# Changing of the Genomic Pattern of *Salmonella* Enteritidis Strains Isolated in Brazil Over a 48 year-period revealed by Whole Genome SNP Analyses

**DOI:** 10.1038/s41598-018-28844-6

**Published:** 2018-07-11

**Authors:** Fábio Campioni, Guojie Cao, George Kastanis, Daniel A. Janies, Alzira Maria Morato Bergamini, Dália dos Prazeres Rodrigues, Robert Stones, Eric Brown, Marc W. Allard, Juliana Pfrimer Falcão

**Affiliations:** 1 Departamento de Análises Clínicas, Toxicológicas e Bromatológicas – Faculdade de Ciências Farmacêuticas de Ribeirão Preto, Universidade de São Paulo, Av. do Café s/n°, Ribeirão Preto, SP Brazil; 2 Division of Microbiology, Center for Food Safety and Applied Nutrition, U.S. Food and Drug Administration, College Park, Maryland, USA; 3 Department of Bioinformatics and Genomics, The University of North Carolina at Charlotte, 9201 University City Blvd, Charlotte, NC 28223 USA; 4 Instituto Adolfo Lutz de Ribeirão Preto, Rua Minas 877, Ribeirão Preto, SP Brazil; 5 Laboratório de Enterobactérias, FIOCRUZ/Fundação Instituto Oswaldo Cruz, Avenida Brasil, 4365, Pavilhão Rocha Lima, 3°andar, Manguinhos, Rio de Janeiro, RJ Brazil; 6 Newcastle University, Newcastle upon Tyne, Newcastle, United Kingdom

## Abstract

*Salmonella* Enteritidis became the main serovar isolated from gastroenteritis cases in Brazil after the 90’s. In this study we used whole genome sequence analysis to determine the phylogenetic relationships among a collection of strains isolated in Brazil to identify possible genomic differences between the strains isolated in the pre and post-epidemic period. Also, we compared our data from strains isolated in Brazil to strains available in the public domain from other South American countries. Illumina technology was used to sequence the genome of 256 *Salmonella* Enteritidis strains isolated over a 48 year-period in Brazil, comprising the pre- and post-epidemic period. Phylogenetic analyses revealed distinct lineages for strains isolated before and after 1994. Moreover, the phage region SE20 that may be related to the emergence of *Salmonella* Enteritidis worldwide was present only in strains of the post-epidemic cluster. In conclusion, our results showed that the genomic profile of *Salmonella* Enteritidis strains isolated in Brazil shifted after 1994, replaced by a global epidemic group of strains. It may be hypothesized that the presence of the prophage SE20 might have conferred to these strains a better ability to colonize chicken and consequently to infect and cause disease in humans, which might better explain the increase in the number of *S*. Enteritidis cases in Brazil and other South American countries. However, to verify this hypothesis further studies are needed.

## Introduction

*Salmonella* Enteritidis (*S*. Enteritidis) is a serovar that colonizes, among other livestock and wild animals, the reproductive tract of chickens, usually asymptomatically, contaminating and surviving in internal egg compounds^1–3^. This interaction with feed-related animals, allowed *S*. Enteritidis to cause a sustained epidemic of infections worldwide through the consumption of raw or undercooked poultry and eggs after the 80’s, becoming one of the top serovars isolated from human salmonellosis^1,4^.

The isolation of *S*. Enteritidis increased from 16% in 1987 to 27% in 1994 in the United States and from 9% to 64% in England^5,6^. In South America, more than 150 outbreaks of *S*. Enteritidis were described in Argentina between 1986 and 1993^7^. In Chile, the increase was from 13.67 strains annually until 1993 to 478 in 1994^8^. In Uruguay, *S*. Enteritidis was sporadically isolated until 1994; however, after 1995 it accounted for more than 50% of the strains received in the *Salmonella* reference center in that country and more than 85% of the strains isolated from humans^9,10^.

In pre-1990’s Brazil, less than 1% of the samples isolated, obtained from human and non-human sources were *S*. Enteritidis^11,12^. However, after 1991 the isolation of this serovar increased and in 1994 it became the most isolated one either from human or non-human sources, found in 65% of the total *Salmonella* isolates^11,13–15^. Moreover, a phage type shift was observed after 1993 and the prevalent phage type isolated, PT-8, was subsequently replaced by PT-4^16^. Currently, although the reports in Brazil do not provide the number of *Salmonella* serovars isolated, this genus was the most isolated agent from foodborne outbreaks between 2007 and 2016, accounting for 7.5% of the total. Moreover, eggs and egg-based products were among the major vehicles of foodborne outbreaks in Brazil at that time^17^.

The reasons for the onset of this epidemic of *S*. Enteritidis are unknown and several factors have been proposed, such as the existence of a rodent reservoir that spread *Salmonella* into farms, the eradication of *S*. Gallinarum by vaccination that enabled *S*. Enteritidis to spread epidemically among chicken flocks and, also a possible pathogen evolution which recently acquired genetic changes that may have facilitated the spread of *S*. Enteritidis among poultry flocks^2,18–21^. However, these facts are not sufficient to explain the recent association of this serovar with eggs, because the *S*. Enteritidis epidemic is composed of different strains defined by the presence of different phage types^9,18^. Moreover, it is not clear why specific phage types are more prevalent in different parts of the world^4^. In Brazil, it is postulated that an epidemic clone of *S*. Enteritidis had been introduced into the country through the commercial exchange of poultry between the United States and European countries^12^. In those countries, the rapid dissemination of this serovar has been related to a probable emergence of a more virulent clone that acquired the ability to colonize and persist in chickens and eggs^18^.

In light of these facts, we used whole genome sequencing (WGS) analyses to assess the phylogenetic relationships among a collection of *S*. Enteritidis strains isolated over a 48 years-period in Brazil, comprising the pre- and post-epidemic period. Previous studies conducted around the world have shown WGS analyses as a powerful tool for evolutionary, phylogenetic and outbreak detection studies of *S*. Enteritidis, a genetically homogenous serovar^22–29^. Our aim was to identify possible genomic differences between the strains isolated in the pre- and post-epidemic period in Brazil that might help to explain how *S*. Enteritidis became the top serovar isolated from gastroenteritis cases in Brazil after the mid 90’s. Also, we compared the strains from Brazil to the strains from other South American countries available online.

## Results

### Whole Genome SNP Analysis

**Figure 1:**
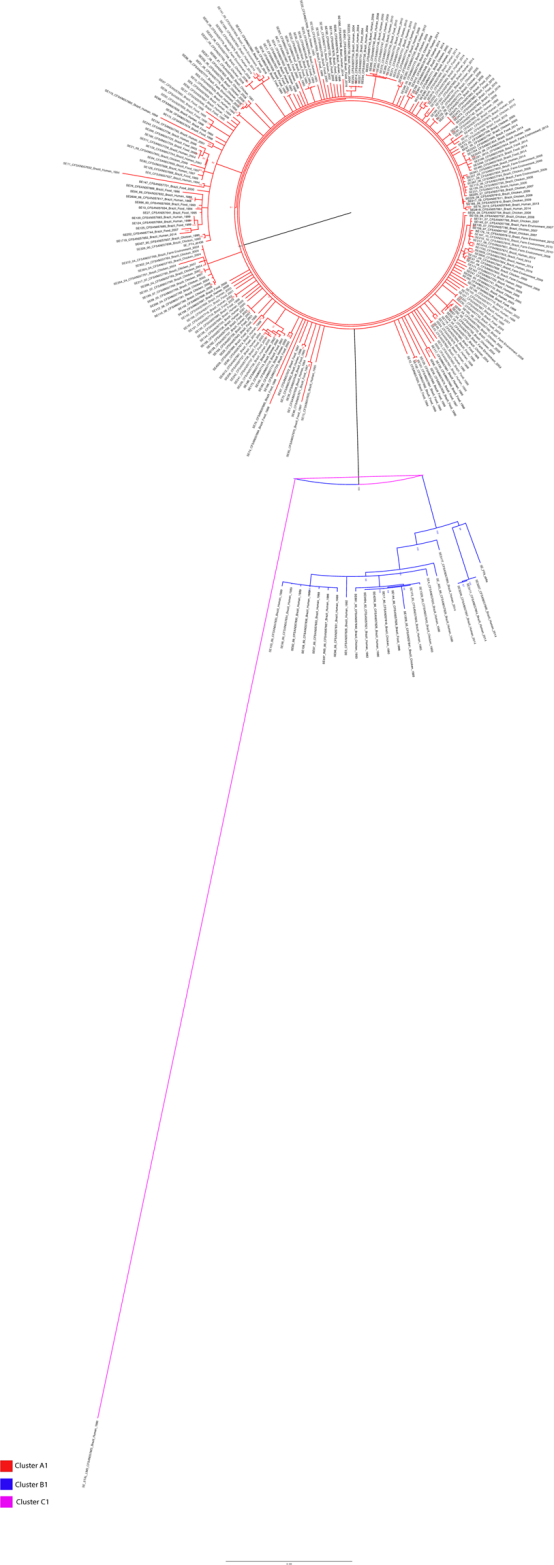
Maximum-likelihood tree based on the SNP-analysis of 256 *Salmonella* Enteritidis strains isolated in Brazil.

**Figure 2:**
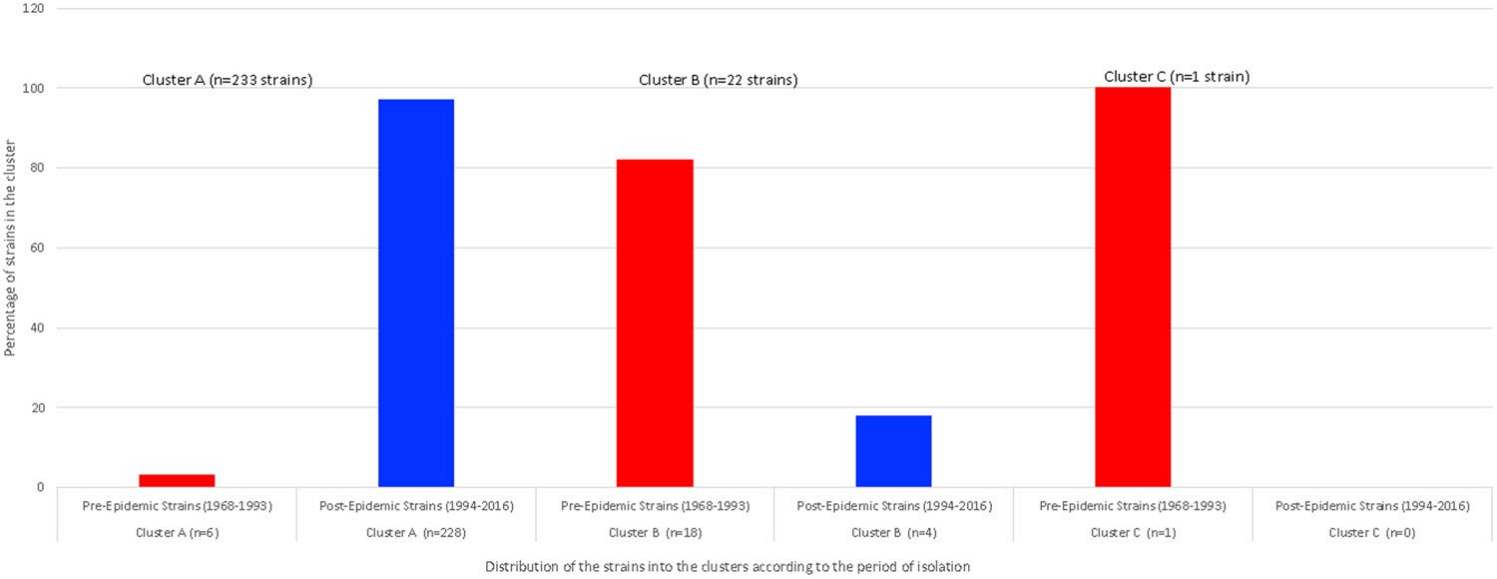
Distribution of the 256 *Salmonella* Enteritidis strains studied into the clusters according to the period of isolation.

The strains isolated in Brazil were divided into two main clusters (Fig. 1). Cluster A consists of 234 strains isolated from humans, food, chickens and farm environments between 1968 and 2016 (Fig. 1). Most of the strains in this cluster, 228 (97%), were isolated between 1994 and 2016 and the remaining six strains (3%) were isolated in 1968, 1969, 1989, 1990 and 1993 (Fig. 2). Moreover, this cluster contained the strain SE_PT4_str436 and the reference P125109, both PT-4 (Fig. 1).

Cluster B consists of 22 strains isolated from humans, food and chickens between 1980 and 2014 (Fig. 1). The majority of these strains, 18 (82%), were isolated between 1980 and 1993 and only four strains (18%) were isolated in 2014 (Fig. 2). Also, this cluster contained the PT-8 strain SE_PT8_str8a (Fig. 1). The strain SE_STAL isolated in 1986 was presented as a single wgSNP-type named as cluster C.

The existence of these clusters was confirmed by statistical analysis using the method Structure (Supplementary Data 1).

When the strains studied were compared to strains from other South American countries, we found the same two main clusters (Supplementary Fig. S1). Cluster A grouped 288 strains isolated in Brazil (n = 237) between 1968 and 2016, in Argentina (n = 17) between 1988 and 2014, in Chile (n = 30) between 2009 and 2012, in Peru in 2012 (n = 2), in Ecuador (n = 1) in 2004 and in Colombia (n = 1) in 2010. Most of the strains in this cluster, 279 (97%), were isolated between 1994 and 2016 and the other nine strains (3%) were isolated in 1968, 1969, 1981, 1988, 1989, 1990. Also, the SE_PT4_str436 strain was found in this cluster (Supplementary Fig. S1). Cluster B grouped 26 strains isolated in Brazil (n = 22) between 2006 and 2014, in Argentina (n = 2) in 1972 and 2006, in Peru (n = 1) in 2008 and also the PT8 strain (n = 1). Most of these strains, 19 (73%), were isolated between 1972 and 1993 and only six strains (23%) were isolated in 2006, 2008 and 2014. Again, the strain SE_STAL isolated in 1986, was presented as a single wgSNP-type in the cluster C (Supplementary Fig. S1).

### Accessory genomes analysis

The φSE20 phage region was absent in all the strains of the pre-epidemic clusters B and C except for the strain SE5, isolated in 1992. In contrast, this region was found in all the strains of the post-epidemic cluster A (Supplementary Table S1).

The Fels-2 (A19) and *S*. Typhi CT18 (A22-24) regions were present in all the strains of the pre-epidemic clusters B and C. These regions were absent in all the strains of the post-epidemic cluster A, except for SE1551, isolated in 2014 and SE3800, isolated in 2016 (Supplementary Table S1).

The p88 region was found only in the strains SE4 and SE5 of the pre-epidemic cluster B and isolated in 1986 and 1992, respectively.

The Gifsy1 region was absent in all but the strain SE1551 of the post-epidemic cluster A and SE_STAL of the pre-epidemic cluster C. The regions ROD21 and SE12 were found in all the strains studied.

## Discussion

In this study, we analyzed *S*. Enteritidis strains isolated in Brazil over a 48 year-period, including the pre- and post-epidemic period. Our findings using WGS improved the characterization of this important poultry-related pathogen circulating in this country, the second largest poultry producer and poultry exporter worldwide^30^.

Whole genome SNP analysis showed that most of the strains isolated between the years 1980 and 1993, cluster together and are divergent from the strains isolated after 1994 to 2016, strongly suggesting that either a new lineage of *S*. Enteritidis was introduced in Brazil and became prevalent after 1994 or an indigenous epidemic lineage spread and prevailed among poultry flocks in the country. The strains isolated before 1994 in this study reflect the isolation of the Enteritidis serovar at that time period and was found to be less than 1% of the total *Salmonella* strains isolated in Brazil^31^. Because of this, the low number of strains isolated in the pre-epidemic period is possibly a limitation of the present study. Once these strains were isolated in the Southern regions of Brazil, a higher number of strains from different regions would better reflect the diversity of *S*. Enteritidis circulating in the country at that time period.

Although we do not know the phage type of the strains studied, the fact that the genomic type shifted corroborates with studies showing that after 1993, the prevalent strains from phage type 8 were surpassed by strains from phage type 4 that became the predominant phage type isolated in Brazil^11,16,31,32^. This fact does not mean that all the pre and post-epidemic strains in this study are necessarily from phage type 8 and 4, respectively, once strains from different phage types can share similar genetic types^25^. However, the results clearly indicate that a new genetic type was inserted into the country after 1994 and became the prevalent one in the country until the present. The clustering of some strains isolated in 2014 with pre-epidemic strains suggest that this lineage was not completely excluded from the environment and is still being isolated in Brazil but in a small number. Similarly, the presence of six strains isolated in 1968, 1969, 1989, 1990 and 1993 in the same cluster of the post-epidemic strains indicates that this lineage was present in the environment in the pre-epidemic period; however it was not the predominant one at that time (Fig. 1).

The fact that in most parts of the world the *S*. Enteritidis epidemic began in the mid-1980’s while in Brazil, it started in the mid-1990’s, can be explained by the broiler production in Brazil that was developed mostly after the 80’s. One hypothesis is that poultry matrices imported from the United States and Europe introduced this lineage in Brazil^12^. Considering that in the United States the predominant phage types are PT-8 and PT-13 and in Europe, the PT-4, it was proposed by some authors that the first *S*. Enteritidis PT-8 strains might have been introduced into Brazil through the exchange of poultry matrices with the United States quickly followed by the European PT-4 strains^12,25^.

In a previous study^33^ comparing 188 *S*. Enteritidis strains from Brazil with 100 strains from North America using Multiple-locus Variable-number tandem repeat analysis (MLVA), the majority of the strains from the United States clustered with two strains from Brazil isolated in 1986 and 1992 (SE5 and SE4), the only two pre-epidemic strains in that study. This cluster was separated from the Brazilian strains isolated after 1994, which showed that the US strains were more similar to the Brazilian pre-epidemic strains than the post-epidemic ones^33^. This fact reinforces the idea that the pre-epidemic strains were introduced in Brazil through the exchange of poultry matrices with the USA.

Our analysis including strains from other South American countries, showed most of the strains isolated from Chile, Ecuador, Colombia and Peru between 2004 and 2012 and subsequently clustering with the post-epidemic strains from Brazil, suggesting that the same epidemic lineage is circulating in various South American countries. The exception was one strain from Peru isolated in 2008 that clustered with the Brazilian pre-epidemic strains. However, the few number of samples from Ecuador, Colombia and Peru available online makes it difficult to infer the predominant genomic type circulating in those countries. Also, the strains from Chile showed to be clonal with 28 of 30 strains clustering together in a sub-cluster with the Brazilian post-epidemic strains (Supplementary Fig. S1).

Two strains from Argentina, isolated in 1972 and 2006, clustered with the Brazilian pre-epidemic strains and the other 17 strains isolated between 1988 and 2014 clustered with the Brazilian post-epidemic strains, showing that both lineages have been isolated in that country in recent years. However, more samples would be necessary for a more accurate analysis (Supplementary Fig. S1).

Although strains from Uruguay were not included in this study, literature data showed an increase of isolation of *S*. Enteritidis after 1995^10,19^. Moreover, phenotypic analysis and comparative genomic data showed differences among strains isolated in the pre- and post-epidemic period in Uruguay, corroborating with data of the present study^9,19^.

The division of the strains studied into two major clusters was also found by Zheng and collaborators that categorized *S*. Enteritidis from 14 different phage types divided into two major clonal lineages. Lineage I included the main phage types isolated in Western Europe like PT1, PT4 and PT21 and lineage II included the most common phage types isolated in North America such as PT8, PT13a and PT13^22^. Although we do not know the phage types of the strains studied, we included a PT8 strain (SE_PT8_str8a) and a PT4 strain (SE_PT4_str426) for comparison purposes and they clustered into the pre- and into the post-epidemic clusters respectively (Fig. 1). Also, the reference strain P125109 from PT4 clustered with the post epidemic strains of this study (Fig. 1). These findings suggest that the strains isolated in the pre-epidemic period in Brazil belonged to lineage II and the post-epidemic strains belonged to lineage I (Fig. 1).

Furthermore, Feasey *et al*. found the genomic diversity of *S*. Enteritidis worldwide clustered into four different groups: the global epidemic clade, outlier clade, Central/Eastern Africa clade and West Africa clade^34^. The P125109 strain and also several strains from Argentina clustered into the global epidemic clade and into the outlier cluster, respectively^34^. In the present study, the same strains mentioned above clustered into the post-epidemic cluster (P125109) and into the pre-epidemic cluster (strains from Argentina), respectively, suggesting that the strains in the pre-epidemic cluster in our study might be part of the outlier cluster and the post-epidemic cluster of the global epidemic clade mentioned by Feasey *et al*., (Supplementary Fig. S1).

Another study conducted by Porwollik *et al*., proposed the *S*. Enteritidis strains divided into two lineages based on the presence or absence of the phage regions SE20, Fels2 and *S*. Typhi CT18, ST27 and ST3^2^. The phage SE20 region is considered a recent acquisition in the PT4 strain P125109 and might be related to the emergence of *Salmonella* Enteritidis worldwide^2^. Our results showed that SE20 is absent in all but one strain in the pre-epidemic cluster and present in all the strains of the post-epidemic cluster and might suggest the hypothesis that the emergence of *S*. Enteritidis in Brazil may also be related to strains that carry that region. However, to verify this hypothesis further studies are needed. Moreover, the regions Fels2 and *S*. Typhi CT18, ST27 and ST35 were present only in the pre-epidemic strains from Brazil. This result is similar to Porwollik’s study that related these regions to strains isolated more than 50 years ago and also confirmed by Betancor *et al*. and Feasey *et al*.^2,19,34^.

Feasey and colleagues reported the prophages Gifsy1 and p88 as related to clades of strains isolated in Africa^34^. In the present study, the region Gifsy1 was found in two strains, the pre-epidemic SE_STAL isolated in 1986 and the post-epidemic SE1551 isolated in 2014 (Supplementary Table S1). Moreover, the region p88 was found in two pre-epidemic strains, the SE4 isolated in 1986 and the SE5 isolated in 1992 (Supplementary Table S1). Also, the prophages ROD21 and SE12 were found in all the strains studied, which is in agreement with Feasey and colleagues that reported these regions as prevalent in all the strains of the global epidemic clade and of the outlier cluster^34^.

In conclusion, our results showed that the genomic profile of *Salmonella* Enteritidis strains isolated in Brazil shifted after 1994, replaced by a global epidemic group of strains. It may be hypothesized that the presence of the prophage SE20 might have conferred to these strains a better ability to colonize chicken and consequently to infect and cause disease in humans, which might better explain the increase in the number of *S*. Enteritidis cases in Brazil and other South American countries. However, to verify this hypothesis further studies are needed.

## Methods

### Bacterial strains

A total of 256 *Salmonella* Enteritidis strains isolated from humans (n = 109), food (n = 78), chickens (n = 52) and farm environments (n = 17) in Brazil between 1968 and 2016 were studied (Supplementary Table S1). Specifically, 26 strains were isolated between 1968 and 1993 (pre-epidemic period) and 230 strains were isolated between 1996 and 2016 (post-epidemic period). The lower number of pre-epidemic strains reflects the isolation of serovar Enteritidis strains in that period that represented less than 1% of the *Salmonella* strains isolated in the country^11,12^. The post-epidemic strains were selected to represent the year and source of isolation of these strains.

The strains were provided by the Adolfo Lutz Institute of Ribeirao Preto (IAL-RP), Oswaldo Cruz Foundation (FIOCRUZ-RJ) and by AVIPA (Avicultura Integral e Patologia S/A) from Brazil. The accession numbers of the strains studied are available in a previous publication^35^.

### Library preparation and sequencing

The libraries and runs were prepared according to Campioni *et al*.^35^ and samples were sequenced at the FDA Center for Food Safety and Applied Nutrition.

### Genomic data analysis

To analyze the phylogenetic relationships among the strains studied, a matrix of SNPs was constructed using the CFSAN SNP pipeline^36^ and the *S*. Enteritidis strain P125109 (GenBank acession n° NC_011294.1) as the reference genome. The GARLI v.2.01 program was used to reconstruct a maximum likelihood phylogenetic tree with the SNP matrix created. We searched the best tree in 100 repetitions and run bootstrap analysis for 1,000 replicates (ratematrix = 6rate; ratehetmodel = gamma). Python program SumTrees was used to generate the ML tree with bootstrap values with threshold 70% (https://pythonhosted.org/DendroPy/programs/sumtrees.html).

A non-phylogenetic statistical analysis using the method Structure was done in order to confirm the groups observed in the phylogenetic tree generated by GARLI^37–39^.

### Genomes for comparison

We used the approaches described above to compare the *S*. Enteritidis strains from Brazil of this study with 57 strains from other South American countries available in GenBank from Brazil (n = 3), Argentina (n = 19), Chile (n = 30), Peru (n = 3), Ecuador (n = 1) and Colombia (n = 1) (Supplementary Table S2). Also, the strains SE_PT4_str436, from PT-4 and SE_PT8_str8a, from PT-8 were included in the analysis^22^.

### Accessory genomes analysis

Basic local alignment search tool (BLAST)^40^ was used to look for the phage regions φSE20 (phage similar to φST64B), Fels-2 regions (A19), the *S*. Typhi CT18 ST27 and ST35 (A22 to A24), P88 (closely related to Enterobacter Phage P88), Gifsy-1 prophage (found in *S*. Bovismorbificans), Gifsy-2 and SE12 (degenerated in the reference P125109) and ROD21 (pathogenicity island exclusive of *S*. Enteritidis), according to the proposed by previous studies^2,19,22,34^.

## Electronic supplementary material

Supplementary Dataset

Supplementary Information
