# Supplementary material for: Changing of the Genomic Pattern of Salmonella Enteritidis Strains Isolated in Brazil Over a 48 year-period revealed by Whole Genome SNP Analyses
Source: Sci Rep. 2018 Jul 11;8:10478. doi: 10.1038/s41598-018-28844-6 (PMC6041339; doi:10.1038/s41598-018-28844-6)
Supplement: Supplementary file 1 — Supplementary Dataset [file 41598_2018_28844_MOESM1_ESM.pdf]

TITLE: Changing of the Genomic Pattern of *Salmonella* Enteritidis Strains Isolated in Brazil Over a 48 year-period revealed by Whole Genome SNP Analyses

Campioni, Fábio[1]; Cao, Guojie[2]; Kastanis, George[2]; Janies, Daniel A.[3] Bergamini, Alzira Maria Morato[4]; Rodrigues, Dália dos Prazeres[5]; Stones, Robert[6]; Brown, Eric[2]; Allard, Marc W.[2*]; Falcão, Juliana Pfrimer[1*]

[1] – Departamento de Análises Clínicas, Toxicológicas e Bromatológicas – Faculdade de Ciências Farmacêuticas de Ribeirão Preto, Universidade de São Paulo, Av. do Café s/ nº, Ribeirão Preto, SP Brasil.

[2] – Division of Microbiology, Center for Food Safety and Applied Nutrition, U.S. Food and Drug Administration, College Park, Maryland, United States of America

[3] – Department of Bioinformatics and Genomics, The University of North Carolina at Charlotte, 9201 University City Blvd, Charlotte, NC 28223, United States of America

[4] – Instituto Adolfo Lutz de Ribeirão Preto, Rua Minas 877, Ribeirão Preto, SP, Brasil.

[5]– Laboratório de Enterobactérias, FIOCRUZ/Fundação Instituto Oswaldo Cruz, Avenida Brasil, 4365, Pavilhão Rocha Lima, 3°andar, Manguinhos, Rio de Janeiro, RJ, Brasil.

[6]- Newcastle University, Newcastle upon Tyne, United Kingdom.

Corresponding authors*:

Juliana Pfrimer Falcão

Phone.: + 55-16-36024896; FAX: + 55-16-36024725

Email: jufalcao@fcfrp.usp.br

Marc Allard

Phone: +1-240-402-2941

E-mail: marc.allard@fda.hhs.gov

Supplementary Table S1 – Metadata for the 256 *Salmonella* Enteritidis strains isolated in Brazil in this study.

Original Strain Number	FDA Number	Year of Isolation	Source of Isolation	Brazilian State of Isolation	wgSNP analysis cluster	φSE20 region	Fels-2 regions	CT18	Gifsy1	Phage p88	ROD21	φSE12
SE6	CFSAN057627	1994	Human	São Paulo	A	+	-	-	-	-	+	+
SE7	CFSAN057628	1994	Human	São Paulo	A	+	-	-	-	-	+	+
SE8	CFSAN057629	1994	Human	São Paulo	A	+	-	-	-	-	+	+
SE9	CFSAN057630	1994	Human	São Paulo	A	+	-	-	-	-	+	+
SE10	CFSAN057631	1994	Human	São Paulo	A	+	-	-	-	-	+	+
SE11	CFSAN057632	1994	Human	São Paulo	A	+	-	-	-	-	+	+
SE12	CFSAN057633	1994	Food	São Paulo	A	+	-	-	-	-	+	+
SE13	CFSAN057634	1994	Food	São Paulo	A	+	-	-	-	-	+	+
SE15	CFSAN057635	1995	Human	São Paulo	A	+	-	-	-	-	+	+
SE16	CFSAN057636	1995	Human	São Paulo	A	+	-	-	-	-	+	+
SE17	CFSAN057637	1995	Food	São Paulo	A	+	-	-	-	-	+	+
SE18	CFSAN057638	1995	Food	São Paulo	A	+	-	-	-	-	+	+
SE22	CFSAN057639	1995	Human	São Paulo	A	+	-	-	-	-	+	+
SE23	CFSAN057640	1995	Human	São Paulo	A	+	-	-	-	-	+	+
SE27	CFSAN057641	1995	Food	São Paulo	A	+	-	-	-	-	+	+
SE28	CFSAN057642	1995	Food	São Paulo	A	+	-	-	-	-	+	+
SE29	CFSAN057643	1995	Food	São Paulo	A	+	-	-	-	-	+	+
SE30	CFSAN057644	1995	Food	São Paulo	A	+	-	-	-	-	+	+
SE32	CFSAN057645	1995	Food	São Paulo	A	+	-	-	-	-	+	+

SE33	CFSAN057646	1995	Food	São Paulo	A	+	-	-	-	-	+	+
SE35	CFSAN057647	1995	Food	São Paulo	A	+	-	-	-	-	+	+
SE36	CFSAN057648	1995	Food	São Paulo	A	+	-	-	-	-	+	+
SE37	CFSAN057649	1995	Food	São Paulo	A	+	-	-	-	-	+	+
SE38	CFSAN057650	1995	Food	São Paulo	A	+	-	-	-	-	+	+
SE39	CFSAN057651	1995	Food	São Paulo	A	+	-	-	-	-	+	+
SE41	CFSAN057652	1996	Human	São Paulo	A	+	-	-	-	-	+	+
SE43	CFSAN057653	1996	Human	São Paulo	A	+	-	-	-	-	+	+
SE48	CFSAN057654	1996	Human	São Paulo	A	+	-	-	-	-	+	+
SE50	CFSAN057655	1996	Human	São Paulo	A	+	-	-	-	-	+	+
SE56	CFSAN057656	1996	Food	São Paulo	A	+	-	-	-	-	+	+
SE59	CFSAN057657	1996	Human	São Paulo	A	+	-	-	-	-	+	+
SE60	CFSAN057658	1996	Food	São Paulo	A	+	-	-	-	-	+	+
SE66	CFSAN057659	1996	Human	São Paulo	A	+	-	-	-	-	+	+
SE67	CFSAN057660	1996	Food	São Paulo	A	+	-	-	-	-	+	+
SE68	CFSAN057661	1996	Human	São Paulo	A	+	-	-	-	-	+	+
SE70	CFSAN057662	1996	Human	São Paulo	A	+	-	-	-	-	+	+
SE73	CFSAN057663	1996	Food	São Paulo	A	+	-	-	-	-	+	+
SE74	CFSAN057664	1996	Food	São Paulo	A	+	-	-	-	-	+	+
SE75	CFSAN057665	1996	Food	São Paulo	A	+	-	-	-	-	+	+
SE76	CFSAN057666	1996	Food	São Paulo	A	+	-	-	-	-	+	+
SE77	CFSAN057667	1996	Human	São Paulo	A	+	-	-	-	-	+	+

SE80	CFSAN057668	1997	Human	São Paulo	A	+	-	-	-	-	+	+
SE84	CFSAN057669	1997	Food	São Paulo	A	+	-	-	-	-	+	+
SE85	CFSAN057670	1997	Food	São Paulo	A	+	-	-	-	-	+	+
SE86	CFSAN057671	1997	Food	São Paulo	A	+	-	-	-	-	+	+
SE87	CFSAN057672	1997	Food	São Paulo	A	+	-	-	-	-	+	+
SE88	CFSAN057673	1997	Food	São Paulo	A	+	-	-	-	-	+	+
SE89	CFSAN057674	1998	Human	São Paulo	A	+	-	-	-	-	+	+
SE94	CFSAN057675	1998	Human	São Paulo	A	+	-	-	-	-	+	+
SE96	CFSAN057676	1998	Human	São Paulo	A	+	-	-	-	-	+	+
SE100	CFSAN057677	1998	Human	São Paulo	A	+	-	-	-	-	+	+
SE114	CFSAN057678	1998	Food	São Paulo	A	+	-	-	-	-	+	+
SE115	CFSAN057679	1998	Food	São Paulo	A	+	-	-	-	-	+	+
SE116	CFSAN057680	1998	Food	São Paulo	A	+	-	-	-	-	+	+
SE117	CFSAN057681	1999	Human	São Paulo	A	+	-	-	-	-	+	+
SE119	CFSAN057682	1999	Human	São Paulo	A	+	-	-	-	-	+	+
SE120	CFSAN057683	1999	Human	São Paulo	A	+	-	-	-	-	+	+
SE124	CFSAN057684	1999	Human	São Paulo	A	+	-	-	-	-	+	+
SE125	CFSAN057685	1999	Food	São Paulo	A	+	-	-	-	-	+	+
SE126	CFSAN057686	1999	Food	São Paulo	A	+	-	-	-	-	+	+
SE127	CFSAN057687	1999	Food	São Paulo	A	+	-	-	-	-	+	+
SE-128	CFSAN057688	1999	Food	São Paulo	A	+	-	-	-	-	+	+
SE-129	CFSAN057689	1999	Food	São Paulo	A	+	-	-	-	-	+	+

SE130	CFSAN057690	1999	Food	São Paulo	A	+	-	-	-	-	+	+
SE131	CFSAN057691	1999	Food	São Paulo	A	+	-	-	-	-	+	+
SE132	CFSAN057692	1999	Food	São Paulo	A	+	-	-	-	-	+	+
SE133	CFSAN057693	1999	Food	São Paulo	A	+	-	-	-	-	+	+
SE134	CFSAN057694	1999	Food	São Paulo	A	+	-	-	-	-	+	+
SE135	CFSAN057695	1999	Food	São Paulo	A	+	-	-	-	-	+	+
SE136	CFSAN057696	2000	Human	São Paulo	A	+	-	-	-	-	+	+
SE139	CFSAN057697	2000	Human	São Paulo	A	+	-	-	-	-	+	+
SE142	CFSAN057698	2000	Food	São Paulo	A	+	-	-	-	-	+	+
SE143	CFSAN057699	2000	Human	São Paulo	A	+	-	-	-	-	+	+
SE144	CFSAN057700	2000	Human	São Paulo	A	+	-	-	-	-	+	+
SE147	CFSAN057701	2000	Food	São Paulo	A	+	-	-	-	-	+	+
SE150	CFSAN057702	2000	Food	São Paulo	A	+	-	-	-	-	+	+
SE151	CFSAN057703	2001	Human	São Paulo	A	+	-	-	-	-	+	+
SE153	CFSAN057704	2001	Human	São Paulo	A	+	-	-	-	-	+	+
SE154	CFSAN057705	2001	Human	São Paulo	A	+	-	-	-	-	+	+
SE156	CFSAN057706	2001	Human	São Paulo	A	+	-	-	-	-	+	+
SE158	CFSAN057707	2001	Human	São Paulo	A	+	-	-	-	-	+	+
SE162	CFSAN057708	2001	Food	São Paulo	A	+	-	-	-	-	+	+
SE164	CFSAN057709	2001	Food	São Paulo	A	+	-	-	-	-	+	+
SE165	CFSAN057710	2001	Food	São Paulo	A	+	-	-	-	-	+	+
SE168	CFSAN057711	2001	Food	São Paulo	A	+	-	-	-	-	+	+

SE171	CFSAN057712	2002	Human	São Paulo	A	+	-	-	-	-	+	+
SE173	CFSAN057713	2002	Human	São Paulo	A	+	-	-	-	-	+	+
SE181	CFSAN057714	2002	Human	São Paulo	A	+	-	-	-	-	+	+
SE183	CFSAN057715	2002	Human	São Paulo	A	+	-	-	-	-	+	+
SE185	CFSAN057716	2002	Human	São Paulo	A	+	-	-	-	-	+	+
SE186	CFSAN057717	2002	Human	São Paulo	A	+	-	-	-	-	+	+
SE187	CFSAN057718	2002	Human	São Paulo	A	+	-	-	-	-	+	+
SE191	CFSAN057719	2002	Food	São Paulo	A	+	-	-	-	-	+	+
SE192	CFSAN057720	2002	Food	São Paulo	A	+	-	-	-	-	+	+
SE193	CFSAN057721	2003	Human	São Paulo	A	+	-	-	-	-	+	+
SE197	CFSAN057722	2003	Human	São Paulo	A	+	-	-	-	-	+	+
SE198	CFSAN057723	2003	Food	São Paulo	A	+	-	-	-	-	+	+
SE199	CFSAN057724	2003	Food	São Paulo	A	+	-	-	-	-	+	+
SE200	CFSAN057725	2003	Food	São Paulo	A	+	-	-	-	-	+	+
SE201	CFSAN057726	2003	Food	São Paulo	A	+	-	-	-	-	+	+
SE206	CFSAN057727	2004	Human	São Paulo	A	+	-	-	-	-	+	+
SE211	CFSAN057728	2004	Human	São Paulo	A	+	-	-	-	-	+	+
SE214	CFSAN057729	2004	Human	São Paulo	A	+	-	-	-	-	+	+
SE223	CFSAN057730	2004	Human	São Paulo	A	+	-	-	-	-	+	+
SE227	CFSAN057731	2004	Food	São Paulo	A	+	-	-	-	-	+	+
SE230	CFSAN057732	2004	Food	São Paulo	A	+	-	-	-	-	+	+
SE231	CFSAN057733	2004	Food	São Paulo	A	+	-	-	-	-	+	+

SE233	CFSAN057734	2004	Human	São Paulo	A	+	-	-	-	-	+	+
SE234	CFSAN057735	2004	Human	São Paulo	A	+	-	-	-	-	+	+
SE239	CFSAN057736	2005	Human	São Paulo	A	+	-	-	-	-	+	+
SE241	CFSAN057737	2005	Food	São Paulo	A	+	-	-	-	-	+	+
SE242	CFSAN057738	2005	Food	São Paulo	A	+	-	-	-	-	+	+
SE244	CFSAN057739	2006	Human	São Paulo	A	+	-	-	-	-	+	+
SE246	CFSAN057740	2006	Human	São Paulo	A	+	-	-	-	-	+	+
SE247	CFSAN057741	2006	Human	São Paulo	A	+	-	-	-	-	+	+
SE249	CFSAN057742	2006	Human	São Paulo	A	+	-	-	-	-	+	+
SE251	CFSAN057743	2006	Human	São Paulo	A	+	-	-	-	-	+	+
SE252	CFSAN057744	2007	Food	São Paulo	A	+	-	-	-	-	+	+
SE253	CFSAN057745	2009	Human	São Paulo	A	+	-	-	-	-	+	+
SE255	CFSAN057746	2009	Human	São Paulo	A	+	-	-	-	-	+	+
SE260	CFSAN057747	2009	Human	São Paulo	A	+	-	-	-	-	+	+
SE263	CFSAN057748	2009	Human	São Paulo	A	+	-	-	-	-	+	+
SE267	CFSAN057749	2009	Human	São Paulo	A	+	-	-	-	-	+	+
SE269	CFSAN057750	2010	Food	São Paulo	A	+	-	-	-	-	+	+
SE270	CFSAN057751	2010	Human	São Paulo	A	+	-	-	-	-	+	+
SE271	CFSAN057752	2010	Human	São Paulo	A	+	-	-	-	-	+	+
SE272	CFSAN057753	2010	Human	São Paulo	A	+	-	-	-	-	+	+
SE273	CFSAN057754	2010	Human	São Paulo	A	+	-	-	-	-	+	+
SE274	CFSAN057755	2010	Human	São Paulo	A	+	-	-	-	-	+	+

SE285	CFSAN057756	2012	Human	São Paulo	A	+	-	-	-	-	+	+
SE34_04	CFSAN057757	2004	Chicken	Mato Grosso	A	+	-	-	-	-	+	+
SE55_04	CFSAN057758	2004	Farm Environment	Pernambuco	A	+	-	-	-	-	+	+
SE67_04	CFSAN057759	2004	Chicken	Paraná	A	+	-	-	-	-	+	+
SE69_04	CFSAN057760	2004	Chicken	Paraná	A	+	-	-	-	-	+	+
SE294_04	CFSAN057761	2004	Chicken	Sergipe	A	+	-	-	-	-	+	+
SE296_04	CFSAN057762	2004	Chicken	Sergipe	A	+	-	-	-	-	+	+
SE298_04	CFSAN057763	2004	Chicken	Sergipe	A	+	-	-	-	-	+	+
SE302_04	CFSAN057764	2004	Chicken	Sergipe	A	+	-	-	-	-	+	+
SE304_04	CFSAN057765	2004	Chicken	Sergipe	A	+	-	-	-	-	+	+
SE312_04	CFSAN057766	2004	Farm Environment	Sergipe	A	+	-	-	-	-	+	+
SE67_05	CFSAN057767	2005	Chicken	Pernambuco	A	+	-	-	-	-	+	+
SE69_05	CFSAN057768	2005	Chicken	Pernambuco	A	+	-	-	-	-	+	+
SE73_05	CFSAN057769	2005	Chicken	Pernambuco	A	+	-	-	-	-	+	+
SE226_05	CFSAN057770	2005	Chicken	São Paulo	A	+	-	-	-	-	+	+
SE275_05	CFSAN057771	2005	Chicken	Pernambuco	A	+	-	-	-	-	+	+
SE332_05	CFSAN057772	2005	Chicken	São Paulo	A	+	-	-	-	-	+	+
SE348_05	CFSAN057773	2005	Chicken	Pernambuco	A	+	-	-	-	-	+	+
SE413_05	CFSAN057774	2005	Chicken	Paraná	A	+	-	-	-	-	+	+
SE27_06	CFSAN057775	2006	Chicken	Pernambuco	A	+	-	-	-	-	+	+
SE31_06	CFSAN057776	2006	Chicken	Pernambuco	A	+	-	-	-	-	+	+
SE88_06	CFSAN057777	2006	Farm Environment	São Paulo	A	+	-	-	-	-	+	+

SE98_06	CFSAN057778	2006	Farm Environment	São Paulo	A	+	-	-	-	-	+	+
SE102_06	CFSAN057779	2006	Chicken	Pernambuco	A	+	-	-	-	-	+	+
SE110_06	CFSAN057780	2006	Chicken	Pernambuco	A	+	-	-	-	-	+	+
SE112_06	CFSAN057781	2006	Chicken	Pernambuco	A	+	-	-	-	-	+	+
SE150_06	CFSAN057782	2006	Farm Environment	São Paulo	A	+	-	-	-	-	+	+
SE355_06	CFSAN057783	2006	Chicken	São Paulo	A	+	-	-	-	-	+	+
SE387_06	CFSAN057784	2006	Chicken	São Paulo	A	+	-	-	-	-	+	+
SE131_07	CFSAN057785	2007	Chicken	Pernambuco	A	+	-	-	-	-	+	+
SE135_07	CFSAN057786	2007	Chicken	Pernambuco	A	+	-	-	-	-	+	+
SE139_07	CFSAN057787	2007	Chicken	São Paulo	A	+	-	-	-	-	+	+
SE143_07	CFSAN057788	2007	Farm Environment	Paraná	A	+	-	-	-	-	+	+
SE189_07	CFSAN057789	2007	Chicken	Pernambuco	A	+	-	-	-	-	+	+
SE191_07	CFSAN057790	2007	Chicken	Paraná	A	+	-	-	-	-	+	+
SE199_07	CFSAN057791	2007	Chicken	Paraná	A	+	-	-	-	-	+	+
SE217_07	CFSAN057792	2007	Chicken	Pernambuco	A	+	-	-	-	-	+	+
SE255_07	CFSAN057793	2007	Chicken	São Paulo	A	+	-	-	-	-	+	+
SE05_08	CFSAN057794	2008	Chicken	São Paulo	A	+	-	-	-	-	+	+
SE27_08	CFSAN057795	2008	Chicken	Pernambuco	A	+	-	-	-	-	+	+
SE129_08	CFSAN057796	2008	Chicken	São Paulo	A	+	-	-	-	-	+	+
SE133_08	CFSAN057797	2008	Chicken	São Paulo	A	+	-	-	-	-	+	+
SE203_08	CFSAN057798	2008	Chicken	São Paulo	A	+	-	-	-	-	+	+
SE227_08	CFSAN057799	2008	Chicken	São Paulo	A	+	-	-	-	-	+	+

SE257_08	CFSAN057800	2008	Chicken	São Paulo	A	+	-	-	-	-	+	+
SE317_08	CFSAN057801	2008	Farm Environment	São Paulo	A	+	-	-	-	-	+	+
SE327_08	CFSAN057802	2008	Farm Environment	São Paulo	A	+	-	-	-	-	+	+
SE331_08	CFSAN057803	2008	Farm Environment	Pernambuco	A	+	-	-	-	-	+	+
SE11_09	CFSAN057804	2009	Farm Environment	Minas Gerais	A	+	-	-	-	-	+	+
SE21_09	CFSAN057805	2009	Chicken	São Paulo	A	+	-	-	-	-	+	+
SE37_09	CFSAN057806	2009	Chicken	Pernambuco	A	+	-	-	-	-	+	+
SE107_09	CFSAN057807	2009	Farm Environment	Pernambuco	A	+	-	-	-	-	+	+
SE113_09	CFSAN057808	2009	Farm Environment	São Paulo	A	+	-	-	-	-	+	+
SE141_09	CFSAN057809	2009	Farm Environment	São Paulo	A	+	-	-	-	-	+	+
SE165_09	CFSAN057810	2009	Chicken	São Paulo	A	+	-	-	-	-	+	+
SE217_09	CFSAN057811	2009	Chicken	São Paulo	A	+	-	-	-	-	+	+
SE229_09	CFSAN057812	2009	Chicken	São Paulo	A	+	-	-	-	-	+	+
SE179_10	CFSAN057813	2010	Farm Environment	São Paulo	A	+	-	-	-	-	+	+
SE191_10	CFSAN057814	2010	Chicken	Pernambuco	A	+	-	-	-	-	+	+
SE197_10	CFSAN057815	2010	Farm Environment	São Paulo	A	+	-	-	-	-	+	+
SE219_10	CFSAN057816	2010	Farm Environment	São Paulo	A	+	-	-	-	-	+	+
SE2639_68	CFSAN057817	1968	Human	Pernambuco	A	+	-	-	-	-	+	+
SE3243_69	CFSAN057818	1969	Human	Pernambuco	A	+	-	-	-	-	+	+
SE569_81	CFSAN057820	1981	Chicken	São Paulo	A	+	-	-	-	-	+	+
SE94_89	CFSAN057832	1989	Human	Minas Gerais	A	+	+	+	-	-	+	+
SE327_90	CFSAN057837	1990	Chicken	São Paulo	A	+	-	-	-	-	+	+

SE326_90	CFSAN057838	1990	Chicken	São Paulo	A	+	-	-	-	-	+	+
SE896_93	CFSAN057839	1993	Food	Rio Grande do Sul	A	+	-	-	-	-	+	+
SE76_2013	CFSAN057843	2013	Human	Bahia	A	+	-	-	-	-	+	+
SE221_2013	CFSAN057844	2013	Farm Environment	Minas Gerais	A	+	-	-	-	-	+	+
SE460	CFSAN057845	2014	Food	Santa Catarina	A	+	-	-	-	-	+	+
SE795	CFSAN057846	2014	Human	Bahia	A	+	-	-	-	-	+	+
SE801	CFSAN057847	2014	Human	Bahia	A	+	-	-	-	-	+	+
SE961	CFSAN057848	2014	Human	Ceará	A	+	-	-	-	-	+	+
SE1356	CFSAN057849	2014	Human	Minas Gerais	A	+	-	-	-	-	+	+
SE1396	CFSAN057850	2014	Human	Rio Grande do Sul	A	+	-	-	-	-	+	+
SE1551	CFSAN057851	2014	Human	Mato Grosso do Sul	A	+	-	-	+	-	+	+
SE1719	CFSAN057852	2014	Human	Minas Gerais	A	+	-	-	-	-	+	+
SE1733	CFSAN057853	2014	Food	Rio de Janeiro	A	+	-	-	-	-	+	+
SE1794	CFSAN057854	2014	Chicken	Minas Gerais	A	+	-	-	-	-	+	+
SE1931	CFSAN057855	2014	Human	Rio Grande do Sul	A	+	-	-	-	-	+	+
SE2198	CFSAN057858	2014	Food	São Paulo	A	+	-	-	-	-	+	+
SE2232	CFSAN057859	2014	Human	Minas Gerais	A	+	-	-	-	-	+	+
SE2252	CFSAN057860	2014	Food	Santa Catarina	A	+	-	-	-	-	+	+
SE2818	CFSAN057861	2014	Human	Bahia	A	+	-	-	-	-	+	+
SE2819	CFSAN057862	2014	Food	Acre	A	+	-	-	-	-	+	+
SE2829	CFSAN057863	2014	Human	Minas Gerais	A	+	-	-	-	-	+	+
SE4245	CFSAN057868	2014	Human	Bahia	A	+	-	-	-	-	+	+

SE4280	CFSAN057869	2014	Human	Distrito Federal	A	+	-	-	-	-	+	+
SE4451	CFSAN057870	2014	Human	Santa Catarina	A	+	-	-	-	-	+	+
SE5012	CFSAN057872	2014	Human	Bahia	A	+	-	-	-	-	+	+
SE5346	CFSAN057873	2014	Food	Mato Grosso do Sul	A	+	-	-	-	-	+	+
SE5413	CFSAN057874	2014	Human	Pará	A	+	-	-	-	-	+	+
SE5535	CFSAN057875	2014	Human	Pernambuco	A	+	-	-	-	-	+	+
SE3472	CFSAN057880	2016	Food	Minas Gerais	A	+	-	-	-	-	+	+
SE3473	CFSAN057881	2016	Food	Minas Gerais	A	+	-	-	-	-	+	+
SE3474	CFSAN057882	2016	Food	Minas Gerais	A	+	-	-	-	-	+	+
SE3475	CFSAN057883	2016	Food	Minas Gerais	A	+	-	-	-	-	+	+
SE3800	CFSAN057884	2016	Human	Minas Gerais	A	+	-	-	-	-	+	+
SE4501	CFSAN057885	2016	Food	Minas Gerais	A	+	-	-	-	-	+	+
SE4509	CFSAN057888	2016	Food	Minas Gerais	A	+	-	-	-	-	+	+
SE4510	CFSAN057889	2016	Food	Minas Gerais	A	+	-	-	-	-	+	+
SE4511	CFSAN057890	2016	Food	Minas Gerais	A	+	-	-	-	-	+	+
SE4512	CFSAN057891	2016	Food	Minas Gerais	A	+	-	-	-	-	+	+
SE4	CFSAN057625	1986	Human	São Paulo	B	-	+	+	-	+	+	+
SE5	CFSAN057626	1992	Human	São Paulo	B	+	+	+	-	+	+	+
SE17_80	CFSAN057819	1980	Chicken	Rio de Janeiro	B	-	+	+	-	-	+	+
SE3494_82	CFSAN057821	1982	Human	Minas Gerais	B	-	+	+	-	-	+	+
SE172_85	CFSAN057823	1985	Human	Rio Grande do Sul	B	-	+	+	-	-	+	+
SE4258_86	CFSAN057826	1986	Human	Bahia	B	-	+	+	-	-	+	+

SE337_R65_86	CFSAN057827	1986	Human	Rio Grande do Sul	B	-	+	+	-	-	+	+
SE144_88	CFSAN057828	1988	Food	Minas Gerais	B	-	+	+	-	-	+	+
SEA65_88	CFSAN057829	1988	Human	Minas Gerais	B	-	+	+	-	-	+	+
SE92_89	CFSAN057830	1989	Human	Minas Gerais	B	-	+	+	-	-	+	+
SE96_89	CFSAN057831	1989	Human	Minas Gerais	B	-	+	+	-	-	+	+
SE97_89	CFSAN057833	1989	Human	Minas Gerais	B	-	+	+	-	-	+	+
SE98_89	CFSAN057834	1989	Human	Minas Gerais	B	-	+	+	-	-	+	+
SE103_89	CFSAN057835	1989	Human	Minas Gerais	B	-	+	+	-	-	+	+
SE108_89	CFSAN057836	1989	Human	Minas Gerais	B	-	+	+	-	-	+	+
SE881_93	CFSAN057840	1993	Chicken	Santa Catarina	B	-	+	+	-	-	+	+
SE1328_93	CFSAN057841	1993	Chicken	Santa Catarina	B	-	+	+	-	-	+	+
SE1329_93	CFSAN057842	1993	Chicken	Santa Catarina	B	-	+	+	-	-	+	+
SE3171	CFSAN057864	2014	Human	Santa Catarina	B	-	+	+	-	-	+	+
SE3177	CFSAN057865	2014	Human	Mato Grosso do Sul	B	-	+	+	-	-	+	+
SE3207	CFSAN057866	2014	Human	Santa Catarina	B	-	+	+	-	-	+	+
SE3209	CFSAN057867	2014	Human	Santa Catarina	B	-	+	+	-	-	+	+
SE_STAL	CFSAN057825	1986	Human	Rio Grande do Sul	C	-	+	+	+	-	+	+

Supplementary Table S2: Metadata for the 57 *Salmonella* Enteritidis strains from South American Countries included in this study.

SRA number	Biosample	Country of isolation	Year of isolation
SRR5173681	SAMN06113950	Brazil	2000
SRR5181396	SAMN06113985	Brazil	1995
SRR4427825	SAMN03743889	Brazil	2006
SRR4427824	SAMN03743890	Argentina	2006
SRR5057134	SAMN03743894	Argentina	2014
SRR5057146	SAMN03743896	Argentina	2005
SRR1998938	SAMN03492334	Argentina	2008
SRR1998945	SAMN03492328	Argentina	2006
SRR1998946	SAMN03492332	Argentina	2009
SRR1998947	SAMN03492333	Argentina	2008
SRR1998964	SAMN03492331	Argentina	2006
SRR1998969	SAMN03492327	Argentina	2006
SRR3721476	SAMN03743858	Argentina	2005
SRR4427823	SAMN03743891	Argentina	2006
ERR353344	SAMEA2156540	Argentina	2011
ERR353345	SAMEA2157002	Argentina	2012
ERR353346	SAMEA2159950	Argentina	1995
ERR353347	SAMEA2159409	Argentina	2005
ERR353348	SAMEA2152972	Argentina	1972
ERR353349	SAMEA2144838	Argentina	1990
ERR353350	SAMEA2155866	Argentina	1988
ERR353351	SAMEA2142557	Argentina	2006
SRR2176194	SAMN03079672	Chile	2009
SRR2176220	SAMN03079675	Chile	2010
SRR2176221	SAMN03079676	Chile	2011
SRR2176222	SAMN03079677	Chile	2012
SRR2176223	SAMN03079678	Chile	2012

SRR2176225	SAMN03079680	Chile	2012
SRR2176195	SAMN03079681	Chile	2012
SRR2176198	SAMN03079684	Chile	2012
SRR2176197	SAMN03079683	Chile	2012
SRR2176199	SAMN03079685	Chile	2012
SRR2176200	SAMN03079686	Chile	2012
SRR2176201	SAMN03079687	Chile	2012
SRR2176205	SAMN03079673	Chile	2009
SRR2176219	SAMN03079674	Chile	2010
SRR2176224	SAMN03079679	Chile	2012
SRR2176196	SAMN03079682	Chile	2012
SRR2176202	SAMN03079688	Chile	2012
SRR2176203	SAMN03079689	Chile	2012
SRR2176204	SAMN03079690	Chile	2012
SRR2176193	SAMN03079671	Chile	2009
SRR2176208	SAMN03079691	Chile	2011
SRR2176209	SAMN03079692	Chile	2012
SRR2176211	SAMN03079694	Chile	2012
SRR2176210	SAMN03079693	Chile	2012
SRR2176213	SAMN03079695	Chile	2012
SRR2176214	SAMN03079696	Chile	2012
SRR2176215	SAMN03079697	Chile	2012
SRR2176216	SAMN03079698	Chile	2012
SRR2176217	SAMN03079699	Chile	2012
SRR2176218	SAMN03079700	Chile	2012
SRR3931883	SAMN05417496	Peru	2012
SRR3931884	SAMN05417497	Peru	2012
SRR4098714	SAMN02846140	Peru	2008
SRR1743998	SAMN02843988	Ecuador	2004
SRR5218453	SAMN06283410	Colombia	2010

Supplementary Figure S1 – Maximum-likelihood tree based on the SNP-analysis of 313 *Salmonella* Enteritidis strains isolated in Argentina, Brazil, Chile, Colombia, Ecuador and Peru.

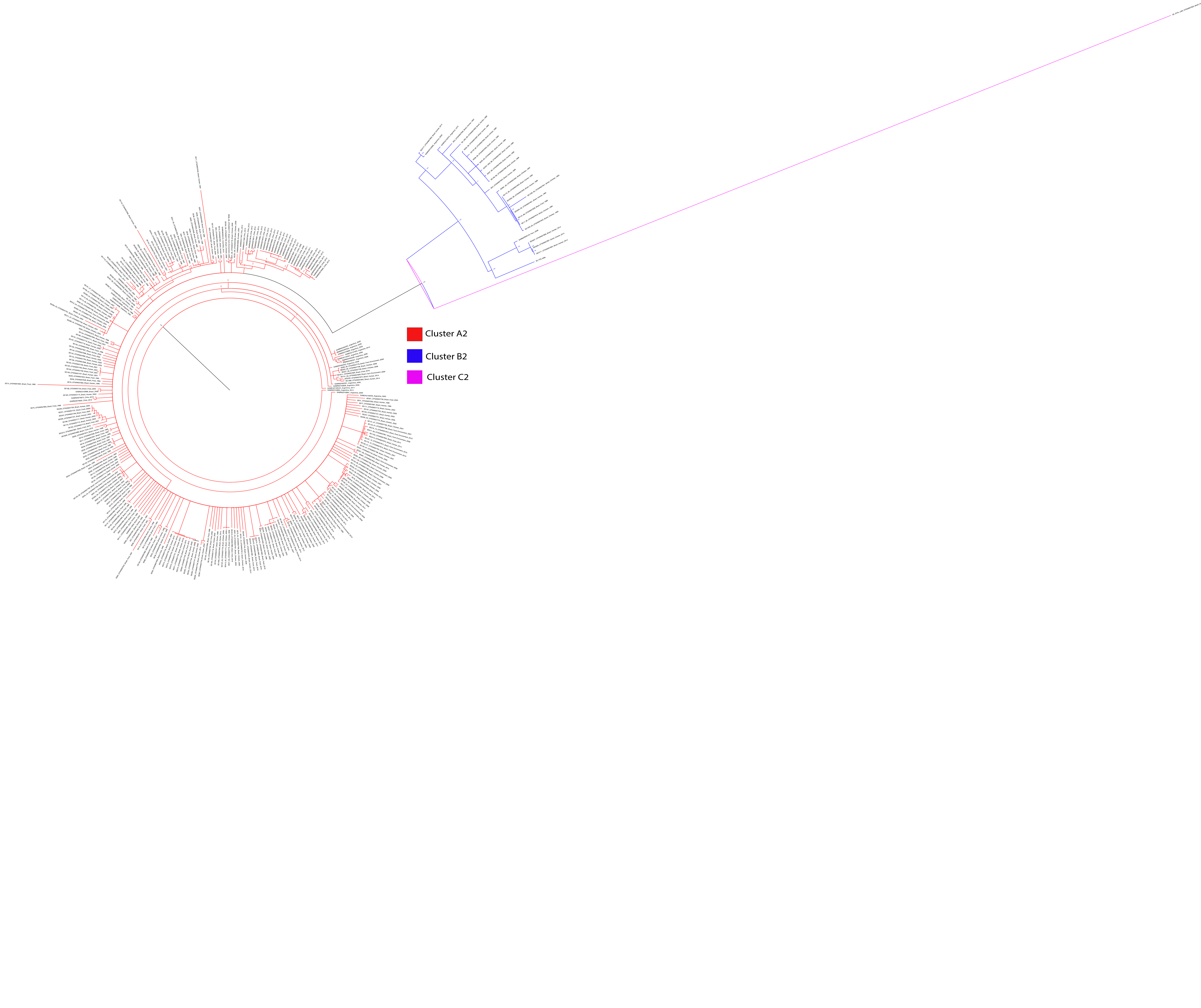
Cluster A2
Cluster B2
Cluster C2
