# Supplementary material for: Changing of the Genomic Pattern of Salmonella Enteritidis Strains Isolated in Brazil Over a 48 year-period revealed by Whole Genome SNP Analyses
Source: Sci Rep. 2018 Jul 11;8:10478. doi: 10.1038/s41598-018-28844-6 (PMC6041339; doi:10.1038/s41598-018-28844-6)
Supplement: Supplementary file 2 — Supplementary Information [file 41598_2018_28844_MOESM2_ESM.zip]

**Title: Changing of the Genomic Pattern of *Salmonella* Enteritidis Strains Isolated in Brazil Over a 48 year-period revealed by Whole Genome SNP Analyses**

**Campioni, Fábio^1^; Cao, Guojie^2^; Kastanis, George^2^; Janies, Daniel A.^3^ Bergamini, Alzira Maria Morato^4^; Rodrigues, Dália dos Prazeres^5^; Stones, Robert^6^; Brown, Eric^2^; Allard, Marc W.^2*^; Falcão, Juliana Pfrimer^1*^**

^1^ – Departamento de Análises Clínicas, Toxicológicas e Bromatológicas – Faculdade de Ciências Farmacêuticas de Ribeirão Preto, Universidade de São Paulo, Av. do Café s/ nº, Ribeirão Preto, SP Brasil.

^2^ – Division of Microbiology, Center for Food Safety and Applied Nutrition, U.S. Food and Drug Administration, College Park, Maryland, United States of America

^3^ – Department of Bioinformatics and Genomics, The University of North Carolina at Charlotte, 9201 University City Blvd, Charlotte, NC 28223, United States of America

^4^ – Instituto Adolfo Lutz de Ribeirão Preto, Rua Minas 877, Ribeirão Preto, SP, Brasil.

^5^– Laboratório de Enterobactérias, FIOCRUZ/Fundação Instituto Oswaldo Cruz, Avenida Brasil, 4365, Pavilhão Rocha Lima, 3°andar, Manguinhos, Rio de Janeiro, RJ, Brasil.

^6^- Newcastle University, Newcastle upon Tyne, United Kingdom.

**Corresponding authors***:

Juliana Pfrimer Falcão

Phone.: + 55-16-36024896; FAX: + 55-16-36024725

Email: [jufalcao@fcfrp.usp.br](mailto:jufalcao@fcfrp.usp.br)

Marc Allard

Phone: +1-240-402-2941

E-mail: [marc.allard@fda.hhs.gov](mailto:marc.allard@fda.hhs.gov)

----------------------------------------------

STRUCTURE by Pritchard, Stephens and Donnelly (2000)

and Falush, Stephens and Pritchard (2003)

Code by Pritchard, Falush and Hubisz

Version 2.3.4 (Jul 2012)

Run parameters:

256 individuals

150322 loci

2 populations assumed

100 Burn-in period

200 Reps

--------------------------------------------

Overall proportion of membership of the

sample in each of the 2 clusters

Inferred Clusters

1	2

0.126 0.874

--------------------------------------------

Allele-freq. divergence among pops (Net nucleotide distance),

computed using point estimates of P.

1	2

1	-	0.0003

2	0.0003	-

Average distances (expected heterozygosity) between individuals in same cluster:

cluster 1 : 0.4152

cluster 2 : 0.4147

--------------------------------------------

Estimated Ln Prob of Data	= -574728.9

Mean value of ln likelihood = -450243.0

Variance of ln likelihood	= 248971.8

Mean value of alpha	= 0.0858

Mean value of Fst_1	= 0.0102

Mean value of Fst_2	= 0.0102

Inferred ancestry of individuals:

Label (%Miss) : Inferred clusters

1 SE_05_08	(93)	: 0.000 1.000

2	SE_10	(95)	: 0.005 0.995

3	SE_100	(95)	: 0.002 0.998

4 SE_102_06	(96)	: 0.000 1.000

5 SE_103_89	(93)	: 0.999 0.001

6 SE_107_09	(95)	: 0.000 1.000

7 SE_108_89	(91)	: 1.000 0.000

8	SE_11	(95)	: 0.020 0.980

9 SE_110_06	(96)	: 0.006 0.994

10 SE_112_06	(96)	: 0.001 0.999

11 SE_113_09	(95)	: 0.007 0.993

12	SE_114	(95)	: 0.003 0.997

13	SE_115	(95)	: 0.002 0.998

14	SE_116	(95)	: 0.003 0.997

15	SE_117	(95)	: 0.023 0.977

16	SE_119	(95)	: 0.324 0.676

17 SE_11_09	(95)	: 0.004 0.996

18	SE_12	(95)	: 0.014 0.986

19	SE_120	(93)	: 0.001 0.999

20	SE_124	(94)	: 0.007 0.993

21	SE_125	(93)	: 0.010 0.990

22	SE_126	(95)	: 0.001 0.999

23	SE_127	(95)	: 0.016 0.984

24	SE_128	(95)	: 0.018 0.982

25	SE_129	(96)	: 0.008 0.992

26 SE_129_08	(95)	: 0.002 0.998

27	SE_13	(95)	: 0.001 0.999

28	SE_130	(96)	: 0.003 0.997

29	SE_131	(96)	: 0.007 0.993

30 SE_131_07	(95)	: 0.002 0.998

31	SE_132	(96)	: 0.002 0.998

32 SE_1328_93	(93)	: 1.000 0.000

33 SE_1329_93	(93)	: 1.000 0.000

34	SE_133	(96)	: 0.004 0.996

35 SE_133_08	(91)	: 0.018 0.982

36	SE_134	(95)	: 0.002 0.998

37	SE_135	(95)	: 0.006 0.994

38 SE_1356	(95)	: 0.002 0.998

39 SE_135_07	(96)	: 0.001 0.999

40	SE_136	(95)	: 0.005 0.995

41	SE_139	(96)	: 0.000 1.000

42 SE_1396	(94)	: 0.001 0.999

43 SE_139_07	(96)	: 0.000 1.000

44 SE_141_09	(94)	: 0.012 0.988

45	SE_142	(95)	: 0.000 1.000

46	SE_143	(95)	: 0.001 0.999

47 SE_143_07	(96)	: 0.011 0.989

48	SE_144	(95)	: 0.007 0.993

49 SE_144_88	(93)	: 0.999 0.001

50	SE_147	(95)	: 0.009 0.991

51	SE_15	(95)	: 0.015 0.985

52	SE_150	(95)	: 0.002 0.998

53 SE_150_06	(95)	: 0.016 0.984

54	SE_151	(95)	: 0.021 0.979

55	SE_153	(95)	: 0.012 0.988

56	SE_154	(95)	: 0.012 0.988

57 SE_1551	(76)	: 0.477 0.523

58	SE_156	(96)	: 0.003 0.997

59	SE_158	(95)	: 0.000 1.000

60	SE_16	(95)	: 0.001 0.999

61	SE_162	(89)	: 0.000 1.000

62	SE_164	(95)	: 0.002 0.998

63	SE_165	(95)	: 0.017 0.983

64 SE_165_09	(94)	: 0.004 0.996

65	SE_168	(95)	: 0.007 0.993

66	SE_17	(95)	: 0.000 1.000

67	SE_171	(95)	: 0.002 0.998

68 SE_1719	(92)	: 0.019 0.981

69 SE_172_85	(93)	: 0.999 0.001

70	SE_173	(89)	: 0.997 0.003

71 SE_1733	(95)	: 0.004 0.996

72 SE_1794	(92)	: 0.002 0.998

73 SE_179_10	(95)	: 0.000 1.000

74 SE_17_80	(93)	: 1.000 0.000

75	SE_18	(95)	: 0.000 1.000

76	SE_181	(95)	: 0.003 0.997

77	SE_183	(92)	: 0.050 0.950

78	SE_185	(95)	: 0.005 0.995

79	SE_186	(96)	: 0.004 0.996

80	SE_187	(95)	: 0.009 0.991

81 SE_189_07	(95)	: 0.017 0.983

82	SE_191	(96)	: 0.000 1.000

83 SE_191_07	(96)	: 0.002 0.998

84 SE_191_10	(88)	: 0.002 0.998

85	SE_192	(95)	: 0.001 0.999

86	SE_193	(92)	: 0.003 0.997

87 SE_1931	(94)	: 0.008 0.992

88	SE_197	(95)	: 0.004 0.996

89 SE_197_10	(95)	: 0.005 0.995

90	SE_198	(92)	: 0.002 0.998

91	SE_199	(95)	: 0.008 0.992

92 SE_199_07	(96)	: 0.001 0.999

93	SE_200	(95)	: 0.000 1.000

94	SE_201	(95)	: 0.009 0.991

95 SE_203_08	(95)	: 0.001 0.999

96	SE_206	(89)	: 0.598 0.402

97	SE_211	(96)	: 0.002 0.998

98	SE_214	(95)	: 0.000 1.000

99 SE_217_07	(96)	: 0.008 0.992

100 SE_217_09	(95)	: 0.003 0.997

101 SE_2198	(84)	: 0.043 0.957

102 SE_219_10	(94)	: 0.001 0.999

103 SE_21_09	(94)	: 0.010 0.990

104	SE_22	(95)	: 0.010 0.990

105 SE_221_2013	(92)	: 0.007 0.993

106	SE_223	(95)	: 0.000 1.000

107 SE_2232	(95)	: 0.008 0.992

108 SE_2252	(92)	: 0.002 0.998

109 SE_226_05	(96)	: 0.003 0.997

110	SE_227	(95)	: 0.001 0.999

111 SE_227_08	(95)	: 0.012 0.988

112 SE_229_09	(95)	: 0.004 0.996

113	SE_23	(95)	: 0.001 0.999

114	SE_230	(95)	: 0.000 1.000

115	SE_231	(95)	: 0.012 0.988

116	SE_233	(95)	: 0.002 0.998

117	SE_234	(95)	: 0.026 0.974

118	SE_239	(95)	: 0.001 0.999

119	SE_241	(95)	: 0.007 0.993

120	SE_242	(95)	: 0.000 1.000

121	SE_244	(95)	: 0.006 0.994

122	SE_246	(95)	: 0.003 0.997

123	SE_247	(95)	: 0.002 0.998

124	SE_249	(95)	: 0.005 0.995

125	SE_251	(95)	: 0.006 0.994

126	SE_252	(92)	: 0.010 0.990

127	SE_253	(95)	: 0.000 1.000

128	SE_255	(95)	: 0.002 0.998

129 SE_255_07	(95)	: 0.010 0.990

130 SE_257_08	(95)	: 0.009 0.991

131	SE_260	(95)	: 0.004 0.996

132	SE_263	(95)	: 0.005 0.995

133 SE_2639_68	(94)	: 0.001 0.999

134	SE_267	(95)	: 0.007 0.993

135	SE_269	(95)	: 0.000 1.000

136	SE_27	(95)	: 0.003 0.997

137	SE_270	(95)	: 0.003 0.997

138	SE_271	(95)	: 0.033 0.967

139	SE_272	(95)	: 0.012 0.988

140	SE_273	(95)	: 0.006 0.994

141	SE_274	(95)	: 0.002 0.998

142 SE_275_05	(96)	: 0.002 0.998

143 SE_27_06	(95)	: 0.001 0.999

144 SE_27_08	(95)	: 0.000 1.000

145	SE_28	(95)	: 0.001 0.999

146 SE_2818	(94)	: 0.003 0.997

147 SE_2819	(95)	: 0.000 1.000

148 SE_2829	(89)	: 0.999 0.001

149	SE_285	(86)	: 0.587 0.413

150	SE_29	(96)	: 0.003 0.997

151 SE_294_04	(95)	: 0.014 0.986

152 SE_296_04	(95)	: 0.025 0.975

153 SE_298_04	(95)	: 0.002 0.998

154	SE_30	(96)	: 0.000 1.000

155 SE_302_04	(96)	: 0.001 0.999

156 SE_304_04	(95)	: 0.001 0.999

157 SE_312_04	(95)	: 0.001 0.999

158 SE_3171	(93)	: 0.996 0.004

159 SE_3177	(93)	: 0.999 0.001

160 SE_317_08	(95)	: 0.017 0.983

161 SE_31_06	(96)	: 0.002 0.998

162	SE_32	(96)	: 0.003 0.997

163 SE_3207	(93)	: 1.000 0.000

164 SE_3209	(93)	: 0.998 0.002

165 SE_3243_69	(92)	: 0.006 0.994

166 SE_326_90	(87)	: 0.996 0.004

167 SE_327_08	(95)	: 0.008 0.992

168 SE_327_90	(88)	: 1.000 0.000

169	SE_33	(96)	: 0.010 0.990

170 SE_331_08	(95)	: 0.001 0.999

171 SE_332_05	(93)	: 0.001 0.999

172 SE_337_R65_	(93)	: 0.998 0.002

173 SE_3472	(92)	: 0.001 0.999

174 SE_3473	(92)	: 0.005 0.995

175 SE_3474	(92)	: 0.000 1.000

176 SE_3475	(92)	: 0.011 0.989

177 SE_348_05	(96)	: 0.000 1.000

178 SE_3494_82	(93)	: 0.996 0.004

179 SE_34_04	(93)	: 0.007 0.993

180	SE_35	(96)	: 0.001 0.999

181 SE_355_06	(95)	: 0.001 0.999

182	SE_36	(95)	: 0.001 0.999

183	SE_37	(96)	: 0.007 0.993

184 SE_37_09	(95)	: 0.009 0.991

185	SE_38	(95)	: 0.004 0.996

186 SE_3800	(84)	: 0.977 0.023

187 SE_387_06	(95)	: 0.000 1.000

188	SE_39	(96)	: 0.005 0.995

189	SE_4	(72)	: 0.996 0.004

190	SE_41	(88)	: 0.998 0.002

191 SE_413_05	(95)	: 0.008 0.992

192 SE_4245	(95)	: 0.028 0.972

193 SE_4258_86	(93)	: 0.999 0.001

194 SE_4280	(95)	: 0.001 0.999

195	SE_43	(96)	: 0.009 0.991

196 SE_4451	(95)	: 0.019 0.981

197 SE_4501	(94)	: 0.002 0.998

198 SE_4509	(93)	: 0.001 0.999

199 SE_4510	(94)	: 0.002 0.998

200 SE_4511	(95)	: 0.003 0.997

201 SE_4512	(95)	: 0.001 0.999

202	SE_460	(92)	: 0.000 1.000

203	SE_48	(96)	: 0.006 0.994

204	SE_5	(93)	: 0.999 0.001

205	SE_50	(96)	: 0.000 1.000

206 SE_5012	(95)	: 0.004 0.996

207 SE_5346	(92)	: 0.003 0.997

208 SE_5413	(95)	: 0.001 0.999

209 SE_5535	(95)	: 0.000 1.000

210 SE_55_04	(92)	: 0.002 0.998

211	SE_56	(96)	: 0.028 0.972

212 SE_569_81	(95)	: 0.002 0.998

213	SE_59	(96)	: 0.004 0.996

214	SE_6	(95)	: 0.000 1.000

215	SE_60	(95)	: 0.004 0.996

216	SE_66	(96)	: 0.002 0.998

217	SE_67	(95)	: 0.014 0.986

218 SE_67_04	(93)	: 0.000 1.000

219 SE_67_05	(96)	: 0.004 0.996

220	SE_68	(96)	: 0.004 0.996

221 SE_69_04	(93)	: 0.024 0.976

222 SE_69_05	(95)	: 0.020 0.980

223	SE_7	(95)	: 0.002 0.998

224	SE_70	(96)	: 0.006 0.994

225	SE_73	(96)	: 0.004 0.996

226 SE_73_05	(96)	: 0.000 1.000

227	SE_74	(95)	: 0.014 0.986

228	SE_75	(96)	: 0.024 0.976

229	SE_76	(95)	: 0.009 0.991

230 SE_76_2013	(95)	: 0.002 0.998

231	SE_77	(95)	: 0.000 1.000

232	SE_795	(95)	: 0.002 0.998

233	SE_8	(95)	: 0.000 1.000

234	SE_80	(95)	: 0.002 0.998

235	SE_801	(94)	: 0.007 0.993

236	SE_84	(96)	: 0.014 0.986

237	SE_85	(95)	: 0.023 0.977

238	SE_86	(95)	: 0.001 0.999

239	SE_87	(96)	: 0.001 0.999

240	SE_88	(95)	: 0.007 0.993

241 SE_881_93	(93)	: 1.000 0.000

242 SE_88_06	(95)	: 0.006 0.994

243	SE_89	(95)	: 0.001 0.999

244 SE_896_93	(95)	: 0.000 1.000

245	SE_9	(95)	: 0.004 0.996

246 SE_92_89	(88)	: 0.998 0.002

247	SE_94	(95)	: 0.006 0.994

248 SE_94_89	(95)	: 0.003 0.997

249	SE_96	(96)	: 0.006 0.994

250	SE_961	(95)	: 0.014 0.986

251 SE_96_89	(93)	: 0.999 0.001

252 SE_97_89	(88)	: 0.997 0.003

253 SE_98_06	(95)	: 0.009 0.991

254 SE_98_89	(93)	: 1.000 0.000

255 SE_A65_88	(93)	: 0.995 0.005

256 SE_STAL_LMS	(86)	: 0.999 0.001

Estimated Allele Frequencies in each cluster

First column gives estimated ancestral frequencies

Locus 1 :

2 alleles

98.8% missing data

3	(0.211) 0.203 0.216

2	(0.789) 0.797 0.784

Locus 2 :

4 alleles

94.5% missing data

1	(0.336) 0.324 0.340

2	(0.214) 0.220 0.214

3	(0.366) 0.364 0.374

4	(0.083) 0.092 0.072

Locus 3 :

2 alleles

24.2% missing data

3	(0.980) 0.974 0.990

1	(0.020) 0.026 0.010

Locus 4 :

3 alleles

98.0% missing data

1	(0.353) 0.345 0.360

3	(0.349) 0.350 0.337

2	(0.298) 0.305 0.303

Locus 5 :

3 alleles

97.3% missing data

1	(0.616) 0.608 0.625

2	(0.172) 0.173 0.172

4	(0.212) 0.219 0.202

Locus 6 :

4 alleles

94.5% missing data

1	(0.731) 0.735 0.728

4	(0.042) 0.041 0.046

3	(0.088) 0.086 0.089

2	(0.139) 0.138 0.137

Locus 7 :

3 alleles

98.4% missing data

2	(0.340) 0.343 0.345

1	(0.304) 0.306 0.300

3	(0.357) 0.351 0.355

Locus 8 :

3 alleles

96.1% missing data

1	(0.792) 0.795 0.790

2	(0.114) 0.110 0.117

4	(0.094) 0.094 0.093

Locus 9 :

4 alleles

94.5% missing data

4	(0.561) 0.589 0.552

3	(0.202) 0.183 0.215

2	(0.097) 0.089 0.098

1	(0.141) 0.140 0.135

Locus 10 :

2 alleles

98.0% missing data

4	(0.475) 0.477 0.468

1	(0.525) 0.523 0.532

Locus 11 :

2 alleles

97.3% missing data

1	(0.582) 0.574 0.604

4	(0.418) 0.426 0.396

Locus 12 :

3 alleles

77.7% missing data

1	(0.916) 0.916 0.929

2	(0.055) 0.058 0.043

4	(0.029) 0.026 0.028

Locus 13 :

3 alleles

84.8% missing data

1	(0.645) 0.662 0.627

2	(0.316) 0.302 0.338

4	(0.038) 0.036 0.035

Locus 14 :

2 alleles

8.2% missing data

1	(0.987) 0.981 0.996

2	(0.013) 0.019 0.004

Locus 15 :

2 alleles

98.8% missing data

4	(0.541) 0.544 0.540

2	(0.459) 0.456 0.460

Locus 16 :

2 alleles

98.4% missing data

3	(0.554) 0.553 0.553

1	(0.446) 0.447 0.447

Locus 17 :

2 alleles

98.8% missing data

3	(0.700) 0.696 0.701

2	(0.300) 0.304 0.299

Locus 18 :

2 alleles

90.2% missing data

1	(0.743) 0.747 0.749

2	(0.257) 0.253 0.251

Locus 19 :

3 alleles

96.9% missing data

2	(0.247) 0.251 0.236

4	(0.524) 0.522 0.539

1	(0.229) 0.227 0.224

Locus 20 :

2 alleles

97.7% missing data

1	(0.694) 0.696 0.698

4	(0.306) 0.304 0.302

Locus 21 :

2 alleles

98.8% missing data

2	(0.548) 0.544 0.554

1	(0.452) 0.456 0.446

Locus 22 :

3 alleles

96.5% missing data

2	(0.245) 0.245 0.234

4	(0.623) 0.612 0.642

1	(0.132) 0.142 0.124

Locus 23 :

2 alleles

99.2% missing data

4	(0.742) 0.744 0.741

1	(0.258) 0.256 0.259

Locus 24 :

2 alleles

99.2% missing data

4	(0.768) 0.766 0.760

3	(0.232) 0.234 0.240

Locus 25 :

2 alleles

99.2% missing data

1	(0.749) 0.748 0.749

4	(0.251) 0.252 0.251

Locus 26 :

2 alleles

99.2% missing data

3	(0.504) 0.494 0.512

2	(0.496) 0.506 0.488

Locus 27 :

3 alleles

98.8% missing data

1	(0.343) 0.342 0.345

3	(0.382) 0.380 0.378

4	(0.275) 0.278 0.277

Locus 28 :

2 alleles

99.2% missing data

3	(0.134) 0.129 0.142

1	(0.866) 0.871 0.858

Locus 29 :

2 alleles

98.8% missing data

4	(0.714) 0.721 0.712

1	(0.286) 0.279 0.288

Locus 30 :

4 alleles

93.8% missing data

3	(0.294) 0.295 0.292

2	(0.043) 0.043 0.043

4	(0.570) 0.578 0.571

1	(0.092) 0.084 0.093

Locus 31 :

2 alleles

97.7% missing data

2	(0.282) 0.278 0.287

1	(0.718) 0.722 0.713

Locus 32 :

4 alleles

98.0% missing data

3	(0.243) 0.246 0.245

4	(0.294) 0.296 0.298

2	(0.288) 0.285 0.281

1	(0.175) 0.173 0.176

Locus 33 :

2 alleles

99.2% missing data

1	(0.622) 0.625 0.618

3	(0.378) 0.375 0.382

Locus 34 :

2 alleles

99.2% missing data

4	(0.429) 0.432 0.428

3	(0.571) 0.568 0.572

Locus 35 :

2 alleles

98.0% missing data

3	(0.448) 0.455 0.447

1	(0.552) 0.545 0.553

Locus 36 :

3 alleles

98.0% missing data

3	(0.382) 0.378 0.382

4	(0.302) 0.301 0.302

1	(0.317) 0.320 0.316

Locus 37 :

2 alleles

99.2% missing data

3	(0.270) 0.262 0.277

4	(0.730) 0.738 0.723

Locus 38 :

2 alleles

98.8% missing data

1	(0.331) 0.334 0.333

3	(0.669) 0.666 0.667

Locus 39 :

3 alleles

98.8% missing data

3	(0.361) 0.357 0.364

4	(0.181) 0.183 0.179

2	(0.458) 0.460 0.457

Locus 40 :

2 alleles

98.8% missing data

4	(0.290) 0.291 0.293

1	(0.710) 0.709 0.707

Locus 41 :

2 alleles

98.8% missing data

1	(0.447) 0.439 0.454

2	(0.553) 0.561 0.546

Locus 42 :

3 alleles

90.2% missing data

1	(0.842) 0.842 0.852

2	(0.078) 0.083 0.071

4	(0.080) 0.075 0.077

Locus 43 :

2 alleles

99.2% missing data

3	(0.358) 0.355 0.362

2	(0.642) 0.645 0.638

Locus 44 :

3 alleles

98.4% missing data

1	(0.624) 0.613 0.632

2	(0.147) 0.152 0.141

3	(0.229) 0.235 0.227

Locus 45 :

2 alleles

98.8% missing data

3	(0.510) 0.514 0.499

1	(0.490) 0.486 0.501

Locus 46 :

2 alleles

99.2% missing data

4	(0.323) 0.321 0.328

1	(0.677) 0.679 0.672

Locus 47 :

3 alleles

87.9% missing data

1	(0.627) 0.634 0.630

2	(0.267) 0.264 0.261

4	(0.106) 0.103 0.109

Locus 48 :

2 alleles

97.7% missing data

2	(0.578) 0.578 0.591

4	(0.422) 0.422 0.409

Locus 49 :

2 alleles

98.8% missing data

2	(0.564) 0.565 0.563

1	(0.436) 0.435 0.437

Locus 50 :

2 alleles

92.6% missing data

1	(0.790) 0.793 0.796

2	(0.210) 0.207 0.204

Locus 51 :

3 alleles

87.5% missing data

1	(0.693) 0.712 0.677

3	(0.053) 0.041 0.063

2	(0.254) 0.247 0.260

Locus 52 :

2 alleles

95.3% missing data

1	(0.199) 0.190 0.207

4	(0.801) 0.810 0.793

Locus 53 :

4 alleles

98.4% missing data

2	(0.177) 0.172 0.173

3	(0.295) 0.301 0.292

1	(0.212) 0.212 0.215

4	(0.316) 0.314 0.320

Locus 54 :

2 alleles

99.2% missing data

4	(0.383) 0.376 0.389

3	(0.617) 0.624 0.611

Locus 55 :

2 alleles

99.2% missing data

4	(0.532) 0.534 0.526

2	(0.468) 0.466 0.474

Locus 56 :

2 alleles

99.2% missing data

1	(0.598) 0.602 0.600

2	(0.402) 0.398 0.400

Locus 57 :

2 alleles

98.8% missing data

3	(0.692) 0.690 0.692

4	(0.308) 0.310 0.308

Locus 58 :

2 alleles

96.9% missing data

2	(0.749) 0.753 0.753

3	(0.251) 0.247 0.247

Locus 59 :

2 alleles

99.2% missing data

2	(0.605) 0.598 0.612

1	(0.395) 0.402 0.388

Locus 60 :

2 alleles

98.8% missing data

1	(0.477) 0.481 0.485

2	(0.523) 0.519 0.515

Locus 61 :

3 alleles

98.0% missing data

2	(0.296) 0.309 0.286

1	(0.264) 0.268 0.257

4	(0.439) 0.423 0.457

Locus 62 :

2 alleles

98.4% missing data

2	(0.726) 0.724 0.720

3	(0.274) 0.276 0.280

Locus 63 :

4 alleles

98.4% missing data

4	(0.304) 0.294 0.304

3	(0.362) 0.367 0.362

1	(0.191) 0.189 0.198

2	(0.143) 0.151 0.137

Locus 64 :

2 alleles

99.2% missing data

2	(0.830) 0.831 0.829

4	(0.170) 0.169 0.171

Locus 65 :

3 alleles

97.7% missing data

1	(0.757) 0.762 0.747

2	(0.157) 0.154 0.164

3	(0.086) 0.084 0.089

Locus 66 :

2 alleles

98.8% missing data

3	(0.533) 0.536 0.525

2	(0.467) 0.464 0.475

Locus 67 :

3 alleles

98.8% missing data

1	(0.288) 0.284 0.292

2	(0.484) 0.483 0.478

4	(0.228) 0.233 0.231

Locus 68 :

2 alleles

99.2% missing data

1	(0.379) 0.378 0.386

2	(0.621) 0.622 0.614

Locus 69 :

3 alleles

97.3% missing data

1	(0.319) 0.322 0.315

2	(0.136) 0.134 0.134

4	(0.545) 0.544 0.550

Locus 70 :

2 alleles

66.0% missing data

1	(0.969) 0.977 0.968

4	(0.031) 0.023 0.032

Locus 71 :

2 alleles

0.8% missing data

2	(0.981) 0.986 0.988

3	(0.019) 0.014 0.012

Locus 72 :

4 alleles

97.3% missing data

1	(0.203) 0.200 0.204

4	(0.166) 0.165 0.168

3	(0.483) 0.496 0.476

2	(0.148) 0.140 0.152

Locus 73 :

2 alleles

99.2% missing data

3	(0.691) 0.696 0.686

4	(0.309) 0.304 0.314

Locus 74 :

2 alleles

94.9% missing data

1	(0.219) 0.212 0.230

3	(0.781) 0.788 0.770

Locus 75 :

2 alleles

99.2% missing data

3	(0.425) 0.419 0.430

1	(0.575) 0.581 0.570

Locus 76 :

2 alleles

99.2% missing data

2	(0.480) 0.476 0.484

1	(0.520) 0.524 0.516

Locus 77 :

2 alleles

99.2% missing data

4	(0.531) 0.531 0.527

2	(0.469) 0.469 0.473

Locus 78 :

2 alleles

99.2% missing data

4	(0.338) 0.341 0.340

3	(0.662) 0.659 0.660

Locus 79 :

2 alleles

99.2% missing data

4	(0.764) 0.759 0.765

1	(0.236) 0.241 0.235

Locus 80 :

2 alleles

98.8% missing data

3	(0.404) 0.401 0.406

1	(0.596) 0.599 0.594

Locus 81 :

2 alleles

99.2% missing data

1	(0.534) 0.534 0.533

3	(0.466) 0.466 0.467

Locus 82 :

2 alleles

99.2% missing data

4	(0.275) 0.275 0.276

1	(0.725) 0.725 0.724

Locus 83 :

2 alleles

98.8% missing data

1	(0.337) 0.334 0.339

3	(0.663) 0.666 0.661

Locus 84 :

2 alleles

99.2% missing data

3	(0.307) 0.307 0.313

4	(0.693) 0.693 0.687

Locus 85 :

2 alleles

98.8% missing data

1	(0.732) 0.729 0.734

2	(0.268) 0.271 0.266

Locus 86 :

2 alleles

99.2% missing data

1	(0.634) 0.642 0.626

4	(0.366) 0.358 0.374

Locus 87 :

2 alleles

99.2% missing data

1	(0.369) 0.365 0.367

3	(0.631) 0.635 0.633

Locus 88 :

3 alleles

95.7% missing data

2	(0.728) 0.745 0.723

1	(0.113) 0.107 0.116

4	(0.159) 0.148 0.161

Locus 89 :

2 alleles

99.2% missing data

1	(0.566) 0.557 0.576

3	(0.434) 0.443 0.424

Locus 90 :

2 alleles

98.8% missing data

3	(0.254) 0.249 0.257

1	(0.746) 0.751 0.743

Locus 91 :

2 alleles

99.2% missing data

3	(0.804) 0.800 0.809

4	(0.196) 0.200 0.191

Locus 92 :

2 alleles

99.2% missing data

2	(0.560) 0.561 0.558

1	(0.440) 0.439 0.442

Locus 93 :

2 alleles

98.8% missing data

2	(0.689) 0.694 0.686

4	(0.311) 0.306 0.314

Locus 94 :

2 alleles

98.4% missing data

1	(0.612) 0.616 0.609

4	(0.388) 0.384 0.391

Locus 95 :

2 alleles

94.9% missing data

3	(0.846) 0.847 0.850

4	(0.154) 0.153 0.150

Locus 96 :

2 alleles

99.2% missing data

1	(0.709) 0.707 0.709

3	(0.291) 0.293 0.291

Locus 97 :

2 alleles

99.2% missing data

1	(0.555) 0.548 0.561

2	(0.445) 0.452 0.439

Locus 98 :

2 alleles

77.3% missing data

1	(0.823) 0.836 0.821

2	(0.177) 0.164 0.179

Locus 99 :

2 alleles

96.1% missing data

1	(0.659) 0.662 0.665

2	(0.341) 0.338 0.335

Locus 100 :

3 alleles

98.4% missing data

1	(0.435) 0.432 0.438

4	(0.366) 0.361 0.370

3	(0.199) 0.207 0.192

Locus 101 :

2 alleles

92.2% missing data

1	(0.142) 0.148 0.134

2	(0.858) 0.852 0.866

Locus 102 :

2 alleles

96.1% missing data

3	(0.693) 0.716 0.672

1	(0.307) 0.284 0.328

Locus 103 :

2 alleles

99.2% missing data

1	(0.419) 0.410 0.427

4	(0.581) 0.590 0.573

Locus 104 :

2 alleles

92.6% missing data

3	(0.900) 0.899 0.907

1	(0.100) 0.101 0.093

Locus 105 :

2 alleles

98.8% missing data

1	(0.806) 0.809 0.807

3	(0.194) 0.191 0.193

Locus 106 :

2 alleles

98.4% missing data

3	(0.411) 0.410 0.411

1	(0.589) 0.590 0.589

Locus 107 :

3 alleles

94.5% missing data

3	(0.320) 0.316 0.310

1	(0.465) 0.468 0.476

2	(0.216) 0.217 0.214

Locus 108 :

3 alleles

94.9% missing data

1	(0.206) 0.209 0.218

2	(0.585) 0.587 0.583

3	(0.209) 0.204 0.199

Locus 109 :

2 alleles

98.8% missing data

2	(0.619) 0.625 0.620

4	(0.381) 0.375 0.380

Locus 110 :

2 alleles

98.8% missing data

1	(0.554) 0.566 0.545

4	(0.446) 0.434 0.455

Locus 111 :

2 alleles

98.4% missing data

3	(0.849) 0.849 0.852

1	(0.151) 0.151 0.148

Locus 112 :

2 alleles

90.2% missing data

4	(0.873) 0.887 0.855

1	(0.127) 0.113 0.145

Locus 113 :

2 alleles

0.0% missing data

2	(0.991) 0.993 0.995

1	(0.009) 0.007 0.005

Locus 114 :

3 alleles

97.3% missing data

1	(0.328) 0.340 0.324

2	(0.344) 0.344 0.345

4	(0.328) 0.316 0.331

Locus 115 :

2 alleles

99.2% missing data

2	(0.512) 0.508 0.516

1	(0.488) 0.492 0.484

Locus 116 :

3 alleles

90.2% missing data

1	(0.638) 0.649 0.647

2	(0.291) 0.285 0.288

4	(0.071) 0.066 0.066

Locus 117 :

2 alleles

99.2% missing data

4	(0.499) 0.491 0.500

1	(0.501) 0.509 0.500

Locus 118 :

3 alleles

89.1% missing data

1	(0.760) 0.755 0.781

2	(0.160) 0.168 0.146

3	(0.080) 0.077 0.072

Locus 119 :

2 alleles

95.3% missing data

1	(0.521) 0.543 0.502

2	(0.479) 0.457 0.498

Locus 120 :

2 alleles

84.4% missing data

1	(0.955) 0.959 0.957

3	(0.045) 0.041 0.043

Locus 121 :

2 alleles

99.2% missing data

3	(0.625) 0.621 0.625

4	(0.375) 0.379 0.375

Locus 122 :

2 alleles

99.2% missing data

4	(0.283) 0.278 0.282

1	(0.717) 0.722 0.718

Locus 123 :

2 alleles

8.6% missing data

4	(0.986) 0.979 0.996

1	(0.014) 0.021 0.004

Locus 124 :

2 alleles

96.1% missing data

1	(0.762) 0.757 0.770

4	(0.238) 0.243 0.230

Locus 125 :

2 alleles

94.9% missing data

2	(0.751) 0.762 0.747

1	(0.249) 0.238 0.253

Locus 126 :

2 alleles

98.8% missing data

4	(0.565) 0.565 0.565

3	(0.435) 0.435 0.435

Locus 127 :

2 alleles

99.2% missing data

4	(0.556) 0.553 0.553

3	(0.444) 0.447 0.447

Locus 128 :

2 alleles

99.2% missing data

3	(0.461) 0.459 0.466

4	(0.539) 0.541 0.534

Locus 129 :

2 alleles

99.2% missing data

3	(0.274) 0.283 0.273

2	(0.726) 0.717 0.727

Locus 130 :

2 alleles

99.2% missing data

2	(0.358) 0.368 0.359

4	(0.642) 0.632 0.641

Locus 131 :

2 alleles

98.8% missing data

4	(0.282) 0.285 0.279

1	(0.718) 0.715 0.721

Locus 132 :

2 alleles

98.8% missing data

3	(0.558) 0.555 0.563

2	(0.442) 0.445 0.437

Locus 133 :

2 alleles

99.2% missing data

1	(0.360) 0.359 0.360

3	(0.640) 0.641 0.640

Locus 134 :

2 alleles

99.2% missing data

3	(0.435) 0.432 0.440

1	(0.565) 0.568 0.560

Locus 135 :

3 alleles

96.9% missing data

2	(0.625) 0.614 0.650

1	(0.206) 0.216 0.191

3	(0.168) 0.170 0.159

Locus 136 :

2 alleles

8.6% missing data

2	(0.984) 0.976 0.995

1	(0.016) 0.024 0.005

Locus 137 :

3 alleles

98.4% missing data

2	(0.401) 0.399 0.408

4	(0.377) 0.379 0.376

3	(0.221) 0.222 0.216

Locus 138 :

2 alleles

88.3% missing data

3	(0.759) 0.803 0.704

2	(0.241) 0.197 0.296

Locus 139 :

3 alleles

89.5% missing data

3	(0.826) 0.858 0.805

4	(0.100) 0.084 0.106

1	(0.074) 0.059 0.089

Locus 140 :

2 alleles

99.2% missing data

1	(0.525) 0.526 0.520

3	(0.475) 0.474 0.480

Locus 141 :

2 alleles

98.8% missing data

2	(0.633) 0.623 0.640

1	(0.367) 0.377 0.360

Locus 142 :

3 alleles

98.8% missing data

4	(0.200) 0.197 0.204

3	(0.384) 0.379 0.380

1	(0.416) 0.424 0.415

Locus 143 :

2 alleles

98.8% missing data

3	(0.559) 0.554 0.564

2	(0.441) 0.446 0.436

Locus 144 :

2 alleles

98.8% missing data

3	(0.530) 0.520 0.533

4	(0.470) 0.480 0.467

Locus 145 :

2 alleles

99.2% missing data

4	(0.542) 0.547 0.537

2	(0.458) 0.453 0.463

Locus 146 :

2 alleles

99.2% missing data

3	(0.318) 0.320 0.317

1	(0.682) 0.680 0.683

Locus 147 :

3 alleles

96.5% missing data

2	(0.636) 0.630 0.652

3	(0.230) 0.231 0.226

1	(0.134) 0.139 0.122

Locus 148 :

2 alleles

99.2% missing data

4	(0.300) 0.298 0.302

3	(0.700) 0.702 0.698

Locus 149 :

2 alleles

99.2% missing data

3	(0.573) 0.567 0.576

2	(0.427) 0.433 0.424

Locus 150 :

3 alleles

96.1% missing data

3	(0.159) 0.153 0.156

4	(0.599) 0.615 0.601

1	(0.242) 0.232 0.243

Locus 151 :

3 alleles

93.4% missing data

1	(0.804) 0.805 0.805

2	(0.120) 0.120 0.123

4	(0.076) 0.075 0.072

Locus 152 :

2 alleles

99.2% missing data

1	(0.211) 0.202 0.218

3	(0.789) 0.798 0.782

Locus 153 :

2 alleles

98.8% missing data

2	(0.580) 0.578 0.585

3	(0.420) 0.422 0.415

Locus 154 :

3 alleles

98.8% missing data

1	(0.382) 0.378 0.385

3	(0.258) 0.267 0.251

4	(0.360) 0.355 0.365

Locus 155 :

2 alleles

91.8% missing data

2	(0.910) 0.903 0.925

4	(0.090) 0.097 0.075

Locus 156 :

3 alleles

97.7% missing data

4	(0.261) 0.247 0.266

1	(0.172) 0.166 0.178

2	(0.567) 0.587 0.556

Locus 157 :

2 alleles

99.2% missing data

3	(0.488) 0.485 0.493

2	(0.512) 0.515 0.507

Locus 158 :

2 alleles

99.2% missing data

4	(0.595) 0.604 0.592

1	(0.405) 0.396 0.408

Locus 159 :

2 alleles

95.3% missing data

2	(0.817) 0.820 0.825

4	(0.183) 0.180 0.175

Locus 160 :

2 alleles

96.5% missing data

3	(0.624) 0.625 0.615

4	(0.376) 0.375 0.385

Locus 161 :

2 alleles

98.8% missing data

1	(0.357) 0.357 0.355

3	(0.643) 0.643 0.645

Locus 162 :

2 alleles

99.2% missing data

3	(0.369) 0.368 0.367

1	(0.631) 0.632 0.633

Locus 163 :

2 alleles

98.8% missing data

2	(0.255) 0.254 0.253

4	(0.745) 0.746 0.747

Locus 164 :

2 alleles

98.0% missing data

2	(0.300) 0.298 0.301

3	(0.700) 0.702 0.699

Locus 165 :

2 alleles

94.9% missing data

4	(0.870) 0.867 0.877

3	(0.130) 0.133 0.123

Locus 166 :

2 alleles

99.2% missing data

3	(0.435) 0.436 0.432

2	(0.565) 0.564 0.568

Locus 167 :

2 alleles

99.2% missing data

4	(0.301) 0.301 0.303

1	(0.699) 0.699 0.697

Locus 168 :

2 alleles

96.5% missing data

2	(0.908) 0.916 0.902

1	(0.092) 0.084 0.098

Locus 169 :

3 alleles

98.4% missing data

1	(0.329) 0.330 0.334

3	(0.530) 0.519 0.530

2	(0.142) 0.151 0.136

Locus 170 :

2 alleles

99.2% missing data

1	(0.678) 0.675 0.680

2	(0.322) 0.325 0.320

Locus 171 :

2 alleles

86.3% missing data

1	(0.954) 0.944 0.967

3	(0.046) 0.056 0.033

Locus 172 :

2 alleles

99.2% missing data

4	(0.537) 0.542 0.534

3	(0.463) 0.458 0.466

Locus 173 :

2 alleles

99.2% missing data

3	(0.761) 0.769 0.759

2	(0.239) 0.231 0.241

Locus 174 :

3 alleles

91.4% missing data

2	(0.685) 0.660 0.722

4	(0.125) 0.122 0.117

3	(0.189) 0.218 0.161

Locus 175 :

2 alleles

98.0% missing data

2	(0.516) 0.499 0.525

3	(0.484) 0.501 0.475

Locus 176 :

2 alleles

98.8% missing data

1	(0.725) 0.721 0.729

4	(0.275) 0.279 0.271

Locus 177 :

3 alleles

91.4% missing data

2	(0.540) 0.554 0.522

4	(0.194) 0.191 0.198

3	(0.266) 0.255 0.280

Locus 178 :

3 alleles

98.8% missing data

3	(0.256) 0.254 0.262

1	(0.227) 0.227 0.230

4	(0.517) 0.519 0.509

Locus 179 :

2 alleles

98.4% missing data

1	(0.570) 0.584 0.567

3	(0.430) 0.416 0.433

Locus 180 :

3 alleles

98.0% missing data

2	(0.594) 0.589 0.604

3	(0.199) 0.201 0.196

4	(0.207) 0.210 0.199

Locus 181 :

2 alleles

97.7% missing data

1	(0.786) 0.790 0.786

3	(0.214) 0.210 0.214

Locus 182 :

2 alleles

98.8% missing data

3	(0.482) 0.489 0.476

2	(0.518) 0.511 0.524

Locus 183 :

2 alleles

98.8% missing data

1	(0.694) 0.696 0.696

3	(0.306) 0.304 0.304

Locus 184 :

2 alleles

99.2% missing data

2	(0.647) 0.645 0.651

3	(0.353) 0.355 0.349

Locus 185 :

2 alleles

98.8% missing data

4	(0.666) 0.662 0.671

2	(0.334) 0.338 0.329

Locus 186 :

3 alleles

97.3% missing data

1	(0.206) 0.197 0.211

2	(0.670) 0.673 0.670

4	(0.124) 0.130 0.119

Locus 187 :

2 alleles

96.1% missing data

2	(0.762) 0.776 0.760

1	(0.238) 0.224 0.240

Locus 188 :

2 alleles

99.2% missing data

4	(0.689) 0.688 0.683

2	(0.311) 0.312 0.317

Locus 189 :

2 alleles

99.2% missing data

1	(0.262) 0.264 0.253

4	(0.738) 0.736 0.747

Locus 190 :

2 alleles

99.2% missing data

2	(0.621) 0.632 0.611

3	(0.379) 0.368 0.389

Locus 191 :

3 alleles

97.7% missing data

2	(0.436) 0.420 0.461

3	(0.245) 0.253 0.234

4	(0.319) 0.327 0.306

Locus 192 :

2 alleles

99.2% missing data

4	(0.791) 0.793 0.788

2	(0.209) 0.207 0.212

Locus 193 :

2 alleles

98.8% missing data

4	(0.705) 0.704 0.706

2	(0.295) 0.296 0.294

Locus 194 :

3 alleles

98.8% missing data

1	(0.253) 0.249 0.263

2	(0.326) 0.327 0.316

3	(0.420) 0.424 0.421

Locus 195 :

2 alleles

99.2% missing data

2	(0.580) 0.572 0.582

4	(0.420) 0.428 0.418

Locus 196 :

2 alleles

98.8% missing data

3	(0.570) 0.575 0.570

1	(0.430) 0.425 0.430

Locus 197 :

2 alleles

96.1% missing data

3	(0.864) 0.855 0.871

4	(0.136) 0.145 0.129

Locus 198 :

2 alleles

99.2% missing data

1	(0.686) 0.689 0.683

2	(0.314) 0.311 0.317

Locus 199 :

2 alleles

98.8% missing data

3	(0.748) 0.756 0.746

1	(0.252) 0.244 0.254

Locus 200 :

2 alleles

99.2% missing data

4	(0.433) 0.430 0.434

2	(0.567) 0.570 0.566

Locus 201 :

2 alleles

99.2% missing data

2	(0.482) 0.482 0.478

1	(0.518) 0.518 0.522

Locus 202 :

2 alleles

99.2% missing data

4	(0.705) 0.698 0.712

1	(0.295) 0.302 0.288

Locus 203 :

2 alleles

99.2% missing data

1	(0.665) 0.662 0.668

3	(0.335) 0.338 0.332

Locus 204 :

2 alleles

98.8% missing data

2	(0.295) 0.289 0.303

3	(0.705) 0.711 0.697

Locus 205 :

2 alleles

98.8% missing data

4	(0.415) 0.413 0.414

1	(0.585) 0.587 0.586

Locus 206 :

2 alleles

99.2% missing data

1	(0.643) 0.645 0.641

4	(0.357) 0.355 0.359

Locus 207 :

2 alleles

99.2% missing data

1	(0.418) 0.425 0.415

2	(0.582) 0.575 0.585

Locus 208 :

2 alleles

99.2% missing data

3	(0.376) 0.379 0.377

1	(0.624) 0.621 0.623

Locus 209 :

2 alleles

99.2% missing data

1	(0.437) 0.428 0.438

4	(0.563) 0.572 0.562

Locus 210 :

2 alleles

91.0% missing data

1	(0.939) 0.952 0.928

2	(0.061) 0.048 0.072

Locus 211 :

2 alleles

99.2% missing data

3	(0.700) 0.702 0.694

2	(0.300) 0.298 0.306

Locus 212 :

2 alleles

92.2% missing data

1	(0.881) 0.874 0.894

3	(0.119) 0.126 0.106

Locus 213 :

2 alleles

99.2% missing data

2	(0.478) 0.478 0.479

1	(0.522) 0.522 0.521

Locus 214 :

3 alleles

98.0% missing data

3	(0.146) 0.141 0.152

4	(0.493) 0.496 0.492

1	(0.361) 0.364 0.357

Locus 215 :

2 alleles

99.2% missing data

3	(0.369) 0.372 0.368

2	(0.631) 0.628 0.632

Locus 216 :

2 alleles

99.2% missing data

4	(0.550) 0.545 0.548

1	(0.450) 0.455 0.452

Locus 217 :

2 alleles

99.2% missing data

1	(0.380) 0.372 0.389

3	(0.620) 0.628 0.611

Locus 218 :

2 alleles

98.8% missing data

1	(0.363) 0.353 0.370

4	(0.637) 0.647 0.630

Locus 219 :

2 alleles

98.8% missing data

1	(0.741) 0.735 0.748

3	(0.259) 0.265 0.252

Locus 220 :

2 alleles

99.2% missing data

3	(0.683) 0.680 0.687

1	(0.317) 0.320 0.313

Locus 221 :

2 alleles

96.1% missing data

3	(0.364) 0.339 0.377

4	(0.636) 0.661 0.623

Locus 222 :

2 alleles

99.2% missing data

4	(0.721) 0.725 0.721

3	(0.279) 0.275 0.279

Locus 223 :

3 alleles

97.7% missing data

2	(0.386) 0.384 0.389

3	(0.323) 0.321 0.331

1	(0.291) 0.296 0.280

Locus 224 :

2 alleles

99.2% missing data

3	(0.485) 0.486 0.484

4	(0.515) 0.514 0.516

Locus 225 :

2 alleles

99.2% missing data

1	(0.387) 0.375 0.394

4	(0.613) 0.625 0.606

Locus 226 :

2 alleles

99.2% missing data

2	(0.506) 0.510 0.513

1	(0.494) 0.490 0.487

Locus 227 :

2 alleles

97.7% missing data

3	(0.318) 0.315 0.327

1	(0.682) 0.685 0.673

Locus 228 :

2 alleles

99.2% missing data

1	(0.322) 0.320 0.329

3	(0.678) 0.680 0.671

Locus 229 :

2 alleles

99.2% missing data

2	(0.360) 0.353 0.367

3	(0.640) 0.647 0.633

Locus 230 :

2 alleles

96.5% missing data

1	(0.450) 0.451 0.441

3	(0.550) 0.549 0.559

Locus 231 :

3 alleles

97.7% missing data

1	(0.458) 0.462 0.460

4	(0.287) 0.282 0.287

3	(0.255) 0.255 0.253

Locus 232 :

2 alleles

99.2% missing data

2	(0.464) 0.462 0.461

4	(0.536) 0.538 0.539

Locus 233 :

2 alleles

98.8% missing data

3	(0.789) 0.783 0.791

4	(0.211) 0.217 0.209

Locus 234 :

2 alleles

98.8% missing data

2	(0.436) 0.436 0.440

1	(0.564) 0.564 0.560

Locus 235 :

2 alleles

99.2% missing data

3	(0.323) 0.321 0.330

4	(0.677) 0.679 0.670

Locus 236 :

2 alleles

99.2% missing data

1	(0.557) 0.554 0.564

3	(0.443) 0.446 0.436

Locus 237 :

2 alleles

0.4% missing data

1	(0.987) 0.982 0.997

3	(0.013) 0.018 0.003

Locus 238 :

3 alleles

98.0% missing data

1	(0.566) 0.570 0.569

4	(0.238) 0.236 0.240

3	(0.195) 0.193 0.191

Locus 239 :

2 alleles

99.2% missing data

1	(0.668) 0.670 0.658

2	(0.332) 0.330 0.342

Locus 240 :

3 alleles

98.8% missing data

1	(0.168) 0.174 0.164

3	(0.511) 0.502 0.519

4	(0.320) 0.324 0.317

Locus 241 :

2 alleles

98.8% missing data

1	(0.707) 0.699 0.716

2	(0.293) 0.301 0.284

Locus 242 :

2 alleles

97.7% missing data

3	(0.179) 0.172 0.183

2	(0.821) 0.828 0.817

Locus 243 :

2 alleles

8.6% missing data

4	(0.982) 0.974 0.995

3	(0.018) 0.026 0.005

Locus 244 :

2 alleles

98.8% missing data

3	(0.643) 0.642 0.643

1	(0.357) 0.358 0.357

Locus 245 :

2 alleles

94.5% missing data

3	(0.895) 0.906 0.891

2	(0.105) 0.094 0.109

Locus 246 :

2 alleles

98.8% missing data

3	(0.671) 0.671 0.676

2	(0.329) 0.329 0.324

Locus 247 :

3 alleles

98.8% missing data

2	(0.214) 0.219 0.211

3	(0.484) 0.484 0.481

1	(0.302) 0.297 0.308

Locus 248 :

2 alleles

99.2% missing data

3	(0.675) 0.679 0.670

1	(0.325) 0.321 0.330

Locus 249 :

2 alleles

99.2% missing data

3	(0.272) 0.271 0.271

2	(0.728) 0.729 0.729

Locus 250 :

2 alleles

98.4% missing data

2	(0.184) 0.180 0.189

1	(0.816) 0.820 0.811

Locus 251 :

3 alleles

76.6% missing data

1	(0.862) 0.866 0.876

2	(0.095) 0.094 0.088

4	(0.043) 0.040 0.036

Locus 252 :

2 alleles

97.7% missing data

1	(0.556) 0.569 0.555

2	(0.444) 0.431 0.445

Locus 253 :

2 alleles

98.8% missing data

1	(0.436) 0.433 0.439

2	(0.564) 0.567 0.561

Locus 254 :

3 alleles

98.8% missing data

1	(0.334) 0.330 0.330

3	(0.513) 0.516 0.520

4	(0.154) 0.154 0.150

Locus 255 :

2 alleles

99.2% missing data

1	(0.339) 0.338 0.336

3	(0.661) 0.662 0.664

Locus 256 :

2 alleles

98.0% missing data

2	(0.537) 0.546 0.530

1	(0.463) 0.454 0.470

Locus 257 :

2 alleles

89.5% missing data

1	(0.793) 0.787 0.808

2	(0.207) 0.213 0.192

Locus 258 :

2 alleles

98.8% missing data

1	(0.644) 0.645 0.646

4	(0.356) 0.355 0.354

Locus 259 :

2 alleles

98.8% missing data

2	(0.305) 0.297 0.304

1	(0.695) 0.703 0.696

Locus 260 :

2 alleles

96.9% missing data

1	(0.768) 0.778 0.766

2	(0.232) 0.222 0.234

Locus 261 :

2 alleles

98.4% missing data

4	(0.594) 0.594 0.598

2	(0.406) 0.406 0.402

Locus 262 :

2 alleles

99.2% missing data

4	(0.579) 0.579 0.579

2	(0.421) 0.421 0.421

Locus 263 :

2 alleles

99.2% missing data

1	(0.686) 0.689 0.680

3	(0.314) 0.311 0.320

Locus 264 :

2 alleles

98.8% missing data

1	(0.315) 0.317 0.323

2	(0.685) 0.683 0.677

Locus 265 :

2 alleles

96.1% missing data

1	(0.711) 0.705 0.732

4	(0.289) 0.295 0.268

Locus 266 :

2 alleles

99.2% missing data

3	(0.557) 0.551 0.562

1	(0.443) 0.449 0.438

Locus 267 :

2 alleles

87.9% missing data

4	(0.940) 0.941 0.946

2	(0.060) 0.059 0.054

Locus 268 :

2 alleles

97.7% missing data

3	(0.605) 0.604 0.618

1	(0.395) 0.396 0.382

Locus 269 :

2 alleles

0.0% missing data

3	(0.982) 0.987 0.991

1	(0.018) 0.013 0.009

Locus 270 :

3 alleles

98.4% missing data

2	(0.292) 0.288 0.302

4	(0.236) 0.234 0.236

1	(0.471) 0.478 0.462

Locus 271 :

2 alleles

99.2% missing data

2	(0.232) 0.224 0.240

1	(0.768) 0.776 0.760

Locus 272 :

2 alleles

97.7% missing data

1	(0.790) 0.779 0.800

4	(0.210) 0.221 0.200

Locus 273 :

2 alleles

98.0% missing data

1	(0.721) 0.717 0.729

3	(0.279) 0.283 0.271

Locus 274 :

2 alleles

99.2% missing data

2	(0.716) 0.713 0.711

3	(0.284) 0.287 0.289

Locus 275 :

3 alleles

96.5% missing data

1	(0.306) 0.304 0.307

2	(0.332) 0.315 0.345

3	(0.362) 0.380 0.348

Locus 276 :

2 alleles

97.7% missing data

2	(0.644) 0.643 0.651

1	(0.356) 0.357 0.349

Locus 277 :

2 alleles

97.3% missing data

1	(0.609) 0.608 0.615

2	(0.391) 0.392 0.385

Locus 278 :

2 alleles

98.8% missing data

2	(0.588) 0.587 0.595

3	(0.412) 0.413 0.405

Locus 279 :

2 alleles

98.4% missing data

2	(0.465) 0.458 0.473

1	(0.535) 0.542 0.527

Locus 280 :

2 alleles

99.2% missing data

4	(0.498) 0.497 0.501

1	(0.502) 0.503 0.499

Locus 281 :

2 alleles

99.2% missing data

1	(0.680) 0.679 0.682

3	(0.320) 0.321 0.318

Locus 282 :

2 alleles

99.2% missing data

1	(0.631) 0.630 0.627

2	(0.369) 0.370 0.373

Locus 283 :

4 alleles

97.7% missing data

4	(0.276) 0.267 0.283

3	(0.162) 0.157 0.161

1	(0.304) 0.309 0.306

2	(0.258) 0.266 0.250

Locus 284 :

2 alleles

98.4% missing data

2	(0.425) 0.417 0.417

4	(0.575) 0.583 0.583

Locus 285 :

2 alleles

98.8% missing data

3	(0.416) 0.419 0.418

4	(0.584) 0.581 0.582

Locus 286 :

2 alleles

99.2% missing data

1	(0.360) 0.364 0.359

4	(0.640) 0.636 0.641

Locus 287 :

3 alleles

98.4% missing data

3	(0.210) 0.209 0.212

1	(0.340) 0.335 0.340

2	(0.451) 0.456 0.448

Locus 288 :

2 alleles

99.2% missing data

2	(0.459) 0.460 0.455

1	(0.541) 0.540 0.545

Locus 289 :

2 alleles

98.8% missing data

4	(0.180) 0.180 0.178

1	(0.820) 0.820 0.822

Locus 290 :

2 alleles

99.2% missing data

2	(0.449) 0.447 0.446

4	(0.551) 0.553 0.554

Locus 291 :

2 alleles

99.2% missing data

1	(0.608) 0.609 0.613

2	(0.392) 0.391 0.387

Locus 292 :

2 alleles

99.2% missing data

1	(0.680) 0.680 0.686

4	(0.320) 0.320 0.314

Locus 293 :

2 alleles

99.2% missing data

4	(0.628) 0.625 0.635

2	(0.372) 0.375 0.365

Locus 294 :

2 alleles

98.4% missing data

4	(0.573) 0.586 0.569

1	(0.427) 0.414 0.431

Locus 295 :

2 alleles

98.8% missing data

4	(0.852) 0.859 0.851

1	(0.148) 0.141 0.149

Locus 296 :

2 alleles

83.2% missing data

4	(0.442) 0.514 0.383

2	(0.558) 0.486 0.617

Locus 297 :

2 alleles

98.0% missing data

1	(0.539) 0.542 0.542

2	(0.461) 0.458 0.458

Locus 298 :

2 alleles

92.6% missing data

1	(0.914) 0.906 0.926

3	(0.086) 0.094 0.074

Locus 299 :

2 alleles

99.2% missing data

2	(0.606) 0.605 0.603

1	(0.394) 0.395 0.397

Locus 300 :

2 alleles

96.1% missing data

4	(0.337) 0.328 0.335

2	(0.663) 0.672 0.665

Locus 301 :

2 alleles

98.8% missing data

4	(0.204) 0.214 0.204

3	(0.796) 0.786 0.796

Locus 302 :

2 alleles

95.7% missing data

2	(0.910) 0.918 0.904

1	(0.090) 0.082 0.096

Locus 303 :

2 alleles

0.8% missing data

4	(0.891) 0.754 0.967

2	(0.109) 0.246 0.033

Locus 304 :

2 alleles

93.0% missing data

2	(0.900) 0.903 0.906

1	(0.100) 0.097 0.094

Locus 305 :

2 alleles

98.8% missing data

2	(0.349) 0.354 0.353

3	(0.651) 0.646 0.647

Locus 306 :

2 alleles

98.0% missing data

4	(0.682) 0.683 0.686

3	(0.318) 0.317 0.314

Locus 307 :

2 alleles

96.9% missing data

2	(0.779) 0.773 0.782

4	(0.221) 0.227 0.218

Locus 308 :

2 alleles

99.2% missing data

1	(0.675) 0.673 0.679

2	(0.325) 0.327 0.321

Locus 309 :

2 alleles

99.2% missing data

2	(0.586) 0.584 0.586

4	(0.414) 0.416 0.414

Locus 310 :

2 alleles

8.6% missing data

2	(0.987) 0.981 0.996

3	(0.013) 0.019 0.004

Locus 311 :

3 alleles

85.5% missing data

1	(0.812) 0.821 0.818

2	(0.128) 0.123 0.127

4	(0.061) 0.056 0.055

Locus 312 :

2 alleles

93.0% missing data

1	(0.772) 0.761 0.785

2	(0.228) 0.239 0.215

Locus 313 :

2 alleles

99.2% missing data

3	(0.521) 0.524 0.524

4	(0.479) 0.476 0.476

Locus 314 :

2 alleles

96.5% missing data

1	(0.734) 0.726 0.749

2	(0.266) 0.274 0.251

Locus 315 :

2 alleles

97.3% missing data

2	(0.315) 0.323 0.313

1	(0.685) 0.677 0.687

Locus 316 :

2 alleles

95.3% missing data

3	(0.189) 0.175 0.205

1	(0.811) 0.825 0.795

Locus 317 :

2 alleles

99.2% missing data

1	(0.547) 0.539 0.550

4	(0.453) 0.461 0.450

Locus 318 :

2 alleles

98.4% missing data

1	(0.332) 0.341 0.330

2	(0.668) 0.659 0.670

Locus 319 :

2 alleles

97.3% missing data

4	(0.241) 0.241 0.237

3	(0.759) 0.759 0.763

Locus 320 :

2 alleles

98.8% missing data

1	(0.658) 0.658 0.657

2	(0.342) 0.342 0.343

Locus 321 :

2 alleles

99.2% missing data

1	(0.687) 0.688 0.688

2	(0.313) 0.312 0.312

Locus 322 :

2 alleles

99.2% missing data

2	(0.437) 0.437 0.440

3	(0.563) 0.563 0.560

Locus 323 :

3 alleles

98.8% missing data

2	(0.186) 0.183 0.189

1	(0.471) 0.476 0.468

3	(0.343) 0.341 0.344

Locus 324 :

3 alleles

98.8% missing data

4	(0.171) 0.164 0.175

1	(0.522) 0.532 0.512

2	(0.307) 0.304 0.312

Locus 325 :

2 alleles

8.6% missing data

3	(0.985) 0.977 0.995

4	(0.015) 0.023 0.005

Locus 326 :

2 alleles

91.0% missing data

3	(0.936) 0.948 0.930

1	(0.064) 0.052 0.070

Locus 327 :

2 alleles

99.2% missing data

1	(0.641) 0.644 0.638

4	(0.359) 0.356 0.362

Locus 328 :

2 alleles

97.3% missing data

3	(0.336) 0.342 0.322

4	(0.664) 0.658 0.678

Locus 329 :

2 alleles

98.0% missing data

3	(0.702) 0.706 0.700

1	(0.298) 0.294 0.300

Locus 330 :

2 alleles

95.7% missing data

1	(0.825) 0.826 0.835

4	(0.175) 0.174 0.165

Locus 331 :

3 alleles

98.4% missing data

1	(0.336) 0.341 0.333

4	(0.409) 0.408 0.411

2	(0.255) 0.251 0.256

Locus 332 :

2 alleles

94.9% missing data

1	(0.776) 0.767 0.794

2	(0.224) 0.233 0.206

Locus 333 :

3 alleles

95.7% missing data

2	(0.570) 0.561 0.587

1	(0.259) 0.274 0.240

3	(0.170) 0.164 0.174

Locus 334 :

2 alleles

99.2% missing data

4	(0.497) 0.493 0.499

3	(0.503) 0.507 0.501

Locus 335 :

4 alleles

94.5% missing data

1	(0.587) 0.581 0.607

4	(0.129) 0.128 0.130

3	(0.113) 0.116 0.105

2	(0.171) 0.175 0.158

Locus 336 :

2 alleles

98.8% missing data

4	(0.603) 0.611 0.594

1	(0.397) 0.389 0.406

Locus 337 :

2 alleles

99.2% missing data

3	(0.392) 0.389 0.405

2	(0.608) 0.611 0.595

Locus 338 :

2 alleles

96.9% missing data

1	(0.576) 0.580 0.569

2	(0.424) 0.420 0.431

Locus 339 :

2 alleles

97.3% missing data

2	(0.851) 0.841 0.851

1	(0.149) 0.159 0.149

Locus 340 :

2 alleles

96.1% missing data

4	(0.620) 0.626 0.608

2	(0.380) 0.374 0.392

Locus 341 :

2 alleles

99.2% missing data

1	(0.206) 0.205 0.208

2	(0.794) 0.795 0.792

Locus 342 :

2 alleles

99.2% missing data

1	(0.581) 0.581 0.583

4	(0.419) 0.419 0.417

Locus 343 :

3 alleles

96.9% missing data

4	(0.400) 0.392 0.398

1	(0.484) 0.498 0.482

2	(0.116) 0.111 0.120

Locus 344 :

2 alleles

99.2% missing data

3	(0.424) 0.422 0.432

4	(0.576) 0.578 0.568

Locus 345 :

2 alleles

98.8% missing data

4	(0.638) 0.643 0.640

3	(0.362) 0.357 0.360

Locus 346 :

2 alleles

99.2% missing data

1	(0.630) 0.630 0.628

2	(0.370) 0.370 0.372

Locus 347 :

2 alleles

98.8% missing data

3	(0.789) 0.787 0.792

4	(0.211) 0.213 0.208

Locus 348 :

2 alleles

99.2% missing data

2	(0.341) 0.348 0.338

3	(0.659) 0.652 0.662

Locus 349 :

2 alleles

93.0% missing data

2	(0.912) 0.910 0.915

4	(0.088) 0.090 0.085

Locus 350 :

2 alleles

98.0% missing data

2	(0.757) 0.758 0.758

3	(0.243) 0.242 0.242

Locus 351 :

2 alleles

99.2% missing data

3	(0.250) 0.249 0.254

2	(0.750) 0.751 0.746

Locus 352 :

2 alleles

97.7% missing data

1	(0.738) 0.735 0.741

2	(0.262) 0.265 0.259

Locus 353 :

3 alleles

98.0% missing data

3	(0.566) 0.560 0.577

4	(0.268) 0.267 0.262

2	(0.167) 0.174 0.161

Locus 354 :

2 alleles

99.2% missing data

1	(0.747) 0.741 0.754

2	(0.253) 0.259 0.246

Locus 355 :

2 alleles

96.5% missing data

2	(0.150) 0.141 0.155

4	(0.850) 0.859 0.845

Locus 356 :

2 alleles

99.2% missing data

2	(0.173) 0.172 0.178

4	(0.827) 0.828 0.822

Locus 357 :

2 alleles

96.1% missing data

4	(0.616) 0.618 0.625

2	(0.384) 0.382 0.375

Locus 358 :

2 alleles

0.0% missing data

4	(0.987) 0.986 0.995

1	(0.013) 0.014 0.005

Locus 359 :

2 alleles

99.2% missing data

3	(0.764) 0.765 0.757

2	(0.236) 0.235 0.243

Locus 360 :

2 alleles

99.2% missing data

3	(0.467) 0.468 0.475

1	(0.533) 0.532 0.525

Locus 361 :

2 alleles

91.0% missing data

2	(0.927) 0.941 0.917

1	(0.073) 0.059 0.083

Locus 362 :

2 alleles

99.2% missing data

3	(0.588) 0.593 0.588

4	(0.412) 0.407 0.412

Locus 363 :

2 alleles

99.2% missing data

4	(0.498) 0.488 0.502

1	(0.502) 0.512 0.498

Locus 364 :

2 alleles

98.4% missing data

3	(0.693) 0.693 0.689

4	(0.307) 0.307 0.311

Locus 365 :

2 alleles

99.2% missing data

3	(0.628) 0.629 0.624

2	(0.372) 0.371 0.376

Locus 366 :

2 alleles

98.0% missing data

4	(0.186) 0.186 0.186

1	(0.814) 0.814 0.814

Locus 367 :

2 alleles

98.0% missing data

4	(0.306) 0.289 0.311

1	(0.694) 0.711 0.689

Locus 368 :

2 alleles

99.2% missing data

2	(0.615) 0.610 0.620

1	(0.385) 0.390 0.380

Locus 369 :

2 alleles

99.2% missing data

1	(0.620) 0.614 0.627

2	(0.380) 0.386 0.373

Locus 370 :

2 alleles

99.2% missing data

1	(0.335) 0.335 0.330

3	(0.665) 0.665 0.670

Locus 371 :

2 alleles

99.2% missing data

2	(0.649) 0.653 0.644

1	(0.351) 0.347 0.356

Locus 372 :

3 alleles

97.7% missing data

3	(0.580) 0.580 0.584

4	(0.205) 0.206 0.198

1	(0.214) 0.213 0.217

Locus 373 :

2 alleles

8.2% missing data

1	(0.983) 0.974 0.995

4	(0.017) 0.026 0.005

Locus 374 :

2 alleles

99.2% missing data

1	(0.660) 0.658 0.656

3	(0.340) 0.342 0.344

Locus 375 :

2 alleles

92.2% missing data

3	(0.923) 0.924 0.928

1	(0.077) 0.076 0.072

Locus 376 :

2 alleles

96.9% missing data

4	(0.831) 0.830 0.837

3	(0.169) 0.170 0.163

Locus 377 :

2 alleles

7.8% missing data

3	(0.966) 0.933 0.989

1	(0.034) 0.067 0.011

Locus 378 :

2 alleles

99.2% missing data

4	(0.534) 0.530 0.541

3	(0.466) 0.470 0.459

Locus 379 :

2 alleles

98.4% missing data

4	(0.605) 0.612 0.602

2	(0.395) 0.388 0.398

Locus 380 :

2 alleles

9.4% missing data

2	(0.984) 0.988 0.991

1	(0.016) 0.012 0.009

Locus 381 :

2 alleles

14.1% missing data

2	(0.980) 0.984 0.986

3	(0.020) 0.016 0.014

Locus 382 :

2 alleles

98.4% missing data

3	(0.125) 0.127 0.123

1	(0.875) 0.873 0.877

Locus 383 :

2 alleles

92.6% missing data

4	(0.893) 0.880 0.908

1	(0.107) 0.120 0.092

Locus 384 :

2 alleles

95.7% missing data

1	(0.222) 0.208 0.232

2	(0.778) 0.792 0.768

Locus 385 :

2 alleles

95.7% missing data

2	(0.175) 0.164 0.189

1	(0.825) 0.836 0.811

Locus 386 :

2 alleles

99.2% missing data

3	(0.302) 0.294 0.303

2	(0.698) 0.706 0.697

Locus 387 :

2 alleles

98.4% missing data

4	(0.576) 0.565 0.596

3	(0.424) 0.435 0.404

Locus 388 :

2 alleles

99.2% missing data

3	(0.728) 0.721 0.736

2	(0.272) 0.279 0.264

Locus 389 :

2 alleles

99.2% missing data

4	(0.606) 0.611 0.597

1	(0.394) 0.389 0.403

Locus 390 :

2 alleles

98.8% missing data

1	(0.683) 0.684 0.682

2	(0.317) 0.316 0.318

Locus 391 :

2 alleles

98.8% missing data

4	(0.576) 0.569 0.584

1	(0.424) 0.431 0.416

Locus 392 :

2 alleles

90.6% missing data

2	(0.893) 0.915 0.882

1	(0.107) 0.085 0.118

Locus 393 :

2 alleles

96.9% missing data

1	(0.446) 0.427 0.447

4	(0.554) 0.573 0.553

Locus 394 :

2 alleles

98.0% missing data

3	(0.654) 0.648 0.667

4	(0.346) 0.352 0.333

Locus 395 :

2 alleles

96.1% missing data

2	(0.870) 0.880 0.873

3	(0.130) 0.120 0.127

Locus 396 :

2 alleles

99.2% missing data

4	(0.487) 0.477 0.493

3	(0.513) 0.523 0.507

Locus 397 :

2 alleles

98.8% missing data

3	(0.588) 0.593 0.584

2	(0.412) 0.407 0.416

Locus 398 :

2 alleles

98.0% missing data

1	(0.253) 0.259 0.242

2	(0.747) 0.741 0.758

Locus 399 :

3 alleles

98.4% missing data

2	(0.525) 0.526 0.525

3	(0.195) 0.191 0.200

1	(0.280) 0.283 0.275

Locus 400 :

2 alleles

99.2% missing data

1	(0.577) 0.569 0.584

3	(0.423) 0.431 0.416

Locus 401 :

2 alleles

99.2% missing data

2	(0.481) 0.482 0.487

1	(0.519) 0.518 0.513

Locus 402 :

2 alleles

99.2% missing data

2	(0.627) 0.622 0.636

1	(0.373) 0.378 0.364

Locus 403 :

2 alleles

99.2% missing data

1	(0.462) 0.464 0.463

2	(0.538) 0.536 0.537

Locus 404 :

2 alleles

99.2% missing data

2	(0.167) 0.171 0.166

4	(0.833) 0.829 0.834

Locus 405 :

2 alleles

99.2% missing data

2	(0.358) 0.362 0.361

4	(0.642) 0.638 0.639

Locus 406 :

2 alleles

99.2% missing data

4	(0.423) 0.432 0.423

1	(0.577) 0.568 0.577

Locus 407 :

3 alleles

98.8% missing data

1	(0.147) 0.148 0.149

4	(0.527) 0.521 0.528

2	(0.326) 0.331 0.323

Locus 408 :

2 alleles

99.2% missing data

4	(0.583) 0.581 0.578

1	(0.417) 0.419 0.422

Locus 409 :

2 alleles

94.9% missing data

3	(0.891) 0.891 0.899

4	(0.109) 0.109 0.101

Locus 410 :

2 alleles

99.2% missing data

1	(0.530) 0.522 0.542

3	(0.470) 0.478 0.458

Locus 411 :

2 alleles

98.8% missing data

4	(0.181) 0.177 0.188

1	(0.819) 0.823 0.812

Locus 412 :

3 alleles

98.4% missing data

1	(0.484) 0.488 0.486

4	(0.164) 0.160 0.166

2	(0.352) 0.352 0.348

Locus 413 :

2 alleles

98.8% missing data

1	(0.529) 0.517 0.537

3	(0.471) 0.483 0.463

Locus 414 :

2 alleles

99.2% missing data

4	(0.403) 0.402 0.401

1	(0.597) 0.598 0.599

Locus 415 :

2 alleles

98.8% missing data

1	(0.606) 0.614 0.600

4	(0.394) 0.386 0.400

Locus 416 :

2 alleles

99.2% missing data

2	(0.612) 0.612 0.612

1	(0.388) 0.388 0.388

Locus 417 :

2 alleles

99.2% missing data

1	(0.272) 0.264 0.275

3	(0.728) 0.736 0.725

Locus 418 :

2 alleles

98.0% missing data

4	(0.780) 0.784 0.777

1	(0.220) 0.216 0.223

Locus 419 :

2 alleles

98.4% missing data

3	(0.656) 0.648 0.662

1	(0.344) 0.352 0.338

Locus 420 :

2 alleles

99.2% missing data

4	(0.270) 0.269 0.277

3	(0.730) 0.731 0.723

Locus 421 :

3 alleles

98.8% missing data

4	(0.237) 0.231 0.237

1	(0.317) 0.323 0.313

3	(0.446) 0.445 0.450

Locus 422 :

2 alleles

99.2% missing data

1	(0.399) 0.402 0.397

3	(0.601) 0.598 0.603

Locus 423 :

2 alleles

98.4% missing data

1	(0.746) 0.745 0.744

4	(0.254) 0.255 0.256

Locus 424 :

2 alleles

92.6% missing data

1	(0.857) 0.854 0.872

3	(0.143) 0.146 0.128

Locus 425 :

2 alleles

98.8% missing data

1	(0.265) 0.263 0.265

4	(0.735) 0.737 0.735

Locus 426 :

2 alleles

96.5% missing data

4	(0.745) 0.763 0.741

3	(0.255) 0.237 0.259

Locus 427 :

2 alleles

96.1% missing data

4	(0.795) 0.805 0.797

3	(0.205) 0.195 0.203

Locus 428 :

2 alleles

99.2% missing data

4	(0.329) 0.323 0.331

3	(0.671) 0.677 0.669

Locus 429 :

2 alleles

98.0% missing data

2	(0.625) 0.623 0.631

1	(0.375) 0.377 0.369

Locus 430 :

2 alleles

98.4% missing data

1	(0.699) 0.706 0.695

2	(0.301) 0.294 0.305

Locus 431 :

2 alleles

97.3% missing data

3	(0.208) 0.208 0.207

1	(0.792) 0.792 0.793

Locus 432 :

2 alleles

99.2% missing data

2	(0.332) 0.334 0.336

3	(0.668) 0.666 0.664

Locus 433 :

2 alleles

99.2% missing data

4	(0.537) 0.533 0.542

1	(0.463) 0.467 0.458

Locus 434 :

2 alleles

99.2% missing data

3	(0.775) 0.780 0.770

1	(0.225) 0.220 0.230

Locus 435 :

2 alleles

98.8% missing data

1	(0.330) 0.327 0.335

4	(0.670) 0.673 0.665

Locus 436 :

2 alleles

99.2% missing data

2	(0.410) 0.405 0.417

4	(0.590) 0.595 0.583

Locus 437 :

2 alleles

99.2% missing data

2	(0.351) 0.346 0.360

4	(0.649) 0.654 0.640

Locus 438 :

2 alleles

99.2% missing data

1	(0.706) 0.699 0.709

3	(0.294) 0.301 0.291

Locus 439 :

3 alleles

98.8% missing data

2	(0.339) 0.335 0.336

1	(0.446) 0.451 0.443

4	(0.215) 0.214 0.220

Locus 440 :

3 alleles

98.8% missing data

3	(0.624) 0.619 0.630

1	(0.190) 0.194 0.185

2	(0.186) 0.187 0.185

Locus 441 :

2 alleles

98.4% missing data

3	(0.509) 0.493 0.515

1	(0.491) 0.507 0.485

Locus 442 :

2 alleles

99.2% missing data

4	(0.593) 0.594 0.591

3	(0.407) 0.406 0.409

Locus 443 :

2 alleles

97.7% missing data

2	(0.270) 0.262 0.270

4	(0.730) 0.738 0.730

Locus 444 :

2 alleles

97.7% missing data

4	(0.775) 0.762 0.785

1	(0.225) 0.238 0.215

Locus 445 :

2 alleles

98.4% missing data

1	(0.723) 0.727 0.716

4	(0.277) 0.273 0.284

Locus 446 :

2 alleles

99.2% missing data

1	(0.529) 0.521 0.537

4	(0.471) 0.479 0.463

Locus 447 :

2 alleles

99.2% missing data

1	(0.590) 0.589 0.593

2	(0.410) 0.411 0.407

Locus 448 :

2 alleles

99.2% missing data

4	(0.622) 0.631 0.621

2	(0.378) 0.369 0.379

Locus 449 :

2 alleles

99.2% missing data

3	(0.420) 0.415 0.425

4	(0.580) 0.585 0.575

Locus 450 :

2 alleles

99.2% missing data

2	(0.569) 0.568 0.579

3	(0.431) 0.432 0.421

Locus 451 :

2 alleles

98.4% missing data

4	(0.583) 0.585 0.590

2	(0.417) 0.415 0.410

Locus 452 :

2 alleles

98.4% missing data

2	(0.639) 0.635 0.641

3	(0.361) 0.365 0.359

Locus 453 :

2 alleles

99.2% missing data

2	(0.270) 0.265 0.271

3	(0.730) 0.735 0.729

Locus 454 :

2 alleles

98.8% missing data

1	(0.632) 0.631 0.635

3	(0.368) 0.369 0.365

Locus 455 :

2 alleles

99.2% missing data

1	(0.653) 0.657 0.647

4	(0.347) 0.343 0.353

Locus 456 :

2 alleles

98.4% missing data

2	(0.301) 0.297 0.304

1	(0.699) 0.703 0.696

Locus 457 :

3 alleles

98.4% missing data

1	(0.550) 0.546 0.557

2	(0.215) 0.211 0.216

4	(0.234) 0.243 0.227

Locus 458 :

2 alleles

99.2% missing data

4	(0.626) 0.628 0.632

1	(0.374) 0.372 0.368

Locus 459 :

2 alleles

96.9% missing data

4	(0.334) 0.332 0.335

3	(0.666) 0.668 0.665

Locus 460 :

2 alleles

99.2% missing data

2	(0.494) 0.494 0.493

3	(0.506) 0.506 0.507

Locus 461 :

3 alleles

98.8% missing data

3	(0.269) 0.268 0.267

2	(0.444) 0.442 0.449

4	(0.287) 0.290 0.284

Locus 462 :

2 alleles

99.2% missing data

3	(0.684) 0.686 0.680

2	(0.316) 0.314 0.320

Locus 463 :

2 alleles

97.7% missing data

3	(0.681) 0.683 0.689

1	(0.319) 0.317 0.311

Locus 464 :

2 alleles

99.2% missing data

1	(0.419) 0.422 0.418

3	(0.581) 0.578 0.582

Locus 465 :

2 alleles

99.2% missing data

4	(0.542) 0.535 0.545

3	(0.458) 0.465 0.455

Locus 466 :

2 alleles

96.9% missing data

2	(0.776) 0.771 0.791

4	(0.224) 0.229 0.209

Locus 467 :

2 alleles

1.2% missing data

4	(0.925) 0.777 0.977

1	(0.075) 0.223 0.023

Locus 468 :

2 alleles

99.2% missing data

1	(0.568) 0.560 0.569

3	(0.432) 0.440 0.431

Locus 469 :

2 alleles

94.1% missing data

1	(0.844) 0.838 0.863

2	(0.156) 0.162 0.137

Locus 470 :

2 alleles

99.2% missing data

1	(0.189) 0.181 0.194

4	(0.811) 0.819 0.806

Locus 471 :

2 alleles

99.2% missing data

1	(0.482) 0.474 0.489

3	(0.518) 0.526 0.511

Locus 472 :

2 alleles

99.2% missing data

2	(0.353) 0.349 0.354

3	(0.647) 0.651 0.646

Locus 473 :

2 alleles

99.2% missing data

4	(0.417) 0.416 0.422

3	(0.583) 0.584 0.578

Locus 474 :

2 alleles

98.8% missing data

3	(0.666) 0.662 0.665

2	(0.334) 0.338 0.335

Locus 475 :

2 alleles

98.8% missing data

2	(0.556) 0.546 0.558

1	(0.444) 0.454 0.442

Locus 476 :

2 alleles

98.0% missing data

3	(0.695) 0.682 0.708

4	(0.305) 0.318 0.292

Locus 477 :

2 alleles

99.2% missing data

4	(0.704) 0.702 0.704

3	(0.296) 0.298 0.296

Locus 478 :

2 alleles

96.1% missing data

2	(0.831) 0.842 0.824

1	(0.169) 0.158 0.176

Locus 479 :

3 alleles

98.4% missing data

4	(0.379) 0.373 0.378

2	(0.187) 0.186 0.193

1	(0.435) 0.441 0.429

Locus 480 :

2 alleles

98.8% missing data

4	(0.831) 0.836 0.820

1	(0.169) 0.164 0.180

Locus 481 :

3 alleles

98.0% missing data

3	(0.305) 0.307 0.302

1	(0.313) 0.316 0.313

4	(0.381) 0.377 0.386

Locus 482 :

2 alleles

0.0% missing data

2	(0.984) 0.982 0.995

3	(0.016) 0.018 0.005

Locus 483 :

3 alleles

94.1% missing data

4	(0.589) 0.611 0.561

3	(0.338) 0.311 0.371

1	(0.073) 0.078 0.068

Locus 484 :

3 alleles

98.8% missing data

3	(0.560) 0.558 0.557

2	(0.221) 0.216 0.228

1	(0.218) 0.226 0.215

Locus 485 :

2 alleles

99.2% missing data

3	(0.365) 0.359 0.373

1	(0.635) 0.641 0.627

Locus 486 :

2 alleles

97.3% missing data

4	(0.766) 0.765 0.771

1	(0.234) 0.235 0.229

Locus 487 :

2 alleles

91.4% missing data

2	(0.934) 0.930 0.944

3	(0.066) 0.070 0.056

Locus 488 :

2 alleles

96.1% missing data

2	(0.840) 0.843 0.838

3	(0.160) 0.157 0.162

Locus 489 :

2 alleles

98.8% missing data

1	(0.379) 0.379 0.390

2	(0.621) 0.621 0.610

Locus 490 :

2 alleles

98.8% missing data

3	(0.377) 0.373 0.384

4	(0.623) 0.627 0.616

Locus 491 :

2 alleles

99.2% missing data

3	(0.486) 0.485 0.488

4	(0.514) 0.515 0.512

Locus 492 :

2 alleles

95.7% missing data

2	(0.848) 0.840 0.859

1	(0.152) 0.160 0.141

Locus 493 :

2 alleles

98.0% missing data

2	(0.666) 0.661 0.680

1	(0.334) 0.339 0.320

Locus 494 :

2 alleles

8.6% missing data

4	(0.984) 0.979 0.995

2	(0.016) 0.021 0.005

Locus 495 :

2 alleles

99.2% missing data

1	(0.619) 0.621 0.623

3	(0.381) 0.379 0.377

Locus 496 :

2 alleles

99.2% missing data

1	(0.778) 0.782 0.770

4	(0.222) 0.218 0.230

Locus 497 :

2 alleles

98.8% missing data

2	(0.449) 0.449 0.457

3	(0.551) 0.551 0.543

Locus 498 :

2 alleles

99.2% missing data

1	(0.537) 0.530 0.540

2	(0.463) 0.470 0.460

Locus 499 :

2 alleles

98.8% missing data

1	(0.683) 0.688 0.680

3	(0.317) 0.312 0.320

Locus 500 :

2 alleles

99.2% missing data

1	(0.755) 0.747 0.760

2	(0.245) 0.253 0.240

Locus 501 :

2 alleles

98.4% missing data

4	(0.661) 0.667 0.651

1	(0.339) 0.333 0.349

Locus 502 :

3 alleles

98.8% missing data

1	(0.481) 0.472 0.486

2	(0.122) 0.131 0.117

4	(0.397) 0.396 0.397

Locus 503 :

2 alleles

99.2% missing data

1	(0.271) 0.274 0.275

4	(0.729) 0.726 0.725

Locus 504 :

2 alleles

99.2% missing data

1	(0.666) 0.673 0.661

3	(0.334) 0.327 0.339

Locus 505 :

2 alleles

98.8% missing data

1	(0.504) 0.498 0.513

2	(0.496) 0.502 0.487

Locus 506 :

2 alleles

99.2% missing data

1	(0.452) 0.454 0.456

2	(0.548) 0.546 0.544

Locus 507 :

2 alleles

99.2% missing data

2	(0.485) 0.482 0.487

1	(0.515) 0.518 0.513

Locus 508 :

2 alleles

19.5% missing data

3	(0.983) 0.985 0.990

4	(0.017) 0.015 0.010

Locus 509 :

2 alleles

97.7% missing data

4	(0.355) 0.343 0.354

1	(0.645) 0.657 0.646

Locus 510 :

2 alleles

99.2% missing data

3	(0.549) 0.553 0.545

2	(0.451) 0.447 0.455

Locus 511 :

2 alleles

99.2% missing data

3	(0.561) 0.555 0.567

1	(0.439) 0.445 0.433

Locus 512 :

3 alleles

95.7% missing data

3	(0.167) 0.164 0.169

1	(0.686) 0.695 0.689

2	(0.147) 0.141 0.142

Locus 513 :

2 alleles

99.2% missing data

2	(0.670) 0.671 0.666

4	(0.330) 0.329 0.334

Locus 514 :

3 alleles

97.7% missing data

2	(0.199) 0.194 0.199

1	(0.612) 0.613 0.612

4	(0.189) 0.193 0.188

Locus 515 :

2 alleles

93.4% missing data

1	(0.708) 0.703 0.721

4	(0.292) 0.297 0.279

Locus 516 :

2 alleles

99.2% missing data

3	(0.605) 0.599 0.604

1	(0.395) 0.401 0.396

Locus 517 :

3 alleles

93.8% missing data

2	(0.144) 0.144 0.141

3	(0.644) 0.640 0.666

4	(0.212) 0.216 0.193

Locus 518 :

2 alleles

91.8% missing data

1	(0.916) 0.917 0.921

2	(0.084) 0.083 0.079

Locus 519 :

2 alleles

99.2% missing data

4	(0.391) 0.381 0.400

1	(0.609) 0.619 0.600

Locus 520 :

2 alleles

99.2% missing data

2	(0.446) 0.450 0.449

1	(0.554) 0.550 0.551

Locus 521 :

2 alleles

99.2% missing data

4	(0.543) 0.543 0.547

1	(0.457) 0.457 0.453

Locus 522 :

2 alleles

99.2% missing data

2	(0.736) 0.735 0.743

1	(0.264) 0.265 0.257

Locus 523 :

2 alleles

99.2% missing data

2	(0.536) 0.532 0.530

3	(0.464) 0.468 0.470

Locus 524 :

2 alleles

99.2% missing data

2	(0.254) 0.252 0.258

3	(0.746) 0.748 0.742

Locus 525 :

2 alleles

99.2% missing data

1	(0.494) 0.492 0.497

2	(0.506) 0.508 0.503

Locus 526 :

2 alleles

98.4% missing data

4	(0.238) 0.239 0.236

1	(0.762) 0.761 0.764

Locus 527 :

2 alleles

99.2% missing data

4	(0.497) 0.497 0.494

2	(0.503) 0.503 0.506

Locus 528 :

2 alleles

99.2% missing data

2	(0.463) 0.462 0.465

3	(0.537) 0.538 0.535

Locus 529 :

2 alleles

98.4% missing data

2	(0.571) 0.579 0.572

1	(0.429) 0.421 0.428

Locus 530 :

2 alleles

98.0% missing data

2	(0.557) 0.568 0.557

4	(0.443) 0.432 0.443

Locus 531 :

2 alleles

99.2% missing data

4	(0.484) 0.486 0.484

2	(0.516) 0.514 0.516

Locus 532 :

2 alleles

99.2% missing data

1	(0.722) 0.717 0.727

2	(0.278) 0.283 0.273

Locus 533 :

2 alleles

97.7% missing data

1	(0.299) 0.290 0.307

2	(0.701) 0.710 0.693

Locus 534 :

3 alleles

96.5% missing data

2	(0.272) 0.277 0.267

1	(0.203) 0.207 0.199

4	(0.524) 0.516 0.535

Locus 535 :

2 alleles

99.2% missing data

2	(0.714) 0.719 0.712

3	(0.286) 0.281 0.288

Locus 536 :

2 alleles

98.8% missing data

1	(0.337) 0.337 0.349

3	(0.663) 0.663 0.651

Locus 537 :

2 alleles

99.2% missing data

2	(0.524) 0.527 0.523

4	(0.476) 0.473 0.477

Locus 538 :

2 alleles

99.2% missing data

2	(0.514) 0.503 0.515

1	(0.486) 0.497 0.485

Locus 539 :

2 alleles

98.0% missing data

4	(0.628) 0.634 0.625

2	(0.372) 0.366 0.375

Locus 540 :

2 alleles

98.4% missing data

2	(0.599) 0.609 0.596

3	(0.401) 0.391 0.404

Locus 541 :

2 alleles

91.4% missing data

2	(0.080) 0.076 0.078

4	(0.920) 0.924 0.922

Locus 542 :

2 alleles

99.2% missing data

2	(0.452) 0.443 0.457

1	(0.548) 0.557 0.543

Locus 543 :

2 alleles

99.2% missing data

4	(0.707) 0.714 0.704

1	(0.293) 0.286 0.296

Locus 544 :

2 alleles

96.5% missing data

1	(0.886) 0.892 0.883

4	(0.114) 0.108 0.117

Locus 545 :

2 alleles

99.2% missing data

4	(0.434) 0.442 0.429

2	(0.566) 0.558 0.571

Locus 546 :

2 alleles

99.2% missing data

2	(0.438) 0.435 0.434

3	(0.562) 0.565 0.566

Locus 547 :

2 alleles

98.8% missing data

1	(0.655) 0.661 0.652

2	(0.345) 0.339 0.348

Locus 548 :

2 alleles

98.8% missing data

4	(0.484) 0.482 0.491

1	(0.516) 0.518 0.509

Locus 549 :

2 alleles

99.2% missing data

4	(0.424) 0.428 0.422

1	(0.576) 0.572 0.578

Locus 550 :

2 alleles

99.2% missing data

1	(0.503) 0.505 0.504

2	(0.497) 0.495 0.496

Locus 551 :

2 alleles

99.2% missing data

2	(0.700) 0.694 0.698

4	(0.300) 0.306 0.302

Locus 552 :

2 alleles

99.2% missing data

1	(0.495) 0.487 0.500

2	(0.505) 0.513 0.500

Locus 553 :

2 alleles

99.2% missing data

3	(0.359) 0.353 0.359

1	(0.641) 0.647 0.641

Locus 554 :

2 alleles

8.2% missing data

3	(0.986) 0.981 0.996

2	(0.014) 0.019 0.004

Locus 555 :

2 alleles

0.4% missing data

2	(0.959) 0.954 0.972

1	(0.041) 0.046 0.028

Locus 556 :

2 alleles

91.0% missing data

1	(0.882) 0.903 0.864

2	(0.118) 0.097 0.136

Locus 557 :

2 alleles

91.0% missing data

3	(0.886) 0.909 0.878

2	(0.114) 0.091 0.122

Locus 558 :

2 alleles

98.4% missing data

3	(0.535) 0.533 0.542

1	(0.465) 0.467 0.458

Locus 559 :

2 alleles

99.2% missing data

3	(0.456) 0.459 0.456

1	(0.544) 0.541 0.544

Locus 560 :

2 alleles

99.2% missing data

2	(0.559) 0.556 0.562

4	(0.441) 0.444 0.438

Locus 561 :

2 alleles

98.8% missing data

3	(0.560) 0.554 0.562

4	(0.440) 0.446 0.438

Locus 562 :

2 alleles

99.2% missing data

2	(0.543) 0.544 0.533

3	(0.457) 0.456 0.467

Locus 563 :

4 alleles

85.5% missing data

2	(0.133) 0.124 0.137

4	(0.712) 0.723 0.710

1	(0.069) 0.070 0.072

3	(0.087) 0.083 0.082

Locus 564 :

2 alleles

99.2% missing data

4	(0.298) 0.295 0.309

2	(0.702) 0.705 0.691

Locus 565 :

2 alleles

99.2% missing data

3	(0.474) 0.469 0.478

4	(0.526) 0.531 0.522

Locus 566 :

2 alleles

99.2% missing data

1	(0.720) 0.723 0.712

2	(0.280) 0.277 0.288

Locus 567 :

2 alleles

99.2% missing data

4	(0.659) 0.658 0.664

1	(0.341) 0.342 0.336

Locus 568 :

2 alleles

99.2% missing data

2	(0.521) 0.523 0.521

4	(0.479) 0.477 0.479

Locus 569 :

3 alleles

98.8% missing data

4	(0.594) 0.588 0.588

2	(0.206) 0.206 0.208

1	(0.200) 0.206 0.204

Locus 570 :

2 alleles

98.8% missing data

4	(0.412) 0.405 0.419

1	(0.588) 0.595 0.581

Locus 571 :

2 alleles

99.2% missing data

1	(0.407) 0.403 0.410

2	(0.593) 0.597 0.590

Locus 572 :

2 alleles

96.9% missing data

1	(0.689) 0.682 0.712

2	(0.311) 0.318 0.288

Locus 573 :

2 alleles

99.2% missing data

1	(0.644) 0.649 0.636

3	(0.356) 0.351 0.364

Locus 574 :

3 alleles

98.8% missing data

1	(0.234) 0.232 0.233

4	(0.172) 0.170 0.179

3	(0.595) 0.598 0.587

Locus 575 :

2 alleles

99.2% missing data

3	(0.232) 0.226 0.234

2	(0.768) 0.774 0.766

Locus 576 :

2 alleles

99.2% missing data

1	(0.325) 0.326 0.323

3	(0.675) 0.674 0.677

Locus 577 :

2 alleles

99.2% missing data

2	(0.528) 0.522 0.523

1	(0.472) 0.478 0.477

Locus 578 :

3 alleles

98.8% missing data

3	(0.134) 0.132 0.141

2	(0.410) 0.422 0.401

1	(0.456) 0.446 0.458

Locus 579 :

2 alleles

99.2% missing data

2	(0.313) 0.308 0.308

3	(0.687) 0.692 0.692

Locus 580 :

3 alleles

98.4% missing data

4	(0.524) 0.531 0.518

3	(0.230) 0.227 0.232

2	(0.245) 0.242 0.250

Locus 581 :

2 alleles

99.2% missing data

4	(0.358) 0.358 0.357

2	(0.642) 0.642 0.643

Locus 582 :

2 alleles

99.2% missing data

1	(0.729) 0.732 0.724

3	(0.271) 0.268 0.276

Locus 583 :

2 alleles

99.2% missing data

2	(0.384) 0.377 0.389

1	(0.616) 0.623 0.611

Locus 584 :

2 alleles

99.2% missing data

2	(0.275) 0.278 0.269

1	(0.725) 0.722 0.731

Locus 585 :

2 alleles

99.2% missing data

1	(0.791) 0.787 0.792

2	(0.209) 0.213 0.208

Locus 586 :

2 alleles

97.3% missing data

1	(0.510) 0.525 0.484

2	(0.490) 0.475 0.516

Locus 587 :

2 alleles

99.2% missing data

2	(0.507) 0.503 0.510

1	(0.493) 0.497 0.490

Locus 588 :

2 alleles

0.4% missing data

2	(0.964) 0.972 0.966

1	(0.036) 0.028 0.034

Locus 589 :

2 alleles

99.2% missing data

4	(0.531) 0.527 0.535

1	(0.469) 0.473 0.465

Locus 590 :

2 alleles

99.2% missing data

4	(0.442) 0.441 0.449

1	(0.558) 0.559 0.551

Locus 591 :

2 alleles

99.2% missing data

4	(0.477) 0.477 0.485

3	(0.523) 0.523 0.515

Locus 592 :

2 alleles

98.8% missing data

1	(0.431) 0.427 0.433

2	(0.569) 0.573 0.567

Locus 593 :

2 alleles

98.4% missing data

4	(0.700) 0.705 0.703

3	(0.300) 0.295 0.297

Locus 594 :

2 alleles

99.2% missing data

2	(0.583) 0.588 0.582

1	(0.417) 0.412 0.418

Locus 595 :

3 alleles

95.7% missing data

1	(0.165) 0.155 0.170

2	(0.144) 0.139 0.145

3	(0.691) 0.706 0.685

Locus 596 :

3 alleles

97.7% missing data

4	(0.580) 0.581 0.583

1	(0.275) 0.276 0.271

3	(0.145) 0.143 0.145

Locus 597 :

3 alleles

95.7% missing data

3	(0.803) 0.810 0.797

1	(0.054) 0.049 0.061

2	(0.143) 0.141 0.142

Locus 598 :

2 alleles

98.4% missing data

3	(0.503) 0.502 0.516

4	(0.497) 0.498 0.484

Locus 599 :

3 alleles

98.4% missing data

3	(0.453) 0.449 0.465

2	(0.182) 0.187 0.178

4	(0.365) 0.364 0.357

Locus 600 :

4 alleles

98.0% missing data

1	(0.148) 0.143 0.147

2	(0.361) 0.353 0.373

3	(0.244) 0.250 0.242

4	(0.247) 0.253 0.238

Locus 601 :

2 alleles

99.2% missing data

1	(0.309) 0.309 0.314

2	(0.691) 0.691 0.686

Locus 602 :

2 alleles

99.2% missing data

3	(0.699) 0.694 0.695

1	(0.301) 0.306 0.305

Locus 603 :

2 alleles

99.2% missing data

3	(0.265) 0.260 0.272

1	(0.735) 0.740 0.728

Locus 604 :

2 alleles

99.2% missing data

2	(0.323) 0.325 0.322

4	(0.677) 0.675 0.678

Locus 605 :

2 alleles

98.4% missing data

4	(0.569) 0.574 0.565

2	(0.431) 0.426 0.435

Locus 606 :

2 alleles

92.6% missing data

2	(0.884) 0.879 0.896

3	(0.116) 0.121 0.104

Locus 607 :

2 alleles

99.2% missing data

2	(0.607) 0.607 0.605

4	(0.393) 0.393 0.395

Locus 608 :

2 alleles

99.2% missing data

1	(0.492) 0.489 0.497

2	(0.508) 0.511 0.503

Locus 609 :

2 alleles

99.2% missing data

3	(0.152) 0.146 0.154

4	(0.848) 0.854 0.846

Locus 610 :

2 alleles

99.2% missing data

4	(0.822) 0.823 0.819

2	(0.178) 0.177 0.181

Locus 611 :

2 alleles

91.4% missing data

2	(0.869) 0.868 0.884

1	(0.131) 0.132 0.116

Locus 612 :

2 alleles

98.8% missing data

3	(0.293) 0.290 0.292

4	(0.707) 0.710 0.708

Locus 613 :

2 alleles

98.0% missing data

4	(0.793) 0.790 0.793

1	(0.207) 0.210 0.207

Locus 614 :

2 alleles

95.3% missing data

4	(0.737) 0.755 0.715

1	(0.263) 0.245 0.285

Locus 615 :

2 alleles

98.8% missing data

4	(0.630) 0.632 0.630

1	(0.370) 0.368 0.370

Locus 616 :

2 alleles

98.8% missing data

3	(0.554) 0.553 0.563

1	(0.446) 0.447 0.437

Locus 617 :

2 alleles

99.2% missing data

1	(0.766) 0.765 0.765

4	(0.234) 0.235 0.235

Locus 618 :

2 alleles

99.2% missing data

1	(0.599) 0.596 0.599

4	(0.401) 0.404 0.401

Locus 619 :

2 alleles

99.2% missing data

1	(0.544) 0.551 0.538

2	(0.456) 0.449 0.462

Locus 620 :

2 alleles

8.2% missing data

1	(0.979) 0.961 0.993

2	(0.021) 0.039 0.007

Locus 621 :

2 alleles

99.2% missing data

4	(0.321) 0.314 0.329

1	(0.679) 0.686 0.671

Locus 622 :

2 alleles

98.4% missing data

1	(0.843) 0.844 0.839

3	(0.157) 0.156 0.161

Locus 623 :

2 alleles

91.4% missing data

1	(0.925) 0.931 0.926

4	(0.075) 0.069 0.074

Locus 624 :

2 alleles

90.2% missing data

4	(0.879) 0.902 0.868

3	(0.121) 0.098 0.132

Locus 625 :

2 alleles

92.6% missing data

2	(0.890) 0.889 0.897

3	(0.110) 0.111 0.103

Locus 626 :

2 alleles

99.2% missing data

1	(0.512) 0.506 0.519

4	(0.488) 0.494 0.481

Locus 627 :

2 alleles

99.2% missing data

2	(0.275) 0.274 0.272

4	(0.725) 0.726 0.728

Locus 628 :

2 alleles

99.2% missing data

1	(0.382) 0.374 0.386

2	(0.618) 0.626 0.614

Locus 629 :

3 alleles

98.8% missing data

3	(0.276) 0.273 0.279

1	(0.434) 0.438 0.428

4	(0.289) 0.289 0.293

Locus 630 :

2 alleles

98.8% missing data

4	(0.542) 0.534 0.550

3	(0.458) 0.466 0.450

Locus 631 :

2 alleles

99.2% missing data

3	(0.377) 0.376 0.375

4	(0.623) 0.624 0.625

Locus 632 :

2 alleles

90.2% missing data

2	(0.807) 0.839 0.776

1	(0.193) 0.161 0.224

Locus 633 :

2 alleles

99.2% missing data

4	(0.348) 0.344 0.354

3	(0.652) 0.656 0.646

Locus 634 :

2 alleles

99.2% missing data

3	(0.285) 0.286 0.286

1	(0.715) 0.714 0.714

Locus 635 :

2 alleles

98.8% missing data

3	(0.506) 0.509 0.501

2	(0.494) 0.491 0.499

Locus 636 :

2 alleles

98.8% missing data

4	(0.492) 0.496 0.491

1	(0.508) 0.504 0.509

Locus 637 :

2 alleles

98.4% missing data

1	(0.591) 0.587 0.600

2	(0.409) 0.413 0.400

Locus 638 :

2 alleles

90.6% missing data

3	(0.928) 0.925 0.941

1	(0.072) 0.075 0.059

Locus 639 :

2 alleles

99.2% missing data

3	(0.799) 0.800 0.803

4	(0.201) 0.200 0.197

Locus 640 :

2 alleles

99.2% missing data

2	(0.501) 0.504 0.497

1	(0.499) 0.496 0.503

Locus 641 :

2 alleles

99.2% missing data

2	(0.666) 0.668 0.660

1	(0.334) 0.332 0.340

Locus 642 :

2 alleles

99.2% missing data

4	(0.342) 0.339 0.349

1	(0.658) 0.661 0.651

Locus 643 :

2 alleles

99.2% missing data

4	(0.432) 0.426 0.437

3	(0.568) 0.574 0.563

Locus 644 :

2 alleles

97.7% missing data

1	(0.197) 0.195 0.194

4	(0.803) 0.805 0.806

Locus 645 :

2 alleles

98.4% missing data

4	(0.537) 0.538 0.540

2	(0.463) 0.462 0.460

Locus 646 :

2 alleles

91.0% missing data

2	(0.920) 0.915 0.931

1	(0.080) 0.085 0.069

Locus 647 :

2 alleles

93.0% missing data

3	(0.908) 0.910 0.916

1	(0.092) 0.090 0.084

Locus 648 :

2 alleles

99.2% missing data

4	(0.521) 0.515 0.525

1	(0.479) 0.485 0.475

Locus 649 :

2 alleles

99.2% missing data

2	(0.464) 0.466 0.459

1	(0.536) 0.534 0.541

Locus 650 :

2 alleles

98.8% missing data

2	(0.464) 0.473 0.460

1	(0.536) 0.527 0.540

Locus 651 :

2 alleles

98.8% missing data

1	(0.746) 0.741 0.744

4	(0.254) 0.259 0.256

Locus 652 :

2 alleles

99.2% missing data

2	(0.205) 0.203 0.213

4	(0.795) 0.797 0.787

Locus 653 :

3 alleles

98.8% missing data

3	(0.267) 0.265 0.271

2	(0.389) 0.390 0.388

1	(0.344) 0.345 0.340

Locus 654 :

2 alleles

99.2% missing data

1	(0.432) 0.429 0.427

4	(0.568) 0.571 0.573

Locus 655 :

3 alleles

98.4% missing data

4	(0.291) 0.287 0.295

2	(0.323) 0.319 0.325

1	(0.385) 0.394 0.380

Locus 656 :

3 alleles

97.3% missing data

3	(0.522) 0.523 0.532

2	(0.235) 0.236 0.231

1	(0.243) 0.241 0.238

Locus 657 :

3 alleles

92.2% missing data

1	(0.856) 0.852 0.873

2	(0.080) 0.078 0.073

3	(0.064) 0.071 0.055

Locus 658 :

2 alleles

99.2% missing data

2	(0.525) 0.525 0.526

4	(0.475) 0.475 0.474

Locus 659 :

2 alleles

98.8% missing data

4	(0.465) 0.473 0.470

1	(0.535) 0.527 0.530

Locus 660 :

2 alleles

99.2% missing data

2	(0.396) 0.396 0.398

3	(0.604) 0.604 0.602

Locus 661 :

2 alleles

7.8% missing data

3	(0.985) 0.981 0.995

4	(0.015) 0.019 0.005

Locus 662 :

2 alleles

98.8% missing data

3	(0.385) 0.382 0.383

1	(0.615) 0.618 0.617

Locus 663 :

2 alleles

99.2% missing data

4	(0.523) 0.526 0.514

2	(0.477) 0.474 0.486

Locus 664 :

2 alleles

98.8% missing data

4	(0.319) 0.317 0.318

3	(0.681) 0.683 0.682

Locus 665 :

3 alleles

98.8% missing data

4	(0.210) 0.219 0.207

3	(0.304) 0.296 0.303

2	(0.487) 0.485 0.490

Locus 666 :

2 alleles

99.2% missing data

1	(0.546) 0.540 0.544

2	(0.454) 0.460 0.456

Locus 667 :

2 alleles

99.2% missing data

3	(0.667) 0.661 0.670

4	(0.333) 0.339 0.330

Locus 668 :

3 alleles

98.8% missing data

3	(0.372) 0.362 0.381

2	(0.447) 0.446 0.441

4	(0.181) 0.192 0.178

Locus 669 :

2 alleles

99.2% missing data

1	(0.447) 0.447 0.447

4	(0.553) 0.553 0.553

Locus 670 :

2 alleles

99.2% missing data

4	(0.372) 0.371 0.378

3	(0.628) 0.629 0.622

Locus 671 :

2 alleles

99.2% missing data

3	(0.307) 0.305 0.306

2	(0.693) 0.695 0.694

Locus 672 :

2 alleles

99.2% missing data

1	(0.771) 0.775 0.770

4	(0.229) 0.225 0.230

Locus 673 :

2 alleles

97.3% missing data

1	(0.375) 0.388 0.379

3	(0.625) 0.612 0.621

Locus 674 :

2 alleles

98.8% missing data

2	(0.349) 0.344 0.352

4	(0.651) 0.656 0.648

Locus 675 :

2 alleles

91.4% missing data

4	(0.935) 0.939 0.934

1	(0.065) 0.061 0.066

Locus 676 :

2 alleles

96.9% missing data

3	(0.703) 0.706 0.706

2	(0.297) 0.294 0.294

Locus 677 :

2 alleles

99.2% missing data

4	(0.525) 0.524 0.523

1	(0.475) 0.476 0.477

Locus 678 :

3 alleles

98.8% missing data

2	(0.404) 0.404 0.406

1	(0.140) 0.134 0.144

4	(0.455) 0.462 0.450

Locus 679 :

2 alleles

96.1% missing data

2	(0.872) 0.877 0.874

1	(0.128) 0.123 0.126

Locus 680 :

3 alleles

95.7% missing data

1	(0.122) 0.115 0.125

4	(0.729) 0.739 0.729

2	(0.149) 0.146 0.146

Locus 681 :

2 alleles

93.0% missing data

1	(0.868) 0.869 0.878

4	(0.132) 0.131 0.122

Locus 682 :

2 alleles

99.2% missing data

3	(0.433) 0.431 0.435

4	(0.567) 0.569 0.565

Locus 683 :

2 alleles

99.2% missing data

4	(0.422) 0.422 0.422

1	(0.578) 0.578 0.578

Locus 684 :

2 alleles

99.2% missing data

2	(0.652) 0.652 0.651

3	(0.348) 0.348 0.349

Locus 685 :

2 alleles

99.2% missing data

3	(0.783) 0.787 0.780

2	(0.217) 0.213 0.220

Locus 686 :

2 alleles

99.2% missing data

4	(0.322) 0.325 0.323

3	(0.678) 0.675 0.677

Locus 687 :

3 alleles

98.8% missing data

4	(0.138) 0.135 0.140

2	(0.563) 0.559 0.559

1	(0.299) 0.306 0.301

Locus 688 :

2 alleles

98.8% missing data

2	(0.641) 0.642 0.643

4	(0.359) 0.358 0.357

Locus 689 :

2 alleles

0.0% missing data

2	(0.974) 0.979 0.980

3	(0.026) 0.021 0.020

Locus 690 :

3 alleles

98.8% missing data

1	(0.470) 0.469 0.474

2	(0.345) 0.350 0.338

4	(0.185) 0.181 0.189

Locus 691 :

2 alleles

99.2% missing data

1	(0.523) 0.514 0.530

4	(0.477) 0.486 0.470

Locus 692 :

2 alleles

97.3% missing data

2	(0.857) 0.851 0.867

1	(0.143) 0.149 0.133

Locus 693 :

2 alleles

97.3% missing data

3	(0.673) 0.688 0.666

4	(0.327) 0.312 0.334

Locus 694 :

3 alleles

98.8% missing data

4	(0.144) 0.145 0.149

3	(0.362) 0.360 0.362

2	(0.494) 0.495 0.489

Locus 695 :

2 alleles

14.1% missing data

3	(0.982) 0.986 0.991

2	(0.018) 0.014 0.009

Locus 696 :

2 alleles

99.2% missing data

3	(0.443) 0.441 0.450

1	(0.557) 0.559 0.550

Locus 697 :

2 alleles

98.8% missing data

1	(0.898) 0.893 0.904

3	(0.102) 0.107 0.096

Locus 698 :

2 alleles

98.4% missing data

1	(0.739) 0.744 0.739

4	(0.261) 0.256 0.261

Locus 699 :

2 alleles

99.2% missing data

2	(0.668) 0.670 0.665

3	(0.332) 0.330 0.335

Locus 700 :

2 alleles

8.6% missing data

3	(0.984) 0.986 0.992

1	(0.016) 0.014 0.008

Locus 701 :

2 alleles

99.2% missing data

3	(0.624) 0.620 0.628

4	(0.376) 0.380 0.372

Locus 702 :

2 alleles

99.2% missing data

3	(0.639) 0.634 0.645

1	(0.361) 0.366 0.355

Locus 703 :

3 alleles

96.1% missing data

2	(0.261) 0.262 0.258

3	(0.581) 0.582 0.592

1	(0.158) 0.156 0.150

Locus 704 :

2 alleles

99.2% missing data

2	(0.803) 0.795 0.809

3	(0.197) 0.205 0.191

Locus 705 :

2 alleles

98.0% missing data

2	(0.632) 0.639 0.641

1	(0.368) 0.361 0.359

Locus 706 :

3 alleles

98.8% missing data

1	(0.266) 0.259 0.266

2	(0.483) 0.483 0.485

4	(0.250) 0.259 0.249

Locus 707 :

2 alleles

99.2% missing data

1	(0.782) 0.776 0.782

4	(0.218) 0.224 0.218

Locus 708 :

2 alleles

98.4% missing data

3	(0.874) 0.881 0.867

1	(0.126) 0.119 0.133

Locus 709 :

3 alleles

98.4% missing data

3	(0.428) 0.420 0.442

4	(0.229) 0.239 0.222

2	(0.343) 0.342 0.337

Locus 710 :

2 alleles

95.7% missing data

1	(0.153) 0.156 0.143

3	(0.847) 0.844 0.857

Locus 711 :

2 alleles

99.2% missing data

4	(0.343) 0.345 0.345

3	(0.657) 0.655 0.655

Locus 712 :

2 alleles

98.0% missing data

3	(0.802) 0.808 0.801

1	(0.198) 0.192 0.199

Locus 713 :

2 alleles

97.7% missing data

4	(0.175) 0.177 0.172

3	(0.825) 0.823 0.828

Locus 714 :

3 alleles

98.8% missing data

1	(0.369) 0.366 0.372

2	(0.192) 0.188 0.197

4	(0.439) 0.446 0.431

Locus 715 :

2 alleles

98.8% missing data

2	(0.289) 0.293 0.297

1	(0.711) 0.707 0.703

Locus 716 :

2 alleles

99.2% missing data

2	(0.222) 0.215 0.228

4	(0.778) 0.785 0.772

Locus 717 :

2 alleles

98.8% missing data

1	(0.234) 0.228 0.239

4	(0.766) 0.772 0.761

Locus 718 :

2 alleles

92.6% missing data

1	(0.896) 0.910 0.891

3	(0.104) 0.090 0.109

Locus 719 :

2 alleles

98.8% missing data

2	(0.346) 0.348 0.340

4	(0.654) 0.652 0.660

Locus 720 :

2 alleles

98.8% missing data

4	(0.617) 0.617 0.620

3	(0.383) 0.383 0.380

Locus 721 :

2 alleles

98.8% missing data

4	(0.745) 0.740 0.754

1	(0.255) 0.260 0.246

Locus 722 :

3 alleles

96.9% missing data

4	(0.473) 0.467 0.484

3	(0.255) 0.253 0.255

1	(0.273) 0.280 0.261

Locus 723 :

2 alleles

98.8% missing data

4	(0.592) 0.599 0.586

2	(0.408) 0.401 0.414

Locus 724 :

3 alleles

98.0% missing data

1	(0.182) 0.179 0.182

4	(0.382) 0.374 0.387

2	(0.435) 0.447 0.431

Locus 725 :

2 alleles

98.8% missing data

3	(0.438) 0.447 0.433

2	(0.562) 0.553 0.567

Locus 726 :

2 alleles

98.0% missing data

4	(0.201) 0.197 0.200

1	(0.799) 0.803 0.800

Locus 727 :

2 alleles

99.2% missing data

4	(0.470) 0.469 0.477

3	(0.530) 0.531 0.523

Locus 728 :

2 alleles

99.2% missing data

3	(0.675) 0.666 0.672

4	(0.325) 0.334 0.328

Locus 729 :

2 alleles

98.8% missing data

2	(0.775) 0.774 0.774

3	(0.225) 0.226 0.226

Locus 730 :

2 alleles

98.8% missing data

4	(0.442) 0.441 0.444

3	(0.558) 0.559 0.556

Locus 731 :

3 alleles

98.4% missing data

4	(0.404) 0.404 0.409

1	(0.267) 0.264 0.274

3	(0.329) 0.332 0.318

Locus 732 :

2 alleles

97.3% missing data

1	(0.806) 0.811 0.802

4	(0.194) 0.189 0.198

Locus 733 :

2 alleles

12.1% missing data

3	(0.980) 0.985 0.986

1	(0.020) 0.015 0.014

Locus 734 :

2 alleles

96.5% missing data

4	(0.676) 0.675 0.682

1	(0.324) 0.325 0.318

Locus 735 :

2 alleles

99.2% missing data

1	(0.382) 0.383 0.381

3	(0.618) 0.617 0.619

Locus 736 :

2 alleles

98.8% missing data

4	(0.659) 0.659 0.656

1	(0.341) 0.341 0.344

Locus 737 :

2 alleles

99.2% missing data

4	(0.331) 0.332 0.335

3	(0.669) 0.668 0.665

Locus 738 :

2 alleles

99.2% missing data

3	(0.632) 0.622 0.634

4	(0.368) 0.378 0.366

Locus 739 :

2 alleles

99.2% missing data

3	(0.539) 0.535 0.547

2	(0.461) 0.465 0.453

Locus 740 :

2 alleles

99.2% missing data

1	(0.579) 0.572 0.585

4	(0.421) 0.428 0.415

Locus 741 :

3 alleles

98.8% missing data

3	(0.245) 0.242 0.251

1	(0.340) 0.339 0.344

4	(0.415) 0.419 0.404

Locus 742 :

2 alleles

99.2% missing data

2	(0.445) 0.442 0.454

3	(0.555) 0.558 0.546

Locus 743 :

2 alleles

99.2% missing data

2	(0.568) 0.565 0.569

1	(0.432) 0.435 0.431

Locus 744 :

2 alleles

98.8% missing data

3	(0.211) 0.205 0.214

1	(0.789) 0.795 0.786

Locus 745 :

2 alleles

98.0% missing data

3	(0.734) 0.741 0.735

2	(0.266) 0.259 0.265

Locus 746 :

2 alleles

99.2% missing data

1	(0.394) 0.392 0.393

4	(0.606) 0.608 0.607

Locus 747 :

2 alleles

96.1% missing data

1	(0.818) 0.823 0.816

3	(0.182) 0.177 0.184

Locus 748 :

2 alleles

99.2% missing data

4	(0.658) 0.656 0.664

3	(0.342) 0.344 0.336

Locus 749 :

2 alleles

94.5% missing data

3	(0.144) 0.143 0.144

1	(0.856) 0.857 0.856

Locus 750 :

3 alleles

89.5% missing data

1	(0.859) 0.881 0.834

2	(0.045) 0.037 0.053

4	(0.096) 0.083 0.113

Locus 751 :

2 alleles

99.2% missing data

2	(0.410) 0.410 0.407

4	(0.590) 0.590 0.593

Locus 752 :

2 alleles

99.2% missing data

2	(0.618) 0.622 0.614

3	(0.382) 0.378 0.386

Locus 753 :

2 alleles

99.2% missing data

1	(0.557) 0.560 0.562

4	(0.443) 0.440 0.438

Locus 754 :

2 alleles

98.8% missing data

1	(0.678) 0.679 0.674

4	(0.322) 0.321 0.326

Locus 755 :

2 alleles

98.8% missing data

4	(0.213) 0.219 0.209

3	(0.787) 0.781 0.791

Locus 756 :

2 alleles

99.2% missing data

2	(0.422) 0.422 0.425

4	(0.578) 0.578 0.575

Locus 757 :

2 alleles

97.7% missing data

1	(0.625) 0.619 0.638

3	(0.375) 0.381 0.362

Locus 758 :

2 alleles

99.2% missing data

3	(0.256) 0.257 0.257

2	(0.744) 0.743 0.743

Locus 759 :

2 alleles

98.0% missing data

1	(0.771) 0.766 0.784

4	(0.229) 0.234 0.216

Locus 760 :

2 alleles

96.9% missing data

4	(0.780) 0.776 0.787

1	(0.220) 0.224 0.213

Locus 761 :

2 alleles

99.2% missing data

2	(0.463) 0.463 0.462

1	(0.537) 0.537 0.538

Locus 762 :

2 alleles

98.4% missing data

3	(0.286) 0.281 0.297

1	(0.714) 0.719 0.703

Locus 763 :

2 alleles

91.0% missing data

2	(0.936) 0.950 0.926

4	(0.064) 0.050 0.074

Locus 764 :

2 alleles

99.2% missing data

2	(0.411) 0.411 0.410

1	(0.589) 0.589 0.590

Locus 765 :

2 alleles

98.8% missing data

3	(0.592) 0.593 0.591

2	(0.408) 0.407 0.409

Locus 766 :

2 alleles

98.8% missing data

3	(0.592) 0.596 0.598

1	(0.408) 0.404 0.402

Locus 767 :

2 alleles

98.8% missing data

3	(0.363) 0.355 0.365

2	(0.637) 0.645 0.635

Locus 768 :

2 alleles

99.2% missing data

3	(0.279) 0.281 0.273

2	(0.721) 0.719 0.727

Locus 769 :

2 alleles

98.8% missing data

3	(0.624) 0.618 0.633

1	(0.376) 0.382 0.367

Locus 770 :

2 alleles

99.2% missing data

4	(0.617) 0.625 0.610

1	(0.383) 0.375 0.390

Locus 771 :

2 alleles

99.2% missing data

1	(0.546) 0.546 0.541

4	(0.454) 0.454 0.459

Locus 772 :

2 alleles

98.0% missing data

3	(0.714) 0.705 0.724

1	(0.286) 0.295 0.276

Locus 773 :

2 alleles

99.2% missing data

4	(0.683) 0.678 0.683

1	(0.317) 0.322 0.317

Locus 774 :

2 alleles

97.3% missing data

3	(0.667) 0.665 0.676

1	(0.333) 0.335 0.324

Locus 775 :

2 alleles

99.2% missing data

3	(0.418) 0.425 0.420

1	(0.582) 0.575 0.580

Locus 776 :

2 alleles

95.3% missing data

1	(0.279) 0.269 0.292

4	(0.721) 0.731 0.708

Locus 777 :

2 alleles

99.2% missing data

1	(0.592) 0.597 0.588

4	(0.408) 0.403 0.412

Locus 778 :

2 alleles

3.9% missing data

1	(0.985) 0.980 0.995

4	(0.015) 0.020 0.005

Locus 779 :

3 alleles

94.9% missing data

1	(0.169) 0.174 0.159

2	(0.334) 0.329 0.346

4	(0.497) 0.497 0.495

Locus 780 :

3 alleles

98.0% missing data

2	(0.665) 0.673 0.659

4	(0.242) 0.239 0.243

3	(0.093) 0.088 0.098

Locus 781 :

3 alleles

92.6% missing data

2	(0.203) 0.206 0.205

1	(0.722) 0.722 0.722

3	(0.075) 0.071 0.074

Locus 782 :

2 alleles

98.8% missing data

2	(0.815) 0.816 0.819

1	(0.185) 0.184 0.181

Locus 783 :

2 alleles

99.2% missing data

3	(0.314) 0.318 0.315

4	(0.686) 0.682 0.685

Locus 784 :

2 alleles

99.2% missing data

4	(0.205) 0.200 0.213

3	(0.795) 0.800 0.787

Locus 785 :

2 alleles

99.2% missing data

4	(0.429) 0.424 0.430

1	(0.571) 0.576 0.570

Locus 786 :

2 alleles

97.7% missing data

1	(0.129) 0.131 0.130

4	(0.871) 0.869 0.870

Locus 787 :

2 alleles

99.2% missing data

1	(0.415) 0.420 0.412

4	(0.585) 0.580 0.588

Locus 788 :

3 alleles

47.7% missing data

2	(0.939) 0.944 0.962

1	(0.032) 0.025 0.020

4	(0.029) 0.031 0.018

Locus 789 :

2 alleles

91.0% missing data

1	(0.897) 0.915 0.886

4	(0.103) 0.085 0.114

Locus 790 :

2 alleles

98.8% missing data

2	(0.838) 0.839 0.833

1	(0.162) 0.161 0.167

Locus 791 :

2 alleles

93.8% missing data

1	(0.870) 0.876 0.873

2	(0.130) 0.124 0.127

Locus 792 :

2 alleles

98.4% missing data

2	(0.847) 0.850 0.843

1	(0.153) 0.150 0.157

Locus 793 :

2 alleles

99.2% missing data

4	(0.432) 0.429 0.431

1	(0.568) 0.571 0.569

Locus 794 :

2 alleles

98.8% missing data

4	(0.490) 0.485 0.500

2	(0.510) 0.515 0.500

Locus 795 :

3 alleles

98.0% missing data

4	(0.125) 0.122 0.127

3	(0.335) 0.334 0.329

2	(0.540) 0.544 0.543

Locus 796 :

2 alleles

14.1% missing data

1	(0.983) 0.986 0.991

4	(0.017) 0.014 0.009

Locus 797 :

2 alleles

96.9% missing data

3	(0.793) 0.803 0.784

4	(0.207) 0.197 0.216

Locus 798 :

2 alleles

99.2% missing data

4	(0.647) 0.640 0.654

1	(0.353) 0.360 0.346

Locus 799 :

2 alleles

98.4% missing data

2	(0.616) 0.621 0.612

1	(0.384) 0.379 0.388

Locus 800 :

4 alleles

95.7% missing data

1	(0.413) 0.403 0.435

2	(0.271) 0.275 0.261

3	(0.219) 0.229 0.202

4	(0.097) 0.092 0.103

Locus 801 :

2 alleles

98.8% missing data

4	(0.363) 0.362 0.349

2	(0.637) 0.638 0.651

Locus 802 :

2 alleles

98.8% missing data

1	(0.705) 0.697 0.711

2	(0.295) 0.303 0.289

Locus 803 :

3 alleles

97.7% missing data

2	(0.309) 0.307 0.311

1	(0.381) 0.385 0.389

3	(0.311) 0.308 0.301

Locus 804 :

2 alleles

96.9% missing data

1	(0.513) 0.514 0.520

2	(0.487) 0.486 0.480

Locus 805 :

2 alleles

98.8% missing data

4	(0.648) 0.654 0.648

1	(0.352) 0.346 0.352

Locus 806 :

2 alleles

99.2% missing data

1	(0.475) 0.475 0.483

4	(0.525) 0.525 0.517

Locus 807 :

2 alleles

98.8% missing data

1	(0.734) 0.728 0.742

4	(0.266) 0.272 0.258

Locus 808 :

2 alleles

99.2% missing data

1	(0.415) 0.414 0.419

4	(0.585) 0.586 0.581

Locus 809 :

2 alleles

99.2% missing data

1	(0.714) 0.712 0.712

4	(0.286) 0.288 0.288

Locus 810 :

2 alleles

99.2% missing data

3	(0.286) 0.289 0.289

1	(0.714) 0.711 0.711

Locus 811 :

2 alleles

98.0% missing data

1	(0.493) 0.480 0.498

2	(0.507) 0.520 0.502

Locus 812 :

2 alleles

99.2% missing data

3	(0.302) 0.304 0.304

1	(0.698) 0.696 0.696

Locus 813 :

2 alleles

97.7% missing data

1	(0.201) 0.212 0.193

2	(0.799) 0.788 0.807

Locus 814 :

3 alleles

98.8% missing data

3	(0.596) 0.602 0.593

4	(0.220) 0.215 0.217

2	(0.184) 0.183 0.190

Locus 815 :

2 alleles

99.2% missing data

2	(0.646) 0.645 0.647

4	(0.354) 0.355 0.353

Locus 816 :

2 alleles

91.8% missing data

1	(0.846) 0.832 0.864

4	(0.154) 0.168 0.136

Locus 817 :

2 alleles

98.8% missing data

4	(0.673) 0.667 0.671

1	(0.327) 0.333 0.329

Locus 818 :

2 alleles

99.2% missing data

1	(0.281) 0.280 0.281

2	(0.719) 0.720 0.719

Locus 819 :

2 alleles

97.3% missing data

1	(0.764) 0.762 0.769

2	(0.236) 0.238 0.231

Locus 820 :

2 alleles

99.2% missing data

3	(0.342) 0.338 0.347

2	(0.658) 0.662 0.653

Locus 821 :

2 alleles

99.2% missing data

1	(0.180) 0.176 0.183

4	(0.820) 0.824 0.817

Locus 822 :

2 alleles

96.5% missing data

4	(0.814) 0.827 0.807

2	(0.186) 0.173 0.193

Locus 823 :

2 alleles

99.2% missing data

2	(0.285) 0.284 0.286

1	(0.715) 0.716 0.714

Locus 824 :

2 alleles

99.2% missing data

4	(0.497) 0.497 0.491

3	(0.503) 0.503 0.509

Locus 825 :

2 alleles

98.8% missing data

2	(0.227) 0.221 0.227

4	(0.773) 0.779 0.773

Locus 826 :

2 alleles

10.2% missing data

2	(0.985) 0.976 0.996

4	(0.015) 0.024 0.004

Locus 827 :

2 alleles

99.2% missing data

4	(0.486) 0.487 0.483

3	(0.514) 0.513 0.517

Locus 828 :

2 alleles

99.2% missing data

4	(0.860) 0.862 0.862

1	(0.140) 0.138 0.138

Locus 829 :

2 alleles

99.2% missing data

1	(0.565) 0.567 0.565

2	(0.435) 0.433 0.435

Locus 830 :

2 alleles

98.0% missing data

1	(0.672) 0.664 0.688

3	(0.328) 0.336 0.312

Locus 831 :

2 alleles

99.2% missing data

1	(0.538) 0.531 0.544

2	(0.462) 0.469 0.456

Locus 832 :

2 alleles

99.2% missing data

3	(0.464) 0.469 0.465

1	(0.536) 0.531 0.535

Locus 833 :

3 alleles

97.7% missing data

1	(0.647) 0.652 0.651

4	(0.145) 0.144 0.142

3	(0.207) 0.204 0.207

Locus 834 :

2 alleles

99.2% missing data

3	(0.394) 0.394 0.395

2	(0.606) 0.606 0.605

Locus 835 :

2 alleles

98.4% missing data

2	(0.619) 0.618 0.622

3	(0.381) 0.382 0.378

Locus 836 :

3 alleles

98.8% missing data

3	(0.179) 0.180 0.186

1	(0.467) 0.457 0.465

4	(0.354) 0.363 0.350

Locus 837 :

2 alleles

99.2% missing data

2	(0.597) 0.597 0.599

1	(0.403) 0.403 0.401

Locus 838 :

2 alleles

99.2% missing data

3	(0.418) 0.427 0.409

2	(0.582) 0.573 0.591

Locus 839 :

2 alleles

94.9% missing data

1	(0.849) 0.848 0.851

4	(0.151) 0.152 0.149

Locus 840 :

2 alleles

99.2% missing data

4	(0.318) 0.313 0.333

3	(0.682) 0.687 0.667

Locus 841 :

2 alleles

92.6% missing data

4	(0.868) 0.863 0.892

3	(0.132) 0.137 0.108

Locus 842 :

2 alleles

99.2% missing data

3	(0.387) 0.386 0.387

1	(0.613) 0.614 0.613

Locus 843 :

2 alleles

98.8% missing data

4	(0.711) 0.704 0.710

2	(0.289) 0.296 0.290

Locus 844 :

2 alleles

99.2% missing data

1	(0.752) 0.748 0.757

4	(0.248) 0.252 0.243

Locus 845 :

2 alleles

99.2% missing data

3	(0.294) 0.290 0.296

2	(0.706) 0.710 0.704

Locus 846 :

2 alleles

99.2% missing data

4	(0.395) 0.398 0.390

1	(0.605) 0.602 0.610

Locus 847 :

2 alleles

99.2% missing data

4	(0.451) 0.446 0.457

2	(0.549) 0.554 0.543

Locus 848 :

2 alleles

98.8% missing data

4	(0.565) 0.562 0.574

2	(0.435) 0.438 0.426

Locus 849 :

3 alleles

91.8% missing data

1	(0.697) 0.684 0.719

2	(0.209) 0.213 0.205

3	(0.093) 0.103 0.076

Locus 850 :

3 alleles

96.9% missing data

1	(0.671) 0.680 0.665

2	(0.182) 0.176 0.185

3	(0.147) 0.143 0.150

Locus 851 :

2 alleles

98.8% missing data

4	(0.833) 0.835 0.826

1	(0.167) 0.165 0.174

Locus 852 :

2 alleles

93.4% missing data

1	(0.669) 0.661 0.677

2	(0.331) 0.339 0.323

Locus 853 :

2 alleles

98.8% missing data

1	(0.564) 0.568 0.566

2	(0.436) 0.432 0.434

Locus 854 :

2 alleles

99.2% missing data

3	(0.464) 0.464 0.463

1	(0.536) 0.536 0.537

Locus 855 :

2 alleles

99.2% missing data

3	(0.689) 0.684 0.690

1	(0.311) 0.316 0.310

Locus 856 :

2 alleles

99.2% missing data

2	(0.377) 0.385 0.370

3	(0.623) 0.615 0.630

Locus 857 :

2 alleles

97.3% missing data

4	(0.765) 0.766 0.771

2	(0.235) 0.234 0.229

Locus 858 :

2 alleles

99.2% missing data

4	(0.388) 0.389 0.390

1	(0.612) 0.611 0.610

Locus 859 :

3 alleles

98.4% missing data

2	(0.372) 0.362 0.375

1	(0.346) 0.354 0.349

4	(0.282) 0.284 0.276

Locus 860 :

2 alleles

99.2% missing data

1	(0.399) 0.398 0.403

2	(0.601) 0.602 0.597

Locus 861 :

2 alleles

99.2% missing data

1	(0.230) 0.230 0.236

4	(0.770) 0.770 0.764

Locus 862 :

2 alleles

99.2% missing data

4	(0.509) 0.511 0.512

2	(0.491) 0.489 0.488

Locus 863 :

3 alleles

98.8% missing data

3	(0.336) 0.331 0.340

1	(0.221) 0.219 0.224

2	(0.443) 0.450 0.436

Locus 864 :

2 alleles

95.3% missing data

1	(0.627) 0.628 0.628

2	(0.373) 0.372 0.372

Locus 865 :

2 alleles

96.1% missing data

3	(0.240) 0.230 0.242

4	(0.760) 0.770 0.758

Locus 866 :

2 alleles

98.4% missing data

1	(0.643) 0.648 0.639

4	(0.357) 0.352 0.361

Locus 867 :

2 alleles

1.6% missing data

4	(0.988) 0.983 0.997

3	(0.012) 0.017 0.003

Locus 868 :

2 alleles

99.2% missing data

1	(0.492) 0.498 0.490

2	(0.508) 0.502 0.510

Locus 869 :

2 alleles

98.8% missing data

3	(0.735) 0.726 0.741

4	(0.265) 0.274 0.259

Locus 870 :

2 alleles

99.2% missing data

4	(0.746) 0.745 0.747

2	(0.254) 0.255 0.253

Locus 871 :

2 alleles

99.2% missing data

4	(0.203) 0.206 0.202

3	(0.797) 0.794 0.798

Locus 872 :

2 alleles

99.2% missing data

4	(0.381) 0.384 0.388

1	(0.619) 0.616 0.612

Locus 873 :

2 alleles

98.4% missing data

4	(0.506) 0.508 0.505

1	(0.494) 0.492 0.495

Locus 874 :

2 alleles

96.5% missing data

4	(0.185) 0.184 0.178

2	(0.815) 0.816 0.822

Locus 875 :

2 alleles

98.8% missing data

4	(0.497) 0.501 0.496

2	(0.503) 0.499 0.504

Locus 876 :

2 alleles

99.2% missing data

3	(0.268) 0.264 0.276

4	(0.732) 0.736 0.724

Locus 877 :

2 alleles

97.3% missing data

1	(0.782) 0.786 0.784

3	(0.218) 0.214 0.216

Locus 878 :

2 alleles

99.2% missing data

2	(0.478) 0.468 0.485

4	(0.522) 0.532 0.515

Locus 879 :

2 alleles

98.8% missing data

2	(0.429) 0.439 0.431

4	(0.571) 0.561 0.569

Locus 880 :

2 alleles

90.6% missing data

4	(0.914) 0.932 0.905

1	(0.086) 0.068 0.095

Locus 881 :

2 alleles

99.2% missing data

4	(0.365) 0.371 0.369

3	(0.635) 0.629 0.631

Locus 882 :

2 alleles

99.2% missing data

1	(0.363) 0.359 0.364

3	(0.637) 0.641 0.636

Locus 883 :

2 alleles

99.2% missing data

2	(0.544) 0.548 0.540

1	(0.456) 0.452 0.460

Locus 884 :

2 alleles

98.8% missing data

4	(0.625) 0.621 0.628

3	(0.375) 0.379 0.372

Locus 885 :

2 alleles

99.2% missing data

4	(0.702) 0.702 0.691

1	(0.298) 0.298 0.309

Locus 886 :

2 alleles

98.8% missing data

3	(0.542) 0.542 0.541

1	(0.458) 0.458 0.459

Locus 887 :

2 alleles

98.4% missing data

2	(0.626) 0.631 0.632

4	(0.374) 0.369 0.368

Locus 888 :

2 alleles

98.8% missing data

3	(0.241) 0.240 0.247

1	(0.759) 0.760 0.753

Locus 889 :

2 alleles

99.2% missing data

2	(0.621) 0.619 0.612

4	(0.379) 0.381 0.388

Locus 890 :

2 alleles

99.2% missing data

1	(0.642) 0.637 0.643

2	(0.358) 0.363 0.357

Locus 891 :

2 alleles

96.5% missing data

2	(0.793) 0.798 0.796

1	(0.207) 0.202 0.204

Locus 892 :

2 alleles

99.2% missing data

3	(0.494) 0.493 0.491

4	(0.506) 0.507 0.509

Locus 893 :

2 alleles

98.8% missing data

4	(0.590) 0.591 0.593

1	(0.410) 0.409 0.407

Locus 894 :

2 alleles

99.2% missing data

3	(0.793) 0.795 0.789

1	(0.207) 0.205 0.211

Locus 895 :

2 alleles

99.2% missing data

1	(0.669) 0.665 0.672

4	(0.331) 0.335 0.328

Locus 896 :

2 alleles

93.0% missing data

4	(0.913) 0.911 0.920

1	(0.087) 0.089 0.080

Locus 897 :

2 alleles

99.2% missing data

2	(0.364) 0.374 0.364

3	(0.636) 0.626 0.636

Locus 898 :

2 alleles

96.9% missing data

2	(0.189) 0.181 0.196

3	(0.811) 0.819 0.804

Locus 899 :

3 alleles

98.8% missing data

3	(0.196) 0.198 0.198

4	(0.573) 0.570 0.571

2	(0.231) 0.232 0.232

Locus 900 :

2 alleles

99.2% missing data

1	(0.532) 0.523 0.538

3	(0.468) 0.477 0.462

Locus 901 :

2 alleles

98.8% missing data

2	(0.372) 0.362 0.380

4	(0.628) 0.638 0.620

Locus 902 :

2 alleles

99.2% missing data

4	(0.729) 0.730 0.725

2	(0.271) 0.270 0.275

Locus 903 :

2 alleles

99.2% missing data

2	(0.498) 0.504 0.492

3	(0.502) 0.496 0.508

Locus 904 :

2 alleles

96.5% missing data

4	(0.169) 0.161 0.169

1	(0.831) 0.839 0.831

Locus 905 :

2 alleles

99.2% missing data

1	(0.454) 0.452 0.459

2	(0.546) 0.548 0.541

Locus 906 :

2 alleles

99.2% missing data

2	(0.497) 0.498 0.496

1	(0.503) 0.502 0.504

Locus 907 :

2 alleles

99.2% missing data

4	(0.365) 0.362 0.371

3	(0.635) 0.638 0.629

Locus 908 :

3 alleles

96.5% missing data

1	(0.160) 0.161 0.154

3	(0.698) 0.688 0.717

4	(0.142) 0.150 0.129

Locus 909 :

3 alleles

97.3% missing data

3	(0.082) 0.082 0.082

1	(0.383) 0.391 0.371

2	(0.535) 0.527 0.546

Locus 910 :

2 alleles

99.2% missing data

4	(0.557) 0.547 0.562

1	(0.443) 0.453 0.438

Locus 911 :

2 alleles

90.6% missing data

3	(0.924) 0.938 0.910

4	(0.076) 0.062 0.090

Locus 912 :

2 alleles

99.2% missing data

2	(0.685) 0.685 0.688

4	(0.315) 0.315 0.312

Locus 913 :

2 alleles

99.2% missing data

4	(0.664) 0.657 0.666

3	(0.336) 0.343 0.334

Locus 914 :

2 alleles

98.8% missing data

1	(0.467) 0.472 0.459

3	(0.533) 0.528 0.541

Locus 915 :

2 alleles

98.4% missing data

2	(0.439) 0.435 0.444

4	(0.561) 0.565 0.556

Locus 916 :

2 alleles

99.2% missing data

3	(0.367) 0.363 0.368

2	(0.633) 0.637 0.632

Locus 917 :

3 alleles

94.5% missing data

2	(0.470) 0.487 0.469

4	(0.389) 0.387 0.391

1	(0.141) 0.126 0.140

Locus 918 :

3 alleles

97.7% missing data

4	(0.165) 0.164 0.169

2	(0.514) 0.527 0.508

3	(0.321) 0.309 0.323

Locus 919 :

2 alleles

99.2% missing data

3	(0.571) 0.564 0.577

4	(0.429) 0.436 0.423

Locus 920 :

2 alleles

98.8% missing data

4	(0.274) 0.269 0.281

3	(0.726) 0.731 0.719

Locus 921 :

2 alleles

99.2% missing data

4	(0.834) 0.836 0.830

3	(0.166) 0.164 0.170

Locus 922 :

2 alleles

99.2% missing data

4	(0.653) 0.650 0.652

3	(0.347) 0.350 0.348

Locus 923 :

2 alleles

97.7% missing data

1	(0.219) 0.205 0.218

4	(0.781) 0.795 0.782

Locus 924 :

2 alleles

98.8% missing data

4	(0.617) 0.615 0.622

1	(0.383) 0.385 0.378

Locus 925 :

2 alleles

98.8% missing data

3	(0.477) 0.484 0.475

2	(0.523) 0.516 0.525

Locus 926 :

2 alleles

96.5% missing data

4	(0.912) 0.913 0.913

1	(0.088) 0.087 0.087

Locus 927 :

2 alleles

98.8% missing data

4	(0.677) 0.671 0.686

2	(0.323) 0.329 0.314

Locus 928 :

3 alleles

98.8% missing data

1	(0.395) 0.400 0.387

4	(0.341) 0.333 0.344

3	(0.265) 0.267 0.269

Locus 929 :

2 alleles

91.0% missing data

1	(0.930) 0.943 0.925

4	(0.070) 0.057 0.075

Locus 930 :

2 alleles

99.2% missing data

2	(0.693) 0.693 0.692

4	(0.307) 0.307 0.308

Locus 931 :

2 alleles

98.8% missing data

4	(0.336) 0.328 0.348

3	(0.664) 0.672 0.652

Locus 932 :

2 alleles

99.2% missing data

2	(0.484) 0.485 0.488

4	(0.516) 0.515 0.512

Locus 933 :

3 alleles

90.6% missing data

1	(0.856) 0.880 0.850

4	(0.074) 0.058 0.083

2	(0.071) 0.063 0.067

Locus 934 :

3 alleles

96.9% missing data

4	(0.298) 0.297 0.289

3	(0.101) 0.103 0.099

1	(0.601) 0.599 0.612

Locus 935 :

2 alleles

98.8% missing data

1	(0.504) 0.506 0.508

3	(0.496) 0.494 0.492

Locus 936 :

2 alleles

93.0% missing data

3	(0.903) 0.904 0.911

1	(0.097) 0.096 0.089

Locus 937 :

2 alleles

99.2% missing data

2	(0.394) 0.390 0.394

4	(0.606) 0.610 0.606

Locus 938 :

2 alleles

98.8% missing data

4	(0.782) 0.790 0.779

1	(0.218) 0.210 0.221

Locus 939 :

2 alleles

98.4% missing data

1	(0.423) 0.405 0.434

3	(0.577) 0.595 0.566

Locus 940 :

2 alleles

98.4% missing data

3	(0.505) 0.513 0.506

1	(0.495) 0.487 0.494

Locus 941 :

2 alleles

99.2% missing data

1	(0.454) 0.449 0.458

4	(0.546) 0.551 0.542

Locus 942 :

4 alleles

98.4% missing data

4	(0.245) 0.242 0.247

3	(0.343) 0.344 0.333

1	(0.309) 0.313 0.309

2	(0.104) 0.101 0.111

Locus 943 :

2 alleles

95.7% missing data

3	(0.311) 0.291 0.327

1	(0.689) 0.709 0.673

Locus 944 :

2 alleles

96.5% missing data

1	(0.839) 0.844 0.835

2	(0.161) 0.156 0.165

Locus 945 :

2 alleles

99.2% missing data

2	(0.400) 0.394 0.405

4	(0.600) 0.606 0.595

Locus 946 :

2 alleles

96.1% missing data

3	(0.876) 0.886 0.868

1	(0.124) 0.114 0.132

Locus 947 :

2 alleles

99.2% missing data

3	(0.358) 0.356 0.362

4	(0.642) 0.644 0.638

Locus 948 :

2 alleles

99.2% missing data

3	(0.362) 0.366 0.363

1	(0.638) 0.634 0.637

Locus 949 :

2 alleles

99.2% missing data

3	(0.659) 0.662 0.656

4	(0.341) 0.338 0.344

Locus 950 :

2 alleles

99.2% missing data

1	(0.692) 0.687 0.697

4	(0.308) 0.313 0.303

Locus 951 :

2 alleles

99.2% missing data

1	(0.359) 0.363 0.363

4	(0.641) 0.637 0.637

Locus 952 :

2 alleles

99.2% missing data

2	(0.316) 0.316 0.318

3	(0.684) 0.684 0.682

Locus 953 :

2 alleles

96.5% missing data

4	(0.170) 0.157 0.178

2	(0.830) 0.843 0.822

Locus 954 :

2 alleles

99.2% missing data

2	(0.359) 0.355 0.364

1	(0.641) 0.645 0.636

Locus 955 :

2 alleles

98.4% missing data

2	(0.615) 0.621 0.605

1	(0.385) 0.379 0.395

Locus 956 :

2 alleles

99.2% missing data

3	(0.276) 0.275 0.278

4	(0.724) 0.725 0.722

Locus 957 :

2 alleles

99.2% missing data

4	(0.635) 0.642 0.629

2	(0.365) 0.358 0.371

Locus 958 :

2 alleles

99.2% missing data

1	(0.555) 0.552 0.559

4	(0.445) 0.448 0.441

Locus 959 :

2 alleles

99.2% missing data

1	(0.614) 0.604 0.612

2	(0.386) 0.396 0.388

Locus 960 :

2 alleles

99.2% missing data

4	(0.458) 0.459 0.458

1	(0.542) 0.541 0.542

Locus 961 :

2 alleles

99.2% missing data

1	(0.313) 0.313 0.317

4	(0.687) 0.687 0.683

Locus 962 :

2 alleles

96.5% missing data

4	(0.908) 0.906 0.911

3	(0.092) 0.094 0.089

Locus 963 :

2 alleles

99.2% missing data

3	(0.409) 0.407 0.407

4	(0.591) 0.593 0.593

Locus 964 :

2 alleles

98.8% missing data

1	(0.609) 0.609 0.612

3	(0.391) 0.391 0.388

Locus 965 :

2 alleles

99.2% missing data

3	(0.213) 0.211 0.212

4	(0.787) 0.789 0.788

Locus 966 :

2 alleles

99.2% missing data

3	(0.316) 0.321 0.318

4	(0.684) 0.679 0.682

Locus 967 :

2 alleles

90.6% missing data

3	(0.102) 0.086 0.122

4	(0.898) 0.914 0.878

Locus 968 :

2 alleles

99.2% missing data

4	(0.288) 0.294 0.285

1	(0.712) 0.706 0.715

Locus 969 :

2 alleles

99.2% missing data

4	(0.580) 0.577 0.581

2	(0.420) 0.423 0.419

Locus 970 :

2 alleles

93.8% missing data

1	(0.708) 0.706 0.714

2	(0.292) 0.294 0.286

Locus 971 :

4 alleles

96.5% missing data

2	(0.349) 0.352 0.351

1	(0.381) 0.380 0.382

3	(0.120) 0.115 0.124

4	(0.150) 0.153 0.143

Locus 972 :

3 alleles

98.0% missing data

1	(0.439) 0.444 0.442

3	(0.224) 0.227 0.224

2	(0.337) 0.329 0.334

Locus 973 :

3 alleles

98.0% missing data

1	(0.486) 0.484 0.484

2	(0.348) 0.353 0.344

4	(0.167) 0.163 0.172

Locus 974 :

3 alleles

98.8% missing data

2	(0.453) 0.455 0.449

3	(0.154) 0.155 0.161

4	(0.393) 0.389 0.389

Locus 975 :

2 alleles

98.8% missing data

1	(0.384) 0.372 0.388

4	(0.616) 0.628 0.612

Locus 976 :

2 alleles

99.2% missing data

2	(0.424) 0.422 0.424

3	(0.576) 0.578 0.576

Locus 977 :

2 alleles

88.3% missing data

2	(0.273) 0.263 0.276

1	(0.727) 0.737 0.724

Locus 978 :

2 alleles

98.8% missing data

2	(0.284) 0.289 0.288

1	(0.716) 0.711 0.712

Locus 979 :

2 alleles

97.7% missing data

1	(0.855) 0.860 0.851

2	(0.145) 0.140 0.149

Locus 980 :

2 alleles

99.2% missing data

1	(0.256) 0.255 0.258

2	(0.744) 0.745 0.742

Locus 981 :

3 alleles

97.7% missing data

1	(0.415) 0.407 0.424

2	(0.330) 0.333 0.323

4	(0.255) 0.260 0.253

Locus 982 :

2 alleles

98.8% missing data

1	(0.790) 0.794 0.782

3	(0.210) 0.206 0.218

Locus 983 :

2 alleles

99.2% missing data

3	(0.374) 0.375 0.372

4	(0.626) 0.625 0.628

Locus 984 :

3 alleles

98.8% missing data

1	(0.410) 0.411 0.404

2	(0.278) 0.278 0.283

4	(0.311) 0.310 0.313

Locus 985 :

2 alleles

98.8% missing data

4	(0.576) 0.584 0.574

1	(0.424) 0.416 0.426

Locus 986 :

2 alleles

99.2% missing data

1	(0.628) 0.628 0.629

3	(0.372) 0.372 0.371

Locus 987 :

2 alleles

99.2% missing data

1	(0.794) 0.784 0.799

2	(0.206) 0.216 0.201

Locus 988 :

2 alleles

98.8% missing data

3	(0.601) 0.599 0.610

4	(0.399) 0.401 0.390

Locus 989 :

4 alleles

96.9% missing data

3	(0.259) 0.254 0.256

4	(0.129) 0.127 0.127

1	(0.480) 0.475 0.497

2	(0.132) 0.144 0.121

Locus 990 :

2 alleles

97.3% missing data

1	(0.524) 0.518 0.532

2	(0.476) 0.482 0.468

Locus 991 :

3 alleles

96.9% missing data

1	(0.645) 0.653 0.637

4	(0.132) 0.129 0.135

2	(0.223) 0.218 0.228

Locus 992 :

2 alleles

99.2% missing data

2	(0.383) 0.388 0.391

1	(0.617) 0.612 0.609

Locus 993 :

2 alleles

98.8% missing data

4	(0.694) 0.683 0.696

2	(0.306) 0.317 0.304

Locus 994 :

2 alleles

95.3% missing data

4	(0.858) 0.851 0.873

2	(0.142) 0.149 0.127

Locus 995 :

3 alleles

98.0% missing data

2	(0.315) 0.323 0.320

3	(0.218) 0.210 0.220

1	(0.467) 0.467 0.460

Locus 996 :

2 alleles

97.7% missing data

2	(0.360) 0.362 0.359

1	(0.640) 0.638 0.641

Locus 997 :

3 alleles

98.0% missing data

2	(0.497) 0.497 0.495

4	(0.178) 0.185 0.175

1	(0.325) 0.319 0.330

Locus 998 :

2 alleles

99.2% missing data

3	(0.577) 0.580 0.581

1	(0.423) 0.420 0.419

Locus 999 :

2 alleles

99.2% missing data

1	(0.448) 0.447 0.451

3	(0.552) 0.553 0.549

Locus 1000 :

2 alleles

0.0% missing data

2	(0.989) 0.993 0.994

3	(0.011) 0.007 0.006

Locus 1001 :

2 alleles

99.2% missing data

3	(0.568) 0.562 0.568

4	(0.432) 0.438 0.432

Locus 1002 :

2 alleles

98.4% missing data

1	(0.386) 0.383 0.378

2	(0.614) 0.617 0.622

Locus 1003 :

2 alleles

99.2% missing data

3	(0.605) 0.597 0.606

4	(0.395) 0.403 0.394

Locus 1004 :

2 alleles

96.5% missing data

1	(0.174) 0.163 0.181

4	(0.826) 0.837 0.819

Locus 1005 :

2 alleles

99.2% missing data

3	(0.625) 0.620 0.629

2	(0.375) 0.380 0.371

Locus 1006 :

2 alleles

99.2% missing data

1	(0.243) 0.241 0.245

4	(0.757) 0.759 0.755

Locus 1007 :

3 alleles

98.8% missing data

4	(0.322) 0.319 0.327

2	(0.495) 0.491 0.496

1	(0.183) 0.190 0.177

Locus 1008 :

2 alleles

99.2% missing data

2	(0.471) 0.473 0.470

3	(0.529) 0.527 0.530

Locus 1009 :

3 alleles

98.4% missing data

4	(0.315) 0.308 0.323

1	(0.354) 0.351 0.354

3	(0.332) 0.342 0.323

Locus 1010 :

2 alleles

99.2% missing data

1	(0.604) 0.606 0.600

3	(0.396) 0.394 0.400

Locus 1011 :

2 alleles

95.7% missing data

1	(0.897) 0.897 0.901

2	(0.103) 0.103 0.099

Locus 1012 :

2 alleles

97.7% missing data

1	(0.724) 0.723 0.730

4	(0.276) 0.277 0.270

Locus 1013 :

2 alleles

99.2% missing data

2	(0.442) 0.443 0.447

1	(0.558) 0.557 0.553

Locus 1014 :

2 alleles

94.5% missing data

1	(0.666) 0.658 0.675

2	(0.334) 0.342 0.325

Locus 1015 :

2 alleles

96.1% missing data

1	(0.862) 0.871 0.859

2	(0.138) 0.129 0.141

Locus 1016 :

2 alleles

99.2% missing data

2	(0.399) 0.401 0.396

4	(0.601) 0.599 0.604

Locus 1017 :

3 alleles

96.9% missing data

2	(0.394) 0.380 0.402

1	(0.461) 0.469 0.461

3	(0.144) 0.151 0.136

Locus 1018 :

2 alleles

98.8% missing data

1	(0.808) 0.810 0.802

3	(0.192) 0.190 0.198

Locus 1019 :

2 alleles

99.2% missing data

4	(0.693) 0.694 0.691

1	(0.307) 0.306 0.309

Locus 1020 :

2 alleles

97.7% missing data

1	(0.650) 0.653 0.651

2	(0.350) 0.347 0.349

Locus 1021 :

2 alleles

99.2% missing data

1	(0.270) 0.265 0.276

4	(0.730) 0.735 0.724

Locus 1022 :

3 alleles

96.5% missing data

1	(0.502) 0.513 0.492

3	(0.308) 0.307 0.312

2	(0.190) 0.180 0.196

Locus 1023 :

2 alleles

96.9% missing data

2	(0.622) 0.623 0.619

1	(0.378) 0.377 0.381

Locus 1024 :

2 alleles

99.2% missing data

2	(0.472) 0.475 0.476

4	(0.528) 0.525 0.524

Locus 1025 :

2 alleles

99.2% missing data

3	(0.445) 0.449 0.442

2	(0.555) 0.551 0.558

Locus 1026 :

2 alleles

99.2% missing data

4	(0.329) 0.326 0.327

1	(0.671) 0.674 0.673

Locus 1027 :

2 alleles

89.1% missing data

1	(0.925) 0.937 0.921

2	(0.075) 0.063 0.079

Locus 1028 :

3 alleles

97.7% missing data

2	(0.700) 0.712 0.690

4	(0.088) 0.084 0.093

1	(0.212) 0.204 0.216

Locus 1029 :

2 alleles

99.2% missing data

4	(0.600) 0.599 0.597

3	(0.400) 0.401 0.403

Locus 1030 :

2 alleles

98.0% missing data

1	(0.562) 0.573 0.551

2	(0.438) 0.427 0.449

Locus 1031 :

2 alleles

98.8% missing data

4	(0.735) 0.728 0.739

1	(0.265) 0.272 0.261

Locus 1032 :

3 alleles

98.4% missing data

1	(0.613) 0.610 0.613

3	(0.176) 0.184 0.174

2	(0.211) 0.207 0.213

Locus 1033 :

2 alleles

98.4% missing data

2	(0.745) 0.751 0.751

4	(0.255) 0.249 0.249

Locus 1034 :

2 alleles

4.7% missing data

4	(0.983) 0.987 0.992

2	(0.017) 0.013 0.008

Locus 1035 :

3 alleles

77.7% missing data

2	(0.888) 0.901 0.892

1	(0.075) 0.066 0.071

4	(0.037) 0.032 0.036

Locus 1036 :

2 alleles

99.2% missing data

1	(0.263) 0.258 0.266

3	(0.737) 0.742 0.734

Locus 1037 :

2 alleles

98.0% missing data

4	(0.310) 0.301 0.309

1	(0.690) 0.699 0.691

Locus 1038 :

2 alleles

96.5% missing data

4	(0.822) 0.829 0.824

2	(0.178) 0.171 0.176

Locus 1039 :

2 alleles

99.2% missing data

4	(0.183) 0.178 0.188

2	(0.817) 0.822 0.812

Locus 1040 :

2 alleles

96.1% missing data

1	(0.771) 0.778 0.779

3	(0.229) 0.222 0.221

Locus 1041 :

2 alleles

97.3% missing data

2	(0.690) 0.703 0.683

1	(0.310) 0.297 0.317

Locus 1042 :

2 alleles

98.8% missing data

4	(0.701) 0.701 0.700

3	(0.299) 0.299 0.300

Locus 1043 :

2 alleles

93.8% missing data

1	(0.885) 0.887 0.891

3	(0.115) 0.113 0.109

Locus 1044 :

2 alleles

96.5% missing data

2	(0.143) 0.132 0.151

1	(0.857) 0.868 0.849

Locus 1045 :

2 alleles

99.2% missing data

3	(0.516) 0.519 0.515

4	(0.484) 0.481 0.485

Locus 1046 :

2 alleles

98.0% missing data

2	(0.725) 0.726 0.725

1	(0.275) 0.274 0.275

Locus 1047 :

2 alleles

98.8% missing data

4	(0.675) 0.671 0.674

2	(0.325) 0.329 0.326

Locus 1048 :

2 alleles

99.2% missing data

2	(0.231) 0.230 0.234

4	(0.769) 0.770 0.766

Locus 1049 :

2 alleles

99.2% missing data

2	(0.443) 0.441 0.449

3	(0.557) 0.559 0.551

Locus 1050 :

2 alleles

99.2% missing data

4	(0.716) 0.715 0.713

2	(0.284) 0.285 0.287

Locus 1051 :

2 alleles

99.2% missing data

4	(0.635) 0.631 0.640

1	(0.365) 0.369 0.360

Locus 1052 :

2 alleles

99.2% missing data

3	(0.638) 0.633 0.637

1	(0.362) 0.367 0.363

Locus 1053 :

2 alleles

98.8% missing data

4	(0.501) 0.494 0.510

1	(0.499) 0.506 0.490

Locus 1054 :

2 alleles

99.2% missing data

4	(0.616) 0.622 0.613

2	(0.384) 0.378 0.387

Locus 1055 :

2 alleles

99.2% missing data

1	(0.538) 0.536 0.539

2	(0.462) 0.464 0.461

Locus 1056 :

2 alleles

99.2% missing data

3	(0.511) 0.516 0.516

1	(0.489) 0.484 0.484

Locus 1057 :

2 alleles

99.2% missing data

3	(0.348) 0.351 0.342

2	(0.652) 0.649 0.658

Locus 1058 :

2 alleles

99.2% missing data

3	(0.455) 0.455 0.453

4	(0.545) 0.545 0.547

Locus 1059 :

2 alleles

99.2% missing data

1	(0.337) 0.342 0.333

4	(0.663) 0.658 0.667

Locus 1060 :

2 alleles

98.8% missing data

1	(0.663) 0.652 0.670

2	(0.337) 0.348 0.330

Locus 1061 :

2 alleles

91.0% missing data

2	(0.923) 0.936 0.911

4	(0.077) 0.064 0.089

Locus 1062 :

2 alleles

97.3% missing data

4	(0.182) 0.178 0.175

3	(0.818) 0.822 0.825

Locus 1063 :

2 alleles

99.2% missing data

3	(0.523) 0.529 0.519

2	(0.477) 0.471 0.481

Locus 1064 :

2 alleles

98.0% missing data

4	(0.753) 0.763 0.749

2	(0.247) 0.237 0.251

Locus 1065 :

2 alleles

99.2% missing data

2	(0.503) 0.510 0.494

1	(0.497) 0.490 0.506

Locus 1066 :

2 alleles

99.2% missing data

1	(0.531) 0.528 0.533

3	(0.469) 0.472 0.467

Locus 1067 :

3 alleles

93.0% missing data

2	(0.782) 0.776 0.808

1	(0.126) 0.126 0.117

4	(0.092) 0.098 0.075

Locus 1068 :

2 alleles

96.5% missing data

4	(0.732) 0.735 0.725

1	(0.268) 0.265 0.275

Locus 1069 :

2 alleles

0.0% missing data

4	(0.983) 0.978 0.995

1	(0.017) 0.022 0.005

Locus 1070 :

2 alleles

99.2% missing data

4	(0.613) 0.609 0.613

1	(0.387) 0.391 0.387

Locus 1071 :

2 alleles

83.2% missing data

3	(0.463) 0.527 0.400

2	(0.537) 0.473 0.600

Locus 1072 :

2 alleles

97.7% missing data

4	(0.379) 0.360 0.389

3	(0.621) 0.640 0.611

Locus 1073 :

3 alleles

98.4% missing data

1	(0.232) 0.233 0.226

2	(0.366) 0.363 0.365

3	(0.403) 0.404 0.409

Locus 1074 :

2 alleles

96.5% missing data

4	(0.197) 0.188 0.204

1	(0.803) 0.812 0.796

Locus 1075 :

2 alleles

97.7% missing data

2	(0.547) 0.541 0.558

3	(0.453) 0.459 0.442

Locus 1076 :

2 alleles

97.3% missing data

1	(0.816) 0.813 0.823

2	(0.184) 0.187 0.177

Locus 1077 :

2 alleles

99.2% missing data

2	(0.659) 0.661 0.656

4	(0.341) 0.339 0.344

Locus 1078 :

3 alleles

98.8% missing data

3	(0.203) 0.202 0.206

1	(0.205) 0.204 0.207

4	(0.592) 0.594 0.588

Locus 1079 :

3 alleles

98.8% missing data

3	(0.306) 0.304 0.312

2	(0.391) 0.385 0.396

4	(0.303) 0.311 0.292

Locus 1080 :

2 alleles

99.2% missing data

1	(0.499) 0.500 0.499

3	(0.501) 0.500 0.501

Locus 1081 :

2 alleles

99.2% missing data

3	(0.538) 0.538 0.538

1	(0.462) 0.462 0.462

Locus 1082 :

2 alleles

98.8% missing data

1	(0.329) 0.318 0.338

4	(0.671) 0.682 0.662

Locus 1083 :

2 alleles

98.0% missing data

1	(0.771) 0.776 0.770

2	(0.229) 0.224 0.230

Locus 1084 :

2 alleles

98.4% missing data

2	(0.698) 0.698 0.707

3	(0.302) 0.302 0.293

Locus 1085 :

2 alleles

98.8% missing data

2	(0.254) 0.247 0.257

4	(0.746) 0.753 0.743

Locus 1086 :

2 alleles

99.2% missing data

4	(0.627) 0.623 0.624

1	(0.373) 0.377 0.376

Locus 1087 :

2 alleles

99.2% missing data

2	(0.754) 0.751 0.749

4	(0.246) 0.249 0.251

Locus 1088 :

2 alleles

96.9% missing data

1	(0.774) 0.775 0.783

3	(0.226) 0.225 0.217

Locus 1089 :

2 alleles

99.2% missing data

4	(0.362) 0.362 0.362

1	(0.638) 0.638 0.638

Locus 1090 :

2 alleles

96.1% missing data

2	(0.779) 0.794 0.774

3	(0.221) 0.206 0.226

Locus 1091 :

2 alleles

90.6% missing data

4	(0.930) 0.938 0.930

1	(0.070) 0.062 0.070

Locus 1092 :

2 alleles

97.3% missing data

4	(0.774) 0.778 0.785

3	(0.226) 0.222 0.215

Locus 1093 :

2 alleles

99.2% missing data

1	(0.369) 0.366 0.363

2	(0.631) 0.634 0.637

Locus 1094 :

2 alleles

99.2% missing data

3	(0.367) 0.364 0.365

1	(0.633) 0.636 0.635

Locus 1095 :

2 alleles

98.0% missing data

1	(0.448) 0.462 0.442

4	(0.552) 0.538 0.558

Locus 1096 :

2 alleles

98.4% missing data

1	(0.747) 0.740 0.750

4	(0.253) 0.260 0.250

Locus 1097 :

2 alleles

99.2% missing data

3	(0.290) 0.287 0.290

1	(0.710) 0.713 0.710

Locus 1098 :

2 alleles

96.5% missing data

2	(0.775) 0.765 0.790

1	(0.225) 0.235 0.210

Locus 1099 :

2 alleles

98.4% missing data

3	(0.186) 0.185 0.183

1	(0.814) 0.815 0.817

Locus 1100 :

2 alleles

99.2% missing data

4	(0.644) 0.641 0.647

3	(0.356) 0.359 0.353

Locus 1101 :

2 alleles

99.2% missing data

1	(0.560) 0.555 0.561

4	(0.440) 0.445 0.439

Locus 1102 :

3 alleles

98.8% missing data

3	(0.131) 0.132 0.135

1	(0.474) 0.474 0.470

2	(0.395) 0.393 0.395

Locus 1103 :

3 alleles

98.4% missing data

4	(0.325) 0.327 0.327

1	(0.181) 0.177 0.186

3	(0.495) 0.496 0.487

Locus 1104 :

2 alleles

96.1% missing data

2	(0.793) 0.810 0.789

3	(0.207) 0.190 0.211

Locus 1105 :

2 alleles

92.2% missing data

1	(0.894) 0.894 0.902

4	(0.106) 0.106 0.098

Locus 1106 :

2 alleles

99.2% missing data

3	(0.539) 0.540 0.541

1	(0.461) 0.460 0.459

Locus 1107 :

3 alleles

98.4% missing data

4	(0.252) 0.257 0.261

3	(0.296) 0.293 0.293

1	(0.452) 0.450 0.446

Locus 1108 :

3 alleles

98.8% missing data

3	(0.475) 0.482 0.472

1	(0.125) 0.122 0.128

4	(0.401) 0.396 0.400

Locus 1109 :

2 alleles

99.2% missing data

1	(0.652) 0.655 0.649

3	(0.348) 0.345 0.351

Locus 1110 :

2 alleles

99.2% missing data

4	(0.443) 0.448 0.442

2	(0.557) 0.552 0.558

Locus 1111 :

2 alleles

99.2% missing data

4	(0.564) 0.564 0.565

2	(0.436) 0.436 0.435

Locus 1112 :

2 alleles

99.2% missing data

2	(0.498) 0.496 0.506

4	(0.502) 0.504 0.494

Locus 1113 :

2 alleles

98.8% missing data

2	(0.355) 0.352 0.361

4	(0.645) 0.648 0.639

Locus 1114 :

3 alleles

98.8% missing data

3	(0.360) 0.359 0.358

1	(0.213) 0.208 0.220

2	(0.427) 0.433 0.421

Locus 1115 :

2 alleles

99.2% missing data

2	(0.574) 0.576 0.576

4	(0.426) 0.424 0.424

Locus 1116 :

2 alleles

99.2% missing data

3	(0.360) 0.358 0.362

4	(0.640) 0.642 0.638

Locus 1117 :

2 alleles

99.2% missing data

2	(0.605) 0.606 0.602

1	(0.395) 0.394 0.398

Locus 1118 :

2 alleles

98.8% missing data

1	(0.579) 0.581 0.572

2	(0.421) 0.419 0.428

Locus 1119 :

3 alleles

98.8% missing data

1	(0.289) 0.284 0.291

3	(0.497) 0.502 0.496

2	(0.214) 0.214 0.214

Locus 1120 :

2 alleles

99.2% missing data

1	(0.552) 0.547 0.560

4	(0.448) 0.453 0.440

Locus 1121 :

3 alleles

98.4% missing data

4	(0.320) 0.317 0.319

3	(0.430) 0.430 0.432

1	(0.250) 0.253 0.248

Locus 1122 :

2 alleles

99.2% missing data

1	(0.344) 0.339 0.351

4	(0.656) 0.661 0.649

Locus 1123 :

2 alleles

99.2% missing data

4	(0.467) 0.469 0.467

3	(0.533) 0.531 0.533

Locus 1124 :

2 alleles

98.8% missing data

4	(0.447) 0.444 0.457

2	(0.553) 0.556 0.543

Locus 1125 :

2 alleles

99.2% missing data

4	(0.532) 0.534 0.524

1	(0.468) 0.466 0.476

Locus 1126 :

2 alleles

98.8% missing data

2	(0.652) 0.645 0.665

1	(0.348) 0.355 0.335

Locus 1127 :

2 alleles

99.2% missing data

2	(0.332) 0.336 0.333

1	(0.668) 0.664 0.667

Locus 1128 :

2 alleles

99.2% missing data

2	(0.563) 0.561 0.569

1	(0.437) 0.439 0.431

Locus 1129 :

2 alleles

94.9% missing data

4	(0.785) 0.776 0.807

1	(0.215) 0.224 0.193

Locus 1130 :

2 alleles

99.2% missing data

3	(0.538) 0.531 0.543

4	(0.462) 0.469 0.457

Locus 1131 :

2 alleles

99.2% missing data

1	(0.762) 0.766 0.759

3	(0.238) 0.234 0.241

Locus 1132 :

2 alleles

99.2% missing data

2	(0.557) 0.555 0.554

3	(0.443) 0.445 0.446

Locus 1133 :

3 alleles

98.8% missing data

1	(0.221) 0.221 0.221

2	(0.290) 0.291 0.291

4	(0.489) 0.488 0.488

Locus 1134 :

2 alleles

99.2% missing data

1	(0.350) 0.347 0.354

3	(0.650) 0.653 0.646

Locus 1135 :

2 alleles

99.2% missing data

3	(0.676) 0.681 0.669

4	(0.324) 0.319 0.331

Locus 1136 :

2 alleles

99.2% missing data

3	(0.681) 0.677 0.680

1	(0.319) 0.323 0.320

Locus 1137 :

2 alleles

99.2% missing data

1	(0.346) 0.344 0.345

4	(0.654) 0.656 0.655

Locus 1138 :

2 alleles

93.0% missing data

4	(0.853) 0.854 0.868

1	(0.147) 0.146 0.132

Locus 1139 :

2 alleles

99.2% missing data

2	(0.388) 0.386 0.394

1	(0.612) 0.614 0.606

Locus 1140 :

2 alleles

99.2% missing data

2	(0.513) 0.511 0.517

1	(0.487) 0.489 0.483

Locus 1141 :

2 alleles

97.7% missing data

1	(0.482) 0.490 0.461

4	(0.518) 0.510 0.539

Locus 1142 :

2 alleles

99.2% missing data

1	(0.286) 0.283 0.286

2	(0.714) 0.717 0.714

Locus 1143 :

2 alleles

96.5% missing data

3	(0.136) 0.128 0.140

1	(0.864) 0.872 0.860

Locus 1144 :

2 alleles

98.8% missing data

1	(0.507) 0.505 0.509

4	(0.493) 0.495 0.491

Locus 1145 :

2 alleles

97.7% missing data

1	(0.852) 0.854 0.850

2	(0.148) 0.146 0.150

Locus 1146 :

2 alleles

99.2% missing data

4	(0.658) 0.664 0.651

2	(0.342) 0.336 0.349

Locus 1147 :

2 alleles

99.2% missing data

1	(0.669) 0.673 0.665

4	(0.331) 0.327 0.335

Locus 1148 :

2 alleles

99.2% missing data

2	(0.560) 0.563 0.560

3	(0.440) 0.437 0.440

Locus 1149 :

2 alleles

89.8% missing data

2	(0.795) 0.831 0.764

4	(0.205) 0.169 0.236

Locus 1150 :

2 alleles

99.2% missing data

4	(0.696) 0.691 0.692

1	(0.304) 0.309 0.308

Locus 1151 :

2 alleles

98.0% missing data

2	(0.850) 0.854 0.845

1	(0.150) 0.146 0.155

Locus 1152 :

2 alleles

98.8% missing data

4	(0.424) 0.420 0.435

1	(0.576) 0.580 0.565

Locus 1153 :

2 alleles

98.8% missing data

1	(0.464) 0.466 0.466

2	(0.536) 0.534 0.534

Locus 1154 :

2 alleles

96.1% missing data

4	(0.812) 0.826 0.804

3	(0.188) 0.174 0.196

Locus 1155 :

2 alleles

99.2% missing data

2	(0.680) 0.681 0.681

1	(0.320) 0.319 0.319

Locus 1156 :

2 alleles

96.1% missing data

3	(0.876) 0.887 0.868

4	(0.124) 0.113 0.132

Locus 1157 :

2 alleles

99.2% missing data

4	(0.472) 0.473 0.478

2	(0.528) 0.527 0.522

Locus 1158 :

2 alleles

99.2% missing data

3	(0.454) 0.458 0.454

4	(0.546) 0.542 0.546

Locus 1159 :

2 alleles

99.2% missing data

4	(0.393) 0.393 0.395

1	(0.607) 0.607 0.605

Locus 1160 :

2 alleles

98.8% missing data

3	(0.688) 0.691 0.690

2	(0.312) 0.309 0.310

Locus 1161 :

2 alleles

98.0% missing data

2	(0.517) 0.526 0.522

1	(0.483) 0.474 0.478

Locus 1162 :

2 alleles

99.2% missing data

4	(0.567) 0.566 0.561

1	(0.433) 0.434 0.439

Locus 1163 :

2 alleles

98.8% missing data

4	(0.734) 0.730 0.741

3	(0.266) 0.270 0.259

Locus 1164 :

2 alleles

99.2% missing data

3	(0.343) 0.348 0.346

2	(0.657) 0.652 0.654

Locus 1165 :

2 alleles

99.2% missing data

4	(0.418) 0.419 0.425

2	(0.582) 0.581 0.575

Locus 1166 :

2 alleles

99.2% missing data

3	(0.236) 0.232 0.238

1	(0.764) 0.768 0.762

Locus 1167 :

2 alleles

0.8% missing data

3	(0.987) 0.989 0.993

2	(0.013) 0.011 0.007

Locus 1168 :

2 alleles

99.2% missing data

1	(0.356) 0.344 0.367

3	(0.644) 0.656 0.633

Locus 1169 :

2 alleles

96.1% missing data

3	(0.909) 0.917 0.903

1	(0.091) 0.083 0.097

Locus 1170 :

2 alleles

99.2% missing data

3	(0.516) 0.511 0.520

1	(0.484) 0.489 0.480

Locus 1171 :

2 alleles

99.2% missing data

2	(0.519) 0.508 0.528

1	(0.481) 0.492 0.472

Locus 1172 :

2 alleles

98.8% missing data

3	(0.588) 0.588 0.591

2	(0.412) 0.412 0.409

Locus 1173 :

2 alleles

99.2% missing data

4	(0.586) 0.584 0.584

2	(0.414) 0.416 0.416

Locus 1174 :

2 alleles

99.2% missing data

4	(0.660) 0.667 0.661

3	(0.340) 0.333 0.339

Locus 1175 :

2 alleles

94.5% missing data

4	(0.886) 0.896 0.880

3	(0.114) 0.104 0.120

Locus 1176 :

2 alleles

99.2% missing data

3	(0.158) 0.161 0.151

4	(0.842) 0.839 0.849

Locus 1177 :

2 alleles

98.8% missing data

4	(0.756) 0.752 0.765

2	(0.244) 0.248 0.235

Locus 1178 :

2 alleles

7.8% missing data

1	(0.985) 0.980 0.995

2	(0.015) 0.020 0.005

Locus 1179 :

2 alleles

98.8% missing data

1	(0.700) 0.704 0.691

3	(0.300) 0.296 0.309

Locus 1180 :

2 alleles

99.2% missing data

2	(0.621) 0.618 0.614

1	(0.379) 0.382 0.386

Locus 1181 :

2 alleles

99.2% missing data

3	(0.564) 0.565 0.569

1	(0.436) 0.435 0.431

Locus 1182 :

2 alleles

99.2% missing data

1	(0.288) 0.288 0.290

2	(0.712) 0.712 0.710

Locus 1183 :

2 alleles

99.2% missing data

4	(0.676) 0.677 0.672

3	(0.324) 0.323 0.328

Locus 1184 :

2 alleles

93.8% missing data

2	(0.873) 0.872 0.884

3	(0.127) 0.128 0.116

Locus 1185 :

2 alleles

99.2% missing data

4	(0.693) 0.698 0.690

3	(0.307) 0.302 0.310

Locus 1186 :

2 alleles

94.5% missing data

1	(0.491) 0.526 0.466

3	(0.509) 0.474 0.534

Locus 1187 :

2 alleles

99.2% missing data

2	(0.431) 0.428 0.435

4	(0.569) 0.572 0.565

Locus 1188 :

2 alleles

99.2% missing data

2	(0.409) 0.413 0.411

4	(0.591) 0.587 0.589

Locus 1189 :

2 alleles

91.4% missing data

4	(0.835) 0.841 0.835

1	(0.165) 0.159 0.165

Locus 1190 :

2 alleles

97.7% missing data

2	(0.224) 0.216 0.227

4	(0.776) 0.784 0.773

Locus 1191 :

2 alleles

99.2% missing data

3	(0.508) 0.510 0.508

4	(0.492) 0.490 0.492

Locus 1192 :

2 alleles

96.1% missing data

4	(0.773) 0.775 0.781

2	(0.227) 0.225 0.219

Locus 1193 :

2 alleles

99.2% missing data

4	(0.612) 0.615 0.612

2	(0.388) 0.385 0.388

Locus 1194 :

2 alleles

99.2% missing data

1	(0.391) 0.393 0.387

3	(0.609) 0.607 0.613

Locus 1195 :

2 alleles

0.0% missing data

2	(0.988) 0.984 0.996

3	(0.012) 0.016 0.004

Locus 1196 :

2 alleles

98.8% missing data

3	(0.359) 0.365 0.356

4	(0.641) 0.635 0.644

Locus 1197 :

3 alleles

92.6% missing data

2	(0.760) 0.744 0.791

4	(0.145) 0.152 0.129

1	(0.095) 0.104 0.080

Locus 1198 :

2 alleles

94.9% missing data

1	(0.831) 0.832 0.840

4	(0.169) 0.168 0.160

Locus 1199 :

2 alleles

98.8% missing data

4	(0.582) 0.579 0.587

1	(0.418) 0.421 0.413

Locus 1200 :

2 alleles

99.2% missing data

1	(0.496) 0.497 0.496

3	(0.504) 0.503 0.504

Locus 1201 :

4 alleles

91.8% missing data

4	(0.187) 0.211 0.158

3	(0.683) 0.659 0.718

2	(0.050) 0.051 0.045

1	(0.081) 0.079 0.079

Locus 1202 :

2 alleles

98.8% missing data

3	(0.579) 0.565 0.582

4	(0.421) 0.435 0.418

Locus 1203 :

2 alleles

99.2% missing data

3	(0.571) 0.570 0.564

1	(0.429) 0.430 0.436

Locus 1204 :

2 alleles

99.2% missing data

1	(0.401) 0.396 0.410

3	(0.599) 0.604 0.590

Locus 1205 :

2 alleles

99.2% missing data

3	(0.644) 0.642 0.641

2	(0.356) 0.358 0.359

Locus 1206 :

2 alleles

99.2% missing data

3	(0.474) 0.478 0.479

4	(0.526) 0.522 0.521

Locus 1207 :

3 alleles

98.8% missing data

1	(0.277) 0.277 0.280

3	(0.348) 0.351 0.347

2	(0.375) 0.372 0.373

Locus 1208 :

2 alleles

99.2% missing data

2	(0.629) 0.625 0.627

4	(0.371) 0.375 0.373

Locus 1209 :

2 alleles

98.0% missing data

2	(0.601) 0.606 0.603

4	(0.399) 0.394 0.397

Locus 1210 :

2 alleles

98.8% missing data

3	(0.525) 0.514 0.529

2	(0.475) 0.486 0.471

Locus 1211 :

2 alleles

98.8% missing data

1	(0.353) 0.351 0.367

2	(0.647) 0.649 0.633

Locus 1212 :

2 alleles

99.2% missing data

1	(0.488) 0.490 0.490

3	(0.512) 0.510 0.510

Locus 1213 :

2 alleles

92.6% missing data

3	(0.867) 0.864 0.881

2	(0.133) 0.136 0.119

Locus 1214 :

2 alleles

98.8% missing data

4	(0.161) 0.153 0.164

1	(0.839) 0.847 0.836

Locus 1215 :

4 alleles

93.4% missing data

3	(0.615) 0.610 0.640

4	(0.118) 0.114 0.110

2	(0.132) 0.143 0.123

1	(0.134) 0.133 0.128

Locus 1216 :

3 alleles

98.8% missing data

3	(0.588) 0.584 0.590

1	(0.196) 0.197 0.197

4	(0.216) 0.219 0.212

Locus 1217 :

2 alleles

99.2% missing data

1	(0.318) 0.319 0.322

2	(0.682) 0.681 0.678

Locus 1218 :

2 alleles

99.2% missing data

1	(0.639) 0.644 0.635

4	(0.361) 0.356 0.365

Locus 1219 :

2 alleles

99.2% missing data

2	(0.375) 0.376 0.384

1	(0.625) 0.624 0.616

Locus 1220 :

3 alleles

94.9% missing data

2	(0.149) 0.148 0.146

3	(0.465) 0.464 0.474

4	(0.387) 0.388 0.380

Locus 1221 :

2 alleles

98.8% missing data

2	(0.721) 0.721 0.716

4	(0.279) 0.279 0.284

Locus 1222 :

2 alleles

98.8% missing data

4	(0.671) 0.663 0.672

1	(0.329) 0.337 0.328

Locus 1223 :

2 alleles

98.8% missing data

2	(0.696) 0.691 0.701

3	(0.304) 0.309 0.299

Locus 1224 :

2 alleles

99.2% missing data

4	(0.556) 0.556 0.551

3	(0.444) 0.444 0.449

Locus 1225 :

2 alleles

99.2% missing data

1	(0.645) 0.649 0.642

2	(0.355) 0.351 0.358

Locus 1226 :

2 alleles

99.2% missing data

4	(0.718) 0.722 0.715

1	(0.282) 0.278 0.285

Locus 1227 :

3 alleles

98.0% missing data

4	(0.519) 0.518 0.522

3	(0.207) 0.213 0.199

1	(0.273) 0.269 0.279

Locus 1228 :

2 alleles

97.7% missing data

4	(0.562) 0.549 0.577

2	(0.438) 0.451 0.423

Locus 1229 :

2 alleles

99.2% missing data

1	(0.463) 0.466 0.468

3	(0.537) 0.534 0.532

Locus 1230 :

2 alleles

91.0% missing data

1	(0.918) 0.933 0.908

2	(0.082) 0.067 0.092

Locus 1231 :

2 alleles

99.2% missing data

3	(0.689) 0.689 0.690

4	(0.311) 0.311 0.310

Locus 1232 :

2 alleles

99.2% missing data

4	(0.610) 0.613 0.606

1	(0.390) 0.387 0.394

Locus 1233 :

2 alleles

14.1% missing data

2	(0.986) 0.988 0.992

4	(0.014) 0.012 0.008

Locus 1234 :

2 alleles

99.2% missing data

3	(0.246) 0.247 0.248

4	(0.754) 0.753 0.752

Locus 1235 :

3 alleles

98.8% missing data

3	(0.266) 0.264 0.270

4	(0.355) 0.363 0.349

2	(0.380) 0.373 0.381

Locus 1236 :

2 alleles

98.8% missing data

4	(0.695) 0.685 0.704

2	(0.305) 0.315 0.296

Locus 1237 :

2 alleles

99.2% missing data

2	(0.639) 0.635 0.641

1	(0.361) 0.365 0.359

Locus 1238 :

2 alleles

92.6% missing data

2	(0.904) 0.897 0.921

1	(0.096) 0.103 0.079

Locus 1239 :

2 alleles

96.5% missing data

4	(0.783) 0.788 0.786

2	(0.217) 0.212 0.214

Locus 1240 :

2 alleles

99.2% missing data

4	(0.639) 0.641 0.633

2	(0.361) 0.359 0.367

Locus 1241 :

2 alleles

99.2% missing data

3	(0.505) 0.504 0.502

2	(0.495) 0.496 0.498

Locus 1242 :

2 alleles

99.2% missing data

4	(0.389) 0.391 0.390

1	(0.611) 0.609 0.610

Locus 1243 :

2 alleles

98.4% missing data

3	(0.267) 0.262 0.269

4	(0.733) 0.738 0.731

Locus 1244 :

2 alleles

99.2% missing data

2	(0.449) 0.452 0.455

1	(0.551) 0.548 0.545

Locus 1245 :

2 alleles

98.8% missing data

4	(0.495) 0.492 0.495

2	(0.505) 0.508 0.505

Locus 1246 :

2 alleles

99.2% missing data

4	(0.708) 0.708 0.699

1	(0.292) 0.292 0.301

Locus 1247 :

2 alleles

99.2% missing data

2	(0.441) 0.437 0.434

3	(0.559) 0.563 0.566

Locus 1248 :

2 alleles

93.0% missing data

2	(0.881) 0.882 0.891

4	(0.119) 0.118 0.109

Locus 1249 :

2 alleles

99.2% missing data

3	(0.577) 0.577 0.584

1	(0.423) 0.423 0.416

Locus 1250 :

2 alleles

93.0% missing data

4	(0.918) 0.915 0.923

1	(0.082) 0.085 0.077

Locus 1251 :

2 alleles

96.9% missing data

3	(0.862) 0.860 0.866

4	(0.138) 0.140 0.134

Locus 1252 :

2 alleles

98.8% missing data

2	(0.357) 0.361 0.359

4	(0.643) 0.639 0.641

Locus 1253 :

2 alleles

99.2% missing data

2	(0.531) 0.531 0.535

1	(0.469) 0.469 0.465

Locus 1254 :

2 alleles

99.2% missing data

4	(0.455) 0.458 0.458

2	(0.545) 0.542 0.542

Locus 1255 :

2 alleles

99.2% missing data

3	(0.876) 0.879 0.870

4	(0.124) 0.121 0.130

Locus 1256 :

2 alleles

96.1% missing data

4	(0.255) 0.251 0.250

1	(0.745) 0.749 0.750

Locus 1257 :

2 alleles

98.8% missing data

3	(0.646) 0.639 0.650

1	(0.354) 0.361 0.350

Locus 1258 :

2 alleles

98.0% missing data

4	(0.533) 0.537 0.542

2	(0.467) 0.463 0.458

Locus 1259 :

2 alleles

99.2% missing data

4	(0.319) 0.314 0.321

2	(0.681) 0.686 0.679

Locus 1260 :

3 alleles

98.8% missing data

4	(0.204) 0.206 0.205

2	(0.533) 0.530 0.527

1	(0.263) 0.264 0.268

Locus 1261 :

2 alleles

98.0% missing data

4	(0.227) 0.228 0.222

3	(0.773) 0.772 0.778

Locus 1262 :

2 alleles

99.2% missing data

3	(0.668) 0.669 0.666

2	(0.332) 0.331 0.334

Locus 1263 :

2 alleles

98.8% missing data

3	(0.761) 0.763 0.761

4	(0.239) 0.237 0.239

Locus 1264 :

2 alleles

99.2% missing data

4	(0.688) 0.682 0.697

3	(0.312) 0.318 0.303

Locus 1265 :

2 alleles

99.2% missing data

1	(0.654) 0.657 0.655

4	(0.346) 0.343 0.345

Locus 1266 :

2 alleles

99.2% missing data

2	(0.470) 0.470 0.457

3	(0.530) 0.530 0.543

Locus 1267 :

2 alleles

97.7% missing data

1	(0.191) 0.193 0.187

4	(0.809) 0.807 0.813

Locus 1268 :

2 alleles

99.2% missing data

3	(0.669) 0.676 0.666

1	(0.331) 0.324 0.334

Locus 1269 :

2 alleles

98.4% missing data

4	(0.648) 0.653 0.647

3	(0.352) 0.347 0.353

Locus 1270 :

2 alleles

99.2% missing data

2	(0.408) 0.405 0.409

3	(0.592) 0.595 0.591

Locus 1271 :

2 alleles

99.2% missing data

4	(0.313) 0.309 0.318

3	(0.687) 0.691 0.682

Locus 1272 :

3 alleles

93.0% missing data

3	(0.314) 0.315 0.305

2	(0.640) 0.641 0.648

4	(0.046) 0.044 0.047

Locus 1273 :

2 alleles

96.5% missing data

1	(0.844) 0.844 0.846

4	(0.156) 0.156 0.154

Locus 1274 :

2 alleles

99.2% missing data

2	(0.735) 0.727 0.738

1	(0.265) 0.273 0.262

Locus 1275 :

2 alleles

99.2% missing data

4	(0.314) 0.313 0.313

1	(0.686) 0.687 0.687

Locus 1276 :

2 alleles

99.2% missing data

2	(0.568) 0.582 0.560

1	(0.432) 0.418 0.440

Locus 1277 :

3 alleles

96.5% missing data

1	(0.565) 0.559 0.579

2	(0.299) 0.311 0.288

4	(0.136) 0.130 0.133

Locus 1278 :

2 alleles

98.0% missing data

4	(0.534) 0.543 0.528

3	(0.466) 0.457 0.472

Locus 1279 :

2 alleles

97.7% missing data

4	(0.256) 0.247 0.260

1	(0.744) 0.753 0.740

Locus 1280 :

2 alleles

99.2% missing data

3	(0.740) 0.740 0.734

4	(0.260) 0.260 0.266

Locus 1281 :

2 alleles

98.0% missing data

3	(0.799) 0.803 0.795

1	(0.201) 0.197 0.205

Locus 1282 :

2 alleles

99.2% missing data

1	(0.386) 0.386 0.391

3	(0.614) 0.614 0.609

Locus 1283 :

2 alleles

99.2% missing data

2	(0.367) 0.370 0.365

1	(0.633) 0.630 0.635

Locus 1284 :

2 alleles

99.2% missing data

2	(0.629) 0.624 0.636

4	(0.371) 0.376 0.364

Locus 1285 :

2 alleles

99.2% missing data

1	(0.506) 0.503 0.506

3	(0.494) 0.497 0.494

Locus 1286 :

2 alleles

99.2% missing data

2	(0.359) 0.357 0.367

4	(0.641) 0.643 0.633

Locus 1287 :

2 alleles

99.2% missing data

1	(0.624) 0.617 0.629

4	(0.376) 0.383 0.371

Locus 1288 :

2 alleles

99.2% missing data

3	(0.592) 0.594 0.592

2	(0.408) 0.406 0.408

Locus 1289 :

2 alleles

99.2% missing data

3	(0.230) 0.236 0.226

2	(0.770) 0.764 0.774

Locus 1290 :

2 alleles

0.4% missing data

4	(0.969) 0.978 0.972

3	(0.031) 0.022 0.028

Locus 1291 :

2 alleles

96.1% missing data

4	(0.158) 0.142 0.166

2	(0.842) 0.858 0.834

Locus 1292 :

2 alleles

99.2% missing data

4	(0.309) 0.309 0.313

3	(0.691) 0.691 0.687

Locus 1293 :

2 alleles

99.2% missing data

2	(0.198) 0.197 0.203

4	(0.802) 0.803 0.797

Locus 1294 :

2 alleles

97.3% missing data

4	(0.419) 0.409 0.420

3	(0.581) 0.591 0.580

Locus 1295 :

2 alleles

99.2% missing data

1	(0.277) 0.278 0.282

4	(0.723) 0.722 0.718

Locus 1296 :

2 alleles

99.2% missing data

4	(0.400) 0.394 0.403

2	(0.600) 0.606 0.597

Locus 1297 :

2 alleles

98.0% missing data

1	(0.258) 0.250 0.265

4	(0.742) 0.750 0.735

Locus 1298 :

2 alleles

99.2% missing data

2	(0.601) 0.604 0.596

4	(0.399) 0.396 0.404

Locus 1299 :

3 alleles

92.2% missing data

2	(0.146) 0.154 0.126

4	(0.702) 0.693 0.736

3	(0.152) 0.153 0.139

Locus 1300 :

2 alleles

99.2% missing data

4	(0.696) 0.707 0.690

3	(0.304) 0.293 0.310

Locus 1301 :

2 alleles

99.2% missing data

4	(0.408) 0.407 0.413

2	(0.592) 0.593 0.587

Locus 1302 :

2 alleles

99.2% missing data

3	(0.241) 0.239 0.240

2	(0.759) 0.761 0.760

Locus 1303 :

2 alleles

99.2% missing data

2	(0.573) 0.571 0.574

4	(0.427) 0.429 0.426

Locus 1304 :

2 alleles

99.2% missing data

4	(0.539) 0.538 0.538

2	(0.461) 0.462 0.462

Locus 1305 :

2 alleles

99.2% missing data

1	(0.384) 0.385 0.384

4	(0.616) 0.615 0.616

Locus 1306 :

2 alleles

99.2% missing data

1	(0.449) 0.455 0.443

3	(0.551) 0.545 0.557

Locus 1307 :

2 alleles

91.0% missing data

1	(0.917) 0.932 0.909

2	(0.083) 0.068 0.091

Locus 1308 :

2 alleles

95.7% missing data

2	(0.212) 0.200 0.221

3	(0.788) 0.800 0.779

Locus 1309 :

2 alleles

99.2% missing data

3	(0.386) 0.381 0.388

1	(0.614) 0.619 0.612

Locus 1310 :

2 alleles

93.0% missing data

3	(0.906) 0.905 0.913

4	(0.094) 0.095 0.087

Locus 1311 :

2 alleles

99.2% missing data

2	(0.433) 0.429 0.436

3	(0.567) 0.571 0.564

Locus 1312 :

2 alleles

98.8% missing data

4	(0.660) 0.656 0.657

1	(0.340) 0.344 0.343

Locus 1313 :

2 alleles

99.2% missing data

3	(0.488) 0.484 0.489

4	(0.512) 0.516 0.511

Locus 1314 :

2 alleles

99.2% missing data

4	(0.636) 0.633 0.633

1	(0.364) 0.367 0.367

Locus 1315 :

3 alleles

98.8% missing data

3	(0.247) 0.240 0.250

4	(0.430) 0.432 0.429

1	(0.322) 0.329 0.321

Locus 1316 :

2 alleles

99.2% missing data

4	(0.716) 0.717 0.711

1	(0.284) 0.283 0.289

Locus 1317 :

2 alleles

99.2% missing data

3	(0.655) 0.651 0.657

4	(0.345) 0.349 0.343

Locus 1318 :

2 alleles

99.2% missing data

2	(0.433) 0.429 0.437

1	(0.567) 0.571 0.563

Locus 1319 :

3 alleles

98.8% missing data

2	(0.290) 0.293 0.290

4	(0.468) 0.457 0.478

1	(0.242) 0.251 0.233

Locus 1320 :

2 alleles

99.2% missing data

1	(0.365) 0.365 0.367

3	(0.635) 0.635 0.633

Locus 1321 :

2 alleles

99.2% missing data

1	(0.804) 0.806 0.797

3	(0.196) 0.194 0.203

Locus 1322 :

2 alleles

98.8% missing data

3	(0.611) 0.605 0.613

4	(0.389) 0.395 0.387

Locus 1323 :

2 alleles

99.2% missing data

3	(0.397) 0.398 0.389

2	(0.603) 0.602 0.611

Locus 1324 :

2 alleles

99.2% missing data

3	(0.479) 0.473 0.486

2	(0.521) 0.527 0.514

Locus 1325 :

2 alleles

98.4% missing data

3	(0.379) 0.373 0.372

4	(0.621) 0.627 0.628

Locus 1326 :

2 alleles

99.2% missing data

3	(0.406) 0.404 0.409

1	(0.594) 0.596 0.591

Locus 1327 :

2 alleles

99.2% missing data

3	(0.496) 0.495 0.497

2	(0.504) 0.505 0.503

Locus 1328 :

2 alleles

99.2% missing data

3	(0.349) 0.348 0.351

1	(0.651) 0.652 0.649

Locus 1329 :

2 alleles

98.8% missing data

4	(0.358) 0.357 0.362

1	(0.642) 0.643 0.638

Locus 1330 :

2 alleles

98.8% missing data

2	(0.416) 0.420 0.412

3	(0.584) 0.580 0.588

Locus 1331 :

2 alleles

99.2% missing data

3	(0.449) 0.453 0.449

4	(0.551) 0.547 0.551

Locus 1332 :

2 alleles

92.6% missing data

2	(0.874) 0.859 0.886

1	(0.126) 0.141 0.114

Locus 1333 :

2 alleles

99.2% missing data

2	(0.456) 0.450 0.463

3	(0.544) 0.550 0.537

Locus 1334 :

2 alleles

97.3% missing data

3	(0.715) 0.725 0.706

4	(0.285) 0.275 0.294

Locus 1335 :

2 alleles

99.2% missing data

1	(0.548) 0.550 0.545

4	(0.452) 0.450 0.455

Locus 1336 :

2 alleles

99.2% missing data

1	(0.325) 0.329 0.328

3	(0.675) 0.671 0.672

Locus 1337 :

2 alleles

94.9% missing data

3	(0.882) 0.879 0.884

1	(0.118) 0.121 0.116

Locus 1338 :

2 alleles

95.3% missing data

4	(0.130) 0.127 0.120

3	(0.870) 0.873 0.880

Locus 1339 :

3 alleles

98.8% missing data

1	(0.397) 0.393 0.400

4	(0.355) 0.359 0.350

3	(0.247) 0.249 0.250

Locus 1340 :

2 alleles

99.2% missing data

4	(0.593) 0.584 0.601

1	(0.407) 0.416 0.399

Locus 1341 :

2 alleles

99.2% missing data

2	(0.434) 0.427 0.442

3	(0.566) 0.573 0.558

Locus 1342 :

2 alleles

98.8% missing data

3	(0.579) 0.568 0.583

4	(0.421) 0.432 0.417

Locus 1343 :

2 alleles

99.2% missing data

3	(0.555) 0.552 0.556

1	(0.445) 0.448 0.444

Locus 1344 :

2 alleles

98.8% missing data

4	(0.529) 0.533 0.527

2	(0.471) 0.467 0.473

Locus 1345 :

2 alleles

99.2% missing data

3	(0.684) 0.691 0.681

1	(0.316) 0.309 0.319

Locus 1346 :

4 alleles

94.9% missing data

3	(0.321) 0.325 0.306

1	(0.335) 0.338 0.345

4	(0.267) 0.262 0.270

2	(0.077) 0.075 0.078

Locus 1347 :

2 alleles

98.8% missing data

4	(0.783) 0.778 0.787

1	(0.217) 0.222 0.213

Locus 1348 :

2 alleles

97.3% missing data

2	(0.489) 0.472 0.495

1	(0.511) 0.528 0.505

Locus 1349 :

2 alleles

92.2% missing data

1	(0.285) 0.301 0.261

4	(0.715) 0.699 0.739

Locus 1350 :

3 alleles

97.3% missing data

2	(0.382) 0.371 0.387

1	(0.423) 0.440 0.418

4	(0.195) 0.189 0.195

Locus 1351 :

2 alleles

99.2% missing data

1	(0.784) 0.782 0.783

4	(0.216) 0.218 0.217

Locus 1352 :

2 alleles

99.2% missing data

2	(0.743) 0.734 0.745

1	(0.257) 0.266 0.255

Locus 1353 :

2 alleles

96.1% missing data

2	(0.509) 0.483 0.528

1	(0.491) 0.517 0.472

Locus 1354 :

2 alleles

99.2% missing data

1	(0.693) 0.695 0.696

4	(0.307) 0.305 0.304

Locus 1355 :

2 alleles

0.4% missing data

4	(0.982) 0.987 0.988

1	(0.018) 0.013 0.012

Locus 1356 :

2 alleles

98.8% missing data

2	(0.667) 0.662 0.668

1	(0.333) 0.338 0.332

Locus 1357 :

2 alleles

99.2% missing data

2	(0.429) 0.423 0.432

1	(0.571) 0.577 0.568

Locus 1358 :

2 alleles

99.2% missing data

1	(0.709) 0.701 0.715

3	(0.291) 0.299 0.285

Locus 1359 :

2 alleles

99.2% missing data

3	(0.544) 0.540 0.547

1	(0.456) 0.460 0.453

Locus 1360 :

2 alleles

98.8% missing data

2	(0.608) 0.605 0.616

3	(0.392) 0.395 0.384

Locus 1361 :

2 alleles

97.7% missing data

2	(0.431) 0.445 0.428

3	(0.569) 0.555 0.572

Locus 1362 :

2 alleles

99.2% missing data

2	(0.227) 0.222 0.234

1	(0.773) 0.778 0.766

Locus 1363 :

2 alleles

98.0% missing data

3	(0.251) 0.252 0.244

1	(0.749) 0.748 0.756

Locus 1364 :

2 alleles

99.2% missing data

2	(0.571) 0.573 0.574

1	(0.429) 0.427 0.426

Locus 1365 :

2 alleles

99.2% missing data

3	(0.497) 0.497 0.496

1	(0.503) 0.503 0.504

Locus 1366 :

2 alleles

99.2% missing data

3	(0.529) 0.529 0.525

1	(0.471) 0.471 0.475

Locus 1367 :

2 alleles

98.4% missing data

3	(0.592) 0.589 0.599

4	(0.408) 0.411 0.401

Locus 1368 :

2 alleles

99.2% missing data

1	(0.339) 0.337 0.344

4	(0.661) 0.663 0.656

Locus 1369 :

2 alleles

98.8% missing data

4	(0.473) 0.473 0.467

1	(0.527) 0.527 0.533

Locus 1370 :

2 alleles

96.9% missing data

3	(0.776) 0.769 0.782

1	(0.224) 0.231 0.218

Locus 1371 :

2 alleles

99.2% missing data

3	(0.594) 0.596 0.586

4	(0.406) 0.404 0.414

Locus 1372 :

2 alleles

98.8% missing data

3	(0.462) 0.458 0.465

1	(0.538) 0.542 0.535

Locus 1373 :

2 alleles

98.8% missing data

1	(0.468) 0.456 0.476

3	(0.532) 0.544 0.524

Locus 1374 :

2 alleles

98.4% missing data

2	(0.747) 0.742 0.756

1	(0.253) 0.258 0.244

Locus 1375 :

2 alleles

99.2% missing data

4	(0.553) 0.546 0.556

1	(0.447) 0.454 0.444

Locus 1376 :

2 alleles

99.2% missing data

4	(0.644) 0.642 0.651

1	(0.356) 0.358 0.349

Locus 1377 :

2 alleles

98.0% missing data

1	(0.858) 0.852 0.865

4	(0.142) 0.148 0.135

Locus 1378 :

2 alleles

98.4% missing data

2	(0.577) 0.584 0.573

1	(0.423) 0.416 0.427

Locus 1379 :

2 alleles

99.2% missing data

3	(0.554) 0.551 0.555

1	(0.446) 0.449 0.445

Locus 1380 :

2 alleles

99.2% missing data

3	(0.392) 0.389 0.399

1	(0.608) 0.611 0.601

Locus 1381 :

2 alleles

99.2% missing data

4	(0.400) 0.398 0.400

3	(0.600) 0.602 0.600

Locus 1382 :

3 alleles

98.4% missing data

3	(0.219) 0.217 0.226

1	(0.436) 0.433 0.432

4	(0.345) 0.350 0.343

Locus 1383 :

2 alleles

96.1% missing data

2	(0.867) 0.868 0.870

1	(0.133) 0.132 0.130

Locus 1384 :

2 alleles

96.9% missing data

1	(0.851) 0.860 0.844

4	(0.149) 0.140 0.156

Locus 1385 :

2 alleles

8.6% missing data

2	(0.991) 0.984 0.998

3	(0.009) 0.016 0.002

Locus 1386 :

2 alleles

99.2% missing data

1	(0.640) 0.634 0.646

2	(0.360) 0.366 0.354

Locus 1387 :

2 alleles

99.2% missing data

2	(0.632) 0.636 0.632

1	(0.368) 0.364 0.368

Locus 1388 :

2 alleles

99.2% missing data

2	(0.445) 0.437 0.445

4	(0.555) 0.563 0.555

Locus 1389 :

2 alleles

99.2% missing data

2	(0.765) 0.765 0.772

1	(0.235) 0.235 0.228

Locus 1390 :

2 alleles

8.2% missing data

1	(0.984) 0.977 0.995

2	(0.016) 0.023 0.005

Locus 1391 :

2 alleles

98.4% missing data

1	(0.678) 0.689 0.676

4	(0.322) 0.311 0.324

Locus 1392 :

2 alleles

1.2% missing data

2	(0.983) 0.979 0.994

3	(0.017) 0.021 0.006

Locus 1393 :

2 alleles

99.2% missing data

1	(0.575) 0.579 0.567

3	(0.425) 0.421 0.433

Locus 1394 :

2 alleles

98.4% missing data

3	(0.442) 0.455 0.438

1	(0.558) 0.545 0.562

Locus 1395 :

2 alleles

99.2% missing data

3	(0.600) 0.591 0.609

4	(0.400) 0.409 0.391

Locus 1396 :

2 alleles

95.3% missing data

3	(0.149) 0.148 0.140

1	(0.851) 0.852 0.860

Locus 1397 :

2 alleles

98.4% missing data

4	(0.561) 0.569 0.550

1	(0.439) 0.431 0.450

Locus 1398 :

2 alleles

91.0% missing data

4	(0.918) 0.930 0.913

2	(0.082) 0.070 0.087

Locus 1399 :

2 alleles

99.2% missing data

1	(0.722) 0.727 0.716

4	(0.278) 0.273 0.284

Locus 1400 :

2 alleles

99.2% missing data

3	(0.238) 0.234 0.239

1	(0.762) 0.766 0.761

Locus 1401 :

3 alleles

98.8% missing data

1	(0.239) 0.242 0.247

4	(0.362) 0.358 0.359

2	(0.398) 0.399 0.394

Locus 1402 :

3 alleles

96.1% missing data

1	(0.626) 0.651 0.616

2	(0.193) 0.179 0.200

3	(0.181) 0.169 0.184

Locus 1403 :

2 alleles

99.2% missing data

1	(0.514) 0.509 0.523

2	(0.486) 0.491 0.477

Locus 1404 :

2 alleles

98.4% missing data

4	(0.560) 0.571 0.556

1	(0.440) 0.429 0.444

Locus 1405 :

2 alleles

99.2% missing data

1	(0.441) 0.435 0.449

4	(0.559) 0.565 0.551

Locus 1406 :

2 alleles

0.0% missing data

1	(0.983) 0.978 0.995

3	(0.017) 0.022 0.005

Locus 1407 :

2 alleles

98.8% missing data

4	(0.599) 0.598 0.603

3	(0.401) 0.402 0.397

Locus 1408 :

2 alleles

98.8% missing data

2	(0.418) 0.421 0.410

3	(0.582) 0.579 0.590

Locus 1409 :

4 alleles

96.9% missing data

2	(0.444) 0.451 0.440

4	(0.161) 0.159 0.154

1	(0.238) 0.233 0.252

3	(0.157) 0.156 0.154

Locus 1410 :

3 alleles

98.8% missing data

4	(0.378) 0.379 0.379

1	(0.362) 0.356 0.364

3	(0.260) 0.264 0.256

Locus 1411 :

2 alleles

99.2% missing data

3	(0.406) 0.404 0.410

1	(0.594) 0.596 0.590

Locus 1412 :

2 alleles

99.2% missing data

2	(0.337) 0.340 0.333

4	(0.663) 0.660 0.667

Locus 1413 :

2 alleles

91.0% missing data

3	(0.922) 0.936 0.912

1	(0.078) 0.064 0.088

Locus 1414 :

2 alleles

99.2% missing data

3	(0.418) 0.421 0.420

1	(0.582) 0.579 0.580

Locus 1415 :

2 alleles

99.2% missing data

3	(0.481) 0.485 0.478

4	(0.519) 0.515 0.522

Locus 1416 :

2 alleles

99.2% missing data

3	(0.585) 0.583 0.586

1	(0.415) 0.417 0.414

Locus 1417 :

2 alleles

95.3% missing data

3	(0.089) 0.092 0.084

2	(0.911) 0.908 0.916

Locus 1418 :

2 alleles

99.2% missing data

4	(0.362) 0.367 0.363

1	(0.638) 0.633 0.637

Locus 1419 :

3 alleles

98.8% missing data

1	(0.321) 0.316 0.321

2	(0.448) 0.446 0.450

4	(0.232) 0.238 0.230

Locus 1420 :

2 alleles

99.2% missing data

3	(0.558) 0.563 0.553

4	(0.442) 0.437 0.447

Locus 1421 :

2 alleles

99.2% missing data

2	(0.289) 0.282 0.289

4	(0.711) 0.718 0.711

Locus 1422 :

3 alleles

98.4% missing data

1	(0.329) 0.333 0.327

4	(0.374) 0.365 0.385

3	(0.297) 0.303 0.288

Locus 1423 :

2 alleles

98.4% missing data

2	(0.313) 0.304 0.319

1	(0.687) 0.696 0.681

Locus 1424 :

3 alleles

98.8% missing data

2	(0.386) 0.383 0.388

1	(0.330) 0.325 0.330

4	(0.284) 0.291 0.282

Locus 1425 :

2 alleles

8.2% missing data

4	(0.981) 0.969 0.994

1	(0.019) 0.031 0.006

Locus 1426 :

2 alleles

99.2% missing data

3	(0.244) 0.243 0.248

1	(0.756) 0.757 0.752

Locus 1427 :

2 alleles

98.8% missing data

3	(0.547) 0.553 0.546

4	(0.453) 0.447 0.454

Locus 1428 :

3 alleles

98.4% missing data

1	(0.322) 0.316 0.327

3	(0.235) 0.238 0.233

4	(0.443) 0.446 0.440

Locus 1429 :

2 alleles

98.8% missing data

4	(0.502) 0.493 0.507

1	(0.498) 0.507 0.493

Locus 1430 :

2 alleles

92.6% missing data

3	(0.906) 0.907 0.912

1	(0.094) 0.093 0.088

Locus 1431 :

2 alleles

99.2% missing data

1	(0.463) 0.458 0.473

4	(0.537) 0.542 0.527

Locus 1432 :

2 alleles

97.3% missing data

1	(0.636) 0.636 0.645

4	(0.364) 0.364 0.355

Locus 1433 :

2 alleles

98.8% missing data

3	(0.408) 0.407 0.405

4	(0.592) 0.593 0.595

Locus 1434 :

2 alleles

99.2% missing data

3	(0.566) 0.560 0.575

1	(0.434) 0.440 0.425

Locus 1435 :

2 alleles

99.2% missing data

1	(0.460) 0.457 0.461

4	(0.540) 0.543 0.539

Locus 1436 :

2 alleles

99.2% missing data

3	(0.398) 0.394 0.404

1	(0.602) 0.606 0.596

Locus 1437 :

3 alleles

98.0% missing data

3	(0.127) 0.118 0.133

1	(0.712) 0.715 0.709

4	(0.161) 0.167 0.157

Locus 1438 :

2 alleles

98.8% missing data

2	(0.795) 0.796 0.792

1	(0.205) 0.204 0.208

Locus 1439 :

3 alleles

94.9% missing data

3	(0.083) 0.086 0.078

1	(0.102) 0.100 0.097

2	(0.815) 0.814 0.825

Locus 1440 :

2 alleles

99.2% missing data

2	(0.549) 0.541 0.555

3	(0.451) 0.459 0.445

Locus 1441 :

3 alleles

98.0% missing data

3	(0.414) 0.422 0.415

4	(0.164) 0.168 0.157

2	(0.422) 0.409 0.428

Locus 1442 :

2 alleles

98.0% missing data

4	(0.787) 0.797 0.783

2	(0.213) 0.203 0.217

Locus 1443 :

2 alleles

99.2% missing data

2	(0.741) 0.742 0.744

1	(0.259) 0.258 0.256

Locus 1444 :

3 alleles

98.8% missing data

3	(0.350) 0.347 0.346

2	(0.440) 0.444 0.438

4	(0.210) 0.210 0.215

Locus 1445 :

2 alleles

99.2% missing data

4	(0.448) 0.441 0.448

2	(0.552) 0.559 0.552

Locus 1446 :

2 alleles

96.1% missing data

4	(0.124) 0.120 0.125

3	(0.876) 0.880 0.875

Locus 1447 :

2 alleles

98.8% missing data

1	(0.301) 0.301 0.305

2	(0.699) 0.699 0.695

Locus 1448 :

3 alleles

98.4% missing data

1	(0.157) 0.153 0.159

3	(0.684) 0.679 0.688

4	(0.159) 0.168 0.152

Locus 1449 :

3 alleles

96.5% missing data

2	(0.143) 0.141 0.138

1	(0.400) 0.403 0.400

4	(0.457) 0.456 0.463

Locus 1450 :

2 alleles

99.2% missing data

4	(0.582) 0.580 0.588

1	(0.418) 0.420 0.412

Locus 1451 :

2 alleles

98.8% missing data

4	(0.501) 0.496 0.510

2	(0.499) 0.504 0.490

Locus 1452 :

2 alleles

98.8% missing data

4	(0.550) 0.551 0.556

1	(0.450) 0.449 0.444

Locus 1453 :

2 alleles

97.7% missing data

1	(0.115) 0.113 0.116

3	(0.885) 0.887 0.884

Locus 1454 :

2 alleles

99.2% missing data

1	(0.526) 0.524 0.529

3	(0.474) 0.476 0.471

Locus 1455 :

2 alleles

99.2% missing data

2	(0.577) 0.588 0.571

3	(0.423) 0.412 0.429

Locus 1456 :

2 alleles

99.2% missing data

4	(0.622) 0.620 0.625

1	(0.378) 0.380 0.375

Locus 1457 :

2 alleles

99.2% missing data

3	(0.619) 0.617 0.619

2	(0.381) 0.383 0.381

Locus 1458 :

2 alleles

97.7% missing data

4	(0.566) 0.569 0.580

3	(0.434) 0.431 0.420

Locus 1459 :

2 alleles

0.4% missing data

2	(0.979) 0.984 0.982

4	(0.021) 0.016 0.018

Locus 1460 :

2 alleles

99.2% missing data

3	(0.520) 0.518 0.522

2	(0.480) 0.482 0.478

Locus 1461 :

2 alleles

88.3% missing data

2	(0.755) 0.802 0.710

1	(0.245) 0.198 0.290

Locus 1462 :

2 alleles

93.0% missing data

3	(0.928) 0.929 0.932

1	(0.072) 0.071 0.068

Locus 1463 :

2 alleles

99.2% missing data

2	(0.313) 0.311 0.320

3	(0.687) 0.689 0.680

Locus 1464 :

2 alleles

98.4% missing data

1	(0.382) 0.381 0.381

2	(0.618) 0.619 0.619

Locus 1465 :

2 alleles

97.7% missing data

1	(0.541) 0.540 0.534

3	(0.459) 0.460 0.466

Locus 1466 :

2 alleles

99.2% missing data

1	(0.386) 0.386 0.385

2	(0.614) 0.614 0.615

Locus 1467 :

2 alleles

99.2% missing data

4	(0.366) 0.367 0.367

1	(0.634) 0.633 0.633

Locus 1468 :

2 alleles

99.2% missing data

3	(0.607) 0.608 0.607

4	(0.393) 0.392 0.393

Locus 1469 :

2 alleles

93.4% missing data

2	(0.553) 0.553 0.560

3	(0.447) 0.447 0.440

Locus 1470 :

2 alleles

99.2% missing data

3	(0.377) 0.379 0.379

1	(0.623) 0.621 0.621

Locus 1471 :

2 alleles

7.8% missing data

2	(0.981) 0.975 0.994

3	(0.019) 0.025 0.006

Locus 1472 :

2 alleles

99.2% missing data

2	(0.426) 0.432 0.426

1	(0.574) 0.568 0.574

Locus 1473 :

2 alleles

98.8% missing data

1	(0.809) 0.806 0.806

2	(0.191) 0.194 0.194

Locus 1474 :

2 alleles

98.4% missing data

1	(0.710) 0.715 0.711

3	(0.290) 0.285 0.289

Locus 1475 :

2 alleles

99.2% missing data

3	(0.213) 0.212 0.220

1	(0.787) 0.788 0.780

Locus 1476 :

2 alleles

99.2% missing data

4	(0.405) 0.402 0.406

1	(0.595) 0.598 0.594

Locus 1477 :

2 alleles

98.8% missing data

1	(0.733) 0.734 0.734

4	(0.267) 0.266 0.266

Locus 1478 :

2 alleles

99.2% missing data

3	(0.756) 0.762 0.753

4	(0.244) 0.238 0.247

Locus 1479 :

2 alleles

99.2% missing data

4	(0.591) 0.585 0.598

3	(0.409) 0.415 0.402

Locus 1480 :

2 alleles

96.9% missing data

3	(0.842) 0.830 0.854

1	(0.158) 0.170 0.146

Locus 1481 :

2 alleles

99.2% missing data

4	(0.690) 0.685 0.689

1	(0.310) 0.315 0.311

Locus 1482 :

2 alleles

99.2% missing data

3	(0.549) 0.543 0.561

1	(0.451) 0.457 0.439

Locus 1483 :

2 alleles

98.8% missing data

1	(0.775) 0.773 0.771

3	(0.225) 0.227 0.229

Locus 1484 :

3 alleles

98.8% missing data

2	(0.168) 0.172 0.171

4	(0.286) 0.286 0.282

1	(0.545) 0.541 0.547

Locus 1485 :

2 alleles

99.2% missing data

1	(0.455) 0.453 0.455

3	(0.545) 0.547 0.545

Locus 1486 :

2 alleles

99.2% missing data

3	(0.121) 0.120 0.131

1	(0.879) 0.880 0.869

Locus 1487 :

2 alleles

99.2% missing data

4	(0.609) 0.610 0.611

1	(0.391) 0.390 0.389

Locus 1488 :

2 alleles

99.2% missing data

2	(0.362) 0.363 0.364

1	(0.638) 0.637 0.636

Locus 1489 :

2 alleles

98.4% missing data

3	(0.838) 0.842 0.837

4	(0.162) 0.158 0.163

Locus 1490 :

2 alleles

99.2% missing data

1	(0.237) 0.235 0.236

3	(0.763) 0.765 0.764

Locus 1491 :

2 alleles

99.2% missing data

3	(0.475) 0.470 0.477

1	(0.525) 0.530 0.523

Locus 1492 :

2 alleles

99.2% missing data

3	(0.848) 0.846 0.848

2	(0.152) 0.154 0.152

Locus 1493 :

3 alleles

98.8% missing data

1	(0.316) 0.311 0.322

2	(0.282) 0.279 0.287

3	(0.401) 0.410 0.391

Locus 1494 :

2 alleles

98.8% missing data

2	(0.662) 0.661 0.670

1	(0.338) 0.339 0.330

Locus 1495 :

2 alleles

99.2% missing data

2	(0.716) 0.713 0.715

4	(0.284) 0.287 0.285

Locus 1496 :

3 alleles

98.8% missing data

3	(0.510) 0.503 0.513

1	(0.209) 0.220 0.203

4	(0.282) 0.277 0.285

Locus 1497 :

2 alleles

99.2% missing data

3	(0.785) 0.788 0.784

4	(0.215) 0.212 0.216

Locus 1498 :

2 alleles

99.2% missing data

2	(0.458) 0.458 0.457

1	(0.542) 0.542 0.543

Locus 1499 :

2 alleles

99.2% missing data

3	(0.615) 0.609 0.620

4	(0.385) 0.391 0.380

Locus 1500 :

2 alleles

98.4% missing data

3	(0.565) 0.564 0.570

4	(0.435) 0.436 0.430

Locus 1501 :

2 alleles

99.2% missing data

1	(0.370) 0.373 0.374

4	(0.630) 0.627 0.626

Locus 1502 :

2 alleles

99.2% missing data

3	(0.290) 0.286 0.292

1	(0.710) 0.714 0.708

Locus 1503 :

2 alleles

99.2% missing data

4	(0.219) 0.225 0.216

1	(0.781) 0.775 0.784

Locus 1504 :

2 alleles

98.8% missing data

1	(0.589) 0.589 0.594

4	(0.411) 0.411 0.406

Locus 1505 :

2 alleles

96.5% missing data

4	(0.592) 0.582 0.613

1	(0.408) 0.418 0.387

Locus 1506 :

2 alleles

99.2% missing data

3	(0.733) 0.734 0.729

2	(0.267) 0.266 0.271

Locus 1507 :

2 alleles

98.4% missing data

4	(0.757) 0.759 0.758

1	(0.243) 0.241 0.242

Locus 1508 :

2 alleles

99.2% missing data

4	(0.565) 0.559 0.569

1	(0.435) 0.441 0.431

Locus 1509 :

3 alleles

97.3% missing data

4	(0.279) 0.290 0.270

1	(0.399) 0.393 0.401

3	(0.322) 0.317 0.329

Locus 1510 :

2 alleles

99.2% missing data

3	(0.700) 0.700 0.702

2	(0.300) 0.300 0.298

Locus 1511 :

2 alleles

98.8% missing data

4	(0.376) 0.375 0.375

2	(0.624) 0.625 0.625

Locus 1512 :

2 alleles

90.6% missing data

1	(0.874) 0.894 0.868

3	(0.126) 0.106 0.132

Locus 1513 :

2 alleles

98.8% missing data

4	(0.551) 0.547 0.553

3	(0.449) 0.453 0.447

Locus 1514 :

2 alleles

98.0% missing data

4	(0.773) 0.775 0.774

1	(0.227) 0.225 0.226

Locus 1515 :

3 alleles

97.3% missing data

3	(0.122) 0.114 0.131

4	(0.673) 0.680 0.667

1	(0.205) 0.206 0.202

Locus 1516 :

2 alleles

98.8% missing data

3	(0.639) 0.628 0.646

2	(0.361) 0.372 0.354

Locus 1517 :

3 alleles

96.5% missing data

3	(0.762) 0.776 0.756

1	(0.164) 0.153 0.166

2	(0.074) 0.071 0.078

Locus 1518 :

2 alleles

98.8% missing data

4	(0.525) 0.525 0.527

1	(0.475) 0.475 0.473

Locus 1519 :

2 alleles

98.0% missing data

1	(0.529) 0.529 0.539

4	(0.471) 0.471 0.461

Locus 1520 :

2 alleles

97.7% missing data

1	(0.852) 0.841 0.860

2	(0.148) 0.159 0.140

Locus 1521 :

2 alleles

91.0% missing data

3	(0.871) 0.897 0.857

2	(0.129) 0.103 0.143

Locus 1522 :

3 alleles

98.8% missing data

2	(0.432) 0.431 0.433

1	(0.240) 0.242 0.240

4	(0.328) 0.327 0.327

Locus 1523 :

2 alleles

99.2% missing data

2	(0.626) 0.627 0.624

1	(0.374) 0.373 0.376

Locus 1524 :

2 alleles

99.2% missing data

2	(0.219) 0.225 0.219

3	(0.781) 0.775 0.781

Locus 1525 :

2 alleles

99.2% missing data

3	(0.439) 0.438 0.443

4	(0.561) 0.562 0.557

Locus 1526 :

2 alleles

99.2% missing data

4	(0.501) 0.499 0.502

1	(0.499) 0.501 0.498

Locus 1527 :

2 alleles

97.3% missing data

4	(0.834) 0.842 0.829

3	(0.166) 0.158 0.171

Locus 1528 :

2 alleles

91.0% missing data

1	(0.903) 0.921 0.893

4	(0.097) 0.079 0.107

Locus 1529 :

2 alleles

99.2% missing data

3	(0.709) 0.710 0.704

1	(0.291) 0.290 0.296

Locus 1530 :

2 alleles

99.2% missing data

3	(0.241) 0.243 0.241

1	(0.759) 0.757 0.759

Locus 1531 :

2 alleles

98.4% missing data

1	(0.525) 0.530 0.531

2	(0.475) 0.470 0.469

Locus 1532 :

2 alleles

96.9% missing data

3	(0.270) 0.268 0.262

1	(0.730) 0.732 0.738

Locus 1533 :

2 alleles

99.2% missing data

3	(0.737) 0.738 0.731

1	(0.263) 0.262 0.269

Locus 1534 :

2 alleles

99.2% missing data

2	(0.428) 0.426 0.426

1	(0.572) 0.574 0.574

Locus 1535 :

2 alleles

99.2% missing data

2	(0.452) 0.443 0.458

1	(0.548) 0.557 0.542

Locus 1536 :

2 alleles

98.8% missing data

2	(0.608) 0.617 0.607

3	(0.392) 0.383 0.393

Locus 1537 :

2 alleles

99.2% missing data

1	(0.429) 0.424 0.428

3	(0.571) 0.576 0.572

Locus 1538 :

2 alleles

99.2% missing data

2	(0.418) 0.417 0.419

1	(0.582) 0.583 0.581

Locus 1539 :

2 alleles

99.2% missing data

2	(0.396) 0.399 0.395

4	(0.604) 0.601 0.605

Locus 1540 :

2 alleles

99.2% missing data

4	(0.604) 0.599 0.600

2	(0.396) 0.401 0.400

Locus 1541 :

2 alleles

99.2% missing data

2	(0.299) 0.304 0.298

1	(0.701) 0.696 0.702

Locus 1542 :

2 alleles

98.8% missing data

4	(0.585) 0.574 0.589

1	(0.415) 0.426 0.411

Locus 1543 :

2 alleles

99.2% missing data

2	(0.388) 0.387 0.394

1	(0.612) 0.613 0.606

Locus 1544 :

2 alleles

98.0% missing data

2	(0.879) 0.875 0.882

4	(0.121) 0.125 0.118

Locus 1545 :

2 alleles

97.7% missing data

4	(0.713) 0.717 0.710

1	(0.287) 0.283 0.290

Locus 1546 :

2 alleles

98.8% missing data

3	(0.476) 0.465 0.490

1	(0.524) 0.535 0.510

Locus 1547 :

2 alleles

99.2% missing data

1	(0.528) 0.532 0.526

4	(0.472) 0.468 0.474

Locus 1548 :

2 alleles

99.2% missing data

2	(0.450) 0.459 0.446

3	(0.550) 0.541 0.554

Locus 1549 :

2 alleles

98.4% missing data

2	(0.688) 0.684 0.687

3	(0.312) 0.316 0.313

Locus 1550 :

2 alleles

98.8% missing data

3	(0.698) 0.702 0.699

4	(0.302) 0.298 0.301

Locus 1551 :

2 alleles

99.2% missing data

3	(0.617) 0.615 0.614

1	(0.383) 0.385 0.386

Locus 1552 :

2 alleles

99.2% missing data

1	(0.398) 0.396 0.402

2	(0.602) 0.604 0.598

Locus 1553 :

3 alleles

97.7% missing data

1	(0.554) 0.555 0.563

4	(0.091) 0.088 0.093

3	(0.355) 0.357 0.344

Locus 1554 :

2 alleles

99.2% missing data

1	(0.483) 0.480 0.489

4	(0.517) 0.520 0.511

Locus 1555 :

2 alleles

99.2% missing data

2	(0.418) 0.422 0.415

1	(0.582) 0.578 0.585

Locus 1556 :

2 alleles

99.2% missing data

1	(0.793) 0.784 0.797

3	(0.207) 0.216 0.203

Locus 1557 :

3 alleles

98.8% missing data

1	(0.159) 0.151 0.170

2	(0.589) 0.595 0.585

3	(0.252) 0.254 0.245

Locus 1558 :

3 alleles

97.3% missing data

3	(0.309) 0.308 0.300

4	(0.221) 0.221 0.214

2	(0.470) 0.471 0.487

Locus 1559 :

2 alleles

99.2% missing data

4	(0.123) 0.121 0.127

2	(0.877) 0.879 0.873

Locus 1560 :

2 alleles

99.2% missing data

3	(0.622) 0.622 0.617

2	(0.378) 0.378 0.383

Locus 1561 :

2 alleles

99.2% missing data

3	(0.567) 0.558 0.578

1	(0.433) 0.442 0.422

Locus 1562 :

2 alleles

96.9% missing data

1	(0.738) 0.751 0.738

4	(0.262) 0.249 0.262

Locus 1563 :

2 alleles

98.8% missing data

3	(0.372) 0.371 0.367

4	(0.628) 0.629 0.633

Locus 1564 :

2 alleles

99.2% missing data

2	(0.708) 0.701 0.713

1	(0.292) 0.299 0.287

Locus 1565 :

2 alleles

99.2% missing data

4	(0.275) 0.282 0.276

3	(0.725) 0.718 0.724

Locus 1566 :

2 alleles

98.0% missing data

2	(0.775) 0.776 0.775

3	(0.225) 0.224 0.225

Locus 1567 :

2 alleles

99.2% missing data

4	(0.573) 0.574 0.579

2	(0.427) 0.426 0.421

Locus 1568 :

2 alleles

99.2% missing data

2	(0.467) 0.466 0.472

3	(0.533) 0.534 0.528

Locus 1569 :

2 alleles

0.0% missing data

4	(0.983) 0.973 0.995

3	(0.017) 0.027 0.005

Locus 1570 :

2 alleles

99.2% missing data

1	(0.369) 0.367 0.372

4	(0.631) 0.633 0.628

Locus 1571 :

2 alleles

95.3% missing data

2	(0.165) 0.164 0.155

1	(0.835) 0.836 0.845

Locus 1572 :

2 alleles

98.8% missing data

4	(0.752) 0.756 0.748

2	(0.248) 0.244 0.252

Locus 1573 :

2 alleles

99.2% missing data

2	(0.341) 0.338 0.344

4	(0.659) 0.662 0.656

Locus 1574 :

2 alleles

99.2% missing data

2	(0.416) 0.419 0.412

3	(0.584) 0.581 0.588

Locus 1575 :

2 alleles

99.2% missing data

2	(0.704) 0.701 0.702

3	(0.296) 0.299 0.298

Locus 1576 :

2 alleles

90.2% missing data

4	(0.843) 0.862 0.833

1	(0.157) 0.138 0.167

Locus 1577 :

2 alleles

96.1% missing data

3	(0.828) 0.833 0.833

4	(0.172) 0.167 0.167

Locus 1578 :

2 alleles

99.2% missing data

2	(0.264) 0.262 0.264

4	(0.736) 0.738 0.736

Locus 1579 :

2 alleles

99.2% missing data

2	(0.331) 0.339 0.321

1	(0.669) 0.661 0.679

Locus 1580 :

2 alleles

99.2% missing data

4	(0.570) 0.572 0.569

2	(0.430) 0.428 0.431

Locus 1581 :

2 alleles

94.9% missing data

4	(0.267) 0.269 0.266

1	(0.733) 0.731 0.734

Locus 1582 :

3 alleles

98.8% missing data

3	(0.366) 0.370 0.362

2	(0.424) 0.421 0.425

4	(0.210) 0.209 0.213

Locus 1583 :

2 alleles

96.9% missing data

3	(0.242) 0.230 0.250

4	(0.758) 0.770 0.750

Locus 1584 :

2 alleles

76.2% missing data

4	(0.960) 0.952 0.976

1	(0.040) 0.048 0.024

Locus 1585 :

3 alleles

98.8% missing data

3	(0.264) 0.264 0.267

1	(0.445) 0.444 0.438

4	(0.291) 0.292 0.296

Locus 1586 :

2 alleles

99.2% missing data

3	(0.563) 0.560 0.570

1	(0.437) 0.440 0.430

Locus 1587 :

2 alleles

98.0% missing data

2	(0.839) 0.844 0.833

4	(0.161) 0.156 0.167

Locus 1588 :

2 alleles

97.7% missing data

2	(0.492) 0.500 0.495

1	(0.508) 0.500 0.505

Locus 1589 :

2 alleles

99.2% missing data

4	(0.765) 0.759 0.767

3	(0.235) 0.241 0.233

Locus 1590 :

2 alleles

99.2% missing data

2	(0.206) 0.202 0.210

1	(0.794) 0.798 0.790

Locus 1591 :

2 alleles

98.0% missing data

3	(0.653) 0.653 0.662

1	(0.347) 0.347 0.338

Locus 1592 :

2 alleles

99.2% missing data

4	(0.591) 0.596 0.586

2	(0.409) 0.404 0.414

Locus 1593 :

2 alleles

99.2% missing data

1	(0.606) 0.602 0.603

4	(0.394) 0.398 0.397

Locus 1594 :

2 alleles

98.0% missing data

2	(0.661) 0.658 0.665

3	(0.339) 0.342 0.335

Locus 1595 :

2 alleles

99.2% missing data

1	(0.560) 0.557 0.563

2	(0.440) 0.443 0.437

Locus 1596 :

3 alleles

98.8% missing data

3	(0.338) 0.335 0.338

2	(0.331) 0.333 0.334

4	(0.330) 0.332 0.328

Locus 1597 :

2 alleles

94.9% missing data

1	(0.815) 0.812 0.817

4	(0.185) 0.188 0.183

Locus 1598 :

2 alleles

98.8% missing data

3	(0.380) 0.384 0.383

1	(0.620) 0.616 0.617

Locus 1599 :

2 alleles

98.8% missing data

1	(0.761) 0.748 0.766

4	(0.239) 0.252 0.234

Locus 1600 :

2 alleles

99.2% missing data

1	(0.654) 0.650 0.656

2	(0.346) 0.350 0.344

Locus 1601 :

2 alleles

99.2% missing data

3	(0.735) 0.737 0.729

1	(0.265) 0.263 0.271

Locus 1602 :

2 alleles

11.3% missing data

4	(0.981) 0.983 0.991

1	(0.019) 0.017 0.009

Locus 1603 :

2 alleles

99.2% missing data

2	(0.656) 0.648 0.663

1	(0.344) 0.352 0.337

Locus 1604 :

3 alleles

98.8% missing data

3	(0.425) 0.419 0.437

1	(0.416) 0.420 0.410

4	(0.159) 0.161 0.154

Locus 1605 :

2 alleles

98.8% missing data

3	(0.485) 0.473 0.490

4	(0.515) 0.527 0.510

Locus 1606 :

2 alleles

99.2% missing data

1	(0.623) 0.629 0.620

3	(0.377) 0.371 0.380

Locus 1607 :

2 alleles

0.4% missing data

2	(0.965) 0.974 0.971

3	(0.035) 0.026 0.029

Locus 1608 :

3 alleles

98.8% missing data

3	(0.138) 0.141 0.138

2	(0.407) 0.404 0.411

1	(0.454) 0.455 0.452

Locus 1609 :

2 alleles

99.2% missing data

1	(0.633) 0.632 0.643

3	(0.367) 0.368 0.357

Locus 1610 :

2 alleles

99.2% missing data

4	(0.512) 0.509 0.518

2	(0.488) 0.491 0.482

Locus 1611 :

2 alleles

90.6% missing data

3	(0.915) 0.928 0.906

2	(0.085) 0.072 0.094

Locus 1612 :

3 alleles

98.4% missing data

1	(0.438) 0.442 0.438

4	(0.287) 0.279 0.291

3	(0.274) 0.279 0.271

Locus 1613 :

2 alleles

98.8% missing data

4	(0.359) 0.361 0.356

1	(0.641) 0.639 0.644

Locus 1614 :

3 alleles

98.8% missing data

4	(0.255) 0.251 0.261

3	(0.451) 0.448 0.453

1	(0.294) 0.301 0.286

Locus 1615 :

2 alleles

98.0% missing data

2	(0.690) 0.696 0.694

3	(0.310) 0.304 0.306

Locus 1616 :

2 alleles

0.8% missing data

2	(0.976) 0.958 0.993

3	(0.024) 0.042 0.007

Locus 1617 :

2 alleles

98.8% missing data

4	(0.489) 0.495 0.487

2	(0.511) 0.505 0.513

Locus 1618 :

2 alleles

99.2% missing data

3	(0.485) 0.491 0.483

2	(0.515) 0.509 0.517

Locus 1619 :

2 alleles

91.0% missing data

2	(0.893) 0.914 0.886

1	(0.107) 0.086 0.114

Locus 1620 :

2 alleles

95.7% missing data

1	(0.144) 0.135 0.145

3	(0.856) 0.865 0.855

Locus 1621 :

2 alleles

99.2% missing data

3	(0.274) 0.275 0.281

1	(0.726) 0.725 0.719

Locus 1622 :

2 alleles

98.4% missing data

4	(0.788) 0.784 0.800

3	(0.212) 0.216 0.200

Locus 1623 :

2 alleles

99.2% missing data

4	(0.341) 0.332 0.344

3	(0.659) 0.668 0.656

Locus 1624 :

3 alleles

98.8% missing data

4	(0.435) 0.434 0.441

3	(0.259) 0.254 0.262

1	(0.306) 0.312 0.297

Locus 1625 :

2 alleles

99.2% missing data

2	(0.633) 0.626 0.638

3	(0.367) 0.374 0.362

Locus 1626 :

2 alleles

99.2% missing data

4	(0.571) 0.569 0.576

3	(0.429) 0.431 0.424

Locus 1627 :

2 alleles

99.2% missing data

1	(0.334) 0.337 0.330

3	(0.666) 0.663 0.670

Locus 1628 :

2 alleles

99.2% missing data

4	(0.515) 0.511 0.518

3	(0.485) 0.489 0.482

Locus 1629 :

2 alleles

99.2% missing data

3	(0.233) 0.229 0.238

4	(0.767) 0.771 0.762

Locus 1630 :

2 alleles

99.2% missing data

4	(0.594) 0.598 0.588

2	(0.406) 0.402 0.412

Locus 1631 :

2 alleles

92.6% missing data

2	(0.124) 0.121 0.112

4	(0.876) 0.879 0.888

Locus 1632 :

2 alleles

99.2% missing data

1	(0.570) 0.573 0.568

2	(0.430) 0.427 0.432

Locus 1633 :

2 alleles

99.2% missing data

1	(0.810) 0.812 0.803

2	(0.190) 0.188 0.197

Locus 1634 :

2 alleles

99.2% missing data

4	(0.301) 0.297 0.301

3	(0.699) 0.703 0.699

Locus 1635 :

2 alleles

99.2% missing data

3	(0.841) 0.845 0.839

1	(0.159) 0.155 0.161

Locus 1636 :

2 alleles

98.4% missing data

4	(0.719) 0.718 0.720

2	(0.281) 0.282 0.280

Locus 1637 :

2 alleles

99.2% missing data

4	(0.235) 0.228 0.241

3	(0.765) 0.772 0.759

Locus 1638 :

2 alleles

99.2% missing data

2	(0.446) 0.450 0.447

1	(0.554) 0.550 0.553

Locus 1639 :

3 alleles

98.4% missing data

1	(0.465) 0.466 0.459

2	(0.317) 0.321 0.315

3	(0.217) 0.213 0.226

Locus 1640 :

2 alleles

99.2% missing data

2	(0.558) 0.563 0.555

3	(0.442) 0.437 0.445

Locus 1641 :

2 alleles

96.5% missing data

4	(0.743) 0.757 0.741

1	(0.257) 0.243 0.259

Locus 1642 :

2 alleles

99.2% missing data

3	(0.314) 0.315 0.314

4	(0.686) 0.685 0.686

Locus 1643 :

2 alleles

93.8% missing data

1	(0.911) 0.912 0.914

4	(0.089) 0.088 0.086

Locus 1644 :

2 alleles

99.2% missing data

2	(0.588) 0.583 0.589

1	(0.412) 0.417 0.411

Locus 1645 :

2 alleles

96.1% missing data

2	(0.805) 0.819 0.800

1	(0.195) 0.181 0.200

Locus 1646 :

2 alleles

95.7% missing data

1	(0.804) 0.807 0.801

2	(0.196) 0.193 0.199

Locus 1647 :

2 alleles

99.2% missing data

4	(0.590) 0.585 0.594

3	(0.410) 0.415 0.406

Locus 1648 :

3 alleles

97.7% missing data

1	(0.556) 0.572 0.553

3	(0.247) 0.242 0.244

4	(0.197) 0.185 0.203

Locus 1649 :

2 alleles

99.2% missing data

1	(0.573) 0.574 0.572

2	(0.427) 0.426 0.428

Locus 1650 :

2 alleles

99.2% missing data

4	(0.395) 0.390 0.401

2	(0.605) 0.610 0.599

Locus 1651 :

2 alleles

99.2% missing data

4	(0.655) 0.651 0.658

1	(0.345) 0.349 0.342

Locus 1652 :

2 alleles

99.2% missing data

3	(0.536) 0.534 0.538

4	(0.464) 0.466 0.462

Locus 1653 :

2 alleles

99.2% missing data

4	(0.330) 0.325 0.336

1	(0.670) 0.675 0.664

Locus 1654 :

2 alleles

96.5% missing data

4	(0.842) 0.844 0.836

3	(0.158) 0.156 0.164

Locus 1655 :

2 alleles

98.8% missing data

4	(0.473) 0.466 0.475

3	(0.527) 0.534 0.525

Locus 1656 :

2 alleles

98.8% missing data

1	(0.416) 0.417 0.425

4	(0.584) 0.583 0.575

Locus 1657 :

2 alleles

96.9% missing data

3	(0.803) 0.807 0.807

1	(0.197) 0.193 0.193

Locus 1658 :

2 alleles

97.3% missing data

3	(0.229) 0.235 0.224

4	(0.771) 0.765 0.776

Locus 1659 :

3 alleles

98.4% missing data

2	(0.523) 0.521 0.521

4	(0.101) 0.095 0.109

3	(0.376) 0.384 0.370

Locus 1660 :

2 alleles

96.1% missing data

1	(0.891) 0.897 0.887

3	(0.109) 0.103 0.113

Locus 1661 :

3 alleles

98.0% missing data

2	(0.366) 0.367 0.378

1	(0.169) 0.164 0.172

3	(0.464) 0.468 0.450

Locus 1662 :

2 alleles

92.6% missing data

4	(0.862) 0.854 0.874

1	(0.138) 0.146 0.126

Locus 1663 :

2 alleles

98.8% missing data

1	(0.824) 0.829 0.820

2	(0.176) 0.171 0.180

Locus 1664 :

2 alleles

99.2% missing data

2	(0.351) 0.352 0.358

4	(0.649) 0.648 0.642

Locus 1665 :

3 alleles

95.7% missing data

3	(0.160) 0.150 0.162

1	(0.679) 0.692 0.681

2	(0.161) 0.158 0.157

Locus 1666 :

2 alleles

98.8% missing data

4	(0.334) 0.329 0.339

2	(0.666) 0.671 0.661

Locus 1667 :

2 alleles

98.0% missing data

1	(0.718) 0.718 0.724

4	(0.282) 0.282 0.276

Locus 1668 :

2 alleles

99.2% missing data

2	(0.668) 0.667 0.661

3	(0.332) 0.333 0.339

Locus 1669 :

2 alleles

99.2% missing data

3	(0.310) 0.310 0.311

1	(0.690) 0.690 0.689

Locus 1670 :

2 alleles

0.8% missing data

2	(0.985) 0.982 0.995

3	(0.015) 0.018 0.005

Locus 1671 :

2 alleles

99.2% missing data

1	(0.686) 0.684 0.681

4	(0.314) 0.316 0.319

Locus 1672 :

2 alleles

98.0% missing data

4	(0.764) 0.763 0.766

2	(0.236) 0.237 0.234

Locus 1673 :

2 alleles

99.2% missing data

4	(0.477) 0.475 0.482

3	(0.523) 0.525 0.518

Locus 1674 :

2 alleles

99.2% missing data

3	(0.600) 0.596 0.608

2	(0.400) 0.404 0.392

Locus 1675 :

2 alleles

96.1% missing data

2	(0.782) 0.787 0.785

4	(0.218) 0.213 0.215

Locus 1676 :

2 alleles

98.8% missing data

1	(0.643) 0.645 0.643

3	(0.357) 0.355 0.357

Locus 1677 :

2 alleles

91.0% missing data

2	(0.916) 0.931 0.907

1	(0.084) 0.069 0.093

Locus 1678 :

2 alleles

91.0% missing data

3	(0.936) 0.939 0.936

4	(0.064) 0.061 0.064

Locus 1679 :

2 alleles

99.2% missing data

2	(0.186) 0.193 0.184

4	(0.814) 0.807 0.816

Locus 1680 :

3 alleles

98.4% missing data

3	(0.285) 0.282 0.291

2	(0.380) 0.375 0.384

1	(0.334) 0.343 0.325

Locus 1681 :

2 alleles

99.2% missing data

1	(0.519) 0.516 0.520

3	(0.481) 0.484 0.480

Locus 1682 :

2 alleles

99.2% missing data

4	(0.264) 0.269 0.263

1	(0.736) 0.731 0.737

Locus 1683 :

2 alleles

99.2% missing data

3	(0.455) 0.464 0.455

2	(0.545) 0.536 0.545

Locus 1684 :

2 alleles

91.4% missing data

1	(0.857) 0.882 0.849

2	(0.143) 0.118 0.151

Locus 1685 :

2 alleles

98.8% missing data

4	(0.709) 0.714 0.709

2	(0.291) 0.286 0.291

Locus 1686 :

2 alleles

99.2% missing data

1	(0.695) 0.694 0.698

4	(0.305) 0.306 0.302

Locus 1687 :

2 alleles

99.2% missing data

4	(0.505) 0.500 0.506

1	(0.495) 0.500 0.494

Locus 1688 :

2 alleles

99.2% missing data

4	(0.730) 0.727 0.735

3	(0.270) 0.273 0.265

Locus 1689 :

2 alleles

99.2% missing data

4	(0.252) 0.246 0.258

2	(0.748) 0.754 0.742

Locus 1690 :

2 alleles

99.2% missing data

1	(0.734) 0.729 0.738

3	(0.266) 0.271 0.262

Locus 1691 :

2 alleles

99.2% missing data

3	(0.570) 0.567 0.573

2	(0.430) 0.433 0.427

Locus 1692 :

2 alleles

99.2% missing data

3	(0.466) 0.463 0.465

4	(0.534) 0.537 0.535

Locus 1693 :

2 alleles

98.8% missing data

3	(0.313) 0.312 0.318

2	(0.687) 0.688 0.682

Locus 1694 :

2 alleles

0.4% missing data

4	(0.986) 0.990 0.993

1	(0.014) 0.010 0.007

Locus 1695 :

2 alleles

91.0% missing data

2	(0.916) 0.933 0.910

4	(0.084) 0.067 0.090

Locus 1696 :

3 alleles

98.0% missing data

1	(0.367) 0.367 0.363

2	(0.470) 0.463 0.478

4	(0.163) 0.169 0.159

Locus 1697 :

2 alleles

99.2% missing data

3	(0.503) 0.503 0.506

2	(0.497) 0.497 0.494

Locus 1698 :

3 alleles

94.9% missing data

4	(0.285) 0.274 0.288

1	(0.618) 0.627 0.616

3	(0.097) 0.099 0.096

Locus 1699 :

2 alleles

98.8% missing data

4	(0.657) 0.656 0.665

2	(0.343) 0.344 0.335

Locus 1700 :

2 alleles

99.2% missing data

3	(0.642) 0.644 0.641

1	(0.358) 0.356 0.359

Locus 1701 :

2 alleles

99.2% missing data

2	(0.451) 0.449 0.453

3	(0.549) 0.551 0.547

Locus 1702 :

2 alleles

99.2% missing data

2	(0.460) 0.456 0.461

1	(0.540) 0.544 0.539

Locus 1703 :

3 alleles

97.7% missing data

4	(0.191) 0.187 0.194

2	(0.545) 0.562 0.534

1	(0.264) 0.250 0.272

Locus 1704 :

2 alleles

99.2% missing data

3	(0.690) 0.689 0.689

4	(0.310) 0.311 0.311

Locus 1705 :

2 alleles

99.2% missing data

4	(0.468) 0.467 0.467

2	(0.532) 0.533 0.533

Locus 1706 :

2 alleles

96.5% missing data

4	(0.870) 0.866 0.880

1	(0.130) 0.134 0.120

Locus 1707 :

2 alleles

96.9% missing data

1	(0.822) 0.815 0.839

3	(0.178) 0.185 0.161

Locus 1708 :

2 alleles

98.0% missing data

1	(0.725) 0.735 0.722

2	(0.275) 0.265 0.278

Locus 1709 :

2 alleles

98.8% missing data

3	(0.461) 0.468 0.470

2	(0.539) 0.532 0.530

Locus 1710 :

2 alleles

99.2% missing data

3	(0.596) 0.598 0.589

2	(0.404) 0.402 0.411

Locus 1711 :

2 alleles

99.2% missing data

4	(0.693) 0.693 0.688

2	(0.307) 0.307 0.312

Locus 1712 :

2 alleles

99.2% missing data

1	(0.541) 0.534 0.540

2	(0.459) 0.466 0.460

Locus 1713 :

2 alleles

99.2% missing data

2	(0.533) 0.533 0.532

1	(0.467) 0.467 0.468

Locus 1714 :

2 alleles

99.2% missing data

2	(0.561) 0.554 0.560

3	(0.439) 0.446 0.440

Locus 1715 :

2 alleles

99.2% missing data

2	(0.711) 0.706 0.710

1	(0.289) 0.294 0.290

Locus 1716 :

2 alleles

99.2% missing data

2	(0.289) 0.289 0.285

1	(0.711) 0.711 0.715

Locus 1717 :

2 alleles

99.2% missing data

2	(0.194) 0.193 0.203

1	(0.806) 0.807 0.797

Locus 1718 :

2 alleles

93.8% missing data

4	(0.890) 0.889 0.897

2	(0.110) 0.111 0.103

Locus 1719 :

2 alleles

99.2% missing data

4	(0.607) 0.605 0.607

2	(0.393) 0.395 0.393

Locus 1720 :

2 alleles

89.8% missing data

2	(0.097) 0.077 0.113

3	(0.903) 0.923 0.887

Locus 1721 :

2 alleles

99.2% missing data

2	(0.371) 0.368 0.375

3	(0.629) 0.632 0.625

Locus 1722 :

2 alleles

0.0% missing data

4	(0.985) 0.982 0.995

1	(0.015) 0.018 0.005

Locus 1723 :

2 alleles

99.2% missing data

4	(0.395) 0.394 0.395

2	(0.605) 0.606 0.605

Locus 1724 :

2 alleles

92.6% missing data

4	(0.886) 0.883 0.894

2	(0.114) 0.117 0.106

Locus 1725 :

2 alleles

90.6% missing data

2	(0.897) 0.907 0.899

1	(0.103) 0.093 0.101

Locus 1726 :

2 alleles

99.2% missing data

4	(0.397) 0.403 0.393

1	(0.603) 0.597 0.607

Locus 1727 :

2 alleles

8.2% missing data

2	(0.982) 0.974 0.995

1	(0.018) 0.026 0.005

Locus 1728 :

2 alleles

97.7% missing data

3	(0.615) 0.630 0.616

2	(0.385) 0.370 0.384

Locus 1729 :

2 alleles

98.8% missing data

4	(0.635) 0.633 0.637

1	(0.365) 0.367 0.363

Locus 1730 :

2 alleles

99.2% missing data

1	(0.739) 0.734 0.739

4	(0.261) 0.266 0.261

Locus 1731 :

3 alleles

85.9% missing data

4	(0.774) 0.783 0.786

3	(0.145) 0.142 0.140

1	(0.081) 0.074 0.075

Locus 1732 :

2 alleles

98.4% missing data

2	(0.511) 0.511 0.512

4	(0.489) 0.489 0.488

Locus 1733 :

2 alleles

99.2% missing data

2	(0.683) 0.673 0.686

3	(0.317) 0.327 0.314

Locus 1734 :

2 alleles

99.2% missing data

3	(0.316) 0.316 0.318

2	(0.684) 0.684 0.682

Locus 1735 :

2 alleles

99.2% missing data

4	(0.435) 0.437 0.441

1	(0.565) 0.563 0.559

Locus 1736 :

2 alleles

99.2% missing data

1	(0.539) 0.539 0.546

2	(0.461) 0.461 0.454

Locus 1737 :

2 alleles

99.2% missing data

4	(0.709) 0.700 0.711

3	(0.291) 0.300 0.289

Locus 1738 :

2 alleles

98.8% missing data

2	(0.594) 0.589 0.603

4	(0.406) 0.411 0.397

Locus 1739 :

2 alleles

99.2% missing data

3	(0.580) 0.582 0.580

4	(0.420) 0.418 0.420

Locus 1740 :

2 alleles

98.8% missing data

1	(0.764) 0.773 0.759

4	(0.236) 0.227 0.241

Locus 1741 :

2 alleles

90.6% missing data

2	(0.920) 0.936 0.916

1	(0.080) 0.064 0.084

Locus 1742 :

2 alleles

98.8% missing data

2	(0.437) 0.433 0.442

1	(0.563) 0.567 0.558

Locus 1743 :

2 alleles

99.2% missing data

2	(0.578) 0.573 0.587

1	(0.422) 0.427 0.413

Locus 1744 :

3 alleles

98.8% missing data

4	(0.603) 0.601 0.604

3	(0.245) 0.250 0.238

2	(0.152) 0.150 0.158

Locus 1745 :

2 alleles

99.2% missing data

3	(0.562) 0.561 0.558

2	(0.438) 0.439 0.442

Locus 1746 :

2 alleles

99.2% missing data

4	(0.708) 0.711 0.705

2	(0.292) 0.289 0.295

Locus 1747 :

2 alleles

99.2% missing data

3	(0.174) 0.169 0.182

2	(0.826) 0.831 0.818

Locus 1748 :

2 alleles

99.2% missing data

1	(0.332) 0.330 0.342

2	(0.668) 0.670 0.658

Locus 1749 :

2 alleles

99.2% missing data

1	(0.228) 0.226 0.235

2	(0.772) 0.774 0.765

Locus 1750 :

2 alleles

99.2% missing data

2	(0.295) 0.296 0.297

1	(0.705) 0.704 0.703

Locus 1751 :

2 alleles

99.2% missing data

1	(0.491) 0.486 0.494

2	(0.509) 0.514 0.506

Locus 1752 :

2 alleles

99.2% missing data

3	(0.493) 0.493 0.493

1	(0.507) 0.507 0.507

Locus 1753 :

2 alleles

97.7% missing data

1	(0.656) 0.647 0.674

3	(0.344) 0.353 0.326

Locus 1754 :

2 alleles

99.2% missing data

3	(0.668) 0.663 0.671

2	(0.332) 0.337 0.329

Locus 1755 :

2 alleles

98.8% missing data

1	(0.492) 0.488 0.500

4	(0.508) 0.512 0.500

Locus 1756 :

2 alleles

99.2% missing data

1	(0.667) 0.668 0.664

2	(0.333) 0.332 0.336

Locus 1757 :

2 alleles

99.2% missing data

2	(0.335) 0.333 0.340

3	(0.665) 0.667 0.660

Locus 1758 :

2 alleles

99.2% missing data

3	(0.695) 0.699 0.691

4	(0.305) 0.301 0.309

Locus 1759 :

2 alleles

99.2% missing data

1	(0.634) 0.640 0.627

4	(0.366) 0.360 0.373

Locus 1760 :

2 alleles

99.2% missing data

3	(0.695) 0.686 0.698

1	(0.305) 0.314 0.302

Locus 1761 :

2 alleles

98.0% missing data

1	(0.700) 0.702 0.701

4	(0.300) 0.298 0.299

Locus 1762 :

2 alleles

99.2% missing data

3	(0.224) 0.227 0.222

1	(0.776) 0.773 0.778

Locus 1763 :

2 alleles

99.2% missing data

1	(0.576) 0.578 0.579

2	(0.424) 0.422 0.421

Locus 1764 :

2 alleles

99.2% missing data

4	(0.601) 0.601 0.604

2	(0.399) 0.399 0.396

Locus 1765 :

2 alleles

96.5% missing data

3	(0.286) 0.269 0.287

1	(0.714) 0.731 0.713

Locus 1766 :

2 alleles

99.2% missing data

4	(0.363) 0.371 0.359

2	(0.637) 0.629 0.641

Locus 1767 :

2 alleles

99.2% missing data

4	(0.769) 0.773 0.767

3	(0.231) 0.227 0.233

Locus 1768 :

2 alleles

99.2% missing data

4	(0.713) 0.712 0.713

2	(0.287) 0.288 0.287

Locus 1769 :

2 alleles

98.8% missing data

1	(0.607) 0.602 0.611

2	(0.393) 0.398 0.389

Locus 1770 :

2 alleles

99.2% missing data

1	(0.379) 0.381 0.375

2	(0.621) 0.619 0.625

Locus 1771 :

2 alleles

98.4% missing data

2	(0.335) 0.340 0.331

3	(0.665) 0.660 0.669

Locus 1772 :

2 alleles

99.2% missing data

3	(0.630) 0.637 0.622

4	(0.370) 0.363 0.378

Locus 1773 :

2 alleles

99.2% missing data

3	(0.387) 0.388 0.383

2	(0.613) 0.612 0.617

Locus 1774 :

2 alleles

99.2% missing data

2	(0.714) 0.710 0.711

4	(0.286) 0.290 0.289

Locus 1775 :

2 alleles

97.7% missing data

3	(0.138) 0.136 0.136

4	(0.862) 0.864 0.864

Locus 1776 :

2 alleles

97.7% missing data

1	(0.242) 0.241 0.231

3	(0.758) 0.759 0.769

Locus 1777 :

2 alleles

96.5% missing data

3	(0.837) 0.851 0.830

2	(0.163) 0.149 0.170

Locus 1778 :

2 alleles

99.2% missing data

3	(0.692) 0.694 0.690

1	(0.308) 0.306 0.310

Locus 1779 :

2 alleles

95.7% missing data

2	(0.726) 0.736 0.723

4	(0.274) 0.264 0.277

Locus 1780 :

2 alleles

98.4% missing data

3	(0.607) 0.602 0.610

4	(0.393) 0.398 0.390

Locus 1781 :

2 alleles

98.0% missing data

1	(0.637) 0.650 0.631

3	(0.363) 0.350 0.369

Locus 1782 :

2 alleles

99.2% missing data

4	(0.546) 0.536 0.553

2	(0.454) 0.464 0.447

Locus 1783 :

2 alleles

99.2% missing data

2	(0.773) 0.763 0.783

3	(0.227) 0.237 0.217

Locus 1784 :

2 alleles

99.2% missing data

4	(0.334) 0.327 0.340

2	(0.666) 0.673 0.660

Locus 1785 :

2 alleles

99.2% missing data

1	(0.799) 0.792 0.804

3	(0.201) 0.208 0.196

Locus 1786 :

2 alleles

99.2% missing data

3	(0.471) 0.477 0.469

1	(0.529) 0.523 0.531

Locus 1787 :

2 alleles

99.2% missing data

3	(0.418) 0.417 0.416

2	(0.582) 0.583 0.584

Locus 1788 :

2 alleles

99.2% missing data

4	(0.469) 0.470 0.470

2	(0.531) 0.530 0.530

Locus 1789 :

2 alleles

98.8% missing data

3	(0.575) 0.574 0.575

2	(0.425) 0.426 0.425

Locus 1790 :

2 alleles

98.8% missing data

1	(0.781) 0.784 0.775

3	(0.219) 0.216 0.225

Locus 1791 :

2 alleles

99.2% missing data

1	(0.415) 0.411 0.419

3	(0.585) 0.589 0.581

Locus 1792 :

2 alleles

99.2% missing data

4	(0.392) 0.392 0.394

3	(0.608) 0.608 0.606

Locus 1793 :

2 alleles

99.2% missing data

1	(0.477) 0.465 0.484

4	(0.523) 0.535 0.516

Locus 1794 :

2 alleles

98.4% missing data

2	(0.379) 0.379 0.374

1	(0.621) 0.621 0.626

Locus 1795 :

2 alleles

99.2% missing data

1	(0.577) 0.576 0.575

3	(0.423) 0.424 0.425

Locus 1796 :

2 alleles

98.4% missing data

3	(0.828) 0.838 0.826

4	(0.172) 0.162 0.174

Locus 1797 :

2 alleles

99.2% missing data

1	(0.531) 0.536 0.530

3	(0.469) 0.464 0.470

Locus 1798 :

2 alleles

99.2% missing data

4	(0.650) 0.652 0.641

3	(0.350) 0.348 0.359

Locus 1799 :

2 alleles

92.6% missing data

3	(0.905) 0.904 0.911

1	(0.095) 0.096 0.089

Locus 1800 :

2 alleles

97.3% missing data

1	(0.378) 0.391 0.364

3	(0.622) 0.609 0.636

Locus 1801 :

2 alleles

98.8% missing data

1	(0.665) 0.662 0.669

2	(0.335) 0.338 0.331

Locus 1802 :

2 alleles

99.2% missing data

1	(0.397) 0.397 0.404

4	(0.603) 0.603 0.596

Locus 1803 :

2 alleles

98.8% missing data

2	(0.555) 0.556 0.555

4	(0.445) 0.444 0.445

Locus 1804 :

2 alleles

97.3% missing data

2	(0.374) 0.355 0.387

3	(0.626) 0.645 0.613

Locus 1805 :

2 alleles

99.2% missing data

4	(0.598) 0.595 0.595

2	(0.402) 0.405 0.405

Locus 1806 :

3 alleles

95.3% missing data

2	(0.101) 0.096 0.101

4	(0.771) 0.787 0.764

1	(0.128) 0.117 0.135

Locus 1807 :

2 alleles

98.8% missing data

3	(0.773) 0.781 0.773

2	(0.227) 0.219 0.227

Locus 1808 :

2 alleles

0.0% missing data

1	(0.981) 0.977 0.995

4	(0.019) 0.023 0.005

Locus 1809 :

2 alleles

99.2% missing data

4	(0.494) 0.502 0.499

3	(0.506) 0.498 0.501

Locus 1810 :

2 alleles

98.4% missing data

2	(0.791) 0.794 0.787

3	(0.209) 0.206 0.213

Locus 1811 :

2 alleles

99.2% missing data

2	(0.475) 0.473 0.479

1	(0.525) 0.527 0.521

Locus 1812 :

2 alleles

93.0% missing data

1	(0.906) 0.905 0.914

4	(0.094) 0.095 0.086

Locus 1813 :

2 alleles

92.6% missing data

4	(0.808) 0.804 0.824

2	(0.192) 0.196 0.176

Locus 1814 :

2 alleles

99.2% missing data

3	(0.517) 0.520 0.518

2	(0.483) 0.480 0.482

Locus 1815 :

2 alleles

99.2% missing data

3	(0.420) 0.423 0.418

1	(0.580) 0.577 0.582

Locus 1816 :

2 alleles

99.2% missing data

4	(0.298) 0.297 0.305

3	(0.702) 0.703 0.695

Locus 1817 :

2 alleles

96.1% missing data

3	(0.848) 0.864 0.841

1	(0.152) 0.136 0.159

Locus 1818 :

3 alleles

98.4% missing data

4	(0.484) 0.484 0.486

2	(0.190) 0.186 0.187

3	(0.327) 0.329 0.328

Locus 1819 :

2 alleles

99.2% missing data

2	(0.344) 0.338 0.350

3	(0.656) 0.662 0.650

Locus 1820 :

2 alleles

99.2% missing data

1	(0.573) 0.570 0.572

2	(0.427) 0.430 0.428

Locus 1821 :

2 alleles

99.2% missing data

1	(0.617) 0.619 0.615

3	(0.383) 0.381 0.385

Locus 1822 :

3 alleles

98.8% missing data

2	(0.209) 0.208 0.210

3	(0.587) 0.582 0.588

4	(0.204) 0.210 0.202

Locus 1823 :

2 alleles

99.2% missing data

1	(0.486) 0.484 0.492

2	(0.514) 0.516 0.508

Locus 1824 :

2 alleles

99.2% missing data

2	(0.469) 0.466 0.465

4	(0.531) 0.534 0.535

Locus 1825 :

3 alleles

98.8% missing data

2	(0.593) 0.587 0.593

3	(0.281) 0.283 0.283

1	(0.126) 0.130 0.124

Locus 1826 :

2 alleles

99.2% missing data

2	(0.656) 0.647 0.661

3	(0.344) 0.353 0.339

Locus 1827 :

2 alleles

99.2% missing data

4	(0.514) 0.518 0.517

2	(0.486) 0.482 0.483

Locus 1828 :

2 alleles

99.2% missing data

1	(0.786) 0.780 0.790

3	(0.214) 0.220 0.210

Locus 1829 :

2 alleles

99.2% missing data

3	(0.606) 0.608 0.608

1	(0.394) 0.392 0.392

Locus 1830 :

2 alleles

96.9% missing data

1	(0.781) 0.776 0.789

4	(0.219) 0.224 0.211

Locus 1831 :

2 alleles

99.2% missing data

3	(0.476) 0.470 0.474

4	(0.524) 0.530 0.526

Locus 1832 :

2 alleles

99.2% missing data

2	(0.517) 0.514 0.518

3	(0.483) 0.486 0.482

Locus 1833 :

2 alleles

99.2% missing data

1	(0.519) 0.523 0.513

4	(0.481) 0.477 0.487

Locus 1834 :

2 alleles

99.2% missing data

3	(0.631) 0.632 0.630

4	(0.369) 0.368 0.370

Locus 1835 :

2 alleles

99.2% missing data

1	(0.594) 0.593 0.590

3	(0.406) 0.407 0.410

Locus 1836 :

2 alleles

99.2% missing data

4	(0.419) 0.418 0.418

1	(0.581) 0.582 0.582

Locus 1837 :

2 alleles

0.4% missing data

1	(0.986) 0.981 0.996

4	(0.014) 0.019 0.004

Locus 1838 :

2 alleles

99.2% missing data

4	(0.643) 0.645 0.645

1	(0.357) 0.355 0.355

Locus 1839 :

2 alleles

92.2% missing data

3	(0.902) 0.893 0.917

2	(0.098) 0.107 0.083

Locus 1840 :

2 alleles

97.3% missing data

3	(0.295) 0.295 0.296

4	(0.705) 0.705 0.704

Locus 1841 :

2 alleles

99.2% missing data

4	(0.386) 0.383 0.387

3	(0.614) 0.617 0.613

Locus 1842 :

2 alleles

98.0% missing data

4	(0.655) 0.656 0.662

1	(0.345) 0.344 0.338

Locus 1843 :

2 alleles

98.0% missing data

3	(0.802) 0.806 0.796

4	(0.198) 0.194 0.204

Locus 1844 :

2 alleles

99.2% missing data

4	(0.482) 0.477 0.488

3	(0.518) 0.523 0.512

Locus 1845 :

2 alleles

99.2% missing data

3	(0.730) 0.734 0.734

1	(0.270) 0.266 0.266

Locus 1846 :

2 alleles

95.3% missing data

1	(0.788) 0.804 0.766

3	(0.212) 0.196 0.234

Locus 1847 :

2 alleles

98.8% missing data

1	(0.428) 0.434 0.425

2	(0.572) 0.566 0.575

Locus 1848 :

2 alleles

99.2% missing data

4	(0.458) 0.457 0.467

3	(0.542) 0.543 0.533

Locus 1849 :

2 alleles

99.2% missing data

2	(0.506) 0.511 0.503

3	(0.494) 0.489 0.497

Locus 1850 :

2 alleles

99.2% missing data

4	(0.182) 0.181 0.184

2	(0.818) 0.819 0.816

Locus 1851 :

2 alleles

99.2% missing data

3	(0.408) 0.402 0.410

1	(0.592) 0.598 0.590

Locus 1852 :

2 alleles

99.2% missing data

4	(0.557) 0.555 0.559

2	(0.443) 0.445 0.441

Locus 1853 :

2 alleles

99.2% missing data

3	(0.361) 0.357 0.366

4	(0.639) 0.643 0.634

Locus 1854 :

2 alleles

99.2% missing data

1	(0.496) 0.486 0.499

3	(0.504) 0.514 0.501

Locus 1855 :

2 alleles

99.2% missing data

1	(0.553) 0.553 0.554

4	(0.447) 0.447 0.446

Locus 1856 :

2 alleles

98.4% missing data

3	(0.572) 0.573 0.573

1	(0.428) 0.427 0.427

Locus 1857 :

2 alleles

99.2% missing data

1	(0.578) 0.576 0.588

2	(0.422) 0.424 0.412

Locus 1858 :

2 alleles

99.2% missing data

4	(0.635) 0.639 0.634

1	(0.365) 0.361 0.366

Locus 1859 :

2 alleles

99.2% missing data

1	(0.532) 0.538 0.527

4	(0.468) 0.462 0.473

Locus 1860 :

2 alleles

98.8% missing data

3	(0.345) 0.346 0.342

1	(0.655) 0.654 0.658

Locus 1861 :

2 alleles

99.2% missing data

3	(0.450) 0.452 0.451

2	(0.550) 0.548 0.549

Locus 1862 :

2 alleles

96.1% missing data

2	(0.869) 0.870 0.875

3	(0.131) 0.130 0.125

Locus 1863 :

2 alleles

99.2% missing data

1	(0.515) 0.515 0.520

2	(0.485) 0.485 0.480

Locus 1864 :

3 alleles

95.7% missing data

1	(0.141) 0.135 0.141

2	(0.732) 0.747 0.727

4	(0.127) 0.118 0.132

Locus 1865 :

2 alleles

99.2% missing data

1	(0.541) 0.542 0.540

3	(0.459) 0.458 0.460

Locus 1866 :

2 alleles

99.2% missing data

4	(0.496) 0.501 0.497

2	(0.504) 0.499 0.503

Locus 1867 :

2 alleles

98.8% missing data

3	(0.611) 0.601 0.614

4	(0.389) 0.399 0.386

Locus 1868 :

2 alleles

96.5% missing data

1	(0.216) 0.215 0.217

4	(0.784) 0.785 0.783

Locus 1869 :

2 alleles

99.2% missing data

2	(0.817) 0.810 0.821

1	(0.183) 0.190 0.179

Locus 1870 :

2 alleles

99.2% missing data

1	(0.383) 0.386 0.393

4	(0.617) 0.614 0.607

Locus 1871 :

2 alleles

98.8% missing data

1	(0.456) 0.448 0.464

4	(0.544) 0.552 0.536

Locus 1872 :

2 alleles

98.4% missing data

1	(0.694) 0.700 0.692

4	(0.306) 0.300 0.308

Locus 1873 :

2 alleles

99.2% missing data

4	(0.341) 0.341 0.344

3	(0.659) 0.659 0.656

Locus 1874 :

2 alleles

99.2% missing data

2	(0.626) 0.628 0.620

4	(0.374) 0.372 0.380

Locus 1875 :

2 alleles

99.2% missing data

3	(0.390) 0.384 0.397

1	(0.610) 0.616 0.603

Locus 1876 :

2 alleles

98.8% missing data

1	(0.579) 0.579 0.580

2	(0.421) 0.421 0.420

Locus 1877 :

2 alleles

98.4% missing data

1	(0.734) 0.737 0.736

4	(0.266) 0.263 0.264

Locus 1878 :

2 alleles

98.8% missing data

4	(0.679) 0.673 0.692

1	(0.321) 0.327 0.308

Locus 1879 :

2 alleles

99.2% missing data

2	(0.308) 0.316 0.307

1	(0.692) 0.684 0.693

Locus 1880 :

3 alleles

98.8% missing data

2	(0.207) 0.205 0.208

1	(0.437) 0.443 0.428

3	(0.356) 0.352 0.364

Locus 1881 :

2 alleles

90.6% missing data

1	(0.865) 0.882 0.857

3	(0.135) 0.118 0.143

Locus 1882 :

2 alleles

98.8% missing data

3	(0.603) 0.602 0.605

2	(0.397) 0.398 0.395

Locus 1883 :

2 alleles

99.2% missing data

2	(0.564) 0.565 0.563

1	(0.436) 0.435 0.437

Locus 1884 :

2 alleles

0.8% missing data

1	(0.981) 0.985 0.991

4	(0.019) 0.015 0.009

Locus 1885 :

2 alleles

99.2% missing data

1	(0.405) 0.405 0.411

4	(0.595) 0.595 0.589

Locus 1886 :

2 alleles

15.2% missing data

3	(0.977) 0.963 0.992

2	(0.023) 0.037 0.008

Locus 1887 :

2 alleles

99.2% missing data

1	(0.493) 0.491 0.492

3	(0.507) 0.509 0.508

Locus 1888 :

2 alleles

91.0% missing data

2	(0.888) 0.906 0.885

1	(0.112) 0.094 0.115

Locus 1889 :

2 alleles

98.8% missing data

1	(0.570) 0.563 0.577

2	(0.430) 0.437 0.423

Locus 1890 :

2 alleles

99.2% missing data

1	(0.523) 0.516 0.533

3	(0.477) 0.484 0.467

Locus 1891 :

2 alleles

98.8% missing data

2	(0.307) 0.308 0.306

1	(0.693) 0.692 0.694

Locus 1892 :

2 alleles

99.2% missing data

1	(0.440) 0.427 0.445

4	(0.560) 0.573 0.555

Locus 1893 :

2 alleles

98.4% missing data

2	(0.650) 0.657 0.647

3	(0.350) 0.343 0.353

Locus 1894 :

2 alleles

96.9% missing data

2	(0.418) 0.423 0.410

3	(0.582) 0.577 0.590

Locus 1895 :

2 alleles

99.2% missing data

2	(0.293) 0.299 0.290

1	(0.707) 0.701 0.710

Locus 1896 :

2 alleles

99.2% missing data

1	(0.612) 0.608 0.615

4	(0.388) 0.392 0.385

Locus 1897 :

2 alleles

99.2% missing data

3	(0.541) 0.543 0.536

4	(0.459) 0.457 0.464

Locus 1898 :

2 alleles

99.2% missing data

4	(0.285) 0.280 0.289

1	(0.715) 0.720 0.711

Locus 1899 :

3 alleles

97.3% missing data

4	(0.516) 0.516 0.536

2	(0.317) 0.319 0.300

3	(0.167) 0.165 0.164

Locus 1900 :

2 alleles

0.0% missing data

3	(0.987) 0.982 0.996

1	(0.013) 0.018 0.004

Locus 1901 :

2 alleles

91.0% missing data

1	(0.892) 0.890 0.905

3	(0.108) 0.110 0.095

Locus 1902 :

2 alleles

0.8% missing data

2	(0.988) 0.991 0.993

1	(0.012) 0.009 0.007

Locus 1903 :

2 alleles

99.2% missing data

1	(0.527) 0.525 0.530

2	(0.473) 0.475 0.470

Locus 1904 :

2 alleles

99.2% missing data

1	(0.621) 0.617 0.619

2	(0.379) 0.383 0.381

Locus 1905 :

2 alleles

99.2% missing data

3	(0.403) 0.408 0.399

2	(0.597) 0.592 0.601

Locus 1906 :

2 alleles

97.7% missing data

2	(0.843) 0.833 0.851

1	(0.157) 0.167 0.149

Locus 1907 :

2 alleles

99.2% missing data

3	(0.546) 0.540 0.543

1	(0.454) 0.460 0.457

Locus 1908 :

2 alleles

98.8% missing data

4	(0.664) 0.666 0.665

3	(0.336) 0.334 0.335

Locus 1909 :

2 alleles

99.2% missing data

3	(0.652) 0.658 0.649

2	(0.348) 0.342 0.351

Locus 1910 :

2 alleles

99.2% missing data

1	(0.579) 0.574 0.576

4	(0.421) 0.426 0.424

Locus 1911 :

2 alleles

98.4% missing data

2	(0.802) 0.807 0.801

1	(0.198) 0.193 0.199

Locus 1912 :

2 alleles

99.2% missing data

4	(0.349) 0.353 0.350

1	(0.651) 0.647 0.650

Locus 1913 :

2 alleles

9.0% missing data

2	(0.982) 0.975 0.995

3	(0.018) 0.025 0.005

Locus 1914 :

2 alleles

96.9% missing data

3	(0.327) 0.315 0.338

2	(0.673) 0.685 0.662

Locus 1915 :

2 alleles

99.2% missing data

1	(0.594) 0.590 0.608

2	(0.406) 0.410 0.392

Locus 1916 :

2 alleles

99.2% missing data

4	(0.385) 0.389 0.387

2	(0.615) 0.611 0.613

Locus 1917 :

2 alleles

97.3% missing data

2	(0.569) 0.557 0.593

3	(0.431) 0.443 0.407

Locus 1918 :

2 alleles

0.4% missing data

4	(0.985) 0.984 0.995

1	(0.015) 0.016 0.005

Locus 1919 :

2 alleles

99.2% missing data

3	(0.712) 0.714 0.708

4	(0.288) 0.286 0.292

Locus 1920 :

2 alleles

99.2% missing data

3	(0.532) 0.536 0.528

4	(0.468) 0.464 0.472

Locus 1921 :

2 alleles

99.2% missing data

2	(0.607) 0.606 0.598

4	(0.393) 0.394 0.402

Locus 1922 :

2 alleles

99.2% missing data

3	(0.493) 0.496 0.488

4	(0.507) 0.504 0.512

Locus 1923 :

2 alleles

99.2% missing data

3	(0.645) 0.650 0.640

1	(0.355) 0.350 0.360

Locus 1924 :

2 alleles

99.2% missing data

4	(0.552) 0.547 0.553

3	(0.448) 0.453 0.447

Locus 1925 :

2 alleles

99.2% missing data

1	(0.563) 0.569 0.566

3	(0.437) 0.431 0.434

Locus 1926 :

3 alleles

98.8% missing data

4	(0.337) 0.337 0.336

2	(0.230) 0.231 0.231

3	(0.433) 0.432 0.433

Locus 1927 :

2 alleles

99.2% missing data

2	(0.634) 0.629 0.634

3	(0.366) 0.371 0.366

Locus 1928 :

2 alleles

99.2% missing data

1	(0.295) 0.297 0.300

4	(0.705) 0.703 0.700

Locus 1929 :

2 alleles

99.2% missing data

1	(0.709) 0.714 0.701

4	(0.291) 0.286 0.299

Locus 1930 :

3 alleles

98.8% missing data

1	(0.384) 0.390 0.387

4	(0.322) 0.318 0.316

3	(0.294) 0.292 0.297

Locus 1931 :

2 alleles

99.2% missing data

2	(0.256) 0.256 0.263

1	(0.744) 0.744 0.737

Locus 1932 :

3 alleles

98.8% missing data

1	(0.143) 0.140 0.148

2	(0.286) 0.286 0.287

4	(0.571) 0.575 0.565

Locus 1933 :

2 alleles

98.8% missing data

4	(0.534) 0.530 0.538

1	(0.466) 0.470 0.462

Locus 1934 :

3 alleles

93.8% missing data

1	(0.737) 0.732 0.765

2	(0.137) 0.134 0.127

3	(0.126) 0.134 0.108

Locus 1935 :

2 alleles

96.5% missing data

1	(0.794) 0.807 0.790

3	(0.206) 0.193 0.210

Locus 1936 :

2 alleles

96.9% missing data

4	(0.814) 0.814 0.816

1	(0.186) 0.186 0.184

Locus 1937 :

2 alleles

92.6% missing data

3	(0.890) 0.889 0.898

1	(0.110) 0.111 0.102

Locus 1938 :

2 alleles

99.2% missing data

3	(0.378) 0.382 0.376

2	(0.622) 0.618 0.624

Locus 1939 :

3 alleles

98.8% missing data

2	(0.327) 0.326 0.330

4	(0.351) 0.338 0.350

3	(0.322) 0.335 0.319

Locus 1940 :

2 alleles

95.7% missing data

4	(0.098) 0.093 0.100

1	(0.902) 0.907 0.900

Locus 1941 :

2 alleles

99.2% missing data

1	(0.472) 0.466 0.479

2	(0.528) 0.534 0.521

Locus 1942 :

2 alleles

99.2% missing data

1	(0.337) 0.335 0.344

2	(0.663) 0.665 0.656

Locus 1943 :

3 alleles

98.4% missing data

4	(0.381) 0.383 0.374

1	(0.378) 0.371 0.387

3	(0.242) 0.246 0.239

Locus 1944 :

2 alleles

91.4% missing data

4	(0.899) 0.909 0.896

1	(0.101) 0.091 0.104

Locus 1945 :

2 alleles

99.2% missing data

1	(0.753) 0.753 0.751

4	(0.247) 0.247 0.249

Locus 1946 :

2 alleles

7.0% missing data

4	(0.982) 0.975 0.994

1	(0.018) 0.025 0.006

Locus 1947 :

2 alleles

99.2% missing data

3	(0.349) 0.343 0.348

4	(0.651) 0.657 0.652

Locus 1948 :

2 alleles

95.7% missing data

3	(0.776) 0.795 0.771

4	(0.224) 0.205 0.229

Locus 1949 :

2 alleles

99.2% missing data

2	(0.455) 0.451 0.453

3	(0.545) 0.549 0.547

Locus 1950 :

2 alleles

99.2% missing data

4	(0.629) 0.637 0.625

3	(0.371) 0.363 0.375

Locus 1951 :

2 alleles

99.2% missing data

1	(0.669) 0.666 0.675

2	(0.331) 0.334 0.325

Locus 1952 :

2 alleles

98.8% missing data

1	(0.421) 0.415 0.430

3	(0.579) 0.585 0.570

Locus 1953 :

2 alleles

98.4% missing data

1	(0.604) 0.603 0.611

2	(0.396) 0.397 0.389

Locus 1954 :

2 alleles

99.2% missing data

3	(0.464) 0.468 0.466

1	(0.536) 0.532 0.534

Locus 1955 :

2 alleles

99.2% missing data

1	(0.534) 0.528 0.534

3	(0.466) 0.472 0.466

Locus 1956 :

4 alleles

98.4% missing data

3	(0.181) 0.181 0.186

1	(0.261) 0.257 0.260

4	(0.222) 0.218 0.226

2	(0.337) 0.344 0.328

Locus 1957 :

2 alleles

99.2% missing data

2	(0.320) 0.317 0.325

3	(0.680) 0.683 0.675

Locus 1958 :

2 alleles

98.8% missing data

3	(0.455) 0.464 0.446

2	(0.545) 0.536 0.554

Locus 1959 :

2 alleles

99.2% missing data

1	(0.384) 0.387 0.385

4	(0.616) 0.613 0.615

Locus 1960 :

2 alleles

99.2% missing data

1	(0.443) 0.445 0.445

4	(0.557) 0.555 0.555

Locus 1961 :

2 alleles

99.2% missing data

2	(0.299) 0.293 0.301

1	(0.701) 0.707 0.699

Locus 1962 :

3 alleles

98.8% missing data

4	(0.439) 0.434 0.445

2	(0.238) 0.237 0.237

3	(0.323) 0.329 0.318

Locus 1963 :

2 alleles

99.2% missing data

3	(0.371) 0.367 0.377

4	(0.629) 0.633 0.623

Locus 1964 :

2 alleles

97.3% missing data

4	(0.152) 0.143 0.160

3	(0.848) 0.857 0.840

Locus 1965 :

2 alleles

97.7% missing data

3	(0.590) 0.584 0.611

1	(0.410) 0.416 0.389

Locus 1966 :

2 alleles

99.2% missing data

4	(0.318) 0.314 0.324

3	(0.682) 0.686 0.676

Locus 1967 :

2 alleles

99.2% missing data

3	(0.223) 0.220 0.229

4	(0.777) 0.780 0.771

Locus 1968 :

2 alleles

99.2% missing data

4	(0.895) 0.900 0.891

1	(0.105) 0.100 0.109

Locus 1969 :

3 alleles

96.5% missing data

1	(0.346) 0.338 0.349

3	(0.403) 0.413 0.397

4	(0.250) 0.250 0.254

Locus 1970 :

2 alleles

98.8% missing data

1	(0.554) 0.541 0.562

4	(0.446) 0.459 0.438

Locus 1971 :

2 alleles

99.2% missing data

4	(0.432) 0.429 0.435

3	(0.568) 0.571 0.565

Locus 1972 :

2 alleles

99.2% missing data

2	(0.517) 0.511 0.525

1	(0.483) 0.489 0.475

Locus 1973 :

2 alleles

0.0% missing data

1	(0.987) 0.991 0.994

4	(0.013) 0.009 0.006

Locus 1974 :

2 alleles

98.8% missing data

1	(0.388) 0.388 0.391

3	(0.612) 0.612 0.609

Locus 1975 :

3 alleles

98.8% missing data

1	(0.424) 0.416 0.430

3	(0.269) 0.269 0.266

4	(0.307) 0.315 0.304

Locus 1976 :

2 alleles

98.8% missing data

3	(0.323) 0.315 0.328

1	(0.677) 0.685 0.672

Locus 1977 :

3 alleles

98.4% missing data

3	(0.329) 0.324 0.323

2	(0.339) 0.337 0.346

1	(0.332) 0.339 0.331

Locus 1978 :

2 alleles

99.2% missing data

4	(0.500) 0.504 0.500

1	(0.500) 0.496 0.500

Locus 1979 :

2 alleles

99.2% missing data

1	(0.597) 0.600 0.598

2	(0.403) 0.400 0.402

Locus 1980 :

2 alleles

8.6% missing data

1	(0.983) 0.970 0.994

3	(0.017) 0.030 0.006

Locus 1981 :

3 alleles

98.4% missing data

1	(0.298) 0.298 0.300

2	(0.298) 0.299 0.306

3	(0.403) 0.403 0.394

Locus 1982 :

3 alleles

96.5% missing data

1	(0.107) 0.120 0.100

3	(0.816) 0.805 0.819

2	(0.077) 0.075 0.082

Locus 1983 :

2 alleles

99.2% missing data

4	(0.527) 0.526 0.531

1	(0.473) 0.474 0.469

Locus 1984 :

2 alleles

98.4% missing data

2	(0.561) 0.547 0.564

1	(0.439) 0.453 0.436

Locus 1985 :

2 alleles

98.4% missing data

3	(0.808) 0.809 0.809

1	(0.192) 0.191 0.191

Locus 1986 :

2 alleles

99.2% missing data

3	(0.368) 0.365 0.368

1	(0.632) 0.635 0.632

Locus 1987 :

2 alleles

98.8% missing data

3	(0.237) 0.236 0.234

1	(0.763) 0.764 0.766

Locus 1988 :

2 alleles

99.2% missing data

3	(0.407) 0.401 0.409

1	(0.593) 0.599 0.591

Locus 1989 :

2 alleles

99.2% missing data

1	(0.586) 0.578 0.593

2	(0.414) 0.422 0.407

Locus 1990 :

2 alleles

98.4% missing data

2	(0.434) 0.424 0.444

1	(0.566) 0.576 0.556

Locus 1991 :

2 alleles

93.0% missing data

1	(0.919) 0.911 0.931

3	(0.081) 0.089 0.069

Locus 1992 :

2 alleles

99.2% missing data

3	(0.684) 0.685 0.683

2	(0.316) 0.315 0.317

Locus 1993 :

2 alleles

99.2% missing data

1	(0.586) 0.590 0.578

3	(0.414) 0.410 0.422

Locus 1994 :

3 alleles

98.8% missing data

3	(0.309) 0.306 0.307

4	(0.228) 0.230 0.228

2	(0.463) 0.464 0.465

Locus 1995 :

2 alleles

99.2% missing data

1	(0.724) 0.721 0.719

4	(0.276) 0.279 0.281

Locus 1996 :

2 alleles

99.2% missing data

4	(0.382) 0.376 0.379

2	(0.618) 0.624 0.621

Locus 1997 :

2 alleles

99.2% missing data

1	(0.482) 0.492 0.475

3	(0.518) 0.508 0.525

Locus 1998 :

2 alleles

99.2% missing data

3	(0.217) 0.216 0.220

2	(0.783) 0.784 0.780

Locus 1999 :

2 alleles

99.2% missing data

3	(0.594) 0.599 0.591

4	(0.406) 0.401 0.409

Locus 2000 :

2 alleles

98.8% missing data

2	(0.540) 0.537 0.549

3	(0.460) 0.463 0.451

Locus 2001 :

3 alleles

98.4% missing data

1	(0.387) 0.387 0.383

4	(0.423) 0.426 0.425

3	(0.189) 0.186 0.192

Locus 2002 :

2 alleles

8.6% missing data

1	(0.987) 0.980 0.996

4	(0.013) 0.020 0.004

Locus 2003 :

4 alleles

98.0% missing data

1	(0.253) 0.250 0.261

4	(0.196) 0.192 0.197

3	(0.163) 0.173 0.156

2	(0.387) 0.385 0.385

Locus 2004 :

2 alleles

99.2% missing data

2	(0.456) 0.453 0.457

1	(0.544) 0.547 0.543

Locus 2005 :

2 alleles

99.2% missing data

3	(0.515) 0.517 0.515

2	(0.485) 0.483 0.485

Locus 2006 :

2 alleles

91.4% missing data

4	(0.902) 0.920 0.894

3	(0.098) 0.080 0.106

Locus 2007 :

2 alleles

98.8% missing data

3	(0.315) 0.312 0.334

1	(0.685) 0.688 0.666

Locus 2008 :

2 alleles

95.7% missing data

3	(0.717) 0.717 0.729

4	(0.283) 0.283 0.271

Locus 2009 :

2 alleles

98.8% missing data

3	(0.402) 0.396 0.411

4	(0.598) 0.604 0.589

Locus 2010 :

2 alleles

99.2% missing data

2	(0.668) 0.673 0.668

3	(0.332) 0.327 0.332

Locus 2011 :

2 alleles

98.8% missing data

4	(0.678) 0.680 0.682

3	(0.322) 0.320 0.318

Locus 2012 :

2 alleles

99.2% missing data

1	(0.319) 0.319 0.321

3	(0.681) 0.681 0.679

Locus 2013 :

2 alleles

96.5% missing data

3	(0.862) 0.861 0.864

1	(0.138) 0.139 0.136

Locus 2014 :

3 alleles

96.1% missing data

1	(0.741) 0.748 0.747

3	(0.137) 0.135 0.133

4	(0.122) 0.118 0.119

Locus 2015 :

2 alleles

98.8% missing data

1	(0.461) 0.449 0.472

4	(0.539) 0.551 0.528

Locus 2016 :

2 alleles

98.8% missing data

3	(0.542) 0.548 0.540

1	(0.458) 0.452 0.460

Locus 2017 :

3 alleles

98.8% missing data

1	(0.313) 0.310 0.319

2	(0.451) 0.448 0.449

3	(0.236) 0.243 0.232

Locus 2018 :

2 alleles

99.2% missing data

2	(0.152) 0.155 0.150

4	(0.848) 0.845 0.850

Locus 2019 :

2 alleles

98.4% missing data

1	(0.446) 0.453 0.456

3	(0.554) 0.547 0.544

Locus 2020 :

2 alleles

99.2% missing data

1	(0.350) 0.355 0.349

2	(0.650) 0.645 0.651

Locus 2021 :

2 alleles

1.6% missing data

2	(0.987) 0.983 0.996

4	(0.013) 0.017 0.004

Locus 2022 :

2 alleles

98.8% missing data

3	(0.162) 0.160 0.168

1	(0.838) 0.840 0.832

Locus 2023 :

2 alleles

92.6% missing data

1	(0.912) 0.912 0.917

4	(0.088) 0.088 0.083

Locus 2024 :

2 alleles

99.2% missing data

4	(0.483) 0.482 0.482

1	(0.517) 0.518 0.518

Locus 2025 :

2 alleles

99.2% missing data

4	(0.821) 0.821 0.825

3	(0.179) 0.179 0.175

Locus 2026 :

2 alleles

98.8% missing data

4	(0.698) 0.699 0.696

1	(0.302) 0.301 0.304

Locus 2027 :

2 alleles

99.2% missing data

4	(0.576) 0.581 0.572

3	(0.424) 0.419 0.428

Locus 2028 :

2 alleles

99.2% missing data

2	(0.581) 0.586 0.578

1	(0.419) 0.414 0.422

Locus 2029 :

2 alleles

99.2% missing data

2	(0.288) 0.290 0.289

1	(0.712) 0.710 0.711

Locus 2030 :

2 alleles

99.2% missing data

4	(0.528) 0.522 0.530

2	(0.472) 0.478 0.470

Locus 2031 :

2 alleles

98.8% missing data

4	(0.317) 0.308 0.329

1	(0.683) 0.692 0.671

Locus 2032 :

2 alleles

97.3% missing data

1	(0.216) 0.228 0.211

2	(0.784) 0.772 0.789

Locus 2033 :

3 alleles

92.2% missing data

4	(0.764) 0.769 0.780

1	(0.117) 0.116 0.109

2	(0.119) 0.115 0.111

Locus 2034 :

2 alleles

99.2% missing data

1	(0.477) 0.476 0.484

2	(0.523) 0.524 0.516

Locus 2035 :

2 alleles

99.2% missing data

4	(0.663) 0.664 0.657

2	(0.337) 0.336 0.343

Locus 2036 :

2 alleles

98.8% missing data

4	(0.719) 0.716 0.721

1	(0.281) 0.284 0.279

Locus 2037 :

2 alleles

99.2% missing data

2	(0.282) 0.280 0.281

1	(0.718) 0.720 0.719

Locus 2038 :

4 alleles

91.8% missing data

2	(0.616) 0.604 0.662

3	(0.080) 0.078 0.077

1	(0.095) 0.094 0.087

4	(0.209) 0.224 0.174

Locus 2039 :

2 alleles

99.2% missing data

3	(0.597) 0.594 0.602

1	(0.403) 0.406 0.398

Locus 2040 :

2 alleles

98.8% missing data

4	(0.573) 0.576 0.574

2	(0.427) 0.424 0.426

Locus 2041 :

2 alleles

99.2% missing data

1	(0.535) 0.538 0.531

4	(0.465) 0.462 0.469

Locus 2042 :

2 alleles

99.2% missing data

2	(0.468) 0.455 0.478

4	(0.532) 0.545 0.522

Locus 2043 :

2 alleles

99.2% missing data

2	(0.343) 0.345 0.337

1	(0.657) 0.655 0.663

Locus 2044 :

2 alleles

98.8% missing data

2	(0.508) 0.508 0.512

3	(0.492) 0.492 0.488

Locus 2045 :

2 alleles

99.2% missing data

2	(0.421) 0.418 0.425

1	(0.579) 0.582 0.575

Locus 2046 :

2 alleles

96.9% missing data

2	(0.800) 0.791 0.815

3	(0.200) 0.209 0.185

Locus 2047 :

2 alleles

99.2% missing data

2	(0.357) 0.360 0.353

4	(0.643) 0.640 0.647

Locus 2048 :

2 alleles

95.7% missing data

4	(0.151) 0.145 0.151

2	(0.849) 0.855 0.849

Locus 2049 :

2 alleles

96.9% missing data

1	(0.778) 0.792 0.772

4	(0.222) 0.208 0.228

Locus 2050 :

2 alleles

98.8% missing data

1	(0.505) 0.501 0.499

2	(0.495) 0.499 0.501

Locus 2051 :

2 alleles

92.6% missing data

2	(0.849) 0.842 0.864

4	(0.151) 0.158 0.136

Locus 2052 :

3 alleles

98.4% missing data

1	(0.203) 0.203 0.212

2	(0.556) 0.554 0.558

4	(0.241) 0.244 0.230

Locus 2053 :

2 alleles

99.2% missing data

1	(0.459) 0.446 0.465

4	(0.541) 0.554 0.535

Locus 2054 :

2 alleles

98.8% missing data

1	(0.649) 0.644 0.659

4	(0.351) 0.356 0.341

Locus 2055 :

2 alleles

99.2% missing data

4	(0.682) 0.681 0.680

2	(0.318) 0.319 0.320

Locus 2056 :

2 alleles

99.2% missing data

3	(0.356) 0.358 0.352

1	(0.644) 0.642 0.648

Locus 2057 :

2 alleles

99.2% missing data

4	(0.546) 0.538 0.546

1	(0.454) 0.462 0.454

Locus 2058 :

2 alleles

97.7% missing data

2	(0.138) 0.135 0.137

4	(0.862) 0.865 0.863

Locus 2059 :

2 alleles

99.2% missing data

1	(0.555) 0.554 0.554

3	(0.445) 0.446 0.446

Locus 2060 :

2 alleles

99.2% missing data

4	(0.242) 0.237 0.246

2	(0.758) 0.763 0.754

Locus 2061 :

2 alleles

99.2% missing data

2	(0.462) 0.463 0.464

3	(0.538) 0.537 0.536

Locus 2062 :

2 alleles

99.2% missing data

4	(0.593) 0.595 0.590

3	(0.407) 0.405 0.410

Locus 2063 :

2 alleles

99.2% missing data

1	(0.256) 0.252 0.265

3	(0.744) 0.748 0.735

Locus 2064 :

2 alleles

99.2% missing data

3	(0.485) 0.490 0.478

1	(0.515) 0.510 0.522

Locus 2065 :

2 alleles

99.2% missing data

1	(0.227) 0.230 0.234

4	(0.773) 0.770 0.766

Locus 2066 :

2 alleles

98.4% missing data

1	(0.778) 0.780 0.776

4	(0.222) 0.220 0.224

Locus 2067 :

2 alleles

99.2% missing data

3	(0.369) 0.369 0.368

2	(0.631) 0.631 0.632

Locus 2068 :

2 alleles

98.4% missing data

4	(0.378) 0.382 0.384

2	(0.622) 0.618 0.616

Locus 2069 :

2 alleles

99.2% missing data

4	(0.790) 0.788 0.784

1	(0.210) 0.212 0.216

Locus 2070 :

2 alleles

98.4% missing data

2	(0.257) 0.252 0.258

1	(0.743) 0.748 0.742

Locus 2071 :

2 alleles

99.2% missing data

1	(0.496) 0.494 0.500

2	(0.504) 0.506 0.500

Locus 2072 :

2 alleles

98.8% missing data

1	(0.290) 0.281 0.298

2	(0.710) 0.719 0.702

Locus 2073 :

2 alleles

99.2% missing data

1	(0.431) 0.428 0.426

3	(0.569) 0.572 0.574

Locus 2074 :

2 alleles

99.2% missing data

4	(0.478) 0.473 0.481

1	(0.522) 0.527 0.519

Locus 2075 :

2 alleles

99.2% missing data

1	(0.501) 0.499 0.506

2	(0.499) 0.501 0.494

Locus 2076 :

2 alleles

99.2% missing data

2	(0.539) 0.549 0.536

4	(0.461) 0.451 0.464

Locus 2077 :

2 alleles

85.2% missing data

1	(0.557) 0.628 0.483

4	(0.443) 0.372 0.517

Locus 2078 :

3 alleles

98.8% missing data

2	(0.456) 0.454 0.458

1	(0.288) 0.284 0.293

3	(0.256) 0.262 0.249

Locus 2079 :

2 alleles

97.7% missing data

3	(0.148) 0.151 0.140

4	(0.852) 0.849 0.860

Locus 2080 :

2 alleles

99.2% missing data

2	(0.717) 0.720 0.707

4	(0.283) 0.280 0.293

Locus 2081 :

3 alleles

98.8% missing data

1	(0.499) 0.502 0.499

2	(0.252) 0.256 0.245

4	(0.249) 0.241 0.256

Locus 2082 :

2 alleles

99.2% missing data

1	(0.185) 0.189 0.188

2	(0.815) 0.811 0.812

Locus 2083 :

2 alleles

99.2% missing data

4	(0.598) 0.600 0.591

1	(0.402) 0.400 0.409

Locus 2084 :

2 alleles

99.2% missing data

4	(0.384) 0.390 0.389

1	(0.616) 0.610 0.611

Locus 2085 :

2 alleles

99.2% missing data

4	(0.264) 0.261 0.271

3	(0.736) 0.739 0.729

Locus 2086 :

2 alleles

99.2% missing data

4	(0.450) 0.456 0.451

2	(0.550) 0.544 0.549

Locus 2087 :

2 alleles

98.8% missing data

4	(0.623) 0.625 0.614

1	(0.377) 0.375 0.386

Locus 2088 :

2 alleles

99.2% missing data

4	(0.440) 0.442 0.443

1	(0.560) 0.558 0.557

Locus 2089 :

2 alleles

99.2% missing data

3	(0.508) 0.501 0.510

2	(0.492) 0.499 0.490

Locus 2090 :

3 alleles

98.8% missing data

2	(0.332) 0.334 0.337

4	(0.427) 0.428 0.422

1	(0.241) 0.238 0.241

Locus 2091 :

2 alleles

99.2% missing data

2	(0.623) 0.618 0.622

3	(0.377) 0.382 0.378

Locus 2092 :

2 alleles

99.2% missing data

4	(0.660) 0.665 0.654

3	(0.340) 0.335 0.346

Locus 2093 :

2 alleles

99.2% missing data

4	(0.775) 0.768 0.772

2	(0.225) 0.232 0.228

Locus 2094 :

2 alleles

91.0% missing data

3	(0.885) 0.906 0.878

4	(0.115) 0.094 0.122

Locus 2095 :

2 alleles

98.8% missing data

1	(0.282) 0.292 0.281

2	(0.718) 0.708 0.719

Locus 2096 :

2 alleles

99.2% missing data

4	(0.712) 0.715 0.708

3	(0.288) 0.285 0.292

Locus 2097 :

2 alleles

99.2% missing data

1	(0.577) 0.580 0.565

3	(0.423) 0.420 0.435

Locus 2098 :

3 alleles

86.3% missing data

1	(0.778) 0.814 0.739

4	(0.166) 0.132 0.212

3	(0.056) 0.054 0.049

Locus 2099 :

2 alleles

99.2% missing data

3	(0.640) 0.641 0.631

2	(0.360) 0.359 0.369

Locus 2100 :

2 alleles

99.2% missing data

3	(0.344) 0.340 0.350

1	(0.656) 0.660 0.650

Locus 2101 :

2 alleles

98.4% missing data

3	(0.479) 0.476 0.489

1	(0.521) 0.524 0.511

Locus 2102 :

2 alleles

99.2% missing data

4	(0.397) 0.397 0.393

2	(0.603) 0.603 0.607

Locus 2103 :

2 alleles

99.2% missing data

4	(0.524) 0.529 0.522

2	(0.476) 0.471 0.478

Locus 2104 :

2 alleles

94.9% missing data

3	(0.866) 0.866 0.870

2	(0.134) 0.134 0.130

Locus 2105 :

2 alleles

99.2% missing data

2	(0.357) 0.362 0.355

1	(0.643) 0.638 0.645

Locus 2106 :

2 alleles

98.8% missing data

3	(0.673) 0.675 0.677

1	(0.327) 0.325 0.323

Locus 2107 :

2 alleles

99.2% missing data

1	(0.660) 0.664 0.658

2	(0.340) 0.336 0.342

Locus 2108 :

2 alleles

99.2% missing data

4	(0.363) 0.362 0.365

1	(0.637) 0.638 0.635

Locus 2109 :

2 alleles

99.2% missing data

4	(0.658) 0.659 0.648

2	(0.342) 0.341 0.352

Locus 2110 :

2 alleles

98.8% missing data

4	(0.678) 0.684 0.678

3	(0.322) 0.316 0.322

Locus 2111 :

3 alleles

98.8% missing data

3	(0.320) 0.324 0.318

1	(0.412) 0.407 0.414

2	(0.268) 0.269 0.268

Locus 2112 :

2 alleles

98.0% missing data

1	(0.663) 0.668 0.664

3	(0.337) 0.332 0.336

Locus 2113 :

2 alleles

99.2% missing data

2	(0.189) 0.191 0.190

3	(0.811) 0.809 0.810

Locus 2114 :

2 alleles

16.4% missing data

2	(0.980) 0.972 0.994

1	(0.020) 0.028 0.006

Locus 2115 :

2 alleles

92.6% missing data

1	(0.940) 0.936 0.950

4	(0.060) 0.064 0.050

Locus 2116 :

2 alleles

99.2% missing data

1	(0.310) 0.308 0.309

3	(0.690) 0.692 0.691

Locus 2117 :

2 alleles

98.8% missing data

1	(0.340) 0.335 0.346

4	(0.660) 0.665 0.654

Locus 2118 :

2 alleles

99.2% missing data

1	(0.780) 0.776 0.787

2	(0.220) 0.224 0.213

Locus 2119 :

2 alleles

91.0% missing data

4	(0.897) 0.894 0.909

2	(0.103) 0.106 0.091

Locus 2120 :

2 alleles

8.6% missing data

1	(0.987) 0.978 0.996

2	(0.013) 0.022 0.004

Locus 2121 :

2 alleles

99.2% missing data

2	(0.433) 0.435 0.427

3	(0.567) 0.565 0.573

Locus 2122 :

2 alleles

98.0% missing data

1	(0.752) 0.758 0.755

3	(0.248) 0.242 0.245

Locus 2123 :

2 alleles

98.8% missing data

2	(0.648) 0.652 0.643

4	(0.352) 0.348 0.357

Locus 2124 :

2 alleles

99.2% missing data

3	(0.210) 0.205 0.220

1	(0.790) 0.795 0.780

Locus 2125 :

2 alleles

93.4% missing data

4	(0.885) 0.877 0.901

3	(0.115) 0.123 0.099

Locus 2126 :

2 alleles

99.2% missing data

2	(0.650) 0.652 0.651

1	(0.350) 0.348 0.349

Locus 2127 :

2 alleles

99.2% missing data

4	(0.625) 0.631 0.623

2	(0.375) 0.369 0.377

Locus 2128 :

2 alleles

98.4% missing data

2	(0.461) 0.453 0.462

4	(0.539) 0.547 0.538

Locus 2129 :

2 alleles

91.0% missing data

4	(0.898) 0.917 0.882

2	(0.102) 0.083 0.118

Locus 2130 :

2 alleles

99.2% missing data

1	(0.526) 0.527 0.530

3	(0.474) 0.473 0.470

Locus 2131 :

2 alleles

98.8% missing data

3	(0.262) 0.262 0.263

4	(0.738) 0.738 0.737

Locus 2132 :

2 alleles

99.2% missing data

1	(0.603) 0.607 0.603

3	(0.397) 0.393 0.397

Locus 2133 :

2 alleles

99.2% missing data

4	(0.690) 0.685 0.692

1	(0.310) 0.315 0.308

Locus 2134 :

2 alleles

99.2% missing data

3	(0.558) 0.548 0.561

4	(0.442) 0.452 0.439

Locus 2135 :

2 alleles

96.9% missing data

1	(0.757) 0.769 0.751

3	(0.243) 0.231 0.249

Locus 2136 :

2 alleles

99.2% missing data

1	(0.707) 0.703 0.707

4	(0.293) 0.297 0.293

Locus 2137 :

2 alleles

98.8% missing data

3	(0.625) 0.620 0.635

1	(0.375) 0.380 0.365

Locus 2138 :

2 alleles

98.4% missing data

1	(0.574) 0.576 0.569

4	(0.426) 0.424 0.431

Locus 2139 :

2 alleles

91.0% missing data

2	(0.901) 0.917 0.889

3	(0.099) 0.083 0.111

Locus 2140 :

2 alleles

99.2% missing data

2	(0.654) 0.648 0.661

1	(0.346) 0.352 0.339

Locus 2141 :

2 alleles

92.6% missing data

3	(0.876) 0.869 0.895

2	(0.124) 0.131 0.105

Locus 2142 :

2 alleles

94.5% missing data

4	(0.848) 0.840 0.865

1	(0.152) 0.160 0.135

Locus 2143 :

2 alleles

99.2% missing data

1	(0.481) 0.478 0.480

4	(0.519) 0.522 0.520

Locus 2144 :

2 alleles

96.9% missing data

2	(0.746) 0.735 0.760

1	(0.254) 0.265 0.240

Locus 2145 :

2 alleles

99.2% missing data

3	(0.333) 0.332 0.337

2	(0.667) 0.668 0.663

Locus 2146 :

2 alleles

98.8% missing data

3	(0.619) 0.616 0.622

1	(0.381) 0.384 0.378

Locus 2147 :

2 alleles

91.0% missing data

1	(0.880) 0.895 0.882

2	(0.120) 0.105 0.118

Locus 2148 :

2 alleles

99.2% missing data

1	(0.488) 0.493 0.487

3	(0.512) 0.507 0.513

Locus 2149 :

2 alleles

99.2% missing data

2	(0.348) 0.353 0.347

1	(0.652) 0.647 0.653

Locus 2150 :

2 alleles

95.3% missing data

2	(0.348) 0.339 0.354

4	(0.652) 0.661 0.646

Locus 2151 :

2 alleles

99.2% missing data

2	(0.594) 0.595 0.591

4	(0.406) 0.405 0.409

Locus 2152 :

3 alleles

98.8% missing data

3	(0.316) 0.316 0.316

1	(0.454) 0.452 0.453

2	(0.230) 0.232 0.231

Locus 2153 :

2 alleles

99.2% missing data

1	(0.246) 0.246 0.253

4	(0.754) 0.754 0.747

Locus 2154 :

2 alleles

99.2% missing data

4	(0.605) 0.603 0.606

2	(0.395) 0.397 0.394

Locus 2155 :

2 alleles

98.0% missing data

2	(0.857) 0.857 0.867

1	(0.143) 0.143 0.133

Locus 2156 :

2 alleles

99.2% missing data

3	(0.402) 0.407 0.404

1	(0.598) 0.593 0.596

Locus 2157 :

2 alleles

95.3% missing data

1	(0.856) 0.854 0.862

2	(0.144) 0.146 0.138

Locus 2158 :

2 alleles

99.2% missing data

3	(0.447) 0.445 0.444

1	(0.553) 0.555 0.556

Locus 2159 :

2 alleles

97.7% missing data

3	(0.690) 0.695 0.693

4	(0.310) 0.305 0.307

Locus 2160 :

2 alleles

99.2% missing data

3	(0.541) 0.541 0.538

4	(0.459) 0.459 0.462

Locus 2161 :

2 alleles

93.0% missing data

3	(0.926) 0.929 0.931

1	(0.074) 0.071 0.069

Locus 2162 :

2 alleles

99.2% missing data

1	(0.406) 0.404 0.409

4	(0.594) 0.596 0.591

Locus 2163 :

3 alleles

98.8% missing data

1	(0.365) 0.353 0.368

3	(0.209) 0.214 0.210

4	(0.426) 0.433 0.422

Locus 2164 :

2 alleles

98.8% missing data

3	(0.709) 0.712 0.706

2	(0.291) 0.288 0.294

Locus 2165 :

2 alleles

99.2% missing data

2	(0.410) 0.410 0.409

1	(0.590) 0.590 0.591

Locus 2166 :

2 alleles

96.1% missing data

4	(0.847) 0.859 0.839

3	(0.153) 0.141 0.161

Locus 2167 :

3 alleles

11.7% missing data

4	(0.964) 0.948 0.989

3	(0.021) 0.029 0.006

2	(0.015) 0.023 0.005

Locus 2168 :

2 alleles

99.2% missing data

1	(0.210) 0.210 0.217

4	(0.790) 0.790 0.783

Locus 2169 :

2 alleles

98.4% missing data

1	(0.700) 0.700 0.703

2	(0.300) 0.300 0.297

Locus 2170 :

2 alleles

76.2% missing data

2	(0.956) 0.953 0.969

1	(0.044) 0.047 0.031

Locus 2171 :

2 alleles

99.2% missing data

3	(0.492) 0.492 0.492

2	(0.508) 0.508 0.508

Locus 2172 :

2 alleles

99.2% missing data

2	(0.518) 0.519 0.512

1	(0.482) 0.481 0.488

Locus 2173 :

2 alleles

96.1% missing data

2	(0.176) 0.163 0.178

1	(0.824) 0.837 0.822

Locus 2174 :

2 alleles

93.0% missing data

1	(0.894) 0.896 0.901

2	(0.106) 0.104 0.099

Locus 2175 :

2 alleles

98.8% missing data

2	(0.495) 0.491 0.499

1	(0.505) 0.509 0.501

Locus 2176 :

2 alleles

99.2% missing data

2	(0.422) 0.422 0.430

3	(0.578) 0.578 0.570

Locus 2177 :

2 alleles

96.1% missing data

2	(0.795) 0.799 0.795

4	(0.205) 0.201 0.205

Locus 2178 :

2 alleles

98.8% missing data

4	(0.618) 0.622 0.606

2	(0.382) 0.378 0.394

Locus 2179 :

2 alleles

99.2% missing data

3	(0.391) 0.388 0.389

1	(0.609) 0.612 0.611

Locus 2180 :

2 alleles

99.2% missing data

4	(0.705) 0.709 0.700

3	(0.295) 0.291 0.300

Locus 2181 :

2 alleles

99.2% missing data

2	(0.616) 0.622 0.617

3	(0.384) 0.378 0.383

Locus 2182 :

2 alleles

96.1% missing data

1	(0.905) 0.916 0.900

4	(0.095) 0.084 0.100

Locus 2183 :

3 alleles

98.0% missing data

2	(0.580) 0.581 0.583

1	(0.141) 0.140 0.139

3	(0.278) 0.279 0.278

Locus 2184 :

2 alleles

99.2% missing data

3	(0.428) 0.425 0.431

4	(0.572) 0.575 0.569

Locus 2185 :

3 alleles

97.3% missing data

4	(0.228) 0.234 0.223

2	(0.580) 0.575 0.587

3	(0.192) 0.191 0.190

Locus 2186 :

2 alleles

99.2% missing data

4	(0.565) 0.556 0.568

2	(0.435) 0.444 0.432

Locus 2187 :

2 alleles

99.2% missing data

1	(0.285) 0.282 0.285

2	(0.715) 0.718 0.715

Locus 2188 :

2 alleles

98.0% missing data

1	(0.733) 0.736 0.733

2	(0.267) 0.264 0.267

Locus 2189 :

2 alleles

98.8% missing data

2	(0.503) 0.507 0.507

1	(0.497) 0.493 0.493

Locus 2190 :

2 alleles

99.2% missing data

2	(0.863) 0.858 0.859

1	(0.137) 0.142 0.141

Locus 2191 :

2 alleles

99.2% missing data

1	(0.638) 0.635 0.637

3	(0.362) 0.365 0.363

Locus 2192 :

2 alleles

99.2% missing data

1	(0.311) 0.309 0.312

4	(0.689) 0.691 0.688

Locus 2193 :

2 alleles

96.5% missing data

4	(0.902) 0.903 0.904

1	(0.098) 0.097 0.096

Locus 2194 :

2 alleles

99.2% missing data

3	(0.737) 0.739 0.741

4	(0.263) 0.261 0.259

Locus 2195 :

2 alleles

98.8% missing data

2	(0.560) 0.552 0.565

4	(0.440) 0.448 0.435

Locus 2196 :

2 alleles

96.9% missing data

4	(0.772) 0.777 0.780

1	(0.228) 0.223 0.220

Locus 2197 :

3 alleles

98.8% missing data

1	(0.244) 0.242 0.245

4	(0.327) 0.325 0.324

2	(0.430) 0.434 0.431

Locus 2198 :

2 alleles

99.2% missing data

1	(0.472) 0.473 0.462

4	(0.528) 0.527 0.538

Locus 2199 :

2 alleles

99.2% missing data

4	(0.367) 0.371 0.366

1	(0.633) 0.629 0.634

Locus 2200 :

2 alleles

98.8% missing data

1	(0.391) 0.389 0.394

2	(0.609) 0.611 0.606

Locus 2201 :

2 alleles

98.4% missing data

1	(0.712) 0.712 0.716

3	(0.288) 0.288 0.284

Locus 2202 :

2 alleles

99.2% missing data

4	(0.528) 0.536 0.520

2	(0.472) 0.464 0.480

Locus 2203 :

2 alleles

99.2% missing data

2	(0.497) 0.495 0.500

3	(0.503) 0.505 0.500

Locus 2204 :

2 alleles

99.2% missing data

4	(0.241) 0.243 0.241

2	(0.759) 0.757 0.759

Locus 2205 :

2 alleles

99.2% missing data

1	(0.399) 0.396 0.401

4	(0.601) 0.604 0.599

Locus 2206 :

2 alleles

99.2% missing data

1	(0.369) 0.369 0.369

4	(0.631) 0.631 0.631

Locus 2207 :

2 alleles

96.9% missing data

3	(0.799) 0.791 0.810

1	(0.201) 0.209 0.190

Locus 2208 :

2 alleles

98.0% missing data

1	(0.199) 0.189 0.201

2	(0.801) 0.811 0.799

Locus 2209 :

2 alleles

97.7% missing data

2	(0.743) 0.738 0.754

3	(0.257) 0.262 0.246

Locus 2210 :

2 alleles

96.1% missing data

1	(0.657) 0.651 0.668

2	(0.343) 0.349 0.332

Locus 2211 :

2 alleles

99.2% missing data

2	(0.825) 0.827 0.822

4	(0.175) 0.173 0.178

Locus 2212 :

2 alleles

98.8% missing data

4	(0.773) 0.773 0.772

2	(0.227) 0.227 0.228

Locus 2213 :

2 alleles

99.2% missing data

1	(0.545) 0.542 0.547

4	(0.455) 0.458 0.453

Locus 2214 :

2 alleles

98.4% missing data

4	(0.883) 0.886 0.878

2	(0.117) 0.114 0.122

Locus 2215 :

2 alleles

99.2% missing data

3	(0.651) 0.655 0.648

4	(0.349) 0.345 0.352

Locus 2216 :

2 alleles

98.8% missing data

1	(0.287) 0.280 0.292

4	(0.713) 0.720 0.708

Locus 2217 :

2 alleles

7.8% missing data

2	(0.975) 0.955 0.992

4	(0.025) 0.045 0.008

Locus 2218 :

2 alleles

99.2% missing data

1	(0.365) 0.370 0.360

4	(0.635) 0.630 0.640

Locus 2219 :

2 alleles

97.7% missing data

1	(0.271) 0.271 0.268

2	(0.729) 0.729 0.732

Locus 2220 :

2 alleles

99.2% missing data

3	(0.751) 0.752 0.747

4	(0.249) 0.248 0.253

Locus 2221 :

2 alleles

99.2% missing data

2	(0.468) 0.465 0.470

1	(0.532) 0.535 0.530

Locus 2222 :

2 alleles

99.2% missing data

4	(0.503) 0.499 0.513

1	(0.497) 0.501 0.487

Locus 2223 :

3 alleles

98.8% missing data

4	(0.361) 0.364 0.361

1	(0.461) 0.463 0.452

2	(0.178) 0.174 0.186

Locus 2224 :

2 alleles

99.2% missing data

4	(0.339) 0.343 0.335

2	(0.661) 0.657 0.665

Locus 2225 :

2 alleles

96.1% missing data

2	(0.798) 0.803 0.802

4	(0.202) 0.197 0.198

Locus 2226 :

2 alleles

95.3% missing data

2	(0.630) 0.629 0.633

3	(0.370) 0.371 0.367

Locus 2227 :

2 alleles

0.0% missing data

3	(0.986) 0.982 0.995

4	(0.014) 0.018 0.005

Locus 2228 :

2 alleles

99.2% missing data

2	(0.451) 0.449 0.449

3	(0.549) 0.551 0.551

Locus 2229 :

2 alleles

99.2% missing data

1	(0.733) 0.726 0.733

3	(0.267) 0.274 0.267

Locus 2230 :

2 alleles

98.8% missing data

1	(0.410) 0.414 0.409

2	(0.590) 0.586 0.591

Locus 2231 :

2 alleles

99.2% missing data

2	(0.707) 0.709 0.701

1	(0.293) 0.291 0.299

Locus 2232 :

2 alleles

99.2% missing data

2	(0.629) 0.629 0.627

3	(0.371) 0.371 0.373

Locus 2233 :

2 alleles

99.2% missing data

3	(0.448) 0.441 0.451

2	(0.552) 0.559 0.549

Locus 2234 :

2 alleles

98.8% missing data

2	(0.538) 0.534 0.547

4	(0.462) 0.466 0.453

Locus 2235 :

2 alleles

99.2% missing data

3	(0.643) 0.641 0.647

1	(0.357) 0.359 0.353

Locus 2236 :

2 alleles

94.5% missing data

4	(0.864) 0.868 0.863

2	(0.136) 0.132 0.137

Locus 2237 :

2 alleles

99.2% missing data

2	(0.399) 0.398 0.409

1	(0.601) 0.602 0.591

Locus 2238 :

2 alleles

99.2% missing data

1	(0.802) 0.796 0.807

3	(0.198) 0.204 0.193

Locus 2239 :

2 alleles

99.2% missing data

3	(0.357) 0.359 0.354

4	(0.643) 0.641 0.646

Locus 2240 :

2 alleles

99.2% missing data

1	(0.700) 0.704 0.695

3	(0.300) 0.296 0.305

Locus 2241 :

2 alleles

99.2% missing data

1	(0.593) 0.593 0.592

4	(0.407) 0.407 0.408

Locus 2242 :

2 alleles

98.8% missing data

2	(0.435) 0.432 0.438

1	(0.565) 0.568 0.562

Locus 2243 :

2 alleles

98.8% missing data

2	(0.491) 0.489 0.494

1	(0.509) 0.511 0.506

Locus 2244 :

2 alleles

96.9% missing data

2	(0.607) 0.593 0.627

4	(0.393) 0.407 0.373

Locus 2245 :

2 alleles

0.0% missing data

1	(0.290) 0.239 0.353

4	(0.710) 0.761 0.647

Locus 2246 :

2 alleles

0.0% missing data

4	(0.987) 0.984 0.996

1	(0.013) 0.016 0.004

Locus 2247 :

2 alleles

99.2% missing data

1	(0.525) 0.514 0.525

2	(0.475) 0.486 0.475

Locus 2248 :

2 alleles

99.2% missing data

4	(0.785) 0.789 0.782

1	(0.215) 0.211 0.218

Locus 2249 :

2 alleles

99.2% missing data

3	(0.474) 0.474 0.471

4	(0.526) 0.526 0.529

Locus 2250 :

2 alleles

99.2% missing data

2	(0.501) 0.507 0.502

3	(0.499) 0.493 0.498

Locus 2251 :

2 alleles

99.2% missing data

3	(0.769) 0.765 0.771

2	(0.231) 0.235 0.229

Locus 2252 :

2 alleles

98.8% missing data

2	(0.356) 0.348 0.367

3	(0.644) 0.652 0.633

Locus 2253 :

2 alleles

98.8% missing data

1	(0.455) 0.449 0.455

2	(0.545) 0.551 0.545

Locus 2254 :

2 alleles

99.2% missing data

2	(0.738) 0.734 0.745

4	(0.262) 0.266 0.255

Locus 2255 :

2 alleles

99.2% missing data

1	(0.645) 0.640 0.649

2	(0.355) 0.360 0.351

Locus 2256 :

2 alleles

98.4% missing data

3	(0.542) 0.542 0.542

4	(0.458) 0.458 0.458

Locus 2257 :

2 alleles

99.2% missing data

1	(0.646) 0.647 0.650

4	(0.354) 0.353 0.350

Locus 2258 :

3 alleles

98.8% missing data

4	(0.395) 0.394 0.396

3	(0.391) 0.389 0.391

1	(0.214) 0.218 0.212

Locus 2259 :

2 alleles

92.6% missing data

4	(0.909) 0.910 0.913

2	(0.091) 0.090 0.087

Locus 2260 :

2 alleles

99.2% missing data

3	(0.205) 0.198 0.206

1	(0.795) 0.802 0.794

Locus 2261 :

2 alleles

99.2% missing data

3	(0.372) 0.367 0.377

1	(0.628) 0.633 0.623

Locus 2262 :

2 alleles

97.7% missing data

1	(0.769) 0.762 0.780

3	(0.231) 0.238 0.220

Locus 2263 :

2 alleles

99.2% missing data

4	(0.275) 0.274 0.276

1	(0.725) 0.726 0.724

Locus 2264 :

2 alleles

99.2% missing data

4	(0.468) 0.466 0.466

1	(0.532) 0.534 0.534

Locus 2265 :

2 alleles

96.9% missing data

1	(0.344) 0.349 0.323

4	(0.656) 0.651 0.677

Locus 2266 :

2 alleles

92.6% missing data

2	(0.932) 0.943 0.929

1	(0.068) 0.057 0.071

Locus 2267 :

3 alleles

98.8% missing data

4	(0.265) 0.269 0.266

3	(0.437) 0.431 0.433

1	(0.297) 0.300 0.301

Locus 2268 :

2 alleles

99.2% missing data

4	(0.156) 0.155 0.161

2	(0.844) 0.845 0.839

Locus 2269 :

2 alleles

97.7% missing data

2	(0.792) 0.787 0.802

1	(0.208) 0.213 0.198

Locus 2270 :

3 alleles

95.7% missing data

1	(0.223) 0.213 0.222

4	(0.642) 0.663 0.642

2	(0.135) 0.124 0.136

Locus 2271 :

2 alleles

91.4% missing data

3	(0.898) 0.911 0.893

4	(0.102) 0.089 0.107

Locus 2272 :

2 alleles

99.2% missing data

2	(0.575) 0.577 0.580

1	(0.425) 0.423 0.420

Locus 2273 :

3 alleles

96.1% missing data

4	(0.223) 0.219 0.224

2	(0.626) 0.628 0.628

3	(0.152) 0.152 0.148

Locus 2274 :

2 alleles

99.2% missing data

3	(0.730) 0.732 0.724

1	(0.270) 0.268 0.276

Locus 2275 :

2 alleles

96.1% missing data

3	(0.137) 0.133 0.136

2	(0.863) 0.867 0.864

Locus 2276 :

2 alleles

99.2% missing data

2	(0.517) 0.512 0.517

3	(0.483) 0.488 0.483

Locus 2277 :

2 alleles

99.2% missing data

2	(0.511) 0.509 0.507

3	(0.489) 0.491 0.493

Locus 2278 :

2 alleles

99.2% missing data

3	(0.525) 0.520 0.524

4	(0.475) 0.480 0.476

Locus 2279 :

2 alleles

99.2% missing data

4	(0.441) 0.442 0.442

3	(0.559) 0.558 0.558

Locus 2280 :

2 alleles

1.2% missing data

3	(0.652) 0.547 0.748

2	(0.348) 0.453 0.252

Locus 2281 :

2 alleles

98.4% missing data

2	(0.414) 0.413 0.415

3	(0.586) 0.587 0.585

Locus 2282 :

2 alleles

99.2% missing data

2	(0.461) 0.468 0.462

1	(0.539) 0.532 0.538

Locus 2283 :

2 alleles

99.2% missing data

4	(0.684) 0.680 0.689

1	(0.316) 0.320 0.311

Locus 2284 :

2 alleles

98.8% missing data

3	(0.298) 0.291 0.298

4	(0.702) 0.709 0.702

Locus 2285 :

2 alleles

99.2% missing data

2	(0.684) 0.685 0.682

4	(0.316) 0.315 0.318

Locus 2286 :

2 alleles

8.6% missing data

3	(0.986) 0.978 0.996

1	(0.014) 0.022 0.004

Locus 2287 :

2 alleles

99.2% missing data

2	(0.649) 0.650 0.645

1	(0.351) 0.350 0.355

Locus 2288 :

2 alleles

99.2% missing data

4	(0.474) 0.466 0.477

1	(0.526) 0.534 0.523

Locus 2289 :

3 alleles

93.8% missing data

4	(0.227) 0.253 0.197

1	(0.209) 0.203 0.206

3	(0.564) 0.545 0.596

Locus 2290 :

2 alleles

98.8% missing data

2	(0.700) 0.707 0.694

1	(0.300) 0.293 0.306

Locus 2291 :

2 alleles

99.2% missing data

3	(0.601) 0.609 0.599

1	(0.399) 0.391 0.401

Locus 2292 :

3 alleles

98.8% missing data

4	(0.347) 0.346 0.348

2	(0.309) 0.312 0.310

1	(0.344) 0.341 0.341

Locus 2293 :

2 alleles

95.3% missing data

2	(0.789) 0.780 0.803

4	(0.211) 0.220 0.197

Locus 2294 :

3 alleles

98.8% missing data

2	(0.236) 0.231 0.241

4	(0.328) 0.334 0.321

1	(0.435) 0.435 0.438

Locus 2295 :

2 alleles

99.2% missing data

2	(0.452) 0.442 0.446

3	(0.548) 0.558 0.554

Locus 2296 :

2 alleles

99.2% missing data

4	(0.223) 0.227 0.223

3	(0.777) 0.773 0.777

Locus 2297 :

2 alleles

99.2% missing data

1	(0.422) 0.427 0.417

4	(0.578) 0.573 0.583

Locus 2298 :

2 alleles

90.6% missing data

1	(0.766) 0.770 0.774

4	(0.234) 0.230 0.226

Locus 2299 :

2 alleles

99.2% missing data

2	(0.440) 0.438 0.442

1	(0.560) 0.562 0.558

Locus 2300 :

2 alleles

99.2% missing data

3	(0.541) 0.535 0.542

1	(0.459) 0.465 0.458

Locus 2301 :

2 alleles

8.2% missing data

1	(0.981) 0.972 0.994

2	(0.019) 0.028 0.006

Locus 2302 :

2 alleles

99.2% missing data

4	(0.646) 0.650 0.643

1	(0.354) 0.350 0.357

Locus 2303 :

2 alleles

97.7% missing data

3	(0.453) 0.445 0.460

2	(0.547) 0.555 0.540

Locus 2304 :

2 alleles

99.2% missing data

2	(0.403) 0.398 0.410

3	(0.597) 0.602 0.590

Locus 2305 :

2 alleles

8.2% missing data

1	(0.968) 0.943 0.990

2	(0.032) 0.057 0.010

Locus 2306 :

3 alleles

98.8% missing data

1	(0.308) 0.307 0.311

3	(0.333) 0.330 0.336

4	(0.359) 0.363 0.353

Locus 2307 :

2 alleles

96.9% missing data

4	(0.788) 0.788 0.793

1	(0.212) 0.212 0.207

Locus 2308 :

2 alleles

99.2% missing data

1	(0.428) 0.438 0.422

2	(0.572) 0.562 0.578

Locus 2309 :

2 alleles

96.9% missing data

3	(0.274) 0.276 0.263

4	(0.726) 0.724 0.737

Locus 2310 :

2 alleles

98.8% missing data

1	(0.678) 0.681 0.681

3	(0.322) 0.319 0.319

Locus 2311 :

2 alleles

99.2% missing data

2	(0.684) 0.676 0.691

4	(0.316) 0.324 0.309

Locus 2312 :

2 alleles

98.8% missing data

3	(0.479) 0.480 0.485

2	(0.521) 0.520 0.515

Locus 2313 :

2 alleles

98.8% missing data

4	(0.759) 0.759 0.751

3	(0.241) 0.241 0.249

Locus 2314 :

3 alleles

98.4% missing data

2	(0.464) 0.455 0.472

3	(0.185) 0.194 0.177

4	(0.352) 0.351 0.351

Locus 2315 :

2 alleles

98.8% missing data

3	(0.792) 0.792 0.795

1	(0.208) 0.208 0.205

Locus 2316 :

2 alleles

96.9% missing data

2	(0.819) 0.812 0.829

1	(0.181) 0.188 0.171

Locus 2317 :

2 alleles

91.0% missing data

4	(0.924) 0.924 0.933

1	(0.076) 0.076 0.067

Locus 2318 :

2 alleles

99.2% missing data

2	(0.564) 0.569 0.557

3	(0.436) 0.431 0.443

Locus 2319 :

2 alleles

98.8% missing data

3	(0.774) 0.778 0.775

2	(0.226) 0.222 0.225

Locus 2320 :

2 alleles

99.2% missing data

3	(0.487) 0.490 0.488

4	(0.513) 0.510 0.512

Locus 2321 :

2 alleles

99.2% missing data

3	(0.537) 0.542 0.528

1	(0.463) 0.458 0.472

Locus 2322 :

2 alleles

98.0% missing data

2	(0.851) 0.854 0.844

4	(0.149) 0.146 0.156

Locus 2323 :

2 alleles

99.2% missing data

4	(0.472) 0.467 0.480

1	(0.528) 0.533 0.520

Locus 2324 :

2 alleles

95.7% missing data

4	(0.751) 0.736 0.774

2	(0.249) 0.264 0.226

Locus 2325 :

2 alleles

98.8% missing data

4	(0.656) 0.662 0.651

2	(0.344) 0.338 0.349

Locus 2326 :

2 alleles

98.0% missing data

3	(0.169) 0.163 0.174

1	(0.831) 0.837 0.826

Locus 2327 :

3 alleles

98.4% missing data

2	(0.286) 0.296 0.287

3	(0.151) 0.150 0.156

1	(0.563) 0.553 0.557

Locus 2328 :

2 alleles

98.8% missing data

3	(0.580) 0.585 0.570

1	(0.420) 0.415 0.430

Locus 2329 :

3 alleles

85.9% missing data

1	(0.611) 0.668 0.549

4	(0.314) 0.249 0.386

3	(0.075) 0.083 0.065

Locus 2330 :

2 alleles

99.2% missing data

3	(0.489) 0.490 0.490

4	(0.511) 0.510 0.510

Locus 2331 :

2 alleles

99.2% missing data

1	(0.712) 0.712 0.708

3	(0.288) 0.288 0.292

Locus 2332 :

2 alleles

98.8% missing data

2	(0.559) 0.559 0.559

1	(0.441) 0.441 0.441

Locus 2333 :

2 alleles

99.2% missing data

1	(0.591) 0.588 0.587

4	(0.409) 0.412 0.413

Locus 2334 :

2 alleles

98.4% missing data

2	(0.401) 0.398 0.414

3	(0.599) 0.602 0.586

Locus 2335 :

2 alleles

98.4% missing data

1	(0.694) 0.692 0.702

4	(0.306) 0.308 0.298

Locus 2336 :

2 alleles

98.8% missing data

2	(0.523) 0.519 0.533

3	(0.477) 0.481 0.467

Locus 2337 :

2 alleles

94.9% missing data

1	(0.827) 0.826 0.837

4	(0.173) 0.174 0.163

Locus 2338 :

2 alleles

99.2% missing data

1	(0.708) 0.707 0.710

4	(0.292) 0.293 0.290

Locus 2339 :

2 alleles

99.2% missing data

4	(0.494) 0.497 0.499

1	(0.506) 0.503 0.501

Locus 2340 :

3 alleles

94.9% missing data

2	(0.782) 0.772 0.798

1	(0.073) 0.073 0.075

3	(0.145) 0.155 0.128

Locus 2341 :

2 alleles

98.8% missing data

1	(0.300) 0.297 0.302

4	(0.700) 0.703 0.698

Locus 2342 :

2 alleles

99.2% missing data

4	(0.547) 0.545 0.548

3	(0.453) 0.455 0.452

Locus 2343 :

2 alleles

99.2% missing data

2	(0.349) 0.338 0.350

3	(0.651) 0.662 0.650

Locus 2344 :

2 alleles

92.6% missing data

3	(0.900) 0.890 0.915

1	(0.100) 0.110 0.085

Locus 2345 :

2 alleles

96.5% missing data

2	(0.155) 0.167 0.145

1	(0.845) 0.833 0.855

Locus 2346 :

2 alleles

98.8% missing data

1	(0.684) 0.689 0.689

3	(0.316) 0.311 0.311

Locus 2347 :

2 alleles

98.8% missing data

2	(0.535) 0.543 0.527

3	(0.465) 0.457 0.473

Locus 2348 :

2 alleles

99.2% missing data

1	(0.292) 0.295 0.290

2	(0.708) 0.705 0.710

Locus 2349 :

2 alleles

99.2% missing data

1	(0.256) 0.260 0.256

4	(0.744) 0.740 0.744

Locus 2350 :

2 alleles

99.2% missing data

1	(0.185) 0.184 0.190

2	(0.815) 0.816 0.810

Locus 2351 :

2 alleles

99.2% missing data

1	(0.656) 0.662 0.656

4	(0.344) 0.338 0.344

Locus 2352 :

2 alleles

98.8% missing data

4	(0.477) 0.471 0.475

1	(0.523) 0.529 0.525

Locus 2353 :

2 alleles

99.2% missing data

1	(0.493) 0.487 0.495

3	(0.507) 0.513 0.505

Locus 2354 :

2 alleles

99.2% missing data

4	(0.373) 0.375 0.376

1	(0.627) 0.625 0.624

Locus 2355 :

3 alleles

98.4% missing data

4	(0.345) 0.352 0.336

3	(0.219) 0.208 0.225

2	(0.435) 0.440 0.439

Locus 2356 :

2 alleles

99.2% missing data

1	(0.462) 0.468 0.456

3	(0.538) 0.532 0.544

Locus 2357 :

2 alleles

98.4% missing data

4	(0.773) 0.776 0.774

1	(0.227) 0.224 0.226

Locus 2358 :

2 alleles

99.2% missing data

4	(0.392) 0.386 0.398

1	(0.608) 0.614 0.602

Locus 2359 :

2 alleles

99.2% missing data

1	(0.799) 0.801 0.794

4	(0.201) 0.199 0.206

Locus 2360 :

2 alleles

98.4% missing data

4	(0.301) 0.295 0.300

2	(0.699) 0.705 0.700

Locus 2361 :

2 alleles

98.0% missing data

1	(0.768) 0.766 0.772

4	(0.232) 0.234 0.228

Locus 2362 :

2 alleles

99.2% missing data

1	(0.705) 0.703 0.712

4	(0.295) 0.297 0.288

Locus 2363 :

2 alleles

99.2% missing data

1	(0.782) 0.777 0.785

4	(0.218) 0.223 0.215

Locus 2364 :

2 alleles

89.1% missing data

3	(0.125) 0.100 0.138

2	(0.875) 0.900 0.862

Locus 2365 :

2 alleles

90.6% missing data

3	(0.889) 0.898 0.884

4	(0.111) 0.102 0.116

Locus 2366 :

3 alleles

98.0% missing data

3	(0.352) 0.357 0.354

2	(0.195) 0.187 0.205

1	(0.454) 0.456 0.441

Locus 2367 :

2 alleles

99.2% missing data

1	(0.510) 0.512 0.510

4	(0.490) 0.488 0.490

Locus 2368 :

2 alleles

99.2% missing data

1	(0.744) 0.737 0.745

3	(0.256) 0.263 0.255

Locus 2369 :

2 alleles

98.8% missing data

1	(0.572) 0.574 0.577

2	(0.428) 0.426 0.423

Locus 2370 :

2 alleles

97.7% missing data

2	(0.641) 0.642 0.645

4	(0.359) 0.358 0.355

Locus 2371 :

2 alleles

88.7% missing data

2	(0.708) 0.722 0.694

3	(0.292) 0.278 0.306

Locus 2372 :

2 alleles

98.0% missing data

1	(0.467) 0.481 0.458

4	(0.533) 0.519 0.542

Locus 2373 :

2 alleles

0.8% missing data

4	(0.985) 0.981 0.995

1	(0.015) 0.019 0.005

Locus 2374 :

2 alleles

99.2% missing data

4	(0.432) 0.434 0.432

3	(0.568) 0.566 0.568

Locus 2375 :

2 alleles

99.2% missing data

2	(0.436) 0.442 0.436

4	(0.564) 0.558 0.564

Locus 2376 :

2 alleles

99.2% missing data

3	(0.493) 0.489 0.500

1	(0.507) 0.511 0.500

Locus 2377 :

2 alleles

99.2% missing data

1	(0.514) 0.515 0.510

2	(0.486) 0.485 0.490

Locus 2378 :

2 alleles

96.9% missing data

3	(0.917) 0.918 0.915

1	(0.083) 0.082 0.085

Locus 2379 :

2 alleles

99.2% missing data

1	(0.691) 0.697 0.689

3	(0.309) 0.303 0.311

Locus 2380 :

2 alleles

90.6% missing data

1	(0.861) 0.884 0.845

3	(0.139) 0.116 0.155

Locus 2381 :

2 alleles

99.2% missing data

1	(0.381) 0.383 0.382

3	(0.619) 0.617 0.618

Locus 2382 :

2 alleles

99.2% missing data

4	(0.613) 0.612 0.608

3	(0.387) 0.388 0.392

Locus 2383 :

2 alleles

95.3% missing data

1	(0.597) 0.568 0.627

2	(0.403) 0.432 0.373

Locus 2384 :

2 alleles

98.4% missing data

4	(0.700) 0.704 0.701

1	(0.300) 0.296 0.299

Locus 2385 :

2 alleles

99.2% missing data

4	(0.555) 0.561 0.549

3	(0.445) 0.439 0.451

Locus 2386 :

2 alleles

98.8% missing data

3	(0.821) 0.825 0.819

1	(0.179) 0.175 0.181

Locus 2387 :

2 alleles

99.2% missing data

4	(0.516) 0.512 0.518

2	(0.484) 0.488 0.482

Locus 2388 :

2 alleles

97.7% missing data

3	(0.367) 0.346 0.374

1	(0.633) 0.654 0.626

Locus 2389 :

2 alleles

98.8% missing data

3	(0.760) 0.767 0.755

1	(0.240) 0.233 0.245

Locus 2390 :

2 alleles

99.2% missing data

3	(0.398) 0.400 0.396

4	(0.602) 0.600 0.604

Locus 2391 :

2 alleles

98.0% missing data

4	(0.708) 0.708 0.711

3	(0.292) 0.292 0.289

Locus 2392 :

2 alleles

99.2% missing data

1	(0.499) 0.493 0.505

4	(0.501) 0.507 0.495

Locus 2393 :

2 alleles

98.8% missing data

4	(0.572) 0.568 0.572

2	(0.428) 0.432 0.428

Locus 2394 :

2 alleles

99.2% missing data

1	(0.486) 0.486 0.483

4	(0.514) 0.514 0.517

Locus 2395 :

2 alleles

99.2% missing data

4	(0.709) 0.704 0.711

3	(0.291) 0.296 0.289

Locus 2396 :

2 alleles

99.2% missing data

4	(0.387) 0.386 0.391

1	(0.613) 0.614 0.609

Locus 2397 :

2 alleles

99.2% missing data

3	(0.814) 0.821 0.807

2	(0.186) 0.179 0.193

Locus 2398 :

2 alleles

99.2% missing data

4	(0.616) 0.618 0.624

3	(0.384) 0.382 0.376

Locus 2399 :

2 alleles

93.0% missing data

4	(0.856) 0.847 0.877

3	(0.144) 0.153 0.123

Locus 2400 :

2 alleles

98.8% missing data

4	(0.446) 0.443 0.459

2	(0.554) 0.557 0.541

Locus 2401 :

2 alleles

99.2% missing data

4	(0.222) 0.220 0.225

3	(0.778) 0.780 0.775

Locus 2402 :

2 alleles

98.8% missing data

4	(0.504) 0.498 0.510

3	(0.496) 0.502 0.490

Locus 2403 :

2 alleles

99.2% missing data

3	(0.379) 0.380 0.386

4	(0.621) 0.620 0.614

Locus 2404 :

2 alleles

99.2% missing data

1	(0.540) 0.540 0.547

4	(0.460) 0.460 0.453

Locus 2405 :

2 alleles

99.2% missing data

1	(0.496) 0.489 0.503

2	(0.504) 0.511 0.497

Locus 2406 :

2 alleles

98.8% missing data

3	(0.561) 0.561 0.556

4	(0.439) 0.439 0.444

Locus 2407 :

2 alleles

0.0% missing data

2	(0.895) 0.739 0.968

3	(0.105) 0.261 0.032

Locus 2408 :

2 alleles

96.9% missing data

3	(0.872) 0.866 0.881

4	(0.128) 0.134 0.119

Locus 2409 :

2 alleles

99.2% missing data

4	(0.471) 0.464 0.475

1	(0.529) 0.536 0.525

Locus 2410 :

2 alleles

90.6% missing data

3	(0.892) 0.907 0.893

1	(0.108) 0.093 0.107

Locus 2411 :

2 alleles

99.2% missing data

2	(0.589) 0.589 0.583

3	(0.411) 0.411 0.417

Locus 2412 :

2 alleles

98.8% missing data

2	(0.396) 0.399 0.391

1	(0.604) 0.601 0.609

Locus 2413 :

3 alleles

98.4% missing data

3	(0.249) 0.251 0.253

1	(0.367) 0.375 0.362

2	(0.384) 0.374 0.385

Locus 2414 :

2 alleles

99.2% missing data

4	(0.382) 0.384 0.389

1	(0.618) 0.616 0.611

Locus 2415 :

2 alleles

99.2% missing data

2	(0.630) 0.626 0.633

3	(0.370) 0.374 0.367

Locus 2416 :

2 alleles

99.2% missing data

3	(0.749) 0.751 0.746

4	(0.251) 0.249 0.254

Locus 2417 :

2 alleles

99.2% missing data

4	(0.350) 0.341 0.355

2	(0.650) 0.659 0.645

Locus 2418 :

3 alleles

96.9% missing data

1	(0.205) 0.205 0.206

2	(0.597) 0.592 0.600

4	(0.198) 0.203 0.194

Locus 2419 :

2 alleles

91.0% missing data

1	(0.904) 0.921 0.894

2	(0.096) 0.079 0.106

Locus 2420 :

3 alleles

97.7% missing data

3	(0.486) 0.499 0.485

2	(0.299) 0.296 0.297

1	(0.215) 0.204 0.217

Locus 2421 :

2 alleles

98.8% missing data

4	(0.583) 0.587 0.578

2	(0.417) 0.413 0.422

Locus 2422 :

2 alleles

98.8% missing data

2	(0.485) 0.487 0.487

1	(0.515) 0.513 0.513

Locus 2423 :

2 alleles

91.8% missing data

3	(0.905) 0.911 0.910

4	(0.095) 0.089 0.090

Locus 2424 :

2 alleles

99.2% missing data

4	(0.580) 0.584 0.579

3	(0.420) 0.416 0.421

Locus 2425 :

2 alleles

99.2% missing data

1	(0.211) 0.208 0.214

4	(0.789) 0.792 0.786

Locus 2426 :

2 alleles

99.2% missing data

4	(0.430) 0.433 0.427

1	(0.570) 0.567 0.573

Locus 2427 :

2 alleles

91.4% missing data

1	(0.919) 0.915 0.930

3	(0.081) 0.085 0.070

Locus 2428 :

2 alleles

99.2% missing data

3	(0.501) 0.499 0.503

1	(0.499) 0.501 0.497

Locus 2429 :

2 alleles

99.2% missing data

4	(0.393) 0.400 0.391

3	(0.607) 0.600 0.609

Locus 2430 :

2 alleles

99.2% missing data

1	(0.685) 0.685 0.680

3	(0.315) 0.315 0.320

Locus 2431 :

2 alleles

99.2% missing data

1	(0.359) 0.357 0.364

2	(0.641) 0.643 0.636

Locus 2432 :

2 alleles

99.2% missing data

3	(0.713) 0.704 0.716

1	(0.287) 0.296 0.284

Locus 2433 :

2 alleles

99.2% missing data

4	(0.650) 0.647 0.643

3	(0.350) 0.353 0.357

Locus 2434 :

2 alleles

99.2% missing data

3	(0.491) 0.490 0.489

1	(0.509) 0.510 0.511

Locus 2435 :

2 alleles

98.8% missing data

3	(0.646) 0.634 0.658

2	(0.354) 0.366 0.342

Locus 2436 :

2 alleles

99.2% missing data

4	(0.442) 0.440 0.443

1	(0.558) 0.560 0.557

Locus 2437 :

2 alleles

95.3% missing data

4	(0.840) 0.839 0.851

3	(0.160) 0.161 0.149

Locus 2438 :

2 alleles

99.2% missing data

1	(0.765) 0.768 0.764

2	(0.235) 0.232 0.236

Locus 2439 :

2 alleles

11.7% missing data

3	(0.977) 0.957 0.993

1	(0.023) 0.043 0.007

Locus 2440 :

2 alleles

99.2% missing data

2	(0.400) 0.401 0.402

1	(0.600) 0.599 0.598

Locus 2441 :

2 alleles

93.4% missing data

1	(0.877) 0.873 0.895

3	(0.123) 0.127 0.105

Locus 2442 :

3 alleles

98.8% missing data

2	(0.557) 0.557 0.553

3	(0.178) 0.173 0.186

4	(0.265) 0.270 0.261

Locus 2443 :

3 alleles

98.4% missing data

4	(0.193) 0.190 0.198

3	(0.304) 0.298 0.316

1	(0.504) 0.512 0.486

Locus 2444 :

2 alleles

98.4% missing data

4	(0.479) 0.478 0.478

2	(0.521) 0.522 0.522

Locus 2445 :

3 alleles

97.7% missing data

1	(0.402) 0.408 0.406

3	(0.421) 0.415 0.420

4	(0.177) 0.177 0.175

Locus 2446 :

3 alleles

80.9% missing data

4	(0.061) 0.074 0.042

1	(0.800) 0.785 0.830

2	(0.139) 0.142 0.128

Locus 2447 :

2 alleles

88.3% missing data

1	(0.727) 0.716 0.753

2	(0.273) 0.284 0.247

Locus 2448 :

2 alleles

99.2% missing data

1	(0.270) 0.261 0.277

3	(0.730) 0.739 0.723

Locus 2449 :

2 alleles

98.4% missing data

1	(0.597) 0.594 0.597

3	(0.403) 0.406 0.403

Locus 2450 :

3 alleles

98.0% missing data

1	(0.332) 0.327 0.343

3	(0.469) 0.467 0.461

2	(0.199) 0.206 0.196

Locus 2451 :

3 alleles

88.7% missing data

3	(0.555) 0.616 0.499

1	(0.296) 0.252 0.346

4	(0.149) 0.132 0.154

Locus 2452 :

2 alleles

99.2% missing data

1	(0.738) 0.735 0.745

3	(0.262) 0.265 0.255

Locus 2453 :

2 alleles

90.6% missing data

1	(0.590) 0.584 0.608

2	(0.410) 0.416 0.392

Locus 2454 :

2 alleles

98.4% missing data

1	(0.395) 0.398 0.396

2	(0.605) 0.602 0.604

Locus 2455 :

4 alleles

97.3% missing data

4	(0.364) 0.360 0.378

3	(0.219) 0.218 0.215

1	(0.228) 0.226 0.230

2	(0.189) 0.196 0.177

Locus 2456 :

2 alleles

99.2% missing data

1	(0.439) 0.435 0.445

4	(0.561) 0.565 0.555

Locus 2457 :

2 alleles

99.2% missing data

4	(0.563) 0.561 0.567

2	(0.437) 0.439 0.433

Locus 2458 :

2 alleles

98.0% missing data

1	(0.419) 0.408 0.429

2	(0.581) 0.592 0.571

Locus 2459 :

2 alleles

98.8% missing data

2	(0.640) 0.639 0.641

3	(0.360) 0.361 0.359

Locus 2460 :

2 alleles

98.8% missing data

1	(0.230) 0.227 0.236

3	(0.770) 0.773 0.764

Locus 2461 :

2 alleles

99.2% missing data

1	(0.409) 0.414 0.407

2	(0.591) 0.586 0.593

Locus 2462 :

2 alleles

99.2% missing data

1	(0.698) 0.695 0.700

4	(0.302) 0.305 0.300

Locus 2463 :

2 alleles

98.8% missing data

1	(0.761) 0.759 0.758

4	(0.239) 0.241 0.242

Locus 2464 :

2 alleles

99.2% missing data

3	(0.526) 0.521 0.525

1	(0.474) 0.479 0.475

Locus 2465 :

2 alleles

97.3% missing data

1	(0.552) 0.541 0.576

2	(0.448) 0.459 0.424

Locus 2466 :

2 alleles

98.8% missing data

1	(0.549) 0.548 0.553

2	(0.451) 0.452 0.447

Locus 2467 :

2 alleles

89.8% missing data

1	(0.617) 0.629 0.604

2	(0.383) 0.371 0.396

Locus 2468 :

2 alleles

93.0% missing data

1	(0.341) 0.332 0.351

2	(0.659) 0.668 0.649

Locus 2469 :

2 alleles

97.7% missing data

1	(0.817) 0.812 0.815

2	(0.183) 0.188 0.185

Locus 2470 :

2 alleles

99.2% missing data

1	(0.515) 0.517 0.510

3	(0.485) 0.483 0.490

Locus 2471 :

2 alleles

99.2% missing data

2	(0.295) 0.296 0.298

1	(0.705) 0.704 0.702

Locus 2472 :

2 alleles

98.8% missing data

2	(0.767) 0.768 0.763

1	(0.233) 0.232 0.237

Locus 2473 :

2 alleles

99.2% missing data

1	(0.549) 0.553 0.547

4	(0.451) 0.447 0.453

Locus 2474 :

2 alleles

94.1% missing data

1	(0.570) 0.574 0.567

2	(0.430) 0.426 0.433

Locus 2475 :

2 alleles

95.7% missing data

2	(0.311) 0.317 0.314

1	(0.689) 0.683 0.686

Locus 2476 :

2 alleles

95.3% missing data

1	(0.757) 0.762 0.752

2	(0.243) 0.238 0.248

Locus 2477 :

3 alleles

97.3% missing data

3	(0.465) 0.461 0.485

1	(0.274) 0.272 0.269

4	(0.261) 0.267 0.246

Locus 2478 :

3 alleles

98.0% missing data

1	(0.758) 0.759 0.755

2	(0.093) 0.094 0.095

3	(0.148) 0.147 0.149

Locus 2479 :

2 alleles

97.7% missing data

2	(0.226) 0.227 0.223

1	(0.774) 0.773 0.777

Locus 2480 :

2 alleles

98.8% missing data

2	(0.538) 0.538 0.545

1	(0.462) 0.462 0.455

Locus 2481 :

3 alleles

98.8% missing data

4	(0.408) 0.407 0.407

1	(0.143) 0.146 0.141

3	(0.449) 0.447 0.452

Locus 2482 :

2 alleles

99.2% missing data

4	(0.807) 0.814 0.796

1	(0.193) 0.186 0.204

Locus 2483 :

2 alleles

96.5% missing data

1	(0.792) 0.803 0.788

4	(0.208) 0.197 0.212

Locus 2484 :

2 alleles

99.2% missing data

2	(0.377) 0.380 0.374

1	(0.623) 0.620 0.626

Locus 2485 :

2 alleles

98.8% missing data

1	(0.469) 0.472 0.476

2	(0.531) 0.528 0.524

Locus 2486 :

2 alleles

98.8% missing data

3	(0.420) 0.417 0.413

1	(0.580) 0.583 0.587

Locus 2487 :

3 alleles

98.8% missing data

1	(0.165) 0.163 0.168

4	(0.303) 0.298 0.309

2	(0.532) 0.539 0.523

Locus 2488 :

2 alleles

99.2% missing data

3	(0.706) 0.710 0.700

4	(0.294) 0.290 0.300

Locus 2489 :

2 alleles

99.2% missing data

3	(0.501) 0.498 0.500

1	(0.499) 0.502 0.500

Locus 2490 :

2 alleles

99.2% missing data

1	(0.402) 0.398 0.406

2	(0.598) 0.602 0.594

Locus 2491 :

2 alleles

96.5% missing data

1	(0.821) 0.823 0.827

4	(0.179) 0.177 0.173

Locus 2492 :

2 alleles

90.6% missing data

1	(0.588) 0.552 0.636

2	(0.412) 0.448 0.364

Locus 2493 :

2 alleles

98.0% missing data

2	(0.797) 0.793 0.801

3	(0.203) 0.207 0.199

Locus 2494 :

2 alleles

89.1% missing data

3	(0.938) 0.942 0.941

2	(0.062) 0.058 0.059

Locus 2495 :

2 alleles

99.2% missing data

1	(0.747) 0.738 0.746

4	(0.253) 0.262 0.254

Locus 2496 :

2 alleles

99.2% missing data

1	(0.374) 0.370 0.374

2	(0.626) 0.630 0.626

Locus 2497 :

2 alleles

0.0% missing data

4	(0.910) 0.765 0.972

3	(0.090) 0.235 0.028

Locus 2498 :

2 alleles

99.2% missing data

4	(0.468) 0.463 0.471

1	(0.532) 0.537 0.529

Locus 2499 :

3 alleles

98.8% missing data

2	(0.406) 0.409 0.402

1	(0.253) 0.248 0.265

4	(0.340) 0.343 0.334

Locus 2500 :

2 alleles

93.0% missing data

2	(0.898) 0.897 0.903

1	(0.102) 0.103 0.097

Locus 2501 :

3 alleles

98.4% missing data

4	(0.237) 0.248 0.233

2	(0.315) 0.311 0.322

1	(0.448) 0.441 0.445

Locus 2502 :

2 alleles

98.0% missing data

3	(0.607) 0.604 0.616

2	(0.393) 0.396 0.384

Locus 2503 :

3 alleles

98.4% missing data

1	(0.315) 0.319 0.314

3	(0.479) 0.473 0.479

4	(0.206) 0.208 0.208

Locus 2504 :

2 alleles

98.8% missing data

4	(0.708) 0.704 0.712

1	(0.292) 0.296 0.288

Locus 2505 :

2 alleles

99.2% missing data

3	(0.628) 0.615 0.630

2	(0.372) 0.385 0.370

Locus 2506 :

2 alleles

99.2% missing data

2	(0.442) 0.441 0.450

1	(0.558) 0.559 0.550

Locus 2507 :

3 alleles

98.4% missing data

2	(0.356) 0.352 0.357

1	(0.359) 0.361 0.360

4	(0.286) 0.287 0.283

Locus 2508 :

2 alleles

99.2% missing data

2	(0.681) 0.681 0.689

1	(0.319) 0.319 0.311

Locus 2509 :

2 alleles

91.4% missing data

1	(0.924) 0.918 0.933

3	(0.076) 0.082 0.067

Locus 2510 :

2 alleles

92.6% missing data

3	(0.892) 0.894 0.902

2	(0.108) 0.106 0.098

Locus 2511 :

2 alleles

98.4% missing data

2	(0.595) 0.606 0.588

1	(0.405) 0.394 0.412

Locus 2512 :

2 alleles

8.6% missing data

2	(0.987) 0.989 0.993

1	(0.013) 0.011 0.007

Locus 2513 :

2 alleles

99.2% missing data

1	(0.708) 0.703 0.716

3	(0.292) 0.297 0.284

Locus 2514 :

2 alleles

99.2% missing data

2	(0.305) 0.309 0.301

3	(0.695) 0.691 0.699

Locus 2515 :

2 alleles

98.8% missing data

1	(0.564) 0.564 0.569

3	(0.436) 0.436 0.431

Locus 2516 :

2 alleles

99.2% missing data

1	(0.391) 0.385 0.398

4	(0.609) 0.615 0.602

Locus 2517 :

3 alleles

91.0% missing data

1	(0.625) 0.608 0.657

2	(0.289) 0.310 0.263

3	(0.086) 0.082 0.080

Locus 2518 :

2 alleles

97.7% missing data

1	(0.597) 0.602 0.607

2	(0.403) 0.398 0.393

Locus 2519 :

2 alleles

8.6% missing data

4	(0.988) 0.981 0.996

3	(0.012) 0.019 0.004

Locus 2520 :

2 alleles

91.8% missing data

1	(0.654) 0.659 0.672

2	(0.346) 0.341 0.328

Locus 2521 :

2 alleles

98.0% missing data

1	(0.748) 0.752 0.745

2	(0.252) 0.248 0.255

Locus 2522 :

2 alleles

98.8% missing data

4	(0.763) 0.753 0.765

2	(0.237) 0.247 0.235

Locus 2523 :

2 alleles

98.4% missing data

1	(0.567) 0.558 0.582

2	(0.433) 0.442 0.418

Locus 2524 :

2 alleles

99.2% missing data

3	(0.759) 0.758 0.752

1	(0.241) 0.242 0.248

Locus 2525 :

2 alleles

99.2% missing data

1	(0.372) 0.365 0.376

2	(0.628) 0.635 0.624

Locus 2526 :

2 alleles

98.8% missing data

2	(0.613) 0.611 0.616

3	(0.387) 0.389 0.384

Locus 2527 :

2 alleles

98.8% missing data

2	(0.513) 0.508 0.513

3	(0.487) 0.492 0.487

Locus 2528 :

2 alleles

91.0% missing data

1	(0.726) 0.724 0.721

2	(0.274) 0.276 0.279

Locus 2529 :

2 alleles

93.8% missing data

1	(0.693) 0.700 0.694

2	(0.307) 0.300 0.306

Locus 2530 :

2 alleles

97.3% missing data

2	(0.191) 0.191 0.196

1	(0.809) 0.809 0.804

Locus 2531 :

2 alleles

94.1% missing data

1	(0.647) 0.650 0.642

2	(0.353) 0.350 0.358

Locus 2532 :

2 alleles

96.9% missing data

2	(0.581) 0.590 0.578

1	(0.419) 0.410 0.422

Locus 2533 :

2 alleles

99.2% missing data

4	(0.712) 0.719 0.704

3	(0.288) 0.281 0.296

Locus 2534 :

2 alleles

96.5% missing data

1	(0.753) 0.760 0.759

3	(0.247) 0.240 0.241

Locus 2535 :

3 alleles

98.8% missing data

4	(0.729) 0.735 0.719

3	(0.136) 0.135 0.143

2	(0.135) 0.130 0.138

Locus 2536 :

2 alleles

97.3% missing data

1	(0.802) 0.802 0.802

4	(0.198) 0.198 0.198

Locus 2537 :

3 alleles

96.5% missing data

4	(0.245) 0.227 0.249

2	(0.572) 0.598 0.569

3	(0.183) 0.175 0.181

Locus 2538 :

2 alleles

98.8% missing data

2	(0.585) 0.585 0.580

3	(0.415) 0.415 0.420

Locus 2539 :

2 alleles

99.2% missing data

1	(0.232) 0.228 0.238

2	(0.768) 0.772 0.762

Locus 2540 :

2 alleles

99.2% missing data

1	(0.494) 0.492 0.495

3	(0.506) 0.508 0.505

Locus 2541 :

2 alleles

98.0% missing data

2	(0.332) 0.325 0.335

4	(0.668) 0.675 0.665

Locus 2542 :

2 alleles

98.8% missing data

2	(0.468) 0.464 0.475

3	(0.532) 0.536 0.525

Locus 2543 :

2 alleles

99.2% missing data

3	(0.353) 0.354 0.355

1	(0.647) 0.646 0.645

Locus 2544 :

2 alleles

96.9% missing data

1	(0.542) 0.547 0.537

2	(0.458) 0.453 0.463

Locus 2545 :

2 alleles

87.1% missing data

1	(0.922) 0.924 0.936

3	(0.078) 0.076 0.064

Locus 2546 :

2 alleles

97.7% missing data

1	(0.327) 0.339 0.325

2	(0.673) 0.661 0.675

Locus 2547 :

3 alleles

98.8% missing data

3	(0.566) 0.559 0.569

2	(0.096) 0.094 0.100

4	(0.338) 0.347 0.331

Locus 2548 :

2 alleles

96.1% missing data

1	(0.478) 0.489 0.466

2	(0.522) 0.511 0.534

Locus 2549 :

2 alleles

99.2% missing data

2	(0.468) 0.468 0.464

3	(0.532) 0.532 0.536

Locus 2550 :

2 alleles

98.8% missing data

4	(0.486) 0.490 0.486

2	(0.514) 0.510 0.514

Locus 2551 :

2 alleles

98.4% missing data

3	(0.778) 0.780 0.780

1	(0.222) 0.220 0.220

Locus 2552 :

2 alleles

99.2% missing data

2	(0.474) 0.467 0.478

1	(0.526) 0.533 0.522

Locus 2553 :

2 alleles

99.2% missing data

2	(0.683) 0.680 0.686

1	(0.317) 0.320 0.314

Locus 2554 :

2 alleles

91.0% missing data

1	(0.931) 0.945 0.923

2	(0.069) 0.055 0.077

Locus 2555 :

2 alleles

99.2% missing data

2	(0.587) 0.590 0.584

4	(0.413) 0.410 0.416

Locus 2556 :

2 alleles

98.0% missing data

2	(0.483) 0.477 0.476

1	(0.517) 0.523 0.524

Locus 2557 :

2 alleles

99.2% missing data

1	(0.605) 0.607 0.606

3	(0.395) 0.393 0.394

Locus 2558 :

2 alleles

98.8% missing data

1	(0.537) 0.533 0.533

4	(0.463) 0.467 0.467

Locus 2559 :

2 alleles

98.0% missing data

2	(0.735) 0.736 0.737

1	(0.265) 0.264 0.263

Locus 2560 :

2 alleles

99.2% missing data

3	(0.635) 0.644 0.631

2	(0.365) 0.356 0.369

Locus 2561 :

2 alleles

99.2% missing data

4	(0.154) 0.156 0.159

1	(0.846) 0.844 0.841

Locus 2562 :

2 alleles

97.3% missing data

1	(0.238) 0.238 0.237

3	(0.762) 0.762 0.763

Locus 2563 :

2 alleles

99.2% missing data

3	(0.349) 0.339 0.352

4	(0.651) 0.661 0.648

Locus 2564 :

2 alleles

97.3% missing data

2	(0.696) 0.679 0.707

3	(0.304) 0.321 0.293

Locus 2565 :

2 alleles

99.2% missing data

4	(0.432) 0.426 0.434

2	(0.568) 0.574 0.566

Locus 2566 :

2 alleles

96.1% missing data

3	(0.183) 0.174 0.181

2	(0.817) 0.826 0.819

Locus 2567 :

2 alleles

99.2% missing data

4	(0.534) 0.533 0.528

1	(0.466) 0.467 0.472

Locus 2568 :

2 alleles

99.2% missing data

1	(0.587) 0.586 0.585

3	(0.413) 0.414 0.415

Locus 2569 :

2 alleles

99.2% missing data

3	(0.517) 0.515 0.516

2	(0.483) 0.485 0.484

Locus 2570 :

2 alleles

99.2% missing data

3	(0.731) 0.730 0.728

2	(0.269) 0.270 0.272

Locus 2571 :

2 alleles

99.2% missing data

2	(0.470) 0.472 0.468

1	(0.530) 0.528 0.532

Locus 2572 :

3 alleles

98.8% missing data

2	(0.220) 0.214 0.227

3	(0.211) 0.220 0.206

1	(0.569) 0.566 0.567

Locus 2573 :

2 alleles

99.2% missing data

4	(0.348) 0.348 0.352

1	(0.652) 0.652 0.648

Locus 2574 :

2 alleles

99.2% missing data

1	(0.504) 0.506 0.507

3	(0.496) 0.494 0.493

Locus 2575 :

2 alleles

98.0% missing data

3	(0.596) 0.604 0.599

1	(0.404) 0.396 0.401

Locus 2576 :

2 alleles

98.8% missing data

1	(0.458) 0.460 0.456

4	(0.542) 0.540 0.544

Locus 2577 :

2 alleles

99.2% missing data

3	(0.348) 0.346 0.348

2	(0.652) 0.654 0.652

Locus 2578 :

2 alleles

97.7% missing data

3	(0.861) 0.863 0.861

2	(0.139) 0.137 0.139

Locus 2579 :

2 alleles

98.4% missing data

2	(0.628) 0.641 0.619

1	(0.372) 0.359 0.381

Locus 2580 :

2 alleles

99.2% missing data

4	(0.511) 0.503 0.512

3	(0.489) 0.497 0.488

Locus 2581 :

2 alleles

97.3% missing data

4	(0.657) 0.652 0.674

3	(0.343) 0.348 0.326

Locus 2582 :

2 alleles

99.2% missing data

2	(0.308) 0.305 0.307

4	(0.692) 0.695 0.693

Locus 2583 :

2 alleles

99.2% missing data

2	(0.190) 0.189 0.190

3	(0.810) 0.811 0.810

Locus 2584 :

2 alleles

98.8% missing data

3	(0.551) 0.560 0.546

1	(0.449) 0.440 0.454

Locus 2585 :

2 alleles

92.6% missing data

1	(0.879) 0.880 0.892

2	(0.121) 0.120 0.108

Locus 2586 :

2 alleles

99.2% missing data

1	(0.390) 0.385 0.395

2	(0.610) 0.615 0.605

Locus 2587 :

2 alleles

99.2% missing data

1	(0.808) 0.804 0.817

3	(0.192) 0.196 0.183

Locus 2588 :

2 alleles

99.2% missing data

2	(0.345) 0.339 0.353

3	(0.655) 0.661 0.647

Locus 2589 :

2 alleles

99.2% missing data

1	(0.493) 0.499 0.483

2	(0.507) 0.501 0.517

Locus 2590 :

2 alleles

99.2% missing data

3	(0.482) 0.483 0.484

1	(0.518) 0.517 0.516

Locus 2591 :

2 alleles

99.2% missing data

1	(0.467) 0.468 0.476

2	(0.533) 0.532 0.524

Locus 2592 :

2 alleles

99.2% missing data

3	(0.743) 0.742 0.740

1	(0.257) 0.258 0.260

Locus 2593 :

2 alleles

9.0% missing data

2	(0.984) 0.979 0.995

1	(0.016) 0.021 0.005

Locus 2594 :

2 alleles

99.2% missing data

3	(0.823) 0.816 0.826

2	(0.177) 0.184 0.174

Locus 2595 :

2 alleles

99.2% missing data

1	(0.500) 0.499 0.498

3	(0.500) 0.501 0.502

Locus 2596 :

2 alleles

98.8% missing data

3	(0.413) 0.404 0.418

4	(0.587) 0.596 0.582

Locus 2597 :

2 alleles

98.8% missing data

1	(0.611) 0.609 0.621

3	(0.389) 0.391 0.379

Locus 2598 :

3 alleles

98.8% missing data

3	(0.348) 0.346 0.354

4	(0.330) 0.326 0.331

1	(0.322) 0.328 0.316

Locus 2599 :

2 alleles

98.8% missing data

3	(0.726) 0.724 0.735

1	(0.274) 0.276 0.265

Locus 2600 :

2 alleles

99.2% missing data

1	(0.483) 0.478 0.486

2	(0.517) 0.522 0.514

Locus 2601 :

2 alleles

98.0% missing data

2	(0.264) 0.255 0.268

1	(0.736) 0.745 0.732

Locus 2602 :

2 alleles

99.2% missing data

2	(0.483) 0.474 0.489

4	(0.517) 0.526 0.511

Locus 2603 :

3 alleles

96.9% missing data

1	(0.219) 0.228 0.210

3	(0.629) 0.622 0.642

2	(0.152) 0.150 0.148

Locus 2604 :

2 alleles

99.2% missing data

2	(0.378) 0.375 0.382

1	(0.622) 0.625 0.618

Locus 2605 :

3 alleles

98.8% missing data

1	(0.216) 0.212 0.219

3	(0.404) 0.409 0.402

2	(0.380) 0.379 0.379

Locus 2606 :

2 alleles

96.1% missing data

2	(0.741) 0.756 0.740

1	(0.259) 0.244 0.260

Locus 2607 :

2 alleles

99.2% missing data

3	(0.373) 0.372 0.373

2	(0.627) 0.628 0.627

Locus 2608 :

2 alleles

90.6% missing data

1	(0.859) 0.876 0.853

3	(0.141) 0.124 0.147

Locus 2609 :

2 alleles

96.1% missing data

1	(0.861) 0.871 0.852

2	(0.139) 0.129 0.148

Locus 2610 :

2 alleles

99.2% missing data

2	(0.436) 0.439 0.435

1	(0.564) 0.561 0.565

Locus 2611 :

2 alleles

99.2% missing data

3	(0.514) 0.520 0.507

2	(0.486) 0.480 0.493

Locus 2612 :

2 alleles

98.0% missing data

1	(0.762) 0.767 0.764

4	(0.238) 0.233 0.236

Locus 2613 :

2 alleles

98.8% missing data

2	(0.297) 0.294 0.302

1	(0.703) 0.706 0.698

Locus 2614 :

3 alleles

98.4% missing data

3	(0.188) 0.192 0.184

2	(0.415) 0.416 0.420

1	(0.397) 0.392 0.396

Locus 2615 :

2 alleles

95.7% missing data

2	(0.783) 0.793 0.782

4	(0.217) 0.207 0.218

Locus 2616 :

2 alleles

98.8% missing data

3	(0.548) 0.549 0.548

1	(0.452) 0.451 0.452

Locus 2617 :

2 alleles

99.2% missing data

3	(0.480) 0.479 0.477

1	(0.520) 0.521 0.523

Locus 2618 :

2 alleles

99.2% missing data

1	(0.389) 0.384 0.383

2	(0.611) 0.616 0.617

Locus 2619 :

2 alleles

99.2% missing data

1	(0.299) 0.296 0.304

4	(0.701) 0.704 0.696

Locus 2620 :

2 alleles

99.2% missing data

3	(0.726) 0.721 0.732

2	(0.274) 0.279 0.268

Locus 2621 :

2 alleles

99.2% missing data

2	(0.765) 0.768 0.761

1	(0.235) 0.232 0.239

Locus 2622 :

3 alleles

98.8% missing data

4	(0.349) 0.349 0.349

1	(0.222) 0.225 0.227

2	(0.429) 0.426 0.424

Locus 2623 :

2 alleles

92.2% missing data

1	(0.894) 0.873 0.914

4	(0.106) 0.127 0.086

Locus 2624 :

2 alleles

98.4% missing data

1	(0.831) 0.837 0.821

2	(0.169) 0.163 0.179

Locus 2625 :

2 alleles

95.3% missing data

1	(0.820) 0.808 0.835

3	(0.180) 0.192 0.165

Locus 2626 :

3 alleles

96.9% missing data

3	(0.116) 0.124 0.105

4	(0.738) 0.724 0.759

2	(0.145) 0.152 0.136

Locus 2627 :

2 alleles

99.2% missing data

4	(0.371) 0.369 0.374

1	(0.629) 0.631 0.626

Locus 2628 :

2 alleles

90.2% missing data

1	(0.773) 0.787 0.776

2	(0.227) 0.213 0.224

Locus 2629 :

2 alleles

90.6% missing data

1	(0.890) 0.890 0.901

2	(0.110) 0.110 0.099

Locus 2630 :

2 alleles

96.5% missing data

1	(0.812) 0.804 0.827

4	(0.188) 0.196 0.173

Locus 2631 :

2 alleles

98.8% missing data

4	(0.378) 0.385 0.375

1	(0.622) 0.615 0.625

Locus 2632 :

2 alleles

96.1% missing data

1	(0.761) 0.759 0.772

2	(0.239) 0.241 0.228

Locus 2633 :

2 alleles

91.0% missing data

1	(0.797) 0.788 0.816

2	(0.203) 0.212 0.184

Locus 2634 :

2 alleles

99.2% missing data

4	(0.249) 0.248 0.254

2	(0.751) 0.752 0.746

Locus 2635 :

2 alleles

98.8% missing data

1	(0.682) 0.682 0.700

4	(0.318) 0.318 0.300

Locus 2636 :

2 alleles

96.9% missing data

1	(0.563) 0.558 0.571

2	(0.437) 0.442 0.429

Locus 2637 :

2 alleles

95.7% missing data

1	(0.848) 0.851 0.855

2	(0.152) 0.149 0.145

Locus 2638 :

2 alleles

98.8% missing data

2	(0.634) 0.631 0.634

1	(0.366) 0.369 0.366

Locus 2639 :

2 alleles

98.0% missing data

1	(0.335) 0.329 0.349

2	(0.665) 0.671 0.651

Locus 2640 :

2 alleles

98.8% missing data

2	(0.331) 0.326 0.336

1	(0.669) 0.674 0.664

Locus 2641 :

2 alleles

99.2% missing data

1	(0.410) 0.408 0.403

2	(0.590) 0.592 0.597

Locus 2642 :

2 alleles

99.2% missing data

2	(0.645) 0.647 0.644

3	(0.355) 0.353 0.356

Locus 2643 :

2 alleles

99.2% missing data

1	(0.731) 0.731 0.722

2	(0.269) 0.269 0.278

Locus 2644 :

2 alleles

99.2% missing data

1	(0.235) 0.240 0.238

2	(0.765) 0.760 0.762

Locus 2645 :

2 alleles

98.4% missing data

2	(0.649) 0.654 0.654

4	(0.351) 0.346 0.346

Locus 2646 :

2 alleles

99.2% missing data

1	(0.531) 0.527 0.529

3	(0.469) 0.473 0.471

Locus 2647 :

2 alleles

99.2% missing data

1	(0.475) 0.464 0.479

2	(0.525) 0.536 0.521

Locus 2648 :

2 alleles

99.2% missing data

2	(0.498) 0.491 0.498

3	(0.502) 0.509 0.502

Locus 2649 :

2 alleles

99.2% missing data

3	(0.261) 0.266 0.256

4	(0.739) 0.734 0.744

Locus 2650 :

2 alleles

99.2% missing data

2	(0.514) 0.508 0.509

1	(0.486) 0.492 0.491

Locus 2651 :

2 alleles

99.2% missing data

4	(0.670) 0.675 0.668

2	(0.330) 0.325 0.332

Locus 2652 :

4 alleles

93.8% missing data

1	(0.665) 0.668 0.683

2	(0.072) 0.069 0.069

4	(0.111) 0.106 0.107

3	(0.152) 0.157 0.140

Locus 2653 :

2 alleles

96.5% missing data

2	(0.388) 0.403 0.375

1	(0.612) 0.597 0.625

Locus 2654 :

3 alleles

98.0% missing data

3	(0.330) 0.333 0.332

2	(0.340) 0.345 0.341

1	(0.330) 0.322 0.327

Locus 2655 :

3 alleles

94.9% missing data

1	(0.396) 0.396 0.389

2	(0.443) 0.449 0.451

3	(0.161) 0.155 0.161

Locus 2656 :

2 alleles

98.8% missing data

2	(0.729) 0.736 0.728

1	(0.271) 0.264 0.272

Locus 2657 :

2 alleles

98.4% missing data

2	(0.528) 0.527 0.527

1	(0.472) 0.473 0.473

Locus 2658 :

2 alleles

98.0% missing data

2	(0.672) 0.678 0.669

1	(0.328) 0.322 0.331

Locus 2659 :

2 alleles

99.2% missing data

2	(0.342) 0.336 0.343

1	(0.658) 0.664 0.657

Locus 2660 :

2 alleles

99.2% missing data

4	(0.687) 0.691 0.689

2	(0.313) 0.309 0.311

Locus 2661 :

2 alleles

99.2% missing data

3	(0.310) 0.307 0.311

2	(0.690) 0.693 0.689

Locus 2662 :

2 alleles

95.3% missing data

2	(0.873) 0.873 0.874

4	(0.127) 0.127 0.126

Locus 2663 :

2 alleles

96.1% missing data

2	(0.854) 0.864 0.845

4	(0.146) 0.136 0.155

Locus 2664 :

2 alleles

99.2% missing data

1	(0.615) 0.610 0.615

2	(0.385) 0.390 0.385

Locus 2665 :

2 alleles

0.4% missing data

2	(0.990) 0.985 0.997

3	(0.010) 0.015 0.003

Locus 2666 :

2 alleles

99.2% missing data

3	(0.274) 0.278 0.274

2	(0.726) 0.722 0.726

Locus 2667 :

2 alleles

99.2% missing data

2	(0.728) 0.726 0.730

3	(0.272) 0.274 0.270

Locus 2668 :

2 alleles

99.2% missing data

4	(0.165) 0.171 0.160

1	(0.835) 0.829 0.840

Locus 2669 :

2 alleles

99.2% missing data

1	(0.314) 0.314 0.316

3	(0.686) 0.686 0.684

Locus 2670 :

2 alleles

98.8% missing data

3	(0.771) 0.766 0.772

1	(0.229) 0.234 0.228

Locus 2671 :

2 alleles

99.2% missing data

1	(0.326) 0.313 0.333

2	(0.674) 0.687 0.667

Locus 2672 :

2 alleles

98.8% missing data

4	(0.573) 0.573 0.568

1	(0.427) 0.427 0.432

Locus 2673 :

2 alleles

99.2% missing data

2	(0.459) 0.465 0.455

4	(0.541) 0.535 0.545

Locus 2674 :

2 alleles

99.2% missing data

1	(0.606) 0.610 0.601

3	(0.394) 0.390 0.399

Locus 2675 :

2 alleles

98.4% missing data

4	(0.320) 0.327 0.319

1	(0.680) 0.673 0.681

Locus 2676 :

2 alleles

99.2% missing data

3	(0.358) 0.350 0.363

4	(0.642) 0.650 0.637

Locus 2677 :

2 alleles

92.6% missing data

3	(0.901) 0.919 0.894

2	(0.099) 0.081 0.106

Locus 2678 :

2 alleles

96.9% missing data

1	(0.668) 0.676 0.674

2	(0.332) 0.324 0.326

Locus 2679 :

2 alleles

96.9% missing data

1	(0.748) 0.738 0.759

2	(0.252) 0.262 0.241

Locus 2680 :

2 alleles

99.2% missing data

1	(0.371) 0.373 0.369

4	(0.629) 0.627 0.631

Locus 2681 :

2 alleles

98.8% missing data

2	(0.569) 0.577 0.565

3	(0.431) 0.423 0.435

Locus 2682 :

2 alleles

97.7% missing data

2	(0.232) 0.240 0.230

4	(0.768) 0.760 0.770

Locus 2683 :

2 alleles

97.3% missing data

1	(0.278) 0.271 0.292

2	(0.722) 0.729 0.708

Locus 2684 :

2 alleles

98.8% missing data

2	(0.729) 0.732 0.727

1	(0.271) 0.268 0.273

Locus 2685 :

2 alleles

99.2% missing data

3	(0.555) 0.554 0.554

2	(0.445) 0.446 0.446

Locus 2686 :

2 alleles

93.8% missing data

2	(0.788) 0.782 0.793

1	(0.212) 0.218 0.207

Locus 2687 :

3 alleles

97.7% missing data

4	(0.170) 0.157 0.180

1	(0.308) 0.304 0.307

2	(0.522) 0.539 0.513

Locus 2688 :

2 alleles

99.2% missing data

3	(0.426) 0.422 0.421

2	(0.574) 0.578 0.579

Locus 2689 :

2 alleles

98.8% missing data

4	(0.177) 0.176 0.182

1	(0.823) 0.824 0.818

Locus 2690 :

2 alleles

15.2% missing data

4	(0.985) 0.980 0.995

1	(0.015) 0.020 0.005

Locus 2691 :

2 alleles

98.4% missing data

1	(0.785) 0.791 0.783

2	(0.215) 0.209 0.217

Locus 2692 :

2 alleles

98.4% missing data

2	(0.652) 0.649 0.665

1	(0.348) 0.351 0.335

Locus 2693 :

2 alleles

97.3% missing data

1	(0.839) 0.832 0.851

4	(0.161) 0.168 0.149

Locus 2694 :

2 alleles

99.2% missing data

4	(0.592) 0.591 0.590

3	(0.408) 0.409 0.410

Locus 2695 :

2 alleles

98.4% missing data

2	(0.491) 0.498 0.482

1	(0.509) 0.502 0.518

Locus 2696 :

2 alleles

99.2% missing data

1	(0.391) 0.396 0.386

2	(0.609) 0.604 0.614

Locus 2697 :

2 alleles

99.2% missing data

1	(0.361) 0.362 0.364

4	(0.639) 0.638 0.636

Locus 2698 :

2 alleles

98.4% missing data

2	(0.818) 0.818 0.816

1	(0.182) 0.182 0.184

Locus 2699 :

2 alleles

98.0% missing data

1	(0.665) 0.675 0.658

2	(0.335) 0.325 0.342

Locus 2700 :

3 alleles

94.5% missing data

1	(0.284) 0.285 0.273

2	(0.560) 0.569 0.575

4	(0.156) 0.146 0.152

Locus 2701 :

2 alleles

99.2% missing data

2	(0.549) 0.551 0.541

1	(0.451) 0.449 0.459

Locus 2702 :

2 alleles

99.2% missing data

2	(0.442) 0.447 0.442

4	(0.558) 0.553 0.558

Locus 2703 :

2 alleles

98.8% missing data

4	(0.652) 0.651 0.654

2	(0.348) 0.349 0.346

Locus 2704 :

2 alleles

98.4% missing data

4	(0.376) 0.382 0.375

1	(0.624) 0.618 0.625

Locus 2705 :

2 alleles

0.0% missing data

2	(0.986) 0.982 0.995

4	(0.014) 0.018 0.005

Locus 2706 :

2 alleles

98.8% missing data

4	(0.753) 0.748 0.753

3	(0.247) 0.252 0.247

Locus 2707 :

2 alleles

99.2% missing data

4	(0.496) 0.492 0.503

3	(0.504) 0.508 0.497

Locus 2708 :

2 alleles

99.2% missing data

3	(0.643) 0.646 0.636

4	(0.357) 0.354 0.364

Locus 2709 :

2 alleles

98.8% missing data

4	(0.643) 0.634 0.648

1	(0.357) 0.366 0.352

Locus 2710 :

2 alleles

96.5% missing data

1	(0.758) 0.765 0.751

2	(0.242) 0.235 0.249

Locus 2711 :

2 alleles

0.0% missing data

2	(0.987) 0.982 0.996

1	(0.013) 0.018 0.004

Locus 2712 :

3 alleles

95.3% missing data

3	(0.235) 0.218 0.246

1	(0.658) 0.681 0.645

4	(0.106) 0.101 0.109

Locus 2713 :

3 alleles

98.8% missing data

4	(0.359) 0.356 0.358

1	(0.328) 0.327 0.330

2	(0.313) 0.317 0.312

Locus 2714 :

2 alleles

95.3% missing data

4	(0.763) 0.763 0.773

2	(0.237) 0.237 0.227

Locus 2715 :

2 alleles

99.2% missing data

4	(0.578) 0.571 0.583

1	(0.422) 0.429 0.417

Locus 2716 :

2 alleles

99.2% missing data

1	(0.451) 0.444 0.460

4	(0.549) 0.556 0.540

Locus 2717 :

2 alleles

99.2% missing data

3	(0.633) 0.628 0.635

2	(0.367) 0.372 0.365

Locus 2718 :

2 alleles

98.4% missing data

4	(0.530) 0.529 0.537

2	(0.470) 0.471 0.463

Locus 2719 :

2 alleles

99.2% missing data

2	(0.439) 0.439 0.438

1	(0.561) 0.561 0.562

Locus 2720 :

2 alleles

99.2% missing data

2	(0.455) 0.457 0.454

1	(0.545) 0.543 0.546

Locus 2721 :

2 alleles

8.2% missing data

3	(0.983) 0.976 0.995

1	(0.017) 0.024 0.005

Locus 2722 :

2 alleles

97.3% missing data

4	(0.252) 0.257 0.249

2	(0.748) 0.743 0.751

Locus 2723 :

2 alleles

99.2% missing data

2	(0.589) 0.579 0.597

4	(0.411) 0.421 0.403

Locus 2724 :

3 alleles

98.4% missing data

2	(0.534) 0.524 0.545

1	(0.309) 0.312 0.302

4	(0.156) 0.165 0.153

Locus 2725 :

3 alleles

96.9% missing data

1	(0.236) 0.221 0.241

3	(0.449) 0.472 0.446

4	(0.314) 0.306 0.314

Locus 2726 :

2 alleles

99.2% missing data

3	(0.232) 0.234 0.226

2	(0.768) 0.766 0.774

Locus 2727 :

2 alleles

99.2% missing data

1	(0.364) 0.362 0.369

2	(0.636) 0.638 0.631

Locus 2728 :

2 alleles

98.8% missing data

3	(0.106) 0.100 0.113

2	(0.894) 0.900 0.887

Locus 2729 :

2 alleles

99.2% missing data

1	(0.392) 0.394 0.392

4	(0.608) 0.606 0.608

Locus 2730 :

2 alleles

99.2% missing data

3	(0.623) 0.625 0.623

4	(0.377) 0.375 0.377

Locus 2731 :

2 alleles

96.1% missing data

3	(0.913) 0.919 0.909

2	(0.087) 0.081 0.091

Locus 2732 :

2 alleles

97.7% missing data

3	(0.846) 0.842 0.857

1	(0.154) 0.158 0.143

Locus 2733 :

2 alleles

99.2% missing data

1	(0.509) 0.511 0.510

3	(0.491) 0.489 0.490

Locus 2734 :

2 alleles

99.2% missing data

1	(0.316) 0.313 0.320

3	(0.684) 0.687 0.680

Locus 2735 :

2 alleles

96.5% missing data

3	(0.202) 0.200 0.203

1	(0.798) 0.800 0.797

Locus 2736 :

2 alleles

99.2% missing data

2	(0.363) 0.362 0.360

3	(0.637) 0.638 0.640

Locus 2737 :

2 alleles

99.2% missing data

4	(0.601) 0.605 0.600

2	(0.399) 0.395 0.400

Locus 2738 :

2 alleles

99.2% missing data

3	(0.640) 0.647 0.637

4	(0.360) 0.353 0.363

Locus 2739 :

2 alleles

98.8% missing data

4	(0.568) 0.575 0.568

1	(0.432) 0.425 0.432

Locus 2740 :

2 alleles

94.5% missing data

2	(0.464) 0.436 0.489

4	(0.536) 0.564 0.511

Locus 2741 :

2 alleles

99.2% missing data

3	(0.541) 0.544 0.545

2	(0.459) 0.456 0.455

Locus 2742 :

2 alleles

99.2% missing data

1	(0.539) 0.546 0.537

2	(0.461) 0.454 0.463

Locus 2743 :

2 alleles

98.4% missing data

1	(0.506) 0.518 0.503

4	(0.494) 0.482 0.497

Locus 2744 :

2 alleles

96.9% missing data

1	(0.171) 0.174 0.164

2	(0.829) 0.826 0.836

Locus 2745 :

2 alleles

99.2% missing data

4	(0.665) 0.659 0.667

1	(0.335) 0.341 0.333

Locus 2746 :

2 alleles

95.7% missing data

1	(0.815) 0.818 0.823

4	(0.185) 0.182 0.177

Locus 2747 :

2 alleles

98.8% missing data

4	(0.774) 0.762 0.782

3	(0.226) 0.238 0.218

Locus 2748 :

2 alleles

99.2% missing data

1	(0.536) 0.538 0.526

3	(0.464) 0.462 0.474

Locus 2749 :

2 alleles

99.2% missing data

2	(0.541) 0.544 0.539

1	(0.459) 0.456 0.461

Locus 2750 :

2 alleles

98.4% missing data

1	(0.853) 0.847 0.857

3	(0.147) 0.153 0.143

Locus 2751 :

2 alleles

96.5% missing data

4	(0.812) 0.817 0.819

2	(0.188) 0.183 0.181

Locus 2752 :

2 alleles

94.9% missing data

3	(0.829) 0.815 0.846

1	(0.171) 0.185 0.154

Locus 2753 :

2 alleles

96.5% missing data

3	(0.809) 0.819 0.806

2	(0.191) 0.181 0.194

Locus 2754 :

2 alleles

96.9% missing data

2	(0.262) 0.252 0.264

3	(0.738) 0.748 0.736

Locus 2755 :

2 alleles

99.2% missing data

4	(0.524) 0.522 0.524

1	(0.476) 0.478 0.476

Locus 2756 :

2 alleles

97.7% missing data

1	(0.694) 0.698 0.695

4	(0.306) 0.302 0.305

Locus 2757 :

2 alleles

99.2% missing data

3	(0.472) 0.468 0.474

1	(0.528) 0.532 0.526

Locus 2758 :

2 alleles

93.0% missing data

3	(0.424) 0.467 0.384

2	(0.576) 0.533 0.616

Locus 2759 :

2 alleles

99.2% missing data

1	(0.243) 0.241 0.249

2	(0.757) 0.759 0.751

Locus 2760 :

2 alleles

0.0% missing data

4	(0.985) 0.988 0.993

1	(0.015) 0.012 0.007

Locus 2761 :

2 alleles

98.0% missing data

1	(0.706) 0.703 0.712

2	(0.294) 0.297 0.288

Locus 2762 :

2 alleles

99.2% missing data

1	(0.547) 0.541 0.549

3	(0.453) 0.459 0.451

Locus 2763 :

2 alleles

99.2% missing data

4	(0.483) 0.483 0.486

2	(0.517) 0.517 0.514

Locus 2764 :

2 alleles

99.2% missing data

2	(0.461) 0.456 0.467

1	(0.539) 0.544 0.533

Locus 2765 :

2 alleles

98.8% missing data

2	(0.696) 0.706 0.690

1	(0.304) 0.294 0.310

Locus 2766 :

2 alleles

99.2% missing data

3	(0.471) 0.468 0.472

2	(0.529) 0.532 0.528

Locus 2767 :

2 alleles

99.2% missing data

3	(0.739) 0.743 0.728

2	(0.261) 0.257 0.272

Locus 2768 :

2 alleles

98.8% missing data

1	(0.515) 0.509 0.517

3	(0.485) 0.491 0.483

Locus 2769 :

2 alleles

10.9% missing data

3	(0.976) 0.948 0.993

1	(0.024) 0.052 0.007

Locus 2770 :

2 alleles

98.8% missing data

4	(0.775) 0.779 0.773

2	(0.225) 0.221 0.227

Locus 2771 :

2 alleles

99.2% missing data

2	(0.444) 0.441 0.446

3	(0.556) 0.559 0.554

Locus 2772 :

2 alleles

9.0% missing data

3	(0.984) 0.978 0.995

2	(0.016) 0.022 0.005

Locus 2773 :

2 alleles

99.2% missing data

1	(0.145) 0.137 0.154

3	(0.855) 0.863 0.846

Locus 2774 :

2 alleles

99.2% missing data

2	(0.555) 0.555 0.553

3	(0.445) 0.445 0.447

Locus 2775 :

2 alleles

99.2% missing data

4	(0.724) 0.726 0.719

1	(0.276) 0.274 0.281

Locus 2776 :

2 alleles

98.4% missing data

3	(0.366) 0.362 0.365

2	(0.634) 0.638 0.635

Locus 2777 :

2 alleles

98.8% missing data

1	(0.572) 0.573 0.575

4	(0.428) 0.427 0.425

Locus 2778 :

2 alleles

99.2% missing data

3	(0.716) 0.716 0.716

1	(0.284) 0.284 0.284

Locus 2779 :

2 alleles

98.4% missing data

1	(0.573) 0.576 0.572

3	(0.427) 0.424 0.428

Locus 2780 :

2 alleles

97.7% missing data

1	(0.196) 0.198 0.193

4	(0.804) 0.802 0.807

Locus 2781 :

2 alleles

99.2% missing data

4	(0.289) 0.290 0.286

1	(0.711) 0.710 0.714

Locus 2782 :

2 alleles

99.2% missing data

1	(0.351) 0.348 0.348

4	(0.649) 0.652 0.652

Locus 2783 :

2 alleles

99.2% missing data

2	(0.617) 0.617 0.617

1	(0.383) 0.383 0.383

Locus 2784 :

2 alleles

98.8% missing data

1	(0.550) 0.549 0.552

2	(0.450) 0.451 0.448

Locus 2785 :

2 alleles

99.2% missing data

1	(0.508) 0.508 0.507

4	(0.492) 0.492 0.493

Locus 2786 :

2 alleles

10.2% missing data

1	(0.986) 0.980 0.995

2	(0.014) 0.020 0.005

Locus 2787 :

3 alleles

97.7% missing data

1	(0.230) 0.225 0.229

4	(0.406) 0.410 0.407

2	(0.364) 0.365 0.365

Locus 2788 :

2 alleles

99.2% missing data

4	(0.622) 0.621 0.626

1	(0.378) 0.379 0.374

Locus 2789 :

3 alleles

93.4% missing data

2	(0.862) 0.856 0.874

3	(0.086) 0.094 0.076

4	(0.052) 0.051 0.050

Locus 2790 :

2 alleles

98.8% missing data

2	(0.472) 0.465 0.482

4	(0.528) 0.535 0.518

Locus 2791 :

2 alleles

99.2% missing data

2	(0.655) 0.658 0.653

1	(0.345) 0.342 0.347

Locus 2792 :

2 alleles

98.4% missing data

1	(0.562) 0.564 0.566

4	(0.438) 0.436 0.434

Locus 2793 :

2 alleles

99.2% missing data

2	(0.653) 0.657 0.651

1	(0.347) 0.343 0.349

Locus 2794 :

2 alleles

99.2% missing data

4	(0.366) 0.371 0.371

1	(0.634) 0.629 0.629

Locus 2795 :

2 alleles

98.4% missing data

2	(0.559) 0.555 0.557

4	(0.441) 0.445 0.443

Locus 2796 :

2 alleles

98.4% missing data

3	(0.326) 0.320 0.333

2	(0.674) 0.680 0.667

Locus 2797 :

2 alleles

99.2% missing data

4	(0.501) 0.504 0.499

1	(0.499) 0.496 0.501

Locus 2798 :

2 alleles

99.2% missing data

1	(0.366) 0.366 0.373

2	(0.634) 0.634 0.627

Locus 2799 :

2 alleles

98.0% missing data

1	(0.298) 0.298 0.288

4	(0.702) 0.702 0.712

Locus 2800 :

2 alleles

98.8% missing data

2	(0.780) 0.772 0.787

1	(0.220) 0.228 0.213

Locus 2801 :

2 alleles

99.2% missing data

1	(0.450) 0.457 0.441

4	(0.550) 0.543 0.559

Locus 2802 :

2 alleles

99.2% missing data

2	(0.632) 0.634 0.630

3	(0.368) 0.366 0.370

Locus 2803 :

2 alleles

99.2% missing data

2	(0.246) 0.246 0.251

1	(0.754) 0.754 0.749

Locus 2804 :

2 alleles

99.2% missing data

4	(0.593) 0.590 0.589

3	(0.407) 0.410 0.411

Locus 2805 :

2 alleles

99.2% missing data

4	(0.572) 0.581 0.558

3	(0.428) 0.419 0.442

Locus 2806 :

2 alleles

96.9% missing data

1	(0.157) 0.154 0.156

3	(0.843) 0.846 0.844

Locus 2807 :

2 alleles

96.1% missing data

3	(0.262) 0.267 0.257

2	(0.738) 0.733 0.743

Locus 2808 :

3 alleles

96.1% missing data

1	(0.638) 0.655 0.633

4	(0.193) 0.188 0.195

2	(0.169) 0.156 0.172

Locus 2809 :

2 alleles

98.4% missing data

2	(0.116) 0.115 0.115

4	(0.884) 0.885 0.885

Locus 2810 :

2 alleles

98.0% missing data

1	(0.478) 0.473 0.487

4	(0.522) 0.527 0.513

Locus 2811 :

2 alleles

96.1% missing data

4	(0.170) 0.156 0.176

2	(0.830) 0.844 0.824

Locus 2812 :

2 alleles

98.0% missing data

2	(0.206) 0.200 0.209

1	(0.794) 0.800 0.791

Locus 2813 :

3 alleles

98.8% missing data

1	(0.173) 0.170 0.174

3	(0.436) 0.436 0.436

2	(0.392) 0.394 0.390

Locus 2814 :

2 alleles

0.4% missing data

1	(0.984) 0.981 0.995

2	(0.016) 0.019 0.005

Locus 2815 :

2 alleles

99.2% missing data

1	(0.248) 0.244 0.253

2	(0.752) 0.756 0.747

Locus 2816 :

2 alleles

99.2% missing data

2	(0.336) 0.337 0.341

1	(0.664) 0.663 0.659

Locus 2817 :

2 alleles

99.2% missing data

1	(0.250) 0.255 0.249

4	(0.750) 0.745 0.751

Locus 2818 :

2 alleles

99.2% missing data

2	(0.386) 0.383 0.383

3	(0.614) 0.617 0.617

Locus 2819 :

3 alleles

98.4% missing data

1	(0.420) 0.419 0.428

3	(0.223) 0.218 0.224

4	(0.357) 0.363 0.348

Locus 2820 :

2 alleles

98.8% missing data

1	(0.751) 0.755 0.752

4	(0.249) 0.245 0.248

Locus 2821 :

2 alleles

97.3% missing data

4	(0.174) 0.180 0.170

3	(0.826) 0.820 0.830

Locus 2822 :

2 alleles

94.5% missing data

1	(0.919) 0.928 0.913

4	(0.081) 0.072 0.087

Locus 2823 :

2 alleles

99.2% missing data

4	(0.719) 0.724 0.716

1	(0.281) 0.276 0.284

Locus 2824 :

2 alleles

99.2% missing data

1	(0.641) 0.642 0.647

4	(0.359) 0.358 0.353

Locus 2825 :

2 alleles

0.0% missing data

1	(0.650) 0.533 0.749

4	(0.350) 0.467 0.251

Locus 2826 :

2 alleles

98.8% missing data

4	(0.541) 0.540 0.538

2	(0.459) 0.460 0.462

Locus 2827 :

2 alleles

99.2% missing data

3	(0.481) 0.479 0.485

2	(0.519) 0.521 0.515

Locus 2828 :

2 alleles

99.2% missing data

1	(0.434) 0.443 0.429

2	(0.566) 0.557 0.571

Locus 2829 :

2 alleles

98.8% missing data

3	(0.724) 0.721 0.731

1	(0.276) 0.279 0.269

Locus 2830 :

2 alleles

99.2% missing data

1	(0.346) 0.350 0.343

2	(0.654) 0.650 0.657

Locus 2831 :

2 alleles

98.4% missing data

4	(0.272) 0.268 0.276

1	(0.728) 0.732 0.724

Locus 2832 :

2 alleles

99.2% missing data

4	(0.644) 0.646 0.648

1	(0.356) 0.354 0.352

Locus 2833 :

2 alleles

99.2% missing data

2	(0.502) 0.502 0.504

4	(0.498) 0.498 0.496

Locus 2834 :

3 alleles

95.7% missing data

2	(0.141) 0.133 0.148

1	(0.792) 0.806 0.777

3	(0.067) 0.060 0.074

Locus 2835 :

2 alleles

92.6% missing data

4	(0.861) 0.862 0.876

1	(0.139) 0.138 0.124

Locus 2836 :

3 alleles

96.5% missing data

1	(0.353) 0.378 0.331

4	(0.147) 0.144 0.147

3	(0.500) 0.477 0.522

Locus 2837 :

2 alleles

94.9% missing data

1	(0.863) 0.864 0.872

2	(0.137) 0.136 0.128

Locus 2838 :

2 alleles

99.2% missing data

3	(0.584) 0.586 0.579

2	(0.416) 0.414 0.421

Locus 2839 :

3 alleles

98.0% missing data

1	(0.510) 0.516 0.506

4	(0.282) 0.277 0.286

3	(0.208) 0.207 0.207

Locus 2840 :

2 alleles

99.2% missing data

2	(0.687) 0.680 0.689

4	(0.313) 0.320 0.311

Locus 2841 :

2 alleles

96.1% missing data

2	(0.862) 0.867 0.863

3	(0.138) 0.133 0.137

Locus 2842 :

2 alleles

0.0% missing data

4	(0.988) 0.991 0.993

1	(0.012) 0.009 0.007

Locus 2843 :

2 alleles

98.8% missing data

2	(0.315) 0.313 0.327

4	(0.685) 0.687 0.673

Locus 2844 :

2 alleles

98.8% missing data

1	(0.761) 0.752 0.771

2	(0.239) 0.248 0.229

Locus 2845 :

3 alleles

92.2% missing data

4	(0.842) 0.834 0.856

3	(0.086) 0.086 0.082

2	(0.072) 0.080 0.062

Locus 2846 :

2 alleles

99.2% missing data

4	(0.684) 0.678 0.687

1	(0.316) 0.322 0.313

Locus 2847 :

2 alleles

99.2% missing data

2	(0.623) 0.618 0.626

4	(0.377) 0.382 0.374

Locus 2848 :

2 alleles

99.2% missing data

2	(0.656) 0.645 0.664

1	(0.344) 0.355 0.336

Locus 2849 :

2 alleles

99.2% missing data

4	(0.620) 0.621 0.618

3	(0.380) 0.379 0.382

Locus 2850 :

3 alleles

92.6% missing data

1	(0.162) 0.166 0.139

2	(0.690) 0.678 0.735

4	(0.148) 0.157 0.126

Locus 2851 :

2 alleles

98.0% missing data

1	(0.772) 0.760 0.784

3	(0.228) 0.240 0.216

Locus 2852 :

2 alleles

97.7% missing data

4	(0.726) 0.729 0.734

2	(0.274) 0.271 0.266

Locus 2853 :

2 alleles

99.2% missing data

1	(0.491) 0.492 0.489

2	(0.509) 0.508 0.511

Locus 2854 :

2 alleles

99.2% missing data

2	(0.276) 0.271 0.276

3	(0.724) 0.729 0.724

Locus 2855 :

2 alleles

97.7% missing data

3	(0.687) 0.697 0.679

1	(0.313) 0.303 0.321

Locus 2856 :

2 alleles

99.2% missing data

1	(0.735) 0.733 0.737

2	(0.265) 0.267 0.263

Locus 2857 :

2 alleles

99.2% missing data

2	(0.625) 0.631 0.618

3	(0.375) 0.369 0.382

Locus 2858 :

2 alleles

99.2% missing data

2	(0.410) 0.412 0.411

4	(0.590) 0.588 0.589

Locus 2859 :

2 alleles

99.2% missing data

4	(0.474) 0.474 0.475

3	(0.526) 0.526 0.525

Locus 2860 :

2 alleles

98.8% missing data

1	(0.325) 0.319 0.327

4	(0.675) 0.681 0.673

Locus 2861 :

3 alleles

97.7% missing data

4	(0.343) 0.345 0.349

1	(0.324) 0.323 0.318

2	(0.333) 0.331 0.333

Locus 2862 :

2 alleles

94.9% missing data

1	(0.365) 0.361 0.364

2	(0.635) 0.639 0.636

Locus 2863 :

2 alleles

99.2% missing data

3	(0.439) 0.431 0.443

2	(0.561) 0.569 0.557

Locus 2864 :

2 alleles

98.4% missing data

1	(0.682) 0.682 0.686

2	(0.318) 0.318 0.314

Locus 2865 :

2 alleles

92.6% missing data

4	(0.883) 0.880 0.895

2	(0.117) 0.120 0.105

Locus 2866 :

2 alleles

98.8% missing data

3	(0.583) 0.582 0.590

1	(0.417) 0.418 0.410

Locus 2867 :

3 alleles

95.7% missing data

4	(0.135) 0.124 0.144

3	(0.656) 0.676 0.646

1	(0.208) 0.200 0.210

Locus 2868 :

3 alleles

97.3% missing data

3	(0.564) 0.557 0.585

1	(0.208) 0.208 0.201

2	(0.228) 0.235 0.214

Locus 2869 :

2 alleles

92.2% missing data

3	(0.522) 0.486 0.558

1	(0.478) 0.514 0.442

Locus 2870 :

3 alleles

98.8% missing data

4	(0.425) 0.417 0.433

1	(0.384) 0.383 0.385

2	(0.191) 0.199 0.182

Locus 2871 :

2 alleles

98.8% missing data

4	(0.523) 0.523 0.529

3	(0.477) 0.477 0.471

Locus 2872 :

2 alleles

99.2% missing data

4	(0.603) 0.600 0.603

3	(0.397) 0.400 0.397

Locus 2873 :

2 alleles

98.4% missing data

1	(0.474) 0.470 0.475

2	(0.526) 0.530 0.525

Locus 2874 :

2 alleles

98.8% missing data

2	(0.493) 0.492 0.494

4	(0.507) 0.508 0.506

Locus 2875 :

2 alleles

98.4% missing data

1	(0.663) 0.656 0.665

2	(0.337) 0.344 0.335

Locus 2876 :

2 alleles

95.3% missing data

3	(0.786) 0.789 0.790

2	(0.214) 0.211 0.210

Locus 2877 :

2 alleles

0.0% missing data

1	(0.985) 0.988 0.989

4	(0.015) 0.012 0.011

Locus 2878 :

2 alleles

99.2% missing data

3	(0.605) 0.598 0.609

1	(0.395) 0.402 0.391

Locus 2879 :

2 alleles

92.6% missing data

3	(0.905) 0.894 0.921

4	(0.095) 0.106 0.079

Locus 2880 :

2 alleles

93.4% missing data

3	(0.844) 0.862 0.840

1	(0.156) 0.138 0.160

Locus 2881 :

2 alleles

99.2% missing data

3	(0.544) 0.549 0.534

4	(0.456) 0.451 0.466

Locus 2882 :

2 alleles

98.8% missing data

2	(0.737) 0.732 0.749

1	(0.263) 0.268 0.251

Locus 2883 :

3 alleles

98.8% missing data

4	(0.141) 0.140 0.144

3	(0.408) 0.411 0.409

2	(0.452) 0.450 0.447

Locus 2884 :

2 alleles

98.0% missing data

4	(0.664) 0.672 0.660

1	(0.336) 0.328 0.340

Locus 2885 :

2 alleles

99.2% missing data

1	(0.255) 0.255 0.258

4	(0.745) 0.745 0.742

Locus 2886 :

3 alleles

98.8% missing data

2	(0.209) 0.213 0.210

4	(0.462) 0.461 0.465

3	(0.329) 0.326 0.325

Locus 2887 :

2 alleles

98.8% missing data

2	(0.533) 0.528 0.539

1	(0.467) 0.472 0.461

Locus 2888 :

2 alleles

99.2% missing data

3	(0.300) 0.296 0.301

4	(0.700) 0.704 0.699

Locus 2889 :

2 alleles

98.8% missing data

3	(0.772) 0.774 0.772

1	(0.228) 0.226 0.228

Locus 2890 :

2 alleles

98.8% missing data

4	(0.700) 0.689 0.702

2	(0.300) 0.311 0.298

Locus 2891 :

2 alleles

98.8% missing data

1	(0.283) 0.280 0.284

4	(0.717) 0.720 0.716

Locus 2892 :

2 alleles

99.2% missing data

3	(0.236) 0.230 0.239

2	(0.764) 0.770 0.761

Locus 2893 :

2 alleles

99.2% missing data

3	(0.474) 0.485 0.472

4	(0.526) 0.515 0.528

Locus 2894 :

2 alleles

99.2% missing data

1	(0.496) 0.490 0.494

2	(0.504) 0.510 0.506

Locus 2895 :

2 alleles

99.2% missing data

2	(0.524) 0.521 0.524

1	(0.476) 0.479 0.476

Locus 2896 :

2 alleles

99.2% missing data

3	(0.574) 0.572 0.570

2	(0.426) 0.428 0.430

Locus 2897 :

2 alleles

98.4% missing data

4	(0.733) 0.729 0.744

3	(0.267) 0.271 0.256

Locus 2898 :

3 alleles

90.2% missing data

1	(0.798) 0.832 0.779

3	(0.109) 0.091 0.121

4	(0.093) 0.077 0.100

Locus 2899 :

2 alleles

97.7% missing data

3	(0.777) 0.774 0.789

1	(0.223) 0.226 0.211

Locus 2900 :

2 alleles

98.8% missing data

1	(0.528) 0.526 0.531

4	(0.472) 0.474 0.469

Locus 2901 :

2 alleles

99.2% missing data

2	(0.648) 0.653 0.639

4	(0.352) 0.347 0.361

Locus 2902 :

2 alleles

98.8% missing data

3	(0.299) 0.294 0.301

4	(0.701) 0.706 0.699

Locus 2903 :

2 alleles

97.3% missing data

2	(0.815) 0.809 0.825

1	(0.185) 0.191 0.175

Locus 2904 :

2 alleles

99.2% missing data

1	(0.561) 0.556 0.565

3	(0.439) 0.444 0.435

Locus 2905 :

2 alleles

99.2% missing data

2	(0.389) 0.391 0.389

1	(0.611) 0.609 0.611

Locus 2906 :

2 alleles

92.6% missing data

1	(0.875) 0.892 0.866

3	(0.125) 0.108 0.134

Locus 2907 :

2 alleles

99.2% missing data

4	(0.660) 0.654 0.662

3	(0.340) 0.346 0.338

Locus 2908 :

2 alleles

99.2% missing data

3	(0.518) 0.520 0.517

4	(0.482) 0.480 0.483

Locus 2909 :

2 alleles

99.2% missing data

1	(0.414) 0.424 0.405

2	(0.586) 0.576 0.595

Locus 2910 :

2 alleles

98.8% missing data

4	(0.427) 0.421 0.444

3	(0.573) 0.579 0.556

Locus 2911 :

2 alleles

99.2% missing data

2	(0.610) 0.619 0.604

1	(0.390) 0.381 0.396

Locus 2912 :

2 alleles

91.4% missing data

1	(0.902) 0.908 0.904

3	(0.098) 0.092 0.096

Locus 2913 :

2 alleles

99.2% missing data

2	(0.433) 0.431 0.433

3	(0.567) 0.569 0.567

Locus 2914 :

2 alleles

99.2% missing data

4	(0.621) 0.624 0.618

2	(0.379) 0.376 0.382

Locus 2915 :

2 alleles

98.4% missing data

2	(0.839) 0.838 0.841

1	(0.161) 0.162 0.159

Locus 2916 :

2 alleles

99.2% missing data

3	(0.629) 0.621 0.629

2	(0.371) 0.379 0.371

Locus 2917 :

2 alleles

99.2% missing data

4	(0.318) 0.311 0.326

1	(0.682) 0.689 0.674

Locus 2918 :

2 alleles

98.0% missing data

2	(0.337) 0.336 0.333

3	(0.663) 0.664 0.667

Locus 2919 :

2 alleles

96.1% missing data

3	(0.803) 0.814 0.796

1	(0.197) 0.186 0.204

Locus 2920 :

2 alleles

94.5% missing data

4	(0.862) 0.858 0.875

3	(0.138) 0.142 0.125

Locus 2921 :

2 alleles

99.2% missing data

3	(0.511) 0.504 0.511

1	(0.489) 0.496 0.489

Locus 2922 :

2 alleles

94.9% missing data

4	(0.880) 0.883 0.882

3	(0.120) 0.117 0.118

Locus 2923 :

2 alleles

95.3% missing data

1	(0.099) 0.106 0.088

2	(0.901) 0.894 0.912

Locus 2924 :

2 alleles

99.2% missing data

2	(0.486) 0.482 0.488

3	(0.514) 0.518 0.512

Locus 2925 :

2 alleles

99.2% missing data

4	(0.290) 0.284 0.299

3	(0.710) 0.716 0.701

Locus 2926 :

2 alleles

99.2% missing data

2	(0.777) 0.779 0.775

4	(0.223) 0.221 0.225

Locus 2927 :

2 alleles

99.2% missing data

1	(0.337) 0.332 0.345

2	(0.663) 0.668 0.655

Locus 2928 :

2 alleles

99.2% missing data

1	(0.841) 0.831 0.844

4	(0.159) 0.169 0.156

Locus 2929 :

2 alleles

96.1% missing data

3	(0.811) 0.816 0.811

2	(0.189) 0.184 0.189

Locus 2930 :

2 alleles

97.7% missing data

1	(0.573) 0.573 0.574

4	(0.427) 0.427 0.426

Locus 2931 :

2 alleles

98.4% missing data

1	(0.352) 0.351 0.354

2	(0.648) 0.649 0.646

Locus 2932 :

2 alleles

99.2% missing data

2	(0.322) 0.315 0.324

4	(0.678) 0.685 0.676

Locus 2933 :

3 alleles

97.7% missing data

1	(0.355) 0.355 0.375

2	(0.295) 0.296 0.291

4	(0.350) 0.349 0.334

Locus 2934 :

2 alleles

98.8% missing data

4	(0.708) 0.711 0.710

2	(0.292) 0.289 0.290

Locus 2935 :

2 alleles

99.2% missing data

3	(0.392) 0.390 0.391

2	(0.608) 0.610 0.609

Locus 2936 :

2 alleles

98.4% missing data

3	(0.450) 0.445 0.452

4	(0.550) 0.555 0.548

Locus 2937 :

2 alleles

83.6% missing data

4	(0.460) 0.534 0.394

3	(0.540) 0.466 0.606

Locus 2938 :

2 alleles

99.2% missing data

1	(0.192) 0.199 0.191

3	(0.808) 0.801 0.809

Locus 2939 :

3 alleles

98.8% missing data

3	(0.446) 0.441 0.447

4	(0.263) 0.258 0.266

1	(0.292) 0.302 0.288

Locus 2940 :

3 alleles

97.3% missing data

3	(0.138) 0.135 0.139

4	(0.297) 0.303 0.281

2	(0.565) 0.562 0.579

Locus 2941 :

2 alleles

99.2% missing data

2	(0.742) 0.736 0.740

1	(0.258) 0.264 0.260

Locus 2942 :

2 alleles

99.2% missing data

1	(0.403) 0.398 0.413

3	(0.597) 0.602 0.587

Locus 2943 :

2 alleles

99.2% missing data

4	(0.636) 0.626 0.645

3	(0.364) 0.374 0.355

Locus 2944 :

3 alleles

98.4% missing data

2	(0.371) 0.361 0.379

3	(0.451) 0.455 0.446

1	(0.178) 0.183 0.175

Locus 2945 :

2 alleles

99.2% missing data

4	(0.357) 0.358 0.355

3	(0.643) 0.642 0.645

Locus 2946 :

2 alleles

9.0% missing data

4	(0.961) 0.930 0.984

3	(0.039) 0.070 0.016

Locus 2947 :

2 alleles

99.2% missing data

4	(0.308) 0.310 0.313

2	(0.692) 0.690 0.687

Locus 2948 :

2 alleles

96.5% missing data

4	(0.636) 0.656 0.637

3	(0.364) 0.344 0.363

Locus 2949 :

2 alleles

98.0% missing data

3	(0.638) 0.637 0.644

1	(0.362) 0.363 0.356

Locus 2950 :

2 alleles

99.2% missing data

2	(0.216) 0.221 0.213

1	(0.784) 0.779 0.787

Locus 2951 :

2 alleles

99.2% missing data

4	(0.375) 0.378 0.375

1	(0.625) 0.622 0.625

Locus 2952 :

2 alleles

99.2% missing data

2	(0.629) 0.632 0.629

4	(0.371) 0.368 0.371

Locus 2953 :

2 alleles

98.8% missing data

3	(0.654) 0.656 0.658

2	(0.346) 0.344 0.342

Locus 2954 :

2 alleles

99.2% missing data

4	(0.782) 0.776 0.785

2	(0.218) 0.224 0.215

Locus 2955 :

2 alleles

97.7% missing data

1	(0.168) 0.172 0.162

4	(0.832) 0.828 0.838

Locus 2956 :

2 alleles

10.9% missing data

3	(0.978) 0.961 0.993

2	(0.022) 0.039 0.007

Locus 2957 :

2 alleles

99.2% missing data

3	(0.521) 0.517 0.529

1	(0.479) 0.483 0.471

Locus 2958 :

2 alleles

99.2% missing data

1	(0.664) 0.670 0.659

4	(0.336) 0.330 0.341

Locus 2959 :

2 alleles

97.7% missing data

1	(0.753) 0.746 0.760

4	(0.247) 0.254 0.240

Locus 2960 :

2 alleles

94.5% missing data

3	(0.858) 0.851 0.875

4	(0.142) 0.149 0.125

Locus 2961 :

2 alleles

98.4% missing data

2	(0.412) 0.420 0.411

1	(0.588) 0.580 0.589

Locus 2962 :

2 alleles

99.2% missing data

2	(0.355) 0.359 0.353

1	(0.645) 0.641 0.647

Locus 2963 :

2 alleles

93.0% missing data

2	(0.906) 0.904 0.911

4	(0.094) 0.096 0.089

Locus 2964 :

3 alleles

98.8% missing data

3	(0.185) 0.177 0.191

4	(0.386) 0.399 0.377

2	(0.429) 0.424 0.432

Locus 2965 :

2 alleles

99.2% missing data

2	(0.387) 0.388 0.384

4	(0.613) 0.612 0.616

Locus 2966 :

2 alleles

14.8% missing data

3	(0.983) 0.987 0.990

4	(0.017) 0.013 0.010

Locus 2967 :

2 alleles

99.2% missing data

2	(0.383) 0.382 0.387

3	(0.617) 0.618 0.613

Locus 2968 :

2 alleles

99.2% missing data

3	(0.682) 0.674 0.685

2	(0.318) 0.326 0.315

Locus 2969 :

2 alleles

99.2% missing data

1	(0.173) 0.170 0.175

2	(0.827) 0.830 0.825

Locus 2970 :

2 alleles

98.0% missing data

2	(0.434) 0.441 0.421

4	(0.566) 0.559 0.579

Locus 2971 :

2 alleles

93.4% missing data

4	(0.909) 0.909 0.914

3	(0.091) 0.091 0.086

Locus 2972 :

2 alleles

87.1% missing data

1	(0.617) 0.693 0.557

3	(0.383) 0.307 0.443

Locus 2973 :

2 alleles

91.0% missing data

4	(0.947) 0.955 0.941

1	(0.053) 0.045 0.059

Locus 2974 :

2 alleles

99.2% missing data

2	(0.601) 0.594 0.603

1	(0.399) 0.406 0.397

Locus 2975 :

2 alleles

98.4% missing data

3	(0.509) 0.502 0.521

4	(0.491) 0.498 0.479

Locus 2976 :

2 alleles

98.8% missing data

4	(0.437) 0.432 0.446

3	(0.563) 0.568 0.554

Locus 2977 :

2 alleles

99.2% missing data

2	(0.684) 0.684 0.677

4	(0.316) 0.316 0.323

Locus 2978 :

2 alleles

99.2% missing data

2	(0.347) 0.350 0.343

1	(0.653) 0.650 0.657

Locus 2979 :

2 alleles

99.2% missing data

1	(0.482) 0.480 0.486

3	(0.518) 0.520 0.514

Locus 2980 :

2 alleles

96.1% missing data

1	(0.129) 0.123 0.128

3	(0.871) 0.877 0.872

Locus 2981 :

2 alleles

99.2% missing data

1	(0.380) 0.379 0.381

4	(0.620) 0.621 0.619

Locus 2982 :

2 alleles

99.2% missing data

1	(0.566) 0.565 0.557

3	(0.434) 0.435 0.443

Locus 2983 :

3 alleles

98.4% missing data

3	(0.603) 0.599 0.603

2	(0.216) 0.214 0.221

1	(0.181) 0.188 0.176

Locus 2984 :

2 alleles

98.4% missing data

3	(0.370) 0.362 0.372

2	(0.630) 0.638 0.628

Locus 2985 :

2 alleles

98.8% missing data

1	(0.531) 0.524 0.544

3	(0.469) 0.476 0.456

Locus 2986 :

2 alleles

96.1% missing data

1	(0.830) 0.840 0.829

4	(0.170) 0.160 0.171

Locus 2987 :

2 alleles

99.2% missing data

2	(0.613) 0.609 0.607

3	(0.387) 0.391 0.393

Locus 2988 :

2 alleles

99.2% missing data

1	(0.217) 0.214 0.220

2	(0.783) 0.786 0.780

Locus 2989 :

2 alleles

99.2% missing data

2	(0.231) 0.234 0.228

1	(0.769) 0.766 0.772

Locus 2990 :

2 alleles

98.4% missing data

1	(0.684) 0.688 0.684

4	(0.316) 0.312 0.316

Locus 2991 :

2 alleles

0.0% missing data

2	(0.986) 0.989 0.993

3	(0.014) 0.011 0.007

Locus 2992 :

2 alleles

99.2% missing data

2	(0.457) 0.454 0.462

3	(0.543) 0.546 0.538

Locus 2993 :

3 alleles

98.4% missing data

2	(0.408) 0.407 0.409

1	(0.164) 0.162 0.167

3	(0.428) 0.430 0.424

Locus 2994 :

2 alleles

99.2% missing data

2	(0.685) 0.682 0.684

3	(0.315) 0.318 0.316

Locus 2995 :

2 alleles

99.2% missing data

2	(0.268) 0.274 0.259

4	(0.732) 0.726 0.741

Locus 2996 :

2 alleles

99.2% missing data

3	(0.529) 0.524 0.528

4	(0.471) 0.476 0.472

Locus 2997 :

2 alleles

99.2% missing data

3	(0.599) 0.601 0.597

2	(0.401) 0.399 0.403

Locus 2998 :

2 alleles

99.2% missing data

3	(0.531) 0.530 0.526

4	(0.469) 0.470 0.474

Locus 2999 :

2 alleles

97.3% missing data

1	(0.829) 0.829 0.828

2	(0.171) 0.171 0.172

Locus 3000 :

2 alleles

99.2% missing data

2	(0.297) 0.293 0.297

3	(0.703) 0.707 0.703

Locus 3001 :

2 alleles

96.5% missing data

2	(0.648) 0.641 0.651

1	(0.352) 0.359 0.349

Locus 3002 :

2 alleles

98.8% missing data

2	(0.675) 0.676 0.668

1	(0.325) 0.324 0.332

Locus 3003 :

3 alleles

98.8% missing data

4	(0.350) 0.351 0.347

1	(0.325) 0.321 0.331

3	(0.325) 0.328 0.323

Locus 3004 :

2 alleles

92.6% missing data

2	(0.659) 0.665 0.643

1	(0.341) 0.335 0.357

Locus 3005 :

3 alleles

96.9% missing data

4	(0.200) 0.190 0.200

2	(0.525) 0.526 0.524

1	(0.275) 0.284 0.276

Locus 3006 :

2 alleles

99.2% missing data

4	(0.490) 0.488 0.493

1	(0.510) 0.512 0.507

Locus 3007 :

2 alleles

98.4% missing data

1	(0.658) 0.654 0.665

2	(0.342) 0.346 0.335

Locus 3008 :

3 alleles

94.5% missing data

4	(0.622) 0.621 0.637

2	(0.224) 0.227 0.213

1	(0.153) 0.152 0.150

Locus 3009 :

2 alleles

98.4% missing data

1	(0.225) 0.217 0.240

4	(0.775) 0.783 0.760

Locus 3010 :

2 alleles

99.2% missing data

1	(0.494) 0.492 0.487

4	(0.506) 0.508 0.513

Locus 3011 :

2 alleles

98.8% missing data

2	(0.478) 0.472 0.486

1	(0.522) 0.528 0.514

Locus 3012 :

2 alleles

99.2% missing data

4	(0.381) 0.379 0.388

1	(0.619) 0.621 0.612

Locus 3013 :

2 alleles

97.3% missing data

2	(0.221) 0.220 0.218

4	(0.779) 0.780 0.782

Locus 3014 :

2 alleles

97.3% missing data

4	(0.655) 0.649 0.670

1	(0.345) 0.351 0.330

Locus 3015 :

2 alleles

99.2% missing data

1	(0.574) 0.568 0.576

4	(0.426) 0.432 0.424

Locus 3016 :

2 alleles

98.0% missing data

2	(0.844) 0.847 0.844

3	(0.156) 0.153 0.156

Locus 3017 :

3 alleles

89.8% missing data

2	(0.255) 0.266 0.248

1	(0.622) 0.603 0.641

3	(0.123) 0.131 0.111

Locus 3018 :

3 alleles

97.7% missing data

1	(0.260) 0.261 0.254

4	(0.507) 0.504 0.512

2	(0.233) 0.235 0.234

Locus 3019 :

2 alleles

99.2% missing data

1	(0.403) 0.403 0.403

4	(0.597) 0.597 0.597

Locus 3020 :

2 alleles

99.2% missing data

3	(0.618) 0.614 0.618

1	(0.382) 0.386 0.382

Locus 3021 :

2 alleles

97.7% missing data

2	(0.831) 0.838 0.820

3	(0.169) 0.162 0.180

Locus 3022 :

2 alleles

99.2% missing data

4	(0.677) 0.677 0.675

2	(0.323) 0.323 0.325

Locus 3023 :

2 alleles

97.3% missing data

1	(0.425) 0.421 0.415

2	(0.575) 0.579 0.585

Locus 3024 :

2 alleles

98.4% missing data

2	(0.356) 0.354 0.351

1	(0.644) 0.646 0.649

Locus 3025 :

3 alleles

98.4% missing data

2	(0.557) 0.563 0.548

1	(0.228) 0.223 0.234

3	(0.215) 0.215 0.218

Locus 3026 :

2 alleles

98.8% missing data

1	(0.555) 0.551 0.565

4	(0.445) 0.449 0.435

Locus 3027 :

2 alleles

98.8% missing data

2	(0.500) 0.492 0.502

1	(0.500) 0.508 0.498

Locus 3028 :

2 alleles

99.2% missing data

2	(0.360) 0.350 0.363

1	(0.640) 0.650 0.637

Locus 3029 :

2 alleles

99.2% missing data

4	(0.373) 0.371 0.379

1	(0.627) 0.629 0.621

Locus 3030 :

2 alleles

99.2% missing data

1	(0.303) 0.306 0.306

2	(0.697) 0.694 0.694

Locus 3031 :

2 alleles

99.2% missing data

2	(0.607) 0.608 0.601

3	(0.393) 0.392 0.399

Locus 3032 :

2 alleles

92.6% missing data

4	(0.881) 0.865 0.900

1	(0.119) 0.135 0.100

Locus 3033 :

3 alleles

98.8% missing data

2	(0.177) 0.174 0.181

1	(0.185) 0.180 0.188

3	(0.638) 0.646 0.631

Locus 3034 :

2 alleles

99.2% missing data

4	(0.631) 0.627 0.626

3	(0.369) 0.373 0.374

Locus 3035 :

2 alleles

99.2% missing data

1	(0.385) 0.386 0.389

3	(0.615) 0.614 0.611

Locus 3036 :

3 alleles

98.8% missing data

3	(0.261) 0.258 0.265

4	(0.257) 0.261 0.261

2	(0.482) 0.480 0.475

Locus 3037 :

2 alleles

99.2% missing data

2	(0.694) 0.684 0.697

4	(0.306) 0.316 0.303

Locus 3038 :

2 alleles

91.0% missing data

3	(0.906) 0.922 0.897

4	(0.094) 0.078 0.103

Locus 3039 :

2 alleles

99.2% missing data

1	(0.284) 0.283 0.283

4	(0.716) 0.717 0.717

Locus 3040 :

2 alleles

97.7% missing data

2	(0.720) 0.716 0.728

4	(0.280) 0.284 0.272

Locus 3041 :

2 alleles

98.8% missing data

4	(0.386) 0.390 0.382

3	(0.614) 0.610 0.618

Locus 3042 :

2 alleles

99.2% missing data

4	(0.540) 0.539 0.544

2	(0.460) 0.461 0.456

Locus 3043 :

2 alleles

99.2% missing data

2	(0.426) 0.430 0.426

3	(0.574) 0.570 0.574

Locus 3044 :

2 alleles

97.7% missing data

4	(0.284) 0.281 0.276

1	(0.716) 0.719 0.724

Locus 3045 :

2 alleles

9.8% missing data

3	(0.986) 0.980 0.995

2	(0.014) 0.020 0.005

Locus 3046 :

2 alleles

97.7% missing data

1	(0.484) 0.481 0.490

4	(0.516) 0.519 0.510

Locus 3047 :

2 alleles

98.4% missing data

1	(0.510) 0.508 0.519

2	(0.490) 0.492 0.481

Locus 3048 :

2 alleles

96.9% missing data

4	(0.717) 0.721 0.724

1	(0.283) 0.279 0.276

Locus 3049 :

2 alleles

99.2% missing data

4	(0.444) 0.435 0.446

1	(0.556) 0.565 0.554

Locus 3050 :

3 alleles

94.1% missing data

4	(0.656) 0.685 0.629

2	(0.105) 0.097 0.108

1	(0.239) 0.218 0.263

Locus 3051 :

2 alleles

99.2% missing data

1	(0.268) 0.268 0.266

2	(0.732) 0.732 0.734

Locus 3052 :

2 alleles

97.3% missing data

3	(0.395) 0.411 0.385

2	(0.605) 0.589 0.615

Locus 3053 :

4 alleles

95.7% missing data

1	(0.246) 0.243 0.250

4	(0.531) 0.550 0.523

2	(0.123) 0.116 0.125

3	(0.100) 0.091 0.102

Locus 3054 :

2 alleles

98.8% missing data

1	(0.530) 0.525 0.535

2	(0.470) 0.475 0.465

Locus 3055 :

2 alleles

96.5% missing data

2	(0.868) 0.877 0.862

1	(0.132) 0.123 0.138

Locus 3056 :

2 alleles

99.2% missing data

3	(0.641) 0.644 0.635

4	(0.359) 0.356 0.365

Locus 3057 :

2 alleles

99.2% missing data

1	(0.333) 0.336 0.336

3	(0.667) 0.664 0.664

Locus 3058 :

2 alleles

89.8% missing data

3	(0.812) 0.821 0.806

4	(0.188) 0.179 0.194

Locus 3059 :

2 alleles

99.2% missing data

4	(0.329) 0.336 0.327

3	(0.671) 0.664 0.673

Locus 3060 :

2 alleles

99.2% missing data

1	(0.375) 0.368 0.381

2	(0.625) 0.632 0.619

Locus 3061 :

2 alleles

99.2% missing data

1	(0.493) 0.502 0.491

4	(0.507) 0.498 0.509

Locus 3062 :

2 alleles

98.4% missing data

2	(0.361) 0.360 0.359

1	(0.639) 0.640 0.641

Locus 3063 :

2 alleles

98.8% missing data

3	(0.344) 0.348 0.342

1	(0.656) 0.652 0.658

Locus 3064 :

2 alleles

99.2% missing data

2	(0.551) 0.547 0.555

1	(0.449) 0.453 0.445

Locus 3065 :

2 alleles

97.7% missing data

1	(0.643) 0.641 0.646

2	(0.357) 0.359 0.354

Locus 3066 :

2 alleles

99.2% missing data

4	(0.620) 0.614 0.619

1	(0.380) 0.386 0.381

Locus 3067 :

3 alleles

98.8% missing data

4	(0.453) 0.449 0.454

1	(0.264) 0.262 0.263

2	(0.283) 0.289 0.283

Locus 3068 :

2 alleles

99.2% missing data

2	(0.604) 0.599 0.606

4	(0.396) 0.401 0.394

Locus 3069 :

2 alleles

98.8% missing data

1	(0.649) 0.649 0.648

2	(0.351) 0.351 0.352

Locus 3070 :

2 alleles

99.2% missing data

2	(0.324) 0.318 0.332

1	(0.676) 0.682 0.668

Locus 3071 :

3 alleles

98.8% missing data

3	(0.220) 0.224 0.220

2	(0.428) 0.429 0.430

4	(0.353) 0.347 0.350

Locus 3072 :

2 alleles

98.4% missing data

2	(0.734) 0.733 0.737

1	(0.266) 0.267 0.263

Locus 3073 :

2 alleles

99.2% missing data

4	(0.690) 0.683 0.689

2	(0.310) 0.317 0.311

Locus 3074 :

2 alleles

98.8% missing data

1	(0.643) 0.648 0.635

2	(0.357) 0.352 0.365

Locus 3075 :

2 alleles

96.9% missing data

2	(0.783) 0.790 0.784

1	(0.217) 0.210 0.216

Locus 3076 :

3 alleles

98.8% missing data

4	(0.501) 0.497 0.496

1	(0.307) 0.308 0.311

2	(0.193) 0.195 0.193

Locus 3077 :

2 alleles

8.6% missing data

3	(0.985) 0.986 0.993

2	(0.015) 0.014 0.007

Locus 3078 :

2 alleles

99.2% missing data

4	(0.653) 0.655 0.655

1	(0.347) 0.345 0.345

Locus 3079 :

2 alleles

98.8% missing data

3	(0.617) 0.609 0.619

4	(0.383) 0.391 0.381

Locus 3080 :

3 alleles

98.0% missing data

2	(0.120) 0.116 0.124

3	(0.461) 0.475 0.453

4	(0.419) 0.408 0.423

Locus 3081 :

2 alleles

97.3% missing data

4	(0.294) 0.291 0.287

2	(0.706) 0.709 0.713

Locus 3082 :

2 alleles

99.2% missing data

1	(0.563) 0.555 0.557

3	(0.437) 0.445 0.443

Locus 3083 :

2 alleles

98.8% missing data

2	(0.536) 0.531 0.538

1	(0.464) 0.469 0.462

Locus 3084 :

2 alleles

98.8% missing data

2	(0.674) 0.677 0.678

3	(0.326) 0.323 0.322

Locus 3085 :

2 alleles

99.2% missing data

2	(0.449) 0.452 0.451

4	(0.551) 0.548 0.549

Locus 3086 :

2 alleles

99.2% missing data

3	(0.414) 0.413 0.418

4	(0.586) 0.587 0.582

Locus 3087 :

2 alleles

99.2% missing data

4	(0.608) 0.608 0.603

2	(0.392) 0.392 0.397

Locus 3088 :

2 alleles

99.2% missing data

3	(0.606) 0.608 0.597

4	(0.394) 0.392 0.403

Locus 3089 :

2 alleles

98.4% missing data

3	(0.762) 0.766 0.760

2	(0.238) 0.234 0.240

Locus 3090 :

2 alleles

99.2% missing data

4	(0.450) 0.446 0.453

1	(0.550) 0.554 0.547

Locus 3091 :

2 alleles

99.2% missing data

1	(0.475) 0.474 0.477

4	(0.525) 0.526 0.523

Locus 3092 :

2 alleles

98.8% missing data

2	(0.798) 0.799 0.792

4	(0.202) 0.201 0.208

Locus 3093 :

2 alleles

97.7% missing data

4	(0.179) 0.184 0.180

2	(0.821) 0.816 0.820

Locus 3094 :

2 alleles

95.7% missing data

4	(0.392) 0.372 0.401

1	(0.608) 0.628 0.599

Locus 3095 :

2 alleles

99.2% missing data

4	(0.247) 0.252 0.241

3	(0.753) 0.748 0.759

Locus 3096 :

2 alleles

0.0% missing data

4	(0.978) 0.961 0.993

1	(0.022) 0.039 0.007

Locus 3097 :

2 alleles

98.8% missing data

2	(0.622) 0.621 0.627

1	(0.378) 0.379 0.373

Locus 3098 :

2 alleles

99.2% missing data

1	(0.566) 0.564 0.565

4	(0.434) 0.436 0.435

Locus 3099 :

2 alleles

99.2% missing data

3	(0.512) 0.507 0.510

4	(0.488) 0.493 0.490

Locus 3100 :

2 alleles

99.2% missing data

2	(0.531) 0.528 0.529

3	(0.469) 0.472 0.471

Locus 3101 :

2 alleles

97.3% missing data

1	(0.308) 0.304 0.297

4	(0.692) 0.696 0.703

Locus 3102 :

2 alleles

0.0% missing data

4	(0.985) 0.980 0.996

1	(0.015) 0.020 0.004

Locus 3103 :

2 alleles

96.5% missing data

2	(0.779) 0.788 0.781

1	(0.221) 0.212 0.219

Locus 3104 :

2 alleles

93.0% missing data

3	(0.870) 0.865 0.884

2	(0.130) 0.135 0.116

Locus 3105 :

2 alleles

99.2% missing data

1	(0.417) 0.411 0.419

4	(0.583) 0.589 0.581

Locus 3106 :

2 alleles

99.2% missing data

4	(0.422) 0.422 0.419

2	(0.578) 0.578 0.581

Locus 3107 :

2 alleles

80.1% missing data

2	(0.956) 0.956 0.967

4	(0.044) 0.044 0.033

Locus 3108 :

2 alleles

99.2% missing data

2	(0.496) 0.499 0.489

1	(0.504) 0.501 0.511

Locus 3109 :

2 alleles

99.2% missing data

4	(0.622) 0.621 0.618

1	(0.378) 0.379 0.382

Locus 3110 :

2 alleles

99.2% missing data

4	(0.494) 0.492 0.493

1	(0.506) 0.508 0.507

Locus 3111 :

2 alleles

99.2% missing data

2	(0.486) 0.486 0.489

4	(0.514) 0.514 0.511

Locus 3112 :

2 alleles

99.2% missing data

2	(0.540) 0.543 0.543

4	(0.460) 0.457 0.457

Locus 3113 :

2 alleles

99.2% missing data

1	(0.789) 0.790 0.778

3	(0.211) 0.210 0.222

Locus 3114 :

2 alleles

99.2% missing data

4	(0.567) 0.563 0.565

3	(0.433) 0.437 0.435

Locus 3115 :

2 alleles

99.2% missing data

4	(0.255) 0.251 0.262

2	(0.745) 0.749 0.738

Locus 3116 :

3 alleles

96.5% missing data

4	(0.698) 0.695 0.714

3	(0.135) 0.131 0.134

2	(0.167) 0.174 0.152

Locus 3117 :

2 alleles

99.2% missing data

1	(0.299) 0.290 0.306

4	(0.701) 0.710 0.694

Locus 3118 :

2 alleles

98.8% missing data

2	(0.489) 0.491 0.491

4	(0.511) 0.509 0.509

Locus 3119 :

2 alleles

90.2% missing data

3	(0.914) 0.927 0.910

1	(0.086) 0.073 0.090

Locus 3120 :

2 alleles

98.8% missing data

2	(0.661) 0.663 0.666

1	(0.339) 0.337 0.334

Locus 3121 :

2 alleles

99.2% missing data

4	(0.524) 0.520 0.520

3	(0.476) 0.480 0.480

Locus 3122 :

2 alleles

99.2% missing data

1	(0.491) 0.488 0.499

3	(0.509) 0.512 0.501

Locus 3123 :

2 alleles

99.2% missing data

4	(0.376) 0.381 0.363

1	(0.624) 0.619 0.637

Locus 3124 :

2 alleles

99.2% missing data

4	(0.662) 0.656 0.661

2	(0.338) 0.344 0.339

Locus 3125 :

2 alleles

91.0% missing data

3	(0.913) 0.906 0.923

2	(0.087) 0.094 0.077

Locus 3126 :

2 alleles

89.5% missing data

4	(0.814) 0.846 0.784

2	(0.186) 0.154 0.216

Locus 3127 :

2 alleles

98.4% missing data

4	(0.591) 0.585 0.604

1	(0.409) 0.415 0.396

Locus 3128 :

2 alleles

90.2% missing data

4	(0.899) 0.912 0.891

2	(0.101) 0.088 0.109

Locus 3129 :

2 alleles

99.2% missing data

1	(0.126) 0.123 0.132

3	(0.874) 0.877 0.868

Locus 3130 :

2 alleles

98.8% missing data

3	(0.551) 0.552 0.550

4	(0.449) 0.448 0.450

Locus 3131 :

2 alleles

99.2% missing data

2	(0.656) 0.655 0.650

4	(0.344) 0.345 0.350

Locus 3132 :

2 alleles

99.2% missing data

4	(0.559) 0.566 0.559

2	(0.441) 0.434 0.441

Locus 3133 :

2 alleles

99.2% missing data

4	(0.743) 0.732 0.747

2	(0.257) 0.268 0.253

Locus 3134 :

2 alleles

98.4% missing data

1	(0.413) 0.403 0.431

3	(0.587) 0.597 0.569

Locus 3135 :

2 alleles

98.8% missing data

4	(0.588) 0.587 0.590

3	(0.412) 0.413 0.410

Locus 3136 :

2 alleles

99.2% missing data

2	(0.386) 0.378 0.392

4	(0.614) 0.622 0.608

Locus 3137 :

2 alleles

99.2% missing data

4	(0.475) 0.471 0.477

3	(0.525) 0.529 0.523

Locus 3138 :

2 alleles

99.2% missing data

2	(0.701) 0.701 0.701

4	(0.299) 0.299 0.299

Locus 3139 :

2 alleles

98.0% missing data

3	(0.731) 0.725 0.745

4	(0.269) 0.275 0.255

Locus 3140 :

3 alleles

97.7% missing data

1	(0.442) 0.450 0.436

2	(0.260) 0.249 0.274

4	(0.299) 0.301 0.290

Locus 3141 :

2 alleles

98.0% missing data

1	(0.698) 0.706 0.685

2	(0.302) 0.294 0.315

Locus 3142 :

3 alleles

98.0% missing data

3	(0.520) 0.524 0.531

2	(0.220) 0.219 0.214

1	(0.260) 0.257 0.256

Locus 3143 :

2 alleles

99.2% missing data

1	(0.448) 0.449 0.448

4	(0.552) 0.551 0.552

Locus 3144 :

2 alleles

97.7% missing data

2	(0.681) 0.678 0.684

1	(0.319) 0.322 0.316

Locus 3145 :

2 alleles

97.3% missing data

2	(0.737) 0.740 0.724

1	(0.263) 0.260 0.276

Locus 3146 :

2 alleles

99.2% missing data

1	(0.431) 0.432 0.433

2	(0.569) 0.568 0.567

Locus 3147 :

2 alleles

98.8% missing data

1	(0.865) 0.869 0.864

2	(0.135) 0.131 0.136

Locus 3148 :

2 alleles

98.0% missing data

2	(0.687) 0.689 0.678

1	(0.313) 0.311 0.322

Locus 3149 :

2 alleles

97.7% missing data

1	(0.574) 0.579 0.567

2	(0.426) 0.421 0.433

Locus 3150 :

2 alleles

99.2% missing data

4	(0.625) 0.619 0.635

1	(0.375) 0.381 0.365

Locus 3151 :

2 alleles

99.2% missing data

4	(0.553) 0.557 0.542

1	(0.447) 0.443 0.458

Locus 3152 :

3 alleles

96.1% missing data

1	(0.586) 0.583 0.603

4	(0.178) 0.174 0.171

2	(0.237) 0.243 0.226

Locus 3153 :

2 alleles

98.4% missing data

1	(0.512) 0.522 0.517

4	(0.488) 0.478 0.483

Locus 3154 :

2 alleles

97.3% missing data

2	(0.772) 0.775 0.776

3	(0.228) 0.225 0.224

Locus 3155 :

3 alleles

98.8% missing data

3	(0.358) 0.359 0.357

2	(0.232) 0.238 0.229

4	(0.410) 0.403 0.413

Locus 3156 :

2 alleles

98.4% missing data

1	(0.397) 0.388 0.416

3	(0.603) 0.612 0.584

Locus 3157 :

2 alleles

99.2% missing data

4	(0.147) 0.146 0.148

2	(0.853) 0.854 0.852

Locus 3158 :

2 alleles

98.8% missing data

2	(0.405) 0.401 0.417

4	(0.595) 0.599 0.583

Locus 3159 :

3 alleles

98.8% missing data

3	(0.413) 0.415 0.415

1	(0.404) 0.400 0.399

2	(0.183) 0.184 0.186

Locus 3160 :

2 alleles

99.2% missing data

2	(0.547) 0.551 0.541

3	(0.453) 0.449 0.459

Locus 3161 :

2 alleles

98.4% missing data

2	(0.515) 0.519 0.523

1	(0.485) 0.481 0.477

Locus 3162 :

4 alleles

97.7% missing data

2	(0.378) 0.386 0.375

4	(0.116) 0.112 0.118

1	(0.195) 0.195 0.197

3	(0.311) 0.307 0.310

Locus 3163 :

2 alleles

98.8% missing data

3	(0.253) 0.254 0.252

4	(0.747) 0.746 0.748

Locus 3164 :

2 alleles

99.2% missing data

1	(0.711) 0.711 0.707

3	(0.289) 0.289 0.293

Locus 3165 :

2 alleles

99.2% missing data

1	(0.628) 0.621 0.637

3	(0.372) 0.379 0.363

Locus 3166 :

2 alleles

99.2% missing data

4	(0.663) 0.661 0.667

2	(0.337) 0.339 0.333

Locus 3167 :

2 alleles

99.2% missing data

1	(0.490) 0.489 0.489

2	(0.510) 0.511 0.511

Locus 3168 :

3 alleles

98.0% missing data

3	(0.219) 0.221 0.220

2	(0.505) 0.492 0.515

4	(0.276) 0.287 0.266

Locus 3169 :

2 alleles

90.2% missing data

4	(0.922) 0.936 0.915

3	(0.078) 0.064 0.085

Locus 3170 :

2 alleles

97.7% missing data

2	(0.756) 0.751 0.770

4	(0.244) 0.249 0.230

Locus 3171 :

3 alleles

98.8% missing data

4	(0.272) 0.273 0.266

3	(0.295) 0.293 0.303

1	(0.434) 0.435 0.431

Locus 3172 :

2 alleles

99.2% missing data

2	(0.485) 0.488 0.485

4	(0.515) 0.512 0.515

Locus 3173 :

2 alleles

98.8% missing data

4	(0.725) 0.718 0.727

2	(0.275) 0.282 0.273

Locus 3174 :

3 alleles

96.1% missing data

2	(0.548) 0.529 0.564

1	(0.303) 0.324 0.286

3	(0.148) 0.147 0.150

Locus 3175 :

2 alleles

98.4% missing data

2	(0.573) 0.572 0.573

1	(0.427) 0.428 0.427

Locus 3176 :

2 alleles

98.4% missing data

1	(0.577) 0.576 0.573

2	(0.423) 0.424 0.427

Locus 3177 :

2 alleles

98.4% missing data

2	(0.468) 0.469 0.472

1	(0.532) 0.531 0.528

Locus 3178 :

2 alleles

99.2% missing data

2	(0.645) 0.653 0.638

1	(0.355) 0.347 0.362

Locus 3179 :

2 alleles

98.4% missing data

2	(0.664) 0.663 0.674

1	(0.336) 0.337 0.326

Locus 3180 :

2 alleles

99.2% missing data

2	(0.749) 0.755 0.740

1	(0.251) 0.245 0.260

Locus 3181 :

3 alleles

94.9% missing data

1	(0.219) 0.204 0.231

4	(0.486) 0.514 0.478

2	(0.295) 0.282 0.292

Locus 3182 :

2 alleles

98.8% missing data

1	(0.354) 0.352 0.354

2	(0.646) 0.648 0.646

Locus 3183 :

2 alleles

98.8% missing data

2	(0.619) 0.618 0.616

1	(0.381) 0.382 0.384

Locus 3184 :

2 alleles

99.2% missing data

2	(0.512) 0.510 0.519

1	(0.488) 0.490 0.481

Locus 3185 :

2 alleles

98.4% missing data

2	(0.727) 0.733 0.726

3	(0.273) 0.267 0.274

Locus 3186 :

2 alleles

93.0% missing data

4	(0.820) 0.818 0.839

2	(0.180) 0.182 0.161

Locus 3187 :

2 alleles

98.8% missing data

3	(0.450) 0.448 0.453

1	(0.550) 0.552 0.547

Locus 3188 :

2 alleles

98.4% missing data

3	(0.559) 0.561 0.562

4	(0.441) 0.439 0.438

Locus 3189 :

3 alleles

97.3% missing data

4	(0.273) 0.271 0.281

1	(0.474) 0.469 0.480

2	(0.253) 0.260 0.240

Locus 3190 :

2 alleles

99.2% missing data

4	(0.714) 0.712 0.712

2	(0.286) 0.288 0.288

Locus 3191 :

2 alleles

98.8% missing data

4	(0.598) 0.603 0.604

2	(0.402) 0.397 0.396

Locus 3192 :

2 alleles

98.4% missing data

1	(0.296) 0.288 0.295

2	(0.704) 0.712 0.705

Locus 3193 :

2 alleles

98.0% missing data

1	(0.323) 0.314 0.329

3	(0.677) 0.686 0.671

Locus 3194 :

2 alleles

99.2% missing data

2	(0.724) 0.723 0.716

1	(0.276) 0.277 0.284

Locus 3195 :

2 alleles

98.8% missing data

1	(0.601) 0.608 0.598

2	(0.399) 0.392 0.402

Locus 3196 :

2 alleles

96.9% missing data

2	(0.637) 0.635 0.641

1	(0.363) 0.365 0.359

Locus 3197 :

3 alleles

98.8% missing data

3	(0.446) 0.449 0.439

4	(0.211) 0.212 0.216

1	(0.344) 0.339 0.345

Locus 3198 :

2 alleles

99.2% missing data

3	(0.464) 0.468 0.463

4	(0.536) 0.532 0.537

Locus 3199 :

2 alleles

98.0% missing data

2	(0.877) 0.881 0.870

1	(0.123) 0.119 0.130

Locus 3200 :

2 alleles

73.4% missing data

2	(0.967) 0.971 0.974

1	(0.033) 0.029 0.026

Locus 3201 :

2 alleles

2.7% missing data

1	(0.984) 0.987 0.992

2	(0.016) 0.013 0.008

Locus 3202 :

2 alleles

95.3% missing data

2	(0.272) 0.259 0.279

4	(0.728) 0.741 0.721

Locus 3203 :

2 alleles

99.2% missing data

4	(0.495) 0.488 0.498

2	(0.505) 0.512 0.502

Locus 3204 :

2 alleles

98.4% missing data

1	(0.244) 0.234 0.254

2	(0.756) 0.766 0.746

Locus 3205 :

2 alleles

99.2% missing data

2	(0.573) 0.563 0.576

4	(0.427) 0.437 0.424

Locus 3206 :

2 alleles

99.2% missing data

1	(0.605) 0.605 0.604

4	(0.395) 0.395 0.396

Locus 3207 :

2 alleles

98.8% missing data

2	(0.472) 0.478 0.469

3	(0.528) 0.522 0.531

Locus 3208 :

3 alleles

98.8% missing data

3	(0.205) 0.206 0.204

2	(0.291) 0.289 0.289

1	(0.504) 0.505 0.507

Locus 3209 :

2 alleles

99.2% missing data

1	(0.386) 0.385 0.379

4	(0.614) 0.615 0.621

Locus 3210 :

2 alleles

98.8% missing data

4	(0.342) 0.335 0.347

2	(0.658) 0.665 0.653

Locus 3211 :

2 alleles

97.7% missing data

2	(0.506) 0.513 0.489

1	(0.494) 0.487 0.511

Locus 3212 :

2 alleles

91.0% missing data

4	(0.953) 0.962 0.946

3	(0.047) 0.038 0.054

Locus 3213 :

2 alleles

90.2% missing data

4	(0.931) 0.947 0.923

2	(0.069) 0.053 0.077

Locus 3214 :

2 alleles

98.8% missing data

4	(0.852) 0.850 0.855

3	(0.148) 0.150 0.145

Locus 3215 :

2 alleles

99.2% missing data

1	(0.376) 0.373 0.378

4	(0.624) 0.627 0.622

Locus 3216 :

2 alleles

97.3% missing data

4	(0.435) 0.422 0.452

2	(0.565) 0.578 0.548

Locus 3217 :

2 alleles

99.2% missing data

1	(0.562) 0.562 0.567

3	(0.438) 0.438 0.433

Locus 3218 :

3 alleles

93.8% missing data

3	(0.546) 0.583 0.526

1	(0.395) 0.366 0.413

4	(0.058) 0.051 0.061

Locus 3219 :

2 alleles

95.3% missing data

1	(0.894) 0.898 0.898

4	(0.106) 0.102 0.102

Locus 3220 :

2 alleles

99.2% missing data

3	(0.329) 0.327 0.342

1	(0.671) 0.673 0.658

Locus 3221 :

2 alleles

96.9% missing data

2	(0.781) 0.795 0.772

3	(0.219) 0.205 0.228

Locus 3222 :

2 alleles

99.2% missing data

1	(0.689) 0.684 0.687

3	(0.311) 0.316 0.313

Locus 3223 :

2 alleles

90.6% missing data

1	(0.919) 0.934 0.910

4	(0.081) 0.066 0.090

Locus 3224 :

3 alleles

98.4% missing data

1	(0.472) 0.458 0.478

4	(0.191) 0.200 0.192

3	(0.336) 0.343 0.331

Locus 3225 :

2 alleles

3.5% missing data

1	(0.978) 0.982 0.981

4	(0.022) 0.018 0.019

Locus 3226 :

2 alleles

98.8% missing data

2	(0.242) 0.244 0.246

3	(0.758) 0.756 0.754

Locus 3227 :

2 alleles

91.0% missing data

3	(0.940) 0.947 0.938

4	(0.060) 0.053 0.062

Locus 3228 :

3 alleles

96.1% missing data

4	(0.188) 0.178 0.189

1	(0.559) 0.582 0.556

2	(0.253) 0.240 0.254

Locus 3229 :

2 alleles

99.2% missing data

3	(0.658) 0.664 0.645

4	(0.342) 0.336 0.355

Locus 3230 :

2 alleles

93.0% missing data

4	(0.888) 0.892 0.888

1	(0.112) 0.108 0.112

Locus 3231 :

2 alleles

97.3% missing data

4	(0.225) 0.225 0.223

1	(0.775) 0.775 0.777

Locus 3232 :

2 alleles

98.4% missing data

3	(0.467) 0.458 0.472

2	(0.533) 0.542 0.528

Locus 3233 :

2 alleles

98.8% missing data

1	(0.570) 0.564 0.574

4	(0.430) 0.436 0.426

Locus 3234 :

2 alleles

98.8% missing data

3	(0.638) 0.633 0.634

4	(0.362) 0.367 0.366

Locus 3235 :

2 alleles

99.2% missing data

1	(0.372) 0.376 0.371

4	(0.628) 0.624 0.629

Locus 3236 :

2 alleles

98.8% missing data

3	(0.617) 0.616 0.622

2	(0.383) 0.384 0.378

Locus 3237 :

2 alleles

97.3% missing data

1	(0.597) 0.607 0.593

3	(0.403) 0.393 0.407

Locus 3238 :

2 alleles

97.3% missing data

3	(0.705) 0.709 0.710

1	(0.295) 0.291 0.290

Locus 3239 :

2 alleles

98.0% missing data

4	(0.397) 0.403 0.387

1	(0.603) 0.597 0.613

Locus 3240 :

4 alleles

98.4% missing data

2	(0.158) 0.156 0.164

4	(0.220) 0.217 0.216

1	(0.272) 0.271 0.278

3	(0.349) 0.356 0.342

Locus 3241 :

2 alleles

98.8% missing data

2	(0.634) 0.643 0.628

3	(0.366) 0.357 0.372

Locus 3242 :

3 alleles

98.4% missing data

3	(0.365) 0.368 0.372

1	(0.447) 0.439 0.443

2	(0.189) 0.193 0.186

Locus 3243 :

2 alleles

98.4% missing data

4	(0.221) 0.224 0.223

2	(0.779) 0.776 0.777

Locus 3244 :

2 alleles

99.2% missing data

1	(0.455) 0.456 0.452

2	(0.545) 0.544 0.548

Locus 3245 :

2 alleles

99.2% missing data

4	(0.594) 0.588 0.596

2	(0.406) 0.412 0.404

Locus 3246 :

2 alleles

99.2% missing data

1	(0.248) 0.241 0.259

4	(0.752) 0.759 0.741

Locus 3247 :

2 alleles

99.2% missing data

4	(0.302) 0.302 0.304

2	(0.698) 0.698 0.696

Locus 3248 :

2 alleles

99.2% missing data

1	(0.753) 0.752 0.745

3	(0.247) 0.248 0.255

Locus 3249 :

3 alleles

98.8% missing data

4	(0.263) 0.263 0.263

2	(0.427) 0.429 0.425

1	(0.310) 0.308 0.312

Locus 3250 :

2 alleles

99.2% missing data

2	(0.399) 0.397 0.404

3	(0.601) 0.603 0.596

Locus 3251 :

2 alleles

96.5% missing data

4	(0.773) 0.776 0.775

2	(0.227) 0.224 0.225

Locus 3252 :

2 alleles

99.2% missing data

3	(0.367) 0.362 0.366

2	(0.633) 0.638 0.634

Locus 3253 :

2 alleles

96.1% missing data

4	(0.806) 0.820 0.798

3	(0.194) 0.180 0.202

Locus 3254 :

2 alleles

98.4% missing data

3	(0.603) 0.604 0.600

4	(0.397) 0.396 0.400

Locus 3255 :

2 alleles

99.2% missing data

1	(0.260) 0.257 0.261

2	(0.740) 0.743 0.739

Locus 3256 :

2 alleles

99.2% missing data

4	(0.208) 0.205 0.216

2	(0.792) 0.795 0.784

Locus 3257 :

2 alleles

99.2% missing data

2	(0.349) 0.348 0.352

1	(0.651) 0.652 0.648

Locus 3258 :

2 alleles

99.2% missing data

1	(0.630) 0.635 0.629

3	(0.370) 0.365 0.371

Locus 3259 :

2 alleles

99.2% missing data

1	(0.683) 0.676 0.686

3	(0.317) 0.324 0.314

Locus 3260 :

2 alleles

98.8% missing data

2	(0.476) 0.484 0.472

4	(0.524) 0.516 0.528

Locus 3261 :

2 alleles

99.2% missing data

3	(0.332) 0.325 0.338

4	(0.668) 0.675 0.662

Locus 3262 :

2 alleles

95.3% missing data

2	(0.849) 0.857 0.847

4	(0.151) 0.143 0.153

Locus 3263 :

2 alleles

99.2% missing data

1	(0.454) 0.450 0.460

2	(0.546) 0.550 0.540

Locus 3264 :

2 alleles

9.4% missing data

2	(0.986) 0.977 0.996

4	(0.014) 0.023 0.004

Locus 3265 :

2 alleles

98.8% missing data

4	(0.285) 0.283 0.284

2	(0.715) 0.717 0.716

Locus 3266 :

2 alleles

99.2% missing data

1	(0.435) 0.440 0.435

4	(0.565) 0.560 0.565

Locus 3267 :

2 alleles

99.2% missing data

4	(0.276) 0.280 0.280

3	(0.724) 0.720 0.720

Locus 3268 :

2 alleles

99.2% missing data

1	(0.629) 0.622 0.633

2	(0.371) 0.378 0.367

Locus 3269 :

2 alleles

99.2% missing data

4	(0.788) 0.786 0.793

1	(0.212) 0.214 0.207

Locus 3270 :

2 alleles

99.2% missing data

2	(0.434) 0.430 0.437

3	(0.566) 0.570 0.563

Locus 3271 :

2 alleles

98.8% missing data

1	(0.471) 0.461 0.476

2	(0.529) 0.539 0.524

Locus 3272 :

2 alleles

99.2% missing data

4	(0.340) 0.342 0.339

3	(0.660) 0.658 0.661

Locus 3273 :

2 alleles

97.7% missing data

3	(0.717) 0.711 0.734

2	(0.283) 0.289 0.266

Locus 3274 :

2 alleles

90.2% missing data

1	(0.865) 0.889 0.839

2	(0.135) 0.111 0.161

Locus 3275 :

2 alleles

8.2% missing data

1	(0.973) 0.950 0.991

4	(0.027) 0.050 0.009

Locus 3276 :

2 alleles

99.2% missing data

3	(0.705) 0.699 0.707

1	(0.295) 0.301 0.293

Locus 3277 :

2 alleles

99.2% missing data

3	(0.426) 0.424 0.424

2	(0.574) 0.576 0.576

Locus 3278 :

2 alleles

99.2% missing data

3	(0.505) 0.499 0.509

4	(0.495) 0.501 0.491

Locus 3279 :

2 alleles

97.7% missing data

2	(0.295) 0.293 0.289

4	(0.705) 0.707 0.711

Locus 3280 :

2 alleles

99.2% missing data

4	(0.516) 0.520 0.511

3	(0.484) 0.480 0.489

Locus 3281 :

2 alleles

90.6% missing data

3	(0.916) 0.924 0.911

2	(0.084) 0.076 0.089

Locus 3282 :

2 alleles

99.2% missing data

3	(0.512) 0.514 0.512

4	(0.488) 0.486 0.488

Locus 3283 :

2 alleles

98.8% missing data

2	(0.559) 0.545 0.564

1	(0.441) 0.455 0.436

Locus 3284 :

2 alleles

99.2% missing data

3	(0.610) 0.607 0.600

2	(0.390) 0.393 0.400

Locus 3285 :

2 alleles

18.0% missing data

4	(0.983) 0.975 0.994

1	(0.017) 0.025 0.006

Locus 3286 :

2 alleles

99.2% missing data

4	(0.692) 0.682 0.696

3	(0.308) 0.318 0.304

Locus 3287 :

2 alleles

96.1% missing data

3	(0.829) 0.831 0.832

4	(0.171) 0.169 0.168

Locus 3288 :

2 alleles

99.2% missing data

1	(0.527) 0.527 0.526

2	(0.473) 0.473 0.474

Locus 3289 :

2 alleles

99.2% missing data

1	(0.204) 0.209 0.210

3	(0.796) 0.791 0.790

Locus 3290 :

3 alleles

98.8% missing data

1	(0.406) 0.403 0.398

4	(0.339) 0.344 0.344

2	(0.255) 0.253 0.258

Locus 3291 :

2 alleles

99.2% missing data

1	(0.129) 0.132 0.129

3	(0.871) 0.868 0.871

Locus 3292 :

2 alleles

98.0% missing data

4	(0.868) 0.869 0.871

3	(0.132) 0.131 0.129

Locus 3293 :

2 alleles

99.2% missing data

4	(0.391) 0.395 0.388

2	(0.609) 0.605 0.612

Locus 3294 :

2 alleles

99.2% missing data

1	(0.397) 0.397 0.393

4	(0.603) 0.603 0.607

Locus 3295 :

2 alleles

99.2% missing data

3	(0.557) 0.554 0.555

1	(0.443) 0.446 0.445

Locus 3296 :

2 alleles

99.2% missing data

4	(0.321) 0.331 0.317

2	(0.679) 0.669 0.683

Locus 3297 :

2 alleles

91.0% missing data

1	(0.883) 0.902 0.872

4	(0.117) 0.098 0.128

Locus 3298 :

3 alleles

98.8% missing data

4	(0.258) 0.255 0.259

2	(0.253) 0.249 0.256

1	(0.488) 0.496 0.485

Locus 3299 :

2 alleles

99.2% missing data

3	(0.696) 0.690 0.695

4	(0.304) 0.310 0.305

Locus 3300 :

2 alleles

92.6% missing data

3	(0.667) 0.665 0.659

2	(0.333) 0.335 0.341

Locus 3301 :

2 alleles

9.4% missing data

3	(0.982) 0.982 0.991

2	(0.018) 0.018 0.009

Locus 3302 :

2 alleles

99.2% missing data

4	(0.400) 0.403 0.398

1	(0.600) 0.597 0.602

Locus 3303 :

3 alleles

98.8% missing data

2	(0.257) 0.257 0.258

3	(0.397) 0.399 0.397

4	(0.346) 0.343 0.345

Locus 3304 :

2 alleles

99.2% missing data

3	(0.364) 0.362 0.370

4	(0.636) 0.638 0.630

Locus 3305 :

2 alleles

96.9% missing data

1	(0.784) 0.799 0.773

4	(0.216) 0.201 0.227

Locus 3306 :

2 alleles

99.2% missing data

4	(0.383) 0.382 0.383

2	(0.617) 0.618 0.617

Locus 3307 :

2 alleles

99.2% missing data

1	(0.463) 0.460 0.464

2	(0.537) 0.540 0.536

Locus 3308 :

2 alleles

98.0% missing data

2	(0.692) 0.685 0.705

1	(0.308) 0.315 0.295

Locus 3309 :

2 alleles

99.2% missing data

2	(0.777) 0.772 0.781

4	(0.223) 0.228 0.219

Locus 3310 :

2 alleles

99.2% missing data

4	(0.714) 0.704 0.720

2	(0.286) 0.296 0.280

Locus 3311 :

3 alleles

98.4% missing data

2	(0.276) 0.271 0.287

4	(0.316) 0.310 0.318

1	(0.408) 0.419 0.395

Locus 3312 :

3 alleles

97.7% missing data

3	(0.371) 0.389 0.351

1	(0.332) 0.321 0.345

2	(0.298) 0.291 0.304

Locus 3313 :

2 alleles

98.0% missing data

1	(0.739) 0.741 0.732

3	(0.261) 0.259 0.268

Locus 3314 :

2 alleles

99.2% missing data

1	(0.235) 0.239 0.234

4	(0.765) 0.761 0.766

Locus 3315 :

2 alleles

98.4% missing data

3	(0.427) 0.424 0.432

2	(0.573) 0.576 0.568

Locus 3316 :

2 alleles

98.8% missing data

1	(0.600) 0.603 0.592

4	(0.400) 0.397 0.408

Locus 3317 :

2 alleles

98.4% missing data

1	(0.270) 0.269 0.267

4	(0.730) 0.731 0.733

Locus 3318 :

2 alleles

98.8% missing data

3	(0.487) 0.485 0.495

2	(0.513) 0.515 0.505

Locus 3319 :

2 alleles

98.8% missing data

4	(0.359) 0.356 0.367

2	(0.641) 0.644 0.633

Locus 3320 :

2 alleles

99.2% missing data

1	(0.641) 0.641 0.633

3	(0.359) 0.359 0.367

Locus 3321 :

2 alleles

99.2% missing data

1	(0.616) 0.612 0.610

3	(0.384) 0.388 0.390

Locus 3322 :

2 alleles

96.9% missing data

1	(0.836) 0.835 0.838

4	(0.164) 0.165 0.162

Locus 3323 :

2 alleles

96.1% missing data

3	(0.798) 0.809 0.794

2	(0.202) 0.191 0.206

Locus 3324 :

2 alleles

99.2% missing data

2	(0.399) 0.399 0.400

3	(0.601) 0.601 0.600

Locus 3325 :

2 alleles

98.8% missing data

1	(0.821) 0.823 0.822

2	(0.179) 0.177 0.178

Locus 3326 :

2 alleles

7.4% missing data

4	(0.985) 0.979 0.995

3	(0.015) 0.021 0.005

Locus 3327 :

2 alleles

94.5% missing data

1	(0.830) 0.832 0.838

2	(0.170) 0.168 0.162

Locus 3328 :

2 alleles

99.2% missing data

4	(0.367) 0.360 0.376

2	(0.633) 0.640 0.624

Locus 3329 :

2 alleles

99.2% missing data

1	(0.563) 0.562 0.566

3	(0.437) 0.438 0.434

Locus 3330 :

2 alleles

99.2% missing data

2	(0.508) 0.504 0.511

4	(0.492) 0.496 0.489

Locus 3331 :

3 alleles

97.3% missing data

4	(0.195) 0.199 0.187

2	(0.492) 0.485 0.513

1	(0.313) 0.316 0.300

Locus 3332 :

2 alleles

97.3% missing data

1	(0.862) 0.852 0.870

3	(0.138) 0.148 0.130

Locus 3333 :

2 alleles

97.7% missing data

2	(0.788) 0.791 0.790

3	(0.212) 0.209 0.210

Locus 3334 :

2 alleles

99.2% missing data

1	(0.711) 0.715 0.702

3	(0.289) 0.285 0.298

Locus 3335 :

2 alleles

91.0% missing data

3	(0.895) 0.912 0.884

4	(0.105) 0.088 0.116

Locus 3336 :

2 alleles

99.2% missing data

4	(0.543) 0.548 0.547

1	(0.457) 0.452 0.453

Locus 3337 :

2 alleles

98.8% missing data

1	(0.533) 0.531 0.534

2	(0.467) 0.469 0.466

Locus 3338 :

2 alleles

99.2% missing data

1	(0.375) 0.376 0.374

4	(0.625) 0.624 0.626

Locus 3339 :

2 alleles

98.8% missing data

2	(0.384) 0.394 0.375

1	(0.616) 0.606 0.625

Locus 3340 :

2 alleles

90.6% missing data

4	(0.904) 0.924 0.896

1	(0.096) 0.076 0.104

Locus 3341 :

2 alleles

98.0% missing data

3	(0.620) 0.621 0.630

2	(0.380) 0.379 0.370

Locus 3342 :

2 alleles

99.2% missing data

2	(0.445) 0.442 0.447

4	(0.555) 0.558 0.553

Locus 3343 :

2 alleles

98.4% missing data

2	(0.715) 0.713 0.717

4	(0.285) 0.287 0.283

Locus 3344 :

2 alleles

91.4% missing data

3	(0.753) 0.755 0.753

1	(0.247) 0.245 0.247

Locus 3345 :

2 alleles

99.2% missing data

3	(0.495) 0.490 0.499

1	(0.505) 0.510 0.501

Locus 3346 :

2 alleles

99.2% missing data

3	(0.577) 0.577 0.573

2	(0.423) 0.423 0.427

Locus 3347 :

2 alleles

97.3% missing data

2	(0.260) 0.241 0.271

4	(0.740) 0.759 0.729

Locus 3348 :

2 alleles

99.2% missing data

2	(0.696) 0.696 0.691

4	(0.304) 0.304 0.309

Locus 3349 :

2 alleles

90.6% missing data

2	(0.897) 0.918 0.889

4	(0.103) 0.082 0.111

Locus 3350 :

2 alleles

8.2% missing data

4	(0.989) 0.982 0.997

3	(0.011) 0.018 0.003

Locus 3351 :

2 alleles

99.2% missing data

3	(0.380) 0.383 0.378

1	(0.620) 0.617 0.622

Locus 3352 :

2 alleles

97.7% missing data

4	(0.704) 0.701 0.710

3	(0.296) 0.299 0.290

Locus 3353 :

2 alleles

98.4% missing data

3	(0.864) 0.861 0.864

2	(0.136) 0.139 0.136

Locus 3354 :

2 alleles

99.2% missing data

2	(0.289) 0.296 0.289

4	(0.711) 0.704 0.711

Locus 3355 :

2 alleles

97.3% missing data

3	(0.754) 0.752 0.755

1	(0.246) 0.248 0.245

Locus 3356 :

2 alleles

99.2% missing data

3	(0.620) 0.630 0.616

4	(0.380) 0.370 0.384

Locus 3357 :

2 alleles

99.2% missing data

3	(0.365) 0.368 0.365

2	(0.635) 0.632 0.635

Locus 3358 :

2 alleles

98.0% missing data

2	(0.698) 0.691 0.709

4	(0.302) 0.309 0.291

Locus 3359 :

2 alleles

99.2% missing data

2	(0.296) 0.289 0.299

4	(0.704) 0.711 0.701

Locus 3360 :

2 alleles

99.2% missing data

1	(0.582) 0.590 0.576

4	(0.418) 0.410 0.424

Locus 3361 :

2 alleles

99.2% missing data

4	(0.609) 0.603 0.610

1	(0.391) 0.397 0.390

Locus 3362 :

2 alleles

99.2% missing data

2	(0.611) 0.615 0.608

3	(0.389) 0.385 0.392

Locus 3363 :

2 alleles

99.2% missing data

1	(0.422) 0.414 0.432

2	(0.578) 0.586 0.568

Locus 3364 :

2 alleles

99.2% missing data

1	(0.565) 0.562 0.566

3	(0.435) 0.438 0.434

Locus 3365 :

2 alleles

99.2% missing data

2	(0.449) 0.448 0.451

4	(0.551) 0.552 0.549

Locus 3366 :

2 alleles

98.8% missing data

2	(0.685) 0.687 0.691

1	(0.315) 0.313 0.309

Locus 3367 :

2 alleles

99.2% missing data

1	(0.707) 0.712 0.700

3	(0.293) 0.288 0.300

Locus 3368 :

2 alleles

99.2% missing data

4	(0.358) 0.358 0.362

2	(0.642) 0.642 0.638

Locus 3369 :

2 alleles

99.2% missing data

2	(0.242) 0.240 0.242

3	(0.758) 0.760 0.758

Locus 3370 :

2 alleles

99.2% missing data

4	(0.691) 0.692 0.689

1	(0.309) 0.308 0.311

Locus 3371 :

2 alleles

99.2% missing data

4	(0.385) 0.392 0.380

2	(0.615) 0.608 0.620

Locus 3372 :

2 alleles

98.8% missing data

4	(0.688) 0.678 0.694

2	(0.312) 0.322 0.306

Locus 3373 :

2 alleles

98.8% missing data

1	(0.780) 0.783 0.773

2	(0.220) 0.217 0.227

Locus 3374 :

2 alleles

99.2% missing data

4	(0.765) 0.763 0.768

1	(0.235) 0.237 0.232

Locus 3375 :

2 alleles

98.0% missing data

1	(0.876) 0.874 0.880

2	(0.124) 0.126 0.120

Locus 3376 :

2 alleles

99.2% missing data

2	(0.547) 0.551 0.545

1	(0.453) 0.449 0.455

Locus 3377 :

3 alleles

98.4% missing data

2	(0.374) 0.373 0.376

3	(0.362) 0.362 0.362

4	(0.264) 0.265 0.262

Locus 3378 :

2 alleles

98.8% missing data

3	(0.667) 0.666 0.670

2	(0.333) 0.334 0.330

Locus 3379 :

2 alleles

90.6% missing data

4	(0.113) 0.091 0.136

1	(0.887) 0.909 0.864

Locus 3380 :

2 alleles

99.2% missing data

2	(0.400) 0.399 0.405

1	(0.600) 0.601 0.595

Locus 3381 :

2 alleles

99.2% missing data

1	(0.475) 0.474 0.480

3	(0.525) 0.526 0.520

Locus 3382 :

2 alleles

99.2% missing data

4	(0.534) 0.534 0.532

3	(0.466) 0.466 0.468

Locus 3383 :

2 alleles

99.2% missing data

1	(0.631) 0.621 0.638

4	(0.369) 0.379 0.362

Locus 3384 :

2 alleles

98.8% missing data

1	(0.328) 0.323 0.332

4	(0.672) 0.677 0.668

Locus 3385 :

2 alleles

99.2% missing data

4	(0.410) 0.408 0.415

2	(0.590) 0.592 0.585

Locus 3386 :

2 alleles

99.2% missing data

4	(0.570) 0.569 0.572

3	(0.430) 0.431 0.428

Locus 3387 :

2 alleles

99.2% missing data

4	(0.538) 0.531 0.544

3	(0.462) 0.469 0.456

Locus 3388 :

2 alleles

99.2% missing data

3	(0.442) 0.442 0.445

1	(0.558) 0.558 0.555

Locus 3389 :

2 alleles

99.2% missing data

4	(0.462) 0.463 0.462

1	(0.538) 0.537 0.538

Locus 3390 :

2 alleles

91.0% missing data

3	(0.908) 0.922 0.903

4	(0.092) 0.078 0.097

Locus 3391 :

2 alleles

99.2% missing data

4	(0.709) 0.702 0.715

2	(0.291) 0.298 0.285

Locus 3392 :

2 alleles

99.2% missing data

1	(0.635) 0.636 0.637

3	(0.365) 0.364 0.363

Locus 3393 :

2 alleles

99.2% missing data

3	(0.396) 0.406 0.394

4	(0.604) 0.594 0.606

Locus 3394 :

2 alleles

98.4% missing data

3	(0.706) 0.718 0.701

4	(0.294) 0.282 0.299

Locus 3395 :

3 alleles

94.9% missing data

3	(0.582) 0.582 0.615

2	(0.102) 0.101 0.094

1	(0.316) 0.317 0.290

Locus 3396 :

2 alleles

99.2% missing data

2	(0.533) 0.527 0.536

4	(0.467) 0.473 0.464

Locus 3397 :

2 alleles

99.2% missing data

1	(0.449) 0.455 0.453

3	(0.551) 0.545 0.547

Locus 3398 :

2 alleles

96.9% missing data

4	(0.607) 0.610 0.616

3	(0.393) 0.390 0.384

Locus 3399 :

2 alleles

99.2% missing data

2	(0.643) 0.644 0.640

3	(0.357) 0.356 0.360

Locus 3400 :

2 alleles

99.2% missing data

4	(0.696) 0.688 0.708

3	(0.304) 0.312 0.292

Locus 3401 :

2 alleles

99.2% missing data

2	(0.395) 0.393 0.401

3	(0.605) 0.607 0.599

Locus 3402 :

2 alleles

99.2% missing data

2	(0.703) 0.696 0.712

1	(0.297) 0.304 0.288

Locus 3403 :

2 alleles

99.2% missing data

2	(0.377) 0.381 0.379

3	(0.623) 0.619 0.621

Locus 3404 :

2 alleles

99.2% missing data

1	(0.616) 0.612 0.629

3	(0.384) 0.388 0.371

Locus 3405 :

2 alleles

99.2% missing data

2	(0.412) 0.410 0.417

3	(0.588) 0.590 0.583

Locus 3406 :

2 alleles

98.8% missing data

4	(0.824) 0.818 0.829

3	(0.176) 0.182 0.171

Locus 3407 :

2 alleles

99.2% missing data

4	(0.631) 0.625 0.637

3	(0.369) 0.375 0.363

Locus 3408 :

2 alleles

96.5% missing data

4	(0.858) 0.855 0.861

3	(0.142) 0.145 0.139

Locus 3409 :

2 alleles

99.2% missing data

3	(0.446) 0.441 0.451

2	(0.554) 0.559 0.549

Locus 3410 :

2 alleles

99.2% missing data

4	(0.625) 0.625 0.623

1	(0.375) 0.375 0.377

Locus 3411 :

2 alleles

99.2% missing data

3	(0.625) 0.614 0.633

4	(0.375) 0.386 0.367

Locus 3412 :

3 alleles

98.8% missing data

2	(0.596) 0.594 0.597

4	(0.241) 0.237 0.246

3	(0.163) 0.168 0.157

Locus 3413 :

2 alleles

99.2% missing data

3	(0.622) 0.615 0.625

4	(0.378) 0.385 0.375

Locus 3414 :

2 alleles

90.6% missing data

3	(0.859) 0.873 0.856

4	(0.141) 0.127 0.144

Locus 3415 :

2 alleles

98.8% missing data

3	(0.469) 0.458 0.472

4	(0.531) 0.542 0.528

Locus 3416 :

3 alleles

98.8% missing data

2	(0.188) 0.181 0.192

1	(0.355) 0.358 0.358

3	(0.457) 0.461 0.451

Locus 3417 :

2 alleles

99.2% missing data

1	(0.298) 0.297 0.300

3	(0.702) 0.703 0.700

Locus 3418 :

2 alleles

99.2% missing data

4	(0.570) 0.561 0.572

3	(0.430) 0.439 0.428

Locus 3419 :

2 alleles

99.2% missing data

1	(0.283) 0.276 0.287

4	(0.717) 0.724 0.713

Locus 3420 :

2 alleles

96.1% missing data

3	(0.141) 0.136 0.139

4	(0.859) 0.864 0.861

Locus 3421 :

2 alleles

99.2% missing data

1	(0.616) 0.618 0.612

3	(0.384) 0.382 0.388

Locus 3422 :

2 alleles

0.0% missing data

4	(0.986) 0.981 0.996

1	(0.014) 0.019 0.004

Locus 3423 :

2 alleles

99.2% missing data

1	(0.653) 0.654 0.653

4	(0.347) 0.346 0.347

Locus 3424 :

2 alleles

99.2% missing data

2	(0.483) 0.486 0.477

4	(0.517) 0.514 0.523

Locus 3425 :

2 alleles

99.2% missing data

4	(0.572) 0.572 0.577

3	(0.428) 0.428 0.423

Locus 3426 :

2 alleles

98.8% missing data

3	(0.635) 0.629 0.641

2	(0.365) 0.371 0.359

Locus 3427 :

2 alleles

99.2% missing data

2	(0.404) 0.400 0.402

3	(0.596) 0.600 0.598

Locus 3428 :

2 alleles

99.2% missing data

2	(0.464) 0.458 0.466

1	(0.536) 0.542 0.534

Locus 3429 :

2 alleles

99.2% missing data

4	(0.181) 0.178 0.187

1	(0.819) 0.822 0.813

Locus 3430 :

2 alleles

99.2% missing data

2	(0.616) 0.619 0.613

4	(0.384) 0.381 0.387

Locus 3431 :

2 alleles

99.2% missing data

1	(0.407) 0.399 0.413

4	(0.593) 0.601 0.587

Locus 3432 :

2 alleles

99.2% missing data

2	(0.837) 0.843 0.828

4	(0.163) 0.157 0.172

Locus 3433 :

2 alleles

99.2% missing data

3	(0.232) 0.232 0.242

2	(0.768) 0.768 0.758

Locus 3434 :

2 alleles

99.2% missing data

1	(0.523) 0.517 0.520

4	(0.477) 0.483 0.480

Locus 3435 :

2 alleles

97.7% missing data

4	(0.341) 0.336 0.331

2	(0.659) 0.664 0.669

Locus 3436 :

2 alleles

99.2% missing data

4	(0.810) 0.807 0.812

1	(0.190) 0.193 0.188

Locus 3437 :

2 alleles

99.2% missing data

4	(0.451) 0.450 0.447

3	(0.549) 0.550 0.553

Locus 3438 :

2 alleles

99.2% missing data

2	(0.300) 0.298 0.305

4	(0.700) 0.702 0.695

Locus 3439 :

2 alleles

99.2% missing data

4	(0.353) 0.353 0.352

1	(0.647) 0.647 0.648

Locus 3440 :

2 alleles

99.2% missing data

4	(0.564) 0.568 0.552

2	(0.436) 0.432 0.448

Locus 3441 :

2 alleles

99.2% missing data

1	(0.563) 0.568 0.557

4	(0.437) 0.432 0.443

Locus 3442 :

2 alleles

99.2% missing data

1	(0.551) 0.559 0.550

2	(0.449) 0.441 0.450

Locus 3443 :

2 alleles

99.2% missing data

4	(0.369) 0.371 0.368

3	(0.631) 0.629 0.632

Locus 3444 :

2 alleles

99.2% missing data

2	(0.283) 0.283 0.288

3	(0.717) 0.717 0.712

Locus 3445 :

2 alleles

98.4% missing data

3	(0.361) 0.360 0.368

1	(0.639) 0.640 0.632

Locus 3446 :

2 alleles

98.0% missing data

3	(0.391) 0.382 0.391

1	(0.609) 0.618 0.609

Locus 3447 :

2 alleles

97.7% missing data

3	(0.689) 0.697 0.688

4	(0.311) 0.303 0.312

Locus 3448 :

2 alleles

99.2% missing data

4	(0.646) 0.641 0.652

1	(0.354) 0.359 0.348

Locus 3449 :

2 alleles

99.2% missing data

1	(0.492) 0.494 0.495

4	(0.508) 0.506 0.505

Locus 3450 :

2 alleles

99.2% missing data

3	(0.746) 0.749 0.743

4	(0.254) 0.251 0.257

Locus 3451 :

2 alleles

99.2% missing data

2	(0.281) 0.285 0.283

3	(0.719) 0.715 0.717

Locus 3452 :

2 alleles

99.2% missing data

1	(0.645) 0.637 0.652

4	(0.355) 0.363 0.348

Locus 3453 :

2 alleles

98.0% missing data

2	(0.780) 0.789 0.777

3	(0.220) 0.211 0.223

Locus 3454 :

3 alleles

98.0% missing data

3	(0.429) 0.443 0.423

4	(0.327) 0.318 0.328

2	(0.244) 0.239 0.249

Locus 3455 :

2 alleles

99.2% missing data

2	(0.567) 0.568 0.563

4	(0.433) 0.432 0.437

Locus 3456 :

2 alleles

99.2% missing data

4	(0.833) 0.826 0.837

2	(0.167) 0.174 0.163

Locus 3457 :

3 alleles

96.5% missing data

4	(0.276) 0.279 0.265

3	(0.596) 0.588 0.620

2	(0.128) 0.133 0.115

Locus 3458 :

2 alleles

99.2% missing data

4	(0.687) 0.684 0.690

1	(0.313) 0.316 0.310

Locus 3459 :

2 alleles

99.2% missing data

3	(0.250) 0.250 0.257

1	(0.750) 0.750 0.743

Locus 3460 :

2 alleles

8.2% missing data

3	(0.986) 0.981 0.996

4	(0.014) 0.019 0.004

Locus 3461 :

2 alleles

99.2% missing data

3	(0.547) 0.533 0.542

1	(0.453) 0.467 0.458

Locus 3462 :

3 alleles

96.9% missing data

3	(0.192) 0.190 0.189

4	(0.169) 0.168 0.166

2	(0.639) 0.641 0.645

Locus 3463 :

2 alleles

98.8% missing data

1	(0.401) 0.409 0.391

2	(0.599) 0.591 0.609

Locus 3464 :

2 alleles

98.8% missing data

4	(0.668) 0.664 0.676

3	(0.332) 0.336 0.324

Locus 3465 :

2 alleles

99.2% missing data

2	(0.346) 0.346 0.346

4	(0.654) 0.654 0.654

Locus 3466 :

2 alleles

99.2% missing data

4	(0.794) 0.792 0.792

3	(0.206) 0.208 0.208

Locus 3467 :

2 alleles

91.0% missing data

3	(0.928) 0.940 0.921

2	(0.072) 0.060 0.079

Locus 3468 :

2 alleles

87.5% missing data

3	(0.673) 0.731 0.617

1	(0.327) 0.269 0.383

Locus 3469 :

2 alleles

96.1% missing data

2	(0.750) 0.766 0.746

1	(0.250) 0.234 0.254

Locus 3470 :

2 alleles

98.8% missing data

1	(0.750) 0.748 0.750

3	(0.250) 0.252 0.250

Locus 3471 :

2 alleles

98.8% missing data

2	(0.457) 0.451 0.463

3	(0.543) 0.549 0.537

Locus 3472 :

2 alleles

95.3% missing data

1	(0.403) 0.401 0.405

2	(0.597) 0.599 0.595

Locus 3473 :

2 alleles

96.9% missing data

3	(0.782) 0.783 0.789

2	(0.218) 0.217 0.211

Locus 3474 :

2 alleles

98.4% missing data

4	(0.881) 0.872 0.887

1	(0.119) 0.128 0.113

Locus 3475 :

2 alleles

99.2% missing data

3	(0.319) 0.311 0.322

1	(0.681) 0.689 0.678

Locus 3476 :

2 alleles

99.2% missing data

3	(0.458) 0.456 0.453

2	(0.542) 0.544 0.547

Locus 3477 :

2 alleles

99.2% missing data

4	(0.415) 0.418 0.417

1	(0.585) 0.582 0.583

Locus 3478 :

2 alleles

99.2% missing data

3	(0.282) 0.282 0.286

1	(0.718) 0.718 0.714

Locus 3479 :

2 alleles

99.2% missing data

3	(0.469) 0.468 0.472

1	(0.531) 0.532 0.528

Locus 3480 :

2 alleles

99.2% missing data

2	(0.689) 0.680 0.694

1	(0.311) 0.320 0.306

Locus 3481 :

2 alleles

99.2% missing data

4	(0.549) 0.539 0.551

2	(0.451) 0.461 0.449

Locus 3482 :

2 alleles

98.8% missing data

2	(0.290) 0.299 0.286

1	(0.710) 0.701 0.714

Locus 3483 :

2 alleles

99.2% missing data

3	(0.335) 0.337 0.335

4	(0.665) 0.663 0.665

Locus 3484 :

2 alleles

99.2% missing data

2	(0.534) 0.538 0.536

3	(0.466) 0.462 0.464

Locus 3485 :

2 alleles

99.2% missing data

3	(0.528) 0.528 0.522

2	(0.472) 0.472 0.478

Locus 3486 :

2 alleles

98.8% missing data

1	(0.200) 0.195 0.207

2	(0.800) 0.805 0.793

Locus 3487 :

2 alleles

8.6% missing data

1	(0.984) 0.976 0.995

2	(0.016) 0.024 0.005

Locus 3488 :

2 alleles

99.2% missing data

4	(0.556) 0.552 0.561

3	(0.444) 0.448 0.439

Locus 3489 :

3 alleles

98.8% missing data

1	(0.414) 0.415 0.414

4	(0.123) 0.126 0.119

2	(0.463) 0.458 0.467

Locus 3490 :

2 alleles

98.8% missing data

2	(0.741) 0.736 0.740

4	(0.259) 0.264 0.260

Locus 3491 :

2 alleles

99.2% missing data

4	(0.620) 0.613 0.625

2	(0.380) 0.387 0.375

Locus 3492 :

2 alleles

99.2% missing data

2	(0.400) 0.398 0.403

4	(0.600) 0.602 0.597

Locus 3493 :

2 alleles

0.0% missing data

2	(0.984) 0.987 0.991

3	(0.016) 0.013 0.009

Locus 3494 :

2 alleles

98.8% missing data

2	(0.472) 0.464 0.488

3	(0.528) 0.536 0.512

Locus 3495 :

2 alleles

99.2% missing data

4	(0.204) 0.197 0.213

3	(0.796) 0.803 0.787

Locus 3496 :

2 alleles

99.2% missing data

4	(0.632) 0.632 0.629

1	(0.368) 0.368 0.371

Locus 3497 :

2 alleles

99.2% missing data

2	(0.698) 0.694 0.707

1	(0.302) 0.306 0.293

Locus 3498 :

2 alleles

99.2% missing data

3	(0.632) 0.629 0.626

1	(0.368) 0.371 0.374

Locus 3499 :

2 alleles

98.4% missing data

3	(0.637) 0.626 0.652

1	(0.363) 0.374 0.348

Locus 3500 :

2 alleles

98.0% missing data

1	(0.701) 0.703 0.697

4	(0.299) 0.297 0.303

Locus 3501 :

2 alleles

99.2% missing data

4	(0.505) 0.503 0.508

3	(0.495) 0.497 0.492

Locus 3502 :

2 alleles

99.2% missing data

4	(0.642) 0.642 0.640

2	(0.358) 0.358 0.360

Locus 3503 :

2 alleles

99.2% missing data

4	(0.318) 0.314 0.326

1	(0.682) 0.686 0.674

Locus 3504 :

2 alleles

99.2% missing data

3	(0.625) 0.623 0.625

2	(0.375) 0.377 0.375

Locus 3505 :

2 alleles

99.2% missing data

1	(0.618) 0.619 0.609

3	(0.382) 0.381 0.391

Locus 3506 :

2 alleles

98.8% missing data

3	(0.748) 0.752 0.743

2	(0.252) 0.248 0.257

Locus 3507 :

2 alleles

99.2% missing data

3	(0.347) 0.355 0.352

4	(0.653) 0.645 0.648

Locus 3508 :

2 alleles

98.8% missing data

3	(0.633) 0.638 0.629

4	(0.367) 0.362 0.371

Locus 3509 :

2 alleles

99.2% missing data

2	(0.500) 0.496 0.504

3	(0.500) 0.504 0.496

Locus 3510 :

2 alleles

99.2% missing data

3	(0.159) 0.159 0.158

1	(0.841) 0.841 0.842

Locus 3511 :

3 alleles

95.7% missing data

2	(0.243) 0.224 0.246

1	(0.189) 0.189 0.190

4	(0.568) 0.587 0.564

Locus 3512 :

2 alleles

99.2% missing data

1	(0.548) 0.547 0.548

3	(0.452) 0.453 0.452

Locus 3513 :

2 alleles

99.2% missing data

4	(0.450) 0.454 0.449

3	(0.550) 0.546 0.551

Locus 3514 :

2 alleles

99.2% missing data

3	(0.402) 0.408 0.398

1	(0.598) 0.592 0.602

Locus 3515 :

2 alleles

97.7% missing data

3	(0.327) 0.333 0.319

2	(0.673) 0.667 0.681

Locus 3516 :

2 alleles

96.1% missing data

3	(0.180) 0.168 0.182

2	(0.820) 0.832 0.818

Locus 3517 :

2 alleles

99.2% missing data

4	(0.517) 0.515 0.522

2	(0.483) 0.485 0.478

Locus 3518 :

2 alleles

99.2% missing data

4	(0.327) 0.331 0.323

1	(0.673) 0.669 0.677

Locus 3519 :

2 alleles

99.2% missing data

4	(0.658) 0.661 0.654

3	(0.342) 0.339 0.346

Locus 3520 :

2 alleles

98.4% missing data

3	(0.353) 0.345 0.356

4	(0.647) 0.655 0.644

Locus 3521 :

2 alleles

99.2% missing data

2	(0.548) 0.546 0.549

1	(0.452) 0.454 0.451

Locus 3522 :

3 alleles

98.0% missing data

3	(0.269) 0.267 0.266

1	(0.500) 0.504 0.506

4	(0.231) 0.229 0.227

Locus 3523 :

2 alleles

96.1% missing data

3	(0.800) 0.814 0.798

2	(0.200) 0.186 0.202

Locus 3524 :

2 alleles

97.7% missing data

1	(0.605) 0.616 0.600

3	(0.395) 0.384 0.400

Locus 3525 :

2 alleles

98.8% missing data

3	(0.537) 0.534 0.537

1	(0.463) 0.466 0.463

Locus 3526 :

2 alleles

99.2% missing data

2	(0.570) 0.571 0.567

1	(0.430) 0.429 0.433

Locus 3527 :

2 alleles

99.2% missing data

3	(0.486) 0.479 0.491

4	(0.514) 0.521 0.509

Locus 3528 :

2 alleles

88.3% missing data

3	(0.739) 0.748 0.727

4	(0.261) 0.252 0.273

Locus 3529 :

2 alleles

99.2% missing data

2	(0.250) 0.248 0.254

3	(0.750) 0.752 0.746

Locus 3530 :

2 alleles

99.2% missing data

1	(0.157) 0.155 0.167

2	(0.843) 0.845 0.833

Locus 3531 :

2 alleles

99.2% missing data

1	(0.311) 0.313 0.314

3	(0.689) 0.687 0.686

Locus 3532 :

2 alleles

97.7% missing data

2	(0.706) 0.704 0.719

4	(0.294) 0.296 0.281

Locus 3533 :

2 alleles

99.2% missing data

4	(0.402) 0.406 0.396

2	(0.598) 0.594 0.604

Locus 3534 :

2 alleles

99.2% missing data

2	(0.494) 0.503 0.485

1	(0.506) 0.497 0.515

Locus 3535 :

2 alleles

98.0% missing data

2	(0.784) 0.776 0.787

3	(0.216) 0.224 0.213

Locus 3536 :

2 alleles

98.8% missing data

3	(0.481) 0.479 0.495

4	(0.519) 0.521 0.505

Locus 3537 :

2 alleles

88.3% missing data

3	(0.856) 0.878 0.838

1	(0.144) 0.122 0.162

Locus 3538 :

2 alleles

8.6% missing data

3	(0.982) 0.973 0.994

1	(0.018) 0.027 0.006

Locus 3539 :

2 alleles

99.2% missing data

4	(0.539) 0.541 0.538

3	(0.461) 0.459 0.462

Locus 3540 :

2 alleles

99.2% missing data

3	(0.344) 0.338 0.348

2	(0.656) 0.662 0.652

Locus 3541 :

2 alleles

9.0% missing data

2	(0.988) 0.981 0.996

3	(0.012) 0.019 0.004

Locus 3542 :

2 alleles

99.2% missing data

2	(0.464) 0.466 0.463

4	(0.536) 0.534 0.537

Locus 3543 :

2 alleles

0.4% missing data

2	(0.989) 0.985 0.997

1	(0.011) 0.015 0.003

Locus 3544 :

2 alleles

99.2% missing data

1	(0.215) 0.211 0.216

4	(0.785) 0.789 0.784

Locus 3545 :

2 alleles

99.2% missing data

4	(0.471) 0.465 0.474

1	(0.529) 0.535 0.526

Locus 3546 :

2 alleles

99.2% missing data

2	(0.559) 0.552 0.566

4	(0.441) 0.448 0.434

Locus 3547 :

2 alleles

99.2% missing data

2	(0.338) 0.345 0.334

3	(0.662) 0.655 0.666

Locus 3548 :

2 alleles

99.2% missing data

3	(0.592) 0.587 0.593

1	(0.408) 0.413 0.407

Locus 3549 :

2 alleles

99.2% missing data

4	(0.470) 0.475 0.465

2	(0.530) 0.525 0.535

Locus 3550 :

2 alleles

99.2% missing data

2	(0.743) 0.742 0.744

1	(0.257) 0.258 0.256

Locus 3551 :

2 alleles

98.8% missing data

4	(0.347) 0.334 0.348

2	(0.653) 0.666 0.652

Locus 3552 :

2 alleles

99.2% missing data

3	(0.652) 0.643 0.659

2	(0.348) 0.357 0.341

Locus 3553 :

2 alleles

99.2% missing data

2	(0.351) 0.349 0.346

1	(0.649) 0.651 0.654

Locus 3554 :

2 alleles

99.2% missing data

2	(0.539) 0.542 0.542

4	(0.461) 0.458 0.458

Locus 3555 :

2 alleles

99.2% missing data

3	(0.835) 0.841 0.834

1	(0.165) 0.159 0.166

Locus 3556 :

2 alleles

99.2% missing data

4	(0.227) 0.227 0.231

3	(0.773) 0.773 0.769

Locus 3557 :

2 alleles

8.6% missing data

4	(0.986) 0.981 0.996

3	(0.014) 0.019 0.004

Locus 3558 :

2 alleles

0.0% missing data

2	(0.923) 0.792 0.977

4	(0.077) 0.208 0.023

Locus 3559 :

2 alleles

99.2% missing data

1	(0.377) 0.372 0.387

4	(0.623) 0.628 0.613

Locus 3560 :

2 alleles

99.2% missing data

2	(0.593) 0.589 0.600

4	(0.407) 0.411 0.400

Locus 3561 :

2 alleles

97.7% missing data

2	(0.779) 0.781 0.779

1	(0.221) 0.219 0.221

Locus 3562 :

3 alleles

98.8% missing data

2	(0.388) 0.386 0.392

4	(0.310) 0.311 0.307

3	(0.302) 0.303 0.301

Locus 3563 :

2 alleles

99.2% missing data

1	(0.680) 0.676 0.678

2	(0.320) 0.324 0.322

Locus 3564 :

2 alleles

99.2% missing data

4	(0.310) 0.305 0.315

1	(0.690) 0.695 0.685

Locus 3565 :

2 alleles

98.0% missing data

2	(0.857) 0.853 0.858

1	(0.143) 0.147 0.142

Locus 3566 :

2 alleles

99.2% missing data

3	(0.437) 0.435 0.441

4	(0.563) 0.565 0.559

Locus 3567 :

2 alleles

99.2% missing data

1	(0.445) 0.448 0.446

2	(0.555) 0.552 0.554

Locus 3568 :

2 alleles

99.2% missing data

2	(0.415) 0.418 0.412

1	(0.585) 0.582 0.588

Locus 3569 :

2 alleles

99.2% missing data

2	(0.628) 0.625 0.630

3	(0.372) 0.375 0.370

Locus 3570 :

2 alleles

99.2% missing data

4	(0.207) 0.205 0.207

2	(0.793) 0.795 0.793

Locus 3571 :

3 alleles

98.8% missing data

4	(0.392) 0.390 0.391

2	(0.366) 0.358 0.369

3	(0.242) 0.253 0.240

Locus 3572 :

2 alleles

98.4% missing data

3	(0.775) 0.784 0.775

4	(0.225) 0.216 0.225

Locus 3573 :

2 alleles

99.2% missing data

1	(0.612) 0.609 0.614

2	(0.388) 0.391 0.386

Locus 3574 :

2 alleles

99.2% missing data

2	(0.576) 0.570 0.578

3	(0.424) 0.430 0.422

Locus 3575 :

2 alleles

99.2% missing data

3	(0.516) 0.516 0.516

4	(0.484) 0.484 0.484

Locus 3576 :

2 alleles

99.2% missing data

2	(0.617) 0.612 0.623

4	(0.383) 0.388 0.377

Locus 3577 :

2 alleles

96.9% missing data

2	(0.821) 0.821 0.822

3	(0.179) 0.179 0.178

Locus 3578 :

2 alleles

99.2% missing data

1	(0.538) 0.536 0.537

3	(0.462) 0.464 0.463

Locus 3579 :

2 alleles

98.4% missing data

2	(0.198) 0.195 0.199

1	(0.802) 0.805 0.801

Locus 3580 :

2 alleles

97.7% missing data

3	(0.866) 0.864 0.866

2	(0.134) 0.136 0.134

Locus 3581 :

3 alleles

88.3% missing data

2	(0.714) 0.735 0.697

3	(0.224) 0.215 0.234

4	(0.063) 0.050 0.069

Locus 3582 :

3 alleles

97.3% missing data

4	(0.351) 0.345 0.343

3	(0.414) 0.408 0.432

1	(0.235) 0.247 0.224

Locus 3583 :

3 alleles

98.8% missing data

3	(0.246) 0.247 0.245

1	(0.360) 0.355 0.366

2	(0.394) 0.398 0.388

Locus 3584 :

2 alleles

98.8% missing data

2	(0.690) 0.696 0.688

3	(0.310) 0.304 0.312

Locus 3585 :

2 alleles

99.2% missing data

2	(0.489) 0.497 0.483

3	(0.511) 0.503 0.517

Locus 3586 :

2 alleles

99.2% missing data

3	(0.566) 0.570 0.559

4	(0.434) 0.430 0.441

Locus 3587 :

2 alleles

93.0% missing data

3	(0.882) 0.882 0.893

1	(0.118) 0.118 0.107

Locus 3588 :

3 alleles

98.8% missing data

3	(0.225) 0.220 0.227

4	(0.578) 0.588 0.569

1	(0.197) 0.191 0.203

Locus 3589 :

2 alleles

99.2% missing data

1	(0.329) 0.328 0.329

2	(0.671) 0.672 0.671

Locus 3590 :

2 alleles

99.2% missing data

2	(0.490) 0.493 0.481

3	(0.510) 0.507 0.519

Locus 3591 :

2 alleles

99.2% missing data

3	(0.266) 0.258 0.274

1	(0.734) 0.742 0.726

Locus 3592 :

2 alleles

96.1% missing data

1	(0.137) 0.133 0.134

2	(0.863) 0.867 0.866

Locus 3593 :

2 alleles

99.2% missing data

4	(0.692) 0.697 0.692

3	(0.308) 0.303 0.308

Locus 3594 :

2 alleles

99.2% missing data

1	(0.249) 0.249 0.251

3	(0.751) 0.751 0.749

Locus 3595 :

2 alleles

99.2% missing data

4	(0.577) 0.579 0.580

2	(0.423) 0.421 0.420

Locus 3596 :

2 alleles

98.8% missing data

2	(0.480) 0.478 0.487

1	(0.520) 0.522 0.513

Locus 3597 :

3 alleles

98.8% missing data

2	(0.248) 0.241 0.253

3	(0.367) 0.372 0.373

1	(0.385) 0.387 0.373

Locus 3598 :

2 alleles

99.2% missing data

1	(0.578) 0.573 0.584

3	(0.422) 0.427 0.416

Locus 3599 :

2 alleles

97.7% missing data

1	(0.249) 0.244 0.243

2	(0.751) 0.756 0.757

Locus 3600 :

2 alleles

99.2% missing data

3	(0.307) 0.309 0.308

2	(0.693) 0.691 0.692

Locus 3601 :

2 alleles

99.2% missing data

3	(0.795) 0.791 0.796

4	(0.205) 0.209 0.204

Locus 3602 :

2 alleles

98.0% missing data

1	(0.753) 0.746 0.761

4	(0.247) 0.254 0.239

Locus 3603 :

2 alleles

99.2% missing data

3	(0.726) 0.724 0.726

2	(0.274) 0.276 0.274

Locus 3604 :

2 alleles

89.8% missing data

3	(0.884) 0.891 0.887

4	(0.116) 0.109 0.113

Locus 3605 :

2 alleles

98.8% missing data

3	(0.314) 0.309 0.321

2	(0.686) 0.691 0.679

Locus 3606 :

2 alleles

99.2% missing data

3	(0.453) 0.454 0.453

2	(0.547) 0.546 0.547

Locus 3607 :

2 alleles

99.2% missing data

2	(0.460) 0.459 0.457

4	(0.540) 0.541 0.543

Locus 3608 :

2 alleles

98.8% missing data

1	(0.711) 0.707 0.719

4	(0.289) 0.293 0.281

Locus 3609 :

2 alleles

96.5% missing data

3	(0.119) 0.117 0.121

4	(0.881) 0.883 0.879

Locus 3610 :

2 alleles

99.2% missing data

3	(0.516) 0.513 0.519

4	(0.484) 0.487 0.481

Locus 3611 :

2 alleles

98.4% missing data

3	(0.778) 0.784 0.776

2	(0.222) 0.216 0.224

Locus 3612 :

2 alleles

96.1% missing data

1	(0.875) 0.887 0.868

4	(0.125) 0.113 0.132

Locus 3613 :

2 alleles

98.8% missing data

4	(0.575) 0.571 0.576

3	(0.425) 0.429 0.424

Locus 3614 :

2 alleles

99.2% missing data

2	(0.613) 0.619 0.608

3	(0.387) 0.381 0.392

Locus 3615 :

2 alleles

99.2% missing data

4	(0.554) 0.556 0.550

2	(0.446) 0.444 0.450

Locus 3616 :

3 alleles

95.7% missing data

1	(0.607) 0.617 0.611

3	(0.195) 0.183 0.200

4	(0.198) 0.199 0.190

Locus 3617 :

2 alleles

99.2% missing data

2	(0.414) 0.409 0.417

1	(0.586) 0.591 0.583

Locus 3618 :

2 alleles

99.2% missing data

2	(0.431) 0.433 0.425

1	(0.569) 0.567 0.575

Locus 3619 :

2 alleles

92.6% missing data

1	(0.893) 0.891 0.903

3	(0.107) 0.109 0.097

Locus 3620 :

2 alleles

99.2% missing data

3	(0.598) 0.607 0.589

4	(0.402) 0.393 0.411

Locus 3621 :

2 alleles

99.2% missing data

3	(0.368) 0.369 0.370

1	(0.632) 0.631 0.630

Locus 3622 :

2 alleles

91.8% missing data

3	(0.936) 0.933 0.945

4	(0.064) 0.067 0.055

Locus 3623 :

2 alleles

99.2% missing data

4	(0.594) 0.598 0.595

2	(0.406) 0.402 0.405

Locus 3624 :

2 alleles

99.2% missing data

2	(0.421) 0.416 0.420

1	(0.579) 0.584 0.580

Locus 3625 :

2 alleles

99.2% missing data

4	(0.587) 0.587 0.582

1	(0.413) 0.413 0.418

Locus 3626 :

2 alleles

98.0% missing data

3	(0.681) 0.687 0.686

2	(0.319) 0.313 0.314

Locus 3627 :

2 alleles

99.2% missing data

4	(0.226) 0.228 0.229

2	(0.774) 0.772 0.771

Locus 3628 :

2 alleles

99.2% missing data

4	(0.272) 0.265 0.281

3	(0.728) 0.735 0.719

Locus 3629 :

2 alleles

99.2% missing data

2	(0.440) 0.435 0.443

1	(0.560) 0.565 0.557

Locus 3630 :

2 alleles

99.2% missing data

1	(0.663) 0.665 0.657

3	(0.337) 0.335 0.343

Locus 3631 :

2 alleles

99.2% missing data

1	(0.467) 0.461 0.472

3	(0.533) 0.539 0.528

Locus 3632 :

3 alleles

98.0% missing data

3	(0.263) 0.256 0.270

2	(0.285) 0.275 0.281

4	(0.452) 0.470 0.449

Locus 3633 :

2 alleles

96.5% missing data

4	(0.815) 0.824 0.807

3	(0.185) 0.176 0.193

Locus 3634 :

2 alleles

99.2% missing data

3	(0.374) 0.373 0.380

4	(0.626) 0.627 0.620

Locus 3635 :

2 alleles

99.2% missing data

4	(0.329) 0.324 0.338

2	(0.671) 0.676 0.662

Locus 3636 :

2 alleles

99.2% missing data

4	(0.625) 0.626 0.626

2	(0.375) 0.374 0.374

Locus 3637 :

2 alleles

90.6% missing data

4	(0.868) 0.893 0.861

2	(0.132) 0.107 0.139

Locus 3638 :

2 alleles

99.2% missing data

4	(0.357) 0.348 0.359

3	(0.643) 0.652 0.641

Locus 3639 :

2 alleles

99.2% missing data

2	(0.326) 0.317 0.330

4	(0.674) 0.683 0.670

Locus 3640 :

2 alleles

99.2% missing data

3	(0.602) 0.601 0.602

1	(0.398) 0.399 0.398

Locus 3641 :

2 alleles

98.8% missing data

4	(0.286) 0.282 0.290

1	(0.714) 0.718 0.710

Locus 3642 :

2 alleles

99.2% missing data

3	(0.623) 0.629 0.623

4	(0.377) 0.371 0.377

Locus 3643 :

2 alleles

90.2% missing data

4	(0.893) 0.912 0.887

3	(0.107) 0.088 0.113

Locus 3644 :

2 alleles

99.2% missing data

1	(0.519) 0.519 0.517

2	(0.481) 0.481 0.483

Locus 3645 :

2 alleles

99.2% missing data

4	(0.654) 0.656 0.647

2	(0.346) 0.344 0.353

Locus 3646 :

2 alleles

93.4% missing data

1	(0.676) 0.676 0.667

4	(0.324) 0.324 0.333

Locus 3647 :

2 alleles

99.2% missing data

1	(0.396) 0.398 0.400

3	(0.604) 0.602 0.600

Locus 3648 :

2 alleles

99.2% missing data

1	(0.532) 0.522 0.535

4	(0.468) 0.478 0.465

Locus 3649 :

2 alleles

96.5% missing data

3	(0.855) 0.855 0.856

2	(0.145) 0.145 0.144

Locus 3650 :

2 alleles

99.2% missing data

2	(0.490) 0.481 0.497

3	(0.510) 0.519 0.503

Locus 3651 :

2 alleles

99.2% missing data

2	(0.331) 0.329 0.334

3	(0.669) 0.671 0.666

Locus 3652 :

2 alleles

99.2% missing data

2	(0.234) 0.236 0.239

1	(0.766) 0.764 0.761

Locus 3653 :

2 alleles

99.2% missing data

3	(0.421) 0.416 0.421

1	(0.579) 0.584 0.579

Locus 3654 :

2 alleles

99.2% missing data

2	(0.469) 0.459 0.474

1	(0.531) 0.541 0.526

Locus 3655 :

3 alleles

98.0% missing data

3	(0.522) 0.531 0.519

4	(0.321) 0.304 0.324

2	(0.156) 0.164 0.157

Locus 3656 :

2 alleles

99.2% missing data

3	(0.370) 0.371 0.371

1	(0.630) 0.629 0.629

Locus 3657 :

2 alleles

96.1% missing data

2	(0.880) 0.877 0.886

1	(0.120) 0.123 0.114

Locus 3658 :

2 alleles

97.3% missing data

4	(0.804) 0.805 0.808

2	(0.196) 0.195 0.192

Locus 3659 :

2 alleles

96.5% missing data

4	(0.752) 0.744 0.764

2	(0.248) 0.256 0.236

Locus 3660 :

2 alleles

47.3% missing data

2	(0.977) 0.982 0.984

3	(0.023) 0.018 0.016

Locus 3661 :

2 alleles

0.4% missing data

1	(0.986) 0.990 0.992

4	(0.014) 0.010 0.008

Locus 3662 :

2 alleles

10.5% missing data

2	(0.986) 0.980 0.996

3	(0.014) 0.020 0.004

Locus 3663 :

2 alleles

98.8% missing data

2	(0.481) 0.483 0.490

3	(0.519) 0.517 0.510

Locus 3664 :

2 alleles

98.4% missing data

3	(0.691) 0.698 0.688

1	(0.309) 0.302 0.312

Locus 3665 :

3 alleles

98.4% missing data

1	(0.238) 0.242 0.234

4	(0.428) 0.422 0.426

3	(0.334) 0.335 0.340

Locus 3666 :

2 alleles

93.0% missing data

1	(0.922) 0.922 0.927

3	(0.078) 0.078 0.073

Locus 3667 :

2 alleles

99.2% missing data

4	(0.449) 0.445 0.454

2	(0.551) 0.555 0.546

Locus 3668 :

2 alleles

99.2% missing data

2	(0.617) 0.615 0.618

3	(0.383) 0.385 0.382

Locus 3669 :

2 alleles

99.2% missing data

4	(0.328) 0.323 0.335

2	(0.672) 0.677 0.665

Locus 3670 :

2 alleles

0.0% missing data

3	(0.983) 0.986 0.991

2	(0.017) 0.014 0.009

Locus 3671 :

2 alleles

99.2% missing data

4	(0.440) 0.438 0.442

2	(0.560) 0.562 0.558

Locus 3672 :

2 alleles

98.8% missing data

3	(0.579) 0.580 0.579

1	(0.421) 0.420 0.421

Locus 3673 :

2 alleles

91.0% missing data

4	(0.872) 0.896 0.860

1	(0.128) 0.104 0.140

Locus 3674 :

2 alleles

99.2% missing data

4	(0.561) 0.561 0.564

2	(0.439) 0.439 0.436

Locus 3675 :

2 alleles

98.4% missing data

3	(0.588) 0.601 0.579

2	(0.412) 0.399 0.421

Locus 3676 :

2 alleles

96.1% missing data

2	(0.804) 0.815 0.795

1	(0.196) 0.185 0.205

Locus 3677 :

2 alleles

98.8% missing data

4	(0.615) 0.618 0.615

1	(0.385) 0.382 0.385

Locus 3678 :

3 alleles

98.4% missing data

4	(0.252) 0.259 0.253

3	(0.552) 0.546 0.548

2	(0.196) 0.195 0.199

Locus 3679 :

2 alleles

99.2% missing data

4	(0.458) 0.456 0.463

2	(0.542) 0.544 0.537

Locus 3680 :

2 alleles

99.2% missing data

1	(0.689) 0.682 0.700

3	(0.311) 0.318 0.300

Locus 3681 :

2 alleles

99.2% missing data

1	(0.662) 0.658 0.668

3	(0.338) 0.342 0.332

Locus 3682 :

2 alleles

99.2% missing data

2	(0.262) 0.258 0.272

3	(0.738) 0.742 0.728

Locus 3683 :

3 alleles

98.8% missing data

2	(0.483) 0.492 0.482

1	(0.372) 0.369 0.370

3	(0.145) 0.139 0.148

Locus 3684 :

2 alleles

99.2% missing data

3	(0.520) 0.524 0.518

1	(0.480) 0.476 0.482

Locus 3685 :

2 alleles

93.8% missing data

2	(0.890) 0.892 0.894

3	(0.110) 0.108 0.106

Locus 3686 :

2 alleles

99.2% missing data

4	(0.591) 0.598 0.589

3	(0.409) 0.402 0.411

Locus 3687 :

2 alleles

98.8% missing data

1	(0.775) 0.778 0.772

3	(0.225) 0.222 0.228

Locus 3688 :

2 alleles

99.2% missing data

1	(0.359) 0.360 0.361

3	(0.641) 0.640 0.639

Locus 3689 :

2 alleles

99.2% missing data

1	(0.270) 0.271 0.279

3	(0.730) 0.729 0.721

Locus 3690 :

2 alleles

98.8% missing data

4	(0.451) 0.451 0.453

1	(0.549) 0.549 0.547

Locus 3691 :

2 alleles

99.2% missing data

3	(0.525) 0.524 0.526

4	(0.475) 0.476 0.474

Locus 3692 :

2 alleles

96.9% missing data

2	(0.832) 0.841 0.828

4	(0.168) 0.159 0.172

Locus 3693 :

2 alleles

99.2% missing data

3	(0.704) 0.693 0.710

2	(0.296) 0.307 0.290

Locus 3694 :

2 alleles

99.2% missing data

2	(0.535) 0.532 0.537

3	(0.465) 0.468 0.463

Locus 3695 :

2 alleles

96.9% missing data

4	(0.170) 0.156 0.180

1	(0.830) 0.844 0.820

Locus 3696 :

2 alleles

99.2% missing data

3	(0.646) 0.639 0.645

4	(0.354) 0.361 0.355

Locus 3697 :

2 alleles

99.2% missing data

3	(0.242) 0.241 0.246

2	(0.758) 0.759 0.754

Locus 3698 :

2 alleles

98.8% missing data

2	(0.518) 0.513 0.520

4	(0.482) 0.487 0.480

Locus 3699 :

2 alleles

99.2% missing data

3	(0.332) 0.330 0.330

2	(0.668) 0.670 0.670

Locus 3700 :

2 alleles

99.2% missing data

2	(0.442) 0.442 0.439

4	(0.558) 0.558 0.561

Locus 3701 :

2 alleles

9.0% missing data

4	(0.983) 0.987 0.988

1	(0.017) 0.013 0.012

Locus 3702 :

2 alleles

99.2% missing data

2	(0.447) 0.446 0.445

4	(0.553) 0.554 0.555

Locus 3703 :

2 alleles

96.1% missing data

1	(0.778) 0.792 0.771

3	(0.222) 0.208 0.229

Locus 3704 :

2 alleles

91.0% missing data

1	(0.924) 0.931 0.923

3	(0.076) 0.069 0.077

Locus 3705 :

2 alleles

99.2% missing data

4	(0.645) 0.644 0.644

2	(0.355) 0.356 0.356

Locus 3706 :

2 alleles

99.2% missing data

1	(0.414) 0.423 0.414

2	(0.586) 0.577 0.586

Locus 3707 :

2 alleles

0.0% missing data

2	(0.923) 0.803 0.977

3	(0.077) 0.197 0.023

Locus 3708 :

2 alleles

91.0% missing data

1	(0.916) 0.930 0.909

4	(0.084) 0.070 0.091

Locus 3709 :

2 alleles

98.0% missing data

2	(0.471) 0.457 0.479

4	(0.529) 0.543 0.521

Locus 3710 :

2 alleles

99.2% missing data

2	(0.477) 0.475 0.479

1	(0.523) 0.525 0.521

Locus 3711 :

2 alleles

99.2% missing data

2	(0.545) 0.543 0.554

4	(0.455) 0.457 0.446

Locus 3712 :

2 alleles

99.2% missing data

4	(0.758) 0.761 0.750

2	(0.242) 0.239 0.250

Locus 3713 :

2 alleles

91.0% missing data

2	(0.939) 0.949 0.931

3	(0.061) 0.051 0.069

Locus 3714 :

2 alleles

99.2% missing data

1	(0.569) 0.566 0.570

2	(0.431) 0.434 0.430

Locus 3715 :

2 alleles

91.0% missing data

2	(0.919) 0.932 0.911

3	(0.081) 0.068 0.089

Locus 3716 :

2 alleles

99.2% missing data

3	(0.379) 0.383 0.378

4	(0.621) 0.617 0.622

Locus 3717 :

2 alleles

98.8% missing data

1	(0.640) 0.632 0.649

4	(0.360) 0.368 0.351

Locus 3718 :

2 alleles

99.2% missing data

4	(0.595) 0.596 0.597

3	(0.405) 0.404 0.403

Locus 3719 :

2 alleles

99.2% missing data

1	(0.558) 0.549 0.562

4	(0.442) 0.451 0.438

Locus 3720 :

2 alleles

99.2% missing data

4	(0.412) 0.402 0.417

2	(0.588) 0.598 0.583

Locus 3721 :

2 alleles

97.3% missing data

1	(0.798) 0.799 0.806

2	(0.202) 0.201 0.194

Locus 3722 :

2 alleles

90.2% missing data

3	(0.913) 0.928 0.906

2	(0.087) 0.072 0.094

Locus 3723 :

2 alleles

94.5% missing data

2	(0.492) 0.525 0.462

3	(0.508) 0.475 0.538

Locus 3724 :

2 alleles

99.2% missing data

4	(0.752) 0.758 0.744

2	(0.248) 0.242 0.256

Locus 3725 :

2 alleles

98.8% missing data

3	(0.503) 0.498 0.512

2	(0.497) 0.502 0.488

Locus 3726 :

2 alleles

92.6% missing data

3	(0.896) 0.896 0.904

4	(0.104) 0.104 0.096

Locus 3727 :

2 alleles

95.3% missing data

2	(0.873) 0.884 0.866

3	(0.127) 0.116 0.134

Locus 3728 :

2 alleles

99.2% missing data

2	(0.111) 0.107 0.120

4	(0.889) 0.893 0.880

Locus 3729 :

2 alleles

99.2% missing data

2	(0.468) 0.471 0.476

3	(0.532) 0.529 0.524

Locus 3730 :

2 alleles

99.2% missing data

4	(0.461) 0.464 0.458

3	(0.539) 0.536 0.542

Locus 3731 :

2 alleles

94.9% missing data

4	(0.863) 0.866 0.871

2	(0.137) 0.134 0.129

Locus 3732 :

2 alleles

99.2% missing data

2	(0.537) 0.536 0.540

4	(0.463) 0.464 0.460

Locus 3733 :

2 alleles

99.2% missing data

2	(0.620) 0.622 0.617

3	(0.380) 0.378 0.383

Locus 3734 :

2 alleles

98.0% missing data

4	(0.223) 0.226 0.220

1	(0.777) 0.774 0.780

Locus 3735 :

2 alleles

98.8% missing data

3	(0.551) 0.559 0.545

1	(0.449) 0.441 0.455

Locus 3736 :

2 alleles

99.2% missing data

1	(0.173) 0.178 0.174

3	(0.827) 0.822 0.826

Locus 3737 :

2 alleles

99.2% missing data

2	(0.409) 0.397 0.418

3	(0.591) 0.603 0.582

Locus 3738 :

2 alleles

99.2% missing data

2	(0.262) 0.268 0.263

1	(0.738) 0.732 0.737

Locus 3739 :

2 alleles

99.2% missing data

1	(0.440) 0.443 0.444

2	(0.560) 0.557 0.556

Locus 3740 :

2 alleles

98.4% missing data

1	(0.604) 0.608 0.608

2	(0.396) 0.392 0.392

Locus 3741 :

2 alleles

99.2% missing data

3	(0.454) 0.448 0.454

1	(0.546) 0.552 0.546

Locus 3742 :

3 alleles

98.4% missing data

3	(0.301) 0.301 0.305

2	(0.319) 0.316 0.315

1	(0.380) 0.383 0.380

Locus 3743 :

2 alleles

99.2% missing data

1	(0.695) 0.692 0.699

3	(0.305) 0.308 0.301

Locus 3744 :

2 alleles

97.7% missing data

4	(0.817) 0.810 0.824

2	(0.183) 0.190 0.176

Locus 3745 :

2 alleles

99.2% missing data

2	(0.602) 0.611 0.596

3	(0.398) 0.389 0.404

Locus 3746 :

2 alleles

98.8% missing data

2	(0.506) 0.502 0.509

4	(0.494) 0.498 0.491

Locus 3747 :

2 alleles

98.4% missing data

3	(0.728) 0.739 0.723

2	(0.272) 0.261 0.277

Locus 3748 :

2 alleles

99.2% missing data

3	(0.349) 0.355 0.344

1	(0.651) 0.645 0.656

Locus 3749 :

2 alleles

98.4% missing data

2	(0.313) 0.312 0.311

1	(0.687) 0.688 0.689

Locus 3750 :

2 alleles

99.2% missing data

1	(0.356) 0.348 0.362

3	(0.644) 0.652 0.638

Locus 3751 :

2 alleles

99.2% missing data

2	(0.635) 0.632 0.635

1	(0.365) 0.368 0.365

Locus 3752 :

2 alleles

99.2% missing data

3	(0.529) 0.525 0.530

2	(0.471) 0.475 0.470

Locus 3753 :

3 alleles

96.5% missing data

3	(0.748) 0.746 0.755

4	(0.189) 0.191 0.182

1	(0.063) 0.063 0.063

Locus 3754 :

2 alleles

98.4% missing data

3	(0.674) 0.681 0.665

1	(0.326) 0.319 0.335

Locus 3755 :

2 alleles

99.2% missing data

3	(0.603) 0.604 0.592

1	(0.397) 0.396 0.408

Locus 3756 :

2 alleles

99.2% missing data

3	(0.460) 0.458 0.468

1	(0.540) 0.542 0.532

Locus 3757 :

2 alleles

99.2% missing data

2	(0.289) 0.285 0.296

4	(0.711) 0.715 0.704

Locus 3758 :

2 alleles

99.2% missing data

3	(0.396) 0.396 0.392

4	(0.604) 0.604 0.608

Locus 3759 :

2 alleles

98.8% missing data

4	(0.541) 0.537 0.552

1	(0.459) 0.463 0.448

Locus 3760 :

2 alleles

91.0% missing data

1	(0.906) 0.914 0.904

2	(0.094) 0.086 0.096

Locus 3761 :

2 alleles

0.0% missing data

1	(0.984) 0.979 0.996

3	(0.016) 0.021 0.004

Locus 3762 :

2 alleles

98.4% missing data

3	(0.262) 0.260 0.266

1	(0.738) 0.740 0.734

Locus 3763 :

2 alleles

99.2% missing data

2	(0.638) 0.638 0.635

1	(0.362) 0.362 0.365

Locus 3764 :

2 alleles

99.2% missing data

4	(0.425) 0.423 0.431

1	(0.575) 0.577 0.569

Locus 3765 :

2 alleles

98.8% missing data

2	(0.188) 0.189 0.182

3	(0.812) 0.811 0.818

Locus 3766 :

2 alleles

98.8% missing data

3	(0.634) 0.631 0.632

1	(0.366) 0.369 0.368

Locus 3767 :

2 alleles

99.2% missing data

3	(0.654) 0.646 0.657

4	(0.346) 0.354 0.343

Locus 3768 :

2 alleles

98.4% missing data

1	(0.653) 0.651 0.652

2	(0.347) 0.349 0.348

Locus 3769 :

2 alleles

99.2% missing data

3	(0.686) 0.684 0.681

4	(0.314) 0.316 0.319

Locus 3770 :

2 alleles

99.2% missing data

1	(0.395) 0.391 0.398

2	(0.605) 0.609 0.602

Locus 3771 :

2 alleles

91.0% missing data

1	(0.894) 0.910 0.890

2	(0.106) 0.090 0.110

Locus 3772 :

2 alleles

90.6% missing data

2	(0.905) 0.924 0.895

3	(0.095) 0.076 0.105

Locus 3773 :

2 alleles

99.2% missing data

4	(0.567) 0.570 0.559

3	(0.433) 0.430 0.441

Locus 3774 :

2 alleles

96.9% missing data

4	(0.209) 0.220 0.198

3	(0.791) 0.780 0.802

Locus 3775 :

2 alleles

0.0% missing data

2	(0.988) 0.984 0.997

3	(0.012) 0.016 0.003

Locus 3776 :

2 alleles

99.2% missing data

2	(0.314) 0.310 0.322

3	(0.686) 0.690 0.678

Locus 3777 :

2 alleles

99.2% missing data

4	(0.277) 0.273 0.286

2	(0.723) 0.727 0.714

Locus 3778 :

2 alleles

99.2% missing data

4	(0.565) 0.567 0.562

1	(0.435) 0.433 0.438

Locus 3779 :

2 alleles

99.2% missing data

2	(0.479) 0.475 0.485

1	(0.521) 0.525 0.515

Locus 3780 :

2 alleles

91.0% missing data

2	(0.203) 0.167 0.240

4	(0.797) 0.833 0.760

Locus 3781 :

2 alleles

99.2% missing data

3	(0.566) 0.572 0.557

4	(0.434) 0.428 0.443

Locus 3782 :

2 alleles

99.2% missing data

3	(0.438) 0.434 0.439

4	(0.562) 0.566 0.561

Locus 3783 :

2 alleles

99.2% missing data

1	(0.707) 0.707 0.701

3	(0.293) 0.293 0.299

Locus 3784 :

2 alleles

98.4% missing data

3	(0.202) 0.204 0.195

1	(0.798) 0.796 0.805

Locus 3785 :

2 alleles

99.2% missing data

3	(0.732) 0.722 0.735

4	(0.268) 0.278 0.265

Locus 3786 :

2 alleles

99.2% missing data

1	(0.540) 0.541 0.541

3	(0.460) 0.459 0.459

Locus 3787 :

2 alleles

99.2% missing data

3	(0.543) 0.541 0.544

2	(0.457) 0.459 0.456

Locus 3788 :

2 alleles

99.2% missing data

1	(0.456) 0.445 0.462

4	(0.544) 0.555 0.538

Locus 3789 :

2 alleles

99.2% missing data

4	(0.575) 0.570 0.575

3	(0.425) 0.430 0.425

Locus 3790 :

2 alleles

94.9% missing data

2	(0.911) 0.913 0.913

3	(0.089) 0.087 0.087

Locus 3791 :

2 alleles

99.2% missing data

3	(0.543) 0.541 0.547

2	(0.457) 0.459 0.453

Locus 3792 :

2 alleles

99.2% missing data

4	(0.406) 0.408 0.408

2	(0.594) 0.592 0.592

Locus 3793 :

2 alleles

99.2% missing data

1	(0.323) 0.331 0.318

3	(0.677) 0.669 0.682

Locus 3794 :

2 alleles

99.2% missing data

2	(0.639) 0.645 0.640

3	(0.361) 0.355 0.360

Locus 3795 :

2 alleles

99.2% missing data

4	(0.755) 0.750 0.762

1	(0.245) 0.250 0.238

Locus 3796 :

2 alleles

99.2% missing data

2	(0.665) 0.662 0.666

3	(0.335) 0.338 0.334

Locus 3797 :

2 alleles

99.2% missing data

3	(0.748) 0.740 0.754

1	(0.252) 0.260 0.246

Locus 3798 :

2 alleles

99.2% missing data

3	(0.599) 0.594 0.610

4	(0.401) 0.406 0.390

Locus 3799 :

2 alleles

99.2% missing data

3	(0.291) 0.283 0.295

1	(0.709) 0.717 0.705

Locus 3800 :

2 alleles

99.2% missing data

4	(0.259) 0.260 0.259

3	(0.741) 0.740 0.741

Locus 3801 :

2 alleles

99.2% missing data

2	(0.509) 0.501 0.512

4	(0.491) 0.499 0.488

Locus 3802 :

2 alleles

99.2% missing data

2	(0.283) 0.277 0.293

4	(0.717) 0.723 0.707

Locus 3803 :

2 alleles

99.2% missing data

3	(0.237) 0.229 0.242

2	(0.763) 0.771 0.758

Locus 3804 :

2 alleles

99.2% missing data

3	(0.694) 0.691 0.700

2	(0.306) 0.309 0.300

Locus 3805 :

2 alleles

99.2% missing data

4	(0.279) 0.286 0.271

1	(0.721) 0.714 0.729

Locus 3806 :

2 alleles

98.8% missing data

3	(0.333) 0.330 0.335

1	(0.667) 0.670 0.665

Locus 3807 :

2 alleles

99.2% missing data

3	(0.554) 0.546 0.557

4	(0.446) 0.454 0.443

Locus 3808 :

2 alleles

99.2% missing data

3	(0.742) 0.736 0.747

2	(0.258) 0.264 0.253

Locus 3809 :

2 alleles

99.2% missing data

2	(0.654) 0.656 0.657

3	(0.346) 0.344 0.343

Locus 3810 :

2 alleles

98.4% missing data

3	(0.321) 0.320 0.316

4	(0.679) 0.680 0.684

Locus 3811 :

2 alleles

98.0% missing data

3	(0.324) 0.320 0.319

1	(0.676) 0.680 0.681

Locus 3812 :

3 alleles

98.8% missing data

2	(0.371) 0.378 0.366

1	(0.201) 0.200 0.201

3	(0.428) 0.421 0.433

Locus 3813 :

2 alleles

99.2% missing data

3	(0.343) 0.350 0.336

4	(0.657) 0.650 0.664

Locus 3814 :

2 alleles

87.5% missing data

4	(0.913) 0.916 0.924

1	(0.087) 0.084 0.076

Locus 3815 :

2 alleles

91.0% missing data

2	(0.899) 0.914 0.892

1	(0.101) 0.086 0.108

Locus 3816 :

2 alleles

9.4% missing data

2	(0.988) 0.982 0.996

3	(0.012) 0.018 0.004

Locus 3817 :

2 alleles

1.2% missing data

3	(0.987) 0.990 0.993

2	(0.013) 0.010 0.007

Locus 3818 :

2 alleles

99.2% missing data

3	(0.582) 0.591 0.573

2	(0.418) 0.409 0.427

Locus 3819 :

2 alleles

98.8% missing data

2	(0.698) 0.701 0.696

1	(0.302) 0.299 0.304

Locus 3820 :

2 alleles

99.2% missing data

1	(0.543) 0.545 0.534

3	(0.457) 0.455 0.466

Locus 3821 :

2 alleles

99.2% missing data

1	(0.727) 0.730 0.722

3	(0.273) 0.270 0.278

Locus 3822 :

2 alleles

99.2% missing data

3	(0.534) 0.539 0.533

1	(0.466) 0.461 0.467

Locus 3823 :

2 alleles

91.0% missing data

2	(0.890) 0.906 0.881

4	(0.110) 0.094 0.119

Locus 3824 :

2 alleles

98.8% missing data

4	(0.258) 0.262 0.258

1	(0.742) 0.738 0.742

Locus 3825 :

2 alleles

90.6% missing data

2	(0.917) 0.933 0.907

4	(0.083) 0.067 0.093

Locus 3826 :

2 alleles

99.2% missing data

4	(0.559) 0.559 0.559

1	(0.441) 0.441 0.441

Locus 3827 :

2 alleles

99.2% missing data

1	(0.370) 0.377 0.368

2	(0.630) 0.623 0.632

Locus 3828 :

2 alleles

98.4% missing data

1	(0.689) 0.691 0.690

2	(0.311) 0.309 0.310

Locus 3829 :

2 alleles

99.2% missing data

3	(0.784) 0.777 0.788

1	(0.216) 0.223 0.212

Locus 3830 :

2 alleles

93.0% missing data

4	(0.909) 0.906 0.910

3	(0.091) 0.094 0.090

Locus 3831 :

2 alleles

93.4% missing data

2	(0.881) 0.866 0.897

3	(0.119) 0.134 0.103

Locus 3832 :

2 alleles

99.2% missing data

2	(0.386) 0.384 0.394

3	(0.614) 0.616 0.606

Locus 3833 :

2 alleles

98.8% missing data

3	(0.599) 0.603 0.591

1	(0.401) 0.397 0.409

Locus 3834 :

2 alleles

91.0% missing data

1	(0.905) 0.905 0.916

4	(0.095) 0.095 0.084

Locus 3835 :

2 alleles

99.2% missing data

1	(0.215) 0.218 0.217

4	(0.785) 0.782 0.783

Locus 3836 :

2 alleles

98.8% missing data

3	(0.826) 0.829 0.822

1	(0.174) 0.171 0.178

Locus 3837 :

2 alleles

99.2% missing data

3	(0.572) 0.571 0.576

4	(0.428) 0.429 0.424

Locus 3838 :

2 alleles

91.0% missing data

3	(0.928) 0.942 0.919

1	(0.072) 0.058 0.081

Locus 3839 :

2 alleles

98.4% missing data

3	(0.603) 0.608 0.607

1	(0.397) 0.392 0.393

Locus 3840 :

2 alleles

97.7% missing data

3	(0.250) 0.254 0.247

1	(0.750) 0.746 0.753

Locus 3841 :

2 alleles

99.2% missing data

2	(0.563) 0.567 0.560

4	(0.437) 0.433 0.440

Locus 3842 :

2 alleles

99.2% missing data

1	(0.373) 0.375 0.370

4	(0.627) 0.625 0.630

Locus 3843 :

2 alleles

99.2% missing data

4	(0.540) 0.541 0.549

2	(0.460) 0.459 0.451

Locus 3844 :

2 alleles

97.7% missing data

1	(0.301) 0.299 0.289

4	(0.699) 0.701 0.711

Locus 3845 :

2 alleles

99.2% missing data

4	(0.811) 0.803 0.810

3	(0.189) 0.197 0.190

Locus 3846 :

2 alleles

97.3% missing data

1	(0.695) 0.694 0.698

4	(0.305) 0.306 0.302

Locus 3847 :

2 alleles

95.7% missing data

3	(0.796) 0.801 0.799

2	(0.204) 0.199 0.201

Locus 3848 :

2 alleles

95.7% missing data

4	(0.284) 0.267 0.294

2	(0.716) 0.733 0.706

Locus 3849 :

2 alleles

99.2% missing data

1	(0.660) 0.668 0.657

2	(0.340) 0.332 0.343

Locus 3850 :

2 alleles

99.2% missing data

1	(0.398) 0.394 0.402

3	(0.602) 0.606 0.598

Locus 3851 :

2 alleles

99.2% missing data

4	(0.372) 0.374 0.377

2	(0.628) 0.626 0.623

Locus 3852 :

2 alleles

99.2% missing data

3	(0.376) 0.376 0.378

1	(0.624) 0.624 0.622

Locus 3853 :

2 alleles

10.5% missing data

2	(0.986) 0.989 0.992

1	(0.014) 0.011 0.008

Locus 3854 :

2 alleles

3.5% missing data

3	(0.983) 0.977 0.995

1	(0.017) 0.023 0.005

Locus 3855 :

2 alleles

98.8% missing data

1	(0.718) 0.716 0.722

4	(0.282) 0.284 0.278

Locus 3856 :

2 alleles

99.2% missing data

4	(0.429) 0.423 0.439

3	(0.571) 0.577 0.561

Locus 3857 :

2 alleles

98.8% missing data

4	(0.620) 0.620 0.623

1	(0.380) 0.380 0.377

Locus 3858 :

2 alleles

99.2% missing data

3	(0.574) 0.576 0.577

1	(0.426) 0.424 0.423

Locus 3859 :

2 alleles

96.1% missing data

3	(0.845) 0.857 0.840

2	(0.155) 0.143 0.160

Locus 3860 :

2 alleles

99.2% missing data

4	(0.801) 0.799 0.803

1	(0.199) 0.201 0.197

Locus 3861 :

2 alleles

91.0% missing data

4	(0.749) 0.753 0.755

3	(0.251) 0.247 0.245

Locus 3862 :

2 alleles

99.2% missing data

3	(0.631) 0.636 0.625

4	(0.369) 0.364 0.375

Locus 3863 :

2 alleles

91.4% missing data

4	(0.904) 0.911 0.910

3	(0.096) 0.089 0.090

Locus 3864 :

2 alleles

98.4% missing data

3	(0.637) 0.641 0.637

4	(0.363) 0.359 0.363

Locus 3865 :

2 alleles

99.2% missing data

4	(0.287) 0.287 0.286

1	(0.713) 0.713 0.714

Locus 3866 :

2 alleles

99.2% missing data

2	(0.234) 0.238 0.235

1	(0.766) 0.762 0.765

Locus 3867 :

2 alleles

98.4% missing data

4	(0.476) 0.477 0.461

3	(0.524) 0.523 0.539

Locus 3868 :

2 alleles

9.0% missing data

3	(0.975) 0.951 0.992

2	(0.025) 0.049 0.008

Locus 3869 :

3 alleles

98.4% missing data

4	(0.433) 0.424 0.443

1	(0.283) 0.279 0.286

2	(0.285) 0.298 0.271

Locus 3870 :

2 alleles

98.0% missing data

1	(0.807) 0.817 0.804

2	(0.193) 0.183 0.196

Locus 3871 :

2 alleles

91.0% missing data

1	(0.910) 0.925 0.901

3	(0.090) 0.075 0.099

Locus 3872 :

2 alleles

98.8% missing data

1	(0.571) 0.567 0.572

4	(0.429) 0.433 0.428

Locus 3873 :

3 alleles

98.4% missing data

3	(0.344) 0.343 0.347

4	(0.356) 0.353 0.351

1	(0.300) 0.304 0.301

Locus 3874 :

2 alleles

99.2% missing data

1	(0.301) 0.301 0.305

3	(0.699) 0.699 0.695

Locus 3875 :

2 alleles

97.7% missing data

1	(0.155) 0.151 0.155

3	(0.845) 0.849 0.845

Locus 3876 :

2 alleles

91.4% missing data

4	(0.917) 0.925 0.918

1	(0.083) 0.075 0.082

Locus 3877 :

2 alleles

98.8% missing data

1	(0.619) 0.610 0.626

4	(0.381) 0.390 0.374

Locus 3878 :

2 alleles

96.9% missing data

3	(0.837) 0.840 0.843

1	(0.163) 0.160 0.157

Locus 3879 :

2 alleles

96.9% missing data

2	(0.679) 0.667 0.698

4	(0.321) 0.333 0.302

Locus 3880 :

2 alleles

99.2% missing data

3	(0.262) 0.261 0.260

2	(0.738) 0.739 0.740

Locus 3881 :

2 alleles

98.4% missing data

3	(0.250) 0.240 0.253

1	(0.750) 0.760 0.747

Locus 3882 :

2 alleles

99.2% missing data

3	(0.600) 0.610 0.595

1	(0.400) 0.390 0.405

Locus 3883 :

2 alleles

99.2% missing data

2	(0.585) 0.583 0.583

3	(0.415) 0.417 0.417

Locus 3884 :

2 alleles

99.2% missing data

1	(0.550) 0.546 0.550

3	(0.450) 0.454 0.450

Locus 3885 :

2 alleles

99.2% missing data

2	(0.291) 0.287 0.298

4	(0.709) 0.713 0.702

Locus 3886 :

2 alleles

98.4% missing data

4	(0.590) 0.606 0.581

3	(0.410) 0.394 0.419

Locus 3887 :

3 alleles

98.8% missing data

3	(0.332) 0.333 0.335

4	(0.328) 0.332 0.328

2	(0.341) 0.336 0.337

Locus 3888 :

2 alleles

99.2% missing data

1	(0.552) 0.550 0.555

2	(0.448) 0.450 0.445

Locus 3889 :

2 alleles

99.2% missing data

2	(0.451) 0.453 0.449

4	(0.549) 0.547 0.551

Locus 3890 :

2 alleles

98.8% missing data

4	(0.421) 0.429 0.417

1	(0.579) 0.571 0.583

Locus 3891 :

2 alleles

97.3% missing data

4	(0.782) 0.777 0.793

2	(0.218) 0.223 0.207

Locus 3892 :

2 alleles

99.2% missing data

4	(0.611) 0.614 0.602

1	(0.389) 0.386 0.398

Locus 3893 :

2 alleles

97.7% missing data

1	(0.823) 0.833 0.816

2	(0.177) 0.167 0.184

Locus 3894 :

2 alleles

99.2% missing data

3	(0.498) 0.501 0.503

2	(0.502) 0.499 0.497

Locus 3895 :

2 alleles

93.4% missing data

3	(0.901) 0.900 0.905

1	(0.099) 0.100 0.095

Locus 3896 :

2 alleles

99.2% missing data

1	(0.490) 0.488 0.494

4	(0.510) 0.512 0.506

Locus 3897 :

2 alleles

97.7% missing data

2	(0.846) 0.843 0.851

3	(0.154) 0.157 0.149

Locus 3898 :

2 alleles

99.2% missing data

3	(0.746) 0.744 0.752

2	(0.254) 0.256 0.248

Locus 3899 :

2 alleles

96.1% missing data

4	(0.126) 0.123 0.129

3	(0.874) 0.877 0.871

Locus 3900 :

2 alleles

99.2% missing data

3	(0.436) 0.441 0.431

1	(0.564) 0.559 0.569

Locus 3901 :

2 alleles

98.8% missing data

1	(0.301) 0.302 0.299

3	(0.699) 0.698 0.701

Locus 3902 :

2 alleles

99.2% missing data

1	(0.386) 0.385 0.393

3	(0.614) 0.615 0.607

Locus 3903 :

2 alleles

95.3% missing data

4	(0.837) 0.842 0.842

2	(0.163) 0.158 0.158

Locus 3904 :

2 alleles

95.3% missing data

1	(0.253) 0.232 0.266

2	(0.747) 0.768 0.734

Locus 3905 :

2 alleles

99.2% missing data

4	(0.467) 0.461 0.472

1	(0.533) 0.539 0.528

Locus 3906 :

2 alleles

99.2% missing data

2	(0.425) 0.426 0.430

4	(0.575) 0.574 0.570

Locus 3907 :

2 alleles

99.2% missing data

3	(0.249) 0.245 0.249

4	(0.751) 0.755 0.751

Locus 3908 :

2 alleles

99.2% missing data

2	(0.737) 0.727 0.738

3	(0.263) 0.273 0.262

Locus 3909 :

2 alleles

98.4% missing data

1	(0.686) 0.690 0.693

2	(0.314) 0.310 0.307

Locus 3910 :

2 alleles

97.7% missing data

2	(0.179) 0.175 0.174

4	(0.821) 0.825 0.826

Locus 3911 :

2 alleles

96.5% missing data

2	(0.760) 0.773 0.754

3	(0.240) 0.227 0.246

Locus 3912 :

2 alleles

98.8% missing data

1	(0.623) 0.633 0.618

4	(0.377) 0.367 0.382

Locus 3913 :

2 alleles

93.0% missing data

4	(0.906) 0.906 0.913

1	(0.094) 0.094 0.087

Locus 3914 :

2 alleles

99.2% missing data

2	(0.620) 0.616 0.630

1	(0.380) 0.384 0.370

Locus 3915 :

2 alleles

99.2% missing data

2	(0.795) 0.791 0.795

3	(0.205) 0.209 0.205

Locus 3916 :

2 alleles

99.2% missing data

2	(0.455) 0.457 0.458

1	(0.545) 0.543 0.542

Locus 3917 :

2 alleles

98.0% missing data

1	(0.290) 0.285 0.292

4	(0.710) 0.715 0.708

Locus 3918 :

2 alleles

99.2% missing data

2	(0.614) 0.612 0.616

1	(0.386) 0.388 0.384

Locus 3919 :

2 alleles

98.4% missing data

1	(0.593) 0.606 0.593

4	(0.407) 0.394 0.407

Locus 3920 :

2 alleles

98.4% missing data

2	(0.493) 0.503 0.490

1	(0.507) 0.497 0.510

Locus 3921 :

2 alleles

99.2% missing data

4	(0.447) 0.443 0.448

1	(0.553) 0.557 0.552

Locus 3922 :

2 alleles

93.0% missing data

1	(0.919) 0.915 0.928

4	(0.081) 0.085 0.072

Locus 3923 :

2 alleles

96.1% missing data

2	(0.828) 0.831 0.830

3	(0.172) 0.169 0.170

Locus 3924 :

2 alleles

98.4% missing data

4	(0.421) 0.428 0.426

2	(0.579) 0.572 0.574

Locus 3925 :

2 alleles

99.2% missing data

1	(0.613) 0.606 0.617

4	(0.387) 0.394 0.383

Locus 3926 :

2 alleles

98.8% missing data

4	(0.579) 0.571 0.585

1	(0.421) 0.429 0.415

Locus 3927 :

2 alleles

99.2% missing data

3	(0.705) 0.709 0.697

4	(0.295) 0.291 0.303

Locus 3928 :

2 alleles

91.0% missing data

3	(0.888) 0.908 0.880

2	(0.112) 0.092 0.120

Locus 3929 :

2 alleles

96.1% missing data

2	(0.172) 0.158 0.178

4	(0.828) 0.842 0.822

Locus 3930 :

2 alleles

99.2% missing data

3	(0.634) 0.639 0.630

1	(0.366) 0.361 0.370

Locus 3931 :

2 alleles

0.0% missing data

4	(0.987) 0.982 0.996

1	(0.013) 0.018 0.004

Locus 3932 :

2 alleles

87.1% missing data

2	(0.649) 0.672 0.632

4	(0.351) 0.328 0.368

Locus 3933 :

2 alleles

98.8% missing data

1	(0.704) 0.702 0.706

4	(0.296) 0.298 0.294

Locus 3934 :

2 alleles

93.0% missing data

4	(0.899) 0.895 0.915

3	(0.101) 0.105 0.085

Locus 3935 :

2 alleles

90.2% missing data

1	(0.928) 0.925 0.939

2	(0.072) 0.075 0.061

Locus 3936 :

2 alleles

96.9% missing data

2	(0.900) 0.893 0.913

4	(0.100) 0.107 0.087

Locus 3937 :

2 alleles

98.8% missing data

4	(0.736) 0.734 0.737

1	(0.264) 0.266 0.263

Locus 3938 :

2 alleles

98.8% missing data

1	(0.436) 0.441 0.434

4	(0.564) 0.559 0.566

Locus 3939 :

2 alleles

96.5% missing data

4	(0.176) 0.162 0.182

3	(0.824) 0.838 0.818

Locus 3940 :

2 alleles

98.8% missing data

2	(0.581) 0.581 0.589

4	(0.419) 0.419 0.411

Locus 3941 :

3 alleles

98.4% missing data

3	(0.316) 0.309 0.321

4	(0.449) 0.453 0.454

1	(0.234) 0.237 0.226

Locus 3942 :

3 alleles

97.3% missing data

4	(0.294) 0.282 0.298

3	(0.553) 0.569 0.549

2	(0.153) 0.149 0.153

Locus 3943 :

2 alleles

99.2% missing data

4	(0.492) 0.498 0.493

1	(0.508) 0.502 0.507

Locus 3944 :

2 alleles

99.2% missing data

1	(0.543) 0.539 0.549

2	(0.457) 0.461 0.451

Locus 3945 :

2 alleles

99.2% missing data

2	(0.296) 0.293 0.299

4	(0.704) 0.707 0.701

Locus 3946 :

2 alleles

99.2% missing data

1	(0.731) 0.732 0.729

2	(0.269) 0.268 0.271

Locus 3947 :

2 alleles

96.1% missing data

1	(0.885) 0.884 0.892

4	(0.115) 0.116 0.108

Locus 3948 :

2 alleles

99.2% missing data

4	(0.568) 0.566 0.573

2	(0.432) 0.434 0.427

Locus 3949 :

2 alleles

99.2% missing data

3	(0.403) 0.404 0.412

2	(0.597) 0.596 0.588

Locus 3950 :

2 alleles

97.7% missing data

3	(0.307) 0.291 0.313

2	(0.693) 0.709 0.687

Locus 3951 :

2 alleles

99.2% missing data

3	(0.723) 0.721 0.722

2	(0.277) 0.279 0.278

Locus 3952 :

2 alleles

98.4% missing data

3	(0.580) 0.591 0.574

2	(0.420) 0.409 0.426

Locus 3953 :

2 alleles

99.2% missing data

2	(0.643) 0.633 0.649

3	(0.357) 0.367 0.351

Locus 3954 :

2 alleles

99.2% missing data

3	(0.452) 0.447 0.457

4	(0.548) 0.553 0.543

Locus 3955 :

2 alleles

99.2% missing data

4	(0.833) 0.834 0.828

1	(0.167) 0.166 0.172

Locus 3956 :

2 alleles

99.2% missing data

2	(0.604) 0.605 0.606

4	(0.396) 0.395 0.394

Locus 3957 :

2 alleles

91.0% missing data

2	(0.878) 0.900 0.867

4	(0.122) 0.100 0.133

Locus 3958 :

2 alleles

99.2% missing data

2	(0.363) 0.365 0.364

3	(0.637) 0.635 0.636

Locus 3959 :

2 alleles

99.2% missing data

1	(0.524) 0.524 0.524

4	(0.476) 0.476 0.476

Locus 3960 :

2 alleles

99.2% missing data

1	(0.787) 0.790 0.785

3	(0.213) 0.210 0.215

Locus 3961 :

2 alleles

99.2% missing data

2	(0.612) 0.611 0.618

1	(0.388) 0.389 0.382

Locus 3962 :

2 alleles

97.7% missing data

1	(0.799) 0.791 0.809

4	(0.201) 0.209 0.191

Locus 3963 :

2 alleles

5.1% missing data

1	(0.986) 0.989 0.992

3	(0.014) 0.011 0.008

Locus 3964 :

2 alleles

99.2% missing data

3	(0.200) 0.197 0.207

2	(0.800) 0.803 0.793

Locus 3965 :

2 alleles

99.2% missing data

4	(0.433) 0.436 0.435

2	(0.567) 0.564 0.565

Locus 3966 :

2 alleles

99.2% missing data

1	(0.730) 0.733 0.730

3	(0.270) 0.267 0.270

Locus 3967 :

2 alleles

99.2% missing data

4	(0.431) 0.428 0.433

3	(0.569) 0.572 0.567

Locus 3968 :

2 alleles

98.4% missing data

4	(0.675) 0.663 0.690

1	(0.325) 0.337 0.310

Locus 3969 :

2 alleles

98.4% missing data

1	(0.734) 0.738 0.733

4	(0.266) 0.262 0.267

Locus 3970 :

2 alleles

99.2% missing data

4	(0.585) 0.587 0.587

3	(0.415) 0.413 0.413

Locus 3971 :

2 alleles

99.2% missing data

4	(0.292) 0.287 0.299

2	(0.708) 0.713 0.701

Locus 3972 :

2 alleles

99.2% missing data

1	(0.524) 0.520 0.526

3	(0.476) 0.480 0.474

Locus 3973 :

2 alleles

99.2% missing data

1	(0.521) 0.524 0.523

4	(0.479) 0.476 0.477

Locus 3974 :

2 alleles

98.8% missing data

3	(0.686) 0.686 0.687

4	(0.314) 0.314 0.313

Locus 3975 :

2 alleles

9.0% missing data

3	(0.981) 0.976 0.994

4	(0.019) 0.024 0.006

Locus 3976 :

2 alleles

0.0% missing data

2	(0.968) 0.913 0.990

3	(0.032) 0.087 0.010

Locus 3977 :

2 alleles

99.2% missing data

1	(0.511) 0.499 0.516

3	(0.489) 0.501 0.484

Locus 3978 :

2 alleles

98.8% missing data

2	(0.746) 0.745 0.747

1	(0.254) 0.255 0.253

Locus 3979 :

2 alleles

99.2% missing data

1	(0.205) 0.206 0.210

2	(0.795) 0.794 0.790

Locus 3980 :

2 alleles

99.2% missing data

2	(0.199) 0.196 0.202

4	(0.801) 0.804 0.798

Locus 3981 :

2 alleles

97.7% missing data

2	(0.672) 0.688 0.666

1	(0.328) 0.312 0.334

Locus 3982 :

2 alleles

98.4% missing data

2	(0.530) 0.535 0.527

4	(0.470) 0.465 0.473

Locus 3983 :

2 alleles

96.5% missing data

1	(0.791) 0.800 0.790

4	(0.209) 0.200 0.210

Locus 3984 :

2 alleles

99.2% missing data

2	(0.514) 0.517 0.518

1	(0.486) 0.483 0.482

Locus 3985 :

2 alleles

99.2% missing data

4	(0.310) 0.310 0.314

2	(0.690) 0.690 0.686

Locus 3986 :

3 alleles

98.4% missing data

3	(0.301) 0.298 0.301

2	(0.409) 0.419 0.409

1	(0.290) 0.283 0.289

Locus 3987 :

2 alleles

99.2% missing data

3	(0.447) 0.453 0.449

2	(0.553) 0.547 0.551

Locus 3988 :

2 alleles

94.5% missing data

3	(0.616) 0.644 0.594

1	(0.384) 0.356 0.406

Locus 3989 :

2 alleles

98.8% missing data

3	(0.252) 0.250 0.255

4	(0.748) 0.750 0.745

Locus 3990 :

2 alleles

99.2% missing data

3	(0.350) 0.342 0.358

2	(0.650) 0.658 0.642

Locus 3991 :

2 alleles

99.2% missing data

2	(0.631) 0.629 0.629

3	(0.369) 0.371 0.371

Locus 3992 :

2 alleles

14.1% missing data

1	(0.984) 0.986 0.992

3	(0.016) 0.014 0.008

Locus 3993 :

3 alleles

98.8% missing data

4	(0.435) 0.429 0.439

3	(0.352) 0.350 0.354

2	(0.213) 0.221 0.208

Locus 3994 :

2 alleles

96.5% missing data

2	(0.423) 0.437 0.402

4	(0.577) 0.563 0.598

Locus 3995 :

3 alleles

89.8% missing data

3	(0.847) 0.879 0.828

2	(0.067) 0.054 0.077

1	(0.085) 0.067 0.095

Locus 3996 :

2 alleles

96.5% missing data

2	(0.647) 0.650 0.658

3	(0.353) 0.350 0.342

Locus 3997 :

2 alleles

98.0% missing data

1	(0.640) 0.632 0.647

2	(0.360) 0.368 0.353

Locus 3998 :

2 alleles

99.2% missing data

3	(0.384) 0.379 0.386

1	(0.616) 0.621 0.614

Locus 3999 :

2 alleles

99.2% missing data

3	(0.655) 0.649 0.655

4	(0.345) 0.351 0.345

Locus 4000 :

2 alleles

8.6% missing data

3	(0.975) 0.959 0.992

4	(0.025) 0.041 0.008

Locus 4001 :

2 alleles

99.2% missing data

1	(0.770) 0.764 0.772

3	(0.230) 0.236 0.228

Locus 4002 :

2 alleles

98.8% missing data

3	(0.431) 0.436 0.428

2	(0.569) 0.564 0.572

Locus 4003 :

2 alleles

99.2% missing data

3	(0.344) 0.351 0.337

2	(0.656) 0.649 0.663

Locus 4004 :

2 alleles

99.2% missing data

2	(0.436) 0.442 0.433

1	(0.564) 0.558 0.567

Locus 4005 :

2 alleles

99.2% missing data

3	(0.624) 0.626 0.621

1	(0.376) 0.374 0.379

Locus 4006 :

2 alleles

99.2% missing data

4	(0.805) 0.808 0.801

1	(0.195) 0.192 0.199

Locus 4007 :

2 alleles

99.2% missing data

1	(0.495) 0.498 0.501

2	(0.505) 0.502 0.499

Locus 4008 :

2 alleles

99.2% missing data

3	(0.704) 0.698 0.704

2	(0.296) 0.302 0.296

Locus 4009 :

2 alleles

99.2% missing data

2	(0.804) 0.805 0.798

4	(0.196) 0.195 0.202

Locus 4010 :

2 alleles

96.1% missing data

2	(0.126) 0.118 0.130

4	(0.874) 0.882 0.870

Locus 4011 :

2 alleles

98.8% missing data

2	(0.761) 0.763 0.760

4	(0.239) 0.237 0.240

Locus 4012 :

2 alleles

98.0% missing data

3	(0.835) 0.843 0.833

4	(0.165) 0.157 0.167

Locus 4013 :

2 alleles

98.8% missing data

1	(0.711) 0.707 0.714

2	(0.289) 0.293 0.286

Locus 4014 :

2 alleles

98.8% missing data

1	(0.694) 0.696 0.698

4	(0.306) 0.304 0.302

Locus 4015 :

2 alleles

99.2% missing data

2	(0.251) 0.257 0.252

1	(0.749) 0.743 0.748

Locus 4016 :

2 alleles

99.2% missing data

4	(0.244) 0.251 0.245

3	(0.756) 0.749 0.755

Locus 4017 :

2 alleles

99.2% missing data

2	(0.343) 0.344 0.341

4	(0.657) 0.656 0.659

Locus 4018 :

2 alleles

99.2% missing data

2	(0.717) 0.723 0.710

3	(0.283) 0.277 0.290

Locus 4019 :

2 alleles

99.2% missing data

3	(0.230) 0.237 0.224

4	(0.770) 0.763 0.776

Locus 4020 :

2 alleles

98.8% missing data

3	(0.598) 0.597 0.589

1	(0.402) 0.403 0.411

Locus 4021 :

2 alleles

99.2% missing data

3	(0.482) 0.484 0.481

2	(0.518) 0.516 0.519

Locus 4022 :

2 alleles

98.8% missing data

4	(0.258) 0.249 0.266

2	(0.742) 0.751 0.734

Locus 4023 :

2 alleles

8.2% missing data

2	(0.984) 0.977 0.995

3	(0.016) 0.023 0.005

Locus 4024 :

2 alleles

96.9% missing data

1	(0.123) 0.122 0.123

4	(0.877) 0.878 0.877

Locus 4025 :

2 alleles

99.2% missing data

4	(0.408) 0.407 0.404

3	(0.592) 0.593 0.596

Locus 4026 :

2 alleles

99.2% missing data

4	(0.430) 0.436 0.424

3	(0.570) 0.564 0.576

Locus 4027 :

2 alleles

99.2% missing data

2	(0.475) 0.469 0.477

3	(0.525) 0.531 0.523

Locus 4028 :

2 alleles

98.8% missing data

1	(0.649) 0.652 0.654

3	(0.351) 0.348 0.346

Locus 4029 :

2 alleles

99.2% missing data

3	(0.499) 0.500 0.504

2	(0.501) 0.500 0.496

Locus 4030 :

2 alleles

99.2% missing data

1	(0.672) 0.664 0.674

2	(0.328) 0.336 0.326

Locus 4031 :

2 alleles

99.2% missing data

4	(0.511) 0.514 0.509

3	(0.489) 0.486 0.491

Locus 4032 :

2 alleles

96.9% missing data

3	(0.889) 0.882 0.898

1	(0.111) 0.118 0.102

Locus 4033 :

2 alleles

99.2% missing data

4	(0.647) 0.650 0.641

3	(0.353) 0.350 0.359

Locus 4034 :

2 alleles

99.2% missing data

1	(0.521) 0.514 0.520

3	(0.479) 0.486 0.480

Locus 4035 :

2 alleles

99.2% missing data

2	(0.484) 0.484 0.486

1	(0.516) 0.516 0.514

Locus 4036 :

2 alleles

98.4% missing data

2	(0.626) 0.630 0.630

3	(0.374) 0.370 0.370

Locus 4037 :

2 alleles

99.2% missing data

3	(0.474) 0.477 0.474

4	(0.526) 0.523 0.526

Locus 4038 :

2 alleles

87.5% missing data

4	(0.749) 0.759 0.749

1	(0.251) 0.241 0.251

Locus 4039 :

2 alleles

98.8% missing data

2	(0.644) 0.636 0.651

3	(0.356) 0.364 0.349

Locus 4040 :

2 alleles

96.5% missing data

1	(0.785) 0.783 0.794

2	(0.215) 0.217 0.206

Locus 4041 :

2 alleles

98.8% missing data

3	(0.631) 0.625 0.635

2	(0.369) 0.375 0.365

Locus 4042 :

2 alleles

99.2% missing data

3	(0.589) 0.587 0.592

4	(0.411) 0.413 0.408

Locus 4043 :

2 alleles

93.8% missing data

4	(0.818) 0.809 0.838

2	(0.182) 0.191 0.162

Locus 4044 :

2 alleles

98.4% missing data

2	(0.621) 0.618 0.628

4	(0.379) 0.382 0.372

Locus 4045 :

2 alleles

89.5% missing data

4	(0.921) 0.913 0.936

1	(0.079) 0.087 0.064

Locus 4046 :

2 alleles

89.5% missing data

2	(0.846) 0.878 0.813

3	(0.154) 0.122 0.187

Locus 4047 :

2 alleles

97.7% missing data

2	(0.744) 0.733 0.757

1	(0.256) 0.267 0.243

Locus 4048 :

2 alleles

99.2% missing data

4	(0.641) 0.646 0.632

3	(0.359) 0.354 0.368

Locus 4049 :

2 alleles

99.2% missing data

2	(0.660) 0.652 0.659

3	(0.340) 0.348 0.341

Locus 4050 :

3 alleles

98.8% missing data

4	(0.343) 0.336 0.348

3	(0.226) 0.229 0.226

1	(0.431) 0.435 0.427

Locus 4051 :

2 alleles

97.3% missing data

3	(0.771) 0.771 0.781

1	(0.229) 0.229 0.219

Locus 4052 :

2 alleles

92.6% missing data

3	(0.867) 0.867 0.877

2	(0.133) 0.133 0.123

Locus 4053 :

2 alleles

99.2% missing data

4	(0.288) 0.280 0.297

1	(0.712) 0.720 0.703

Locus 4054 :

2 alleles

96.9% missing data

1	(0.830) 0.831 0.831

4	(0.170) 0.169 0.169

Locus 4055 :

2 alleles

91.4% missing data

3	(0.902) 0.909 0.906

1	(0.098) 0.091 0.094

Locus 4056 :

2 alleles

98.4% missing data

3	(0.433) 0.439 0.441

4	(0.567) 0.561 0.559

Locus 4057 :

4 alleles

98.4% missing data

1	(0.315) 0.319 0.311

4	(0.270) 0.270 0.269

2	(0.165) 0.158 0.170

3	(0.250) 0.253 0.250

Locus 4058 :

2 alleles

99.2% missing data

4	(0.447) 0.447 0.451

3	(0.553) 0.553 0.549

Locus 4059 :

2 alleles

99.2% missing data

1	(0.495) 0.497 0.491

3	(0.505) 0.503 0.509

Locus 4060 :

2 alleles

99.2% missing data

2	(0.480) 0.481 0.480

1	(0.520) 0.519 0.520

Locus 4061 :

2 alleles

99.2% missing data

3	(0.716) 0.722 0.716

1	(0.284) 0.278 0.284

Locus 4062 :

2 alleles

99.2% missing data

1	(0.520) 0.522 0.519

3	(0.480) 0.478 0.481

Locus 4063 :

2 alleles

99.2% missing data

1	(0.774) 0.774 0.770

3	(0.226) 0.226 0.230

Locus 4064 :

2 alleles

99.2% missing data

4	(0.525) 0.530 0.526

2	(0.475) 0.470 0.474

Locus 4065 :

2 alleles

99.2% missing data

3	(0.391) 0.395 0.388

4	(0.609) 0.605 0.612

Locus 4066 :

2 alleles

99.2% missing data

3	(0.773) 0.765 0.775

2	(0.227) 0.235 0.225

Locus 4067 :

2 alleles

97.3% missing data

2	(0.774) 0.760 0.788

3	(0.226) 0.240 0.212

Locus 4068 :

2 alleles

98.4% missing data

4	(0.714) 0.714 0.714

2	(0.286) 0.286 0.286

Locus 4069 :

2 alleles

99.2% missing data

2	(0.461) 0.460 0.461

3	(0.539) 0.540 0.539

Locus 4070 :

2 alleles

94.9% missing data

2	(0.863) 0.874 0.858

3	(0.137) 0.126 0.142

Locus 4071 :

2 alleles

99.2% missing data

2	(0.388) 0.385 0.396

4	(0.612) 0.615 0.604

Locus 4072 :

2 alleles

98.8% missing data

1	(0.516) 0.515 0.521

3	(0.484) 0.485 0.479

Locus 4073 :

2 alleles

99.2% missing data

1	(0.522) 0.527 0.520

3	(0.478) 0.473 0.480

Locus 4074 :

3 alleles

98.8% missing data

3	(0.196) 0.193 0.199

1	(0.665) 0.665 0.659

2	(0.139) 0.142 0.142

Locus 4075 :

2 alleles

93.0% missing data

4	(0.871) 0.862 0.884

3	(0.129) 0.138 0.116

Locus 4076 :

2 alleles

99.2% missing data

3	(0.397) 0.400 0.400

1	(0.603) 0.600 0.600

Locus 4077 :

3 alleles

96.9% missing data

3	(0.638) 0.631 0.650

4	(0.122) 0.120 0.122

2	(0.240) 0.249 0.227

Locus 4078 :

2 alleles

98.8% missing data

1	(0.747) 0.742 0.755

4	(0.253) 0.258 0.245

Locus 4079 :

3 alleles

98.4% missing data

2	(0.405) 0.406 0.401

1	(0.377) 0.377 0.383

4	(0.217) 0.217 0.216

Locus 4080 :

2 alleles

99.2% missing data

2	(0.199) 0.199 0.203

4	(0.801) 0.801 0.797

Locus 4081 :

2 alleles

99.2% missing data

1	(0.829) 0.822 0.831

4	(0.171) 0.178 0.169

Locus 4082 :

2 alleles

98.0% missing data

1	(0.217) 0.222 0.211

2	(0.783) 0.778 0.789

Locus 4083 :

2 alleles

98.8% missing data

2	(0.323) 0.317 0.327

1	(0.677) 0.683 0.673

Locus 4084 :

2 alleles

93.8% missing data

1	(0.905) 0.894 0.920

4	(0.095) 0.106 0.080

Locus 4085 :

3 alleles

98.8% missing data

1	(0.399) 0.401 0.394

4	(0.236) 0.226 0.246

3	(0.365) 0.373 0.360

Locus 4086 :

2 alleles

95.7% missing data

1	(0.695) 0.670 0.723

4	(0.305) 0.330 0.277

Locus 4087 :

2 alleles

94.5% missing data

1	(0.904) 0.908 0.908

2	(0.096) 0.092 0.092

Locus 4088 :

2 alleles

97.7% missing data

3	(0.826) 0.820 0.837

2	(0.174) 0.180 0.163

Locus 4089 :

2 alleles

10.5% missing data

3	(0.983) 0.973 0.995

1	(0.017) 0.027 0.005

Locus 4090 :

2 alleles

97.3% missing data

1	(0.680) 0.681 0.687

4	(0.320) 0.319 0.313

Locus 4091 :

2 alleles

96.5% missing data

3	(0.096) 0.096 0.090

1	(0.904) 0.904 0.910

Locus 4092 :

2 alleles

98.8% missing data

4	(0.699) 0.702 0.698

3	(0.301) 0.298 0.302

Locus 4093 :

2 alleles

96.9% missing data

1	(0.693) 0.693 0.706

3	(0.307) 0.307 0.294

Locus 4094 :

2 alleles

99.2% missing data

1	(0.546) 0.553 0.551

3	(0.454) 0.447 0.449

Locus 4095 :

2 alleles

99.2% missing data

3	(0.346) 0.345 0.346

2	(0.654) 0.655 0.654

Locus 4096 :

2 alleles

98.4% missing data

2	(0.555) 0.550 0.553

1	(0.445) 0.450 0.447

Locus 4097 :

2 alleles

99.2% missing data

1	(0.869) 0.864 0.880

3	(0.131) 0.136 0.120

Locus 4098 :

2 alleles

99.2% missing data

1	(0.570) 0.573 0.568

4	(0.430) 0.427 0.432

Locus 4099 :

2 alleles

99.2% missing data

4	(0.711) 0.711 0.713

1	(0.289) 0.289 0.287

Locus 4100 :

3 alleles

98.8% missing data

2	(0.434) 0.426 0.440

1	(0.390) 0.391 0.385

4	(0.177) 0.184 0.175

Locus 4101 :

3 alleles

98.4% missing data

4	(0.227) 0.220 0.231

3	(0.395) 0.394 0.404

1	(0.378) 0.386 0.365

Locus 4102 :

2 alleles

99.2% missing data

1	(0.606) 0.608 0.605

2	(0.394) 0.392 0.395

Locus 4103 :

2 alleles

99.2% missing data

1	(0.637) 0.643 0.634

4	(0.363) 0.357 0.366

Locus 4104 :

3 alleles

97.3% missing data

4	(0.246) 0.250 0.237

2	(0.281) 0.279 0.276

1	(0.472) 0.471 0.488

Locus 4105 :

3 alleles

98.4% missing data

1	(0.665) 0.661 0.673

2	(0.162) 0.164 0.162

4	(0.172) 0.175 0.165

Locus 4106 :

2 alleles

98.4% missing data

1	(0.486) 0.478 0.498

2	(0.514) 0.522 0.502

Locus 4107 :

2 alleles

99.2% missing data

1	(0.417) 0.417 0.417

3	(0.583) 0.583 0.583

Locus 4108 :

3 alleles

98.8% missing data

1	(0.389) 0.384 0.397

3	(0.352) 0.359 0.344

4	(0.259) 0.257 0.260

Locus 4109 :

2 alleles

99.2% missing data

4	(0.679) 0.674 0.683

1	(0.321) 0.326 0.317

Locus 4110 :

2 alleles

99.2% missing data

3	(0.457) 0.455 0.456

1	(0.543) 0.545 0.544

Locus 4111 :

2 alleles

94.9% missing data

1	(0.870) 0.863 0.883

4	(0.130) 0.137 0.117

Locus 4112 :

2 alleles

99.2% missing data

3	(0.426) 0.422 0.428

1	(0.574) 0.578 0.572

Locus 4113 :

2 alleles

99.2% missing data

3	(0.554) 0.550 0.555

1	(0.446) 0.450 0.445

Locus 4114 :

2 alleles

98.4% missing data

1	(0.527) 0.527 0.539

4	(0.473) 0.473 0.461

Locus 4115 :

2 alleles

95.3% missing data

3	(0.748) 0.752 0.743

2	(0.252) 0.248 0.257

Locus 4116 :

2 alleles

92.2% missing data

2	(0.862) 0.854 0.874

1	(0.138) 0.146 0.126

Locus 4117 :

2 alleles

98.8% missing data

1	(0.403) 0.396 0.414

4	(0.597) 0.604 0.586

Locus 4118 :

2 alleles

8.2% missing data

1	(0.985) 0.976 0.996

4	(0.015) 0.024 0.004

Locus 4119 :

2 alleles

99.2% missing data

3	(0.497) 0.494 0.496

1	(0.503) 0.506 0.504

Locus 4120 :

2 alleles

99.2% missing data

1	(0.363) 0.356 0.367

3	(0.637) 0.644 0.633

Locus 4121 :

2 alleles

97.3% missing data

3	(0.770) 0.772 0.774

4	(0.230) 0.228 0.226

Locus 4122 :

2 alleles

93.0% missing data

1	(0.874) 0.871 0.882

4	(0.126) 0.129 0.118

Locus 4123 :

2 alleles

96.9% missing data

3	(0.863) 0.856 0.875

1	(0.137) 0.144 0.125

Locus 4124 :

2 alleles

91.0% missing data

4	(0.933) 0.945 0.924

3	(0.067) 0.055 0.076

Locus 4125 :

2 alleles

98.0% missing data

3	(0.705) 0.698 0.719

1	(0.295) 0.302 0.281

Locus 4126 :

2 alleles

99.2% missing data

4	(0.453) 0.447 0.455

1	(0.547) 0.553 0.545

Locus 4127 :

2 alleles

96.5% missing data

3	(0.798) 0.803 0.801

1	(0.202) 0.197 0.199

Locus 4128 :

2 alleles

99.2% missing data

1	(0.239) 0.240 0.243

2	(0.761) 0.760 0.757

Locus 4129 :

2 alleles

99.2% missing data

3	(0.665) 0.667 0.664

1	(0.335) 0.333 0.336

Locus 4130 :

2 alleles

85.2% missing data

1	(0.957) 0.960 0.961

3	(0.043) 0.040 0.039

Locus 4131 :

2 alleles

99.2% missing data

1	(0.325) 0.317 0.329

4	(0.675) 0.683 0.671

Locus 4132 :

2 alleles

99.2% missing data

4	(0.704) 0.707 0.703

3	(0.296) 0.293 0.297

Locus 4133 :

4 alleles

93.0% missing data

2	(0.779) 0.765 0.802

3	(0.082) 0.080 0.079

4	(0.037) 0.045 0.031

1	(0.102) 0.111 0.087

Locus 4134 :

2 alleles

99.2% missing data

2	(0.531) 0.538 0.525

3	(0.469) 0.462 0.475

Locus 4135 :

2 alleles

96.1% missing data

4	(0.834) 0.845 0.834

2	(0.166) 0.155 0.166

Locus 4136 :

2 alleles

99.2% missing data

4	(0.590) 0.587 0.599

3	(0.410) 0.413 0.401

Locus 4137 :

2 alleles

99.2% missing data

3	(0.696) 0.699 0.690

1	(0.304) 0.301 0.310

Locus 4138 :

2 alleles

98.8% missing data

1	(0.625) 0.630 0.618

2	(0.375) 0.370 0.382

Locus 4139 :

2 alleles

64.8% missing data

1	(0.979) 0.985 0.981

2	(0.021) 0.015 0.019

Locus 4140 :

2 alleles

99.2% missing data

4	(0.382) 0.377 0.386

3	(0.618) 0.623 0.614

Locus 4141 :

2 alleles

98.8% missing data

2	(0.639) 0.640 0.640

3	(0.361) 0.360 0.360

Locus 4142 :

2 alleles

99.2% missing data

2	(0.519) 0.517 0.529

4	(0.481) 0.483 0.471

Locus 4143 :

2 alleles

99.2% missing data

4	(0.690) 0.686 0.693

2	(0.310) 0.314 0.307

Locus 4144 :

2 alleles

96.5% missing data

3	(0.328) 0.327 0.328

2	(0.672) 0.673 0.672

Locus 4145 :

2 alleles

98.4% missing data

2	(0.686) 0.693 0.685

1	(0.314) 0.307 0.315

Locus 4146 :

2 alleles

99.2% missing data

1	(0.420) 0.420 0.422

3	(0.580) 0.580 0.578

Locus 4147 :

2 alleles

99.2% missing data

1	(0.625) 0.629 0.623

2	(0.375) 0.371 0.377

Locus 4148 :

2 alleles

98.0% missing data

4	(0.849) 0.844 0.853

3	(0.151) 0.156 0.147

Locus 4149 :

2 alleles

99.2% missing data

1	(0.415) 0.412 0.421

4	(0.585) 0.588 0.579

Locus 4150 :

2 alleles

90.6% missing data

4	(0.924) 0.934 0.922

1	(0.076) 0.066 0.078

Locus 4151 :

2 alleles

99.2% missing data

3	(0.506) 0.503 0.509

1	(0.494) 0.497 0.491

Locus 4152 :

2 alleles

98.4% missing data

1	(0.746) 0.745 0.742

4	(0.254) 0.255 0.258

Locus 4153 :

2 alleles

99.2% missing data

1	(0.634) 0.635 0.629

4	(0.366) 0.365 0.371

Locus 4154 :

2 alleles

98.8% missing data

1	(0.435) 0.424 0.438

4	(0.565) 0.576 0.562

Locus 4155 :

2 alleles

99.2% missing data

4	(0.509) 0.514 0.508

3	(0.491) 0.486 0.492

Locus 4156 :

2 alleles

99.2% missing data

4	(0.687) 0.689 0.677

2	(0.313) 0.311 0.323

Locus 4157 :

2 alleles

92.6% missing data

3	(0.887) 0.882 0.906

4	(0.113) 0.118 0.094

Locus 4158 :

2 alleles

99.2% missing data

2	(0.583) 0.580 0.586

1	(0.417) 0.420 0.414

Locus 4159 :

2 alleles

98.8% missing data

2	(0.356) 0.355 0.358

4	(0.644) 0.645 0.642

Locus 4160 :

2 alleles

99.2% missing data

1	(0.394) 0.385 0.405

3	(0.606) 0.615 0.595

Locus 4161 :

2 alleles

96.1% missing data

1	(0.764) 0.779 0.766

3	(0.236) 0.221 0.234

Locus 4162 :

2 alleles

99.2% missing data

2	(0.492) 0.490 0.490

4	(0.508) 0.510 0.510

Locus 4163 :

2 alleles

92.2% missing data

4	(0.861) 0.855 0.883

3	(0.139) 0.145 0.117

Locus 4164 :

2 alleles

92.6% missing data

2	(0.942) 0.937 0.947

4	(0.058) 0.063 0.053

Locus 4165 :

2 alleles

96.1% missing data

2	(0.880) 0.883 0.885

1	(0.120) 0.117 0.115

Locus 4166 :

2 alleles

99.2% missing data

2	(0.529) 0.530 0.535

1	(0.471) 0.470 0.465

Locus 4167 :

2 alleles

99.2% missing data

4	(0.461) 0.463 0.465

2	(0.539) 0.537 0.535

Locus 4168 :

2 alleles

99.2% missing data

1	(0.563) 0.564 0.569

3	(0.437) 0.436 0.431

Locus 4169 :

2 alleles

99.2% missing data

2	(0.576) 0.575 0.576

4	(0.424) 0.425 0.424

Locus 4170 :

2 alleles

99.2% missing data

4	(0.531) 0.525 0.536

3	(0.469) 0.475 0.464

Locus 4171 :

2 alleles

97.7% missing data

4	(0.255) 0.245 0.252

3	(0.745) 0.755 0.748

Locus 4172 :

2 alleles

96.1% missing data

2	(0.776) 0.794 0.771

1	(0.224) 0.206 0.229

Locus 4173 :

2 alleles

92.6% missing data

2	(0.895) 0.899 0.902

3	(0.105) 0.101 0.098

Locus 4174 :

2 alleles

92.6% missing data

3	(0.917) 0.920 0.922

1	(0.083) 0.080 0.078

Locus 4175 :

2 alleles

92.2% missing data

1	(0.949) 0.951 0.949

4	(0.051) 0.049 0.051

Locus 4176 :

2 alleles

99.2% missing data

3	(0.370) 0.377 0.362

1	(0.630) 0.623 0.638

Locus 4177 :

2 alleles

96.5% missing data

1	(0.728) 0.738 0.725

4	(0.272) 0.262 0.275

Locus 4178 :

2 alleles

99.2% missing data

1	(0.284) 0.288 0.283

4	(0.716) 0.712 0.717

Locus 4179 :

2 alleles

99.2% missing data

2	(0.464) 0.461 0.470

4	(0.536) 0.539 0.530

Locus 4180 :

2 alleles

99.2% missing data

1	(0.409) 0.403 0.414

3	(0.591) 0.597 0.586

Locus 4181 :

2 alleles

97.7% missing data

4	(0.718) 0.722 0.728

2	(0.282) 0.278 0.272

Locus 4182 :

2 alleles

98.8% missing data

1	(0.728) 0.731 0.722

2	(0.272) 0.269 0.278

Locus 4183 :

3 alleles

98.8% missing data

4	(0.523) 0.520 0.523

2	(0.351) 0.351 0.356

1	(0.126) 0.129 0.121

Locus 4184 :

3 alleles

98.8% missing data

3	(0.583) 0.588 0.581

2	(0.214) 0.212 0.214

4	(0.203) 0.200 0.206

Locus 4185 :

2 alleles

98.8% missing data

4	(0.576) 0.581 0.564

2	(0.424) 0.419 0.436

Locus 4186 :

2 alleles

99.2% missing data

3	(0.232) 0.234 0.228

2	(0.768) 0.766 0.772

Locus 4187 :

2 alleles

99.2% missing data

4	(0.775) 0.772 0.779

1	(0.225) 0.228 0.221

Locus 4188 :

3 alleles

98.8% missing data

3	(0.510) 0.502 0.523

1	(0.205) 0.209 0.199

4	(0.284) 0.289 0.279

Locus 4189 :

2 alleles

99.2% missing data

3	(0.430) 0.433 0.423

1	(0.570) 0.567 0.577

Locus 4190 :

2 alleles

98.8% missing data

4	(0.732) 0.725 0.733

1	(0.268) 0.275 0.267

Locus 4191 :

2 alleles

0.4% missing data

4	(0.973) 0.926 0.992

3	(0.027) 0.074 0.008

Locus 4192 :

3 alleles

98.8% missing data

1	(0.220) 0.225 0.216

4	(0.549) 0.545 0.548

3	(0.231) 0.229 0.236

Locus 4193 :

2 alleles

3.9% missing data

3	(0.985) 0.988 0.992

2	(0.015) 0.012 0.008

Locus 4194 :

2 alleles

99.2% missing data

4	(0.233) 0.234 0.233

1	(0.767) 0.766 0.767

Locus 4195 :

2 alleles

99.2% missing data

4	(0.457) 0.461 0.459

2	(0.543) 0.539 0.541

Locus 4196 :

2 alleles

99.2% missing data

2	(0.444) 0.448 0.440

4	(0.556) 0.552 0.560

Locus 4197 :

2 alleles

9.0% missing data

1	(0.977) 0.960 0.992

3	(0.023) 0.040 0.008

Locus 4198 :

2 alleles

99.2% missing data

1	(0.260) 0.256 0.259

3	(0.740) 0.744 0.741

Locus 4199 :

2 alleles

98.4% missing data

2	(0.819) 0.831 0.810

4	(0.181) 0.169 0.190

Locus 4200 :

2 alleles

99.2% missing data

4	(0.294) 0.292 0.295

2	(0.706) 0.708 0.705

Locus 4201 :

2 alleles

99.2% missing data

1	(0.307) 0.302 0.313

3	(0.693) 0.698 0.687

Locus 4202 :

2 alleles

96.1% missing data

1	(0.780) 0.788 0.782

3	(0.220) 0.212 0.218

Locus 4203 :

2 alleles

99.2% missing data

4	(0.558) 0.561 0.556

2	(0.442) 0.439 0.444

Locus 4204 :

2 alleles

98.4% missing data

2	(0.778) 0.784 0.775

3	(0.222) 0.216 0.225

Locus 4205 :

3 alleles

98.8% missing data

2	(0.151) 0.155 0.154

3	(0.538) 0.530 0.536

4	(0.312) 0.315 0.311

Locus 4206 :

2 alleles

99.2% missing data

2	(0.514) 0.516 0.512

1	(0.486) 0.484 0.488

Locus 4207 :

2 alleles

99.2% missing data

2	(0.259) 0.261 0.260

1	(0.741) 0.739 0.740

Locus 4208 :

2 alleles

99.2% missing data

4	(0.440) 0.445 0.440

1	(0.560) 0.555 0.560

Locus 4209 :

2 alleles

99.2% missing data

1	(0.551) 0.550 0.551

4	(0.449) 0.450 0.449

Locus 4210 :

2 alleles

99.2% missing data

1	(0.625) 0.624 0.627

2	(0.375) 0.376 0.373

Locus 4211 :

3 alleles

97.3% missing data

1	(0.653) 0.661 0.651

4	(0.166) 0.158 0.173

2	(0.181) 0.182 0.177

Locus 4212 :

2 alleles

99.2% missing data

3	(0.490) 0.484 0.494

1	(0.510) 0.516 0.506

Locus 4213 :

2 alleles

98.0% missing data

4	(0.583) 0.573 0.592

1	(0.417) 0.427 0.408

Locus 4214 :

2 alleles

99.2% missing data

3	(0.581) 0.577 0.573

1	(0.419) 0.423 0.427

Locus 4215 :

2 alleles

92.6% missing data

1	(0.914) 0.905 0.926

3	(0.086) 0.095 0.074

Locus 4216 :

2 alleles

99.2% missing data

2	(0.411) 0.406 0.419

1	(0.589) 0.594 0.581

Locus 4217 :

2 alleles

99.2% missing data

1	(0.450) 0.442 0.459

3	(0.550) 0.558 0.541

Locus 4218 :

2 alleles

98.4% missing data

4	(0.532) 0.534 0.531

1	(0.468) 0.466 0.469

Locus 4219 :

2 alleles

98.8% missing data

3	(0.501) 0.500 0.496

2	(0.499) 0.500 0.504

Locus 4220 :

2 alleles

99.2% missing data

1	(0.357) 0.354 0.363

4	(0.643) 0.646 0.637

Locus 4221 :

2 alleles

75.0% missing data

1	(0.964) 0.965 0.971

3	(0.036) 0.035 0.029

Locus 4222 :

2 alleles

98.4% missing data

2	(0.630) 0.631 0.634

3	(0.370) 0.369 0.366

Locus 4223 :

2 alleles

99.2% missing data

4	(0.324) 0.324 0.330

1	(0.676) 0.676 0.670

Locus 4224 :

2 alleles

99.2% missing data

4	(0.430) 0.434 0.435

3	(0.570) 0.566 0.565

Locus 4225 :

2 alleles

98.0% missing data

1	(0.677) 0.680 0.679

2	(0.323) 0.320 0.321

Locus 4226 :

2 alleles

96.1% missing data

2	(0.714) 0.724 0.716

4	(0.286) 0.276 0.284

Locus 4227 :

2 alleles

99.2% missing data

3	(0.491) 0.492 0.487

2	(0.509) 0.508 0.513

Locus 4228 :

2 alleles

99.2% missing data

4	(0.256) 0.259 0.249

3	(0.744) 0.741 0.751

Locus 4229 :

2 alleles

8.6% missing data

1	(0.986) 0.981 0.996

2	(0.014) 0.019 0.004

Locus 4230 :

2 alleles

98.8% missing data

4	(0.838) 0.840 0.832

2	(0.162) 0.160 0.168

Locus 4231 :

2 alleles

99.2% missing data

1	(0.356) 0.354 0.355

3	(0.644) 0.646 0.645

Locus 4232 :

2 alleles

94.5% missing data

1	(0.887) 0.877 0.900

3	(0.113) 0.123 0.100

Locus 4233 :

2 alleles

99.2% missing data

4	(0.512) 0.507 0.511

1	(0.488) 0.493 0.489

Locus 4234 :

2 alleles

99.2% missing data

2	(0.359) 0.360 0.357

3	(0.641) 0.640 0.643

Locus 4235 :

3 alleles

98.4% missing data

4	(0.200) 0.197 0.203

1	(0.265) 0.265 0.262

3	(0.535) 0.537 0.535

Locus 4236 :

2 alleles

98.4% missing data

1	(0.673) 0.673 0.678

2	(0.327) 0.327 0.322

Locus 4237 :

2 alleles

99.2% missing data

4	(0.527) 0.524 0.532

1	(0.473) 0.476 0.468

Locus 4238 :

2 alleles

98.8% missing data

3	(0.687) 0.683 0.697

1	(0.313) 0.317 0.303

Locus 4239 :

3 alleles

98.8% missing data

4	(0.422) 0.417 0.424

2	(0.407) 0.401 0.410

1	(0.171) 0.182 0.167

Locus 4240 :

2 alleles

99.2% missing data

1	(0.665) 0.664 0.666

2	(0.335) 0.336 0.334

Locus 4241 :

2 alleles

99.2% missing data

3	(0.747) 0.742 0.747

2	(0.253) 0.258 0.253

Locus 4242 :

2 alleles

98.0% missing data

3	(0.596) 0.596 0.600

4	(0.404) 0.404 0.400

Locus 4243 :

2 alleles

98.8% missing data

1	(0.665) 0.672 0.662

3	(0.335) 0.328 0.338

Locus 4244 :

2 alleles

99.2% missing data

1	(0.430) 0.432 0.424

4	(0.570) 0.568 0.576

Locus 4245 :

2 alleles

96.5% missing data

1	(0.721) 0.712 0.740

2	(0.279) 0.288 0.260

Locus 4246 :

3 alleles

98.0% missing data

1	(0.679) 0.680 0.679

3	(0.100) 0.104 0.095

4	(0.222) 0.216 0.227

Locus 4247 :

2 alleles

99.2% missing data

2	(0.572) 0.575 0.578

4	(0.428) 0.425 0.422

Locus 4248 :

2 alleles

99.2% missing data

2	(0.256) 0.256 0.257

3	(0.744) 0.744 0.743

Locus 4249 :

2 alleles

16.0% missing data

2	(0.987) 0.979 0.996

3	(0.013) 0.021 0.004

Locus 4250 :

3 alleles

90.6% missing data

1	(0.848) 0.877 0.836

3	(0.096) 0.080 0.102

4	(0.056) 0.044 0.062

Locus 4251 :

2 alleles

92.6% missing data

3	(0.876) 0.877 0.877

1	(0.124) 0.123 0.123

Locus 4252 :

2 alleles

98.8% missing data

1	(0.756) 0.761 0.747

2	(0.244) 0.239 0.253

Locus 4253 :

2 alleles

99.2% missing data

3	(0.749) 0.753 0.740

1	(0.251) 0.247 0.260

Locus 4254 :

2 alleles

98.8% missing data

4	(0.648) 0.651 0.644

1	(0.352) 0.349 0.356

Locus 4255 :

2 alleles

99.2% missing data

4	(0.429) 0.421 0.437

1	(0.571) 0.579 0.563

Locus 4256 :

3 alleles

98.8% missing data

1	(0.313) 0.308 0.322

4	(0.339) 0.335 0.334

2	(0.347) 0.357 0.345

Locus 4257 :

2 alleles

98.8% missing data

1	(0.713) 0.710 0.713

4	(0.287) 0.290 0.287

Locus 4258 :

2 alleles

96.5% missing data

1	(0.652) 0.657 0.668

4	(0.348) 0.343 0.332

Locus 4259 :

2 alleles

96.5% missing data

1	(0.712) 0.711 0.724

2	(0.288) 0.289 0.276

Locus 4260 :

2 alleles

99.2% missing data

4	(0.671) 0.675 0.665

2	(0.329) 0.325 0.335

Locus 4261 :

2 alleles

90.2% missing data

3	(0.876) 0.897 0.860

4	(0.124) 0.103 0.140

Locus 4262 :

2 alleles

93.4% missing data

3	(0.206) 0.209 0.192

1	(0.794) 0.791 0.808

Locus 4263 :

2 alleles

99.2% missing data

1	(0.707) 0.699 0.705

4	(0.293) 0.301 0.295

Locus 4264 :

2 alleles

98.8% missing data

4	(0.279) 0.282 0.285

3	(0.721) 0.718 0.715

Locus 4265 :

2 alleles

99.2% missing data

4	(0.597) 0.588 0.605

2	(0.403) 0.412 0.395

Locus 4266 :

2 alleles

99.2% missing data

4	(0.663) 0.655 0.669

3	(0.337) 0.345 0.331

Locus 4267 :

2 alleles

99.2% missing data

1	(0.587) 0.583 0.587

2	(0.413) 0.417 0.413

Locus 4268 :

2 alleles

99.2% missing data

1	(0.329) 0.329 0.330

4	(0.671) 0.671 0.670

Locus 4269 :

2 alleles

99.2% missing data

2	(0.582) 0.586 0.577

3	(0.418) 0.414 0.423

Locus 4270 :

2 alleles

98.0% missing data

1	(0.599) 0.595 0.609

2	(0.401) 0.405 0.391

Locus 4271 :

2 alleles

99.2% missing data

2	(0.388) 0.388 0.384

1	(0.612) 0.612 0.616

Locus 4272 :

2 alleles

98.4% missing data

1	(0.195) 0.195 0.192

2	(0.805) 0.805 0.808

Locus 4273 :

2 alleles

99.2% missing data

2	(0.434) 0.437 0.440

1	(0.566) 0.563 0.560

Locus 4274 :

2 alleles

99.2% missing data

1	(0.178) 0.181 0.179

4	(0.822) 0.819 0.821

Locus 4275 :

3 alleles

94.1% missing data

4	(0.490) 0.514 0.474

2	(0.407) 0.382 0.432

1	(0.104) 0.104 0.094

Locus 4276 :

2 alleles

98.4% missing data

1	(0.564) 0.552 0.575

3	(0.436) 0.448 0.425

Locus 4277 :

2 alleles

99.2% missing data

1	(0.592) 0.590 0.598

4	(0.408) 0.410 0.402

Locus 4278 :

3 alleles

98.8% missing data

4	(0.222) 0.218 0.232

1	(0.337) 0.338 0.339

2	(0.441) 0.444 0.429

Locus 4279 :

2 alleles

93.0% missing data

4	(0.887) 0.888 0.896

3	(0.113) 0.112 0.104

Locus 4280 :

4 alleles

98.4% missing data

1	(0.310) 0.313 0.310

4	(0.133) 0.129 0.140

2	(0.353) 0.350 0.355

3	(0.204) 0.208 0.195

Locus 4281 :

2 alleles

99.2% missing data

3	(0.363) 0.362 0.365

4	(0.637) 0.638 0.635

Locus 4282 :

2 alleles

99.2% missing data

3	(0.696) 0.699 0.693

4	(0.304) 0.301 0.307

Locus 4283 :

2 alleles

98.4% missing data

1	(0.711) 0.712 0.708

4	(0.289) 0.288 0.292

Locus 4284 :

2 alleles

99.2% missing data

4	(0.315) 0.315 0.320

2	(0.685) 0.685 0.680

Locus 4285 :

2 alleles

98.4% missing data

3	(0.564) 0.566 0.568

4	(0.436) 0.434 0.432

Locus 4286 :

2 alleles

98.0% missing data

1	(0.734) 0.732 0.734

2	(0.266) 0.268 0.266

Locus 4287 :

2 alleles

99.2% missing data

4	(0.382) 0.376 0.382

2	(0.618) 0.624 0.618

Locus 4288 :

2 alleles

99.2% missing data

4	(0.385) 0.380 0.390

3	(0.615) 0.620 0.610

Locus 4289 :

2 alleles

99.2% missing data

1	(0.391) 0.398 0.398

4	(0.609) 0.602 0.602

Locus 4290 :

2 alleles

99.2% missing data

1	(0.358) 0.356 0.361

4	(0.642) 0.644 0.639

Locus 4291 :

2 alleles

99.2% missing data

1	(0.372) 0.358 0.379

3	(0.628) 0.642 0.621

Locus 4292 :

3 alleles

90.6% missing data

4	(0.878) 0.891 0.874

2	(0.064) 0.057 0.068

1	(0.058) 0.052 0.059

Locus 4293 :

2 alleles

96.9% missing data

4	(0.196) 0.196 0.201

1	(0.804) 0.804 0.799

Locus 4294 :

2 alleles

99.2% missing data

3	(0.390) 0.392 0.387

4	(0.610) 0.608 0.613

Locus 4295 :

2 alleles

93.0% missing data

2	(0.857) 0.858 0.872

4	(0.143) 0.142 0.128

Locus 4296 :

2 alleles

98.8% missing data

4	(0.676) 0.675 0.671

2	(0.324) 0.325 0.329

Locus 4297 :

2 alleles

99.2% missing data

2	(0.540) 0.542 0.534

4	(0.460) 0.458 0.466

Locus 4298 :

2 alleles

99.2% missing data

4	(0.406) 0.401 0.401

3	(0.594) 0.599 0.599

Locus 4299 :

2 alleles

91.0% missing data

4	(0.946) 0.956 0.938

3	(0.054) 0.044 0.062

Locus 4300 :

2 alleles

99.2% missing data

4	(0.603) 0.596 0.602

3	(0.397) 0.404 0.398

Locus 4301 :

2 alleles

99.2% missing data

4	(0.547) 0.543 0.548

3	(0.453) 0.457 0.452

Locus 4302 :

2 alleles

99.2% missing data

4	(0.563) 0.572 0.558

1	(0.437) 0.428 0.442

Locus 4303 :

2 alleles

92.2% missing data

3	(0.917) 0.915 0.922

4	(0.083) 0.085 0.078

Locus 4304 :

2 alleles

99.2% missing data

3	(0.301) 0.295 0.306

1	(0.699) 0.705 0.694

Locus 4305 :

2 alleles

99.2% missing data

4	(0.546) 0.548 0.546

2	(0.454) 0.452 0.454

Locus 4306 :

2 alleles

97.7% missing data

4	(0.795) 0.791 0.808

2	(0.205) 0.209 0.192

Locus 4307 :

2 alleles

99.2% missing data

1	(0.213) 0.219 0.209

4	(0.787) 0.781 0.791

Locus 4308 :

2 alleles

95.7% missing data

4	(0.807) 0.820 0.798

1	(0.193) 0.180 0.202

Locus 4309 :

2 alleles

98.8% missing data

1	(0.515) 0.512 0.511

3	(0.485) 0.488 0.489

Locus 4310 :

2 alleles

97.7% missing data

1	(0.580) 0.589 0.576

3	(0.420) 0.411 0.424

Locus 4311 :

2 alleles

8.2% missing data

4	(0.981) 0.974 0.994

3	(0.019) 0.026 0.006

Locus 4312 :

2 alleles

99.2% missing data

3	(0.731) 0.724 0.736

1	(0.269) 0.276 0.264

Locus 4313 :

2 alleles

98.0% missing data

4	(0.265) 0.274 0.255

1	(0.735) 0.726 0.745

Locus 4314 :

2 alleles

99.2% missing data

3	(0.642) 0.636 0.648

1	(0.358) 0.364 0.352

Locus 4315 :

2 alleles

94.9% missing data

1	(0.728) 0.732 0.732

4	(0.272) 0.268 0.268

Locus 4316 :

2 alleles

98.4% missing data

1	(0.730) 0.733 0.727

2	(0.270) 0.267 0.273

Locus 4317 :

2 alleles

99.2% missing data

2	(0.478) 0.479 0.479

4	(0.522) 0.521 0.521

Locus 4318 :

2 alleles

99.2% missing data

4	(0.552) 0.544 0.557

3	(0.448) 0.456 0.443

Locus 4319 :

2 alleles

99.2% missing data

4	(0.708) 0.707 0.709

3	(0.292) 0.293 0.291

Locus 4320 :

3 alleles

98.4% missing data

3	(0.222) 0.214 0.230

4	(0.435) 0.444 0.428

1	(0.343) 0.342 0.342

Locus 4321 :

2 alleles

99.2% missing data

2	(0.360) 0.353 0.361

3	(0.640) 0.647 0.639

Locus 4322 :

2 alleles

99.2% missing data

4	(0.717) 0.713 0.718

3	(0.283) 0.287 0.282

Locus 4323 :

2 alleles

99.2% missing data

3	(0.873) 0.873 0.872

2	(0.127) 0.127 0.128

Locus 4324 :

2 alleles

99.2% missing data

2	(0.359) 0.365 0.357

4	(0.641) 0.635 0.643

Locus 4325 :

2 alleles

93.0% missing data

2	(0.893) 0.888 0.902

4	(0.107) 0.112 0.098

Locus 4326 :

2 alleles

99.2% missing data

3	(0.476) 0.471 0.482

4	(0.524) 0.529 0.518

Locus 4327 :

2 alleles

99.2% missing data

1	(0.309) 0.306 0.311

2	(0.691) 0.694 0.689

Locus 4328 :

2 alleles

99.2% missing data

1	(0.409) 0.403 0.411

4	(0.591) 0.597 0.589

Locus 4329 :

2 alleles

99.2% missing data

4	(0.403) 0.403 0.400

3	(0.597) 0.597 0.600

Locus 4330 :

2 alleles

99.2% missing data

3	(0.421) 0.423 0.420

1	(0.579) 0.577 0.580

Locus 4331 :

2 alleles

98.8% missing data

4	(0.631) 0.629 0.637

1	(0.369) 0.371 0.363

Locus 4332 :

2 alleles

99.2% missing data

4	(0.607) 0.606 0.607

1	(0.393) 0.394 0.393

Locus 4333 :

2 alleles

99.2% missing data

4	(0.474) 0.476 0.478

1	(0.526) 0.524 0.522

Locus 4334 :

2 alleles

99.2% missing data

1	(0.576) 0.571 0.578

3	(0.424) 0.429 0.422

Locus 4335 :

2 alleles

99.2% missing data

2	(0.682) 0.681 0.683

3	(0.318) 0.319 0.317

Locus 4336 :

2 alleles

98.8% missing data

1	(0.856) 0.855 0.863

2	(0.144) 0.145 0.137

Locus 4337 :

2 alleles

92.6% missing data

4	(0.906) 0.898 0.920

3	(0.094) 0.102 0.080

Locus 4338 :

2 alleles

99.2% missing data

3	(0.206) 0.205 0.210

2	(0.794) 0.795 0.790

Locus 4339 :

2 alleles

99.2% missing data

3	(0.499) 0.502 0.500

1	(0.501) 0.498 0.500

Locus 4340 :

2 alleles

99.2% missing data

1	(0.677) 0.684 0.672

2	(0.323) 0.316 0.328

Locus 4341 :

2 alleles

98.8% missing data

1	(0.155) 0.162 0.157

4	(0.845) 0.838 0.843

Locus 4342 :

3 alleles

98.8% missing data

3	(0.444) 0.435 0.441

4	(0.245) 0.246 0.251

1	(0.311) 0.319 0.308

Locus 4343 :

2 alleles

99.2% missing data

2	(0.366) 0.362 0.367

4	(0.634) 0.638 0.633

Locus 4344 :

2 alleles

99.2% missing data

3	(0.510) 0.517 0.504

4	(0.490) 0.483 0.496

Locus 4345 :

2 alleles

99.2% missing data

3	(0.524) 0.520 0.531

2	(0.476) 0.480 0.469

Locus 4346 :

2 alleles

99.2% missing data

2	(0.184) 0.192 0.180

4	(0.816) 0.808 0.820

Locus 4347 :

2 alleles

0.0% missing data

2	(0.978) 0.982 0.984

3	(0.022) 0.018 0.016

Locus 4348 :

2 alleles

99.2% missing data

1	(0.689) 0.693 0.685

2	(0.311) 0.307 0.315

Locus 4349 :

2 alleles

98.8% missing data

2	(0.618) 0.617 0.623

3	(0.382) 0.383 0.377

Locus 4350 :

2 alleles

99.2% missing data

3	(0.768) 0.770 0.761

2	(0.232) 0.230 0.239

Locus 4351 :

2 alleles

99.2% missing data

1	(0.457) 0.456 0.457

3	(0.543) 0.544 0.543

Locus 4352 :

2 alleles

99.2% missing data

1	(0.749) 0.746 0.750

4	(0.251) 0.254 0.250

Locus 4353 :

2 alleles

99.2% missing data

4	(0.320) 0.319 0.326

3	(0.680) 0.681 0.674

Locus 4354 :

2 alleles

96.9% missing data

2	(0.236) 0.236 0.227

3	(0.764) 0.764 0.773

Locus 4355 :

2 alleles

99.2% missing data

4	(0.215) 0.213 0.222

2	(0.785) 0.787 0.778

Locus 4356 :

2 alleles

97.7% missing data

1	(0.649) 0.639 0.663

2	(0.351) 0.361 0.337

Locus 4357 :

2 alleles

99.2% missing data

1	(0.465) 0.461 0.463

2	(0.535) 0.539 0.537

Locus 4358 :

2 alleles

96.1% missing data

1	(0.201) 0.189 0.205

4	(0.799) 0.811 0.795

Locus 4359 :

2 alleles

99.2% missing data

3	(0.681) 0.673 0.689

2	(0.319) 0.327 0.311

Locus 4360 :

3 alleles

95.3% missing data

4	(0.673) 0.679 0.672

2	(0.174) 0.162 0.180

3	(0.152) 0.159 0.148

Locus 4361 :

2 alleles

99.2% missing data

1	(0.323) 0.317 0.329

3	(0.677) 0.683 0.671

Locus 4362 :

2 alleles

98.0% missing data

1	(0.669) 0.674 0.662

2	(0.331) 0.326 0.338

Locus 4363 :

2 alleles

98.0% missing data

4	(0.339) 0.333 0.344

3	(0.661) 0.667 0.656

Locus 4364 :

2 alleles

99.2% missing data

1	(0.767) 0.766 0.767

3	(0.233) 0.234 0.233

Locus 4365 :

2 alleles

99.2% missing data

4	(0.318) 0.311 0.323

1	(0.682) 0.689 0.677

Locus 4366 :

2 alleles

91.0% missing data

4	(0.873) 0.887 0.866

1	(0.127) 0.113 0.134

Locus 4367 :

2 alleles

99.2% missing data

2	(0.696) 0.689 0.695

4	(0.304) 0.311 0.305

Locus 4368 :

3 alleles

98.8% missing data

2	(0.204) 0.201 0.210

1	(0.372) 0.371 0.375

3	(0.423) 0.428 0.416

Locus 4369 :

2 alleles

97.7% missing data

1	(0.646) 0.646 0.662

2	(0.354) 0.354 0.338

Locus 4370 :

2 alleles

99.2% missing data

4	(0.594) 0.587 0.607

2	(0.406) 0.413 0.393

Locus 4371 :

2 alleles

86.7% missing data

2	(0.626) 0.696 0.562

1	(0.374) 0.304 0.438

Locus 4372 :

2 alleles

99.2% missing data

4	(0.359) 0.359 0.366

1	(0.641) 0.641 0.634

Locus 4373 :

3 alleles

98.8% missing data

2	(0.166) 0.158 0.174

1	(0.391) 0.400 0.384

4	(0.443) 0.442 0.442

Locus 4374 :

2 alleles

96.9% missing data

2	(0.865) 0.877 0.857

1	(0.135) 0.123 0.143

Locus 4375 :

2 alleles

99.2% missing data

4	(0.408) 0.412 0.407

1	(0.592) 0.588 0.593

Locus 4376 :

2 alleles

99.2% missing data

3	(0.359) 0.366 0.354

4	(0.641) 0.634 0.646

Locus 4377 :

2 alleles

99.2% missing data

1	(0.287) 0.297 0.288

2	(0.713) 0.703 0.712

Locus 4378 :

2 alleles

99.2% missing data

4	(0.446) 0.442 0.448

3	(0.554) 0.558 0.552

Locus 4379 :

2 alleles

99.2% missing data

4	(0.351) 0.349 0.359

1	(0.649) 0.651 0.641

Locus 4380 :

2 alleles

3.1% missing data

3	(0.988) 0.991 0.993

2	(0.012) 0.009 0.007

Locus 4381 :

2 alleles

99.2% missing data

4	(0.542) 0.543 0.540

1	(0.458) 0.457 0.460

Locus 4382 :

2 alleles

99.2% missing data

1	(0.442) 0.448 0.435

2	(0.558) 0.552 0.565

Locus 4383 :

3 alleles

98.8% missing data

2	(0.232) 0.236 0.232

4	(0.348) 0.347 0.346

3	(0.420) 0.417 0.423

Locus 4384 :

2 alleles

98.8% missing data

1	(0.256) 0.253 0.271

3	(0.744) 0.747 0.729

Locus 4385 :

2 alleles

99.2% missing data

2	(0.198) 0.194 0.204

4	(0.802) 0.806 0.796

Locus 4386 :

2 alleles

91.0% missing data

4	(0.913) 0.930 0.905

1	(0.087) 0.070 0.095

Locus 4387 :

2 alleles

99.2% missing data

2	(0.624) 0.619 0.630

3	(0.376) 0.381 0.370

Locus 4388 :

2 alleles

99.2% missing data

4	(0.443) 0.433 0.451

2	(0.557) 0.567 0.549

Locus 4389 :

3 alleles

98.8% missing data

1	(0.591) 0.589 0.587

2	(0.158) 0.155 0.167

3	(0.251) 0.256 0.246

Locus 4390 :

3 alleles

90.6% missing data

1	(0.849) 0.866 0.846

4	(0.093) 0.080 0.097

2	(0.058) 0.054 0.056

Locus 4391 :

2 alleles

91.0% missing data

2	(0.943) 0.948 0.941

4	(0.057) 0.052 0.059

Locus 4392 :

2 alleles

99.2% missing data

4	(0.313) 0.306 0.317

2	(0.687) 0.694 0.683

Locus 4393 :

2 alleles

99.2% missing data

2	(0.523) 0.527 0.524

3	(0.477) 0.473 0.476

Locus 4394 :

2 alleles

99.2% missing data

4	(0.749) 0.745 0.754

2	(0.251) 0.255 0.246

Locus 4395 :

2 alleles

99.2% missing data

4	(0.414) 0.417 0.412

2	(0.586) 0.583 0.588

Locus 4396 :

2 alleles

98.8% missing data

1	(0.744) 0.740 0.745

4	(0.256) 0.260 0.255

Locus 4397 :

2 alleles

96.9% missing data

3	(0.831) 0.839 0.826

2	(0.169) 0.161 0.174

Locus 4398 :

2 alleles

99.2% missing data

1	(0.576) 0.571 0.581

2	(0.424) 0.429 0.419

Locus 4399 :

2 alleles

92.6% missing data

2	(0.842) 0.833 0.864

4	(0.158) 0.167 0.136

Locus 4400 :

2 alleles

99.2% missing data

3	(0.340) 0.338 0.341

1	(0.660) 0.662 0.659

Locus 4401 :

2 alleles

99.2% missing data

3	(0.309) 0.311 0.310

2	(0.691) 0.689 0.690

Locus 4402 :

2 alleles

99.2% missing data

3	(0.748) 0.737 0.748

2	(0.252) 0.263 0.252

Locus 4403 :

2 alleles

91.4% missing data

3	(0.077) 0.074 0.071

4	(0.923) 0.926 0.929

Locus 4404 :

2 alleles

99.2% missing data

2	(0.519) 0.515 0.520

1	(0.481) 0.485 0.480

Locus 4405 :

2 alleles

98.0% missing data

3	(0.853) 0.844 0.861

1	(0.147) 0.156 0.139

Locus 4406 :

2 alleles

98.0% missing data

1	(0.686) 0.686 0.690

2	(0.314) 0.314 0.310

Locus 4407 :

2 alleles

99.2% missing data

2	(0.258) 0.262 0.259

4	(0.742) 0.738 0.741

Locus 4408 :

2 alleles

89.8% missing data

2	(0.928) 0.942 0.923

4	(0.072) 0.058 0.077

Locus 4409 :

2 alleles

99.2% missing data

1	(0.578) 0.573 0.579

4	(0.422) 0.427 0.421

Locus 4410 :

2 alleles

99.2% missing data

3	(0.598) 0.590 0.602

4	(0.402) 0.410 0.398

Locus 4411 :

2 alleles

99.2% missing data

4	(0.540) 0.543 0.533

2	(0.460) 0.457 0.467

Locus 4412 :

2 alleles

99.2% missing data

4	(0.592) 0.597 0.588

2	(0.408) 0.403 0.412

Locus 4413 :

2 alleles

96.5% missing data

1	(0.752) 0.774 0.745

3	(0.248) 0.226 0.255

Locus 4414 :

2 alleles

92.6% missing data

2	(0.892) 0.894 0.900

3	(0.108) 0.106 0.100

Locus 4415 :

2 alleles

99.2% missing data

4	(0.395) 0.394 0.401

2	(0.605) 0.606 0.599

Locus 4416 :

2 alleles

87.5% missing data

4	(0.694) 0.712 0.683

2	(0.306) 0.288 0.317

Locus 4417 :

2 alleles

99.2% missing data

2	(0.358) 0.352 0.362

3	(0.642) 0.648 0.638

Locus 4418 :

2 alleles

97.7% missing data

1	(0.737) 0.729 0.754

2	(0.263) 0.271 0.246

Locus 4419 :

2 alleles

99.2% missing data

1	(0.274) 0.271 0.273

3	(0.726) 0.729 0.727

Locus 4420 :

2 alleles

99.2% missing data

3	(0.658) 0.661 0.657

2	(0.342) 0.339 0.343

Locus 4421 :

2 alleles

99.2% missing data

2	(0.716) 0.707 0.715

1	(0.284) 0.293 0.285

Locus 4422 :

2 alleles

98.8% missing data

2	(0.372) 0.368 0.368

1	(0.628) 0.632 0.632

Locus 4423 :

2 alleles

99.2% missing data

4	(0.730) 0.736 0.723

1	(0.270) 0.264 0.277

Locus 4424 :

2 alleles

96.9% missing data

2	(0.306) 0.312 0.295

1	(0.694) 0.688 0.705

Locus 4425 :

2 alleles

96.9% missing data

4	(0.890) 0.892 0.886

3	(0.110) 0.108 0.114

Locus 4426 :

2 alleles

0.0% missing data

4	(0.979) 0.961 0.994

1	(0.021) 0.039 0.006

Locus 4427 :

2 alleles

0.0% missing data

2	(0.990) 0.992 0.994

3	(0.010) 0.008 0.006

Locus 4428 :

2 alleles

97.7% missing data

4	(0.402) 0.410 0.387

2	(0.598) 0.590 0.613

Locus 4429 :

2 alleles

99.2% missing data

4	(0.405) 0.399 0.415

3	(0.595) 0.601 0.585

Locus 4430 :

2 alleles

99.2% missing data

1	(0.338) 0.341 0.336

3	(0.662) 0.659 0.664

Locus 4431 :

2 alleles

99.2% missing data

3	(0.297) 0.299 0.289

4	(0.703) 0.701 0.711

Locus 4432 :

2 alleles

99.2% missing data

3	(0.483) 0.486 0.482

4	(0.517) 0.514 0.518

Locus 4433 :

2 alleles

99.2% missing data

4	(0.521) 0.506 0.529

2	(0.479) 0.494 0.471

Locus 4434 :

2 alleles

99.2% missing data

2	(0.407) 0.412 0.405

3	(0.593) 0.588 0.595

Locus 4435 :

2 alleles

98.8% missing data

4	(0.313) 0.305 0.325

3	(0.687) 0.695 0.675

Locus 4436 :

2 alleles

99.2% missing data

2	(0.439) 0.441 0.428

1	(0.561) 0.559 0.572

Locus 4437 :

2 alleles

99.2% missing data

1	(0.270) 0.270 0.277

4	(0.730) 0.730 0.723

Locus 4438 :

2 alleles

99.2% missing data

3	(0.559) 0.551 0.566

1	(0.441) 0.449 0.434

Locus 4439 :

3 alleles

98.4% missing data

2	(0.320) 0.313 0.327

3	(0.422) 0.426 0.417

1	(0.258) 0.261 0.256

Locus 4440 :

2 alleles

98.8% missing data

2	(0.689) 0.685 0.685

1	(0.311) 0.315 0.315

Locus 4441 :

2 alleles

99.2% missing data

3	(0.292) 0.296 0.289

4	(0.708) 0.704 0.711

Locus 4442 :

2 alleles

99.2% missing data

1	(0.466) 0.462 0.471

3	(0.534) 0.538 0.529

Locus 4443 :

2 alleles

98.8% missing data

3	(0.258) 0.256 0.260

1	(0.742) 0.744 0.740

Locus 4444 :

2 alleles

91.0% missing data

2	(0.919) 0.934 0.911

3	(0.081) 0.066 0.089

Locus 4445 :

2 alleles

96.5% missing data

3	(0.786) 0.788 0.793

1	(0.214) 0.212 0.207

Locus 4446 :

2 alleles

99.2% missing data

2	(0.410) 0.401 0.417

1	(0.590) 0.599 0.583

Locus 4447 :

2 alleles

99.2% missing data

1	(0.484) 0.481 0.482

3	(0.516) 0.519 0.518

Locus 4448 :

2 alleles

99.2% missing data

4	(0.358) 0.359 0.362

2	(0.642) 0.641 0.638

Locus 4449 :

2 alleles

99.2% missing data

2	(0.489) 0.485 0.495

4	(0.511) 0.515 0.505

Locus 4450 :

2 alleles

99.2% missing data

2	(0.671) 0.673 0.670

4	(0.329) 0.327 0.330

Locus 4451 :

2 alleles

99.2% missing data

2	(0.674) 0.669 0.679

4	(0.326) 0.331 0.321

Locus 4452 :

2 alleles

98.8% missing data

3	(0.160) 0.159 0.162

4	(0.840) 0.841 0.838

Locus 4453 :

2 alleles

99.2% missing data

4	(0.334) 0.330 0.341

2	(0.666) 0.670 0.659

Locus 4454 :

2 alleles

99.2% missing data

1	(0.369) 0.367 0.375

4	(0.631) 0.633 0.625

Locus 4455 :

2 alleles

98.8% missing data

4	(0.312) 0.311 0.315

3	(0.688) 0.689 0.685

Locus 4456 :

2 alleles

98.8% missing data

3	(0.522) 0.526 0.524

1	(0.478) 0.474 0.476

Locus 4457 :

2 alleles

98.8% missing data

2	(0.431) 0.430 0.427

1	(0.569) 0.570 0.573

Locus 4458 :

2 alleles

99.2% missing data

2	(0.807) 0.802 0.812

1	(0.193) 0.198 0.188

Locus 4459 :

2 alleles

99.2% missing data

1	(0.498) 0.493 0.502

4	(0.502) 0.507 0.498

Locus 4460 :

2 alleles

8.2% missing data

3	(0.980) 0.971 0.994

2	(0.020) 0.029 0.006

Locus 4461 :

2 alleles

90.6% missing data

2	(0.867) 0.875 0.868

1	(0.133) 0.125 0.132

Locus 4462 :

2 alleles

99.2% missing data

1	(0.681) 0.677 0.685

3	(0.319) 0.323 0.315

Locus 4463 :

3 alleles

98.8% missing data

2	(0.498) 0.492 0.500

3	(0.192) 0.186 0.195

1	(0.309) 0.322 0.305

Locus 4464 :

2 alleles

98.0% missing data

4	(0.826) 0.822 0.827

3	(0.174) 0.178 0.173

Locus 4465 :

2 alleles

99.2% missing data

2	(0.369) 0.367 0.377

3	(0.631) 0.633 0.623

Locus 4466 :

2 alleles

98.4% missing data

1	(0.733) 0.726 0.739

3	(0.267) 0.274 0.261

Locus 4467 :

2 alleles

96.5% missing data

2	(0.773) 0.783 0.774

1	(0.227) 0.217 0.226

Locus 4468 :

2 alleles

99.2% missing data

1	(0.169) 0.170 0.171

4	(0.831) 0.830 0.829

Locus 4469 :

2 alleles

99.2% missing data

1	(0.505) 0.500 0.510

4	(0.495) 0.500 0.490

Locus 4470 :

2 alleles

98.4% missing data

3	(0.645) 0.648 0.640

1	(0.355) 0.352 0.360

Locus 4471 :

2 alleles

99.2% missing data

3	(0.684) 0.682 0.684

4	(0.316) 0.318 0.316

Locus 4472 :

2 alleles

96.1% missing data

2	(0.760) 0.771 0.757

4	(0.240) 0.229 0.243

Locus 4473 :

2 alleles

95.3% missing data

3	(0.846) 0.843 0.852

2	(0.154) 0.157 0.148

Locus 4474 :

2 alleles

98.4% missing data

3	(0.259) 0.249 0.264

4	(0.741) 0.751 0.736

Locus 4475 :

2 alleles

98.4% missing data

3	(0.603) 0.602 0.605

1	(0.397) 0.398 0.395

Locus 4476 :

2 alleles

98.8% missing data

1	(0.761) 0.764 0.757

3	(0.239) 0.236 0.243

Locus 4477 :

3 alleles

98.0% missing data

3	(0.594) 0.587 0.609

4	(0.131) 0.142 0.120

1	(0.274) 0.271 0.271

Locus 4478 :

2 alleles

99.2% missing data

3	(0.320) 0.313 0.330

1	(0.680) 0.687 0.670

Locus 4479 :

2 alleles

99.2% missing data

3	(0.449) 0.455 0.449

4	(0.551) 0.545 0.551

Locus 4480 :

2 alleles

99.2% missing data

2	(0.353) 0.362 0.347

4	(0.647) 0.638 0.653

Locus 4481 :

2 alleles

99.2% missing data

4	(0.621) 0.617 0.622

2	(0.379) 0.383 0.378

Locus 4482 :

2 alleles

96.9% missing data

1	(0.769) 0.770 0.779

2	(0.231) 0.230 0.221

Locus 4483 :

2 alleles

99.2% missing data

3	(0.528) 0.529 0.531

2	(0.472) 0.471 0.469

Locus 4484 :

2 alleles

98.8% missing data

2	(0.450) 0.446 0.461

4	(0.550) 0.554 0.539

Locus 4485 :

2 alleles

99.2% missing data

3	(0.387) 0.389 0.384

4	(0.613) 0.611 0.616

Locus 4486 :

3 alleles

98.8% missing data

1	(0.230) 0.228 0.228

2	(0.301) 0.302 0.307

4	(0.469) 0.469 0.465

Locus 4487 :

2 alleles

99.2% missing data

4	(0.368) 0.374 0.365

1	(0.632) 0.626 0.635

Locus 4488 :

3 alleles

96.5% missing data

1	(0.280) 0.287 0.264

3	(0.592) 0.587 0.607

4	(0.129) 0.127 0.129

Locus 4489 :

2 alleles

98.0% missing data

1	(0.772) 0.768 0.784

4	(0.228) 0.232 0.216

Locus 4490 :

2 alleles

99.2% missing data

4	(0.366) 0.370 0.366

3	(0.634) 0.630 0.634

Locus 4491 :

2 alleles

98.4% missing data

1	(0.642) 0.642 0.646

2	(0.358) 0.358 0.354

Locus 4492 :

2 alleles

91.4% missing data

4	(0.937) 0.942 0.939

1	(0.063) 0.058 0.061

Locus 4493 :

2 alleles

99.2% missing data

3	(0.689) 0.695 0.683

2	(0.311) 0.305 0.317

Locus 4494 :

2 alleles

92.6% missing data

1	(0.934) 0.931 0.938

3	(0.066) 0.069 0.062

Locus 4495 :

2 alleles

99.2% missing data

3	(0.478) 0.477 0.480

4	(0.522) 0.523 0.520

Locus 4496 :

3 alleles

98.0% missing data

4	(0.511) 0.519 0.510

3	(0.252) 0.242 0.252

2	(0.236) 0.239 0.238

Locus 4497 :

2 alleles

99.2% missing data

3	(0.680) 0.682 0.682

4	(0.320) 0.318 0.318

Locus 4498 :

2 alleles

98.0% missing data

4	(0.738) 0.746 0.736

3	(0.262) 0.254 0.264

Locus 4499 :

3 alleles

98.4% missing data

1	(0.548) 0.534 0.550

4	(0.305) 0.309 0.310

2	(0.147) 0.156 0.140

Locus 4500 :

2 alleles

99.2% missing data

1	(0.452) 0.448 0.461

2	(0.548) 0.552 0.539

Locus 4501 :

2 alleles

99.2% missing data

4	(0.453) 0.452 0.457

1	(0.547) 0.548 0.543

Locus 4502 :

2 alleles

99.2% missing data

3	(0.489) 0.487 0.490

4	(0.511) 0.513 0.510

Locus 4503 :

2 alleles

95.3% missing data

4	(0.596) 0.568 0.627

3	(0.404) 0.432 0.373

Locus 4504 :

3 alleles

98.8% missing data

4	(0.486) 0.489 0.477

1	(0.359) 0.358 0.368

2	(0.155) 0.152 0.155

Locus 4505 :

2 alleles

98.4% missing data

4	(0.683) 0.679 0.689

3	(0.317) 0.321 0.311

Locus 4506 :

2 alleles

99.2% missing data

3	(0.243) 0.250 0.243

4	(0.757) 0.750 0.757

Locus 4507 :

2 alleles

99.2% missing data

4	(0.404) 0.406 0.407

1	(0.596) 0.594 0.593

Locus 4508 :

2 alleles

97.3% missing data

4	(0.657) 0.646 0.676

3	(0.343) 0.354 0.324

Locus 4509 :

2 alleles

99.2% missing data

3	(0.176) 0.175 0.180

2	(0.824) 0.825 0.820

Locus 4510 :

2 alleles

93.0% missing data

1	(0.920) 0.915 0.928

4	(0.080) 0.085 0.072

Locus 4511 :

2 alleles

98.8% missing data

4	(0.745) 0.738 0.752

3	(0.255) 0.262 0.248

Locus 4512 :

2 alleles

98.8% missing data

4	(0.715) 0.722 0.702

1	(0.285) 0.278 0.298

Locus 4513 :

2 alleles

99.2% missing data

1	(0.533) 0.535 0.532

4	(0.467) 0.465 0.468

Locus 4514 :

2 alleles

99.2% missing data

4	(0.589) 0.586 0.588

1	(0.411) 0.414 0.412

Locus 4515 :

2 alleles

99.2% missing data

4	(0.807) 0.808 0.801

1	(0.193) 0.192 0.199

Locus 4516 :

2 alleles

99.2% missing data

2	(0.320) 0.313 0.322

1	(0.680) 0.687 0.678

Locus 4517 :

2 alleles

99.2% missing data

4	(0.587) 0.583 0.591

1	(0.413) 0.417 0.409

Locus 4518 :

2 alleles

99.2% missing data

2	(0.584) 0.574 0.593

1	(0.416) 0.426 0.407

Locus 4519 :

2 alleles

98.0% missing data

1	(0.309) 0.313 0.303

4	(0.691) 0.687 0.697

Locus 4520 :

2 alleles

98.8% missing data

2	(0.397) 0.386 0.405

4	(0.603) 0.614 0.595

Locus 4521 :

2 alleles

99.2% missing data

2	(0.334) 0.330 0.341

3	(0.666) 0.670 0.659

Locus 4522 :

2 alleles

99.2% missing data

3	(0.540) 0.541 0.535

4	(0.460) 0.459 0.465

Locus 4523 :

2 alleles

98.8% missing data

2	(0.668) 0.666 0.673

3	(0.332) 0.334 0.327

Locus 4524 :

2 alleles

97.3% missing data

4	(0.847) 0.848 0.848

2	(0.153) 0.152 0.152

Locus 4525 :

2 alleles

98.8% missing data

4	(0.679) 0.679 0.683

3	(0.321) 0.321 0.317

Locus 4526 :

2 alleles

99.2% missing data

1	(0.385) 0.388 0.386

3	(0.615) 0.612 0.614

Locus 4527 :

2 alleles

75.8% missing data

3	(0.963) 0.960 0.976

4	(0.037) 0.040 0.024

Locus 4528 :

2 alleles

99.2% missing data

1	(0.719) 0.714 0.724

3	(0.281) 0.286 0.276

Locus 4529 :

2 alleles

97.7% missing data

4	(0.660) 0.651 0.678

3	(0.340) 0.349 0.322

Locus 4530 :

2 alleles

98.8% missing data

2	(0.454) 0.444 0.462

3	(0.546) 0.556 0.538

Locus 4531 :

2 alleles

98.8% missing data

2	(0.708) 0.702 0.716

1	(0.292) 0.298 0.284

Locus 4532 :

2 alleles

99.2% missing data

1	(0.660) 0.653 0.661

2	(0.340) 0.347 0.339

Locus 4533 :

2 alleles

99.2% missing data

1	(0.758) 0.758 0.754

4	(0.242) 0.242 0.246

Locus 4534 :

2 alleles

99.2% missing data

2	(0.252) 0.252 0.254

1	(0.748) 0.748 0.746

Locus 4535 :

2 alleles

98.8% missing data

4	(0.683) 0.680 0.686

1	(0.317) 0.320 0.314

Locus 4536 :

2 alleles

98.8% missing data

4	(0.852) 0.851 0.849

3	(0.148) 0.149 0.151

Locus 4537 :

2 alleles

99.2% missing data

2	(0.279) 0.286 0.280

4	(0.721) 0.714 0.720

Locus 4538 :

2 alleles

99.2% missing data

4	(0.534) 0.530 0.537

2	(0.466) 0.470 0.463

Locus 4539 :

2 alleles

99.2% missing data

3	(0.600) 0.598 0.602

2	(0.400) 0.402 0.398

Locus 4540 :

2 alleles

98.8% missing data

2	(0.750) 0.747 0.755

4	(0.250) 0.253 0.245

Locus 4541 :

2 alleles

99.2% missing data

3	(0.269) 0.274 0.267

4	(0.731) 0.726 0.733

Locus 4542 :

2 alleles

99.2% missing data

4	(0.197) 0.200 0.200

3	(0.803) 0.800 0.800

Locus 4543 :

2 alleles

91.0% missing data

3	(0.904) 0.922 0.895

2	(0.096) 0.078 0.105

Locus 4544 :

2 alleles

98.4% missing data

4	(0.656) 0.664 0.649

3	(0.344) 0.336 0.351

Locus 4545 :

2 alleles

99.2% missing data

3	(0.466) 0.474 0.465

2	(0.534) 0.526 0.535

Locus 4546 :

2 alleles

99.2% missing data

4	(0.485) 0.479 0.494

2	(0.515) 0.521 0.506

Locus 4547 :

2 alleles

98.8% missing data

2	(0.725) 0.728 0.726

3	(0.275) 0.272 0.274

Locus 4548 :

2 alleles

98.8% missing data

2	(0.685) 0.689 0.678

1	(0.315) 0.311 0.322

Locus 4549 :

2 alleles

96.1% missing data

1	(0.812) 0.814 0.817

2	(0.188) 0.186 0.183

Locus 4550 :

2 alleles

96.9% missing data

4	(0.849) 0.845 0.861

2	(0.151) 0.155 0.139

Locus 4551 :

2 alleles

98.8% missing data

1	(0.491) 0.487 0.500

2	(0.509) 0.513 0.500

Locus 4552 :

2 alleles

99.2% missing data

1	(0.504) 0.494 0.508

2	(0.496) 0.506 0.492

Locus 4553 :

2 alleles

99.2% missing data

1	(0.540) 0.537 0.546

3	(0.460) 0.463 0.454

Locus 4554 :

2 alleles

99.2% missing data

3	(0.401) 0.396 0.405

4	(0.599) 0.604 0.595

Locus 4555 :

2 alleles

99.2% missing data

2	(0.395) 0.400 0.390

4	(0.605) 0.600 0.610

Locus 4556 :

2 alleles

99.2% missing data

4	(0.630) 0.626 0.636

2	(0.370) 0.374 0.364

Locus 4557 :

2 alleles

99.2% missing data

3	(0.537) 0.546 0.531

1	(0.463) 0.454 0.469

Locus 4558 :

2 alleles

99.2% missing data

2	(0.570) 0.573 0.570

3	(0.430) 0.427 0.430

Locus 4559 :

2 alleles

99.2% missing data

4	(0.485) 0.482 0.484

3	(0.515) 0.518 0.516

Locus 4560 :

2 alleles

99.2% missing data

2	(0.449) 0.456 0.446

1	(0.551) 0.544 0.554

Locus 4561 :

2 alleles

90.2% missing data

3	(0.914) 0.930 0.906

2	(0.086) 0.070 0.094

Locus 4562 :

2 alleles

98.8% missing data

3	(0.527) 0.521 0.529

1	(0.473) 0.479 0.471

Locus 4563 :

2 alleles

98.8% missing data

3	(0.737) 0.744 0.736

1	(0.263) 0.256 0.264

Locus 4564 :

2 alleles

98.4% missing data

4	(0.753) 0.759 0.749

2	(0.247) 0.241 0.251

Locus 4565 :

2 alleles

99.2% missing data

4	(0.595) 0.597 0.597

2	(0.405) 0.403 0.403

Locus 4566 :

2 alleles

99.2% missing data

3	(0.566) 0.558 0.570

1	(0.434) 0.442 0.430

Locus 4567 :

2 alleles

98.4% missing data

4	(0.612) 0.612 0.617

1	(0.388) 0.388 0.383

Locus 4568 :

2 alleles

98.8% missing data

1	(0.432) 0.424 0.441

4	(0.568) 0.576 0.559

Locus 4569 :

2 alleles

99.2% missing data

4	(0.282) 0.282 0.284

2	(0.718) 0.718 0.716

Locus 4570 :

2 alleles

98.8% missing data

2	(0.467) 0.463 0.465

3	(0.533) 0.537 0.535

Locus 4571 :

2 alleles

92.6% missing data

3	(0.909) 0.912 0.916

1	(0.091) 0.088 0.084

Locus 4572 :

2 alleles

99.2% missing data

4	(0.745) 0.740 0.747

3	(0.255) 0.260 0.253

Locus 4573 :

2 alleles

99.2% missing data

4	(0.740) 0.738 0.733

3	(0.260) 0.262 0.267

Locus 4574 :

2 alleles

99.2% missing data

2	(0.408) 0.408 0.406

3	(0.592) 0.592 0.594

Locus 4575 :

3 alleles

98.0% missing data

3	(0.494) 0.492 0.496

2	(0.194) 0.186 0.198

1	(0.312) 0.322 0.306

Locus 4576 :

2 alleles

99.2% missing data

1	(0.480) 0.472 0.483

4	(0.520) 0.528 0.517

Locus 4577 :

2 alleles

99.2% missing data

4	(0.523) 0.527 0.524

1	(0.477) 0.473 0.476

Locus 4578 :

3 alleles

98.8% missing data

1	(0.562) 0.557 0.563

2	(0.186) 0.191 0.186

4	(0.252) 0.252 0.250

Locus 4579 :

2 alleles

99.2% missing data

2	(0.724) 0.725 0.723

1	(0.276) 0.275 0.277

Locus 4580 :

2 alleles

99.2% missing data

1	(0.759) 0.756 0.762

3	(0.241) 0.244 0.238

Locus 4581 :

2 alleles

99.2% missing data

1	(0.486) 0.480 0.491

4	(0.514) 0.520 0.509

Locus 4582 :

2 alleles

98.8% missing data

2	(0.671) 0.669 0.675

1	(0.329) 0.331 0.325

Locus 4583 :

2 alleles

99.2% missing data

2	(0.236) 0.230 0.244

4	(0.764) 0.770 0.756

Locus 4584 :

2 alleles

98.8% missing data

1	(0.572) 0.566 0.577

3	(0.428) 0.434 0.423

Locus 4585 :

2 alleles

98.0% missing data

1	(0.548) 0.557 0.542

2	(0.452) 0.443 0.458

Locus 4586 :

2 alleles

99.2% missing data

3	(0.525) 0.525 0.532

1	(0.475) 0.475 0.468

Locus 4587 :

2 alleles

99.2% missing data

2	(0.371) 0.376 0.372

3	(0.629) 0.624 0.628

Locus 4588 :

2 alleles

99.2% missing data

1	(0.562) 0.568 0.566

4	(0.438) 0.432 0.434

Locus 4589 :

2 alleles

95.7% missing data

2	(0.804) 0.813 0.801

4	(0.196) 0.187 0.199

Locus 4590 :

2 alleles

96.1% missing data

4	(0.821) 0.825 0.827

1	(0.179) 0.175 0.173

Locus 4591 :

2 alleles

98.4% missing data

4	(0.385) 0.399 0.384

1	(0.615) 0.601 0.616

Locus 4592 :

2 alleles

0.0% missing data

3	(0.982) 0.986 0.991

2	(0.018) 0.014 0.009

Locus 4593 :

2 alleles

96.1% missing data

2	(0.836) 0.849 0.830

4	(0.164) 0.151 0.170

Locus 4594 :

2 alleles

99.2% missing data

1	(0.417) 0.418 0.415

2	(0.583) 0.582 0.585

Locus 4595 :

2 alleles

99.2% missing data

1	(0.473) 0.476 0.477

3	(0.527) 0.524 0.523

Locus 4596 :

2 alleles

99.2% missing data

3	(0.272) 0.264 0.278

4	(0.728) 0.736 0.722

Locus 4597 :

2 alleles

99.2% missing data

1	(0.740) 0.736 0.744

2	(0.260) 0.264 0.256

Locus 4598 :

2 alleles

99.2% missing data

3	(0.637) 0.640 0.637

4	(0.363) 0.360 0.363

Locus 4599 :

3 alleles

98.4% missing data

2	(0.317) 0.308 0.322

3	(0.335) 0.340 0.332

4	(0.347) 0.352 0.346

Locus 4600 :

2 alleles

99.2% missing data

2	(0.570) 0.580 0.570

1	(0.430) 0.420 0.430

Locus 4601 :

2 alleles

99.2% missing data

4	(0.517) 0.509 0.521

3	(0.483) 0.491 0.479

Locus 4602 :

2 alleles

99.2% missing data

2	(0.762) 0.760 0.761

1	(0.238) 0.240 0.239

Locus 4603 :

2 alleles

99.2% missing data

1	(0.697) 0.694 0.700

2	(0.303) 0.306 0.300

Locus 4604 :

2 alleles

99.2% missing data

4	(0.242) 0.249 0.239

2	(0.758) 0.751 0.761

Locus 4605 :

2 alleles

99.2% missing data

1	(0.423) 0.415 0.430

4	(0.577) 0.585 0.570

Locus 4606 :

2 alleles

96.1% missing data

2	(0.847) 0.857 0.841

1	(0.153) 0.143 0.159

Locus 4607 :

2 alleles

99.2% missing data

3	(0.382) 0.380 0.375

1	(0.618) 0.620 0.625

Locus 4608 :

2 alleles

99.2% missing data

4	(0.465) 0.470 0.465

2	(0.535) 0.530 0.535

Locus 4609 :

2 alleles

99.2% missing data

1	(0.423) 0.427 0.420

3	(0.577) 0.573 0.580

Locus 4610 :

2 alleles

99.2% missing data

3	(0.785) 0.782 0.790

2	(0.215) 0.218 0.210

Locus 4611 :

2 alleles

96.5% missing data

2	(0.676) 0.682 0.682

4	(0.324) 0.318 0.318

Locus 4612 :

2 alleles

99.2% missing data

3	(0.600) 0.598 0.602

1	(0.400) 0.402 0.398

Locus 4613 :

2 alleles

97.3% missing data

4	(0.753) 0.758 0.752

1	(0.247) 0.242 0.248

Locus 4614 :

2 alleles

99.2% missing data

2	(0.514) 0.508 0.520

1	(0.486) 0.492 0.480

Locus 4615 :

2 alleles

99.2% missing data

2	(0.349) 0.351 0.349

4	(0.651) 0.649 0.651

Locus 4616 :

2 alleles

99.2% missing data

4	(0.424) 0.429 0.424

1	(0.576) 0.571 0.576

Locus 4617 :

2 alleles

98.0% missing data

3	(0.536) 0.524 0.544

4	(0.464) 0.476 0.456

Locus 4618 :

2 alleles

98.8% missing data

2	(0.531) 0.531 0.535

3	(0.469) 0.469 0.465

Locus 4619 :

2 alleles

98.8% missing data

3	(0.561) 0.565 0.562

4	(0.439) 0.435 0.438

Locus 4620 :

2 alleles

99.2% missing data

2	(0.136) 0.133 0.143

1	(0.864) 0.867 0.857

Locus 4621 :

2 alleles

99.2% missing data

4	(0.590) 0.591 0.590

1	(0.410) 0.409 0.410

Locus 4622 :

2 alleles

98.8% missing data

2	(0.490) 0.482 0.508

1	(0.510) 0.518 0.492

Locus 4623 :

3 alleles

98.8% missing data

2	(0.315) 0.316 0.308

4	(0.308) 0.314 0.309

3	(0.377) 0.370 0.383

Locus 4624 :

2 alleles

95.3% missing data

3	(0.896) 0.888 0.905

4	(0.104) 0.112 0.095

Locus 4625 :

3 alleles

98.8% missing data

3	(0.387) 0.379 0.396

1	(0.347) 0.352 0.342

4	(0.266) 0.269 0.261

Locus 4626 :

2 alleles

99.2% missing data

1	(0.278) 0.274 0.285

4	(0.722) 0.726 0.715

Locus 4627 :

2 alleles

90.2% missing data

4	(0.916) 0.930 0.909

3	(0.084) 0.070 0.091

Locus 4628 :

2 alleles

98.8% missing data

4	(0.520) 0.518 0.527

2	(0.480) 0.482 0.473

Locus 4629 :

2 alleles

99.2% missing data

3	(0.492) 0.483 0.498

1	(0.508) 0.517 0.502

Locus 4630 :

2 alleles

99.2% missing data

4	(0.632) 0.632 0.631

2	(0.368) 0.368 0.369

Locus 4631 :

2 alleles

98.4% missing data

4	(0.620) 0.626 0.617

3	(0.380) 0.374 0.383

Locus 4632 :

2 alleles

99.2% missing data

4	(0.757) 0.753 0.761

2	(0.243) 0.247 0.239

Locus 4633 :

2 alleles

98.4% missing data

1	(0.386) 0.375 0.395

2	(0.614) 0.625 0.605

Locus 4634 :

2 alleles

99.2% missing data

4	(0.775) 0.768 0.778

3	(0.225) 0.232 0.222

Locus 4635 :

2 alleles

96.1% missing data

2	(0.192) 0.183 0.191

3	(0.808) 0.817 0.809

Locus 4636 :

2 alleles

99.2% missing data

4	(0.501) 0.499 0.500

1	(0.499) 0.501 0.500

Locus 4637 :

2 alleles

98.8% missing data

4	(0.742) 0.738 0.739

2	(0.258) 0.262 0.261

Locus 4638 :

2 alleles

99.2% missing data

3	(0.677) 0.672 0.680

4	(0.323) 0.328 0.320

Locus 4639 :

2 alleles

99.2% missing data

1	(0.601) 0.595 0.601

3	(0.399) 0.405 0.399

Locus 4640 :

3 alleles

98.8% missing data

2	(0.227) 0.226 0.228

1	(0.553) 0.555 0.546

4	(0.219) 0.219 0.226

Locus 4641 :

2 alleles

99.2% missing data

3	(0.520) 0.520 0.515

1	(0.480) 0.480 0.485

Locus 4642 :

2 alleles

99.2% missing data

1	(0.726) 0.722 0.729

4	(0.274) 0.278 0.271

Locus 4643 :

2 alleles

93.0% missing data

4	(0.866) 0.868 0.875

3	(0.134) 0.132 0.125

Locus 4644 :

2 alleles

99.2% missing data

2	(0.442) 0.435 0.448

3	(0.558) 0.565 0.552

Locus 4645 :

3 alleles

96.5% missing data

4	(0.144) 0.135 0.146

3	(0.675) 0.695 0.671

2	(0.181) 0.170 0.184

Locus 4646 :

2 alleles

99.2% missing data

1	(0.663) 0.662 0.663

4	(0.337) 0.338 0.337

Locus 4647 :

2 alleles

92.2% missing data

4	(0.843) 0.836 0.856

1	(0.157) 0.164 0.144

Locus 4648 :

2 alleles

99.2% missing data

2	(0.388) 0.381 0.389

4	(0.612) 0.619 0.611

Locus 4649 :

2 alleles

99.2% missing data

3	(0.420) 0.420 0.420

1	(0.580) 0.580 0.580

Locus 4650 :

2 alleles

99.2% missing data

4	(0.398) 0.397 0.404

2	(0.602) 0.603 0.596

Locus 4651 :

2 alleles

99.2% missing data

3	(0.572) 0.564 0.583

2	(0.428) 0.436 0.417

Locus 4652 :

2 alleles

95.7% missing data

2	(0.876) 0.867 0.891

3	(0.124) 0.133 0.109

Locus 4653 :

2 alleles

99.2% missing data

4	(0.323) 0.319 0.333

2	(0.677) 0.681 0.667

Locus 4654 :

2 alleles

99.2% missing data

3	(0.291) 0.295 0.297

4	(0.709) 0.705 0.703

Locus 4655 :

2 alleles

99.2% missing data

4	(0.465) 0.460 0.474

2	(0.535) 0.540 0.526

Locus 4656 :

2 alleles

98.8% missing data

1	(0.787) 0.789 0.783

4	(0.213) 0.211 0.217

Locus 4657 :

3 alleles

98.8% missing data

4	(0.375) 0.375 0.375

1	(0.267) 0.269 0.267

2	(0.358) 0.356 0.358

Locus 4658 :

2 alleles

95.3% missing data

2	(0.850) 0.852 0.852

3	(0.150) 0.148 0.148

Locus 4659 :

2 alleles

99.2% missing data

2	(0.439) 0.441 0.447

1	(0.561) 0.559 0.553

Locus 4660 :

2 alleles

83.6% missing data

3	(0.872) 0.873 0.885

1	(0.128) 0.127 0.115

Locus 4661 :

2 alleles

96.5% missing data

3	(0.820) 0.812 0.833

4	(0.180) 0.188 0.167

Locus 4662 :

2 alleles

98.8% missing data

3	(0.811) 0.810 0.810

2	(0.189) 0.190 0.190

Locus 4663 :

2 alleles

92.6% missing data

4	(0.933) 0.925 0.947

3	(0.067) 0.075 0.053

Locus 4664 :

2 alleles

99.2% missing data

4	(0.698) 0.700 0.695

1	(0.302) 0.300 0.305

Locus 4665 :

2 alleles

99.2% missing data

4	(0.606) 0.605 0.606

2	(0.394) 0.395 0.394

Locus 4666 :

2 alleles

98.4% missing data

1	(0.390) 0.386 0.384

3	(0.610) 0.614 0.616

Locus 4667 :

3 alleles

98.8% missing data

3	(0.217) 0.213 0.221

1	(0.467) 0.471 0.470

2	(0.316) 0.316 0.309

Locus 4668 :

2 alleles

99.2% missing data

4	(0.713) 0.707 0.717

3	(0.287) 0.293 0.283

Locus 4669 :

2 alleles

99.2% missing data

4	(0.465) 0.463 0.469

3	(0.535) 0.537 0.531

Locus 4670 :

2 alleles

99.2% missing data

2	(0.377) 0.369 0.381

1	(0.623) 0.631 0.619

Locus 4671 :

2 alleles

99.2% missing data

4	(0.288) 0.287 0.292

2	(0.712) 0.713 0.708

Locus 4672 :

2 alleles

98.4% missing data

4	(0.615) 0.622 0.615

2	(0.385) 0.378 0.385

Locus 4673 :

2 alleles

99.2% missing data

1	(0.474) 0.468 0.479

3	(0.526) 0.532 0.521

Locus 4674 :

2 alleles

99.2% missing data

2	(0.761) 0.763 0.759

3	(0.239) 0.237 0.241

Locus 4675 :

2 alleles

96.9% missing data

1	(0.755) 0.751 0.767

2	(0.245) 0.249 0.233

Locus 4676 :

2 alleles

99.2% missing data

1	(0.606) 0.605 0.609

3	(0.394) 0.395 0.391

Locus 4677 :

2 alleles

98.4% missing data

3	(0.382) 0.371 0.389

2	(0.618) 0.629 0.611

Locus 4678 :

2 alleles

99.2% missing data

2	(0.553) 0.546 0.556

3	(0.447) 0.454 0.444

Locus 4679 :

2 alleles

99.2% missing data

3	(0.133) 0.130 0.139

2	(0.867) 0.870 0.861

Locus 4680 :

2 alleles

99.2% missing data

2	(0.437) 0.430 0.442

4	(0.563) 0.570 0.558

Locus 4681 :

2 alleles

99.2% missing data

2	(0.623) 0.621 0.630

4	(0.377) 0.379 0.370

Locus 4682 :

2 alleles

98.8% missing data

1	(0.391) 0.385 0.392

3	(0.609) 0.615 0.608

Locus 4683 :

2 alleles

99.2% missing data

1	(0.679) 0.681 0.676

2	(0.321) 0.319 0.324

Locus 4684 :

2 alleles

97.3% missing data

2	(0.761) 0.763 0.762

1	(0.239) 0.237 0.238

Locus 4685 :

2 alleles

99.2% missing data

4	(0.552) 0.551 0.552

3	(0.448) 0.449 0.448

Locus 4686 :

2 alleles

99.2% missing data

2	(0.281) 0.284 0.279

3	(0.719) 0.716 0.721

Locus 4687 :

2 alleles

99.2% missing data

4	(0.389) 0.387 0.394

3	(0.611) 0.613 0.606

Locus 4688 :

2 alleles

99.2% missing data

2	(0.404) 0.410 0.406

3	(0.596) 0.590 0.594

Locus 4689 :

2 alleles

99.2% missing data

2	(0.620) 0.622 0.617

4	(0.380) 0.378 0.383

Locus 4690 :

2 alleles

99.2% missing data

3	(0.559) 0.555 0.561

1	(0.441) 0.445 0.439

Locus 4691 :

2 alleles

98.8% missing data

3	(0.317) 0.314 0.322

2	(0.683) 0.686 0.678

Locus 4692 :

2 alleles

98.8% missing data

2	(0.810) 0.804 0.815

4	(0.190) 0.196 0.185

Locus 4693 :

2 alleles

99.2% missing data

3	(0.396) 0.390 0.402

1	(0.604) 0.610 0.598

Locus 4694 :

2 alleles

99.2% missing data

1	(0.586) 0.578 0.590

2	(0.414) 0.422 0.410

Locus 4695 :

2 alleles

99.2% missing data

4	(0.818) 0.817 0.817

3	(0.182) 0.183 0.183

Locus 4696 :

2 alleles

98.0% missing data

4	(0.305) 0.305 0.305

2	(0.695) 0.695 0.695

Locus 4697 :

2 alleles

0.4% missing data

3	(0.987) 0.983 0.996

4	(0.013) 0.017 0.004

Locus 4698 :

2 alleles

98.8% missing data

2	(0.362) 0.354 0.370

1	(0.638) 0.646 0.630

Locus 4699 :

2 alleles

99.2% missing data

2	(0.773) 0.770 0.776

1	(0.227) 0.230 0.224

Locus 4700 :

2 alleles

98.8% missing data

3	(0.540) 0.542 0.530

1	(0.460) 0.458 0.470

Locus 4701 :

2 alleles

99.2% missing data

1	(0.590) 0.595 0.582

4	(0.410) 0.405 0.418

Locus 4702 :

2 alleles

98.0% missing data

4	(0.416) 0.414 0.412

2	(0.584) 0.586 0.588

Locus 4703 :

2 alleles

99.2% missing data

3	(0.246) 0.238 0.249

4	(0.754) 0.762 0.751

Locus 4704 :

3 alleles

92.2% missing data

4	(0.799) 0.798 0.810

3	(0.132) 0.131 0.125

1	(0.069) 0.071 0.065

Locus 4705 :

2 alleles

96.9% missing data

1	(0.838) 0.846 0.836

4	(0.162) 0.154 0.164

Locus 4706 :

2 alleles

99.2% missing data

3	(0.291) 0.291 0.296

1	(0.709) 0.709 0.704

Locus 4707 :

2 alleles

95.7% missing data

3	(0.831) 0.820 0.839

4	(0.169) 0.180 0.161

Locus 4708 :

2 alleles

7.4% missing data

4	(0.978) 0.944 0.993

3	(0.022) 0.056 0.007

Locus 4709 :

2 alleles

98.4% missing data

2	(0.610) 0.601 0.616

4	(0.390) 0.399 0.384

Locus 4710 :

2 alleles

99.2% missing data

1	(0.775) 0.777 0.769

4	(0.225) 0.223 0.231

Locus 4711 :

2 alleles

98.8% missing data

3	(0.683) 0.686 0.683

1	(0.317) 0.314 0.317

Locus 4712 :

2 alleles

99.2% missing data

4	(0.305) 0.302 0.303

1	(0.695) 0.698 0.697

Locus 4713 :

2 alleles

91.0% missing data

1	(0.951) 0.953 0.950

4	(0.049) 0.047 0.050

Locus 4714 :

2 alleles

99.2% missing data

4	(0.363) 0.360 0.366

1	(0.637) 0.640 0.634

Locus 4715 :

2 alleles

99.2% missing data

2	(0.555) 0.551 0.559

1	(0.445) 0.449 0.441

Locus 4716 :

2 alleles

94.9% missing data

4	(0.925) 0.927 0.922

1	(0.075) 0.073 0.078

Locus 4717 :

2 alleles

91.0% missing data

1	(0.876) 0.898 0.867

3	(0.124) 0.102 0.133

Locus 4718 :

2 alleles

96.9% missing data

1	(0.835) 0.835 0.840

4	(0.165) 0.165 0.160

Locus 4719 :

2 alleles

99.2% missing data

1	(0.606) 0.608 0.607

4	(0.394) 0.392 0.393

Locus 4720 :

2 alleles

98.8% missing data

1	(0.458) 0.449 0.471

4	(0.542) 0.551 0.529

Locus 4721 :

2 alleles

99.2% missing data

3	(0.385) 0.388 0.383

4	(0.615) 0.612 0.617

Locus 4722 :

2 alleles

99.2% missing data

3	(0.488) 0.487 0.493

1	(0.512) 0.513 0.507

Locus 4723 :

2 alleles

98.8% missing data

4	(0.596) 0.590 0.602

3	(0.404) 0.410 0.398

Locus 4724 :

2 alleles

98.8% missing data

4	(0.497) 0.496 0.501

2	(0.503) 0.504 0.499

Locus 4725 :

2 alleles

99.2% missing data

2	(0.750) 0.748 0.752

1	(0.250) 0.252 0.248

Locus 4726 :

2 alleles

98.8% missing data

4	(0.504) 0.501 0.505

3	(0.496) 0.499 0.495

Locus 4727 :

2 alleles

99.2% missing data

4	(0.454) 0.453 0.452

2	(0.546) 0.547 0.548

Locus 4728 :

3 alleles

98.4% missing data

1	(0.316) 0.316 0.313

4	(0.509) 0.510 0.510

2	(0.174) 0.174 0.178

Locus 4729 :

2 alleles

99.2% missing data

1	(0.406) 0.400 0.412

2	(0.594) 0.600 0.588

Locus 4730 :

2 alleles

99.2% missing data

3	(0.566) 0.557 0.571

2	(0.434) 0.443 0.429

Locus 4731 :

2 alleles

97.7% missing data

4	(0.204) 0.210 0.198

2	(0.796) 0.790 0.802

Locus 4732 :

2 alleles

99.2% missing data

4	(0.173) 0.179 0.170

2	(0.827) 0.821 0.830

Locus 4733 :

2 alleles

99.2% missing data

4	(0.644) 0.650 0.639

1	(0.356) 0.350 0.361

Locus 4734 :

2 alleles

99.2% missing data

3	(0.491) 0.498 0.492

2	(0.509) 0.502 0.508

Locus 4735 :

2 alleles

98.8% missing data

4	(0.557) 0.556 0.560

2	(0.443) 0.444 0.440

Locus 4736 :

2 alleles

99.2% missing data

2	(0.716) 0.718 0.716

4	(0.284) 0.282 0.284

Locus 4737 :

2 alleles

99.2% missing data

2	(0.278) 0.270 0.284

3	(0.722) 0.730 0.716

Locus 4738 :

2 alleles

99.2% missing data

2	(0.761) 0.762 0.761

1	(0.239) 0.238 0.239

Locus 4739 :

2 alleles

99.2% missing data

2	(0.511) 0.506 0.515

1	(0.489) 0.494 0.485

Locus 4740 :

2 alleles

99.2% missing data

2	(0.721) 0.717 0.722

4	(0.279) 0.283 0.278

Locus 4741 :

2 alleles

99.2% missing data

3	(0.522) 0.522 0.524

1	(0.478) 0.478 0.476

Locus 4742 :

2 alleles

99.2% missing data

1	(0.493) 0.490 0.496

4	(0.507) 0.510 0.504

Locus 4743 :

2 alleles

99.2% missing data

3	(0.617) 0.614 0.607

1	(0.383) 0.386 0.393

Locus 4744 :

2 alleles

99.2% missing data

4	(0.469) 0.472 0.465

1	(0.531) 0.528 0.535

Locus 4745 :

2 alleles

98.4% missing data

2	(0.369) 0.361 0.367

4	(0.631) 0.639 0.633

Locus 4746 :

2 alleles

97.7% missing data

3	(0.811) 0.807 0.823

1	(0.189) 0.193 0.177

Locus 4747 :

2 alleles

99.2% missing data

2	(0.524) 0.524 0.525

4	(0.476) 0.476 0.475

Locus 4748 :

2 alleles

96.9% missing data

4	(0.226) 0.222 0.218

1	(0.774) 0.778 0.782

Locus 4749 :

2 alleles

99.2% missing data

4	(0.357) 0.360 0.363

1	(0.643) 0.640 0.637

Locus 4750 :

2 alleles

98.0% missing data

2	(0.803) 0.806 0.798

3	(0.197) 0.194 0.202

Locus 4751 :

2 alleles

99.2% missing data

3	(0.483) 0.485 0.485

1	(0.517) 0.515 0.515

Locus 4752 :

2 alleles

94.5% missing data

4	(0.674) 0.700 0.639

2	(0.326) 0.300 0.361

Locus 4753 :

2 alleles

98.4% missing data

1	(0.736) 0.733 0.745

4	(0.264) 0.267 0.255

Locus 4754 :

2 alleles

99.2% missing data

1	(0.577) 0.572 0.585

4	(0.423) 0.428 0.415

Locus 4755 :

2 alleles

99.2% missing data

2	(0.307) 0.305 0.311

4	(0.693) 0.695 0.689

Locus 4756 :

2 alleles

99.2% missing data

4	(0.549) 0.558 0.537

1	(0.451) 0.442 0.463

Locus 4757 :

2 alleles

97.7% missing data

4	(0.266) 0.270 0.262

2	(0.734) 0.730 0.738

Locus 4758 :

2 alleles

99.2% missing data

2	(0.247) 0.244 0.252

3	(0.753) 0.756 0.748

Locus 4759 :

2 alleles

98.8% missing data

4	(0.518) 0.513 0.523

3	(0.482) 0.487 0.477

Locus 4760 :

2 alleles

99.2% missing data

2	(0.670) 0.676 0.670

3	(0.330) 0.324 0.330

Locus 4761 :

2 alleles

99.2% missing data

4	(0.243) 0.242 0.248

3	(0.757) 0.758 0.752

Locus 4762 :

2 alleles

99.2% missing data

3	(0.691) 0.690 0.695

4	(0.309) 0.310 0.305

Locus 4763 :

2 alleles

99.2% missing data

1	(0.300) 0.297 0.301

3	(0.700) 0.703 0.699

Locus 4764 :

2 alleles

8.6% missing data

1	(0.983) 0.976 0.995

3	(0.017) 0.024 0.005

Locus 4765 :

2 alleles

97.7% missing data

4	(0.396) 0.406 0.380

2	(0.604) 0.594 0.620

Locus 4766 :

2 alleles

93.0% missing data

3	(0.895) 0.890 0.906

4	(0.105) 0.110 0.094

Locus 4767 :

2 alleles

90.2% missing data

2	(0.919) 0.927 0.920

4	(0.081) 0.073 0.080

Locus 4768 :

2 alleles

98.8% missing data

1	(0.703) 0.696 0.707

2	(0.297) 0.304 0.293

Locus 4769 :

2 alleles

97.3% missing data

3	(0.754) 0.754 0.762

4	(0.246) 0.246 0.238

Locus 4770 :

2 alleles

99.2% missing data

3	(0.473) 0.480 0.474

1	(0.527) 0.520 0.526

Locus 4771 :

2 alleles

98.8% missing data

4	(0.208) 0.210 0.206

2	(0.792) 0.790 0.794

Locus 4772 :

2 alleles

99.2% missing data

2	(0.425) 0.424 0.418

4	(0.575) 0.576 0.582

Locus 4773 :

2 alleles

97.3% missing data

2	(0.166) 0.166 0.162

3	(0.834) 0.834 0.838

Locus 4774 :

2 alleles

99.2% missing data

2	(0.613) 0.611 0.615

4	(0.387) 0.389 0.385

Locus 4775 :

2 alleles

89.5% missing data

2	(0.816) 0.849 0.786

1	(0.184) 0.151 0.214

Locus 4776 :

2 alleles

98.4% missing data

2	(0.858) 0.857 0.863

1	(0.142) 0.143 0.137

Locus 4777 :

2 alleles

92.6% missing data

3	(0.883) 0.892 0.882

4	(0.117) 0.108 0.118

Locus 4778 :

3 alleles

91.4% missing data

3	(0.766) 0.765 0.784

1	(0.173) 0.178 0.158

4	(0.061) 0.057 0.059

Locus 4779 :

2 alleles

99.2% missing data

3	(0.649) 0.643 0.646

1	(0.351) 0.357 0.354

Locus 4780 :

2 alleles

98.8% missing data

1	(0.810) 0.812 0.807

3	(0.190) 0.188 0.193

Locus 4781 :

2 alleles

99.2% missing data

3	(0.475) 0.473 0.472

1	(0.525) 0.527 0.528

Locus 4782 :

2 alleles

90.2% missing data

2	(0.772) 0.803 0.749

1	(0.228) 0.197 0.251

Locus 4783 :

3 alleles

98.8% missing data

3	(0.311) 0.307 0.313

4	(0.355) 0.361 0.348

1	(0.334) 0.332 0.339

Locus 4784 :

2 alleles

93.8% missing data

1	(0.912) 0.901 0.926

4	(0.088) 0.099 0.074

Locus 4785 :

2 alleles

99.2% missing data

2	(0.561) 0.556 0.563

4	(0.439) 0.444 0.437

Locus 4786 :

2 alleles

99.2% missing data

4	(0.275) 0.273 0.279

1	(0.725) 0.727 0.721

Locus 4787 :

2 alleles

99.2% missing data

2	(0.302) 0.310 0.302

1	(0.698) 0.690 0.698

Locus 4788 :

3 alleles

98.8% missing data

2	(0.335) 0.321 0.347

4	(0.294) 0.304 0.286

1	(0.371) 0.375 0.367

Locus 4789 :

2 alleles

99.2% missing data

1	(0.617) 0.616 0.621

3	(0.383) 0.384 0.379

Locus 4790 :

2 alleles

99.2% missing data

1	(0.418) 0.419 0.417

2	(0.582) 0.581 0.583

Locus 4791 :

2 alleles

90.2% missing data

1	(0.899) 0.922 0.895

2	(0.101) 0.078 0.105

Locus 4792 :

2 alleles

99.2% missing data

2	(0.369) 0.373 0.368

4	(0.631) 0.627 0.632

Locus 4793 :

2 alleles

97.7% missing data

4	(0.264) 0.272 0.258

1	(0.736) 0.728 0.742

Locus 4794 :

2 alleles

0.0% missing data

3	(0.989) 0.984 0.997

2	(0.011) 0.016 0.003

Locus 4795 :

2 alleles

99.2% missing data

4	(0.696) 0.704 0.688

1	(0.304) 0.296 0.312

Locus 4796 :

3 alleles

98.4% missing data

4	(0.254) 0.256 0.259

1	(0.403) 0.407 0.398

3	(0.343) 0.337 0.343

Locus 4797 :

2 alleles

91.0% missing data

4	(0.928) 0.934 0.932

3	(0.072) 0.066 0.068

Locus 4798 :

2 alleles

99.2% missing data

1	(0.326) 0.316 0.330

2	(0.674) 0.684 0.670

Locus 4799 :

2 alleles

88.7% missing data

2	(0.317) 0.346 0.270

1	(0.683) 0.654 0.730

Locus 4800 :

2 alleles

98.8% missing data

3	(0.393) 0.393 0.397

1	(0.607) 0.607 0.603

Locus 4801 :

2 alleles

96.1% missing data

4	(0.752) 0.769 0.745

3	(0.248) 0.231 0.255

Locus 4802 :

2 alleles

99.2% missing data

4	(0.581) 0.582 0.584

2	(0.419) 0.418 0.416

Locus 4803 :

2 alleles

99.2% missing data

3	(0.632) 0.638 0.633

1	(0.368) 0.362 0.367

Locus 4804 :

2 alleles

99.2% missing data

1	(0.427) 0.420 0.432

4	(0.573) 0.580 0.568

Locus 4805 :

2 alleles

98.4% missing data

4	(0.767) 0.771 0.763

3	(0.233) 0.229 0.237

Locus 4806 :

2 alleles

99.2% missing data

4	(0.681) 0.672 0.683

2	(0.319) 0.328 0.317

Locus 4807 :

2 alleles

99.2% missing data

1	(0.487) 0.481 0.497

3	(0.513) 0.519 0.503

Locus 4808 :

2 alleles

98.8% missing data

2	(0.657) 0.644 0.663

3	(0.343) 0.356 0.337

Locus 4809 :

2 alleles

99.2% missing data

4	(0.548) 0.549 0.546

1	(0.452) 0.451 0.454

Locus 4810 :

2 alleles

95.7% missing data

1	(0.892) 0.894 0.896

3	(0.108) 0.106 0.104

Locus 4811 :

2 alleles

99.2% missing data

2	(0.646) 0.636 0.649

3	(0.354) 0.364 0.351

Locus 4812 :

2 alleles

99.2% missing data

1	(0.774) 0.782 0.767

3	(0.226) 0.218 0.233

Locus 4813 :

2 alleles

99.2% missing data

1	(0.479) 0.480 0.475

4	(0.521) 0.520 0.525

Locus 4814 :

2 alleles

99.2% missing data

1	(0.635) 0.637 0.639

4	(0.365) 0.363 0.361

Locus 4815 :

2 alleles

99.2% missing data

4	(0.355) 0.358 0.353

3	(0.645) 0.642 0.647

Locus 4816 :

2 alleles

97.3% missing data

1	(0.224) 0.228 0.216

4	(0.776) 0.772 0.784

Locus 4817 :

2 alleles

98.4% missing data

4	(0.478) 0.472 0.481

3	(0.522) 0.528 0.519

Locus 4818 :

2 alleles

98.8% missing data

4	(0.785) 0.778 0.791

3	(0.215) 0.222 0.209

Locus 4819 :

2 alleles

98.8% missing data

4	(0.411) 0.407 0.409

3	(0.589) 0.593 0.591

Locus 4820 :

3 alleles

98.4% missing data

3	(0.171) 0.172 0.168

2	(0.440) 0.437 0.446

4	(0.389) 0.392 0.386

Locus 4821 :

2 alleles

99.2% missing data

4	(0.840) 0.833 0.843

3	(0.160) 0.167 0.157

Locus 4822 :

2 alleles

99.2% missing data

2	(0.225) 0.217 0.232

1	(0.775) 0.783 0.768

Locus 4823 :

2 alleles

85.9% missing data

2	(0.693) 0.753 0.617

1	(0.307) 0.247 0.383

Locus 4824 :

2 alleles

99.2% missing data

4	(0.475) 0.465 0.486

1	(0.525) 0.535 0.514

Locus 4825 :

2 alleles

97.3% missing data

1	(0.312) 0.318 0.296

3	(0.688) 0.682 0.704

Locus 4826 :

2 alleles

99.2% missing data

4	(0.692) 0.694 0.691

1	(0.308) 0.306 0.309

Locus 4827 :

2 alleles

99.2% missing data

4	(0.468) 0.469 0.466

3	(0.532) 0.531 0.534

Locus 4828 :

2 alleles

99.2% missing data

3	(0.803) 0.801 0.804

4	(0.197) 0.199 0.196

Locus 4829 :

2 alleles

96.5% missing data

4	(0.827) 0.840 0.822

3	(0.173) 0.160 0.178

Locus 4830 :

2 alleles

93.8% missing data

1	(0.895) 0.896 0.899

2	(0.105) 0.104 0.101

Locus 4831 :

2 alleles

99.2% missing data

4	(0.648) 0.642 0.658

3	(0.352) 0.358 0.342

Locus 4832 :

2 alleles

92.6% missing data

4	(0.925) 0.925 0.929

2	(0.075) 0.075 0.071

Locus 4833 :

2 alleles

8.2% missing data

2	(0.982) 0.975 0.994

3	(0.018) 0.025 0.006

Locus 4834 :

2 alleles

98.0% missing data

4	(0.691) 0.704 0.686

2	(0.309) 0.296 0.314

Locus 4835 :

2 alleles

99.2% missing data

2	(0.391) 0.387 0.394

3	(0.609) 0.613 0.606

Locus 4836 :

2 alleles

99.2% missing data

4	(0.696) 0.694 0.687

1	(0.304) 0.306 0.313

Locus 4837 :

2 alleles

99.2% missing data

4	(0.511) 0.505 0.508

1	(0.489) 0.495 0.492

Locus 4838 :

2 alleles

99.2% missing data

2	(0.557) 0.556 0.560

4	(0.443) 0.444 0.440

Locus 4839 :

3 alleles

97.3% missing data

4	(0.325) 0.338 0.317

2	(0.374) 0.372 0.386

3	(0.301) 0.290 0.297

Locus 4840 :

2 alleles

99.2% missing data

1	(0.674) 0.676 0.669

4	(0.326) 0.324 0.331

Locus 4841 :

2 alleles

99.2% missing data

1	(0.334) 0.332 0.332

3	(0.666) 0.668 0.668

Locus 4842 :

2 alleles

96.1% missing data

1	(0.894) 0.903 0.891

2	(0.106) 0.097 0.109

Locus 4843 :

2 alleles

99.2% missing data

4	(0.578) 0.580 0.568

2	(0.422) 0.420 0.432

Locus 4844 :

2 alleles

99.2% missing data

3	(0.501) 0.500 0.497

1	(0.499) 0.500 0.503

Locus 4845 :

2 alleles

99.2% missing data

1	(0.259) 0.264 0.258

2	(0.741) 0.736 0.742

Locus 4846 :

2 alleles

8.6% missing data

4	(0.964) 0.927 0.989

3	(0.036) 0.073 0.011

Locus 4847 :

2 alleles

99.2% missing data

2	(0.259) 0.259 0.262

4	(0.741) 0.741 0.738

Locus 4848 :

2 alleles

99.2% missing data

3	(0.509) 0.509 0.508

4	(0.491) 0.491 0.492

Locus 4849 :

2 alleles

99.2% missing data

1	(0.317) 0.311 0.319

2	(0.683) 0.689 0.681

Locus 4850 :

2 alleles

0.0% missing data

2	(0.985) 0.982 0.996

4	(0.015) 0.018 0.004

Locus 4851 :

2 alleles

99.2% missing data

2	(0.453) 0.456 0.458

3	(0.547) 0.544 0.542

Locus 4852 :

2 alleles

99.2% missing data

4	(0.413) 0.409 0.421

2	(0.587) 0.591 0.579

Locus 4853 :

2 alleles

98.8% missing data

2	(0.682) 0.687 0.678

3	(0.318) 0.313 0.322

Locus 4854 :

2 alleles

99.2% missing data

3	(0.494) 0.496 0.493

4	(0.506) 0.504 0.507

Locus 4855 :

2 alleles

99.2% missing data

3	(0.422) 0.423 0.423

1	(0.578) 0.577 0.577

Locus 4856 :

2 alleles

99.2% missing data

4	(0.476) 0.472 0.487

3	(0.524) 0.528 0.513

Locus 4857 :

2 alleles

98.4% missing data

1	(0.642) 0.649 0.637

2	(0.358) 0.351 0.363

Locus 4858 :

2 alleles

87.1% missing data

4	(0.697) 0.709 0.681

3	(0.303) 0.291 0.319

Locus 4859 :

3 alleles

98.8% missing data

3	(0.573) 0.570 0.564

1	(0.095) 0.093 0.103

4	(0.332) 0.337 0.333

Locus 4860 :

2 alleles

98.8% missing data

2	(0.638) 0.642 0.642

3	(0.362) 0.358 0.358

Locus 4861 :

3 alleles

96.9% missing data

2	(0.280) 0.266 0.289

3	(0.099) 0.097 0.103

1	(0.621) 0.637 0.609

Locus 4862 :

2 alleles

97.3% missing data

3	(0.786) 0.782 0.798

1	(0.214) 0.218 0.202

Locus 4863 :

2 alleles

91.0% missing data

2	(0.914) 0.933 0.903

1	(0.086) 0.067 0.097

Locus 4864 :

2 alleles

99.2% missing data

2	(0.488) 0.492 0.482

4	(0.512) 0.508 0.518

Locus 4865 :

2 alleles

99.2% missing data

2	(0.561) 0.557 0.562

4	(0.439) 0.443 0.438

Locus 4866 :

2 alleles

99.2% missing data

3	(0.521) 0.515 0.516

4	(0.479) 0.485 0.484

Locus 4867 :

2 alleles

99.2% missing data

2	(0.424) 0.423 0.431

1	(0.576) 0.577 0.569

Locus 4868 :

2 alleles

99.2% missing data

2	(0.597) 0.594 0.598

4	(0.403) 0.406 0.402

Locus 4869 :

2 alleles

98.8% missing data

1	(0.393) 0.384 0.401

2	(0.607) 0.616 0.599

Locus 4870 :

2 alleles

98.4% missing data

1	(0.716) 0.714 0.720

2	(0.284) 0.286 0.280

Locus 4871 :

3 alleles

95.7% missing data

3	(0.810) 0.802 0.810

2	(0.099) 0.103 0.100

1	(0.091) 0.094 0.090

Locus 4872 :

2 alleles

96.9% missing data

4	(0.843) 0.845 0.844

3	(0.157) 0.155 0.156

Locus 4873 :

2 alleles

99.2% missing data

4	(0.581) 0.583 0.579

3	(0.419) 0.417 0.421

Locus 4874 :

2 alleles

97.7% missing data

3	(0.389) 0.369 0.398

2	(0.611) 0.631 0.602

Locus 4875 :

2 alleles

99.2% missing data

4	(0.719) 0.725 0.714

3	(0.281) 0.275 0.286

Locus 4876 :

2 alleles

90.6% missing data

2	(0.912) 0.928 0.904

4	(0.088) 0.072 0.096

Locus 4877 :

3 alleles

97.7% missing data

3	(0.236) 0.230 0.241

4	(0.443) 0.446 0.446

2	(0.321) 0.324 0.313

Locus 4878 :

2 alleles

87.1% missing data

4	(0.659) 0.719 0.601

2	(0.341) 0.281 0.399

Locus 4879 :

3 alleles

96.5% missing data

3	(0.597) 0.599 0.613

1	(0.177) 0.174 0.175

4	(0.226) 0.227 0.213

Locus 4880 :

2 alleles

99.2% missing data

3	(0.490) 0.493 0.483

2	(0.510) 0.507 0.517

Locus 4881 :

2 alleles

99.2% missing data

4	(0.591) 0.592 0.586

2	(0.409) 0.408 0.414

Locus 4882 :

2 alleles

99.2% missing data

1	(0.654) 0.652 0.661

3	(0.346) 0.348 0.339

Locus 4883 :

3 alleles

96.5% missing data

1	(0.657) 0.678 0.652

3	(0.232) 0.217 0.233

4	(0.111) 0.105 0.115

Locus 4884 :

3 alleles

98.8% missing data

4	(0.233) 0.240 0.233

2	(0.240) 0.235 0.245

3	(0.527) 0.524 0.522

Locus 4885 :

2 alleles

99.2% missing data

2	(0.330) 0.323 0.336

3	(0.670) 0.677 0.664

Locus 4886 :

2 alleles

97.3% missing data

3	(0.732) 0.728 0.745

2	(0.268) 0.272 0.255

Locus 4887 :

2 alleles

97.7% missing data

4	(0.184) 0.186 0.179

1	(0.816) 0.814 0.821

Locus 4888 :

2 alleles

98.8% missing data

2	(0.274) 0.274 0.268

1	(0.726) 0.726 0.732

Locus 4889 :

2 alleles

99.2% missing data

3	(0.541) 0.544 0.536

1	(0.459) 0.456 0.464

Locus 4890 :

2 alleles

99.2% missing data

2	(0.669) 0.667 0.675

1	(0.331) 0.333 0.325

Locus 4891 :

2 alleles

99.2% missing data

1	(0.352) 0.351 0.351

3	(0.648) 0.649 0.649

Locus 4892 :

3 alleles

98.8% missing data

2	(0.431) 0.431 0.435

3	(0.226) 0.227 0.227

4	(0.343) 0.342 0.337

Locus 4893 :

2 alleles

96.5% missing data

2	(0.154) 0.151 0.158

4	(0.846) 0.849 0.842

Locus 4894 :

2 alleles

99.2% missing data

1	(0.544) 0.544 0.544

2	(0.456) 0.456 0.456

Locus 4895 :

2 alleles

99.2% missing data

1	(0.632) 0.628 0.631

4	(0.368) 0.372 0.369

Locus 4896 :

2 alleles

99.2% missing data

4	(0.392) 0.393 0.388

1	(0.608) 0.607 0.612

Locus 4897 :

2 alleles

97.7% missing data

4	(0.401) 0.395 0.390

2	(0.599) 0.605 0.610

Locus 4898 :

2 alleles

99.2% missing data

1	(0.586) 0.578 0.579

3	(0.414) 0.422 0.421

Locus 4899 :

2 alleles

99.2% missing data

1	(0.257) 0.252 0.262

4	(0.743) 0.748 0.738

Locus 4900 :

2 alleles

98.8% missing data

2	(0.637) 0.634 0.636

4	(0.363) 0.366 0.364

Locus 4901 :

2 alleles

99.2% missing data

4	(0.543) 0.546 0.543

1	(0.457) 0.454 0.457

Locus 4902 :

2 alleles

99.2% missing data

2	(0.277) 0.285 0.274

4	(0.723) 0.715 0.726

Locus 4903 :

2 alleles

98.8% missing data

1	(0.572) 0.572 0.574

4	(0.428) 0.428 0.426

Locus 4904 :

2 alleles

99.2% missing data

4	(0.570) 0.571 0.573

3	(0.430) 0.429 0.427

Locus 4905 :

3 alleles

98.8% missing data

2	(0.255) 0.250 0.258

4	(0.195) 0.188 0.201

3	(0.550) 0.561 0.540

Locus 4906 :

2 alleles

97.3% missing data

1	(0.710) 0.707 0.713

4	(0.290) 0.293 0.287

Locus 4907 :

2 alleles

99.2% missing data

4	(0.452) 0.450 0.450

2	(0.548) 0.550 0.550

Locus 4908 :

2 alleles

99.2% missing data

4	(0.339) 0.342 0.339

3	(0.661) 0.658 0.661

Locus 4909 :

3 alleles

98.8% missing data

3	(0.648) 0.653 0.641

2	(0.158) 0.153 0.161

1	(0.194) 0.194 0.198

Locus 4910 :

2 alleles

99.2% missing data

4	(0.586) 0.580 0.587

2	(0.414) 0.420 0.413

Locus 4911 :

2 alleles

99.2% missing data

2	(0.593) 0.598 0.583

1	(0.407) 0.402 0.417

Locus 4912 :

2 alleles

98.8% missing data

1	(0.806) 0.802 0.805

3	(0.194) 0.198 0.195

Locus 4913 :

2 alleles

99.2% missing data

4	(0.631) 0.629 0.631

3	(0.369) 0.371 0.369

Locus 4914 :

2 alleles

93.4% missing data

1	(0.826) 0.826 0.844

4	(0.174) 0.174 0.156

Locus 4915 :

2 alleles

98.0% missing data

4	(0.725) 0.720 0.733

3	(0.275) 0.280 0.267

Locus 4916 :

2 alleles

91.4% missing data

2	(0.906) 0.912 0.909

4	(0.094) 0.088 0.091

Locus 4917 :

2 alleles

99.2% missing data

1	(0.495) 0.498 0.493

4	(0.505) 0.502 0.507

Locus 4918 :

2 alleles

2.7% missing data

4	(0.982) 0.978 0.991

1	(0.018) 0.022 0.009

Locus 4919 :

2 alleles

99.2% missing data

2	(0.663) 0.661 0.663

4	(0.337) 0.339 0.337

Locus 4920 :

2 alleles

99.2% missing data

4	(0.504) 0.503 0.508

1	(0.496) 0.497 0.492

Locus 4921 :

2 alleles

98.8% missing data

3	(0.614) 0.618 0.606

4	(0.386) 0.382 0.394

Locus 4922 :

2 alleles

99.2% missing data

2	(0.350) 0.349 0.358

1	(0.650) 0.651 0.642

Locus 4923 :

2 alleles

99.2% missing data

1	(0.618) 0.621 0.625

3	(0.382) 0.379 0.375

Locus 4924 :

2 alleles

99.2% missing data

3	(0.589) 0.591 0.589

1	(0.411) 0.409 0.411

Locus 4925 :

2 alleles

91.0% missing data

3	(0.919) 0.933 0.913

2	(0.081) 0.067 0.087

Locus 4926 :

2 alleles

99.2% missing data

4	(0.524) 0.518 0.526

3	(0.476) 0.482 0.474

Locus 4927 :

2 alleles

99.2% missing data

1	(0.494) 0.490 0.498

2	(0.506) 0.510 0.502

Locus 4928 :

2 alleles

99.2% missing data

3	(0.515) 0.519 0.513

1	(0.485) 0.481 0.487

Locus 4929 :

3 alleles

98.0% missing data

1	(0.281) 0.280 0.285

4	(0.454) 0.456 0.444

2	(0.265) 0.264 0.271

Locus 4930 :

2 alleles

99.2% missing data

2	(0.542) 0.543 0.536

3	(0.458) 0.457 0.464

Locus 4931 :

3 alleles

98.8% missing data

3	(0.371) 0.364 0.365

1	(0.316) 0.318 0.317

4	(0.313) 0.318 0.317

Locus 4932 :

2 alleles

99.2% missing data

4	(0.512) 0.504 0.520

3	(0.488) 0.496 0.480

Locus 4933 :

2 alleles

99.2% missing data

4	(0.497) 0.504 0.491

1	(0.503) 0.496 0.509

Locus 4934 :

2 alleles

98.8% missing data

2	(0.347) 0.344 0.347

4	(0.653) 0.656 0.653

Locus 4935 :

2 alleles

99.2% missing data

2	(0.359) 0.356 0.357

3	(0.641) 0.644 0.643

Locus 4936 :

3 alleles

96.9% missing data

2	(0.176) 0.180 0.172

3	(0.273) 0.266 0.273

4	(0.551) 0.554 0.555

Locus 4937 :

2 alleles

91.0% missing data

3	(0.911) 0.928 0.903

1	(0.089) 0.072 0.097

Locus 4938 :

2 alleles

94.5% missing data

4	(0.886) 0.886 0.891

3	(0.114) 0.114 0.109

Locus 4939 :

2 alleles

99.2% missing data

2	(0.365) 0.365 0.367

4	(0.635) 0.635 0.633

Locus 4940 :

2 alleles

98.8% missing data

4	(0.635) 0.634 0.637

2	(0.365) 0.366 0.363

Locus 4941 :

3 alleles

98.4% missing data

2	(0.263) 0.256 0.260

4	(0.359) 0.365 0.363

3	(0.378) 0.379 0.377

Locus 4942 :

2 alleles

99.2% missing data

2	(0.389) 0.393 0.386

4	(0.611) 0.607 0.614

Locus 4943 :

2 alleles

97.3% missing data

4	(0.770) 0.764 0.778

3	(0.230) 0.236 0.222

Locus 4944 :

2 alleles

99.2% missing data

4	(0.759) 0.751 0.763

2	(0.241) 0.249 0.237

Locus 4945 :

2 alleles

96.5% missing data

1	(0.790) 0.792 0.798

4	(0.210) 0.208 0.202

Locus 4946 :

2 alleles

99.2% missing data

3	(0.263) 0.264 0.261

4	(0.737) 0.736 0.739

Locus 4947 :

2 alleles

99.2% missing data

4	(0.703) 0.702 0.701

3	(0.297) 0.298 0.299

Locus 4948 :

2 alleles

90.6% missing data

3	(0.921) 0.936 0.914

4	(0.079) 0.064 0.086

Locus 4949 :

2 alleles

96.5% missing data

1	(0.153) 0.144 0.165

2	(0.847) 0.856 0.835

Locus 4950 :

2 alleles

99.2% missing data

3	(0.191) 0.193 0.193

1	(0.809) 0.807 0.807

Locus 4951 :

2 alleles

98.8% missing data

1	(0.128) 0.127 0.132

4	(0.872) 0.873 0.868

Locus 4952 :

2 alleles

98.4% missing data

4	(0.544) 0.552 0.543

2	(0.456) 0.448 0.457

Locus 4953 :

2 alleles

99.2% missing data

4	(0.658) 0.653 0.666

3	(0.342) 0.347 0.334

Locus 4954 :

2 alleles

98.8% missing data

4	(0.841) 0.839 0.846

2	(0.159) 0.161 0.154

Locus 4955 :

2 alleles

99.2% missing data

3	(0.567) 0.562 0.571

2	(0.433) 0.438 0.429

Locus 4956 :

2 alleles

99.2% missing data

4	(0.433) 0.431 0.437

1	(0.567) 0.569 0.563

Locus 4957 :

2 alleles

99.2% missing data

1	(0.792) 0.792 0.787

2	(0.208) 0.208 0.213

Locus 4958 :

2 alleles

99.2% missing data

2	(0.353) 0.352 0.354

4	(0.647) 0.648 0.646

Locus 4959 :

2 alleles

98.8% missing data

2	(0.401) 0.402 0.400

3	(0.599) 0.598 0.600

Locus 4960 :

2 alleles

98.8% missing data

2	(0.710) 0.715 0.710

4	(0.290) 0.285 0.290

Locus 4961 :

2 alleles

99.2% missing data

1	(0.229) 0.233 0.223

4	(0.771) 0.767 0.777

Locus 4962 :

2 alleles

99.2% missing data

3	(0.533) 0.532 0.529

4	(0.467) 0.468 0.471

Locus 4963 :

2 alleles

99.2% missing data

2	(0.351) 0.342 0.361

4	(0.649) 0.658 0.639

Locus 4964 :

2 alleles

98.8% missing data

2	(0.517) 0.519 0.511

1	(0.483) 0.481 0.489

Locus 4965 :

2 alleles

99.2% missing data

4	(0.786) 0.779 0.789

1	(0.214) 0.221 0.211

Locus 4966 :

2 alleles

99.2% missing data

4	(0.382) 0.380 0.385

2	(0.618) 0.620 0.615

Locus 4967 :

2 alleles

99.2% missing data

3	(0.678) 0.683 0.670

4	(0.322) 0.317 0.330

Locus 4968 :

2 alleles

98.8% missing data

4	(0.576) 0.586 0.571

1	(0.424) 0.414 0.429

Locus 4969 :

2 alleles

98.4% missing data

1	(0.672) 0.665 0.682

3	(0.328) 0.335 0.318

Locus 4970 :

2 alleles

0.0% missing data

4	(0.989) 0.984 0.997

1	(0.011) 0.016 0.003

Locus 4971 :

2 alleles

99.2% missing data

4	(0.747) 0.738 0.751

1	(0.253) 0.262 0.249

Locus 4972 :

2 alleles

99.2% missing data

2	(0.621) 0.621 0.618

3	(0.379) 0.379 0.382

Locus 4973 :

2 alleles

99.2% missing data

3	(0.604) 0.598 0.602

1	(0.396) 0.402 0.398

Locus 4974 :

2 alleles

99.2% missing data

3	(0.352) 0.353 0.353

4	(0.648) 0.647 0.647

Locus 4975 :

3 alleles

98.4% missing data

1	(0.448) 0.438 0.452

3	(0.308) 0.313 0.305

2	(0.244) 0.250 0.242

Locus 4976 :

2 alleles

99.2% missing data

3	(0.374) 0.364 0.377

1	(0.626) 0.636 0.623

Locus 4977 :

3 alleles

92.2% missing data

4	(0.908) 0.902 0.918

3	(0.042) 0.040 0.041

2	(0.050) 0.058 0.041

Locus 4978 :

2 alleles

99.2% missing data

4	(0.434) 0.437 0.432

3	(0.566) 0.563 0.568

Locus 4979 :

2 alleles

99.2% missing data

3	(0.522) 0.525 0.525

1	(0.478) 0.475 0.475

Locus 4980 :

2 alleles

99.2% missing data

4	(0.503) 0.493 0.507

1	(0.497) 0.507 0.493

Locus 4981 :

2 alleles

99.2% missing data

1	(0.620) 0.621 0.614

3	(0.380) 0.379 0.386

Locus 4982 :

2 alleles

99.2% missing data

1	(0.574) 0.579 0.569

4	(0.426) 0.421 0.431

Locus 4983 :

2 alleles

98.8% missing data

1	(0.552) 0.540 0.559

2	(0.448) 0.460 0.441

Locus 4984 :

2 alleles

99.2% missing data

2	(0.751) 0.750 0.751

1	(0.249) 0.250 0.249

Locus 4985 :

2 alleles

98.4% missing data

1	(0.477) 0.484 0.478

3	(0.523) 0.516 0.522

Locus 4986 :

2 alleles

98.8% missing data

4	(0.361) 0.359 0.360

1	(0.639) 0.641 0.640

Locus 4987 :

2 alleles

99.2% missing data

1	(0.665) 0.667 0.660

4	(0.335) 0.333 0.340

Locus 4988 :

2 alleles

96.1% missing data

3	(0.161) 0.150 0.169

4	(0.839) 0.850 0.831

Locus 4989 :

2 alleles

99.2% missing data

4	(0.659) 0.658 0.661

2	(0.341) 0.342 0.339

Locus 4990 :

2 alleles

98.8% missing data

4	(0.513) 0.514 0.519

1	(0.487) 0.486 0.481

Locus 4991 :

2 alleles

99.2% missing data

4	(0.539) 0.544 0.538

2	(0.461) 0.456 0.462

Locus 4992 :

2 alleles

99.2% missing data

2	(0.693) 0.694 0.691

4	(0.307) 0.306 0.309

Locus 4993 :

3 alleles

98.8% missing data

3	(0.379) 0.373 0.377

2	(0.366) 0.369 0.371

4	(0.255) 0.258 0.251

Locus 4994 :

2 alleles

99.2% missing data

1	(0.375) 0.376 0.376

4	(0.625) 0.624 0.624

Locus 4995 :

2 alleles

96.9% missing data

3	(0.186) 0.190 0.181

2	(0.814) 0.810 0.819

Locus 4996 :

2 alleles

99.2% missing data

1	(0.347) 0.344 0.346

3	(0.653) 0.656 0.654

Locus 4997 :

2 alleles

93.8% missing data

1	(0.909) 0.899 0.922

2	(0.091) 0.101 0.078

Locus 4998 :

2 alleles

99.2% missing data

2	(0.532) 0.531 0.538

1	(0.468) 0.469 0.462

Locus 4999 :

2 alleles

99.2% missing data

4	(0.784) 0.780 0.789

2	(0.216) 0.220 0.211

Locus 5000 :

2 alleles

99.2% missing data

2	(0.397) 0.405 0.392

1	(0.603) 0.595 0.608

Locus 5001 :

2 alleles

99.2% missing data

3	(0.610) 0.611 0.611

1	(0.390) 0.389 0.389

Locus 5002 :

2 alleles

99.2% missing data

1	(0.454) 0.454 0.454

3	(0.546) 0.546 0.546

Locus 5003 :

2 alleles

99.2% missing data

2	(0.850) 0.848 0.850

3	(0.150) 0.152 0.150

Locus 5004 :

2 alleles

98.8% missing data

1	(0.580) 0.580 0.573

4	(0.420) 0.420 0.427

Locus 5005 :

2 alleles

97.3% missing data

4	(0.451) 0.470 0.438

1	(0.549) 0.530 0.562

Locus 5006 :

3 alleles

93.4% missing data

3	(0.782) 0.773 0.800

4	(0.071) 0.072 0.072

1	(0.147) 0.155 0.127

Locus 5007 :

2 alleles

98.0% missing data

4	(0.735) 0.731 0.741

3	(0.265) 0.269 0.259

Locus 5008 :

2 alleles

99.2% missing data

4	(0.643) 0.639 0.645

1	(0.357) 0.361 0.355

Locus 5009 :

2 alleles

99.2% missing data

2	(0.192) 0.190 0.194

1	(0.808) 0.810 0.806

Locus 5010 :

2 alleles

99.2% missing data

1	(0.753) 0.761 0.749

2	(0.247) 0.239 0.251

Locus 5011 :

2 alleles

94.5% missing data

1	(0.117) 0.123 0.105

3	(0.883) 0.877 0.895

Locus 5012 :

2 alleles

99.2% missing data

1	(0.364) 0.363 0.367

4	(0.636) 0.637 0.633

Locus 5013 :

2 alleles

99.2% missing data

1	(0.575) 0.580 0.569

2	(0.425) 0.420 0.431

Locus 5014 :

2 alleles

99.2% missing data

2	(0.693) 0.689 0.705

1	(0.307) 0.311 0.295

Locus 5015 :

2 alleles

98.8% missing data

1	(0.440) 0.440 0.449

4	(0.560) 0.560 0.551

Locus 5016 :

2 alleles

99.2% missing data

1	(0.362) 0.358 0.367

3	(0.638) 0.642 0.633

Locus 5017 :

2 alleles

98.8% missing data

2	(0.696) 0.698 0.691

3	(0.304) 0.302 0.309

Locus 5018 :

2 alleles

7.8% missing data

1	(0.982) 0.979 0.994

3	(0.018) 0.021 0.006

Locus 5019 :

2 alleles

99.2% missing data

3	(0.427) 0.424 0.438

4	(0.573) 0.576 0.562

Locus 5020 :

2 alleles

99.2% missing data

3	(0.685) 0.691 0.679

1	(0.315) 0.309 0.321

Locus 5021 :

2 alleles

99.2% missing data

1	(0.673) 0.660 0.680

2	(0.327) 0.340 0.320

Locus 5022 :

2 alleles

99.2% missing data

4	(0.737) 0.730 0.737

3	(0.263) 0.270 0.263

Locus 5023 :

2 alleles

98.8% missing data

4	(0.412) 0.417 0.415

2	(0.588) 0.583 0.585

Locus 5024 :

2 alleles

98.4% missing data

3	(0.429) 0.427 0.425

1	(0.571) 0.573 0.575

Locus 5025 :

2 alleles

99.2% missing data

2	(0.497) 0.496 0.495

1	(0.503) 0.504 0.505

Locus 5026 :

2 alleles

98.8% missing data

2	(0.416) 0.411 0.425

1	(0.584) 0.589 0.575

Locus 5027 :

2 alleles

99.2% missing data

4	(0.320) 0.327 0.316

1	(0.680) 0.673 0.684

Locus 5028 :

3 alleles

96.1% missing data

4	(0.180) 0.178 0.183

3	(0.181) 0.186 0.172

1	(0.639) 0.636 0.645

Locus 5029 :

3 alleles

98.0% missing data

4	(0.152) 0.158 0.144

2	(0.683) 0.674 0.687

3	(0.165) 0.168 0.168

Locus 5030 :

2 alleles

96.5% missing data

3	(0.587) 0.566 0.614

4	(0.413) 0.434 0.386

Locus 5031 :

2 alleles

96.1% missing data

2	(0.871) 0.869 0.873

3	(0.129) 0.131 0.127

Locus 5032 :

2 alleles

96.9% missing data

1	(0.883) 0.874 0.893

3	(0.117) 0.126 0.107

Locus 5033 :

2 alleles

98.8% missing data

4	(0.566) 0.571 0.566

1	(0.434) 0.429 0.434

Locus 5034 :

2 alleles

97.3% missing data

3	(0.200) 0.202 0.198

4	(0.800) 0.798 0.802

Locus 5035 :

2 alleles

98.8% missing data

3	(0.450) 0.447 0.456

1	(0.550) 0.553 0.544

Locus 5036 :

2 alleles

98.8% missing data

3	(0.825) 0.820 0.830

4	(0.175) 0.180 0.170

Locus 5037 :

2 alleles

98.0% missing data

2	(0.777) 0.782 0.783

3	(0.223) 0.218 0.217

Locus 5038 :

2 alleles

99.2% missing data

1	(0.496) 0.492 0.500

3	(0.504) 0.508 0.500

Locus 5039 :

2 alleles

99.2% missing data

3	(0.551) 0.552 0.543

1	(0.449) 0.448 0.457

Locus 5040 :

2 alleles

99.2% missing data

2	(0.623) 0.620 0.623

4	(0.377) 0.380 0.377

Locus 5041 :

2 alleles

99.2% missing data

4	(0.361) 0.356 0.368

3	(0.639) 0.644 0.632

Locus 5042 :

2 alleles

99.2% missing data

2	(0.677) 0.674 0.679

3	(0.323) 0.326 0.321

Locus 5043 :

2 alleles

99.2% missing data

4	(0.485) 0.476 0.490

1	(0.515) 0.524 0.510

Locus 5044 :

2 alleles

99.2% missing data

4	(0.292) 0.287 0.295

3	(0.708) 0.713 0.705

Locus 5045 :

2 alleles

99.2% missing data

1	(0.668) 0.663 0.664

2	(0.332) 0.337 0.336

Locus 5046 :

2 alleles

99.2% missing data

3	(0.426) 0.420 0.429

4	(0.574) 0.580 0.571

Locus 5047 :

2 alleles

97.3% missing data

4	(0.826) 0.837 0.819

1	(0.174) 0.163 0.181

Locus 5048 :

2 alleles

99.2% missing data

3	(0.689) 0.696 0.686

4	(0.311) 0.304 0.314

Locus 5049 :

2 alleles

98.8% missing data

4	(0.382) 0.372 0.390

3	(0.618) 0.628 0.610

Locus 5050 :

2 alleles

90.6% missing data

4	(0.876) 0.889 0.866

2	(0.124) 0.111 0.134

Locus 5051 :

2 alleles

99.2% missing data

2	(0.303) 0.297 0.306

3	(0.697) 0.703 0.694

Locus 5052 :

2 alleles

85.2% missing data

3	(0.928) 0.926 0.939

1	(0.072) 0.074 0.061

Locus 5053 :

2 alleles

99.2% missing data

4	(0.606) 0.608 0.610

3	(0.394) 0.392 0.390

Locus 5054 :

2 alleles

99.2% missing data

3	(0.733) 0.736 0.733

1	(0.267) 0.264 0.267

Locus 5055 :

2 alleles

98.8% missing data

3	(0.636) 0.630 0.641

4	(0.364) 0.370 0.359

Locus 5056 :

2 alleles

98.0% missing data

4	(0.401) 0.401 0.408

1	(0.599) 0.599 0.592

Locus 5057 :

2 alleles

99.2% missing data

4	(0.721) 0.712 0.726

1	(0.279) 0.288 0.274

Locus 5058 :

2 alleles

99.2% missing data

1	(0.622) 0.617 0.633

3	(0.378) 0.383 0.367

Locus 5059 :

2 alleles

95.3% missing data

3	(0.895) 0.901 0.894

1	(0.105) 0.099 0.106

Locus 5060 :

2 alleles

96.5% missing data

4	(0.206) 0.193 0.208

3	(0.794) 0.807 0.792

Locus 5061 :

2 alleles

98.4% missing data

3	(0.630) 0.619 0.644

1	(0.370) 0.381 0.356

Locus 5062 :

2 alleles

91.0% missing data

1	(0.935) 0.949 0.923

2	(0.065) 0.051 0.077

Locus 5063 :

2 alleles

93.0% missing data

2	(0.902) 0.901 0.909

1	(0.098) 0.099 0.091

Locus 5064 :

2 alleles

98.8% missing data

1	(0.555) 0.547 0.561

3	(0.445) 0.453 0.439

Locus 5065 :

2 alleles

91.0% missing data

1	(0.933) 0.947 0.927

2	(0.067) 0.053 0.073

Locus 5066 :

2 alleles

99.2% missing data

1	(0.497) 0.495 0.498

3	(0.503) 0.505 0.502

Locus 5067 :

2 alleles

98.8% missing data

3	(0.764) 0.760 0.773

1	(0.236) 0.240 0.227

Locus 5068 :

2 alleles

99.2% missing data

1	(0.598) 0.599 0.599

4	(0.402) 0.401 0.401

Locus 5069 :

2 alleles

99.2% missing data

1	(0.355) 0.360 0.351

3	(0.645) 0.640 0.649

Locus 5070 :

2 alleles

99.2% missing data

2	(0.449) 0.434 0.455

1	(0.551) 0.566 0.545

Locus 5071 :

2 alleles

99.2% missing data

1	(0.669) 0.657 0.666

3	(0.331) 0.343 0.334

Locus 5072 :

2 alleles

99.2% missing data

2	(0.369) 0.363 0.377

3	(0.631) 0.637 0.623

Locus 5073 :

2 alleles

92.6% missing data

3	(0.861) 0.861 0.875

4	(0.139) 0.139 0.125

Locus 5074 :

2 alleles

99.2% missing data

4	(0.469) 0.464 0.478

3	(0.531) 0.536 0.522

Locus 5075 :

2 alleles

96.9% missing data

2	(0.852) 0.850 0.854

4	(0.148) 0.150 0.146

Locus 5076 :

2 alleles

99.2% missing data

4	(0.545) 0.547 0.541

1	(0.455) 0.453 0.459

Locus 5077 :

2 alleles

98.8% missing data

1	(0.666) 0.670 0.660

2	(0.334) 0.330 0.340

Locus 5078 :

2 alleles

99.2% missing data

2	(0.529) 0.527 0.520

3	(0.471) 0.473 0.480

Locus 5079 :

2 alleles

99.2% missing data

1	(0.273) 0.282 0.270

4	(0.727) 0.718 0.730

Locus 5080 :

2 alleles

98.0% missing data

3	(0.265) 0.256 0.271

1	(0.735) 0.744 0.729

Locus 5081 :

2 alleles

99.2% missing data

1	(0.109) 0.113 0.112

4	(0.891) 0.887 0.888

Locus 5082 :

2 alleles

98.8% missing data

1	(0.719) 0.718 0.723

4	(0.281) 0.282 0.277

Locus 5083 :

2 alleles

96.9% missing data

3	(0.842) 0.837 0.852

1	(0.158) 0.163 0.148

Locus 5084 :

2 alleles

99.2% missing data

4	(0.501) 0.494 0.507

3	(0.499) 0.506 0.493

Locus 5085 :

2 alleles

99.2% missing data

3	(0.300) 0.303 0.308

1	(0.700) 0.697 0.692

Locus 5086 :

2 alleles

8.2% missing data

3	(0.983) 0.976 0.995

1	(0.017) 0.024 0.005

Locus 5087 :

2 alleles

98.0% missing data

3	(0.680) 0.670 0.694

4	(0.320) 0.330 0.306

Locus 5088 :

2 alleles

99.2% missing data

2	(0.359) 0.362 0.351

4	(0.641) 0.638 0.649

Locus 5089 :

3 alleles

98.8% missing data

4	(0.378) 0.378 0.379

3	(0.239) 0.241 0.239

2	(0.382) 0.381 0.382

Locus 5090 :

3 alleles

98.8% missing data

4	(0.244) 0.242 0.251

2	(0.437) 0.437 0.436

3	(0.319) 0.321 0.312

Locus 5091 :

2 alleles

99.2% missing data

1	(0.225) 0.222 0.227

3	(0.775) 0.778 0.773

Locus 5092 :

2 alleles

99.2% missing data

1	(0.702) 0.700 0.694

4	(0.298) 0.300 0.306

Locus 5093 :

2 alleles

97.7% missing data

1	(0.524) 0.511 0.522

2	(0.476) 0.489 0.478

Locus 5094 :

2 alleles

99.2% missing data

2	(0.644) 0.645 0.637

4	(0.356) 0.355 0.363

Locus 5095 :

2 alleles

99.2% missing data

1	(0.500) 0.498 0.509

2	(0.500) 0.502 0.491

Locus 5096 :

2 alleles

99.2% missing data

2	(0.769) 0.763 0.767

4	(0.231) 0.237 0.233

Locus 5097 :

2 alleles

96.9% missing data

2	(0.289) 0.277 0.288

3	(0.711) 0.723 0.712

Locus 5098 :

2 alleles

97.3% missing data

4	(0.701) 0.702 0.712

2	(0.299) 0.298 0.288

Locus 5099 :

2 alleles

97.3% missing data

4	(0.772) 0.772 0.776

1	(0.228) 0.228 0.224

Locus 5100 :

3 alleles

98.0% missing data

4	(0.464) 0.473 0.457

1	(0.137) 0.131 0.148

3	(0.399) 0.396 0.395

Locus 5101 :

2 alleles

99.2% missing data

1	(0.239) 0.236 0.239

2	(0.761) 0.764 0.761

Locus 5102 :

3 alleles

98.8% missing data

1	(0.147) 0.144 0.149

4	(0.342) 0.342 0.348

3	(0.511) 0.514 0.504

Locus 5103 :

2 alleles

99.2% missing data

2	(0.531) 0.535 0.525

4	(0.469) 0.465 0.475

Locus 5104 :

2 alleles

98.8% missing data

4	(0.592) 0.598 0.589

1	(0.408) 0.402 0.411

Locus 5105 :

3 alleles

98.8% missing data

4	(0.220) 0.226 0.215

1	(0.409) 0.403 0.406

2	(0.371) 0.371 0.379

Locus 5106 :

2 alleles

98.8% missing data

3	(0.270) 0.264 0.270

2	(0.730) 0.736 0.730

Locus 5107 :

2 alleles

99.2% missing data

4	(0.191) 0.185 0.198

3	(0.809) 0.815 0.802

Locus 5108 :

2 alleles

98.8% missing data

2	(0.588) 0.581 0.595

3	(0.412) 0.419 0.405

Locus 5109 :

2 alleles

93.0% missing data

1	(0.932) 0.935 0.934

4	(0.068) 0.065 0.066

Locus 5110 :

2 alleles

98.8% missing data

4	(0.549) 0.545 0.548

1	(0.451) 0.455 0.452

Locus 5111 :

2 alleles

98.8% missing data

4	(0.479) 0.476 0.481

1	(0.521) 0.524 0.519

Locus 5112 :

2 alleles

98.8% missing data

1	(0.656) 0.662 0.649

3	(0.344) 0.338 0.351

Locus 5113 :

2 alleles

99.2% missing data

1	(0.440) 0.438 0.439

2	(0.560) 0.562 0.561

Locus 5114 :

3 alleles

98.0% missing data

2	(0.485) 0.488 0.488

3	(0.256) 0.253 0.257

1	(0.259) 0.259 0.256

Locus 5115 :

2 alleles

98.8% missing data

4	(0.413) 0.401 0.419

1	(0.587) 0.599 0.581

Locus 5116 :

2 alleles

99.2% missing data

1	(0.797) 0.802 0.793

3	(0.203) 0.198 0.207

Locus 5117 :

2 alleles

99.2% missing data

1	(0.646) 0.643 0.649

2	(0.354) 0.357 0.351

Locus 5118 :

2 alleles

99.2% missing data

1	(0.390) 0.400 0.386

4	(0.610) 0.600 0.614

Locus 5119 :

2 alleles

99.2% missing data

4	(0.510) 0.510 0.510

2	(0.490) 0.490 0.490

Locus 5120 :

2 alleles

99.2% missing data

4	(0.811) 0.815 0.809

2	(0.189) 0.185 0.191

Locus 5121 :

2 alleles

99.2% missing data

3	(0.598) 0.598 0.596

2	(0.402) 0.402 0.404

Locus 5122 :

3 alleles

98.8% missing data

4	(0.567) 0.575 0.557

2	(0.173) 0.166 0.183

1	(0.260) 0.259 0.261

Locus 5123 :

2 alleles

98.0% missing data

4	(0.859) 0.854 0.865

2	(0.141) 0.146 0.135

Locus 5124 :

2 alleles

99.2% missing data

4	(0.429) 0.427 0.432

2	(0.571) 0.573 0.568

Locus 5125 :

2 alleles

93.0% missing data

2	(0.857) 0.846 0.881

3	(0.143) 0.154 0.119

Locus 5126 :

2 alleles

96.1% missing data

4	(0.827) 0.836 0.821

2	(0.173) 0.164 0.179

Locus 5127 :

2 alleles

99.2% missing data

1	(0.615) 0.607 0.610

3	(0.385) 0.393 0.390

Locus 5128 :

2 alleles

99.2% missing data

1	(0.512) 0.509 0.516

3	(0.488) 0.491 0.484

Locus 5129 :

2 alleles

99.2% missing data

4	(0.670) 0.671 0.669

3	(0.330) 0.329 0.331

Locus 5130 :

2 alleles

99.2% missing data

1	(0.559) 0.555 0.558

3	(0.441) 0.445 0.442

Locus 5131 :

2 alleles

89.8% missing data

1	(0.740) 0.774 0.707

2	(0.260) 0.226 0.293

Locus 5132 :

2 alleles

99.2% missing data

4	(0.740) 0.745 0.734

2	(0.260) 0.255 0.266

Locus 5133 :

2 alleles

99.2% missing data

2	(0.641) 0.643 0.635

1	(0.359) 0.357 0.365

Locus 5134 :

2 alleles

99.2% missing data

4	(0.630) 0.630 0.628

2	(0.370) 0.370 0.372

Locus 5135 :

2 alleles

0.0% missing data

4	(0.988) 0.990 0.994

3	(0.012) 0.010 0.006

Locus 5136 :

2 alleles

99.2% missing data

4	(0.635) 0.623 0.641

1	(0.365) 0.377 0.359

Locus 5137 :

2 alleles

99.2% missing data

2	(0.389) 0.384 0.399

4	(0.611) 0.616 0.601

Locus 5138 :

2 alleles

99.2% missing data

4	(0.423) 0.418 0.428

3	(0.577) 0.582 0.572

Locus 5139 :

2 alleles

99.2% missing data

4	(0.659) 0.660 0.660

2	(0.341) 0.340 0.340

Locus 5140 :

2 alleles

99.2% missing data

1	(0.558) 0.556 0.555

2	(0.442) 0.444 0.445

Locus 5141 :

2 alleles

96.5% missing data

4	(0.845) 0.850 0.848

2	(0.155) 0.150 0.152

Locus 5142 :

2 alleles

99.2% missing data

1	(0.640) 0.635 0.640

4	(0.360) 0.365 0.360

Locus 5143 :

2 alleles

8.2% missing data

2	(0.985) 0.980 0.995

3	(0.015) 0.020 0.005

Locus 5144 :

2 alleles

98.8% missing data

1	(0.458) 0.453 0.464

3	(0.542) 0.547 0.536

Locus 5145 :

2 alleles

0.0% missing data

1	(0.988) 0.991 0.993

4	(0.012) 0.009 0.007

Locus 5146 :

2 alleles

96.9% missing data

1	(0.843) 0.846 0.845

4	(0.157) 0.154 0.155

Locus 5147 :

2 alleles

98.8% missing data

1	(0.420) 0.414 0.414

2	(0.580) 0.586 0.586

Locus 5148 :

2 alleles

99.2% missing data

4	(0.413) 0.414 0.411

3	(0.587) 0.586 0.589

Locus 5149 :

2 alleles

99.2% missing data

1	(0.417) 0.415 0.412

3	(0.583) 0.585 0.588

Locus 5150 :

2 alleles

99.2% missing data

2	(0.496) 0.488 0.500

1	(0.504) 0.512 0.500

Locus 5151 :

2 alleles

99.2% missing data

4	(0.418) 0.421 0.419

1	(0.582) 0.579 0.581

Locus 5152 :

2 alleles

98.0% missing data

4	(0.779) 0.773 0.781

2	(0.221) 0.227 0.219

Locus 5153 :

2 alleles

99.2% missing data

2	(0.304) 0.305 0.306

3	(0.696) 0.695 0.694

Locus 5154 :

2 alleles

99.2% missing data

3	(0.560) 0.564 0.557

4	(0.440) 0.436 0.443

Locus 5155 :

2 alleles

99.2% missing data

2	(0.466) 0.468 0.460

3	(0.534) 0.532 0.540

Locus 5156 :

2 alleles

99.2% missing data

3	(0.577) 0.572 0.578

2	(0.423) 0.428 0.422

Locus 5157 :

2 alleles

99.2% missing data

3	(0.541) 0.537 0.545

4	(0.459) 0.463 0.455

Locus 5158 :

2 alleles

99.2% missing data

4	(0.295) 0.292 0.305

3	(0.705) 0.708 0.695

Locus 5159 :

2 alleles

99.2% missing data

3	(0.466) 0.464 0.471

1	(0.534) 0.536 0.529

Locus 5160 :

2 alleles

98.4% missing data

4	(0.559) 0.566 0.558

1	(0.441) 0.434 0.442

Locus 5161 :

2 alleles

98.8% missing data

2	(0.583) 0.582 0.586

3	(0.417) 0.418 0.414

Locus 5162 :

2 alleles

99.2% missing data

2	(0.772) 0.774 0.772

3	(0.228) 0.226 0.228

Locus 5163 :

3 alleles

8.2% missing data

3	(0.961) 0.937 0.988

4	(0.018) 0.027 0.006

1	(0.020) 0.036 0.007

Locus 5164 :

2 alleles

99.2% missing data

3	(0.637) 0.634 0.636

1	(0.363) 0.366 0.364

Locus 5165 :

2 alleles

99.2% missing data

4	(0.412) 0.409 0.413

1	(0.588) 0.591 0.587

Locus 5166 :

2 alleles

99.2% missing data

2	(0.363) 0.357 0.367

1	(0.637) 0.643 0.633

Locus 5167 :

2 alleles

99.2% missing data

2	(0.360) 0.357 0.360

3	(0.640) 0.643 0.640

Locus 5168 :

2 alleles

98.4% missing data

1	(0.857) 0.863 0.853

3	(0.143) 0.137 0.147

Locus 5169 :

2 alleles

99.2% missing data

4	(0.333) 0.329 0.338

1	(0.667) 0.671 0.662

Locus 5170 :

2 alleles

98.8% missing data

3	(0.408) 0.413 0.413

1	(0.592) 0.587 0.587

Locus 5171 :

3 alleles

98.8% missing data

3	(0.340) 0.330 0.345

2	(0.386) 0.397 0.381

4	(0.274) 0.273 0.274

Locus 5172 :

2 alleles

96.9% missing data

3	(0.683) 0.679 0.688

1	(0.317) 0.321 0.312

Locus 5173 :

2 alleles

99.2% missing data

1	(0.659) 0.657 0.657

3	(0.341) 0.343 0.343

Locus 5174 :

2 alleles

8.2% missing data

4	(0.984) 0.976 0.995

2	(0.016) 0.024 0.005

Locus 5175 :

2 alleles

99.2% missing data

4	(0.371) 0.362 0.373

1	(0.629) 0.638 0.627

Locus 5176 :

2 alleles

98.4% missing data

2	(0.757) 0.763 0.756

3	(0.243) 0.237 0.244

Locus 5177 :

2 alleles

99.2% missing data

3	(0.686) 0.686 0.684

2	(0.314) 0.314 0.316

Locus 5178 :

2 alleles

98.8% missing data

3	(0.399) 0.401 0.401

1	(0.601) 0.599 0.599

Locus 5179 :

2 alleles

98.8% missing data

2	(0.420) 0.426 0.420

1	(0.580) 0.574 0.580

Locus 5180 :

2 alleles

99.2% missing data

3	(0.607) 0.604 0.609

1	(0.393) 0.396 0.391

Locus 5181 :

2 alleles

99.2% missing data

4	(0.621) 0.621 0.617

3	(0.379) 0.379 0.383

Locus 5182 :

2 alleles

99.2% missing data

4	(0.163) 0.162 0.167

1	(0.837) 0.838 0.833

Locus 5183 :

2 alleles

98.8% missing data

4	(0.802) 0.801 0.800

1	(0.198) 0.199 0.200

Locus 5184 :

2 alleles

97.7% missing data

1	(0.231) 0.230 0.227

3	(0.769) 0.770 0.773

Locus 5185 :

2 alleles

99.2% missing data

3	(0.625) 0.627 0.625

4	(0.375) 0.373 0.375

Locus 5186 :

2 alleles

99.2% missing data

1	(0.519) 0.514 0.522

2	(0.481) 0.486 0.478

Locus 5187 :

2 alleles

98.8% missing data

1	(0.723) 0.723 0.722

4	(0.277) 0.277 0.278

Locus 5188 :

3 alleles

98.8% missing data

1	(0.420) 0.421 0.418

4	(0.274) 0.277 0.269

3	(0.306) 0.302 0.313

Locus 5189 :

2 alleles

99.2% missing data

4	(0.370) 0.365 0.370

3	(0.630) 0.635 0.630

Locus 5190 :

2 alleles

99.2% missing data

1	(0.281) 0.284 0.287

4	(0.719) 0.716 0.713

Locus 5191 :

2 alleles

99.2% missing data

2	(0.321) 0.321 0.324

4	(0.679) 0.679 0.676

Locus 5192 :

2 alleles

99.2% missing data

1	(0.434) 0.430 0.435

2	(0.566) 0.570 0.565

Locus 5193 :

2 alleles

90.6% missing data

4	(0.896) 0.914 0.885

3	(0.104) 0.086 0.115

Locus 5194 :

2 alleles

99.2% missing data

4	(0.834) 0.839 0.830

1	(0.166) 0.161 0.170

Locus 5195 :

2 alleles

99.2% missing data

3	(0.279) 0.285 0.280

2	(0.721) 0.715 0.720

Locus 5196 :

2 alleles

99.2% missing data

3	(0.534) 0.537 0.535

4	(0.466) 0.463 0.465

Locus 5197 :

3 alleles

96.1% missing data

3	(0.670) 0.662 0.686

2	(0.212) 0.208 0.206

4	(0.118) 0.130 0.108

Locus 5198 :

2 alleles

91.0% missing data

4	(0.904) 0.919 0.897

1	(0.096) 0.081 0.103

Locus 5199 :

3 alleles

98.8% missing data

1	(0.486) 0.482 0.488

2	(0.264) 0.259 0.270

3	(0.250) 0.259 0.242

Locus 5200 :

2 alleles

98.8% missing data

4	(0.706) 0.704 0.705

1	(0.294) 0.296 0.295

Locus 5201 :

2 alleles

97.7% missing data

1	(0.749) 0.744 0.753

2	(0.251) 0.256 0.247

Locus 5202 :

2 alleles

99.2% missing data

3	(0.454) 0.453 0.452

1	(0.546) 0.547 0.548

Locus 5203 :

2 alleles

91.4% missing data

1	(0.871) 0.891 0.860

4	(0.129) 0.109 0.140

Locus 5204 :

2 alleles

98.8% missing data

1	(0.547) 0.547 0.548

2	(0.453) 0.453 0.452

Locus 5205 :

2 alleles

98.8% missing data

3	(0.583) 0.575 0.588

1	(0.417) 0.425 0.412

Locus 5206 :

2 alleles

98.8% missing data

4	(0.520) 0.519 0.528

3	(0.480) 0.481 0.472

Locus 5207 :

2 alleles

99.2% missing data

3	(0.456) 0.451 0.462

4	(0.544) 0.549 0.538

Locus 5208 :

2 alleles

98.0% missing data

2	(0.118) 0.113 0.127

1	(0.882) 0.887 0.873

Locus 5209 :

2 alleles

99.2% missing data

4	(0.429) 0.429 0.433

2	(0.571) 0.571 0.567

Locus 5210 :

2 alleles

98.8% missing data

4	(0.605) 0.599 0.605

2	(0.395) 0.401 0.395

Locus 5211 :

2 alleles

98.8% missing data

2	(0.540) 0.538 0.547

1	(0.460) 0.462 0.453

Locus 5212 :

2 alleles

99.2% missing data

1	(0.251) 0.247 0.258

3	(0.749) 0.753 0.742

Locus 5213 :

2 alleles

99.2% missing data

4	(0.818) 0.818 0.822

1	(0.182) 0.182 0.178

Locus 5214 :

2 alleles

96.9% missing data

1	(0.736) 0.728 0.753

4	(0.264) 0.272 0.247

Locus 5215 :

2 alleles

99.2% missing data

2	(0.615) 0.611 0.620

4	(0.385) 0.389 0.380

Locus 5216 :

2 alleles

97.7% missing data

2	(0.608) 0.611 0.616

1	(0.392) 0.389 0.384

Locus 5217 :

2 alleles

99.2% missing data

1	(0.795) 0.794 0.793

4	(0.205) 0.206 0.207

Locus 5218 :

3 alleles

98.4% missing data

1	(0.604) 0.597 0.603

3	(0.228) 0.235 0.223

4	(0.168) 0.168 0.174

Locus 5219 :

2 alleles

3.5% missing data

1	(0.984) 0.979 0.996

2	(0.016) 0.021 0.004

Locus 5220 :

2 alleles

99.2% missing data

1	(0.657) 0.661 0.650

4	(0.343) 0.339 0.350

Locus 5221 :

2 alleles

99.2% missing data

2	(0.569) 0.568 0.573

3	(0.431) 0.432 0.427

Locus 5222 :

2 alleles

97.7% missing data

3	(0.557) 0.561 0.569

1	(0.443) 0.439 0.431

Locus 5223 :

2 alleles

99.2% missing data

3	(0.334) 0.337 0.335

2	(0.666) 0.663 0.665

Locus 5224 :

2 alleles

97.3% missing data

1	(0.600) 0.595 0.620

4	(0.400) 0.405 0.380

Locus 5225 :

2 alleles

99.2% missing data

2	(0.519) 0.518 0.530

4	(0.481) 0.482 0.470

Locus 5226 :

2 alleles

0.4% missing data

1	(0.988) 0.986 0.995

4	(0.012) 0.014 0.005

Locus 5227 :

2 alleles

99.2% missing data

4	(0.590) 0.590 0.591

1	(0.410) 0.410 0.409

Locus 5228 :

2 alleles

0.4% missing data

1	(0.969) 0.971 0.979

4	(0.031) 0.029 0.021

Locus 5229 :

2 alleles

99.2% missing data

1	(0.633) 0.626 0.636

2	(0.367) 0.374 0.364

Locus 5230 :

2 alleles

99.2% missing data

2	(0.274) 0.273 0.276

3	(0.726) 0.727 0.724

Locus 5231 :

3 alleles

96.9% missing data

1	(0.807) 0.808 0.812

3	(0.073) 0.076 0.070

4	(0.119) 0.116 0.118

Locus 5232 :

2 alleles

99.2% missing data

4	(0.315) 0.328 0.309

2	(0.685) 0.672 0.691

Locus 5233 :

2 alleles

99.2% missing data

4	(0.517) 0.522 0.514

2	(0.483) 0.478 0.486

Locus 5234 :

2 alleles

99.2% missing data

2	(0.522) 0.524 0.522

4	(0.478) 0.476 0.478

Locus 5235 :

3 alleles

98.8% missing data

4	(0.247) 0.249 0.242

3	(0.417) 0.422 0.414

2	(0.337) 0.329 0.345

Locus 5236 :

2 alleles

99.2% missing data

1	(0.661) 0.657 0.669

4	(0.339) 0.343 0.331

Locus 5237 :

2 alleles

98.8% missing data

4	(0.816) 0.815 0.816

3	(0.184) 0.185 0.184

Locus 5238 :

2 alleles

0.4% missing data

3	(0.965) 0.960 0.980

4	(0.035) 0.040 0.020

Locus 5239 :

2 alleles

99.2% missing data

3	(0.308) 0.299 0.313

4	(0.692) 0.701 0.687

Locus 5240 :

2 alleles

99.2% missing data

2	(0.327) 0.326 0.322

4	(0.673) 0.674 0.678

Locus 5241 :

2 alleles

98.8% missing data

4	(0.427) 0.436 0.427

3	(0.573) 0.564 0.573

Locus 5242 :

2 alleles

99.2% missing data

4	(0.291) 0.292 0.297

3	(0.709) 0.708 0.703

Locus 5243 :

2 alleles

97.3% missing data

3	(0.775) 0.773 0.782

4	(0.225) 0.227 0.218

Locus 5244 :

2 alleles

99.2% missing data

2	(0.449) 0.445 0.451

1	(0.551) 0.555 0.549

Locus 5245 :

2 alleles

99.2% missing data

3	(0.456) 0.449 0.463

1	(0.544) 0.551 0.537

Locus 5246 :

3 alleles

98.8% missing data

3	(0.298) 0.299 0.302

4	(0.147) 0.146 0.148

1	(0.554) 0.556 0.550

Locus 5247 :

2 alleles

8.2% missing data

3	(0.985) 0.978 0.995

2	(0.015) 0.022 0.005

Locus 5248 :

2 alleles

98.0% missing data

4	(0.623) 0.624 0.623

3	(0.377) 0.376 0.377

Locus 5249 :

2 alleles

98.4% missing data

4	(0.627) 0.623 0.624

3	(0.373) 0.377 0.376

Locus 5250 :

2 alleles

91.4% missing data

3	(0.914) 0.910 0.925

4	(0.086) 0.090 0.075

Locus 5251 :

2 alleles

0.0% missing data

4	(0.985) 0.982 0.996

1	(0.015) 0.018 0.004

Locus 5252 :

2 alleles

96.9% missing data

4	(0.196) 0.187 0.201

1	(0.804) 0.813 0.799

Locus 5253 :

2 alleles

95.3% missing data

4	(0.901) 0.902 0.900

2	(0.099) 0.098 0.100

Locus 5254 :

2 alleles

99.2% missing data

3	(0.293) 0.289 0.294

4	(0.707) 0.711 0.706

Locus 5255 :

2 alleles

94.9% missing data

3	(0.917) 0.928 0.914

2	(0.083) 0.072 0.086

Locus 5256 :

2 alleles

99.2% missing data

4	(0.469) 0.473 0.468

2	(0.531) 0.527 0.532

Locus 5257 :

2 alleles

99.2% missing data

4	(0.685) 0.676 0.688

1	(0.315) 0.324 0.312

Locus 5258 :

2 alleles

98.8% missing data

3	(0.883) 0.880 0.887

4	(0.117) 0.120 0.113

Locus 5259 :

2 alleles

99.2% missing data

1	(0.236) 0.239 0.238

3	(0.764) 0.761 0.762

Locus 5260 :

2 alleles

99.2% missing data

3	(0.594) 0.592 0.591

4	(0.406) 0.408 0.409

Locus 5261 :

2 alleles

92.6% missing data

3	(0.921) 0.910 0.934

1	(0.079) 0.090 0.066

Locus 5262 :

2 alleles

85.2% missing data

4	(0.937) 0.934 0.942

2	(0.063) 0.066 0.058

Locus 5263 :

3 alleles

98.0% missing data

4	(0.334) 0.343 0.337

3	(0.436) 0.428 0.438

1	(0.230) 0.229 0.225

Locus 5264 :

2 alleles

99.2% missing data

4	(0.846) 0.848 0.843

2	(0.154) 0.152 0.157

Locus 5265 :

2 alleles

99.2% missing data

4	(0.525) 0.524 0.522

2	(0.475) 0.476 0.478

Locus 5266 :

2 alleles

99.2% missing data

4	(0.467) 0.465 0.468

3	(0.533) 0.535 0.532

Locus 5267 :

2 alleles

93.0% missing data

4	(0.867) 0.865 0.880

1	(0.133) 0.135 0.120

Locus 5268 :

2 alleles

98.8% missing data

1	(0.482) 0.471 0.485

3	(0.518) 0.529 0.515

Locus 5269 :

2 alleles

98.4% missing data

3	(0.672) 0.668 0.678

2	(0.328) 0.332 0.322

Locus 5270 :

2 alleles

99.2% missing data

4	(0.530) 0.528 0.533

3	(0.470) 0.472 0.467

Locus 5271 :

2 alleles

99.2% missing data

1	(0.437) 0.436 0.441

2	(0.563) 0.564 0.559

Locus 5272 :

3 alleles

98.8% missing data

1	(0.269) 0.264 0.272

2	(0.382) 0.387 0.380

3	(0.349) 0.349 0.347

Locus 5273 :

2 alleles

99.2% missing data

4	(0.626) 0.634 0.613

1	(0.374) 0.366 0.387

Locus 5274 :

2 alleles

99.2% missing data

4	(0.406) 0.399 0.418

2	(0.594) 0.601 0.582

Locus 5275 :

2 alleles

99.2% missing data

1	(0.602) 0.597 0.605

4	(0.398) 0.403 0.395

Locus 5276 :

2 alleles

99.2% missing data

2	(0.712) 0.710 0.714

3	(0.288) 0.290 0.286

Locus 5277 :

2 alleles

98.4% missing data

2	(0.666) 0.664 0.666

1	(0.334) 0.336 0.334

Locus 5278 :

2 alleles

99.2% missing data

3	(0.362) 0.361 0.364

1	(0.638) 0.639 0.636

Locus 5279 :

2 alleles

98.8% missing data

1	(0.831) 0.826 0.838

4	(0.169) 0.174 0.162

Locus 5280 :

3 alleles

94.1% missing data

4	(0.808) 0.796 0.831

1	(0.071) 0.077 0.062

3	(0.121) 0.127 0.106

Locus 5281 :

2 alleles

99.2% missing data

3	(0.707) 0.711 0.707

2	(0.293) 0.289 0.293

Locus 5282 :

2 alleles

99.2% missing data

4	(0.670) 0.664 0.672

3	(0.330) 0.336 0.328

Locus 5283 :

2 alleles

99.2% missing data

2	(0.462) 0.455 0.468

3	(0.538) 0.545 0.532

Locus 5284 :

2 alleles

96.5% missing data

1	(0.796) 0.803 0.798

4	(0.204) 0.197 0.202

Locus 5285 :

2 alleles

99.2% missing data

4	(0.332) 0.333 0.336

2	(0.668) 0.667 0.664

Locus 5286 :

3 alleles

98.4% missing data

1	(0.230) 0.226 0.233

3	(0.349) 0.342 0.355

2	(0.420) 0.432 0.412

Locus 5287 :

2 alleles

99.2% missing data

4	(0.272) 0.268 0.273

3	(0.728) 0.732 0.727

Locus 5288 :

2 alleles

98.4% missing data

4	(0.577) 0.583 0.578

3	(0.423) 0.417 0.422

Locus 5289 :

2 alleles

97.3% missing data

2	(0.401) 0.409 0.381

4	(0.599) 0.591 0.619

Locus 5290 :

2 alleles

99.2% missing data

4	(0.591) 0.589 0.596

3	(0.409) 0.411 0.404

Locus 5291 :

2 alleles

8.2% missing data

4	(0.983) 0.977 0.995

1	(0.017) 0.023 0.005

Locus 5292 :

2 alleles

7.8% missing data

4	(0.982) 0.968 0.995

1	(0.018) 0.032 0.005

Locus 5293 :

2 alleles

95.3% missing data

3	(0.901) 0.900 0.903

1	(0.099) 0.100 0.097

Locus 5294 :

3 alleles

98.4% missing data

1	(0.467) 0.473 0.476

3	(0.375) 0.367 0.370

2	(0.158) 0.161 0.155

Locus 5295 :

2 alleles

99.2% missing data

3	(0.675) 0.665 0.681

1	(0.325) 0.335 0.319

Locus 5296 :

2 alleles

99.2% missing data

1	(0.612) 0.615 0.610

3	(0.388) 0.385 0.390

Locus 5297 :

2 alleles

99.2% missing data

2	(0.573) 0.566 0.572

1	(0.427) 0.434 0.428

Locus 5298 :

2 alleles

94.9% missing data

1	(0.872) 0.871 0.876

4	(0.128) 0.129 0.124

Locus 5299 :

2 alleles

99.2% missing data

4	(0.674) 0.668 0.680

2	(0.326) 0.332 0.320

Locus 5300 :

2 alleles

90.6% missing data

2	(0.919) 0.931 0.914

3	(0.081) 0.069 0.086

Locus 5301 :

2 alleles

99.2% missing data

1	(0.496) 0.487 0.499

2	(0.504) 0.513 0.501

Locus 5302 :

2 alleles

99.2% missing data

1	(0.430) 0.427 0.430

4	(0.570) 0.573 0.570

Locus 5303 :

2 alleles

98.8% missing data

2	(0.664) 0.668 0.664

3	(0.336) 0.332 0.336

Locus 5304 :

2 alleles

99.2% missing data

1	(0.460) 0.461 0.464

3	(0.540) 0.539 0.536

Locus 5305 :

2 alleles

99.2% missing data

2	(0.566) 0.557 0.572

1	(0.434) 0.443 0.428

Locus 5306 :

2 alleles

99.2% missing data

3	(0.703) 0.704 0.700

4	(0.297) 0.296 0.300

Locus 5307 :

2 alleles

98.8% missing data

4	(0.629) 0.624 0.633

2	(0.371) 0.376 0.367

Locus 5308 :

2 alleles

98.8% missing data

2	(0.261) 0.255 0.272

1	(0.739) 0.745 0.728

Locus 5309 :

2 alleles

99.2% missing data

2	(0.269) 0.276 0.266

4	(0.731) 0.724 0.734

Locus 5310 :

2 alleles

99.2% missing data

3	(0.374) 0.373 0.382

1	(0.626) 0.627 0.618

Locus 5311 :

2 alleles

96.5% missing data

2	(0.773) 0.789 0.767

4	(0.227) 0.211 0.233

Locus 5312 :

2 alleles

99.2% missing data

4	(0.724) 0.722 0.730

2	(0.276) 0.278 0.270

Locus 5313 :

2 alleles

97.7% missing data

3	(0.117) 0.114 0.116

1	(0.883) 0.886 0.884

Locus 5314 :

2 alleles

99.2% missing data

3	(0.570) 0.566 0.582

4	(0.430) 0.434 0.418

Locus 5315 :

2 alleles

99.2% missing data

3	(0.366) 0.373 0.365

1	(0.634) 0.627 0.635

Locus 5316 :

2 alleles

99.2% missing data

2	(0.547) 0.544 0.554

3	(0.453) 0.456 0.446

Locus 5317 :

2 alleles

99.2% missing data

3	(0.339) 0.339 0.342

4	(0.661) 0.661 0.658

Locus 5318 :

2 alleles

99.2% missing data

1	(0.452) 0.455 0.454

3	(0.548) 0.545 0.546

Locus 5319 :

2 alleles

99.2% missing data

1	(0.287) 0.279 0.291

2	(0.713) 0.721 0.709

Locus 5320 :

2 alleles

99.2% missing data

3	(0.869) 0.865 0.873

1	(0.131) 0.135 0.127

Locus 5321 :

2 alleles

99.2% missing data

1	(0.595) 0.593 0.593

3	(0.405) 0.407 0.407

Locus 5322 :

2 alleles

99.2% missing data

1	(0.369) 0.377 0.361

3	(0.631) 0.623 0.639

Locus 5323 :

2 alleles

95.3% missing data

3	(0.487) 0.514 0.459

4	(0.513) 0.486 0.541

Locus 5324 :

2 alleles

98.4% missing data

3	(0.761) 0.766 0.760

4	(0.239) 0.234 0.240

Locus 5325 :

2 alleles

98.8% missing data

1	(0.628) 0.621 0.634

3	(0.372) 0.379 0.366

Locus 5326 :

2 alleles

98.4% missing data

1	(0.714) 0.710 0.727

2	(0.286) 0.290 0.273

Locus 5327 :

2 alleles

99.2% missing data

1	(0.446) 0.446 0.448

3	(0.554) 0.554 0.552

Locus 5328 :

3 alleles

90.6% missing data

1	(0.808) 0.844 0.791

4	(0.099) 0.080 0.107

2	(0.092) 0.076 0.102

Locus 5329 :

2 alleles

99.2% missing data

3	(0.289) 0.292 0.293

4	(0.711) 0.708 0.707

Locus 5330 :

2 alleles

99.2% missing data

2	(0.350) 0.352 0.350

4	(0.650) 0.648 0.650

Locus 5331 :

3 alleles

98.8% missing data

2	(0.297) 0.292 0.305

1	(0.388) 0.386 0.385

4	(0.315) 0.322 0.311

Locus 5332 :

2 alleles

99.2% missing data

1	(0.631) 0.624 0.629

4	(0.369) 0.376 0.371

Locus 5333 :

2 alleles

99.2% missing data

3	(0.719) 0.721 0.712

4	(0.281) 0.279 0.288

Locus 5334 :

2 alleles

99.2% missing data

3	(0.417) 0.415 0.425

1	(0.583) 0.585 0.575

Locus 5335 :

2 alleles

0.0% missing data

3	(0.913) 0.763 0.974

1	(0.087) 0.237 0.026

Locus 5336 :

2 alleles

99.2% missing data

3	(0.342) 0.338 0.337

1	(0.658) 0.662 0.663

Locus 5337 :

2 alleles

99.2% missing data

3	(0.324) 0.326 0.325

2	(0.676) 0.674 0.675

Locus 5338 :

2 alleles

99.2% missing data

4	(0.361) 0.363 0.367

3	(0.639) 0.637 0.633

Locus 5339 :

2 alleles

99.2% missing data

4	(0.457) 0.458 0.463

3	(0.543) 0.542 0.537

Locus 5340 :

2 alleles

98.8% missing data

4	(0.619) 0.618 0.617

1	(0.381) 0.382 0.383

Locus 5341 :

2 alleles

99.2% missing data

1	(0.564) 0.564 0.561

3	(0.436) 0.436 0.439

Locus 5342 :

2 alleles

99.2% missing data

1	(0.516) 0.517 0.518

3	(0.484) 0.483 0.482

Locus 5343 :

2 alleles

98.8% missing data

3	(0.614) 0.612 0.612

2	(0.386) 0.388 0.388

Locus 5344 :

2 alleles

95.3% missing data

2	(0.131) 0.136 0.125

4	(0.869) 0.864 0.875

Locus 5345 :

2 alleles

99.2% missing data

4	(0.452) 0.458 0.448

1	(0.548) 0.542 0.552

Locus 5346 :

2 alleles

97.7% missing data

4	(0.778) 0.778 0.787

3	(0.222) 0.222 0.213

Locus 5347 :

2 alleles

99.2% missing data

2	(0.560) 0.559 0.554

4	(0.440) 0.441 0.446

Locus 5348 :

3 alleles

97.7% missing data

4	(0.495) 0.492 0.504

2	(0.133) 0.132 0.135

1	(0.372) 0.376 0.360

Locus 5349 :

2 alleles

99.2% missing data

4	(0.581) 0.582 0.583

3	(0.419) 0.418 0.417

Locus 5350 :

2 alleles

99.2% missing data

2	(0.862) 0.865 0.853

4	(0.138) 0.135 0.147

Locus 5351 :

2 alleles

96.9% missing data

3	(0.866) 0.869 0.870

4	(0.134) 0.131 0.130

Locus 5352 :

2 alleles

98.8% missing data

4	(0.527) 0.534 0.525

3	(0.473) 0.466 0.475

Locus 5353 :

2 alleles

89.1% missing data

1	(0.771) 0.811 0.724

3	(0.229) 0.189 0.276

Locus 5354 :

2 alleles

99.2% missing data

4	(0.841) 0.831 0.846

1	(0.159) 0.169 0.154

Locus 5355 :

2 alleles

99.2% missing data

3	(0.587) 0.585 0.584

4	(0.413) 0.415 0.416

Locus 5356 :

2 alleles

99.2% missing data

3	(0.561) 0.568 0.552

4	(0.439) 0.432 0.448

Locus 5357 :

2 alleles

99.2% missing data

4	(0.486) 0.485 0.488

3	(0.514) 0.515 0.512

Locus 5358 :

2 alleles

99.2% missing data

1	(0.411) 0.417 0.411

4	(0.589) 0.583 0.589

Locus 5359 :

2 alleles

99.2% missing data

1	(0.761) 0.754 0.766

4	(0.239) 0.246 0.234

Locus 5360 :

2 alleles

99.2% missing data

4	(0.556) 0.554 0.550

2	(0.444) 0.446 0.450

Locus 5361 :

2 alleles

99.2% missing data

3	(0.647) 0.639 0.655

1	(0.353) 0.361 0.345

Locus 5362 :

2 alleles

96.9% missing data

2	(0.850) 0.852 0.852

4	(0.150) 0.148 0.148

Locus 5363 :

2 alleles

99.2% missing data

3	(0.588) 0.587 0.590

1	(0.412) 0.413 0.410

Locus 5364 :

2 alleles

99.2% missing data

1	(0.563) 0.560 0.569

3	(0.437) 0.440 0.431

Locus 5365 :

2 alleles

99.2% missing data

3	(0.464) 0.456 0.468

1	(0.536) 0.544 0.532

Locus 5366 :

2 alleles

99.2% missing data

3	(0.611) 0.602 0.615

4	(0.389) 0.398 0.385

Locus 5367 :

2 alleles

99.2% missing data

2	(0.412) 0.407 0.413

3	(0.588) 0.593 0.587

Locus 5368 :

2 alleles

99.2% missing data

3	(0.598) 0.593 0.600

4	(0.402) 0.407 0.400

Locus 5369 :

2 alleles

99.2% missing data

3	(0.849) 0.843 0.851

4	(0.151) 0.157 0.149

Locus 5370 :

2 alleles

98.0% missing data

1	(0.176) 0.179 0.166

2	(0.824) 0.821 0.834

Locus 5371 :

2 alleles

99.2% missing data

4	(0.701) 0.708 0.700

2	(0.299) 0.292 0.300

Locus 5372 :

2 alleles

99.2% missing data

4	(0.654) 0.651 0.654

1	(0.346) 0.349 0.346

Locus 5373 :

2 alleles

99.2% missing data

3	(0.332) 0.335 0.331

4	(0.668) 0.665 0.669

Locus 5374 :

2 alleles

98.4% missing data

1	(0.402) 0.404 0.395

4	(0.598) 0.596 0.605

Locus 5375 :

2 alleles

99.2% missing data

4	(0.500) 0.507 0.495

2	(0.500) 0.493 0.505

Locus 5376 :

2 alleles

99.2% missing data

3	(0.399) 0.398 0.403

2	(0.601) 0.602 0.597

Locus 5377 :

2 alleles

98.8% missing data

1	(0.568) 0.568 0.572

4	(0.432) 0.432 0.428

Locus 5378 :

2 alleles

99.2% missing data

1	(0.266) 0.263 0.274

3	(0.734) 0.737 0.726

Locus 5379 :

2 alleles

97.3% missing data

1	(0.861) 0.859 0.865

2	(0.139) 0.141 0.135

Locus 5380 :

2 alleles

96.5% missing data

4	(0.719) 0.738 0.715

3	(0.281) 0.262 0.285

Locus 5381 :

2 alleles

99.2% missing data

4	(0.530) 0.529 0.526

3	(0.470) 0.471 0.474

Locus 5382 :

2 alleles

98.8% missing data

1	(0.600) 0.601 0.598

4	(0.400) 0.399 0.402

Locus 5383 :

2 alleles

98.4% missing data

2	(0.417) 0.411 0.416

4	(0.583) 0.589 0.584

Locus 5384 :

2 alleles

96.1% missing data

1	(0.223) 0.215 0.225

3	(0.777) 0.785 0.775

Locus 5385 :

2 alleles

96.9% missing data

3	(0.852) 0.855 0.856

1	(0.148) 0.145 0.144

Locus 5386 :

2 alleles

97.3% missing data

4	(0.759) 0.758 0.761

2	(0.241) 0.242 0.239

Locus 5387 :

3 alleles

97.7% missing data

1	(0.849) 0.848 0.849

4	(0.073) 0.079 0.068

2	(0.078) 0.072 0.084

Locus 5388 :

2 alleles

91.0% missing data

2	(0.885) 0.899 0.881

3	(0.115) 0.101 0.119

Locus 5389 :

2 alleles

99.2% missing data

2	(0.719) 0.727 0.715

1	(0.281) 0.273 0.285

Locus 5390 :

2 alleles

96.1% missing data

2	(0.866) 0.874 0.861

1	(0.134) 0.126 0.139

Locus 5391 :

2 alleles

98.8% missing data

3	(0.717) 0.728 0.714

4	(0.283) 0.272 0.286

Locus 5392 :

2 alleles

98.8% missing data

1	(0.656) 0.648 0.668

3	(0.344) 0.352 0.332

Locus 5393 :

2 alleles

96.9% missing data

3	(0.781) 0.777 0.799

1	(0.219) 0.223 0.201

Locus 5394 :

2 alleles

99.2% missing data

2	(0.362) 0.352 0.367

1	(0.638) 0.648 0.633

Locus 5395 :

2 alleles

98.0% missing data

1	(0.792) 0.781 0.802

2	(0.208) 0.219 0.198

Locus 5396 :

2 alleles

99.2% missing data

2	(0.712) 0.709 0.715

1	(0.288) 0.291 0.285

Locus 5397 :

2 alleles

99.2% missing data

3	(0.173) 0.179 0.172

1	(0.827) 0.821 0.828

Locus 5398 :

2 alleles

99.2% missing data

1	(0.532) 0.532 0.535

4	(0.468) 0.468 0.465

Locus 5399 :

2 alleles

98.8% missing data

4	(0.556) 0.552 0.568

3	(0.444) 0.448 0.432

Locus 5400 :

2 alleles

99.2% missing data

1	(0.437) 0.438 0.439

3	(0.563) 0.562 0.561

Locus 5401 :

2 alleles

99.2% missing data

1	(0.628) 0.620 0.630

3	(0.372) 0.380 0.370

Locus 5402 :

2 alleles

99.2% missing data

1	(0.655) 0.662 0.656

4	(0.345) 0.338 0.344

Locus 5403 :

2 alleles

99.2% missing data

1	(0.695) 0.700 0.695

2	(0.305) 0.300 0.305

Locus 5404 :

2 alleles

99.2% missing data

3	(0.214) 0.218 0.211

1	(0.786) 0.782 0.789

Locus 5405 :

2 alleles

99.2% missing data

2	(0.573) 0.576 0.571

3	(0.427) 0.424 0.429

Locus 5406 :

2 alleles

99.2% missing data

1	(0.364) 0.364 0.368

3	(0.636) 0.636 0.632

Locus 5407 :

2 alleles

99.2% missing data

2	(0.559) 0.555 0.567

1	(0.441) 0.445 0.433

Locus 5408 :

2 alleles

99.2% missing data

4	(0.510) 0.512 0.507

2	(0.490) 0.488 0.493

Locus 5409 :

2 alleles

99.2% missing data

1	(0.407) 0.402 0.410

2	(0.593) 0.598 0.590

Locus 5410 :

2 alleles

94.9% missing data

1	(0.192) 0.198 0.182

4	(0.808) 0.802 0.818

Locus 5411 :

2 alleles

98.4% missing data

1	(0.672) 0.673 0.676

2	(0.328) 0.327 0.324

Locus 5412 :

2 alleles

99.2% missing data

1	(0.711) 0.713 0.712

3	(0.289) 0.287 0.288

Locus 5413 :

3 alleles

98.8% missing data

1	(0.335) 0.333 0.335

4	(0.148) 0.144 0.154

3	(0.517) 0.523 0.511

Locus 5414 :

3 alleles

98.4% missing data

4	(0.241) 0.243 0.242

3	(0.500) 0.501 0.494

1	(0.259) 0.256 0.264

Locus 5415 :

2 alleles

99.2% missing data

4	(0.715) 0.708 0.723

3	(0.285) 0.292 0.277

Locus 5416 :

2 alleles

99.2% missing data

2	(0.324) 0.318 0.332

4	(0.676) 0.682 0.668

Locus 5417 :

2 alleles

99.2% missing data

3	(0.627) 0.621 0.635

1	(0.373) 0.379 0.365

Locus 5418 :

2 alleles

92.2% missing data

3	(0.867) 0.885 0.858

2	(0.133) 0.115 0.142

Locus 5419 :

2 alleles

99.2% missing data

2	(0.175) 0.182 0.173

3	(0.825) 0.818 0.827

Locus 5420 :

2 alleles

99.2% missing data

4	(0.790) 0.786 0.800

2	(0.210) 0.214 0.200

Locus 5421 :

2 alleles

98.4% missing data

2	(0.627) 0.631 0.629

1	(0.373) 0.369 0.371

Locus 5422 :

2 alleles

99.2% missing data

3	(0.386) 0.380 0.394

4	(0.614) 0.620 0.606

Locus 5423 :

2 alleles

99.2% missing data

4	(0.480) 0.470 0.489

1	(0.520) 0.530 0.511

Locus 5424 :

2 alleles

99.2% missing data

3	(0.243) 0.241 0.250

1	(0.757) 0.759 0.750

Locus 5425 :

2 alleles

98.8% missing data

3	(0.252) 0.249 0.259

1	(0.748) 0.751 0.741

Locus 5426 :

2 alleles

96.5% missing data

3	(0.817) 0.828 0.814

4	(0.183) 0.172 0.186

Locus 5427 :

2 alleles

99.2% missing data

1	(0.542) 0.541 0.540

2	(0.458) 0.459 0.460

Locus 5428 :

2 alleles

99.2% missing data

1	(0.525) 0.523 0.526

4	(0.475) 0.477 0.474

Locus 5429 :

2 alleles

96.1% missing data

2	(0.774) 0.787 0.770

4	(0.226) 0.213 0.230

Locus 5430 :

2 alleles

99.2% missing data

2	(0.260) 0.259 0.270

1	(0.740) 0.741 0.730

Locus 5431 :

2 alleles

93.0% missing data

3	(0.943) 0.934 0.953

1	(0.057) 0.066 0.047

Locus 5432 :

3 alleles

92.2% missing data

1	(0.756) 0.751 0.777

2	(0.157) 0.153 0.151

3	(0.086) 0.096 0.071

Locus 5433 :

2 alleles

99.2% missing data

3	(0.565) 0.568 0.560

4	(0.435) 0.432 0.440

Locus 5434 :

2 alleles

99.2% missing data

2	(0.646) 0.642 0.648

4	(0.354) 0.358 0.352

Locus 5435 :

2 alleles

97.3% missing data

3	(0.284) 0.284 0.286

1	(0.716) 0.716 0.714

Locus 5436 :

2 alleles

99.2% missing data

4	(0.362) 0.367 0.363

2	(0.638) 0.633 0.637

Locus 5437 :

2 alleles

99.2% missing data

2	(0.565) 0.564 0.564

1	(0.435) 0.436 0.436

Locus 5438 :

2 alleles

98.8% missing data

2	(0.557) 0.545 0.565

1	(0.443) 0.455 0.435

Locus 5439 :

2 alleles

99.2% missing data

1	(0.476) 0.472 0.478

2	(0.524) 0.528 0.522

Locus 5440 :

2 alleles

98.8% missing data

1	(0.695) 0.688 0.699

4	(0.305) 0.312 0.301

Locus 5441 :

2 alleles

99.2% missing data

4	(0.290) 0.291 0.293

2	(0.710) 0.709 0.707

Locus 5442 :

2 alleles

98.8% missing data

2	(0.242) 0.250 0.236

3	(0.758) 0.750 0.764

Locus 5443 :

2 alleles

99.2% missing data

1	(0.501) 0.503 0.504

2	(0.499) 0.497 0.496

Locus 5444 :

2 alleles

96.5% missing data

1	(0.771) 0.784 0.770

4	(0.229) 0.216 0.230

Locus 5445 :

3 alleles

98.8% missing data

3	(0.418) 0.417 0.426

2	(0.211) 0.210 0.212

1	(0.372) 0.373 0.362

Locus 5446 :

2 alleles

99.2% missing data

4	(0.589) 0.585 0.587

1	(0.411) 0.415 0.413

Locus 5447 :

2 alleles

99.2% missing data

4	(0.556) 0.550 0.556

2	(0.444) 0.450 0.444

Locus 5448 :

2 alleles

95.3% missing data

4	(0.168) 0.175 0.158

1	(0.832) 0.825 0.842

Locus 5449 :

2 alleles

98.8% missing data

1	(0.567) 0.566 0.568

3	(0.433) 0.434 0.432

Locus 5450 :

2 alleles

99.2% missing data

3	(0.335) 0.332 0.343

1	(0.665) 0.668 0.657

Locus 5451 :

2 alleles

99.2% missing data

2	(0.270) 0.266 0.273

1	(0.730) 0.734 0.727

Locus 5452 :

2 alleles

99.2% missing data

3	(0.317) 0.319 0.313

2	(0.683) 0.681 0.687

Locus 5453 :

2 alleles

96.5% missing data

3	(0.851) 0.849 0.855

1	(0.149) 0.151 0.145

Locus 5454 :

3 alleles

97.3% missing data

4	(0.656) 0.663 0.645

3	(0.164) 0.160 0.166

1	(0.180) 0.177 0.189

Locus 5455 :

2 alleles

99.2% missing data

1	(0.715) 0.714 0.706

3	(0.285) 0.286 0.294

Locus 5456 :

2 alleles

99.2% missing data

1	(0.727) 0.725 0.728

3	(0.273) 0.275 0.272

Locus 5457 :

2 alleles

99.2% missing data

4	(0.438) 0.432 0.441

1	(0.562) 0.568 0.559

Locus 5458 :

2 alleles

99.2% missing data

4	(0.424) 0.426 0.432

3	(0.576) 0.574 0.568

Locus 5459 :

2 alleles

99.2% missing data

3	(0.340) 0.338 0.341

2	(0.660) 0.662 0.659

Locus 5460 :

2 alleles

99.2% missing data

3	(0.563) 0.561 0.569

4	(0.437) 0.439 0.431

Locus 5461 :

2 alleles

99.2% missing data

3	(0.618) 0.619 0.618

1	(0.382) 0.381 0.382

Locus 5462 :

3 alleles

93.8% missing data

2	(0.684) 0.673 0.695

3	(0.263) 0.271 0.255

4	(0.053) 0.055 0.050

Locus 5463 :

2 alleles

98.8% missing data

3	(0.388) 0.399 0.381

1	(0.612) 0.601 0.619

Locus 5464 :

2 alleles

99.2% missing data

3	(0.850) 0.844 0.851

1	(0.150) 0.156 0.149

Locus 5465 :

2 alleles

99.2% missing data

4	(0.303) 0.304 0.300

3	(0.697) 0.696 0.700

Locus 5466 :

2 alleles

99.2% missing data

4	(0.255) 0.258 0.255

3	(0.745) 0.742 0.745

Locus 5467 :

3 alleles

98.8% missing data

3	(0.179) 0.172 0.182

1	(0.340) 0.337 0.341

2	(0.481) 0.491 0.476

Locus 5468 :

2 alleles

98.8% missing data

2	(0.218) 0.218 0.224

1	(0.782) 0.782 0.776

Locus 5469 :

2 alleles

98.0% missing data

1	(0.776) 0.779 0.781

3	(0.224) 0.221 0.219

Locus 5470 :

2 alleles

99.2% missing data

1	(0.515) 0.520 0.513

2	(0.485) 0.480 0.487

Locus 5471 :

2 alleles

98.8% missing data

4	(0.524) 0.523 0.519

1	(0.476) 0.477 0.481

Locus 5472 :

2 alleles

87.5% missing data

4	(0.937) 0.931 0.949

1	(0.063) 0.069 0.051

Locus 5473 :

2 alleles

99.2% missing data

3	(0.561) 0.550 0.565

4	(0.439) 0.450 0.435

Locus 5474 :

2 alleles

99.2% missing data

3	(0.430) 0.429 0.432

1	(0.570) 0.571 0.568

Locus 5475 :

2 alleles

99.2% missing data

2	(0.360) 0.363 0.363

3	(0.640) 0.637 0.637

Locus 5476 :

2 alleles

99.2% missing data

4	(0.562) 0.557 0.561

2	(0.438) 0.443 0.439

Locus 5477 :

2 alleles

98.8% missing data

4	(0.546) 0.538 0.557

1	(0.454) 0.462 0.443

Locus 5478 :

2 alleles

98.8% missing data

1	(0.339) 0.347 0.335

4	(0.661) 0.653 0.665

Locus 5479 :

2 alleles

99.2% missing data

1	(0.153) 0.155 0.157

4	(0.847) 0.845 0.843

Locus 5480 :

2 alleles

99.2% missing data

4	(0.611) 0.619 0.604

1	(0.389) 0.381 0.396

Locus 5481 :

3 alleles

98.8% missing data

1	(0.498) 0.506 0.494

2	(0.211) 0.206 0.213

3	(0.291) 0.289 0.292

Locus 5482 :

2 alleles

99.2% missing data

3	(0.333) 0.330 0.341

4	(0.667) 0.670 0.659

Locus 5483 :

2 alleles

99.2% missing data

1	(0.600) 0.604 0.591

2	(0.400) 0.396 0.409

Locus 5484 :

2 alleles

99.2% missing data

2	(0.646) 0.650 0.644

4	(0.354) 0.350 0.356

Locus 5485 :

2 alleles

97.7% missing data

4	(0.469) 0.485 0.464

3	(0.531) 0.515 0.536

Locus 5486 :

2 alleles

99.2% missing data

1	(0.627) 0.626 0.622

3	(0.373) 0.374 0.378

Locus 5487 :

2 alleles

90.6% missing data

4	(0.894) 0.911 0.888

1	(0.106) 0.089 0.112

Locus 5488 :

2 alleles

99.2% missing data

3	(0.299) 0.296 0.299

1	(0.701) 0.704 0.701

Locus 5489 :

2 alleles

99.2% missing data

4	(0.491) 0.498 0.491

2	(0.509) 0.502 0.509

Locus 5490 :

2 alleles

99.2% missing data

3	(0.328) 0.323 0.330

2	(0.672) 0.677 0.670

Locus 5491 :

2 alleles

98.0% missing data

3	(0.674) 0.680 0.676

1	(0.326) 0.320 0.324

Locus 5492 :

2 alleles

99.2% missing data

1	(0.398) 0.403 0.405

2	(0.602) 0.597 0.595

Locus 5493 :

2 alleles

99.2% missing data

1	(0.372) 0.365 0.374

2	(0.628) 0.635 0.626

Locus 5494 :

2 alleles

99.2% missing data

1	(0.865) 0.862 0.872

3	(0.135) 0.138 0.128

Locus 5495 :

2 alleles

99.2% missing data

2	(0.660) 0.663 0.654

4	(0.340) 0.337 0.346

Locus 5496 :

2 alleles

99.2% missing data

2	(0.358) 0.357 0.365

1	(0.642) 0.643 0.635

Locus 5497 :

2 alleles

99.2% missing data

2	(0.223) 0.229 0.222

1	(0.777) 0.771 0.778

Locus 5498 :

2 alleles

99.2% missing data

1	(0.329) 0.326 0.328

3	(0.671) 0.674 0.672

Locus 5499 :

2 alleles

90.2% missing data

1	(0.900) 0.919 0.894

4	(0.100) 0.081 0.106

Locus 5500 :

2 alleles

95.7% missing data

2	(0.195) 0.178 0.209

3	(0.805) 0.822 0.791

Locus 5501 :

2 alleles

0.0% missing data

1	(0.982) 0.979 0.994

2	(0.018) 0.021 0.006

Locus 5502 :

2 alleles

0.0% missing data

1	(0.970) 0.971 0.978

2	(0.030) 0.029 0.022

Locus 5503 :

2 alleles

98.4% missing data

4	(0.567) 0.574 0.563

1	(0.433) 0.426 0.437

Locus 5504 :

2 alleles

97.7% missing data

1	(0.834) 0.838 0.839

2	(0.166) 0.162 0.161

Locus 5505 :

2 alleles

94.9% missing data

1	(0.866) 0.869 0.873

2	(0.134) 0.131 0.127

Locus 5506 :

2 alleles

99.2% missing data

1	(0.618) 0.613 0.619

4	(0.382) 0.387 0.381

Locus 5507 :

2 alleles

99.2% missing data

1	(0.501) 0.502 0.505

3	(0.499) 0.498 0.495

Locus 5508 :

2 alleles

99.2% missing data

4	(0.232) 0.230 0.234

2	(0.768) 0.770 0.766

Locus 5509 :

3 alleles

98.0% missing data

1	(0.428) 0.438 0.419

3	(0.190) 0.180 0.200

4	(0.383) 0.383 0.381

Locus 5510 :

2 alleles

99.2% missing data

3	(0.615) 0.611 0.615

4	(0.385) 0.389 0.385

Locus 5511 :

2 alleles

99.2% missing data

1	(0.372) 0.375 0.363

3	(0.628) 0.625 0.637

Locus 5512 :

2 alleles

99.2% missing data

4	(0.353) 0.352 0.353

1	(0.647) 0.648 0.647

Locus 5513 :

2 alleles

99.2% missing data

3	(0.554) 0.555 0.551

2	(0.446) 0.445 0.449

Locus 5514 :

2 alleles

99.2% missing data

1	(0.430) 0.424 0.437

4	(0.570) 0.576 0.563

Locus 5515 :

2 alleles

96.5% missing data

3	(0.806) 0.808 0.813

1	(0.194) 0.192 0.187

Locus 5516 :

2 alleles

99.2% missing data

4	(0.553) 0.561 0.546

3	(0.447) 0.439 0.454

Locus 5517 :

2 alleles

96.1% missing data

3	(0.907) 0.906 0.910

1	(0.093) 0.094 0.090

Locus 5518 :

2 alleles

99.2% missing data

1	(0.375) 0.376 0.372

4	(0.625) 0.624 0.628

Locus 5519 :

2 alleles

99.2% missing data

1	(0.363) 0.366 0.357

4	(0.637) 0.634 0.643

Locus 5520 :

2 alleles

99.2% missing data

3	(0.345) 0.334 0.350

1	(0.655) 0.666 0.650

Locus 5521 :

2 alleles

92.6% missing data

3	(0.896) 0.913 0.885

1	(0.104) 0.087 0.115

Locus 5522 :

2 alleles

99.2% missing data

2	(0.593) 0.597 0.591

4	(0.407) 0.403 0.409

Locus 5523 :

2 alleles

99.2% missing data

2	(0.567) 0.569 0.572

3	(0.433) 0.431 0.428

Locus 5524 :

2 alleles

97.7% missing data

2	(0.869) 0.872 0.869

3	(0.131) 0.128 0.131

Locus 5525 :

3 alleles

98.8% missing data

1	(0.271) 0.265 0.275

3	(0.467) 0.460 0.471

4	(0.262) 0.275 0.254

Locus 5526 :

2 alleles

99.2% missing data

4	(0.621) 0.616 0.625

1	(0.379) 0.384 0.375

Locus 5527 :

2 alleles

93.0% missing data

3	(0.902) 0.898 0.909

2	(0.098) 0.102 0.091

Locus 5528 :

2 alleles

99.2% missing data

1	(0.522) 0.519 0.523

2	(0.478) 0.481 0.477

Locus 5529 :

2 alleles

94.9% missing data

4	(0.523) 0.516 0.534

3	(0.477) 0.484 0.466

Locus 5530 :

2 alleles

96.1% missing data

4	(0.184) 0.168 0.189

3	(0.816) 0.832 0.811

Locus 5531 :

2 alleles

99.2% missing data

4	(0.769) 0.760 0.776

2	(0.231) 0.240 0.224

Locus 5532 :

2 alleles

99.2% missing data

3	(0.692) 0.684 0.694

4	(0.308) 0.316 0.306

Locus 5533 :

2 alleles

98.8% missing data

2	(0.392) 0.385 0.399

4	(0.608) 0.615 0.601

Locus 5534 :

3 alleles

98.8% missing data

4	(0.602) 0.596 0.607

3	(0.257) 0.264 0.248

1	(0.142) 0.140 0.145

Locus 5535 :

2 alleles

98.0% missing data

1	(0.663) 0.665 0.666

4	(0.337) 0.335 0.334

Locus 5536 :

2 alleles

8.2% missing data

1	(0.986) 0.978 0.996

2	(0.014) 0.022 0.004

Locus 5537 :

2 alleles

98.0% missing data

2	(0.715) 0.711 0.722

1	(0.285) 0.289 0.278

Locus 5538 :

3 alleles

98.8% missing data

1	(0.220) 0.219 0.221

3	(0.502) 0.504 0.495

4	(0.278) 0.277 0.284

Locus 5539 :

2 alleles

99.2% missing data

2	(0.262) 0.258 0.263

1	(0.738) 0.742 0.737

Locus 5540 :

2 alleles

97.7% missing data

2	(0.647) 0.648 0.655

3	(0.353) 0.352 0.345

Locus 5541 :

2 alleles

99.2% missing data

4	(0.565) 0.556 0.568

1	(0.435) 0.444 0.432

Locus 5542 :

2 alleles

99.2% missing data

2	(0.470) 0.475 0.467

1	(0.530) 0.525 0.533

Locus 5543 :

2 alleles

99.2% missing data

4	(0.542) 0.534 0.546

2	(0.458) 0.466 0.454

Locus 5544 :

2 alleles

99.2% missing data

1	(0.646) 0.645 0.640

4	(0.354) 0.355 0.360

Locus 5545 :

2 alleles

99.2% missing data

3	(0.332) 0.330 0.337

2	(0.668) 0.670 0.663

Locus 5546 :

2 alleles

99.2% missing data

3	(0.551) 0.547 0.554

1	(0.449) 0.453 0.446

Locus 5547 :

2 alleles

99.2% missing data

4	(0.368) 0.362 0.367

3	(0.632) 0.638 0.633

Locus 5548 :

2 alleles

99.2% missing data

1	(0.414) 0.408 0.416

3	(0.586) 0.592 0.584

Locus 5549 :

2 alleles

99.2% missing data

1	(0.639) 0.640 0.637

4	(0.361) 0.360 0.363

Locus 5550 :

2 alleles

93.0% missing data

3	(0.843) 0.832 0.868

2	(0.157) 0.168 0.132

Locus 5551 :

2 alleles

98.8% missing data

3	(0.435) 0.436 0.440

2	(0.565) 0.564 0.560

Locus 5552 :

2 alleles

97.3% missing data

4	(0.745) 0.753 0.752

3	(0.255) 0.247 0.248

Locus 5553 :

2 alleles

99.2% missing data

4	(0.485) 0.485 0.483

1	(0.515) 0.515 0.517

Locus 5554 :

2 alleles

98.8% missing data

1	(0.248) 0.252 0.242

2	(0.752) 0.748 0.758

Locus 5555 :

2 alleles

99.2% missing data

1	(0.619) 0.619 0.619

4	(0.381) 0.381 0.381

Locus 5556 :

2 alleles

99.2% missing data

1	(0.395) 0.397 0.394

2	(0.605) 0.603 0.606

Locus 5557 :

2 alleles

98.8% missing data

1	(0.487) 0.482 0.497

3	(0.513) 0.518 0.503

Locus 5558 :

2 alleles

99.2% missing data

2	(0.427) 0.418 0.431

1	(0.573) 0.582 0.569

Locus 5559 :

2 alleles

99.2% missing data

2	(0.683) 0.686 0.678

3	(0.317) 0.314 0.322

Locus 5560 :

2 alleles

99.2% missing data

1	(0.337) 0.341 0.333

4	(0.663) 0.659 0.667

Locus 5561 :

2 alleles

98.8% missing data

1	(0.588) 0.585 0.591

4	(0.412) 0.415 0.409

Locus 5562 :

2 alleles

95.3% missing data

3	(0.138) 0.143 0.128

1	(0.862) 0.857 0.872

Locus 5563 :

2 alleles

99.2% missing data

1	(0.325) 0.321 0.331

4	(0.675) 0.679 0.669

Locus 5564 :

2 alleles

99.2% missing data

1	(0.231) 0.226 0.239

4	(0.769) 0.774 0.761

Locus 5565 :

2 alleles

99.2% missing data

4	(0.584) 0.587 0.588

2	(0.416) 0.413 0.412

Locus 5566 :

2 alleles

99.2% missing data

1	(0.344) 0.341 0.351

2	(0.656) 0.659 0.649

Locus 5567 :

2 alleles

98.8% missing data

1	(0.640) 0.632 0.646

2	(0.360) 0.368 0.354

Locus 5568 :

2 alleles

96.9% missing data

1	(0.815) 0.815 0.817

3	(0.185) 0.185 0.183

Locus 5569 :

2 alleles

91.0% missing data

4	(0.670) 0.628 0.713

2	(0.330) 0.372 0.287

Locus 5570 :

2 alleles

90.2% missing data

2	(0.930) 0.927 0.936

4	(0.070) 0.073 0.064

Locus 5571 :

2 alleles

96.9% missing data

4	(0.809) 0.810 0.814

2	(0.191) 0.190 0.186

Locus 5572 :

2 alleles

95.3% missing data

4	(0.887) 0.887 0.891

1	(0.113) 0.113 0.109

Locus 5573 :

2 alleles

98.4% missing data

4	(0.592) 0.599 0.588

2	(0.408) 0.401 0.412

Locus 5574 :

2 alleles

96.1% missing data

4	(0.783) 0.788 0.787

2	(0.217) 0.212 0.213

Locus 5575 :

2 alleles

99.2% missing data

2	(0.313) 0.312 0.316

1	(0.687) 0.688 0.684

Locus 5576 :

3 alleles

98.8% missing data

3	(0.307) 0.310 0.304

4	(0.370) 0.363 0.372

1	(0.323) 0.328 0.324

Locus 5577 :

2 alleles

99.2% missing data

1	(0.425) 0.423 0.425

2	(0.575) 0.577 0.575

Locus 5578 :

2 alleles

99.2% missing data

2	(0.704) 0.714 0.697

1	(0.296) 0.286 0.303

Locus 5579 :

2 alleles

97.7% missing data

2	(0.701) 0.701 0.707

4	(0.299) 0.299 0.293

Locus 5580 :

2 alleles

99.2% missing data

2	(0.750) 0.745 0.753

1	(0.250) 0.255 0.247

Locus 5581 :

2 alleles

96.9% missing data

2	(0.801) 0.812 0.793

4	(0.199) 0.188 0.207

Locus 5582 :

2 alleles

98.8% missing data

4	(0.773) 0.770 0.778

2	(0.227) 0.230 0.222

Locus 5583 :

3 alleles

95.7% missing data

1	(0.711) 0.727 0.701

2	(0.189) 0.180 0.194

3	(0.101) 0.093 0.105

Locus 5584 :

2 alleles

98.4% missing data

3	(0.221) 0.224 0.220

2	(0.779) 0.776 0.780

Locus 5585 :

2 alleles

96.9% missing data

2	(0.775) 0.771 0.779

1	(0.225) 0.229 0.221

Locus 5586 :

2 alleles

99.2% missing data

3	(0.629) 0.628 0.629

1	(0.371) 0.372 0.371

Locus 5587 :

2 alleles

99.2% missing data

1	(0.340) 0.335 0.345

3	(0.660) 0.665 0.655

Locus 5588 :

2 alleles

99.2% missing data

1	(0.731) 0.734 0.727

2	(0.269) 0.266 0.273

Locus 5589 :

2 alleles

91.0% missing data

4	(0.925) 0.936 0.917

2	(0.075) 0.064 0.083

Locus 5590 :

2 alleles

99.2% missing data

1	(0.319) 0.317 0.318

3	(0.681) 0.683 0.682

Locus 5591 :

3 alleles

98.4% missing data

2	(0.208) 0.208 0.209

3	(0.290) 0.290 0.287

4	(0.502) 0.503 0.504

Locus 5592 :

2 alleles

99.2% missing data

2	(0.567) 0.568 0.560

3	(0.433) 0.432 0.440

Locus 5593 :

2 alleles

98.0% missing data

3	(0.551) 0.562 0.553

1	(0.449) 0.438 0.447

Locus 5594 :

2 alleles

0.4% missing data

2	(0.984) 0.988 0.992

3	(0.016) 0.012 0.008

Locus 5595 :

2 alleles

96.9% missing data

4	(0.806) 0.817 0.803

2	(0.194) 0.183 0.197

Locus 5596 :

2 alleles

99.2% missing data

4	(0.556) 0.551 0.561

3	(0.444) 0.449 0.439

Locus 5597 :

2 alleles

99.2% missing data

2	(0.508) 0.510 0.503

1	(0.492) 0.490 0.497

Locus 5598 :

2 alleles

98.0% missing data

3	(0.671) 0.682 0.664

2	(0.329) 0.318 0.336

Locus 5599 :

2 alleles

97.3% missing data

2	(0.354) 0.356 0.342

1	(0.646) 0.644 0.658

Locus 5600 :

2 alleles

99.2% missing data

4	(0.373) 0.376 0.378

1	(0.627) 0.624 0.622

Locus 5601 :

2 alleles

98.8% missing data

1	(0.544) 0.547 0.548

4	(0.456) 0.453 0.452

Locus 5602 :

3 alleles

98.8% missing data

3	(0.238) 0.232 0.244

2	(0.319) 0.320 0.321

1	(0.443) 0.448 0.435

Locus 5603 :

2 alleles

99.2% missing data

2	(0.566) 0.566 0.571

4	(0.434) 0.434 0.429

Locus 5604 :

2 alleles

99.2% missing data

2	(0.609) 0.603 0.613

3	(0.391) 0.397 0.387

Locus 5605 :

2 alleles

91.0% missing data

4	(0.900) 0.918 0.892

2	(0.100) 0.082 0.108

Locus 5606 :

2 alleles

99.2% missing data

2	(0.382) 0.386 0.378

1	(0.618) 0.614 0.622

Locus 5607 :

2 alleles

99.2% missing data

3	(0.637) 0.629 0.644

1	(0.363) 0.371 0.356

Locus 5608 :

2 alleles

92.2% missing data

4	(0.805) 0.798 0.821

2	(0.195) 0.202 0.179

Locus 5609 :

3 alleles

98.8% missing data

1	(0.380) 0.376 0.386

3	(0.382) 0.380 0.377

4	(0.238) 0.244 0.237

Locus 5610 :

2 alleles

99.2% missing data

3	(0.475) 0.471 0.483

1	(0.525) 0.529 0.517

Locus 5611 :

2 alleles

99.2% missing data

1	(0.179) 0.177 0.187

2	(0.821) 0.823 0.813

Locus 5612 :

2 alleles

94.1% missing data

2	(0.857) 0.854 0.868

3	(0.143) 0.146 0.132

Locus 5613 :

2 alleles

99.2% missing data

4	(0.378) 0.373 0.385

3	(0.622) 0.627 0.615

Locus 5614 :

2 alleles

99.2% missing data

4	(0.288) 0.284 0.291

1	(0.712) 0.716 0.709

Locus 5615 :

2 alleles

98.8% missing data

1	(0.593) 0.595 0.591

4	(0.407) 0.405 0.409

Locus 5616 :

2 alleles

98.8% missing data

3	(0.454) 0.453 0.458

1	(0.546) 0.547 0.542

Locus 5617 :

2 alleles

99.2% missing data

1	(0.474) 0.465 0.474

3	(0.526) 0.535 0.526

Locus 5618 :

2 alleles

98.8% missing data

2	(0.558) 0.554 0.571

3	(0.442) 0.446 0.429

Locus 5619 :

2 alleles

99.2% missing data

4	(0.436) 0.444 0.432

3	(0.564) 0.556 0.568

Locus 5620 :

2 alleles

99.2% missing data

3	(0.699) 0.699 0.696

1	(0.301) 0.301 0.304

Locus 5621 :

2 alleles

98.8% missing data

3	(0.373) 0.367 0.380

1	(0.627) 0.633 0.620

Locus 5622 :

2 alleles

98.8% missing data

4	(0.807) 0.810 0.799

1	(0.193) 0.190 0.201

Locus 5623 :

2 alleles

99.2% missing data

3	(0.443) 0.442 0.443

1	(0.557) 0.558 0.557

Locus 5624 :

2 alleles

97.3% missing data

3	(0.324) 0.330 0.313

2	(0.676) 0.670 0.687

Locus 5625 :

3 alleles

98.8% missing data

4	(0.325) 0.331 0.321

3	(0.182) 0.178 0.187

1	(0.493) 0.491 0.492

Locus 5626 :

2 alleles

97.3% missing data

3	(0.846) 0.849 0.848

4	(0.154) 0.151 0.152

Locus 5627 :

3 alleles

98.8% missing data

3	(0.420) 0.417 0.419

1	(0.357) 0.357 0.354

2	(0.224) 0.226 0.227

Locus 5628 :

2 alleles

99.2% missing data

2	(0.467) 0.463 0.461

1	(0.533) 0.537 0.539

Locus 5629 :

2 alleles

99.2% missing data

4	(0.651) 0.645 0.651

2	(0.349) 0.355 0.349

Locus 5630 :

2 alleles

99.2% missing data

3	(0.312) 0.316 0.320

4	(0.688) 0.684 0.680

Locus 5631 :

2 alleles

96.9% missing data

1	(0.391) 0.381 0.401

4	(0.609) 0.619 0.599

Locus 5632 :

2 alleles

99.2% missing data

4	(0.713) 0.714 0.715

1	(0.287) 0.286 0.285

Locus 5633 :

2 alleles

98.0% missing data

3	(0.670) 0.670 0.680

4	(0.330) 0.330 0.320

Locus 5634 :

2 alleles

98.4% missing data

1	(0.492) 0.481 0.505

2	(0.508) 0.519 0.495

Locus 5635 :

2 alleles

99.2% missing data

1	(0.294) 0.295 0.297

2	(0.706) 0.705 0.703

Locus 5636 :

3 alleles

98.8% missing data

2	(0.384) 0.382 0.383

3	(0.438) 0.431 0.443

4	(0.179) 0.187 0.175

Locus 5637 :

2 alleles

99.2% missing data

2	(0.617) 0.618 0.618

1	(0.383) 0.382 0.382

Locus 5638 :

2 alleles

98.8% missing data

1	(0.750) 0.751 0.753

2	(0.250) 0.249 0.247

Locus 5639 :

3 alleles

98.8% missing data

4	(0.274) 0.273 0.277

1	(0.488) 0.482 0.488

3	(0.239) 0.244 0.235

Locus 5640 :

2 alleles

99.2% missing data

1	(0.238) 0.236 0.240

2	(0.762) 0.764 0.760

Locus 5641 :

2 alleles

99.2% missing data

4	(0.371) 0.375 0.369

1	(0.629) 0.625 0.631

Locus 5642 :

2 alleles

99.2% missing data

2	(0.413) 0.413 0.409

4	(0.587) 0.587 0.591

Locus 5643 :

2 alleles

99.2% missing data

1	(0.683) 0.681 0.679

3	(0.317) 0.319 0.321

Locus 5644 :

2 alleles

99.2% missing data

1	(0.634) 0.631 0.643

4	(0.366) 0.369 0.357

Locus 5645 :

2 alleles

93.8% missing data

4	(0.913) 0.908 0.924

1	(0.087) 0.092 0.076

Locus 5646 :

2 alleles

98.8% missing data

1	(0.343) 0.340 0.346

4	(0.657) 0.660 0.654

Locus 5647 :

2 alleles

99.2% missing data

4	(0.657) 0.650 0.663

3	(0.343) 0.350 0.337

Locus 5648 :

2 alleles

99.2% missing data

4	(0.427) 0.430 0.429

3	(0.573) 0.570 0.571

Locus 5649 :

2 alleles

99.2% missing data

1	(0.409) 0.409 0.409

4	(0.591) 0.591 0.591

Locus 5650 :

2 alleles

99.2% missing data

3	(0.707) 0.702 0.712

4	(0.293) 0.298 0.288

Locus 5651 :

2 alleles

99.2% missing data

1	(0.574) 0.578 0.577

3	(0.426) 0.422 0.423

Locus 5652 :

2 alleles

16.8% missing data

1	(0.982) 0.973 0.994

4	(0.018) 0.027 0.006

Locus 5653 :

2 alleles

99.2% missing data

4	(0.630) 0.633 0.627

3	(0.370) 0.367 0.373

Locus 5654 :

2 alleles

96.9% missing data

1	(0.744) 0.743 0.754

4	(0.256) 0.257 0.246

Locus 5655 :

2 alleles

99.2% missing data

4	(0.732) 0.737 0.723

3	(0.268) 0.263 0.277

Locus 5656 :

2 alleles

98.8% missing data

2	(0.682) 0.683 0.689

1	(0.318) 0.317 0.311

Locus 5657 :

2 alleles

99.2% missing data

2	(0.748) 0.749 0.741

3	(0.252) 0.251 0.259

Locus 5658 :

2 alleles

97.7% missing data

4	(0.187) 0.190 0.187

1	(0.813) 0.810 0.813

Locus 5659 :

2 alleles

99.2% missing data

1	(0.705) 0.709 0.703

3	(0.295) 0.291 0.297

Locus 5660 :

2 alleles

99.2% missing data

2	(0.752) 0.750 0.755

3	(0.248) 0.250 0.245

Locus 5661 :

3 alleles

98.4% missing data

4	(0.127) 0.125 0.131

3	(0.477) 0.484 0.478

1	(0.397) 0.391 0.391

Locus 5662 :

2 alleles

99.2% missing data

2	(0.791) 0.784 0.794

4	(0.209) 0.216 0.206

Locus 5663 :

2 alleles

99.2% missing data

1	(0.594) 0.589 0.591

2	(0.406) 0.411 0.409

Locus 5664 :

2 alleles

99.2% missing data

3	(0.250) 0.245 0.260

1	(0.750) 0.755 0.740

Locus 5665 :

2 alleles

99.2% missing data

2	(0.334) 0.329 0.332

1	(0.666) 0.671 0.668

Locus 5666 :

3 alleles

98.8% missing data

1	(0.226) 0.224 0.230

3	(0.296) 0.292 0.299

2	(0.478) 0.484 0.472

Locus 5667 :

2 alleles

99.2% missing data

4	(0.768) 0.762 0.776

2	(0.232) 0.238 0.224

Locus 5668 :

2 alleles

98.8% missing data

2	(0.717) 0.719 0.715

3	(0.283) 0.281 0.285

Locus 5669 :

2 alleles

99.2% missing data

4	(0.242) 0.239 0.251

3	(0.758) 0.761 0.749

Locus 5670 :

3 alleles

98.8% missing data

3	(0.295) 0.293 0.296

4	(0.302) 0.301 0.305

1	(0.402) 0.406 0.399

Locus 5671 :

2 alleles

99.2% missing data

3	(0.466) 0.461 0.477

4	(0.534) 0.539 0.523

Locus 5672 :

2 alleles

1.2% missing data

4	(0.988) 0.982 0.996

1	(0.012) 0.018 0.004

Locus 5673 :

2 alleles

99.2% missing data

4	(0.506) 0.509 0.506

3	(0.494) 0.491 0.494

Locus 5674 :

2 alleles

99.2% missing data

4	(0.781) 0.774 0.780

3	(0.219) 0.226 0.220

Locus 5675 :

2 alleles

99.2% missing data

3	(0.689) 0.682 0.691

1	(0.311) 0.318 0.309

Locus 5676 :

2 alleles

99.2% missing data

3	(0.213) 0.208 0.219

2	(0.787) 0.792 0.781

Locus 5677 :

2 alleles

99.2% missing data

1	(0.365) 0.369 0.364

3	(0.635) 0.631 0.636

Locus 5678 :

2 alleles

99.2% missing data

4	(0.565) 0.559 0.570

1	(0.435) 0.441 0.430

Locus 5679 :

2 alleles

0.4% missing data

2	(0.968) 0.976 0.968

1	(0.032) 0.024 0.032

Locus 5680 :

3 alleles

98.8% missing data

3	(0.388) 0.385 0.389

2	(0.318) 0.323 0.317

4	(0.294) 0.292 0.295

Locus 5681 :

2 alleles

99.2% missing data

1	(0.581) 0.577 0.585

2	(0.419) 0.423 0.415

Locus 5682 :

2 alleles

96.1% missing data

3	(0.848) 0.864 0.841

1	(0.152) 0.136 0.159

Locus 5683 :

2 alleles

98.8% missing data

4	(0.728) 0.726 0.728

2	(0.272) 0.274 0.272

Locus 5684 :

2 alleles

99.2% missing data

2	(0.402) 0.396 0.416

1	(0.598) 0.604 0.584

Locus 5685 :

2 alleles

98.4% missing data

3	(0.774) 0.775 0.769

1	(0.226) 0.225 0.231

Locus 5686 :

3 alleles

98.4% missing data

3	(0.398) 0.405 0.394

4	(0.472) 0.467 0.474

1	(0.130) 0.128 0.132

Locus 5687 :

2 alleles

93.0% missing data

3	(0.900) 0.901 0.910

4	(0.100) 0.099 0.090

Locus 5688 :

2 alleles

99.2% missing data

1	(0.303) 0.307 0.299

3	(0.697) 0.693 0.701

Locus 5689 :

2 alleles

99.2% missing data

3	(0.330) 0.327 0.334

1	(0.670) 0.673 0.666

Locus 5690 :

3 alleles

83.2% missing data

2	(0.475) 0.565 0.395

3	(0.452) 0.375 0.533

4	(0.072) 0.060 0.071

Locus 5691 :

2 alleles

99.2% missing data

3	(0.567) 0.568 0.568

4	(0.433) 0.432 0.432

Locus 5692 :

2 alleles

98.8% missing data

3	(0.780) 0.780 0.784

4	(0.220) 0.220 0.216

Locus 5693 :

2 alleles

99.2% missing data

2	(0.416) 0.417 0.410

3	(0.584) 0.583 0.590

Locus 5694 :

2 alleles

99.2% missing data

1	(0.576) 0.577 0.578

4	(0.424) 0.423 0.422

Locus 5695 :

2 alleles

99.2% missing data

1	(0.286) 0.285 0.281

4	(0.714) 0.715 0.719

Locus 5696 :

2 alleles

99.2% missing data

4	(0.672) 0.668 0.669

1	(0.328) 0.332 0.331

Locus 5697 :

2 alleles

99.2% missing data

1	(0.695) 0.703 0.697

2	(0.305) 0.297 0.303

Locus 5698 :

2 alleles

99.2% missing data

3	(0.767) 0.771 0.764

2	(0.233) 0.229 0.236

Locus 5699 :

2 alleles

96.9% missing data

3	(0.315) 0.301 0.315

4	(0.685) 0.699 0.685

Locus 5700 :

2 alleles

98.8% missing data

2	(0.640) 0.629 0.648

4	(0.360) 0.371 0.352

Locus 5701 :

2 alleles

99.2% missing data

2	(0.429) 0.425 0.430

4	(0.571) 0.575 0.570

Locus 5702 :

2 alleles

99.2% missing data

2	(0.290) 0.287 0.291

1	(0.710) 0.713 0.709

Locus 5703 :

3 alleles

98.0% missing data

1	(0.255) 0.248 0.261

3	(0.577) 0.573 0.579

4	(0.168) 0.179 0.160

Locus 5704 :

2 alleles

99.2% missing data

3	(0.617) 0.621 0.616

1	(0.383) 0.379 0.384

Locus 5705 :

2 alleles

99.2% missing data

1	(0.505) 0.508 0.505

3	(0.495) 0.492 0.495

Locus 5706 :

2 alleles

98.8% missing data

4	(0.573) 0.571 0.580

3	(0.427) 0.429 0.420

Locus 5707 :

2 alleles

99.2% missing data

3	(0.887) 0.893 0.879

1	(0.113) 0.107 0.121

Locus 5708 :

2 alleles

98.0% missing data

2	(0.811) 0.807 0.813

3	(0.189) 0.193 0.187

Locus 5709 :

2 alleles

99.2% missing data

1	(0.278) 0.279 0.283

4	(0.722) 0.721 0.717

Locus 5710 :

2 alleles

99.2% missing data

1	(0.827) 0.825 0.824

4	(0.173) 0.175 0.176

Locus 5711 :

2 alleles

8.6% missing data

1	(0.984) 0.976 0.994

4	(0.016) 0.024 0.006

Locus 5712 :

2 alleles

98.8% missing data

1	(0.692) 0.692 0.693

4	(0.308) 0.308 0.307

Locus 5713 :

2 alleles

99.2% missing data

3	(0.261) 0.256 0.266

1	(0.739) 0.744 0.734

Locus 5714 :

2 alleles

99.2% missing data

1	(0.343) 0.347 0.347

2	(0.657) 0.653 0.653

Locus 5715 :

2 alleles

96.1% missing data

3	(0.867) 0.878 0.857

4	(0.133) 0.122 0.143

Locus 5716 :

2 alleles

99.2% missing data

2	(0.502) 0.500 0.506

1	(0.498) 0.500 0.494

Locus 5717 :

2 alleles

98.4% missing data

4	(0.430) 0.448 0.424

3	(0.570) 0.552 0.576

Locus 5718 :

3 alleles

98.8% missing data

1	(0.601) 0.594 0.598

4	(0.227) 0.236 0.223

3	(0.172) 0.171 0.179

Locus 5719 :

3 alleles

96.1% missing data

1	(0.081) 0.074 0.088

3	(0.722) 0.742 0.709

4	(0.197) 0.184 0.203

Locus 5720 :

3 alleles

98.4% missing data

1	(0.524) 0.527 0.529

4	(0.292) 0.294 0.282

2	(0.183) 0.179 0.190

Locus 5721 :

2 alleles

99.2% missing data

4	(0.575) 0.574 0.581

2	(0.425) 0.426 0.419

Locus 5722 :

3 alleles

98.8% missing data

3	(0.356) 0.354 0.354

4	(0.354) 0.353 0.357

1	(0.290) 0.293 0.289

Locus 5723 :

2 alleles

16.4% missing data

2	(0.987) 0.977 0.996

1	(0.013) 0.023 0.004

Locus 5724 :

2 alleles

99.2% missing data

1	(0.410) 0.414 0.410

3	(0.590) 0.586 0.590

Locus 5725 :

2 alleles

99.2% missing data

3	(0.519) 0.521 0.518

4	(0.481) 0.479 0.482

Locus 5726 :

2 alleles

99.2% missing data

4	(0.348) 0.346 0.344

2	(0.652) 0.654 0.656

Locus 5727 :

2 alleles

98.8% missing data

2	(0.476) 0.469 0.481

1	(0.524) 0.531 0.519

Locus 5728 :

2 alleles

98.0% missing data

3	(0.327) 0.330 0.316

1	(0.673) 0.670 0.684

Locus 5729 :

2 alleles

98.4% missing data

1	(0.759) 0.761 0.749

2	(0.241) 0.239 0.251

Locus 5730 :

2 alleles

99.2% missing data

2	(0.200) 0.202 0.199

1	(0.800) 0.798 0.801

Locus 5731 :

2 alleles

98.8% missing data

1	(0.630) 0.632 0.632

4	(0.370) 0.368 0.368

Locus 5732 :

2 alleles

96.9% missing data

1	(0.591) 0.605 0.574

4	(0.409) 0.395 0.426

Locus 5733 :

2 alleles

99.2% missing data

4	(0.719) 0.721 0.716

3	(0.281) 0.279 0.284

Locus 5734 :

2 alleles

95.3% missing data

3	(0.723) 0.738 0.716

1	(0.277) 0.262 0.284

Locus 5735 :

2 alleles

99.2% missing data

3	(0.559) 0.553 0.561

4	(0.441) 0.447 0.439

Locus 5736 :

2 alleles

4.3% missing data

1	(0.984) 0.981 0.995

4	(0.016) 0.019 0.005

Locus 5737 :

3 alleles

98.0% missing data

4	(0.483) 0.485 0.492

3	(0.326) 0.321 0.318

2	(0.191) 0.193 0.189

Locus 5738 :

2 alleles

9.8% missing data

3	(0.987) 0.989 0.993

1	(0.013) 0.011 0.007

Locus 5739 :

2 alleles

98.0% missing data

1	(0.737) 0.744 0.734

2	(0.263) 0.256 0.266

Locus 5740 :

2 alleles

98.8% missing data

1	(0.337) 0.340 0.335

4	(0.663) 0.660 0.665

Locus 5741 :

2 alleles

98.4% missing data

2	(0.696) 0.702 0.688

3	(0.304) 0.298 0.312

Locus 5742 :

2 alleles

99.2% missing data

2	(0.653) 0.664 0.648

1	(0.347) 0.336 0.352

Locus 5743 :

2 alleles

99.2% missing data

1	(0.470) 0.464 0.466

3	(0.530) 0.536 0.534

Locus 5744 :

2 alleles

98.4% missing data

1	(0.255) 0.256 0.259

3	(0.745) 0.744 0.741

Locus 5745 :

2 alleles

99.2% missing data

3	(0.758) 0.750 0.760

1	(0.242) 0.250 0.240

Locus 5746 :

3 alleles

98.8% missing data

2	(0.405) 0.405 0.404

1	(0.332) 0.331 0.337

3	(0.264) 0.265 0.259

Locus 5747 :

2 alleles

99.2% missing data

1	(0.376) 0.370 0.382

2	(0.624) 0.630 0.618

Locus 5748 :

2 alleles

98.8% missing data

2	(0.592) 0.599 0.585

1	(0.408) 0.401 0.415

Locus 5749 :

2 alleles

98.8% missing data

2	(0.590) 0.580 0.594

3	(0.410) 0.420 0.406

Locus 5750 :

2 alleles

99.2% missing data

1	(0.315) 0.313 0.312

2	(0.685) 0.687 0.688

Locus 5751 :

2 alleles

8.2% missing data

4	(0.986) 0.978 0.996

1	(0.014) 0.022 0.004

Locus 5752 :

2 alleles

99.2% missing data

1	(0.465) 0.463 0.461

4	(0.535) 0.537 0.539

Locus 5753 :

2 alleles

94.9% missing data

1	(0.309) 0.327 0.283

3	(0.691) 0.673 0.717

Locus 5754 :

2 alleles

99.2% missing data

3	(0.320) 0.310 0.322

1	(0.680) 0.690 0.678

Locus 5755 :

2 alleles

97.3% missing data

2	(0.292) 0.297 0.290

3	(0.708) 0.703 0.710

Locus 5756 :

2 alleles

97.7% missing data

1	(0.585) 0.580 0.598

3	(0.415) 0.420 0.402

Locus 5757 :

2 alleles

98.8% missing data

3	(0.558) 0.557 0.557

2	(0.442) 0.443 0.443

Locus 5758 :

2 alleles

99.2% missing data

2	(0.406) 0.403 0.408

4	(0.594) 0.597 0.592

Locus 5759 :

2 alleles

99.2% missing data

3	(0.321) 0.320 0.320

2	(0.679) 0.680 0.680

Locus 5760 :

2 alleles

99.2% missing data

4	(0.442) 0.437 0.447

3	(0.558) 0.563 0.553

Locus 5761 :

2 alleles

98.8% missing data

1	(0.469) 0.463 0.471

2	(0.531) 0.537 0.529

Locus 5762 :

3 alleles

98.8% missing data

4	(0.309) 0.311 0.302

3	(0.384) 0.385 0.390

2	(0.308) 0.304 0.309

Locus 5763 :

2 alleles

99.2% missing data

4	(0.289) 0.279 0.290

1	(0.711) 0.721 0.710

Locus 5764 :

2 alleles

99.2% missing data

4	(0.689) 0.696 0.683

2	(0.311) 0.304 0.317

Locus 5765 :

2 alleles

98.8% missing data

3	(0.645) 0.637 0.647

4	(0.355) 0.363 0.353

Locus 5766 :

2 alleles

98.4% missing data

3	(0.543) 0.549 0.544

1	(0.457) 0.451 0.456

Locus 5767 :

2 alleles

91.4% missing data

1	(0.917) 0.923 0.920

3	(0.083) 0.077 0.080

Locus 5768 :

2 alleles

99.2% missing data

1	(0.678) 0.686 0.672

3	(0.322) 0.314 0.328

Locus 5769 :

2 alleles

99.2% missing data

4	(0.600) 0.599 0.604

3	(0.400) 0.401 0.396

Locus 5770 :

2 alleles

99.2% missing data

3	(0.191) 0.190 0.199

1	(0.809) 0.810 0.801

Locus 5771 :

3 alleles

98.8% missing data

4	(0.248) 0.241 0.261

1	(0.576) 0.585 0.561

3	(0.176) 0.174 0.178

Locus 5772 :

2 alleles

99.2% missing data

1	(0.294) 0.288 0.296

3	(0.706) 0.712 0.704

Locus 5773 :

2 alleles

99.2% missing data

2	(0.507) 0.500 0.507

1	(0.493) 0.500 0.493

Locus 5774 :

2 alleles

99.2% missing data

1	(0.346) 0.344 0.347

2	(0.654) 0.656 0.653

Locus 5775 :

2 alleles

98.4% missing data

1	(0.681) 0.683 0.691

3	(0.319) 0.317 0.309

Locus 5776 :

2 alleles

99.2% missing data

2	(0.434) 0.436 0.435

3	(0.566) 0.564 0.565

Locus 5777 :

2 alleles

99.2% missing data

4	(0.552) 0.546 0.565

2	(0.448) 0.454 0.435

Locus 5778 :

2 alleles

99.2% missing data

1	(0.675) 0.664 0.684

3	(0.325) 0.336 0.316

Locus 5779 :

2 alleles

98.8% missing data

2	(0.472) 0.467 0.478

4	(0.528) 0.533 0.522

Locus 5780 :

2 alleles

99.2% missing data

1	(0.537) 0.533 0.542

4	(0.463) 0.467 0.458

Locus 5781 :

2 alleles

99.2% missing data

3	(0.562) 0.561 0.562

2	(0.438) 0.439 0.438

Locus 5782 :

2 alleles

96.5% missing data

3	(0.376) 0.383 0.384

4	(0.624) 0.617 0.616

Locus 5783 :

3 alleles

92.6% missing data

3	(0.865) 0.855 0.882

2	(0.067) 0.068 0.064

4	(0.067) 0.077 0.054

Locus 5784 :

2 alleles

98.8% missing data

2	(0.673) 0.665 0.678

3	(0.327) 0.335 0.322

Locus 5785 :

4 alleles

98.4% missing data

1	(0.172) 0.174 0.171

2	(0.250) 0.250 0.255

3	(0.243) 0.240 0.236

4	(0.335) 0.337 0.337

Locus 5786 :

2 alleles

99.2% missing data

1	(0.772) 0.778 0.769

2	(0.228) 0.222 0.231

Locus 5787 :

3 alleles

94.5% missing data

1	(0.848) 0.852 0.853

2	(0.084) 0.082 0.083

3	(0.068) 0.066 0.064

Locus 5788 :

2 alleles

98.4% missing data

1	(0.673) 0.668 0.673

2	(0.327) 0.332 0.327

Locus 5789 :

2 alleles

99.2% missing data

4	(0.770) 0.766 0.770

1	(0.230) 0.234 0.230

Locus 5790 :

2 alleles

99.2% missing data

3	(0.517) 0.513 0.521

4	(0.483) 0.487 0.479

Locus 5791 :

2 alleles

90.6% missing data

4	(0.841) 0.870 0.824

1	(0.159) 0.130 0.176

Locus 5792 :

2 alleles

98.4% missing data

3	(0.534) 0.536 0.537

4	(0.466) 0.464 0.463

Locus 5793 :

2 alleles

96.9% missing data

3	(0.206) 0.200 0.204

4	(0.794) 0.800 0.796

Locus 5794 :

2 alleles

99.2% missing data

1	(0.385) 0.389 0.386

2	(0.615) 0.611 0.614

Locus 5795 :

2 alleles

99.2% missing data

4	(0.373) 0.372 0.376

1	(0.627) 0.628 0.624

Locus 5796 :

2 alleles

98.8% missing data

1	(0.282) 0.283 0.283

3	(0.718) 0.717 0.717

Locus 5797 :

2 alleles

99.2% missing data

3	(0.621) 0.625 0.619

2	(0.379) 0.375 0.381

Locus 5798 :

3 alleles

98.8% missing data

2	(0.439) 0.444 0.444

1	(0.290) 0.283 0.292

3	(0.271) 0.273 0.264

Locus 5799 :

2 alleles

91.0% missing data

3	(0.904) 0.909 0.897

2	(0.096) 0.091 0.103

Locus 5800 :

2 alleles

99.2% missing data

1	(0.496) 0.488 0.500

4	(0.504) 0.512 0.500

Locus 5801 :

3 alleles

98.8% missing data

3	(0.138) 0.146 0.135

2	(0.481) 0.471 0.487

1	(0.381) 0.383 0.378

Locus 5802 :

2 alleles

96.1% missing data

4	(0.156) 0.146 0.160

3	(0.844) 0.854 0.840

Locus 5803 :

2 alleles

97.7% missing data

2	(0.705) 0.701 0.724

1	(0.295) 0.299 0.276

Locus 5804 :

2 alleles

98.0% missing data

1	(0.218) 0.222 0.217

3	(0.782) 0.778 0.783

Locus 5805 :

2 alleles

99.2% missing data

1	(0.498) 0.489 0.503

4	(0.502) 0.511 0.497

Locus 5806 :

2 alleles

97.7% missing data

2	(0.172) 0.168 0.174

1	(0.828) 0.832 0.826

Locus 5807 :

2 alleles

99.2% missing data

4	(0.473) 0.468 0.479

3	(0.527) 0.532 0.521

Locus 5808 :

3 alleles

96.9% missing data

1	(0.182) 0.187 0.175

2	(0.324) 0.333 0.308

4	(0.494) 0.480 0.517

Locus 5809 :

2 alleles

98.8% missing data

1	(0.595) 0.599 0.596

3	(0.405) 0.401 0.404

Locus 5810 :

2 alleles

99.2% missing data

3	(0.314) 0.316 0.311

2	(0.686) 0.684 0.689

Locus 5811 :

2 alleles

99.2% missing data

4	(0.175) 0.177 0.171

3	(0.825) 0.823 0.829

Locus 5812 :

2 alleles

99.2% missing data

3	(0.519) 0.518 0.519

2	(0.481) 0.482 0.481

Locus 5813 :

2 alleles

99.2% missing data

2	(0.586) 0.578 0.591

1	(0.414) 0.422 0.409

Locus 5814 :

4 alleles

94.9% missing data

1	(0.142) 0.139 0.139

3	(0.576) 0.603 0.564

4	(0.173) 0.161 0.184

2	(0.109) 0.098 0.112

Locus 5815 :

2 alleles

99.2% missing data

4	(0.562) 0.558 0.567

3	(0.438) 0.442 0.433

Locus 5816 :

2 alleles

96.9% missing data

3	(0.850) 0.858 0.845

4	(0.150) 0.142 0.155

Locus 5817 :

2 alleles

99.2% missing data

2	(0.725) 0.721 0.728

3	(0.275) 0.279 0.272

Locus 5818 :

2 alleles

99.2% missing data

2	(0.507) 0.507 0.505

3	(0.493) 0.493 0.495

Locus 5819 :

2 alleles

99.2% missing data

4	(0.287) 0.293 0.283

2	(0.713) 0.707 0.717

Locus 5820 :

2 alleles

85.2% missing data

2	(0.625) 0.695 0.537

1	(0.375) 0.305 0.463

Locus 5821 :

2 alleles

94.1% missing data

3	(0.521) 0.551 0.505

2	(0.479) 0.449 0.495

Locus 5822 :

2 alleles

99.2% missing data

3	(0.716) 0.716 0.708

4	(0.284) 0.284 0.292

Locus 5823 :

2 alleles

99.2% missing data

1	(0.514) 0.504 0.512

4	(0.486) 0.496 0.488

Locus 5824 :

2 alleles

98.8% missing data

1	(0.499) 0.499 0.499

3	(0.501) 0.501 0.501

Locus 5825 :

2 alleles

99.2% missing data

1	(0.467) 0.465 0.472

3	(0.533) 0.535 0.528

Locus 5826 :

2 alleles

98.8% missing data

1	(0.610) 0.611 0.611

2	(0.390) 0.389 0.389

Locus 5827 :

2 alleles

99.2% missing data

3	(0.476) 0.475 0.475

4	(0.524) 0.525 0.525

Locus 5828 :

2 alleles

99.2% missing data

2	(0.610) 0.612 0.606

1	(0.390) 0.388 0.394

Locus 5829 :

3 alleles

98.0% missing data

1	(0.181) 0.195 0.175

3	(0.311) 0.313 0.319

2	(0.507) 0.492 0.506

Locus 5830 :

3 alleles

98.0% missing data

4	(0.368) 0.370 0.382

3	(0.512) 0.511 0.496

2	(0.119) 0.119 0.122

Locus 5831 :

2 alleles

99.2% missing data

3	(0.542) 0.538 0.546

2	(0.458) 0.462 0.454

Locus 5832 :

2 alleles

96.5% missing data

2	(0.119) 0.111 0.126

3	(0.881) 0.889 0.874

Locus 5833 :

2 alleles

99.2% missing data

3	(0.594) 0.594 0.596

1	(0.406) 0.406 0.404

Locus 5834 :

2 alleles

97.3% missing data

4	(0.765) 0.772 0.754

1	(0.235) 0.228 0.246

Locus 5835 :

3 alleles

98.8% missing data

4	(0.399) 0.395 0.402

3	(0.252) 0.247 0.254

2	(0.348) 0.358 0.344

Locus 5836 :

2 alleles

98.4% missing data

4	(0.220) 0.218 0.222

3	(0.780) 0.782 0.778

Locus 5837 :

2 alleles

99.2% missing data

1	(0.259) 0.261 0.255

2	(0.741) 0.739 0.745

Locus 5838 :

2 alleles

97.7% missing data

3	(0.225) 0.224 0.216

1	(0.775) 0.776 0.784

Locus 5839 :

2 alleles

99.2% missing data

3	(0.397) 0.394 0.403

2	(0.603) 0.606 0.597

Locus 5840 :

2 alleles

99.2% missing data

1	(0.680) 0.685 0.671

4	(0.320) 0.315 0.329

Locus 5841 :

2 alleles

99.2% missing data

1	(0.649) 0.650 0.656

4	(0.351) 0.350 0.344

Locus 5842 :

2 alleles

99.2% missing data

4	(0.270) 0.263 0.274

3	(0.730) 0.737 0.726

Locus 5843 :

2 alleles

99.2% missing data

3	(0.225) 0.229 0.225

1	(0.775) 0.771 0.775

Locus 5844 :

2 alleles

99.2% missing data

3	(0.281) 0.279 0.285

4	(0.719) 0.721 0.715

Locus 5845 :

2 alleles

99.2% missing data

3	(0.310) 0.312 0.309

1	(0.690) 0.688 0.691

Locus 5846 :

2 alleles

99.2% missing data

3	(0.686) 0.689 0.686

4	(0.314) 0.311 0.314

Locus 5847 :

2 alleles

99.2% missing data

1	(0.362) 0.359 0.361

3	(0.638) 0.641 0.639

Locus 5848 :

2 alleles

99.2% missing data

2	(0.681) 0.680 0.680

1	(0.319) 0.320 0.320

Locus 5849 :

2 alleles

98.4% missing data

1	(0.668) 0.670 0.667

2	(0.332) 0.330 0.333

Locus 5850 :

3 alleles

98.8% missing data

2	(0.296) 0.291 0.298

3	(0.300) 0.306 0.299

4	(0.403) 0.403 0.403

Locus 5851 :

3 alleles

98.8% missing data

4	(0.413) 0.419 0.417

1	(0.433) 0.430 0.426

2	(0.154) 0.151 0.157

Locus 5852 :

2 alleles

99.2% missing data

3	(0.526) 0.530 0.522

2	(0.474) 0.470 0.478

Locus 5853 :

2 alleles

99.2% missing data

1	(0.608) 0.609 0.608

3	(0.392) 0.391 0.392

Locus 5854 :

2 alleles

98.0% missing data

4	(0.695) 0.698 0.698

2	(0.305) 0.302 0.302

Locus 5855 :

2 alleles

96.9% missing data

1	(0.464) 0.466 0.450

3	(0.536) 0.534 0.550

Locus 5856 :

2 alleles

99.2% missing data

2	(0.704) 0.701 0.705

3	(0.296) 0.299 0.295

Locus 5857 :

2 alleles

99.2% missing data

1	(0.493) 0.487 0.500

3	(0.507) 0.513 0.500

Locus 5858 :

2 alleles

98.8% missing data

1	(0.637) 0.629 0.642

3	(0.363) 0.371 0.358

Locus 5859 :

2 alleles

98.0% missing data

2	(0.656) 0.652 0.666

4	(0.344) 0.348 0.334

Locus 5860 :

2 alleles

99.2% missing data

3	(0.397) 0.399 0.402

1	(0.603) 0.601 0.598

Locus 5861 :

2 alleles

98.8% missing data

2	(0.451) 0.442 0.456

3	(0.549) 0.558 0.544

Locus 5862 :

2 alleles

96.5% missing data

3	(0.812) 0.803 0.821

1	(0.188) 0.197 0.179

Locus 5863 :

2 alleles

99.2% missing data

4	(0.593) 0.590 0.597

3	(0.407) 0.410 0.403

Locus 5864 :

2 alleles

99.2% missing data

1	(0.788) 0.792 0.783

3	(0.212) 0.208 0.217

Locus 5865 :

2 alleles

99.2% missing data

2	(0.438) 0.444 0.433

1	(0.562) 0.556 0.567

Locus 5866 :

2 alleles

99.2% missing data

2	(0.455) 0.459 0.450

3	(0.545) 0.541 0.550

Locus 5867 :

2 alleles

96.5% missing data

2	(0.739) 0.748 0.739

3	(0.261) 0.252 0.261

Locus 5868 :

2 alleles

96.9% missing data

1	(0.741) 0.727 0.756

4	(0.259) 0.273 0.244

Locus 5869 :

2 alleles

0.4% missing data

4	(0.985) 0.980 0.995

1	(0.015) 0.020 0.005

Locus 5870 :

2 alleles

99.2% missing data

1	(0.753) 0.746 0.757

2	(0.247) 0.254 0.243

Locus 5871 :

2 alleles

88.3% missing data

3	(0.662) 0.722 0.613

1	(0.338) 0.278 0.387

Locus 5872 :

2 alleles

97.3% missing data

1	(0.266) 0.265 0.255

3	(0.734) 0.735 0.745

Locus 5873 :

2 alleles

91.8% missing data

2	(0.869) 0.884 0.867

1	(0.131) 0.116 0.133

Locus 5874 :

2 alleles

99.2% missing data

3	(0.632) 0.641 0.627

2	(0.368) 0.359 0.373

Locus 5875 :

2 alleles

7.8% missing data

1	(0.988) 0.981 0.996

3	(0.012) 0.019 0.004

Locus 5876 :

2 alleles

96.5% missing data

1	(0.851) 0.850 0.857

3	(0.149) 0.150 0.143

Locus 5877 :

3 alleles

98.0% missing data

1	(0.288) 0.286 0.292

2	(0.355) 0.355 0.349

3	(0.357) 0.359 0.359

Locus 5878 :

2 alleles

99.2% missing data

3	(0.571) 0.577 0.571

4	(0.429) 0.423 0.429

Locus 5879 :

2 alleles

99.2% missing data

1	(0.272) 0.271 0.275

4	(0.728) 0.729 0.725

Locus 5880 :

2 alleles

99.2% missing data

1	(0.841) 0.844 0.834

2	(0.159) 0.156 0.166

Locus 5881 :

2 alleles

98.4% missing data

1	(0.734) 0.730 0.743

2	(0.266) 0.270 0.257

Locus 5882 :

2 alleles

99.2% missing data

3	(0.520) 0.519 0.528

1	(0.480) 0.481 0.472

Locus 5883 :

2 alleles

99.2% missing data

1	(0.510) 0.506 0.518

2	(0.490) 0.494 0.482

Locus 5884 :

2 alleles

99.2% missing data

4	(0.663) 0.650 0.673

2	(0.337) 0.350 0.327

Locus 5885 :

2 alleles

99.2% missing data

1	(0.529) 0.528 0.531

2	(0.471) 0.472 0.469

Locus 5886 :

2 alleles

98.4% missing data

3	(0.559) 0.570 0.549

1	(0.441) 0.430 0.451

Locus 5887 :

2 alleles

99.2% missing data

2	(0.329) 0.325 0.336

1	(0.671) 0.675 0.664

Locus 5888 :

2 alleles

99.2% missing data

3	(0.505) 0.505 0.514

2	(0.495) 0.495 0.486

Locus 5889 :

2 alleles

99.2% missing data

1	(0.483) 0.479 0.485

3	(0.517) 0.521 0.515

Locus 5890 :

2 alleles

99.2% missing data

2	(0.653) 0.654 0.649

4	(0.347) 0.346 0.351

Locus 5891 :

2 alleles

99.2% missing data

1	(0.494) 0.489 0.496

2	(0.506) 0.511 0.504

Locus 5892 :

3 alleles

98.8% missing data

4	(0.511) 0.505 0.512

2	(0.213) 0.214 0.217

1	(0.276) 0.280 0.271

Locus 5893 :

2 alleles

99.2% missing data

3	(0.459) 0.467 0.459

1	(0.541) 0.533 0.541

Locus 5894 :

2 alleles

99.2% missing data

1	(0.450) 0.446 0.455

2	(0.550) 0.554 0.545

Locus 5895 :

2 alleles

99.2% missing data

1	(0.187) 0.185 0.190

3	(0.813) 0.815 0.810

Locus 5896 :

2 alleles

99.2% missing data

1	(0.096) 0.101 0.091

2	(0.904) 0.899 0.909

Locus 5897 :

2 alleles

99.2% missing data

4	(0.689) 0.688 0.686

2	(0.311) 0.312 0.314

Locus 5898 :

2 alleles

99.2% missing data

4	(0.381) 0.384 0.372

3	(0.619) 0.616 0.628

Locus 5899 :

2 alleles

99.2% missing data

3	(0.496) 0.494 0.505

2	(0.504) 0.506 0.495

Locus 5900 :

2 alleles

99.2% missing data

3	(0.681) 0.684 0.678

1	(0.319) 0.316 0.322

Locus 5901 :

2 alleles

96.1% missing data

1	(0.850) 0.856 0.848

4	(0.150) 0.144 0.152

Locus 5902 :

2 alleles

99.2% missing data

1	(0.901) 0.902 0.895

2	(0.099) 0.098 0.105

Locus 5903 :

2 alleles

96.9% missing data

4	(0.180) 0.176 0.181

2	(0.820) 0.824 0.819

Locus 5904 :

2 alleles

99.2% missing data

1	(0.571) 0.571 0.572

4	(0.429) 0.429 0.428

Locus 5905 :

2 alleles

99.2% missing data

2	(0.537) 0.537 0.536

3	(0.463) 0.463 0.464

Locus 5906 :

2 alleles

98.8% missing data

3	(0.413) 0.419 0.413

1	(0.587) 0.581 0.587

Locus 5907 :

2 alleles

98.8% missing data

1	(0.403) 0.407 0.403

2	(0.597) 0.593 0.597

Locus 5908 :

2 alleles

99.2% missing data

2	(0.646) 0.647 0.645

1	(0.354) 0.353 0.355

Locus 5909 :

2 alleles

99.2% missing data

2	(0.472) 0.476 0.473

4	(0.528) 0.524 0.527

Locus 5910 :

2 alleles

99.2% missing data

3	(0.472) 0.474 0.476

2	(0.528) 0.526 0.524

Locus 5911 :

2 alleles

99.2% missing data

4	(0.580) 0.583 0.581

3	(0.420) 0.417 0.419

Locus 5912 :

2 alleles

99.2% missing data

4	(0.321) 0.326 0.320

3	(0.679) 0.674 0.680

Locus 5913 :

2 alleles

96.9% missing data

1	(0.182) 0.187 0.173

2	(0.818) 0.813 0.827

Locus 5914 :

2 alleles

99.2% missing data

2	(0.450) 0.450 0.456

4	(0.550) 0.550 0.544

Locus 5915 :

3 alleles

98.8% missing data

4	(0.370) 0.367 0.373

2	(0.254) 0.264 0.254

1	(0.376) 0.369 0.373

Locus 5916 :

2 alleles

99.2% missing data

3	(0.645) 0.644 0.649

4	(0.355) 0.356 0.351

Locus 5917 :

2 alleles

99.2% missing data

3	(0.466) 0.461 0.470

4	(0.534) 0.539 0.530

Locus 5918 :

2 alleles

99.2% missing data

4	(0.507) 0.508 0.512

1	(0.493) 0.492 0.488

Locus 5919 :

2 alleles

99.2% missing data

3	(0.227) 0.227 0.230

1	(0.773) 0.773 0.770

Locus 5920 :

2 alleles

99.2% missing data

3	(0.531) 0.536 0.533

2	(0.469) 0.464 0.467

Locus 5921 :

2 alleles

98.8% missing data

2	(0.553) 0.552 0.555

3	(0.447) 0.448 0.445

Locus 5922 :

2 alleles

99.2% missing data

4	(0.361) 0.362 0.362

3	(0.639) 0.638 0.638

Locus 5923 :

2 alleles

99.2% missing data

1	(0.287) 0.287 0.282

4	(0.713) 0.713 0.718

Locus 5924 :

2 alleles

98.8% missing data

2	(0.729) 0.734 0.728

3	(0.271) 0.266 0.272

Locus 5925 :

2 alleles

99.2% missing data

1	(0.656) 0.654 0.655

2	(0.344) 0.346 0.345

Locus 5926 :

3 alleles

98.8% missing data

1	(0.263) 0.261 0.261

4	(0.490) 0.487 0.494

2	(0.247) 0.252 0.245

Locus 5927 :

2 alleles

99.2% missing data

3	(0.506) 0.502 0.510

2	(0.494) 0.498 0.490

Locus 5928 :

2 alleles

99.2% missing data

4	(0.704) 0.710 0.696

3	(0.296) 0.290 0.304

Locus 5929 :

2 alleles

99.2% missing data

4	(0.393) 0.392 0.393

3	(0.607) 0.608 0.607

Locus 5930 :

2 alleles

8.6% missing data

4	(0.987) 0.982 0.996

1	(0.013) 0.018 0.004

Locus 5931 :

2 alleles

98.4% missing data

1	(0.403) 0.413 0.399

4	(0.597) 0.587 0.601

Locus 5932 :

2 alleles

99.2% missing data

3	(0.338) 0.338 0.340

4	(0.662) 0.662 0.660

Locus 5933 :

2 alleles

99.2% missing data

2	(0.463) 0.465 0.467

3	(0.537) 0.535 0.533

Locus 5934 :

2 alleles

99.2% missing data

3	(0.430) 0.430 0.430

2	(0.570) 0.570 0.570

Locus 5935 :

2 alleles

96.5% missing data

2	(0.095) 0.093 0.100

1	(0.905) 0.907 0.900

Locus 5936 :

2 alleles

99.2% missing data

2	(0.556) 0.556 0.561

1	(0.444) 0.444 0.439

Locus 5937 :

2 alleles

99.2% missing data

4	(0.650) 0.646 0.653

1	(0.350) 0.354 0.347

Locus 5938 :

2 alleles

99.2% missing data

1	(0.185) 0.176 0.188

2	(0.815) 0.824 0.812

Locus 5939 :

2 alleles

99.2% missing data

2	(0.514) 0.512 0.516

3	(0.486) 0.488 0.484

Locus 5940 :

2 alleles

99.2% missing data

4	(0.717) 0.712 0.716

3	(0.283) 0.288 0.284

Locus 5941 :

2 alleles

99.2% missing data

3	(0.216) 0.219 0.216

4	(0.784) 0.781 0.784

Locus 5942 :

2 alleles

91.0% missing data

2	(0.862) 0.883 0.853

4	(0.138) 0.117 0.147

Locus 5943 :

2 alleles

99.2% missing data

2	(0.158) 0.155 0.161

3	(0.842) 0.845 0.839

Locus 5944 :

2 alleles

98.8% missing data

3	(0.523) 0.522 0.516

2	(0.477) 0.478 0.484

Locus 5945 :

2 alleles

99.2% missing data

3	(0.773) 0.768 0.773

4	(0.227) 0.232 0.227

Locus 5946 :

2 alleles

99.2% missing data

3	(0.162) 0.162 0.166

4	(0.838) 0.838 0.834

Locus 5947 :

2 alleles

99.2% missing data

1	(0.602) 0.593 0.607

3	(0.398) 0.407 0.393

Locus 5948 :

2 alleles

99.2% missing data

3	(0.417) 0.415 0.418

4	(0.583) 0.585 0.582

Locus 5949 :

2 alleles

96.1% missing data

3	(0.763) 0.777 0.753

1	(0.237) 0.223 0.247

Locus 5950 :

2 alleles

99.2% missing data

2	(0.297) 0.301 0.299

1	(0.703) 0.699 0.701

Locus 5951 :

2 alleles

99.2% missing data

4	(0.376) 0.373 0.384

3	(0.624) 0.627 0.616

Locus 5952 :

2 alleles

98.8% missing data

2	(0.618) 0.615 0.619

1	(0.382) 0.385 0.381

Locus 5953 :

3 alleles

97.7% missing data

2	(0.370) 0.357 0.369

3	(0.308) 0.309 0.308

1	(0.323) 0.334 0.323

Locus 5954 :

2 alleles

99.2% missing data

4	(0.347) 0.343 0.349

3	(0.653) 0.657 0.651

Locus 5955 :

2 alleles

99.2% missing data

1	(0.622) 0.625 0.618

3	(0.378) 0.375 0.382

Locus 5956 :

2 alleles

99.2% missing data

3	(0.596) 0.599 0.592

2	(0.404) 0.401 0.408

Locus 5957 :

2 alleles

98.0% missing data

3	(0.103) 0.102 0.102

2	(0.897) 0.898 0.898

Locus 5958 :

2 alleles

99.2% missing data

2	(0.207) 0.204 0.213

3	(0.793) 0.796 0.787

Locus 5959 :

2 alleles

99.2% missing data

2	(0.509) 0.508 0.511

4	(0.491) 0.492 0.489

Locus 5960 :

2 alleles

91.0% missing data

4	(0.945) 0.958 0.938

3	(0.055) 0.042 0.062

Locus 5961 :

2 alleles

99.2% missing data

2	(0.143) 0.147 0.139

3	(0.857) 0.853 0.861

Locus 5962 :

2 alleles

99.2% missing data

3	(0.552) 0.543 0.555

1	(0.448) 0.457 0.445

Locus 5963 :

2 alleles

98.8% missing data

1	(0.573) 0.576 0.578

3	(0.427) 0.424 0.422

Locus 5964 :

2 alleles

98.4% missing data

2	(0.606) 0.615 0.609

4	(0.394) 0.385 0.391

Locus 5965 :

2 alleles

89.5% missing data

4	(0.928) 0.934 0.933

2	(0.072) 0.066 0.067

Locus 5966 :

2 alleles

99.2% missing data

2	(0.610) 0.612 0.608

4	(0.390) 0.388 0.392

Locus 5967 :

2 alleles

99.2% missing data

1	(0.581) 0.580 0.582

2	(0.419) 0.420 0.418

Locus 5968 :

2 alleles

99.2% missing data

4	(0.747) 0.746 0.748

2	(0.253) 0.254 0.252

Locus 5969 :

2 alleles

91.0% missing data

1	(0.926) 0.940 0.919

2	(0.074) 0.060 0.081

Locus 5970 :

2 alleles

99.2% missing data

3	(0.571) 0.569 0.569

1	(0.429) 0.431 0.431

Locus 5971 :

2 alleles

95.3% missing data

3	(0.710) 0.730 0.695

2	(0.290) 0.270 0.305

Locus 5972 :

2 alleles

98.8% missing data

2	(0.407) 0.398 0.415

3	(0.593) 0.602 0.585

Locus 5973 :

2 alleles

98.8% missing data

1	(0.451) 0.451 0.446

2	(0.549) 0.549 0.554

Locus 5974 :

3 alleles

97.7% missing data

2	(0.580) 0.582 0.586

3	(0.208) 0.209 0.204

4	(0.211) 0.208 0.209

Locus 5975 :

2 alleles

99.2% missing data

1	(0.365) 0.364 0.369

2	(0.635) 0.636 0.631

Locus 5976 :

2 alleles

0.0% missing data

3	(0.987) 0.982 0.996

1	(0.013) 0.018 0.004

Locus 5977 :

2 alleles

96.9% missing data

3	(0.529) 0.531 0.528

1	(0.471) 0.469 0.472

Locus 5978 :

2 alleles

98.8% missing data

4	(0.742) 0.736 0.739

3	(0.258) 0.264 0.261

Locus 5979 :

2 alleles

99.2% missing data

3	(0.354) 0.356 0.353

4	(0.646) 0.644 0.647

Locus 5980 :

3 alleles

98.8% missing data

3	(0.275) 0.274 0.276

1	(0.268) 0.271 0.268

2	(0.457) 0.456 0.456

Locus 5981 :

2 alleles

99.2% missing data

1	(0.473) 0.473 0.465

3	(0.527) 0.527 0.535

Locus 5982 :

2 alleles

99.2% missing data

4	(0.656) 0.659 0.652

1	(0.344) 0.341 0.348

Locus 5983 :

2 alleles

99.2% missing data

4	(0.622) 0.616 0.627

1	(0.378) 0.384 0.373

Locus 5984 :

2 alleles

96.5% missing data

3	(0.723) 0.721 0.731

1	(0.277) 0.279 0.269

Locus 5985 :

2 alleles

98.0% missing data

2	(0.704) 0.698 0.713

3	(0.296) 0.302 0.287

Locus 5986 :

2 alleles

99.2% missing data

1	(0.276) 0.277 0.276

3	(0.724) 0.723 0.724

Locus 5987 :

3 alleles

98.4% missing data

3	(0.345) 0.340 0.346

2	(0.344) 0.345 0.346

1	(0.311) 0.315 0.308

Locus 5988 :

2 alleles

98.8% missing data

1	(0.304) 0.299 0.316

4	(0.696) 0.701 0.684

Locus 5989 :

2 alleles

97.7% missing data

3	(0.812) 0.814 0.816

2	(0.188) 0.186 0.184

Locus 5990 :

2 alleles

10.2% missing data

2	(0.983) 0.985 0.992

1	(0.017) 0.015 0.008

Locus 5991 :

2 alleles

99.2% missing data

2	(0.288) 0.287 0.293

4	(0.712) 0.713 0.707

Locus 5992 :

2 alleles

96.1% missing data

2	(0.897) 0.905 0.889

4	(0.103) 0.095 0.111

Locus 5993 :

2 alleles

95.7% missing data

2	(0.888) 0.899 0.884

1	(0.112) 0.101 0.116

Locus 5994 :

2 alleles

0.0% missing data

2	(0.985) 0.980 0.996

3	(0.015) 0.020 0.004

Locus 5995 :

2 alleles

96.1% missing data

3	(0.113) 0.112 0.109

2	(0.887) 0.888 0.891

Locus 5996 :

2 alleles

98.8% missing data

2	(0.801) 0.805 0.795

4	(0.199) 0.195 0.205

Locus 5997 :

2 alleles

88.3% missing data

3	(0.858) 0.887 0.814

1	(0.142) 0.113 0.186

Locus 5998 :

2 alleles

99.2% missing data

3	(0.398) 0.399 0.398

2	(0.602) 0.601 0.602

Locus 5999 :

2 alleles

97.3% missing data

2	(0.850) 0.845 0.859

3	(0.150) 0.155 0.141

Locus 6000 :

2 alleles

99.2% missing data

3	(0.427) 0.427 0.422

4	(0.573) 0.573 0.578

Locus 6001 :

2 alleles

99.2% missing data

3	(0.352) 0.352 0.358

2	(0.648) 0.648 0.642

Locus 6002 :

2 alleles

99.2% missing data

4	(0.313) 0.314 0.320

1	(0.687) 0.686 0.680

Locus 6003 :

2 alleles

99.2% missing data

3	(0.542) 0.543 0.540

2	(0.458) 0.457 0.460

Locus 6004 :

2 alleles

99.2% missing data

3	(0.524) 0.521 0.529

4	(0.476) 0.479 0.471

Locus 6005 :

2 alleles

99.2% missing data

1	(0.179) 0.184 0.175

3	(0.821) 0.816 0.825

Locus 6006 :

3 alleles

98.4% missing data

4	(0.271) 0.273 0.275

3	(0.423) 0.415 0.429

2	(0.306) 0.312 0.296

Locus 6007 :

2 alleles

99.2% missing data

3	(0.697) 0.695 0.699

4	(0.303) 0.305 0.301

Locus 6008 :

2 alleles

98.8% missing data

1	(0.757) 0.767 0.754

2	(0.243) 0.233 0.246

Locus 6009 :

2 alleles

0.0% missing data

2	(0.981) 0.985 0.985

3	(0.019) 0.015 0.015

Locus 6010 :

2 alleles

99.2% missing data

4	(0.464) 0.466 0.466

3	(0.536) 0.534 0.534

Locus 6011 :

2 alleles

98.0% missing data

3	(0.179) 0.175 0.183

1	(0.821) 0.825 0.817

Locus 6012 :

2 alleles

99.2% missing data

3	(0.717) 0.713 0.723

1	(0.283) 0.287 0.277

Locus 6013 :

3 alleles

98.8% missing data

2	(0.249) 0.242 0.256

3	(0.300) 0.313 0.289

1	(0.451) 0.445 0.455

Locus 6014 :

2 alleles

99.2% missing data

1	(0.671) 0.667 0.675

3	(0.329) 0.333 0.325

Locus 6015 :

2 alleles

99.2% missing data

2	(0.275) 0.277 0.268

3	(0.725) 0.723 0.732

Locus 6016 :

2 alleles

99.2% missing data

2	(0.487) 0.487 0.491

4	(0.513) 0.513 0.509

Locus 6017 :

2 alleles

99.2% missing data

1	(0.502) 0.506 0.497

3	(0.498) 0.494 0.503

Locus 6018 :

2 alleles

98.8% missing data

2	(0.285) 0.299 0.280

1	(0.715) 0.701 0.720

Locus 6019 :

3 alleles

98.8% missing data

2	(0.227) 0.238 0.222

4	(0.231) 0.224 0.239

1	(0.542) 0.538 0.539

Locus 6020 :

2 alleles

99.2% missing data

3	(0.519) 0.523 0.517

1	(0.481) 0.477 0.483

Locus 6021 :

2 alleles

98.8% missing data

4	(0.896) 0.889 0.904

1	(0.104) 0.111 0.096

Locus 6022 :

2 alleles

99.2% missing data

1	(0.534) 0.527 0.537

2	(0.466) 0.473 0.463

Locus 6023 :

2 alleles

99.2% missing data

4	(0.524) 0.517 0.526

2	(0.476) 0.483 0.474

Locus 6024 :

2 alleles

98.8% missing data

1	(0.661) 0.661 0.663

2	(0.339) 0.339 0.337

Locus 6025 :

2 alleles

99.2% missing data

2	(0.572) 0.581 0.568

4	(0.428) 0.419 0.432

Locus 6026 :

2 alleles

99.2% missing data

2	(0.546) 0.541 0.538

4	(0.454) 0.459 0.462

Locus 6027 :

2 alleles

99.2% missing data

4	(0.549) 0.547 0.555

1	(0.451) 0.453 0.445

Locus 6028 :

2 alleles

99.2% missing data

4	(0.647) 0.643 0.645

1	(0.353) 0.357 0.355

Locus 6029 :

2 alleles

99.2% missing data

2	(0.545) 0.538 0.545

1	(0.455) 0.462 0.455

Locus 6030 :

2 alleles

99.2% missing data

3	(0.485) 0.486 0.489

4	(0.515) 0.514 0.511

Locus 6031 :

2 alleles

99.2% missing data

2	(0.577) 0.572 0.582

4	(0.423) 0.428 0.418

Locus 6032 :

2 alleles

99.2% missing data

4	(0.212) 0.215 0.216

1	(0.788) 0.785 0.784

Locus 6033 :

2 alleles

99.2% missing data

1	(0.566) 0.569 0.571

2	(0.434) 0.431 0.429

Locus 6034 :

2 alleles

99.2% missing data

1	(0.214) 0.215 0.217

4	(0.786) 0.785 0.783

Locus 6035 :

2 alleles

99.2% missing data

2	(0.483) 0.479 0.490

3	(0.517) 0.521 0.510

Locus 6036 :

2 alleles

99.2% missing data

2	(0.465) 0.460 0.466

3	(0.535) 0.540 0.534

Locus 6037 :

2 alleles

99.2% missing data

3	(0.556) 0.555 0.558

2	(0.444) 0.445 0.442

Locus 6038 :

2 alleles

97.7% missing data

1	(0.564) 0.576 0.565

3	(0.436) 0.424 0.435

Locus 6039 :

2 alleles

98.0% missing data

1	(0.402) 0.399 0.402

3	(0.598) 0.601 0.598

Locus 6040 :

2 alleles

98.4% missing data

3	(0.513) 0.523 0.514

2	(0.487) 0.477 0.486

Locus 6041 :

2 alleles

98.4% missing data

1	(0.682) 0.676 0.689

2	(0.318) 0.324 0.311

Locus 6042 :

2 alleles

99.2% missing data

2	(0.607) 0.601 0.608

4	(0.393) 0.399 0.392

Locus 6043 :

2 alleles

99.2% missing data

3	(0.440) 0.444 0.443

1	(0.560) 0.556 0.557

Locus 6044 :

2 alleles

99.2% missing data

2	(0.398) 0.400 0.398

1	(0.602) 0.600 0.602

Locus 6045 :

2 alleles

99.2% missing data

2	(0.425) 0.416 0.430

3	(0.575) 0.584 0.570

Locus 6046 :

2 alleles

99.2% missing data

2	(0.186) 0.185 0.191

1	(0.814) 0.815 0.809

Locus 6047 :

2 alleles

96.9% missing data

3	(0.820) 0.810 0.834

1	(0.180) 0.190 0.166

Locus 6048 :

2 alleles

91.0% missing data

1	(0.919) 0.934 0.908

2	(0.081) 0.066 0.092

Locus 6049 :

2 alleles

99.2% missing data

1	(0.551) 0.547 0.556

4	(0.449) 0.453 0.444

Locus 6050 :

2 alleles

99.2% missing data

1	(0.666) 0.668 0.662

3	(0.334) 0.332 0.338

Locus 6051 :

2 alleles

99.2% missing data

4	(0.347) 0.350 0.343

3	(0.653) 0.650 0.657

Locus 6052 :

2 alleles

99.2% missing data

2	(0.643) 0.642 0.644

3	(0.357) 0.358 0.356

Locus 6053 :

2 alleles

99.2% missing data

2	(0.771) 0.774 0.766

1	(0.229) 0.226 0.234

Locus 6054 :

2 alleles

99.2% missing data

4	(0.557) 0.554 0.557

1	(0.443) 0.446 0.443

Locus 6055 :

2 alleles

99.2% missing data

1	(0.471) 0.475 0.466

3	(0.529) 0.525 0.534

Locus 6056 :

2 alleles

99.2% missing data

4	(0.717) 0.721 0.713

1	(0.283) 0.279 0.287

Locus 6057 :

2 alleles

99.2% missing data

2	(0.725) 0.727 0.725

1	(0.275) 0.273 0.275

Locus 6058 :

2 alleles

99.2% missing data

2	(0.380) 0.372 0.379

3	(0.620) 0.628 0.621

Locus 6059 :

2 alleles

99.2% missing data

3	(0.485) 0.483 0.490

4	(0.515) 0.517 0.510

Locus 6060 :

2 alleles

98.8% missing data

3	(0.382) 0.378 0.383

2	(0.618) 0.622 0.617

Locus 6061 :

2 alleles

99.2% missing data

1	(0.402) 0.400 0.400

4	(0.598) 0.600 0.600

Locus 6062 :

2 alleles

99.2% missing data

4	(0.288) 0.289 0.293

3	(0.712) 0.711 0.707

Locus 6063 :

2 alleles

91.0% missing data

1	(0.944) 0.953 0.936

3	(0.056) 0.047 0.064

Locus 6064 :

2 alleles

99.2% missing data

2	(0.301) 0.297 0.304

4	(0.699) 0.703 0.696

Locus 6065 :

2 alleles

99.2% missing data

1	(0.527) 0.524 0.530

2	(0.473) 0.476 0.470

Locus 6066 :

2 alleles

8.2% missing data

3	(0.984) 0.977 0.996

4	(0.016) 0.023 0.004

Locus 6067 :

2 alleles

8.6% missing data

3	(0.970) 0.935 0.990

1	(0.030) 0.065 0.010

Locus 6068 :

2 alleles

96.9% missing data

2	(0.765) 0.777 0.754

3	(0.235) 0.223 0.246

Locus 6069 :

2 alleles

99.2% missing data

3	(0.447) 0.443 0.442

4	(0.553) 0.557 0.558

Locus 6070 :

3 alleles

98.4% missing data

4	(0.296) 0.293 0.293

2	(0.209) 0.220 0.206

3	(0.494) 0.486 0.501

Locus 6071 :

2 alleles

98.8% missing data

3	(0.796) 0.796 0.796

1	(0.204) 0.204 0.204

Locus 6072 :

2 alleles

99.2% missing data

2	(0.182) 0.186 0.186

1	(0.818) 0.814 0.814

Locus 6073 :

2 alleles

98.8% missing data

2	(0.371) 0.377 0.371

1	(0.629) 0.623 0.629

Locus 6074 :

2 alleles

99.2% missing data

2	(0.781) 0.786 0.775

1	(0.219) 0.214 0.225

Locus 6075 :

2 alleles

99.2% missing data

2	(0.275) 0.270 0.282

1	(0.725) 0.730 0.718

Locus 6076 :

2 alleles

96.1% missing data

2	(0.759) 0.764 0.764

1	(0.241) 0.236 0.236

Locus 6077 :

2 alleles

99.2% missing data

2	(0.630) 0.623 0.636

1	(0.370) 0.377 0.364

Locus 6078 :

2 alleles

98.8% missing data

1	(0.514) 0.507 0.522

2	(0.486) 0.493 0.478

Locus 6079 :

2 alleles

0.0% missing data

2	(0.988) 0.991 0.993

3	(0.012) 0.009 0.007

Locus 6080 :

2 alleles

97.7% missing data

2	(0.226) 0.231 0.216

1	(0.774) 0.769 0.784

Locus 6081 :

2 alleles

99.2% missing data

1	(0.779) 0.782 0.777

4	(0.221) 0.218 0.223

Locus 6082 :

3 alleles

98.4% missing data

3	(0.387) 0.390 0.379

4	(0.329) 0.330 0.336

1	(0.284) 0.280 0.285

Locus 6083 :

2 alleles

99.2% missing data

1	(0.424) 0.417 0.422

4	(0.576) 0.583 0.578

Locus 6084 :

3 alleles

98.4% missing data

1	(0.241) 0.238 0.247

4	(0.485) 0.486 0.480

3	(0.274) 0.276 0.274

Locus 6085 :

2 alleles

99.2% missing data

2	(0.855) 0.851 0.855

3	(0.145) 0.149 0.145

Locus 6086 :

2 alleles

90.2% missing data

4	(0.921) 0.922 0.927

1	(0.079) 0.078 0.073

Locus 6087 :

2 alleles

99.2% missing data

4	(0.773) 0.770 0.779

1	(0.227) 0.230 0.221

Locus 6088 :

2 alleles

98.4% missing data

4	(0.354) 0.355 0.359

1	(0.646) 0.645 0.641

Locus 6089 :

2 alleles

99.2% missing data

2	(0.497) 0.494 0.508

4	(0.503) 0.506 0.492

Locus 6090 :

2 alleles

99.2% missing data

4	(0.812) 0.808 0.821

2	(0.188) 0.192 0.179

Locus 6091 :

2 alleles

91.0% missing data

3	(0.934) 0.947 0.925

2	(0.066) 0.053 0.075

Locus 6092 :

2 alleles

99.2% missing data

2	(0.614) 0.617 0.614

1	(0.386) 0.383 0.386

Locus 6093 :

2 alleles

94.5% missing data

1	(0.828) 0.832 0.836

3	(0.172) 0.168 0.164

Locus 6094 :

2 alleles

99.2% missing data

4	(0.455) 0.457 0.460

1	(0.545) 0.543 0.540

Locus 6095 :

2 alleles

99.2% missing data

3	(0.762) 0.767 0.760

1	(0.238) 0.233 0.240

Locus 6096 :

2 alleles

99.2% missing data

1	(0.230) 0.230 0.230

4	(0.770) 0.770 0.770

Locus 6097 :

2 alleles

99.2% missing data

2	(0.337) 0.340 0.342

1	(0.663) 0.660 0.658

Locus 6098 :

2 alleles

99.2% missing data

1	(0.493) 0.488 0.496

3	(0.507) 0.512 0.504

Locus 6099 :

2 alleles

98.4% missing data

2	(0.638) 0.636 0.650

1	(0.362) 0.364 0.350

Locus 6100 :

2 alleles

99.2% missing data

3	(0.477) 0.475 0.474

1	(0.523) 0.525 0.526

Locus 6101 :

2 alleles

9.8% missing data

1	(0.986) 0.980 0.995

3	(0.014) 0.020 0.005

Locus 6102 :

2 alleles

99.2% missing data

4	(0.695) 0.689 0.697

3	(0.305) 0.311 0.303

Locus 6103 :

2 alleles

99.2% missing data

3	(0.365) 0.372 0.359

1	(0.635) 0.628 0.641

Locus 6104 :

2 alleles

96.1% missing data

1	(0.894) 0.905 0.887

4	(0.106) 0.095 0.113

Locus 6105 :

3 alleles

98.4% missing data

1	(0.329) 0.327 0.332

3	(0.359) 0.358 0.359

2	(0.312) 0.316 0.309

Locus 6106 :

2 alleles

99.2% missing data

1	(0.716) 0.717 0.713

2	(0.284) 0.283 0.287

Locus 6107 :

2 alleles

99.2% missing data

3	(0.300) 0.303 0.300

4	(0.700) 0.697 0.700

Locus 6108 :

2 alleles

99.2% missing data

4	(0.392) 0.400 0.392

2	(0.608) 0.600 0.608

Locus 6109 :

2 alleles

96.1% missing data

2	(0.790) 0.796 0.790

4	(0.210) 0.204 0.210

Locus 6110 :

2 alleles

98.8% missing data

2	(0.364) 0.365 0.368

3	(0.636) 0.635 0.632

Locus 6111 :

2 alleles

97.7% missing data

4	(0.249) 0.239 0.255

1	(0.751) 0.761 0.745

Locus 6112 :

2 alleles

99.2% missing data

2	(0.436) 0.446 0.429

1	(0.564) 0.554 0.571

Locus 6113 :

2 alleles

99.2% missing data

4	(0.698) 0.702 0.690

3	(0.302) 0.298 0.310

Locus 6114 :

3 alleles

98.8% missing data

3	(0.488) 0.483 0.491

4	(0.220) 0.223 0.223

2	(0.291) 0.294 0.286

Locus 6115 :

2 alleles

91.0% missing data

4	(0.894) 0.906 0.890

2	(0.106) 0.094 0.110

Locus 6116 :

2 alleles

99.2% missing data

2	(0.407) 0.406 0.409

3	(0.593) 0.594 0.591

Locus 6117 :

3 alleles

97.3% missing data

4	(0.151) 0.147 0.156

1	(0.641) 0.648 0.645

2	(0.207) 0.205 0.199

Locus 6118 :

2 alleles

90.6% missing data

1	(0.939) 0.951 0.929

3	(0.061) 0.049 0.071

Locus 6119 :

2 alleles

99.2% missing data

2	(0.415) 0.416 0.412

4	(0.585) 0.584 0.588

Locus 6120 :

2 alleles

98.8% missing data

4	(0.523) 0.519 0.515

2	(0.477) 0.481 0.485

Locus 6121 :

2 alleles

93.0% missing data

2	(0.832) 0.831 0.851

1	(0.168) 0.169 0.149

Locus 6122 :

2 alleles

99.2% missing data

4	(0.298) 0.294 0.297

1	(0.702) 0.706 0.703

Locus 6123 :

2 alleles

92.6% missing data

1	(0.880) 0.882 0.890

3	(0.120) 0.118 0.110

Locus 6124 :

2 alleles

98.8% missing data

1	(0.399) 0.394 0.401

4	(0.601) 0.606 0.599

Locus 6125 :

2 alleles

98.8% missing data

2	(0.677) 0.679 0.672

1	(0.323) 0.321 0.328

Locus 6126 :

3 alleles

95.3% missing data

3	(0.708) 0.710 0.723

4	(0.115) 0.114 0.109

2	(0.178) 0.175 0.168

Locus 6127 :

3 alleles

98.8% missing data

3	(0.465) 0.462 0.466

2	(0.245) 0.244 0.250

1	(0.289) 0.295 0.283

Locus 6128 :

2 alleles

99.2% missing data

3	(0.433) 0.431 0.433

2	(0.567) 0.569 0.567

Locus 6129 :

2 alleles

99.2% missing data

3	(0.694) 0.691 0.696

1	(0.306) 0.309 0.304

Locus 6130 :

2 alleles

99.2% missing data

1	(0.485) 0.484 0.489

3	(0.515) 0.516 0.511

Locus 6131 :

2 alleles

98.8% missing data

4	(0.647) 0.649 0.652

2	(0.353) 0.351 0.348

Locus 6132 :

2 alleles

99.2% missing data

3	(0.768) 0.772 0.762

1	(0.232) 0.228 0.238

Locus 6133 :

2 alleles

90.6% missing data

1	(0.936) 0.945 0.932

4	(0.064) 0.055 0.068

Locus 6134 :

2 alleles

91.0% missing data

2	(0.922) 0.935 0.918

3	(0.078) 0.065 0.082

Locus 6135 :

2 alleles

99.2% missing data

1	(0.348) 0.344 0.351

3	(0.652) 0.656 0.649

Locus 6136 :

2 alleles

99.2% missing data

1	(0.629) 0.616 0.634

2	(0.371) 0.384 0.366

Locus 6137 :

2 alleles

0.0% missing data

1	(0.988) 0.984 0.997

4	(0.012) 0.016 0.003

Locus 6138 :

2 alleles

98.0% missing data

1	(0.611) 0.620 0.616

4	(0.389) 0.380 0.384

Locus 6139 :

2 alleles

98.8% missing data

2	(0.729) 0.728 0.730

4	(0.271) 0.272 0.270

Locus 6140 :

2 alleles

99.2% missing data

4	(0.351) 0.346 0.349

1	(0.649) 0.654 0.651

Locus 6141 :

2 alleles

99.2% missing data

3	(0.496) 0.494 0.500

1	(0.504) 0.506 0.500

Locus 6142 :

2 alleles

99.2% missing data

4	(0.448) 0.447 0.445

1	(0.552) 0.553 0.555

Locus 6143 :

2 alleles

98.4% missing data

2	(0.720) 0.720 0.714

3	(0.280) 0.280 0.286

Locus 6144 :

2 alleles

97.7% missing data

2	(0.854) 0.848 0.864

1	(0.146) 0.152 0.136

Locus 6145 :

2 alleles

98.8% missing data

2	(0.168) 0.161 0.176

3	(0.832) 0.839 0.824

Locus 6146 :

2 alleles

99.2% missing data

2	(0.324) 0.320 0.329

3	(0.676) 0.680 0.671

Locus 6147 :

2 alleles

97.3% missing data

1	(0.821) 0.821 0.828

2	(0.179) 0.179 0.172

Locus 6148 :

2 alleles

99.2% missing data

1	(0.450) 0.448 0.449

2	(0.550) 0.552 0.551

Locus 6149 :

2 alleles

98.8% missing data

3	(0.686) 0.685 0.688

4	(0.314) 0.315 0.312

Locus 6150 :

2 alleles

98.8% missing data

4	(0.676) 0.668 0.677

3	(0.324) 0.332 0.323

Locus 6151 :

3 alleles

94.9% missing data

2	(0.744) 0.738 0.764

1	(0.132) 0.132 0.126

4	(0.124) 0.130 0.110

Locus 6152 :

2 alleles

99.2% missing data

3	(0.411) 0.407 0.416

2	(0.589) 0.593 0.584

Locus 6153 :

2 alleles

98.0% missing data

1	(0.781) 0.784 0.783

4	(0.219) 0.216 0.217

Locus 6154 :

2 alleles

99.2% missing data

2	(0.460) 0.455 0.464

4	(0.540) 0.545 0.536

Locus 6155 :

2 alleles

98.8% missing data

1	(0.746) 0.749 0.743

3	(0.254) 0.251 0.257

Locus 6156 :

2 alleles

98.8% missing data

3	(0.243) 0.239 0.252

4	(0.757) 0.761 0.748

Locus 6157 :

2 alleles

99.2% missing data

4	(0.452) 0.452 0.455

1	(0.548) 0.548 0.545

Locus 6158 :

2 alleles

99.2% missing data

2	(0.389) 0.385 0.396

3	(0.611) 0.615 0.604

Locus 6159 :

2 alleles

99.2% missing data

4	(0.651) 0.650 0.661

3	(0.349) 0.350 0.339

Locus 6160 :

2 alleles

98.8% missing data

3	(0.652) 0.654 0.651

4	(0.348) 0.346 0.349

Locus 6161 :

2 alleles

99.2% missing data

1	(0.610) 0.600 0.611

3	(0.390) 0.400 0.389

Locus 6162 :

3 alleles

96.9% missing data

2	(0.185) 0.186 0.174

1	(0.650) 0.643 0.666

3	(0.165) 0.171 0.159

Locus 6163 :

2 alleles

88.7% missing data

4	(0.761) 0.808 0.712

3	(0.239) 0.192 0.288

Locus 6164 :

2 alleles

99.2% missing data

1	(0.519) 0.514 0.521

3	(0.481) 0.486 0.479

Locus 6165 :

2 alleles

96.5% missing data

1	(0.878) 0.883 0.871

3	(0.122) 0.117 0.129

Locus 6166 :

2 alleles

91.0% missing data

2	(0.918) 0.935 0.910

1	(0.082) 0.065 0.090

Locus 6167 :

3 alleles

98.4% missing data

4	(0.359) 0.351 0.356

2	(0.308) 0.321 0.309

1	(0.333) 0.327 0.334

Locus 6168 :

2 alleles

99.2% missing data

2	(0.598) 0.597 0.599

4	(0.402) 0.403 0.401

Locus 6169 :

2 alleles

98.8% missing data

1	(0.632) 0.639 0.626

2	(0.368) 0.361 0.374

Locus 6170 :

2 alleles

99.2% missing data

3	(0.510) 0.503 0.512

2	(0.490) 0.497 0.488

Locus 6171 :

2 alleles

99.2% missing data

1	(0.485) 0.493 0.484

2	(0.515) 0.507 0.516

Locus 6172 :

2 alleles

96.9% missing data

2	(0.764) 0.755 0.776

1	(0.236) 0.245 0.224

Locus 6173 :

2 alleles

99.2% missing data

1	(0.789) 0.785 0.791

2	(0.211) 0.215 0.209

Locus 6174 :

2 alleles

99.2% missing data

2	(0.437) 0.433 0.446

1	(0.563) 0.567 0.554

Locus 6175 :

3 alleles

98.0% missing data

2	(0.410) 0.403 0.424

1	(0.395) 0.394 0.389

4	(0.194) 0.203 0.187

Locus 6176 :

3 alleles

98.4% missing data

1	(0.335) 0.330 0.338

2	(0.460) 0.449 0.463

3	(0.205) 0.221 0.199

Locus 6177 :

2 alleles

99.2% missing data

1	(0.600) 0.596 0.601

2	(0.400) 0.404 0.399

Locus 6178 :

2 alleles

96.5% missing data

3	(0.284) 0.269 0.294

2	(0.716) 0.731 0.706

Locus 6179 :

2 alleles

99.2% missing data

1	(0.784) 0.787 0.777

3	(0.216) 0.213 0.223

Locus 6180 :

2 alleles

96.1% missing data

4	(0.695) 0.706 0.693

3	(0.305) 0.294 0.307

Locus 6181 :

2 alleles

99.2% missing data

4	(0.705) 0.700 0.706

1	(0.295) 0.300 0.294

Locus 6182 :

2 alleles

99.2% missing data

1	(0.380) 0.379 0.382

3	(0.620) 0.621 0.618

Locus 6183 :

2 alleles

99.2% missing data

1	(0.617) 0.618 0.619

4	(0.383) 0.382 0.381

Locus 6184 :

3 alleles

98.8% missing data

2	(0.267) 0.263 0.272

1	(0.191) 0.189 0.201

4	(0.542) 0.548 0.526

Locus 6185 :

2 alleles

96.1% missing data

3	(0.815) 0.822 0.819

2	(0.185) 0.178 0.181

Locus 6186 :

2 alleles

99.2% missing data

1	(0.324) 0.320 0.325

3	(0.676) 0.680 0.675

Locus 6187 :

2 alleles

96.1% missing data

2	(0.193) 0.188 0.189

3	(0.807) 0.812 0.811

Locus 6188 :

2 alleles

98.8% missing data

4	(0.756) 0.763 0.753

2	(0.244) 0.237 0.247

Locus 6189 :

2 alleles

88.3% missing data

1	(0.767) 0.810 0.723

4	(0.233) 0.190 0.277

Locus 6190 :

2 alleles

99.2% missing data

2	(0.507) 0.506 0.510

1	(0.493) 0.494 0.490

Locus 6191 :

2 alleles

98.8% missing data

1	(0.193) 0.192 0.198

2	(0.807) 0.808 0.802

Locus 6192 :

2 alleles

99.2% missing data

4	(0.293) 0.289 0.290

3	(0.707) 0.711 0.710

Locus 6193 :

2 alleles

92.6% missing data

2	(0.858) 0.859 0.869

4	(0.142) 0.141 0.131

Locus 6194 :

2 alleles

8.2% missing data

3	(0.981) 0.971 0.994

1	(0.019) 0.029 0.006

Locus 6195 :

2 alleles

99.2% missing data

4	(0.309) 0.307 0.312

3	(0.691) 0.693 0.688

Locus 6196 :

2 alleles

96.1% missing data

2	(0.238) 0.227 0.243

4	(0.762) 0.773 0.757

Locus 6197 :

2 alleles

99.2% missing data

2	(0.703) 0.708 0.693

1	(0.297) 0.292 0.307

Locus 6198 :

2 alleles

99.2% missing data

1	(0.662) 0.659 0.663

4	(0.338) 0.341 0.337

Locus 6199 :

2 alleles

99.2% missing data

3	(0.798) 0.796 0.801

4	(0.202) 0.204 0.199

Locus 6200 :

2 alleles

99.2% missing data

2	(0.719) 0.716 0.713

4	(0.281) 0.284 0.287

Locus 6201 :

2 alleles

99.2% missing data

2	(0.694) 0.687 0.697

3	(0.306) 0.313 0.303

Locus 6202 :

2 alleles

91.0% missing data

3	(0.929) 0.935 0.930

1	(0.071) 0.065 0.070

Locus 6203 :

2 alleles

99.2% missing data

3	(0.806) 0.809 0.800

4	(0.194) 0.191 0.200

Locus 6204 :

2 alleles

99.2% missing data

3	(0.616) 0.612 0.623

1	(0.384) 0.388 0.377

Locus 6205 :

2 alleles

0.0% missing data

4	(0.984) 0.989 0.992

1	(0.016) 0.011 0.008

Locus 6206 :

2 alleles

99.2% missing data

3	(0.536) 0.537 0.537

1	(0.464) 0.463 0.463

Locus 6207 :

2 alleles

99.2% missing data

1	(0.554) 0.553 0.557

2	(0.446) 0.447 0.443

Locus 6208 :

2 alleles

99.2% missing data

4	(0.542) 0.532 0.552

2	(0.458) 0.468 0.448

Locus 6209 :

2 alleles

99.2% missing data

3	(0.170) 0.165 0.179

2	(0.830) 0.835 0.821

Locus 6210 :

2 alleles

99.2% missing data

4	(0.343) 0.346 0.335

2	(0.657) 0.654 0.665

Locus 6211 :

2 alleles

95.7% missing data

4	(0.861) 0.870 0.856

1	(0.139) 0.130 0.144

Locus 6212 :

2 alleles

99.2% missing data

3	(0.176) 0.172 0.184

4	(0.824) 0.828 0.816

Locus 6213 :

2 alleles

99.2% missing data

3	(0.800) 0.797 0.805

4	(0.200) 0.203 0.195

Locus 6214 :

2 alleles

98.0% missing data

2	(0.245) 0.245 0.237

3	(0.755) 0.755 0.763

Locus 6215 :

2 alleles

98.4% missing data

3	(0.367) 0.362 0.372

1	(0.633) 0.638 0.628

Locus 6216 :

2 alleles

99.2% missing data

3	(0.156) 0.154 0.162

2	(0.844) 0.846 0.838

Locus 6217 :

2 alleles

99.2% missing data

4	(0.245) 0.248 0.248

1	(0.755) 0.752 0.752

Locus 6218 :

2 alleles

98.8% missing data

1	(0.286) 0.283 0.295

4	(0.714) 0.717 0.705

Locus 6219 :

2 alleles

99.2% missing data

2	(0.615) 0.619 0.613

4	(0.385) 0.381 0.387

Locus 6220 :

2 alleles

98.8% missing data

4	(0.593) 0.595 0.593

1	(0.407) 0.405 0.407

Locus 6221 :

2 alleles

86.3% missing data

4	(0.945) 0.945 0.950

2	(0.055) 0.055 0.050

Locus 6222 :

2 alleles

98.8% missing data

1	(0.766) 0.767 0.767

2	(0.234) 0.233 0.233

Locus 6223 :

2 alleles

91.4% missing data

2	(0.901) 0.921 0.893

3	(0.099) 0.079 0.107

Locus 6224 :

2 alleles

8.2% missing data

3	(0.984) 0.975 0.995

2	(0.016) 0.025 0.005

Locus 6225 :

2 alleles

99.2% missing data

1	(0.671) 0.679 0.666

3	(0.329) 0.321 0.334

Locus 6226 :

2 alleles

99.2% missing data

4	(0.412) 0.413 0.418

3	(0.588) 0.587 0.582

Locus 6227 :

2 alleles

98.8% missing data

3	(0.575) 0.576 0.574

2	(0.425) 0.424 0.426

Locus 6228 :

2 alleles

96.9% missing data

3	(0.161) 0.162 0.156

1	(0.839) 0.838 0.844

Locus 6229 :

2 alleles

99.2% missing data

2	(0.528) 0.530 0.528

4	(0.472) 0.470 0.472

Locus 6230 :

3 alleles

98.8% missing data

1	(0.543) 0.541 0.542

4	(0.268) 0.275 0.266

3	(0.189) 0.184 0.193

Locus 6231 :

2 alleles

99.2% missing data

3	(0.377) 0.375 0.378

2	(0.623) 0.625 0.622

Locus 6232 :

2 alleles

0.0% missing data

1	(0.987) 0.983 0.996

4	(0.013) 0.017 0.004

Locus 6233 :

2 alleles

99.2% missing data

3	(0.489) 0.498 0.491

1	(0.511) 0.502 0.509

Locus 6234 :

2 alleles

98.8% missing data

3	(0.622) 0.619 0.626

2	(0.378) 0.381 0.374

Locus 6235 :

2 alleles

98.4% missing data

3	(0.449) 0.465 0.445

2	(0.551) 0.535 0.555

Locus 6236 :

2 alleles

98.8% missing data

3	(0.362) 0.356 0.373

1	(0.638) 0.644 0.627

Locus 6237 :

2 alleles

99.2% missing data

3	(0.491) 0.491 0.490

1	(0.509) 0.509 0.510

Locus 6238 :

2 alleles

99.2% missing data

1	(0.433) 0.429 0.438

3	(0.567) 0.571 0.562

Locus 6239 :

2 alleles

99.2% missing data

3	(0.390) 0.394 0.388

1	(0.610) 0.606 0.612

Locus 6240 :

2 alleles

99.2% missing data

4	(0.456) 0.455 0.463

3	(0.544) 0.545 0.537

Locus 6241 :

2 alleles

99.2% missing data

3	(0.752) 0.752 0.748

1	(0.248) 0.248 0.252

Locus 6242 :

3 alleles

98.4% missing data

3	(0.240) 0.236 0.241

1	(0.366) 0.372 0.366

2	(0.394) 0.392 0.393

Locus 6243 :

2 alleles

99.2% missing data

3	(0.366) 0.363 0.367

1	(0.634) 0.637 0.633

Locus 6244 :

3 alleles

94.5% missing data

2	(0.737) 0.724 0.770

4	(0.100) 0.107 0.088

3	(0.163) 0.169 0.143

Locus 6245 :

2 alleles

96.9% missing data

3	(0.707) 0.714 0.708

4	(0.293) 0.286 0.292

Locus 6246 :

2 alleles

98.8% missing data

4	(0.537) 0.533 0.539

3	(0.463) 0.467 0.461

Locus 6247 :

2 alleles

98.8% missing data

3	(0.712) 0.711 0.710

4	(0.288) 0.289 0.290

Locus 6248 :

3 alleles

98.4% missing data

4	(0.417) 0.421 0.414

1	(0.239) 0.234 0.241

3	(0.343) 0.345 0.344

Locus 6249 :

2 alleles

98.8% missing data

1	(0.333) 0.328 0.335

3	(0.667) 0.672 0.665

Locus 6250 :

2 alleles

98.4% missing data

4	(0.540) 0.541 0.537

3	(0.460) 0.459 0.463

Locus 6251 :

2 alleles

99.2% missing data

1	(0.701) 0.692 0.708

2	(0.299) 0.308 0.292

Locus 6252 :

2 alleles

99.2% missing data

4	(0.660) 0.658 0.658

1	(0.340) 0.342 0.342

Locus 6253 :

2 alleles

99.2% missing data

3	(0.487) 0.487 0.488

4	(0.513) 0.513 0.512

Locus 6254 :

2 alleles

99.2% missing data

3	(0.108) 0.106 0.113

1	(0.892) 0.894 0.887

Locus 6255 :

2 alleles

0.0% missing data

1	(0.985) 0.980 0.995

4	(0.015) 0.020 0.005

Locus 6256 :

2 alleles

99.2% missing data

2	(0.627) 0.623 0.630

1	(0.373) 0.377 0.370

Locus 6257 :

2 alleles

98.8% missing data

1	(0.719) 0.725 0.712

3	(0.281) 0.275 0.288

Locus 6258 :

2 alleles

98.4% missing data

4	(0.705) 0.711 0.709

1	(0.295) 0.289 0.291

Locus 6259 :

2 alleles

99.2% missing data

3	(0.494) 0.496 0.489

1	(0.506) 0.504 0.511

Locus 6260 :

2 alleles

99.2% missing data

1	(0.200) 0.201 0.202

2	(0.800) 0.799 0.798

Locus 6261 :

2 alleles

98.8% missing data

3	(0.702) 0.694 0.705

4	(0.298) 0.306 0.295

Locus 6262 :

2 alleles

96.9% missing data

3	(0.722) 0.715 0.742

2	(0.278) 0.285 0.258

Locus 6263 :

3 alleles

98.4% missing data

4	(0.176) 0.178 0.178

1	(0.636) 0.639 0.630

2	(0.188) 0.183 0.193

Locus 6264 :

2 alleles

99.2% missing data

3	(0.693) 0.686 0.698

1	(0.307) 0.314 0.302

Locus 6265 :

3 alleles

98.0% missing data

1	(0.379) 0.366 0.383

3	(0.197) 0.198 0.201

4	(0.425) 0.436 0.416

Locus 6266 :

2 alleles

99.2% missing data

1	(0.444) 0.443 0.441

3	(0.556) 0.557 0.559

Locus 6267 :

2 alleles

95.3% missing data

3	(0.880) 0.882 0.885

2	(0.120) 0.118 0.115

Locus 6268 :

2 alleles

99.2% missing data

3	(0.452) 0.445 0.451

1	(0.548) 0.555 0.549

Locus 6269 :

2 alleles

99.2% missing data

3	(0.640) 0.631 0.645

1	(0.360) 0.369 0.355

Locus 6270 :

3 alleles

92.2% missing data

3	(0.676) 0.650 0.719

1	(0.155) 0.173 0.136

2	(0.169) 0.177 0.145

Locus 6271 :

2 alleles

91.0% missing data

1	(0.929) 0.944 0.920

4	(0.071) 0.056 0.080

Locus 6272 :

2 alleles

98.4% missing data

1	(0.597) 0.597 0.594

4	(0.403) 0.403 0.406

Locus 6273 :

2 alleles

99.2% missing data

1	(0.725) 0.717 0.729

2	(0.275) 0.283 0.271

Locus 6274 :

2 alleles

99.2% missing data

3	(0.272) 0.273 0.276

2	(0.728) 0.727 0.724

Locus 6275 :

2 alleles

98.8% missing data

1	(0.534) 0.533 0.537

2	(0.466) 0.467 0.463

Locus 6276 :

2 alleles

99.2% missing data

1	(0.464) 0.465 0.461

3	(0.536) 0.535 0.539

Locus 6277 :

2 alleles

92.6% missing data

2	(0.904) 0.905 0.912

1	(0.096) 0.095 0.088

Locus 6278 :

2 alleles

99.2% missing data

3	(0.487) 0.484 0.489

2	(0.513) 0.516 0.511

Locus 6279 :

2 alleles

99.2% missing data

3	(0.555) 0.560 0.557

2	(0.445) 0.440 0.443

Locus 6280 :

2 alleles

99.2% missing data

1	(0.397) 0.398 0.401

3	(0.603) 0.602 0.599

Locus 6281 :

2 alleles

99.2% missing data

3	(0.392) 0.387 0.401

1	(0.608) 0.613 0.599

Locus 6282 :

2 alleles

99.2% missing data

1	(0.599) 0.595 0.602

3	(0.401) 0.405 0.398

Locus 6283 :

2 alleles

98.0% missing data

4	(0.695) 0.686 0.700

1	(0.305) 0.314 0.300

Locus 6284 :

2 alleles

99.2% missing data

4	(0.621) 0.615 0.624

3	(0.379) 0.385 0.376

Locus 6285 :

3 alleles

98.8% missing data

2	(0.555) 0.553 0.553

3	(0.219) 0.219 0.224

1	(0.226) 0.228 0.223

Locus 6286 :

3 alleles

98.8% missing data

1	(0.443) 0.442 0.438

2	(0.127) 0.120 0.134

3	(0.431) 0.437 0.428

Locus 6287 :

2 alleles

96.1% missing data

4	(0.767) 0.790 0.763

1	(0.233) 0.210 0.237

Locus 6288 :

2 alleles

90.6% missing data

3	(0.926) 0.923 0.937

4	(0.074) 0.077 0.063

Locus 6289 :

2 alleles

99.2% missing data

1	(0.675) 0.680 0.671

4	(0.325) 0.320 0.329

Locus 6290 :

2 alleles

99.2% missing data

1	(0.402) 0.402 0.401

3	(0.598) 0.598 0.599

Locus 6291 :

2 alleles

99.2% missing data

2	(0.512) 0.508 0.515

1	(0.488) 0.492 0.485

Locus 6292 :

2 alleles

98.8% missing data

1	(0.338) 0.332 0.337

3	(0.662) 0.668 0.663

Locus 6293 :

3 alleles

98.8% missing data

3	(0.294) 0.285 0.292

1	(0.395) 0.392 0.400

2	(0.311) 0.322 0.307

Locus 6294 :

3 alleles

94.9% missing data

4	(0.727) 0.729 0.740

3	(0.161) 0.158 0.150

2	(0.112) 0.113 0.110

Locus 6295 :

2 alleles

99.2% missing data

1	(0.789) 0.789 0.785

3	(0.211) 0.211 0.215

Locus 6296 :

2 alleles

96.1% missing data

3	(0.853) 0.864 0.849

4	(0.147) 0.136 0.151

Locus 6297 :

2 alleles

98.8% missing data

4	(0.440) 0.440 0.439

1	(0.560) 0.560 0.561

Locus 6298 :

2 alleles

99.2% missing data

4	(0.446) 0.444 0.449

2	(0.554) 0.556 0.551

Locus 6299 :

2 alleles

98.4% missing data

1	(0.830) 0.826 0.837

4	(0.170) 0.174 0.163

Locus 6300 :

2 alleles

99.2% missing data

2	(0.446) 0.449 0.442

4	(0.554) 0.551 0.558

Locus 6301 :

2 alleles

99.2% missing data

2	(0.342) 0.336 0.348

3	(0.658) 0.664 0.652

Locus 6302 :

2 alleles

96.1% missing data

4	(0.128) 0.121 0.132

1	(0.872) 0.879 0.868

Locus 6303 :

2 alleles

99.2% missing data

2	(0.501) 0.497 0.505

1	(0.499) 0.503 0.495

Locus 6304 :

2 alleles

99.2% missing data

1	(0.481) 0.475 0.482

2	(0.519) 0.525 0.518

Locus 6305 :

2 alleles

93.8% missing data

4	(0.869) 0.868 0.877

1	(0.131) 0.132 0.123

Locus 6306 :

2 alleles

98.0% missing data

3	(0.293) 0.299 0.290

4	(0.707) 0.701 0.710

Locus 6307 :

2 alleles

98.0% missing data

4	(0.844) 0.849 0.843

3	(0.156) 0.151 0.157

Locus 6308 :

2 alleles

99.2% missing data

1	(0.423) 0.424 0.420

4	(0.577) 0.576 0.580

Locus 6309 :

2 alleles

99.2% missing data

1	(0.394) 0.401 0.382

2	(0.606) 0.599 0.618

Locus 6310 :

2 alleles

98.4% missing data

1	(0.584) 0.578 0.588

3	(0.416) 0.422 0.412

Locus 6311 :

2 alleles

99.2% missing data

4	(0.305) 0.308 0.306

1	(0.695) 0.692 0.694

Locus 6312 :

2 alleles

92.6% missing data

1	(0.138) 0.141 0.131

4	(0.862) 0.859 0.869

Locus 6313 :

2 alleles

99.2% missing data

1	(0.530) 0.519 0.538

2	(0.470) 0.481 0.462

Locus 6314 :

2 alleles

98.4% missing data

2	(0.695) 0.704 0.690

4	(0.305) 0.296 0.310

Locus 6315 :

3 alleles

90.2% missing data

4	(0.561) 0.517 0.617

3	(0.355) 0.398 0.314

2	(0.084) 0.085 0.069

Locus 6316 :

2 alleles

99.2% missing data

2	(0.615) 0.618 0.609

3	(0.385) 0.382 0.391

Locus 6317 :

3 alleles

98.8% missing data

4	(0.230) 0.237 0.226

2	(0.272) 0.269 0.279

1	(0.498) 0.493 0.495

Locus 6318 :

2 alleles

98.8% missing data

3	(0.391) 0.388 0.395

1	(0.609) 0.612 0.605

Locus 6319 :

2 alleles

99.2% missing data

2	(0.672) 0.666 0.677

4	(0.328) 0.334 0.323

Locus 6320 :

2 alleles

90.6% missing data

2	(0.870) 0.889 0.854

4	(0.130) 0.111 0.146

Locus 6321 :

2 alleles

98.8% missing data

2	(0.510) 0.516 0.508

1	(0.490) 0.484 0.492

Locus 6322 :

2 alleles

99.2% missing data

4	(0.532) 0.532 0.539

3	(0.468) 0.468 0.461

Locus 6323 :

2 alleles

8.2% missing data

2	(0.986) 0.978 0.996

1	(0.014) 0.022 0.004

Locus 6324 :

2 alleles

99.2% missing data

3	(0.660) 0.657 0.666

4	(0.340) 0.343 0.334

Locus 6325 :

3 alleles

95.7% missing data

4	(0.172) 0.165 0.173

2	(0.707) 0.712 0.710

3	(0.122) 0.123 0.116

Locus 6326 :

2 alleles

98.4% missing data

1	(0.279) 0.274 0.282

2	(0.721) 0.726 0.718

Locus 6327 :

2 alleles

98.4% missing data

4	(0.604) 0.611 0.595

3	(0.396) 0.389 0.405

Locus 6328 :

2 alleles

97.7% missing data

4	(0.330) 0.337 0.323

3	(0.670) 0.663 0.677

Locus 6329 :

2 alleles

8.6% missing data

4	(0.985) 0.980 0.994

1	(0.015) 0.020 0.006

Locus 6330 :

2 alleles

99.2% missing data

4	(0.406) 0.400 0.411

1	(0.594) 0.600 0.589

Locus 6331 :

2 alleles

91.0% missing data

2	(0.652) 0.620 0.700

4	(0.348) 0.380 0.300

Locus 6332 :

2 alleles

92.2% missing data

4	(0.887) 0.878 0.905

2	(0.113) 0.122 0.095

Locus 6333 :

2 alleles

96.1% missing data

1	(0.840) 0.849 0.836

3	(0.160) 0.151 0.164

Locus 6334 :

2 alleles

99.2% missing data

1	(0.461) 0.453 0.463

3	(0.539) 0.547 0.537

Locus 6335 :

2 alleles

99.2% missing data

2	(0.626) 0.630 0.627

4	(0.374) 0.370 0.373

Locus 6336 :

2 alleles

97.7% missing data

1	(0.265) 0.264 0.260

2	(0.735) 0.736 0.740

Locus 6337 :

2 alleles

8.6% missing data

3	(0.985) 0.976 0.995

4	(0.015) 0.024 0.005

Locus 6338 :

3 alleles

95.3% missing data

2	(0.809) 0.794 0.829

3	(0.068) 0.073 0.061

1	(0.122) 0.133 0.110

Locus 6339 :

2 alleles

97.7% missing data

4	(0.647) 0.651 0.649

2	(0.353) 0.349 0.351

Locus 6340 :

2 alleles

99.2% missing data

4	(0.490) 0.487 0.495

3	(0.510) 0.513 0.505

Locus 6341 :

2 alleles

99.2% missing data

1	(0.614) 0.607 0.625

2	(0.386) 0.393 0.375

Locus 6342 :

2 alleles

97.7% missing data

1	(0.360) 0.366 0.348

3	(0.640) 0.634 0.652

Locus 6343 :

2 alleles

17.2% missing data

4	(0.984) 0.973 0.995

1	(0.016) 0.027 0.005

Locus 6344 :

2 alleles

98.8% missing data

2	(0.639) 0.638 0.639

3	(0.361) 0.362 0.361

Locus 6345 :

2 alleles

99.2% missing data

3	(0.462) 0.462 0.462

4	(0.538) 0.538 0.538

Locus 6346 :

2 alleles

99.2% missing data

2	(0.495) 0.498 0.496

1	(0.505) 0.502 0.504

Locus 6347 :

2 alleles

8.6% missing data

1	(0.980) 0.963 0.994

4	(0.020) 0.037 0.006

Locus 6348 :

2 alleles

99.2% missing data

4	(0.597) 0.592 0.600

2	(0.403) 0.408 0.400

Locus 6349 :

2 alleles

98.0% missing data

4	(0.729) 0.738 0.730

3	(0.271) 0.262 0.270

Locus 6350 :

2 alleles

98.8% missing data

2	(0.444) 0.438 0.449

4	(0.556) 0.562 0.551

Locus 6351 :

2 alleles

99.2% missing data

4	(0.282) 0.291 0.276

2	(0.718) 0.709 0.724

Locus 6352 :

2 alleles

97.3% missing data

1	(0.134) 0.127 0.142

4	(0.866) 0.873 0.858

Locus 6353 :

2 alleles

99.2% missing data

2	(0.434) 0.429 0.442

1	(0.566) 0.571 0.558

Locus 6354 :

2 alleles

97.7% missing data

4	(0.208) 0.210 0.203

2	(0.792) 0.790 0.797

Locus 6355 :

2 alleles

97.3% missing data

2	(0.368) 0.375 0.363

1	(0.632) 0.625 0.637

Locus 6356 :

2 alleles

99.2% missing data

2	(0.760) 0.758 0.757

4	(0.240) 0.242 0.243

Locus 6357 :

2 alleles

96.9% missing data

1	(0.263) 0.252 0.272

4	(0.737) 0.748 0.728

Locus 6358 :

2 alleles

97.3% missing data

2	(0.804) 0.799 0.815

3	(0.196) 0.201 0.185

Locus 6359 :

2 alleles

98.8% missing data

1	(0.523) 0.515 0.522

4	(0.477) 0.485 0.478

Locus 6360 :

2 alleles

99.2% missing data

1	(0.370) 0.374 0.370

4	(0.630) 0.626 0.630

Locus 6361 :

2 alleles

99.2% missing data

2	(0.261) 0.263 0.263

4	(0.739) 0.737 0.737

Locus 6362 :

2 alleles

98.8% missing data

3	(0.323) 0.321 0.319

2	(0.677) 0.679 0.681

Locus 6363 :

2 alleles

97.3% missing data

4	(0.555) 0.541 0.568

2	(0.445) 0.459 0.432

Locus 6364 :

2 alleles

98.4% missing data

3	(0.622) 0.630 0.615

2	(0.378) 0.370 0.385

Locus 6365 :

2 alleles

99.2% missing data

1	(0.352) 0.343 0.355

4	(0.648) 0.657 0.645

Locus 6366 :

2 alleles

99.2% missing data

2	(0.787) 0.791 0.785

4	(0.213) 0.209 0.215

Locus 6367 :

2 alleles

90.2% missing data

2	(0.876) 0.901 0.859

4	(0.124) 0.099 0.141

Locus 6368 :

2 alleles

99.2% missing data

4	(0.474) 0.463 0.477

1	(0.526) 0.537 0.523

Locus 6369 :

2 alleles

95.3% missing data

4	(0.789) 0.788 0.806

3	(0.211) 0.212 0.194

Locus 6370 :

2 alleles

0.0% missing data

4	(0.985) 0.981 0.996

1	(0.015) 0.019 0.004

Locus 6371 :

2 alleles

99.2% missing data

2	(0.251) 0.250 0.252

3	(0.749) 0.750 0.748

Locus 6372 :

2 alleles

99.2% missing data

1	(0.356) 0.354 0.355

3	(0.644) 0.646 0.645

Locus 6373 :

2 alleles

99.2% missing data

2	(0.302) 0.300 0.308

4	(0.698) 0.700 0.692

Locus 6374 :

2 alleles

93.0% missing data

3	(0.919) 0.919 0.921

1	(0.081) 0.081 0.079

Locus 6375 :

2 alleles

95.3% missing data

4	(0.914) 0.912 0.917

1	(0.086) 0.088 0.083

Locus 6376 :

2 alleles

99.2% missing data

2	(0.676) 0.675 0.670

3	(0.324) 0.325 0.330

Locus 6377 :

2 alleles

99.2% missing data

4	(0.575) 0.572 0.572

3	(0.425) 0.428 0.428

Locus 6378 :

2 alleles

98.8% missing data

2	(0.580) 0.584 0.582

4	(0.420) 0.416 0.418

Locus 6379 :

2 alleles

99.2% missing data

2	(0.368) 0.368 0.373

3	(0.632) 0.632 0.627

Locus 6380 :

2 alleles

99.2% missing data

4	(0.393) 0.395 0.394

2	(0.607) 0.605 0.606

Locus 6381 :

2 alleles

99.2% missing data

2	(0.670) 0.673 0.662

4	(0.330) 0.327 0.338

Locus 6382 :

3 alleles

98.8% missing data

2	(0.308) 0.300 0.312

1	(0.461) 0.458 0.464

4	(0.230) 0.242 0.224

Locus 6383 :

2 alleles

96.1% missing data

3	(0.848) 0.858 0.845

4	(0.152) 0.142 0.155

Locus 6384 :

2 alleles

97.3% missing data

3	(0.562) 0.557 0.580

4	(0.438) 0.443 0.420

Locus 6385 :

2 alleles

99.2% missing data

3	(0.180) 0.178 0.181

2	(0.820) 0.822 0.819

Locus 6386 :

3 alleles

98.8% missing data

1	(0.371) 0.366 0.367

4	(0.311) 0.307 0.319

2	(0.317) 0.327 0.314

Locus 6387 :

3 alleles

98.8% missing data

1	(0.168) 0.165 0.171

3	(0.346) 0.343 0.344

4	(0.486) 0.491 0.485

Locus 6388 :

2 alleles

99.2% missing data

1	(0.470) 0.474 0.470

4	(0.530) 0.526 0.530

Locus 6389 :

2 alleles

99.2% missing data

2	(0.480) 0.485 0.477

4	(0.520) 0.515 0.523

Locus 6390 :

2 alleles

99.2% missing data

3	(0.662) 0.658 0.655

4	(0.338) 0.342 0.345

Locus 6391 :

2 alleles

99.2% missing data

4	(0.452) 0.461 0.452

1	(0.548) 0.539 0.548

Locus 6392 :

2 alleles

99.2% missing data

3	(0.325) 0.324 0.324

4	(0.675) 0.676 0.676

Locus 6393 :

2 alleles

95.3% missing data

1	(0.133) 0.133 0.129

3	(0.867) 0.867 0.871

Locus 6394 :

3 alleles

92.2% missing data

4	(0.064) 0.062 0.060

3	(0.869) 0.872 0.878

1	(0.067) 0.066 0.063

Locus 6395 :

2 alleles

99.2% missing data

4	(0.764) 0.761 0.767

2	(0.236) 0.239 0.233

Locus 6396 :

2 alleles

8.6% missing data

2	(0.990) 0.984 0.997

1	(0.010) 0.016 0.003

Locus 6397 :

2 alleles

95.7% missing data

1	(0.841) 0.837 0.850

3	(0.159) 0.163 0.150

Locus 6398 :

2 alleles

91.0% missing data

3	(0.890) 0.908 0.884

4	(0.110) 0.092 0.116

Locus 6399 :

2 alleles

98.8% missing data

1	(0.254) 0.247 0.254

3	(0.746) 0.753 0.746

Locus 6400 :

2 alleles

98.0% missing data

1	(0.768) 0.765 0.776

4	(0.232) 0.235 0.224

Locus 6401 :

2 alleles

88.7% missing data

1	(0.958) 0.951 0.968

3	(0.042) 0.049 0.032

Locus 6402 :

2 alleles

99.2% missing data

2	(0.605) 0.611 0.601

3	(0.395) 0.389 0.399

Locus 6403 :

2 alleles

99.2% missing data

1	(0.648) 0.645 0.648

2	(0.352) 0.355 0.352

Locus 6404 :

2 alleles

98.8% missing data

1	(0.308) 0.302 0.304

3	(0.692) 0.698 0.696

Locus 6405 :

2 alleles

90.2% missing data

3	(0.895) 0.910 0.892

4	(0.105) 0.090 0.108

Locus 6406 :

2 alleles

98.8% missing data

1	(0.669) 0.672 0.664

4	(0.331) 0.328 0.336

Locus 6407 :

2 alleles

99.2% missing data

4	(0.428) 0.420 0.429

1	(0.572) 0.580 0.571

Locus 6408 :

2 alleles

99.2% missing data

4	(0.410) 0.416 0.402

2	(0.590) 0.584 0.598

Locus 6409 :

2 alleles

98.4% missing data

3	(0.620) 0.627 0.614

4	(0.380) 0.373 0.386

Locus 6410 :

2 alleles

99.2% missing data

2	(0.579) 0.574 0.576

3	(0.421) 0.426 0.424

Locus 6411 :

3 alleles

98.8% missing data

1	(0.224) 0.218 0.232

4	(0.482) 0.485 0.484

3	(0.294) 0.298 0.285

Locus 6412 :

2 alleles

99.2% missing data

1	(0.521) 0.517 0.523

3	(0.479) 0.483 0.477

Locus 6413 :

2 alleles

99.2% missing data

2	(0.570) 0.558 0.576

1	(0.430) 0.442 0.424

Locus 6414 :

2 alleles

99.2% missing data

4	(0.523) 0.523 0.527

3	(0.477) 0.477 0.473

Locus 6415 :

2 alleles

98.0% missing data

3	(0.488) 0.496 0.469

4	(0.512) 0.504 0.531

Locus 6416 :

2 alleles

99.2% missing data

4	(0.717) 0.716 0.720

2	(0.283) 0.284 0.280

Locus 6417 :

2 alleles

99.2% missing data

3	(0.352) 0.352 0.357

1	(0.648) 0.648 0.643

Locus 6418 :

2 alleles

99.2% missing data

3	(0.409) 0.412 0.407

2	(0.591) 0.588 0.593

Locus 6419 :

2 alleles

99.2% missing data

3	(0.480) 0.484 0.480

2	(0.520) 0.516 0.520

Locus 6420 :

2 alleles

99.2% missing data

1	(0.383) 0.388 0.383

4	(0.617) 0.612 0.617

Locus 6421 :

2 alleles

96.1% missing data

4	(0.798) 0.807 0.793

3	(0.202) 0.193 0.207

Locus 6422 :

2 alleles

98.4% missing data

4	(0.674) 0.675 0.677

2	(0.326) 0.325 0.323

Locus 6423 :

2 alleles

98.4% missing data

4	(0.522) 0.506 0.532

1	(0.478) 0.494 0.468

Locus 6424 :

2 alleles

99.2% missing data

2	(0.447) 0.451 0.448

4	(0.553) 0.549 0.552

Locus 6425 :

2 alleles

99.2% missing data

3	(0.620) 0.612 0.629

4	(0.380) 0.388 0.371

Locus 6426 :

2 alleles

8.2% missing data

3	(0.984) 0.977 0.995

1	(0.016) 0.023 0.005

Locus 6427 :

2 alleles

99.2% missing data

2	(0.684) 0.682 0.692

4	(0.316) 0.318 0.308

Locus 6428 :

2 alleles

99.2% missing data

1	(0.180) 0.174 0.187

3	(0.820) 0.826 0.813

Locus 6429 :

2 alleles

99.2% missing data

1	(0.737) 0.727 0.742

3	(0.263) 0.273 0.258

Locus 6430 :

2 alleles

99.2% missing data

4	(0.665) 0.660 0.677

3	(0.335) 0.340 0.323

Locus 6431 :

2 alleles

90.6% missing data

3	(0.149) 0.123 0.167

2	(0.851) 0.877 0.833

Locus 6432 :

2 alleles

97.3% missing data

4	(0.165) 0.172 0.153

1	(0.835) 0.828 0.847

Locus 6433 :

2 alleles

93.8% missing data

3	(0.818) 0.816 0.834

2	(0.182) 0.184 0.166

Locus 6434 :

2 alleles

99.2% missing data

4	(0.260) 0.261 0.264

2	(0.740) 0.739 0.736

Locus 6435 :

2 alleles

99.2% missing data

4	(0.336) 0.335 0.335

2	(0.664) 0.665 0.665

Locus 6436 :

2 alleles

98.0% missing data

2	(0.726) 0.721 0.739

3	(0.274) 0.279 0.261

Locus 6437 :

2 alleles

99.2% missing data

3	(0.518) 0.512 0.517

4	(0.482) 0.488 0.483

Locus 6438 :

2 alleles

96.1% missing data

4	(0.847) 0.849 0.845

1	(0.153) 0.151 0.155

Locus 6439 :

2 alleles

99.2% missing data

3	(0.444) 0.433 0.451

2	(0.556) 0.567 0.549

Locus 6440 :

2 alleles

99.2% missing data

4	(0.173) 0.177 0.170

2	(0.827) 0.823 0.830

Locus 6441 :

2 alleles

98.8% missing data

4	(0.683) 0.693 0.678

1	(0.317) 0.307 0.322

Locus 6442 :

2 alleles

97.7% missing data

4	(0.633) 0.618 0.646

2	(0.367) 0.382 0.354

Locus 6443 :

2 alleles

96.1% missing data

2	(0.837) 0.845 0.833

4	(0.163) 0.155 0.167

Locus 6444 :

2 alleles

99.2% missing data

3	(0.214) 0.214 0.214

2	(0.786) 0.786 0.786

Locus 6445 :

2 alleles

99.2% missing data

3	(0.740) 0.734 0.744

1	(0.260) 0.266 0.256

Locus 6446 :

2 alleles

99.2% missing data

2	(0.427) 0.428 0.424

3	(0.573) 0.572 0.576

Locus 6447 :

2 alleles

99.2% missing data

3	(0.533) 0.532 0.537

1	(0.467) 0.468 0.463

Locus 6448 :

2 alleles

99.2% missing data

3	(0.818) 0.819 0.813

4	(0.182) 0.181 0.187

Locus 6449 :

2 alleles

99.2% missing data

2	(0.384) 0.386 0.393

1	(0.616) 0.614 0.607

Locus 6450 :

2 alleles

98.0% missing data

3	(0.459) 0.448 0.465

4	(0.541) 0.552 0.535

Locus 6451 :

2 alleles

99.2% missing data

4	(0.548) 0.545 0.549

1	(0.452) 0.455 0.451

Locus 6452 :

2 alleles

97.7% missing data

4	(0.290) 0.291 0.282

3	(0.710) 0.709 0.718

Locus 6453 :

2 alleles

97.3% missing data

1	(0.761) 0.760 0.765

2	(0.239) 0.240 0.235

Locus 6454 :

2 alleles

99.2% missing data

3	(0.325) 0.327 0.323

4	(0.675) 0.673 0.677

Locus 6455 :

2 alleles

99.2% missing data

1	(0.678) 0.685 0.677

3	(0.322) 0.315 0.323

Locus 6456 :

2 alleles

98.0% missing data

2	(0.813) 0.814 0.817

3	(0.187) 0.186 0.183

Locus 6457 :

2 alleles

87.9% missing data

4	(0.725) 0.737 0.713

2	(0.275) 0.263 0.287

Locus 6458 :

2 alleles

98.8% missing data

4	(0.275) 0.272 0.285

1	(0.725) 0.728 0.715

Locus 6459 :

2 alleles

99.2% missing data

2	(0.683) 0.685 0.674

4	(0.317) 0.315 0.326

Locus 6460 :

2 alleles

99.2% missing data

4	(0.529) 0.522 0.533

3	(0.471) 0.478 0.467

Locus 6461 :

2 alleles

99.2% missing data

4	(0.380) 0.378 0.379

2	(0.620) 0.622 0.621

Locus 6462 :

2 alleles

93.0% missing data

2	(0.912) 0.902 0.924

3	(0.088) 0.098 0.076

Locus 6463 :

2 alleles

98.8% missing data

2	(0.371) 0.363 0.371

4	(0.629) 0.637 0.629

Locus 6464 :

2 alleles

99.2% missing data

3	(0.256) 0.251 0.259

1	(0.744) 0.749 0.741

Locus 6465 :

2 alleles

8.2% missing data

4	(0.980) 0.973 0.994

1	(0.020) 0.027 0.006

Locus 6466 :

2 alleles

98.0% missing data

2	(0.731) 0.729 0.742

1	(0.269) 0.271 0.258

Locus 6467 :

2 alleles

8.2% missing data

1	(0.974) 0.939 0.992

2	(0.026) 0.061 0.008

Locus 6468 :

2 alleles

99.2% missing data

2	(0.551) 0.545 0.552

3	(0.449) 0.455 0.448

Locus 6469 :

3 alleles

98.4% missing data

3	(0.220) 0.220 0.220

2	(0.606) 0.606 0.602

4	(0.173) 0.174 0.178

Locus 6470 :

2 alleles

99.2% missing data

3	(0.506) 0.505 0.509

2	(0.494) 0.495 0.491

Locus 6471 :

2 alleles

98.8% missing data

2	(0.549) 0.553 0.543

3	(0.451) 0.447 0.457

Locus 6472 :

2 alleles

92.6% missing data

4	(0.891) 0.884 0.908

1	(0.109) 0.116 0.092

Locus 6473 :

3 alleles

98.8% missing data

2	(0.421) 0.421 0.417

4	(0.288) 0.287 0.293

3	(0.291) 0.292 0.290

Locus 6474 :

2 alleles

99.2% missing data

4	(0.358) 0.356 0.362

1	(0.642) 0.644 0.638

Locus 6475 :

2 alleles

99.2% missing data

3	(0.570) 0.573 0.570

1	(0.430) 0.427 0.430

Locus 6476 :

2 alleles

97.7% missing data

4	(0.789) 0.780 0.797

3	(0.211) 0.220 0.203

Locus 6477 :

2 alleles

99.2% missing data

4	(0.344) 0.346 0.342

2	(0.656) 0.654 0.658

Locus 6478 :

2 alleles

91.4% missing data

1	(0.929) 0.933 0.932

3	(0.071) 0.067 0.068

Locus 6479 :

2 alleles

99.2% missing data

1	(0.665) 0.663 0.662

4	(0.335) 0.337 0.338

Locus 6480 :

2 alleles

99.2% missing data

3	(0.460) 0.458 0.461

1	(0.540) 0.542 0.539

Locus 6481 :

2 alleles

98.4% missing data

3	(0.473) 0.482 0.472

2	(0.527) 0.518 0.528

Locus 6482 :

2 alleles

96.1% missing data

1	(0.261) 0.243 0.270

3	(0.739) 0.757 0.730

Locus 6483 :

2 alleles

99.2% missing data

3	(0.526) 0.535 0.527

1	(0.474) 0.465 0.473

Locus 6484 :

2 alleles

0.0% missing data

1	(0.986) 0.981 0.996

3	(0.014) 0.019 0.004

Locus 6485 :

2 alleles

99.2% missing data

1	(0.635) 0.634 0.642

2	(0.365) 0.366 0.358

Locus 6486 :

2 alleles

99.2% missing data

1	(0.627) 0.629 0.625

3	(0.373) 0.371 0.375

Locus 6487 :

3 alleles

98.0% missing data

2	(0.577) 0.574 0.579

4	(0.203) 0.203 0.205

3	(0.220) 0.223 0.216

Locus 6488 :

2 alleles

99.2% missing data

2	(0.669) 0.673 0.667

3	(0.331) 0.327 0.333

Locus 6489 :

2 alleles

99.2% missing data

4	(0.173) 0.170 0.171

1	(0.827) 0.830 0.829

Locus 6490 :

2 alleles

96.5% missing data

4	(0.833) 0.845 0.820

2	(0.167) 0.155 0.180

Locus 6491 :

3 alleles

96.9% missing data

4	(0.340) 0.348 0.330

3	(0.456) 0.461 0.460

1	(0.204) 0.191 0.210

Locus 6492 :

2 alleles

99.2% missing data

3	(0.822) 0.816 0.824

1	(0.178) 0.184 0.176

Locus 6493 :

2 alleles

99.2% missing data

1	(0.762) 0.765 0.756

3	(0.238) 0.235 0.244

Locus 6494 :

2 alleles

98.8% missing data

2	(0.606) 0.604 0.610

1	(0.394) 0.396 0.390

Locus 6495 :

2 alleles

97.7% missing data

2	(0.443) 0.441 0.430

3	(0.557) 0.559 0.570

Locus 6496 :

2 alleles

8.2% missing data

2	(0.975) 0.960 0.992

4	(0.025) 0.040 0.008

Locus 6497 :

2 alleles

99.2% missing data

2	(0.492) 0.490 0.494

1	(0.508) 0.510 0.506

Locus 6498 :

2 alleles

94.9% missing data

3	(0.145) 0.137 0.144

2	(0.855) 0.863 0.856

Locus 6499 :

3 alleles

98.8% missing data

1	(0.432) 0.420 0.434

2	(0.194) 0.191 0.203

4	(0.373) 0.389 0.363

Locus 6500 :

2 alleles

93.8% missing data

3	(0.938) 0.928 0.950

1	(0.062) 0.072 0.050

Locus 6501 :

2 alleles

99.2% missing data

3	(0.570) 0.569 0.570

1	(0.430) 0.431 0.430

Locus 6502 :

2 alleles

98.8% missing data

3	(0.717) 0.718 0.717

4	(0.283) 0.282 0.283

Locus 6503 :

3 alleles

92.2% missing data

2	(0.852) 0.874 0.835

3	(0.080) 0.067 0.089

1	(0.068) 0.059 0.076

Locus 6504 :

2 alleles

99.2% missing data

4	(0.410) 0.411 0.405

2	(0.590) 0.589 0.595

Locus 6505 :

2 alleles

97.7% missing data

2	(0.256) 0.250 0.254

3	(0.744) 0.750 0.746

Locus 6506 :

2 alleles

14.8% missing data

4	(0.985) 0.986 0.992

1	(0.015) 0.014 0.008

Locus 6507 :

2 alleles

90.6% missing data

3	(0.843) 0.849 0.839

4	(0.157) 0.151 0.161

Locus 6508 :

2 alleles

97.7% missing data

4	(0.379) 0.371 0.385

1	(0.621) 0.629 0.615

Locus 6509 :

2 alleles

99.2% missing data

1	(0.405) 0.397 0.404

2	(0.595) 0.603 0.596

Locus 6510 :

2 alleles

99.2% missing data

1	(0.546) 0.537 0.544

3	(0.454) 0.463 0.456

Locus 6511 :

2 alleles

99.2% missing data

4	(0.727) 0.734 0.722

2	(0.273) 0.266 0.278

Locus 6512 :

2 alleles

8.2% missing data

3	(0.983) 0.966 0.994

1	(0.017) 0.034 0.006

Locus 6513 :

2 alleles

99.2% missing data

4	(0.820) 0.813 0.827

1	(0.180) 0.187 0.173

Locus 6514 :

2 alleles

96.5% missing data

1	(0.406) 0.420 0.383

2	(0.594) 0.580 0.617

Locus 6515 :

2 alleles

99.2% missing data

4	(0.449) 0.441 0.453

1	(0.551) 0.559 0.547

Locus 6516 :

2 alleles

99.2% missing data

1	(0.552) 0.551 0.552

4	(0.448) 0.449 0.448

Locus 6517 :

2 alleles

99.2% missing data

1	(0.297) 0.306 0.292

2	(0.703) 0.694 0.708

Locus 6518 :

2 alleles

98.8% missing data

3	(0.291) 0.287 0.296

2	(0.709) 0.713 0.704

Locus 6519 :

2 alleles

97.3% missing data

4	(0.717) 0.723 0.721

1	(0.283) 0.277 0.279

Locus 6520 :

2 alleles

99.2% missing data

2	(0.281) 0.287 0.274

4	(0.719) 0.713 0.726

Locus 6521 :

2 alleles

99.2% missing data

4	(0.488) 0.478 0.495

3	(0.512) 0.522 0.505

Locus 6522 :

2 alleles

91.0% missing data

1	(0.890) 0.909 0.879

2	(0.110) 0.091 0.121

Locus 6523 :

2 alleles

99.2% missing data

1	(0.728) 0.727 0.725

2	(0.272) 0.273 0.275

Locus 6524 :

2 alleles

99.2% missing data

3	(0.475) 0.471 0.477

1	(0.525) 0.529 0.523

Locus 6525 :

2 alleles

99.2% missing data

3	(0.533) 0.522 0.537

1	(0.467) 0.478 0.463

Locus 6526 :

3 alleles

98.4% missing data

3	(0.485) 0.480 0.489

4	(0.227) 0.233 0.223

1	(0.288) 0.287 0.288

Locus 6527 :

2 alleles

97.7% missing data

4	(0.799) 0.790 0.813

2	(0.201) 0.210 0.187

Locus 6528 :

2 alleles

99.2% missing data

2	(0.496) 0.497 0.493

3	(0.504) 0.503 0.507

Locus 6529 :

2 alleles

99.2% missing data

2	(0.424) 0.428 0.424

1	(0.576) 0.572 0.576

Locus 6530 :

3 alleles

95.7% missing data

4	(0.262) 0.246 0.264

1	(0.582) 0.609 0.577

3	(0.156) 0.146 0.158

Locus 6531 :

2 alleles

98.4% missing data

3	(0.666) 0.674 0.660

2	(0.334) 0.326 0.340

Locus 6532 :

2 alleles

99.2% missing data

2	(0.585) 0.583 0.585

3	(0.415) 0.417 0.415

Locus 6533 :

2 alleles

99.2% missing data

3	(0.328) 0.332 0.329

1	(0.672) 0.668 0.671

Locus 6534 :

2 alleles

99.2% missing data

4	(0.226) 0.225 0.233

1	(0.774) 0.775 0.767

Locus 6535 :

2 alleles

99.2% missing data

2	(0.362) 0.365 0.367

3	(0.638) 0.635 0.633

Locus 6536 :

2 alleles

98.8% missing data

2	(0.319) 0.309 0.323

3	(0.681) 0.691 0.677

Locus 6537 :

3 alleles

98.8% missing data

2	(0.385) 0.379 0.383

1	(0.425) 0.426 0.427

4	(0.189) 0.195 0.190

Locus 6538 :

2 alleles

99.2% missing data

4	(0.678) 0.679 0.682

3	(0.322) 0.321 0.318

Locus 6539 :

2 alleles

98.8% missing data

3	(0.536) 0.532 0.547

1	(0.464) 0.468 0.453

Locus 6540 :

2 alleles

0.8% missing data

4	(0.933) 0.782 0.980

2	(0.067) 0.218 0.020

Locus 6541 :

2 alleles

99.2% missing data

1	(0.660) 0.653 0.665

3	(0.340) 0.347 0.335

Locus 6542 :

2 alleles

99.2% missing data

1	(0.287) 0.278 0.290

4	(0.713) 0.722 0.710

Locus 6543 :

2 alleles

99.2% missing data

1	(0.612) 0.606 0.617

2	(0.388) 0.394 0.383

Locus 6544 :

2 alleles

8.2% missing data

2	(0.989) 0.981 0.997

1	(0.011) 0.019 0.003

Locus 6545 :

2 alleles

99.2% missing data

3	(0.367) 0.360 0.372

1	(0.633) 0.640 0.628

Locus 6546 :

2 alleles

88.7% missing data

2	(0.461) 0.515 0.407

1	(0.539) 0.485 0.593

Locus 6547 :

2 alleles

98.0% missing data

1	(0.753) 0.756 0.750

2	(0.247) 0.244 0.250

Locus 6548 :

2 alleles

96.1% missing data

3	(0.255) 0.243 0.267

2	(0.745) 0.757 0.733

Locus 6549 :

2 alleles

99.2% missing data

1	(0.494) 0.489 0.499

2	(0.506) 0.511 0.501

Locus 6550 :

2 alleles

97.3% missing data

2	(0.776) 0.771 0.784

3	(0.224) 0.229 0.216

Locus 6551 :

2 alleles

99.2% missing data

1	(0.383) 0.380 0.389

3	(0.617) 0.620 0.611

Locus 6552 :

2 alleles

98.4% missing data

2	(0.664) 0.661 0.664

1	(0.336) 0.339 0.336

Locus 6553 :

3 alleles

96.5% missing data

1	(0.225) 0.225 0.226

2	(0.687) 0.686 0.686

3	(0.088) 0.089 0.088

Locus 6554 :

2 alleles

99.2% missing data

4	(0.451) 0.456 0.443

1	(0.549) 0.544 0.557

Locus 6555 :

2 alleles

99.2% missing data

1	(0.328) 0.335 0.325

3	(0.672) 0.665 0.675

Locus 6556 :

2 alleles

99.2% missing data

2	(0.614) 0.610 0.614

3	(0.386) 0.390 0.386

Locus 6557 :

2 alleles

99.2% missing data

4	(0.656) 0.661 0.652

3	(0.344) 0.339 0.348

Locus 6558 :

2 alleles

99.2% missing data

3	(0.292) 0.297 0.297

1	(0.708) 0.703 0.703

Locus 6559 :

2 alleles

99.2% missing data

2	(0.292) 0.292 0.300

1	(0.708) 0.708 0.700

Locus 6560 :

2 alleles

99.2% missing data

3	(0.696) 0.697 0.694

2	(0.304) 0.303 0.306

Locus 6561 :

2 alleles

99.2% missing data

3	(0.644) 0.643 0.642

2	(0.356) 0.357 0.358

Locus 6562 :

2 alleles

99.2% missing data

4	(0.601) 0.598 0.602

2	(0.399) 0.402 0.398

Locus 6563 :

2 alleles

99.2% missing data

1	(0.625) 0.626 0.625

2	(0.375) 0.374 0.375

Locus 6564 :

3 alleles

98.8% missing data

2	(0.439) 0.445 0.432

3	(0.199) 0.193 0.200

1	(0.362) 0.362 0.369

Locus 6565 :

2 alleles

98.8% missing data

4	(0.529) 0.532 0.521

2	(0.471) 0.468 0.479

Locus 6566 :

2 alleles

99.2% missing data

4	(0.131) 0.129 0.138

2	(0.869) 0.871 0.862

Locus 6567 :

2 alleles

99.2% missing data

1	(0.338) 0.341 0.340

2	(0.662) 0.659 0.660

Locus 6568 :

2 alleles

99.2% missing data

4	(0.754) 0.755 0.746

2	(0.246) 0.245 0.254

Locus 6569 :

2 alleles

8.6% missing data

2	(0.984) 0.984 0.992

3	(0.016) 0.016 0.008

Locus 6570 :

2 alleles

98.8% missing data

1	(0.463) 0.464 0.462

4	(0.537) 0.536 0.538

Locus 6571 :

2 alleles

96.1% missing data

3	(0.826) 0.840 0.818

1	(0.174) 0.160 0.182

Locus 6572 :

2 alleles

99.2% missing data

2	(0.352) 0.349 0.357

1	(0.648) 0.651 0.643

Locus 6573 :

2 alleles

99.2% missing data

3	(0.664) 0.661 0.669

4	(0.336) 0.339 0.331

Locus 6574 :

2 alleles

99.2% missing data

3	(0.682) 0.675 0.687

2	(0.318) 0.325 0.313

Locus 6575 :

2 alleles

99.2% missing data

3	(0.501) 0.501 0.499

4	(0.499) 0.499 0.501

Locus 6576 :

2 alleles

97.7% missing data

4	(0.777) 0.772 0.790

1	(0.223) 0.228 0.210

Locus 6577 :

2 alleles

99.2% missing data

3	(0.733) 0.735 0.735

1	(0.267) 0.265 0.265

Locus 6578 :

2 alleles

99.2% missing data

1	(0.406) 0.411 0.401

2	(0.594) 0.589 0.599

Locus 6579 :

2 alleles

98.8% missing data

4	(0.438) 0.438 0.446

3	(0.562) 0.562 0.554

Locus 6580 :

2 alleles

98.0% missing data

1	(0.856) 0.857 0.856

2	(0.144) 0.143 0.144

Locus 6581 :

2 alleles

98.4% missing data

4	(0.632) 0.632 0.631

2	(0.368) 0.368 0.369

Locus 6582 :

3 alleles

98.8% missing data

1	(0.203) 0.197 0.206

4	(0.199) 0.192 0.204

3	(0.597) 0.611 0.590

Locus 6583 :

2 alleles

99.2% missing data

3	(0.416) 0.414 0.417

4	(0.584) 0.586 0.583

Locus 6584 :

2 alleles

97.3% missing data

2	(0.151) 0.153 0.150

1	(0.849) 0.847 0.850

Locus 6585 :

2 alleles

99.2% missing data

3	(0.385) 0.385 0.387

1	(0.615) 0.615 0.613

Locus 6586 :

2 alleles

98.8% missing data

3	(0.730) 0.727 0.733

4	(0.270) 0.273 0.267

Locus 6587 :

2 alleles

99.2% missing data

1	(0.524) 0.525 0.526

2	(0.476) 0.475 0.474

Locus 6588 :

2 alleles

99.2% missing data

3	(0.250) 0.249 0.255

2	(0.750) 0.751 0.745

Locus 6589 :

2 alleles

99.2% missing data

4	(0.522) 0.526 0.525

2	(0.478) 0.474 0.475

Locus 6590 :

2 alleles

99.2% missing data

1	(0.616) 0.614 0.611

4	(0.384) 0.386 0.389

Locus 6591 :

2 alleles

99.2% missing data

2	(0.487) 0.483 0.491

3	(0.513) 0.517 0.509

Locus 6592 :

2 alleles

98.4% missing data

4	(0.783) 0.787 0.777

1	(0.217) 0.213 0.223

Locus 6593 :

2 alleles

99.2% missing data

4	(0.565) 0.565 0.566

2	(0.435) 0.435 0.434

Locus 6594 :

2 alleles

99.2% missing data

1	(0.470) 0.469 0.470

3	(0.530) 0.531 0.530

Locus 6595 :

2 alleles

99.2% missing data

4	(0.643) 0.641 0.640

1	(0.357) 0.359 0.360

Locus 6596 :

3 alleles

98.8% missing data

3	(0.416) 0.414 0.419

2	(0.284) 0.279 0.286

4	(0.300) 0.307 0.295

Locus 6597 :

2 alleles

98.8% missing data

3	(0.584) 0.580 0.582

4	(0.416) 0.420 0.418

Locus 6598 :

2 alleles

99.2% missing data

1	(0.426) 0.423 0.424

2	(0.574) 0.577 0.576

Locus 6599 :

2 alleles

99.2% missing data

3	(0.697) 0.702 0.697

1	(0.303) 0.298 0.303

Locus 6600 :

2 alleles

96.1% missing data

1	(0.128) 0.124 0.131

2	(0.872) 0.876 0.869

Locus 6601 :

2 alleles

98.8% missing data

1	(0.563) 0.561 0.558

3	(0.437) 0.439 0.442

Locus 6602 :

3 alleles

98.4% missing data

2	(0.279) 0.286 0.275

4	(0.597) 0.597 0.592

1	(0.124) 0.118 0.132

Locus 6603 :

2 alleles

98.8% missing data

3	(0.490) 0.488 0.494

2	(0.510) 0.512 0.506

Locus 6604 :

2 alleles

99.2% missing data

3	(0.577) 0.569 0.584

1	(0.423) 0.431 0.416

Locus 6605 :

2 alleles

99.2% missing data

1	(0.423) 0.423 0.422

2	(0.577) 0.577 0.578

Locus 6606 :

2 alleles

98.8% missing data

4	(0.725) 0.726 0.730

2	(0.275) 0.274 0.270

Locus 6607 :

2 alleles

99.2% missing data

2	(0.394) 0.393 0.394

3	(0.606) 0.607 0.606

Locus 6608 :

2 alleles

98.8% missing data

4	(0.571) 0.578 0.565

1	(0.429) 0.422 0.435

Locus 6609 :

2 alleles

98.4% missing data

3	(0.277) 0.269 0.287

2	(0.723) 0.731 0.713

Locus 6610 :

2 alleles

99.2% missing data

4	(0.368) 0.373 0.370

3	(0.632) 0.627 0.630

Locus 6611 :

2 alleles

0.4% missing data

3	(0.986) 0.989 0.993

2	(0.014) 0.011 0.007

Locus 6612 :

2 alleles

98.8% missing data

2	(0.389) 0.382 0.404

1	(0.611) 0.618 0.596

Locus 6613 :

2 alleles

99.2% missing data

4	(0.505) 0.506 0.500

3	(0.495) 0.494 0.500

Locus 6614 :

2 alleles

15.6% missing data

1	(0.983) 0.977 0.995

3	(0.017) 0.023 0.005

Locus 6615 :

2 alleles

98.8% missing data

3	(0.478) 0.487 0.484

1	(0.522) 0.513 0.516

Locus 6616 :

2 alleles

99.2% missing data

3	(0.465) 0.460 0.463

2	(0.535) 0.540 0.537

Locus 6617 :

2 alleles

97.7% missing data

3	(0.797) 0.785 0.806

2	(0.203) 0.215 0.194

Locus 6618 :

2 alleles

99.2% missing data

4	(0.230) 0.228 0.229

1	(0.770) 0.772 0.771

Locus 6619 :

2 alleles

1.2% missing data

3	(0.987) 0.982 0.996

2	(0.013) 0.018 0.004

Locus 6620 :

2 alleles

99.2% missing data

4	(0.690) 0.693 0.695

1	(0.310) 0.307 0.305

Locus 6621 :

2 alleles

99.2% missing data

4	(0.418) 0.422 0.423

2	(0.582) 0.578 0.577

Locus 6622 :

3 alleles

98.4% missing data

1	(0.415) 0.413 0.423

3	(0.338) 0.345 0.331

2	(0.247) 0.242 0.246

Locus 6623 :

2 alleles

99.2% missing data

4	(0.323) 0.317 0.330

1	(0.677) 0.683 0.670

Locus 6624 :

2 alleles

99.2% missing data

2	(0.303) 0.299 0.312

4	(0.697) 0.701 0.688

Locus 6625 :

2 alleles

99.2% missing data

2	(0.695) 0.696 0.689

3	(0.305) 0.304 0.311

Locus 6626 :

3 alleles

98.4% missing data

2	(0.346) 0.336 0.353

4	(0.256) 0.263 0.258

3	(0.397) 0.402 0.389

Locus 6627 :

2 alleles

96.9% missing data

1	(0.785) 0.775 0.800

2	(0.215) 0.225 0.200

Locus 6628 :

3 alleles

98.0% missing data

2	(0.354) 0.358 0.351

3	(0.542) 0.539 0.539

4	(0.103) 0.103 0.110

Locus 6629 :

2 alleles

99.2% missing data

2	(0.231) 0.235 0.226

4	(0.769) 0.765 0.774

Locus 6630 :

2 alleles

99.2% missing data

4	(0.252) 0.244 0.261

1	(0.748) 0.756 0.739

Locus 6631 :

2 alleles

99.2% missing data

2	(0.203) 0.210 0.198

4	(0.797) 0.790 0.802

Locus 6632 :

2 alleles

99.2% missing data

3	(0.564) 0.563 0.561

4	(0.436) 0.437 0.439

Locus 6633 :

2 alleles

98.4% missing data

4	(0.805) 0.806 0.805

2	(0.195) 0.194 0.195

Locus 6634 :

2 alleles

10.2% missing data

3	(0.982) 0.971 0.994

4	(0.018) 0.029 0.006

Locus 6635 :

2 alleles

99.2% missing data

4	(0.583) 0.583 0.586

1	(0.417) 0.417 0.414

Locus 6636 :

2 alleles

99.2% missing data

4	(0.622) 0.617 0.624

1	(0.378) 0.383 0.376

Locus 6637 :

2 alleles

98.8% missing data

2	(0.597) 0.598 0.601

1	(0.403) 0.402 0.399

Locus 6638 :

2 alleles

99.2% missing data

1	(0.435) 0.438 0.442

2	(0.565) 0.562 0.558

Locus 6639 :

2 alleles

99.2% missing data

1	(0.266) 0.262 0.262

4	(0.734) 0.738 0.738

Locus 6640 :

2 alleles

98.8% missing data

4	(0.336) 0.342 0.338

2	(0.664) 0.658 0.662

Locus 6641 :

2 alleles

97.3% missing data

1	(0.754) 0.754 0.758

2	(0.246) 0.246 0.242

Locus 6642 :

2 alleles

8.6% missing data

3	(0.985) 0.977 0.996

2	(0.015) 0.023 0.004

Locus 6643 :

2 alleles

99.2% missing data

4	(0.653) 0.660 0.652

2	(0.347) 0.340 0.348

Locus 6644 :

2 alleles

98.8% missing data

1	(0.574) 0.567 0.588

3	(0.426) 0.433 0.412

Locus 6645 :

2 alleles

99.2% missing data

4	(0.451) 0.448 0.455

3	(0.549) 0.552 0.545

Locus 6646 :

2 alleles

99.2% missing data

4	(0.718) 0.721 0.710

2	(0.282) 0.279 0.290

Locus 6647 :

2 alleles

99.2% missing data

4	(0.646) 0.638 0.644

1	(0.354) 0.362 0.356

Locus 6648 :

2 alleles

99.2% missing data

4	(0.423) 0.423 0.422

2	(0.577) 0.577 0.578

Locus 6649 :

2 alleles

98.0% missing data

4	(0.419) 0.404 0.420

1	(0.581) 0.596 0.580

Locus 6650 :

2 alleles

98.4% missing data

1	(0.603) 0.602 0.609

3	(0.397) 0.398 0.391

Locus 6651 :

3 alleles

96.1% missing data

1	(0.175) 0.177 0.169

4	(0.281) 0.282 0.274

2	(0.544) 0.541 0.557

Locus 6652 :

2 alleles

98.4% missing data

4	(0.430) 0.418 0.440

3	(0.570) 0.582 0.560

Locus 6653 :

2 alleles

99.2% missing data

3	(0.470) 0.478 0.463

2	(0.530) 0.522 0.537

Locus 6654 :

3 alleles

98.8% missing data

4	(0.328) 0.324 0.333

1	(0.368) 0.374 0.366

2	(0.304) 0.302 0.302

Locus 6655 :

2 alleles

98.0% missing data

2	(0.274) 0.275 0.268

3	(0.726) 0.725 0.732

Locus 6656 :

2 alleles

99.2% missing data

2	(0.531) 0.536 0.525

4	(0.469) 0.464 0.475

Locus 6657 :

2 alleles

99.2% missing data

3	(0.499) 0.499 0.502

2	(0.501) 0.501 0.498

Locus 6658 :

2 alleles

99.2% missing data

4	(0.419) 0.418 0.416

3	(0.581) 0.582 0.584

Locus 6659 :

3 alleles

98.8% missing data

1	(0.190) 0.187 0.196

4	(0.301) 0.298 0.302

3	(0.508) 0.515 0.502

Locus 6660 :

2 alleles

99.2% missing data

2	(0.345) 0.344 0.345

4	(0.655) 0.656 0.655

Locus 6661 :

2 alleles

99.2% missing data

1	(0.444) 0.446 0.440

4	(0.556) 0.554 0.560

Locus 6662 :

2 alleles

99.2% missing data

2	(0.598) 0.597 0.599

3	(0.402) 0.403 0.401

Locus 6663 :

2 alleles

99.2% missing data

4	(0.687) 0.683 0.691

1	(0.313) 0.317 0.309

Locus 6664 :

2 alleles

98.8% missing data

4	(0.494) 0.495 0.493

3	(0.506) 0.505 0.507

Locus 6665 :

2 alleles

99.2% missing data

3	(0.443) 0.437 0.439

1	(0.557) 0.563 0.561

Locus 6666 :

2 alleles

96.1% missing data

4	(0.187) 0.178 0.194

2	(0.813) 0.822 0.806

Locus 6667 :

2 alleles

99.2% missing data

4	(0.253) 0.251 0.257

2	(0.747) 0.749 0.743

Locus 6668 :

2 alleles

99.2% missing data

4	(0.859) 0.862 0.850

2	(0.141) 0.138 0.150

Locus 6669 :

2 alleles

99.2% missing data

4	(0.443) 0.438 0.448

3	(0.557) 0.562 0.552

Locus 6670 :

2 alleles

99.2% missing data

4	(0.397) 0.398 0.400

1	(0.603) 0.602 0.600

Locus 6671 :

2 alleles

99.2% missing data

4	(0.452) 0.448 0.457

3	(0.548) 0.552 0.543

Locus 6672 :

2 alleles

98.8% missing data

4	(0.468) 0.468 0.478

2	(0.532) 0.532 0.522

Locus 6673 :

2 alleles

99.2% missing data

2	(0.323) 0.316 0.323

3	(0.677) 0.684 0.677

Locus 6674 :

2 alleles

99.2% missing data

2	(0.299) 0.301 0.296

4	(0.701) 0.699 0.704

Locus 6675 :

2 alleles

7.8% missing data

2	(0.986) 0.983 0.996

4	(0.014) 0.017 0.004

Locus 6676 :

3 alleles

94.9% missing data

4	(0.695) 0.680 0.718

2	(0.149) 0.156 0.141

1	(0.156) 0.164 0.141

Locus 6677 :

2 alleles

98.8% missing data

1	(0.199) 0.198 0.204

4	(0.801) 0.802 0.796

Locus 6678 :

2 alleles

99.2% missing data

1	(0.415) 0.411 0.418

4	(0.585) 0.589 0.582

Locus 6679 :

2 alleles

99.2% missing data

2	(0.690) 0.690 0.689

4	(0.310) 0.310 0.311

Locus 6680 :

2 alleles

90.6% missing data

4	(0.919) 0.936 0.908

3	(0.081) 0.064 0.092

Locus 6681 :

2 alleles

99.2% missing data

1	(0.425) 0.420 0.426

4	(0.575) 0.580 0.574

Locus 6682 :

2 alleles

99.2% missing data

3	(0.564) 0.565 0.560

1	(0.436) 0.435 0.440

Locus 6683 :

2 alleles

99.2% missing data

2	(0.431) 0.430 0.432

4	(0.569) 0.570 0.568

Locus 6684 :

2 alleles

99.2% missing data

4	(0.444) 0.440 0.452

3	(0.556) 0.560 0.548

Locus 6685 :

2 alleles

99.2% missing data

3	(0.437) 0.437 0.438

1	(0.563) 0.563 0.562

Locus 6686 :

2 alleles

99.2% missing data

3	(0.687) 0.690 0.679

2	(0.313) 0.310 0.321

Locus 6687 :

2 alleles

98.0% missing data

4	(0.828) 0.836 0.823

2	(0.172) 0.164 0.177

Locus 6688 :

2 alleles

93.4% missing data

3	(0.875) 0.876 0.877

4	(0.125) 0.124 0.123

Locus 6689 :

2 alleles

99.2% missing data

1	(0.551) 0.554 0.551

4	(0.449) 0.446 0.449

Locus 6690 :

2 alleles

0.8% missing data

4	(0.979) 0.984 0.985

1	(0.021) 0.016 0.015

Locus 6691 :

2 alleles

91.0% missing data

3	(0.914) 0.931 0.904

4	(0.086) 0.069 0.096

Locus 6692 :

2 alleles

98.8% missing data

4	(0.413) 0.405 0.413

3	(0.587) 0.595 0.587

Locus 6693 :

2 alleles

98.4% missing data

1	(0.637) 0.639 0.623

4	(0.363) 0.361 0.377

Locus 6694 :

2 alleles

98.8% missing data

1	(0.541) 0.540 0.545

2	(0.459) 0.460 0.455

Locus 6695 :

2 alleles

99.2% missing data

4	(0.643) 0.651 0.636

3	(0.357) 0.349 0.364

Locus 6696 :

2 alleles

99.2% missing data

3	(0.597) 0.599 0.600

4	(0.403) 0.401 0.400

Locus 6697 :

2 alleles

99.2% missing data

1	(0.281) 0.282 0.280

4	(0.719) 0.718 0.720

Locus 6698 :

2 alleles

99.2% missing data

3	(0.372) 0.369 0.372

2	(0.628) 0.631 0.628

Locus 6699 :

2 alleles

98.0% missing data

3	(0.681) 0.678 0.690

2	(0.319) 0.322 0.310

Locus 6700 :

2 alleles

94.9% missing data

3	(0.114) 0.112 0.111

4	(0.886) 0.888 0.889

Locus 6701 :

2 alleles

99.2% missing data

4	(0.783) 0.776 0.788

1	(0.217) 0.224 0.212

Locus 6702 :

2 alleles

99.2% missing data

3	(0.543) 0.547 0.543

4	(0.457) 0.453 0.457

Locus 6703 :

2 alleles

99.2% missing data

3	(0.408) 0.405 0.411

2	(0.592) 0.595 0.589

Locus 6704 :

2 alleles

98.8% missing data

3	(0.575) 0.583 0.576

4	(0.425) 0.417 0.424

Locus 6705 :

2 alleles

98.8% missing data

3	(0.681) 0.691 0.677

4	(0.319) 0.309 0.323

Locus 6706 :

2 alleles

99.2% missing data

4	(0.225) 0.233 0.219

3	(0.775) 0.767 0.781

Locus 6707 :

2 alleles

99.2% missing data

1	(0.338) 0.339 0.340

4	(0.662) 0.661 0.660

Locus 6708 :

2 alleles

99.2% missing data

3	(0.365) 0.363 0.367

1	(0.635) 0.637 0.633

Locus 6709 :

2 alleles

99.2% missing data

3	(0.315) 0.325 0.307

4	(0.685) 0.675 0.693

Locus 6710 :

2 alleles

98.0% missing data

1	(0.764) 0.774 0.760

3	(0.236) 0.226 0.240

Locus 6711 :

2 alleles

99.2% missing data

2	(0.530) 0.532 0.528

1	(0.470) 0.468 0.472

Locus 6712 :

2 alleles

99.2% missing data

4	(0.231) 0.230 0.232

3	(0.769) 0.770 0.768

Locus 6713 :

3 alleles

97.3% missing data

1	(0.620) 0.626 0.625

2	(0.221) 0.220 0.217

3	(0.159) 0.154 0.157

Locus 6714 :

2 alleles

97.7% missing data

4	(0.811) 0.817 0.808

1	(0.189) 0.183 0.192

Locus 6715 :

2 alleles

99.2% missing data

1	(0.508) 0.505 0.508

4	(0.492) 0.495 0.492

Locus 6716 :

2 alleles

99.2% missing data

2	(0.260) 0.267 0.255

1	(0.740) 0.733 0.745

Locus 6717 :

2 alleles

99.2% missing data

1	(0.694) 0.689 0.702

2	(0.306) 0.311 0.298

Locus 6718 :

2 alleles

91.0% missing data

3	(0.945) 0.956 0.932

1	(0.055) 0.044 0.068

Locus 6719 :

2 alleles

99.2% missing data

1	(0.520) 0.517 0.520

3	(0.480) 0.483 0.480

Locus 6720 :

2 alleles

99.2% missing data

3	(0.426) 0.422 0.430

4	(0.574) 0.578 0.570

Locus 6721 :

2 alleles

99.2% missing data

4	(0.226) 0.223 0.233

3	(0.774) 0.777 0.767

Locus 6722 :

2 alleles

91.0% missing data

4	(0.897) 0.911 0.892

1	(0.103) 0.089 0.108

Locus 6723 :

2 alleles

98.8% missing data

1	(0.341) 0.337 0.352

4	(0.659) 0.663 0.648

Locus 6724 :

2 alleles

99.2% missing data

1	(0.587) 0.582 0.587

2	(0.413) 0.418 0.413

Locus 6725 :

2 alleles

99.2% missing data

1	(0.736) 0.732 0.735

2	(0.264) 0.268 0.265

Locus 6726 :

2 alleles

99.2% missing data

4	(0.336) 0.333 0.343

2	(0.664) 0.667 0.657

Locus 6727 :

2 alleles

96.5% missing data

2	(0.180) 0.170 0.183

3	(0.820) 0.830 0.817

Locus 6728 :

2 alleles

96.1% missing data

4	(0.146) 0.133 0.152

1	(0.854) 0.867 0.848

Locus 6729 :

2 alleles

98.8% missing data

4	(0.544) 0.541 0.535

3	(0.456) 0.459 0.465

Locus 6730 :

2 alleles

99.2% missing data

3	(0.537) 0.530 0.545

1	(0.463) 0.470 0.455

Locus 6731 :

2 alleles

93.0% missing data

1	(0.918) 0.921 0.922

3	(0.082) 0.079 0.078

Locus 6732 :

2 alleles

96.9% missing data

3	(0.823) 0.817 0.840

1	(0.177) 0.183 0.160

Locus 6733 :

2 alleles

97.7% missing data

1	(0.252) 0.251 0.244

3	(0.748) 0.749 0.756

Locus 6734 :

2 alleles

99.2% missing data

2	(0.375) 0.377 0.371

3	(0.625) 0.623 0.629

Locus 6735 :

2 alleles

98.0% missing data

2	(0.546) 0.562 0.532

1	(0.454) 0.438 0.468

Locus 6736 :

2 alleles

98.8% missing data

1	(0.233) 0.225 0.239

3	(0.767) 0.775 0.761

Locus 6737 :

2 alleles

96.1% missing data

3	(0.233) 0.217 0.241

1	(0.767) 0.783 0.759

Locus 6738 :

2 alleles

99.2% missing data

1	(0.664) 0.664 0.658

4	(0.336) 0.336 0.342

Locus 6739 :

2 alleles

96.1% missing data

1	(0.387) 0.402 0.365

3	(0.613) 0.598 0.635

Locus 6740 :

2 alleles

99.2% missing data

1	(0.656) 0.649 0.652

2	(0.344) 0.351 0.348

Locus 6741 :

2 alleles

95.3% missing data

3	(0.846) 0.839 0.860

1	(0.154) 0.161 0.140

Locus 6742 :

2 alleles

8.6% missing data

2	(0.985) 0.986 0.993

3	(0.015) 0.014 0.007

Locus 6743 :

2 alleles

96.1% missing data

4	(0.100) 0.099 0.096

2	(0.900) 0.901 0.904

Locus 6744 :

2 alleles

99.2% missing data

1	(0.524) 0.532 0.513

2	(0.476) 0.468 0.487

Locus 6745 :

2 alleles

98.4% missing data

4	(0.599) 0.604 0.597

3	(0.401) 0.396 0.403

Locus 6746 :

2 alleles

99.2% missing data

2	(0.365) 0.358 0.372

4	(0.635) 0.642 0.628

Locus 6747 :

2 alleles

93.0% missing data

2	(0.897) 0.886 0.913

4	(0.103) 0.114 0.087

Locus 6748 :

2 alleles

99.2% missing data

2	(0.613) 0.612 0.611

1	(0.387) 0.388 0.389

Locus 6749 :

2 alleles

99.2% missing data

4	(0.795) 0.794 0.794

1	(0.205) 0.206 0.206

Locus 6750 :

3 alleles

98.4% missing data

1	(0.435) 0.432 0.439

3	(0.375) 0.376 0.374

2	(0.190) 0.192 0.186

Locus 6751 :

2 alleles

99.2% missing data

1	(0.364) 0.352 0.370

2	(0.636) 0.648 0.630

Locus 6752 :

2 alleles

99.2% missing data

3	(0.420) 0.430 0.413

4	(0.580) 0.570 0.587

Locus 6753 :

2 alleles

98.8% missing data

3	(0.283) 0.281 0.281

1	(0.717) 0.719 0.719

Locus 6754 :

3 alleles

98.8% missing data

1	(0.178) 0.181 0.176

3	(0.275) 0.278 0.274

2	(0.548) 0.541 0.549

Locus 6755 :

2 alleles

98.8% missing data

2	(0.405) 0.404 0.417

1	(0.595) 0.596 0.583

Locus 6756 :

2 alleles

96.5% missing data

1	(0.349) 0.335 0.349

4	(0.651) 0.665 0.651

Locus 6757 :

2 alleles

99.2% missing data

2	(0.354) 0.357 0.361

4	(0.646) 0.643 0.639

Locus 6758 :

2 alleles

90.6% missing data

4	(0.912) 0.921 0.906

1	(0.088) 0.079 0.094

Locus 6759 :

3 alleles

98.0% missing data

2	(0.591) 0.588 0.593

1	(0.232) 0.227 0.237

4	(0.177) 0.185 0.170

Locus 6760 :

3 alleles

98.8% missing data

4	(0.245) 0.249 0.241

2	(0.582) 0.588 0.578

3	(0.173) 0.163 0.181

Locus 6761 :

2 alleles

8.6% missing data

2	(0.983) 0.985 0.992

3	(0.017) 0.015 0.008

Locus 6762 :

2 alleles

97.7% missing data

1	(0.206) 0.205 0.205

4	(0.794) 0.795 0.795

Locus 6763 :

2 alleles

99.2% missing data

2	(0.560) 0.555 0.563

4	(0.440) 0.445 0.437

Locus 6764 :

2 alleles

99.2% missing data

4	(0.590) 0.589 0.597

3	(0.410) 0.411 0.403

Locus 6765 :

2 alleles

99.2% missing data

2	(0.218) 0.220 0.219

3	(0.782) 0.780 0.781

Locus 6766 :

2 alleles

98.8% missing data

3	(0.628) 0.637 0.622

1	(0.372) 0.363 0.378

Locus 6767 :

2 alleles

99.2% missing data

3	(0.559) 0.552 0.561

2	(0.441) 0.448 0.439

Locus 6768 :

2 alleles

99.2% missing data

2	(0.589) 0.586 0.588

1	(0.411) 0.414 0.412

Locus 6769 :

2 alleles

99.2% missing data

3	(0.809) 0.805 0.813

2	(0.191) 0.195 0.187

Locus 6770 :

2 alleles

99.2% missing data

1	(0.363) 0.360 0.363

4	(0.637) 0.640 0.637

Locus 6771 :

2 alleles

99.2% missing data

3	(0.770) 0.766 0.773

2	(0.230) 0.234 0.227

Locus 6772 :

2 alleles

99.2% missing data

3	(0.596) 0.588 0.607

1	(0.404) 0.412 0.393

Locus 6773 :

2 alleles

96.5% missing data

2	(0.188) 0.178 0.192

4	(0.812) 0.822 0.808

Locus 6774 :

3 alleles

98.8% missing data

4	(0.246) 0.245 0.248

1	(0.149) 0.150 0.148

2	(0.605) 0.605 0.604

Locus 6775 :

2 alleles

99.2% missing data

4	(0.316) 0.317 0.316

3	(0.684) 0.683 0.684

Locus 6776 :

2 alleles

98.8% missing data

1	(0.752) 0.755 0.749

3	(0.248) 0.245 0.251

Locus 6777 :

2 alleles

98.8% missing data

1	(0.593) 0.594 0.591

4	(0.407) 0.406 0.409

Locus 6778 :

2 alleles

99.2% missing data

2	(0.302) 0.301 0.311

1	(0.698) 0.699 0.689

Locus 6779 :

2 alleles

99.2% missing data

2	(0.669) 0.672 0.668

1	(0.331) 0.328 0.332

Locus 6780 :

2 alleles

96.1% missing data

1	(0.130) 0.121 0.133

3	(0.870) 0.879 0.867

Locus 6781 :

2 alleles

99.2% missing data

4	(0.273) 0.273 0.276

1	(0.727) 0.727 0.724

Locus 6782 :

2 alleles

99.2% missing data

4	(0.496) 0.495 0.495

2	(0.504) 0.505 0.505

Locus 6783 :

2 alleles

0.0% missing data

2	(0.987) 0.986 0.994

3	(0.013) 0.014 0.006

Locus 6784 :

2 alleles

93.0% missing data

4	(0.875) 0.876 0.886

2	(0.125) 0.124 0.114

Locus 6785 :

2 alleles

97.7% missing data

4	(0.887) 0.882 0.894

2	(0.113) 0.118 0.106

Locus 6786 :

2 alleles

98.8% missing data

3	(0.601) 0.603 0.600

4	(0.399) 0.397 0.400

Locus 6787 :

2 alleles

99.2% missing data

1	(0.411) 0.408 0.412

2	(0.589) 0.592 0.588

Locus 6788 :

2 alleles

99.2% missing data

3	(0.451) 0.446 0.451

2	(0.549) 0.554 0.549

Locus 6789 :

3 alleles

98.8% missing data

3	(0.314) 0.316 0.311

2	(0.319) 0.312 0.328

1	(0.367) 0.372 0.360

Locus 6790 :

2 alleles

99.2% missing data

1	(0.378) 0.380 0.380

2	(0.622) 0.620 0.620

Locus 6791 :

2 alleles

99.2% missing data

3	(0.603) 0.598 0.606

4	(0.397) 0.402 0.394

Locus 6792 :

2 alleles

96.1% missing data

4	(0.122) 0.115 0.128

1	(0.878) 0.885 0.872

Locus 6793 :

2 alleles

96.5% missing data

1	(0.809) 0.821 0.803

3	(0.191) 0.179 0.197

Locus 6794 :

2 alleles

99.2% missing data

2	(0.428) 0.434 0.431

4	(0.572) 0.566 0.569

Locus 6795 :

4 alleles

98.4% missing data

3	(0.138) 0.134 0.143

1	(0.392) 0.403 0.388

4	(0.217) 0.216 0.220

2	(0.253) 0.246 0.250

Locus 6796 :

2 alleles

99.2% missing data

1	(0.246) 0.247 0.250

3	(0.754) 0.753 0.750

Locus 6797 :

2 alleles

96.1% missing data

3	(0.787) 0.792 0.792

2	(0.213) 0.208 0.208

Locus 6798 :

2 alleles

99.2% missing data

2	(0.613) 0.612 0.606

3	(0.387) 0.388 0.394

Locus 6799 :

2 alleles

97.7% missing data

3	(0.743) 0.754 0.743

1	(0.257) 0.246 0.257

Locus 6800 :

3 alleles

98.4% missing data

4	(0.256) 0.252 0.255

3	(0.398) 0.397 0.404

1	(0.346) 0.351 0.341

Locus 6801 :

2 alleles

98.4% missing data

3	(0.605) 0.606 0.607

1	(0.395) 0.394 0.393

Locus 6802 :

2 alleles

99.2% missing data

1	(0.296) 0.295 0.299

3	(0.704) 0.705 0.701

Locus 6803 :

2 alleles

0.8% missing data

4	(0.985) 0.981 0.995

1	(0.015) 0.019 0.005

Locus 6804 :

2 alleles

99.2% missing data

2	(0.614) 0.614 0.610

1	(0.386) 0.386 0.390

Locus 6805 :

2 alleles

99.2% missing data

1	(0.786) 0.780 0.790

2	(0.214) 0.220 0.210

Locus 6806 :

3 alleles

98.8% missing data

2	(0.289) 0.287 0.290

1	(0.123) 0.130 0.120

4	(0.588) 0.583 0.590

Locus 6807 :

2 alleles

99.2% missing data

1	(0.519) 0.522 0.523

2	(0.481) 0.478 0.477

Locus 6808 :

2 alleles

96.5% missing data

1	(0.273) 0.273 0.262

3	(0.727) 0.727 0.738

Locus 6809 :

2 alleles

97.3% missing data

3	(0.227) 0.233 0.224

2	(0.773) 0.767 0.776

Locus 6810 :

3 alleles

97.3% missing data

2	(0.513) 0.503 0.524

3	(0.346) 0.349 0.338

4	(0.141) 0.148 0.138

Locus 6811 :

2 alleles

0.0% missing data

1	(0.988) 0.984 0.997

3	(0.012) 0.016 0.003

Locus 6812 :

2 alleles

99.2% missing data

1	(0.608) 0.610 0.609

2	(0.392) 0.390 0.391

Locus 6813 :

2 alleles

96.1% missing data

1	(0.448) 0.430 0.475

2	(0.552) 0.570 0.525

Locus 6814 :

2 alleles

99.2% missing data

1	(0.694) 0.702 0.689

4	(0.306) 0.298 0.311

Locus 6815 :

2 alleles

99.2% missing data

2	(0.467) 0.461 0.466

4	(0.533) 0.539 0.534

Locus 6816 :

2 alleles

99.2% missing data

2	(0.599) 0.600 0.596

1	(0.401) 0.400 0.404

Locus 6817 :

2 alleles

99.2% missing data

3	(0.379) 0.387 0.374

2	(0.621) 0.613 0.626

Locus 6818 :

2 alleles

0.0% missing data

3	(0.988) 0.983 0.997

4	(0.012) 0.017 0.003

Locus 6819 :

2 alleles

91.8% missing data

2	(0.854) 0.863 0.856

3	(0.146) 0.137 0.144

Locus 6820 :

2 alleles

98.8% missing data

1	(0.315) 0.313 0.319

2	(0.685) 0.687 0.681

Locus 6821 :

2 alleles

91.0% missing data

2	(0.755) 0.722 0.795

4	(0.245) 0.278 0.205

Locus 6822 :

2 alleles

98.0% missing data

3	(0.436) 0.426 0.439

1	(0.564) 0.574 0.561

Locus 6823 :

2 alleles

99.2% missing data

1	(0.168) 0.164 0.176

4	(0.832) 0.836 0.824

Locus 6824 :

2 alleles

99.2% missing data

3	(0.646) 0.643 0.644

1	(0.354) 0.357 0.356

Locus 6825 :

2 alleles

99.2% missing data

3	(0.594) 0.592 0.600

1	(0.406) 0.408 0.400

Locus 6826 :

2 alleles

98.8% missing data

3	(0.314) 0.320 0.306

2	(0.686) 0.680 0.694

Locus 6827 :

2 alleles

99.2% missing data

2	(0.530) 0.531 0.527

4	(0.470) 0.469 0.473

Locus 6828 :

2 alleles

8.2% missing data

4	(0.983) 0.979 0.994

2	(0.017) 0.021 0.006

Locus 6829 :

2 alleles

96.9% missing data

3	(0.792) 0.788 0.793

1	(0.208) 0.212 0.207

Locus 6830 :

2 alleles

99.2% missing data

4	(0.356) 0.352 0.358

1	(0.644) 0.648 0.642

Locus 6831 :

3 alleles

98.4% missing data

1	(0.292) 0.289 0.305

3	(0.405) 0.406 0.398

4	(0.303) 0.305 0.297

Locus 6832 :

2 alleles

99.2% missing data

4	(0.646) 0.643 0.653

3	(0.354) 0.357 0.347

Locus 6833 :

2 alleles

98.8% missing data

1	(0.440) 0.441 0.442

3	(0.560) 0.559 0.558

Locus 6834 :

3 alleles

98.8% missing data

1	(0.352) 0.359 0.343

3	(0.155) 0.156 0.160

2	(0.493) 0.486 0.497

Locus 6835 :

3 alleles

97.7% missing data

4	(0.175) 0.174 0.178

1	(0.706) 0.709 0.699

2	(0.118) 0.117 0.123

Locus 6836 :

2 alleles

98.8% missing data

1	(0.310) 0.309 0.311

3	(0.690) 0.691 0.689

Locus 6837 :

2 alleles

96.9% missing data

3	(0.142) 0.144 0.140

1	(0.858) 0.856 0.860

Locus 6838 :

2 alleles

99.2% missing data

3	(0.649) 0.653 0.650

1	(0.351) 0.347 0.350

Locus 6839 :

2 alleles

97.7% missing data

2	(0.644) 0.654 0.634

1	(0.356) 0.346 0.366

Locus 6840 :

2 alleles

96.1% missing data

2	(0.129) 0.128 0.127

4	(0.871) 0.872 0.873

Locus 6841 :

2 alleles

98.8% missing data

3	(0.505) 0.505 0.496

1	(0.495) 0.495 0.504

Locus 6842 :

2 alleles

98.8% missing data

2	(0.600) 0.604 0.605

1	(0.400) 0.396 0.395

Locus 6843 :

2 alleles

99.2% missing data

2	(0.542) 0.532 0.548

1	(0.458) 0.468 0.452

Locus 6844 :

2 alleles

99.2% missing data

1	(0.555) 0.555 0.560

4	(0.445) 0.445 0.440

Locus 6845 :

2 alleles

96.9% missing data

3	(0.867) 0.871 0.869

1	(0.133) 0.129 0.131

Locus 6846 :

2 alleles

98.4% missing data

1	(0.805) 0.803 0.810

4	(0.195) 0.197 0.190

Locus 6847 :

2 alleles

98.8% missing data

3	(0.593) 0.583 0.600

4	(0.407) 0.417 0.400

Locus 6848 :

2 alleles

99.2% missing data

3	(0.542) 0.538 0.543

2	(0.458) 0.462 0.457

Locus 6849 :

2 alleles

94.9% missing data

3	(0.872) 0.867 0.881

4	(0.128) 0.133 0.119

Locus 6850 :

2 alleles

98.8% missing data

2	(0.559) 0.557 0.558

4	(0.441) 0.443 0.442

Locus 6851 :

2 alleles

99.2% missing data

1	(0.508) 0.514 0.506

3	(0.492) 0.486 0.494

Locus 6852 :

2 alleles

99.2% missing data

3	(0.594) 0.600 0.597

2	(0.406) 0.400 0.403

Locus 6853 :

2 alleles

99.2% missing data

4	(0.679) 0.675 0.684

1	(0.321) 0.325 0.316

Locus 6854 :

2 alleles

98.8% missing data

1	(0.557) 0.557 0.561

2	(0.443) 0.443 0.439

Locus 6855 :

3 alleles

98.0% missing data

4	(0.317) 0.320 0.311

1	(0.326) 0.325 0.336

2	(0.357) 0.355 0.353

Locus 6856 :

2 alleles

98.8% missing data

1	(0.538) 0.542 0.553

2	(0.462) 0.458 0.447

Locus 6857 :

2 alleles

99.2% missing data

3	(0.397) 0.397 0.400

1	(0.603) 0.603 0.600

Locus 6858 :

2 alleles

92.6% missing data

3	(0.918) 0.915 0.927

4	(0.082) 0.085 0.073

Locus 6859 :

2 alleles

98.8% missing data

2	(0.637) 0.634 0.647

3	(0.363) 0.366 0.353

Locus 6860 :

3 alleles

98.8% missing data

4	(0.269) 0.266 0.273

1	(0.513) 0.513 0.510

3	(0.219) 0.221 0.216

Locus 6861 :

2 alleles

99.2% missing data

4	(0.565) 0.560 0.562

1	(0.435) 0.440 0.438

Locus 6862 :

2 alleles

99.2% missing data

2	(0.595) 0.594 0.587

4	(0.405) 0.406 0.413

Locus 6863 :

2 alleles

98.8% missing data

3	(0.498) 0.495 0.503

1	(0.502) 0.505 0.497

Locus 6864 :

2 alleles

0.0% missing data

4	(0.984) 0.981 0.995

1	(0.016) 0.019 0.005

Locus 6865 :

2 alleles

98.8% missing data

2	(0.602) 0.607 0.602

3	(0.398) 0.393 0.398

Locus 6866 :

2 alleles

95.7% missing data

2	(0.796) 0.813 0.795

3	(0.204) 0.187 0.205

Locus 6867 :

2 alleles

99.2% missing data

4	(0.508) 0.504 0.511

3	(0.492) 0.496 0.489

Locus 6868 :

2 alleles

99.2% missing data

4	(0.475) 0.470 0.484

2	(0.525) 0.530 0.516

Locus 6869 :

2 alleles

99.2% missing data

3	(0.655) 0.653 0.648

1	(0.345) 0.347 0.352

Locus 6870 :

2 alleles

97.7% missing data

3	(0.252) 0.254 0.245

1	(0.748) 0.746 0.755

Locus 6871 :

2 alleles

99.2% missing data

4	(0.836) 0.830 0.842

1	(0.164) 0.170 0.158

Locus 6872 :

2 alleles

99.2% missing data

1	(0.467) 0.461 0.472

2	(0.533) 0.539 0.528

Locus 6873 :

2 alleles

99.2% missing data

2	(0.656) 0.654 0.657

1	(0.344) 0.346 0.343

Locus 6874 :

2 alleles

98.4% missing data

4	(0.721) 0.732 0.715

2	(0.279) 0.268 0.285

Locus 6875 :

2 alleles

99.2% missing data

2	(0.227) 0.220 0.233

3	(0.773) 0.780 0.767

Locus 6876 :

2 alleles

98.8% missing data

1	(0.721) 0.728 0.710

2	(0.279) 0.272 0.290

Locus 6877 :

2 alleles

0.0% missing data

3	(0.987) 0.983 0.996

2	(0.013) 0.017 0.004

Locus 6878 :

2 alleles

99.2% missing data

1	(0.294) 0.293 0.298

4	(0.706) 0.707 0.702

Locus 6879 :

2 alleles

99.2% missing data

1	(0.551) 0.549 0.553

3	(0.449) 0.451 0.447

Locus 6880 :

3 alleles

98.8% missing data

3	(0.329) 0.328 0.328

1	(0.294) 0.289 0.295

4	(0.377) 0.383 0.376

Locus 6881 :

2 alleles

99.2% missing data

2	(0.519) 0.510 0.516

1	(0.481) 0.490 0.484

Locus 6882 :

2 alleles

99.2% missing data

1	(0.430) 0.432 0.429

2	(0.570) 0.568 0.571

Locus 6883 :

2 alleles

99.2% missing data

2	(0.431) 0.434 0.430

4	(0.569) 0.566 0.570

Locus 6884 :

2 alleles

96.5% missing data

4	(0.209) 0.197 0.215

2	(0.791) 0.803 0.785

Locus 6885 :

2 alleles

0.4% missing data

4	(0.986) 0.982 0.996

1	(0.014) 0.018 0.004

Locus 6886 :

2 alleles

95.3% missing data

1	(0.807) 0.806 0.818

4	(0.193) 0.194 0.182

Locus 6887 :

2 alleles

98.4% missing data

1	(0.230) 0.224 0.236

2	(0.770) 0.776 0.764

Locus 6888 :

2 alleles

99.2% missing data

4	(0.255) 0.253 0.262

1	(0.745) 0.747 0.738

Locus 6889 :

2 alleles

85.2% missing data

2	(0.945) 0.950 0.950

3	(0.055) 0.050 0.050

Locus 6890 :

2 alleles

90.6% missing data

4	(0.904) 0.924 0.895

2	(0.096) 0.076 0.105

Locus 6891 :

2 alleles

99.2% missing data

1	(0.361) 0.358 0.368

4	(0.639) 0.642 0.632

Locus 6892 :

2 alleles

99.2% missing data

4	(0.476) 0.467 0.486

3	(0.524) 0.533 0.514

Locus 6893 :

2 alleles

99.2% missing data

1	(0.419) 0.417 0.427

4	(0.581) 0.583 0.573

Locus 6894 :

2 alleles

99.2% missing data

3	(0.653) 0.653 0.667

4	(0.347) 0.347 0.333

Locus 6895 :

2 alleles

99.2% missing data

2	(0.592) 0.581 0.604

3	(0.408) 0.419 0.396

Locus 6896 :

2 alleles

99.2% missing data

2	(0.491) 0.494 0.483

1	(0.509) 0.506 0.517

Locus 6897 :

2 alleles

99.2% missing data

1	(0.680) 0.680 0.673

4	(0.320) 0.320 0.327

Locus 6898 :

2 alleles

98.8% missing data

2	(0.294) 0.292 0.300

3	(0.706) 0.708 0.700

Locus 6899 :

3 alleles

98.0% missing data

1	(0.391) 0.392 0.401

3	(0.153) 0.149 0.154

2	(0.456) 0.459 0.445

Locus 6900 :

2 alleles

96.1% missing data

2	(0.803) 0.807 0.805

3	(0.197) 0.193 0.195

Locus 6901 :

2 alleles

98.8% missing data

1	(0.637) 0.635 0.637

2	(0.363) 0.365 0.363

Locus 6902 :

2 alleles

98.8% missing data

1	(0.528) 0.520 0.540

3	(0.472) 0.480 0.460

Locus 6903 :

2 alleles

99.2% missing data

3	(0.359) 0.355 0.361

1	(0.641) 0.645 0.639

Locus 6904 :

2 alleles

98.8% missing data

1	(0.538) 0.533 0.546

3	(0.462) 0.467 0.454

Locus 6905 :

2 alleles

99.2% missing data

4	(0.319) 0.318 0.321

3	(0.681) 0.682 0.679

Locus 6906 :

2 alleles

99.2% missing data

1	(0.758) 0.753 0.761

3	(0.242) 0.247 0.239

Locus 6907 :

3 alleles

98.8% missing data

1	(0.515) 0.515 0.515

2	(0.224) 0.228 0.223

3	(0.260) 0.257 0.263

Locus 6908 :

2 alleles

97.7% missing data

3	(0.530) 0.535 0.531

2	(0.470) 0.465 0.469

Locus 6909 :

2 alleles

98.4% missing data

2	(0.800) 0.801 0.801

1	(0.200) 0.199 0.199

Locus 6910 :

2 alleles

94.1% missing data

3	(0.850) 0.848 0.852

1	(0.150) 0.152 0.148

Locus 6911 :

2 alleles

98.8% missing data

4	(0.623) 0.624 0.619

1	(0.377) 0.376 0.381

Locus 6912 :

3 alleles

89.1% missing data

3	(0.794) 0.831 0.749

1	(0.074) 0.063 0.076

2	(0.132) 0.107 0.175

Locus 6913 :

2 alleles

94.9% missing data

4	(0.853) 0.864 0.850

1	(0.147) 0.136 0.150

Locus 6914 :

2 alleles

99.2% missing data

2	(0.676) 0.667 0.676

3	(0.324) 0.333 0.324

Locus 6915 :

2 alleles

99.2% missing data

4	(0.519) 0.509 0.526

3	(0.481) 0.491 0.474

Locus 6916 :

2 alleles

89.8% missing data

3	(0.949) 0.954 0.950

1	(0.051) 0.046 0.050

Locus 6917 :

2 alleles

98.0% missing data

2	(0.485) 0.489 0.477

3	(0.515) 0.511 0.523

Locus 6918 :

2 alleles

98.8% missing data

3	(0.558) 0.557 0.561

2	(0.442) 0.443 0.439

Locus 6919 :

2 alleles

99.2% missing data

2	(0.504) 0.500 0.507

1	(0.496) 0.500 0.493

Locus 6920 :

2 alleles

99.2% missing data

4	(0.644) 0.637 0.647

1	(0.356) 0.363 0.353

Locus 6921 :

2 alleles

99.2% missing data

4	(0.340) 0.340 0.347

2	(0.660) 0.660 0.653

Locus 6922 :

2 alleles

98.4% missing data

1	(0.690) 0.691 0.692

4	(0.310) 0.309 0.308

Locus 6923 :

2 alleles

96.1% missing data

1	(0.162) 0.154 0.163

3	(0.838) 0.846 0.837

Locus 6924 :

2 alleles

99.2% missing data

1	(0.754) 0.749 0.758

4	(0.246) 0.251 0.242

Locus 6925 :

2 alleles

95.3% missing data

4	(0.882) 0.872 0.893

2	(0.118) 0.128 0.107

Locus 6926 :

2 alleles

99.2% missing data

1	(0.307) 0.310 0.307

3	(0.693) 0.690 0.693

Locus 6927 :

2 alleles

99.2% missing data

3	(0.575) 0.566 0.584

1	(0.425) 0.434 0.416

Locus 6928 :

2 alleles

99.2% missing data

1	(0.679) 0.673 0.682

3	(0.321) 0.327 0.318

Locus 6929 :

2 alleles

98.4% missing data

4	(0.606) 0.621 0.597

1	(0.394) 0.379 0.403

Locus 6930 :

3 alleles

97.7% missing data

2	(0.175) 0.166 0.176

4	(0.364) 0.361 0.366

1	(0.462) 0.473 0.458

Locus 6931 :

2 alleles

99.2% missing data

3	(0.419) 0.414 0.425

2	(0.581) 0.586 0.575

Locus 6932 :

2 alleles

99.2% missing data

2	(0.563) 0.563 0.570

3	(0.437) 0.437 0.430

Locus 6933 :

2 alleles

99.2% missing data

2	(0.494) 0.492 0.500

3	(0.506) 0.508 0.500

Locus 6934 :

2 alleles

99.2% missing data

2	(0.623) 0.624 0.618

4	(0.377) 0.376 0.382

Locus 6935 :

2 alleles

99.2% missing data

1	(0.256) 0.257 0.258

3	(0.744) 0.743 0.742

Locus 6936 :

2 alleles

99.2% missing data

2	(0.281) 0.286 0.279

1	(0.719) 0.714 0.721

Locus 6937 :

2 alleles

98.8% missing data

4	(0.694) 0.693 0.693

1	(0.306) 0.307 0.307

Locus 6938 :

2 alleles

99.2% missing data

4	(0.774) 0.772 0.775

1	(0.226) 0.228 0.225

Locus 6939 :

3 alleles

98.8% missing data

1	(0.406) 0.405 0.408

4	(0.318) 0.315 0.319

2	(0.276) 0.280 0.273

Locus 6940 :

2 alleles

94.9% missing data

2	(0.902) 0.902 0.906

1	(0.098) 0.098 0.094

Locus 6941 :

2 alleles

98.4% missing data

2	(0.445) 0.450 0.451

1	(0.555) 0.550 0.549

Locus 6942 :

3 alleles

98.8% missing data

1	(0.359) 0.363 0.351

2	(0.392) 0.382 0.406

3	(0.248) 0.255 0.243

Locus 6943 :

2 alleles

96.1% missing data

4	(0.314) 0.312 0.305

3	(0.686) 0.688 0.695

Locus 6944 :

2 alleles

99.2% missing data

1	(0.435) 0.440 0.435

3	(0.565) 0.560 0.565

Locus 6945 :

2 alleles

98.0% missing data

4	(0.711) 0.703 0.722

2	(0.289) 0.297 0.278

Locus 6946 :

2 alleles

96.9% missing data

4	(0.839) 0.843 0.841

2	(0.161) 0.157 0.159

Locus 6947 :

2 alleles

99.2% missing data

1	(0.691) 0.691 0.695

4	(0.309) 0.309 0.305

Locus 6948 :

2 alleles

98.0% missing data

4	(0.235) 0.234 0.228

1	(0.765) 0.766 0.772

Locus 6949 :

2 alleles

99.2% missing data

3	(0.708) 0.698 0.710

4	(0.292) 0.302 0.290

Locus 6950 :

2 alleles

99.2% missing data

4	(0.174) 0.165 0.183

3	(0.826) 0.835 0.817

Locus 6951 :

2 alleles

98.8% missing data

2	(0.546) 0.548 0.546

1	(0.454) 0.452 0.454

Locus 6952 :

2 alleles

99.2% missing data

2	(0.829) 0.828 0.821

4	(0.171) 0.172 0.179

Locus 6953 :

2 alleles

98.4% missing data

4	(0.748) 0.747 0.759

2	(0.252) 0.253 0.241

Locus 6954 :

2 alleles

98.8% missing data

1	(0.837) 0.841 0.837

2	(0.163) 0.159 0.163

Locus 6955 :

2 alleles

99.2% missing data

1	(0.522) 0.524 0.525

3	(0.478) 0.476 0.475

Locus 6956 :

2 alleles

99.2% missing data

3	(0.605) 0.596 0.604

2	(0.395) 0.404 0.396

Locus 6957 :

2 alleles

99.2% missing data

4	(0.735) 0.729 0.731

2	(0.265) 0.271 0.269

Locus 6958 :

2 alleles

97.7% missing data

3	(0.448) 0.441 0.445

2	(0.552) 0.559 0.555

Locus 6959 :

2 alleles

99.2% missing data

2	(0.410) 0.402 0.409

1	(0.590) 0.598 0.591

Locus 6960 :

2 alleles

96.1% missing data

2	(0.116) 0.114 0.113

1	(0.884) 0.886 0.887

Locus 6961 :

2 alleles

90.6% missing data

1	(0.914) 0.932 0.904

2	(0.086) 0.068 0.096

Locus 6962 :

2 alleles

98.4% missing data

2	(0.248) 0.254 0.246

1	(0.752) 0.746 0.754

Locus 6963 :

2 alleles

99.2% missing data

1	(0.344) 0.344 0.354

4	(0.656) 0.656 0.646

Locus 6964 :

2 alleles

99.2% missing data

3	(0.451) 0.443 0.455

1	(0.549) 0.557 0.545

Locus 6965 :

2 alleles

0.0% missing data

3	(0.989) 0.991 0.994

2	(0.011) 0.009 0.006

Locus 6966 :

2 alleles

9.0% missing data

3	(0.986) 0.981 0.995

2	(0.014) 0.019 0.005

Locus 6967 :

2 alleles

99.2% missing data

4	(0.270) 0.273 0.266

2	(0.730) 0.727 0.734

Locus 6968 :

2 alleles

99.2% missing data

3	(0.396) 0.393 0.401

1	(0.604) 0.607 0.599

Locus 6969 :

2 alleles

97.7% missing data

4	(0.795) 0.797 0.792

1	(0.205) 0.203 0.208

Locus 6970 :

2 alleles

91.0% missing data

2	(0.932) 0.946 0.925

4	(0.068) 0.054 0.075

Locus 6971 :

2 alleles

99.2% missing data

3	(0.183) 0.189 0.177

2	(0.817) 0.811 0.823

Locus 6972 :

2 alleles

99.2% missing data

2	(0.568) 0.563 0.572

1	(0.432) 0.437 0.428

Locus 6973 :

2 alleles

99.2% missing data

2	(0.315) 0.319 0.317

1	(0.685) 0.681 0.683

Locus 6974 :

2 alleles

99.2% missing data

4	(0.261) 0.263 0.264

1	(0.739) 0.737 0.736

Locus 6975 :

2 alleles

99.2% missing data

2	(0.334) 0.330 0.343

1	(0.666) 0.670 0.657

Locus 6976 :

2 alleles

99.2% missing data

4	(0.678) 0.677 0.677

3	(0.322) 0.323 0.323

Locus 6977 :

2 alleles

99.2% missing data

2	(0.518) 0.519 0.516

3	(0.482) 0.481 0.484

Locus 6978 :

2 alleles

93.4% missing data

3	(0.904) 0.907 0.909

1	(0.096) 0.093 0.091

Locus 6979 :

2 alleles

99.2% missing data

4	(0.627) 0.627 0.622

3	(0.373) 0.373 0.378

Locus 6980 :

2 alleles

99.2% missing data

1	(0.294) 0.293 0.296

2	(0.706) 0.707 0.704

Locus 6981 :

2 alleles

99.2% missing data

1	(0.561) 0.566 0.554

4	(0.439) 0.434 0.446

Locus 6982 :

2 alleles

99.2% missing data

3	(0.460) 0.453 0.467

4	(0.540) 0.547 0.533

Locus 6983 :

2 alleles

95.3% missing data

3	(0.900) 0.896 0.908

4	(0.100) 0.104 0.092

Locus 6984 :

2 alleles

95.7% missing data

3	(0.608) 0.627 0.590

2	(0.392) 0.373 0.410

Locus 6985 :

2 alleles

99.2% missing data

4	(0.685) 0.690 0.681

2	(0.315) 0.310 0.319

Locus 6986 :

2 alleles

99.2% missing data

3	(0.513) 0.518 0.508

4	(0.487) 0.482 0.492

Locus 6987 :

2 alleles

99.2% missing data

4	(0.690) 0.698 0.686

2	(0.310) 0.302 0.314

Locus 6988 :

2 alleles

98.0% missing data

3	(0.843) 0.836 0.844

4	(0.157) 0.164 0.156

Locus 6989 :

2 alleles

96.5% missing data

3	(0.908) 0.917 0.901

1	(0.092) 0.083 0.099

Locus 6990 :

2 alleles

0.8% missing data

4	(0.986) 0.982 0.996

1	(0.014) 0.018 0.004

Locus 6991 :

2 alleles

99.2% missing data

1	(0.334) 0.332 0.336

2	(0.666) 0.668 0.664

Locus 6992 :

2 alleles

98.4% missing data

2	(0.840) 0.843 0.830

3	(0.160) 0.157 0.170

Locus 6993 :

2 alleles

99.2% missing data

4	(0.249) 0.252 0.249

1	(0.751) 0.748 0.751

Locus 6994 :

2 alleles

99.2% missing data

2	(0.423) 0.429 0.419

4	(0.577) 0.571 0.581

Locus 6995 :

2 alleles

98.4% missing data

3	(0.678) 0.668 0.684

1	(0.322) 0.332 0.316

Locus 6996 :

2 alleles

98.8% missing data

4	(0.643) 0.638 0.648

1	(0.357) 0.362 0.352

Locus 6997 :

2 alleles

98.8% missing data

2	(0.696) 0.688 0.705

4	(0.304) 0.312 0.295

Locus 6998 :

2 alleles

96.9% missing data

4	(0.762) 0.768 0.764

1	(0.238) 0.232 0.236

Locus 6999 :

2 alleles

99.2% missing data

1	(0.610) 0.607 0.616

4	(0.390) 0.393 0.384

Locus 7000 :

2 alleles

99.2% missing data

2	(0.535) 0.537 0.536

3	(0.465) 0.463 0.464

Locus 7001 :

2 alleles

98.8% missing data

4	(0.345) 0.340 0.338

2	(0.655) 0.660 0.662

Locus 7002 :

2 alleles

99.2% missing data

2	(0.432) 0.431 0.438

4	(0.568) 0.569 0.562

Locus 7003 :

2 alleles

98.8% missing data

4	(0.483) 0.480 0.482

2	(0.517) 0.520 0.518

Locus 7004 :

2 alleles

96.1% missing data

4	(0.183) 0.174 0.190

3	(0.817) 0.826 0.810

Locus 7005 :

2 alleles

99.2% missing data

3	(0.717) 0.720 0.709

4	(0.283) 0.280 0.291

Locus 7006 :

2 alleles

98.8% missing data

3	(0.357) 0.358 0.364

4	(0.643) 0.642 0.636

Locus 7007 :

2 alleles

96.9% missing data

4	(0.736) 0.734 0.743

1	(0.264) 0.266 0.257

Locus 7008 :

2 alleles

98.0% missing data

4	(0.722) 0.723 0.726

3	(0.278) 0.277 0.274

Locus 7009 :

2 alleles

96.9% missing data

1	(0.854) 0.847 0.864

4	(0.146) 0.153 0.136

Locus 7010 :

2 alleles

99.2% missing data

1	(0.355) 0.358 0.354

3	(0.645) 0.642 0.646

Locus 7011 :

2 alleles

99.2% missing data

1	(0.414) 0.419 0.413

3	(0.586) 0.581 0.587

Locus 7012 :

2 alleles

99.2% missing data

2	(0.221) 0.222 0.221

1	(0.779) 0.778 0.779

Locus 7013 :

3 alleles

98.8% missing data

2	(0.213) 0.207 0.216

1	(0.528) 0.531 0.522

4	(0.259) 0.262 0.261

Locus 7014 :

2 alleles

91.0% missing data

4	(0.888) 0.907 0.878

2	(0.112) 0.093 0.122

Locus 7015 :

2 alleles

98.8% missing data

2	(0.705) 0.701 0.713

4	(0.295) 0.299 0.287

Locus 7016 :

2 alleles

0.0% missing data

3	(0.987) 0.984 0.996

2	(0.013) 0.016 0.004

Locus 7017 :

2 alleles

99.2% missing data

2	(0.446) 0.450 0.448

1	(0.554) 0.550 0.552

Locus 7018 :

2 alleles

99.2% missing data

4	(0.483) 0.482 0.480

2	(0.517) 0.518 0.520

Locus 7019 :

2 alleles

99.2% missing data

3	(0.574) 0.571 0.577

2	(0.426) 0.429 0.423

Locus 7020 :

2 alleles

96.1% missing data

3	(0.863) 0.871 0.858

4	(0.137) 0.129 0.142

Locus 7021 :

2 alleles

97.3% missing data

2	(0.153) 0.155 0.147

1	(0.847) 0.845 0.853

Locus 7022 :

2 alleles

99.2% missing data

4	(0.573) 0.577 0.575

2	(0.427) 0.423 0.425

Locus 7023 :

2 alleles

99.2% missing data

2	(0.583) 0.575 0.589

3	(0.417) 0.425 0.411

Locus 7024 :

2 alleles

97.7% missing data

2	(0.632) 0.637 0.640

4	(0.368) 0.363 0.360

Locus 7025 :

2 alleles

98.8% missing data

3	(0.730) 0.727 0.725

2	(0.270) 0.273 0.275

Locus 7026 :

2 alleles

99.2% missing data

3	(0.689) 0.692 0.683

2	(0.311) 0.308 0.317

Locus 7027 :

2 alleles

98.8% missing data

2	(0.708) 0.702 0.713

4	(0.292) 0.298 0.287

Locus 7028 :

2 alleles

0.4% missing data

2	(0.987) 0.982 0.996

3	(0.013) 0.018 0.004

Locus 7029 :

2 alleles

99.2% missing data

1	(0.260) 0.268 0.258

3	(0.740) 0.732 0.742

Locus 7030 :

2 alleles

99.2% missing data

3	(0.430) 0.425 0.427

1	(0.570) 0.575 0.573

Locus 7031 :

2 alleles

98.4% missing data

1	(0.297) 0.289 0.295

4	(0.703) 0.711 0.705

Locus 7032 :

2 alleles

99.2% missing data

2	(0.307) 0.304 0.306

4	(0.693) 0.696 0.694

Locus 7033 :

2 alleles

99.2% missing data

2	(0.490) 0.488 0.491

1	(0.510) 0.512 0.509

Locus 7034 :

2 alleles

99.2% missing data

1	(0.476) 0.476 0.479

3	(0.524) 0.524 0.521

Locus 7035 :

2 alleles

99.2% missing data

4	(0.815) 0.818 0.814

3	(0.185) 0.182 0.186

Locus 7036 :

2 alleles

99.2% missing data

1	(0.537) 0.532 0.539

2	(0.463) 0.468 0.461

Locus 7037 :

2 alleles

99.2% missing data

4	(0.632) 0.634 0.632

3	(0.368) 0.366 0.368

Locus 7038 :

2 alleles

8.2% missing data

1	(0.986) 0.978 0.996

4	(0.014) 0.022 0.004

Locus 7039 :

2 alleles

98.8% missing data

2	(0.609) 0.596 0.609

4	(0.391) 0.404 0.391

Locus 7040 :

2 alleles

99.2% missing data

1	(0.691) 0.693 0.689

2	(0.309) 0.307 0.311

Locus 7041 :

2 alleles

98.4% missing data

1	(0.549) 0.547 0.554

2	(0.451) 0.453 0.446

Locus 7042 :

2 alleles

99.2% missing data

4	(0.437) 0.435 0.431

3	(0.563) 0.565 0.569

Locus 7043 :

2 alleles

99.2% missing data

3	(0.541) 0.537 0.544

2	(0.459) 0.463 0.456

Locus 7044 :

2 alleles

99.2% missing data

3	(0.338) 0.347 0.331

2	(0.662) 0.653 0.669

Locus 7045 :

2 alleles

99.2% missing data

1	(0.783) 0.785 0.780

3	(0.217) 0.215 0.220

Locus 7046 :

2 alleles

98.8% missing data

2	(0.472) 0.479 0.465

1	(0.528) 0.521 0.535

Locus 7047 :

2 alleles

96.9% missing data

2	(0.250) 0.255 0.241

1	(0.750) 0.745 0.759

Locus 7048 :

2 alleles

98.0% missing data

2	(0.338) 0.327 0.339

1	(0.662) 0.673 0.661

Locus 7049 :

3 alleles

98.8% missing data

3	(0.415) 0.413 0.422

4	(0.289) 0.292 0.286

1	(0.296) 0.295 0.292

Locus 7050 :

2 alleles

98.8% missing data

2	(0.748) 0.742 0.756

1	(0.252) 0.258 0.244

Locus 7051 :

2 alleles

99.2% missing data

4	(0.693) 0.690 0.696

2	(0.307) 0.310 0.304

Locus 7052 :

2 alleles

99.2% missing data

2	(0.798) 0.793 0.801

1	(0.202) 0.207 0.199

Locus 7053 :

3 alleles

98.0% missing data

2	(0.494) 0.483 0.489

1	(0.158) 0.153 0.166

3	(0.347) 0.363 0.345

Locus 7054 :

2 alleles

99.2% missing data

3	(0.331) 0.336 0.326

1	(0.669) 0.664 0.674

Locus 7055 :

2 alleles

99.2% missing data

2	(0.325) 0.319 0.333

1	(0.675) 0.681 0.667

Locus 7056 :

2 alleles

99.2% missing data

4	(0.586) 0.587 0.582

3	(0.414) 0.413 0.418

Locus 7057 :

2 alleles

98.8% missing data

3	(0.204) 0.202 0.210

2	(0.796) 0.798 0.790

Locus 7058 :

2 alleles

98.8% missing data

2	(0.648) 0.650 0.642

1	(0.352) 0.350 0.358

Locus 7059 :

2 alleles

99.2% missing data

1	(0.442) 0.436 0.441

2	(0.558) 0.564 0.559

Locus 7060 :

2 alleles

98.0% missing data

3	(0.665) 0.658 0.683

1	(0.335) 0.342 0.317

Locus 7061 :

2 alleles

99.2% missing data

4	(0.303) 0.301 0.305

3	(0.697) 0.699 0.695

Locus 7062 :

2 alleles

90.6% missing data

2	(0.896) 0.905 0.894

3	(0.104) 0.095 0.106

Locus 7063 :

3 alleles

98.4% missing data

1	(0.331) 0.332 0.317

3	(0.351) 0.339 0.369

2	(0.318) 0.329 0.314

Locus 7064 :

2 alleles

99.2% missing data

1	(0.222) 0.223 0.223

2	(0.778) 0.777 0.777

Locus 7065 :

2 alleles

99.2% missing data

1	(0.683) 0.682 0.680

4	(0.317) 0.318 0.320

Locus 7066 :

3 alleles

98.8% missing data

4	(0.421) 0.420 0.420

2	(0.224) 0.219 0.234

3	(0.354) 0.361 0.347

Locus 7067 :

2 alleles

99.2% missing data

3	(0.489) 0.484 0.489

2	(0.511) 0.516 0.511

Locus 7068 :

2 alleles

99.2% missing data

3	(0.301) 0.297 0.307

2	(0.699) 0.703 0.693

Locus 7069 :

2 alleles

99.2% missing data

3	(0.479) 0.483 0.481

4	(0.521) 0.517 0.519

Locus 7070 :

2 alleles

92.6% missing data

1	(0.899) 0.918 0.892

2	(0.101) 0.082 0.108

Locus 7071 :

2 alleles

98.8% missing data

2	(0.500) 0.504 0.500

4	(0.500) 0.496 0.500

Locus 7072 :

2 alleles

99.2% missing data

3	(0.589) 0.586 0.586

4	(0.411) 0.414 0.414

Locus 7073 :

2 alleles

99.2% missing data

4	(0.513) 0.517 0.522

3	(0.487) 0.483 0.478

Locus 7074 :

2 alleles

99.2% missing data

2	(0.535) 0.531 0.538

3	(0.465) 0.469 0.462

Locus 7075 :

2 alleles

99.2% missing data

3	(0.543) 0.543 0.539

2	(0.457) 0.457 0.461

Locus 7076 :

2 alleles

98.8% missing data

2	(0.753) 0.754 0.751

4	(0.247) 0.246 0.249

Locus 7077 :

2 alleles

99.2% missing data

3	(0.519) 0.518 0.519

4	(0.481) 0.482 0.481

Locus 7078 :

2 alleles

97.7% missing data

3	(0.580) 0.570 0.597

1	(0.420) 0.430 0.403

Locus 7079 :

2 alleles

99.2% missing data

3	(0.549) 0.547 0.554

1	(0.451) 0.453 0.446

Locus 7080 :

2 alleles

97.7% missing data

1	(0.756) 0.753 0.759

4	(0.244) 0.247 0.241

Locus 7081 :

2 alleles

99.2% missing data

3	(0.339) 0.341 0.338

1	(0.661) 0.659 0.662

Locus 7082 :

2 alleles

97.7% missing data

1	(0.346) 0.335 0.350

2	(0.654) 0.665 0.650

Locus 7083 :

2 alleles

92.6% missing data

1	(0.890) 0.882 0.900

4	(0.110) 0.118 0.100

Locus 7084 :

2 alleles

98.8% missing data

3	(0.396) 0.392 0.401

2	(0.604) 0.608 0.599

Locus 7085 :

2 alleles

99.2% missing data

2	(0.688) 0.683 0.684

1	(0.312) 0.317 0.316

Locus 7086 :

2 alleles

99.2% missing data

2	(0.459) 0.463 0.462

1	(0.541) 0.537 0.538

Locus 7087 :

2 alleles

98.8% missing data

1	(0.664) 0.657 0.672

3	(0.336) 0.343 0.328

Locus 7088 :

2 alleles

98.8% missing data

1	(0.623) 0.620 0.629

4	(0.377) 0.380 0.371

Locus 7089 :

2 alleles

99.2% missing data

2	(0.425) 0.426 0.425

4	(0.575) 0.574 0.575

Locus 7090 :

2 alleles

99.2% missing data

3	(0.328) 0.336 0.323

2	(0.672) 0.664 0.677

Locus 7091 :

2 alleles

99.2% missing data

2	(0.618) 0.614 0.628

3	(0.382) 0.386 0.372

Locus 7092 :

2 alleles

99.2% missing data

4	(0.592) 0.594 0.588

2	(0.408) 0.406 0.412

Locus 7093 :

3 alleles

96.1% missing data

4	(0.648) 0.639 0.664

1	(0.154) 0.153 0.150

2	(0.198) 0.208 0.185

Locus 7094 :

2 alleles

99.2% missing data

4	(0.494) 0.494 0.492

1	(0.506) 0.506 0.508

Locus 7095 :

2 alleles

99.2% missing data

2	(0.538) 0.535 0.547

3	(0.462) 0.465 0.453

Locus 7096 :

2 alleles

99.2% missing data

1	(0.522) 0.516 0.523

3	(0.478) 0.484 0.477

Locus 7097 :

2 alleles

98.8% missing data

2	(0.545) 0.550 0.547

4	(0.455) 0.450 0.453

Locus 7098 :

2 alleles

98.4% missing data

2	(0.443) 0.447 0.443

3	(0.557) 0.553 0.557

Locus 7099 :

2 alleles

99.2% missing data

3	(0.721) 0.712 0.728

1	(0.279) 0.288 0.272

Locus 7100 :

2 alleles

99.2% missing data

3	(0.779) 0.770 0.786

1	(0.221) 0.230 0.214

Locus 7101 :

2 alleles

99.2% missing data

3	(0.710) 0.715 0.696

4	(0.290) 0.285 0.304

Locus 7102 :

2 alleles

98.8% missing data

3	(0.492) 0.490 0.496

1	(0.508) 0.510 0.504

Locus 7103 :

2 alleles

0.0% missing data

2	(0.987) 0.991 0.993

3	(0.013) 0.009 0.007

Locus 7104 :

2 alleles

99.2% missing data

2	(0.292) 0.289 0.298

1	(0.708) 0.711 0.702

Locus 7105 :

2 alleles

99.2% missing data

4	(0.336) 0.340 0.338

3	(0.664) 0.660 0.662

Locus 7106 :

2 alleles

99.2% missing data

1	(0.245) 0.243 0.245

2	(0.755) 0.757 0.755

Locus 7107 :

2 alleles

99.2% missing data

1	(0.402) 0.403 0.405

3	(0.598) 0.597 0.595

Locus 7108 :

2 alleles

99.2% missing data

1	(0.748) 0.752 0.745

3	(0.252) 0.248 0.255

Locus 7109 :

2 alleles

97.7% missing data

3	(0.293) 0.279 0.306

1	(0.707) 0.721 0.694

Locus 7110 :

2 alleles

96.5% missing data

1	(0.815) 0.810 0.832

4	(0.185) 0.190 0.168

Locus 7111 :

2 alleles

99.2% missing data

3	(0.425) 0.424 0.424

1	(0.575) 0.576 0.576

Locus 7112 :

2 alleles

98.8% missing data

3	(0.363) 0.358 0.362

4	(0.637) 0.642 0.638

Locus 7113 :

2 alleles

98.8% missing data

4	(0.414) 0.415 0.414

2	(0.586) 0.585 0.586

Locus 7114 :

2 alleles

93.0% missing data

3	(0.888) 0.880 0.903

2	(0.112) 0.120 0.097

Locus 7115 :

2 alleles

96.5% missing data

3	(0.480) 0.480 0.484

4	(0.520) 0.520 0.516

Locus 7116 :

2 alleles

98.8% missing data

1	(0.763) 0.758 0.769

2	(0.237) 0.242 0.231

Locus 7117 :

3 alleles

98.8% missing data

4	(0.408) 0.414 0.404

3	(0.289) 0.284 0.297

2	(0.302) 0.302 0.299

Locus 7118 :

2 alleles

99.2% missing data

1	(0.395) 0.389 0.401

4	(0.605) 0.611 0.599

Locus 7119 :

2 alleles

97.7% missing data

2	(0.273) 0.258 0.281

1	(0.727) 0.742 0.719

Locus 7120 :

2 alleles

99.2% missing data

4	(0.468) 0.471 0.463

1	(0.532) 0.529 0.537

Locus 7121 :

2 alleles

99.2% missing data

3	(0.426) 0.417 0.433

1	(0.574) 0.583 0.567

Locus 7122 :

2 alleles

99.2% missing data

2	(0.258) 0.253 0.268

4	(0.742) 0.747 0.732

Locus 7123 :

2 alleles

89.8% missing data

1	(0.762) 0.804 0.729

2	(0.238) 0.196 0.271

Locus 7124 :

2 alleles

98.4% missing data

1	(0.726) 0.724 0.726

2	(0.274) 0.276 0.274

Locus 7125 :

2 alleles

99.2% missing data

1	(0.614) 0.617 0.608

4	(0.386) 0.383 0.392

Locus 7126 :

2 alleles

25.0% missing data

2	(0.981) 0.973 0.994

3	(0.019) 0.027 0.006

Locus 7127 :

2 alleles

99.2% missing data

2	(0.697) 0.695 0.699

1	(0.303) 0.305 0.301

Locus 7128 :

2 alleles

99.2% missing data

4	(0.199) 0.200 0.203

1	(0.801) 0.800 0.797

Locus 7129 :

2 alleles

98.8% missing data

2	(0.660) 0.657 0.662

1	(0.340) 0.343 0.338

Locus 7130 :

2 alleles

99.2% missing data

2	(0.446) 0.448 0.442

4	(0.554) 0.552 0.558

Locus 7131 :

2 alleles

99.2% missing data

3	(0.305) 0.307 0.310

2	(0.695) 0.693 0.690

Locus 7132 :

2 alleles

99.2% missing data

1	(0.627) 0.625 0.628

3	(0.373) 0.375 0.372

Locus 7133 :

2 alleles

97.7% missing data

1	(0.764) 0.755 0.776

3	(0.236) 0.245 0.224

Locus 7134 :

2 alleles

75.0% missing data

2	(0.945) 0.935 0.964

3	(0.055) 0.065 0.036

Locus 7135 :

2 alleles

99.2% missing data

2	(0.411) 0.415 0.411

1	(0.589) 0.585 0.589

Locus 7136 :

2 alleles

99.2% missing data

1	(0.375) 0.368 0.380

3	(0.625) 0.632 0.620

Locus 7137 :

2 alleles

97.7% missing data

3	(0.148) 0.149 0.151

4	(0.852) 0.851 0.849

Locus 7138 :

2 alleles

99.2% missing data

4	(0.712) 0.713 0.710

3	(0.288) 0.287 0.290

Locus 7139 :

2 alleles

99.2% missing data

4	(0.567) 0.562 0.571

1	(0.433) 0.438 0.429

Locus 7140 :

2 alleles

98.8% missing data

1	(0.751) 0.754 0.749

2	(0.249) 0.246 0.251

Locus 7141 :

2 alleles

98.8% missing data

3	(0.717) 0.724 0.712

4	(0.283) 0.276 0.288

Locus 7142 :

2 alleles

98.4% missing data

1	(0.452) 0.456 0.446

2	(0.548) 0.544 0.554

Locus 7143 :

2 alleles

99.2% missing data

2	(0.324) 0.322 0.328

1	(0.676) 0.678 0.672

Locus 7144 :

3 alleles

98.8% missing data

1	(0.554) 0.558 0.551

3	(0.134) 0.140 0.129

2	(0.312) 0.302 0.320

Locus 7145 :

2 alleles

99.2% missing data

2	(0.336) 0.339 0.333

1	(0.664) 0.661 0.667

Locus 7146 :

3 alleles

98.0% missing data

1	(0.414) 0.408 0.426

4	(0.265) 0.272 0.255

2	(0.321) 0.319 0.319

Locus 7147 :

2 alleles

99.2% missing data

2	(0.481) 0.480 0.483

4	(0.519) 0.520 0.517

Locus 7148 :

2 alleles

99.2% missing data

1	(0.330) 0.328 0.333

2	(0.670) 0.672 0.667

Locus 7149 :

2 alleles

99.2% missing data

4	(0.252) 0.248 0.255

2	(0.748) 0.752 0.745

Locus 7150 :

2 alleles

98.8% missing data

3	(0.389) 0.390 0.392

1	(0.611) 0.610 0.608

Locus 7151 :

2 alleles

99.2% missing data

4	(0.664) 0.656 0.673

2	(0.336) 0.344 0.327

Locus 7152 :

2 alleles

99.2% missing data

3	(0.362) 0.355 0.363

1	(0.638) 0.645 0.637

Locus 7153 :

2 alleles

99.2% missing data

3	(0.584) 0.578 0.582

1	(0.416) 0.422 0.418

Locus 7154 :

2 alleles

94.9% missing data

4	(0.914) 0.916 0.915

3	(0.086) 0.084 0.085

Locus 7155 :

2 alleles

98.8% missing data

2	(0.579) 0.581 0.585

1	(0.421) 0.419 0.415

Locus 7156 :

2 alleles

98.8% missing data

4	(0.446) 0.451 0.442

1	(0.554) 0.549 0.558

Locus 7157 :

2 alleles

97.3% missing data

3	(0.819) 0.816 0.820

4	(0.181) 0.184 0.180

Locus 7158 :

2 alleles

98.8% missing data

4	(0.793) 0.791 0.796

1	(0.207) 0.209 0.204

Locus 7159 :

2 alleles

99.2% missing data

1	(0.255) 0.249 0.260

3	(0.745) 0.751 0.740

Locus 7160 :

2 alleles

98.8% missing data

1	(0.809) 0.810 0.805

2	(0.191) 0.190 0.195

Locus 7161 :

2 alleles

98.0% missing data

1	(0.564) 0.562 0.572

4	(0.436) 0.438 0.428

Locus 7162 :

2 alleles

98.8% missing data

4	(0.305) 0.308 0.301

1	(0.695) 0.692 0.699

Locus 7163 :

3 alleles

98.8% missing data

4	(0.275) 0.278 0.274

3	(0.193) 0.193 0.192

2	(0.532) 0.529 0.534

Locus 7164 :

3 alleles

91.8% missing data

1	(0.893) 0.888 0.903

2	(0.059) 0.060 0.056

4	(0.048) 0.052 0.041

Locus 7165 :

2 alleles

99.2% missing data

3	(0.729) 0.721 0.729

4	(0.271) 0.279 0.271

Locus 7166 :

2 alleles

91.4% missing data

1	(0.863) 0.885 0.858

2	(0.137) 0.115 0.142

Locus 7167 :

2 alleles

99.2% missing data

2	(0.334) 0.329 0.347

4	(0.666) 0.671 0.653

Locus 7168 :

2 alleles

99.2% missing data

2	(0.628) 0.634 0.622

1	(0.372) 0.366 0.378

Locus 7169 :

2 alleles

99.2% missing data

1	(0.510) 0.504 0.507

4	(0.490) 0.496 0.493

Locus 7170 :

2 alleles

99.2% missing data

1	(0.445) 0.458 0.443

3	(0.555) 0.542 0.557

Locus 7171 :

3 alleles

98.8% missing data

1	(0.272) 0.274 0.275

3	(0.237) 0.240 0.233

4	(0.491) 0.486 0.492

Locus 7172 :

3 alleles

98.4% missing data

2	(0.114) 0.112 0.113

3	(0.532) 0.535 0.532

1	(0.355) 0.353 0.356

Locus 7173 :

2 alleles

99.2% missing data

2	(0.275) 0.275 0.276

4	(0.725) 0.725 0.724

Locus 7174 :

2 alleles

98.8% missing data

2	(0.707) 0.698 0.715

3	(0.293) 0.302 0.285

Locus 7175 :

2 alleles

99.2% missing data

1	(0.630) 0.632 0.627

2	(0.370) 0.368 0.373

Locus 7176 :

2 alleles

99.2% missing data

1	(0.757) 0.758 0.750

3	(0.243) 0.242 0.250

Locus 7177 :

2 alleles

92.6% missing data

1	(0.903) 0.893 0.913

3	(0.097) 0.107 0.087

Locus 7178 :

2 alleles

98.8% missing data

1	(0.666) 0.659 0.673

4	(0.334) 0.341 0.327

Locus 7179 :

2 alleles

99.2% missing data

2	(0.225) 0.218 0.227

3	(0.775) 0.782 0.773

Locus 7180 :

2 alleles

98.8% missing data

2	(0.675) 0.664 0.684

3	(0.325) 0.336 0.316

Locus 7181 :

2 alleles

99.2% missing data

1	(0.451) 0.448 0.456

3	(0.549) 0.552 0.544

Locus 7182 :

2 alleles

99.2% missing data

3	(0.684) 0.692 0.679

4	(0.316) 0.308 0.321

Locus 7183 :

2 alleles

95.3% missing data

2	(0.096) 0.095 0.096

1	(0.904) 0.905 0.904

Locus 7184 :

3 alleles

95.3% missing data

3	(0.709) 0.720 0.710

2	(0.111) 0.098 0.116

4	(0.181) 0.182 0.174

Locus 7185 :

2 alleles

99.2% missing data

4	(0.577) 0.577 0.573

2	(0.423) 0.423 0.427

Locus 7186 :

2 alleles

99.2% missing data

2	(0.518) 0.519 0.512

4	(0.482) 0.481 0.488

Locus 7187 :

2 alleles

99.2% missing data

1	(0.401) 0.396 0.409

3	(0.599) 0.604 0.591

Locus 7188 :

2 alleles

99.2% missing data

2	(0.719) 0.721 0.715

3	(0.281) 0.279 0.285

Locus 7189 :

2 alleles

99.2% missing data

1	(0.612) 0.600 0.620

3	(0.388) 0.400 0.380

Locus 7190 :

2 alleles

99.2% missing data

3	(0.389) 0.394 0.387

4	(0.611) 0.606 0.613

Locus 7191 :

2 alleles

91.0% missing data

4	(0.910) 0.919 0.911

3	(0.090) 0.081 0.089

Locus 7192 :

2 alleles

98.0% missing data

3	(0.514) 0.523 0.523

4	(0.486) 0.477 0.477

Locus 7193 :

2 alleles

8.2% missing data

2	(0.983) 0.975 0.995

3	(0.017) 0.025 0.005

Locus 7194 :

3 alleles

98.8% missing data

2	(0.487) 0.487 0.484

4	(0.327) 0.332 0.324

3	(0.186) 0.182 0.193

Locus 7195 :

4 alleles

91.8% missing data

2	(0.722) 0.716 0.750

3	(0.107) 0.104 0.101

4	(0.079) 0.088 0.063

1	(0.092) 0.092 0.085

Locus 7196 :

2 alleles

95.3% missing data

3	(0.858) 0.869 0.850

2	(0.142) 0.131 0.150

Locus 7197 :

2 alleles

99.2% missing data

1	(0.548) 0.552 0.555

4	(0.452) 0.448 0.445

Locus 7198 :

2 alleles

99.2% missing data

2	(0.538) 0.541 0.534

4	(0.462) 0.459 0.466

Locus 7199 :

2 alleles

98.0% missing data

2	(0.315) 0.316 0.306

4	(0.685) 0.684 0.694

Locus 7200 :

2 alleles

99.2% missing data

1	(0.528) 0.529 0.532

3	(0.472) 0.471 0.468

Locus 7201 :

2 alleles

91.0% missing data

2	(0.895) 0.914 0.887

4	(0.105) 0.086 0.113

Locus 7202 :

2 alleles

99.2% missing data

2	(0.686) 0.686 0.684

3	(0.314) 0.314 0.316

Locus 7203 :

2 alleles

96.1% missing data

2	(0.667) 0.659 0.682

4	(0.333) 0.341 0.318

Locus 7204 :

2 alleles

98.8% missing data

3	(0.557) 0.554 0.564

1	(0.443) 0.446 0.436

Locus 7205 :

2 alleles

96.9% missing data

1	(0.787) 0.795 0.790

3	(0.213) 0.205 0.210

Locus 7206 :

2 alleles

99.2% missing data

3	(0.368) 0.372 0.371

2	(0.632) 0.628 0.629

Locus 7207 :

2 alleles

99.2% missing data

4	(0.714) 0.721 0.707

1	(0.286) 0.279 0.293

Locus 7208 :

2 alleles

99.2% missing data

2	(0.292) 0.292 0.296

3	(0.708) 0.708 0.704

Locus 7209 :

2 alleles

93.0% missing data

3	(0.154) 0.178 0.132

2	(0.846) 0.822 0.868

Locus 7210 :

2 alleles

99.2% missing data

3	(0.288) 0.284 0.294

2	(0.712) 0.716 0.706

Locus 7211 :

2 alleles

99.2% missing data

2	(0.667) 0.672 0.665

3	(0.333) 0.328 0.335

Locus 7212 :

2 alleles

99.2% missing data

1	(0.501) 0.500 0.501

4	(0.499) 0.500 0.499

Locus 7213 :

2 alleles

96.9% missing data

1	(0.178) 0.167 0.181

2	(0.822) 0.833 0.819

Locus 7214 :

2 alleles

99.2% missing data

3	(0.586) 0.582 0.595

2	(0.414) 0.418 0.405

Locus 7215 :

2 alleles

99.2% missing data

3	(0.706) 0.710 0.703

1	(0.294) 0.290 0.297

Locus 7216 :

2 alleles

99.2% missing data

4	(0.403) 0.390 0.409

2	(0.597) 0.610 0.591

Locus 7217 :

3 alleles

97.3% missing data

2	(0.238) 0.230 0.239

1	(0.403) 0.425 0.402

3	(0.359) 0.345 0.359

Locus 7218 :

2 alleles

98.8% missing data

1	(0.654) 0.658 0.655

2	(0.346) 0.342 0.345

Locus 7219 :

2 alleles

99.2% missing data

3	(0.640) 0.643 0.641

2	(0.360) 0.357 0.359

Locus 7220 :

2 alleles

99.2% missing data

1	(0.706) 0.706 0.710

2	(0.294) 0.294 0.290

Locus 7221 :

2 alleles

99.2% missing data

4	(0.499) 0.503 0.499

3	(0.501) 0.497 0.501

Locus 7222 :

2 alleles

99.2% missing data

1	(0.597) 0.598 0.599

3	(0.403) 0.402 0.401

Locus 7223 :

2 alleles

99.2% missing data

3	(0.549) 0.543 0.547

4	(0.451) 0.457 0.453

Locus 7224 :

2 alleles

99.2% missing data

2	(0.687) 0.683 0.696

1	(0.313) 0.317 0.304

Locus 7225 :

2 alleles

99.2% missing data

2	(0.499) 0.506 0.497

4	(0.501) 0.494 0.503

Locus 7226 :

2 alleles

0.0% missing data

3	(0.988) 0.982 0.996

2	(0.012) 0.018 0.004

Locus 7227 :

2 alleles

99.2% missing data

3	(0.791) 0.795 0.783

2	(0.209) 0.205 0.217

Locus 7228 :

2 alleles

98.0% missing data

4	(0.777) 0.778 0.784

3	(0.223) 0.222 0.216

Locus 7229 :

2 alleles

99.2% missing data

3	(0.573) 0.565 0.583

1	(0.427) 0.435 0.417

Locus 7230 :

2 alleles

93.8% missing data

1	(0.881) 0.882 0.889

3	(0.119) 0.118 0.111

Locus 7231 :

2 alleles

99.2% missing data

1	(0.584) 0.577 0.589

3	(0.416) 0.423 0.411

Locus 7232 :

2 alleles

99.2% missing data

1	(0.375) 0.378 0.375

4	(0.625) 0.622 0.625

Locus 7233 :

2 alleles

99.2% missing data

4	(0.508) 0.510 0.505

3	(0.492) 0.490 0.495

Locus 7234 :

2 alleles

98.8% missing data

1	(0.730) 0.735 0.727

2	(0.270) 0.265 0.273

Locus 7235 :

2 alleles

99.2% missing data

1	(0.449) 0.445 0.450

4	(0.551) 0.555 0.550

Locus 7236 :

2 alleles

98.0% missing data

1	(0.286) 0.274 0.286

4	(0.714) 0.726 0.714

Locus 7237 :

2 alleles

99.2% missing data

3	(0.812) 0.807 0.815

1	(0.188) 0.193 0.185

Locus 7238 :

2 alleles

99.2% missing data

3	(0.519) 0.516 0.521

2	(0.481) 0.484 0.479

Locus 7239 :

2 alleles

99.2% missing data

4	(0.520) 0.516 0.527

2	(0.480) 0.484 0.473

Locus 7240 :

2 alleles

0.0% missing data

3	(0.987) 0.983 0.996

2	(0.013) 0.017 0.004

Locus 7241 :

2 alleles

99.2% missing data

1	(0.703) 0.707 0.699

2	(0.297) 0.293 0.301

Locus 7242 :

2 alleles

98.8% missing data

4	(0.467) 0.455 0.474

1	(0.533) 0.545 0.526

Locus 7243 :

2 alleles

99.2% missing data

4	(0.425) 0.427 0.430

3	(0.575) 0.573 0.570

Locus 7244 :

2 alleles

99.2% missing data

4	(0.350) 0.359 0.348

1	(0.650) 0.641 0.652

Locus 7245 :

2 alleles

99.2% missing data

1	(0.667) 0.667 0.670

3	(0.333) 0.333 0.330

Locus 7246 :

2 alleles

98.8% missing data

3	(0.462) 0.455 0.471

2	(0.538) 0.545 0.529

Locus 7247 :

2 alleles

99.2% missing data

3	(0.539) 0.540 0.543

1	(0.461) 0.460 0.457

Locus 7248 :

2 alleles

91.0% missing data

1	(0.900) 0.906 0.902

3	(0.100) 0.094 0.098

Locus 7249 :

2 alleles

99.2% missing data

3	(0.144) 0.141 0.146

4	(0.856) 0.859 0.854

Locus 7250 :

2 alleles

98.0% missing data

3	(0.658) 0.659 0.662

1	(0.342) 0.341 0.338

Locus 7251 :

2 alleles

96.9% missing data

4	(0.137) 0.137 0.128

1	(0.863) 0.863 0.872

Locus 7252 :

2 alleles

99.2% missing data

3	(0.774) 0.769 0.778

4	(0.226) 0.231 0.222

Locus 7253 :

2 alleles

99.2% missing data

3	(0.618) 0.624 0.616

2	(0.382) 0.376 0.384

Locus 7254 :

3 alleles

98.8% missing data

1	(0.227) 0.219 0.234

2	(0.409) 0.412 0.414

3	(0.364) 0.369 0.352

Locus 7255 :

2 alleles

99.2% missing data

2	(0.621) 0.608 0.629

3	(0.379) 0.392 0.371

Locus 7256 :

3 alleles

98.8% missing data

1	(0.272) 0.272 0.271

4	(0.260) 0.265 0.258

2	(0.469) 0.463 0.471

Locus 7257 :

2 alleles

98.8% missing data

2	(0.704) 0.696 0.711

1	(0.296) 0.304 0.289

Locus 7258 :

2 alleles

98.8% missing data

3	(0.781) 0.786 0.772

2	(0.219) 0.214 0.228

Locus 7259 :

2 alleles

98.8% missing data

1	(0.458) 0.465 0.458

4	(0.542) 0.535 0.542

Locus 7260 :

2 alleles

98.4% missing data

1	(0.421) 0.420 0.420

3	(0.579) 0.580 0.580

Locus 7261 :

2 alleles

99.2% missing data

1	(0.706) 0.703 0.707

2	(0.294) 0.297 0.293

Locus 7262 :

2 alleles

99.2% missing data

1	(0.629) 0.633 0.625

2	(0.371) 0.367 0.375

Locus 7263 :

2 alleles

98.0% missing data

3	(0.809) 0.803 0.818

2	(0.191) 0.197 0.182

Locus 7264 :

2 alleles

98.8% missing data

1	(0.474) 0.477 0.477

2	(0.526) 0.523 0.523

Locus 7265 :

2 alleles

98.8% missing data

4	(0.625) 0.615 0.628

3	(0.375) 0.385 0.372

Locus 7266 :

2 alleles

98.4% missing data

2	(0.765) 0.769 0.760

3	(0.235) 0.231 0.240

Locus 7267 :

2 alleles

99.2% missing data

1	(0.535) 0.530 0.542

4	(0.465) 0.470 0.458

Locus 7268 :

2 alleles

99.2% missing data

3	(0.663) 0.672 0.652

4	(0.337) 0.328 0.348

Locus 7269 :

2 alleles

99.2% missing data

4	(0.425) 0.421 0.431

1	(0.575) 0.579 0.569

Locus 7270 :

2 alleles

99.2% missing data

1	(0.604) 0.602 0.598

3	(0.396) 0.398 0.402

Locus 7271 :

2 alleles

91.0% missing data

1	(0.910) 0.928 0.900

3	(0.090) 0.072 0.100

Locus 7272 :

2 alleles

98.8% missing data

3	(0.751) 0.750 0.746

2	(0.249) 0.250 0.254

Locus 7273 :

2 alleles

99.2% missing data

3	(0.781) 0.783 0.778

1	(0.219) 0.217 0.222

Locus 7274 :

2 alleles

98.8% missing data

3	(0.350) 0.345 0.359

1	(0.650) 0.655 0.641

Locus 7275 :

2 alleles

98.8% missing data

1	(0.512) 0.516 0.510

2	(0.488) 0.484 0.490

Locus 7276 :

2 alleles

99.2% missing data

4	(0.401) 0.397 0.398

1	(0.599) 0.603 0.602

Locus 7277 :

2 alleles

98.4% missing data

1	(0.698) 0.703 0.689

4	(0.302) 0.297 0.311

Locus 7278 :

2 alleles

99.2% missing data

4	(0.465) 0.467 0.465

1	(0.535) 0.533 0.535

Locus 7279 :

2 alleles

96.1% missing data

1	(0.899) 0.906 0.893

4	(0.101) 0.094 0.107

Locus 7280 :

2 alleles

99.2% missing data

3	(0.689) 0.687 0.689

1	(0.311) 0.313 0.311

Locus 7281 :

2 alleles

99.2% missing data

3	(0.657) 0.657 0.658

2	(0.343) 0.343 0.342

Locus 7282 :

2 alleles

99.2% missing data

1	(0.464) 0.462 0.461

2	(0.536) 0.538 0.539

Locus 7283 :

2 alleles

93.8% missing data

2	(0.914) 0.913 0.919

1	(0.086) 0.087 0.081

Locus 7284 :

2 alleles

99.2% missing data

2	(0.208) 0.208 0.209

3	(0.792) 0.792 0.791

Locus 7285 :

2 alleles

99.2% missing data

3	(0.554) 0.550 0.559

2	(0.446) 0.450 0.441

Locus 7286 :

2 alleles

90.6% missing data

2	(0.914) 0.930 0.906

4	(0.086) 0.070 0.094

Locus 7287 :

2 alleles

91.0% missing data

1	(0.897) 0.912 0.892

4	(0.103) 0.088 0.108

Locus 7288 :

2 alleles

99.2% missing data

1	(0.551) 0.548 0.551

2	(0.449) 0.452 0.449

Locus 7289 :

2 alleles

99.2% missing data

1	(0.532) 0.530 0.533

2	(0.468) 0.470 0.467

Locus 7290 :

2 alleles

99.2% missing data

1	(0.572) 0.570 0.579

2	(0.428) 0.430 0.421

Locus 7291 :

2 alleles

98.8% missing data

2	(0.328) 0.328 0.329

1	(0.672) 0.672 0.671

Locus 7292 :

2 alleles

99.2% missing data

2	(0.787) 0.790 0.784

3	(0.213) 0.210 0.216

Locus 7293 :

2 alleles

99.2% missing data

4	(0.636) 0.636 0.631

1	(0.364) 0.364 0.369

Locus 7294 :

3 alleles

98.8% missing data

3	(0.126) 0.120 0.132

1	(0.266) 0.271 0.262

2	(0.608) 0.609 0.606

Locus 7295 :

2 alleles

99.2% missing data

4	(0.550) 0.542 0.553

3	(0.450) 0.458 0.447

Locus 7296 :

2 alleles

99.2% missing data

4	(0.630) 0.626 0.639

2	(0.370) 0.374 0.361

Locus 7297 :

2 alleles

99.2% missing data

1	(0.542) 0.540 0.545

4	(0.458) 0.460 0.455

Locus 7298 :

2 alleles

98.8% missing data

2	(0.596) 0.589 0.600

3	(0.404) 0.411 0.400

Locus 7299 :

2 alleles

98.8% missing data

4	(0.800) 0.797 0.804

2	(0.200) 0.203 0.196

Locus 7300 :

2 alleles

99.2% missing data

1	(0.599) 0.599 0.596

3	(0.401) 0.401 0.404

Locus 7301 :

2 alleles

99.2% missing data

1	(0.380) 0.375 0.381

3	(0.620) 0.625 0.619

Locus 7302 :

2 alleles

99.2% missing data

4	(0.492) 0.495 0.493

1	(0.508) 0.505 0.507

Locus 7303 :

2 alleles

98.8% missing data

2	(0.545) 0.535 0.552

1	(0.455) 0.465 0.448

Locus 7304 :

2 alleles

98.8% missing data

1	(0.579) 0.585 0.578

4	(0.421) 0.415 0.422

Locus 7305 :

2 alleles

99.2% missing data

1	(0.497) 0.500 0.494

2	(0.503) 0.500 0.506

Locus 7306 :

2 alleles

99.2% missing data

4	(0.423) 0.423 0.420

2	(0.577) 0.577 0.580

Locus 7307 :

2 alleles

98.8% missing data

4	(0.505) 0.491 0.516

2	(0.495) 0.509 0.484

Locus 7308 :

2 alleles

99.2% missing data

2	(0.488) 0.483 0.495

4	(0.512) 0.517 0.505

Locus 7309 :

2 alleles

99.2% missing data

4	(0.292) 0.285 0.292

1	(0.708) 0.715 0.708

Locus 7310 :

2 alleles

98.0% missing data

2	(0.558) 0.561 0.552

1	(0.442) 0.439 0.448

Locus 7311 :

2 alleles

99.2% missing data

4	(0.492) 0.488 0.498

1	(0.508) 0.512 0.502

Locus 7312 :

2 alleles

99.2% missing data

1	(0.493) 0.498 0.500

3	(0.507) 0.502 0.500

Locus 7313 :

2 alleles

99.2% missing data

4	(0.412) 0.406 0.421

2	(0.588) 0.594 0.579

Locus 7314 :

2 alleles

99.2% missing data

1	(0.469) 0.468 0.468

2	(0.531) 0.532 0.532

Locus 7315 :

2 alleles

99.2% missing data

2	(0.833) 0.835 0.829

4	(0.167) 0.165 0.171

Locus 7316 :

2 alleles

98.8% missing data

2	(0.356) 0.347 0.368

3	(0.644) 0.653 0.632

Locus 7317 :

2 alleles

99.2% missing data

3	(0.275) 0.277 0.278

2	(0.725) 0.723 0.722

Locus 7318 :

2 alleles

99.2% missing data

3	(0.371) 0.370 0.376

1	(0.629) 0.630 0.624

Locus 7319 :

2 alleles

99.2% missing data

4	(0.638) 0.643 0.637

3	(0.362) 0.357 0.363

Locus 7320 :

2 alleles

99.2% missing data

3	(0.584) 0.578 0.588

4	(0.416) 0.422 0.412

Locus 7321 :

3 alleles

98.4% missing data

3	(0.216) 0.214 0.220

1	(0.330) 0.332 0.326

2	(0.455) 0.453 0.454

Locus 7322 :

2 alleles

99.2% missing data

2	(0.743) 0.745 0.744

1	(0.257) 0.255 0.256

Locus 7323 :

2 alleles

15.6% missing data

3	(0.984) 0.977 0.995

2	(0.016) 0.023 0.005

Locus 7324 :

2 alleles

99.2% missing data

3	(0.485) 0.483 0.488

4	(0.515) 0.517 0.512

Locus 7325 :

2 alleles

93.0% missing data

1	(0.924) 0.921 0.933

3	(0.076) 0.079 0.067

Locus 7326 :

2 alleles

99.2% missing data

3	(0.504) 0.501 0.502

4	(0.496) 0.499 0.498

Locus 7327 :

2 alleles

93.0% missing data

3	(0.841) 0.845 0.852

4	(0.159) 0.155 0.148

Locus 7328 :

2 alleles

99.2% missing data

3	(0.515) 0.508 0.518

1	(0.485) 0.492 0.482

Locus 7329 :

2 alleles

99.2% missing data

3	(0.559) 0.557 0.559

1	(0.441) 0.443 0.441

Locus 7330 :

2 alleles

99.2% missing data

3	(0.799) 0.797 0.803

4	(0.201) 0.203 0.197

Locus 7331 :

2 alleles

99.2% missing data

1	(0.516) 0.522 0.510

4	(0.484) 0.478 0.490

Locus 7332 :

2 alleles

99.2% missing data

1	(0.370) 0.369 0.375

2	(0.630) 0.631 0.625

Locus 7333 :

2 alleles

99.2% missing data

3	(0.495) 0.492 0.498

1	(0.505) 0.508 0.502

Locus 7334 :

3 alleles

95.7% missing data

2	(0.297) 0.321 0.280

3	(0.503) 0.487 0.521

1	(0.200) 0.192 0.199

Locus 7335 :

2 alleles

99.2% missing data

3	(0.686) 0.677 0.690

2	(0.314) 0.323 0.310

Locus 7336 :

2 alleles

99.2% missing data

4	(0.744) 0.739 0.746

3	(0.256) 0.261 0.254

Locus 7337 :

2 alleles

99.2% missing data

3	(0.176) 0.171 0.185

1	(0.824) 0.829 0.815

Locus 7338 :

2 alleles

91.4% missing data

3	(0.965) 0.967 0.964

2	(0.035) 0.033 0.036

Locus 7339 :

2 alleles

99.2% missing data

3	(0.613) 0.611 0.614

4	(0.387) 0.389 0.386

Locus 7340 :

2 alleles

99.2% missing data

2	(0.568) 0.560 0.569

3	(0.432) 0.440 0.431

Locus 7341 :

2 alleles

99.2% missing data

3	(0.342) 0.346 0.343

4	(0.658) 0.654 0.657

Locus 7342 :

2 alleles

98.8% missing data

3	(0.690) 0.695 0.683

4	(0.310) 0.305 0.317

Locus 7343 :

2 alleles

98.8% missing data

1	(0.631) 0.623 0.637

4	(0.369) 0.377 0.363

Locus 7344 :

2 alleles

99.2% missing data

1	(0.595) 0.596 0.593

3	(0.405) 0.404 0.407

Locus 7345 :

2 alleles

99.2% missing data

1	(0.415) 0.412 0.414

3	(0.585) 0.588 0.586

Locus 7346 :

2 alleles

96.5% missing data

3	(0.862) 0.869 0.855

1	(0.138) 0.131 0.145

Locus 7347 :

2 alleles

99.2% missing data

1	(0.744) 0.734 0.747

2	(0.256) 0.266 0.253

Locus 7348 :

2 alleles

99.2% missing data

4	(0.512) 0.513 0.512

1	(0.488) 0.487 0.488

Locus 7349 :

2 alleles

98.4% missing data

1	(0.440) 0.426 0.450

3	(0.560) 0.574 0.550

Locus 7350 :

2 alleles

98.0% missing data

1	(0.460) 0.457 0.454

3	(0.540) 0.543 0.546

Locus 7351 :

2 alleles

99.2% missing data

3	(0.458) 0.458 0.456

4	(0.542) 0.542 0.544

Locus 7352 :

2 alleles

97.3% missing data

1	(0.708) 0.705 0.713

3	(0.292) 0.295 0.287

Locus 7353 :

2 alleles

99.2% missing data

3	(0.394) 0.393 0.391

1	(0.606) 0.607 0.609

Locus 7354 :

2 alleles

96.5% missing data

1	(0.194) 0.189 0.201

2	(0.806) 0.811 0.799

Locus 7355 :

2 alleles

98.8% missing data

1	(0.303) 0.302 0.305

3	(0.697) 0.698 0.695

Locus 7356 :

2 alleles

99.2% missing data

1	(0.365) 0.370 0.364

3	(0.635) 0.630 0.636

Locus 7357 :

2 alleles

99.2% missing data

1	(0.381) 0.376 0.380

2	(0.619) 0.624 0.620

Locus 7358 :

2 alleles

98.8% missing data

1	(0.595) 0.600 0.588

2	(0.405) 0.400 0.412

Locus 7359 :

2 alleles

98.8% missing data

1	(0.427) 0.422 0.427

2	(0.573) 0.578 0.573

Locus 7360 :

2 alleles

99.2% missing data

1	(0.489) 0.487 0.496

4	(0.511) 0.513 0.504

Locus 7361 :

2 alleles

99.2% missing data

3	(0.436) 0.439 0.437

1	(0.564) 0.561 0.563

Locus 7362 :

2 alleles

99.2% missing data

4	(0.304) 0.297 0.310

3	(0.696) 0.703 0.690

Locus 7363 :

2 alleles

9.4% missing data

1	(0.987) 0.981 0.997

2	(0.013) 0.019 0.003

Locus 7364 :

2 alleles

96.1% missing data

3	(0.603) 0.600 0.606

4	(0.397) 0.400 0.394

Locus 7365 :

2 alleles

99.2% missing data

2	(0.712) 0.707 0.715

1	(0.288) 0.293 0.285

Locus 7366 :

2 alleles

99.2% missing data

2	(0.350) 0.353 0.347

1	(0.650) 0.647 0.653

Locus 7367 :

3 alleles

98.0% missing data

1	(0.393) 0.395 0.397

4	(0.274) 0.286 0.270

3	(0.332) 0.319 0.332

Locus 7368 :

2 alleles

99.2% missing data

3	(0.373) 0.373 0.373

1	(0.627) 0.627 0.627

Locus 7369 :

2 alleles

98.8% missing data

3	(0.443) 0.446 0.448

2	(0.557) 0.554 0.552

Locus 7370 :

2 alleles

98.4% missing data

1	(0.634) 0.638 0.633

3	(0.366) 0.362 0.367

Locus 7371 :

2 alleles

98.8% missing data

2	(0.804) 0.800 0.803

3	(0.196) 0.200 0.197

Locus 7372 :

2 alleles

98.8% missing data

3	(0.581) 0.569 0.588

1	(0.419) 0.431 0.412

Locus 7373 :

2 alleles

95.7% missing data

3	(0.164) 0.157 0.173

1	(0.836) 0.843 0.827

Locus 7374 :

2 alleles

98.8% missing data

1	(0.613) 0.612 0.619

2	(0.387) 0.388 0.381

Locus 7375 :

2 alleles

7.8% missing data

2	(0.976) 0.951 0.993

1	(0.024) 0.049 0.007

Locus 7376 :

2 alleles

99.2% missing data

1	(0.609) 0.609 0.611

4	(0.391) 0.391 0.389

Locus 7377 :

2 alleles

91.4% missing data

1	(0.861) 0.861 0.875

4	(0.139) 0.139 0.125

Locus 7378 :

2 alleles

99.2% missing data

1	(0.571) 0.572 0.568

4	(0.429) 0.428 0.432

Locus 7379 :

2 alleles

98.4% missing data

2	(0.789) 0.798 0.786

1	(0.211) 0.202 0.214

Locus 7380 :

2 alleles

99.2% missing data

1	(0.342) 0.336 0.345

4	(0.658) 0.664 0.655

Locus 7381 :

2 alleles

7.8% missing data

2	(0.988) 0.990 0.994

1	(0.012) 0.010 0.006

Locus 7382 :

2 alleles

97.7% missing data

1	(0.601) 0.612 0.602

4	(0.399) 0.388 0.398

Locus 7383 :

2 alleles

99.2% missing data

2	(0.733) 0.723 0.741

1	(0.267) 0.277 0.259

Locus 7384 :

2 alleles

99.2% missing data

3	(0.749) 0.752 0.750

1	(0.251) 0.248 0.250

Locus 7385 :

2 alleles

99.2% missing data

2	(0.624) 0.625 0.625

1	(0.376) 0.375 0.375

Locus 7386 :

2 alleles

99.2% missing data

2	(0.491) 0.486 0.498

4	(0.509) 0.514 0.502

Locus 7387 :

2 alleles

99.2% missing data

4	(0.501) 0.508 0.491

2	(0.499) 0.492 0.509

Locus 7388 :

2 alleles

99.2% missing data

3	(0.459) 0.450 0.466

4	(0.541) 0.550 0.534

Locus 7389 :

2 alleles

99.2% missing data

1	(0.742) 0.735 0.745

4	(0.258) 0.265 0.255

Locus 7390 :

2 alleles

98.8% missing data

2	(0.631) 0.633 0.632

4	(0.369) 0.367 0.368

Locus 7391 :

2 alleles

99.2% missing data

2	(0.381) 0.379 0.381

1	(0.619) 0.621 0.619

Locus 7392 :

2 alleles

99.2% missing data

2	(0.332) 0.336 0.335

1	(0.668) 0.664 0.665

Locus 7393 :

2 alleles

96.5% missing data

1	(0.803) 0.804 0.809

3	(0.197) 0.196 0.191

Locus 7394 :

2 alleles

99.2% missing data

1	(0.482) 0.476 0.488

2	(0.518) 0.524 0.512

Locus 7395 :

2 alleles

99.2% missing data

3	(0.502) 0.496 0.510

4	(0.498) 0.504 0.490

Locus 7396 :

2 alleles

99.2% missing data

4	(0.424) 0.427 0.429

3	(0.576) 0.573 0.571

Locus 7397 :

2 alleles

99.2% missing data

4	(0.731) 0.726 0.733

3	(0.269) 0.274 0.267

Locus 7398 :

2 alleles

99.2% missing data

1	(0.403) 0.402 0.397

4	(0.597) 0.598 0.603

Locus 7399 :

2 alleles

99.2% missing data

2	(0.627) 0.619 0.627

1	(0.373) 0.381 0.373

Locus 7400 :

2 alleles

99.2% missing data

3	(0.381) 0.382 0.384

2	(0.619) 0.618 0.616

Locus 7401 :

2 alleles

99.2% missing data

1	(0.230) 0.230 0.233

3	(0.770) 0.770 0.767

Locus 7402 :

2 alleles

96.9% missing data

2	(0.803) 0.804 0.807

3	(0.197) 0.196 0.193

Locus 7403 :

2 alleles

99.2% missing data

2	(0.701) 0.704 0.697

4	(0.299) 0.296 0.303

Locus 7404 :

2 alleles

99.2% missing data

3	(0.549) 0.551 0.547

1	(0.451) 0.449 0.453

Locus 7405 :

2 alleles

99.2% missing data

2	(0.555) 0.553 0.561

1	(0.445) 0.447 0.439

Locus 7406 :

2 alleles

98.8% missing data

2	(0.724) 0.726 0.724

3	(0.276) 0.274 0.276

Locus 7407 :

2 alleles

98.8% missing data

3	(0.734) 0.734 0.731

2	(0.266) 0.266 0.269

Locus 7408 :

2 alleles

98.4% missing data

2	(0.330) 0.331 0.324

1	(0.670) 0.669 0.676

Locus 7409 :

2 alleles

99.2% missing data

3	(0.378) 0.375 0.390

4	(0.622) 0.625 0.610

Locus 7410 :

2 alleles

98.8% missing data

3	(0.347) 0.343 0.349

1	(0.653) 0.657 0.651

Locus 7411 :

2 alleles

98.0% missing data

2	(0.678) 0.674 0.691

3	(0.322) 0.326 0.309

Locus 7412 :

2 alleles

98.0% missing data

1	(0.825) 0.830 0.822

3	(0.175) 0.170 0.178

Locus 7413 :

2 alleles

99.2% missing data

1	(0.638) 0.633 0.649

4	(0.362) 0.367 0.351

Locus 7414 :

2 alleles

98.4% missing data

3	(0.300) 0.293 0.307

1	(0.700) 0.707 0.693

Locus 7415 :

2 alleles

98.8% missing data

4	(0.604) 0.606 0.599

3	(0.396) 0.394 0.401

Locus 7416 :

2 alleles

99.2% missing data

3	(0.558) 0.558 0.557

4	(0.442) 0.442 0.443

Locus 7417 :

2 alleles

99.2% missing data

2	(0.496) 0.491 0.502

4	(0.504) 0.509 0.498

Locus 7418 :

2 alleles

99.2% missing data

3	(0.594) 0.590 0.591

1	(0.406) 0.410 0.409

Locus 7419 :

2 alleles

99.2% missing data

3	(0.668) 0.663 0.673

2	(0.332) 0.337 0.327

Locus 7420 :

2 alleles

99.2% missing data

4	(0.398) 0.400 0.398

3	(0.602) 0.600 0.602

Locus 7421 :

2 alleles

98.8% missing data

2	(0.475) 0.467 0.476

3	(0.525) 0.533 0.524

Locus 7422 :

2 alleles

98.8% missing data

4	(0.534) 0.535 0.542

3	(0.466) 0.465 0.458

Locus 7423 :

2 alleles

98.8% missing data

1	(0.358) 0.363 0.346

2	(0.642) 0.637 0.654

Locus 7424 :

2 alleles

99.2% missing data

1	(0.658) 0.653 0.662

2	(0.342) 0.347 0.338

Locus 7425 :

2 alleles

92.6% missing data

3	(0.895) 0.887 0.913

2	(0.105) 0.113 0.087

Locus 7426 :

2 alleles

99.2% missing data

3	(0.785) 0.787 0.779

2	(0.215) 0.213 0.221

Locus 7427 :

2 alleles

99.2% missing data

1	(0.434) 0.435 0.441

2	(0.566) 0.565 0.559

Locus 7428 :

2 alleles

96.9% missing data

4	(0.212) 0.197 0.229

1	(0.788) 0.803 0.771

Locus 7429 :

3 alleles

98.8% missing data

1	(0.406) 0.408 0.403

2	(0.360) 0.358 0.363

3	(0.234) 0.233 0.234

Locus 7430 :

2 alleles

98.4% missing data

2	(0.450) 0.456 0.446

3	(0.550) 0.544 0.554

Locus 7431 :

2 alleles

99.2% missing data

3	(0.727) 0.727 0.727

1	(0.273) 0.273 0.273

Locus 7432 :

2 alleles

99.2% missing data

1	(0.342) 0.337 0.351

3	(0.658) 0.663 0.649

Locus 7433 :

2 alleles

95.7% missing data

1	(0.877) 0.866 0.889

2	(0.123) 0.134 0.111

Locus 7434 :

2 alleles

99.2% missing data

1	(0.500) 0.509 0.495

2	(0.500) 0.491 0.505

Locus 7435 :

3 alleles

97.7% missing data

3	(0.660) 0.654 0.674

1	(0.166) 0.162 0.166

2	(0.173) 0.185 0.160

Locus 7436 :

2 alleles

98.8% missing data

3	(0.460) 0.454 0.470

4	(0.540) 0.546 0.530

Locus 7437 :

2 alleles

99.2% missing data

4	(0.627) 0.628 0.626

3	(0.373) 0.372 0.374

Locus 7438 :

2 alleles

98.8% missing data

3	(0.602) 0.604 0.602

4	(0.398) 0.396 0.398

Locus 7439 :

3 alleles

95.7% missing data

2	(0.578) 0.574 0.598

1	(0.109) 0.107 0.112

3	(0.312) 0.318 0.290

Locus 7440 :

2 alleles

97.7% missing data

2	(0.718) 0.718 0.720

1	(0.282) 0.282 0.280

Locus 7441 :

2 alleles

98.8% missing data

4	(0.426) 0.434 0.414

2	(0.574) 0.566 0.586

Locus 7442 :

2 alleles

91.8% missing data

1	(0.713) 0.710 0.716

2	(0.287) 0.290 0.284

Locus 7443 :

2 alleles

99.2% missing data

2	(0.505) 0.501 0.504

4	(0.495) 0.499 0.496

Locus 7444 :

2 alleles

98.8% missing data

2	(0.728) 0.720 0.738

1	(0.272) 0.280 0.262

Locus 7445 :

2 alleles

97.7% missing data

1	(0.684) 0.689 0.684

4	(0.316) 0.311 0.316

Locus 7446 :

2 alleles

99.2% missing data

2	(0.642) 0.643 0.638

1	(0.358) 0.357 0.362

Locus 7447 :

2 alleles

99.2% missing data

3	(0.614) 0.610 0.616

4	(0.386) 0.390 0.384

Locus 7448 :

2 alleles

98.8% missing data

1	(0.404) 0.407 0.396

4	(0.596) 0.593 0.604

Locus 7449 :

2 alleles

99.2% missing data

3	(0.181) 0.184 0.177

1	(0.819) 0.816 0.823

Locus 7450 :

2 alleles

99.2% missing data

1	(0.509) 0.505 0.507

3	(0.491) 0.495 0.493

Locus 7451 :

2 alleles

96.1% missing data

2	(0.869) 0.875 0.864

3	(0.131) 0.125 0.136

Locus 7452 :

2 alleles

92.6% missing data

1	(0.915) 0.909 0.926

3	(0.085) 0.091 0.074

Locus 7453 :

2 alleles

99.2% missing data

1	(0.411) 0.414 0.411

3	(0.589) 0.586 0.589

Locus 7454 :

2 alleles

99.2% missing data

1	(0.376) 0.375 0.371

3	(0.624) 0.625 0.629

Locus 7455 :

2 alleles

98.4% missing data

1	(0.682) 0.685 0.677

4	(0.318) 0.315 0.323

Locus 7456 :

3 alleles

98.4% missing data

3	(0.393) 0.391 0.399

1	(0.334) 0.327 0.334

4	(0.273) 0.282 0.267

Locus 7457 :

2 alleles

99.2% missing data

2	(0.634) 0.638 0.629

1	(0.366) 0.362 0.371

Locus 7458 :

2 alleles

99.2% missing data

4	(0.733) 0.736 0.728

1	(0.267) 0.264 0.272

Locus 7459 :

2 alleles

99.2% missing data

3	(0.300) 0.300 0.295

1	(0.700) 0.700 0.705

Locus 7460 :

2 alleles

99.2% missing data

3	(0.376) 0.372 0.384

1	(0.624) 0.628 0.616

Locus 7461 :

2 alleles

99.2% missing data

1	(0.246) 0.248 0.244

4	(0.754) 0.752 0.756

Locus 7462 :

2 alleles

99.2% missing data

4	(0.603) 0.606 0.595

1	(0.397) 0.394 0.405

Locus 7463 :

2 alleles

98.8% missing data

3	(0.765) 0.765 0.763

2	(0.235) 0.235 0.237

Locus 7464 :

2 alleles

98.0% missing data

1	(0.328) 0.332 0.316

2	(0.672) 0.668 0.684

Locus 7465 :

2 alleles

99.2% missing data

2	(0.375) 0.367 0.383

1	(0.625) 0.633 0.617

Locus 7466 :

2 alleles

89.5% missing data

2	(0.782) 0.821 0.742

4	(0.218) 0.179 0.258

Locus 7467 :

2 alleles

98.8% missing data

2	(0.444) 0.435 0.449

4	(0.556) 0.565 0.551

Locus 7468 :

2 alleles

98.4% missing data

2	(0.573) 0.562 0.588

1	(0.427) 0.438 0.412

Locus 7469 :

3 alleles

98.8% missing data

3	(0.399) 0.407 0.394

4	(0.426) 0.421 0.422

2	(0.175) 0.172 0.184

Locus 7470 :

2 alleles

98.8% missing data

1	(0.629) 0.628 0.634

4	(0.371) 0.372 0.366

Locus 7471 :

2 alleles

99.2% missing data

4	(0.757) 0.761 0.754

2	(0.243) 0.239 0.246

Locus 7472 :

2 alleles

98.8% missing data

3	(0.483) 0.482 0.489

4	(0.517) 0.518 0.511

Locus 7473 :

2 alleles

98.8% missing data

2	(0.490) 0.485 0.496

1	(0.510) 0.515 0.504

Locus 7474 :

3 alleles

98.4% missing data

3	(0.391) 0.401 0.383

2	(0.358) 0.351 0.360

1	(0.251) 0.248 0.257

Locus 7475 :

2 alleles

99.2% missing data

2	(0.229) 0.230 0.226

3	(0.771) 0.770 0.774

Locus 7476 :

2 alleles

98.8% missing data

2	(0.268) 0.274 0.268

4	(0.732) 0.726 0.732

Locus 7477 :

2 alleles

98.8% missing data

4	(0.471) 0.472 0.474

3	(0.529) 0.528 0.526

Locus 7478 :

2 alleles

99.2% missing data

3	(0.476) 0.474 0.478

4	(0.524) 0.526 0.522

Locus 7479 :

2 alleles

8.2% missing data

3	(0.979) 0.970 0.993

2	(0.021) 0.030 0.007

Locus 7480 :

2 alleles

99.2% missing data

1	(0.756) 0.761 0.751

2	(0.244) 0.239 0.249

Locus 7481 :

2 alleles

98.8% missing data

3	(0.413) 0.412 0.422

1	(0.587) 0.588 0.578

Locus 7482 :

2 alleles

99.2% missing data

2	(0.443) 0.439 0.451

4	(0.557) 0.561 0.549

Locus 7483 :

2 alleles

99.2% missing data

3	(0.506) 0.509 0.502

4	(0.494) 0.491 0.498

Locus 7484 :

2 alleles

99.2% missing data

1	(0.656) 0.658 0.647

4	(0.344) 0.342 0.353

Locus 7485 :

2 alleles

99.2% missing data

4	(0.601) 0.599 0.601

3	(0.399) 0.401 0.399

Locus 7486 :

2 alleles

99.2% missing data

1	(0.617) 0.615 0.615

4	(0.383) 0.385 0.385

Locus 7487 :

2 alleles

99.2% missing data

1	(0.389) 0.384 0.395

3	(0.611) 0.616 0.605

Locus 7488 :

2 alleles

98.8% missing data

1	(0.400) 0.394 0.405

3	(0.600) 0.606 0.595

Locus 7489 :

2 alleles

99.2% missing data

1	(0.321) 0.323 0.324

3	(0.679) 0.677 0.676

Locus 7490 :

3 alleles

98.8% missing data

1	(0.297) 0.296 0.299

3	(0.366) 0.370 0.361

2	(0.337) 0.334 0.340

Locus 7491 :

2 alleles

99.2% missing data

2	(0.336) 0.328 0.338

4	(0.664) 0.672 0.662

Locus 7492 :

2 alleles

98.8% missing data

4	(0.339) 0.331 0.342

1	(0.661) 0.669 0.658

Locus 7493 :

2 alleles

99.2% missing data

2	(0.563) 0.562 0.560

1	(0.437) 0.438 0.440

Locus 7494 :

2 alleles

97.3% missing data

2	(0.185) 0.186 0.179

4	(0.815) 0.814 0.821

Locus 7495 :

2 alleles

99.2% missing data

3	(0.546) 0.551 0.540

4	(0.454) 0.449 0.460

Locus 7496 :

2 alleles

99.2% missing data

2	(0.354) 0.359 0.360

1	(0.646) 0.641 0.640

Locus 7497 :

2 alleles

99.2% missing data

4	(0.661) 0.650 0.673

1	(0.339) 0.350 0.327

Locus 7498 :

2 alleles

99.2% missing data

2	(0.783) 0.789 0.779

4	(0.217) 0.211 0.221

Locus 7499 :

2 alleles

98.8% missing data

3	(0.277) 0.270 0.281

4	(0.723) 0.730 0.719

Locus 7500 :

2 alleles

99.2% missing data

2	(0.311) 0.322 0.303

1	(0.689) 0.678 0.697

Locus 7501 :

2 alleles

99.2% missing data

4	(0.628) 0.626 0.634

1	(0.372) 0.374 0.366

Locus 7502 :

2 alleles

99.2% missing data

1	(0.612) 0.606 0.612

2	(0.388) 0.394 0.388

Locus 7503 :

2 alleles

99.2% missing data

1	(0.435) 0.442 0.429

3	(0.565) 0.558 0.571

Locus 7504 :

2 alleles

99.2% missing data

4	(0.603) 0.605 0.599

1	(0.397) 0.395 0.401

Locus 7505 :

2 alleles

99.2% missing data

1	(0.452) 0.445 0.454

3	(0.548) 0.555 0.546

Locus 7506 :

2 alleles

93.0% missing data

4	(0.868) 0.858 0.887

2	(0.132) 0.142 0.113

Locus 7507 :

2 alleles

99.2% missing data

2	(0.665) 0.669 0.661

1	(0.335) 0.331 0.339

Locus 7508 :

2 alleles

99.2% missing data

1	(0.642) 0.635 0.645

2	(0.358) 0.365 0.355

Locus 7509 :

2 alleles

96.5% missing data

1	(0.866) 0.868 0.871

2	(0.134) 0.132 0.129

Locus 7510 :

2 alleles

99.2% missing data

3	(0.756) 0.754 0.755

1	(0.244) 0.246 0.245

Locus 7511 :

2 alleles

99.2% missing data

4	(0.533) 0.540 0.524

2	(0.467) 0.460 0.476

Locus 7512 :

2 alleles

99.2% missing data

3	(0.380) 0.379 0.381

4	(0.620) 0.621 0.619

Locus 7513 :

2 alleles

8.2% missing data

3	(0.980) 0.972 0.994

4	(0.020) 0.028 0.006

Locus 7514 :

2 alleles

90.6% missing data

3	(0.903) 0.922 0.892

1	(0.097) 0.078 0.108

Locus 7515 :

2 alleles

98.8% missing data

4	(0.550) 0.545 0.552

3	(0.450) 0.455 0.448

Locus 7516 :

2 alleles

99.2% missing data

2	(0.707) 0.712 0.705

3	(0.293) 0.288 0.295

Locus 7517 :

2 alleles

99.2% missing data

3	(0.486) 0.482 0.487

1	(0.514) 0.518 0.513

Locus 7518 :

2 alleles

98.8% missing data

1	(0.534) 0.531 0.535

2	(0.466) 0.469 0.465

Locus 7519 :

3 alleles

97.7% missing data

3	(0.532) 0.536 0.527

2	(0.233) 0.238 0.230

1	(0.235) 0.226 0.242

Locus 7520 :

2 alleles

98.8% missing data

3	(0.604) 0.599 0.610

2	(0.396) 0.401 0.390

Locus 7521 :

2 alleles

99.2% missing data

3	(0.489) 0.482 0.494

2	(0.511) 0.518 0.506

Locus 7522 :

2 alleles

98.0% missing data

1	(0.577) 0.576 0.589

2	(0.423) 0.424 0.411

Locus 7523 :

2 alleles

98.0% missing data

1	(0.626) 0.638 0.635

3	(0.374) 0.362 0.365

Locus 7524 :

2 alleles

99.2% missing data

2	(0.620) 0.614 0.620

1	(0.380) 0.386 0.380

Locus 7525 :

2 alleles

99.2% missing data

4	(0.647) 0.654 0.640

2	(0.353) 0.346 0.360

Locus 7526 :

3 alleles

98.8% missing data

2	(0.304) 0.307 0.305

1	(0.461) 0.462 0.460

4	(0.235) 0.231 0.235

Locus 7527 :

2 alleles

99.2% missing data

3	(0.358) 0.355 0.364

4	(0.642) 0.645 0.636

Locus 7528 :

2 alleles

99.2% missing data

1	(0.411) 0.407 0.407

3	(0.589) 0.593 0.593

Locus 7529 :

2 alleles

99.2% missing data

3	(0.507) 0.504 0.504

4	(0.493) 0.496 0.496

Locus 7530 :

2 alleles

99.2% missing data

2	(0.292) 0.293 0.293

1	(0.708) 0.707 0.707

Locus 7531 :

2 alleles

91.8% missing data

1	(0.919) 0.929 0.911

3	(0.081) 0.071 0.089

Locus 7532 :

2 alleles

98.4% missing data

2	(0.663) 0.670 0.667

3	(0.337) 0.330 0.333

Locus 7533 :

2 alleles

99.2% missing data

2	(0.437) 0.443 0.433

4	(0.563) 0.557 0.567

Locus 7534 :

2 alleles

9.4% missing data

2	(0.987) 0.991 0.993

3	(0.013) 0.009 0.007

Locus 7535 :

2 alleles

98.4% missing data

2	(0.590) 0.599 0.584

1	(0.410) 0.401 0.416

Locus 7536 :

2 alleles

99.2% missing data

1	(0.570) 0.573 0.569

3	(0.430) 0.427 0.431

Locus 7537 :

2 alleles

98.8% missing data

3	(0.656) 0.653 0.662

2	(0.344) 0.347 0.338

Locus 7538 :

2 alleles

99.2% missing data

1	(0.348) 0.346 0.350

3	(0.652) 0.654 0.650

Locus 7539 :

2 alleles

98.4% missing data

3	(0.584) 0.584 0.578

1	(0.416) 0.416 0.422

Locus 7540 :

2 alleles

99.2% missing data

3	(0.605) 0.602 0.599

2	(0.395) 0.398 0.401

Locus 7541 :

2 alleles

98.8% missing data

4	(0.717) 0.718 0.721

3	(0.283) 0.282 0.279

Locus 7542 :

2 alleles

97.3% missing data

3	(0.795) 0.796 0.800

1	(0.205) 0.204 0.200

Locus 7543 :

2 alleles

99.2% missing data

2	(0.598) 0.594 0.605

4	(0.402) 0.406 0.395

Locus 7544 :

2 alleles

98.0% missing data

4	(0.327) 0.328 0.321

3	(0.673) 0.672 0.679

Locus 7545 :

3 alleles

98.8% missing data

3	(0.439) 0.440 0.442

4	(0.126) 0.130 0.121

1	(0.435) 0.430 0.437

Locus 7546 :

2 alleles

99.2% missing data

1	(0.652) 0.650 0.650

3	(0.348) 0.350 0.350

Locus 7547 :

2 alleles

91.0% missing data

2	(0.904) 0.915 0.906

3	(0.096) 0.085 0.094

Locus 7548 :

2 alleles

99.2% missing data

1	(0.773) 0.775 0.767

3	(0.227) 0.225 0.233

Locus 7549 :

2 alleles

99.2% missing data

4	(0.527) 0.527 0.526

2	(0.473) 0.473 0.474

Locus 7550 :

3 alleles

98.8% missing data

3	(0.151) 0.155 0.156

2	(0.563) 0.554 0.566

4	(0.285) 0.291 0.278

Locus 7551 :

2 alleles

99.2% missing data

1	(0.527) 0.523 0.530

4	(0.473) 0.477 0.470

Locus 7552 :

2 alleles

99.2% missing data

3	(0.763) 0.763 0.762

2	(0.237) 0.237 0.238

Locus 7553 :

2 alleles

99.2% missing data

3	(0.359) 0.363 0.359

1	(0.641) 0.637 0.641

Locus 7554 :

2 alleles

99.2% missing data

2	(0.278) 0.278 0.275

1	(0.722) 0.722 0.725

Locus 7555 :

2 alleles

99.2% missing data

2	(0.703) 0.696 0.707

3	(0.297) 0.304 0.293

Locus 7556 :

2 alleles

99.2% missing data

4	(0.417) 0.408 0.420

1	(0.583) 0.592 0.580

Locus 7557 :

2 alleles

98.8% missing data

3	(0.799) 0.793 0.804

1	(0.201) 0.207 0.196

Locus 7558 :

2 alleles

99.2% missing data

2	(0.609) 0.610 0.611

3	(0.391) 0.390 0.389

Locus 7559 :

2 alleles

98.4% missing data

1	(0.803) 0.799 0.808

2	(0.197) 0.201 0.192

Locus 7560 :

4 alleles

98.0% missing data

1	(0.218) 0.215 0.224

2	(0.299) 0.308 0.302

3	(0.176) 0.170 0.179

4	(0.307) 0.307 0.296

Locus 7561 :

2 alleles

98.4% missing data

3	(0.745) 0.750 0.741

4	(0.255) 0.250 0.259

Locus 7562 :

2 alleles

99.2% missing data

4	(0.441) 0.439 0.443

3	(0.559) 0.561 0.557

Locus 7563 :

3 alleles

98.4% missing data

2	(0.179) 0.184 0.176

1	(0.365) 0.357 0.378

3	(0.456) 0.459 0.446

Locus 7564 :

2 alleles

83.2% missing data

3	(0.499) 0.556 0.437

2	(0.501) 0.444 0.563

Locus 7565 :

2 alleles

99.2% missing data

1	(0.270) 0.262 0.276

2	(0.730) 0.738 0.724

Locus 7566 :

2 alleles

98.8% missing data

3	(0.580) 0.575 0.590

4	(0.420) 0.425 0.410

Locus 7567 :

2 alleles

99.2% missing data

1	(0.224) 0.221 0.230

3	(0.776) 0.779 0.770

Locus 7568 :

2 alleles

99.2% missing data

4	(0.375) 0.372 0.386

3	(0.625) 0.628 0.614

Locus 7569 :

2 alleles

98.0% missing data

3	(0.741) 0.735 0.750

1	(0.259) 0.265 0.250

Locus 7570 :

2 alleles

1.2% missing data

2	(0.988) 0.987 0.996

1	(0.012) 0.013 0.004

Locus 7571 :

2 alleles

98.8% missing data

3	(0.451) 0.458 0.445

2	(0.549) 0.542 0.555

Locus 7572 :

2 alleles

99.2% missing data

1	(0.609) 0.605 0.613

2	(0.391) 0.395 0.387

Locus 7573 :

3 alleles

96.1% missing data

3	(0.606) 0.624 0.612

4	(0.306) 0.287 0.304

1	(0.088) 0.089 0.084

Locus 7574 :

2 alleles

99.2% missing data

4	(0.393) 0.395 0.388

1	(0.607) 0.605 0.612

Locus 7575 :

2 alleles

92.2% missing data

3	(0.824) 0.797 0.852

1	(0.176) 0.203 0.148

Locus 7576 :

2 alleles

99.2% missing data

3	(0.510) 0.505 0.507

1	(0.490) 0.495 0.493

Locus 7577 :

2 alleles

99.2% missing data

3	(0.701) 0.707 0.694

4	(0.299) 0.293 0.306

Locus 7578 :

2 alleles

99.2% missing data

3	(0.782) 0.777 0.787

1	(0.218) 0.223 0.213

Locus 7579 :

2 alleles

99.2% missing data

1	(0.722) 0.724 0.725

3	(0.278) 0.276 0.275

Locus 7580 :

2 alleles

98.8% missing data

3	(0.520) 0.525 0.526

1	(0.480) 0.475 0.474

Locus 7581 :

2 alleles

99.2% missing data

3	(0.726) 0.729 0.723

2	(0.274) 0.271 0.277

Locus 7582 :

2 alleles

99.2% missing data

4	(0.567) 0.561 0.579

3	(0.433) 0.439 0.421

Locus 7583 :

2 alleles

46.5% missing data

1	(0.972) 0.973 0.983

3	(0.028) 0.027 0.017

Locus 7584 :

2 alleles

32.4% missing data

3	(0.969) 0.942 0.987

2	(0.031) 0.058 0.013

Locus 7585 :

3 alleles

98.8% missing data

2	(0.393) 0.402 0.389

1	(0.179) 0.169 0.186

4	(0.428) 0.429 0.424

Locus 7586 :

2 alleles

99.2% missing data

3	(0.222) 0.223 0.223

2	(0.778) 0.777 0.777

Locus 7587 :

2 alleles

99.2% missing data

3	(0.260) 0.265 0.260

2	(0.740) 0.735 0.740

Locus 7588 :

2 alleles

99.2% missing data

3	(0.570) 0.570 0.571

4	(0.430) 0.430 0.429

Locus 7589 :

2 alleles

99.2% missing data

2	(0.245) 0.247 0.243

3	(0.755) 0.753 0.757

Locus 7590 :

2 alleles

98.0% missing data

3	(0.687) 0.692 0.686

1	(0.313) 0.308 0.314

Locus 7591 :

2 alleles

98.8% missing data

1	(0.284) 0.284 0.284

3	(0.716) 0.716 0.716

Locus 7592 :

2 alleles

91.0% missing data

2	(0.887) 0.904 0.884

3	(0.113) 0.096 0.116

Locus 7593 :

2 alleles

99.2% missing data

4	(0.749) 0.748 0.749

2	(0.251) 0.252 0.251

Locus 7594 :

2 alleles

99.2% missing data

1	(0.615) 0.620 0.614

2	(0.385) 0.380 0.386

Locus 7595 :

2 alleles

98.8% missing data

1	(0.502) 0.505 0.498

2	(0.498) 0.495 0.502

Locus 7596 :

3 alleles

98.4% missing data

3	(0.451) 0.443 0.452

1	(0.360) 0.368 0.354

2	(0.189) 0.189 0.194

Locus 7597 :

2 alleles

98.0% missing data

2	(0.891) 0.886 0.896

3	(0.109) 0.114 0.104

Locus 7598 :

2 alleles

98.8% missing data

3	(0.747) 0.742 0.751

4	(0.253) 0.258 0.249

Locus 7599 :

2 alleles

98.8% missing data

1	(0.612) 0.608 0.619

3	(0.388) 0.392 0.381

Locus 7600 :

2 alleles

98.8% missing data

3	(0.353) 0.355 0.352

1	(0.647) 0.645 0.648

Locus 7601 :

2 alleles

99.2% missing data

3	(0.641) 0.645 0.641

4	(0.359) 0.355 0.359

Locus 7602 :

2 alleles

98.8% missing data

2	(0.745) 0.740 0.740

3	(0.255) 0.260 0.260

Locus 7603 :

2 alleles

93.8% missing data

3	(0.850) 0.848 0.865

2	(0.150) 0.152 0.135

Locus 7604 :

2 alleles

96.5% missing data

1	(0.918) 0.922 0.914

4	(0.082) 0.078 0.086

Locus 7605 :

2 alleles

11.7% missing data

1	(0.982) 0.984 0.991

2	(0.018) 0.016 0.009

Locus 7606 :

2 alleles

98.0% missing data

1	(0.750) 0.755 0.744

3	(0.250) 0.245 0.256

Locus 7607 :

2 alleles

98.8% missing data

1	(0.418) 0.415 0.418

3	(0.582) 0.585 0.582

Locus 7608 :

2 alleles

99.2% missing data

3	(0.521) 0.524 0.525

1	(0.479) 0.476 0.475

Locus 7609 :

2 alleles

99.2% missing data

3	(0.666) 0.667 0.662

4	(0.334) 0.333 0.338

Locus 7610 :

2 alleles

89.5% missing data

1	(0.676) 0.688 0.668

2	(0.324) 0.312 0.332

Locus 7611 :

3 alleles

98.8% missing data

4	(0.167) 0.163 0.176

2	(0.333) 0.333 0.334

1	(0.500) 0.504 0.490

Locus 7612 :

4 alleles

98.4% missing data

3	(0.170) 0.167 0.172

4	(0.430) 0.427 0.428

2	(0.207) 0.215 0.205

1	(0.192) 0.190 0.194

Locus 7613 :

2 alleles

97.7% missing data

2	(0.339) 0.340 0.327

1	(0.661) 0.660 0.673

Locus 7614 :

2 alleles

97.3% missing data

3	(0.396) 0.395 0.394

1	(0.604) 0.605 0.606

Locus 7615 :

2 alleles

78.1% missing data

1	(0.858) 0.855 0.870

2	(0.142) 0.145 0.130

Locus 7616 :

2 alleles

14.1% missing data

3	(0.983) 0.985 0.990

1	(0.017) 0.015 0.010

Locus 7617 :

2 alleles

98.0% missing data

3	(0.186) 0.184 0.181

1	(0.814) 0.816 0.819

Locus 7618 :

2 alleles

97.7% missing data

2	(0.100) 0.098 0.103

1	(0.900) 0.902 0.897

Locus 7619 :

2 alleles

99.2% missing data

4	(0.397) 0.407 0.392

2	(0.603) 0.593 0.608

Locus 7620 :

2 alleles

99.2% missing data

1	(0.765) 0.758 0.770

4	(0.235) 0.242 0.230

Locus 7621 :

3 alleles

98.4% missing data

4	(0.160) 0.157 0.159

1	(0.588) 0.592 0.585

2	(0.253) 0.251 0.256

Locus 7622 :

3 alleles

98.8% missing data

2	(0.411) 0.403 0.416

3	(0.258) 0.265 0.252

1	(0.331) 0.332 0.331

Locus 7623 :

2 alleles

99.2% missing data

1	(0.259) 0.258 0.263

3	(0.741) 0.742 0.737

Locus 7624 :

2 alleles

93.8% missing data

1	(0.708) 0.718 0.701

2	(0.292) 0.282 0.299

Locus 7625 :

3 alleles

90.2% missing data

1	(0.541) 0.542 0.565

2	(0.382) 0.375 0.371

3	(0.077) 0.083 0.064

Locus 7626 :

2 alleles

93.8% missing data

2	(0.456) 0.467 0.448

1	(0.544) 0.533 0.552

Locus 7627 :

2 alleles

99.2% missing data

1	(0.206) 0.201 0.210

2	(0.794) 0.799 0.790

Locus 7628 :

2 alleles

95.3% missing data

1	(0.536) 0.532 0.551

2	(0.464) 0.468 0.449

Locus 7629 :

3 alleles

96.1% missing data

1	(0.318) 0.332 0.310

2	(0.514) 0.507 0.522

4	(0.168) 0.161 0.167

Locus 7630 :

2 alleles

89.5% missing data

1	(0.727) 0.735 0.720

2	(0.273) 0.265 0.280

Locus 7631 :

2 alleles

98.8% missing data

2	(0.413) 0.410 0.417

1	(0.587) 0.590 0.583

Locus 7632 :

2 alleles

98.0% missing data

1	(0.507) 0.506 0.505

2	(0.493) 0.494 0.495

Locus 7633 :

2 alleles

98.8% missing data

1	(0.555) 0.562 0.551

2	(0.445) 0.438 0.449

Locus 7634 :

2 alleles

99.2% missing data

3	(0.406) 0.403 0.413

4	(0.594) 0.597 0.587

Locus 7635 :

2 alleles

91.0% missing data

2	(0.915) 0.920 0.917

3	(0.085) 0.080 0.083

Locus 7636 :

2 alleles

96.9% missing data

1	(0.873) 0.874 0.874

4	(0.127) 0.126 0.126

Locus 7637 :

2 alleles

99.2% missing data

3	(0.380) 0.374 0.386

1	(0.620) 0.626 0.614

Locus 7638 :

2 alleles

99.2% missing data

4	(0.543) 0.536 0.553

1	(0.457) 0.464 0.447

Locus 7639 :

2 alleles

99.2% missing data

2	(0.393) 0.389 0.397

4	(0.607) 0.611 0.603

Locus 7640 :

2 alleles

99.2% missing data

1	(0.343) 0.342 0.344

3	(0.657) 0.658 0.656

Locus 7641 :

2 alleles

99.2% missing data

1	(0.487) 0.484 0.485

2	(0.513) 0.516 0.515

Locus 7642 :

2 alleles

96.5% missing data

2	(0.828) 0.834 0.836

4	(0.172) 0.166 0.164

Locus 7643 :

2 alleles

99.2% missing data

1	(0.371) 0.373 0.375

4	(0.629) 0.627 0.625

Locus 7644 :

3 alleles

96.9% missing data

2	(0.603) 0.591 0.625

3	(0.243) 0.251 0.231

1	(0.154) 0.158 0.144

Locus 7645 :

2 alleles

99.2% missing data

1	(0.339) 0.334 0.349

4	(0.661) 0.666 0.651

Locus 7646 :

3 alleles

98.8% missing data

1	(0.417) 0.419 0.419

2	(0.310) 0.307 0.307

4	(0.273) 0.273 0.274

Locus 7647 :

2 alleles

93.4% missing data

2	(0.316) 0.311 0.323

3	(0.684) 0.689 0.677

Locus 7648 :

2 alleles

98.4% missing data

2	(0.384) 0.378 0.385

1	(0.616) 0.622 0.615

Locus 7649 :

2 alleles

98.4% missing data

2	(0.568) 0.576 0.570

4	(0.432) 0.424 0.430

Locus 7650 :

2 alleles

98.8% missing data

2	(0.408) 0.398 0.423

4	(0.592) 0.602 0.577

Locus 7651 :

2 alleles

99.2% missing data

3	(0.351) 0.346 0.362

4	(0.649) 0.654 0.638

Locus 7652 :

2 alleles

99.2% missing data

1	(0.161) 0.169 0.155

3	(0.839) 0.831 0.845

Locus 7653 :

2 alleles

99.2% missing data

4	(0.240) 0.245 0.244

3	(0.760) 0.755 0.756

Locus 7654 :

2 alleles

98.4% missing data

4	(0.295) 0.297 0.298

1	(0.705) 0.703 0.702

Locus 7655 :

2 alleles

99.2% missing data

1	(0.558) 0.554 0.563

3	(0.442) 0.446 0.437

Locus 7656 :

3 alleles

98.8% missing data

2	(0.324) 0.324 0.329

4	(0.204) 0.206 0.202

3	(0.472) 0.470 0.469

Locus 7657 :

2 alleles

99.2% missing data

2	(0.228) 0.226 0.235

4	(0.772) 0.774 0.765

Locus 7658 :

2 alleles

96.5% missing data

1	(0.817) 0.828 0.809

3	(0.183) 0.172 0.191

Locus 7659 :

3 alleles

89.8% missing data

1	(0.846) 0.878 0.831

2	(0.065) 0.051 0.072

4	(0.089) 0.071 0.097

Locus 7660 :

3 alleles

90.2% missing data

4	(0.147) 0.117 0.163

3	(0.764) 0.803 0.749

1	(0.089) 0.080 0.088

Locus 7661 :

3 alleles

91.8% missing data

1	(0.503) 0.467 0.545

3	(0.370) 0.415 0.334

4	(0.127) 0.119 0.121

Locus 7662 :

2 alleles

0.4% missing data

2	(0.992) 0.995 0.995

1	(0.008) 0.005 0.005

Locus 7663 :

2 alleles

99.2% missing data

1	(0.601) 0.598 0.604

4	(0.399) 0.402 0.396

Locus 7664 :

3 alleles

90.6% missing data

3	(0.811) 0.842 0.799

4	(0.088) 0.076 0.092

1	(0.101) 0.083 0.109

Locus 7665 :

2 alleles

99.2% missing data

4	(0.350) 0.348 0.350

1	(0.650) 0.652 0.650

Locus 7666 :

2 alleles

96.1% missing data

1	(0.694) 0.689 0.698

2	(0.306) 0.311 0.302

Locus 7667 :

2 alleles

97.3% missing data

1	(0.598) 0.597 0.602

2	(0.402) 0.403 0.398

Locus 7668 :

2 alleles

97.3% missing data

3	(0.256) 0.258 0.248

2	(0.744) 0.742 0.752

Locus 7669 :

2 alleles

98.4% missing data

1	(0.537) 0.532 0.544

4	(0.463) 0.468 0.456

Locus 7670 :

3 alleles

98.8% missing data

4	(0.169) 0.171 0.173

2	(0.545) 0.536 0.543

3	(0.286) 0.293 0.284

Locus 7671 :

2 alleles

98.0% missing data

2	(0.266) 0.256 0.270

4	(0.734) 0.744 0.730

Locus 7672 :

2 alleles

93.0% missing data

1	(0.873) 0.876 0.883

2	(0.127) 0.124 0.117

Locus 7673 :

2 alleles

98.4% missing data

3	(0.793) 0.791 0.790

1	(0.207) 0.209 0.210

Locus 7674 :

2 alleles

96.1% missing data

2	(0.869) 0.879 0.865

3	(0.131) 0.121 0.135

Locus 7675 :

2 alleles

90.6% missing data

1	(0.281) 0.297 0.258

4	(0.719) 0.703 0.742

Locus 7676 :

2 alleles

96.9% missing data

3	(0.587) 0.587 0.581

1	(0.413) 0.413 0.419

Locus 7677 :

2 alleles

99.2% missing data

1	(0.306) 0.305 0.309

2	(0.694) 0.695 0.691

Locus 7678 :

2 alleles

99.2% missing data

2	(0.469) 0.466 0.469

4	(0.531) 0.534 0.531

Locus 7679 :

2 alleles

98.4% missing data

1	(0.459) 0.455 0.465

3	(0.541) 0.545 0.535

Locus 7680 :

2 alleles

99.2% missing data

2	(0.765) 0.759 0.766

3	(0.235) 0.241 0.234

Locus 7681 :

3 alleles

97.7% missing data

2	(0.402) 0.394 0.406

1	(0.398) 0.395 0.403

4	(0.201) 0.211 0.190

Locus 7682 :

2 alleles

98.8% missing data

1	(0.813) 0.815 0.805

2	(0.187) 0.185 0.195

Locus 7683 :

2 alleles

96.9% missing data

2	(0.789) 0.795 0.783

1	(0.211) 0.205 0.217

Locus 7684 :

2 alleles

99.2% missing data

4	(0.314) 0.319 0.313

2	(0.686) 0.681 0.687

Locus 7685 :

2 alleles

99.2% missing data

1	(0.527) 0.531 0.526

4	(0.473) 0.469 0.474

Locus 7686 :

2 alleles

96.1% missing data

3	(0.200) 0.189 0.204

1	(0.800) 0.811 0.796

Locus 7687 :

2 alleles

98.0% missing data

1	(0.630) 0.624 0.646

3	(0.370) 0.376 0.354

Locus 7688 :

2 alleles

99.2% missing data

2	(0.528) 0.525 0.528

4	(0.472) 0.475 0.472

Locus 7689 :

2 alleles

99.2% missing data

4	(0.478) 0.482 0.477

3	(0.522) 0.518 0.523

Locus 7690 :

2 alleles

99.2% missing data

4	(0.561) 0.567 0.552

1	(0.439) 0.433 0.448

Locus 7691 :

2 alleles

0.0% missing data

2	(0.984) 0.987 0.990

3	(0.016) 0.013 0.010

Locus 7692 :

2 alleles

98.8% missing data

4	(0.466) 0.471 0.463

3	(0.534) 0.529 0.537

Locus 7693 :

2 alleles

92.6% missing data

1	(0.914) 0.914 0.920

2	(0.086) 0.086 0.080

Locus 7694 :

2 alleles

93.0% missing data

3	(0.900) 0.902 0.906

1	(0.100) 0.098 0.094

Locus 7695 :

3 alleles

98.4% missing data

4	(0.365) 0.375 0.359

1	(0.322) 0.321 0.322

2	(0.313) 0.304 0.319

Locus 7696 :

2 alleles

99.2% missing data

1	(0.444) 0.447 0.444

3	(0.556) 0.553 0.556

Locus 7697 :

2 alleles

92.2% missing data

4	(0.144) 0.146 0.137

1	(0.856) 0.854 0.863

Locus 7698 :

2 alleles

99.2% missing data

1	(0.605) 0.604 0.605

3	(0.395) 0.396 0.395

Locus 7699 :

2 alleles

99.2% missing data

2	(0.485) 0.484 0.482

4	(0.515) 0.516 0.518

Locus 7700 :

2 alleles

99.2% missing data

4	(0.605) 0.599 0.611

3	(0.395) 0.401 0.389

Locus 7701 :

2 alleles

99.2% missing data

1	(0.701) 0.696 0.702

3	(0.299) 0.304 0.298

Locus 7702 :

2 alleles

98.8% missing data

4	(0.286) 0.277 0.292

1	(0.714) 0.723 0.708

Locus 7703 :

2 alleles

97.7% missing data

3	(0.784) 0.785 0.788

4	(0.216) 0.215 0.212

Locus 7704 :

2 alleles

99.2% missing data

4	(0.252) 0.257 0.247

3	(0.748) 0.743 0.753

Locus 7705 :

3 alleles

95.7% missing data

3	(0.162) 0.162 0.162

1	(0.764) 0.762 0.767

2	(0.074) 0.076 0.071

Locus 7706 :

2 alleles

99.2% missing data

1	(0.489) 0.489 0.485

3	(0.511) 0.511 0.515

Locus 7707 :

2 alleles

94.5% missing data

1	(0.872) 0.870 0.881

4	(0.128) 0.130 0.119

Locus 7708 :

2 alleles

98.8% missing data

3	(0.776) 0.774 0.770

4	(0.224) 0.226 0.230

Locus 7709 :

2 alleles

98.0% missing data

1	(0.227) 0.220 0.236

4	(0.773) 0.780 0.764

Locus 7710 :

2 alleles

99.2% missing data

1	(0.527) 0.527 0.530

4	(0.473) 0.473 0.470

Locus 7711 :

2 alleles

99.2% missing data

2	(0.365) 0.361 0.360

1	(0.635) 0.639 0.640

Locus 7712 :

2 alleles

99.2% missing data

1	(0.440) 0.435 0.449

3	(0.560) 0.565 0.551

Locus 7713 :

2 alleles

91.0% missing data

4	(0.901) 0.919 0.891

3	(0.099) 0.081 0.109

Locus 7714 :

2 alleles

99.2% missing data

1	(0.239) 0.232 0.240

4	(0.761) 0.768 0.760

Locus 7715 :

2 alleles

97.3% missing data

1	(0.215) 0.216 0.211

2	(0.785) 0.784 0.789

Locus 7716 :

2 alleles

99.2% missing data

2	(0.340) 0.340 0.341

4	(0.660) 0.660 0.659

Locus 7717 :

2 alleles

98.8% missing data

2	(0.736) 0.738 0.732

4	(0.264) 0.262 0.268

Locus 7718 :

2 alleles

98.8% missing data

2	(0.535) 0.525 0.531

1	(0.465) 0.475 0.469

Locus 7719 :

2 alleles

99.2% missing data

3	(0.661) 0.666 0.660

4	(0.339) 0.334 0.340

Locus 7720 :

2 alleles

99.2% missing data

2	(0.433) 0.436 0.432

3	(0.567) 0.564 0.568

Locus 7721 :

2 alleles

99.2% missing data

2	(0.659) 0.656 0.661

4	(0.341) 0.344 0.339

Locus 7722 :

2 alleles

97.7% missing data

4	(0.786) 0.788 0.787

3	(0.214) 0.212 0.213

Locus 7723 :

2 alleles

99.2% missing data

4	(0.461) 0.458 0.464

3	(0.539) 0.542 0.536

Locus 7724 :

2 alleles

98.8% missing data

3	(0.756) 0.741 0.764

1	(0.244) 0.259 0.236

Locus 7725 :

2 alleles

0.0% missing data

1	(0.920) 0.763 0.976

4	(0.080) 0.237 0.024

Locus 7726 :

2 alleles

98.4% missing data

2	(0.683) 0.691 0.679

1	(0.317) 0.309 0.321

Locus 7727 :

2 alleles

98.8% missing data

2	(0.459) 0.456 0.467

4	(0.541) 0.544 0.533

Locus 7728 :

2 alleles

98.8% missing data

4	(0.315) 0.323 0.307

2	(0.685) 0.677 0.693

Locus 7729 :

4 alleles

97.3% missing data

2	(0.320) 0.329 0.318

3	(0.257) 0.250 0.256

4	(0.152) 0.146 0.159

1	(0.271) 0.275 0.267

Locus 7730 :

2 alleles

98.0% missing data

3	(0.664) 0.660 0.675

1	(0.336) 0.340 0.325

Locus 7731 :

2 alleles

99.2% missing data

3	(0.376) 0.378 0.376

1	(0.624) 0.622 0.624

Locus 7732 :

2 alleles

99.2% missing data

3	(0.493) 0.488 0.500

2	(0.507) 0.512 0.500

Locus 7733 :

3 alleles

98.8% missing data

1	(0.213) 0.212 0.216

2	(0.424) 0.419 0.431

3	(0.364) 0.369 0.353

Locus 7734 :

2 alleles

98.4% missing data

3	(0.770) 0.775 0.769

2	(0.230) 0.225 0.231

Locus 7735 :

2 alleles

98.8% missing data

3	(0.877) 0.879 0.871

4	(0.123) 0.121 0.129

Locus 7736 :

2 alleles

99.2% missing data

3	(0.410) 0.405 0.416

1	(0.590) 0.595 0.584

Locus 7737 :

2 alleles

97.7% missing data

1	(0.327) 0.326 0.316

3	(0.673) 0.674 0.684

Locus 7738 :

2 alleles

99.2% missing data

3	(0.590) 0.585 0.587

4	(0.410) 0.415 0.413

Locus 7739 :

2 alleles

93.0% missing data

4	(0.864) 0.849 0.885

3	(0.136) 0.151 0.115

Locus 7740 :

2 alleles

98.0% missing data

2	(0.724) 0.725 0.724

4	(0.276) 0.275 0.276

Locus 7741 :

2 alleles

99.2% missing data

1	(0.549) 0.549 0.553

3	(0.451) 0.451 0.447

Locus 7742 :

2 alleles

93.0% missing data

1	(0.906) 0.907 0.915

3	(0.094) 0.093 0.085

Locus 7743 :

3 alleles

98.4% missing data

3	(0.348) 0.345 0.342

2	(0.398) 0.397 0.410

1	(0.253) 0.258 0.248

Locus 7744 :

2 alleles

98.4% missing data

3	(0.650) 0.649 0.654

1	(0.350) 0.351 0.346

Locus 7745 :

2 alleles

98.0% missing data

1	(0.792) 0.785 0.804

4	(0.208) 0.215 0.196

Locus 7746 :

2 alleles

99.2% missing data

1	(0.495) 0.495 0.495

3	(0.505) 0.505 0.505

Locus 7747 :

2 alleles

7.8% missing data

3	(0.974) 0.948 0.992

1	(0.026) 0.052 0.008

Locus 7748 :

2 alleles

97.7% missing data

2	(0.905) 0.909 0.905

1	(0.095) 0.091 0.095

Locus 7749 :

2 alleles

98.8% missing data

1	(0.672) 0.668 0.673

4	(0.328) 0.332 0.327

Locus 7750 :

2 alleles

99.2% missing data

3	(0.352) 0.356 0.347

1	(0.648) 0.644 0.653

Locus 7751 :

2 alleles

98.8% missing data

2	(0.507) 0.502 0.518

1	(0.493) 0.498 0.482

Locus 7752 :

2 alleles

98.4% missing data

4	(0.661) 0.662 0.662

1	(0.339) 0.338 0.338

Locus 7753 :

2 alleles

98.8% missing data

3	(0.418) 0.417 0.418

4	(0.582) 0.583 0.582

Locus 7754 :

2 alleles

99.2% missing data

2	(0.695) 0.695 0.700

3	(0.305) 0.305 0.300

Locus 7755 :

2 alleles

97.3% missing data

2	(0.267) 0.271 0.259

1	(0.733) 0.729 0.741

Locus 7756 :

2 alleles

99.2% missing data

1	(0.548) 0.544 0.551

3	(0.452) 0.456 0.449

Locus 7757 :

2 alleles

97.3% missing data

1	(0.157) 0.168 0.148

2	(0.843) 0.832 0.852

Locus 7758 :

2 alleles

99.2% missing data

1	(0.490) 0.492 0.485

3	(0.510) 0.508 0.515

Locus 7759 :

2 alleles

98.8% missing data

3	(0.281) 0.284 0.278

1	(0.719) 0.716 0.722

Locus 7760 :

2 alleles

99.2% missing data

1	(0.546) 0.539 0.554

3	(0.454) 0.461 0.446

Locus 7761 :

2 alleles

87.1% missing data

1	(0.899) 0.908 0.897

2	(0.101) 0.092 0.103

Locus 7762 :

2 alleles

96.5% missing data

4	(0.198) 0.186 0.204

1	(0.802) 0.814 0.796

Locus 7763 :

2 alleles

97.7% missing data

2	(0.183) 0.178 0.189

1	(0.817) 0.822 0.811

Locus 7764 :

2 alleles

97.7% missing data

1	(0.656) 0.653 0.661

3	(0.344) 0.347 0.339

Locus 7765 :

2 alleles

99.2% missing data

2	(0.627) 0.629 0.622

4	(0.373) 0.371 0.378

Locus 7766 :

2 alleles

91.8% missing data

1	(0.855) 0.858 0.870

4	(0.145) 0.142 0.130

Locus 7767 :

3 alleles

98.8% missing data

4	(0.240) 0.243 0.238

3	(0.253) 0.245 0.256

2	(0.507) 0.511 0.506

Locus 7768 :

3 alleles

98.8% missing data

4	(0.280) 0.279 0.286

2	(0.260) 0.254 0.264

3	(0.460) 0.467 0.450

Locus 7769 :

2 alleles

93.0% missing data

1	(0.701) 0.697 0.713

2	(0.299) 0.303 0.287

Locus 7770 :

2 alleles

96.5% missing data

1	(0.683) 0.693 0.677

2	(0.317) 0.307 0.323

Locus 7771 :

2 alleles

95.7% missing data

1	(0.840) 0.849 0.841

2	(0.160) 0.151 0.159

Locus 7772 :

3 alleles

98.4% missing data

1	(0.474) 0.479 0.472

4	(0.304) 0.301 0.301

2	(0.222) 0.220 0.227

Locus 7773 :

2 alleles

99.2% missing data

2	(0.424) 0.417 0.427

4	(0.576) 0.583 0.573

Locus 7774 :

2 alleles

99.2% missing data

4	(0.446) 0.444 0.446

1	(0.554) 0.556 0.554

Locus 7775 :

2 alleles

98.0% missing data

1	(0.820) 0.815 0.824

3	(0.180) 0.185 0.176

Locus 7776 :

2 alleles

98.4% missing data

2	(0.379) 0.370 0.384

3	(0.621) 0.630 0.616

Locus 7777 :

2 alleles

99.2% missing data

2	(0.451) 0.444 0.451

1	(0.549) 0.556 0.549

Locus 7778 :

2 alleles

99.2% missing data

1	(0.447) 0.447 0.451

2	(0.553) 0.553 0.549

Locus 7779 :

2 alleles

99.2% missing data

4	(0.719) 0.716 0.718

1	(0.281) 0.284 0.282

Locus 7780 :

2 alleles

8.2% missing data

4	(0.989) 0.979 0.996

3	(0.011) 0.021 0.004

Locus 7781 :

2 alleles

99.2% missing data

2	(0.468) 0.465 0.473

1	(0.532) 0.535 0.527

Locus 7782 :

2 alleles

99.2% missing data

1	(0.767) 0.770 0.759

4	(0.233) 0.230 0.241

Locus 7783 :

2 alleles

98.8% missing data

4	(0.805) 0.796 0.806

2	(0.195) 0.204 0.194

Locus 7784 :

2 alleles

95.7% missing data

1	(0.608) 0.606 0.612

2	(0.392) 0.394 0.388

Locus 7785 :

2 alleles

98.0% missing data

1	(0.369) 0.367 0.371

2	(0.631) 0.633 0.629

Locus 7786 :

2 alleles

98.4% missing data

1	(0.791) 0.792 0.794

2	(0.209) 0.208 0.206

Locus 7787 :

2 alleles

99.2% missing data

4	(0.621) 0.613 0.621

1	(0.379) 0.387 0.379

Locus 7788 :

2 alleles

94.1% missing data

1	(0.676) 0.684 0.665

2	(0.324) 0.316 0.335

Locus 7789 :

2 alleles

97.3% missing data

4	(0.133) 0.125 0.142

2	(0.867) 0.875 0.858

Locus 7790 :

3 alleles

97.7% missing data

2	(0.469) 0.470 0.463

1	(0.367) 0.362 0.377

3	(0.164) 0.167 0.161

Locus 7791 :

2 alleles

99.2% missing data

1	(0.300) 0.305 0.291

2	(0.700) 0.695 0.709

Locus 7792 :

2 alleles

99.2% missing data

1	(0.416) 0.411 0.413

2	(0.584) 0.589 0.587

Locus 7793 :

2 alleles

99.2% missing data

1	(0.759) 0.758 0.759

4	(0.241) 0.242 0.241

Locus 7794 :

2 alleles

96.5% missing data

1	(0.844) 0.849 0.845

3	(0.156) 0.151 0.155

Locus 7795 :

2 alleles

97.7% missing data

1	(0.509) 0.501 0.526

4	(0.491) 0.499 0.474

Locus 7796 :

2 alleles

99.2% missing data

3	(0.518) 0.518 0.515

4	(0.482) 0.482 0.485

Locus 7797 :

2 alleles

99.2% missing data

4	(0.540) 0.537 0.546

1	(0.460) 0.463 0.454

Locus 7798 :

2 alleles

99.2% missing data

1	(0.579) 0.574 0.577

3	(0.421) 0.426 0.423

Locus 7799 :

2 alleles

99.2% missing data

4	(0.357) 0.358 0.357

2	(0.643) 0.642 0.643

Locus 7800 :

2 alleles

99.2% missing data

2	(0.670) 0.669 0.662

4	(0.330) 0.331 0.338

Locus 7801 :

2 alleles

98.8% missing data

4	(0.260) 0.258 0.266

2	(0.740) 0.742 0.734

Locus 7802 :

3 alleles

98.4% missing data

3	(0.178) 0.181 0.180

2	(0.506) 0.504 0.510

1	(0.316) 0.315 0.311

Locus 7803 :

2 alleles

99.2% missing data

4	(0.371) 0.365 0.376

3	(0.629) 0.635 0.624

Locus 7804 :

3 alleles

96.1% missing data

1	(0.152) 0.142 0.157

4	(0.223) 0.208 0.224

2	(0.626) 0.650 0.618

Locus 7805 :

2 alleles

93.0% missing data

2	(0.901) 0.904 0.900

1	(0.099) 0.096 0.100

Locus 7806 :

2 alleles

99.2% missing data

4	(0.650) 0.655 0.647

2	(0.350) 0.345 0.353

Locus 7807 :

2 alleles

98.8% missing data

3	(0.394) 0.391 0.397

2	(0.606) 0.609 0.603

Locus 7808 :

2 alleles

98.4% missing data

1	(0.252) 0.255 0.251

2	(0.748) 0.745 0.749

Locus 7809 :

2 alleles

99.2% missing data

4	(0.416) 0.413 0.422

2	(0.584) 0.587 0.578

Locus 7810 :

2 alleles

98.8% missing data

1	(0.460) 0.458 0.461

2	(0.540) 0.542 0.539

Locus 7811 :

2 alleles

97.3% missing data

2	(0.868) 0.877 0.864

4	(0.132) 0.123 0.136

Locus 7812 :

2 alleles

99.2% missing data

1	(0.517) 0.508 0.520

3	(0.483) 0.492 0.480

Locus 7813 :

2 alleles

99.2% missing data

4	(0.419) 0.420 0.420

1	(0.581) 0.580 0.580

Locus 7814 :

2 alleles

99.2% missing data

2	(0.494) 0.489 0.502

4	(0.506) 0.511 0.498

Locus 7815 :

2 alleles

96.1% missing data

1	(0.844) 0.847 0.847

4	(0.156) 0.153 0.153

Locus 7816 :

3 alleles

98.8% missing data

4	(0.325) 0.329 0.318

2	(0.524) 0.522 0.525

1	(0.151) 0.149 0.157

Locus 7817 :

2 alleles

2.0% missing data

2	(0.986) 0.989 0.993

3	(0.014) 0.011 0.007

Locus 7818 :

3 alleles

95.3% missing data

1	(0.688) 0.690 0.693

2	(0.215) 0.214 0.213

3	(0.097) 0.095 0.094

Locus 7819 :

3 alleles

98.8% missing data

4	(0.412) 0.414 0.413

1	(0.177) 0.171 0.185

2	(0.410) 0.415 0.403

Locus 7820 :

2 alleles

99.2% missing data

2	(0.564) 0.559 0.567

1	(0.436) 0.441 0.433

Locus 7821 :

2 alleles

99.2% missing data

1	(0.610) 0.617 0.613

4	(0.390) 0.383 0.387

Locus 7822 :

2 alleles

96.5% missing data

2	(0.387) 0.392 0.382

1	(0.613) 0.608 0.618

Locus 7823 :

2 alleles

98.4% missing data

1	(0.255) 0.251 0.256

2	(0.745) 0.749 0.744

Locus 7824 :

2 alleles

96.5% missing data

1	(0.394) 0.400 0.392

2	(0.606) 0.600 0.608

Locus 7825 :

2 alleles

99.2% missing data

2	(0.337) 0.330 0.348

1	(0.663) 0.670 0.652

Locus 7826 :

2 alleles

95.7% missing data

4	(0.876) 0.884 0.873

1	(0.124) 0.116 0.127

Locus 7827 :

2 alleles

99.2% missing data

3	(0.680) 0.672 0.682

2	(0.320) 0.328 0.318

Locus 7828 :

2 alleles

91.0% missing data

3	(0.931) 0.946 0.919

1	(0.069) 0.054 0.081

Locus 7829 :

2 alleles

95.7% missing data

3	(0.865) 0.865 0.873

4	(0.135) 0.135 0.127

Locus 7830 :

3 alleles

98.8% missing data

1	(0.530) 0.531 0.528

3	(0.288) 0.281 0.290

4	(0.182) 0.188 0.181

Locus 7831 :

2 alleles

98.0% missing data

2	(0.542) 0.556 0.537

4	(0.458) 0.444 0.463

Locus 7832 :

2 alleles

98.4% missing data

1	(0.639) 0.633 0.649

2	(0.361) 0.367 0.351

Locus 7833 :

3 alleles

97.3% missing data

4	(0.138) 0.138 0.130

2	(0.277) 0.279 0.282

1	(0.585) 0.584 0.588

Locus 7834 :

2 alleles

96.9% missing data

2	(0.327) 0.342 0.316

1	(0.673) 0.658 0.684

Locus 7835 :

3 alleles

96.5% missing data

1	(0.369) 0.373 0.367

2	(0.463) 0.455 0.469

3	(0.168) 0.173 0.163

Locus 7836 :

3 alleles

98.4% missing data

3	(0.414) 0.419 0.402

1	(0.369) 0.368 0.378

2	(0.217) 0.213 0.221

Locus 7837 :

2 alleles

99.2% missing data

2	(0.459) 0.460 0.457

1	(0.541) 0.540 0.543

Locus 7838 :

3 alleles

97.7% missing data

3	(0.437) 0.433 0.439

2	(0.466) 0.461 0.470

1	(0.097) 0.105 0.091

Locus 7839 :

3 alleles

95.3% missing data

1	(0.167) 0.163 0.162

2	(0.754) 0.748 0.769

4	(0.079) 0.088 0.070

Locus 7840 :

2 alleles

99.2% missing data

1	(0.735) 0.736 0.729

2	(0.265) 0.264 0.271

Locus 7841 :

2 alleles

99.2% missing data

2	(0.445) 0.447 0.443

1	(0.555) 0.553 0.557

Locus 7842 :

2 alleles

95.3% missing data

1	(0.906) 0.896 0.916

3	(0.094) 0.104 0.084

Locus 7843 :

2 alleles

96.1% missing data

4	(0.759) 0.769 0.761

2	(0.241) 0.231 0.239

Locus 7844 :

2 alleles

99.2% missing data

4	(0.486) 0.494 0.482

2	(0.514) 0.506 0.518

Locus 7845 :

2 alleles

99.2% missing data

2	(0.528) 0.522 0.533

4	(0.472) 0.478 0.467

Locus 7846 :

2 alleles

99.2% missing data

2	(0.432) 0.433 0.437

1	(0.568) 0.567 0.563

Locus 7847 :

2 alleles

99.2% missing data

2	(0.284) 0.282 0.290

1	(0.716) 0.718 0.710

Locus 7848 :

2 alleles

99.2% missing data

4	(0.608) 0.608 0.607

1	(0.392) 0.392 0.393

Locus 7849 :

2 alleles

99.2% missing data

4	(0.422) 0.418 0.427

1	(0.578) 0.582 0.573

Locus 7850 :

2 alleles

98.4% missing data

4	(0.782) 0.789 0.779

1	(0.218) 0.211 0.221

Locus 7851 :

2 alleles

99.2% missing data

4	(0.559) 0.556 0.557

2	(0.441) 0.444 0.443

Locus 7852 :

3 alleles

94.5% missing data

4	(0.821) 0.836 0.821

3	(0.073) 0.061 0.081

2	(0.106) 0.103 0.098

Locus 7853 :

2 alleles

98.8% missing data

1	(0.366) 0.377 0.369

3	(0.634) 0.623 0.631

Locus 7854 :

2 alleles

99.2% missing data

2	(0.746) 0.749 0.746

1	(0.254) 0.251 0.254

Locus 7855 :

2 alleles

99.2% missing data

4	(0.629) 0.627 0.626

1	(0.371) 0.373 0.374

Locus 7856 :

2 alleles

99.2% missing data

4	(0.780) 0.778 0.784

1	(0.220) 0.222 0.216

Locus 7857 :

2 alleles

98.8% missing data

3	(0.414) 0.410 0.414

1	(0.586) 0.590 0.586

Locus 7858 :

2 alleles

98.8% missing data

1	(0.663) 0.669 0.652

3	(0.337) 0.331 0.348

Locus 7859 :

2 alleles

93.4% missing data

2	(0.860) 0.862 0.870

4	(0.140) 0.138 0.130

Locus 7860 :

2 alleles

92.6% missing data

2	(0.117) 0.114 0.115

3	(0.883) 0.886 0.885

Locus 7861 :

2 alleles

92.6% missing data

3	(0.901) 0.892 0.915

4	(0.099) 0.108 0.085

Locus 7862 :

3 alleles

98.0% missing data

3	(0.475) 0.479 0.473

2	(0.334) 0.326 0.340

1	(0.191) 0.195 0.187

Locus 7863 :

2 alleles

93.0% missing data

3	(0.864) 0.846 0.882

1	(0.136) 0.154 0.118

Locus 7864 :

2 alleles

94.5% missing data

4	(0.832) 0.838 0.837

1	(0.168) 0.162 0.163

Locus 7865 :

2 alleles

58.6% missing data

2	(0.798) 0.658 0.890

1	(0.202) 0.342 0.110

Locus 7866 :

2 alleles

0.0% missing data

4	(0.983) 0.979 0.995

1	(0.017) 0.021 0.005

Locus 7867 :

2 alleles

99.2% missing data

1	(0.306) 0.303 0.312

4	(0.694) 0.697 0.688

Locus 7868 :

3 alleles

98.8% missing data

1	(0.240) 0.234 0.240

3	(0.505) 0.514 0.495

4	(0.254) 0.251 0.265

Locus 7869 :

2 alleles

95.3% missing data

2	(0.523) 0.552 0.494

4	(0.477) 0.448 0.506

Locus 7870 :

2 alleles

98.8% missing data

4	(0.616) 0.625 0.607

3	(0.384) 0.375 0.393

Locus 7871 :

2 alleles

98.8% missing data

4	(0.420) 0.417 0.420

2	(0.580) 0.583 0.580

Locus 7872 :

2 alleles

97.7% missing data

2	(0.735) 0.746 0.735

4	(0.265) 0.254 0.265

Locus 7873 :

2 alleles

97.7% missing data

2	(0.241) 0.247 0.232

1	(0.759) 0.753 0.768

Locus 7874 :

2 alleles

98.0% missing data

1	(0.795) 0.800 0.793

2	(0.205) 0.200 0.207

Locus 7875 :

2 alleles

99.2% missing data

4	(0.350) 0.346 0.355

3	(0.650) 0.654 0.645

Locus 7876 :

2 alleles

98.8% missing data

1	(0.381) 0.378 0.388

3	(0.619) 0.622 0.612

Locus 7877 :

2 alleles

96.9% missing data

1	(0.794) 0.795 0.802

2	(0.206) 0.205 0.198

Locus 7878 :

2 alleles

93.8% missing data

4	(0.189) 0.186 0.176

2	(0.811) 0.814 0.824

Locus 7879 :

2 alleles

91.0% missing data

1	(0.920) 0.926 0.925

3	(0.080) 0.074 0.075

Locus 7880 :

2 alleles

99.2% missing data

2	(0.516) 0.511 0.524

3	(0.484) 0.489 0.476

Locus 7881 :

2 alleles

97.7% missing data

2	(0.810) 0.816 0.812

4	(0.190) 0.184 0.188

Locus 7882 :

2 alleles

99.2% missing data

2	(0.509) 0.508 0.511

3	(0.491) 0.492 0.489

Locus 7883 :

2 alleles

99.2% missing data

2	(0.270) 0.267 0.276

4	(0.730) 0.733 0.724

Locus 7884 :

3 alleles

93.4% missing data

2	(0.741) 0.729 0.770

1	(0.145) 0.154 0.128

3	(0.114) 0.117 0.102

Locus 7885 :

2 alleles

99.2% missing data

4	(0.399) 0.401 0.403

2	(0.601) 0.599 0.597

Locus 7886 :

2 alleles

97.7% missing data

1	(0.212) 0.209 0.207

4	(0.788) 0.791 0.793

Locus 7887 :

2 alleles

98.4% missing data

4	(0.707) 0.717 0.701

1	(0.293) 0.283 0.299

Locus 7888 :

3 alleles

98.4% missing data

1	(0.482) 0.476 0.485

4	(0.293) 0.307 0.287

3	(0.225) 0.218 0.228

Locus 7889 :

2 alleles

99.2% missing data

4	(0.776) 0.770 0.779

1	(0.224) 0.230 0.221

Locus 7890 :

3 alleles

97.7% missing data

1	(0.694) 0.697 0.688

3	(0.181) 0.175 0.191

2	(0.125) 0.128 0.122

Locus 7891 :

2 alleles

99.2% missing data

3	(0.406) 0.403 0.415

4	(0.594) 0.597 0.585

Locus 7892 :

3 alleles

98.8% missing data

4	(0.078) 0.074 0.085

1	(0.498) 0.498 0.491

2	(0.424) 0.427 0.423

Locus 7893 :

2 alleles

98.8% missing data

2	(0.454) 0.439 0.462

3	(0.546) 0.561 0.538

Locus 7894 :

2 alleles

92.6% missing data

1	(0.904) 0.906 0.910

2	(0.096) 0.094 0.090

Locus 7895 :

2 alleles

97.7% missing data

1	(0.117) 0.118 0.119

3	(0.883) 0.882 0.881

Locus 7896 :

2 alleles

99.2% missing data

4	(0.385) 0.389 0.376

1	(0.615) 0.611 0.624

Locus 7897 :

2 alleles

83.2% missing data

4	(0.479) 0.544 0.412

2	(0.521) 0.456 0.588

Locus 7898 :

2 alleles

96.1% missing data

3	(0.410) 0.388 0.417

1	(0.590) 0.612 0.583

Locus 7899 :

2 alleles

99.2% missing data

2	(0.619) 0.617 0.620

3	(0.381) 0.383 0.380

Locus 7900 :

2 alleles

99.2% missing data

4	(0.485) 0.485 0.487

1	(0.515) 0.515 0.513

Locus 7901 :

2 alleles

91.8% missing data

3	(0.949) 0.941 0.958

4	(0.051) 0.059 0.042

Locus 7902 :

2 alleles

99.2% missing data

1	(0.668) 0.668 0.665

2	(0.332) 0.332 0.335

Locus 7903 :

3 alleles

98.8% missing data

4	(0.566) 0.562 0.570

3	(0.224) 0.223 0.224

2	(0.209) 0.216 0.206

Locus 7904 :

2 alleles

98.4% missing data

4	(0.564) 0.569 0.561

1	(0.436) 0.431 0.439

Locus 7905 :

2 alleles

0.4% missing data

4	(0.910) 0.761 0.973

1	(0.090) 0.239 0.027

Locus 7906 :

2 alleles

99.2% missing data

1	(0.485) 0.485 0.490

4	(0.515) 0.515 0.510

Locus 7907 :

2 alleles

99.2% missing data

1	(0.429) 0.419 0.437

3	(0.571) 0.581 0.563

Locus 7908 :

2 alleles

99.2% missing data

3	(0.425) 0.422 0.427

4	(0.575) 0.578 0.573

Locus 7909 :

2 alleles

99.2% missing data

2	(0.508) 0.515 0.505

1	(0.492) 0.485 0.495

Locus 7910 :

3 alleles

98.4% missing data

3	(0.440) 0.447 0.433

4	(0.301) 0.291 0.309

1	(0.259) 0.262 0.258

Locus 7911 :

3 alleles

97.7% missing data

3	(0.342) 0.336 0.363

4	(0.148) 0.152 0.147

1	(0.509) 0.512 0.490

Locus 7912 :

3 alleles

95.7% missing data

3	(0.122) 0.111 0.126

1	(0.758) 0.776 0.747

4	(0.121) 0.113 0.127

Locus 7913 :

2 alleles

99.2% missing data

2	(0.507) 0.502 0.506

3	(0.493) 0.498 0.494

Locus 7914 :

2 alleles

91.0% missing data

2	(0.907) 0.924 0.898

4	(0.093) 0.076 0.102

Locus 7915 :

2 alleles

98.8% missing data

4	(0.602) 0.606 0.597

2	(0.398) 0.394 0.403

Locus 7916 :

2 alleles

96.1% missing data

2	(0.802) 0.805 0.811

3	(0.198) 0.195 0.189

Locus 7917 :

2 alleles

99.2% missing data

2	(0.283) 0.277 0.284

4	(0.717) 0.723 0.716

Locus 7918 :

2 alleles

98.0% missing data

1	(0.751) 0.750 0.753

2	(0.249) 0.250 0.247

Locus 7919 :

2 alleles

99.2% missing data

1	(0.596) 0.592 0.604

3	(0.404) 0.408 0.396

Locus 7920 :

2 alleles

96.5% missing data

3	(0.338) 0.323 0.345

2	(0.662) 0.677 0.655

Locus 7921 :

2 alleles

99.2% missing data

3	(0.528) 0.514 0.533

4	(0.472) 0.486 0.467

Locus 7922 :

2 alleles

99.2% missing data

2	(0.595) 0.600 0.595

1	(0.405) 0.400 0.405

Locus 7923 :

2 alleles

99.2% missing data

4	(0.539) 0.534 0.543

1	(0.461) 0.466 0.457

Locus 7924 :

3 alleles

98.8% missing data

2	(0.297) 0.292 0.297

1	(0.334) 0.344 0.331

4	(0.369) 0.364 0.372

Locus 7925 :

2 alleles

99.2% missing data

1	(0.327) 0.333 0.321

2	(0.673) 0.667 0.679

Locus 7926 :

2 alleles

99.2% missing data

2	(0.553) 0.547 0.552

4	(0.447) 0.453 0.448

Locus 7927 :

2 alleles

99.2% missing data

2	(0.449) 0.446 0.446

1	(0.551) 0.554 0.554

Locus 7928 :

2 alleles

99.2% missing data

2	(0.588) 0.587 0.592

3	(0.412) 0.413 0.408

Locus 7929 :

2 alleles

99.2% missing data

4	(0.429) 0.417 0.437

1	(0.571) 0.583 0.563

Locus 7930 :

3 alleles

98.4% missing data

3	(0.414) 0.412 0.416

4	(0.292) 0.294 0.283

1	(0.294) 0.294 0.301

Locus 7931 :

2 alleles

99.2% missing data

1	(0.584) 0.583 0.583

4	(0.416) 0.417 0.417

Locus 7932 :

2 alleles

99.2% missing data

2	(0.639) 0.631 0.645

4	(0.361) 0.369 0.355

Locus 7933 :

2 alleles

98.8% missing data

2	(0.318) 0.312 0.322

4	(0.682) 0.688 0.678

Locus 7934 :

3 alleles

98.4% missing data

4	(0.351) 0.338 0.362

2	(0.212) 0.213 0.208

3	(0.437) 0.449 0.430

Locus 7935 :

2 alleles

96.1% missing data

1	(0.820) 0.832 0.817

2	(0.180) 0.168 0.183

Locus 7936 :

2 alleles

98.8% missing data

2	(0.570) 0.570 0.569

1	(0.430) 0.430 0.431

Locus 7937 :

2 alleles

90.6% missing data

4	(0.758) 0.762 0.758

2	(0.242) 0.238 0.242

Locus 7938 :

2 alleles

98.8% missing data

2	(0.586) 0.586 0.591

4	(0.414) 0.414 0.409

Locus 7939 :

2 alleles

99.2% missing data

1	(0.487) 0.489 0.485

4	(0.513) 0.511 0.515

Locus 7940 :

2 alleles

96.5% missing data

1	(0.779) 0.784 0.776

3	(0.221) 0.216 0.224

Locus 7941 :

2 alleles

96.5% missing data

1	(0.459) 0.454 0.464

4	(0.541) 0.546 0.536

Locus 7942 :

3 alleles

98.8% missing data

4	(0.428) 0.427 0.432

2	(0.248) 0.247 0.247

1	(0.324) 0.326 0.321

Locus 7943 :

2 alleles

99.2% missing data

2	(0.211) 0.204 0.214

4	(0.789) 0.796 0.786

Locus 7944 :

3 alleles

98.4% missing data

4	(0.418) 0.419 0.423

2	(0.297) 0.295 0.300

1	(0.285) 0.287 0.277

Locus 7945 :

2 alleles

98.4% missing data

2	(0.765) 0.767 0.762

4	(0.235) 0.233 0.238

Locus 7946 :

2 alleles

99.2% missing data

4	(0.312) 0.304 0.315

1	(0.688) 0.696 0.685

Locus 7947 :

2 alleles

99.2% missing data

3	(0.346) 0.356 0.343

2	(0.654) 0.644 0.657

Locus 7948 :

2 alleles

99.2% missing data

2	(0.476) 0.479 0.476

1	(0.524) 0.521 0.524

Locus 7949 :

2 alleles

99.2% missing data

3	(0.632) 0.625 0.635

1	(0.368) 0.375 0.365

Locus 7950 :

2 alleles

97.3% missing data

4	(0.741) 0.740 0.755

1	(0.259) 0.260 0.245

Locus 7951 :

2 alleles

99.2% missing data

2	(0.702) 0.701 0.704

4	(0.298) 0.299 0.296

Locus 7952 :

2 alleles

92.2% missing data

2	(0.920) 0.923 0.927

3	(0.080) 0.077 0.073

Locus 7953 :

2 alleles

99.2% missing data

1	(0.708) 0.710 0.704

3	(0.292) 0.290 0.296

Locus 7954 :

2 alleles

94.5% missing data

1	(0.833) 0.835 0.842

2	(0.167) 0.165 0.158

Locus 7955 :

2 alleles

92.6% missing data

2	(0.859) 0.852 0.876

1	(0.141) 0.148 0.124

Locus 7956 :

2 alleles

96.9% missing data

4	(0.746) 0.742 0.756

3	(0.254) 0.258 0.244

Locus 7957 :

2 alleles

98.8% missing data

4	(0.670) 0.678 0.666

2	(0.330) 0.322 0.334

Locus 7958 :

3 alleles

98.8% missing data

2	(0.157) 0.160 0.157

4	(0.388) 0.380 0.390

3	(0.455) 0.459 0.454

Locus 7959 :

2 alleles

99.2% missing data

1	(0.466) 0.475 0.464

2	(0.534) 0.525 0.536

Locus 7960 :

2 alleles

97.3% missing data

4	(0.731) 0.730 0.736

2	(0.269) 0.270 0.264

Locus 7961 :

2 alleles

0.4% missing data

4	(0.987) 0.981 0.996

3	(0.013) 0.019 0.004

Locus 7962 :

2 alleles

97.7% missing data

2	(0.762) 0.758 0.771

3	(0.238) 0.242 0.229

Locus 7963 :

2 alleles

98.8% missing data

3	(0.334) 0.336 0.330

4	(0.666) 0.664 0.670

Locus 7964 :

2 alleles

0.0% missing data

4	(0.985) 0.981 0.996

1	(0.015) 0.019 0.004

Locus 7965 :

2 alleles

97.3% missing data

4	(0.392) 0.372 0.406

1	(0.608) 0.628 0.594

Locus 7966 :

2 alleles

91.0% missing data

1	(0.877) 0.887 0.877

4	(0.123) 0.113 0.123

Locus 7967 :

2 alleles

99.2% missing data

2	(0.440) 0.439 0.443

1	(0.560) 0.561 0.557

Locus 7968 :

2 alleles

99.2% missing data

1	(0.497) 0.489 0.500

3	(0.503) 0.511 0.500

Locus 7969 :

2 alleles

99.2% missing data

4	(0.272) 0.270 0.276

3	(0.728) 0.730 0.724

Locus 7970 :

2 alleles

99.2% missing data

4	(0.585) 0.577 0.592

3	(0.415) 0.423 0.408

Locus 7971 :

2 alleles

99.2% missing data

1	(0.694) 0.692 0.692

4	(0.306) 0.308 0.308

Locus 7972 :

2 alleles

96.5% missing data

1	(0.841) 0.831 0.851

4	(0.159) 0.169 0.149

Locus 7973 :

2 alleles

0.4% missing data

2	(0.987) 0.991 0.993

1	(0.013) 0.009 0.007

Locus 7974 :

2 alleles

98.4% missing data

3	(0.663) 0.667 0.662

1	(0.337) 0.333 0.338

Locus 7975 :

2 alleles

99.2% missing data

3	(0.215) 0.207 0.221

1	(0.785) 0.793 0.779

Locus 7976 :

2 alleles

0.0% missing data

3	(0.987) 0.990 0.993

2	(0.013) 0.010 0.007

Locus 7977 :

2 alleles

98.0% missing data

3	(0.261) 0.263 0.255

1	(0.739) 0.737 0.745

Locus 7978 :

2 alleles

99.2% missing data

2	(0.538) 0.531 0.539

4	(0.462) 0.469 0.461

Locus 7979 :

3 alleles

95.3% missing data

4	(0.160) 0.149 0.176

2	(0.742) 0.752 0.730

3	(0.098) 0.099 0.094

Locus 7980 :

2 alleles

99.2% missing data

4	(0.395) 0.390 0.400

2	(0.605) 0.610 0.600

Locus 7981 :

2 alleles

96.9% missing data

3	(0.171) 0.167 0.165

4	(0.829) 0.833 0.835

Locus 7982 :

2 alleles

99.2% missing data

3	(0.688) 0.685 0.691

2	(0.312) 0.315 0.309

Locus 7983 :

2 alleles

99.2% missing data

2	(0.494) 0.494 0.490

1	(0.506) 0.506 0.510

Locus 7984 :

2 alleles

96.1% missing data

2	(0.862) 0.858 0.864

1	(0.138) 0.142 0.136

Locus 7985 :

2 alleles

99.2% missing data

1	(0.592) 0.592 0.583

4	(0.408) 0.408 0.417

Locus 7986 :

2 alleles

99.2% missing data

3	(0.414) 0.405 0.417

2	(0.586) 0.595 0.583

Locus 7987 :

2 alleles

99.2% missing data

1	(0.618) 0.619 0.622

2	(0.382) 0.381 0.378

Locus 7988 :

2 alleles

99.2% missing data

3	(0.287) 0.286 0.298

4	(0.713) 0.714 0.702

Locus 7989 :

2 alleles

96.1% missing data

4	(0.844) 0.857 0.835

1	(0.156) 0.143 0.165

Locus 7990 :

2 alleles

94.5% missing data

1	(0.825) 0.829 0.838

2	(0.175) 0.171 0.162

Locus 7991 :

3 alleles

97.7% missing data

3	(0.220) 0.212 0.221

1	(0.506) 0.518 0.501

4	(0.274) 0.270 0.278

Locus 7992 :

2 alleles

99.2% missing data

2	(0.346) 0.346 0.350

4	(0.654) 0.654 0.650

Locus 7993 :

2 alleles

98.4% missing data

2	(0.541) 0.559 0.532

3	(0.459) 0.441 0.468

Locus 7994 :

2 alleles

96.9% missing data

4	(0.738) 0.739 0.747

1	(0.262) 0.261 0.253

Locus 7995 :

2 alleles

99.2% missing data

2	(0.393) 0.393 0.400

1	(0.607) 0.607 0.600

Locus 7996 :

2 alleles

84.4% missing data

3	(0.932) 0.929 0.946

2	(0.068) 0.071 0.054

Locus 7997 :

2 alleles

98.8% missing data

2	(0.738) 0.742 0.737

3	(0.262) 0.258 0.263

Locus 7998 :

2 alleles

99.2% missing data

2	(0.239) 0.244 0.240

1	(0.761) 0.756 0.760

Locus 7999 :

2 alleles

99.2% missing data

1	(0.719) 0.716 0.727

3	(0.281) 0.284 0.273

Locus 8000 :

2 alleles

99.2% missing data

1	(0.278) 0.276 0.280

2	(0.722) 0.724 0.720

Locus 8001 :

2 alleles

99.2% missing data

3	(0.631) 0.636 0.628

1	(0.369) 0.364 0.372

Locus 8002 :

2 alleles

98.8% missing data

3	(0.649) 0.650 0.653

1	(0.351) 0.350 0.347

Locus 8003 :

2 alleles

99.2% missing data

4	(0.236) 0.236 0.239

1	(0.764) 0.764 0.761

Locus 8004 :

2 alleles

99.2% missing data

3	(0.603) 0.597 0.610

4	(0.397) 0.403 0.390

Locus 8005 :

2 alleles

98.8% missing data

1	(0.390) 0.379 0.394

4	(0.610) 0.621 0.606

Locus 8006 :

2 alleles

98.8% missing data

2	(0.520) 0.522 0.518

1	(0.480) 0.478 0.482

Locus 8007 :

2 alleles

97.7% missing data

2	(0.173) 0.171 0.175

1	(0.827) 0.829 0.825

Locus 8008 :

2 alleles

96.1% missing data

3	(0.700) 0.718 0.695

4	(0.300) 0.282 0.305

Locus 8009 :

2 alleles

99.2% missing data

2	(0.748) 0.751 0.749

4	(0.252) 0.249 0.251

Locus 8010 :

2 alleles

99.2% missing data

4	(0.501) 0.496 0.502

2	(0.499) 0.504 0.498

Locus 8011 :

2 alleles

99.2% missing data

4	(0.557) 0.552 0.558

1	(0.443) 0.448 0.442

Locus 8012 :

2 alleles

99.2% missing data

4	(0.816) 0.821 0.813

2	(0.184) 0.179 0.187

Locus 8013 :

2 alleles

99.2% missing data

1	(0.564) 0.567 0.564

2	(0.436) 0.433 0.436

Locus 8014 :

2 alleles

99.2% missing data

1	(0.624) 0.617 0.628

3	(0.376) 0.383 0.372

Locus 8015 :

2 alleles

99.2% missing data

1	(0.389) 0.387 0.387

2	(0.611) 0.613 0.613

Locus 8016 :

2 alleles

83.6% missing data

4	(0.507) 0.566 0.444

1	(0.493) 0.434 0.556

Locus 8017 :

2 alleles

99.2% missing data

2	(0.676) 0.673 0.674

1	(0.324) 0.327 0.326

Locus 8018 :

2 alleles

99.2% missing data

3	(0.381) 0.377 0.384

1	(0.619) 0.623 0.616

Locus 8019 :

2 alleles

99.2% missing data

3	(0.521) 0.519 0.526

4	(0.479) 0.481 0.474

Locus 8020 :

3 alleles

98.4% missing data

1	(0.328) 0.332 0.328

2	(0.244) 0.249 0.247

3	(0.428) 0.419 0.425

Locus 8021 :

3 alleles

98.8% missing data

1	(0.192) 0.193 0.191

2	(0.467) 0.472 0.460

4	(0.341) 0.336 0.349

Locus 8022 :

2 alleles

97.7% missing data

4	(0.822) 0.803 0.829

1	(0.178) 0.197 0.171

Locus 8023 :

4 alleles

98.4% missing data

2	(0.265) 0.263 0.268

4	(0.244) 0.242 0.249

1	(0.185) 0.186 0.178

3	(0.306) 0.309 0.305

Locus 8024 :

2 alleles

98.0% missing data

1	(0.282) 0.275 0.283

3	(0.718) 0.725 0.717

Locus 8025 :

2 alleles

98.8% missing data

4	(0.585) 0.575 0.590

3	(0.415) 0.425 0.410

Locus 8026 :

2 alleles

11.3% missing data

1	(0.976) 0.955 0.993

4	(0.024) 0.045 0.007

Locus 8027 :

2 alleles

8.2% missing data

2	(0.992) 0.984 0.998

1	(0.008) 0.016 0.002

Locus 8028 :

2 alleles

99.2% missing data

2	(0.472) 0.471 0.472

1	(0.528) 0.529 0.528

Locus 8029 :

2 alleles

98.4% missing data

4	(0.777) 0.775 0.774

2	(0.223) 0.225 0.226

Locus 8030 :

2 alleles

99.2% missing data

3	(0.296) 0.298 0.294

4	(0.704) 0.702 0.706

Locus 8031 :

2 alleles

96.1% missing data

4	(0.153) 0.147 0.152

1	(0.847) 0.853 0.848

Locus 8032 :

2 alleles

99.2% missing data

3	(0.355) 0.353 0.364

1	(0.645) 0.647 0.636

Locus 8033 :

2 alleles

99.2% missing data

4	(0.467) 0.465 0.467

2	(0.533) 0.535 0.533

Locus 8034 :

2 alleles

96.1% missing data

1	(0.801) 0.814 0.800

2	(0.199) 0.186 0.200

Locus 8035 :

2 alleles

99.2% missing data

1	(0.267) 0.270 0.262

4	(0.733) 0.730 0.738

Locus 8036 :

2 alleles

98.0% missing data

1	(0.723) 0.725 0.728

2	(0.277) 0.275 0.272

Locus 8037 :

4 alleles

98.4% missing data

3	(0.220) 0.213 0.226

1	(0.380) 0.378 0.383

2	(0.187) 0.192 0.182

4	(0.213) 0.217 0.208

Locus 8038 :

2 alleles

98.8% missing data

1	(0.791) 0.797 0.788

4	(0.209) 0.203 0.212

Locus 8039 :

2 alleles

99.2% missing data

3	(0.529) 0.521 0.537

1	(0.471) 0.479 0.463

Locus 8040 :

2 alleles

98.4% missing data

1	(0.719) 0.726 0.714

3	(0.281) 0.274 0.286

Locus 8041 :

2 alleles

99.2% missing data

1	(0.519) 0.514 0.513

4	(0.481) 0.486 0.487

Locus 8042 :

2 alleles

98.8% missing data

1	(0.181) 0.183 0.181

2	(0.819) 0.817 0.819

Locus 8043 :

2 alleles

98.4% missing data

1	(0.564) 0.575 0.557

4	(0.436) 0.425 0.443

Locus 8044 :

2 alleles

96.5% missing data

4	(0.093) 0.087 0.099

2	(0.907) 0.913 0.901

Locus 8045 :

2 alleles

99.2% missing data

3	(0.401) 0.409 0.395

4	(0.599) 0.591 0.605

Locus 8046 :

2 alleles

98.8% missing data

4	(0.603) 0.603 0.608

1	(0.397) 0.397 0.392

Locus 8047 :

2 alleles

99.2% missing data

1	(0.388) 0.387 0.382

4	(0.612) 0.613 0.618

Locus 8048 :

2 alleles

99.2% missing data

2	(0.576) 0.571 0.584

3	(0.424) 0.429 0.416

Locus 8049 :

2 alleles

99.2% missing data

4	(0.447) 0.445 0.447

1	(0.553) 0.555 0.553

Locus 8050 :

2 alleles

99.2% missing data

3	(0.356) 0.350 0.362

4	(0.644) 0.650 0.638

Locus 8051 :

2 alleles

99.2% missing data

1	(0.637) 0.636 0.635

4	(0.363) 0.364 0.365

Locus 8052 :

2 alleles

0.0% missing data

4	(0.983) 0.987 0.992

3	(0.017) 0.013 0.008

Locus 8053 :

2 alleles

99.2% missing data

2	(0.381) 0.380 0.386

3	(0.619) 0.620 0.614

Locus 8054 :

2 alleles

99.2% missing data

1	(0.399) 0.402 0.404

4	(0.601) 0.598 0.596

Locus 8055 :

2 alleles

98.8% missing data

1	(0.294) 0.285 0.300

4	(0.706) 0.715 0.700

Locus 8056 :

2 alleles

99.2% missing data

1	(0.628) 0.630 0.623

4	(0.372) 0.370 0.377

Locus 8057 :

2 alleles

99.2% missing data

4	(0.486) 0.484 0.490

3	(0.514) 0.516 0.510

Locus 8058 :

2 alleles

95.7% missing data

4	(0.883) 0.885 0.881

3	(0.117) 0.115 0.119

Locus 8059 :

3 alleles

98.8% missing data

3	(0.247) 0.249 0.240

1	(0.311) 0.306 0.321

4	(0.441) 0.445 0.439

Locus 8060 :

2 alleles

99.2% missing data

1	(0.518) 0.521 0.518

3	(0.482) 0.479 0.482

Locus 8061 :

2 alleles

99.2% missing data

1	(0.396) 0.395 0.397

2	(0.604) 0.605 0.603

Locus 8062 :

2 alleles

99.2% missing data

2	(0.316) 0.315 0.321

1	(0.684) 0.685 0.679

Locus 8063 :

2 alleles

98.4% missing data

1	(0.742) 0.742 0.736

3	(0.258) 0.258 0.264

Locus 8064 :

3 alleles

98.8% missing data

2	(0.359) 0.356 0.366

1	(0.299) 0.308 0.290

3	(0.342) 0.337 0.344

Locus 8065 :

2 alleles

98.8% missing data

2	(0.127) 0.127 0.135

3	(0.873) 0.873 0.865

Locus 8066 :

2 alleles

99.2% missing data

4	(0.792) 0.787 0.794

3	(0.208) 0.213 0.206

Locus 8067 :

2 alleles

93.0% missing data

3	(0.862) 0.854 0.879

2	(0.138) 0.146 0.121

Locus 8068 :

2 alleles

99.2% missing data

4	(0.703) 0.697 0.710

1	(0.297) 0.303 0.290

Locus 8069 :

2 alleles

99.2% missing data

1	(0.563) 0.562 0.562

3	(0.437) 0.438 0.438

Locus 8070 :

2 alleles

99.2% missing data

1	(0.516) 0.515 0.516

3	(0.484) 0.485 0.484

Locus 8071 :

2 alleles

98.8% missing data

1	(0.571) 0.578 0.569

4	(0.429) 0.422 0.431

Locus 8072 :

3 alleles

98.8% missing data

2	(0.204) 0.199 0.206

1	(0.351) 0.355 0.350

4	(0.445) 0.446 0.444

Locus 8073 :

2 alleles

98.0% missing data

1	(0.657) 0.656 0.655

3	(0.343) 0.344 0.345

Locus 8074 :

2 alleles

98.8% missing data

3	(0.444) 0.439 0.448

2	(0.556) 0.561 0.552

Locus 8075 :

2 alleles

93.0% missing data

1	(0.922) 0.918 0.930

2	(0.078) 0.082 0.070

Locus 8076 :

2 alleles

98.4% missing data

1	(0.803) 0.804 0.797

3	(0.197) 0.196 0.203

Locus 8077 :

3 alleles

92.2% missing data

3	(0.065) 0.066 0.062

1	(0.842) 0.831 0.859

4	(0.093) 0.103 0.078

Locus 8078 :

2 alleles

99.2% missing data

3	(0.689) 0.687 0.682

2	(0.311) 0.313 0.318

Locus 8079 :

2 alleles

99.2% missing data

1	(0.758) 0.753 0.763

4	(0.242) 0.247 0.237

Locus 8080 :

2 alleles

98.0% missing data

1	(0.724) 0.717 0.733

3	(0.276) 0.283 0.267

Locus 8081 :

2 alleles

99.2% missing data

2	(0.646) 0.642 0.643

1	(0.354) 0.358 0.357

Locus 8082 :

3 alleles

98.8% missing data

1	(0.523) 0.516 0.529

2	(0.144) 0.148 0.146

3	(0.333) 0.336 0.325

Locus 8083 :

2 alleles

98.8% missing data

4	(0.284) 0.285 0.282

2	(0.716) 0.715 0.718

Locus 8084 :

2 alleles

99.2% missing data

1	(0.421) 0.421 0.425

3	(0.579) 0.579 0.575

Locus 8085 :

2 alleles

99.2% missing data

3	(0.681) 0.682 0.675

1	(0.319) 0.318 0.325

Locus 8086 :

2 alleles

99.2% missing data

4	(0.651) 0.661 0.644

2	(0.349) 0.339 0.356

Locus 8087 :

2 alleles

97.7% missing data

3	(0.623) 0.619 0.638

1	(0.377) 0.381 0.362

Locus 8088 :

2 alleles

90.2% missing data

1	(0.700) 0.716 0.699

2	(0.300) 0.284 0.301

Locus 8089 :

3 alleles

83.6% missing data

1	(0.469) 0.508 0.443

3	(0.374) 0.343 0.408

2	(0.157) 0.149 0.149

Locus 8090 :

2 alleles

98.0% missing data

1	(0.730) 0.735 0.732

3	(0.270) 0.265 0.268

Locus 8091 :

2 alleles

99.2% missing data

1	(0.727) 0.724 0.731

2	(0.273) 0.276 0.269

Locus 8092 :

2 alleles

85.5% missing data

1	(0.914) 0.917 0.923

2	(0.086) 0.083 0.077

Locus 8093 :

2 alleles

95.3% missing data

1	(0.791) 0.792 0.803

2	(0.209) 0.208 0.197

Locus 8094 :

3 alleles

96.9% missing data

1	(0.533) 0.527 0.546

2	(0.286) 0.291 0.283

4	(0.181) 0.182 0.171

Locus 8095 :

3 alleles

94.9% missing data

1	(0.504) 0.506 0.509

3	(0.142) 0.142 0.133

4	(0.354) 0.352 0.358

Locus 8096 :

2 alleles

99.2% missing data

3	(0.564) 0.554 0.569

2	(0.436) 0.446 0.431

Locus 8097 :

2 alleles

98.8% missing data

1	(0.742) 0.740 0.739

2	(0.258) 0.260 0.261

Locus 8098 :

2 alleles

96.1% missing data

2	(0.318) 0.313 0.322

1	(0.682) 0.687 0.678

Locus 8099 :

2 alleles

5.1% missing data

4	(0.986) 0.989 0.993

1	(0.014) 0.011 0.007

Locus 8100 :

2 alleles

99.2% missing data

4	(0.732) 0.737 0.730

3	(0.268) 0.263 0.270

Locus 8101 :

2 alleles

99.2% missing data

4	(0.469) 0.465 0.475

2	(0.531) 0.535 0.525

Locus 8102 :

2 alleles

98.8% missing data

1	(0.607) 0.609 0.606

3	(0.393) 0.391 0.394

Locus 8103 :

2 alleles

99.2% missing data

4	(0.429) 0.426 0.423

1	(0.571) 0.574 0.577

Locus 8104 :

2 alleles

94.9% missing data

4	(0.766) 0.749 0.791

1	(0.234) 0.251 0.209

Locus 8105 :

2 alleles

95.7% missing data

1	(0.774) 0.773 0.773

2	(0.226) 0.227 0.227

Locus 8106 :

2 alleles

98.4% missing data

2	(0.505) 0.505 0.517

1	(0.495) 0.495 0.483

Locus 8107 :

2 alleles

96.1% missing data

2	(0.297) 0.305 0.286

1	(0.703) 0.695 0.714

Locus 8108 :

2 alleles

97.3% missing data

1	(0.657) 0.654 0.664

2	(0.343) 0.346 0.336

Locus 8109 :

2 alleles

98.0% missing data

2	(0.584) 0.588 0.580

1	(0.416) 0.412 0.420

Locus 8110 :

2 alleles

98.8% missing data

1	(0.445) 0.444 0.453

4	(0.555) 0.556 0.547

Locus 8111 :

2 alleles

99.2% missing data

1	(0.412) 0.414 0.414

2	(0.588) 0.586 0.586

Locus 8112 :

2 alleles

98.4% missing data

2	(0.390) 0.393 0.386

1	(0.610) 0.607 0.614

Locus 8113 :

2 alleles

98.8% missing data

1	(0.500) 0.501 0.501

2	(0.500) 0.499 0.499

Locus 8114 :

2 alleles

98.8% missing data

1	(0.770) 0.776 0.760

2	(0.230) 0.224 0.240

Locus 8115 :

2 alleles

97.7% missing data

1	(0.657) 0.650 0.660

2	(0.343) 0.350 0.340

Locus 8116 :

2 alleles

98.8% missing data

1	(0.740) 0.741 0.742

4	(0.260) 0.259 0.258

Locus 8117 :

2 alleles

98.8% missing data

1	(0.458) 0.453 0.466

3	(0.542) 0.547 0.534

Locus 8118 :

2 alleles

98.4% missing data

2	(0.509) 0.517 0.507

1	(0.491) 0.483 0.493

Locus 8119 :

2 alleles

99.2% missing data

1	(0.510) 0.515 0.503

2	(0.490) 0.485 0.497

Locus 8120 :

2 alleles

98.8% missing data

4	(0.609) 0.613 0.600

2	(0.391) 0.387 0.400

Locus 8121 :

2 alleles

99.2% missing data

1	(0.477) 0.473 0.478

3	(0.523) 0.527 0.522

Locus 8122 :

2 alleles

99.2% missing data

1	(0.669) 0.667 0.674

4	(0.331) 0.333 0.326

Locus 8123 :

2 alleles

96.9% missing data

4	(0.809) 0.801 0.821

3	(0.191) 0.199 0.179

Locus 8124 :

2 alleles

94.1% missing data

4	(0.637) 0.663 0.614

3	(0.363) 0.337 0.386

Locus 8125 :

2 alleles

99.2% missing data

1	(0.228) 0.238 0.225

3	(0.772) 0.762 0.775

Locus 8126 :

2 alleles

98.8% missing data

4	(0.476) 0.467 0.481

3	(0.524) 0.533 0.519

Locus 8127 :

2 alleles

99.2% missing data

1	(0.322) 0.326 0.325

2	(0.678) 0.674 0.675

Locus 8128 :

2 alleles

99.2% missing data

1	(0.487) 0.483 0.492

2	(0.513) 0.517 0.508

Locus 8129 :

2 alleles

99.2% missing data

4	(0.369) 0.372 0.373

3	(0.631) 0.628 0.627

Locus 8130 :

2 alleles

98.4% missing data

2	(0.680) 0.684 0.683

1	(0.320) 0.316 0.317

Locus 8131 :

2 alleles

99.2% missing data

4	(0.611) 0.609 0.609

3	(0.389) 0.391 0.391

Locus 8132 :

3 alleles

96.5% missing data

1	(0.594) 0.603 0.583

3	(0.059) 0.063 0.058

2	(0.347) 0.334 0.359

Locus 8133 :

3 alleles

97.3% missing data

1	(0.475) 0.473 0.483

2	(0.221) 0.217 0.222

3	(0.304) 0.309 0.295

Locus 8134 :

2 alleles

98.8% missing data

2	(0.347) 0.349 0.347

1	(0.653) 0.651 0.653

Locus 8135 :

2 alleles

97.7% missing data

2	(0.177) 0.181 0.166

1	(0.823) 0.819 0.834

Locus 8136 :

3 alleles

94.5% missing data

4	(0.680) 0.677 0.699

1	(0.151) 0.156 0.131

2	(0.168) 0.167 0.170

Locus 8137 :

3 alleles

98.8% missing data

2	(0.408) 0.415 0.398

1	(0.437) 0.433 0.440

3	(0.155) 0.152 0.162

Locus 8138 :

2 alleles

99.2% missing data

1	(0.487) 0.485 0.481

3	(0.513) 0.515 0.519

Locus 8139 :

2 alleles

99.2% missing data

4	(0.687) 0.690 0.684

1	(0.313) 0.310 0.316

Locus 8140 :

2 alleles

99.2% missing data

3	(0.650) 0.652 0.644

4	(0.350) 0.348 0.356

Locus 8141 :

2 alleles

99.2% missing data

3	(0.495) 0.494 0.494

2	(0.505) 0.506 0.506

Locus 8142 :

2 alleles

97.3% missing data

1	(0.433) 0.447 0.432

2	(0.567) 0.553 0.568

Locus 8143 :

3 alleles

98.0% missing data

1	(0.363) 0.372 0.356

2	(0.550) 0.541 0.552

3	(0.087) 0.087 0.092

Locus 8144 :

2 alleles

97.3% missing data

1	(0.593) 0.591 0.592

2	(0.407) 0.409 0.408

Locus 8145 :

2 alleles

99.2% missing data

3	(0.272) 0.276 0.268

1	(0.728) 0.724 0.732

Locus 8146 :

2 alleles

98.8% missing data

2	(0.367) 0.368 0.362

1	(0.633) 0.632 0.638

Locus 8147 :

2 alleles

99.2% missing data

2	(0.455) 0.454 0.447

1	(0.545) 0.546 0.553

Locus 8148 :

2 alleles

97.7% missing data

4	(0.314) 0.301 0.319

3	(0.686) 0.699 0.681

Locus 8149 :

2 alleles

99.2% missing data

1	(0.673) 0.671 0.671

2	(0.327) 0.329 0.329

Locus 8150 :

2 alleles

99.2% missing data

3	(0.655) 0.649 0.658

4	(0.345) 0.351 0.342

Locus 8151 :

2 alleles

98.0% missing data

2	(0.444) 0.447 0.437

1	(0.556) 0.553 0.563

Locus 8152 :

2 alleles

99.2% missing data

1	(0.592) 0.585 0.598

2	(0.408) 0.415 0.402

Locus 8153 :

2 alleles

96.1% missing data

2	(0.843) 0.850 0.845

1	(0.157) 0.150 0.155

Locus 8154 :

2 alleles

98.8% missing data

1	(0.395) 0.401 0.397

4	(0.605) 0.599 0.603

Locus 8155 :

2 alleles

99.2% missing data

1	(0.145) 0.149 0.142

4	(0.855) 0.851 0.858

Locus 8156 :

2 alleles

98.8% missing data

4	(0.790) 0.793 0.787

1	(0.210) 0.207 0.213

Locus 8157 :

3 alleles

94.1% missing data

3	(0.654) 0.636 0.688

4	(0.221) 0.233 0.205

2	(0.124) 0.131 0.108

Locus 8158 :

2 alleles

97.7% missing data

2	(0.750) 0.751 0.755

4	(0.250) 0.249 0.245

Locus 8159 :

2 alleles

99.2% missing data

3	(0.421) 0.416 0.421

1	(0.579) 0.584 0.579

Locus 8160 :

2 alleles

99.2% missing data

4	(0.666) 0.664 0.664

1	(0.334) 0.336 0.336

Locus 8161 :

2 alleles

99.2% missing data

3	(0.394) 0.386 0.402

1	(0.606) 0.614 0.598

Locus 8162 :

3 alleles

97.3% missing data

1	(0.630) 0.633 0.631

4	(0.186) 0.187 0.182

2	(0.184) 0.179 0.187

Locus 8163 :

2 alleles

99.2% missing data

4	(0.487) 0.486 0.488

2	(0.513) 0.514 0.512

Locus 8164 :

2 alleles

99.2% missing data

4	(0.572) 0.564 0.578

2	(0.428) 0.436 0.422

Locus 8165 :

2 alleles

99.2% missing data

2	(0.436) 0.427 0.441

4	(0.564) 0.573 0.559

Locus 8166 :

2 alleles

99.2% missing data

1	(0.742) 0.743 0.735

3	(0.258) 0.257 0.265

Locus 8167 :

2 alleles

96.9% missing data

3	(0.803) 0.804 0.810

2	(0.197) 0.196 0.190

Locus 8168 :

2 alleles

99.2% missing data

1	(0.336) 0.341 0.332

2	(0.664) 0.659 0.668

Locus 8169 :

2 alleles

98.8% missing data

1	(0.559) 0.558 0.561

4	(0.441) 0.442 0.439

Locus 8170 :

2 alleles

99.2% missing data

1	(0.178) 0.183 0.179

3	(0.822) 0.817 0.821

Locus 8171 :

2 alleles

99.2% missing data

4	(0.734) 0.735 0.731

3	(0.266) 0.265 0.269

Locus 8172 :

2 alleles

99.2% missing data

2	(0.532) 0.528 0.536

4	(0.468) 0.472 0.464

Locus 8173 :

2 alleles

0.0% missing data

2	(0.919) 0.771 0.975

3	(0.081) 0.229 0.025

Locus 8174 :

2 alleles

93.8% missing data

4	(0.769) 0.769 0.792

2	(0.231) 0.231 0.208

Locus 8175 :

2 alleles

8.2% missing data

4	(0.987) 0.980 0.996

3	(0.013) 0.020 0.004

Locus 8176 :

2 alleles

96.1% missing data

1	(0.186) 0.180 0.185

4	(0.814) 0.820 0.815

Locus 8177 :

2 alleles

99.2% missing data

1	(0.693) 0.695 0.687

4	(0.307) 0.305 0.313

Locus 8178 :

2 alleles

97.7% missing data

1	(0.856) 0.851 0.863

4	(0.144) 0.149 0.137

Locus 8179 :

2 alleles

96.1% missing data

4	(0.749) 0.758 0.748

3	(0.251) 0.242 0.252

Locus 8180 :

2 alleles

99.2% missing data

2	(0.481) 0.480 0.485

4	(0.519) 0.520 0.515

Locus 8181 :

2 alleles

99.2% missing data

3	(0.422) 0.420 0.428

1	(0.578) 0.580 0.572

Locus 8182 :

2 alleles

96.9% missing data

4	(0.777) 0.772 0.787

1	(0.223) 0.228 0.213

Locus 8183 :

2 alleles

99.2% missing data

4	(0.699) 0.695 0.704

2	(0.301) 0.305 0.296

Locus 8184 :

2 alleles

96.9% missing data

3	(0.708) 0.705 0.716

1	(0.292) 0.295 0.284

Locus 8185 :

2 alleles

99.2% missing data

3	(0.608) 0.605 0.608

2	(0.392) 0.395 0.392

Locus 8186 :

2 alleles

93.0% missing data

2	(0.899) 0.901 0.908

3	(0.101) 0.099 0.092

Locus 8187 :

2 alleles

99.2% missing data

1	(0.638) 0.639 0.629

3	(0.362) 0.361 0.371

Locus 8188 :

2 alleles

99.2% missing data

3	(0.492) 0.490 0.502

1	(0.508) 0.510 0.498

Locus 8189 :

2 alleles

99.2% missing data

4	(0.465) 0.470 0.463

1	(0.535) 0.530 0.537

Locus 8190 :

2 alleles

98.4% missing data

2	(0.448) 0.444 0.458

1	(0.552) 0.556 0.542

Locus 8191 :

2 alleles

98.8% missing data

4	(0.647) 0.652 0.648

3	(0.353) 0.348 0.352

Locus 8192 :

2 alleles

99.2% missing data

3	(0.606) 0.608 0.604

1	(0.394) 0.392 0.396

Locus 8193 :

3 alleles

98.8% missing data

4	(0.465) 0.460 0.463

1	(0.137) 0.144 0.133

3	(0.398) 0.396 0.404

Locus 8194 :

2 alleles

99.2% missing data

2	(0.404) 0.399 0.409

3	(0.596) 0.601 0.591

Locus 8195 :

2 alleles

99.2% missing data

3	(0.548) 0.545 0.559

1	(0.452) 0.455 0.441

Locus 8196 :

2 alleles

99.2% missing data

1	(0.315) 0.314 0.319

2	(0.685) 0.686 0.681

Locus 8197 :

2 alleles

99.2% missing data

2	(0.301) 0.298 0.305

3	(0.699) 0.702 0.695

Locus 8198 :

2 alleles

99.2% missing data

2	(0.813) 0.809 0.815

3	(0.187) 0.191 0.185

Locus 8199 :

2 alleles

99.2% missing data

4	(0.406) 0.408 0.405

3	(0.594) 0.592 0.595

Locus 8200 :

2 alleles

96.1% missing data

4	(0.866) 0.879 0.863

1	(0.134) 0.121 0.137

Locus 8201 :

2 alleles

0.0% missing data

2	(0.987) 0.981 0.996

3	(0.013) 0.019 0.004

Locus 8202 :

2 alleles

99.2% missing data

4	(0.410) 0.404 0.412

3	(0.590) 0.596 0.588

Locus 8203 :

2 alleles

99.2% missing data

2	(0.635) 0.628 0.639

1	(0.365) 0.372 0.361

Locus 8204 :

2 alleles

99.2% missing data

1	(0.687) 0.691 0.684

3	(0.313) 0.309 0.316

Locus 8205 :

2 alleles

98.8% missing data

4	(0.644) 0.640 0.649

1	(0.356) 0.360 0.351

Locus 8206 :

2 alleles

99.2% missing data

1	(0.388) 0.383 0.388

3	(0.612) 0.617 0.612

Locus 8207 :

2 alleles

99.2% missing data

2	(0.339) 0.341 0.349

4	(0.661) 0.659 0.651

Locus 8208 :

2 alleles

99.2% missing data

2	(0.639) 0.648 0.639

4	(0.361) 0.352 0.361

Locus 8209 :

2 alleles

98.8% missing data

2	(0.542) 0.536 0.550

1	(0.458) 0.464 0.450

Locus 8210 :

2 alleles

99.2% missing data

2	(0.612) 0.608 0.608

1	(0.388) 0.392 0.392

Locus 8211 :

2 alleles

90.6% missing data

1	(0.756) 0.745 0.770

2	(0.244) 0.255 0.230

Locus 8212 :

2 alleles

96.9% missing data

2	(0.414) 0.415 0.409

1	(0.586) 0.585 0.591

Locus 8213 :

2 alleles

94.9% missing data

1	(0.699) 0.702 0.701

2	(0.301) 0.298 0.299

Locus 8214 :

3 alleles

98.4% missing data

4	(0.276) 0.272 0.279

2	(0.443) 0.453 0.437

1	(0.281) 0.276 0.284

Locus 8215 :

2 alleles

98.8% missing data

1	(0.290) 0.287 0.290

4	(0.710) 0.713 0.710

Locus 8216 :

3 alleles

94.9% missing data

1	(0.757) 0.775 0.749

2	(0.121) 0.108 0.124

4	(0.123) 0.117 0.127

Locus 8217 :

2 alleles

99.2% missing data

2	(0.677) 0.681 0.670

3	(0.323) 0.319 0.330

Locus 8218 :

2 alleles

98.8% missing data

1	(0.790) 0.788 0.791

2	(0.210) 0.212 0.209

Locus 8219 :

2 alleles

98.8% missing data

1	(0.760) 0.765 0.759

2	(0.240) 0.235 0.241

Locus 8220 :

2 alleles

99.2% missing data

2	(0.283) 0.291 0.285

1	(0.717) 0.709 0.715

Locus 8221 :

2 alleles

99.2% missing data

1	(0.436) 0.435 0.433

4	(0.564) 0.565 0.567

Locus 8222 :

2 alleles

97.7% missing data

1	(0.385) 0.386 0.373

4	(0.615) 0.614 0.627

Locus 8223 :

2 alleles

98.8% missing data

1	(0.526) 0.519 0.534

2	(0.474) 0.481 0.466

Locus 8224 :

2 alleles

91.0% missing data

1	(0.924) 0.938 0.915

2	(0.076) 0.062 0.085

Locus 8225 :

2 alleles

99.2% missing data

3	(0.455) 0.452 0.454

2	(0.545) 0.548 0.546

Locus 8226 :

3 alleles

98.8% missing data

2	(0.566) 0.568 0.560

1	(0.255) 0.253 0.258

4	(0.179) 0.179 0.181

Locus 8227 :

3 alleles

93.8% missing data

1	(0.587) 0.581 0.603

3	(0.089) 0.099 0.078

4	(0.323) 0.320 0.319

Locus 8228 :

2 alleles

96.9% missing data

1	(0.735) 0.741 0.730

2	(0.265) 0.259 0.270

Locus 8229 :

2 alleles

98.0% missing data

1	(0.720) 0.710 0.709

2	(0.280) 0.290 0.291

Locus 8230 :

2 alleles

97.3% missing data

4	(0.651) 0.663 0.644

1	(0.349) 0.337 0.356

Locus 8231 :

3 alleles

98.0% missing data

1	(0.592) 0.589 0.595

4	(0.193) 0.192 0.192

2	(0.215) 0.220 0.213

Locus 8232 :

2 alleles

99.2% missing data

1	(0.259) 0.252 0.267

2	(0.741) 0.748 0.733

Locus 8233 :

2 alleles

93.0% missing data

4	(0.875) 0.887 0.880

1	(0.125) 0.113 0.120

Locus 8234 :

2 alleles

97.3% missing data

3	(0.729) 0.728 0.745

1	(0.271) 0.272 0.255

Locus 8235 :

2 alleles

98.4% missing data

1	(0.702) 0.699 0.710

2	(0.298) 0.301 0.290

Locus 8236 :

3 alleles

98.8% missing data

2	(0.188) 0.182 0.189

1	(0.281) 0.283 0.289

4	(0.530) 0.535 0.522

Locus 8237 :

2 alleles

99.2% missing data

4	(0.579) 0.576 0.581

3	(0.421) 0.424 0.419

Locus 8238 :

2 alleles

98.0% missing data

1	(0.523) 0.535 0.523

2	(0.477) 0.465 0.477

Locus 8239 :

2 alleles

98.4% missing data

2	(0.580) 0.587 0.580

1	(0.420) 0.413 0.420

Locus 8240 :

2 alleles

99.2% missing data

3	(0.712) 0.712 0.706

4	(0.288) 0.288 0.294

Locus 8241 :

2 alleles

94.5% missing data

2	(0.891) 0.893 0.898

1	(0.109) 0.107 0.102

Locus 8242 :

2 alleles

99.2% missing data

2	(0.521) 0.518 0.522

1	(0.479) 0.482 0.478

Locus 8243 :

2 alleles

99.2% missing data

2	(0.479) 0.482 0.487

3	(0.521) 0.518 0.513

Locus 8244 :

2 alleles

99.2% missing data

1	(0.230) 0.231 0.229

2	(0.770) 0.769 0.771

Locus 8245 :

2 alleles

99.2% missing data

3	(0.174) 0.173 0.179

4	(0.826) 0.827 0.821

Locus 8246 :

2 alleles

95.7% missing data

4	(0.887) 0.895 0.879

1	(0.113) 0.105 0.121

Locus 8247 :

2 alleles

99.2% missing data

4	(0.610) 0.612 0.612

1	(0.390) 0.388 0.388

Locus 8248 :

2 alleles

98.0% missing data

2	(0.530) 0.536 0.528

1	(0.470) 0.464 0.472

Locus 8249 :

2 alleles

99.2% missing data

2	(0.565) 0.565 0.565

4	(0.435) 0.435 0.435

Locus 8250 :

2 alleles

96.1% missing data

3	(0.782) 0.790 0.783

2	(0.218) 0.210 0.217

Locus 8251 :

2 alleles

99.2% missing data

1	(0.438) 0.439 0.444

2	(0.562) 0.561 0.556

Locus 8252 :

2 alleles

97.7% missing data

4	(0.381) 0.389 0.367

1	(0.619) 0.611 0.633

Locus 8253 :

2 alleles

99.2% missing data

1	(0.202) 0.208 0.210

4	(0.798) 0.792 0.790

Locus 8254 :

3 alleles

96.9% missing data

2	(0.273) 0.281 0.271

1	(0.589) 0.575 0.601

4	(0.137) 0.144 0.128

Locus 8255 :

2 alleles

96.9% missing data

1	(0.630) 0.630 0.632

2	(0.370) 0.370 0.368

Locus 8256 :

2 alleles

98.4% missing data

1	(0.608) 0.613 0.606

2	(0.392) 0.387 0.394

Locus 8257 :

2 alleles

97.7% missing data

2	(0.551) 0.560 0.552

1	(0.449) 0.440 0.448

Locus 8258 :

2 alleles

99.2% missing data

2	(0.257) 0.249 0.266

3	(0.743) 0.751 0.734

Locus 8259 :

2 alleles

99.2% missing data

3	(0.308) 0.303 0.313

2	(0.692) 0.697 0.687

Locus 8260 :

2 alleles

97.7% missing data

1	(0.532) 0.527 0.541

2	(0.468) 0.473 0.459

Locus 8261 :

3 alleles

98.4% missing data

4	(0.300) 0.291 0.303

2	(0.361) 0.367 0.356

1	(0.340) 0.342 0.341

Locus 8262 :

3 alleles

98.8% missing data

3	(0.441) 0.448 0.437

4	(0.215) 0.210 0.218

1	(0.344) 0.342 0.345

Locus 8263 :

2 alleles

98.8% missing data

1	(0.658) 0.655 0.663

4	(0.342) 0.345 0.337

Locus 8264 :

2 alleles

99.2% missing data

4	(0.624) 0.624 0.628

2	(0.376) 0.376 0.372

Locus 8265 :

2 alleles

98.8% missing data

3	(0.639) 0.645 0.634

1	(0.361) 0.355 0.366

Locus 8266 :

4 alleles

97.3% missing data

4	(0.199) 0.202 0.193

1	(0.133) 0.132 0.134

2	(0.397) 0.396 0.403

3	(0.271) 0.269 0.270

Locus 8267 :

2 alleles

99.2% missing data

2	(0.417) 0.409 0.425

1	(0.583) 0.591 0.575

Locus 8268 :

2 alleles

9.4% missing data

3	(0.980) 0.970 0.994

1	(0.020) 0.030 0.006

Locus 8269 :

2 alleles

99.2% missing data

4	(0.896) 0.898 0.891

2	(0.104) 0.102 0.109

Locus 8270 :

3 alleles

97.3% missing data

2	(0.526) 0.531 0.528

4	(0.204) 0.197 0.204

1	(0.270) 0.272 0.268

Locus 8271 :

2 alleles

91.4% missing data

2	(0.889) 0.889 0.891

1	(0.111) 0.111 0.109

Locus 8272 :

3 alleles

98.0% missing data

4	(0.423) 0.415 0.433

1	(0.304) 0.296 0.306

3	(0.273) 0.288 0.261

Locus 8273 :

2 alleles

99.2% missing data

2	(0.420) 0.416 0.414

4	(0.580) 0.584 0.586

Locus 8274 :

2 alleles

99.2% missing data

2	(0.363) 0.357 0.367

1	(0.637) 0.643 0.633

Locus 8275 :

2 alleles

98.0% missing data

4	(0.702) 0.706 0.704

1	(0.298) 0.294 0.296

Locus 8276 :

2 alleles

0.0% missing data

1	(0.986) 0.990 0.993

2	(0.014) 0.010 0.007

Locus 8277 :

2 alleles

99.2% missing data

1	(0.213) 0.208 0.214

2	(0.787) 0.792 0.786

Locus 8278 :

2 alleles

96.1% missing data

4	(0.181) 0.173 0.181

3	(0.819) 0.827 0.819

Locus 8279 :

2 alleles

99.2% missing data

4	(0.442) 0.442 0.440

3	(0.558) 0.558 0.560

Locus 8280 :

2 alleles

99.2% missing data

2	(0.499) 0.501 0.501

3	(0.501) 0.499 0.499

Locus 8281 :

2 alleles

99.2% missing data

1	(0.638) 0.641 0.641

2	(0.362) 0.359 0.359

Locus 8282 :

2 alleles

99.2% missing data

1	(0.383) 0.383 0.383

4	(0.617) 0.617 0.617

Locus 8283 :

2 alleles

99.2% missing data

4	(0.252) 0.245 0.259

2	(0.748) 0.755 0.741

Locus 8284 :

2 alleles

99.2% missing data

2	(0.592) 0.588 0.597

4	(0.408) 0.412 0.403

Locus 8285 :

2 alleles

99.2% missing data

1	(0.741) 0.744 0.739

3	(0.259) 0.256 0.261

Locus 8286 :

2 alleles

98.8% missing data

4	(0.715) 0.716 0.715

3	(0.285) 0.284 0.285

Locus 8287 :

2 alleles

99.2% missing data

4	(0.392) 0.394 0.398

1	(0.608) 0.606 0.602

Locus 8288 :

2 alleles

99.2% missing data

4	(0.729) 0.731 0.721

3	(0.271) 0.269 0.279

Locus 8289 :

2 alleles

98.4% missing data

1	(0.857) 0.866 0.850

4	(0.143) 0.134 0.150

Locus 8290 :

2 alleles

90.6% missing data

3	(0.890) 0.911 0.878

1	(0.110) 0.089 0.122

Locus 8291 :

2 alleles

99.2% missing data

4	(0.802) 0.792 0.803

3	(0.198) 0.208 0.197

Locus 8292 :

2 alleles

99.2% missing data

3	(0.332) 0.325 0.343

4	(0.668) 0.675 0.657

Locus 8293 :

3 alleles

98.4% missing data

1	(0.411) 0.402 0.420

3	(0.241) 0.251 0.236

2	(0.348) 0.347 0.344

Locus 8294 :

2 alleles

98.8% missing data

3	(0.670) 0.673 0.670

1	(0.330) 0.327 0.330

Locus 8295 :

2 alleles

99.2% missing data

4	(0.390) 0.390 0.393

3	(0.610) 0.610 0.607

Locus 8296 :

2 alleles

99.2% missing data

2	(0.197) 0.200 0.193

4	(0.803) 0.800 0.807

Locus 8297 :

2 alleles

99.2% missing data

4	(0.664) 0.668 0.658

1	(0.336) 0.332 0.342

Locus 8298 :

2 alleles

99.2% missing data

1	(0.859) 0.862 0.854

3	(0.141) 0.138 0.146

Locus 8299 :

2 alleles

99.2% missing data

4	(0.537) 0.541 0.530

2	(0.463) 0.459 0.470

Locus 8300 :

2 alleles

99.2% missing data

2	(0.683) 0.682 0.683

4	(0.317) 0.318 0.317

Locus 8301 :

2 alleles

98.8% missing data

2	(0.795) 0.798 0.790

1	(0.205) 0.202 0.210

Locus 8302 :

2 alleles

99.2% missing data

3	(0.589) 0.588 0.589

2	(0.411) 0.412 0.411

Locus 8303 :

2 alleles

99.2% missing data

4	(0.504) 0.504 0.496

1	(0.496) 0.496 0.504

Locus 8304 :

2 alleles

98.8% missing data

3	(0.239) 0.246 0.233

4	(0.761) 0.754 0.767

Locus 8305 :

2 alleles

98.4% missing data

4	(0.494) 0.503 0.496

2	(0.506) 0.497 0.504

Locus 8306 :

2 alleles

99.2% missing data

4	(0.244) 0.238 0.246

1	(0.756) 0.762 0.754

Locus 8307 :

2 alleles

99.2% missing data

1	(0.505) 0.502 0.513

3	(0.495) 0.498 0.487

Locus 8308 :

2 alleles

99.2% missing data

4	(0.401) 0.394 0.409

1	(0.599) 0.606 0.591

Locus 8309 :

2 alleles

99.2% missing data

4	(0.291) 0.295 0.289

3	(0.709) 0.705 0.711

Locus 8310 :

2 alleles

99.2% missing data

3	(0.540) 0.545 0.543

4	(0.460) 0.455 0.457

Locus 8311 :

2 alleles

98.8% missing data

2	(0.685) 0.688 0.675

4	(0.315) 0.312 0.325

Locus 8312 :

2 alleles

0.0% missing data

1	(0.980) 0.977 0.991

2	(0.020) 0.023 0.009

Locus 8313 :

2 alleles

91.0% missing data

4	(0.908) 0.922 0.900

3	(0.092) 0.078 0.100

Locus 8314 :

2 alleles

99.2% missing data

3	(0.232) 0.233 0.234

2	(0.768) 0.767 0.766

Locus 8315 :

2 alleles

99.2% missing data

4	(0.628) 0.622 0.632

3	(0.372) 0.378 0.368

Locus 8316 :

2 alleles

98.8% missing data

4	(0.323) 0.318 0.328

1	(0.677) 0.682 0.672

Locus 8317 :

2 alleles

98.8% missing data

1	(0.523) 0.517 0.526

3	(0.477) 0.483 0.474

Locus 8318 :

2 alleles

99.2% missing data

3	(0.319) 0.319 0.320

4	(0.681) 0.681 0.680

Locus 8319 :

2 alleles

99.2% missing data

2	(0.431) 0.431 0.431

3	(0.569) 0.569 0.569

Locus 8320 :

2 alleles

99.2% missing data

4	(0.628) 0.629 0.629

2	(0.372) 0.371 0.371

Locus 8321 :

3 alleles

98.4% missing data

1	(0.336) 0.334 0.347

3	(0.423) 0.429 0.414

2	(0.240) 0.236 0.238

Locus 8322 :

2 alleles

99.2% missing data

1	(0.336) 0.333 0.338

2	(0.664) 0.667 0.662

Locus 8323 :

2 alleles

99.2% missing data

2	(0.456) 0.454 0.464

3	(0.544) 0.546 0.536

Locus 8324 :

2 alleles

99.2% missing data

4	(0.140) 0.143 0.142

1	(0.860) 0.857 0.858

Locus 8325 :

2 alleles

99.2% missing data

2	(0.802) 0.802 0.802

3	(0.198) 0.198 0.198

Locus 8326 :

2 alleles

99.2% missing data

2	(0.308) 0.307 0.310

3	(0.692) 0.693 0.690

Locus 8327 :

2 alleles

0.0% missing data

2	(0.985) 0.981 0.995

3	(0.015) 0.019 0.005

Locus 8328 :

2 alleles

99.2% missing data

2	(0.448) 0.445 0.449

1	(0.552) 0.555 0.551

Locus 8329 :

3 alleles

98.8% missing data

2	(0.552) 0.557 0.544

4	(0.234) 0.228 0.235

3	(0.214) 0.215 0.221

Locus 8330 :

2 alleles

91.4% missing data

2	(0.891) 0.902 0.897

1	(0.109) 0.098 0.103

Locus 8331 :

2 alleles

98.4% missing data

3	(0.583) 0.590 0.584

2	(0.417) 0.410 0.416

Locus 8332 :

2 alleles

99.2% missing data

4	(0.392) 0.396 0.396

1	(0.608) 0.604 0.604

Locus 8333 :

2 alleles

96.9% missing data

4	(0.188) 0.179 0.189

2	(0.812) 0.821 0.811

Locus 8334 :

2 alleles

99.2% missing data

1	(0.232) 0.228 0.239

2	(0.768) 0.772 0.761

Locus 8335 :

2 alleles

99.2% missing data

3	(0.685) 0.685 0.683

2	(0.315) 0.315 0.317

Locus 8336 :

2 alleles

99.2% missing data

4	(0.417) 0.413 0.424

3	(0.583) 0.587 0.576

Locus 8337 :

2 alleles

99.2% missing data

4	(0.512) 0.507 0.520

2	(0.488) 0.493 0.480

Locus 8338 :

2 alleles

98.8% missing data

4	(0.507) 0.504 0.507

3	(0.493) 0.496 0.493

Locus 8339 :

2 alleles

99.2% missing data

3	(0.531) 0.529 0.527

4	(0.469) 0.471 0.473

Locus 8340 :

2 alleles

94.9% missing data

4	(0.891) 0.900 0.886

3	(0.109) 0.100 0.114

Locus 8341 :

2 alleles

99.2% missing data

2	(0.602) 0.609 0.599

1	(0.398) 0.391 0.401

Locus 8342 :

2 alleles

99.2% missing data

1	(0.536) 0.539 0.530

4	(0.464) 0.461 0.470

Locus 8343 :

2 alleles

98.4% missing data

3	(0.527) 0.528 0.531

2	(0.473) 0.472 0.469

Locus 8344 :

2 alleles

97.7% missing data

1	(0.771) 0.773 0.773

2	(0.229) 0.227 0.227

Locus 8345 :

3 alleles

98.8% missing data

1	(0.570) 0.561 0.575

4	(0.211) 0.209 0.215

3	(0.219) 0.230 0.210

Locus 8346 :

2 alleles

99.2% missing data

4	(0.340) 0.336 0.339

2	(0.660) 0.664 0.661

Locus 8347 :

2 alleles

99.2% missing data

3	(0.761) 0.760 0.767

1	(0.239) 0.240 0.233

Locus 8348 :

3 alleles

98.8% missing data

3	(0.242) 0.242 0.239

2	(0.416) 0.408 0.427

4	(0.341) 0.350 0.334

Locus 8349 :

3 alleles

98.8% missing data

1	(0.538) 0.529 0.543

3	(0.182) 0.180 0.185

2	(0.280) 0.291 0.272

Locus 8350 :

2 alleles

98.4% missing data

1	(0.591) 0.606 0.588

2	(0.409) 0.394 0.412

Locus 8351 :

2 alleles

99.2% missing data

1	(0.705) 0.706 0.700

3	(0.295) 0.294 0.300

Locus 8352 :

2 alleles

99.2% missing data

1	(0.519) 0.523 0.514

2	(0.481) 0.477 0.486

Locus 8353 :

2 alleles

99.2% missing data

1	(0.439) 0.439 0.439

4	(0.561) 0.561 0.561

Locus 8354 :

2 alleles

99.2% missing data

2	(0.417) 0.416 0.419

4	(0.583) 0.584 0.581

Locus 8355 :

2 alleles

99.2% missing data

4	(0.434) 0.431 0.435

1	(0.566) 0.569 0.565

Locus 8356 :

3 alleles

94.9% missing data

4	(0.796) 0.798 0.802

1	(0.105) 0.104 0.104

2	(0.100) 0.098 0.093

Locus 8357 :

2 alleles

99.2% missing data

3	(0.704) 0.707 0.700

4	(0.296) 0.293 0.300

Locus 8358 :

2 alleles

8.2% missing data

2	(0.982) 0.974 0.995

4	(0.018) 0.026 0.005

Locus 8359 :

2 alleles

99.2% missing data

3	(0.585) 0.589 0.586

4	(0.415) 0.411 0.414

Locus 8360 :

2 alleles

94.9% missing data

2	(0.652) 0.678 0.624

4	(0.348) 0.322 0.376

Locus 8361 :

3 alleles

96.1% missing data

1	(0.152) 0.142 0.159

2	(0.405) 0.396 0.402

3	(0.443) 0.462 0.439

Locus 8362 :

2 alleles

7.8% missing data

1	(0.973) 0.946 0.992

2	(0.027) 0.054 0.008

Locus 8363 :

2 alleles

99.2% missing data

3	(0.737) 0.735 0.735

4	(0.263) 0.265 0.265

Locus 8364 :

2 alleles

99.2% missing data

3	(0.696) 0.700 0.693

2	(0.304) 0.300 0.307

Locus 8365 :

2 alleles

99.2% missing data

4	(0.693) 0.696 0.691

2	(0.307) 0.304 0.309

Locus 8366 :

2 alleles

99.2% missing data

2	(0.493) 0.498 0.498

3	(0.507) 0.502 0.502

Locus 8367 :

2 alleles

98.4% missing data

4	(0.813) 0.817 0.809

1	(0.187) 0.183 0.191

Locus 8368 :

2 alleles

99.2% missing data

4	(0.516) 0.513 0.515

2	(0.484) 0.487 0.485

Locus 8369 :

2 alleles

8.6% missing data

2	(0.982) 0.974 0.994

4	(0.018) 0.026 0.006

Locus 8370 :

2 alleles

99.2% missing data

2	(0.528) 0.521 0.531

4	(0.472) 0.479 0.469

Locus 8371 :

2 alleles

97.7% missing data

4	(0.409) 0.400 0.417

3	(0.591) 0.600 0.583

Locus 8372 :

2 alleles

99.2% missing data

4	(0.491) 0.491 0.497

1	(0.509) 0.509 0.503

Locus 8373 :

2 alleles

98.4% missing data

3	(0.750) 0.746 0.750

2	(0.250) 0.254 0.250

Locus 8374 :

2 alleles

99.2% missing data

3	(0.552) 0.541 0.556

1	(0.448) 0.459 0.444

Locus 8375 :

2 alleles

99.2% missing data

1	(0.476) 0.482 0.470

4	(0.524) 0.518 0.530

Locus 8376 :

2 alleles

98.0% missing data

3	(0.326) 0.319 0.338

1	(0.674) 0.681 0.662

Locus 8377 :

2 alleles

99.2% missing data

3	(0.275) 0.269 0.285

4	(0.725) 0.731 0.715

Locus 8378 :

2 alleles

87.9% missing data

4	(0.668) 0.690 0.653

1	(0.332) 0.310 0.347

Locus 8379 :

2 alleles

99.2% missing data

2	(0.760) 0.764 0.754

1	(0.240) 0.236 0.246

Locus 8380 :

2 alleles

99.2% missing data

2	(0.510) 0.505 0.513

1	(0.490) 0.495 0.487

Locus 8381 :

2 alleles

97.3% missing data

2	(0.789) 0.784 0.803

4	(0.211) 0.216 0.197

Locus 8382 :

2 alleles

99.2% missing data

1	(0.320) 0.325 0.318

3	(0.680) 0.675 0.682

Locus 8383 :

2 alleles

99.2% missing data

1	(0.552) 0.548 0.559

4	(0.448) 0.452 0.441

Locus 8384 :

2 alleles

99.2% missing data

3	(0.406) 0.408 0.411

1	(0.594) 0.592 0.589

Locus 8385 :

2 alleles

98.8% missing data

3	(0.611) 0.613 0.608

4	(0.389) 0.387 0.392

Locus 8386 :

2 alleles

95.3% missing data

2	(0.914) 0.913 0.917

1	(0.086) 0.087 0.083

Locus 8387 :

2 alleles

99.2% missing data

4	(0.339) 0.334 0.344

2	(0.661) 0.666 0.656

Locus 8388 :

2 alleles

99.2% missing data

4	(0.764) 0.767 0.759

3	(0.236) 0.233 0.241

Locus 8389 :

2 alleles

99.2% missing data

3	(0.233) 0.238 0.228

2	(0.767) 0.762 0.772

Locus 8390 :

2 alleles

99.2% missing data

1	(0.808) 0.805 0.799

3	(0.192) 0.195 0.201

Locus 8391 :

2 alleles

99.2% missing data

1	(0.497) 0.496 0.496

3	(0.503) 0.504 0.504

Locus 8392 :

2 alleles

99.2% missing data

4	(0.295) 0.291 0.306

2	(0.705) 0.709 0.694

Locus 8393 :

2 alleles

99.2% missing data

2	(0.518) 0.516 0.523

1	(0.482) 0.484 0.477

Locus 8394 :

2 alleles

99.2% missing data

3	(0.166) 0.161 0.174

2	(0.834) 0.839 0.826

Locus 8395 :

2 alleles

96.1% missing data

4	(0.169) 0.158 0.174

3	(0.831) 0.842 0.826

Locus 8396 :

2 alleles

99.2% missing data

1	(0.455) 0.448 0.461

4	(0.545) 0.552 0.539

Locus 8397 :

2 alleles

99.2% missing data

4	(0.202) 0.196 0.208

1	(0.798) 0.804 0.792

Locus 8398 :

2 alleles

98.0% missing data

1	(0.811) 0.806 0.818

3	(0.189) 0.194 0.182

Locus 8399 :

3 alleles

95.7% missing data

1	(0.663) 0.658 0.686

3	(0.164) 0.163 0.155

2	(0.173) 0.179 0.158

Locus 8400 :

2 alleles

99.2% missing data

2	(0.429) 0.430 0.432

1	(0.571) 0.570 0.568

Locus 8401 :

2 alleles

96.5% missing data

4	(0.716) 0.732 0.715

1	(0.284) 0.268 0.285

Locus 8402 :

2 alleles

99.2% missing data

2	(0.726) 0.723 0.733

1	(0.274) 0.277 0.267

Locus 8403 :

2 alleles

98.4% missing data

4	(0.748) 0.747 0.757

3	(0.252) 0.253 0.243

Locus 8404 :

2 alleles

99.2% missing data

4	(0.396) 0.397 0.400

3	(0.604) 0.603 0.600

Locus 8405 :

2 alleles

99.2% missing data

1	(0.467) 0.464 0.468

3	(0.533) 0.536 0.532

Locus 8406 :

2 alleles

98.8% missing data

4	(0.677) 0.673 0.688

3	(0.323) 0.327 0.312

Locus 8407 :

2 alleles

99.2% missing data

4	(0.201) 0.204 0.202

1	(0.799) 0.796 0.798

Locus 8408 :

2 alleles

97.3% missing data

2	(0.746) 0.747 0.753

4	(0.254) 0.253 0.247

Locus 8409 :

2 alleles

99.2% missing data

4	(0.274) 0.273 0.273

2	(0.726) 0.727 0.727

Locus 8410 :

2 alleles

99.2% missing data

1	(0.482) 0.483 0.479

4	(0.518) 0.517 0.521

Locus 8411 :

2 alleles

99.2% missing data

4	(0.555) 0.554 0.551

1	(0.445) 0.446 0.449

Locus 8412 :

2 alleles

97.3% missing data

1	(0.451) 0.437 0.456

3	(0.549) 0.563 0.544

Locus 8413 :

3 alleles

98.0% missing data

3	(0.457) 0.459 0.451

4	(0.321) 0.323 0.325

2	(0.222) 0.218 0.224

Locus 8414 :

2 alleles

99.2% missing data

4	(0.620) 0.620 0.616

3	(0.380) 0.380 0.384

Locus 8415 :

2 alleles

99.2% missing data

4	(0.246) 0.247 0.251

2	(0.754) 0.753 0.749

Locus 8416 :

2 alleles

96.1% missing data

3	(0.832) 0.833 0.835

2	(0.168) 0.167 0.165

Locus 8417 :

2 alleles

98.8% missing data

4	(0.844) 0.842 0.839

2	(0.156) 0.158 0.161

Locus 8418 :

2 alleles

99.2% missing data

3	(0.220) 0.225 0.219

2	(0.780) 0.775 0.781

Locus 8419 :

2 alleles

90.6% missing data

1	(0.899) 0.916 0.883

4	(0.101) 0.084 0.117

Locus 8420 :

2 alleles

91.0% missing data

4	(0.881) 0.902 0.872

1	(0.119) 0.098 0.128

Locus 8421 :

2 alleles

98.8% missing data

4	(0.507) 0.502 0.516

3	(0.493) 0.498 0.484

Locus 8422 :

2 alleles

99.2% missing data

2	(0.627) 0.628 0.629

1	(0.373) 0.372 0.371

Locus 8423 :

2 alleles

98.4% missing data

3	(0.799) 0.799 0.797

4	(0.201) 0.201 0.203

Locus 8424 :

2 alleles

0.0% missing data

1	(0.989) 0.991 0.993

4	(0.011) 0.009 0.007

Locus 8425 :

2 alleles

99.2% missing data

3	(0.366) 0.361 0.365

1	(0.634) 0.639 0.635

Locus 8426 :

2 alleles

99.2% missing data

4	(0.345) 0.343 0.346

3	(0.655) 0.657 0.654

Locus 8427 :

2 alleles

99.2% missing data

1	(0.335) 0.336 0.338

3	(0.665) 0.664 0.662

Locus 8428 :

2 alleles

99.2% missing data

4	(0.522) 0.513 0.531

2	(0.478) 0.487 0.469

Locus 8429 :

2 alleles

99.2% missing data

1	(0.526) 0.530 0.522

4	(0.474) 0.470 0.478

Locus 8430 :

2 alleles

98.4% missing data

2	(0.730) 0.729 0.739

3	(0.270) 0.271 0.261

Locus 8431 :

2 alleles

99.2% missing data

2	(0.681) 0.686 0.674

3	(0.319) 0.314 0.326

Locus 8432 :

2 alleles

99.2% missing data

4	(0.398) 0.391 0.403

3	(0.602) 0.609 0.597

Locus 8433 :

2 alleles

91.0% missing data

4	(0.906) 0.922 0.898

2	(0.094) 0.078 0.102

Locus 8434 :

2 alleles

95.3% missing data

1	(0.146) 0.152 0.135

4	(0.854) 0.848 0.865

Locus 8435 :

2 alleles

99.2% missing data

4	(0.416) 0.406 0.421

3	(0.584) 0.594 0.579

Locus 8436 :

2 alleles

99.2% missing data

1	(0.568) 0.567 0.566

4	(0.432) 0.433 0.434

Locus 8437 :

2 alleles

99.2% missing data

4	(0.461) 0.454 0.465

1	(0.539) 0.546 0.535

Locus 8438 :

2 alleles

93.8% missing data

3	(0.123) 0.132 0.105

4	(0.877) 0.868 0.895

Locus 8439 :

2 alleles

99.2% missing data

2	(0.295) 0.299 0.293

3	(0.705) 0.701 0.707

Locus 8440 :

2 alleles

91.0% missing data

2	(0.924) 0.934 0.917

1	(0.076) 0.066 0.083

Locus 8441 :

2 alleles

99.2% missing data

2	(0.260) 0.262 0.255

3	(0.740) 0.738 0.745

Locus 8442 :

2 alleles

99.2% missing data

3	(0.454) 0.456 0.449

1	(0.546) 0.544 0.551

Locus 8443 :

2 alleles

99.2% missing data

3	(0.487) 0.480 0.493

1	(0.513) 0.520 0.507

Locus 8444 :

3 alleles

98.8% missing data

4	(0.199) 0.199 0.203

2	(0.421) 0.417 0.420

3	(0.379) 0.384 0.377

Locus 8445 :

2 alleles

99.2% missing data

1	(0.550) 0.541 0.553

3	(0.450) 0.459 0.447

Locus 8446 :

2 alleles

99.2% missing data

1	(0.708) 0.700 0.710

3	(0.292) 0.300 0.290

Locus 8447 :

2 alleles

99.2% missing data

4	(0.256) 0.250 0.260

1	(0.744) 0.750 0.740

Locus 8448 :

2 alleles

96.9% missing data

1	(0.216) 0.214 0.208

3	(0.784) 0.786 0.792

Locus 8449 :

2 alleles

98.8% missing data

2	(0.326) 0.323 0.323

1	(0.674) 0.677 0.677

Locus 8450 :

2 alleles

98.8% missing data

3	(0.453) 0.453 0.460

4	(0.547) 0.547 0.540

Locus 8451 :

2 alleles

99.2% missing data

3	(0.604) 0.597 0.609

1	(0.396) 0.403 0.391

Locus 8452 :

2 alleles

99.2% missing data

4	(0.728) 0.733 0.727

3	(0.272) 0.267 0.273

Locus 8453 :

2 alleles

98.8% missing data

3	(0.516) 0.515 0.509

2	(0.484) 0.485 0.491

Locus 8454 :

2 alleles

99.2% missing data

1	(0.774) 0.784 0.769

2	(0.226) 0.216 0.231

Locus 8455 :

2 alleles

93.0% missing data

1	(0.884) 0.903 0.871

3	(0.116) 0.097 0.129

Locus 8456 :

2 alleles

99.2% missing data

1	(0.448) 0.452 0.448

3	(0.552) 0.548 0.552

Locus 8457 :

2 alleles

91.0% missing data

2	(0.900) 0.917 0.894

4	(0.100) 0.083 0.106

Locus 8458 :

2 alleles

99.2% missing data

1	(0.286) 0.280 0.298

3	(0.714) 0.720 0.702

Locus 8459 :

2 alleles

99.2% missing data

4	(0.239) 0.234 0.243

3	(0.761) 0.766 0.757

Locus 8460 :

2 alleles

99.2% missing data

3	(0.521) 0.521 0.525

2	(0.479) 0.479 0.475

Locus 8461 :

2 alleles

98.8% missing data

3	(0.631) 0.637 0.631

2	(0.369) 0.363 0.369

Locus 8462 :

2 alleles

69.1% missing data

1	(0.970) 0.960 0.984

3	(0.030) 0.040 0.016

Locus 8463 :

2 alleles

97.7% missing data

3	(0.293) 0.284 0.300

1	(0.707) 0.716 0.700

Locus 8464 :

2 alleles

99.2% missing data

3	(0.588) 0.598 0.583

4	(0.412) 0.402 0.417

Locus 8465 :

2 alleles

99.2% missing data

1	(0.566) 0.573 0.565

2	(0.434) 0.427 0.435

Locus 8466 :

2 alleles

98.8% missing data

1	(0.817) 0.822 0.813

2	(0.183) 0.178 0.187

Locus 8467 :

2 alleles

99.2% missing data

1	(0.686) 0.687 0.679

3	(0.314) 0.313 0.321

Locus 8468 :

2 alleles

99.2% missing data

3	(0.360) 0.353 0.370

2	(0.640) 0.647 0.630

Locus 8469 :

2 alleles

99.2% missing data

3	(0.245) 0.241 0.248

2	(0.755) 0.759 0.752

Locus 8470 :

2 alleles

98.8% missing data

1	(0.621) 0.620 0.623

4	(0.379) 0.380 0.377

Locus 8471 :

2 alleles

96.1% missing data

3	(0.167) 0.163 0.165

1	(0.833) 0.837 0.835

Locus 8472 :

2 alleles

99.2% missing data

3	(0.297) 0.291 0.306

2	(0.703) 0.709 0.694

Locus 8473 :

2 alleles

98.0% missing data

3	(0.132) 0.129 0.134

2	(0.868) 0.871 0.866

Locus 8474 :

2 alleles

99.2% missing data

4	(0.616) 0.611 0.623

1	(0.384) 0.389 0.377

Locus 8475 :

2 alleles

98.8% missing data

3	(0.399) 0.403 0.392

2	(0.601) 0.597 0.608

Locus 8476 :

2 alleles

97.7% missing data

3	(0.178) 0.170 0.184

2	(0.822) 0.830 0.816

Locus 8477 :

2 alleles

96.1% missing data

1	(0.172) 0.160 0.177

3	(0.828) 0.840 0.823

Locus 8478 :

2 alleles

99.2% missing data

2	(0.615) 0.608 0.619

1	(0.385) 0.392 0.381

Locus 8479 :

2 alleles

99.2% missing data

3	(0.692) 0.697 0.685

4	(0.308) 0.303 0.315

Locus 8480 :

2 alleles

91.4% missing data

1	(0.930) 0.944 0.922

3	(0.070) 0.056 0.078

Locus 8481 :

3 alleles

98.8% missing data

4	(0.539) 0.532 0.540

3	(0.282) 0.283 0.281

1	(0.179) 0.185 0.179

Locus 8482 :

2 alleles

99.2% missing data

1	(0.459) 0.457 0.461

4	(0.541) 0.543 0.539

Locus 8483 :

2 alleles

99.2% missing data

2	(0.296) 0.285 0.303

1	(0.704) 0.715 0.697

Locus 8484 :

2 alleles

98.4% missing data

3	(0.241) 0.242 0.237

1	(0.759) 0.758 0.763

Locus 8485 :

2 alleles

99.2% missing data

1	(0.727) 0.722 0.726

2	(0.273) 0.278 0.274

Locus 8486 :

3 alleles

98.8% missing data

2	(0.329) 0.330 0.327

4	(0.499) 0.501 0.497

1	(0.172) 0.169 0.176

Locus 8487 :

2 alleles

8.6% missing data

3	(0.981) 0.972 0.994

1	(0.019) 0.028 0.006

Locus 8488 :

2 alleles

99.2% missing data

3	(0.510) 0.512 0.509

2	(0.490) 0.488 0.491

Locus 8489 :

2 alleles

99.2% missing data

1	(0.448) 0.449 0.444

2	(0.552) 0.551 0.556

Locus 8490 :

2 alleles

99.2% missing data

2	(0.730) 0.727 0.734

1	(0.270) 0.273 0.266

Locus 8491 :

2 alleles

92.6% missing data

3	(0.840) 0.840 0.854

1	(0.160) 0.160 0.146

Locus 8492 :

2 alleles

93.4% missing data

2	(0.857) 0.849 0.879

1	(0.143) 0.151 0.121

Locus 8493 :

4 alleles

98.0% missing data

4	(0.406) 0.406 0.403

3	(0.105) 0.102 0.112

1	(0.327) 0.330 0.321

2	(0.162) 0.162 0.164

Locus 8494 :

2 alleles

96.5% missing data

1	(0.851) 0.856 0.853

2	(0.149) 0.144 0.147

Locus 8495 :

2 alleles

99.2% missing data

1	(0.423) 0.422 0.428

3	(0.577) 0.578 0.572

Locus 8496 :

2 alleles

98.8% missing data

2	(0.342) 0.344 0.341

4	(0.658) 0.656 0.659

Locus 8497 :

2 alleles

97.7% missing data

1	(0.868) 0.866 0.871

3	(0.132) 0.134 0.129

Locus 8498 :

2 alleles

98.8% missing data

2	(0.499) 0.492 0.510

4	(0.501) 0.508 0.490

Locus 8499 :

2 alleles

99.2% missing data

1	(0.218) 0.214 0.214

3	(0.782) 0.786 0.786

Locus 8500 :

2 alleles

96.1% missing data

1	(0.234) 0.225 0.232

2	(0.766) 0.775 0.768

Locus 8501 :

2 alleles

98.0% missing data

2	(0.715) 0.708 0.727

1	(0.285) 0.292 0.273

Locus 8502 :

2 alleles

0.4% missing data

3	(0.987) 0.982 0.996

1	(0.013) 0.018 0.004

Locus 8503 :

2 alleles

97.3% missing data

4	(0.750) 0.761 0.747

1	(0.250) 0.239 0.253

Locus 8504 :

2 alleles

99.2% missing data

1	(0.619) 0.617 0.617

4	(0.381) 0.383 0.383

Locus 8505 :

2 alleles

98.0% missing data

3	(0.690) 0.700 0.681

1	(0.310) 0.300 0.319

Locus 8506 :

2 alleles

98.8% missing data

1	(0.389) 0.390 0.392

3	(0.611) 0.610 0.608

Locus 8507 :

2 alleles

92.6% missing data

3	(0.884) 0.873 0.903

2	(0.116) 0.127 0.097

Locus 8508 :

2 alleles

99.2% missing data

4	(0.369) 0.365 0.371

1	(0.631) 0.635 0.629

Locus 8509 :

2 alleles

98.8% missing data

2	(0.461) 0.462 0.467

3	(0.539) 0.538 0.533

Locus 8510 :

2 alleles

98.4% missing data

3	(0.257) 0.251 0.259

1	(0.743) 0.749 0.741

Locus 8511 :

2 alleles

99.2% missing data

2	(0.309) 0.312 0.308

1	(0.691) 0.688 0.692

Locus 8512 :

2 alleles

99.2% missing data

1	(0.683) 0.686 0.684

3	(0.317) 0.314 0.316

Locus 8513 :

2 alleles

90.2% missing data

1	(0.902) 0.923 0.891

3	(0.098) 0.077 0.109

Locus 8514 :

2 alleles

0.0% missing data

3	(0.990) 0.992 0.994

2	(0.010) 0.008 0.006

Locus 8515 :

2 alleles

99.2% missing data

4	(0.499) 0.499 0.502

1	(0.501) 0.501 0.498

Locus 8516 :

2 alleles

99.2% missing data

1	(0.337) 0.341 0.338

4	(0.663) 0.659 0.662

Locus 8517 :

2 alleles

99.2% missing data

4	(0.415) 0.405 0.416

2	(0.585) 0.595 0.584

Locus 8518 :

2 alleles

90.6% missing data

2	(0.907) 0.918 0.902

1	(0.093) 0.082 0.098

Locus 8519 :

2 alleles

99.2% missing data

2	(0.402) 0.401 0.399

1	(0.598) 0.599 0.601

Locus 8520 :

2 alleles

98.4% missing data

2	(0.658) 0.663 0.658

3	(0.342) 0.337 0.342

Locus 8521 :

2 alleles

98.8% missing data

1	(0.778) 0.772 0.779

3	(0.222) 0.228 0.221

Locus 8522 :

2 alleles

90.6% missing data

1	(0.900) 0.919 0.887

2	(0.100) 0.081 0.113

Locus 8523 :

2 alleles

93.0% missing data

4	(0.761) 0.765 0.755

1	(0.239) 0.235 0.245

Locus 8524 :

2 alleles

99.2% missing data

3	(0.703) 0.698 0.706

1	(0.297) 0.302 0.294

Locus 8525 :

2 alleles

99.2% missing data

3	(0.676) 0.671 0.680

4	(0.324) 0.329 0.320

Locus 8526 :

2 alleles

98.8% missing data

2	(0.613) 0.616 0.616

3	(0.387) 0.384 0.384

Locus 8527 :

2 alleles

97.3% missing data

4	(0.288) 0.291 0.280

1	(0.712) 0.709 0.720

Locus 8528 :

2 alleles

99.2% missing data

4	(0.570) 0.573 0.570

3	(0.430) 0.427 0.430

Locus 8529 :

2 alleles

94.9% missing data

3	(0.748) 0.735 0.773

2	(0.252) 0.265 0.227

Locus 8530 :

2 alleles

96.5% missing data

1	(0.147) 0.136 0.153

3	(0.853) 0.864 0.847

Locus 8531 :

2 alleles

99.2% missing data

1	(0.292) 0.295 0.294

4	(0.708) 0.705 0.706

Locus 8532 :

3 alleles

98.0% missing data

2	(0.536) 0.543 0.531

4	(0.203) 0.195 0.213

3	(0.261) 0.262 0.256

Locus 8533 :

2 alleles

99.2% missing data

1	(0.482) 0.482 0.478

2	(0.518) 0.518 0.522

Locus 8534 :

2 alleles

98.0% missing data

4	(0.685) 0.688 0.689

3	(0.315) 0.312 0.311

Locus 8535 :

2 alleles

96.9% missing data

3	(0.786) 0.798 0.785

2	(0.214) 0.202 0.215

Locus 8536 :

2 alleles

99.2% missing data

3	(0.319) 0.321 0.320

1	(0.681) 0.679 0.680

Locus 8537 :

2 alleles

99.2% missing data

4	(0.739) 0.744 0.739

1	(0.261) 0.256 0.261

Locus 8538 :

2 alleles

99.2% missing data

1	(0.336) 0.338 0.330

2	(0.664) 0.662 0.670

Locus 8539 :

2 alleles

99.2% missing data

2	(0.441) 0.442 0.444

3	(0.559) 0.558 0.556

Locus 8540 :

2 alleles

99.2% missing data

2	(0.357) 0.356 0.359

3	(0.643) 0.644 0.641

Locus 8541 :

3 alleles

98.4% missing data

2	(0.442) 0.436 0.434

1	(0.232) 0.228 0.247

4	(0.326) 0.336 0.318

Locus 8542 :

2 alleles

99.2% missing data

2	(0.642) 0.640 0.646

1	(0.358) 0.360 0.354

Locus 8543 :

3 alleles

98.4% missing data

3	(0.425) 0.416 0.429

4	(0.254) 0.267 0.253

2	(0.321) 0.317 0.318

Locus 8544 :

2 alleles

99.2% missing data

2	(0.597) 0.595 0.600

1	(0.403) 0.405 0.400

Locus 8545 :

2 alleles

96.1% missing data

3	(0.802) 0.814 0.799

2	(0.198) 0.186 0.201

Locus 8546 :

2 alleles

99.2% missing data

2	(0.421) 0.421 0.423

1	(0.579) 0.579 0.577

Locus 8547 :

2 alleles

97.7% missing data

4	(0.286) 0.283 0.277

3	(0.714) 0.717 0.723

Locus 8548 :

2 alleles

99.2% missing data

1	(0.257) 0.250 0.260

3	(0.743) 0.750 0.740

Locus 8549 :

2 alleles

98.0% missing data

1	(0.883) 0.890 0.877

3	(0.117) 0.110 0.123

Locus 8550 :

2 alleles

98.4% missing data

1	(0.457) 0.455 0.454

3	(0.543) 0.545 0.546

Locus 8551 :

2 alleles

99.2% missing data

3	(0.511) 0.513 0.513

1	(0.489) 0.487 0.487

Locus 8552 :

2 alleles

94.5% missing data

2	(0.739) 0.762 0.732

1	(0.261) 0.238 0.268

Locus 8553 :

2 alleles

98.8% missing data

3	(0.331) 0.325 0.343

1	(0.669) 0.675 0.657

Locus 8554 :

2 alleles

99.2% missing data

3	(0.285) 0.277 0.292

1	(0.715) 0.723 0.708

Locus 8555 :

2 alleles

99.2% missing data

4	(0.458) 0.456 0.462

1	(0.542) 0.544 0.538

Locus 8556 :

3 alleles

98.0% missing data

4	(0.338) 0.353 0.328

3	(0.302) 0.294 0.302

2	(0.361) 0.353 0.370

Locus 8557 :

2 alleles

99.2% missing data

3	(0.463) 0.464 0.464

2	(0.537) 0.536 0.536

Locus 8558 :

2 alleles

98.8% missing data

3	(0.623) 0.627 0.619

1	(0.377) 0.373 0.381

Locus 8559 :

2 alleles

99.2% missing data

4	(0.599) 0.604 0.597

3	(0.401) 0.396 0.403

Locus 8560 :

2 alleles

28.5% missing data

2	(0.980) 0.982 0.990

3	(0.020) 0.018 0.010

Locus 8561 :

2 alleles

99.2% missing data

3	(0.400) 0.399 0.397

4	(0.600) 0.601 0.603

Locus 8562 :

2 alleles

99.2% missing data

1	(0.393) 0.402 0.385

4	(0.607) 0.598 0.615

Locus 8563 :

2 alleles

96.9% missing data

1	(0.197) 0.182 0.204

4	(0.803) 0.818 0.796

Locus 8564 :

2 alleles

98.8% missing data

3	(0.623) 0.622 0.627

1	(0.377) 0.378 0.373

Locus 8565 :

2 alleles

99.2% missing data

3	(0.296) 0.295 0.302

1	(0.704) 0.705 0.698

Locus 8566 :

2 alleles

98.4% missing data

3	(0.747) 0.748 0.747

1	(0.253) 0.252 0.253

Locus 8567 :

2 alleles

94.9% missing data

3	(0.686) 0.662 0.716

1	(0.314) 0.338 0.284

Locus 8568 :

2 alleles

99.2% missing data

4	(0.435) 0.438 0.435

2	(0.565) 0.562 0.565

Locus 8569 :

2 alleles

98.8% missing data

4	(0.683) 0.682 0.683

1	(0.317) 0.318 0.317

Locus 8570 :

2 alleles

99.2% missing data

1	(0.650) 0.652 0.650

2	(0.350) 0.348 0.350

Locus 8571 :

2 alleles

99.2% missing data

4	(0.399) 0.396 0.404

2	(0.601) 0.604 0.596

Locus 8572 :

2 alleles

99.2% missing data

2	(0.626) 0.626 0.633

1	(0.374) 0.374 0.367

Locus 8573 :

2 alleles

98.8% missing data

1	(0.631) 0.623 0.636

4	(0.369) 0.377 0.364

Locus 8574 :

2 alleles

98.8% missing data

3	(0.283) 0.281 0.282

1	(0.717) 0.719 0.718

Locus 8575 :

2 alleles

98.4% missing data

3	(0.332) 0.328 0.335

2	(0.668) 0.672 0.665

Locus 8576 :

2 alleles

97.7% missing data

2	(0.746) 0.738 0.755

4	(0.254) 0.262 0.245

Locus 8577 :

2 alleles

98.8% missing data

4	(0.636) 0.639 0.635

2	(0.364) 0.361 0.365

Locus 8578 :

2 alleles

98.4% missing data

4	(0.501) 0.517 0.497

3	(0.499) 0.483 0.503

Locus 8579 :

2 alleles

99.2% missing data

1	(0.566) 0.568 0.569

3	(0.434) 0.432 0.431

Locus 8580 :

2 alleles

99.2% missing data

4	(0.371) 0.362 0.375

3	(0.629) 0.638 0.625

Locus 8581 :

2 alleles

99.2% missing data

2	(0.372) 0.365 0.375

3	(0.628) 0.635 0.625

Locus 8582 :

2 alleles

99.2% missing data

1	(0.293) 0.297 0.288

4	(0.707) 0.703 0.712

Locus 8583 :

2 alleles

99.2% missing data

1	(0.354) 0.359 0.355

3	(0.646) 0.641 0.645

Locus 8584 :

2 alleles

96.1% missing data

2	(0.224) 0.209 0.227

3	(0.776) 0.791 0.773

Locus 8585 :

2 alleles

99.2% missing data

1	(0.609) 0.610 0.615

3	(0.391) 0.390 0.385

Locus 8586 :

2 alleles

96.1% missing data

1	(0.755) 0.762 0.760

3	(0.245) 0.238 0.240

Locus 8587 :

2 alleles

98.8% missing data

1	(0.466) 0.467 0.466

4	(0.534) 0.533 0.534

Locus 8588 :

2 alleles

99.2% missing data

1	(0.461) 0.465 0.459

4	(0.539) 0.535 0.541

Locus 8589 :

2 alleles

99.2% missing data

3	(0.705) 0.713 0.698

1	(0.295) 0.287 0.302

Locus 8590 :

2 alleles

99.2% missing data

1	(0.727) 0.728 0.725

3	(0.273) 0.272 0.275

Locus 8591 :

2 alleles

93.8% missing data

4	(0.913) 0.907 0.924

1	(0.087) 0.093 0.076

Locus 8592 :

2 alleles

99.2% missing data

2	(0.493) 0.493 0.494

3	(0.507) 0.507 0.506

Locus 8593 :

2 alleles

98.8% missing data

3	(0.542) 0.537 0.540

2	(0.458) 0.463 0.460

Locus 8594 :

2 alleles

99.2% missing data

1	(0.597) 0.599 0.597

2	(0.403) 0.401 0.403

Locus 8595 :

2 alleles

99.2% missing data

1	(0.414) 0.411 0.418

2	(0.586) 0.589 0.582

Locus 8596 :

2 alleles

99.2% missing data

2	(0.485) 0.493 0.478

4	(0.515) 0.507 0.522

Locus 8597 :

2 alleles

98.8% missing data

1	(0.658) 0.656 0.660

2	(0.342) 0.344 0.340

Locus 8598 :

2 alleles

99.2% missing data

3	(0.406) 0.410 0.404

1	(0.594) 0.590 0.596

Locus 8599 :

2 alleles

99.2% missing data

4	(0.649) 0.651 0.655

3	(0.351) 0.349 0.345

Locus 8600 :

2 alleles

99.2% missing data

2	(0.640) 0.641 0.639

1	(0.360) 0.359 0.361

Locus 8601 :

2 alleles

99.2% missing data

1	(0.266) 0.271 0.263

4	(0.734) 0.729 0.737

Locus 8602 :

2 alleles

97.3% missing data

4	(0.844) 0.846 0.846

1	(0.156) 0.154 0.154

Locus 8603 :

2 alleles

99.2% missing data

1	(0.451) 0.450 0.451

4	(0.549) 0.550 0.549

Locus 8604 :

2 alleles

99.2% missing data

4	(0.706) 0.711 0.702

1	(0.294) 0.289 0.298

Locus 8605 :

2 alleles

97.7% missing data

2	(0.729) 0.723 0.740

1	(0.271) 0.277 0.260

Locus 8606 :

2 alleles

99.2% missing data

2	(0.404) 0.407 0.408

1	(0.596) 0.593 0.592

Locus 8607 :

2 alleles

99.2% missing data

2	(0.495) 0.500 0.487

4	(0.505) 0.500 0.513

Locus 8608 :

2 alleles

99.2% missing data

3	(0.436) 0.437 0.438

4	(0.564) 0.563 0.562

Locus 8609 :

2 alleles

99.2% missing data

2	(0.640) 0.642 0.638

4	(0.360) 0.358 0.362

Locus 8610 :

2 alleles

99.2% missing data

2	(0.543) 0.544 0.545

3	(0.457) 0.456 0.455

Locus 8611 :

2 alleles

98.8% missing data

3	(0.773) 0.773 0.776

4	(0.227) 0.227 0.224

Locus 8612 :

2 alleles

99.2% missing data

1	(0.513) 0.512 0.518

3	(0.487) 0.488 0.482

Locus 8613 :

2 alleles

99.2% missing data

3	(0.567) 0.570 0.572

4	(0.433) 0.430 0.428

Locus 8614 :

2 alleles

98.8% missing data

3	(0.761) 0.755 0.765

2	(0.239) 0.245 0.235

Locus 8615 :

2 alleles

99.2% missing data

4	(0.244) 0.239 0.247

1	(0.756) 0.761 0.753

Locus 8616 :

2 alleles

98.8% missing data

1	(0.379) 0.383 0.374

2	(0.621) 0.617 0.626

Locus 8617 :

2 alleles

97.7% missing data

1	(0.746) 0.753 0.746

2	(0.254) 0.247 0.254

Locus 8618 :

2 alleles

98.8% missing data

2	(0.687) 0.675 0.689

4	(0.313) 0.325 0.311

Locus 8619 :

2 alleles

90.6% missing data

4	(0.919) 0.934 0.910

3	(0.081) 0.066 0.090

Locus 8620 :

2 alleles

99.2% missing data

2	(0.486) 0.486 0.483

3	(0.514) 0.514 0.517

Locus 8621 :

2 alleles

99.2% missing data

3	(0.540) 0.531 0.548

1	(0.460) 0.469 0.452

Locus 8622 :

2 alleles

98.8% missing data

1	(0.716) 0.714 0.719

4	(0.284) 0.286 0.281

Locus 8623 :

2 alleles

99.2% missing data

4	(0.424) 0.414 0.430

1	(0.576) 0.586 0.570

Locus 8624 :

2 alleles

94.5% missing data

1	(0.913) 0.907 0.923

4	(0.087) 0.093 0.077

Locus 8625 :

2 alleles

97.3% missing data

4	(0.825) 0.829 0.827

1	(0.175) 0.171 0.173

Locus 8626 :

2 alleles

7.8% missing data

1	(0.984) 0.976 0.995

4	(0.016) 0.024 0.005

Locus 8627 :

2 alleles

93.0% missing data

4	(0.877) 0.872 0.895

3	(0.123) 0.128 0.105

Locus 8628 :

2 alleles

99.2% missing data

3	(0.295) 0.300 0.292

4	(0.705) 0.700 0.708

Locus 8629 :

2 alleles

99.2% missing data

4	(0.627) 0.634 0.623

2	(0.373) 0.366 0.377

Locus 8630 :

2 alleles

7.8% missing data

2	(0.975) 0.943 0.992

3	(0.025) 0.057 0.008

Locus 8631 :

2 alleles

48.0% missing data

3	(0.933) 0.917 0.954

2	(0.067) 0.083 0.046

Locus 8632 :

2 alleles

99.2% missing data

3	(0.530) 0.523 0.531

4	(0.470) 0.477 0.469

Locus 8633 :

2 alleles

99.2% missing data

3	(0.228) 0.227 0.228

4	(0.772) 0.773 0.772

Locus 8634 :

2 alleles

99.2% missing data

2	(0.684) 0.683 0.681

4	(0.316) 0.317 0.319

Locus 8635 :

2 alleles

98.0% missing data

4	(0.829) 0.824 0.838

1	(0.171) 0.176 0.162

Locus 8636 :

2 alleles

99.2% missing data

4	(0.205) 0.200 0.212

1	(0.795) 0.800 0.788

Locus 8637 :

3 alleles

98.8% missing data

1	(0.416) 0.417 0.412

3	(0.160) 0.160 0.165

4	(0.424) 0.422 0.424

Locus 8638 :

2 alleles

91.0% missing data

3	(0.904) 0.922 0.895

1	(0.096) 0.078 0.105

Locus 8639 :

2 alleles

99.2% missing data

2	(0.209) 0.208 0.214

4	(0.791) 0.792 0.786

Locus 8640 :

2 alleles

99.2% missing data

2	(0.530) 0.524 0.537

4	(0.470) 0.476 0.463

Locus 8641 :

2 alleles

98.0% missing data

2	(0.209) 0.204 0.211

1	(0.791) 0.796 0.789

Locus 8642 :

2 alleles

99.2% missing data

2	(0.552) 0.554 0.550

3	(0.448) 0.446 0.450

Locus 8643 :

2 alleles

96.9% missing data

3	(0.703) 0.695 0.722

2	(0.297) 0.305 0.278

Locus 8644 :

2 alleles

98.8% missing data

1	(0.601) 0.602 0.605

4	(0.399) 0.398 0.395

Locus 8645 :

2 alleles

98.8% missing data

3	(0.320) 0.320 0.328

1	(0.680) 0.680 0.672

Locus 8646 :

2 alleles

97.7% missing data

3	(0.287) 0.283 0.281

1	(0.713) 0.717 0.719

Locus 8647 :

2 alleles

99.2% missing data

3	(0.441) 0.447 0.434

2	(0.559) 0.553 0.566

Locus 8648 :

2 alleles

93.0% missing data

1	(0.826) 0.821 0.852

2	(0.174) 0.179 0.148

Locus 8649 :

2 alleles

99.2% missing data

3	(0.478) 0.476 0.475

2	(0.522) 0.524 0.525

Locus 8650 :

2 alleles

99.2% missing data

4	(0.629) 0.622 0.630

2	(0.371) 0.378 0.370

Locus 8651 :

2 alleles

98.8% missing data

4	(0.662) 0.658 0.670

1	(0.338) 0.342 0.330

Locus 8652 :

2 alleles

99.2% missing data

2	(0.573) 0.569 0.572

3	(0.427) 0.431 0.428

Locus 8653 :

2 alleles

99.2% missing data

2	(0.753) 0.747 0.753

1	(0.247) 0.253 0.247

Locus 8654 :

2 alleles

99.2% missing data

4	(0.640) 0.638 0.645

1	(0.360) 0.362 0.355

Locus 8655 :

2 alleles

99.2% missing data

3	(0.471) 0.465 0.477

1	(0.529) 0.535 0.523

Locus 8656 :

2 alleles

99.2% missing data

3	(0.550) 0.551 0.551

2	(0.450) 0.449 0.449

Locus 8657 :

2 alleles

9.0% missing data

2	(0.984) 0.977 0.995

3	(0.016) 0.023 0.005

Locus 8658 :

3 alleles

98.8% missing data

2	(0.196) 0.202 0.194

4	(0.373) 0.364 0.375

1	(0.432) 0.434 0.431

Locus 8659 :

2 alleles

99.2% missing data

3	(0.348) 0.342 0.354

4	(0.652) 0.658 0.646

Locus 8660 :

2 alleles

99.2% missing data

1	(0.677) 0.678 0.675

3	(0.323) 0.322 0.325

Locus 8661 :

2 alleles

99.2% missing data

3	(0.344) 0.343 0.344

4	(0.656) 0.657 0.656

Locus 8662 :

2 alleles

98.4% missing data

1	(0.617) 0.624 0.621

4	(0.383) 0.376 0.379

Locus 8663 :

2 alleles

98.8% missing data

2	(0.433) 0.436 0.432

4	(0.567) 0.564 0.568

Locus 8664 :

2 alleles

99.2% missing data

4	(0.189) 0.196 0.192

3	(0.811) 0.804 0.808

Locus 8665 :

2 alleles

99.2% missing data

1	(0.380) 0.376 0.387

4	(0.620) 0.624 0.613

Locus 8666 :

2 alleles

99.2% missing data

1	(0.522) 0.518 0.527

3	(0.478) 0.482 0.473

Locus 8667 :

2 alleles

98.4% missing data

1	(0.500) 0.503 0.502

4	(0.500) 0.497 0.498

Locus 8668 :

2 alleles

99.2% missing data

3	(0.755) 0.750 0.760

4	(0.245) 0.250 0.240

Locus 8669 :

2 alleles

99.2% missing data

3	(0.334) 0.341 0.330

1	(0.666) 0.659 0.670

Locus 8670 :

2 alleles

98.8% missing data

4	(0.755) 0.754 0.752

2	(0.245) 0.246 0.248

Locus 8671 :

2 alleles

97.3% missing data

4	(0.372) 0.369 0.363

2	(0.628) 0.631 0.637

Locus 8672 :

2 alleles

99.2% missing data

4	(0.284) 0.289 0.281

3	(0.716) 0.711 0.719

Locus 8673 :

2 alleles

99.2% missing data

4	(0.326) 0.327 0.323

2	(0.674) 0.673 0.677

Locus 8674 :

2 alleles

99.2% missing data

4	(0.690) 0.689 0.684

2	(0.310) 0.311 0.316

Locus 8675 :

2 alleles

99.2% missing data

2	(0.119) 0.124 0.117

4	(0.881) 0.876 0.883

Locus 8676 :

2 alleles

99.2% missing data

4	(0.515) 0.516 0.516

1	(0.485) 0.484 0.484

Locus 8677 :

2 alleles

99.2% missing data

4	(0.450) 0.455 0.449

3	(0.550) 0.545 0.551

Locus 8678 :

2 alleles

99.2% missing data

4	(0.758) 0.754 0.760

1	(0.242) 0.246 0.240

Locus 8679 :

2 alleles

98.8% missing data

1	(0.678) 0.681 0.674

3	(0.322) 0.319 0.326

Locus 8680 :

2 alleles

99.2% missing data

3	(0.522) 0.526 0.526

4	(0.478) 0.474 0.474

Locus 8681 :

2 alleles

99.2% missing data

2	(0.485) 0.480 0.489

3	(0.515) 0.520 0.511

Locus 8682 :

2 alleles

99.2% missing data

2	(0.661) 0.656 0.665

4	(0.339) 0.344 0.335

Locus 8683 :

3 alleles

98.4% missing data

1	(0.464) 0.462 0.459

3	(0.150) 0.146 0.155

2	(0.386) 0.392 0.386

Locus 8684 :

2 alleles

98.0% missing data

4	(0.796) 0.799 0.796

2	(0.204) 0.201 0.204

Locus 8685 :

2 alleles

98.8% missing data

2	(0.461) 0.467 0.454

3	(0.539) 0.533 0.546

Locus 8686 :

2 alleles

99.2% missing data

1	(0.656) 0.655 0.654

3	(0.344) 0.345 0.346

Locus 8687 :

2 alleles

99.2% missing data

1	(0.520) 0.507 0.528

2	(0.480) 0.493 0.472

Locus 8688 :

2 alleles

98.4% missing data

1	(0.498) 0.510 0.496

2	(0.502) 0.490 0.504

Locus 8689 :

2 alleles

99.2% missing data

2	(0.693) 0.691 0.692

1	(0.307) 0.309 0.308

Locus 8690 :

2 alleles

99.2% missing data

4	(0.268) 0.266 0.275

2	(0.732) 0.734 0.725

Locus 8691 :

2 alleles

91.0% missing data

1	(0.916) 0.933 0.911

3	(0.084) 0.067 0.089

Locus 8692 :

2 alleles

99.2% missing data

2	(0.540) 0.532 0.543

3	(0.460) 0.468 0.457

Locus 8693 :

3 alleles

98.8% missing data

3	(0.144) 0.141 0.143

2	(0.374) 0.373 0.385

1	(0.482) 0.486 0.472

Locus 8694 :

2 alleles

9.8% missing data

3	(0.985) 0.982 0.995

2	(0.015) 0.018 0.005

Locus 8695 :

2 alleles

97.7% missing data

2	(0.830) 0.838 0.825

4	(0.170) 0.162 0.175

Locus 8696 :

2 alleles

99.2% missing data

4	(0.483) 0.481 0.486

2	(0.517) 0.519 0.514

Locus 8697 :

3 alleles

98.8% missing data

3	(0.333) 0.333 0.330

4	(0.339) 0.334 0.344

1	(0.327) 0.334 0.326

Locus 8698 :

2 alleles

0.0% missing data

1	(0.986) 0.990 0.992

4	(0.014) 0.010 0.008

Locus 8699 :

2 alleles

99.2% missing data

2	(0.529) 0.529 0.531

1	(0.471) 0.471 0.469

Locus 8700 :

2 alleles

98.0% missing data

4	(0.589) 0.594 0.590

2	(0.411) 0.406 0.410

Locus 8701 :

2 alleles

94.5% missing data

2	(0.890) 0.891 0.895

4	(0.110) 0.109 0.105

Locus 8702 :

2 alleles

98.8% missing data

1	(0.673) 0.660 0.676

4	(0.327) 0.340 0.324

Locus 8703 :

2 alleles

99.2% missing data

4	(0.480) 0.479 0.478

1	(0.520) 0.521 0.522

Locus 8704 :

2 alleles

96.9% missing data

1	(0.809) 0.821 0.807

2	(0.191) 0.179 0.193

Locus 8705 :

2 alleles

97.7% missing data

3	(0.714) 0.707 0.722

4	(0.286) 0.293 0.278

Locus 8706 :

2 alleles

99.2% missing data

4	(0.742) 0.743 0.737

3	(0.258) 0.257 0.263

Locus 8707 :

2 alleles

92.6% missing data

4	(0.909) 0.908 0.914

3	(0.091) 0.092 0.086

Locus 8708 :

2 alleles

99.2% missing data

3	(0.677) 0.679 0.674

1	(0.323) 0.321 0.326

Locus 8709 :

2 alleles

99.2% missing data

1	(0.645) 0.642 0.648

4	(0.355) 0.358 0.352

Locus 8710 :

2 alleles

99.2% missing data

3	(0.659) 0.668 0.658

2	(0.341) 0.332 0.342

Locus 8711 :

2 alleles

99.2% missing data

3	(0.468) 0.470 0.462

1	(0.532) 0.530 0.538

Locus 8712 :

3 alleles

97.3% missing data

2	(0.251) 0.249 0.248

1	(0.486) 0.479 0.503

3	(0.263) 0.272 0.249

Locus 8713 :

2 alleles

93.4% missing data

3	(0.899) 0.896 0.908

1	(0.101) 0.104 0.092

Locus 8714 :

2 alleles

98.8% missing data

2	(0.855) 0.855 0.851

3	(0.145) 0.145 0.149

Locus 8715 :

2 alleles

96.5% missing data

3	(0.923) 0.925 0.922

2	(0.077) 0.075 0.078

Locus 8716 :

2 alleles

93.0% missing data

4	(0.904) 0.904 0.911

2	(0.096) 0.096 0.089

Locus 8717 :

2 alleles

98.8% missing data

1	(0.515) 0.510 0.523

4	(0.485) 0.490 0.477

Locus 8718 :

2 alleles

99.2% missing data

2	(0.386) 0.387 0.391

1	(0.614) 0.613 0.609

Locus 8719 :

2 alleles

99.2% missing data

2	(0.457) 0.453 0.462

4	(0.543) 0.547 0.538

Locus 8720 :

2 alleles

99.2% missing data

2	(0.361) 0.364 0.363

1	(0.639) 0.636 0.637

Locus 8721 :

2 alleles

98.8% missing data

4	(0.321) 0.314 0.328

1	(0.679) 0.686 0.672

Locus 8722 :

2 alleles

99.2% missing data

3	(0.480) 0.479 0.479

2	(0.520) 0.521 0.521

Locus 8723 :

2 alleles

96.5% missing data

2	(0.872) 0.873 0.875

1	(0.128) 0.127 0.125

Locus 8724 :

2 alleles

98.8% missing data

1	(0.562) 0.558 0.564

4	(0.438) 0.442 0.436

Locus 8725 :

2 alleles

99.2% missing data

4	(0.681) 0.685 0.674

3	(0.319) 0.315 0.326

Locus 8726 :

2 alleles

97.7% missing data

1	(0.618) 0.613 0.630

3	(0.382) 0.387 0.370

Locus 8727 :

2 alleles

99.2% missing data

2	(0.589) 0.593 0.585

1	(0.411) 0.407 0.415

Locus 8728 :

2 alleles

99.2% missing data

1	(0.485) 0.483 0.489

2	(0.515) 0.517 0.511

Locus 8729 :

2 alleles

95.7% missing data

1	(0.298) 0.292 0.305

2	(0.702) 0.708 0.695

Locus 8730 :

2 alleles

95.7% missing data

3	(0.872) 0.885 0.869

1	(0.128) 0.115 0.131

Locus 8731 :

3 alleles

98.4% missing data

4	(0.169) 0.166 0.175

3	(0.365) 0.370 0.367

2	(0.466) 0.464 0.458

Locus 8732 :

2 alleles

97.3% missing data

1	(0.236) 0.223 0.235

3	(0.764) 0.777 0.765

Locus 8733 :

2 alleles

93.0% missing data

3	(0.907) 0.899 0.924

1	(0.093) 0.101 0.076

Locus 8734 :

2 alleles

98.8% missing data

2	(0.438) 0.430 0.448

1	(0.562) 0.570 0.552

Locus 8735 :

2 alleles

99.2% missing data

3	(0.453) 0.453 0.456

2	(0.547) 0.547 0.544

Locus 8736 :

2 alleles

90.2% missing data

1	(0.681) 0.639 0.719

2	(0.319) 0.361 0.281

Locus 8737 :

2 alleles

98.0% missing data

2	(0.592) 0.600 0.588

3	(0.408) 0.400 0.412

Locus 8738 :

2 alleles

99.2% missing data

2	(0.215) 0.214 0.213

1	(0.785) 0.786 0.787

Locus 8739 :

2 alleles

99.2% missing data

4	(0.490) 0.499 0.480

2	(0.510) 0.501 0.520

Locus 8740 :

2 alleles

99.2% missing data

1	(0.512) 0.521 0.512

2	(0.488) 0.479 0.488

Locus 8741 :

2 alleles

99.2% missing data

4	(0.466) 0.467 0.463

1	(0.534) 0.533 0.537

Locus 8742 :

2 alleles

97.3% missing data

4	(0.347) 0.346 0.337

3	(0.653) 0.654 0.663

Locus 8743 :

2 alleles

98.4% missing data

2	(0.830) 0.840 0.825

1	(0.170) 0.160 0.175

Locus 8744 :

2 alleles

96.5% missing data

1	(0.671) 0.677 0.676

3	(0.329) 0.323 0.324

Locus 8745 :

2 alleles

99.2% missing data

3	(0.673) 0.678 0.667

2	(0.327) 0.322 0.333

Locus 8746 :

2 alleles

99.2% missing data

4	(0.544) 0.530 0.555

3	(0.456) 0.470 0.445

Locus 8747 :

2 alleles

93.4% missing data

1	(0.926) 0.914 0.939

4	(0.074) 0.086 0.061

Locus 8748 :

2 alleles

98.8% missing data

1	(0.494) 0.496 0.493

3	(0.506) 0.504 0.507

Locus 8749 :

2 alleles

99.2% missing data

2	(0.340) 0.337 0.344

3	(0.660) 0.663 0.656

Locus 8750 :

2 alleles

99.2% missing data

4	(0.598) 0.603 0.595

2	(0.402) 0.397 0.405

Locus 8751 :

2 alleles

98.8% missing data

4	(0.410) 0.403 0.420

3	(0.590) 0.597 0.580

Locus 8752 :

2 alleles

99.2% missing data

1	(0.687) 0.689 0.684

4	(0.313) 0.311 0.316

Locus 8753 :

2 alleles

98.8% missing data

3	(0.376) 0.370 0.386

1	(0.624) 0.630 0.614

Locus 8754 :

2 alleles

99.2% missing data

1	(0.556) 0.559 0.560

3	(0.444) 0.441 0.440

Locus 8755 :

3 alleles

98.8% missing data

4	(0.197) 0.191 0.206

3	(0.416) 0.415 0.418

2	(0.387) 0.394 0.376

Locus 8756 :

2 alleles

97.7% missing data

3	(0.156) 0.150 0.156

2	(0.844) 0.850 0.844

Locus 8757 :

2 alleles

93.0% missing data

2	(0.918) 0.917 0.922

1	(0.082) 0.083 0.078

Locus 8758 :

2 alleles

99.2% missing data

4	(0.846) 0.839 0.850

2	(0.154) 0.161 0.150

Locus 8759 :

2 alleles

99.2% missing data

4	(0.617) 0.619 0.617

1	(0.383) 0.381 0.383

Locus 8760 :

2 alleles

99.2% missing data

4	(0.597) 0.596 0.603

3	(0.403) 0.404 0.397

Locus 8761 :

2 alleles

98.0% missing data

2	(0.811) 0.811 0.807

3	(0.189) 0.189 0.193

Locus 8762 :

3 alleles

98.8% missing data

4	(0.244) 0.244 0.250

3	(0.544) 0.548 0.538

2	(0.212) 0.208 0.211

Locus 8763 :

2 alleles

97.3% missing data

1	(0.270) 0.276 0.261

3	(0.730) 0.724 0.739

Locus 8764 :

2 alleles

98.8% missing data

3	(0.148) 0.139 0.156

4	(0.852) 0.861 0.844

Locus 8765 :

2 alleles

99.2% missing data

3	(0.369) 0.376 0.368

2	(0.631) 0.624 0.632

Locus 8766 :

2 alleles

90.6% missing data

2	(0.901) 0.916 0.897

3	(0.099) 0.084 0.103

Locus 8767 :

2 alleles

99.2% missing data

1	(0.624) 0.616 0.630

3	(0.376) 0.384 0.370

Locus 8768 :

2 alleles

99.2% missing data

4	(0.716) 0.724 0.710

2	(0.284) 0.276 0.290

Locus 8769 :

2 alleles

99.2% missing data

3	(0.642) 0.644 0.638

2	(0.358) 0.356 0.362

Locus 8770 :

2 alleles

98.8% missing data

4	(0.247) 0.243 0.261

1	(0.753) 0.757 0.739

Locus 8771 :

2 alleles

99.2% missing data

3	(0.258) 0.251 0.267

1	(0.742) 0.749 0.733

Locus 8772 :

2 alleles

99.2% missing data

2	(0.733) 0.727 0.736

3	(0.267) 0.273 0.264

Locus 8773 :

2 alleles

99.2% missing data

1	(0.707) 0.711 0.702

2	(0.293) 0.289 0.298

Locus 8774 :

2 alleles

98.8% missing data

4	(0.743) 0.739 0.743

1	(0.257) 0.261 0.257

Locus 8775 :

2 alleles

96.5% missing data

2	(0.672) 0.679 0.681

4	(0.328) 0.321 0.319

Locus 8776 :

2 alleles

99.2% missing data

3	(0.386) 0.380 0.393

1	(0.614) 0.620 0.607

Locus 8777 :

2 alleles

89.1% missing data

3	(0.810) 0.845 0.778

4	(0.190) 0.155 0.222

Locus 8778 :

2 alleles

99.2% missing data

1	(0.427) 0.423 0.429

3	(0.573) 0.577 0.571

Locus 8779 :

2 alleles

99.2% missing data

3	(0.423) 0.426 0.429

4	(0.577) 0.574 0.571

Locus 8780 :

2 alleles

99.2% missing data

2	(0.507) 0.503 0.506

1	(0.493) 0.497 0.494

Locus 8781 :

2 alleles

99.2% missing data

3	(0.618) 0.614 0.626

1	(0.382) 0.386 0.374

Locus 8782 :

2 alleles

99.2% missing data

4	(0.598) 0.602 0.600

1	(0.402) 0.398 0.400

Locus 8783 :

2 alleles

99.2% missing data

2	(0.519) 0.520 0.518

4	(0.481) 0.480 0.482

Locus 8784 :

2 alleles

98.8% missing data

3	(0.641) 0.643 0.638

1	(0.359) 0.357 0.362

Locus 8785 :

3 alleles

96.1% missing data

2	(0.241) 0.257 0.229

4	(0.281) 0.275 0.278

1	(0.478) 0.468 0.493

Locus 8786 :

2 alleles

99.2% missing data

2	(0.319) 0.315 0.319

4	(0.681) 0.685 0.681

Locus 8787 :

2 alleles

99.2% missing data

2	(0.493) 0.495 0.498

1	(0.507) 0.505 0.502

Locus 8788 :

2 alleles

93.0% missing data

2	(0.899) 0.900 0.909

1	(0.101) 0.100 0.091

Locus 8789 :

2 alleles

98.8% missing data

2	(0.326) 0.319 0.338

4	(0.674) 0.681 0.662

Locus 8790 :

2 alleles

92.6% missing data

3	(0.901) 0.894 0.914

2	(0.099) 0.106 0.086

Locus 8791 :

2 alleles

98.8% missing data

2	(0.449) 0.451 0.457

3	(0.551) 0.549 0.543

Locus 8792 :

2 alleles

99.2% missing data

1	(0.395) 0.394 0.396

3	(0.605) 0.606 0.604

Locus 8793 :

2 alleles

98.8% missing data

1	(0.444) 0.443 0.459

4	(0.556) 0.557 0.541

Locus 8794 :

2 alleles

98.0% missing data

2	(0.713) 0.708 0.726

3	(0.287) 0.292 0.274

Locus 8795 :

2 alleles

98.8% missing data

3	(0.304) 0.300 0.311

1	(0.696) 0.700 0.689

Locus 8796 :

2 alleles

98.8% missing data

1	(0.687) 0.688 0.685

4	(0.313) 0.312 0.315

Locus 8797 :

2 alleles

99.2% missing data

4	(0.523) 0.521 0.520

1	(0.477) 0.479 0.480

Locus 8798 :

2 alleles

99.2% missing data

2	(0.294) 0.292 0.297

4	(0.706) 0.708 0.703

Locus 8799 :

2 alleles

92.6% missing data

4	(0.853) 0.837 0.876

1	(0.147) 0.163 0.124

Locus 8800 :

2 alleles

99.2% missing data

1	(0.621) 0.615 0.628

4	(0.379) 0.385 0.372

Locus 8801 :

2 alleles

99.2% missing data

4	(0.390) 0.382 0.393

1	(0.610) 0.618 0.607

Locus 8802 :

2 alleles

97.7% missing data

4	(0.789) 0.780 0.798

2	(0.211) 0.220 0.202

Locus 8803 :

2 alleles

96.1% missing data

1	(0.216) 0.201 0.220

4	(0.784) 0.799 0.780

Locus 8804 :

2 alleles

98.8% missing data

3	(0.551) 0.549 0.557

4	(0.449) 0.451 0.443

Locus 8805 :

2 alleles

99.2% missing data

3	(0.526) 0.523 0.526

2	(0.474) 0.477 0.474

Locus 8806 :

2 alleles

99.2% missing data

4	(0.327) 0.324 0.332

3	(0.673) 0.676 0.668

Locus 8807 :

2 alleles

99.2% missing data

1	(0.497) 0.494 0.501

4	(0.503) 0.506 0.499

Locus 8808 :

2 alleles

7.8% missing data

2	(0.982) 0.975 0.995

3	(0.018) 0.025 0.005

Locus 8809 :

2 alleles

99.2% missing data

3	(0.284) 0.277 0.290

1	(0.716) 0.723 0.710

Locus 8810 :

2 alleles

95.7% missing data

3	(0.833) 0.831 0.845

1	(0.167) 0.169 0.155

Locus 8811 :

2 alleles

96.9% missing data

3	(0.802) 0.809 0.798

1	(0.198) 0.191 0.202

Locus 8812 :

2 alleles

94.5% missing data

4	(0.853) 0.849 0.863

1	(0.147) 0.151 0.137

Locus 8813 :

2 alleles

99.2% missing data

4	(0.384) 0.381 0.386

2	(0.616) 0.619 0.614

Locus 8814 :

2 alleles

99.2% missing data

3	(0.597) 0.597 0.595

1	(0.403) 0.403 0.405

Locus 8815 :

2 alleles

0.4% missing data

2	(0.914) 0.762 0.974

3	(0.086) 0.238 0.026

Locus 8816 :

3 alleles

98.4% missing data

4	(0.404) 0.406 0.410

1	(0.244) 0.237 0.248

3	(0.352) 0.357 0.342

Locus 8817 :

2 alleles

98.0% missing data

3	(0.776) 0.773 0.775

1	(0.224) 0.227 0.225

Locus 8818 :

2 alleles

98.0% missing data

3	(0.511) 0.521 0.507

4	(0.489) 0.479 0.493

Locus 8819 :

2 alleles

99.2% missing data

1	(0.712) 0.710 0.714

4	(0.288) 0.290 0.286

Locus 8820 :

2 alleles

99.2% missing data

1	(0.818) 0.819 0.812

2	(0.182) 0.181 0.188

Locus 8821 :

2 alleles

99.2% missing data

1	(0.416) 0.417 0.413

2	(0.584) 0.583 0.587

Locus 8822 :

2 alleles

98.0% missing data

3	(0.826) 0.838 0.823

4	(0.174) 0.162 0.177

Locus 8823 :

2 alleles

98.8% missing data

1	(0.402) 0.389 0.410

4	(0.598) 0.611 0.590

Locus 8824 :

2 alleles

97.7% missing data

2	(0.585) 0.591 0.591

4	(0.415) 0.409 0.409

Locus 8825 :

2 alleles

98.8% missing data

4	(0.639) 0.640 0.635

3	(0.361) 0.360 0.365

Locus 8826 :

2 alleles

98.8% missing data

3	(0.304) 0.306 0.308

4	(0.696) 0.694 0.692

Locus 8827 :

2 alleles

94.1% missing data

4	(0.357) 0.345 0.366

1	(0.643) 0.655 0.634

Locus 8828 :

2 alleles

98.4% missing data

4	(0.713) 0.722 0.710

3	(0.287) 0.278 0.290

Locus 8829 :

2 alleles

96.1% missing data

1	(0.794) 0.804 0.788

4	(0.206) 0.196 0.212

Locus 8830 :

2 alleles

99.2% missing data

4	(0.673) 0.674 0.668

2	(0.327) 0.326 0.332

Locus 8831 :

2 alleles

98.8% missing data

4	(0.499) 0.495 0.512

2	(0.501) 0.505 0.488

Locus 8832 :

2 alleles

8.2% missing data

3	(0.986) 0.978 0.996

4	(0.014) 0.022 0.004

Locus 8833 :

2 alleles

99.2% missing data

1	(0.462) 0.458 0.470

4	(0.538) 0.542 0.530

Locus 8834 :

2 alleles

99.2% missing data

1	(0.372) 0.371 0.376

3	(0.628) 0.629 0.624

Locus 8835 :

2 alleles

91.8% missing data

2	(0.630) 0.626 0.634

3	(0.370) 0.374 0.366

Locus 8836 :

3 alleles

98.8% missing data

2	(0.293) 0.292 0.295

3	(0.534) 0.527 0.531

4	(0.173) 0.181 0.173

Locus 8837 :

2 alleles

99.2% missing data

4	(0.474) 0.473 0.477

3	(0.526) 0.527 0.523

Locus 8838 :

2 alleles

99.2% missing data

4	(0.415) 0.418 0.412

3	(0.585) 0.582 0.588

Locus 8839 :

2 alleles

98.8% missing data

4	(0.516) 0.523 0.508

2	(0.484) 0.477 0.492

Locus 8840 :

2 alleles

99.2% missing data

3	(0.676) 0.675 0.673

1	(0.324) 0.325 0.327

Locus 8841 :

3 alleles

98.8% missing data

4	(0.672) 0.663 0.674

2	(0.124) 0.136 0.120

3	(0.204) 0.201 0.206

Locus 8842 :

2 alleles

97.7% missing data

2	(0.552) 0.537 0.573

1	(0.448) 0.463 0.427

Locus 8843 :

2 alleles

99.2% missing data

1	(0.350) 0.346 0.354

3	(0.650) 0.654 0.646

Locus 8844 :

2 alleles

99.2% missing data

4	(0.556) 0.555 0.555

2	(0.444) 0.445 0.445

Locus 8845 :

2 alleles

99.2% missing data

2	(0.341) 0.334 0.352

4	(0.659) 0.666 0.648

Locus 8846 :

3 alleles

93.8% missing data

3	(0.792) 0.787 0.812

4	(0.096) 0.095 0.091

2	(0.112) 0.118 0.097

Locus 8847 :

2 alleles

97.3% missing data

4	(0.450) 0.454 0.443

1	(0.550) 0.546 0.557

Locus 8848 :

2 alleles

92.6% missing data

3	(0.121) 0.123 0.106

1	(0.879) 0.877 0.894

Locus 8849 :

3 alleles

98.4% missing data

2	(0.248) 0.246 0.250

3	(0.260) 0.260 0.259

4	(0.493) 0.494 0.492

Locus 8850 :

2 alleles

99.2% missing data

4	(0.697) 0.697 0.691

3	(0.303) 0.303 0.309

Locus 8851 :

2 alleles

99.2% missing data

3	(0.192) 0.188 0.198

4	(0.808) 0.812 0.802

Locus 8852 :

2 alleles

99.2% missing data

4	(0.425) 0.420 0.425

2	(0.575) 0.580 0.575

Locus 8853 :

2 alleles

98.4% missing data

4	(0.755) 0.758 0.755

1	(0.245) 0.242 0.245

Locus 8854 :

2 alleles

99.2% missing data

1	(0.782) 0.782 0.782

4	(0.218) 0.218 0.218

Locus 8855 :

2 alleles

91.0% missing data

1	(0.924) 0.940 0.915

4	(0.076) 0.060 0.085

Locus 8856 :

2 alleles

99.2% missing data

3	(0.190) 0.193 0.185

4	(0.810) 0.807 0.815

Locus 8857 :

2 alleles

98.0% missing data

2	(0.314) 0.317 0.311

4	(0.686) 0.683 0.689

Locus 8858 :

2 alleles

98.4% missing data

4	(0.276) 0.287 0.276

3	(0.724) 0.713 0.724

Locus 8859 :

2 alleles

98.8% missing data

3	(0.700) 0.705 0.703

2	(0.300) 0.295 0.297

Locus 8860 :

2 alleles

99.2% missing data

3	(0.441) 0.445 0.436

2	(0.559) 0.555 0.564

Locus 8861 :

2 alleles

99.2% missing data

1	(0.368) 0.369 0.365

4	(0.632) 0.631 0.635

Locus 8862 :

2 alleles

96.9% missing data

3	(0.258) 0.259 0.247

1	(0.742) 0.741 0.753

Locus 8863 :

2 alleles

99.2% missing data

3	(0.428) 0.441 0.426

4	(0.572) 0.559 0.574

Locus 8864 :

2 alleles

99.2% missing data

3	(0.633) 0.626 0.638

1	(0.367) 0.374 0.362

Locus 8865 :

2 alleles

98.4% missing data

2	(0.734) 0.741 0.730

3	(0.266) 0.259 0.270

Locus 8866 :

2 alleles

99.2% missing data

1	(0.666) 0.663 0.665

2	(0.334) 0.337 0.335

Locus 8867 :

2 alleles

99.2% missing data

4	(0.311) 0.305 0.315

1	(0.689) 0.695 0.685

Locus 8868 :

2 alleles

0.0% missing data

4	(0.987) 0.990 0.992

1	(0.013) 0.010 0.008

Locus 8869 :

2 alleles

99.2% missing data

1	(0.745) 0.750 0.743

4	(0.255) 0.250 0.257

Locus 8870 :

2 alleles

87.5% missing data

3	(0.721) 0.737 0.716

1	(0.279) 0.263 0.284

Locus 8871 :

2 alleles

99.2% missing data

3	(0.309) 0.312 0.307

2	(0.691) 0.688 0.693

Locus 8872 :

2 alleles

17.6% missing data

1	(0.981) 0.971 0.994

4	(0.019) 0.029 0.006

Locus 8873 :

2 alleles

99.2% missing data

2	(0.314) 0.315 0.314

3	(0.686) 0.685 0.686

Locus 8874 :

2 alleles

98.4% missing data

3	(0.499) 0.496 0.502

1	(0.501) 0.504 0.498

Locus 8875 :

2 alleles

99.2% missing data

1	(0.394) 0.390 0.396

2	(0.606) 0.610 0.604

Locus 8876 :

2 alleles

92.6% missing data

1	(0.902) 0.913 0.899

2	(0.098) 0.087 0.101

Locus 8877 :

2 alleles

98.0% missing data

1	(0.706) 0.709 0.705

2	(0.294) 0.291 0.295

Locus 8878 :

2 alleles

99.2% missing data

2	(0.355) 0.353 0.352

1	(0.645) 0.647 0.648

Locus 8879 :

2 alleles

98.4% missing data

1	(0.727) 0.727 0.731

3	(0.273) 0.273 0.269

Locus 8880 :

2 alleles

99.2% missing data

1	(0.450) 0.449 0.447

3	(0.550) 0.551 0.553

Locus 8881 :

2 alleles

97.7% missing data

1	(0.826) 0.833 0.824

3	(0.174) 0.167 0.176

Locus 8882 :

2 alleles

99.2% missing data

1	(0.454) 0.457 0.450

3	(0.546) 0.543 0.550

Locus 8883 :

2 alleles

96.5% missing data

4	(0.710) 0.722 0.698

1	(0.290) 0.278 0.302

Locus 8884 :

2 alleles

97.7% missing data

4	(0.804) 0.802 0.807

1	(0.196) 0.198 0.193

Locus 8885 :

2 alleles

99.2% missing data

1	(0.349) 0.358 0.346

3	(0.651) 0.642 0.654

Locus 8886 :

2 alleles

99.2% missing data

4	(0.474) 0.472 0.472

1	(0.526) 0.528 0.528

Locus 8887 :

2 alleles

98.8% missing data

1	(0.559) 0.560 0.562

3	(0.441) 0.440 0.438

Locus 8888 :

2 alleles

97.7% missing data

3	(0.244) 0.234 0.247

1	(0.756) 0.766 0.753

Locus 8889 :

2 alleles

99.2% missing data

3	(0.658) 0.655 0.659

1	(0.342) 0.345 0.341

Locus 8890 :

2 alleles

99.2% missing data

4	(0.617) 0.616 0.619

1	(0.383) 0.384 0.381

Locus 8891 :

2 alleles

99.2% missing data

4	(0.495) 0.499 0.494

3	(0.505) 0.501 0.506

Locus 8892 :

2 alleles

99.2% missing data

4	(0.543) 0.532 0.552

3	(0.457) 0.468 0.448

Locus 8893 :

2 alleles

99.2% missing data

3	(0.615) 0.610 0.618

2	(0.385) 0.390 0.382

Locus 8894 :

2 alleles

99.2% missing data

3	(0.306) 0.302 0.308

2	(0.694) 0.698 0.692

Locus 8895 :

2 alleles

94.1% missing data

2	(0.555) 0.556 0.555

3	(0.445) 0.444 0.445

Locus 8896 :

2 alleles

99.2% missing data

4	(0.281) 0.284 0.285

1	(0.719) 0.716 0.715

Locus 8897 :

2 alleles

99.2% missing data

1	(0.478) 0.474 0.476

4	(0.522) 0.526 0.524

Locus 8898 :

2 alleles

99.2% missing data

4	(0.519) 0.516 0.524

3	(0.481) 0.484 0.476

Locus 8899 :

2 alleles

98.0% missing data

1	(0.649) 0.643 0.659

4	(0.351) 0.357 0.341

Locus 8900 :

2 alleles

99.2% missing data

2	(0.611) 0.607 0.614

3	(0.389) 0.393 0.386

Locus 8901 :

2 alleles

99.2% missing data

3	(0.491) 0.490 0.495

1	(0.509) 0.510 0.505

Locus 8902 :

2 alleles

98.8% missing data

1	(0.426) 0.422 0.420

4	(0.574) 0.578 0.580

Locus 8903 :

2 alleles

99.2% missing data

3	(0.509) 0.504 0.512

4	(0.491) 0.496 0.488

Locus 8904 :

2 alleles

99.2% missing data

1	(0.610) 0.601 0.617

4	(0.390) 0.399 0.383

Locus 8905 :

3 alleles

95.7% missing data

4	(0.173) 0.156 0.180

3	(0.691) 0.711 0.688

2	(0.136) 0.133 0.132

Locus 8906 :

2 alleles

98.8% missing data

1	(0.513) 0.503 0.523

2	(0.487) 0.497 0.477

Locus 8907 :

2 alleles

99.2% missing data

1	(0.513) 0.513 0.513

4	(0.487) 0.487 0.487

Locus 8908 :

2 alleles

97.3% missing data

3	(0.185) 0.173 0.193

4	(0.815) 0.827 0.807

Locus 8909 :

2 alleles

99.2% missing data

1	(0.381) 0.380 0.382

2	(0.619) 0.620 0.618

Locus 8910 :

2 alleles

98.8% missing data

2	(0.725) 0.728 0.729

4	(0.275) 0.272 0.271

Locus 8911 :

2 alleles

99.2% missing data

4	(0.495) 0.496 0.496

3	(0.505) 0.504 0.504

Locus 8912 :

2 alleles

98.4% missing data

4	(0.590) 0.588 0.595

1	(0.410) 0.412 0.405

Locus 8913 :

2 alleles

98.8% missing data

3	(0.469) 0.461 0.480

1	(0.531) 0.539 0.520

Locus 8914 :

2 alleles

0.0% missing data

2	(0.912) 0.762 0.973

3	(0.088) 0.238 0.027

Locus 8915 :

2 alleles

97.7% missing data

3	(0.783) 0.789 0.774

2	(0.217) 0.211 0.226

Locus 8916 :

2 alleles

98.0% missing data

2	(0.739) 0.741 0.740

4	(0.261) 0.259 0.260

Locus 8917 :

2 alleles

99.2% missing data

2	(0.448) 0.449 0.449

4	(0.552) 0.551 0.551

Locus 8918 :

2 alleles

99.2% missing data

3	(0.491) 0.485 0.494

4	(0.509) 0.515 0.506

Locus 8919 :

2 alleles

99.2% missing data

2	(0.223) 0.219 0.226

3	(0.777) 0.781 0.774

Locus 8920 :

2 alleles

99.2% missing data

3	(0.505) 0.506 0.502

4	(0.495) 0.494 0.498

Locus 8921 :

2 alleles

99.2% missing data

3	(0.463) 0.460 0.461

1	(0.537) 0.540 0.539

Locus 8922 :

2 alleles

99.2% missing data

2	(0.546) 0.546 0.548

3	(0.454) 0.454 0.452

Locus 8923 :

2 alleles

99.2% missing data

4	(0.618) 0.613 0.619

3	(0.382) 0.387 0.381

Locus 8924 :

2 alleles

98.8% missing data

1	(0.407) 0.398 0.413

4	(0.593) 0.602 0.587

Locus 8925 :

2 alleles

96.1% missing data

3	(0.893) 0.893 0.898

4	(0.107) 0.107 0.102

Locus 8926 :

2 alleles

99.2% missing data

3	(0.512) 0.506 0.516

4	(0.488) 0.494 0.484

Locus 8927 :

2 alleles

98.4% missing data

1	(0.777) 0.773 0.780

3	(0.223) 0.227 0.220

Locus 8928 :

2 alleles

96.9% missing data

3	(0.777) 0.789 0.772

1	(0.223) 0.211 0.228

Locus 8929 :

2 alleles

99.2% missing data

1	(0.325) 0.328 0.325

3	(0.675) 0.672 0.675

Locus 8930 :

2 alleles

99.2% missing data

1	(0.516) 0.510 0.516

4	(0.484) 0.490 0.484

Locus 8931 :

2 alleles

91.0% missing data

1	(0.882) 0.903 0.876

3	(0.118) 0.097 0.124

Locus 8932 :

2 alleles

99.2% missing data

3	(0.348) 0.348 0.350

2	(0.652) 0.652 0.650

Locus 8933 :

2 alleles

99.2% missing data

3	(0.680) 0.682 0.679

1	(0.320) 0.318 0.321

Locus 8934 :

2 alleles

99.2% missing data

4	(0.666) 0.667 0.661

3	(0.334) 0.333 0.339

Locus 8935 :

2 alleles

99.2% missing data

4	(0.313) 0.320 0.318

2	(0.687) 0.680 0.682

Locus 8936 :

2 alleles

99.2% missing data

1	(0.776) 0.768 0.781

2	(0.224) 0.232 0.219

Locus 8937 :

2 alleles

96.9% missing data

1	(0.718) 0.719 0.723

2	(0.282) 0.281 0.277

Locus 8938 :

2 alleles

98.4% missing data

4	(0.678) 0.686 0.677

3	(0.322) 0.314 0.323

Locus 8939 :

2 alleles

99.2% missing data

3	(0.494) 0.501 0.489

4	(0.506) 0.499 0.511

Locus 8940 :

2 alleles

99.2% missing data

3	(0.328) 0.326 0.331

4	(0.672) 0.674 0.669

Locus 8941 :

3 alleles

98.8% missing data

3	(0.198) 0.190 0.200

2	(0.589) 0.599 0.581

1	(0.213) 0.211 0.220

Locus 8942 :

2 alleles

99.2% missing data

2	(0.718) 0.716 0.711

1	(0.282) 0.284 0.289

Locus 8943 :

2 alleles

98.8% missing data

1	(0.357) 0.353 0.361

2	(0.643) 0.647 0.639

Locus 8944 :

2 alleles

99.2% missing data

3	(0.605) 0.603 0.604

2	(0.395) 0.397 0.396

Locus 8945 :

2 alleles

98.0% missing data

1	(0.520) 0.516 0.531

4	(0.480) 0.484 0.469

Locus 8946 :

2 alleles

98.4% missing data

1	(0.427) 0.428 0.422

3	(0.573) 0.572 0.578

Locus 8947 :

2 alleles

99.2% missing data

1	(0.474) 0.475 0.469

4	(0.526) 0.525 0.531

Locus 8948 :

2 alleles

98.4% missing data

3	(0.262) 0.260 0.264

2	(0.738) 0.740 0.736

Locus 8949 :

2 alleles

99.2% missing data

4	(0.400) 0.410 0.395

1	(0.600) 0.590 0.605

Locus 8950 :

2 alleles

96.5% missing data

1	(0.777) 0.792 0.767

2	(0.223) 0.208 0.233

Locus 8951 :

2 alleles

91.0% missing data

3	(0.950) 0.956 0.947

1	(0.050) 0.044 0.053

Locus 8952 :

2 alleles

98.8% missing data

1	(0.486) 0.491 0.489

4	(0.514) 0.509 0.511

Locus 8953 :

2 alleles

99.2% missing data

4	(0.662) 0.657 0.660

1	(0.338) 0.343 0.340

Locus 8954 :

2 alleles

99.2% missing data

2	(0.511) 0.507 0.509

3	(0.489) 0.493 0.491

Locus 8955 :

2 alleles

97.3% missing data

1	(0.825) 0.827 0.826

2	(0.175) 0.173 0.174

Locus 8956 :

2 alleles

92.2% missing data

3	(0.918) 0.921 0.926

4	(0.082) 0.079 0.074

Locus 8957 :

2 alleles

98.8% missing data

2	(0.710) 0.708 0.708

1	(0.290) 0.292 0.292

Locus 8958 :

2 alleles

99.2% missing data

1	(0.608) 0.600 0.617

4	(0.392) 0.400 0.383

Locus 8959 :

2 alleles

91.4% missing data

1	(0.890) 0.893 0.897

2	(0.110) 0.107 0.103

Locus 8960 :

2 alleles

93.4% missing data

3	(0.725) 0.726 0.712

2	(0.275) 0.274 0.288

Locus 8961 :

2 alleles

99.2% missing data

3	(0.324) 0.322 0.333

4	(0.676) 0.678 0.667

Locus 8962 :

2 alleles

99.2% missing data

2	(0.834) 0.830 0.838

1	(0.166) 0.170 0.162

Locus 8963 :

3 alleles

98.8% missing data

2	(0.316) 0.310 0.320

4	(0.290) 0.287 0.292

1	(0.394) 0.403 0.388

Locus 8964 :

2 alleles

98.8% missing data

1	(0.417) 0.421 0.413

2	(0.583) 0.579 0.587

Locus 8965 :

3 alleles

98.8% missing data

1	(0.355) 0.354 0.357

3	(0.444) 0.445 0.437

2	(0.200) 0.202 0.206

Locus 8966 :

2 alleles

99.2% missing data

1	(0.510) 0.505 0.512

4	(0.490) 0.495 0.488

Locus 8967 :

2 alleles

97.7% missing data

2	(0.172) 0.168 0.176

1	(0.828) 0.832 0.824

Locus 8968 :

2 alleles

0.0% missing data

4	(0.987) 0.990 0.993

3	(0.013) 0.010 0.007

Locus 8969 :

2 alleles

97.3% missing data

1	(0.771) 0.771 0.777

2	(0.229) 0.229 0.223

Locus 8970 :

2 alleles

99.2% missing data

1	(0.834) 0.822 0.838

2	(0.166) 0.178 0.162

Locus 8971 :

3 alleles

98.8% missing data

2	(0.531) 0.524 0.527

1	(0.145) 0.152 0.144

4	(0.324) 0.324 0.329

Locus 8972 :

2 alleles

99.2% missing data

2	(0.593) 0.602 0.586

1	(0.407) 0.398 0.414

Locus 8973 :

2 alleles

97.7% missing data

1	(0.494) 0.505 0.475

2	(0.506) 0.495 0.525

Locus 8974 :

2 alleles

99.2% missing data

4	(0.666) 0.661 0.657

2	(0.334) 0.339 0.343

Locus 8975 :

2 alleles

99.2% missing data

4	(0.315) 0.308 0.319

2	(0.685) 0.692 0.681

Locus 8976 :

2 alleles

99.2% missing data

2	(0.456) 0.453 0.458

1	(0.544) 0.547 0.542

Locus 8977 :

2 alleles

98.0% missing data

2	(0.529) 0.526 0.542

4	(0.471) 0.474 0.458

Locus 8978 :

2 alleles

97.3% missing data

1	(0.807) 0.800 0.819

3	(0.193) 0.200 0.181

Locus 8979 :

2 alleles

98.8% missing data

3	(0.426) 0.414 0.431

2	(0.574) 0.586 0.569

Locus 8980 :

2 alleles

99.2% missing data

2	(0.503) 0.504 0.498

3	(0.497) 0.496 0.502

Locus 8981 :

2 alleles

99.2% missing data

4	(0.285) 0.284 0.291

3	(0.715) 0.716 0.709

Locus 8982 :

3 alleles

9.0% missing data

2	(0.963) 0.952 0.989

3	(0.021) 0.026 0.006

4	(0.016) 0.021 0.005

Locus 8983 :

2 alleles

99.2% missing data

1	(0.609) 0.606 0.609

4	(0.391) 0.394 0.391

Locus 8984 :

2 alleles

99.2% missing data

1	(0.827) 0.830 0.818

3	(0.173) 0.170 0.182

Locus 8985 :

2 alleles

98.4% missing data

2	(0.503) 0.500 0.510

1	(0.497) 0.500 0.490

Locus 8986 :

2 alleles

98.8% missing data

4	(0.715) 0.716 0.716

2	(0.285) 0.284 0.284

Locus 8987 :

2 alleles

0.4% missing data

4	(0.984) 0.987 0.992

1	(0.016) 0.013 0.008

Locus 8988 :

2 alleles

99.2% missing data

3	(0.581) 0.577 0.581

1	(0.419) 0.423 0.419

Locus 8989 :

2 alleles

94.9% missing data

1	(0.353) 0.350 0.357

2	(0.647) 0.650 0.643

Locus 8990 :

2 alleles

99.2% missing data

4	(0.663) 0.666 0.660

3	(0.337) 0.334 0.340

Locus 8991 :

2 alleles

99.2% missing data

1	(0.363) 0.357 0.369

3	(0.637) 0.643 0.631

Locus 8992 :

2 alleles

99.2% missing data

1	(0.782) 0.790 0.778

3	(0.218) 0.210 0.222

Locus 8993 :

2 alleles

99.2% missing data

4	(0.436) 0.436 0.440

2	(0.564) 0.564 0.560

Locus 8994 :

2 alleles

99.2% missing data

4	(0.464) 0.456 0.470

2	(0.536) 0.544 0.530

Locus 8995 :

2 alleles

0.0% missing data

4	(0.988) 0.990 0.993

1	(0.012) 0.010 0.007

Locus 8996 :

2 alleles

98.8% missing data

2	(0.508) 0.510 0.503

4	(0.492) 0.490 0.497

Locus 8997 :

2 alleles

99.2% missing data

3	(0.640) 0.645 0.634

1	(0.360) 0.355 0.366

Locus 8998 :

2 alleles

99.2% missing data

3	(0.509) 0.517 0.505

2	(0.491) 0.483 0.495

Locus 8999 :

2 alleles

99.2% missing data

1	(0.500) 0.493 0.502

2	(0.500) 0.507 0.498

Locus 9000 :

2 alleles

99.2% missing data

3	(0.761) 0.760 0.759

4	(0.239) 0.240 0.241

Locus 9001 :

2 alleles

96.9% missing data

2	(0.901) 0.904 0.901

3	(0.099) 0.096 0.099

Locus 9002 :

2 alleles

94.1% missing data

1	(0.877) 0.873 0.890

3	(0.123) 0.127 0.110

Locus 9003 :

2 alleles

97.7% missing data

3	(0.192) 0.183 0.197

1	(0.808) 0.817 0.803

Locus 9004 :

2 alleles

98.4% missing data

1	(0.674) 0.674 0.675

4	(0.326) 0.326 0.325

Locus 9005 :

2 alleles

99.2% missing data

2	(0.375) 0.380 0.375

4	(0.625) 0.620 0.625

Locus 9006 :

3 alleles

98.8% missing data

2	(0.253) 0.246 0.257

4	(0.497) 0.499 0.498

3	(0.249) 0.255 0.245

Locus 9007 :

2 alleles

99.2% missing data

4	(0.699) 0.693 0.700

1	(0.301) 0.307 0.300

Locus 9008 :

2 alleles

90.2% missing data

2	(0.857) 0.885 0.841

4	(0.143) 0.115 0.159

Locus 9009 :

2 alleles

99.2% missing data

2	(0.475) 0.476 0.475

1	(0.525) 0.524 0.525

Locus 9010 :

2 alleles

96.9% missing data

2	(0.164) 0.165 0.161

3	(0.836) 0.835 0.839

Locus 9011 :

2 alleles

99.2% missing data

1	(0.383) 0.388 0.381

4	(0.617) 0.612 0.619

Locus 9012 :

2 alleles

98.8% missing data

3	(0.391) 0.388 0.396

1	(0.609) 0.612 0.604

Locus 9013 :

2 alleles

98.0% missing data

1	(0.578) 0.580 0.578

2	(0.422) 0.420 0.422

Locus 9014 :

2 alleles

99.2% missing data

1	(0.324) 0.316 0.331

2	(0.676) 0.684 0.669

Locus 9015 :

2 alleles

96.5% missing data

1	(0.827) 0.819 0.839

2	(0.173) 0.181 0.161

Locus 9016 :

2 alleles

99.2% missing data

4	(0.431) 0.418 0.436

1	(0.569) 0.582 0.564

Locus 9017 :

2 alleles

91.0% missing data

1	(0.910) 0.924 0.901

2	(0.090) 0.076 0.099

Locus 9018 :

2 alleles

8.2% missing data

3	(0.983) 0.975 0.995

1	(0.017) 0.025 0.005

Locus 9019 :

2 alleles

99.2% missing data

1	(0.257) 0.258 0.264

4	(0.743) 0.742 0.736

Locus 9020 :

2 alleles

99.2% missing data

2	(0.528) 0.534 0.527

1	(0.472) 0.466 0.473

Locus 9021 :

2 alleles

96.9% missing data

2	(0.555) 0.561 0.541

3	(0.445) 0.439 0.459

Locus 9022 :

2 alleles

99.2% missing data

1	(0.545) 0.536 0.547

4	(0.455) 0.464 0.453

Locus 9023 :

2 alleles

97.3% missing data

1	(0.297) 0.285 0.305

2	(0.703) 0.715 0.695

Locus 9024 :

2 alleles

92.6% missing data

2	(0.921) 0.924 0.925

3	(0.079) 0.076 0.075

Locus 9025 :

2 alleles

99.2% missing data

1	(0.347) 0.350 0.341

4	(0.653) 0.650 0.659

Locus 9026 :

2 alleles

99.2% missing data

4	(0.618) 0.620 0.618

3	(0.382) 0.380 0.382

Locus 9027 :

3 alleles

98.0% missing data

1	(0.276) 0.269 0.276

2	(0.296) 0.282 0.305

3	(0.428) 0.449 0.419

Locus 9028 :

2 alleles

98.8% missing data

1	(0.625) 0.627 0.630

3	(0.375) 0.373 0.370

Locus 9029 :

2 alleles

99.2% missing data

1	(0.501) 0.508 0.497

2	(0.499) 0.492 0.503

Locus 9030 :

2 alleles

99.2% missing data

4	(0.570) 0.568 0.569

1	(0.430) 0.432 0.431

Locus 9031 :

2 alleles

98.8% missing data

1	(0.506) 0.505 0.510

3	(0.494) 0.495 0.490

Locus 9032 :

2 alleles

96.1% missing data

1	(0.771) 0.784 0.769

3	(0.229) 0.216 0.231

Locus 9033 :

2 alleles

99.2% missing data

2	(0.281) 0.282 0.285

3	(0.719) 0.718 0.715

Locus 9034 :

2 alleles

99.2% missing data

1	(0.660) 0.663 0.651

3	(0.340) 0.337 0.349

Locus 9035 :

2 alleles

98.8% missing data

3	(0.641) 0.648 0.641

2	(0.359) 0.352 0.359

Locus 9036 :

2 alleles

96.9% missing data

3	(0.160) 0.164 0.149

1	(0.840) 0.836 0.851

Locus 9037 :

2 alleles

98.8% missing data

1	(0.558) 0.558 0.554

3	(0.442) 0.442 0.446

Locus 9038 :

2 alleles

99.2% missing data

1	(0.477) 0.475 0.479

4	(0.523) 0.525 0.521

Locus 9039 :

2 alleles

99.2% missing data

1	(0.576) 0.579 0.577

2	(0.424) 0.421 0.423

Locus 9040 :

2 alleles

99.2% missing data

4	(0.633) 0.632 0.633

1	(0.367) 0.368 0.367

Locus 9041 :

2 alleles

98.4% missing data

1	(0.492) 0.481 0.492

4	(0.508) 0.519 0.508

Locus 9042 :

2 alleles

99.2% missing data

1	(0.248) 0.252 0.250

3	(0.752) 0.748 0.750

Locus 9043 :

2 alleles

96.5% missing data

1	(0.869) 0.877 0.862

4	(0.131) 0.123 0.138

Locus 9044 :

2 alleles

8.2% missing data

4	(0.987) 0.980 0.996

2	(0.013) 0.020 0.004

Locus 9045 :

2 alleles

99.2% missing data

4	(0.634) 0.630 0.635

2	(0.366) 0.370 0.365

Locus 9046 :

2 alleles

99.2% missing data

4	(0.544) 0.545 0.545

3	(0.456) 0.455 0.455

Locus 9047 :

2 alleles

99.2% missing data

4	(0.247) 0.243 0.258

1	(0.753) 0.757 0.742

Locus 9048 :

2 alleles

98.8% missing data

3	(0.657) 0.661 0.655

4	(0.343) 0.339 0.345

Locus 9049 :

2 alleles

99.2% missing data

3	(0.663) 0.661 0.661

1	(0.337) 0.339 0.339

Locus 9050 :

3 alleles

97.7% missing data

1	(0.491) 0.488 0.506

2	(0.254) 0.252 0.246

3	(0.255) 0.259 0.248

Locus 9051 :

2 alleles

99.2% missing data

1	(0.608) 0.616 0.598

4	(0.392) 0.384 0.402

Locus 9052 :

2 alleles

99.2% missing data

1	(0.157) 0.161 0.153

4	(0.843) 0.839 0.847

Locus 9053 :

2 alleles

98.8% missing data

1	(0.715) 0.716 0.710

2	(0.285) 0.284 0.290

Locus 9054 :

3 alleles

98.8% missing data

1	(0.238) 0.232 0.242

4	(0.243) 0.245 0.242

2	(0.519) 0.523 0.516

Locus 9055 :

2 alleles

99.2% missing data

4	(0.465) 0.467 0.466

1	(0.535) 0.533 0.534

Locus 9056 :

2 alleles

99.2% missing data

1	(0.267) 0.266 0.269

4	(0.733) 0.734 0.731

Locus 9057 :

2 alleles

99.2% missing data

2	(0.551) 0.545 0.562

1	(0.449) 0.455 0.438

Locus 9058 :

2 alleles

99.2% missing data

2	(0.355) 0.350 0.354

4	(0.645) 0.650 0.646

Locus 9059 :

2 alleles

96.5% missing data

3	(0.860) 0.854 0.869

2	(0.140) 0.146 0.131

Locus 9060 :

2 alleles

99.2% missing data

4	(0.581) 0.574 0.586

3	(0.419) 0.426 0.414

Locus 9061 :

2 alleles

95.3% missing data

4	(0.128) 0.127 0.121

1	(0.872) 0.873 0.879

Locus 9062 :

2 alleles

7.8% missing data

4	(0.988) 0.986 0.995

2	(0.012) 0.014 0.005

Locus 9063 :

2 alleles

99.2% missing data

4	(0.589) 0.593 0.588

1	(0.411) 0.407 0.412

Locus 9064 :

2 alleles

94.9% missing data

1	(0.828) 0.825 0.838

4	(0.172) 0.175 0.162

Locus 9065 :

2 alleles

98.8% missing data

2	(0.708) 0.697 0.709

4	(0.292) 0.303 0.291

Locus 9066 :

2 alleles

97.3% missing data

2	(0.834) 0.831 0.837

1	(0.166) 0.169 0.163

Locus 9067 :

2 alleles

98.4% missing data

2	(0.745) 0.734 0.752

4	(0.255) 0.266 0.248

Locus 9068 :

2 alleles

98.8% missing data

4	(0.731) 0.723 0.742

2	(0.269) 0.277 0.258

Locus 9069 :

2 alleles

99.2% missing data

1	(0.745) 0.746 0.743

3	(0.255) 0.254 0.257

Locus 9070 :

3 alleles

98.0% missing data

3	(0.635) 0.640 0.632

2	(0.174) 0.168 0.180

4	(0.191) 0.192 0.188

Locus 9071 :

2 alleles

99.2% missing data

3	(0.726) 0.722 0.724

2	(0.274) 0.278 0.276

Locus 9072 :

2 alleles

99.2% missing data

1	(0.455) 0.460 0.457

3	(0.545) 0.540 0.543

Locus 9073 :

2 alleles

0.4% missing data

1	(0.987) 0.991 0.993

4	(0.013) 0.009 0.007

Locus 9074 :

2 alleles

99.2% missing data

4	(0.330) 0.325 0.332

2	(0.670) 0.675 0.668

Locus 9075 :

2 alleles

99.2% missing data

1	(0.709) 0.709 0.708

4	(0.291) 0.291 0.292

Locus 9076 :

2 alleles

8.6% missing data

2	(0.985) 0.986 0.993

4	(0.015) 0.014 0.007

Locus 9077 :

2 alleles

99.2% missing data

3	(0.802) 0.806 0.794

2	(0.198) 0.194 0.206

Locus 9078 :

2 alleles

97.7% missing data

4	(0.678) 0.672 0.692

3	(0.322) 0.328 0.308

Locus 9079 :

2 alleles

98.8% missing data

2	(0.574) 0.566 0.576

4	(0.426) 0.434 0.424

Locus 9080 :

2 alleles

99.2% missing data

2	(0.195) 0.193 0.196

4	(0.805) 0.807 0.804

Locus 9081 :

2 alleles

99.2% missing data

2	(0.393) 0.385 0.398

1	(0.607) 0.615 0.602

Locus 9082 :

2 alleles

99.2% missing data

3	(0.204) 0.207 0.206

1	(0.796) 0.793 0.794

Locus 9083 :

2 alleles

99.2% missing data

1	(0.399) 0.396 0.401

4	(0.601) 0.604 0.599

Locus 9084 :

2 alleles

99.2% missing data

3	(0.545) 0.544 0.543

1	(0.455) 0.456 0.457

Locus 9085 :

2 alleles

99.2% missing data

2	(0.503) 0.498 0.510

3	(0.497) 0.502 0.490

Locus 9086 :

2 alleles

98.8% missing data

1	(0.680) 0.682 0.681

3	(0.320) 0.318 0.319

Locus 9087 :

2 alleles

98.8% missing data

1	(0.422) 0.419 0.433

2	(0.578) 0.581 0.567

Locus 9088 :

2 alleles

0.0% missing data

1	(0.984) 0.986 0.989

4	(0.016) 0.014 0.011

Locus 9089 :

2 alleles

98.4% missing data

1	(0.587) 0.596 0.583

3	(0.413) 0.404 0.417

Locus 9090 :

2 alleles

98.8% missing data

2	(0.555) 0.549 0.568

4	(0.445) 0.451 0.432

Locus 9091 :

2 alleles

99.2% missing data

1	(0.301) 0.299 0.309

2	(0.699) 0.701 0.691

Locus 9092 :

2 alleles

9.0% missing data

4	(0.985) 0.978 0.995

1	(0.015) 0.022 0.005

Locus 9093 :

2 alleles

99.2% missing data

1	(0.700) 0.706 0.692

4	(0.300) 0.294 0.308

Locus 9094 :

3 alleles

97.3% missing data

3	(0.286) 0.286 0.275

1	(0.196) 0.202 0.190

2	(0.518) 0.511 0.535

Locus 9095 :

2 alleles

96.1% missing data

2	(0.197) 0.186 0.199

1	(0.803) 0.814 0.801

Locus 9096 :

2 alleles

98.8% missing data

2	(0.741) 0.738 0.744

3	(0.259) 0.262 0.256

Locus 9097 :

3 alleles

98.4% missing data

1	(0.185) 0.182 0.203

4	(0.166) 0.173 0.163

2	(0.650) 0.645 0.634

Locus 9098 :

2 alleles

98.0% missing data

1	(0.779) 0.783 0.781

4	(0.221) 0.217 0.219

Locus 9099 :

2 alleles

98.8% missing data

4	(0.426) 0.426 0.428

2	(0.574) 0.574 0.572

Locus 9100 :

2 alleles

89.8% missing data

3	(0.802) 0.816 0.791

4	(0.198) 0.184 0.209

Locus 9101 :

3 alleles

98.0% missing data

3	(0.214) 0.221 0.209

1	(0.565) 0.565 0.566

4	(0.221) 0.214 0.225

Locus 9102 :

3 alleles

96.5% missing data

4	(0.162) 0.166 0.155

1	(0.707) 0.704 0.715

2	(0.131) 0.130 0.130

Locus 9103 :

2 alleles

99.2% missing data

1	(0.364) 0.363 0.368

2	(0.636) 0.637 0.632

Locus 9104 :

2 alleles

8.2% missing data

4	(0.982) 0.975 0.995

3	(0.018) 0.025 0.005

Locus 9105 :

2 alleles

99.2% missing data

1	(0.469) 0.468 0.473

2	(0.531) 0.532 0.527

Locus 9106 :

2 alleles

99.2% missing data

4	(0.725) 0.720 0.729

1	(0.275) 0.280 0.271

Locus 9107 :

2 alleles

98.8% missing data

1	(0.500) 0.501 0.506

2	(0.500) 0.499 0.494

Locus 9108 :

2 alleles

95.7% missing data

3	(0.888) 0.886 0.889

1	(0.112) 0.114 0.111

Locus 9109 :

2 alleles

97.3% missing data

3	(0.551) 0.558 0.547

4	(0.449) 0.442 0.453

Locus 9110 :

2 alleles

99.2% missing data

1	(0.546) 0.544 0.544

2	(0.454) 0.456 0.456

Locus 9111 :

2 alleles

99.2% missing data

1	(0.288) 0.291 0.294

2	(0.712) 0.709 0.706

Locus 9112 :

2 alleles

99.2% missing data

2	(0.371) 0.371 0.370

1	(0.629) 0.629 0.630

Locus 9113 :

2 alleles

99.2% missing data

1	(0.363) 0.362 0.372

4	(0.637) 0.638 0.628

Locus 9114 :

3 alleles

97.3% missing data

3	(0.063) 0.061 0.067

2	(0.247) 0.258 0.235

1	(0.690) 0.681 0.698

Locus 9115 :

2 alleles

99.2% missing data

1	(0.634) 0.637 0.638

2	(0.366) 0.363 0.362

Locus 9116 :

2 alleles

99.2% missing data

3	(0.530) 0.529 0.535

1	(0.470) 0.471 0.465

Locus 9117 :

2 alleles

98.8% missing data

2	(0.181) 0.178 0.184

3	(0.819) 0.822 0.816

Locus 9118 :

2 alleles

99.2% missing data

3	(0.593) 0.593 0.589

2	(0.407) 0.407 0.411

Locus 9119 :

2 alleles

99.2% missing data

1	(0.559) 0.560 0.561

4	(0.441) 0.440 0.439

Locus 9120 :

2 alleles

98.8% missing data

4	(0.486) 0.493 0.487

1	(0.514) 0.507 0.513

Locus 9121 :

2 alleles

99.2% missing data

1	(0.673) 0.671 0.673

2	(0.327) 0.329 0.327

Locus 9122 :

2 alleles

90.6% missing data

4	(0.913) 0.927 0.904

3	(0.087) 0.073 0.096

Locus 9123 :

2 alleles

99.2% missing data

1	(0.454) 0.452 0.452

3	(0.546) 0.548 0.548

Locus 9124 :

2 alleles

99.2% missing data

3	(0.648) 0.649 0.649

1	(0.352) 0.351 0.351

Locus 9125 :

2 alleles

99.2% missing data

2	(0.162) 0.161 0.166

4	(0.838) 0.839 0.834

Locus 9126 :

2 alleles

8.2% missing data

2	(0.981) 0.975 0.994

4	(0.019) 0.025 0.006

Locus 9127 :

3 alleles

98.8% missing data

4	(0.303) 0.306 0.301

1	(0.101) 0.098 0.111

2	(0.596) 0.596 0.588

Locus 9128 :

2 alleles

99.2% missing data

2	(0.766) 0.764 0.761

4	(0.234) 0.236 0.239

Locus 9129 :

2 alleles

99.2% missing data

1	(0.206) 0.206 0.210

2	(0.794) 0.794 0.790

Locus 9130 :

2 alleles

94.1% missing data

4	(0.896) 0.901 0.898

1	(0.104) 0.099 0.102

Locus 9131 :

2 alleles

98.8% missing data

2	(0.392) 0.392 0.391

3	(0.608) 0.608 0.609

Locus 9132 :

2 alleles

99.2% missing data

1	(0.218) 0.218 0.228

4	(0.782) 0.782 0.772

Locus 9133 :

2 alleles

99.2% missing data

2	(0.737) 0.731 0.740

1	(0.263) 0.269 0.260

Locus 9134 :

2 alleles

96.9% missing data

2	(0.125) 0.121 0.130

3	(0.875) 0.879 0.870

Locus 9135 :

2 alleles

99.2% missing data

4	(0.338) 0.339 0.339

3	(0.662) 0.661 0.661

Locus 9136 :

2 alleles

98.8% missing data

1	(0.578) 0.579 0.578

2	(0.422) 0.421 0.422

Locus 9137 :

2 alleles

0.8% missing data

3	(0.986) 0.981 0.996

1	(0.014) 0.019 0.004

Locus 9138 :

2 alleles

98.8% missing data

1	(0.565) 0.568 0.564

3	(0.435) 0.432 0.436

Locus 9139 :

2 alleles

98.0% missing data

3	(0.809) 0.808 0.812

4	(0.191) 0.192 0.188

Locus 9140 :

2 alleles

99.2% missing data

1	(0.553) 0.554 0.544

4	(0.447) 0.446 0.456

Locus 9141 :

2 alleles

99.2% missing data

3	(0.238) 0.236 0.244

2	(0.762) 0.764 0.756

Locus 9142 :

2 alleles

99.2% missing data

2	(0.481) 0.473 0.488

1	(0.519) 0.527 0.512

Locus 9143 :

2 alleles

97.3% missing data

3	(0.795) 0.788 0.804

1	(0.205) 0.212 0.196

Locus 9144 :

3 alleles

96.5% missing data

3	(0.659) 0.672 0.656

1	(0.079) 0.072 0.085

2	(0.262) 0.255 0.260

Locus 9145 :

2 alleles

99.2% missing data

3	(0.650) 0.639 0.653

1	(0.350) 0.361 0.347

Locus 9146 :

2 alleles

99.2% missing data

3	(0.602) 0.593 0.607

2	(0.398) 0.407 0.393

Locus 9147 :

2 alleles

96.9% missing data

3	(0.830) 0.831 0.832

1	(0.170) 0.169 0.168

Locus 9148 :

2 alleles

99.2% missing data

2	(0.405) 0.410 0.405

4	(0.595) 0.590 0.595

Locus 9149 :

2 alleles

99.2% missing data

3	(0.541) 0.539 0.544

2	(0.459) 0.461 0.456

Locus 9150 :

2 alleles

99.2% missing data

4	(0.386) 0.384 0.386

3	(0.614) 0.616 0.614

Locus 9151 :

2 alleles

99.2% missing data

1	(0.558) 0.557 0.559

2	(0.442) 0.443 0.441

Locus 9152 :

2 alleles

99.2% missing data

1	(0.456) 0.457 0.456

4	(0.544) 0.543 0.544

Locus 9153 :

2 alleles

0.0% missing data

2	(0.985) 0.980 0.995

3	(0.015) 0.020 0.005

Locus 9154 :

2 alleles

99.2% missing data

2	(0.438) 0.435 0.442

1	(0.562) 0.565 0.558

Locus 9155 :

2 alleles

99.2% missing data

2	(0.201) 0.199 0.205

4	(0.799) 0.801 0.795

Locus 9156 :

2 alleles

99.2% missing data

1	(0.525) 0.519 0.531

4	(0.475) 0.481 0.469

Locus 9157 :

2 alleles

99.2% missing data

3	(0.379) 0.385 0.375

1	(0.621) 0.615 0.625

Locus 9158 :

2 alleles

99.2% missing data

3	(0.744) 0.744 0.740

4	(0.256) 0.256 0.260

Locus 9159 :

2 alleles

0.0% missing data

2	(0.987) 0.982 0.997

3	(0.013) 0.018 0.003

Locus 9160 :

2 alleles

98.8% missing data

4	(0.622) 0.615 0.633

1	(0.378) 0.385 0.367

Locus 9161 :

2 alleles

99.2% missing data

2	(0.574) 0.572 0.575

3	(0.426) 0.428 0.425

Locus 9162 :

2 alleles

99.2% missing data

2	(0.685) 0.678 0.688

3	(0.315) 0.322 0.312

Locus 9163 :

2 alleles

0.0% missing data

2	(0.984) 0.984 0.993

3	(0.016) 0.016 0.007

Locus 9164 :

3 alleles

98.8% missing data

3	(0.367) 0.368 0.367

1	(0.230) 0.225 0.234

4	(0.403) 0.406 0.398

Locus 9165 :

2 alleles

99.2% missing data

2	(0.808) 0.802 0.809

1	(0.192) 0.198 0.191

Locus 9166 :

2 alleles

98.8% missing data

2	(0.771) 0.772 0.771

4	(0.229) 0.228 0.229

Locus 9167 :

2 alleles

99.2% missing data

3	(0.783) 0.789 0.778

2	(0.217) 0.211 0.222

Locus 9168 :

2 alleles

0.0% missing data

4	(0.983) 0.989 0.992

1	(0.017) 0.011 0.008

Locus 9169 :

2 alleles

98.8% missing data

1	(0.516) 0.513 0.521

3	(0.484) 0.487 0.479

Locus 9170 :

2 alleles

99.2% missing data

4	(0.319) 0.312 0.328

1	(0.681) 0.688 0.672

Locus 9171 :

3 alleles

98.8% missing data

1	(0.405) 0.403 0.410

4	(0.292) 0.288 0.294

3	(0.303) 0.309 0.296

Locus 9172 :

2 alleles

98.8% missing data

3	(0.472) 0.477 0.468

4	(0.528) 0.523 0.532

Locus 9173 :

2 alleles

99.2% missing data

1	(0.794) 0.796 0.794

2	(0.206) 0.204 0.206

Locus 9174 :

2 alleles

99.2% missing data

3	(0.289) 0.289 0.296

1	(0.711) 0.711 0.704

Locus 9175 :

2 alleles

99.2% missing data

3	(0.575) 0.569 0.574

4	(0.425) 0.431 0.426

Locus 9176 :

2 alleles

98.8% missing data

1	(0.607) 0.615 0.604

3	(0.393) 0.385 0.396

Locus 9177 :

2 alleles

99.2% missing data

3	(0.221) 0.224 0.219

1	(0.779) 0.776 0.781

Locus 9178 :

3 alleles

98.4% missing data

4	(0.513) 0.513 0.502

1	(0.253) 0.251 0.262

2	(0.234) 0.236 0.236

Locus 9179 :

2 alleles

98.4% missing data

1	(0.656) 0.655 0.654

4	(0.344) 0.345 0.346

Locus 9180 :

2 alleles

41.8% missing data

1	(0.984) 0.977 0.994

3	(0.016) 0.023 0.006

Locus 9181 :

2 alleles

99.2% missing data

2	(0.760) 0.762 0.753

3	(0.240) 0.238 0.247

Locus 9182 :

2 alleles

99.2% missing data

1	(0.671) 0.673 0.666

3	(0.329) 0.327 0.334

Locus 9183 :

2 alleles

98.8% missing data

2	(0.686) 0.691 0.683

4	(0.314) 0.309 0.317

Locus 9184 :

2 alleles

99.2% missing data

4	(0.611) 0.615 0.604

1	(0.389) 0.385 0.396

Locus 9185 :

2 alleles

99.2% missing data

3	(0.357) 0.357 0.359

2	(0.643) 0.643 0.641

Locus 9186 :

2 alleles

87.9% missing data

4	(0.675) 0.695 0.668

2	(0.325) 0.305 0.332

Locus 9187 :

2 alleles

99.2% missing data

1	(0.494) 0.496 0.492

3	(0.506) 0.504 0.508

Locus 9188 :

2 alleles

98.8% missing data

4	(0.479) 0.472 0.490

3	(0.521) 0.528 0.510

Locus 9189 :

2 alleles

99.2% missing data

1	(0.716) 0.705 0.725

3	(0.284) 0.295 0.275

Locus 9190 :

3 alleles

94.1% missing data

4	(0.298) 0.303 0.297

1	(0.426) 0.424 0.440

2	(0.277) 0.274 0.264

Locus 9191 :

2 alleles

97.3% missing data

1	(0.825) 0.819 0.827

2	(0.175) 0.181 0.173

Locus 9192 :

2 alleles

8.2% missing data

1	(0.982) 0.968 0.995

2	(0.018) 0.032 0.005

Locus 9193 :

3 alleles

96.9% missing data

1	(0.429) 0.427 0.443

2	(0.166) 0.162 0.165

4	(0.405) 0.411 0.393

Locus 9194 :

2 alleles

98.0% missing data

2	(0.310) 0.308 0.309

1	(0.690) 0.692 0.691

Locus 9195 :

2 alleles

98.0% missing data

1	(0.828) 0.832 0.824

2	(0.172) 0.168 0.176

Locus 9196 :

2 alleles

98.8% missing data

1	(0.570) 0.567 0.575

3	(0.430) 0.433 0.425

Locus 9197 :

3 alleles

98.0% missing data

2	(0.556) 0.557 0.557

1	(0.252) 0.251 0.249

4	(0.192) 0.193 0.194

Locus 9198 :

2 alleles

91.0% missing data

2	(0.896) 0.910 0.895

3	(0.104) 0.090 0.105

Locus 9199 :

2 alleles

99.2% missing data

4	(0.573) 0.561 0.576

1	(0.427) 0.439 0.424

Locus 9200 :

3 alleles

98.8% missing data

1	(0.324) 0.315 0.334

4	(0.320) 0.324 0.317

2	(0.355) 0.360 0.348

Locus 9201 :

3 alleles

98.8% missing data

3	(0.282) 0.283 0.280

4	(0.449) 0.455 0.445

1	(0.269) 0.262 0.275

Locus 9202 :

2 alleles

99.2% missing data

4	(0.655) 0.656 0.653

1	(0.345) 0.344 0.347

Locus 9203 :

2 alleles

96.9% missing data

1	(0.601) 0.609 0.599

2	(0.399) 0.391 0.401

Locus 9204 :

2 alleles

99.2% missing data

1	(0.616) 0.611 0.618

3	(0.384) 0.389 0.382

Locus 9205 :

3 alleles

97.3% missing data

4	(0.234) 0.233 0.232

1	(0.560) 0.563 0.562

3	(0.206) 0.204 0.206

Locus 9206 :

3 alleles

98.4% missing data

2	(0.244) 0.249 0.249

1	(0.294) 0.287 0.295

3	(0.461) 0.464 0.455

Locus 9207 :

2 alleles

98.0% missing data

2	(0.442) 0.447 0.436

1	(0.558) 0.553 0.564

Locus 9208 :

2 alleles

98.8% missing data

1	(0.457) 0.452 0.468

2	(0.543) 0.548 0.532

Locus 9209 :

2 alleles

98.8% missing data

2	(0.416) 0.420 0.417

1	(0.584) 0.580 0.583

Locus 9210 :

2 alleles

99.2% missing data

2	(0.477) 0.478 0.477

3	(0.523) 0.522 0.523

Locus 9211 :

2 alleles

99.2% missing data

4	(0.521) 0.521 0.521

1	(0.479) 0.479 0.479

Locus 9212 :

2 alleles

99.2% missing data

3	(0.340) 0.335 0.340

4	(0.660) 0.665 0.660

Locus 9213 :

2 alleles

99.2% missing data

2	(0.163) 0.160 0.173

1	(0.837) 0.840 0.827

Locus 9214 :

2 alleles

99.2% missing data

2	(0.207) 0.208 0.202

1	(0.793) 0.792 0.798

Locus 9215 :

2 alleles

99.2% missing data

4	(0.774) 0.767 0.777

3	(0.226) 0.233 0.223

Locus 9216 :

2 alleles

98.4% missing data

3	(0.637) 0.643 0.638

2	(0.363) 0.357 0.362

Locus 9217 :

2 alleles

96.5% missing data

4	(0.871) 0.866 0.875

2	(0.129) 0.134 0.125

Locus 9218 :

2 alleles

99.2% missing data

3	(0.312) 0.311 0.311

2	(0.688) 0.689 0.689

Locus 9219 :

2 alleles

99.2% missing data

4	(0.491) 0.491 0.497

3	(0.509) 0.509 0.503

Locus 9220 :

2 alleles

99.2% missing data

1	(0.555) 0.546 0.556

3	(0.445) 0.454 0.444

Locus 9221 :

2 alleles

99.2% missing data

4	(0.822) 0.826 0.814

2	(0.178) 0.174 0.186

Locus 9222 :

2 alleles

98.8% missing data

4	(0.635) 0.640 0.633

1	(0.365) 0.360 0.367

Locus 9223 :

2 alleles

98.4% missing data

3	(0.351) 0.350 0.351

2	(0.649) 0.650 0.649

Locus 9224 :

2 alleles

98.0% missing data

2	(0.695) 0.684 0.707

3	(0.305) 0.316 0.293

Locus 9225 :

2 alleles

99.2% missing data

1	(0.625) 0.615 0.626

2	(0.375) 0.385 0.374

Locus 9226 :

2 alleles

99.2% missing data

3	(0.687) 0.680 0.689

1	(0.313) 0.320 0.311

Locus 9227 :

2 alleles

99.2% missing data

2	(0.471) 0.472 0.472

3	(0.529) 0.528 0.528

Locus 9228 :

2 alleles

99.2% missing data

1	(0.548) 0.548 0.546

3	(0.452) 0.452 0.454

Locus 9229 :

2 alleles

97.3% missing data

4	(0.777) 0.775 0.779

3	(0.223) 0.225 0.221

Locus 9230 :

2 alleles

98.4% missing data

4	(0.850) 0.855 0.843

2	(0.150) 0.145 0.157

Locus 9231 :

3 alleles

98.8% missing data

2	(0.361) 0.371 0.354

4	(0.269) 0.268 0.270

1	(0.369) 0.361 0.376

Locus 9232 :

2 alleles

90.6% missing data

1	(0.732) 0.725 0.740

2	(0.268) 0.275 0.260

Locus 9233 :

2 alleles

96.1% missing data

1	(0.527) 0.517 0.536

2	(0.473) 0.483 0.464

Locus 9234 :

2 alleles

99.2% missing data

2	(0.757) 0.748 0.760

3	(0.243) 0.252 0.240

Locus 9235 :

3 alleles

96.9% missing data

1	(0.500) 0.495 0.521

2	(0.259) 0.260 0.246

4	(0.241) 0.245 0.233

Locus 9236 :

2 alleles

99.2% missing data

2	(0.690) 0.690 0.689

4	(0.310) 0.310 0.311

Locus 9237 :

2 alleles

98.4% missing data

1	(0.713) 0.717 0.713

2	(0.287) 0.283 0.287

Locus 9238 :

2 alleles

98.8% missing data

2	(0.738) 0.732 0.746

1	(0.262) 0.268 0.254

Locus 9239 :

2 alleles

99.2% missing data

2	(0.638) 0.631 0.646

1	(0.362) 0.369 0.354

Locus 9240 :

2 alleles

96.1% missing data

2	(0.259) 0.254 0.268

1	(0.741) 0.746 0.732

Locus 9241 :

3 alleles

97.7% missing data

2	(0.355) 0.357 0.354

1	(0.451) 0.455 0.448

3	(0.195) 0.189 0.198

Locus 9242 :

2 alleles

99.2% missing data

1	(0.637) 0.638 0.636

2	(0.363) 0.362 0.364

Locus 9243 :

2 alleles

99.2% missing data

2	(0.228) 0.231 0.228

1	(0.772) 0.769 0.772

Locus 9244 :

2 alleles

99.2% missing data

2	(0.472) 0.471 0.473

4	(0.528) 0.529 0.527

Locus 9245 :

2 alleles

98.8% missing data

4	(0.733) 0.727 0.743

3	(0.267) 0.273 0.257

Locus 9246 :

2 alleles

96.5% missing data

1	(0.847) 0.851 0.849

3	(0.153) 0.149 0.151

Locus 9247 :

2 alleles

97.7% missing data

1	(0.632) 0.631 0.638

2	(0.368) 0.369 0.362

Locus 9248 :

2 alleles

97.7% missing data

1	(0.521) 0.519 0.526

4	(0.479) 0.481 0.474

Locus 9249 :

2 alleles

99.2% missing data

2	(0.283) 0.279 0.289

3	(0.717) 0.721 0.711

Locus 9250 :

3 alleles

98.0% missing data

4	(0.450) 0.446 0.454

2	(0.291) 0.284 0.298

1	(0.259) 0.270 0.248

Locus 9251 :

2 alleles

99.2% missing data

1	(0.642) 0.641 0.647

3	(0.358) 0.359 0.353

Locus 9252 :

2 alleles

99.2% missing data

4	(0.437) 0.442 0.431

2	(0.563) 0.558 0.569

Locus 9253 :

2 alleles

98.4% missing data

2	(0.456) 0.459 0.461

1	(0.544) 0.541 0.539

Locus 9254 :

2 alleles

98.4% missing data

1	(0.630) 0.632 0.635

3	(0.370) 0.368 0.365

Locus 9255 :

2 alleles

99.2% missing data

2	(0.612) 0.608 0.612

1	(0.388) 0.392 0.388

Locus 9256 :

2 alleles

99.2% missing data

2	(0.301) 0.300 0.309

1	(0.699) 0.700 0.691

Locus 9257 :

2 alleles

97.3% missing data

2	(0.706) 0.714 0.705

1	(0.294) 0.286 0.295

Locus 9258 :

2 alleles

99.2% missing data

1	(0.273) 0.268 0.278

4	(0.727) 0.732 0.722

Locus 9259 :

2 alleles

98.4% missing data

2	(0.725) 0.728 0.732

1	(0.275) 0.272 0.268

Locus 9260 :

2 alleles

98.8% missing data

3	(0.302) 0.302 0.302

4	(0.698) 0.698 0.698

Locus 9261 :

2 alleles

99.2% missing data

1	(0.360) 0.351 0.360

2	(0.640) 0.649 0.640

Locus 9262 :

2 alleles

99.2% missing data

1	(0.297) 0.288 0.301

2	(0.703) 0.712 0.699

Locus 9263 :

2 alleles

98.8% missing data

3	(0.622) 0.618 0.626

4	(0.378) 0.382 0.374

Locus 9264 :

2 alleles

99.2% missing data

3	(0.457) 0.465 0.451

1	(0.543) 0.535 0.549

Locus 9265 :

2 alleles

99.2% missing data

3	(0.384) 0.381 0.381

1	(0.616) 0.619 0.619

Locus 9266 :

2 alleles

99.2% missing data

3	(0.536) 0.529 0.541

1	(0.464) 0.471 0.459

Locus 9267 :

2 alleles

99.2% missing data

1	(0.332) 0.327 0.344

2	(0.668) 0.673 0.656

Locus 9268 :

2 alleles

98.4% missing data

2	(0.666) 0.671 0.661

3	(0.334) 0.329 0.339

Locus 9269 :

2 alleles

99.2% missing data

1	(0.520) 0.517 0.527

4	(0.480) 0.483 0.473

Locus 9270 :

2 alleles

99.2% missing data

2	(0.710) 0.707 0.711

3	(0.290) 0.293 0.289

Locus 9271 :

2 alleles

99.2% missing data

3	(0.680) 0.677 0.678

1	(0.320) 0.323 0.322

Locus 9272 :

2 alleles

91.0% missing data

1	(0.927) 0.933 0.923

3	(0.073) 0.067 0.077

Locus 9273 :

2 alleles

99.2% missing data

1	(0.483) 0.484 0.480

3	(0.517) 0.516 0.520

Locus 9274 :

2 alleles

99.2% missing data

3	(0.342) 0.333 0.349

4	(0.658) 0.667 0.651

Locus 9275 :

2 alleles

99.2% missing data

4	(0.374) 0.368 0.377

3	(0.626) 0.632 0.623

Locus 9276 :

2 alleles

99.2% missing data

1	(0.453) 0.441 0.457

3	(0.547) 0.559 0.543

Locus 9277 :

2 alleles

98.8% missing data

2	(0.640) 0.639 0.643

3	(0.360) 0.361 0.357

Locus 9278 :

2 alleles

99.2% missing data

3	(0.249) 0.255 0.241

1	(0.751) 0.745 0.759

Locus 9279 :

2 alleles

91.0% missing data

1	(0.924) 0.933 0.923

4	(0.076) 0.067 0.077

Locus 9280 :

2 alleles

99.2% missing data

3	(0.684) 0.681 0.689

1	(0.316) 0.319 0.311

Locus 9281 :

2 alleles

99.2% missing data

1	(0.313) 0.311 0.320

2	(0.687) 0.689 0.680

Locus 9282 :

2 alleles

99.2% missing data

2	(0.579) 0.580 0.571

1	(0.421) 0.420 0.429

Locus 9283 :

2 alleles

97.3% missing data

4	(0.642) 0.632 0.662

3	(0.358) 0.368 0.338

Locus 9284 :

2 alleles

99.2% missing data

2	(0.549) 0.552 0.552

1	(0.451) 0.448 0.448

Locus 9285 :

2 alleles

99.2% missing data

1	(0.488) 0.479 0.493

3	(0.512) 0.521 0.507

Locus 9286 :

2 alleles

98.8% missing data

2	(0.641) 0.652 0.646

4	(0.359) 0.348 0.354

Locus 9287 :

2 alleles

99.2% missing data

4	(0.481) 0.486 0.478

2	(0.519) 0.514 0.522

Locus 9288 :

2 alleles

99.2% missing data

4	(0.744) 0.743 0.737

3	(0.256) 0.257 0.263

Locus 9289 :

2 alleles

99.2% missing data

1	(0.475) 0.479 0.470

2	(0.525) 0.521 0.530

Locus 9290 :

2 alleles

99.2% missing data

4	(0.609) 0.615 0.600

1	(0.391) 0.385 0.400

Locus 9291 :

2 alleles

96.1% missing data

2	(0.899) 0.900 0.902

3	(0.101) 0.100 0.098

Locus 9292 :

2 alleles

99.2% missing data

3	(0.454) 0.456 0.457

2	(0.546) 0.544 0.543

Locus 9293 :

3 alleles

98.8% missing data

3	(0.396) 0.389 0.400

2	(0.435) 0.433 0.439

4	(0.170) 0.178 0.161

Locus 9294 :

2 alleles

99.2% missing data

2	(0.685) 0.688 0.683

1	(0.315) 0.312 0.317

Locus 9295 :

2 alleles

96.9% missing data

1	(0.855) 0.859 0.858

2	(0.145) 0.141 0.142

Locus 9296 :

3 alleles

96.9% missing data

4	(0.255) 0.250 0.245

2	(0.397) 0.404 0.400

1	(0.348) 0.346 0.355

Locus 9297 :

2 alleles

97.3% missing data

2	(0.428) 0.415 0.442

1	(0.572) 0.585 0.558

Locus 9298 :

2 alleles

98.0% missing data

2	(0.637) 0.631 0.650

1	(0.363) 0.369 0.350

Locus 9299 :

2 alleles

96.5% missing data

4	(0.804) 0.812 0.800

1	(0.196) 0.188 0.200

Locus 9300 :

2 alleles

96.9% missing data

1	(0.715) 0.712 0.721

2	(0.285) 0.288 0.279

Locus 9301 :

2 alleles

98.0% missing data

1	(0.438) 0.440 0.439

2	(0.562) 0.560 0.561

Locus 9302 :

2 alleles

98.8% missing data

1	(0.861) 0.863 0.856

2	(0.139) 0.137 0.144

Locus 9303 :

2 alleles

97.7% missing data

2	(0.764) 0.775 0.755

1	(0.236) 0.225 0.245

Locus 9304 :

2 alleles

98.8% missing data

1	(0.420) 0.415 0.426

2	(0.580) 0.585 0.574

Locus 9305 :

2 alleles

98.0% missing data

1	(0.731) 0.721 0.739

2	(0.269) 0.279 0.261

Locus 9306 :

3 alleles

98.0% missing data

3	(0.174) 0.180 0.166

2	(0.297) 0.288 0.304

1	(0.529) 0.531 0.531

Locus 9307 :

3 alleles

97.7% missing data

1	(0.594) 0.594 0.591

4	(0.209) 0.202 0.218

3	(0.197) 0.204 0.191

Locus 9308 :

2 alleles

98.8% missing data

1	(0.774) 0.771 0.776

2	(0.226) 0.229 0.224

Locus 9309 :

2 alleles

99.2% missing data

2	(0.594) 0.594 0.586

1	(0.406) 0.406 0.414

Locus 9310 :

2 alleles

98.0% missing data

2	(0.640) 0.640 0.634

1	(0.360) 0.360 0.366

Locus 9311 :

3 alleles

98.4% missing data

2	(0.459) 0.453 0.463

3	(0.222) 0.221 0.233

1	(0.319) 0.325 0.304

Locus 9312 :

3 alleles

98.0% missing data

1	(0.633) 0.621 0.636

4	(0.135) 0.143 0.130

2	(0.232) 0.236 0.234

Locus 9313 :

2 alleles

99.2% missing data

2	(0.752) 0.752 0.747

1	(0.248) 0.248 0.253

Locus 9314 :

2 alleles

99.2% missing data

4	(0.521) 0.521 0.520

1	(0.479) 0.479 0.480

Locus 9315 :

2 alleles

99.2% missing data

1	(0.509) 0.509 0.512

4	(0.491) 0.491 0.488

Locus 9316 :

2 alleles

99.2% missing data

1	(0.722) 0.722 0.719

2	(0.278) 0.278 0.281

Locus 9317 :

2 alleles

8.2% missing data

2	(0.984) 0.977 0.995

1	(0.016) 0.023 0.005

Locus 9318 :

2 alleles

99.2% missing data

1	(0.279) 0.276 0.285

4	(0.721) 0.724 0.715

Locus 9319 :

2 alleles

91.0% missing data

3	(0.932) 0.942 0.926

1	(0.068) 0.058 0.074

Locus 9320 :

2 alleles

8.2% missing data

2	(0.983) 0.973 0.995

3	(0.017) 0.027 0.005

Locus 9321 :

2 alleles

99.2% missing data

2	(0.807) 0.806 0.804

3	(0.193) 0.194 0.196

Locus 9322 :

2 alleles

99.2% missing data

4	(0.370) 0.373 0.370

2	(0.630) 0.627 0.630

Locus 9323 :

2 alleles

99.2% missing data

1	(0.490) 0.494 0.490

4	(0.510) 0.506 0.510

Locus 9324 :

2 alleles

99.2% missing data

3	(0.465) 0.460 0.464

4	(0.535) 0.540 0.536

Locus 9325 :

2 alleles

99.2% missing data

1	(0.749) 0.752 0.745

2	(0.251) 0.248 0.255

Locus 9326 :

2 alleles

98.8% missing data

2	(0.777) 0.779 0.776

1	(0.223) 0.221 0.224

Locus 9327 :

2 alleles

98.4% missing data

2	(0.250) 0.254 0.244

1	(0.750) 0.746 0.756

Locus 9328 :

2 alleles

97.3% missing data

1	(0.223) 0.239 0.210

3	(0.777) 0.761 0.790

Locus 9329 :

2 alleles

98.4% missing data

1	(0.791) 0.795 0.785

2	(0.209) 0.205 0.215

Locus 9330 :

3 alleles

98.0% missing data

4	(0.362) 0.356 0.362

1	(0.383) 0.392 0.384

2	(0.255) 0.252 0.254

Locus 9331 :

2 alleles

99.2% missing data

3	(0.559) 0.555 0.567

2	(0.441) 0.445 0.433

Locus 9332 :

2 alleles

98.8% missing data

2	(0.489) 0.478 0.498

4	(0.511) 0.522 0.502

Locus 9333 :

2 alleles

98.8% missing data

4	(0.601) 0.603 0.601

3	(0.399) 0.397 0.399

Locus 9334 :

2 alleles

96.5% missing data

1	(0.465) 0.472 0.465

2	(0.535) 0.528 0.535

Locus 9335 :

2 alleles

97.7% missing data

3	(0.344) 0.350 0.342

1	(0.656) 0.650 0.658

Locus 9336 :

2 alleles

98.8% missing data

1	(0.634) 0.640 0.637

4	(0.366) 0.360 0.363

Locus 9337 :

4 alleles

98.0% missing data

2	(0.278) 0.273 0.290

1	(0.246) 0.243 0.242

4	(0.163) 0.158 0.168

3	(0.313) 0.326 0.300

Locus 9338 :

2 alleles

99.2% missing data

3	(0.776) 0.767 0.786

1	(0.224) 0.233 0.214

Locus 9339 :

2 alleles

97.7% missing data

4	(0.203) 0.190 0.214

1	(0.797) 0.810 0.786

Locus 9340 :

3 alleles

98.0% missing data

2	(0.332) 0.336 0.325

1	(0.346) 0.345 0.350

4	(0.322) 0.318 0.324

Locus 9341 :

3 alleles

97.7% missing data

1	(0.403) 0.400 0.412

2	(0.300) 0.304 0.302

3	(0.297) 0.295 0.286

Locus 9342 :

2 alleles

98.8% missing data

2	(0.730) 0.734 0.717

1	(0.270) 0.266 0.283

Locus 9343 :

2 alleles

99.2% missing data

4	(0.614) 0.616 0.618

1	(0.386) 0.384 0.382

Locus 9344 :

2 alleles

98.4% missing data

4	(0.670) 0.667 0.669

1	(0.330) 0.333 0.331

Locus 9345 :

2 alleles

98.4% missing data

1	(0.573) 0.576 0.579

2	(0.427) 0.424 0.421

Locus 9346 :

2 alleles

98.4% missing data

2	(0.697) 0.701 0.699

1	(0.303) 0.299 0.301

Locus 9347 :

3 alleles

87.1% missing data

4	(0.570) 0.637 0.497

2	(0.297) 0.249 0.352

1	(0.133) 0.114 0.151

Locus 9348 :

2 alleles

99.2% missing data

4	(0.454) 0.459 0.451

3	(0.546) 0.541 0.549

Locus 9349 :

2 alleles

99.2% missing data

1	(0.606) 0.610 0.602

3	(0.394) 0.390 0.398

Locus 9350 :

2 alleles

99.2% missing data

4	(0.319) 0.314 0.323

2	(0.681) 0.686 0.677

Locus 9351 :

2 alleles

99.2% missing data

3	(0.624) 0.618 0.629

1	(0.376) 0.382 0.371

Locus 9352 :

3 alleles

95.3% missing data

3	(0.122) 0.119 0.124

4	(0.674) 0.690 0.668

1	(0.204) 0.191 0.208

Locus 9353 :

2 alleles

0.4% missing data

4	(0.986) 0.982 0.996

2	(0.014) 0.018 0.004

Locus 9354 :

2 alleles

99.2% missing data

3	(0.292) 0.288 0.294

1	(0.708) 0.712 0.706

Locus 9355 :

3 alleles

98.8% missing data

2	(0.340) 0.336 0.346

3	(0.336) 0.345 0.328

4	(0.325) 0.319 0.326

Locus 9356 :

2 alleles

99.2% missing data

4	(0.602) 0.595 0.606

2	(0.398) 0.405 0.394

Locus 9357 :

2 alleles

99.2% missing data

4	(0.366) 0.370 0.360

2	(0.634) 0.630 0.640

Locus 9358 :

2 alleles

99.2% missing data

3	(0.401) 0.398 0.403

2	(0.599) 0.602 0.597

Locus 9359 :

2 alleles

99.2% missing data

3	(0.462) 0.464 0.458

2	(0.538) 0.536 0.542

Locus 9360 :

2 alleles

93.4% missing data

3	(0.216) 0.215 0.222

2	(0.784) 0.785 0.778

Locus 9361 :

2 alleles

99.2% missing data

2	(0.555) 0.554 0.558

1	(0.445) 0.446 0.442

Locus 9362 :

2 alleles

99.2% missing data

3	(0.610) 0.617 0.607

2	(0.390) 0.383 0.393

Locus 9363 :

2 alleles

98.8% missing data

1	(0.441) 0.444 0.433

3	(0.559) 0.556 0.567

Locus 9364 :

2 alleles

91.0% missing data

3	(0.910) 0.928 0.903

1	(0.090) 0.072 0.097

Locus 9365 :

3 alleles

96.1% missing data

4	(0.356) 0.349 0.362

1	(0.539) 0.545 0.535

2	(0.106) 0.106 0.104

Locus 9366 :

2 alleles

99.2% missing data

2	(0.709) 0.708 0.712

3	(0.291) 0.292 0.288

Locus 9367 :

2 alleles

98.8% missing data

4	(0.424) 0.415 0.437

3	(0.576) 0.585 0.563

Locus 9368 :

2 alleles

98.8% missing data

2	(0.569) 0.575 0.566

1	(0.431) 0.425 0.434

Locus 9369 :

2 alleles

97.7% missing data

2	(0.251) 0.254 0.250

1	(0.749) 0.746 0.750

Locus 9370 :

2 alleles

87.1% missing data

2	(0.948) 0.951 0.953

1	(0.052) 0.049 0.047

Locus 9371 :

2 alleles

99.2% missing data

1	(0.560) 0.561 0.559

2	(0.440) 0.439 0.441

Locus 9372 :

2 alleles

99.2% missing data

1	(0.439) 0.435 0.436

2	(0.561) 0.565 0.564

Locus 9373 :

2 alleles

99.2% missing data

2	(0.626) 0.632 0.618

1	(0.374) 0.368 0.382

Locus 9374 :

2 alleles

99.2% missing data

1	(0.447) 0.444 0.455

2	(0.553) 0.556 0.545

Locus 9375 :

2 alleles

98.8% missing data

3	(0.662) 0.662 0.657

2	(0.338) 0.338 0.343

Locus 9376 :

2 alleles

99.2% missing data

4	(0.422) 0.422 0.420

3	(0.578) 0.578 0.580

Locus 9377 :

2 alleles

98.8% missing data

1	(0.534) 0.528 0.540

3	(0.466) 0.472 0.460

Locus 9378 :

2 alleles

97.3% missing data

2	(0.863) 0.866 0.863

1	(0.137) 0.134 0.137

Locus 9379 :

2 alleles

99.2% missing data

1	(0.434) 0.430 0.428

4	(0.566) 0.570 0.572

Locus 9380 :

2 alleles

99.2% missing data

4	(0.386) 0.379 0.394

2	(0.614) 0.621 0.606

Locus 9381 :

2 alleles

96.5% missing data

4	(0.439) 0.459 0.418

2	(0.561) 0.541 0.582

Locus 9382 :

2 alleles

98.8% missing data

3	(0.548) 0.536 0.549

2	(0.452) 0.464 0.451

Locus 9383 :

2 alleles

97.7% missing data

4	(0.605) 0.597 0.622

1	(0.395) 0.403 0.378

Locus 9384 :

2 alleles

98.0% missing data

3	(0.651) 0.648 0.657

2	(0.349) 0.352 0.343

Locus 9385 :

2 alleles

99.2% missing data

1	(0.807) 0.809 0.802

2	(0.193) 0.191 0.198

Locus 9386 :

2 alleles

92.2% missing data

2	(0.885) 0.903 0.878

1	(0.115) 0.097 0.122

Locus 9387 :

2 alleles

9.8% missing data

1	(0.987) 0.980 0.996

4	(0.013) 0.020 0.004

Locus 9388 :

2 alleles

98.4% missing data

3	(0.189) 0.185 0.192

2	(0.811) 0.815 0.808

Locus 9389 :

2 alleles

99.2% missing data

1	(0.296) 0.291 0.300

2	(0.704) 0.709 0.700

Locus 9390 :

2 alleles

99.2% missing data

3	(0.713) 0.709 0.715

1	(0.287) 0.291 0.285

Locus 9391 :

2 alleles

99.2% missing data

1	(0.216) 0.227 0.217

4	(0.784) 0.773 0.783

Locus 9392 :

2 alleles

99.2% missing data

1	(0.550) 0.536 0.562

3	(0.450) 0.464 0.438

Locus 9393 :

2 alleles

99.2% missing data

3	(0.512) 0.506 0.517

2	(0.488) 0.494 0.483

Locus 9394 :

2 alleles

8.2% missing data

2	(0.984) 0.976 0.995

3	(0.016) 0.024 0.005

Locus 9395 :

2 alleles

0.0% missing data

1	(0.987) 0.984 0.997

4	(0.013) 0.016 0.003

Locus 9396 :

2 alleles

99.2% missing data

3	(0.749) 0.741 0.751

4	(0.251) 0.259 0.249

Locus 9397 :

2 alleles

98.8% missing data

4	(0.554) 0.551 0.552

1	(0.446) 0.449 0.448

Locus 9398 :

2 alleles

97.7% missing data

1	(0.556) 0.546 0.565

3	(0.444) 0.454 0.435

Locus 9399 :

2 alleles

99.2% missing data

3	(0.502) 0.501 0.510

1	(0.498) 0.499 0.490

Locus 9400 :

2 alleles

99.2% missing data

4	(0.445) 0.447 0.445

2	(0.555) 0.553 0.555

Locus 9401 :

2 alleles

99.2% missing data

1	(0.492) 0.503 0.490

2	(0.508) 0.497 0.510

Locus 9402 :

2 alleles

96.1% missing data

3	(0.802) 0.809 0.804

1	(0.198) 0.191 0.196

Locus 9403 :

2 alleles

98.0% missing data

2	(0.756) 0.764 0.744

4	(0.244) 0.236 0.256

Locus 9404 :

2 alleles

99.2% missing data

2	(0.568) 0.564 0.572

3	(0.432) 0.436 0.428

Locus 9405 :

2 alleles

97.7% missing data

2	(0.854) 0.845 0.868

4	(0.146) 0.155 0.132

Locus 9406 :

2 alleles

96.9% missing data

2	(0.791) 0.798 0.793

4	(0.209) 0.202 0.207

Locus 9407 :

3 alleles

98.8% missing data

2	(0.314) 0.312 0.325

1	(0.344) 0.349 0.339

4	(0.342) 0.339 0.335

Locus 9408 :

2 alleles

99.2% missing data

4	(0.530) 0.531 0.525

1	(0.470) 0.469 0.475

Locus 9409 :

2 alleles

96.1% missing data

2	(0.846) 0.859 0.836

3	(0.154) 0.141 0.164

Locus 9410 :

2 alleles

95.7% missing data

2	(0.814) 0.829 0.812

3	(0.186) 0.171 0.188

Locus 9411 :

2 alleles

97.7% missing data

3	(0.355) 0.359 0.347

4	(0.645) 0.641 0.653

Locus 9412 :

3 alleles

98.8% missing data

1	(0.319) 0.317 0.322

3	(0.242) 0.246 0.241

2	(0.440) 0.437 0.437

Locus 9413 :

2 alleles

90.6% missing data

1	(0.918) 0.934 0.906

2	(0.082) 0.066 0.094

Locus 9414 :

2 alleles

95.7% missing data

1	(0.799) 0.796 0.799

2	(0.201) 0.204 0.201

Locus 9415 :

2 alleles

99.2% missing data

1	(0.580) 0.579 0.577

3	(0.420) 0.421 0.423

Locus 9416 :

3 alleles

98.8% missing data

3	(0.319) 0.321 0.319

1	(0.294) 0.293 0.292

2	(0.386) 0.386 0.389

Locus 9417 :

2 alleles

98.4% missing data

3	(0.711) 0.717 0.705

1	(0.289) 0.283 0.295

Locus 9418 :

2 alleles

98.8% missing data

3	(0.759) 0.760 0.758

2	(0.241) 0.240 0.242

Locus 9419 :

2 alleles

99.2% missing data

3	(0.452) 0.455 0.451

4	(0.548) 0.545 0.549

Locus 9420 :

2 alleles

97.7% missing data

2	(0.383) 0.385 0.372

1	(0.617) 0.615 0.628

Locus 9421 :

2 alleles

99.2% missing data

1	(0.504) 0.509 0.497

2	(0.496) 0.491 0.503

Locus 9422 :

2 alleles

99.2% missing data

1	(0.709) 0.712 0.704

3	(0.291) 0.288 0.296

Locus 9423 :

2 alleles

99.2% missing data

4	(0.481) 0.478 0.489

1	(0.519) 0.522 0.511

Locus 9424 :

2 alleles

99.2% missing data

2	(0.758) 0.758 0.753

1	(0.242) 0.242 0.247

Locus 9425 :

2 alleles

99.2% missing data

1	(0.502) 0.498 0.502

4	(0.498) 0.502 0.498

Locus 9426 :

2 alleles

90.2% missing data

3	(0.905) 0.924 0.899

1	(0.095) 0.076 0.101

Locus 9427 :

2 alleles

99.2% missing data

4	(0.310) 0.307 0.315

2	(0.690) 0.693 0.685

Locus 9428 :

2 alleles

96.1% missing data

1	(0.801) 0.802 0.801

4	(0.199) 0.198 0.199

Locus 9429 :

3 alleles

97.7% missing data

2	(0.414) 0.413 0.423

4	(0.231) 0.228 0.233

1	(0.355) 0.359 0.345

Locus 9430 :

2 alleles

96.9% missing data

2	(0.280) 0.295 0.272

1	(0.720) 0.705 0.728

Locus 9431 :

2 alleles

99.2% missing data

1	(0.698) 0.697 0.698

3	(0.302) 0.303 0.302

Locus 9432 :

2 alleles

98.8% missing data

2	(0.739) 0.738 0.741

3	(0.261) 0.262 0.259

Locus 9433 :

2 alleles

91.4% missing data

2	(0.913) 0.921 0.913

3	(0.087) 0.079 0.087

Locus 9434 :

2 alleles

98.4% missing data

1	(0.643) 0.650 0.642

4	(0.357) 0.350 0.358

Locus 9435 :

2 alleles

99.2% missing data

4	(0.158) 0.158 0.161

2	(0.842) 0.842 0.839

Locus 9436 :

2 alleles

98.8% missing data

2	(0.592) 0.598 0.594

4	(0.408) 0.402 0.406

Locus 9437 :

2 alleles

98.8% missing data

1	(0.777) 0.779 0.776

4	(0.223) 0.221 0.224

Locus 9438 :

2 alleles

99.2% missing data

2	(0.288) 0.287 0.294

1	(0.712) 0.713 0.706

Locus 9439 :

2 alleles

98.8% missing data

1	(0.800) 0.810 0.787

3	(0.200) 0.190 0.213

Locus 9440 :

4 alleles

93.0% missing data

2	(0.252) 0.236 0.264

1	(0.122) 0.112 0.130

4	(0.371) 0.400 0.356

3	(0.255) 0.252 0.251

Locus 9441 :

2 alleles

99.2% missing data

2	(0.614) 0.608 0.622

1	(0.386) 0.392 0.378

Locus 9442 :

2 alleles

99.2% missing data

1	(0.632) 0.638 0.628

3	(0.368) 0.362 0.372

Locus 9443 :

2 alleles

96.1% missing data

4	(0.858) 0.859 0.863

2	(0.142) 0.141 0.137

Locus 9444 :

2 alleles

99.2% missing data

2	(0.687) 0.683 0.689

4	(0.313) 0.317 0.311

Locus 9445 :

2 alleles

99.2% missing data

4	(0.386) 0.383 0.392

1	(0.614) 0.617 0.608

Locus 9446 :

2 alleles

97.7% missing data

3	(0.145) 0.149 0.141

2	(0.855) 0.851 0.859

Locus 9447 :

2 alleles

87.5% missing data

4	(0.710) 0.724 0.692

3	(0.290) 0.276 0.308

Locus 9448 :

2 alleles

99.2% missing data

1	(0.633) 0.634 0.625

3	(0.367) 0.366 0.375

Locus 9449 :

2 alleles

98.4% missing data

4	(0.429) 0.423 0.432

2	(0.571) 0.577 0.568

Locus 9450 :

2 alleles

98.8% missing data

3	(0.631) 0.632 0.636

4	(0.369) 0.368 0.364

Locus 9451 :

2 alleles

99.2% missing data

3	(0.401) 0.403 0.404

4	(0.599) 0.597 0.596

Locus 9452 :

2 alleles

99.2% missing data

4	(0.512) 0.509 0.517

2	(0.488) 0.491 0.483

Locus 9453 :

2 alleles

99.2% missing data

4	(0.397) 0.400 0.401

3	(0.603) 0.600 0.599

Locus 9454 :

2 alleles

96.9% missing data

1	(0.770) 0.775 0.776

2	(0.230) 0.225 0.224

Locus 9455 :

2 alleles

98.4% missing data

1	(0.650) 0.660 0.645

4	(0.350) 0.340 0.355

Locus 9456 :

2 alleles

98.8% missing data

3	(0.584) 0.579 0.589

1	(0.416) 0.421 0.411

Locus 9457 :

2 alleles

99.2% missing data

4	(0.569) 0.571 0.565

1	(0.431) 0.429 0.435

Locus 9458 :

2 alleles

99.2% missing data

3	(0.600) 0.595 0.602

1	(0.400) 0.405 0.398

Locus 9459 :

2 alleles

99.2% missing data

1	(0.675) 0.672 0.682

2	(0.325) 0.328 0.318

Locus 9460 :

2 alleles

98.8% missing data

1	(0.545) 0.544 0.548

2	(0.455) 0.456 0.452

Locus 9461 :

2 alleles

99.2% missing data

4	(0.808) 0.809 0.803

3	(0.192) 0.191 0.197

Locus 9462 :

2 alleles

93.0% missing data

2	(0.879) 0.877 0.891

1	(0.121) 0.123 0.109

Locus 9463 :

2 alleles

98.8% missing data

1	(0.747) 0.743 0.750

2	(0.253) 0.257 0.250

Locus 9464 :

2 alleles

99.2% missing data

4	(0.772) 0.767 0.773

3	(0.228) 0.233 0.227

Locus 9465 :

2 alleles

97.7% missing data

1	(0.312) 0.312 0.309

2	(0.688) 0.688 0.691

Locus 9466 :

2 alleles

99.2% missing data

4	(0.462) 0.463 0.473

1	(0.538) 0.537 0.527

Locus 9467 :

2 alleles

0.0% missing data

2	(0.988) 0.987 0.995

3	(0.012) 0.013 0.005

Locus 9468 :

2 alleles

99.2% missing data

4	(0.637) 0.635 0.648

2	(0.363) 0.365 0.352

Locus 9469 :

2 alleles

95.7% missing data

2	(0.872) 0.878 0.878

3	(0.128) 0.122 0.122

Locus 9470 :

2 alleles

98.8% missing data

1	(0.309) 0.308 0.310

3	(0.691) 0.692 0.690

Locus 9471 :

2 alleles

90.6% missing data

3	(0.905) 0.921 0.897

1	(0.095) 0.079 0.103

Locus 9472 :

2 alleles

98.8% missing data

2	(0.370) 0.371 0.376

4	(0.630) 0.629 0.624

Locus 9473 :

2 alleles

99.2% missing data

2	(0.709) 0.706 0.709

4	(0.291) 0.294 0.291

Locus 9474 :

2 alleles

99.2% missing data

2	(0.320) 0.318 0.323

3	(0.680) 0.682 0.677

Locus 9475 :

2 alleles

99.2% missing data

1	(0.289) 0.292 0.287

2	(0.711) 0.708 0.713

Locus 9476 :

2 alleles

99.2% missing data

3	(0.568) 0.566 0.576

4	(0.432) 0.434 0.424

Locus 9477 :

2 alleles

99.2% missing data

1	(0.513) 0.518 0.507

2	(0.487) 0.482 0.493

Locus 9478 :

2 alleles

99.2% missing data

3	(0.709) 0.709 0.707

2	(0.291) 0.291 0.293

Locus 9479 :

2 alleles

98.8% missing data

3	(0.695) 0.694 0.696

1	(0.305) 0.306 0.304

Locus 9480 :

2 alleles

8.2% missing data

2	(0.985) 0.980 0.996

3	(0.015) 0.020 0.004

Locus 9481 :

2 alleles

98.8% missing data

4	(0.394) 0.384 0.398

2	(0.606) 0.616 0.602

Locus 9482 :

2 alleles

99.2% missing data

1	(0.497) 0.506 0.491

4	(0.503) 0.494 0.509

Locus 9483 :

2 alleles

96.1% missing data

3	(0.157) 0.147 0.161

1	(0.843) 0.853 0.839

Locus 9484 :

2 alleles

96.5% missing data

3	(0.430) 0.442 0.416

1	(0.570) 0.558 0.584

Locus 9485 :

2 alleles

99.2% missing data

1	(0.575) 0.572 0.585

3	(0.425) 0.428 0.415

Locus 9486 :

2 alleles

96.1% missing data

2	(0.152) 0.141 0.156

3	(0.848) 0.859 0.844

Locus 9487 :

2 alleles

99.2% missing data

3	(0.244) 0.243 0.251

1	(0.756) 0.757 0.749

Locus 9488 :

2 alleles

99.2% missing data

2	(0.426) 0.429 0.427

1	(0.574) 0.571 0.573

Locus 9489 :

2 alleles

99.2% missing data

1	(0.647) 0.643 0.653

2	(0.353) 0.357 0.347

Locus 9490 :

2 alleles

99.2% missing data

4	(0.516) 0.516 0.511

1	(0.484) 0.484 0.489

Locus 9491 :

2 alleles

97.7% missing data

1	(0.573) 0.574 0.573

4	(0.427) 0.426 0.427

Locus 9492 :

2 alleles

99.2% missing data

4	(0.452) 0.447 0.462

2	(0.548) 0.553 0.538

Locus 9493 :

2 alleles

99.2% missing data

4	(0.319) 0.315 0.327

1	(0.681) 0.685 0.673

Locus 9494 :

2 alleles

99.2% missing data

1	(0.504) 0.499 0.505

2	(0.496) 0.501 0.495

Locus 9495 :

3 alleles

98.0% missing data

2	(0.713) 0.711 0.712

4	(0.113) 0.108 0.122

1	(0.174) 0.181 0.166

Locus 9496 :

2 alleles

99.2% missing data

3	(0.468) 0.466 0.474

1	(0.532) 0.534 0.526

Locus 9497 :

2 alleles

99.2% missing data

2	(0.397) 0.395 0.403

1	(0.603) 0.605 0.597

Locus 9498 :

2 alleles

99.2% missing data

3	(0.643) 0.646 0.640

2	(0.357) 0.354 0.360

Locus 9499 :

2 alleles

97.7% missing data

2	(0.854) 0.855 0.857

3	(0.146) 0.145 0.143

Locus 9500 :

3 alleles

98.8% missing data

2	(0.160) 0.155 0.166

3	(0.513) 0.509 0.514

1	(0.328) 0.336 0.320

Locus 9501 :

2 alleles

99.2% missing data

1	(0.583) 0.579 0.583

3	(0.417) 0.421 0.417

Locus 9502 :

2 alleles

99.2% missing data

2	(0.706) 0.702 0.715

3	(0.294) 0.298 0.285

Locus 9503 :

2 alleles

99.2% missing data

2	(0.740) 0.736 0.750

1	(0.260) 0.264 0.250

Locus 9504 :

2 alleles

98.8% missing data

2	(0.508) 0.517 0.501

3	(0.492) 0.483 0.499

Locus 9505 :

2 alleles

98.4% missing data

2	(0.850) 0.858 0.843

4	(0.150) 0.142 0.157

Locus 9506 :

2 alleles

99.2% missing data

4	(0.768) 0.762 0.771

1	(0.232) 0.238 0.229

Locus 9507 :

2 alleles

99.2% missing data

1	(0.683) 0.681 0.683

2	(0.317) 0.319 0.317

Locus 9508 :

2 alleles

98.0% missing data

2	(0.678) 0.681 0.677

4	(0.322) 0.319 0.323

Locus 9509 :

2 alleles

93.0% missing data

3	(0.917) 0.917 0.925

1	(0.083) 0.083 0.075

Locus 9510 :

2 alleles

0.0% missing data

1	(0.988) 0.992 0.993

4	(0.012) 0.008 0.007

Locus 9511 :

2 alleles

88.7% missing data

1	(0.711) 0.764 0.662

3	(0.289) 0.236 0.338

Locus 9512 :

2 alleles

97.7% missing data

1	(0.723) 0.728 0.728

2	(0.277) 0.272 0.272

Locus 9513 :

2 alleles

99.2% missing data

2	(0.512) 0.516 0.503

3	(0.488) 0.484 0.497

Locus 9514 :

2 alleles

98.8% missing data

1	(0.759) 0.761 0.758

3	(0.241) 0.239 0.242

Locus 9515 :

2 alleles

98.8% missing data

4	(0.695) 0.695 0.694

1	(0.305) 0.305 0.306

Locus 9516 :

2 alleles

90.6% missing data

4	(0.907) 0.922 0.897

3	(0.093) 0.078 0.103

Locus 9517 :

2 alleles

98.8% missing data

2	(0.645) 0.636 0.654

1	(0.355) 0.364 0.346

Locus 9518 :

2 alleles

98.8% missing data

2	(0.759) 0.761 0.757

1	(0.241) 0.239 0.243

Locus 9519 :

2 alleles

99.2% missing data

4	(0.585) 0.588 0.589

2	(0.415) 0.412 0.411

Locus 9520 :

2 alleles

98.0% missing data

1	(0.701) 0.706 0.702

3	(0.299) 0.294 0.298

Locus 9521 :

2 alleles

99.2% missing data

3	(0.511) 0.504 0.520

2	(0.489) 0.496 0.480

Locus 9522 :

2 alleles

99.2% missing data

1	(0.653) 0.656 0.650

2	(0.347) 0.344 0.350

Locus 9523 :

2 alleles

96.5% missing data

2	(0.888) 0.897 0.881

3	(0.112) 0.103 0.119

Locus 9524 :

2 alleles

99.2% missing data

2	(0.443) 0.441 0.438

4	(0.557) 0.559 0.562

Locus 9525 :

2 alleles

99.2% missing data

2	(0.669) 0.666 0.668

3	(0.331) 0.334 0.332

Locus 9526 :

2 alleles

99.2% missing data

1	(0.350) 0.349 0.353

4	(0.650) 0.651 0.647

Locus 9527 :

2 alleles

98.8% missing data

2	(0.619) 0.625 0.617

3	(0.381) 0.375 0.383

Locus 9528 :

2 alleles

96.5% missing data

3	(0.109) 0.100 0.120

1	(0.891) 0.900 0.880

Locus 9529 :

2 alleles

99.2% missing data

4	(0.799) 0.805 0.793

2	(0.201) 0.195 0.207

Locus 9530 :

2 alleles

96.1% missing data

1	(0.821) 0.811 0.830

2	(0.179) 0.189 0.170

Locus 9531 :

2 alleles

99.2% missing data

2	(0.463) 0.458 0.464

4	(0.537) 0.542 0.536

Locus 9532 :

2 alleles

99.2% missing data

2	(0.767) 0.773 0.764

4	(0.233) 0.227 0.236

Locus 9533 :

2 alleles

98.8% missing data

3	(0.467) 0.468 0.470

2	(0.533) 0.532 0.530

Locus 9534 :

2 alleles

96.1% missing data

3	(0.738) 0.747 0.746

2	(0.262) 0.253 0.254

Locus 9535 :

2 alleles

99.2% missing data

3	(0.816) 0.807 0.817

2	(0.184) 0.193 0.183

Locus 9536 :

3 alleles

97.7% missing data

1	(0.321) 0.322 0.322

4	(0.205) 0.206 0.206

2	(0.474) 0.472 0.472

Locus 9537 :

2 alleles

99.2% missing data

3	(0.284) 0.284 0.286

2	(0.716) 0.716 0.714

Locus 9538 :

2 alleles

99.2% missing data

3	(0.559) 0.554 0.566

2	(0.441) 0.446 0.434

Locus 9539 :

3 alleles

98.8% missing data

2	(0.405) 0.403 0.404

3	(0.276) 0.270 0.280

4	(0.319) 0.327 0.316

Locus 9540 :

2 alleles

99.2% missing data

4	(0.602) 0.595 0.606

3	(0.398) 0.405 0.394

Locus 9541 :

2 alleles

99.2% missing data

2	(0.277) 0.275 0.281

3	(0.723) 0.725 0.719

Locus 9542 :

2 alleles

99.2% missing data

2	(0.751) 0.751 0.752

1	(0.249) 0.249 0.248

Locus 9543 :

2 alleles

98.8% missing data

1	(0.556) 0.556 0.550

2	(0.444) 0.444 0.450

Locus 9544 :

3 alleles

89.1% missing data

4	(0.737) 0.760 0.730

3	(0.062) 0.053 0.069

2	(0.201) 0.187 0.202

Locus 9545 :

2 alleles

98.4% missing data

3	(0.689) 0.689 0.693

2	(0.311) 0.311 0.307

Locus 9546 :

2 alleles

95.3% missing data

4	(0.829) 0.826 0.835

2	(0.171) 0.174 0.165

Locus 9547 :

2 alleles

94.5% missing data

4	(0.761) 0.750 0.788

1	(0.239) 0.250 0.212

Locus 9548 :

2 alleles

99.2% missing data

3	(0.438) 0.431 0.437

2	(0.562) 0.569 0.563

Locus 9549 :

2 alleles

99.2% missing data

4	(0.612) 0.621 0.602

1	(0.388) 0.379 0.398

Locus 9550 :

2 alleles

9.8% missing data

4	(0.963) 0.930 0.988

3	(0.037) 0.070 0.012

Locus 9551 :

2 alleles

99.2% missing data

3	(0.388) 0.393 0.386

2	(0.612) 0.607 0.614

Locus 9552 :

2 alleles

98.8% missing data

3	(0.277) 0.276 0.286

4	(0.723) 0.724 0.714

Locus 9553 :

2 alleles

8.6% missing data

3	(0.986) 0.978 0.996

1	(0.014) 0.022 0.004

Locus 9554 :

2 alleles

99.2% missing data

4	(0.561) 0.559 0.559

1	(0.439) 0.441 0.441

Locus 9555 :

2 alleles

99.2% missing data

4	(0.373) 0.371 0.379

1	(0.627) 0.629 0.621

Locus 9556 :

3 alleles

98.8% missing data

1	(0.373) 0.378 0.364

4	(0.372) 0.368 0.375

2	(0.255) 0.253 0.261

Locus 9557 :

2 alleles

99.2% missing data

3	(0.567) 0.569 0.562

4	(0.433) 0.431 0.438

Locus 9558 :

2 alleles

98.8% missing data

3	(0.353) 0.351 0.355

2	(0.647) 0.649 0.645

Locus 9559 :

2 alleles

98.0% missing data

2	(0.737) 0.735 0.744

3	(0.263) 0.265 0.256

Locus 9560 :

2 alleles

98.8% missing data

4	(0.341) 0.334 0.346

3	(0.659) 0.666 0.654

Locus 9561 :

2 alleles

98.4% missing data

3	(0.472) 0.473 0.463

2	(0.528) 0.527 0.537

Locus 9562 :

2 alleles

99.2% missing data

3	(0.375) 0.376 0.381

2	(0.625) 0.624 0.619

Locus 9563 :

2 alleles

99.2% missing data

2	(0.660) 0.655 0.665

3	(0.340) 0.345 0.335

Locus 9564 :

2 alleles

99.2% missing data

1	(0.483) 0.479 0.490

2	(0.517) 0.521 0.510

Locus 9565 :

2 alleles

99.2% missing data

4	(0.506) 0.508 0.507

2	(0.494) 0.492 0.493

Locus 9566 :

2 alleles

99.2% missing data

1	(0.498) 0.494 0.498

3	(0.502) 0.506 0.502

Locus 9567 :

2 alleles

99.2% missing data

4	(0.562) 0.557 0.563

3	(0.438) 0.443 0.437

Locus 9568 :

2 alleles

99.2% missing data

1	(0.577) 0.566 0.581

3	(0.423) 0.434 0.419

Locus 9569 :

2 alleles

99.2% missing data

3	(0.463) 0.459 0.468

2	(0.537) 0.541 0.532

Locus 9570 :

2 alleles

96.5% missing data

4	(0.135) 0.129 0.140

3	(0.865) 0.871 0.860

Locus 9571 :

2 alleles

91.4% missing data

2	(0.838) 0.857 0.819

3	(0.162) 0.143 0.181

Locus 9572 :

2 alleles

99.2% missing data

4	(0.657) 0.652 0.660

1	(0.343) 0.348 0.340

Locus 9573 :

2 alleles

98.8% missing data

4	(0.741) 0.738 0.745

2	(0.259) 0.262 0.255

Locus 9574 :

2 alleles

99.2% missing data

3	(0.485) 0.487 0.485

4	(0.515) 0.513 0.515

Locus 9575 :

2 alleles

99.2% missing data

4	(0.840) 0.841 0.838

2	(0.160) 0.159 0.162

Locus 9576 :

2 alleles

99.2% missing data

4	(0.281) 0.274 0.282

1	(0.719) 0.726 0.718

Locus 9577 :

2 alleles

99.2% missing data

3	(0.272) 0.273 0.273

2	(0.728) 0.727 0.727

Locus 9578 :

2 alleles

99.2% missing data

3	(0.653) 0.649 0.654

2	(0.347) 0.351 0.346

Locus 9579 :

2 alleles

94.9% missing data

1	(0.114) 0.112 0.118

2	(0.886) 0.888 0.882

Locus 9580 :

2 alleles

99.2% missing data

2	(0.456) 0.455 0.455

1	(0.544) 0.545 0.545

Locus 9581 :

2 alleles

99.2% missing data

1	(0.347) 0.343 0.349

3	(0.653) 0.657 0.651

Locus 9582 :

3 alleles

98.0% missing data

1	(0.165) 0.166 0.159

4	(0.701) 0.711 0.698

3	(0.134) 0.124 0.142

Locus 9583 :

2 alleles

99.2% missing data

1	(0.698) 0.700 0.693

3	(0.302) 0.300 0.307

Locus 9584 :

2 alleles

99.2% missing data

2	(0.338) 0.343 0.339

3	(0.662) 0.657 0.661

Locus 9585 :

3 alleles

98.8% missing data

4	(0.320) 0.322 0.320

2	(0.355) 0.358 0.354

1	(0.326) 0.320 0.326

Locus 9586 :

3 alleles

98.8% missing data

3	(0.372) 0.366 0.375

4	(0.283) 0.290 0.274

2	(0.345) 0.345 0.350

Locus 9587 :

3 alleles

98.8% missing data

2	(0.376) 0.380 0.375

4	(0.173) 0.171 0.177

1	(0.451) 0.449 0.449

Locus 9588 :

2 alleles

99.2% missing data

4	(0.496) 0.496 0.500

3	(0.504) 0.504 0.500

Locus 9589 :

2 alleles

99.2% missing data

3	(0.220) 0.221 0.222

4	(0.780) 0.779 0.778

Locus 9590 :

2 alleles

98.8% missing data

3	(0.517) 0.505 0.527

2	(0.483) 0.495 0.473

Locus 9591 :

2 alleles

99.2% missing data

2	(0.386) 0.378 0.387

1	(0.614) 0.622 0.613

Locus 9592 :

2 alleles

99.2% missing data

3	(0.561) 0.566 0.554

2	(0.439) 0.434 0.446

Locus 9593 :

2 alleles

99.2% missing data

4	(0.604) 0.595 0.606

1	(0.396) 0.405 0.394

Locus 9594 :

2 alleles

99.2% missing data

2	(0.341) 0.342 0.347

4	(0.659) 0.658 0.653

Locus 9595 :

2 alleles

93.0% missing data

2	(0.819) 0.819 0.833

4	(0.181) 0.181 0.167

Locus 9596 :

3 alleles

98.0% missing data

1	(0.171) 0.169 0.180

3	(0.310) 0.312 0.299

4	(0.519) 0.519 0.521

Locus 9597 :

3 alleles

92.2% missing data

1	(0.320) 0.307 0.326

2	(0.301) 0.282 0.316

4	(0.379) 0.411 0.357

Locus 9598 :

2 alleles

97.7% missing data

2	(0.454) 0.441 0.455

1	(0.546) 0.559 0.545

Locus 9599 :

4 alleles

97.3% missing data

4	(0.383) 0.382 0.389

1	(0.149) 0.146 0.151

3	(0.171) 0.183 0.163

2	(0.296) 0.289 0.297

Locus 9600 :

2 alleles

99.2% missing data

1	(0.461) 0.461 0.462

2	(0.539) 0.539 0.538

Locus 9601 :

2 alleles

98.8% missing data

2	(0.274) 0.280 0.270

1	(0.726) 0.720 0.730

Locus 9602 :

2 alleles

97.7% missing data

2	(0.532) 0.538 0.539

4	(0.468) 0.462 0.461

Locus 9603 :

3 alleles

98.4% missing data

1	(0.418) 0.413 0.428

2	(0.231) 0.232 0.231

3	(0.351) 0.355 0.341

Locus 9604 :

2 alleles

98.8% missing data

4	(0.404) 0.404 0.402

1	(0.596) 0.596 0.598

Locus 9605 :

2 alleles

99.2% missing data

2	(0.415) 0.420 0.408

4	(0.585) 0.580 0.592

Locus 9606 :

2 alleles

99.2% missing data

1	(0.483) 0.480 0.480

2	(0.517) 0.520 0.520

Locus 9607 :

2 alleles

98.8% missing data

3	(0.692) 0.701 0.693

1	(0.308) 0.299 0.307

Locus 9608 :

2 alleles

96.1% missing data

3	(0.837) 0.843 0.839

4	(0.163) 0.157 0.161

Locus 9609 :

2 alleles

98.4% missing data

2	(0.749) 0.748 0.744

1	(0.251) 0.252 0.256

Locus 9610 :

2 alleles

99.2% missing data

2	(0.505) 0.510 0.502

1	(0.495) 0.490 0.498

Locus 9611 :

3 alleles

98.4% missing data

3	(0.273) 0.272 0.274

1	(0.363) 0.362 0.365

2	(0.364) 0.366 0.361

Locus 9612 :

2 alleles

98.8% missing data

3	(0.615) 0.624 0.609

1	(0.385) 0.376 0.391

Locus 9613 :

2 alleles

99.2% missing data

1	(0.198) 0.201 0.203

2	(0.802) 0.799 0.797

Locus 9614 :

2 alleles

98.4% missing data

1	(0.797) 0.786 0.806

4	(0.203) 0.214 0.194

Locus 9615 :

2 alleles

98.8% missing data

2	(0.656) 0.656 0.654

1	(0.344) 0.344 0.346

Locus 9616 :

2 alleles

99.2% missing data

4	(0.520) 0.518 0.519

2	(0.480) 0.482 0.481

Locus 9617 :

2 alleles

98.8% missing data

1	(0.510) 0.507 0.512

2	(0.490) 0.493 0.488

Locus 9618 :

3 alleles

98.4% missing data

1	(0.615) 0.616 0.611

3	(0.212) 0.218 0.210

2	(0.174) 0.166 0.178

Locus 9619 :

2 alleles

99.2% missing data

3	(0.512) 0.510 0.519

1	(0.488) 0.490 0.481

Locus 9620 :

2 alleles

94.9% missing data

2	(0.847) 0.839 0.862

3	(0.153) 0.161 0.138

Locus 9621 :

2 alleles

99.2% missing data

1	(0.614) 0.613 0.611

4	(0.386) 0.387 0.389

Locus 9622 :

2 alleles

99.2% missing data

1	(0.546) 0.535 0.547

3	(0.454) 0.465 0.453

Locus 9623 :

2 alleles

99.2% missing data

2	(0.768) 0.765 0.773

1	(0.232) 0.235 0.227

Locus 9624 :

2 alleles

99.2% missing data

2	(0.345) 0.345 0.348

1	(0.655) 0.655 0.652

Locus 9625 :

2 alleles

99.2% missing data

2	(0.550) 0.547 0.557

3	(0.450) 0.453 0.443

Locus 9626 :

3 alleles

90.2% missing data

3	(0.548) 0.506 0.606

1	(0.377) 0.415 0.330

2	(0.075) 0.079 0.064

Locus 9627 :

3 alleles

96.9% missing data

1	(0.446) 0.443 0.459

4	(0.187) 0.189 0.178

2	(0.367) 0.368 0.363

Locus 9628 :

2 alleles

98.0% missing data

1	(0.719) 0.700 0.731

2	(0.281) 0.300 0.269

Locus 9629 :

2 alleles

99.2% missing data

4	(0.504) 0.501 0.505

2	(0.496) 0.499 0.495

Locus 9630 :

2 alleles

99.2% missing data

2	(0.370) 0.373 0.366

1	(0.630) 0.627 0.634

Locus 9631 :

2 alleles

98.8% missing data

1	(0.785) 0.790 0.777

3	(0.215) 0.210 0.223

Locus 9632 :

3 alleles

97.7% missing data

2	(0.647) 0.655 0.647

4	(0.122) 0.115 0.130

1	(0.230) 0.229 0.223

Locus 9633 :

2 alleles

98.8% missing data

4	(0.389) 0.381 0.397

2	(0.611) 0.619 0.603

Locus 9634 :

3 alleles

98.4% missing data

2	(0.418) 0.417 0.417

1	(0.276) 0.274 0.278

4	(0.306) 0.310 0.305

Locus 9635 :

2 alleles

7.4% missing data

4	(0.976) 0.952 0.993

1	(0.024) 0.048 0.007

Locus 9636 :

2 alleles

97.7% missing data

2	(0.784) 0.777 0.797

4	(0.216) 0.223 0.203

Locus 9637 :

2 alleles

99.2% missing data

4	(0.399) 0.393 0.406

3	(0.601) 0.607 0.594

Locus 9638 :

2 alleles

99.2% missing data

1	(0.807) 0.812 0.799

2	(0.193) 0.188 0.201

Locus 9639 :

2 alleles

99.2% missing data

1	(0.230) 0.226 0.231

2	(0.770) 0.774 0.769

Locus 9640 :

2 alleles

99.2% missing data

3	(0.794) 0.794 0.788

2	(0.206) 0.206 0.212

Locus 9641 :

2 alleles

99.2% missing data

1	(0.408) 0.400 0.414

3	(0.592) 0.600 0.586

Locus 9642 :

2 alleles

99.2% missing data

2	(0.552) 0.554 0.547

1	(0.448) 0.446 0.453

Locus 9643 :

2 alleles

99.2% missing data

2	(0.488) 0.491 0.488

1	(0.512) 0.509 0.512

Locus 9644 :

2 alleles

98.4% missing data

2	(0.688) 0.685 0.703

1	(0.312) 0.315 0.297

Locus 9645 :

3 alleles

98.8% missing data

4	(0.333) 0.333 0.338

1	(0.239) 0.241 0.236

2	(0.428) 0.426 0.426

Locus 9646 :

2 alleles

99.2% missing data

1	(0.494) 0.500 0.492

2	(0.506) 0.500 0.508

Locus 9647 :

3 alleles

93.8% missing data

2	(0.624) 0.626 0.632

3	(0.095) 0.086 0.095

1	(0.280) 0.288 0.273

Locus 9648 :

2 alleles

99.2% missing data

4	(0.608) 0.601 0.613

1	(0.392) 0.399 0.387

Locus 9649 :

2 alleles

99.2% missing data

1	(0.662) 0.665 0.660

4	(0.338) 0.335 0.340

Locus 9650 :

2 alleles

98.4% missing data

4	(0.298) 0.288 0.304

3	(0.702) 0.712 0.696

Locus 9651 :

2 alleles

99.2% missing data

3	(0.395) 0.393 0.397

1	(0.605) 0.607 0.603

Locus 9652 :

2 alleles

99.2% missing data

2	(0.614) 0.620 0.606

4	(0.386) 0.380 0.394

Locus 9653 :

2 alleles

99.2% missing data

4	(0.616) 0.612 0.618

1	(0.384) 0.388 0.382

Locus 9654 :

2 alleles

91.0% missing data

4	(0.917) 0.925 0.916

2	(0.083) 0.075 0.084

Locus 9655 :

2 alleles

99.2% missing data

2	(0.342) 0.343 0.340

1	(0.658) 0.657 0.660

Locus 9656 :

3 alleles

98.4% missing data

4	(0.390) 0.393 0.384

1	(0.247) 0.235 0.256

2	(0.363) 0.372 0.360

Locus 9657 :

2 alleles

98.0% missing data

3	(0.189) 0.189 0.186

4	(0.811) 0.811 0.814

Locus 9658 :

2 alleles

99.2% missing data

3	(0.391) 0.397 0.396

2	(0.609) 0.603 0.604

Locus 9659 :

2 alleles

99.2% missing data

4	(0.479) 0.481 0.481

2	(0.521) 0.519 0.519

Locus 9660 :

2 alleles

99.2% missing data

4	(0.399) 0.400 0.398

1	(0.601) 0.600 0.602

Locus 9661 :

2 alleles

92.6% missing data

4	(0.932) 0.927 0.943

1	(0.068) 0.073 0.057

Locus 9662 :

2 alleles

99.2% missing data

1	(0.727) 0.721 0.729

2	(0.273) 0.279 0.271

Locus 9663 :

2 alleles

89.8% missing data

4	(0.922) 0.930 0.925

1	(0.078) 0.070 0.075

Locus 9664 :

2 alleles

99.2% missing data

1	(0.568) 0.562 0.576

3	(0.432) 0.438 0.424

Locus 9665 :

2 alleles

99.2% missing data

3	(0.603) 0.603 0.603

4	(0.397) 0.397 0.397

Locus 9666 :

2 alleles

99.2% missing data

2	(0.335) 0.331 0.341

3	(0.665) 0.669 0.659

Locus 9667 :

2 alleles

94.1% missing data

4	(0.195) 0.195 0.190

2	(0.805) 0.805 0.810

Locus 9668 :

2 alleles

99.2% missing data

1	(0.538) 0.532 0.545

3	(0.462) 0.468 0.455

Locus 9669 :

2 alleles

0.0% missing data

4	(0.986) 0.981 0.996

1	(0.014) 0.019 0.004

Locus 9670 :

2 alleles

98.4% missing data

2	(0.663) 0.675 0.656

1	(0.337) 0.325 0.344

Locus 9671 :

2 alleles

99.2% missing data

2	(0.317) 0.315 0.322

3	(0.683) 0.685 0.678

Locus 9672 :

2 alleles

99.2% missing data

4	(0.368) 0.362 0.373

1	(0.632) 0.638 0.627

Locus 9673 :

2 alleles

99.2% missing data

3	(0.738) 0.739 0.734

4	(0.262) 0.261 0.266

Locus 9674 :

2 alleles

98.8% missing data

1	(0.233) 0.233 0.231

4	(0.767) 0.767 0.769

Locus 9675 :

2 alleles

99.2% missing data

3	(0.343) 0.341 0.345

4	(0.657) 0.659 0.655

Locus 9676 :

2 alleles

89.8% missing data

4	(0.920) 0.938 0.906

2	(0.080) 0.062 0.094

Locus 9677 :

2 alleles

99.2% missing data

1	(0.451) 0.443 0.452

4	(0.549) 0.557 0.548

Locus 9678 :

2 alleles

99.2% missing data

4	(0.480) 0.474 0.488

2	(0.520) 0.526 0.512

Locus 9679 :

2 alleles

99.2% missing data

1	(0.386) 0.383 0.388

2	(0.614) 0.617 0.612

Locus 9680 :

2 alleles

99.2% missing data

4	(0.205) 0.213 0.202

3	(0.795) 0.787 0.798

Locus 9681 :

2 alleles

96.5% missing data

2	(0.275) 0.279 0.281

1	(0.725) 0.721 0.719

Locus 9682 :

2 alleles

91.0% missing data

4	(0.916) 0.919 0.915

2	(0.084) 0.081 0.085

Locus 9683 :

2 alleles

91.0% missing data

3	(0.936) 0.951 0.928

4	(0.064) 0.049 0.072

Locus 9684 :

2 alleles

90.6% missing data

2	(0.930) 0.941 0.926

1	(0.070) 0.059 0.074

Locus 9685 :

2 alleles

99.2% missing data

3	(0.594) 0.592 0.594

4	(0.406) 0.408 0.406

Locus 9686 :

2 alleles

96.9% missing data

2	(0.148) 0.147 0.149

1	(0.852) 0.853 0.851

Locus 9687 :

2 alleles

99.2% missing data

3	(0.420) 0.418 0.422

1	(0.580) 0.582 0.578

Locus 9688 :

3 alleles

98.8% missing data

3	(0.290) 0.288 0.295

1	(0.532) 0.537 0.521

4	(0.178) 0.175 0.184

Locus 9689 :

2 alleles

96.9% missing data

2	(0.406) 0.406 0.406

1	(0.594) 0.594 0.594

Locus 9690 :

2 alleles

96.9% missing data

2	(0.580) 0.594 0.572

1	(0.420) 0.406 0.428

Locus 9691 :

3 alleles

97.7% missing data

1	(0.233) 0.243 0.234

2	(0.489) 0.482 0.492

4	(0.278) 0.275 0.274

Locus 9692 :

2 alleles

98.0% missing data

1	(0.232) 0.225 0.237

2	(0.768) 0.775 0.763

Locus 9693 :

3 alleles

98.0% missing data

1	(0.396) 0.392 0.396

4	(0.178) 0.175 0.184

2	(0.425) 0.433 0.420

Locus 9694 :

2 alleles

99.2% missing data

4	(0.797) 0.800 0.789

2	(0.203) 0.200 0.211

Locus 9695 :

3 alleles

96.1% missing data

1	(0.607) 0.627 0.597

2	(0.175) 0.158 0.185

4	(0.218) 0.215 0.218

Locus 9696 :

2 alleles

97.7% missing data

1	(0.750) 0.749 0.755

2	(0.250) 0.251 0.245

Locus 9697 :

3 alleles

98.8% missing data

4	(0.452) 0.455 0.452

2	(0.349) 0.349 0.340

1	(0.199) 0.195 0.208

Locus 9698 :

2 alleles

99.2% missing data

1	(0.678) 0.670 0.685

2	(0.322) 0.330 0.315

Locus 9699 :

2 alleles

99.2% missing data

2	(0.814) 0.817 0.810

1	(0.186) 0.183 0.190

Locus 9700 :

2 alleles

98.4% missing data

3	(0.276) 0.272 0.281

2	(0.724) 0.728 0.719

Locus 9701 :

2 alleles

96.1% missing data

4	(0.772) 0.779 0.779

1	(0.228) 0.221 0.221

Locus 9702 :

2 alleles

98.4% missing data

2	(0.271) 0.263 0.273

1	(0.729) 0.737 0.727

Locus 9703 :

2 alleles

98.8% missing data

1	(0.338) 0.334 0.337

2	(0.662) 0.666 0.663

Locus 9704 :

3 alleles

98.4% missing data

4	(0.144) 0.142 0.146

1	(0.745) 0.748 0.737

2	(0.111) 0.110 0.117

Locus 9705 :

2 alleles

98.0% missing data

1	(0.612) 0.613 0.610

2	(0.388) 0.387 0.390

Locus 9706 :

2 alleles

98.8% missing data

1	(0.588) 0.588 0.592

2	(0.412) 0.412 0.408

Locus 9707 :

2 alleles

99.2% missing data

2	(0.578) 0.575 0.582

1	(0.422) 0.425 0.418

Locus 9708 :

2 alleles

98.8% missing data

2	(0.245) 0.241 0.251

1	(0.755) 0.759 0.749

Locus 9709 :

2 alleles

99.2% missing data

1	(0.732) 0.733 0.728

2	(0.268) 0.267 0.272

Locus 9710 :

2 alleles

95.3% missing data

2	(0.729) 0.743 0.719

1	(0.271) 0.257 0.281

Locus 9711 :

2 alleles

99.2% missing data

1	(0.761) 0.766 0.757

4	(0.239) 0.234 0.243

Locus 9712 :

2 alleles

99.2% missing data

1	(0.254) 0.249 0.258

2	(0.746) 0.751 0.742

Locus 9713 :

2 alleles

99.2% missing data

2	(0.341) 0.344 0.344

1	(0.659) 0.656 0.656

Locus 9714 :

2 alleles

99.2% missing data

4	(0.665) 0.667 0.659

2	(0.335) 0.333 0.341

Locus 9715 :

2 alleles

99.2% missing data

2	(0.586) 0.585 0.590

4	(0.414) 0.415 0.410

Locus 9716 :

2 alleles

99.2% missing data

2	(0.352) 0.352 0.354

1	(0.648) 0.648 0.646

Locus 9717 :

2 alleles

99.2% missing data

3	(0.356) 0.352 0.364

1	(0.644) 0.648 0.636

Locus 9718 :

2 alleles

99.2% missing data

4	(0.686) 0.686 0.689

1	(0.314) 0.314 0.311

Locus 9719 :

2 alleles

97.7% missing data

4	(0.853) 0.853 0.849

1	(0.147) 0.147 0.151

Locus 9720 :

2 alleles

99.2% missing data

2	(0.327) 0.330 0.334

1	(0.673) 0.670 0.666

Locus 9721 :

2 alleles

98.4% missing data

3	(0.279) 0.277 0.273

1	(0.721) 0.723 0.727

Locus 9722 :

2 alleles

8.6% missing data

4	(0.990) 0.983 0.997

1	(0.010) 0.017 0.003

Locus 9723 :

2 alleles

94.9% missing data

4	(0.851) 0.846 0.866

3	(0.149) 0.154 0.134

Locus 9724 :

2 alleles

97.3% missing data

1	(0.595) 0.610 0.584

2	(0.405) 0.390 0.416

Locus 9725 :

2 alleles

98.0% missing data

2	(0.162) 0.155 0.176

1	(0.838) 0.845 0.824

Locus 9726 :

2 alleles

97.3% missing data

3	(0.130) 0.120 0.138

1	(0.870) 0.880 0.862

Locus 9727 :

2 alleles

8.2% missing data

4	(0.984) 0.976 0.995

1	(0.016) 0.024 0.005

Locus 9728 :

2 alleles

98.0% missing data

1	(0.393) 0.400 0.400

2	(0.607) 0.600 0.600

Locus 9729 :

2 alleles

98.4% missing data

1	(0.654) 0.657 0.646

2	(0.346) 0.343 0.354

Locus 9730 :

2 alleles

99.2% missing data

2	(0.558) 0.563 0.556

3	(0.442) 0.437 0.444

Locus 9731 :

2 alleles

98.8% missing data

1	(0.796) 0.801 0.793

2	(0.204) 0.199 0.207

Locus 9732 :

2 alleles

98.8% missing data

4	(0.601) 0.603 0.599

1	(0.399) 0.397 0.401

Locus 9733 :

2 alleles

99.2% missing data

2	(0.276) 0.284 0.272

1	(0.724) 0.716 0.728

Locus 9734 :

2 alleles

98.8% missing data

1	(0.538) 0.546 0.529

2	(0.462) 0.454 0.471

Locus 9735 :

2 alleles

98.4% missing data

2	(0.688) 0.684 0.694

4	(0.312) 0.316 0.306

Locus 9736 :

2 alleles

96.1% missing data

2	(0.649) 0.642 0.655

3	(0.351) 0.358 0.345

Locus 9737 :

2 alleles

97.7% missing data

3	(0.158) 0.160 0.162

2	(0.842) 0.840 0.838

Locus 9738 :

2 alleles

96.1% missing data

2	(0.557) 0.558 0.564

1	(0.443) 0.442 0.436

Locus 9739 :

2 alleles

99.2% missing data

1	(0.520) 0.527 0.513

2	(0.480) 0.473 0.487

Locus 9740 :

3 alleles

97.3% missing data

1	(0.575) 0.585 0.573

4	(0.118) 0.113 0.118

2	(0.307) 0.302 0.309

Locus 9741 :

3 alleles

97.7% missing data

3	(0.262) 0.262 0.265

1	(0.404) 0.399 0.413

2	(0.334) 0.338 0.322

Locus 9742 :

2 alleles

97.7% missing data

2	(0.737) 0.743 0.728

4	(0.263) 0.257 0.272

Locus 9743 :

3 alleles

62.9% missing data

2	(0.930) 0.936 0.943

1	(0.042) 0.037 0.036

4	(0.029) 0.027 0.022

Locus 9744 :

2 alleles

94.9% missing data

1	(0.233) 0.227 0.232

2	(0.767) 0.773 0.768

Locus 9745 :

2 alleles

98.8% missing data

2	(0.633) 0.633 0.631

4	(0.367) 0.367 0.369

Locus 9746 :

3 alleles

89.1% missing data

4	(0.672) 0.677 0.674

1	(0.265) 0.263 0.266

2	(0.063) 0.060 0.059

Locus 9747 :

2 alleles

19.9% missing data

4	(0.986) 0.980 0.995

3	(0.014) 0.020 0.005

Locus 9748 :

2 alleles

98.8% missing data

2	(0.686) 0.695 0.682

1	(0.314) 0.305 0.318

Locus 9749 :

3 alleles

97.3% missing data

2	(0.759) 0.762 0.755

4	(0.120) 0.118 0.125

1	(0.120) 0.120 0.120

Locus 9750 :

3 alleles

96.5% missing data

2	(0.735) 0.739 0.743

3	(0.187) 0.179 0.187

4	(0.078) 0.081 0.070

Locus 9751 :

3 alleles

98.4% missing data

4	(0.265) 0.270 0.263

2	(0.309) 0.303 0.315

1	(0.426) 0.426 0.421

Locus 9752 :

2 alleles

99.2% missing data

4	(0.505) 0.511 0.504

1	(0.495) 0.489 0.496

Locus 9753 :

2 alleles

98.8% missing data

2	(0.630) 0.629 0.635

4	(0.370) 0.371 0.365

Locus 9754 :

2 alleles

99.2% missing data

3	(0.651) 0.640 0.655

2	(0.349) 0.360 0.345

Locus 9755 :

3 alleles

97.7% missing data

3	(0.669) 0.678 0.662

4	(0.168) 0.164 0.175

2	(0.163) 0.157 0.164

Locus 9756 :

2 alleles

99.2% missing data

4	(0.473) 0.468 0.472

3	(0.527) 0.532 0.528

Locus 9757 :

2 alleles

99.2% missing data

2	(0.385) 0.381 0.394

3	(0.615) 0.619 0.606

Locus 9758 :

2 alleles

99.2% missing data

2	(0.765) 0.772 0.759

4	(0.235) 0.228 0.241

Locus 9759 :

2 alleles

99.2% missing data

4	(0.597) 0.586 0.604

3	(0.403) 0.414 0.396

Locus 9760 :

2 alleles

98.4% missing data

2	(0.715) 0.718 0.697

1	(0.285) 0.282 0.303

Locus 9761 :

2 alleles

99.2% missing data

2	(0.405) 0.397 0.412

4	(0.595) 0.603 0.588

Locus 9762 :

2 alleles

98.8% missing data

2	(0.654) 0.647 0.661

3	(0.346) 0.353 0.339

Locus 9763 :

2 alleles

99.2% missing data

1	(0.377) 0.382 0.378

2	(0.623) 0.618 0.622

Locus 9764 :

2 alleles

99.2% missing data

2	(0.754) 0.757 0.747

4	(0.246) 0.243 0.253

Locus 9765 :

2 alleles

97.7% missing data

4	(0.630) 0.619 0.644

2	(0.370) 0.381 0.356

Locus 9766 :

2 alleles

84.4% missing data

2	(0.945) 0.948 0.953

3	(0.055) 0.052 0.047

Locus 9767 :

2 alleles

99.2% missing data

1	(0.535) 0.528 0.541

3	(0.465) 0.472 0.459

Locus 9768 :

2 alleles

98.8% missing data

3	(0.807) 0.816 0.801

1	(0.193) 0.184 0.199

Locus 9769 :

2 alleles

98.4% missing data

2	(0.687) 0.693 0.683

4	(0.313) 0.307 0.317

Locus 9770 :

2 alleles

99.2% missing data

4	(0.333) 0.335 0.336

1	(0.667) 0.665 0.664

Locus 9771 :

2 alleles

98.0% missing data

4	(0.601) 0.603 0.605

2	(0.399) 0.397 0.395

Locus 9772 :

2 alleles

99.2% missing data

2	(0.536) 0.539 0.534

4	(0.464) 0.461 0.466

Locus 9773 :

2 alleles

98.8% missing data

2	(0.330) 0.336 0.328

1	(0.670) 0.664 0.672

Locus 9774 :

2 alleles

0.8% missing data

1	(0.991) 0.986 0.997

4	(0.009) 0.014 0.003

Locus 9775 :

2 alleles

98.4% missing data

4	(0.529) 0.528 0.532

1	(0.471) 0.472 0.468

Locus 9776 :

2 alleles

99.2% missing data

4	(0.378) 0.376 0.386

3	(0.622) 0.624 0.614

Locus 9777 :

2 alleles

98.4% missing data

3	(0.430) 0.440 0.422

4	(0.570) 0.560 0.578

Locus 9778 :

2 alleles

99.2% missing data

1	(0.393) 0.396 0.393

2	(0.607) 0.604 0.607

Locus 9779 :

2 alleles

99.2% missing data

4	(0.313) 0.308 0.317

2	(0.687) 0.692 0.683

Locus 9780 :

2 alleles

99.2% missing data

2	(0.613) 0.602 0.618

1	(0.387) 0.398 0.382

Locus 9781 :

2 alleles

99.2% missing data

2	(0.365) 0.368 0.363

3	(0.635) 0.632 0.637

Locus 9782 :

2 alleles

99.2% missing data

4	(0.421) 0.424 0.423

2	(0.579) 0.576 0.577

Locus 9783 :

2 alleles

99.2% missing data

3	(0.371) 0.367 0.379

2	(0.629) 0.633 0.621

Locus 9784 :

2 alleles

98.4% missing data

1	(0.502) 0.511 0.503

4	(0.498) 0.489 0.497

Locus 9785 :

2 alleles

91.4% missing data

3	(0.920) 0.920 0.930

1	(0.080) 0.080 0.070

Locus 9786 :

2 alleles

98.4% missing data

4	(0.589) 0.597 0.586

2	(0.411) 0.403 0.414

Locus 9787 :

2 alleles

99.2% missing data

3	(0.434) 0.426 0.441

4	(0.566) 0.574 0.559

Locus 9788 :

4 alleles

98.4% missing data

1	(0.275) 0.278 0.270

2	(0.194) 0.187 0.194

4	(0.214) 0.216 0.217

3	(0.318) 0.319 0.319

Locus 9789 :

2 alleles

99.2% missing data

2	(0.457) 0.454 0.458

3	(0.543) 0.546 0.542

Locus 9790 :

2 alleles

99.2% missing data

3	(0.544) 0.549 0.549

4	(0.456) 0.451 0.451

Locus 9791 :

2 alleles

99.2% missing data

3	(0.759) 0.764 0.750

2	(0.241) 0.236 0.250

Locus 9792 :

2 alleles

96.9% missing data

2	(0.679) 0.669 0.691

4	(0.321) 0.331 0.309

Locus 9793 :

3 alleles

95.3% missing data

1	(0.125) 0.117 0.129

2	(0.736) 0.756 0.721

4	(0.139) 0.127 0.150

Locus 9794 :

2 alleles

97.3% missing data

3	(0.671) 0.671 0.685

4	(0.329) 0.329 0.315

Locus 9795 :

2 alleles

97.3% missing data

2	(0.545) 0.555 0.534

4	(0.455) 0.445 0.466

Locus 9796 :

2 alleles

99.2% missing data

1	(0.350) 0.357 0.348

2	(0.650) 0.643 0.652

Locus 9797 :

2 alleles

98.0% missing data

3	(0.871) 0.874 0.866

2	(0.129) 0.126 0.134

Locus 9798 :

2 alleles

99.2% missing data

4	(0.446) 0.436 0.449

2	(0.554) 0.564 0.551

Locus 9799 :

2 alleles

99.2% missing data

3	(0.451) 0.449 0.455

1	(0.549) 0.551 0.545

Locus 9800 :

2 alleles

93.0% missing data

1	(0.899) 0.899 0.904

2	(0.101) 0.101 0.096

Locus 9801 :

2 alleles

99.2% missing data

4	(0.410) 0.403 0.415

3	(0.590) 0.597 0.585

Locus 9802 :

2 alleles

98.8% missing data

4	(0.595) 0.593 0.598

2	(0.405) 0.407 0.402

Locus 9803 :

2 alleles

99.2% missing data

1	(0.672) 0.671 0.669

2	(0.328) 0.329 0.331

Locus 9804 :

2 alleles

99.2% missing data

2	(0.268) 0.260 0.270

3	(0.732) 0.740 0.730

Locus 9805 :

2 alleles

99.2% missing data

3	(0.423) 0.423 0.427

1	(0.577) 0.577 0.573

Locus 9806 :

2 alleles

98.4% missing data

2	(0.578) 0.569 0.592

3	(0.422) 0.431 0.408

Locus 9807 :

2 alleles

98.0% missing data

3	(0.644) 0.640 0.657

4	(0.356) 0.360 0.343

Locus 9808 :

2 alleles

99.2% missing data

2	(0.602) 0.599 0.604

4	(0.398) 0.401 0.396

Locus 9809 :

2 alleles

99.2% missing data

4	(0.676) 0.675 0.680

3	(0.324) 0.325 0.320

Locus 9810 :

2 alleles

96.1% missing data

2	(0.905) 0.915 0.898

1	(0.095) 0.085 0.102

Locus 9811 :

2 alleles

97.7% missing data

1	(0.573) 0.574 0.567

4	(0.427) 0.426 0.433

Locus 9812 :

2 alleles

99.2% missing data

1	(0.719) 0.714 0.723

3	(0.281) 0.286 0.277

Locus 9813 :

2 alleles

97.7% missing data

1	(0.215) 0.223 0.202

3	(0.785) 0.777 0.798

Locus 9814 :

2 alleles

99.2% missing data

2	(0.753) 0.752 0.755

1	(0.247) 0.248 0.245

Locus 9815 :

2 alleles

99.2% missing data

3	(0.270) 0.276 0.273

2	(0.730) 0.724 0.727

Locus 9816 :

2 alleles

99.2% missing data

4	(0.493) 0.494 0.489

2	(0.507) 0.506 0.511

Locus 9817 :

2 alleles

99.2% missing data

2	(0.304) 0.304 0.305

3	(0.696) 0.696 0.695

Locus 9818 :

2 alleles

99.2% missing data

3	(0.295) 0.294 0.297

4	(0.705) 0.706 0.703

Locus 9819 :

2 alleles

96.9% missing data

1	(0.460) 0.460 0.458

3	(0.540) 0.540 0.542

Locus 9820 :

2 alleles

99.2% missing data

4	(0.683) 0.679 0.682

3	(0.317) 0.321 0.318

Locus 9821 :

2 alleles

97.7% missing data

2	(0.287) 0.285 0.278

4	(0.713) 0.715 0.722

Locus 9822 :

2 alleles

97.3% missing data

4	(0.855) 0.854 0.856

1	(0.145) 0.146 0.144

Locus 9823 :

2 alleles

99.2% missing data

3	(0.660) 0.664 0.656

4	(0.340) 0.336 0.344

Locus 9824 :

2 alleles

99.2% missing data

2	(0.453) 0.451 0.458

3	(0.547) 0.549 0.542

Locus 9825 :

2 alleles

97.7% missing data

4	(0.807) 0.812 0.801

1	(0.193) 0.188 0.199

Locus 9826 :

2 alleles

98.4% missing data

4	(0.766) 0.772 0.766

1	(0.234) 0.228 0.234

Locus 9827 :

2 alleles

99.2% missing data

3	(0.175) 0.170 0.183

2	(0.825) 0.830 0.817

Locus 9828 :

2 alleles

93.0% missing data

2	(0.909) 0.902 0.923

3	(0.091) 0.098 0.077

Locus 9829 :

2 alleles

98.8% missing data

4	(0.581) 0.579 0.587

1	(0.419) 0.421 0.413

Locus 9830 :

2 alleles

99.2% missing data

4	(0.223) 0.223 0.228

1	(0.777) 0.777 0.772

Locus 9831 :

2 alleles

99.2% missing data

2	(0.677) 0.682 0.672

3	(0.323) 0.318 0.328

Locus 9832 :

2 alleles

99.2% missing data

2	(0.613) 0.605 0.616

4	(0.387) 0.395 0.384

Locus 9833 :

2 alleles

98.4% missing data

4	(0.681) 0.691 0.678

1	(0.319) 0.309 0.322

Locus 9834 :

2 alleles

97.3% missing data

4	(0.782) 0.784 0.787

2	(0.218) 0.216 0.213

Locus 9835 :

2 alleles

96.1% missing data

4	(0.852) 0.855 0.857

2	(0.148) 0.145 0.143

Locus 9836 :

2 alleles

99.2% missing data

3	(0.500) 0.499 0.503

1	(0.500) 0.501 0.497

Locus 9837 :

2 alleles

99.2% missing data

2	(0.569) 0.569 0.576

3	(0.431) 0.431 0.424

Locus 9838 :

2 alleles

98.8% missing data

4	(0.570) 0.565 0.578

1	(0.430) 0.435 0.422

Locus 9839 :

2 alleles

99.2% missing data

3	(0.561) 0.565 0.554

4	(0.439) 0.435 0.446

Locus 9840 :

2 alleles

90.2% missing data

4	(0.930) 0.940 0.929

3	(0.070) 0.060 0.071

Locus 9841 :

2 alleles

99.2% missing data

2	(0.675) 0.682 0.672

1	(0.325) 0.318 0.328

Locus 9842 :

2 alleles

99.2% missing data

2	(0.305) 0.303 0.316

4	(0.695) 0.697 0.684

Locus 9843 :

2 alleles

99.2% missing data

1	(0.405) 0.411 0.402

3	(0.595) 0.589 0.598

Locus 9844 :

2 alleles

90.2% missing data

2	(0.914) 0.926 0.910

4	(0.086) 0.074 0.090

Locus 9845 :

2 alleles

99.2% missing data

4	(0.563) 0.561 0.570

1	(0.437) 0.439 0.430

Locus 9846 :

2 alleles

99.2% missing data

4	(0.138) 0.135 0.141

1	(0.862) 0.865 0.859

Locus 9847 :

2 alleles

99.2% missing data

2	(0.438) 0.435 0.441

4	(0.562) 0.565 0.559

Locus 9848 :

2 alleles

98.4% missing data

3	(0.732) 0.734 0.726

1	(0.268) 0.266 0.274

Locus 9849 :

2 alleles

95.7% missing data

1	(0.164) 0.159 0.162

2	(0.836) 0.841 0.838

Locus 9850 :

2 alleles

96.1% missing data

2	(0.855) 0.864 0.850

3	(0.145) 0.136 0.150

Locus 9851 :

3 alleles

98.0% missing data

2	(0.462) 0.480 0.455

4	(0.326) 0.318 0.330

1	(0.212) 0.202 0.215

Locus 9852 :

2 alleles

0.0% missing data

4	(0.587) 0.488 0.691

1	(0.413) 0.512 0.309

Locus 9853 :

2 alleles

98.0% missing data

3	(0.560) 0.567 0.566

2	(0.440) 0.433 0.434

Locus 9854 :

2 alleles

99.2% missing data

4	(0.191) 0.191 0.194

1	(0.809) 0.809 0.806

Locus 9855 :

2 alleles

98.4% missing data

2	(0.464) 0.464 0.476

4	(0.536) 0.536 0.524

Locus 9856 :

2 alleles

96.1% missing data

4	(0.203) 0.190 0.204

2	(0.797) 0.810 0.796

Locus 9857 :

2 alleles

98.0% missing data

3	(0.561) 0.558 0.566

4	(0.439) 0.442 0.434

Locus 9858 :

2 alleles

99.2% missing data

4	(0.611) 0.613 0.610

3	(0.389) 0.387 0.390

Locus 9859 :

2 alleles

0.0% missing data

2	(0.983) 0.987 0.992

3	(0.017) 0.013 0.008

Locus 9860 :

2 alleles

99.2% missing data

4	(0.677) 0.683 0.667

3	(0.323) 0.317 0.333

Locus 9861 :

2 alleles

99.2% missing data

3	(0.634) 0.625 0.638

2	(0.366) 0.375 0.362

Locus 9862 :

2 alleles

99.2% missing data

4	(0.587) 0.591 0.584

3	(0.413) 0.409 0.416

Locus 9863 :

2 alleles

99.2% missing data

3	(0.482) 0.477 0.484

2	(0.518) 0.523 0.516

Locus 9864 :

2 alleles

90.6% missing data

2	(0.909) 0.921 0.903

4	(0.091) 0.079 0.097

Locus 9865 :

2 alleles

98.8% missing data

2	(0.392) 0.386 0.389

4	(0.608) 0.614 0.611

Locus 9866 :

2 alleles

92.6% missing data

2	(0.620) 0.619 0.628

3	(0.380) 0.381 0.372

Locus 9867 :

2 alleles

99.2% missing data

4	(0.614) 0.617 0.611

2	(0.386) 0.383 0.389

Locus 9868 :

2 alleles

0.0% missing data

2	(0.981) 0.981 0.990

1	(0.019) 0.019 0.010

Locus 9869 :

2 alleles

91.4% missing data

2	(0.931) 0.944 0.924

3	(0.069) 0.056 0.076

Locus 9870 :

2 alleles

98.8% missing data

3	(0.451) 0.457 0.446

2	(0.549) 0.543 0.554

Locus 9871 :

2 alleles

99.2% missing data

2	(0.332) 0.325 0.338

4	(0.668) 0.675 0.662

Locus 9872 :

2 alleles

99.2% missing data

4	(0.642) 0.633 0.648

2	(0.358) 0.367 0.352

Locus 9873 :

2 alleles

99.2% missing data

4	(0.187) 0.185 0.193

1	(0.813) 0.815 0.807

Locus 9874 :

2 alleles

99.2% missing data

1	(0.469) 0.467 0.478

4	(0.531) 0.533 0.522

Locus 9875 :

2 alleles

98.8% missing data

3	(0.877) 0.881 0.876

2	(0.123) 0.119 0.124

Locus 9876 :

2 alleles

99.2% missing data

4	(0.584) 0.578 0.585

2	(0.416) 0.422 0.415

Locus 9877 :

2 alleles

99.2% missing data

3	(0.542) 0.542 0.537

1	(0.458) 0.458 0.463

Locus 9878 :

2 alleles

96.5% missing data

1	(0.169) 0.164 0.173

4	(0.831) 0.836 0.827

Locus 9879 :

2 alleles

99.2% missing data

2	(0.391) 0.388 0.393

1	(0.609) 0.612 0.607

Locus 9880 :

2 alleles

99.2% missing data

4	(0.590) 0.591 0.585

3	(0.410) 0.409 0.415

Locus 9881 :

2 alleles

99.2% missing data

4	(0.283) 0.273 0.290

3	(0.717) 0.727 0.710

Locus 9882 :

2 alleles

8.2% missing data

1	(0.986) 0.978 0.996

4	(0.014) 0.022 0.004

Locus 9883 :

2 alleles

98.8% missing data

3	(0.374) 0.377 0.381

2	(0.626) 0.623 0.619

Locus 9884 :

2 alleles

0.0% missing data

2	(0.986) 0.982 0.996

4	(0.014) 0.018 0.004

Locus 9885 :

3 alleles

98.8% missing data

4	(0.412) 0.408 0.411

1	(0.202) 0.199 0.209

2	(0.386) 0.393 0.381

Locus 9886 :

2 alleles

99.2% missing data

3	(0.577) 0.581 0.570

2	(0.423) 0.419 0.430

Locus 9887 :

2 alleles

99.2% missing data

4	(0.742) 0.746 0.740

2	(0.258) 0.254 0.260

Locus 9888 :

2 alleles

99.2% missing data

1	(0.307) 0.306 0.314

2	(0.693) 0.694 0.686

Locus 9889 :

2 alleles

98.8% missing data

1	(0.286) 0.283 0.294

4	(0.714) 0.717 0.706

Locus 9890 :

2 alleles

99.2% missing data

4	(0.343) 0.347 0.339

3	(0.657) 0.653 0.661

Locus 9891 :

2 alleles

98.8% missing data

2	(0.646) 0.646 0.647

4	(0.354) 0.354 0.353

Locus 9892 :

2 alleles

98.4% missing data

2	(0.304) 0.309 0.301

1	(0.696) 0.691 0.699

Locus 9893 :

2 alleles

99.2% missing data

2	(0.695) 0.692 0.698

3	(0.305) 0.308 0.302

Locus 9894 :

2 alleles

98.8% missing data

3	(0.829) 0.825 0.831

4	(0.171) 0.175 0.169

Locus 9895 :

2 alleles

99.2% missing data

4	(0.421) 0.428 0.417

2	(0.579) 0.572 0.583

Locus 9896 :

2 alleles

99.2% missing data

3	(0.676) 0.669 0.678

4	(0.324) 0.331 0.322

Locus 9897 :

2 alleles

99.2% missing data

2	(0.246) 0.241 0.254

3	(0.754) 0.759 0.746

Locus 9898 :

2 alleles

99.2% missing data

4	(0.365) 0.367 0.364

3	(0.635) 0.633 0.636

Locus 9899 :

2 alleles

9.0% missing data

2	(0.985) 0.988 0.992

3	(0.015) 0.012 0.008

Locus 9900 :

2 alleles

91.4% missing data

2	(0.884) 0.904 0.870

4	(0.116) 0.096 0.130

Locus 9901 :

2 alleles

96.9% missing data

2	(0.415) 0.428 0.407

4	(0.585) 0.572 0.593

Locus 9902 :

2 alleles

98.8% missing data

3	(0.572) 0.575 0.571

4	(0.428) 0.425 0.429

Locus 9903 :

3 alleles

98.8% missing data

1	(0.516) 0.514 0.513

3	(0.366) 0.369 0.363

4	(0.118) 0.117 0.124

Locus 9904 :

2 alleles

98.8% missing data

4	(0.626) 0.617 0.637

1	(0.374) 0.383 0.363

Locus 9905 :

2 alleles

89.8% missing data

2	(0.893) 0.916 0.884

1	(0.107) 0.084 0.116

Locus 9906 :

2 alleles

99.2% missing data

4	(0.600) 0.599 0.595

3	(0.400) 0.401 0.405

Locus 9907 :

2 alleles

99.2% missing data

2	(0.333) 0.334 0.331

3	(0.667) 0.666 0.669

Locus 9908 :

2 alleles

99.2% missing data

2	(0.643) 0.644 0.642

4	(0.357) 0.356 0.358

Locus 9909 :

2 alleles

91.0% missing data

1	(0.915) 0.925 0.912

4	(0.085) 0.075 0.088

Locus 9910 :

2 alleles

99.2% missing data

1	(0.418) 0.416 0.419

2	(0.582) 0.584 0.581

Locus 9911 :

2 alleles

99.2% missing data

4	(0.344) 0.339 0.350

1	(0.656) 0.661 0.650

Locus 9912 :

2 alleles

91.0% missing data

1	(0.928) 0.942 0.916

2	(0.072) 0.058 0.084

Locus 9913 :

2 alleles

99.2% missing data

3	(0.485) 0.481 0.491

2	(0.515) 0.519 0.509

Locus 9914 :

2 alleles

99.2% missing data

2	(0.627) 0.616 0.629

3	(0.373) 0.384 0.371

Locus 9915 :

2 alleles

99.2% missing data

2	(0.530) 0.529 0.538

4	(0.470) 0.471 0.462

Locus 9916 :

2 alleles

99.2% missing data

1	(0.549) 0.540 0.547

4	(0.451) 0.460 0.453

Locus 9917 :

2 alleles

39.1% missing data

3	(0.979) 0.973 0.992

1	(0.021) 0.027 0.008

Locus 9918 :

2 alleles

99.2% missing data

1	(0.361) 0.360 0.367

4	(0.639) 0.640 0.633

Locus 9919 :

2 alleles

98.8% missing data

3	(0.538) 0.540 0.538

1	(0.462) 0.460 0.462

Locus 9920 :

2 alleles

99.2% missing data

4	(0.705) 0.701 0.703

2	(0.295) 0.299 0.297

Locus 9921 :

2 alleles

98.8% missing data

4	(0.835) 0.842 0.829

3	(0.165) 0.158 0.171

Locus 9922 :

2 alleles

99.2% missing data

4	(0.550) 0.552 0.546

3	(0.450) 0.448 0.454

Locus 9923 :

2 alleles

99.2% missing data

2	(0.594) 0.592 0.597

4	(0.406) 0.408 0.403

Locus 9924 :

3 alleles

98.4% missing data

4	(0.370) 0.373 0.372

2	(0.258) 0.260 0.254

1	(0.372) 0.368 0.374

Locus 9925 :

3 alleles

89.8% missing data

3	(0.825) 0.857 0.808

1	(0.070) 0.059 0.078

4	(0.105) 0.084 0.113

Locus 9926 :

2 alleles

98.8% missing data

4	(0.329) 0.330 0.328

1	(0.671) 0.670 0.672

Locus 9927 :

2 alleles

99.2% missing data

3	(0.235) 0.237 0.231

2	(0.765) 0.763 0.769

Locus 9928 :

2 alleles

96.1% missing data

3	(0.874) 0.881 0.868

2	(0.126) 0.119 0.132

Locus 9929 :

2 alleles

99.2% missing data

1	(0.784) 0.786 0.776

2	(0.216) 0.214 0.224

Locus 9930 :

2 alleles

99.2% missing data

2	(0.598) 0.605 0.594

4	(0.402) 0.395 0.406

Locus 9931 :

2 alleles

98.8% missing data

4	(0.620) 0.623 0.618

1	(0.380) 0.377 0.382

Locus 9932 :

2 alleles

99.2% missing data

1	(0.458) 0.458 0.459

3	(0.542) 0.542 0.541

Locus 9933 :

3 alleles

98.4% missing data

4	(0.542) 0.550 0.535

1	(0.328) 0.321 0.328

3	(0.130) 0.129 0.137

Locus 9934 :

2 alleles

98.4% missing data

1	(0.734) 0.742 0.727

4	(0.266) 0.258 0.273

Locus 9935 :

2 alleles

98.8% missing data

2	(0.729) 0.721 0.737

3	(0.271) 0.279 0.263

Locus 9936 :

2 alleles

99.2% missing data

4	(0.436) 0.433 0.437

2	(0.564) 0.567 0.563

Locus 9937 :

2 alleles

99.2% missing data

4	(0.417) 0.418 0.416

2	(0.583) 0.582 0.584

Locus 9938 :

2 alleles

99.2% missing data

4	(0.404) 0.398 0.412

3	(0.596) 0.602 0.588

Locus 9939 :

2 alleles

0.0% missing data

4	(0.984) 0.981 0.995

1	(0.016) 0.019 0.005

Locus 9940 :

2 alleles

99.2% missing data

3	(0.393) 0.401 0.388

2	(0.607) 0.599 0.612

Locus 9941 :

2 alleles

99.2% missing data

3	(0.508) 0.507 0.511

4	(0.492) 0.493 0.489

Locus 9942 :

2 alleles

9.4% missing data

2	(0.977) 0.961 0.993

4	(0.023) 0.039 0.007

Locus 9943 :

2 alleles

0.4% missing data

1	(0.989) 0.985 0.996

4	(0.011) 0.015 0.004

Locus 9944 :

2 alleles

99.2% missing data

2	(0.672) 0.667 0.674

1	(0.328) 0.333 0.326

Locus 9945 :

2 alleles

0.4% missing data

4	(0.984) 0.979 0.995

1	(0.016) 0.021 0.005

Locus 9946 :

2 alleles

95.3% missing data

2	(0.680) 0.690 0.679

3	(0.320) 0.310 0.321

Locus 9947 :

2 alleles

92.6% missing data

4	(0.926) 0.925 0.930

2	(0.074) 0.075 0.070

Locus 9948 :

2 alleles

99.2% missing data

4	(0.412) 0.407 0.412

3	(0.588) 0.593 0.588

Locus 9949 :

2 alleles

96.9% missing data

1	(0.901) 0.902 0.902

3	(0.099) 0.098 0.098

Locus 9950 :

2 alleles

91.0% missing data

2	(0.901) 0.919 0.891

3	(0.099) 0.081 0.109

Locus 9951 :

2 alleles

99.2% missing data

2	(0.397) 0.402 0.395

4	(0.603) 0.598 0.605

Locus 9952 :

2 alleles

90.2% missing data

4	(0.925) 0.940 0.916

3	(0.075) 0.060 0.084

Locus 9953 :

2 alleles

99.2% missing data

4	(0.235) 0.227 0.239

3	(0.765) 0.773 0.761

Locus 9954 :

2 alleles

99.2% missing data

4	(0.404) 0.405 0.400

2	(0.596) 0.595 0.600

Locus 9955 :

2 alleles

99.2% missing data

1	(0.613) 0.616 0.611

4	(0.387) 0.384 0.389

Locus 9956 :

2 alleles

98.0% missing data

1	(0.225) 0.224 0.222

4	(0.775) 0.776 0.778

Locus 9957 :

2 alleles

91.0% missing data

4	(0.931) 0.943 0.922

2	(0.069) 0.057 0.078

Locus 9958 :

2 alleles

94.9% missing data

4	(0.220) 0.216 0.209

1	(0.780) 0.784 0.791

Locus 9959 :

2 alleles

99.2% missing data

1	(0.407) 0.405 0.414

3	(0.593) 0.595 0.586

Locus 9960 :

2 alleles

94.1% missing data

4	(0.858) 0.848 0.878

3	(0.142) 0.152 0.122

Locus 9961 :

2 alleles

99.2% missing data

4	(0.495) 0.494 0.498

3	(0.505) 0.506 0.502

Locus 9962 :

2 alleles

99.2% missing data

4	(0.378) 0.372 0.379

1	(0.622) 0.628 0.621

Locus 9963 :

2 alleles

99.2% missing data

1	(0.209) 0.206 0.215

3	(0.791) 0.794 0.785

Locus 9964 :

3 alleles

98.4% missing data

1	(0.463) 0.456 0.474

4	(0.117) 0.114 0.123

3	(0.420) 0.431 0.403

Locus 9965 :

2 alleles

99.2% missing data

1	(0.611) 0.605 0.622

2	(0.389) 0.395 0.378

Locus 9966 :

2 alleles

99.2% missing data

3	(0.799) 0.805 0.793

2	(0.201) 0.195 0.207

Locus 9967 :

2 alleles

98.8% missing data

2	(0.414) 0.408 0.420

3	(0.586) 0.592 0.580

Locus 9968 :

2 alleles

99.2% missing data

2	(0.685) 0.686 0.684

1	(0.315) 0.314 0.316

Locus 9969 :

2 alleles

99.2% missing data

2	(0.431) 0.433 0.437

4	(0.569) 0.567 0.563

Locus 9970 :

2 alleles

99.2% missing data

3	(0.307) 0.320 0.304

1	(0.693) 0.680 0.696

Locus 9971 :

2 alleles

98.8% missing data

2	(0.650) 0.657 0.643

3	(0.350) 0.343 0.357

Locus 9972 :

2 alleles

94.1% missing data

2	(0.910) 0.911 0.914

3	(0.090) 0.089 0.086

Locus 9973 :

2 alleles

99.2% missing data

3	(0.399) 0.394 0.404

2	(0.601) 0.606 0.596

Locus 9974 :

2 alleles

99.2% missing data

1	(0.404) 0.408 0.407

4	(0.596) 0.592 0.593

Locus 9975 :

2 alleles

92.6% missing data

3	(0.891) 0.892 0.900

1	(0.109) 0.108 0.100

Locus 9976 :

2 alleles

99.2% missing data

2	(0.798) 0.795 0.805

4	(0.202) 0.205 0.195

Locus 9977 :

3 alleles

90.2% missing data

4	(0.898) 0.911 0.891

2	(0.047) 0.045 0.047

1	(0.055) 0.044 0.063

Locus 9978 :

2 alleles

99.2% missing data

3	(0.170) 0.171 0.175

2	(0.830) 0.829 0.825

Locus 9979 :

3 alleles

96.9% missing data

1	(0.121) 0.117 0.117

2	(0.256) 0.260 0.253

3	(0.623) 0.622 0.629

Locus 9980 :

2 alleles

99.2% missing data

3	(0.845) 0.839 0.848

1	(0.155) 0.161 0.152

Locus 9981 :

2 alleles

99.2% missing data

4	(0.719) 0.725 0.712

3	(0.281) 0.275 0.288

Locus 9982 :

2 alleles

97.7% missing data

3	(0.696) 0.701 0.705

4	(0.304) 0.299 0.295

Locus 9983 :

2 alleles

98.8% missing data

3	(0.623) 0.622 0.629

1	(0.377) 0.378 0.371

Locus 9984 :

2 alleles

99.2% missing data

2	(0.531) 0.536 0.529

3	(0.469) 0.464 0.471

Locus 9985 :

2 alleles

99.2% missing data

2	(0.516) 0.508 0.520

4	(0.484) 0.492 0.480

Locus 9986 :

2 alleles

98.0% missing data

1	(0.648) 0.652 0.651

3	(0.352) 0.348 0.349

Locus 9987 :

2 alleles

99.2% missing data

4	(0.579) 0.578 0.588

2	(0.421) 0.422 0.412

Locus 9988 :

3 alleles

98.8% missing data

3	(0.402) 0.399 0.408

1	(0.378) 0.378 0.375

2	(0.220) 0.223 0.218

Locus 9989 :

2 alleles

99.2% missing data

4	(0.598) 0.605 0.593

2	(0.402) 0.395 0.407

Locus 9990 :

2 alleles

99.2% missing data

2	(0.720) 0.719 0.718

3	(0.280) 0.281 0.282

Locus 9991 :

2 alleles

99.2% missing data

4	(0.631) 0.632 0.633

2	(0.369) 0.368 0.367

Locus 9992 :

2 alleles

8.6% missing data

3	(0.986) 0.978 0.996

1	(0.014) 0.022 0.004

Locus 9993 :

2 alleles

99.2% missing data

1	(0.562) 0.551 0.565

3	(0.438) 0.449 0.435

Locus 9994 :

2 alleles

99.2% missing data

1	(0.411) 0.413 0.409

3	(0.589) 0.587 0.591

Locus 9995 :

2 alleles

99.2% missing data

1	(0.549) 0.551 0.550

4	(0.451) 0.449 0.450

Locus 9996 :

2 alleles

0.4% missing data

4	(0.984) 0.987 0.993

3	(0.016) 0.013 0.007

Locus 9997 :

2 alleles

98.8% missing data

3	(0.536) 0.540 0.540

4	(0.464) 0.460 0.460

Locus 9998 :

2 alleles

96.9% missing data

3	(0.316) 0.315 0.315

1	(0.684) 0.685 0.685

Locus 9999 :

2 alleles

99.2% missing data

4	(0.687) 0.692 0.685

1	(0.313) 0.308 0.315

Locus 10000 :

2 alleles

99.2% missing data

3	(0.556) 0.553 0.554

1	(0.444) 0.447 0.446

Locus 10001 :

2 alleles

97.7% missing data

3	(0.563) 0.566 0.572

2	(0.437) 0.434 0.428

Locus 10002 :

2 alleles

0.0% missing data

2	(0.988) 0.992 0.994

3	(0.012) 0.008 0.006

Locus 10003 :

3 alleles

98.8% missing data

4	(0.262) 0.257 0.270

3	(0.399) 0.395 0.400

1	(0.339) 0.348 0.330

Locus 10004 :

2 alleles

99.2% missing data

4	(0.473) 0.466 0.478

1	(0.527) 0.534 0.522

Locus 10005 :

2 alleles

98.8% missing data

3	(0.547) 0.552 0.545

1	(0.453) 0.448 0.455

Locus 10006 :

2 alleles

97.7% missing data

2	(0.297) 0.298 0.291

4	(0.703) 0.702 0.709

Locus 10007 :

2 alleles

99.2% missing data

1	(0.486) 0.491 0.483

3	(0.514) 0.509 0.517

Locus 10008 :

2 alleles

99.2% missing data

4	(0.487) 0.485 0.498

1	(0.513) 0.515 0.502

Locus 10009 :

2 alleles

98.0% missing data

3	(0.682) 0.695 0.685

2	(0.318) 0.305 0.315

Locus 10010 :

2 alleles

99.2% missing data

3	(0.444) 0.441 0.451

4	(0.556) 0.559 0.549

Locus 10011 :

2 alleles

97.7% missing data

1	(0.210) 0.213 0.214

2	(0.790) 0.787 0.786

Locus 10012 :

2 alleles

99.2% missing data

2	(0.310) 0.310 0.317

3	(0.690) 0.690 0.683

Locus 10013 :

2 alleles

99.2% missing data

3	(0.401) 0.397 0.406

4	(0.599) 0.603 0.594

Locus 10014 :

2 alleles

98.4% missing data

2	(0.680) 0.687 0.680

4	(0.320) 0.313 0.320

Locus 10015 :

2 alleles

99.2% missing data

3	(0.620) 0.618 0.618

1	(0.380) 0.382 0.382

Locus 10016 :

2 alleles

99.2% missing data

3	(0.483) 0.484 0.481

4	(0.517) 0.516 0.519

Locus 10017 :

2 alleles

99.2% missing data

4	(0.315) 0.317 0.314

3	(0.685) 0.683 0.686

Locus 10018 :

2 alleles

98.8% missing data

2	(0.407) 0.396 0.408

3	(0.593) 0.604 0.592

Locus 10019 :

2 alleles

85.9% missing data

4	(0.939) 0.938 0.948

2	(0.061) 0.062 0.052

Locus 10020 :

2 alleles

97.7% missing data

3	(0.466) 0.476 0.468

1	(0.534) 0.524 0.532

Locus 10021 :

2 alleles

71.1% missing data

4	(0.970) 0.974 0.975

2	(0.030) 0.026 0.025

Locus 10022 :

2 alleles

99.2% missing data

2	(0.678) 0.673 0.682

1	(0.322) 0.327 0.318

Locus 10023 :

2 alleles

98.8% missing data

3	(0.687) 0.689 0.691

4	(0.313) 0.311 0.309

Locus 10024 :

2 alleles

99.2% missing data

3	(0.297) 0.299 0.303

1	(0.703) 0.701 0.697

Locus 10025 :

2 alleles

98.4% missing data

2	(0.750) 0.755 0.744

1	(0.250) 0.245 0.256

Locus 10026 :

2 alleles

99.2% missing data

3	(0.386) 0.380 0.389

4	(0.614) 0.620 0.611

Locus 10027 :

2 alleles

98.8% missing data

4	(0.610) 0.607 0.613

3	(0.390) 0.393 0.387

Locus 10028 :

2 alleles

99.2% missing data

1	(0.451) 0.450 0.447

2	(0.549) 0.550 0.553

Locus 10029 :

3 alleles

98.8% missing data

1	(0.410) 0.410 0.415

3	(0.207) 0.215 0.197

4	(0.383) 0.375 0.388

Locus 10030 :

2 alleles

93.0% missing data

1	(0.860) 0.857 0.874

3	(0.140) 0.143 0.126

Locus 10031 :

2 alleles

99.2% missing data

2	(0.757) 0.757 0.755

3	(0.243) 0.243 0.245

Locus 10032 :

2 alleles

99.2% missing data

2	(0.728) 0.721 0.733

1	(0.272) 0.279 0.267

Locus 10033 :

2 alleles

89.8% missing data

3	(0.935) 0.940 0.938

4	(0.065) 0.060 0.062

Locus 10034 :

2 alleles

99.2% missing data

1	(0.424) 0.431 0.423

2	(0.576) 0.569 0.577

Locus 10035 :

2 alleles

99.2% missing data

3	(0.543) 0.546 0.542

4	(0.457) 0.454 0.458

Locus 10036 :

2 alleles

99.2% missing data

2	(0.515) 0.517 0.511

3	(0.485) 0.483 0.489

Locus 10037 :

3 alleles

98.8% missing data

2	(0.466) 0.468 0.459

1	(0.353) 0.357 0.354

4	(0.181) 0.175 0.187

Locus 10038 :

2 alleles

0.4% missing data

2	(0.986) 0.990 0.993

3	(0.014) 0.010 0.007

Locus 10039 :

2 alleles

92.6% missing data

1	(0.931) 0.925 0.941

2	(0.069) 0.075 0.059

Locus 10040 :

2 alleles

99.2% missing data

1	(0.433) 0.433 0.429

4	(0.567) 0.567 0.571

Locus 10041 :

2 alleles

98.8% missing data

2	(0.395) 0.391 0.406

3	(0.605) 0.609 0.594

Locus 10042 :

3 alleles

98.4% missing data

3	(0.400) 0.402 0.399

4	(0.413) 0.410 0.409

1	(0.187) 0.188 0.192

Locus 10043 :

2 alleles

99.2% missing data

4	(0.491) 0.498 0.490

3	(0.509) 0.502 0.510

Locus 10044 :

2 alleles

99.2% missing data

3	(0.539) 0.537 0.549

1	(0.461) 0.463 0.451

Locus 10045 :

2 alleles

99.2% missing data

3	(0.290) 0.293 0.288

4	(0.710) 0.707 0.712

Locus 10046 :

2 alleles

99.2% missing data

4	(0.261) 0.257 0.261

3	(0.739) 0.743 0.739

Locus 10047 :

2 alleles

98.0% missing data

3	(0.580) 0.596 0.585

4	(0.420) 0.404 0.415

Locus 10048 :

2 alleles

99.2% missing data

4	(0.491) 0.489 0.496

1	(0.509) 0.511 0.504

Locus 10049 :

2 alleles

99.2% missing data

2	(0.512) 0.508 0.514

3	(0.488) 0.492 0.486

Locus 10050 :

3 alleles

98.8% missing data

3	(0.366) 0.369 0.366

4	(0.318) 0.316 0.317

1	(0.316) 0.315 0.317

Locus 10051 :

2 alleles

98.8% missing data

2	(0.493) 0.488 0.497

4	(0.507) 0.512 0.503

Locus 10052 :

2 alleles

96.9% missing data

2	(0.556) 0.567 0.558

4	(0.444) 0.433 0.442

Locus 10053 :

3 alleles

98.8% missing data

2	(0.443) 0.447 0.434

4	(0.259) 0.258 0.266

1	(0.298) 0.295 0.300

Locus 10054 :

2 alleles

99.2% missing data

4	(0.671) 0.671 0.665

2	(0.329) 0.329 0.335

Locus 10055 :

2 alleles

99.2% missing data

2	(0.319) 0.315 0.324

4	(0.681) 0.685 0.676

Locus 10056 :

2 alleles

99.2% missing data

2	(0.223) 0.217 0.225

1	(0.777) 0.783 0.775

Locus 10057 :

2 alleles

98.0% missing data

1	(0.448) 0.437 0.453

2	(0.552) 0.563 0.547

Locus 10058 :

2 alleles

98.0% missing data

1	(0.479) 0.468 0.483

3	(0.521) 0.532 0.517

Locus 10059 :

2 alleles

98.0% missing data

4	(0.748) 0.744 0.754

3	(0.252) 0.256 0.246

Locus 10060 :

2 alleles

99.2% missing data

3	(0.450) 0.447 0.455

1	(0.550) 0.553 0.545

Locus 10061 :

2 alleles

99.2% missing data

3	(0.373) 0.373 0.381

1	(0.627) 0.627 0.619

Locus 10062 :

2 alleles

97.7% missing data

2	(0.119) 0.113 0.120

4	(0.881) 0.887 0.880

Locus 10063 :

2 alleles

99.2% missing data

2	(0.347) 0.345 0.349

1	(0.653) 0.655 0.651

Locus 10064 :

2 alleles

99.2% missing data

1	(0.661) 0.660 0.657

4	(0.339) 0.340 0.343

Locus 10065 :

2 alleles

99.2% missing data

2	(0.315) 0.309 0.316

3	(0.685) 0.691 0.684

Locus 10066 :

2 alleles

98.8% missing data

4	(0.489) 0.487 0.495

1	(0.511) 0.513 0.505

Locus 10067 :

2 alleles

99.2% missing data

1	(0.747) 0.751 0.741

2	(0.253) 0.249 0.259

Locus 10068 :

2 alleles

98.8% missing data

1	(0.736) 0.731 0.743

3	(0.264) 0.269 0.257

Locus 10069 :

2 alleles

91.0% missing data

1	(0.917) 0.933 0.906

2	(0.083) 0.067 0.094

Locus 10070 :

2 alleles

96.5% missing data

3	(0.746) 0.743 0.754

4	(0.254) 0.257 0.246

Locus 10071 :

2 alleles

96.1% missing data

2	(0.823) 0.835 0.816

4	(0.177) 0.165 0.184

Locus 10072 :

2 alleles

99.2% missing data

3	(0.432) 0.432 0.431

1	(0.568) 0.568 0.569

Locus 10073 :

2 alleles

99.2% missing data

4	(0.367) 0.367 0.372

2	(0.633) 0.633 0.628

Locus 10074 :

2 alleles

99.2% missing data

3	(0.606) 0.607 0.605

2	(0.394) 0.393 0.395

Locus 10075 :

2 alleles

91.0% missing data

2	(0.908) 0.914 0.912

1	(0.092) 0.086 0.088

Locus 10076 :

2 alleles

99.2% missing data

1	(0.674) 0.670 0.676

3	(0.326) 0.330 0.324

Locus 10077 :

2 alleles

98.0% missing data

2	(0.701) 0.710 0.705

1	(0.299) 0.290 0.295

Locus 10078 :

2 alleles

98.4% missing data

3	(0.704) 0.714 0.695

1	(0.296) 0.286 0.305

Locus 10079 :

2 alleles

99.2% missing data

3	(0.684) 0.678 0.684

2	(0.316) 0.322 0.316

Locus 10080 :

2 alleles

99.2% missing data

4	(0.552) 0.552 0.546

2	(0.448) 0.448 0.454

Locus 10081 :

2 alleles

98.8% missing data

1	(0.766) 0.770 0.758

2	(0.234) 0.230 0.242

Locus 10082 :

2 alleles

99.2% missing data

3	(0.477) 0.474 0.480

1	(0.523) 0.526 0.520

Locus 10083 :

2 alleles

99.2% missing data

4	(0.485) 0.480 0.486

1	(0.515) 0.520 0.514

Locus 10084 :

2 alleles

99.2% missing data

4	(0.313) 0.319 0.317

1	(0.687) 0.681 0.683

Locus 10085 :

2 alleles

8.2% missing data

2	(0.983) 0.986 0.991

1	(0.017) 0.014 0.009

Locus 10086 :

2 alleles

99.2% missing data

3	(0.189) 0.186 0.195

1	(0.811) 0.814 0.805

Locus 10087 :

2 alleles

99.2% missing data

2	(0.625) 0.620 0.631

4	(0.375) 0.380 0.369

Locus 10088 :

2 alleles

98.4% missing data

1	(0.717) 0.715 0.716

2	(0.283) 0.285 0.284

Locus 10089 :

2 alleles

0.4% missing data

3	(0.988) 0.992 0.994

2	(0.012) 0.008 0.006

Locus 10090 :

2 alleles

99.2% missing data

3	(0.246) 0.248 0.259

4	(0.754) 0.752 0.741

Locus 10091 :

2 alleles

97.7% missing data

3	(0.694) 0.690 0.706

1	(0.306) 0.310 0.294

Locus 10092 :

2 alleles

99.2% missing data

2	(0.542) 0.539 0.541

3	(0.458) 0.461 0.459

Locus 10093 :

2 alleles

99.2% missing data

3	(0.384) 0.381 0.387

1	(0.616) 0.619 0.613

Locus 10094 :

2 alleles

99.2% missing data

4	(0.438) 0.442 0.444

3	(0.562) 0.558 0.556

Locus 10095 :

2 alleles

98.8% missing data

4	(0.139) 0.139 0.142

1	(0.861) 0.861 0.858

Locus 10096 :

2 alleles

98.8% missing data

2	(0.761) 0.762 0.760

4	(0.239) 0.238 0.240

Locus 10097 :

2 alleles

99.2% missing data

2	(0.682) 0.681 0.686

4	(0.318) 0.319 0.314

Locus 10098 :

2 alleles

0.0% missing data

2	(0.989) 0.984 0.996

3	(0.011) 0.016 0.004

Locus 10099 :

3 alleles

96.9% missing data

2	(0.291) 0.301 0.278

3	(0.189) 0.188 0.189

1	(0.520) 0.511 0.532

Locus 10100 :

2 alleles

98.8% missing data

4	(0.549) 0.554 0.542

1	(0.451) 0.446 0.458

Locus 10101 :

2 alleles

99.2% missing data

3	(0.379) 0.374 0.385

4	(0.621) 0.626 0.615

Locus 10102 :

2 alleles

99.2% missing data

2	(0.587) 0.588 0.583

4	(0.413) 0.412 0.417

Locus 10103 :

2 alleles

99.2% missing data

4	(0.604) 0.609 0.600

2	(0.396) 0.391 0.400

Locus 10104 :

2 alleles

98.0% missing data

4	(0.295) 0.293 0.301

2	(0.705) 0.707 0.699

Locus 10105 :

2 alleles

99.2% missing data

2	(0.769) 0.770 0.760

1	(0.231) 0.230 0.240

Locus 10106 :

3 alleles

98.4% missing data

4	(0.284) 0.285 0.275

2	(0.345) 0.334 0.350

1	(0.371) 0.380 0.374

Locus 10107 :

2 alleles

8.6% missing data

4	(0.986) 0.980 0.995

3	(0.014) 0.020 0.005

Locus 10108 :

2 alleles

99.2% missing data

2	(0.598) 0.599 0.596

3	(0.402) 0.401 0.404

Locus 10109 :

2 alleles

99.2% missing data

3	(0.606) 0.606 0.605

4	(0.394) 0.394 0.395

Locus 10110 :

2 alleles

99.2% missing data

1	(0.598) 0.597 0.600

2	(0.402) 0.403 0.400

Locus 10111 :

2 alleles

99.2% missing data

4	(0.437) 0.438 0.435

2	(0.563) 0.562 0.565

Locus 10112 :

2 alleles

96.1% missing data

1	(0.808) 0.812 0.808

2	(0.192) 0.188 0.192

Locus 10113 :

2 alleles

98.8% missing data

4	(0.517) 0.516 0.520

1	(0.483) 0.484 0.480

Locus 10114 :

2 alleles

96.5% missing data

2	(0.351) 0.361 0.339

3	(0.649) 0.639 0.661

Locus 10115 :

2 alleles

99.2% missing data

4	(0.193) 0.186 0.197

3	(0.807) 0.814 0.803

Locus 10116 :

2 alleles

99.2% missing data

4	(0.514) 0.517 0.512

3	(0.486) 0.483 0.488

Locus 10117 :

2 alleles

99.2% missing data

4	(0.293) 0.290 0.291

1	(0.707) 0.710 0.709

Locus 10118 :

2 alleles

99.2% missing data

3	(0.403) 0.400 0.405

4	(0.597) 0.600 0.595

Locus 10119 :

2 alleles

99.2% missing data

4	(0.573) 0.578 0.569

1	(0.427) 0.422 0.431

Locus 10120 :

2 alleles

99.2% missing data

1	(0.509) 0.511 0.507

4	(0.491) 0.489 0.493

Locus 10121 :

2 alleles

99.2% missing data

1	(0.507) 0.510 0.512

2	(0.493) 0.490 0.488

Locus 10122 :

2 alleles

98.8% missing data

1	(0.431) 0.430 0.425

2	(0.569) 0.570 0.575

Locus 10123 :

2 alleles

98.8% missing data

2	(0.721) 0.719 0.724

1	(0.279) 0.281 0.276

Locus 10124 :

2 alleles

96.5% missing data

1	(0.913) 0.919 0.909

3	(0.087) 0.081 0.091

Locus 10125 :

2 alleles

98.0% missing data

1	(0.760) 0.763 0.765

4	(0.240) 0.237 0.235

Locus 10126 :

2 alleles

99.2% missing data

2	(0.499) 0.496 0.496

4	(0.501) 0.504 0.504

Locus 10127 :

2 alleles

0.0% missing data

2	(0.988) 0.986 0.995

3	(0.012) 0.014 0.005

Locus 10128 :

2 alleles

98.8% missing data

1	(0.515) 0.518 0.520

4	(0.485) 0.482 0.480

Locus 10129 :

2 alleles

94.5% missing data

2	(0.804) 0.824 0.794

3	(0.196) 0.176 0.206

Locus 10130 :

2 alleles

93.0% missing data

3	(0.917) 0.924 0.916

4	(0.083) 0.076 0.084

Locus 10131 :

2 alleles

99.2% missing data

4	(0.471) 0.460 0.475

3	(0.529) 0.540 0.525

Locus 10132 :

2 alleles

89.8% missing data

2	(0.895) 0.900 0.896

1	(0.105) 0.100 0.104

Locus 10133 :

2 alleles

99.2% missing data

2	(0.323) 0.320 0.326

1	(0.677) 0.680 0.674

Locus 10134 :

2 alleles

99.2% missing data

1	(0.401) 0.400 0.405

2	(0.599) 0.600 0.595

Locus 10135 :

2 alleles

97.7% missing data

1	(0.229) 0.225 0.230

4	(0.771) 0.775 0.770

Locus 10136 :

2 alleles

96.1% missing data

2	(0.871) 0.885 0.866

3	(0.129) 0.115 0.134

Locus 10137 :

2 alleles

99.2% missing data

1	(0.545) 0.539 0.555

3	(0.455) 0.461 0.445

Locus 10138 :

2 alleles

98.0% missing data

4	(0.198) 0.198 0.193

3	(0.802) 0.802 0.807

Locus 10139 :

2 alleles

97.3% missing data

2	(0.499) 0.495 0.500

4	(0.501) 0.505 0.500

Locus 10140 :

2 alleles

0.0% missing data

3	(0.982) 0.987 0.989

1	(0.018) 0.013 0.011

Locus 10141 :

2 alleles

90.2% missing data

4	(0.834) 0.868 0.801

1	(0.166) 0.132 0.199

Locus 10142 :

2 alleles

99.2% missing data

4	(0.403) 0.405 0.402

3	(0.597) 0.595 0.598

Locus 10143 :

2 alleles

99.2% missing data

4	(0.638) 0.630 0.645

1	(0.362) 0.370 0.355

Locus 10144 :

3 alleles

98.4% missing data

3	(0.360) 0.354 0.356

2	(0.209) 0.207 0.215

4	(0.431) 0.440 0.430

Locus 10145 :

2 alleles

99.2% missing data

2	(0.413) 0.419 0.413

3	(0.587) 0.581 0.587

Locus 10146 :

2 alleles

77.3% missing data

2	(0.956) 0.958 0.958

3	(0.044) 0.042 0.042

Locus 10147 :

2 alleles

99.2% missing data

4	(0.484) 0.488 0.480

2	(0.516) 0.512 0.520

Locus 10148 :

2 alleles

98.8% missing data

2	(0.401) 0.393 0.404

4	(0.599) 0.607 0.596

Locus 10149 :

2 alleles

98.4% missing data

1	(0.492) 0.493 0.496

4	(0.508) 0.507 0.504

Locus 10150 :

2 alleles

94.9% missing data

4	(0.280) 0.283 0.285

3	(0.720) 0.717 0.715

Locus 10151 :

2 alleles

96.1% missing data

2	(0.154) 0.143 0.153

4	(0.846) 0.857 0.847

Locus 10152 :

2 alleles

99.2% missing data

2	(0.252) 0.249 0.257

4	(0.748) 0.751 0.743

Locus 10153 :

2 alleles

99.2% missing data

4	(0.555) 0.553 0.555

1	(0.445) 0.447 0.445

Locus 10154 :

2 alleles

89.5% missing data

2	(0.825) 0.848 0.797

4	(0.175) 0.152 0.203

Locus 10155 :

2 alleles

99.2% missing data

2	(0.468) 0.464 0.474

4	(0.532) 0.536 0.526

Locus 10156 :

2 alleles

96.1% missing data

1	(0.384) 0.383 0.384

4	(0.616) 0.617 0.616

Locus 10157 :

2 alleles

99.2% missing data

2	(0.474) 0.474 0.476

1	(0.526) 0.526 0.524

Locus 10158 :

2 alleles

99.2% missing data

3	(0.250) 0.257 0.246

4	(0.750) 0.743 0.754

Locus 10159 :

2 alleles

97.7% missing data

3	(0.185) 0.180 0.181

4	(0.815) 0.820 0.819

Locus 10160 :

2 alleles

0.0% missing data

4	(0.986) 0.989 0.993

1	(0.014) 0.011 0.007

Locus 10161 :

2 alleles

98.8% missing data

1	(0.554) 0.548 0.557

4	(0.446) 0.452 0.443

Locus 10162 :

2 alleles

98.4% missing data

4	(0.575) 0.571 0.574

1	(0.425) 0.429 0.426

Locus 10163 :

2 alleles

91.0% missing data

2	(0.937) 0.949 0.929

1	(0.063) 0.051 0.071

Locus 10164 :

2 alleles

99.2% missing data

4	(0.342) 0.340 0.345

3	(0.658) 0.660 0.655

Locus 10165 :

2 alleles

99.2% missing data

4	(0.517) 0.513 0.518

3	(0.483) 0.487 0.482

Locus 10166 :

2 alleles

98.0% missing data

2	(0.561) 0.560 0.571

3	(0.439) 0.440 0.429

Locus 10167 :

2 alleles

90.6% missing data

2	(0.912) 0.917 0.916

1	(0.088) 0.083 0.084

Locus 10168 :

2 alleles

99.2% missing data

3	(0.573) 0.575 0.568

2	(0.427) 0.425 0.432

Locus 10169 :

4 alleles

98.4% missing data

3	(0.185) 0.191 0.186

1	(0.378) 0.373 0.378

4	(0.182) 0.176 0.189

2	(0.255) 0.260 0.247

Locus 10170 :

2 alleles

99.2% missing data

4	(0.578) 0.577 0.575

2	(0.422) 0.423 0.425

Locus 10171 :

2 alleles

99.2% missing data

4	(0.460) 0.471 0.454

3	(0.540) 0.529 0.546

Locus 10172 :

2 alleles

95.7% missing data

4	(0.797) 0.805 0.793

1	(0.203) 0.195 0.207

Locus 10173 :

2 alleles

97.3% missing data

4	(0.208) 0.206 0.205

1	(0.792) 0.794 0.795

Locus 10174 :

2 alleles

99.2% missing data

2	(0.579) 0.582 0.580

3	(0.421) 0.418 0.420

Locus 10175 :

2 alleles

96.1% missing data

4	(0.723) 0.740 0.713

1	(0.277) 0.260 0.287

Locus 10176 :

2 alleles

98.8% missing data

1	(0.493) 0.492 0.500

2	(0.507) 0.508 0.500

Locus 10177 :

2 alleles

99.2% missing data

3	(0.560) 0.554 0.562

4	(0.440) 0.446 0.438

Locus 10178 :

2 alleles

98.8% missing data

2	(0.563) 0.565 0.565

1	(0.437) 0.435 0.435

Locus 10179 :

2 alleles

99.2% missing data

3	(0.785) 0.787 0.781

1	(0.215) 0.213 0.219

Locus 10180 :

2 alleles

99.2% missing data

4	(0.603) 0.597 0.609

3	(0.397) 0.403 0.391

Locus 10181 :

2 alleles

10.2% missing data

2	(0.985) 0.978 0.995

3	(0.015) 0.022 0.005

Locus 10182 :

2 alleles

99.2% missing data

3	(0.520) 0.517 0.525

4	(0.480) 0.483 0.475

Locus 10183 :

2 alleles

99.2% missing data

2	(0.202) 0.200 0.210

4	(0.798) 0.800 0.790

Locus 10184 :

2 alleles

99.2% missing data

1	(0.162) 0.160 0.166

3	(0.838) 0.840 0.834

Locus 10185 :

2 alleles

8.2% missing data

4	(0.981) 0.975 0.994

2	(0.019) 0.025 0.006

Locus 10186 :

2 alleles

99.2% missing data

3	(0.751) 0.750 0.748

1	(0.249) 0.250 0.252

Locus 10187 :

2 alleles

95.7% missing data

2	(0.153) 0.140 0.161

1	(0.847) 0.860 0.839

Locus 10188 :

2 alleles

97.7% missing data

1	(0.783) 0.783 0.788

4	(0.217) 0.217 0.212

Locus 10189 :

2 alleles

97.7% missing data

3	(0.886) 0.885 0.887

1	(0.114) 0.115 0.113

Locus 10190 :

2 alleles

99.2% missing data

2	(0.603) 0.601 0.601

4	(0.397) 0.399 0.399

Locus 10191 :

2 alleles

98.8% missing data

2	(0.392) 0.389 0.393

3	(0.608) 0.611 0.607

Locus 10192 :

2 alleles

0.4% missing data

3	(0.984) 0.982 0.995

2	(0.016) 0.018 0.005

Locus 10193 :

2 alleles

99.2% missing data

1	(0.309) 0.312 0.313

2	(0.691) 0.688 0.687

Locus 10194 :

2 alleles

99.2% missing data

4	(0.557) 0.561 0.557

3	(0.443) 0.439 0.443

Locus 10195 :

2 alleles

98.8% missing data

2	(0.081) 0.082 0.084

3	(0.919) 0.918 0.916

Locus 10196 :

2 alleles

98.8% missing data

1	(0.341) 0.341 0.348

4	(0.659) 0.659 0.652

Locus 10197 :

2 alleles

99.2% missing data

3	(0.498) 0.498 0.496

2	(0.502) 0.502 0.504

Locus 10198 :

2 alleles

99.2% missing data

1	(0.662) 0.664 0.652

3	(0.338) 0.336 0.348

Locus 10199 :

2 alleles

99.2% missing data

1	(0.686) 0.683 0.687

3	(0.314) 0.317 0.313

Locus 10200 :

2 alleles

91.0% missing data

2	(0.904) 0.922 0.895

4	(0.096) 0.078 0.105

Locus 10201 :

2 alleles

99.2% missing data

1	(0.274) 0.276 0.275

4	(0.726) 0.724 0.725

Locus 10202 :

2 alleles

98.8% missing data

3	(0.755) 0.760 0.752

4	(0.245) 0.240 0.248

Locus 10203 :

2 alleles

99.2% missing data

2	(0.614) 0.615 0.612

4	(0.386) 0.385 0.388

Locus 10204 :

2 alleles

99.2% missing data

2	(0.340) 0.334 0.343

4	(0.660) 0.666 0.657

Locus 10205 :

2 alleles

99.2% missing data

2	(0.451) 0.453 0.446

4	(0.549) 0.547 0.554

Locus 10206 :

2 alleles

99.2% missing data

3	(0.573) 0.576 0.575

4	(0.427) 0.424 0.425

Locus 10207 :

2 alleles

99.2% missing data

1	(0.282) 0.282 0.285

2	(0.718) 0.718 0.715

Locus 10208 :

2 alleles

99.2% missing data

3	(0.252) 0.250 0.256

4	(0.748) 0.750 0.744

Locus 10209 :

2 alleles

96.9% missing data

1	(0.183) 0.187 0.178

3	(0.817) 0.813 0.822

Locus 10210 :

2 alleles

99.2% missing data

1	(0.431) 0.429 0.424

3	(0.569) 0.571 0.576

Locus 10211 :

2 alleles

99.2% missing data

2	(0.629) 0.621 0.628

1	(0.371) 0.379 0.372

Locus 10212 :

2 alleles

98.0% missing data

1	(0.628) 0.617 0.630

3	(0.372) 0.383 0.370

Locus 10213 :

2 alleles

99.2% missing data

1	(0.534) 0.531 0.532

2	(0.466) 0.469 0.468

Locus 10214 :

2 alleles

98.8% missing data

1	(0.387) 0.381 0.393

4	(0.613) 0.619 0.607

Locus 10215 :

2 alleles

98.8% missing data

3	(0.591) 0.582 0.600

4	(0.409) 0.418 0.400

Locus 10216 :

2 alleles

99.2% missing data

1	(0.632) 0.628 0.627

2	(0.368) 0.372 0.373

Locus 10217 :

2 alleles

96.5% missing data

2	(0.508) 0.494 0.514

4	(0.492) 0.506 0.486

Locus 10218 :

2 alleles

97.7% missing data

2	(0.751) 0.766 0.747

4	(0.249) 0.234 0.253

Locus 10219 :

2 alleles

99.2% missing data

1	(0.720) 0.718 0.716

2	(0.280) 0.282 0.284

Locus 10220 :

2 alleles

98.4% missing data

1	(0.510) 0.519 0.503

4	(0.490) 0.481 0.497

Locus 10221 :

2 alleles

98.8% missing data

4	(0.530) 0.536 0.523

1	(0.470) 0.464 0.477

Locus 10222 :

2 alleles

96.5% missing data

2	(0.702) 0.713 0.705

4	(0.298) 0.287 0.295

Locus 10223 :

2 alleles

98.8% missing data

4	(0.305) 0.306 0.316

1	(0.695) 0.694 0.684

Locus 10224 :

2 alleles

99.2% missing data

3	(0.521) 0.519 0.512

2	(0.479) 0.481 0.488

Locus 10225 :

2 alleles

91.0% missing data

2	(0.938) 0.944 0.938

3	(0.062) 0.056 0.062

Locus 10226 :

2 alleles

99.2% missing data

2	(0.625) 0.621 0.627

3	(0.375) 0.379 0.373

Locus 10227 :

2 alleles

99.2% missing data

2	(0.580) 0.576 0.583

3	(0.420) 0.424 0.417

Locus 10228 :

2 alleles

96.5% missing data

3	(0.258) 0.249 0.261

2	(0.742) 0.751 0.739

Locus 10229 :

2 alleles

0.0% missing data

4	(0.987) 0.982 0.995

1	(0.013) 0.018 0.005

Locus 10230 :

2 alleles

9.8% missing data

3	(0.984) 0.978 0.995

4	(0.016) 0.022 0.005

Locus 10231 :

2 alleles

99.2% missing data

3	(0.224) 0.229 0.222

4	(0.776) 0.771 0.778

Locus 10232 :

2 alleles

99.2% missing data

2	(0.679) 0.670 0.682

1	(0.321) 0.330 0.318

Locus 10233 :

2 alleles

99.2% missing data

1	(0.450) 0.451 0.448

4	(0.550) 0.549 0.552

Locus 10234 :

2 alleles

96.5% missing data

3	(0.853) 0.855 0.853

2	(0.147) 0.145 0.147

Locus 10235 :

2 alleles

98.8% missing data

1	(0.523) 0.520 0.516

4	(0.477) 0.480 0.484

Locus 10236 :

2 alleles

98.8% missing data

4	(0.727) 0.731 0.724

3	(0.273) 0.269 0.276

Locus 10237 :

2 alleles

99.2% missing data

2	(0.528) 0.529 0.527

4	(0.472) 0.471 0.473

Locus 10238 :

2 alleles

99.2% missing data

3	(0.508) 0.509 0.503

2	(0.492) 0.491 0.497

Locus 10239 :

2 alleles

99.2% missing data

2	(0.466) 0.468 0.458

1	(0.534) 0.532 0.542

Locus 10240 :

2 alleles

98.4% missing data

3	(0.764) 0.777 0.755

4	(0.236) 0.223 0.245

Locus 10241 :

2 alleles

99.2% missing data

2	(0.229) 0.223 0.241

3	(0.771) 0.777 0.759

Locus 10242 :

2 alleles

99.2% missing data

4	(0.264) 0.259 0.272

2	(0.736) 0.741 0.728

Locus 10243 :

2 alleles

99.2% missing data

4	(0.413) 0.419 0.407

3	(0.587) 0.581 0.593

Locus 10244 :

2 alleles

97.3% missing data

2	(0.721) 0.714 0.726

4	(0.279) 0.286 0.274

Locus 10245 :

2 alleles

98.4% missing data

1	(0.583) 0.587 0.584

3	(0.417) 0.413 0.416

Locus 10246 :

2 alleles

95.7% missing data

3	(0.141) 0.134 0.142

2	(0.859) 0.866 0.858

Locus 10247 :

2 alleles

99.2% missing data

1	(0.268) 0.261 0.276

3	(0.732) 0.739 0.724

Locus 10248 :

2 alleles

99.2% missing data

3	(0.492) 0.493 0.490

2	(0.508) 0.507 0.510

Locus 10249 :

2 alleles

0.0% missing data

3	(0.915) 0.762 0.976

2	(0.085) 0.238 0.024

Locus 10250 :

2 alleles

99.2% missing data

4	(0.446) 0.439 0.454

1	(0.554) 0.561 0.546

Locus 10251 :

2 alleles

99.2% missing data

4	(0.377) 0.372 0.380

3	(0.623) 0.628 0.620

Locus 10252 :

2 alleles

99.2% missing data

3	(0.481) 0.481 0.481

2	(0.519) 0.519 0.519

Locus 10253 :

2 alleles

99.2% missing data

1	(0.642) 0.638 0.648

4	(0.358) 0.362 0.352

Locus 10254 :

3 alleles

98.8% missing data

1	(0.361) 0.359 0.358

4	(0.305) 0.309 0.297

3	(0.334) 0.332 0.345

Locus 10255 :

3 alleles

98.8% missing data

1	(0.307) 0.306 0.309

4	(0.392) 0.396 0.390

2	(0.301) 0.299 0.301

Locus 10256 :

2 alleles

99.2% missing data

1	(0.180) 0.185 0.180

3	(0.820) 0.815 0.820

Locus 10257 :

2 alleles

99.2% missing data

3	(0.668) 0.664 0.677

2	(0.332) 0.336 0.323

Locus 10258 :

2 alleles

99.2% missing data

2	(0.556) 0.557 0.550

3	(0.444) 0.443 0.450

Locus 10259 :

2 alleles

99.2% missing data

3	(0.287) 0.286 0.291

4	(0.713) 0.714 0.709

Locus 10260 :

2 alleles

99.2% missing data

2	(0.776) 0.777 0.769

3	(0.224) 0.223 0.231

Locus 10261 :

2 alleles

99.2% missing data

1	(0.577) 0.581 0.576

3	(0.423) 0.419 0.424

Locus 10262 :

2 alleles

8.2% missing data

3	(0.989) 0.980 0.997

1	(0.011) 0.020 0.003

Locus 10263 :

2 alleles

99.2% missing data

2	(0.286) 0.286 0.294

1	(0.714) 0.714 0.706

Locus 10264 :

3 alleles

98.8% missing data

2	(0.542) 0.546 0.538

1	(0.112) 0.109 0.123

3	(0.345) 0.345 0.340

Locus 10265 :

2 alleles

98.4% missing data

1	(0.508) 0.522 0.503

3	(0.492) 0.478 0.497

Locus 10266 :

2 alleles

98.4% missing data

2	(0.331) 0.322 0.343

4	(0.669) 0.678 0.657

Locus 10267 :

2 alleles

99.2% missing data

4	(0.395) 0.391 0.400

2	(0.605) 0.609 0.600

Locus 10268 :

2 alleles

98.8% missing data

1	(0.703) 0.704 0.703

4	(0.297) 0.296 0.297

Locus 10269 :

2 alleles

99.2% missing data

1	(0.192) 0.199 0.192

4	(0.808) 0.801 0.808

Locus 10270 :

2 alleles

98.8% missing data

1	(0.642) 0.636 0.651

3	(0.358) 0.364 0.349

Locus 10271 :

2 alleles

99.2% missing data

3	(0.538) 0.534 0.543

1	(0.462) 0.466 0.457

Locus 10272 :

2 alleles

99.2% missing data

1	(0.570) 0.571 0.574

2	(0.430) 0.429 0.426

Locus 10273 :

2 alleles

97.3% missing data

1	(0.725) 0.727 0.734

4	(0.275) 0.273 0.266

Locus 10274 :

2 alleles

99.2% missing data

3	(0.246) 0.243 0.248

1	(0.754) 0.757 0.752

Locus 10275 :

2 alleles

99.2% missing data

4	(0.685) 0.693 0.678

3	(0.315) 0.307 0.322

Locus 10276 :

2 alleles

99.2% missing data

4	(0.509) 0.503 0.516

2	(0.491) 0.497 0.484

Locus 10277 :

2 alleles

99.2% missing data

3	(0.893) 0.881 0.898

1	(0.107) 0.119 0.102

Locus 10278 :

2 alleles

93.0% missing data

2	(0.914) 0.922 0.913

1	(0.086) 0.078 0.087

Locus 10279 :

2 alleles

98.4% missing data

1	(0.281) 0.278 0.285

3	(0.719) 0.722 0.715

Locus 10280 :

2 alleles

99.2% missing data

4	(0.495) 0.493 0.496

2	(0.505) 0.507 0.504

Locus 10281 :

2 alleles

98.8% missing data

2	(0.705) 0.696 0.713

3	(0.295) 0.304 0.287

Locus 10282 :

2 alleles

98.8% missing data

4	(0.264) 0.267 0.263

3	(0.736) 0.733 0.737

Locus 10283 :

2 alleles

99.2% missing data

2	(0.533) 0.537 0.533

3	(0.467) 0.463 0.467

Locus 10284 :

2 alleles

98.8% missing data

3	(0.407) 0.403 0.411

2	(0.593) 0.597 0.589

Locus 10285 :

2 alleles

99.2% missing data

2	(0.499) 0.495 0.496

4	(0.501) 0.505 0.504

Locus 10286 :

2 alleles

99.2% missing data

1	(0.631) 0.625 0.638

3	(0.369) 0.375 0.362

Locus 10287 :

2 alleles

99.2% missing data

3	(0.495) 0.497 0.488

1	(0.505) 0.503 0.512

Locus 10288 :

2 alleles

99.2% missing data

2	(0.353) 0.351 0.355

3	(0.647) 0.649 0.645

Locus 10289 :

2 alleles

99.2% missing data

2	(0.251) 0.252 0.251

4	(0.749) 0.748 0.749

Locus 10290 :

2 alleles

99.2% missing data

4	(0.302) 0.299 0.301

1	(0.698) 0.701 0.699

Locus 10291 :

2 alleles

96.1% missing data

2	(0.210) 0.202 0.212

1	(0.790) 0.798 0.788

Locus 10292 :

2 alleles

99.2% missing data

1	(0.450) 0.449 0.452

2	(0.550) 0.551 0.548

Locus 10293 :

2 alleles

99.2% missing data

1	(0.188) 0.192 0.180

3	(0.812) 0.808 0.820

Locus 10294 :

2 alleles

96.1% missing data

4	(0.338) 0.336 0.344

1	(0.662) 0.664 0.656

Locus 10295 :

2 alleles

91.0% missing data

1	(0.912) 0.924 0.909

2	(0.088) 0.076 0.091

Locus 10296 :

2 alleles

99.2% missing data

4	(0.439) 0.442 0.445

1	(0.561) 0.558 0.555

Locus 10297 :

2 alleles

98.8% missing data

3	(0.357) 0.355 0.355

2	(0.643) 0.645 0.645

Locus 10298 :

2 alleles

99.2% missing data

4	(0.520) 0.524 0.517

1	(0.480) 0.476 0.483

Locus 10299 :

3 alleles

98.8% missing data

4	(0.384) 0.383 0.383

2	(0.287) 0.281 0.296

1	(0.328) 0.336 0.321

Locus 10300 :

2 alleles

99.2% missing data

2	(0.294) 0.290 0.297

1	(0.706) 0.710 0.703

Locus 10301 :

2 alleles

99.2% missing data

3	(0.253) 0.250 0.259

1	(0.747) 0.750 0.741

Locus 10302 :

2 alleles

99.2% missing data

2	(0.619) 0.625 0.615

1	(0.381) 0.375 0.385

Locus 10303 :

2 alleles

99.2% missing data

1	(0.335) 0.330 0.344

2	(0.665) 0.670 0.656

Locus 10304 :

2 alleles

99.2% missing data

1	(0.863) 0.853 0.867

4	(0.137) 0.147 0.133

Locus 10305 :

2 alleles

99.2% missing data

4	(0.618) 0.614 0.618

3	(0.382) 0.386 0.382

Locus 10306 :

2 alleles

99.2% missing data

3	(0.333) 0.334 0.337

1	(0.667) 0.666 0.663

Locus 10307 :

2 alleles

99.2% missing data

1	(0.780) 0.777 0.784

3	(0.220) 0.223 0.216

Locus 10308 :

2 alleles

97.3% missing data

2	(0.736) 0.736 0.735

3	(0.264) 0.264 0.265

Locus 10309 :

2 alleles

98.8% missing data

1	(0.422) 0.423 0.419

2	(0.578) 0.577 0.581

Locus 10310 :

2 alleles

98.8% missing data

1	(0.502) 0.496 0.505

4	(0.498) 0.504 0.495

Locus 10311 :

2 alleles

99.2% missing data

1	(0.307) 0.310 0.303

4	(0.693) 0.690 0.697

Locus 10312 :

2 alleles

99.2% missing data

1	(0.525) 0.524 0.516

4	(0.475) 0.476 0.484

Locus 10313 :

2 alleles

98.0% missing data

2	(0.711) 0.714 0.715

1	(0.289) 0.286 0.285

Locus 10314 :

2 alleles

93.8% missing data

3	(0.935) 0.925 0.946

1	(0.065) 0.075 0.054

Locus 10315 :

2 alleles

99.2% missing data

2	(0.459) 0.458 0.458

4	(0.541) 0.542 0.542

Locus 10316 :

2 alleles

99.2% missing data

3	(0.375) 0.370 0.381

2	(0.625) 0.630 0.619

Locus 10317 :

2 alleles

99.2% missing data

3	(0.568) 0.569 0.564

4	(0.432) 0.431 0.436

Locus 10318 :

2 alleles

99.2% missing data

2	(0.267) 0.270 0.259

1	(0.733) 0.730 0.741

Locus 10319 :

2 alleles

99.2% missing data

3	(0.243) 0.242 0.246

2	(0.757) 0.758 0.754

Locus 10320 :

2 alleles

99.2% missing data

3	(0.369) 0.371 0.377

4	(0.631) 0.629 0.623

Locus 10321 :

2 alleles

99.2% missing data

4	(0.303) 0.306 0.298

3	(0.697) 0.694 0.702

Locus 10322 :

3 alleles

98.4% missing data

4	(0.194) 0.188 0.203

1	(0.176) 0.174 0.175

2	(0.631) 0.638 0.622

Locus 10323 :

2 alleles

97.7% missing data

2	(0.666) 0.671 0.662

1	(0.334) 0.329 0.338

Locus 10324 :

2 alleles

99.2% missing data

2	(0.450) 0.444 0.459

1	(0.550) 0.556 0.541

Locus 10325 :

2 alleles

98.4% missing data

3	(0.396) 0.385 0.399

4	(0.604) 0.615 0.601

Locus 10326 :

2 alleles

99.2% missing data

2	(0.395) 0.396 0.393

4	(0.605) 0.604 0.607

Locus 10327 :

2 alleles

99.2% missing data

2	(0.717) 0.720 0.716

3	(0.283) 0.280 0.284

Locus 10328 :

2 alleles

97.7% missing data

4	(0.171) 0.166 0.171

1	(0.829) 0.834 0.829

Locus 10329 :

2 alleles

99.2% missing data

3	(0.401) 0.401 0.408

4	(0.599) 0.599 0.592

Locus 10330 :

2 alleles

99.2% missing data

1	(0.604) 0.599 0.610

2	(0.396) 0.401 0.390

Locus 10331 :

2 alleles

99.2% missing data

4	(0.544) 0.546 0.546

1	(0.456) 0.454 0.454

Locus 10332 :

2 alleles

99.2% missing data

3	(0.381) 0.383 0.378

1	(0.619) 0.617 0.622

Locus 10333 :

2 alleles

99.2% missing data

1	(0.631) 0.638 0.629

4	(0.369) 0.362 0.371

Locus 10334 :

2 alleles

99.2% missing data

3	(0.278) 0.274 0.280

1	(0.722) 0.726 0.720

Locus 10335 :

2 alleles

99.2% missing data

3	(0.277) 0.273 0.275

2	(0.723) 0.727 0.725

Locus 10336 :

3 alleles

97.3% missing data

3	(0.391) 0.394 0.384

2	(0.427) 0.427 0.437

1	(0.182) 0.179 0.179

Locus 10337 :

2 alleles

99.2% missing data

3	(0.495) 0.490 0.497

1	(0.505) 0.510 0.503

Locus 10338 :

2 alleles

97.7% missing data

4	(0.584) 0.578 0.601

2	(0.416) 0.422 0.399

Locus 10339 :

2 alleles

94.5% missing data

3	(0.656) 0.678 0.632

2	(0.344) 0.322 0.368

Locus 10340 :

2 alleles

99.2% missing data

3	(0.685) 0.680 0.689

4	(0.315) 0.320 0.311

Locus 10341 :

2 alleles

97.3% missing data

3	(0.318) 0.327 0.315

1	(0.682) 0.673 0.685

Locus 10342 :

2 alleles

99.2% missing data

1	(0.615) 0.616 0.611

3	(0.385) 0.384 0.389

Locus 10343 :

2 alleles

98.0% missing data

3	(0.779) 0.769 0.788

1	(0.221) 0.231 0.212

Locus 10344 :

2 alleles

93.8% missing data

3	(0.872) 0.872 0.883

4	(0.128) 0.128 0.117

Locus 10345 :

2 alleles

99.2% missing data

3	(0.327) 0.320 0.324

1	(0.673) 0.680 0.676

Locus 10346 :

2 alleles

99.2% missing data

3	(0.813) 0.809 0.821

4	(0.187) 0.191 0.179

Locus 10347 :

2 alleles

99.2% missing data

3	(0.362) 0.354 0.369

4	(0.638) 0.646 0.631

Locus 10348 :

2 alleles

99.2% missing data

2	(0.238) 0.230 0.239

1	(0.762) 0.770 0.761

Locus 10349 :

2 alleles

99.2% missing data

3	(0.596) 0.599 0.593

1	(0.404) 0.401 0.407

Locus 10350 :

2 alleles

99.2% missing data

1	(0.293) 0.290 0.299

3	(0.707) 0.710 0.701

Locus 10351 :

2 alleles

99.2% missing data

3	(0.391) 0.389 0.394

1	(0.609) 0.611 0.606

Locus 10352 :

2 alleles

92.6% missing data

2	(0.901) 0.899 0.909

1	(0.099) 0.101 0.091

Locus 10353 :

2 alleles

99.2% missing data

3	(0.330) 0.329 0.337

1	(0.670) 0.671 0.663

Locus 10354 :

2 alleles

91.4% missing data

2	(0.943) 0.948 0.939

1	(0.057) 0.052 0.061

Locus 10355 :

2 alleles

97.7% missing data

2	(0.201) 0.202 0.195

1	(0.799) 0.798 0.805

Locus 10356 :

2 alleles

98.8% missing data

3	(0.665) 0.658 0.675

1	(0.335) 0.342 0.325

Locus 10357 :

2 alleles

97.3% missing data

1	(0.574) 0.596 0.567

3	(0.426) 0.404 0.433

Locus 10358 :

2 alleles

99.2% missing data

3	(0.301) 0.307 0.300

4	(0.699) 0.693 0.700

Locus 10359 :

2 alleles

97.7% missing data

3	(0.286) 0.288 0.278

1	(0.714) 0.712 0.722

Locus 10360 :

2 alleles

0.8% missing data

1	(0.906) 0.755 0.971

2	(0.094) 0.245 0.029

Locus 10361 :

2 alleles

97.7% missing data

3	(0.747) 0.749 0.749

2	(0.253) 0.251 0.251

Locus 10362 :

2 alleles

99.2% missing data

2	(0.381) 0.379 0.381

4	(0.619) 0.621 0.619

Locus 10363 :

2 alleles

99.2% missing data

3	(0.391) 0.389 0.399

4	(0.609) 0.611 0.601

Locus 10364 :

2 alleles

99.2% missing data

3	(0.211) 0.214 0.213

1	(0.789) 0.786 0.787

Locus 10365 :

2 alleles

99.2% missing data

4	(0.353) 0.348 0.356

1	(0.647) 0.652 0.644

Locus 10366 :

2 alleles

97.7% missing data

3	(0.115) 0.112 0.115

1	(0.885) 0.888 0.885

Locus 10367 :

2 alleles

99.2% missing data

4	(0.502) 0.505 0.502

2	(0.498) 0.495 0.498

Locus 10368 :

2 alleles

96.9% missing data

2	(0.801) 0.795 0.818

1	(0.199) 0.205 0.182

Locus 10369 :

2 alleles

99.2% missing data

4	(0.574) 0.576 0.570

1	(0.426) 0.424 0.430

Locus 10370 :

2 alleles

98.4% missing data

2	(0.223) 0.218 0.227

1	(0.777) 0.782 0.773

Locus 10371 :

2 alleles

99.2% missing data

2	(0.394) 0.393 0.393

1	(0.606) 0.607 0.607

Locus 10372 :

2 alleles

98.8% missing data

4	(0.438) 0.437 0.441

3	(0.562) 0.563 0.559

Locus 10373 :

2 alleles

99.2% missing data

1	(0.628) 0.620 0.631

2	(0.372) 0.380 0.369

Locus 10374 :

2 alleles

91.4% missing data

3	(0.801) 0.816 0.796

1	(0.199) 0.184 0.204

Locus 10375 :

2 alleles

99.2% missing data

1	(0.515) 0.510 0.519

2	(0.485) 0.490 0.481

Locus 10376 :

2 alleles

97.3% missing data

3	(0.657) 0.661 0.669

2	(0.343) 0.339 0.331

Locus 10377 :

2 alleles

99.2% missing data

3	(0.830) 0.823 0.835

1	(0.170) 0.177 0.165

Locus 10378 :

2 alleles

96.9% missing data

4	(0.852) 0.861 0.847

3	(0.148) 0.139 0.153

Locus 10379 :

2 alleles

96.1% missing data

3	(0.171) 0.156 0.183

4	(0.829) 0.844 0.817

Locus 10380 :

2 alleles

99.2% missing data

4	(0.353) 0.351 0.351

3	(0.647) 0.649 0.649

Locus 10381 :

2 alleles

99.2% missing data

3	(0.728) 0.725 0.724

2	(0.272) 0.275 0.276

Locus 10382 :

2 alleles

93.0% missing data

2	(0.473) 0.508 0.432

3	(0.527) 0.492 0.568

Locus 10383 :

2 alleles

89.1% missing data

4	(0.676) 0.692 0.670

1	(0.324) 0.308 0.330

Locus 10384 :

2 alleles

99.2% missing data

3	(0.641) 0.631 0.641

4	(0.359) 0.369 0.359

Locus 10385 :

2 alleles

99.2% missing data

4	(0.428) 0.424 0.435

2	(0.572) 0.576 0.565

Locus 10386 :

2 alleles

99.2% missing data

2	(0.503) 0.499 0.508

1	(0.497) 0.501 0.492

Locus 10387 :

2 alleles

99.2% missing data

2	(0.217) 0.214 0.221

3	(0.783) 0.786 0.779

Locus 10388 :

2 alleles

98.4% missing data

1	(0.667) 0.673 0.660

2	(0.333) 0.327 0.340

Locus 10389 :

2 alleles

99.2% missing data

1	(0.495) 0.495 0.498

4	(0.505) 0.505 0.502

Locus 10390 :

2 alleles

99.2% missing data

4	(0.398) 0.394 0.399

1	(0.602) 0.606 0.601

Locus 10391 :

2 alleles

99.2% missing data

3	(0.789) 0.785 0.797

2	(0.211) 0.215 0.203

Locus 10392 :

2 alleles

99.2% missing data

1	(0.673) 0.670 0.677

2	(0.327) 0.330 0.323

Locus 10393 :

2 alleles

99.2% missing data

2	(0.481) 0.482 0.487

4	(0.519) 0.518 0.513

Locus 10394 :

2 alleles

94.5% missing data

1	(0.904) 0.903 0.908

4	(0.096) 0.097 0.092

Locus 10395 :

2 alleles

99.2% missing data

3	(0.575) 0.565 0.579

4	(0.425) 0.435 0.421

Locus 10396 :

2 alleles

96.1% missing data

4	(0.141) 0.134 0.148

3	(0.859) 0.866 0.852

Locus 10397 :

2 alleles

98.0% missing data

2	(0.705) 0.700 0.710

1	(0.295) 0.300 0.290

Locus 10398 :

2 alleles

99.2% missing data

1	(0.397) 0.394 0.400

3	(0.603) 0.606 0.600

Locus 10399 :

2 alleles

98.0% missing data

3	(0.392) 0.401 0.380

4	(0.608) 0.599 0.620

Locus 10400 :

2 alleles

99.2% missing data

2	(0.766) 0.765 0.765

1	(0.234) 0.235 0.235

Locus 10401 :

3 alleles

98.8% missing data

1	(0.508) 0.509 0.506

4	(0.376) 0.377 0.374

2	(0.115) 0.113 0.120

Locus 10402 :

2 alleles

97.7% missing data

2	(0.155) 0.164 0.147

4	(0.845) 0.836 0.853

Locus 10403 :

2 alleles

88.3% missing data

2	(0.934) 0.940 0.937

1	(0.066) 0.060 0.063

Locus 10404 :

2 alleles

99.2% missing data

3	(0.555) 0.556 0.551

1	(0.445) 0.444 0.449

Locus 10405 :

2 alleles

98.8% missing data

2	(0.598) 0.596 0.601

1	(0.402) 0.404 0.399

Locus 10406 :

2 alleles

99.2% missing data

3	(0.457) 0.449 0.462

4	(0.543) 0.551 0.538

Locus 10407 :

2 alleles

99.2% missing data

4	(0.772) 0.775 0.763

2	(0.228) 0.225 0.237

Locus 10408 :

2 alleles

99.2% missing data

3	(0.435) 0.430 0.433

1	(0.565) 0.570 0.567

Locus 10409 :

2 alleles

99.2% missing data

1	(0.727) 0.729 0.720

3	(0.273) 0.271 0.280

Locus 10410 :

2 alleles

98.8% missing data

1	(0.244) 0.243 0.244

2	(0.756) 0.757 0.756

Locus 10411 :

3 alleles

98.4% missing data

3	(0.377) 0.370 0.384

1	(0.325) 0.330 0.315

2	(0.298) 0.300 0.302

Locus 10412 :

2 alleles

96.1% missing data

4	(0.156) 0.147 0.158

2	(0.844) 0.853 0.842

Locus 10413 :

2 alleles

99.2% missing data

4	(0.209) 0.208 0.214

2	(0.791) 0.792 0.786

Locus 10414 :

2 alleles

99.2% missing data

4	(0.736) 0.739 0.730

3	(0.264) 0.261 0.270

Locus 10415 :

2 alleles

98.8% missing data

4	(0.611) 0.618 0.611

1	(0.389) 0.382 0.389

Locus 10416 :

3 alleles

96.1% missing data

4	(0.166) 0.156 0.168

2	(0.684) 0.698 0.683

3	(0.150) 0.146 0.149

Locus 10417 :

2 alleles

94.9% missing data

4	(0.785) 0.776 0.806

2	(0.215) 0.224 0.194

Locus 10418 :

2 alleles

0.8% missing data

4	(0.899) 0.757 0.968

3	(0.101) 0.243 0.032

Locus 10419 :

2 alleles

99.2% missing data

4	(0.362) 0.357 0.368

1	(0.638) 0.643 0.632

Locus 10420 :

2 alleles

99.2% missing data

3	(0.654) 0.658 0.645

2	(0.346) 0.342 0.355

Locus 10421 :

2 alleles

99.2% missing data

4	(0.811) 0.816 0.805

3	(0.189) 0.184 0.195

Locus 10422 :

2 alleles

96.5% missing data

2	(0.160) 0.151 0.161

3	(0.840) 0.849 0.839

Locus 10423 :

2 alleles

98.0% missing data

2	(0.687) 0.692 0.690

3	(0.313) 0.308 0.310

Locus 10424 :

2 alleles

99.2% missing data

2	(0.733) 0.730 0.733

1	(0.267) 0.270 0.267

Locus 10425 :

2 alleles

93.0% missing data

4	(0.946) 0.949 0.947

1	(0.054) 0.051 0.053

Locus 10426 :

2 alleles

98.4% missing data

3	(0.446) 0.456 0.448

4	(0.554) 0.544 0.552

Locus 10427 :

2 alleles

98.8% missing data

1	(0.532) 0.540 0.523

2	(0.468) 0.460 0.477

Locus 10428 :

2 alleles

98.4% missing data

1	(0.452) 0.458 0.452

2	(0.548) 0.542 0.548

Locus 10429 :

2 alleles

4.3% missing data

4	(0.988) 0.992 0.993

1	(0.012) 0.008 0.007

Locus 10430 :

2 alleles

98.0% missing data

4	(0.182) 0.177 0.187

2	(0.818) 0.823 0.813

Locus 10431 :

2 alleles

98.8% missing data

3	(0.620) 0.609 0.622

2	(0.380) 0.391 0.378

Locus 10432 :

2 alleles

98.8% missing data

3	(0.496) 0.486 0.513

2	(0.504) 0.514 0.487

Locus 10433 :

2 alleles

98.8% missing data

1	(0.652) 0.657 0.651

3	(0.348) 0.343 0.349

Locus 10434 :

3 alleles

98.8% missing data

2	(0.304) 0.304 0.307

1	(0.389) 0.384 0.392

4	(0.307) 0.312 0.301

Locus 10435 :

2 alleles

0.0% missing data

1	(0.986) 0.988 0.993

2	(0.014) 0.012 0.007

Locus 10436 :

2 alleles

98.8% missing data

1	(0.651) 0.658 0.651

3	(0.349) 0.342 0.349

Locus 10437 :

2 alleles

0.0% missing data

2	(0.984) 0.980 0.995

1	(0.016) 0.020 0.005

Locus 10438 :

2 alleles

95.7% missing data

2	(0.375) 0.397 0.357

1	(0.625) 0.603 0.643

Locus 10439 :

2 alleles

99.2% missing data

1	(0.656) 0.655 0.663

2	(0.344) 0.345 0.337

Locus 10440 :

2 alleles

93.4% missing data

1	(0.910) 0.898 0.922

4	(0.090) 0.102 0.078

Locus 10441 :

3 alleles

95.3% missing data

4	(0.097) 0.090 0.103

3	(0.723) 0.739 0.714

1	(0.180) 0.171 0.183

Locus 10442 :

2 alleles

98.0% missing data

2	(0.738) 0.739 0.740

1	(0.262) 0.261 0.260

Locus 10443 :

2 alleles

99.2% missing data

4	(0.574) 0.577 0.574

2	(0.426) 0.423 0.426

Locus 10444 :

2 alleles

98.4% missing data

4	(0.554) 0.556 0.543

3	(0.446) 0.444 0.457

Locus 10445 :

2 alleles

99.2% missing data

4	(0.621) 0.627 0.608

1	(0.379) 0.373 0.392

Locus 10446 :

2 alleles

97.7% missing data

4	(0.806) 0.815 0.797

1	(0.194) 0.185 0.203

Locus 10447 :

2 alleles

0.8% missing data

2	(0.894) 0.751 0.969

4	(0.106) 0.249 0.031

Locus 10448 :

2 alleles

99.2% missing data

3	(0.407) 0.404 0.412

4	(0.593) 0.596 0.588

Locus 10449 :

2 alleles

99.2% missing data

2	(0.500) 0.499 0.499

3	(0.500) 0.501 0.501

Locus 10450 :

3 alleles

98.4% missing data

2	(0.394) 0.395 0.389

1	(0.357) 0.353 0.362

3	(0.249) 0.252 0.248

Locus 10451 :

2 alleles

99.2% missing data

4	(0.327) 0.323 0.337

3	(0.673) 0.677 0.663

Locus 10452 :

2 alleles

99.2% missing data

4	(0.352) 0.356 0.354

3	(0.648) 0.644 0.646

Locus 10453 :

2 alleles

8.6% missing data

2	(0.985) 0.979 0.995

1	(0.015) 0.021 0.005

Locus 10454 :

2 alleles

99.2% missing data

1	(0.534) 0.541 0.529

3	(0.466) 0.459 0.471

Locus 10455 :

2 alleles

99.2% missing data

1	(0.648) 0.645 0.644

4	(0.352) 0.355 0.356

Locus 10456 :

2 alleles

99.2% missing data

2	(0.194) 0.190 0.196

1	(0.806) 0.810 0.804

Locus 10457 :

2 alleles

96.5% missing data

3	(0.833) 0.834 0.835

4	(0.167) 0.166 0.165

Locus 10458 :

2 alleles

99.2% missing data

1	(0.552) 0.547 0.556

4	(0.448) 0.453 0.444

Locus 10459 :

2 alleles

99.2% missing data

1	(0.476) 0.474 0.476

3	(0.524) 0.526 0.524

Locus 10460 :

2 alleles

99.2% missing data

2	(0.790) 0.787 0.788

3	(0.210) 0.213 0.212

Locus 10461 :

2 alleles

99.2% missing data

3	(0.528) 0.530 0.530

1	(0.472) 0.470 0.470

Locus 10462 :

2 alleles

95.7% missing data

2	(0.195) 0.180 0.205

3	(0.805) 0.820 0.795

Locus 10463 :

2 alleles

10.5% missing data

3	(0.978) 0.952 0.992

2	(0.022) 0.048 0.008

Locus 10464 :

2 alleles

99.2% missing data

2	(0.402) 0.400 0.408

1	(0.598) 0.600 0.592

Locus 10465 :

2 alleles

99.2% missing data

2	(0.506) 0.506 0.504

3	(0.494) 0.494 0.496

Locus 10466 :

2 alleles

99.2% missing data

1	(0.345) 0.339 0.347

3	(0.655) 0.661 0.653

Locus 10467 :

2 alleles

99.2% missing data

3	(0.602) 0.599 0.605

1	(0.398) 0.401 0.395

Locus 10468 :

2 alleles

99.2% missing data

4	(0.520) 0.526 0.518

1	(0.480) 0.474 0.482

Locus 10469 :

2 alleles

99.2% missing data

4	(0.313) 0.308 0.317

2	(0.687) 0.692 0.683

Locus 10470 :

2 alleles

99.2% missing data

4	(0.690) 0.685 0.693

1	(0.310) 0.315 0.307

Locus 10471 :

2 alleles

99.2% missing data

4	(0.546) 0.534 0.553

2	(0.454) 0.466 0.447

Locus 10472 :

2 alleles

99.2% missing data

3	(0.711) 0.710 0.709

1	(0.289) 0.290 0.291

Locus 10473 :

2 alleles

8.2% missing data

2	(0.984) 0.978 0.995

1	(0.016) 0.022 0.005

Locus 10474 :

2 alleles

99.2% missing data

1	(0.739) 0.738 0.733

4	(0.261) 0.262 0.267

Locus 10475 :

2 alleles

99.2% missing data

3	(0.517) 0.518 0.524

4	(0.483) 0.482 0.476

Locus 10476 :

2 alleles

99.2% missing data

2	(0.299) 0.295 0.304

3	(0.701) 0.705 0.696

Locus 10477 :

2 alleles

99.2% missing data

3	(0.505) 0.507 0.499

2	(0.495) 0.493 0.501

Locus 10478 :

2 alleles

90.6% missing data

3	(0.906) 0.922 0.897

4	(0.094) 0.078 0.103

Locus 10479 :

2 alleles

98.0% missing data

4	(0.693) 0.686 0.703

3	(0.307) 0.314 0.297

Locus 10480 :

2 alleles

99.2% missing data

2	(0.571) 0.562 0.570

3	(0.429) 0.438 0.430

Locus 10481 :

2 alleles

95.3% missing data

2	(0.198) 0.196 0.184

3	(0.802) 0.804 0.816

Locus 10482 :

2 alleles

98.8% missing data

4	(0.698) 0.698 0.701

2	(0.302) 0.302 0.299

Locus 10483 :

2 alleles

99.2% missing data

1	(0.493) 0.489 0.497

2	(0.507) 0.511 0.503

Locus 10484 :

2 alleles

99.2% missing data

3	(0.418) 0.419 0.423

2	(0.582) 0.581 0.577

Locus 10485 :

2 alleles

99.2% missing data

1	(0.524) 0.527 0.519

4	(0.476) 0.473 0.481

Locus 10486 :

2 alleles

95.3% missing data

4	(0.861) 0.852 0.877

2	(0.139) 0.148 0.123

Locus 10487 :

2 alleles

99.2% missing data

2	(0.638) 0.633 0.642

4	(0.362) 0.367 0.358

Locus 10488 :

2 alleles

99.2% missing data

2	(0.669) 0.664 0.664

4	(0.331) 0.336 0.336

Locus 10489 :

2 alleles

99.2% missing data

2	(0.706) 0.710 0.701

3	(0.294) 0.290 0.299

Locus 10490 :

2 alleles

99.2% missing data

3	(0.425) 0.430 0.429

1	(0.575) 0.570 0.571

Locus 10491 :

2 alleles

99.2% missing data

1	(0.558) 0.559 0.563

2	(0.442) 0.441 0.437

Locus 10492 :

2 alleles

99.2% missing data

1	(0.573) 0.561 0.580

4	(0.427) 0.439 0.420

Locus 10493 :

2 alleles

99.2% missing data

2	(0.721) 0.717 0.721

1	(0.279) 0.283 0.279

Locus 10494 :

2 alleles

99.2% missing data

1	(0.584) 0.583 0.587

2	(0.416) 0.417 0.413

Locus 10495 :

2 alleles

98.0% missing data

3	(0.610) 0.620 0.607

2	(0.390) 0.380 0.393

Locus 10496 :

3 alleles

98.8% missing data

3	(0.377) 0.371 0.379

4	(0.297) 0.293 0.306

1	(0.326) 0.336 0.314

Locus 10497 :

3 alleles

98.8% missing data

2	(0.380) 0.389 0.374

3	(0.422) 0.414 0.427

1	(0.198) 0.196 0.199

Locus 10498 :

2 alleles

98.4% missing data

4	(0.468) 0.471 0.471

3	(0.532) 0.529 0.529

Locus 10499 :

2 alleles

99.2% missing data

4	(0.427) 0.423 0.429

2	(0.573) 0.577 0.571

Locus 10500 :

2 alleles

99.2% missing data

1	(0.324) 0.316 0.327

4	(0.676) 0.684 0.673

Locus 10501 :

2 alleles

96.9% missing data

1	(0.368) 0.377 0.355

3	(0.632) 0.623 0.645

Locus 10502 :

2 alleles

99.2% missing data

3	(0.258) 0.252 0.262

2	(0.742) 0.748 0.738

Locus 10503 :

3 alleles

98.0% missing data

1	(0.585) 0.587 0.582

4	(0.183) 0.180 0.189

2	(0.232) 0.233 0.230

Locus 10504 :

2 alleles

99.2% missing data

3	(0.470) 0.456 0.469

1	(0.530) 0.544 0.531

Locus 10505 :

2 alleles

97.7% missing data

1	(0.757) 0.760 0.760

2	(0.243) 0.240 0.240

Locus 10506 :

2 alleles

99.2% missing data

3	(0.286) 0.287 0.291

2	(0.714) 0.713 0.709

Locus 10507 :

2 alleles

90.6% missing data

2	(0.929) 0.936 0.932

1	(0.071) 0.064 0.068

Locus 10508 :

2 alleles

99.2% missing data

3	(0.656) 0.649 0.660

2	(0.344) 0.351 0.340

Locus 10509 :

2 alleles

98.4% missing data

1	(0.735) 0.738 0.732

3	(0.265) 0.262 0.268

Locus 10510 :

2 alleles

99.2% missing data

2	(0.403) 0.409 0.398

3	(0.597) 0.591 0.602

Locus 10511 :

2 alleles

95.3% missing data

3	(0.851) 0.849 0.859

2	(0.149) 0.151 0.141

Locus 10512 :

2 alleles

99.2% missing data

1	(0.528) 0.522 0.532

2	(0.472) 0.478 0.468

Locus 10513 :

2 alleles

98.4% missing data

1	(0.686) 0.682 0.689

3	(0.314) 0.318 0.311

Locus 10514 :

3 alleles

98.0% missing data

2	(0.227) 0.224 0.231

3	(0.337) 0.336 0.343

1	(0.436) 0.440 0.426

Locus 10515 :

2 alleles

99.2% missing data

3	(0.580) 0.583 0.569

1	(0.420) 0.417 0.431

Locus 10516 :

2 alleles

98.0% missing data

1	(0.676) 0.685 0.672

3	(0.324) 0.315 0.328

Locus 10517 :

2 alleles

99.2% missing data

1	(0.433) 0.432 0.438

4	(0.567) 0.568 0.562

Locus 10518 :

2 alleles

98.4% missing data

3	(0.697) 0.689 0.704

2	(0.303) 0.311 0.296

Locus 10519 :

2 alleles

98.4% missing data

3	(0.748) 0.750 0.749

1	(0.252) 0.250 0.251

Locus 10520 :

2 alleles

8.2% missing data

2	(0.985) 0.977 0.995

3	(0.015) 0.023 0.005

Locus 10521 :

2 alleles

98.8% missing data

1	(0.396) 0.392 0.408

4	(0.604) 0.608 0.592

Locus 10522 :

2 alleles

99.2% missing data

3	(0.754) 0.754 0.748

1	(0.246) 0.246 0.252

Locus 10523 :

2 alleles

98.8% missing data

1	(0.389) 0.387 0.394

3	(0.611) 0.613 0.606

Locus 10524 :

2 alleles

96.1% missing data

3	(0.776) 0.794 0.771

1	(0.224) 0.206 0.229

Locus 10525 :

2 alleles

99.2% missing data

3	(0.280) 0.272 0.286

2	(0.720) 0.728 0.714

Locus 10526 :

2 alleles

99.2% missing data

2	(0.705) 0.697 0.707

3	(0.295) 0.303 0.293

Locus 10527 :

2 alleles

99.2% missing data

4	(0.391) 0.388 0.390

1	(0.609) 0.612 0.610

Locus 10528 :

2 alleles

99.2% missing data

1	(0.398) 0.391 0.393

3	(0.602) 0.609 0.607

Locus 10529 :

2 alleles

99.2% missing data

3	(0.423) 0.426 0.420

1	(0.577) 0.574 0.580

Locus 10530 :

2 alleles

99.2% missing data

1	(0.211) 0.209 0.217

3	(0.789) 0.791 0.783

Locus 10531 :

2 alleles

98.8% missing data

1	(0.564) 0.556 0.577

2	(0.436) 0.444 0.423

Locus 10532 :

2 alleles

98.8% missing data

2	(0.333) 0.326 0.342

3	(0.667) 0.674 0.658

Locus 10533 :

2 alleles

99.2% missing data

4	(0.654) 0.657 0.652

1	(0.346) 0.343 0.348

Locus 10534 :

3 alleles

97.3% missing data

1	(0.822) 0.823 0.815

4	(0.087) 0.084 0.090

2	(0.091) 0.092 0.094

Locus 10535 :

3 alleles

98.4% missing data

1	(0.278) 0.280 0.289

3	(0.221) 0.216 0.223

2	(0.501) 0.504 0.488

Locus 10536 :

2 alleles

98.8% missing data

1	(0.740) 0.728 0.748

2	(0.260) 0.272 0.252

Locus 10537 :

2 alleles

99.2% missing data

2	(0.602) 0.597 0.603

4	(0.398) 0.403 0.397

Locus 10538 :

2 alleles

99.2% missing data

1	(0.664) 0.666 0.660

3	(0.336) 0.334 0.340

Locus 10539 :

2 alleles

99.2% missing data

4	(0.331) 0.334 0.331

3	(0.669) 0.666 0.669

Locus 10540 :

2 alleles

96.1% missing data

4	(0.734) 0.739 0.739

2	(0.266) 0.261 0.261

Locus 10541 :

2 alleles

99.2% missing data

3	(0.737) 0.743 0.727

1	(0.263) 0.257 0.273

Locus 10542 :

2 alleles

99.2% missing data

4	(0.608) 0.601 0.607

3	(0.392) 0.399 0.393

Locus 10543 :

2 alleles

98.8% missing data

4	(0.282) 0.275 0.294

3	(0.718) 0.725 0.706

Locus 10544 :

2 alleles

99.2% missing data

2	(0.167) 0.168 0.169

3	(0.833) 0.832 0.831

Locus 10545 :

2 alleles

99.2% missing data

4	(0.729) 0.733 0.723

1	(0.271) 0.267 0.277

Locus 10546 :

2 alleles

99.2% missing data

2	(0.834) 0.828 0.835

4	(0.166) 0.172 0.165

Locus 10547 :

2 alleles

99.2% missing data

2	(0.769) 0.774 0.764

4	(0.231) 0.226 0.236

Locus 10548 :

2 alleles

99.2% missing data

1	(0.653) 0.655 0.653

4	(0.347) 0.345 0.347

Locus 10549 :

2 alleles

98.8% missing data

1	(0.481) 0.480 0.477

4	(0.519) 0.520 0.523

Locus 10550 :

2 alleles

99.2% missing data

1	(0.769) 0.773 0.765

2	(0.231) 0.227 0.235

Locus 10551 :

2 alleles

96.5% missing data

3	(0.759) 0.765 0.761

2	(0.241) 0.235 0.239

Locus 10552 :

2 alleles

99.2% missing data

4	(0.473) 0.470 0.480

3	(0.527) 0.530 0.520

Locus 10553 :

2 alleles

98.8% missing data

1	(0.477) 0.475 0.491

2	(0.523) 0.525 0.509

Locus 10554 :

3 alleles

98.8% missing data

1	(0.433) 0.429 0.433

2	(0.323) 0.331 0.314

4	(0.244) 0.240 0.253

Locus 10555 :

2 alleles

99.2% missing data

4	(0.691) 0.694 0.687

1	(0.309) 0.306 0.313

Locus 10556 :

3 alleles

98.8% missing data

4	(0.189) 0.192 0.186

1	(0.405) 0.402 0.403

3	(0.406) 0.406 0.411

Locus 10557 :

2 alleles

99.2% missing data

4	(0.609) 0.611 0.602

1	(0.391) 0.389 0.398

Locus 10558 :

2 alleles

99.2% missing data

2	(0.612) 0.612 0.609

4	(0.388) 0.388 0.391

Locus 10559 :

2 alleles

99.2% missing data

2	(0.646) 0.642 0.650

3	(0.354) 0.358 0.350

Locus 10560 :

2 alleles

99.2% missing data

2	(0.657) 0.654 0.661

3	(0.343) 0.346 0.339

Locus 10561 :

2 alleles

99.2% missing data

3	(0.210) 0.206 0.216

2	(0.790) 0.794 0.784

Locus 10562 :

2 alleles

96.1% missing data

1	(0.926) 0.933 0.920

4	(0.074) 0.067 0.080

Locus 10563 :

2 alleles

98.4% missing data

2	(0.746) 0.736 0.755

1	(0.254) 0.264 0.245

Locus 10564 :

2 alleles

99.2% missing data

3	(0.624) 0.620 0.627

2	(0.376) 0.380 0.373

Locus 10565 :

2 alleles

97.7% missing data

1	(0.802) 0.801 0.808

3	(0.198) 0.199 0.192

Locus 10566 :

2 alleles

99.2% missing data

2	(0.363) 0.356 0.374

3	(0.637) 0.644 0.626

Locus 10567 :

2 alleles

99.2% missing data

3	(0.646) 0.645 0.648

4	(0.354) 0.355 0.352

Locus 10568 :

2 alleles

98.8% missing data

1	(0.607) 0.606 0.607

3	(0.393) 0.394 0.393

Locus 10569 :

2 alleles

96.9% missing data

4	(0.792) 0.796 0.791

2	(0.208) 0.204 0.209

Locus 10570 :

3 alleles

98.8% missing data

3	(0.412) 0.405 0.409

2	(0.321) 0.324 0.330

1	(0.267) 0.271 0.262

Locus 10571 :

2 alleles

99.2% missing data

1	(0.437) 0.425 0.441

3	(0.563) 0.575 0.559

Locus 10572 :

2 alleles

99.2% missing data

2	(0.489) 0.484 0.487

4	(0.511) 0.516 0.513

Locus 10573 :

2 alleles

99.2% missing data

4	(0.575) 0.581 0.577

3	(0.425) 0.419 0.423

Locus 10574 :

2 alleles

96.1% missing data

1	(0.648) 0.663 0.654

3	(0.352) 0.337 0.346

Locus 10575 :

2 alleles

99.2% missing data

3	(0.356) 0.352 0.358

2	(0.644) 0.648 0.642

Locus 10576 :

2 alleles

99.2% missing data

2	(0.431) 0.437 0.428

4	(0.569) 0.563 0.572

Locus 10577 :

2 alleles

99.2% missing data

1	(0.463) 0.455 0.467

2	(0.537) 0.545 0.533

Locus 10578 :

2 alleles

97.7% missing data

2	(0.701) 0.693 0.715

4	(0.299) 0.307 0.285

Locus 10579 :

2 alleles

98.8% missing data

2	(0.478) 0.484 0.477

1	(0.522) 0.516 0.523

Locus 10580 :

2 alleles

98.8% missing data

2	(0.773) 0.769 0.787

1	(0.227) 0.231 0.213

Locus 10581 :

2 alleles

91.0% missing data

4	(0.858) 0.862 0.859

2	(0.142) 0.138 0.141

Locus 10582 :

2 alleles

98.0% missing data

1	(0.747) 0.756 0.740

4	(0.253) 0.244 0.260

Locus 10583 :

2 alleles

97.7% missing data

3	(0.783) 0.774 0.790

4	(0.217) 0.226 0.210

Locus 10584 :

2 alleles

8.2% missing data

2	(0.985) 0.977 0.995

3	(0.015) 0.023 0.005

Locus 10585 :

2 alleles

98.4% missing data

2	(0.617) 0.622 0.618

3	(0.383) 0.378 0.382

Locus 10586 :

2 alleles

99.2% missing data

2	(0.475) 0.470 0.478

3	(0.525) 0.530 0.522

Locus 10587 :

2 alleles

96.5% missing data

1	(0.791) 0.801 0.784

2	(0.209) 0.199 0.216

Locus 10588 :

2 alleles

99.2% missing data

4	(0.587) 0.582 0.590

3	(0.413) 0.418 0.410

Locus 10589 :

2 alleles

99.2% missing data

3	(0.370) 0.364 0.378

1	(0.630) 0.636 0.622

Locus 10590 :

2 alleles

98.8% missing data

3	(0.645) 0.636 0.656

4	(0.355) 0.364 0.344

Locus 10591 :

2 alleles

99.2% missing data

2	(0.696) 0.694 0.692

3	(0.304) 0.306 0.308

Locus 10592 :

2 alleles

99.2% missing data

1	(0.295) 0.292 0.299

3	(0.705) 0.708 0.701

Locus 10593 :

2 alleles

99.2% missing data

1	(0.579) 0.587 0.577

4	(0.421) 0.413 0.423

Locus 10594 :

2 alleles

99.2% missing data

1	(0.532) 0.521 0.534

4	(0.468) 0.479 0.466

Locus 10595 :

2 alleles

0.4% missing data

3	(0.986) 0.981 0.996

2	(0.014) 0.019 0.004

Locus 10596 :

2 alleles

98.8% missing data

2	(0.685) 0.683 0.684

1	(0.315) 0.317 0.316

Locus 10597 :

2 alleles

98.8% missing data

1	(0.650) 0.650 0.653

3	(0.350) 0.350 0.347

Locus 10598 :

2 alleles

98.0% missing data

3	(0.727) 0.733 0.722

1	(0.273) 0.267 0.278

Locus 10599 :

2 alleles

99.2% missing data

3	(0.456) 0.453 0.458

4	(0.544) 0.547 0.542

Locus 10600 :

2 alleles

99.2% missing data

3	(0.318) 0.316 0.321

1	(0.682) 0.684 0.679

Locus 10601 :

2 alleles

95.7% missing data

2	(0.897) 0.907 0.890

3	(0.103) 0.093 0.110

Locus 10602 :

2 alleles

99.2% missing data

4	(0.143) 0.147 0.142

2	(0.857) 0.853 0.858

Locus 10603 :

2 alleles

98.8% missing data

1	(0.351) 0.345 0.350

3	(0.649) 0.655 0.650

Locus 10604 :

2 alleles

99.2% missing data

1	(0.670) 0.671 0.668

2	(0.330) 0.329 0.332

Locus 10605 :

2 alleles

98.8% missing data

3	(0.626) 0.620 0.631

2	(0.374) 0.380 0.369

Locus 10606 :

2 alleles

98.0% missing data

1	(0.731) 0.720 0.740

3	(0.269) 0.280 0.260

Locus 10607 :

2 alleles

98.4% missing data

3	(0.510) 0.512 0.506

2	(0.490) 0.488 0.494

Locus 10608 :

2 alleles

99.2% missing data

3	(0.229) 0.235 0.226

1	(0.771) 0.765 0.774

Locus 10609 :

2 alleles

99.2% missing data

4	(0.571) 0.568 0.576

1	(0.429) 0.432 0.424

Locus 10610 :

2 alleles

99.2% missing data

4	(0.488) 0.494 0.490

1	(0.512) 0.506 0.510

Locus 10611 :

2 alleles

98.8% missing data

1	(0.546) 0.542 0.552

3	(0.454) 0.458 0.448

Locus 10612 :

2 alleles

99.2% missing data

2	(0.253) 0.250 0.255

4	(0.747) 0.750 0.745

Locus 10613 :

2 alleles

99.2% missing data

4	(0.762) 0.762 0.761

3	(0.238) 0.238 0.239

Locus 10614 :

2 alleles

96.5% missing data

1	(0.883) 0.883 0.887

4	(0.117) 0.117 0.113

Locus 10615 :

2 alleles

99.2% missing data

4	(0.262) 0.261 0.269

1	(0.738) 0.739 0.731

Locus 10616 :

2 alleles

96.1% missing data

1	(0.197) 0.185 0.200

3	(0.803) 0.815 0.800

Locus 10617 :

2 alleles

99.2% missing data

4	(0.583) 0.586 0.575

2	(0.417) 0.414 0.425

Locus 10618 :

2 alleles

99.2% missing data

1	(0.423) 0.426 0.421

4	(0.577) 0.574 0.579

Locus 10619 :

2 alleles

99.2% missing data

2	(0.751) 0.748 0.758

4	(0.249) 0.252 0.242

Locus 10620 :

2 alleles

96.1% missing data

4	(0.295) 0.277 0.310

3	(0.705) 0.723 0.690

Locus 10621 :

2 alleles

98.4% missing data

3	(0.846) 0.852 0.837

4	(0.154) 0.148 0.163

Locus 10622 :

2 alleles

97.7% missing data

1	(0.207) 0.210 0.206

4	(0.793) 0.790 0.794

Locus 10623 :

2 alleles

99.2% missing data

2	(0.738) 0.739 0.734

4	(0.262) 0.261 0.266

Locus 10624 :

2 alleles

99.2% missing data

2	(0.636) 0.643 0.631

4	(0.364) 0.357 0.369

Locus 10625 :

2 alleles

99.2% missing data

4	(0.810) 0.809 0.808

2	(0.190) 0.191 0.192

Locus 10626 :

2 alleles

98.8% missing data

1	(0.329) 0.332 0.336

2	(0.671) 0.668 0.664

Locus 10627 :

2 alleles

91.0% missing data

3	(0.909) 0.925 0.901

2	(0.091) 0.075 0.099

Locus 10628 :

2 alleles

98.4% missing data

2	(0.686) 0.693 0.679

4	(0.314) 0.307 0.321

Locus 10629 :

2 alleles

93.0% missing data

3	(0.865) 0.866 0.865

1	(0.135) 0.134 0.135

Locus 10630 :

2 alleles

99.2% missing data

1	(0.580) 0.576 0.588

4	(0.420) 0.424 0.412

Locus 10631 :

2 alleles

99.2% missing data

4	(0.430) 0.427 0.432

3	(0.570) 0.573 0.568

Locus 10632 :

2 alleles

95.3% missing data

1	(0.918) 0.926 0.914

4	(0.082) 0.074 0.086

Locus 10633 :

2 alleles

98.4% missing data

2	(0.437) 0.426 0.451

4	(0.563) 0.574 0.549

Locus 10634 :

2 alleles

99.2% missing data

1	(0.696) 0.698 0.693

2	(0.304) 0.302 0.307

Locus 10635 :

2 alleles

8.2% missing data

4	(0.985) 0.978 0.996

3	(0.015) 0.022 0.004

Locus 10636 :

2 alleles

99.2% missing data

2	(0.357) 0.353 0.364

1	(0.643) 0.647 0.636

Locus 10637 :

2 alleles

96.1% missing data

4	(0.845) 0.849 0.845

3	(0.155) 0.151 0.155

Locus 10638 :

3 alleles

98.4% missing data

1	(0.294) 0.282 0.301

3	(0.390) 0.394 0.392

2	(0.316) 0.325 0.307

Locus 10639 :

2 alleles

99.2% missing data

2	(0.476) 0.477 0.473

1	(0.524) 0.523 0.527

Locus 10640 :

2 alleles

99.2% missing data

4	(0.761) 0.764 0.761

1	(0.239) 0.236 0.239

Locus 10641 :

2 alleles

99.2% missing data

2	(0.640) 0.636 0.634

1	(0.360) 0.364 0.366

Locus 10642 :

2 alleles

99.2% missing data

1	(0.387) 0.384 0.390

4	(0.613) 0.616 0.610

Locus 10643 :

4 alleles

89.5% missing data

3	(0.749) 0.791 0.725

4	(0.102) 0.079 0.117

2	(0.066) 0.051 0.075

1	(0.084) 0.078 0.082

Locus 10644 :

2 alleles

96.5% missing data

4	(0.574) 0.590 0.554

1	(0.426) 0.410 0.446

Locus 10645 :

2 alleles

96.5% missing data

1	(0.142) 0.138 0.140

4	(0.858) 0.862 0.860

Locus 10646 :

2 alleles

0.0% missing data

1	(0.914) 0.791 0.974

3	(0.086) 0.209 0.026

Locus 10647 :

2 alleles

99.2% missing data

3	(0.263) 0.260 0.267

2	(0.737) 0.740 0.733

Locus 10648 :

2 alleles

98.8% missing data

3	(0.733) 0.738 0.725

1	(0.267) 0.262 0.275

Locus 10649 :

2 alleles

99.2% missing data

1	(0.494) 0.503 0.485

4	(0.506) 0.497 0.515

Locus 10650 :

2 alleles

99.2% missing data

1	(0.558) 0.562 0.554

2	(0.442) 0.438 0.446

Locus 10651 :

2 alleles

99.2% missing data

4	(0.380) 0.376 0.393

1	(0.620) 0.624 0.607

Locus 10652 :

2 alleles

98.8% missing data

3	(0.679) 0.685 0.674

1	(0.321) 0.315 0.326

Locus 10653 :

2 alleles

99.2% missing data

3	(0.629) 0.634 0.626

2	(0.371) 0.366 0.374

Locus 10654 :

2 alleles

99.2% missing data

1	(0.662) 0.663 0.667

4	(0.338) 0.337 0.333

Locus 10655 :

2 alleles

99.2% missing data

4	(0.615) 0.619 0.609

2	(0.385) 0.381 0.391

Locus 10656 :

2 alleles

99.2% missing data

4	(0.499) 0.499 0.503

3	(0.501) 0.501 0.497

Locus 10657 :

2 alleles

99.2% missing data

1	(0.426) 0.419 0.431

2	(0.574) 0.581 0.569

Locus 10658 :

2 alleles

99.2% missing data

1	(0.490) 0.491 0.493

3	(0.510) 0.509 0.507

Locus 10659 :

2 alleles

99.2% missing data

4	(0.557) 0.561 0.547

1	(0.443) 0.439 0.453

Locus 10660 :

2 alleles

99.2% missing data

3	(0.790) 0.790 0.786

2	(0.210) 0.210 0.214

Locus 10661 :

2 alleles

98.0% missing data

4	(0.765) 0.759 0.774

3	(0.235) 0.241 0.226

Locus 10662 :

2 alleles

98.8% missing data

1	(0.377) 0.380 0.377

2	(0.623) 0.620 0.623

Locus 10663 :

2 alleles

99.2% missing data

2	(0.131) 0.128 0.136

1	(0.869) 0.872 0.864

Locus 10664 :

2 alleles

98.4% missing data

3	(0.676) 0.669 0.675

4	(0.324) 0.331 0.325

Locus 10665 :

2 alleles

99.2% missing data

3	(0.729) 0.728 0.723

2	(0.271) 0.272 0.277

Locus 10666 :

2 alleles

91.4% missing data

1	(0.917) 0.926 0.918

2	(0.083) 0.074 0.082

Locus 10667 :

2 alleles

96.5% missing data

3	(0.821) 0.825 0.823

2	(0.179) 0.175 0.177

Locus 10668 :

2 alleles

91.8% missing data

3	(0.909) 0.904 0.919

2	(0.091) 0.096 0.081

Locus 10669 :

3 alleles

98.4% missing data

3	(0.307) 0.319 0.307

2	(0.556) 0.546 0.556

4	(0.137) 0.135 0.137

Locus 10670 :

2 alleles

99.2% missing data

2	(0.575) 0.580 0.575

3	(0.425) 0.420 0.425

Locus 10671 :

2 alleles

99.2% missing data

4	(0.415) 0.415 0.415

2	(0.585) 0.585 0.585

Locus 10672 :

2 alleles

98.8% missing data

2	(0.480) 0.471 0.487

1	(0.520) 0.529 0.513

Locus 10673 :

2 alleles

99.2% missing data

4	(0.210) 0.208 0.208

2	(0.790) 0.792 0.792

Locus 10674 :

2 alleles

98.0% missing data

2	(0.842) 0.842 0.847

3	(0.158) 0.158 0.153

Locus 10675 :

2 alleles

99.2% missing data

2	(0.560) 0.561 0.559

1	(0.440) 0.439 0.441

Locus 10676 :

2 alleles

99.2% missing data

3	(0.522) 0.525 0.525

2	(0.478) 0.475 0.475

Locus 10677 :

2 alleles

99.2% missing data

1	(0.209) 0.208 0.212

2	(0.791) 0.792 0.788

Locus 10678 :

2 alleles

98.8% missing data

4	(0.503) 0.507 0.509

1	(0.497) 0.493 0.491

Locus 10679 :

2 alleles

99.2% missing data

4	(0.416) 0.417 0.415

1	(0.584) 0.583 0.585

Locus 10680 :

2 alleles

98.4% missing data

3	(0.526) 0.517 0.532

2	(0.474) 0.483 0.468

Locus 10681 :

2 alleles

99.2% missing data

3	(0.400) 0.399 0.404

4	(0.600) 0.601 0.596

Locus 10682 :

2 alleles

98.0% missing data

4	(0.582) 0.576 0.600

1	(0.418) 0.424 0.400

Locus 10683 :

2 alleles

99.2% missing data

4	(0.327) 0.325 0.330

2	(0.673) 0.675 0.670

Locus 10684 :

2 alleles

95.7% missing data

4	(0.830) 0.830 0.838

2	(0.170) 0.170 0.162

Locus 10685 :

2 alleles

99.2% missing data

2	(0.450) 0.450 0.454

4	(0.550) 0.550 0.546

Locus 10686 :

2 alleles

99.2% missing data

4	(0.626) 0.632 0.626

2	(0.374) 0.368 0.374

Locus 10687 :

2 alleles

99.2% missing data

1	(0.405) 0.403 0.413

4	(0.595) 0.597 0.587

Locus 10688 :

2 alleles

99.2% missing data

2	(0.484) 0.494 0.481

4	(0.516) 0.506 0.519

Locus 10689 :

2 alleles

98.4% missing data

2	(0.545) 0.557 0.546

3	(0.455) 0.443 0.454

Locus 10690 :

2 alleles

99.2% missing data

4	(0.767) 0.764 0.767

3	(0.233) 0.236 0.233

Locus 10691 :

2 alleles

99.2% missing data

4	(0.493) 0.492 0.492

2	(0.507) 0.508 0.508

Locus 10692 :

2 alleles

0.0% missing data

2	(0.986) 0.983 0.995

3	(0.014) 0.017 0.005

Locus 10693 :

2 alleles

99.2% missing data

3	(0.265) 0.267 0.261

2	(0.735) 0.733 0.739

Locus 10694 :

2 alleles

98.4% missing data

2	(0.558) 0.563 0.557

3	(0.442) 0.437 0.443

Locus 10695 :

2 alleles

98.0% missing data

2	(0.310) 0.310 0.310

4	(0.690) 0.690 0.690

Locus 10696 :

2 alleles

98.0% missing data

3	(0.697) 0.701 0.702

2	(0.303) 0.299 0.298

Locus 10697 :

2 alleles

97.7% missing data

4	(0.586) 0.582 0.589

1	(0.414) 0.418 0.411

Locus 10698 :

2 alleles

99.2% missing data

2	(0.511) 0.510 0.510

4	(0.489) 0.490 0.490

Locus 10699 :

2 alleles

98.8% missing data

3	(0.614) 0.611 0.610

4	(0.386) 0.389 0.390

Locus 10700 :

2 alleles

98.4% missing data

3	(0.760) 0.752 0.764

4	(0.240) 0.248 0.236

Locus 10701 :

2 alleles

99.2% missing data

4	(0.644) 0.637 0.651

3	(0.356) 0.363 0.349

Locus 10702 :

2 alleles

99.2% missing data

3	(0.472) 0.476 0.472

2	(0.528) 0.524 0.528

Locus 10703 :

2 alleles

99.2% missing data

3	(0.711) 0.710 0.711

4	(0.289) 0.290 0.289

Locus 10704 :

2 alleles

99.2% missing data

1	(0.653) 0.648 0.654

2	(0.347) 0.352 0.346

Locus 10705 :

3 alleles

98.8% missing data

4	(0.311) 0.310 0.309

2	(0.221) 0.218 0.224

1	(0.468) 0.472 0.467

Locus 10706 :

2 alleles

99.2% missing data

2	(0.434) 0.426 0.437

4	(0.566) 0.574 0.563

Locus 10707 :

2 alleles

99.2% missing data

1	(0.258) 0.259 0.272

4	(0.742) 0.741 0.728

Locus 10708 :

2 alleles

98.8% missing data

2	(0.857) 0.853 0.857

3	(0.143) 0.147 0.143

Locus 10709 :

2 alleles

98.8% missing data

1	(0.337) 0.337 0.349

3	(0.663) 0.663 0.651

Locus 10710 :

2 alleles

99.2% missing data

3	(0.298) 0.304 0.294

2	(0.702) 0.696 0.706

Locus 10711 :

2 alleles

99.2% missing data

1	(0.679) 0.681 0.677

4	(0.321) 0.319 0.323

Locus 10712 :

2 alleles

99.2% missing data

4	(0.376) 0.379 0.373

1	(0.624) 0.621 0.627

Locus 10713 :

2 alleles

99.2% missing data

4	(0.499) 0.508 0.497

1	(0.501) 0.492 0.503

Locus 10714 :

2 alleles

98.8% missing data

1	(0.633) 0.634 0.629

2	(0.367) 0.366 0.371

Locus 10715 :

2 alleles

99.2% missing data

2	(0.241) 0.238 0.249

4	(0.759) 0.762 0.751

Locus 10716 :

2 alleles

97.7% missing data

3	(0.180) 0.181 0.180

4	(0.820) 0.819 0.820

Locus 10717 :

2 alleles

99.2% missing data

2	(0.674) 0.670 0.673

3	(0.326) 0.330 0.327

Locus 10718 :

2 alleles

98.8% missing data

1	(0.728) 0.733 0.716

3	(0.272) 0.267 0.284

Locus 10719 :

2 alleles

99.2% missing data

1	(0.603) 0.604 0.605

2	(0.397) 0.396 0.395

Locus 10720 :

2 alleles

98.0% missing data

1	(0.611) 0.616 0.614

3	(0.389) 0.384 0.386

Locus 10721 :

2 alleles

99.2% missing data

1	(0.554) 0.548 0.554

2	(0.446) 0.452 0.446

Locus 10722 :

2 alleles

99.2% missing data

3	(0.458) 0.456 0.460

1	(0.542) 0.544 0.540

Locus 10723 :

2 alleles

98.8% missing data

2	(0.701) 0.693 0.705

1	(0.299) 0.307 0.295

Locus 10724 :

2 alleles

98.4% missing data

2	(0.312) 0.316 0.311

1	(0.688) 0.684 0.689

Locus 10725 :

2 alleles

99.2% missing data

3	(0.597) 0.592 0.604

2	(0.403) 0.408 0.396

Locus 10726 :

2 alleles

99.2% missing data

2	(0.501) 0.487 0.509

1	(0.499) 0.513 0.491

Locus 10727 :

2 alleles

99.2% missing data

4	(0.467) 0.474 0.458

1	(0.533) 0.526 0.542

Locus 10728 :

2 alleles

99.2% missing data

2	(0.366) 0.367 0.364

1	(0.634) 0.633 0.636

Locus 10729 :

2 alleles

99.2% missing data

3	(0.763) 0.768 0.749

4	(0.237) 0.232 0.251

Locus 10730 :

2 alleles

99.2% missing data

4	(0.254) 0.259 0.253

3	(0.746) 0.741 0.747

Locus 10731 :

2 alleles

99.2% missing data

2	(0.353) 0.357 0.351

1	(0.647) 0.643 0.649

Locus 10732 :

2 alleles

99.2% missing data

3	(0.363) 0.357 0.367

1	(0.637) 0.643 0.633

Locus 10733 :

2 alleles

98.0% missing data

4	(0.223) 0.214 0.237

3	(0.777) 0.786 0.763

Locus 10734 :

2 alleles

99.2% missing data

2	(0.624) 0.618 0.627

1	(0.376) 0.382 0.373

Locus 10735 :

2 alleles

99.2% missing data

3	(0.297) 0.302 0.289

4	(0.703) 0.698 0.711

Locus 10736 :

2 alleles

99.2% missing data

3	(0.573) 0.568 0.578

2	(0.427) 0.432 0.422

Locus 10737 :

2 alleles

97.3% missing data

4	(0.676) 0.680 0.682

1	(0.324) 0.320 0.318

Locus 10738 :

2 alleles

99.2% missing data

1	(0.634) 0.635 0.631

3	(0.366) 0.365 0.369

Locus 10739 :

2 alleles

99.2% missing data

1	(0.839) 0.844 0.837

4	(0.161) 0.156 0.163

Locus 10740 :

2 alleles

99.2% missing data

4	(0.610) 0.607 0.607

1	(0.390) 0.393 0.393

Locus 10741 :

2 alleles

99.2% missing data

2	(0.594) 0.595 0.591

4	(0.406) 0.405 0.409

Locus 10742 :

2 alleles

97.7% missing data

4	(0.625) 0.620 0.652

3	(0.375) 0.380 0.348

Locus 10743 :

2 alleles

98.8% missing data

1	(0.632) 0.631 0.635

2	(0.368) 0.369 0.365

Locus 10744 :

2 alleles

99.2% missing data

4	(0.486) 0.474 0.490

1	(0.514) 0.526 0.510

Locus 10745 :

2 alleles

99.2% missing data

3	(0.446) 0.444 0.449

4	(0.554) 0.556 0.551

Locus 10746 :

2 alleles

98.0% missing data

4	(0.618) 0.617 0.619

3	(0.382) 0.383 0.381

Locus 10747 :

2 alleles

99.2% missing data

1	(0.276) 0.275 0.281

2	(0.724) 0.725 0.719

Locus 10748 :

2 alleles

99.2% missing data

2	(0.420) 0.420 0.420

3	(0.580) 0.580 0.580

Locus 10749 :

2 alleles

98.8% missing data

2	(0.582) 0.584 0.576

4	(0.418) 0.416 0.424

Locus 10750 :

2 alleles

99.2% missing data

4	(0.350) 0.347 0.358

2	(0.650) 0.653 0.642

Locus 10751 :

2 alleles

94.5% missing data

2	(0.903) 0.905 0.908

4	(0.097) 0.095 0.092

Locus 10752 :

2 alleles

99.2% missing data

4	(0.411) 0.406 0.414

2	(0.589) 0.594 0.586

Locus 10753 :

2 alleles

99.2% missing data

4	(0.370) 0.371 0.375

2	(0.630) 0.629 0.625

Locus 10754 :

2 alleles

99.2% missing data

4	(0.301) 0.304 0.302

3	(0.699) 0.696 0.698

Locus 10755 :

2 alleles

96.9% missing data

2	(0.740) 0.755 0.736

4	(0.260) 0.245 0.264

Locus 10756 :

2 alleles

98.0% missing data

3	(0.769) 0.778 0.763

2	(0.231) 0.222 0.237

Locus 10757 :

2 alleles

91.4% missing data

2	(0.927) 0.940 0.919

4	(0.073) 0.060 0.081

Locus 10758 :

2 alleles

98.8% missing data

3	(0.723) 0.726 0.721

1	(0.277) 0.274 0.279

Locus 10759 :

2 alleles

99.2% missing data

3	(0.517) 0.513 0.517

1	(0.483) 0.487 0.483

Locus 10760 :

2 alleles

99.2% missing data

1	(0.111) 0.109 0.115

3	(0.889) 0.891 0.885

Locus 10761 :

2 alleles

99.2% missing data

3	(0.763) 0.766 0.757

1	(0.237) 0.234 0.243

Locus 10762 :

2 alleles

91.0% missing data

4	(0.772) 0.805 0.748

2	(0.228) 0.195 0.252

Locus 10763 :

2 alleles

99.2% missing data

3	(0.706) 0.705 0.705

2	(0.294) 0.295 0.295

Locus 10764 :

2 alleles

96.5% missing data

1	(0.251) 0.233 0.262

2	(0.749) 0.767 0.738

Locus 10765 :

2 alleles

99.2% missing data

3	(0.610) 0.611 0.611

1	(0.390) 0.389 0.389

Locus 10766 :

2 alleles

99.2% missing data

3	(0.667) 0.659 0.674

4	(0.333) 0.341 0.326

Locus 10767 :

2 alleles

99.2% missing data

2	(0.294) 0.303 0.294

3	(0.706) 0.697 0.706

Locus 10768 :

2 alleles

98.8% missing data

2	(0.203) 0.199 0.219

3	(0.797) 0.801 0.781

Locus 10769 :

2 alleles

98.8% missing data

1	(0.790) 0.791 0.785

3	(0.210) 0.209 0.215

Locus 10770 :

2 alleles

8.6% missing data

4	(0.985) 0.976 0.996

1	(0.015) 0.024 0.004

Locus 10771 :

2 alleles

92.2% missing data

1	(0.855) 0.879 0.846

3	(0.145) 0.121 0.154

Locus 10772 :

2 alleles

90.2% missing data

3	(0.940) 0.952 0.930

4	(0.060) 0.048 0.070

Locus 10773 :

2 alleles

99.2% missing data

2	(0.312) 0.310 0.316

3	(0.688) 0.690 0.684

Locus 10774 :

2 alleles

99.2% missing data

4	(0.766) 0.760 0.767

2	(0.234) 0.240 0.233

Locus 10775 :

2 alleles

99.2% missing data

1	(0.366) 0.366 0.367

2	(0.634) 0.634 0.633

Locus 10776 :

2 alleles

99.2% missing data

1	(0.754) 0.747 0.759

3	(0.246) 0.253 0.241

Locus 10777 :

2 alleles

98.8% missing data

2	(0.557) 0.560 0.559

4	(0.443) 0.440 0.441

Locus 10778 :

2 alleles

3.1% missing data

4	(0.983) 0.980 0.994

1	(0.017) 0.020 0.006

Locus 10779 :

2 alleles

94.5% missing data

1	(0.165) 0.168 0.151

2	(0.835) 0.832 0.849

Locus 10780 :

2 alleles

99.2% missing data

1	(0.598) 0.592 0.600

2	(0.402) 0.408 0.400

Locus 10781 :

2 alleles

99.2% missing data

2	(0.526) 0.521 0.524

1	(0.474) 0.479 0.476

Locus 10782 :

2 alleles

99.2% missing data

3	(0.458) 0.455 0.454

1	(0.542) 0.545 0.546

Locus 10783 :

2 alleles

99.2% missing data

1	(0.425) 0.430 0.423

4	(0.575) 0.570 0.577

Locus 10784 :

2 alleles

99.2% missing data

3	(0.498) 0.495 0.502

1	(0.502) 0.505 0.498

Locus 10785 :

2 alleles

98.4% missing data

3	(0.564) 0.573 0.566

2	(0.436) 0.427 0.434

Locus 10786 :

2 alleles

93.0% missing data

4	(0.881) 0.882 0.891

1	(0.119) 0.118 0.109

Locus 10787 :

2 alleles

99.2% missing data

4	(0.424) 0.422 0.423

3	(0.576) 0.578 0.577

Locus 10788 :

2 alleles

95.7% missing data

4	(0.904) 0.902 0.906

2	(0.096) 0.098 0.094

Locus 10789 :

2 alleles

98.8% missing data

1	(0.416) 0.417 0.411

4	(0.584) 0.583 0.589

Locus 10790 :

2 alleles

98.4% missing data

1	(0.353) 0.347 0.364

3	(0.647) 0.653 0.636

Locus 10791 :

3 alleles

95.7% missing data

3	(0.079) 0.071 0.087

4	(0.752) 0.762 0.749

2	(0.169) 0.167 0.164

Locus 10792 :

2 alleles

99.2% missing data

3	(0.541) 0.542 0.538

4	(0.459) 0.458 0.462

Locus 10793 :

2 alleles

99.2% missing data

1	(0.564) 0.568 0.566

4	(0.436) 0.432 0.434

Locus 10794 :

2 alleles

98.8% missing data

2	(0.601) 0.604 0.606

4	(0.399) 0.396 0.394

Locus 10795 :

2 alleles

99.2% missing data

1	(0.287) 0.288 0.290

4	(0.713) 0.712 0.710

Locus 10796 :

2 alleles

99.2% missing data

4	(0.460) 0.459 0.457

3	(0.540) 0.541 0.543

Locus 10797 :

2 alleles

99.2% missing data

4	(0.337) 0.337 0.340

3	(0.663) 0.663 0.660

Locus 10798 :

2 alleles

99.2% missing data

3	(0.455) 0.456 0.450

4	(0.545) 0.544 0.550

Locus 10799 :

2 alleles

99.2% missing data

3	(0.453) 0.452 0.455

2	(0.547) 0.548 0.545

Locus 10800 :

2 alleles

99.2% missing data

1	(0.487) 0.486 0.482

4	(0.513) 0.514 0.518

Locus 10801 :

2 alleles

99.2% missing data

4	(0.618) 0.614 0.613

3	(0.382) 0.386 0.387

Locus 10802 :

2 alleles

99.2% missing data

1	(0.205) 0.200 0.204

4	(0.795) 0.800 0.796

Locus 10803 :

2 alleles

99.2% missing data

3	(0.281) 0.282 0.282

2	(0.719) 0.718 0.718

Locus 10804 :

2 alleles

98.8% missing data

3	(0.274) 0.270 0.281

4	(0.726) 0.730 0.719

Locus 10805 :

2 alleles

99.2% missing data

1	(0.563) 0.562 0.564

3	(0.437) 0.438 0.436

Locus 10806 :

2 alleles

99.2% missing data

4	(0.642) 0.644 0.633

1	(0.358) 0.356 0.367

Locus 10807 :

2 alleles

8.2% missing data

3	(0.984) 0.978 0.995

1	(0.016) 0.022 0.005

Locus 10808 :

2 alleles

98.4% missing data

4	(0.500) 0.506 0.504

1	(0.500) 0.494 0.496

Locus 10809 :

2 alleles

99.2% missing data

3	(0.769) 0.768 0.768

4	(0.231) 0.232 0.232

Locus 10810 :

2 alleles

99.2% missing data

4	(0.278) 0.274 0.285

2	(0.722) 0.726 0.715

Locus 10811 :

2 alleles

96.1% missing data

4	(0.904) 0.918 0.898

1	(0.096) 0.082 0.102

Locus 10812 :

2 alleles

98.8% missing data

1	(0.674) 0.677 0.677

4	(0.326) 0.323 0.323

Locus 10813 :

2 alleles

99.2% missing data

4	(0.564) 0.565 0.563

1	(0.436) 0.435 0.437

Locus 10814 :

2 alleles

99.2% missing data

4	(0.219) 0.214 0.227

2	(0.781) 0.786 0.773

Locus 10815 :

2 alleles

91.0% missing data

4	(0.923) 0.938 0.916

1	(0.077) 0.062 0.084

Locus 10816 :

2 alleles

98.8% missing data

4	(0.554) 0.544 0.568

2	(0.446) 0.456 0.432

Locus 10817 :

2 alleles

99.2% missing data

4	(0.302) 0.300 0.305

3	(0.698) 0.700 0.695

Locus 10818 :

2 alleles

96.1% missing data

4	(0.845) 0.857 0.839

2	(0.155) 0.143 0.161

Locus 10819 :

2 alleles

99.2% missing data

4	(0.698) 0.701 0.693

1	(0.302) 0.299 0.307

Locus 10820 :

2 alleles

97.7% missing data

1	(0.338) 0.343 0.333

4	(0.662) 0.657 0.667

Locus 10821 :

2 alleles

99.2% missing data

1	(0.815) 0.819 0.804

4	(0.185) 0.181 0.196

Locus 10822 :

2 alleles

99.2% missing data

3	(0.786) 0.782 0.786

4	(0.214) 0.218 0.214

Locus 10823 :

2 alleles

99.2% missing data

3	(0.697) 0.701 0.693

2	(0.303) 0.299 0.307

Locus 10824 :

2 alleles

99.2% missing data

4	(0.318) 0.322 0.314

2	(0.682) 0.678 0.686

Locus 10825 :

2 alleles

99.2% missing data

3	(0.694) 0.701 0.695

4	(0.306) 0.299 0.305

Locus 10826 :

2 alleles

96.1% missing data

1	(0.161) 0.155 0.163

4	(0.839) 0.845 0.837

Locus 10827 :

2 alleles

99.2% missing data

4	(0.438) 0.435 0.446

1	(0.562) 0.565 0.554

Locus 10828 :

2 alleles

99.2% missing data

4	(0.246) 0.248 0.245

1	(0.754) 0.752 0.755

Locus 10829 :

2 alleles

92.6% missing data

1	(0.907) 0.899 0.920

2	(0.093) 0.101 0.080

Locus 10830 :

2 alleles

99.2% missing data

3	(0.395) 0.397 0.399

1	(0.605) 0.603 0.601

Locus 10831 :

2 alleles

99.2% missing data

1	(0.213) 0.208 0.217

3	(0.787) 0.792 0.783

Locus 10832 :

2 alleles

99.2% missing data

1	(0.525) 0.523 0.533

3	(0.475) 0.477 0.467

Locus 10833 :

2 alleles

95.3% missing data

4	(0.834) 0.833 0.844

1	(0.166) 0.167 0.156

Locus 10834 :

2 alleles

98.8% missing data

3	(0.782) 0.785 0.774

1	(0.218) 0.215 0.226

Locus 10835 :

2 alleles

0.0% missing data

3	(0.988) 0.984 0.996

2	(0.012) 0.016 0.004

Locus 10836 :

2 alleles

98.4% missing data

2	(0.228) 0.228 0.223

4	(0.772) 0.772 0.777

Locus 10837 :

2 alleles

98.8% missing data

2	(0.769) 0.770 0.764

1	(0.231) 0.230 0.236

Locus 10838 :

2 alleles

99.2% missing data

2	(0.412) 0.411 0.413

3	(0.588) 0.589 0.587

Locus 10839 :

2 alleles

99.2% missing data

3	(0.623) 0.628 0.615

2	(0.377) 0.372 0.385

Locus 10840 :

2 alleles

98.0% missing data

3	(0.603) 0.609 0.600

1	(0.397) 0.391 0.400

Locus 10841 :

2 alleles

99.2% missing data

3	(0.536) 0.542 0.536

1	(0.464) 0.458 0.464

Locus 10842 :

2 alleles

99.2% missing data

3	(0.468) 0.472 0.467

1	(0.532) 0.528 0.533

Locus 10843 :

2 alleles

97.3% missing data

3	(0.194) 0.182 0.198

1	(0.806) 0.818 0.802

Locus 10844 :

2 alleles

99.2% missing data

2	(0.574) 0.565 0.582

4	(0.426) 0.435 0.418

Locus 10845 :

2 alleles

96.5% missing data

2	(0.781) 0.794 0.781

1	(0.219) 0.206 0.219

Locus 10846 :

2 alleles

99.2% missing data

4	(0.165) 0.167 0.167

1	(0.835) 0.833 0.833

Locus 10847 :

2 alleles

99.2% missing data

4	(0.154) 0.151 0.160

1	(0.846) 0.849 0.840

Locus 10848 :

2 alleles

99.2% missing data

1	(0.554) 0.548 0.559

2	(0.446) 0.452 0.441

Locus 10849 :

2 alleles

99.2% missing data

1	(0.413) 0.411 0.417

2	(0.587) 0.589 0.583

Locus 10850 :

2 alleles

98.8% missing data

4	(0.566) 0.563 0.569

3	(0.434) 0.437 0.431

Locus 10851 :

2 alleles

99.2% missing data

4	(0.471) 0.469 0.472

2	(0.529) 0.531 0.528

Locus 10852 :

2 alleles

96.9% missing data

1	(0.780) 0.782 0.787

4	(0.220) 0.218 0.213

Locus 10853 :

2 alleles

91.0% missing data

4	(0.911) 0.930 0.905

1	(0.089) 0.070 0.095

Locus 10854 :

2 alleles

99.2% missing data

1	(0.358) 0.357 0.362

3	(0.642) 0.643 0.638

Locus 10855 :

2 alleles

99.2% missing data

1	(0.229) 0.222 0.236

2	(0.771) 0.778 0.764

Locus 10856 :

2 alleles

99.2% missing data

1	(0.580) 0.577 0.586

4	(0.420) 0.423 0.414

Locus 10857 :

2 alleles

96.1% missing data

3	(0.274) 0.256 0.281

4	(0.726) 0.744 0.719

Locus 10858 :

2 alleles

98.4% missing data

4	(0.592) 0.588 0.593

1	(0.408) 0.412 0.407

Locus 10859 :

2 alleles

99.2% missing data

1	(0.416) 0.418 0.410

2	(0.584) 0.582 0.590

Locus 10860 :

2 alleles

99.2% missing data

4	(0.472) 0.474 0.472

1	(0.528) 0.526 0.528

Locus 10861 :

2 alleles

99.2% missing data

3	(0.366) 0.366 0.370

4	(0.634) 0.634 0.630

Locus 10862 :

2 alleles

99.2% missing data

1	(0.595) 0.589 0.597

3	(0.405) 0.411 0.403

Locus 10863 :

2 alleles

99.2% missing data

2	(0.400) 0.395 0.404

4	(0.600) 0.605 0.596

Locus 10864 :

2 alleles

99.2% missing data

2	(0.193) 0.192 0.200

4	(0.807) 0.808 0.800

Locus 10865 :

2 alleles

96.5% missing data

2	(0.798) 0.794 0.801

4	(0.202) 0.206 0.199

Locus 10866 :

3 alleles

98.8% missing data

3	(0.164) 0.167 0.167

2	(0.213) 0.221 0.211

4	(0.623) 0.613 0.622

Locus 10867 :

2 alleles

99.2% missing data

2	(0.554) 0.551 0.550

1	(0.446) 0.449 0.450

Locus 10868 :

2 alleles

97.7% missing data

3	(0.248) 0.248 0.238

1	(0.752) 0.752 0.762

Locus 10869 :

2 alleles

98.4% missing data

2	(0.622) 0.629 0.619

4	(0.378) 0.371 0.381

Locus 10870 :

2 alleles

96.1% missing data

3	(0.817) 0.815 0.826

4	(0.183) 0.185 0.174

Locus 10871 :

2 alleles

90.6% missing data

2	(0.625) 0.579 0.671

4	(0.375) 0.421 0.329

Locus 10872 :

2 alleles

98.8% missing data

4	(0.600) 0.602 0.600

1	(0.400) 0.398 0.400

Locus 10873 :

2 alleles

99.2% missing data

1	(0.569) 0.564 0.572

4	(0.431) 0.436 0.428

Locus 10874 :

2 alleles

99.2% missing data

4	(0.555) 0.548 0.560

1	(0.445) 0.452 0.440

Locus 10875 :

2 alleles

99.2% missing data

1	(0.480) 0.468 0.481

2	(0.520) 0.532 0.519

Locus 10876 :

2 alleles

8.2% missing data

4	(0.986) 0.978 0.996

1	(0.014) 0.022 0.004

Locus 10877 :

2 alleles

99.2% missing data

1	(0.383) 0.380 0.390

3	(0.617) 0.620 0.610

Locus 10878 :

2 alleles

99.2% missing data

2	(0.418) 0.421 0.413

4	(0.582) 0.579 0.587

Locus 10879 :

2 alleles

91.0% missing data

1	(0.925) 0.942 0.917

3	(0.075) 0.058 0.083

Locus 10880 :

2 alleles

99.2% missing data

1	(0.252) 0.253 0.253

3	(0.748) 0.747 0.747

Locus 10881 :

2 alleles

99.2% missing data

3	(0.461) 0.460 0.456

4	(0.539) 0.540 0.544

Locus 10882 :

2 alleles

98.8% missing data

3	(0.599) 0.609 0.595

4	(0.401) 0.391 0.405

Locus 10883 :

2 alleles

99.2% missing data

4	(0.524) 0.518 0.524

3	(0.476) 0.482 0.476

Locus 10884 :

2 alleles

99.2% missing data

3	(0.301) 0.294 0.315

1	(0.699) 0.706 0.685

Locus 10885 :

2 alleles

98.8% missing data

4	(0.732) 0.724 0.741

1	(0.268) 0.276 0.259

Locus 10886 :

2 alleles

99.2% missing data

3	(0.508) 0.508 0.509

2	(0.492) 0.492 0.491

Locus 10887 :

2 alleles

99.2% missing data

2	(0.272) 0.277 0.263

1	(0.728) 0.723 0.737

Locus 10888 :

2 alleles

94.1% missing data

2	(0.892) 0.879 0.905

4	(0.108) 0.121 0.095

Locus 10889 :

2 alleles

99.2% missing data

2	(0.464) 0.460 0.467

1	(0.536) 0.540 0.533

Locus 10890 :

2 alleles

99.2% missing data

3	(0.603) 0.600 0.598

4	(0.397) 0.400 0.402

Locus 10891 :

2 alleles

91.0% missing data

1	(0.882) 0.890 0.888

4	(0.118) 0.110 0.112

Locus 10892 :

2 alleles

99.2% missing data

3	(0.389) 0.393 0.390

1	(0.611) 0.607 0.610

Locus 10893 :

2 alleles

98.0% missing data

4	(0.638) 0.646 0.635

2	(0.362) 0.354 0.365

Locus 10894 :

3 alleles

98.8% missing data

4	(0.173) 0.177 0.177

3	(0.354) 0.352 0.355

2	(0.473) 0.471 0.468

Locus 10895 :

2 alleles

99.2% missing data

1	(0.679) 0.681 0.678

2	(0.321) 0.319 0.322

Locus 10896 :

2 alleles

99.2% missing data

3	(0.188) 0.190 0.188

2	(0.812) 0.810 0.812

Locus 10897 :

2 alleles

99.2% missing data

4	(0.327) 0.325 0.330

3	(0.673) 0.675 0.670

Locus 10898 :

3 alleles

98.4% missing data

1	(0.290) 0.285 0.297

3	(0.386) 0.385 0.385

4	(0.324) 0.329 0.318

Locus 10899 :

2 alleles

99.2% missing data

1	(0.608) 0.604 0.613

2	(0.392) 0.396 0.387

Locus 10900 :

2 alleles

99.2% missing data

4	(0.860) 0.862 0.853

2	(0.140) 0.138 0.147

Locus 10901 :

2 alleles

99.2% missing data

4	(0.320) 0.314 0.329

3	(0.680) 0.686 0.671

Locus 10902 :

2 alleles

99.2% missing data

3	(0.449) 0.449 0.453

2	(0.551) 0.551 0.547

Locus 10903 :

2 alleles

98.8% missing data

3	(0.681) 0.687 0.680

1	(0.319) 0.313 0.320

Locus 10904 :

2 alleles

98.8% missing data

2	(0.389) 0.392 0.396

1	(0.611) 0.608 0.604

Locus 10905 :

2 alleles

99.2% missing data

1	(0.630) 0.628 0.630

3	(0.370) 0.372 0.370

Locus 10906 :

2 alleles

99.2% missing data

2	(0.839) 0.835 0.838

4	(0.161) 0.165 0.162

Locus 10907 :

2 alleles

95.3% missing data

1	(0.353) 0.341 0.354

4	(0.647) 0.659 0.646

Locus 10908 :

2 alleles

99.2% missing data

3	(0.384) 0.383 0.382

2	(0.616) 0.617 0.618

Locus 10909 :

2 alleles

98.8% missing data

3	(0.567) 0.565 0.560

4	(0.433) 0.435 0.440

Locus 10910 :

2 alleles

99.2% missing data

3	(0.490) 0.484 0.496

2	(0.510) 0.516 0.504

Locus 10911 :

2 alleles

98.0% missing data

4	(0.295) 0.285 0.301

2	(0.705) 0.715 0.699

Locus 10912 :

2 alleles

98.8% missing data

3	(0.284) 0.283 0.290

4	(0.716) 0.717 0.710

Locus 10913 :

2 alleles

99.2% missing data

3	(0.717) 0.715 0.711

1	(0.283) 0.285 0.289

Locus 10914 :

2 alleles

99.2% missing data

2	(0.661) 0.663 0.662

3	(0.339) 0.337 0.338

Locus 10915 :

2 alleles

90.2% missing data

4	(0.927) 0.940 0.919

1	(0.073) 0.060 0.081

Locus 10916 :

2 alleles

99.2% missing data

3	(0.328) 0.330 0.327

4	(0.672) 0.670 0.673

Locus 10917 :

2 alleles

99.2% missing data

2	(0.735) 0.737 0.734

4	(0.265) 0.263 0.266

Locus 10918 :

2 alleles

99.2% missing data

2	(0.746) 0.748 0.739

4	(0.254) 0.252 0.261

Locus 10919 :

2 alleles

99.2% missing data

3	(0.254) 0.260 0.252

2	(0.746) 0.740 0.748

Locus 10920 :

3 alleles

97.3% missing data

4	(0.699) 0.687 0.711

1	(0.176) 0.177 0.174

2	(0.125) 0.136 0.115

Locus 10921 :

2 alleles

99.2% missing data

2	(0.517) 0.521 0.517

1	(0.483) 0.479 0.483

Locus 10922 :

2 alleles

99.2% missing data

4	(0.281) 0.280 0.281

2	(0.719) 0.720 0.719

Locus 10923 :

2 alleles

90.6% missing data

2	(0.144) 0.117 0.163

4	(0.856) 0.883 0.837

Locus 10924 :

2 alleles

99.2% missing data

3	(0.548) 0.547 0.549

2	(0.452) 0.453 0.451

Locus 10925 :

2 alleles

99.2% missing data

1	(0.421) 0.418 0.420

4	(0.579) 0.582 0.580

Locus 10926 :

2 alleles

91.8% missing data

2	(0.948) 0.949 0.949

4	(0.052) 0.051 0.051

Locus 10927 :

2 alleles

99.2% missing data

4	(0.633) 0.624 0.639

2	(0.367) 0.376 0.361

Locus 10928 :

2 alleles

99.2% missing data

3	(0.457) 0.463 0.459

2	(0.543) 0.537 0.541

Locus 10929 :

2 alleles

99.2% missing data

4	(0.703) 0.703 0.699

2	(0.297) 0.297 0.301

Locus 10930 :

2 alleles

99.2% missing data

1	(0.657) 0.659 0.649

2	(0.343) 0.341 0.351

Locus 10931 :

2 alleles

92.2% missing data

3	(0.896) 0.889 0.910

1	(0.104) 0.111 0.090

Locus 10932 :

2 alleles

99.2% missing data

4	(0.434) 0.428 0.442

1	(0.566) 0.572 0.558

Locus 10933 :

2 alleles

96.9% missing data

3	(0.756) 0.756 0.768

4	(0.244) 0.244 0.232

Locus 10934 :

2 alleles

97.7% missing data

1	(0.365) 0.365 0.347

2	(0.635) 0.635 0.653

Locus 10935 :

3 alleles

98.4% missing data

1	(0.358) 0.350 0.361

2	(0.152) 0.152 0.158

3	(0.490) 0.498 0.480

Locus 10936 :

3 alleles

98.4% missing data

4	(0.384) 0.380 0.388

3	(0.222) 0.225 0.218

2	(0.394) 0.395 0.393

Locus 10937 :

2 alleles

99.2% missing data

1	(0.509) 0.510 0.510

4	(0.491) 0.490 0.490

Locus 10938 :

2 alleles

97.7% missing data

4	(0.141) 0.138 0.143

2	(0.859) 0.862 0.857

Locus 10939 :

2 alleles

99.2% missing data

2	(0.688) 0.689 0.683

4	(0.312) 0.311 0.317

Locus 10940 :

2 alleles

98.8% missing data

4	(0.742) 0.735 0.745

2	(0.258) 0.265 0.255

Locus 10941 :

2 alleles

96.1% missing data

1	(0.842) 0.855 0.839

3	(0.158) 0.145 0.161

Locus 10942 :

2 alleles

99.2% missing data

1	(0.636) 0.638 0.628

2	(0.364) 0.362 0.372

Locus 10943 :

2 alleles

99.2% missing data

4	(0.314) 0.308 0.325

3	(0.686) 0.692 0.675

Locus 10944 :

2 alleles

99.2% missing data

1	(0.540) 0.533 0.540

4	(0.460) 0.467 0.460

Locus 10945 :

2 alleles

99.2% missing data

4	(0.586) 0.586 0.583

2	(0.414) 0.414 0.417

Locus 10946 :

2 alleles

99.2% missing data

2	(0.734) 0.735 0.730

4	(0.266) 0.265 0.270

Locus 10947 :

2 alleles

99.2% missing data

4	(0.736) 0.735 0.742

2	(0.264) 0.265 0.258

Locus 10948 :

2 alleles

93.0% missing data

4	(0.893) 0.893 0.903

3	(0.107) 0.107 0.097

Locus 10949 :

2 alleles

99.2% missing data

3	(0.595) 0.591 0.597

4	(0.405) 0.409 0.403

Locus 10950 :

2 alleles

97.3% missing data

4	(0.802) 0.793 0.817

2	(0.198) 0.207 0.183

Locus 10951 :

2 alleles

96.9% missing data

4	(0.815) 0.814 0.817

1	(0.185) 0.186 0.183

Locus 10952 :

2 alleles

95.7% missing data

4	(0.457) 0.477 0.444

2	(0.543) 0.523 0.556

Locus 10953 :

2 alleles

99.2% missing data

4	(0.669) 0.664 0.676

3	(0.331) 0.336 0.324

Locus 10954 :

2 alleles

99.2% missing data

1	(0.382) 0.383 0.383

4	(0.618) 0.617 0.617

Locus 10955 :

2 alleles

98.8% missing data

4	(0.499) 0.503 0.505

2	(0.501) 0.497 0.495

Locus 10956 :

2 alleles

98.4% missing data

2	(0.674) 0.682 0.665

1	(0.326) 0.318 0.335

Locus 10957 :

2 alleles

98.4% missing data

2	(0.659) 0.666 0.657

3	(0.341) 0.334 0.343

Locus 10958 :

2 alleles

90.6% missing data

4	(0.933) 0.946 0.922

1	(0.067) 0.054 0.078

Locus 10959 :

2 alleles

99.2% missing data

4	(0.317) 0.316 0.322

2	(0.683) 0.684 0.678

Locus 10960 :

2 alleles

99.2% missing data

2	(0.324) 0.325 0.327

3	(0.676) 0.675 0.673

Locus 10961 :

2 alleles

98.8% missing data

1	(0.672) 0.671 0.678

3	(0.328) 0.329 0.322

Locus 10962 :

2 alleles

98.8% missing data

2	(0.697) 0.694 0.699

3	(0.303) 0.306 0.301

Locus 10963 :

2 alleles

98.8% missing data

2	(0.782) 0.776 0.786

4	(0.218) 0.224 0.214

Locus 10964 :

2 alleles

98.4% missing data

4	(0.662) 0.666 0.659

1	(0.338) 0.334 0.341

Locus 10965 :

2 alleles

99.2% missing data

4	(0.305) 0.301 0.312

3	(0.695) 0.699 0.688

Locus 10966 :

2 alleles

99.2% missing data

2	(0.628) 0.629 0.631

1	(0.372) 0.371 0.369

Locus 10967 :

2 alleles

98.8% missing data

2	(0.525) 0.520 0.529

4	(0.475) 0.480 0.471

Locus 10968 :

2 alleles

98.4% missing data

3	(0.534) 0.517 0.545

4	(0.466) 0.483 0.455

Locus 10969 :

2 alleles

98.4% missing data

1	(0.632) 0.618 0.643

2	(0.368) 0.382 0.357

Locus 10970 :

2 alleles

99.2% missing data

1	(0.775) 0.778 0.763

4	(0.225) 0.222 0.237

Locus 10971 :

2 alleles

99.2% missing data

3	(0.647) 0.648 0.646

4	(0.353) 0.352 0.354

Locus 10972 :

2 alleles

99.2% missing data

3	(0.262) 0.261 0.270

1	(0.738) 0.739 0.730

Locus 10973 :

2 alleles

98.4% missing data

4	(0.470) 0.468 0.482

3	(0.530) 0.532 0.518

Locus 10974 :

2 alleles

98.0% missing data

4	(0.209) 0.210 0.203

3	(0.791) 0.790 0.797

Locus 10975 :

2 alleles

93.4% missing data

2	(0.850) 0.845 0.867

3	(0.150) 0.155 0.133

Locus 10976 :

2 alleles

97.7% missing data

2	(0.605) 0.600 0.616

4	(0.395) 0.400 0.384

Locus 10977 :

2 alleles

99.2% missing data

4	(0.297) 0.289 0.303

2	(0.703) 0.711 0.697

Locus 10978 :

2 alleles

98.8% missing data

3	(0.768) 0.769 0.764

2	(0.232) 0.231 0.236

Locus 10979 :

2 alleles

99.2% missing data

1	(0.420) 0.413 0.426

2	(0.580) 0.587 0.574

Locus 10980 :

2 alleles

98.4% missing data

3	(0.748) 0.753 0.748

1	(0.252) 0.247 0.252

Locus 10981 :

2 alleles

98.8% missing data

3	(0.488) 0.491 0.495

4	(0.512) 0.509 0.505

Locus 10982 :

2 alleles

93.8% missing data

4	(0.828) 0.827 0.840

2	(0.172) 0.173 0.160

Locus 10983 :

2 alleles

98.0% missing data

4	(0.765) 0.773 0.760

3	(0.235) 0.227 0.240

Locus 10984 :

2 alleles

98.0% missing data

4	(0.810) 0.813 0.808

2	(0.190) 0.187 0.192

Locus 10985 :

2 alleles

99.2% missing data

2	(0.378) 0.386 0.376

3	(0.622) 0.614 0.624

Locus 10986 :

2 alleles

97.3% missing data

3	(0.819) 0.822 0.820

1	(0.181) 0.178 0.180

Locus 10987 :

2 alleles

99.2% missing data

3	(0.390) 0.384 0.385

4	(0.610) 0.616 0.615

Locus 10988 :

2 alleles

90.2% missing data

3	(0.878) 0.901 0.867

1	(0.122) 0.099 0.133

Locus 10989 :

2 alleles

99.2% missing data

3	(0.734) 0.732 0.730

1	(0.266) 0.268 0.270

Locus 10990 :

2 alleles

99.2% missing data

1	(0.573) 0.571 0.571

3	(0.427) 0.429 0.429

Locus 10991 :

2 alleles

99.2% missing data

1	(0.508) 0.505 0.510

3	(0.492) 0.495 0.490

Locus 10992 :

2 alleles

99.2% missing data

2	(0.448) 0.449 0.439

1	(0.552) 0.551 0.561

Locus 10993 :

2 alleles

97.3% missing data

4	(0.775) 0.785 0.771

1	(0.225) 0.215 0.229

Locus 10994 :

2 alleles

29.3% missing data

1	(0.969) 0.967 0.982

3	(0.031) 0.033 0.018

Locus 10995 :

2 alleles

97.7% missing data

4	(0.903) 0.905 0.899

1	(0.097) 0.095 0.101

Locus 10996 :

2 alleles

91.0% missing data

1	(0.889) 0.905 0.884

4	(0.111) 0.095 0.116

Locus 10997 :

2 alleles

99.2% missing data

3	(0.825) 0.819 0.831

4	(0.175) 0.181 0.169

Locus 10998 :

2 alleles

99.2% missing data

2	(0.820) 0.819 0.820

1	(0.180) 0.181 0.180

Locus 10999 :

2 alleles

99.2% missing data

1	(0.783) 0.769 0.788

2	(0.217) 0.231 0.212

Locus 11000 :

2 alleles

99.2% missing data

4	(0.159) 0.160 0.161

1	(0.841) 0.840 0.839

Locus 11001 :

3 alleles

98.0% missing data

3	(0.719) 0.710 0.729

1	(0.151) 0.158 0.147

2	(0.129) 0.132 0.124

Locus 11002 :

3 alleles

86.3% missing data

2	(0.594) 0.659 0.541

1	(0.349) 0.285 0.408

3	(0.057) 0.056 0.051

Locus 11003 :

2 alleles

98.0% missing data

3	(0.491) 0.494 0.501

1	(0.509) 0.506 0.499

Locus 11004 :

2 alleles

98.4% missing data

3	(0.228) 0.224 0.233

1	(0.772) 0.776 0.767

Locus 11005 :

3 alleles

98.8% missing data

2	(0.226) 0.223 0.234

3	(0.231) 0.231 0.225

1	(0.543) 0.546 0.541

Locus 11006 :

2 alleles

16.8% missing data

4	(0.982) 0.974 0.994

3	(0.018) 0.026 0.006

Locus 11007 :

2 alleles

98.8% missing data

1	(0.410) 0.408 0.420

4	(0.590) 0.592 0.580

Locus 11008 :

2 alleles

99.2% missing data

3	(0.442) 0.448 0.440

1	(0.558) 0.552 0.560

Locus 11009 :

2 alleles

98.8% missing data

4	(0.438) 0.437 0.443

1	(0.562) 0.563 0.557

Locus 11010 :

3 alleles

98.8% missing data

2	(0.282) 0.277 0.285

1	(0.279) 0.278 0.287

4	(0.439) 0.445 0.428

Locus 11011 :

2 alleles

98.8% missing data

3	(0.604) 0.600 0.604

2	(0.396) 0.400 0.396

Locus 11012 :

2 alleles

98.8% missing data

3	(0.728) 0.731 0.726

1	(0.272) 0.269 0.274

Locus 11013 :

2 alleles

98.0% missing data

2	(0.670) 0.670 0.677

1	(0.330) 0.330 0.323

Locus 11014 :

2 alleles

99.2% missing data

2	(0.237) 0.236 0.242

1	(0.763) 0.764 0.758

Locus 11015 :

2 alleles

99.2% missing data

3	(0.526) 0.523 0.532

1	(0.474) 0.477 0.468

Locus 11016 :

2 alleles

16.8% missing data

4	(0.980) 0.973 0.993

1	(0.020) 0.027 0.007

Locus 11017 :

2 alleles

96.1% missing data

1	(0.756) 0.767 0.758

4	(0.244) 0.233 0.242

Locus 11018 :

2 alleles

99.2% missing data

2	(0.651) 0.645 0.650

4	(0.349) 0.355 0.350

Locus 11019 :

2 alleles

99.2% missing data

3	(0.349) 0.347 0.354

1	(0.651) 0.653 0.646

Locus 11020 :

2 alleles

92.6% missing data

1	(0.889) 0.890 0.901

2	(0.111) 0.110 0.099

Locus 11021 :

2 alleles

96.1% missing data

1	(0.296) 0.284 0.295

3	(0.704) 0.716 0.705

Locus 11022 :

2 alleles

99.2% missing data

4	(0.228) 0.225 0.229

1	(0.772) 0.775 0.771

Locus 11023 :

2 alleles

99.2% missing data

1	(0.463) 0.465 0.464

4	(0.537) 0.535 0.536

Locus 11024 :

2 alleles

91.0% missing data

2	(0.927) 0.942 0.919

3	(0.073) 0.058 0.081

Locus 11025 :

2 alleles

96.1% missing data

2	(0.744) 0.758 0.742

3	(0.256) 0.242 0.258

Locus 11026 :

2 alleles

99.2% missing data

4	(0.689) 0.684 0.698

1	(0.311) 0.316 0.302

Locus 11027 :

2 alleles

91.4% missing data

4	(0.917) 0.925 0.918

2	(0.083) 0.075 0.082

Locus 11028 :

2 alleles

91.0% missing data

3	(0.902) 0.922 0.892

4	(0.098) 0.078 0.108

Locus 11029 :

2 alleles

90.6% missing data

3	(0.864) 0.871 0.869

2	(0.136) 0.129 0.131

Locus 11030 :

2 alleles

99.2% missing data

4	(0.854) 0.847 0.861

1	(0.146) 0.153 0.139

Locus 11031 :

2 alleles

99.2% missing data

4	(0.299) 0.297 0.302

1	(0.701) 0.703 0.698

Locus 11032 :

2 alleles

99.2% missing data

3	(0.449) 0.453 0.444

1	(0.551) 0.547 0.556

Locus 11033 :

2 alleles

97.7% missing data

3	(0.654) 0.657 0.661

1	(0.346) 0.343 0.339

Locus 11034 :

2 alleles

98.8% missing data

4	(0.769) 0.771 0.766

2	(0.231) 0.229 0.234

Locus 11035 :

2 alleles

92.6% missing data

3	(0.882) 0.882 0.892

2	(0.118) 0.118 0.108

Locus 11036 :

2 alleles

99.2% missing data

1	(0.543) 0.544 0.548

2	(0.457) 0.456 0.452

Locus 11037 :

2 alleles

9.0% missing data

4	(0.968) 0.935 0.990

2	(0.032) 0.065 0.010

Locus 11038 :

3 alleles

88.3% missing data

3	(0.585) 0.626 0.560

4	(0.332) 0.306 0.348

2	(0.083) 0.068 0.092

Locus 11039 :

2 alleles

96.9% missing data

2	(0.243) 0.241 0.232

1	(0.757) 0.759 0.768

Locus 11040 :

2 alleles

96.1% missing data

1	(0.898) 0.907 0.891

2	(0.102) 0.093 0.109

Locus 11041 :

2 alleles

98.8% missing data

4	(0.483) 0.482 0.475

1	(0.517) 0.518 0.525

Locus 11042 :

2 alleles

96.1% missing data

1	(0.473) 0.499 0.455

2	(0.527) 0.501 0.545

Locus 11043 :

2 alleles

99.2% missing data

3	(0.496) 0.497 0.493

2	(0.504) 0.503 0.507

Locus 11044 :

2 alleles

96.1% missing data

3	(0.737) 0.757 0.735

4	(0.263) 0.243 0.265

Locus 11045 :

2 alleles

99.2% missing data

1	(0.810) 0.811 0.803

4	(0.190) 0.189 0.197

Locus 11046 :

2 alleles

97.7% missing data

1	(0.392) 0.395 0.379

2	(0.608) 0.605 0.621

Locus 11047 :

2 alleles

99.2% missing data

4	(0.434) 0.429 0.437

1	(0.566) 0.571 0.563

Locus 11048 :

2 alleles

99.2% missing data

3	(0.614) 0.616 0.609

4	(0.386) 0.384 0.391

Locus 11049 :

2 alleles

99.2% missing data

3	(0.592) 0.593 0.588

1	(0.408) 0.407 0.412

Locus 11050 :

2 alleles

99.2% missing data

1	(0.599) 0.593 0.596

3	(0.401) 0.407 0.404

Locus 11051 :

3 alleles

91.0% missing data

3	(0.689) 0.689 0.698

1	(0.186) 0.185 0.190

2	(0.125) 0.126 0.112

Locus 11052 :

2 alleles

98.8% missing data

2	(0.679) 0.674 0.677

3	(0.321) 0.326 0.323

Locus 11053 :

2 alleles

98.8% missing data

3	(0.734) 0.729 0.737

4	(0.266) 0.271 0.263

Locus 11054 :

2 alleles

99.2% missing data

1	(0.368) 0.371 0.370

3	(0.632) 0.629 0.630

Locus 11055 :

2 alleles

99.2% missing data

4	(0.545) 0.535 0.553

3	(0.455) 0.465 0.447

Locus 11056 :

2 alleles

92.6% missing data

4	(0.879) 0.869 0.898

1	(0.121) 0.131 0.102

Locus 11057 :

2 alleles

99.2% missing data

1	(0.453) 0.451 0.460

3	(0.547) 0.549 0.540

Locus 11058 :

2 alleles

99.2% missing data

2	(0.322) 0.325 0.332

3	(0.678) 0.675 0.668

Locus 11059 :

2 alleles

98.8% missing data

1	(0.679) 0.675 0.688

2	(0.321) 0.325 0.312

Locus 11060 :

2 alleles

99.2% missing data

3	(0.486) 0.483 0.492

1	(0.514) 0.517 0.508

Locus 11061 :

2 alleles

99.2% missing data

2	(0.400) 0.396 0.401

3	(0.600) 0.604 0.599

Locus 11062 :

2 alleles

98.8% missing data

1	(0.376) 0.370 0.382

2	(0.624) 0.630 0.618

Locus 11063 :

2 alleles

99.2% missing data

4	(0.514) 0.510 0.526

2	(0.486) 0.490 0.474

Locus 11064 :

2 alleles

99.2% missing data

1	(0.223) 0.218 0.229

3	(0.777) 0.782 0.771

Locus 11065 :

2 alleles

98.0% missing data

2	(0.714) 0.720 0.707

4	(0.286) 0.280 0.293

Locus 11066 :

2 alleles

98.0% missing data

2	(0.276) 0.287 0.268

3	(0.724) 0.713 0.732

Locus 11067 :

2 alleles

99.2% missing data

4	(0.467) 0.466 0.466

3	(0.533) 0.534 0.534

Locus 11068 :

3 alleles

97.7% missing data

4	(0.490) 0.504 0.488

2	(0.171) 0.164 0.176

3	(0.339) 0.332 0.336

Locus 11069 :

2 alleles

98.8% missing data

1	(0.607) 0.606 0.604

3	(0.393) 0.394 0.396

Locus 11070 :

2 alleles

98.8% missing data

4	(0.540) 0.537 0.551

2	(0.460) 0.463 0.449

Locus 11071 :

2 alleles

95.7% missing data

1	(0.790) 0.797 0.785

4	(0.210) 0.203 0.215

Locus 11072 :

2 alleles

99.2% missing data

2	(0.262) 0.260 0.267

4	(0.738) 0.740 0.733

Locus 11073 :

2 alleles

99.2% missing data

3	(0.565) 0.564 0.573

4	(0.435) 0.436 0.427

Locus 11074 :

2 alleles

98.4% missing data

4	(0.604) 0.593 0.616

1	(0.396) 0.407 0.384

Locus 11075 :

2 alleles

93.8% missing data

2	(0.879) 0.880 0.887

4	(0.121) 0.120 0.113

Locus 11076 :

2 alleles

93.0% missing data

4	(0.918) 0.930 0.913

2	(0.082) 0.070 0.087

Locus 11077 :

2 alleles

99.2% missing data

2	(0.554) 0.556 0.552

1	(0.446) 0.444 0.448

Locus 11078 :

2 alleles

99.2% missing data

4	(0.478) 0.477 0.479

2	(0.522) 0.523 0.521

Locus 11079 :

2 alleles

99.2% missing data

2	(0.472) 0.480 0.472

4	(0.528) 0.520 0.528

Locus 11080 :

2 alleles

0.4% missing data

2	(0.987) 0.983 0.996

1	(0.013) 0.017 0.004

Locus 11081 :

2 alleles

99.2% missing data

1	(0.222) 0.219 0.227

2	(0.778) 0.781 0.773

Locus 11082 :

2 alleles

99.2% missing data

2	(0.656) 0.657 0.653

3	(0.344) 0.343 0.347

Locus 11083 :

2 alleles

96.5% missing data

1	(0.892) 0.892 0.894

2	(0.108) 0.108 0.106

Locus 11084 :

2 alleles

98.8% missing data

4	(0.531) 0.527 0.538

1	(0.469) 0.473 0.462

Locus 11085 :

2 alleles

99.2% missing data

1	(0.726) 0.726 0.724

2	(0.274) 0.274 0.276

Locus 11086 :

2 alleles

99.2% missing data

4	(0.583) 0.589 0.579

2	(0.417) 0.411 0.421

Locus 11087 :

2 alleles

98.0% missing data

1	(0.704) 0.703 0.716

4	(0.296) 0.297 0.284

Locus 11088 :

2 alleles

99.2% missing data

2	(0.361) 0.358 0.360

3	(0.639) 0.642 0.640

Locus 11089 :

3 alleles

98.8% missing data

2	(0.193) 0.188 0.205

4	(0.303) 0.302 0.295

1	(0.504) 0.510 0.500

Locus 11090 :

2 alleles

93.4% missing data

4	(0.857) 0.852 0.871

3	(0.143) 0.148 0.129

Locus 11091 :

3 alleles

98.4% missing data

4	(0.266) 0.254 0.277

3	(0.449) 0.454 0.444

2	(0.286) 0.292 0.279

Locus 11092 :

2 alleles

99.2% missing data

1	(0.547) 0.544 0.546

3	(0.453) 0.456 0.454

Locus 11093 :

2 alleles

99.2% missing data

1	(0.516) 0.521 0.511

4	(0.484) 0.479 0.489

Locus 11094 :

2 alleles

98.8% missing data

2	(0.708) 0.709 0.711

4	(0.292) 0.291 0.289

Locus 11095 :

2 alleles

99.2% missing data

1	(0.698) 0.701 0.691

4	(0.302) 0.299 0.309

Locus 11096 :

2 alleles

98.8% missing data

4	(0.577) 0.569 0.585

1	(0.423) 0.431 0.415

Locus 11097 :

2 alleles

98.4% missing data

1	(0.463) 0.461 0.457

4	(0.537) 0.539 0.543

Locus 11098 :

2 alleles

99.2% missing data

4	(0.552) 0.550 0.558

1	(0.448) 0.450 0.442

Locus 11099 :

2 alleles

99.2% missing data

2	(0.430) 0.429 0.431

3	(0.570) 0.571 0.569

Locus 11100 :

3 alleles

98.4% missing data

4	(0.443) 0.449 0.444

2	(0.200) 0.201 0.202

1	(0.357) 0.350 0.355

Locus 11101 :

2 alleles

98.0% missing data

3	(0.337) 0.328 0.344

4	(0.663) 0.672 0.656

Locus 11102 :

2 alleles

99.2% missing data

1	(0.496) 0.488 0.497

2	(0.504) 0.512 0.503

Locus 11103 :

3 alleles

98.8% missing data

4	(0.269) 0.273 0.269

1	(0.404) 0.400 0.402

3	(0.327) 0.327 0.329

Locus 11104 :

2 alleles

99.2% missing data

1	(0.714) 0.714 0.711

2	(0.286) 0.286 0.289

Locus 11105 :

2 alleles

7.0% missing data

1	(0.989) 0.992 0.994

3	(0.011) 0.008 0.006

Locus 11106 :

2 alleles

98.8% missing data

3	(0.817) 0.818 0.813

1	(0.183) 0.182 0.187

Locus 11107 :

2 alleles

98.8% missing data

1	(0.323) 0.326 0.323

3	(0.677) 0.674 0.677

Locus 11108 :

2 alleles

98.8% missing data

1	(0.683) 0.678 0.687

2	(0.317) 0.322 0.313

Locus 11109 :

2 alleles

98.8% missing data

1	(0.685) 0.680 0.687

4	(0.315) 0.320 0.313

Locus 11110 :

2 alleles

96.5% missing data

3	(0.845) 0.852 0.841

4	(0.155) 0.148 0.159

Locus 11111 :

2 alleles

99.2% missing data

1	(0.482) 0.485 0.481

2	(0.518) 0.515 0.519

Locus 11112 :

2 alleles

98.4% missing data

1	(0.447) 0.452 0.445

3	(0.553) 0.548 0.555

Locus 11113 :

3 alleles

94.9% missing data

3	(0.567) 0.572 0.567

1	(0.080) 0.081 0.079

4	(0.353) 0.348 0.355

Locus 11114 :

2 alleles

90.2% missing data

1	(0.902) 0.905 0.907

3	(0.098) 0.095 0.093

Locus 11115 :

2 alleles

99.2% missing data

1	(0.769) 0.777 0.763

4	(0.231) 0.223 0.237

Locus 11116 :

2 alleles

99.2% missing data

3	(0.306) 0.304 0.309

4	(0.694) 0.696 0.691

Locus 11117 :

2 alleles

98.8% missing data

2	(0.355) 0.355 0.353

1	(0.645) 0.645 0.647

Locus 11118 :

2 alleles

94.9% missing data

4	(0.473) 0.467 0.475

1	(0.527) 0.533 0.525

Locus 11119 :

2 alleles

99.2% missing data

4	(0.363) 0.361 0.367

3	(0.637) 0.639 0.633

Locus 11120 :

3 alleles

98.8% missing data

1	(0.290) 0.290 0.284

4	(0.247) 0.245 0.252

2	(0.463) 0.465 0.464

Locus 11121 :

2 alleles

98.4% missing data

1	(0.765) 0.766 0.770

4	(0.235) 0.234 0.230

Locus 11122 :

2 alleles

98.0% missing data

4	(0.360) 0.352 0.361

3	(0.640) 0.648 0.639

Locus 11123 :

2 alleles

98.4% missing data

4	(0.668) 0.682 0.661

1	(0.332) 0.318 0.339

Locus 11124 :

2 alleles

99.2% missing data

4	(0.337) 0.332 0.339

1	(0.663) 0.668 0.661

Locus 11125 :

2 alleles

94.5% missing data

4	(0.726) 0.748 0.699

1	(0.274) 0.252 0.301

Locus 11126 :

2 alleles

99.2% missing data

4	(0.738) 0.735 0.739

2	(0.262) 0.265 0.261

Locus 11127 :

2 alleles

99.2% missing data

2	(0.521) 0.526 0.515

4	(0.479) 0.474 0.485

Locus 11128 :

2 alleles

99.2% missing data

2	(0.716) 0.710 0.721

1	(0.284) 0.290 0.279

Locus 11129 :

2 alleles

99.2% missing data

3	(0.672) 0.675 0.672

4	(0.328) 0.325 0.328

Locus 11130 :

2 alleles

93.8% missing data

3	(0.873) 0.871 0.881

2	(0.127) 0.129 0.119

Locus 11131 :

2 alleles

96.1% missing data

4	(0.374) 0.372 0.381

1	(0.626) 0.628 0.619

Locus 11132 :

2 alleles

99.2% missing data

2	(0.708) 0.711 0.704

1	(0.292) 0.289 0.296

Locus 11133 :

2 alleles

98.8% missing data

2	(0.588) 0.590 0.591

1	(0.412) 0.410 0.409

Locus 11134 :

2 alleles

99.2% missing data

4	(0.700) 0.703 0.697

1	(0.300) 0.297 0.303

Locus 11135 :

2 alleles

98.8% missing data

4	(0.725) 0.724 0.726

2	(0.275) 0.276 0.274

Locus 11136 :

2 alleles

96.1% missing data

1	(0.759) 0.763 0.762

2	(0.241) 0.237 0.238

Locus 11137 :

2 alleles

99.2% missing data

2	(0.696) 0.702 0.691

3	(0.304) 0.298 0.309

Locus 11138 :

2 alleles

99.2% missing data

4	(0.529) 0.531 0.529

2	(0.471) 0.469 0.471

Locus 11139 :

2 alleles

99.2% missing data

1	(0.287) 0.290 0.294

3	(0.713) 0.710 0.706

Locus 11140 :

2 alleles

98.8% missing data

1	(0.552) 0.555 0.552

3	(0.448) 0.445 0.448

Locus 11141 :

3 alleles

97.7% missing data

1	(0.434) 0.435 0.430

4	(0.421) 0.418 0.424

2	(0.146) 0.147 0.146

Locus 11142 :

2 alleles

97.7% missing data

1	(0.705) 0.703 0.718

3	(0.295) 0.297 0.282

Locus 11143 :

2 alleles

99.2% missing data

2	(0.367) 0.366 0.370

3	(0.633) 0.634 0.630

Locus 11144 :

2 alleles

96.9% missing data

3	(0.803) 0.815 0.797

2	(0.197) 0.185 0.203

Locus 11145 :

2 alleles

96.1% missing data

1	(0.893) 0.904 0.886

3	(0.107) 0.096 0.114

Locus 11146 :

2 alleles

99.2% missing data

4	(0.441) 0.440 0.442

1	(0.559) 0.560 0.558

Locus 11147 :

2 alleles

98.8% missing data

4	(0.351) 0.362 0.345

2	(0.649) 0.638 0.655

Locus 11148 :

2 alleles

98.4% missing data

4	(0.727) 0.740 0.715

1	(0.273) 0.260 0.285

Locus 11149 :

2 alleles

99.2% missing data

1	(0.693) 0.689 0.692

3	(0.307) 0.311 0.308

Locus 11150 :

2 alleles

99.2% missing data

1	(0.598) 0.598 0.600

2	(0.402) 0.402 0.400

Locus 11151 :

2 alleles

99.2% missing data

4	(0.413) 0.411 0.406

3	(0.587) 0.589 0.594

Locus 11152 :

2 alleles

98.4% missing data

3	(0.815) 0.814 0.818

2	(0.185) 0.186 0.182

Locus 11153 :

2 alleles

99.2% missing data

1	(0.696) 0.688 0.701

4	(0.304) 0.312 0.299

Locus 11154 :

2 alleles

99.2% missing data

1	(0.420) 0.422 0.422

4	(0.580) 0.578 0.578

Locus 11155 :

2 alleles

98.8% missing data

2	(0.615) 0.615 0.620

4	(0.385) 0.385 0.380

Locus 11156 :

2 alleles

99.2% missing data

4	(0.476) 0.470 0.482

3	(0.524) 0.530 0.518

Locus 11157 :

2 alleles

95.3% missing data

2	(0.769) 0.780 0.760

3	(0.231) 0.220 0.240

Locus 11158 :

2 alleles

99.2% missing data

1	(0.311) 0.305 0.315

4	(0.689) 0.695 0.685

Locus 11159 :

2 alleles

98.0% missing data

2	(0.722) 0.724 0.723

4	(0.278) 0.276 0.277

Locus 11160 :

2 alleles

99.2% missing data

1	(0.650) 0.654 0.642

3	(0.350) 0.346 0.358

Locus 11161 :

2 alleles

99.2% missing data

4	(0.132) 0.140 0.128

1	(0.868) 0.860 0.872

Locus 11162 :

2 alleles

98.4% missing data

3	(0.616) 0.622 0.612

1	(0.384) 0.378 0.388

Locus 11163 :

2 alleles

96.1% missing data

3	(0.065) 0.066 0.067

1	(0.935) 0.934 0.933

Locus 11164 :

2 alleles

97.7% missing data

4	(0.280) 0.273 0.283

1	(0.720) 0.727 0.717

Locus 11165 :

2 alleles

99.2% missing data

2	(0.470) 0.466 0.470

3	(0.530) 0.534 0.530

Locus 11166 :

2 alleles

99.2% missing data

2	(0.223) 0.222 0.226

3	(0.777) 0.778 0.774

Locus 11167 :

2 alleles

99.2% missing data

2	(0.333) 0.336 0.333

4	(0.667) 0.664 0.667

Locus 11168 :

2 alleles

99.2% missing data

4	(0.362) 0.355 0.367

3	(0.638) 0.645 0.633

Locus 11169 :

2 alleles

98.8% missing data

2	(0.251) 0.244 0.266

4	(0.749) 0.756 0.734

Locus 11170 :

2 alleles

99.2% missing data

4	(0.313) 0.316 0.321

3	(0.687) 0.684 0.679

Locus 11171 :

2 alleles

98.4% missing data

3	(0.853) 0.845 0.858

1	(0.147) 0.155 0.142

Locus 11172 :

2 alleles

89.8% missing data

4	(0.943) 0.956 0.937

1	(0.057) 0.044 0.063

Locus 11173 :

2 alleles

16.8% missing data

4	(0.978) 0.968 0.992

2	(0.022) 0.032 0.008

Locus 11174 :

2 alleles

0.0% missing data

4	(0.913) 0.760 0.974

3	(0.087) 0.240 0.026

Locus 11175 :

2 alleles

96.1% missing data

3	(0.739) 0.757 0.733

4	(0.261) 0.243 0.267

Locus 11176 :

2 alleles

99.2% missing data

3	(0.574) 0.578 0.572

2	(0.426) 0.422 0.428

Locus 11177 :

2 alleles

97.7% missing data

4	(0.656) 0.669 0.647

3	(0.344) 0.331 0.353

Locus 11178 :

2 alleles

97.3% missing data

3	(0.724) 0.716 0.739

4	(0.276) 0.284 0.261

Locus 11179 :

2 alleles

97.3% missing data

4	(0.299) 0.300 0.295

1	(0.701) 0.700 0.705

Locus 11180 :

2 alleles

99.2% missing data

4	(0.565) 0.563 0.571

2	(0.435) 0.437 0.429

Locus 11181 :

3 alleles

95.7% missing data

2	(0.133) 0.125 0.136

1	(0.743) 0.760 0.734

4	(0.123) 0.115 0.130

Locus 11182 :

3 alleles

91.4% missing data

2	(0.654) 0.651 0.672

3	(0.117) 0.117 0.106

4	(0.229) 0.232 0.222

Locus 11183 :

2 alleles

99.2% missing data

2	(0.675) 0.670 0.679

4	(0.325) 0.330 0.321

Locus 11184 :

3 alleles

90.6% missing data

2	(0.237) 0.219 0.256

1	(0.449) 0.484 0.422

3	(0.314) 0.297 0.322

Locus 11185 :

2 alleles

99.2% missing data

1	(0.362) 0.355 0.367

4	(0.638) 0.645 0.633

Locus 11186 :

2 alleles

99.2% missing data

3	(0.657) 0.657 0.665

2	(0.343) 0.343 0.335

Locus 11187 :

2 alleles

94.1% missing data

1	(0.828) 0.825 0.843

2	(0.172) 0.175 0.157

Locus 11188 :

3 alleles

98.4% missing data

1	(0.465) 0.467 0.462

3	(0.281) 0.280 0.281

4	(0.254) 0.253 0.257

Locus 11189 :

2 alleles

99.2% missing data

4	(0.278) 0.276 0.280

3	(0.722) 0.724 0.720

Locus 11190 :

2 alleles

99.2% missing data

1	(0.335) 0.332 0.337

3	(0.665) 0.668 0.663

Locus 11191 :

2 alleles

99.2% missing data

2	(0.598) 0.602 0.594

3	(0.402) 0.398 0.406

Locus 11192 :

3 alleles

98.8% missing data

1	(0.218) 0.211 0.224

4	(0.349) 0.354 0.344

3	(0.433) 0.435 0.432

Locus 11193 :

2 alleles

99.2% missing data

2	(0.292) 0.287 0.299

1	(0.708) 0.713 0.701

Locus 11194 :

2 alleles

97.7% missing data

4	(0.334) 0.345 0.320

2	(0.666) 0.655 0.680

Locus 11195 :

2 alleles

16.4% missing data

1	(0.976) 0.958 0.993

2	(0.024) 0.042 0.007

Locus 11196 :

2 alleles

99.2% missing data

2	(0.359) 0.352 0.368

3	(0.641) 0.648 0.632

Locus 11197 :

2 alleles

99.2% missing data

2	(0.656) 0.652 0.657

3	(0.344) 0.348 0.343

Locus 11198 :

3 alleles

98.4% missing data

2	(0.405) 0.410 0.404

3	(0.315) 0.318 0.312

1	(0.279) 0.272 0.284

Locus 11199 :

2 alleles

96.9% missing data

2	(0.820) 0.822 0.827

1	(0.180) 0.178 0.173

Locus 11200 :

2 alleles

98.0% missing data

3	(0.503) 0.491 0.512

4	(0.497) 0.509 0.488

Locus 11201 :

2 alleles

99.2% missing data

4	(0.267) 0.265 0.273

2	(0.733) 0.735 0.727

Locus 11202 :

2 alleles

99.2% missing data

1	(0.598) 0.592 0.601

4	(0.402) 0.408 0.399

Locus 11203 :

2 alleles

99.2% missing data

3	(0.493) 0.493 0.498

2	(0.507) 0.507 0.502

Locus 11204 :

2 alleles

96.1% missing data

1	(0.842) 0.844 0.847

3	(0.158) 0.156 0.153

Locus 11205 :

2 alleles

99.2% missing data

1	(0.682) 0.689 0.683

3	(0.318) 0.311 0.317

Locus 11206 :

2 alleles

97.3% missing data

2	(0.817) 0.826 0.811

4	(0.183) 0.174 0.189

Locus 11207 :

2 alleles

96.5% missing data

4	(0.194) 0.178 0.198

1	(0.806) 0.822 0.802

Locus 11208 :

2 alleles

99.2% missing data

1	(0.721) 0.724 0.716

2	(0.279) 0.276 0.284

Locus 11209 :

2 alleles

99.2% missing data

1	(0.488) 0.487 0.488

4	(0.512) 0.513 0.512

Locus 11210 :

2 alleles

97.7% missing data

2	(0.612) 0.624 0.608

1	(0.388) 0.376 0.392

Locus 11211 :

2 alleles

97.3% missing data

3	(0.452) 0.456 0.445

2	(0.548) 0.544 0.555

Locus 11212 :

2 alleles

99.2% missing data

1	(0.715) 0.711 0.720

3	(0.285) 0.289 0.280

Locus 11213 :

2 alleles

98.8% missing data

1	(0.564) 0.562 0.567

2	(0.436) 0.438 0.433

Locus 11214 :

2 alleles

99.2% missing data

2	(0.751) 0.743 0.757

3	(0.249) 0.257 0.243

Locus 11215 :

2 alleles

98.8% missing data

3	(0.525) 0.517 0.533

4	(0.475) 0.483 0.467

Locus 11216 :

2 alleles

94.9% missing data

4	(0.805) 0.806 0.815

2	(0.195) 0.194 0.185

Locus 11217 :

2 alleles

98.8% missing data

4	(0.437) 0.449 0.437

2	(0.563) 0.551 0.563

Locus 11218 :

2 alleles

99.2% missing data

1	(0.782) 0.785 0.778

3	(0.218) 0.215 0.222

Locus 11219 :

2 alleles

99.2% missing data

3	(0.468) 0.466 0.472

2	(0.532) 0.534 0.528

Locus 11220 :

2 alleles

98.4% missing data

4	(0.415) 0.415 0.418

3	(0.585) 0.585 0.582

Locus 11221 :

3 alleles

98.0% missing data

1	(0.389) 0.378 0.393

3	(0.353) 0.367 0.351

4	(0.258) 0.255 0.256

Locus 11222 :

2 alleles

92.6% missing data

4	(0.881) 0.879 0.893

3	(0.119) 0.121 0.107

Locus 11223 :

2 alleles

98.8% missing data

3	(0.386) 0.372 0.391

2	(0.614) 0.628 0.609

Locus 11224 :

2 alleles

99.2% missing data

3	(0.713) 0.711 0.715

1	(0.287) 0.289 0.285

Locus 11225 :

2 alleles

99.2% missing data

2	(0.401) 0.403 0.400

3	(0.599) 0.597 0.600

Locus 11226 :

2 alleles

96.1% missing data

1	(0.777) 0.784 0.784

3	(0.223) 0.216 0.216

Locus 11227 :

2 alleles

96.1% missing data

4	(0.128) 0.126 0.129

3	(0.872) 0.874 0.871

Locus 11228 :

2 alleles

8.2% missing data

2	(0.987) 0.977 0.996

3	(0.013) 0.023 0.004

Locus 11229 :

2 alleles

99.2% missing data

1	(0.550) 0.546 0.555

4	(0.450) 0.454 0.445

Locus 11230 :

2 alleles

99.2% missing data

3	(0.495) 0.504 0.491

4	(0.505) 0.496 0.509

Locus 11231 :

2 alleles

96.1% missing data

2	(0.822) 0.831 0.819

3	(0.178) 0.169 0.181

Locus 11232 :

2 alleles

99.2% missing data

4	(0.295) 0.297 0.292

3	(0.705) 0.703 0.708

Locus 11233 :

2 alleles

99.2% missing data

2	(0.493) 0.482 0.500

3	(0.507) 0.518 0.500

Locus 11234 :

2 alleles

98.4% missing data

1	(0.229) 0.222 0.232

2	(0.771) 0.778 0.768

Locus 11235 :

2 alleles

90.2% missing data

1	(0.847) 0.876 0.833

4	(0.153) 0.124 0.167

Locus 11236 :

2 alleles

99.2% missing data

1	(0.855) 0.857 0.850

4	(0.145) 0.143 0.150

Locus 11237 :

2 alleles

99.2% missing data

2	(0.533) 0.523 0.541

1	(0.467) 0.477 0.459

Locus 11238 :

2 alleles

93.8% missing data

3	(0.891) 0.892 0.896

2	(0.109) 0.108 0.104

Locus 11239 :

2 alleles

99.2% missing data

1	(0.618) 0.614 0.622

2	(0.382) 0.386 0.378

Locus 11240 :

2 alleles

98.0% missing data

1	(0.584) 0.578 0.601

4	(0.416) 0.422 0.399

Locus 11241 :

2 alleles

99.2% missing data

4	(0.324) 0.328 0.331

3	(0.676) 0.672 0.669

Locus 11242 :

2 alleles

99.2% missing data

3	(0.239) 0.246 0.236

1	(0.761) 0.754 0.764

Locus 11243 :

2 alleles

99.2% missing data

3	(0.654) 0.652 0.650

2	(0.346) 0.348 0.350

Locus 11244 :

2 alleles

97.3% missing data

3	(0.652) 0.664 0.653

2	(0.348) 0.336 0.347

Locus 11245 :

2 alleles

99.2% missing data

1	(0.159) 0.151 0.165

4	(0.841) 0.849 0.835

Locus 11246 :

2 alleles

99.2% missing data

4	(0.259) 0.262 0.254

3	(0.741) 0.738 0.746

Locus 11247 :

2 alleles

99.2% missing data

4	(0.516) 0.516 0.517

1	(0.484) 0.484 0.483

Locus 11248 :

3 alleles

98.8% missing data

4	(0.364) 0.370 0.357

3	(0.242) 0.243 0.249

1	(0.393) 0.387 0.394

Locus 11249 :

2 alleles

99.2% missing data

1	(0.586) 0.596 0.582

2	(0.414) 0.404 0.418

Locus 11250 :

2 alleles

99.2% missing data

2	(0.505) 0.501 0.513

3	(0.495) 0.499 0.487

Locus 11251 :

2 alleles

99.2% missing data

2	(0.252) 0.248 0.258

1	(0.748) 0.752 0.742

Locus 11252 :

2 alleles

99.2% missing data

1	(0.474) 0.470 0.477

3	(0.526) 0.530 0.523

Locus 11253 :

2 alleles

99.2% missing data

2	(0.688) 0.690 0.686

1	(0.312) 0.310 0.314

Locus 11254 :

2 alleles

99.2% missing data

1	(0.488) 0.492 0.484

4	(0.512) 0.508 0.516

Locus 11255 :

2 alleles

99.2% missing data

3	(0.368) 0.365 0.369

2	(0.632) 0.635 0.631

Locus 11256 :

2 alleles

99.2% missing data

1	(0.657) 0.653 0.654

3	(0.343) 0.347 0.346

Locus 11257 :

2 alleles

99.2% missing data

4	(0.243) 0.246 0.240

1	(0.757) 0.754 0.760

Locus 11258 :

3 alleles

98.4% missing data

4	(0.350) 0.346 0.362

3	(0.319) 0.317 0.316

1	(0.331) 0.337 0.322

Locus 11259 :

2 alleles

97.7% missing data

4	(0.659) 0.657 0.669

3	(0.341) 0.343 0.331

Locus 11260 :

2 alleles

98.8% missing data

1	(0.279) 0.277 0.282

4	(0.721) 0.723 0.718

Locus 11261 :

2 alleles

99.2% missing data

1	(0.778) 0.767 0.782

3	(0.222) 0.233 0.218

Locus 11262 :

2 alleles

99.2% missing data

2	(0.282) 0.283 0.291

1	(0.718) 0.717 0.709

Locus 11263 :

2 alleles

98.8% missing data

1	(0.501) 0.500 0.492

3	(0.499) 0.500 0.508

Locus 11264 :

2 alleles

99.2% missing data

1	(0.598) 0.601 0.598

2	(0.402) 0.399 0.402

Locus 11265 :

2 alleles

99.2% missing data

1	(0.648) 0.642 0.659

4	(0.352) 0.358 0.341

Locus 11266 :

2 alleles

90.2% missing data

4	(0.109) 0.087 0.127

1	(0.891) 0.913 0.873

Locus 11267 :

2 alleles

99.2% missing data

3	(0.540) 0.546 0.535

4	(0.460) 0.454 0.465

Locus 11268 :

2 alleles

98.8% missing data

1	(0.753) 0.759 0.750

2	(0.247) 0.241 0.250

Locus 11269 :

2 alleles

99.2% missing data

4	(0.561) 0.565 0.559

3	(0.439) 0.435 0.441

Locus 11270 :

2 alleles

96.1% missing data

4	(0.874) 0.884 0.871

2	(0.126) 0.116 0.129

Locus 11271 :

2 alleles

99.2% missing data

1	(0.307) 0.313 0.308

2	(0.693) 0.687 0.692

Locus 11272 :

2 alleles

99.2% missing data

2	(0.338) 0.336 0.339

3	(0.662) 0.664 0.661

Locus 11273 :

2 alleles

93.8% missing data

3	(0.845) 0.835 0.866

4	(0.155) 0.165 0.134

Locus 11274 :

2 alleles

98.8% missing data

3	(0.460) 0.454 0.456

2	(0.540) 0.546 0.544

Locus 11275 :

2 alleles

98.0% missing data

1	(0.860) 0.858 0.864

2	(0.140) 0.142 0.136

Locus 11276 :

2 alleles

98.8% missing data

2	(0.697) 0.695 0.688

1	(0.303) 0.305 0.312

Locus 11277 :

2 alleles

98.4% missing data

3	(0.680) 0.690 0.677

1	(0.320) 0.310 0.323

Locus 11278 :

2 alleles

99.2% missing data

3	(0.407) 0.411 0.410

1	(0.593) 0.589 0.590

Locus 11279 :

2 alleles

99.2% missing data

4	(0.499) 0.489 0.506

3	(0.501) 0.511 0.494

Locus 11280 :

2 alleles

96.1% missing data

3	(0.905) 0.913 0.897

4	(0.095) 0.087 0.103

Locus 11281 :

2 alleles

0.0% missing data

2	(0.984) 0.979 0.995

3	(0.016) 0.021 0.005

Locus 11282 :

2 alleles

90.6% missing data

3	(0.906) 0.904 0.917

4	(0.094) 0.096 0.083

Locus 11283 :

2 alleles

99.2% missing data

4	(0.602) 0.589 0.609

1	(0.398) 0.411 0.391

Locus 11284 :

2 alleles

92.6% missing data

3	(0.874) 0.865 0.894

1	(0.126) 0.135 0.106

Locus 11285 :

2 alleles

99.2% missing data

2	(0.318) 0.319 0.322

1	(0.682) 0.681 0.678

Locus 11286 :

2 alleles

99.2% missing data

4	(0.551) 0.555 0.541

1	(0.449) 0.445 0.459

Locus 11287 :

2 alleles

99.2% missing data

1	(0.578) 0.575 0.575

3	(0.422) 0.425 0.425

Locus 11288 :

2 alleles

99.2% missing data

3	(0.479) 0.475 0.480

2	(0.521) 0.525 0.520

Locus 11289 :

2 alleles

99.2% missing data

2	(0.256) 0.261 0.251

4	(0.744) 0.739 0.749

Locus 11290 :

2 alleles

99.2% missing data

1	(0.387) 0.387 0.385

3	(0.613) 0.613 0.615

Locus 11291 :

2 alleles

98.4% missing data

3	(0.748) 0.752 0.754

2	(0.252) 0.248 0.246

Locus 11292 :

2 alleles

96.1% missing data

1	(0.115) 0.105 0.123

2	(0.885) 0.895 0.877

Locus 11293 :

2 alleles

99.2% missing data

2	(0.310) 0.317 0.306

1	(0.690) 0.683 0.694

Locus 11294 :

2 alleles

98.8% missing data

1	(0.515) 0.518 0.516

3	(0.485) 0.482 0.484

Locus 11295 :

2 alleles

99.2% missing data

3	(0.433) 0.433 0.439

2	(0.567) 0.567 0.561

Locus 11296 :

2 alleles

0.4% missing data

4	(0.989) 0.992 0.994

1	(0.011) 0.008 0.006

Locus 11297 :

2 alleles

99.2% missing data

4	(0.614) 0.614 0.615

1	(0.386) 0.386 0.385

Locus 11298 :

2 alleles

99.2% missing data

3	(0.303) 0.301 0.300

1	(0.697) 0.699 0.700

Locus 11299 :

2 alleles

99.2% missing data

2	(0.581) 0.576 0.580

3	(0.419) 0.424 0.420

Locus 11300 :

2 alleles

99.2% missing data

1	(0.712) 0.714 0.703

2	(0.288) 0.286 0.297

Locus 11301 :

2 alleles

92.6% missing data

1	(0.926) 0.917 0.939

2	(0.074) 0.083 0.061

Locus 11302 :

2 alleles

99.2% missing data

2	(0.351) 0.346 0.356

1	(0.649) 0.654 0.644

Locus 11303 :

2 alleles

99.2% missing data

3	(0.353) 0.350 0.353

2	(0.647) 0.650 0.647

Locus 11304 :

2 alleles

99.2% missing data

2	(0.756) 0.754 0.767

4	(0.244) 0.246 0.233

Locus 11305 :

2 alleles

96.1% missing data

1	(0.832) 0.842 0.828

4	(0.168) 0.158 0.172

Locus 11306 :

2 alleles

99.2% missing data

1	(0.736) 0.730 0.737

2	(0.264) 0.270 0.263

Locus 11307 :

2 alleles

98.0% missing data

1	(0.803) 0.806 0.801

4	(0.197) 0.194 0.199

Locus 11308 :

2 alleles

99.2% missing data

2	(0.188) 0.182 0.192

4	(0.812) 0.818 0.808

Locus 11309 :

2 alleles

99.2% missing data

4	(0.556) 0.563 0.549

1	(0.444) 0.437 0.451

Locus 11310 :

2 alleles

99.2% missing data

4	(0.483) 0.480 0.482

1	(0.517) 0.520 0.518

Locus 11311 :

2 alleles

99.2% missing data

2	(0.657) 0.660 0.655

1	(0.343) 0.340 0.345

Locus 11312 :

2 alleles

99.2% missing data

1	(0.462) 0.457 0.464

3	(0.538) 0.543 0.536

Locus 11313 :

2 alleles

99.2% missing data

4	(0.691) 0.693 0.691

2	(0.309) 0.307 0.309

Locus 11314 :

2 alleles

97.7% missing data

1	(0.462) 0.468 0.450

3	(0.538) 0.532 0.550

Locus 11315 :

2 alleles

98.0% missing data

4	(0.700) 0.707 0.696

1	(0.300) 0.293 0.304

Locus 11316 :

2 alleles

99.2% missing data

4	(0.617) 0.614 0.621

1	(0.383) 0.386 0.379

Locus 11317 :

2 alleles

99.2% missing data

1	(0.532) 0.529 0.539

3	(0.468) 0.471 0.461

Locus 11318 :

2 alleles

99.2% missing data

4	(0.686) 0.689 0.683

1	(0.314) 0.311 0.317

Locus 11319 :

2 alleles

98.8% missing data

3	(0.708) 0.705 0.713

4	(0.292) 0.295 0.287

Locus 11320 :

2 alleles

99.2% missing data

3	(0.760) 0.760 0.754

1	(0.240) 0.240 0.246

Locus 11321 :

2 alleles

97.7% missing data

4	(0.245) 0.248 0.237

1	(0.755) 0.752 0.763

Locus 11322 :

2 alleles

98.8% missing data

1	(0.843) 0.843 0.838

3	(0.157) 0.157 0.162

Locus 11323 :

2 alleles

99.2% missing data

2	(0.273) 0.270 0.277

3	(0.727) 0.730 0.723

Locus 11324 :

2 alleles

99.2% missing data

4	(0.274) 0.277 0.279

2	(0.726) 0.723 0.721

Locus 11325 :

2 alleles

99.2% missing data

4	(0.493) 0.491 0.502

2	(0.507) 0.509 0.498

Locus 11326 :

2 alleles

96.1% missing data

4	(0.249) 0.234 0.247

3	(0.751) 0.766 0.753

Locus 11327 :

2 alleles

99.2% missing data

1	(0.455) 0.454 0.456

2	(0.545) 0.546 0.544

Locus 11328 :

2 alleles

96.9% missing data

1	(0.497) 0.493 0.500

3	(0.503) 0.507 0.500

Locus 11329 :

2 alleles

93.8% missing data

2	(0.117) 0.122 0.103

1	(0.883) 0.878 0.897

Locus 11330 :

2 alleles

99.2% missing data

3	(0.427) 0.433 0.418

1	(0.573) 0.567 0.582

Locus 11331 :

2 alleles

96.1% missing data

2	(0.195) 0.184 0.199

1	(0.805) 0.816 0.801

Locus 11332 :

2 alleles

98.4% missing data

3	(0.423) 0.423 0.429

4	(0.577) 0.577 0.571

Locus 11333 :

2 alleles

96.9% missing data

3	(0.597) 0.620 0.593

1	(0.403) 0.380 0.407

Locus 11334 :

2 alleles

99.2% missing data

3	(0.735) 0.733 0.730

4	(0.265) 0.267 0.270

Locus 11335 :

2 alleles

92.2% missing data

1	(0.899) 0.902 0.909

3	(0.101) 0.098 0.091

Locus 11336 :

2 alleles

96.1% missing data

1	(0.850) 0.859 0.844

2	(0.150) 0.141 0.156

Locus 11337 :

2 alleles

99.2% missing data

3	(0.558) 0.551 0.565

2	(0.442) 0.449 0.435

Locus 11338 :

2 alleles

99.2% missing data

3	(0.323) 0.319 0.328

4	(0.677) 0.681 0.672

Locus 11339 :

2 alleles

99.2% missing data

1	(0.306) 0.308 0.308

3	(0.694) 0.692 0.692

Locus 11340 :

2 alleles

97.7% missing data

1	(0.145) 0.147 0.146

2	(0.855) 0.853 0.854

Locus 11341 :

2 alleles

99.2% missing data

4	(0.410) 0.402 0.412

3	(0.590) 0.598 0.588

Locus 11342 :

2 alleles

96.1% missing data

4	(0.802) 0.810 0.804

3	(0.198) 0.190 0.196

Locus 11343 :

2 alleles

91.0% missing data

1	(0.921) 0.936 0.913

3	(0.079) 0.064 0.087

Locus 11344 :

2 alleles

99.2% missing data

1	(0.631) 0.629 0.629

3	(0.369) 0.371 0.371

Locus 11345 :

2 alleles

99.2% missing data

4	(0.638) 0.640 0.635

1	(0.362) 0.360 0.365

Locus 11346 :

2 alleles

98.4% missing data

4	(0.648) 0.647 0.650

2	(0.352) 0.353 0.350

Locus 11347 :

2 alleles

99.2% missing data

4	(0.518) 0.519 0.520

3	(0.482) 0.481 0.480

Locus 11348 :

2 alleles

92.6% missing data

4	(0.936) 0.930 0.944

2	(0.064) 0.070 0.056

Locus 11349 :

2 alleles

96.5% missing data

1	(0.818) 0.820 0.822

3	(0.182) 0.180 0.178

Locus 11350 :

2 alleles

99.2% missing data

2	(0.649) 0.648 0.648

4	(0.351) 0.352 0.352

Locus 11351 :

2 alleles

98.8% missing data

2	(0.728) 0.721 0.736

1	(0.272) 0.279 0.264

Locus 11352 :

2 alleles

98.8% missing data

1	(0.800) 0.790 0.802

3	(0.200) 0.210 0.198

Locus 11353 :

2 alleles

97.7% missing data

1	(0.254) 0.259 0.244

3	(0.746) 0.741 0.756

Locus 11354 :

2 alleles

99.2% missing data

1	(0.456) 0.456 0.461

2	(0.544) 0.544 0.539

Locus 11355 :

2 alleles

99.2% missing data

1	(0.719) 0.714 0.714

4	(0.281) 0.286 0.286

Locus 11356 :

2 alleles

98.8% missing data

3	(0.711) 0.718 0.704

1	(0.289) 0.282 0.296

Locus 11357 :

2 alleles

99.2% missing data

4	(0.557) 0.547 0.561

1	(0.443) 0.453 0.439

Locus 11358 :

2 alleles

99.2% missing data

1	(0.482) 0.489 0.479

2	(0.518) 0.511 0.521

Locus 11359 :

2 alleles

99.2% missing data

1	(0.419) 0.421 0.424

2	(0.581) 0.579 0.576

Locus 11360 :

2 alleles

99.2% missing data

4	(0.658) 0.660 0.656

3	(0.342) 0.340 0.344

Locus 11361 :

2 alleles

97.7% missing data

3	(0.118) 0.113 0.120

1	(0.882) 0.887 0.880

Locus 11362 :

2 alleles

96.9% missing data

2	(0.890) 0.898 0.886

3	(0.110) 0.102 0.114

Locus 11363 :

2 alleles

99.2% missing data

1	(0.762) 0.755 0.765

4	(0.238) 0.245 0.235

Locus 11364 :

2 alleles

99.2% missing data

1	(0.439) 0.444 0.439

3	(0.561) 0.556 0.561

Locus 11365 :

2 alleles

99.2% missing data

1	(0.526) 0.519 0.526

4	(0.474) 0.481 0.474

Locus 11366 :

2 alleles

91.0% missing data

2	(0.908) 0.927 0.898

1	(0.092) 0.073 0.102

Locus 11367 :

2 alleles

99.2% missing data

4	(0.746) 0.743 0.744

2	(0.254) 0.257 0.256

Locus 11368 :

2 alleles

99.2% missing data

4	(0.620) 0.613 0.625

3	(0.380) 0.387 0.375

Locus 11369 :

2 alleles

99.2% missing data

1	(0.314) 0.314 0.312

2	(0.686) 0.686 0.688

Locus 11370 :

2 alleles

99.2% missing data

3	(0.514) 0.506 0.516

2	(0.486) 0.494 0.484

Locus 11371 :

3 alleles

98.8% missing data

3	(0.375) 0.375 0.379

1	(0.294) 0.297 0.295

2	(0.331) 0.328 0.326

Locus 11372 :

3 alleles

98.8% missing data

2	(0.342) 0.340 0.343

1	(0.344) 0.346 0.346

3	(0.314) 0.313 0.311

Locus 11373 :

2 alleles

99.2% missing data

4	(0.349) 0.357 0.344

2	(0.651) 0.643 0.656

Locus 11374 :

2 alleles

96.5% missing data

1	(0.191) 0.181 0.198

4	(0.809) 0.819 0.802

Locus 11375 :

2 alleles

96.1% missing data

3	(0.786) 0.786 0.792

1	(0.214) 0.214 0.208

Locus 11376 :

2 alleles

99.2% missing data

1	(0.739) 0.733 0.747

4	(0.261) 0.267 0.253

Locus 11377 :

2 alleles

98.4% missing data

3	(0.254) 0.251 0.255

4	(0.746) 0.749 0.745

Locus 11378 :

2 alleles

99.2% missing data

4	(0.654) 0.650 0.653

1	(0.346) 0.350 0.347

Locus 11379 :

3 alleles

97.7% missing data

4	(0.545) 0.555 0.541

1	(0.260) 0.249 0.262

2	(0.195) 0.196 0.197

Locus 11380 :

2 alleles

97.7% missing data

1	(0.157) 0.157 0.155

3	(0.843) 0.843 0.845

Locus 11381 :

2 alleles

99.2% missing data

4	(0.612) 0.609 0.611

1	(0.388) 0.391 0.389

Locus 11382 :

2 alleles

97.7% missing data

4	(0.680) 0.691 0.674

3	(0.320) 0.309 0.326

Locus 11383 :

2 alleles

99.2% missing data

1	(0.321) 0.312 0.330

2	(0.679) 0.688 0.670

Locus 11384 :

2 alleles

99.2% missing data

4	(0.531) 0.530 0.531

3	(0.469) 0.470 0.469

Locus 11385 :

2 alleles

99.2% missing data

3	(0.452) 0.451 0.453

2	(0.548) 0.549 0.547

Locus 11386 :

2 alleles

98.8% missing data

1	(0.444) 0.450 0.436

3	(0.556) 0.550 0.564

Locus 11387 :

2 alleles

99.2% missing data

4	(0.639) 0.635 0.647

2	(0.361) 0.365 0.353

Locus 11388 :

2 alleles

98.8% missing data

1	(0.527) 0.524 0.530

4	(0.473) 0.476 0.470

Locus 11389 :

2 alleles

98.4% missing data

3	(0.540) 0.555 0.537

1	(0.460) 0.445 0.463

Locus 11390 :

2 alleles

99.2% missing data

1	(0.486) 0.481 0.482

4	(0.514) 0.519 0.518

Locus 11391 :

2 alleles

98.4% missing data

1	(0.280) 0.283 0.276

2	(0.720) 0.717 0.724

Locus 11392 :

2 alleles

0.0% missing data

4	(0.984) 0.987 0.992

1	(0.016) 0.013 0.008

Locus 11393 :

2 alleles

97.3% missing data

3	(0.265) 0.250 0.263

2	(0.735) 0.750 0.737

Locus 11394 :

2 alleles

91.0% missing data

4	(0.840) 0.855 0.841

2	(0.160) 0.145 0.159

Locus 11395 :

2 alleles

99.2% missing data

1	(0.390) 0.386 0.395

3	(0.610) 0.614 0.605

Locus 11396 :

2 alleles

99.2% missing data

3	(0.325) 0.326 0.321

1	(0.675) 0.674 0.679

Locus 11397 :

2 alleles

99.2% missing data

2	(0.572) 0.576 0.571

1	(0.428) 0.424 0.429

Locus 11398 :

2 alleles

99.2% missing data

2	(0.585) 0.573 0.589

4	(0.415) 0.427 0.411

Locus 11399 :

2 alleles

96.1% missing data

3	(0.127) 0.121 0.132

2	(0.873) 0.879 0.868

Locus 11400 :

2 alleles

99.2% missing data

1	(0.660) 0.662 0.655

3	(0.340) 0.338 0.345

Locus 11401 :

2 alleles

98.4% missing data

4	(0.655) 0.658 0.650

1	(0.345) 0.342 0.350

Locus 11402 :

2 alleles

99.2% missing data

4	(0.533) 0.535 0.533

3	(0.467) 0.465 0.467

Locus 11403 :

2 alleles

99.2% missing data

1	(0.367) 0.358 0.372

2	(0.633) 0.642 0.628

Locus 11404 :

2 alleles

99.2% missing data

1	(0.602) 0.593 0.610

4	(0.398) 0.407 0.390

Locus 11405 :

2 alleles

99.2% missing data

2	(0.626) 0.620 0.627

4	(0.374) 0.380 0.373

Locus 11406 :

2 alleles

98.8% missing data

2	(0.589) 0.592 0.594

1	(0.411) 0.408 0.406

Locus 11407 :

2 alleles

96.1% missing data

1	(0.782) 0.793 0.777

3	(0.218) 0.207 0.223

Locus 11408 :

2 alleles

96.1% missing data

4	(0.126) 0.121 0.124

2	(0.874) 0.879 0.876

Locus 11409 :

2 alleles

98.8% missing data

2	(0.405) 0.403 0.411

1	(0.595) 0.597 0.589

Locus 11410 :

2 alleles

99.2% missing data

4	(0.576) 0.570 0.578

3	(0.424) 0.430 0.422

Locus 11411 :

2 alleles

99.2% missing data

1	(0.554) 0.548 0.560

4	(0.446) 0.452 0.440

Locus 11412 :

2 alleles

99.2% missing data

3	(0.568) 0.564 0.567

4	(0.432) 0.436 0.433

Locus 11413 :

2 alleles

99.2% missing data

3	(0.711) 0.714 0.716

2	(0.289) 0.286 0.284

Locus 11414 :

2 alleles

98.4% missing data

1	(0.713) 0.723 0.708

3	(0.287) 0.277 0.292

Locus 11415 :

2 alleles

99.2% missing data

4	(0.475) 0.474 0.478

1	(0.525) 0.526 0.522

Locus 11416 :

2 alleles

97.7% missing data

2	(0.751) 0.758 0.746

3	(0.249) 0.242 0.254

Locus 11417 :

2 alleles

99.2% missing data

3	(0.256) 0.262 0.254

1	(0.744) 0.738 0.746

Locus 11418 :

2 alleles

99.2% missing data

3	(0.372) 0.375 0.364

1	(0.628) 0.625 0.636

Locus 11419 :

2 alleles

96.1% missing data

1	(0.881) 0.881 0.882

4	(0.119) 0.119 0.118

Locus 11420 :

2 alleles

0.0% missing data

4	(0.968) 0.977 0.967

3	(0.032) 0.023 0.033

Locus 11421 :

2 alleles

94.9% missing data

4	(0.837) 0.834 0.839

3	(0.163) 0.166 0.161

Locus 11422 :

2 alleles

98.8% missing data

2	(0.497) 0.495 0.506

1	(0.503) 0.505 0.494

Locus 11423 :

2 alleles

99.2% missing data

4	(0.383) 0.392 0.383

3	(0.617) 0.608 0.617

Locus 11424 :

2 alleles

99.2% missing data

3	(0.140) 0.142 0.139

2	(0.860) 0.858 0.861

Locus 11425 :

2 alleles

99.2% missing data

1	(0.578) 0.576 0.584

3	(0.422) 0.424 0.416

Locus 11426 :

2 alleles

99.2% missing data

4	(0.307) 0.305 0.311

1	(0.693) 0.695 0.689

Locus 11427 :

2 alleles

99.2% missing data

2	(0.503) 0.501 0.500

4	(0.497) 0.499 0.500

Locus 11428 :

2 alleles

0.0% missing data

4	(0.983) 0.986 0.992

1	(0.017) 0.014 0.008

Locus 11429 :

3 alleles

98.8% missing data

4	(0.138) 0.135 0.144

1	(0.348) 0.347 0.350

3	(0.515) 0.518 0.506

Locus 11430 :

2 alleles

99.2% missing data

2	(0.453) 0.451 0.452

1	(0.547) 0.549 0.548

Locus 11431 :

2 alleles

99.2% missing data

3	(0.244) 0.245 0.249

4	(0.756) 0.755 0.751

Locus 11432 :

2 alleles

99.2% missing data

3	(0.206) 0.204 0.209

4	(0.794) 0.796 0.791

Locus 11433 :

2 alleles

98.4% missing data

2	(0.564) 0.557 0.580

4	(0.436) 0.443 0.420

Locus 11434 :

2 alleles

96.5% missing data

1	(0.227) 0.212 0.233

4	(0.773) 0.788 0.767

Locus 11435 :

2 alleles

98.8% missing data

3	(0.638) 0.633 0.643

1	(0.362) 0.367 0.357

Locus 11436 :

2 alleles

99.2% missing data

4	(0.594) 0.589 0.601

3	(0.406) 0.411 0.399

Locus 11437 :

2 alleles

99.2% missing data

4	(0.629) 0.625 0.630

2	(0.371) 0.375 0.370

Locus 11438 :

2 alleles

99.2% missing data

4	(0.684) 0.684 0.685

2	(0.316) 0.316 0.315

Locus 11439 :

2 alleles

98.8% missing data

3	(0.463) 0.468 0.456

4	(0.537) 0.532 0.544

Locus 11440 :

2 alleles

99.2% missing data

1	(0.535) 0.531 0.533

2	(0.465) 0.469 0.467

Locus 11441 :

2 alleles

99.2% missing data

4	(0.480) 0.479 0.477

1	(0.520) 0.521 0.523

Locus 11442 :

2 alleles

99.2% missing data

1	(0.631) 0.632 0.625

3	(0.369) 0.368 0.375

Locus 11443 :

2 alleles

99.2% missing data

1	(0.467) 0.474 0.468

2	(0.533) 0.526 0.532

Locus 11444 :

2 alleles

98.0% missing data

1	(0.785) 0.779 0.796

3	(0.215) 0.221 0.204

Locus 11445 :

2 alleles

99.2% missing data

4	(0.947) 0.948 0.940

3	(0.053) 0.052 0.060

Locus 11446 :

2 alleles

96.5% missing data

4	(0.755) 0.742 0.775

1	(0.245) 0.258 0.225

Locus 11447 :

2 alleles

99.2% missing data

3	(0.441) 0.448 0.438

1	(0.559) 0.552 0.562

Locus 11448 :

2 alleles

99.2% missing data

3	(0.292) 0.291 0.291

2	(0.708) 0.709 0.709

Locus 11449 :

2 alleles

99.2% missing data

1	(0.611) 0.609 0.612

2	(0.389) 0.391 0.388

Locus 11450 :

3 alleles

98.8% missing data

1	(0.292) 0.302 0.283

4	(0.397) 0.394 0.399

3	(0.311) 0.304 0.319

Locus 11451 :

2 alleles

89.5% missing data

3	(0.770) 0.817 0.731

4	(0.230) 0.183 0.269

Locus 11452 :

2 alleles

99.2% missing data

1	(0.580) 0.580 0.583

3	(0.420) 0.420 0.417

Locus 11453 :

2 alleles

99.2% missing data

1	(0.343) 0.350 0.340

4	(0.657) 0.650 0.660

Locus 11454 :

2 alleles

99.2% missing data

3	(0.311) 0.321 0.306

1	(0.689) 0.679 0.694

Locus 11455 :

2 alleles

89.5% missing data

1	(0.878) 0.886 0.884

2	(0.122) 0.114 0.116

Locus 11456 :

2 alleles

99.2% missing data

1	(0.270) 0.268 0.277

3	(0.730) 0.732 0.723

Locus 11457 :

2 alleles

99.2% missing data

1	(0.458) 0.456 0.455

2	(0.542) 0.544 0.545

Locus 11458 :

2 alleles

99.2% missing data

1	(0.376) 0.370 0.384

3	(0.624) 0.630 0.616

Locus 11459 :

2 alleles

99.2% missing data

2	(0.724) 0.723 0.714

3	(0.276) 0.277 0.286

Locus 11460 :

2 alleles

99.2% missing data

1	(0.407) 0.411 0.405

4	(0.593) 0.589 0.595

Locus 11461 :

2 alleles

99.2% missing data

3	(0.486) 0.479 0.495

4	(0.514) 0.521 0.505

Locus 11462 :

3 alleles

98.0% missing data

1	(0.451) 0.447 0.449

2	(0.372) 0.365 0.380

3	(0.177) 0.188 0.171

Locus 11463 :

2 alleles

99.2% missing data

2	(0.369) 0.367 0.373

3	(0.631) 0.633 0.627

Locus 11464 :

2 alleles

99.2% missing data

1	(0.486) 0.487 0.491

3	(0.514) 0.513 0.509

Locus 11465 :

2 alleles

99.2% missing data

1	(0.535) 0.539 0.533

3	(0.465) 0.461 0.467

Locus 11466 :

2 alleles

91.0% missing data

3	(0.918) 0.935 0.909

1	(0.082) 0.065 0.091

Locus 11467 :

2 alleles

98.4% missing data

2	(0.462) 0.453 0.473

4	(0.538) 0.547 0.527

Locus 11468 :

2 alleles

99.2% missing data

4	(0.777) 0.780 0.775

2	(0.223) 0.220 0.225

Locus 11469 :

2 alleles

98.8% missing data

4	(0.341) 0.347 0.341

1	(0.659) 0.653 0.659

Locus 11470 :

2 alleles

97.7% missing data

4	(0.353) 0.358 0.336

3	(0.647) 0.642 0.664

Locus 11471 :

2 alleles

99.2% missing data

4	(0.453) 0.456 0.448

3	(0.547) 0.544 0.552

Locus 11472 :

2 alleles

99.2% missing data

4	(0.471) 0.469 0.478

3	(0.529) 0.531 0.522

Locus 11473 :

2 alleles

91.0% missing data

2	(0.939) 0.953 0.930

3	(0.061) 0.047 0.070

Locus 11474 :

2 alleles

98.8% missing data

2	(0.525) 0.531 0.524

4	(0.475) 0.469 0.476

Locus 11475 :

2 alleles

99.2% missing data

3	(0.671) 0.673 0.668

1	(0.329) 0.327 0.332

Locus 11476 :

2 alleles

99.2% missing data

3	(0.296) 0.297 0.296

2	(0.704) 0.703 0.704

Locus 11477 :

2 alleles

99.2% missing data

2	(0.308) 0.303 0.314

1	(0.692) 0.697 0.686

Locus 11478 :

2 alleles

98.8% missing data

1	(0.780) 0.766 0.791

3	(0.220) 0.234 0.209

Locus 11479 :

3 alleles

98.8% missing data

1	(0.368) 0.363 0.370

3	(0.358) 0.357 0.356

2	(0.274) 0.279 0.274

Locus 11480 :

2 alleles

99.2% missing data

3	(0.534) 0.531 0.541

2	(0.466) 0.469 0.459

Locus 11481 :

2 alleles

99.2% missing data

3	(0.624) 0.631 0.613

2	(0.376) 0.369 0.387

Locus 11482 :

2 alleles

99.2% missing data

4	(0.635) 0.633 0.632

1	(0.365) 0.367 0.368

Locus 11483 :

2 alleles

99.2% missing data

4	(0.561) 0.555 0.566

3	(0.439) 0.445 0.434

Locus 11484 :

2 alleles

99.2% missing data

3	(0.453) 0.453 0.449

4	(0.547) 0.547 0.551

Locus 11485 :

2 alleles

99.2% missing data

1	(0.605) 0.595 0.613

3	(0.395) 0.405 0.387

Locus 11486 :

2 alleles

99.2% missing data

2	(0.475) 0.472 0.476

3	(0.525) 0.528 0.524

Locus 11487 :

2 alleles

99.2% missing data

1	(0.768) 0.771 0.767

3	(0.232) 0.229 0.233

Locus 11488 :

2 alleles

99.2% missing data

3	(0.700) 0.697 0.699

1	(0.300) 0.303 0.301

Locus 11489 :

2 alleles

98.8% missing data

2	(0.473) 0.473 0.482

1	(0.527) 0.527 0.518

Locus 11490 :

2 alleles

99.2% missing data

4	(0.213) 0.215 0.218

2	(0.787) 0.785 0.782

Locus 11491 :

2 alleles

97.3% missing data

1	(0.847) 0.844 0.854

4	(0.153) 0.156 0.146

Locus 11492 :

2 alleles

99.2% missing data

3	(0.570) 0.574 0.574

4	(0.430) 0.426 0.426

Locus 11493 :

2 alleles

99.2% missing data

1	(0.492) 0.490 0.498

3	(0.508) 0.510 0.502

Locus 11494 :

2 alleles

98.8% missing data

1	(0.293) 0.293 0.299

3	(0.707) 0.707 0.701

Locus 11495 :

2 alleles

98.4% missing data

4	(0.741) 0.743 0.746

3	(0.259) 0.257 0.254

Locus 11496 :

2 alleles

99.2% missing data

1	(0.583) 0.584 0.584

3	(0.417) 0.416 0.416

Locus 11497 :

2 alleles

99.2% missing data

1	(0.525) 0.526 0.523

4	(0.475) 0.474 0.477

Locus 11498 :

2 alleles

99.2% missing data

2	(0.399) 0.394 0.401

4	(0.601) 0.606 0.599

Locus 11499 :

2 alleles

99.2% missing data

3	(0.479) 0.477 0.483

4	(0.521) 0.523 0.517

Locus 11500 :

2 alleles

98.8% missing data

1	(0.309) 0.301 0.316

4	(0.691) 0.699 0.684

Locus 11501 :

2 alleles

99.2% missing data

2	(0.419) 0.416 0.430

3	(0.581) 0.584 0.570

Locus 11502 :

2 alleles

98.8% missing data

3	(0.455) 0.453 0.457

1	(0.545) 0.547 0.543

Locus 11503 :

2 alleles

99.2% missing data

3	(0.319) 0.317 0.321

2	(0.681) 0.683 0.679

Locus 11504 :

2 alleles

99.2% missing data

4	(0.279) 0.273 0.279

1	(0.721) 0.727 0.721

Locus 11505 :

2 alleles

98.8% missing data

1	(0.385) 0.389 0.379

3	(0.615) 0.611 0.621

Locus 11506 :

2 alleles

98.8% missing data

4	(0.494) 0.492 0.492

1	(0.506) 0.508 0.508

Locus 11507 :

2 alleles

97.7% missing data

1	(0.615) 0.618 0.618

2	(0.385) 0.382 0.382

Locus 11508 :

2 alleles

99.2% missing data

2	(0.750) 0.753 0.747

1	(0.250) 0.247 0.253

Locus 11509 :

2 alleles

99.2% missing data

1	(0.536) 0.524 0.539

2	(0.464) 0.476 0.461

Locus 11510 :

3 alleles

98.8% missing data

1	(0.434) 0.433 0.435

4	(0.280) 0.278 0.282

3	(0.286) 0.288 0.283

Locus 11511 :

2 alleles

99.2% missing data

4	(0.693) 0.693 0.692

2	(0.307) 0.307 0.308

Locus 11512 :

2 alleles

99.2% missing data

3	(0.637) 0.640 0.636

4	(0.363) 0.360 0.364

Locus 11513 :

2 alleles

98.8% missing data

1	(0.685) 0.677 0.693

4	(0.315) 0.323 0.307

Locus 11514 :

2 alleles

99.2% missing data

2	(0.523) 0.518 0.532

4	(0.477) 0.482 0.468

Locus 11515 :

2 alleles

99.2% missing data

1	(0.315) 0.319 0.318

2	(0.685) 0.681 0.682

Locus 11516 :

2 alleles

99.2% missing data

1	(0.335) 0.327 0.344

3	(0.665) 0.673 0.656

Locus 11517 :

2 alleles

8.2% missing data

3	(0.980) 0.971 0.994

2	(0.020) 0.029 0.006

Locus 11518 :

2 alleles

99.2% missing data

3	(0.742) 0.729 0.747

4	(0.258) 0.271 0.253

Locus 11519 :

2 alleles

91.0% missing data

3	(0.934) 0.947 0.925

1	(0.066) 0.053 0.075

Locus 11520 :

2 alleles

99.2% missing data

1	(0.723) 0.720 0.729

2	(0.277) 0.280 0.271

Locus 11521 :

2 alleles

99.2% missing data

1	(0.393) 0.387 0.395

3	(0.607) 0.613 0.605

Locus 11522 :

2 alleles

98.8% missing data

4	(0.517) 0.524 0.517

2	(0.483) 0.476 0.483

Locus 11523 :

2 alleles

99.2% missing data

3	(0.584) 0.579 0.582

2	(0.416) 0.421 0.418

Locus 11524 :

2 alleles

90.6% missing data

3	(0.913) 0.932 0.900

1	(0.087) 0.068 0.100

Locus 11525 :

2 alleles

91.0% missing data

2	(0.905) 0.923 0.895

3	(0.095) 0.077 0.105

Locus 11526 :

2 alleles

99.2% missing data

3	(0.707) 0.702 0.716

1	(0.293) 0.298 0.284

Locus 11527 :

2 alleles

99.2% missing data

1	(0.441) 0.436 0.447

3	(0.559) 0.564 0.553

Locus 11528 :

2 alleles

88.7% missing data

4	(0.932) 0.930 0.943

2	(0.068) 0.070 0.057

Locus 11529 :

2 alleles

99.2% missing data

3	(0.528) 0.530 0.527

4	(0.472) 0.470 0.473

Locus 11530 :

2 alleles

99.2% missing data

1	(0.759) 0.759 0.758

4	(0.241) 0.241 0.242

Locus 11531 :

2 alleles

97.7% missing data

1	(0.136) 0.137 0.133

4	(0.864) 0.863 0.867

Locus 11532 :

2 alleles

98.8% missing data

2	(0.693) 0.694 0.692

1	(0.307) 0.306 0.308

Locus 11533 :

2 alleles

91.0% missing data

1	(0.919) 0.933 0.912

4	(0.081) 0.067 0.088

Locus 11534 :

2 alleles

99.2% missing data

2	(0.422) 0.422 0.429

3	(0.578) 0.578 0.571

Locus 11535 :

2 alleles

99.2% missing data

2	(0.427) 0.422 0.438

3	(0.573) 0.578 0.562

Locus 11536 :

2 alleles

0.0% missing data

3	(0.902) 0.744 0.971

4	(0.098) 0.256 0.029

Locus 11537 :

2 alleles

99.2% missing data

4	(0.455) 0.452 0.460

1	(0.545) 0.548 0.540

Locus 11538 :

3 alleles

98.4% missing data

2	(0.290) 0.293 0.296

1	(0.303) 0.303 0.295

3	(0.407) 0.405 0.408

Locus 11539 :

2 alleles

99.2% missing data

1	(0.570) 0.574 0.567

3	(0.430) 0.426 0.433

Locus 11540 :

2 alleles

96.9% missing data

1	(0.768) 0.770 0.771

2	(0.232) 0.230 0.229

Locus 11541 :

2 alleles

99.2% missing data

2	(0.584) 0.576 0.586

3	(0.416) 0.424 0.414

Locus 11542 :

2 alleles

99.2% missing data

4	(0.671) 0.679 0.663

1	(0.329) 0.321 0.337

Locus 11543 :

2 alleles

99.2% missing data

1	(0.449) 0.444 0.453

2	(0.551) 0.556 0.547

Locus 11544 :

2 alleles

99.2% missing data

1	(0.373) 0.361 0.381

3	(0.627) 0.639 0.619

Locus 11545 :

2 alleles

99.2% missing data

1	(0.389) 0.384 0.398

3	(0.611) 0.616 0.602

Locus 11546 :

2 alleles

99.2% missing data

3	(0.346) 0.342 0.349

4	(0.654) 0.658 0.651

Locus 11547 :

3 alleles

85.2% missing data

4	(0.877) 0.868 0.903

2	(0.054) 0.053 0.045

1	(0.069) 0.079 0.052

Locus 11548 :

2 alleles

98.4% missing data

4	(0.838) 0.834 0.840

3	(0.162) 0.166 0.160

Locus 11549 :

2 alleles

99.2% missing data

2	(0.559) 0.552 0.568

1	(0.441) 0.448 0.432

Locus 11550 :

2 alleles

99.2% missing data

4	(0.552) 0.553 0.549

1	(0.448) 0.447 0.451

Locus 11551 :

2 alleles

99.2% missing data

3	(0.265) 0.263 0.271

1	(0.735) 0.737 0.729

Locus 11552 :

2 alleles

99.2% missing data

1	(0.436) 0.436 0.440

3	(0.564) 0.564 0.560

Locus 11553 :

2 alleles

99.2% missing data

1	(0.592) 0.596 0.588

3	(0.408) 0.404 0.412

Locus 11554 :

2 alleles

98.8% missing data

1	(0.571) 0.572 0.571

3	(0.429) 0.428 0.429

Locus 11555 :

2 alleles

99.2% missing data

1	(0.338) 0.330 0.344

4	(0.662) 0.670 0.656

Locus 11556 :

2 alleles

98.8% missing data

1	(0.492) 0.495 0.495

4	(0.508) 0.505 0.505

Locus 11557 :

3 alleles

98.8% missing data

1	(0.266) 0.265 0.273

3	(0.288) 0.299 0.280

2	(0.445) 0.436 0.448

Locus 11558 :

2 alleles

99.2% missing data

1	(0.539) 0.538 0.541

3	(0.461) 0.462 0.459

Locus 11559 :

2 alleles

92.6% missing data

2	(0.868) 0.861 0.888

1	(0.132) 0.139 0.112

Locus 11560 :

2 alleles

92.2% missing data

1	(0.923) 0.938 0.912

2	(0.077) 0.062 0.088

Locus 11561 :

2 alleles

99.2% missing data

3	(0.543) 0.549 0.534

2	(0.457) 0.451 0.466

Locus 11562 :

2 alleles

96.9% missing data

1	(0.217) 0.225 0.206

4	(0.783) 0.775 0.794

Locus 11563 :

2 alleles

96.9% missing data

4	(0.797) 0.793 0.808

2	(0.203) 0.207 0.192

Locus 11564 :

2 alleles

96.5% missing data

1	(0.811) 0.808 0.823

2	(0.189) 0.192 0.177

Locus 11565 :

2 alleles

99.2% missing data

3	(0.666) 0.661 0.672

4	(0.334) 0.339 0.328

Locus 11566 :

2 alleles

96.1% missing data

4	(0.137) 0.131 0.138

2	(0.863) 0.869 0.862

Locus 11567 :

2 alleles

99.2% missing data

3	(0.396) 0.388 0.405

1	(0.604) 0.612 0.595

Locus 11568 :

2 alleles

99.2% missing data

3	(0.392) 0.390 0.396

1	(0.608) 0.610 0.604

Locus 11569 :

2 alleles

97.3% missing data

1	(0.295) 0.293 0.282

2	(0.705) 0.707 0.718

Locus 11570 :

2 alleles

99.2% missing data

1	(0.356) 0.359 0.361

2	(0.644) 0.641 0.639

Locus 11571 :

2 alleles

98.8% missing data

1	(0.679) 0.674 0.684

2	(0.321) 0.326 0.316

Locus 11572 :

2 alleles

99.2% missing data

2	(0.260) 0.254 0.268

4	(0.740) 0.746 0.732

Locus 11573 :

2 alleles

99.2% missing data

2	(0.391) 0.385 0.399

1	(0.609) 0.615 0.601

Locus 11574 :

2 alleles

99.2% missing data

4	(0.560) 0.561 0.557

1	(0.440) 0.439 0.443

Locus 11575 :

2 alleles

98.8% missing data

4	(0.578) 0.573 0.583

1	(0.422) 0.427 0.417

Locus 11576 :

2 alleles

99.2% missing data

2	(0.437) 0.429 0.444

1	(0.563) 0.571 0.556

Locus 11577 :

2 alleles

99.2% missing data

2	(0.410) 0.409 0.407

4	(0.590) 0.591 0.593

Locus 11578 :

2 alleles

99.2% missing data

1	(0.349) 0.349 0.348

2	(0.651) 0.651 0.652

Locus 11579 :

3 alleles

98.0% missing data

1	(0.239) 0.237 0.236

3	(0.521) 0.522 0.529

2	(0.240) 0.241 0.235

Locus 11580 :

2 alleles

99.2% missing data

3	(0.304) 0.311 0.294

4	(0.696) 0.689 0.706

Locus 11581 :

2 alleles

99.2% missing data

1	(0.340) 0.336 0.342

3	(0.660) 0.664 0.658

Locus 11582 :

2 alleles

98.4% missing data

3	(0.503) 0.500 0.503

1	(0.497) 0.500 0.497

Locus 11583 :

2 alleles

98.0% missing data

3	(0.761) 0.760 0.770

1	(0.239) 0.240 0.230

Locus 11584 :

2 alleles

99.2% missing data

4	(0.685) 0.682 0.686

3	(0.315) 0.318 0.314

Locus 11585 :

2 alleles

93.0% missing data

2	(0.919) 0.910 0.934

1	(0.081) 0.090 0.066

Locus 11586 :

2 alleles

99.2% missing data

4	(0.584) 0.584 0.587

2	(0.416) 0.416 0.413

Locus 11587 :

2 alleles

99.2% missing data

3	(0.482) 0.486 0.485

4	(0.518) 0.514 0.515

Locus 11588 :

2 alleles

96.9% missing data

3	(0.897) 0.889 0.908

1	(0.103) 0.111 0.092

Locus 11589 :

2 alleles

99.2% missing data

4	(0.324) 0.320 0.328

1	(0.676) 0.680 0.672

Locus 11590 :

2 alleles

99.2% missing data

1	(0.305) 0.304 0.308

2	(0.695) 0.696 0.692

Locus 11591 :

2 alleles

98.4% missing data

2	(0.618) 0.623 0.620

3	(0.382) 0.377 0.380

Locus 11592 :

2 alleles

99.2% missing data

2	(0.548) 0.548 0.548

1	(0.452) 0.452 0.452

Locus 11593 :

2 alleles

99.2% missing data

3	(0.539) 0.543 0.537

4	(0.461) 0.457 0.463

Locus 11594 :

2 alleles

99.2% missing data

2	(0.539) 0.530 0.541

4	(0.461) 0.470 0.459

Locus 11595 :

2 alleles

99.2% missing data

3	(0.687) 0.688 0.685

2	(0.313) 0.312 0.315

Locus 11596 :

2 alleles

9.4% missing data

2	(0.986) 0.980 0.995

1	(0.014) 0.020 0.005

Locus 11597 :

2 alleles

97.3% missing data

2	(0.521) 0.525 0.511

4	(0.479) 0.475 0.489

Locus 11598 :

2 alleles

91.0% missing data

4	(0.905) 0.925 0.894

1	(0.095) 0.075 0.106

Locus 11599 :

2 alleles

99.2% missing data

4	(0.607) 0.612 0.601

3	(0.393) 0.388 0.399

Locus 11600 :

2 alleles

99.2% missing data

2	(0.676) 0.680 0.671

3	(0.324) 0.320 0.329

Locus 11601 :

2 alleles

99.2% missing data

2	(0.778) 0.778 0.777

1	(0.222) 0.222 0.223

Locus 11602 :

2 alleles

99.2% missing data

4	(0.335) 0.333 0.347

2	(0.665) 0.667 0.653

Locus 11603 :

2 alleles

99.2% missing data

1	(0.464) 0.462 0.467

4	(0.536) 0.538 0.533

Locus 11604 :

2 alleles

98.8% missing data

3	(0.591) 0.601 0.581

1	(0.409) 0.399 0.419

Locus 11605 :

2 alleles

99.2% missing data

1	(0.485) 0.481 0.486

2	(0.515) 0.519 0.514

Locus 11606 :

2 alleles

99.2% missing data

3	(0.562) 0.565 0.557

4	(0.438) 0.435 0.443

Locus 11607 :

2 alleles

99.2% missing data

4	(0.575) 0.571 0.576

3	(0.425) 0.429 0.424

Locus 11608 :

2 alleles

99.2% missing data

3	(0.341) 0.340 0.343

1	(0.659) 0.660 0.657

Locus 11609 :

2 alleles

99.2% missing data

2	(0.277) 0.283 0.277

3	(0.723) 0.717 0.723

Locus 11610 :

2 alleles

99.2% missing data

1	(0.624) 0.630 0.617

3	(0.376) 0.370 0.383

Locus 11611 :

2 alleles

97.7% missing data

2	(0.372) 0.381 0.352

4	(0.628) 0.619 0.648

Locus 11612 :

2 alleles

98.4% missing data

3	(0.860) 0.861 0.861

4	(0.140) 0.139 0.139

Locus 11613 :

2 alleles

99.2% missing data

3	(0.406) 0.402 0.412

1	(0.594) 0.598 0.588

Locus 11614 :

2 alleles

99.2% missing data

4	(0.403) 0.409 0.395

1	(0.597) 0.591 0.605

Locus 11615 :

2 alleles

99.2% missing data

2	(0.439) 0.441 0.443

1	(0.561) 0.559 0.557

Locus 11616 :

2 alleles

99.2% missing data

4	(0.425) 0.418 0.432

3	(0.575) 0.582 0.568

Locus 11617 :

2 alleles

96.1% missing data

1	(0.347) 0.326 0.354

3	(0.653) 0.674 0.646

Locus 11618 :

2 alleles

98.8% missing data

2	(0.609) 0.608 0.611

3	(0.391) 0.392 0.389

Locus 11619 :

2 alleles

99.2% missing data

3	(0.586) 0.582 0.586

1	(0.414) 0.418 0.414

Locus 11620 :

2 alleles

99.2% missing data

1	(0.696) 0.702 0.692

3	(0.304) 0.298 0.308

Locus 11621 :

2 alleles

97.3% missing data

2	(0.751) 0.753 0.753

1	(0.249) 0.247 0.247

Locus 11622 :

2 alleles

99.2% missing data

4	(0.395) 0.399 0.391

1	(0.605) 0.601 0.609

Locus 11623 :

2 alleles

98.8% missing data

1	(0.540) 0.542 0.539

4	(0.460) 0.458 0.461

Locus 11624 :

2 alleles

99.2% missing data

4	(0.631) 0.631 0.633

2	(0.369) 0.369 0.367

Locus 11625 :

2 alleles

99.2% missing data

4	(0.430) 0.423 0.435

2	(0.570) 0.577 0.565

Locus 11626 :

2 alleles

96.5% missing data

4	(0.631) 0.624 0.645

3	(0.369) 0.376 0.355

Locus 11627 :

2 alleles

99.2% missing data

4	(0.504) 0.512 0.502

3	(0.496) 0.488 0.498

Locus 11628 :

2 alleles

99.2% missing data

4	(0.269) 0.263 0.271

1	(0.731) 0.737 0.729

Locus 11629 :

2 alleles

99.2% missing data

1	(0.536) 0.540 0.528

2	(0.464) 0.460 0.472

Locus 11630 :

2 alleles

99.2% missing data

1	(0.336) 0.335 0.337

3	(0.664) 0.665 0.663

Locus 11631 :

2 alleles

99.2% missing data

2	(0.679) 0.674 0.675

1	(0.321) 0.326 0.325

Locus 11632 :

2 alleles

99.2% missing data

2	(0.683) 0.681 0.688

3	(0.317) 0.319 0.312

Locus 11633 :

2 alleles

99.2% missing data

2	(0.228) 0.231 0.219

1	(0.772) 0.769 0.781

Locus 11634 :

2 alleles

99.2% missing data

1	(0.322) 0.316 0.326

2	(0.678) 0.684 0.674

Locus 11635 :

2 alleles

99.2% missing data

2	(0.231) 0.238 0.228

3	(0.769) 0.762 0.772

Locus 11636 :

2 alleles

99.2% missing data

2	(0.532) 0.532 0.531

1	(0.468) 0.468 0.469

Locus 11637 :

2 alleles

99.2% missing data

1	(0.770) 0.766 0.774

2	(0.230) 0.234 0.226

Locus 11638 :

2 alleles

96.9% missing data

3	(0.320) 0.324 0.313

1	(0.680) 0.676 0.687

Locus 11639 :

2 alleles

98.0% missing data

2	(0.801) 0.803 0.809

3	(0.199) 0.197 0.191

Locus 11640 :

2 alleles

99.2% missing data

4	(0.250) 0.240 0.257

2	(0.750) 0.760 0.743

Locus 11641 :

2 alleles

99.2% missing data

3	(0.543) 0.542 0.549

2	(0.457) 0.458 0.451

Locus 11642 :

3 alleles

98.8% missing data

3	(0.332) 0.330 0.330

4	(0.292) 0.295 0.289

1	(0.377) 0.375 0.381

Locus 11643 :

2 alleles

99.2% missing data

2	(0.213) 0.215 0.214

4	(0.787) 0.785 0.786

Locus 11644 :

2 alleles

95.3% missing data

4	(0.107) 0.106 0.103

2	(0.893) 0.894 0.897

Locus 11645 :

2 alleles

99.2% missing data

1	(0.704) 0.705 0.705

4	(0.296) 0.295 0.295

Locus 11646 :

2 alleles

99.2% missing data

1	(0.493) 0.488 0.492

2	(0.507) 0.512 0.508

Locus 11647 :

2 alleles

97.3% missing data

3	(0.877) 0.871 0.885

2	(0.123) 0.129 0.115

Locus 11648 :

2 alleles

99.2% missing data

2	(0.492) 0.490 0.497

1	(0.508) 0.510 0.503

Locus 11649 :

2 alleles

99.2% missing data

2	(0.159) 0.158 0.163

3	(0.841) 0.842 0.837

Locus 11650 :

3 alleles

98.8% missing data

2	(0.393) 0.391 0.395

3	(0.242) 0.239 0.250

1	(0.364) 0.370 0.355

Locus 11651 :

2 alleles

99.2% missing data

4	(0.334) 0.330 0.332

3	(0.666) 0.670 0.668

Locus 11652 :

2 alleles

99.2% missing data

3	(0.485) 0.486 0.493

1	(0.515) 0.514 0.507

Locus 11653 :

2 alleles

98.8% missing data

4	(0.433) 0.423 0.443

1	(0.567) 0.577 0.557

Locus 11654 :

2 alleles

98.8% missing data

2	(0.669) 0.664 0.671

4	(0.331) 0.336 0.329

Locus 11655 :

2 alleles

99.2% missing data

3	(0.349) 0.354 0.348

1	(0.651) 0.646 0.652

Locus 11656 :

3 alleles

95.7% missing data

1	(0.349) 0.358 0.343

2	(0.173) 0.179 0.169

3	(0.479) 0.464 0.488

Locus 11657 :

2 alleles

99.2% missing data

3	(0.429) 0.424 0.436

1	(0.571) 0.576 0.564

Locus 11658 :

2 alleles

99.2% missing data

1	(0.679) 0.681 0.673

2	(0.321) 0.319 0.327

Locus 11659 :

2 alleles

99.2% missing data

1	(0.359) 0.363 0.352

3	(0.641) 0.637 0.648

Locus 11660 :

2 alleles

99.2% missing data

1	(0.357) 0.356 0.354

4	(0.643) 0.644 0.646

Locus 11661 :

3 alleles

98.4% missing data

3	(0.234) 0.235 0.239

4	(0.540) 0.542 0.537

1	(0.226) 0.222 0.224

Locus 11662 :

2 alleles

99.2% missing data

2	(0.362) 0.362 0.363

1	(0.638) 0.638 0.637

Locus 11663 :

2 alleles

99.2% missing data

1	(0.599) 0.597 0.600

4	(0.401) 0.403 0.400

Locus 11664 :

2 alleles

99.2% missing data

1	(0.580) 0.580 0.579

3	(0.420) 0.420 0.421

Locus 11665 :

2 alleles

99.2% missing data

4	(0.407) 0.404 0.415

2	(0.593) 0.596 0.585

Locus 11666 :

2 alleles

89.5% missing data

2	(0.805) 0.814 0.802

1	(0.195) 0.186 0.198

Locus 11667 :

2 alleles

99.2% missing data

3	(0.438) 0.436 0.436

1	(0.562) 0.564 0.564

Locus 11668 :

2 alleles

98.8% missing data

1	(0.497) 0.491 0.502

2	(0.503) 0.509 0.498

Locus 11669 :

2 alleles

99.2% missing data

1	(0.463) 0.455 0.475

4	(0.537) 0.545 0.525

Locus 11670 :

3 alleles

96.1% missing data

3	(0.710) 0.727 0.698

1	(0.101) 0.092 0.110

2	(0.189) 0.181 0.192

Locus 11671 :

2 alleles

99.2% missing data

3	(0.733) 0.739 0.733

1	(0.267) 0.261 0.267

Locus 11672 :

2 alleles

97.7% missing data

1	(0.213) 0.218 0.205

4	(0.787) 0.782 0.795

Locus 11673 :

2 alleles

99.2% missing data

4	(0.626) 0.633 0.617

1	(0.374) 0.367 0.383

Locus 11674 :

2 alleles

97.3% missing data

4	(0.129) 0.125 0.128

2	(0.871) 0.875 0.872

Locus 11675 :

2 alleles

98.0% missing data

1	(0.725) 0.723 0.726

4	(0.275) 0.277 0.274

Locus 11676 :

3 alleles

96.5% missing data

3	(0.713) 0.714 0.711

1	(0.118) 0.122 0.113

2	(0.169) 0.164 0.176

Locus 11677 :

2 alleles

94.9% missing data

4	(0.120) 0.116 0.122

3	(0.880) 0.884 0.878

Locus 11678 :

2 alleles

99.2% missing data

4	(0.247) 0.240 0.254

1	(0.753) 0.760 0.746

Locus 11679 :

2 alleles

98.0% missing data

3	(0.811) 0.809 0.815

2	(0.189) 0.191 0.185

Locus 11680 :

2 alleles

99.2% missing data

4	(0.491) 0.488 0.496

3	(0.509) 0.512 0.504

Locus 11681 :

2 alleles

98.8% missing data

4	(0.804) 0.805 0.797

1	(0.196) 0.195 0.203

Locus 11682 :

2 alleles

90.2% missing data

3	(0.920) 0.934 0.911

1	(0.080) 0.066 0.089

Locus 11683 :

2 alleles

99.2% missing data

1	(0.522) 0.523 0.519

2	(0.478) 0.477 0.481

Locus 11684 :

2 alleles

98.8% missing data

3	(0.565) 0.561 0.558

1	(0.435) 0.439 0.442

Locus 11685 :

2 alleles

99.2% missing data

2	(0.296) 0.294 0.294

4	(0.704) 0.706 0.706

Locus 11686 :

2 alleles

97.3% missing data

4	(0.260) 0.266 0.254

2	(0.740) 0.734 0.746

Locus 11687 :

2 alleles

99.2% missing data

1	(0.489) 0.490 0.491

4	(0.511) 0.510 0.509

Locus 11688 :

2 alleles

99.2% missing data

1	(0.221) 0.222 0.224

4	(0.779) 0.778 0.776

Locus 11689 :

2 alleles

99.2% missing data

2	(0.575) 0.577 0.572

3	(0.425) 0.423 0.428

Locus 11690 :

2 alleles

99.2% missing data

1	(0.693) 0.689 0.695

3	(0.307) 0.311 0.305

Locus 11691 :

2 alleles

99.2% missing data

1	(0.592) 0.599 0.596

4	(0.408) 0.401 0.404

Locus 11692 :

2 alleles

99.2% missing data

2	(0.361) 0.357 0.359

1	(0.639) 0.643 0.641

Locus 11693 :

2 alleles

8.2% missing data

2	(0.987) 0.981 0.996

3	(0.013) 0.019 0.004

Locus 11694 :

2 alleles

99.2% missing data

4	(0.605) 0.610 0.606

2	(0.395) 0.390 0.394

Locus 11695 :

2 alleles

98.8% missing data

1	(0.638) 0.648 0.640

2	(0.362) 0.352 0.360

Locus 11696 :

2 alleles

99.2% missing data

3	(0.444) 0.444 0.442

2	(0.556) 0.556 0.558

Locus 11697 :

2 alleles

99.2% missing data

1	(0.572) 0.569 0.566

2	(0.428) 0.431 0.434

Locus 11698 :

2 alleles

98.8% missing data

3	(0.453) 0.452 0.461

1	(0.547) 0.548 0.539

Locus 11699 :

2 alleles

99.2% missing data

4	(0.874) 0.882 0.868

3	(0.126) 0.118 0.132

Locus 11700 :

2 alleles

99.2% missing data

2	(0.197) 0.205 0.193

3	(0.803) 0.795 0.807

Locus 11701 :

2 alleles

98.0% missing data

1	(0.156) 0.146 0.162

3	(0.844) 0.854 0.838

Locus 11702 :

2 alleles

98.4% missing data

2	(0.657) 0.663 0.659

3	(0.343) 0.337 0.341

Locus 11703 :

2 alleles

98.4% missing data

3	(0.335) 0.335 0.340

2	(0.665) 0.665 0.660

Locus 11704 :

2 alleles

99.2% missing data

4	(0.753) 0.743 0.755

3	(0.247) 0.257 0.245

Locus 11705 :

2 alleles

95.3% missing data

4	(0.675) 0.672 0.672

1	(0.325) 0.328 0.328

Locus 11706 :

2 alleles

0.0% missing data

2	(0.987) 0.984 0.995

3	(0.013) 0.016 0.005

Locus 11707 :

2 alleles

98.4% missing data

4	(0.334) 0.323 0.335

3	(0.666) 0.677 0.665

Locus 11708 :

2 alleles

99.2% missing data

1	(0.454) 0.453 0.447

3	(0.546) 0.547 0.553

Locus 11709 :

2 alleles

99.2% missing data

4	(0.705) 0.706 0.703

1	(0.295) 0.294 0.297

Locus 11710 :

2 alleles

99.2% missing data

1	(0.345) 0.345 0.345

3	(0.655) 0.655 0.655

Locus 11711 :

2 alleles

99.2% missing data

3	(0.565) 0.561 0.568

4	(0.435) 0.439 0.432

Locus 11712 :

2 alleles

99.2% missing data

4	(0.659) 0.661 0.649

1	(0.341) 0.339 0.351

Locus 11713 :

2 alleles

99.2% missing data

2	(0.380) 0.391 0.372

4	(0.620) 0.609 0.628

Locus 11714 :

2 alleles

99.2% missing data

3	(0.751) 0.747 0.752

2	(0.249) 0.253 0.248

Locus 11715 :

2 alleles

99.2% missing data

1	(0.398) 0.404 0.396

3	(0.602) 0.596 0.604

Locus 11716 :

2 alleles

28.9% missing data

4	(0.982) 0.985 0.989

2	(0.018) 0.015 0.011

Locus 11717 :

2 alleles

99.2% missing data

3	(0.709) 0.708 0.697

4	(0.291) 0.292 0.303

Locus 11718 :

2 alleles

99.2% missing data

2	(0.732) 0.734 0.727

3	(0.268) 0.266 0.273

Locus 11719 :

2 alleles

99.2% missing data

2	(0.284) 0.279 0.287

3	(0.716) 0.721 0.713

Locus 11720 :

2 alleles

99.2% missing data

4	(0.678) 0.673 0.679

2	(0.322) 0.327 0.321

Locus 11721 :

2 alleles

98.8% missing data

1	(0.737) 0.741 0.734

4	(0.263) 0.259 0.266

Locus 11722 :

2 alleles

0.0% missing data

3	(0.984) 0.980 0.995

1	(0.016) 0.020 0.005

Locus 11723 :

2 alleles

99.2% missing data

4	(0.655) 0.645 0.657

2	(0.345) 0.355 0.343

Locus 11724 :

3 alleles

98.8% missing data

1	(0.164) 0.163 0.171

3	(0.453) 0.451 0.454

4	(0.383) 0.386 0.375

Locus 11725 :

2 alleles

99.2% missing data

1	(0.601) 0.595 0.603

4	(0.399) 0.405 0.397

Locus 11726 :

2 alleles

99.2% missing data

3	(0.657) 0.657 0.657

4	(0.343) 0.343 0.343

Locus 11727 :

2 alleles

98.8% missing data

2	(0.306) 0.310 0.313

3	(0.694) 0.690 0.687

Locus 11728 :

2 alleles

98.8% missing data

3	(0.440) 0.441 0.434

1	(0.560) 0.559 0.566

Locus 11729 :

2 alleles

99.2% missing data

4	(0.315) 0.312 0.324

2	(0.685) 0.688 0.676

Locus 11730 :

2 alleles

96.1% missing data

1	(0.713) 0.701 0.737

3	(0.287) 0.299 0.263

Locus 11731 :

2 alleles

97.3% missing data

2	(0.860) 0.860 0.860

4	(0.140) 0.140 0.140

Locus 11732 :

2 alleles

99.2% missing data

1	(0.353) 0.350 0.362

4	(0.647) 0.650 0.638

Locus 11733 :

2 alleles

99.2% missing data

4	(0.347) 0.346 0.350

3	(0.653) 0.654 0.650

Locus 11734 :

2 alleles

99.2% missing data

4	(0.775) 0.776 0.769

1	(0.225) 0.224 0.231

Locus 11735 :

3 alleles

7.8% missing data

2	(0.967) 0.951 0.990

3	(0.019) 0.027 0.006

1	(0.014) 0.022 0.004

Locus 11736 :

2 alleles

99.2% missing data

2	(0.791) 0.785 0.792

1	(0.209) 0.215 0.208

Locus 11737 :

2 alleles

99.2% missing data

2	(0.507) 0.509 0.511

1	(0.493) 0.491 0.489

Locus 11738 :

2 alleles

99.2% missing data

4	(0.515) 0.509 0.514

3	(0.485) 0.491 0.486

Locus 11739 :

2 alleles

99.2% missing data

2	(0.646) 0.649 0.643

1	(0.354) 0.351 0.357

Locus 11740 :

2 alleles

99.2% missing data

1	(0.298) 0.294 0.297

3	(0.702) 0.706 0.703

Locus 11741 :

2 alleles

98.8% missing data

2	(0.702) 0.707 0.701

3	(0.298) 0.293 0.299

Locus 11742 :

2 alleles

97.3% missing data

4	(0.392) 0.396 0.377

1	(0.608) 0.604 0.623

Locus 11743 :

2 alleles

98.0% missing data

4	(0.618) 0.615 0.622

1	(0.382) 0.385 0.378

Locus 11744 :

2 alleles

99.2% missing data

4	(0.791) 0.794 0.785

1	(0.209) 0.206 0.215

Locus 11745 :

2 alleles

0.0% missing data

4	(0.969) 0.977 0.969

3	(0.031) 0.023 0.031

Locus 11746 :

3 alleles

96.1% missing data

2	(0.798) 0.813 0.786

4	(0.083) 0.079 0.088

3	(0.119) 0.108 0.125

Locus 11747 :

2 alleles

99.2% missing data

3	(0.475) 0.474 0.472

4	(0.525) 0.526 0.528

Locus 11748 :

2 alleles

98.8% missing data

4	(0.491) 0.495 0.491

3	(0.509) 0.505 0.509

Locus 11749 :

2 alleles

99.2% missing data

2	(0.373) 0.370 0.375

3	(0.627) 0.630 0.625

Locus 11750 :

2 alleles

99.2% missing data

3	(0.701) 0.697 0.700

1	(0.299) 0.303 0.300

Locus 11751 :

2 alleles

99.2% missing data

3	(0.254) 0.256 0.256

2	(0.746) 0.744 0.744

Locus 11752 :

2 alleles

99.2% missing data

4	(0.348) 0.344 0.350

1	(0.652) 0.656 0.650

Locus 11753 :

2 alleles

97.7% missing data

2	(0.861) 0.852 0.873

1	(0.139) 0.148 0.127

Locus 11754 :

2 alleles

99.2% missing data

4	(0.552) 0.543 0.555

2	(0.448) 0.457 0.445

Locus 11755 :

2 alleles

99.2% missing data

1	(0.446) 0.444 0.447

2	(0.554) 0.556 0.553

Locus 11756 :

2 alleles

99.2% missing data

3	(0.386) 0.390 0.388

1	(0.614) 0.610 0.612

Locus 11757 :

2 alleles

99.2% missing data

1	(0.439) 0.445 0.435

4	(0.561) 0.555 0.565

Locus 11758 :

2 alleles

99.2% missing data

4	(0.752) 0.747 0.757

1	(0.248) 0.253 0.243

Locus 11759 :

2 alleles

97.3% missing data

3	(0.650) 0.646 0.667

1	(0.350) 0.354 0.333

Locus 11760 :

2 alleles

98.8% missing data

1	(0.423) 0.425 0.417

4	(0.577) 0.575 0.583

Locus 11761 :

2 alleles

99.2% missing data

3	(0.244) 0.242 0.249

2	(0.756) 0.758 0.751

Locus 11762 :

2 alleles

0.0% missing data

2	(0.991) 0.994 0.995

3	(0.009) 0.006 0.005

Locus 11763 :

2 alleles

96.1% missing data

1	(0.118) 0.107 0.125

2	(0.882) 0.893 0.875

Locus 11764 :

2 alleles

96.5% missing data

3	(0.794) 0.803 0.788

2	(0.206) 0.197 0.212

Locus 11765 :

2 alleles

99.2% missing data

1	(0.530) 0.537 0.523

3	(0.470) 0.463 0.477

Locus 11766 :

3 alleles

98.0% missing data

3	(0.483) 0.476 0.489

2	(0.356) 0.356 0.358

4	(0.161) 0.169 0.153

Locus 11767 :

2 alleles

96.1% missing data

1	(0.212) 0.208 0.209

3	(0.788) 0.792 0.791

Locus 11768 :

3 alleles

98.8% missing data

3	(0.394) 0.391 0.392

1	(0.350) 0.343 0.354

4	(0.256) 0.266 0.253

Locus 11769 :

2 alleles

99.2% missing data

3	(0.590) 0.592 0.588

1	(0.410) 0.408 0.412

Locus 11770 :

2 alleles

99.2% missing data

4	(0.404) 0.402 0.406

3	(0.596) 0.598 0.594

Locus 11771 :

2 alleles

99.2% missing data

4	(0.641) 0.644 0.635

1	(0.359) 0.356 0.365

Locus 11772 :

2 alleles

99.2% missing data

3	(0.560) 0.563 0.560

2	(0.440) 0.437 0.440

Locus 11773 :

2 alleles

98.8% missing data

3	(0.220) 0.219 0.223

1	(0.780) 0.781 0.777

Locus 11774 :

2 alleles

99.2% missing data

1	(0.386) 0.380 0.386

4	(0.614) 0.620 0.614

Locus 11775 :

2 alleles

99.2% missing data

1	(0.114) 0.109 0.121

3	(0.886) 0.891 0.879

Locus 11776 :

2 alleles

94.5% missing data

4	(0.835) 0.829 0.841

2	(0.165) 0.171 0.159

Locus 11777 :

2 alleles

97.7% missing data

2	(0.206) 0.211 0.198

4	(0.794) 0.789 0.802

Locus 11778 :

2 alleles

99.2% missing data

3	(0.480) 0.485 0.480

1	(0.520) 0.515 0.520

Locus 11779 :

2 alleles

99.2% missing data

4	(0.718) 0.720 0.717

3	(0.282) 0.280 0.283

Locus 11780 :

2 alleles

98.0% missing data

4	(0.770) 0.776 0.764

3	(0.230) 0.224 0.236

Locus 11781 :

2 alleles

98.8% missing data

1	(0.753) 0.744 0.761

2	(0.247) 0.256 0.239

Locus 11782 :

2 alleles

99.2% missing data

3	(0.276) 0.283 0.272

1	(0.724) 0.717 0.728

Locus 11783 :

2 alleles

99.2% missing data

3	(0.573) 0.575 0.574

4	(0.427) 0.425 0.426

Locus 11784 :

2 alleles

99.2% missing data

1	(0.183) 0.189 0.184

3	(0.817) 0.811 0.816

Locus 11785 :

2 alleles

99.2% missing data

1	(0.540) 0.550 0.532

3	(0.460) 0.450 0.468

Locus 11786 :

2 alleles

99.2% missing data

2	(0.482) 0.484 0.474

4	(0.518) 0.516 0.526

Locus 11787 :

2 alleles

99.2% missing data

1	(0.481) 0.488 0.477

3	(0.519) 0.512 0.523

Locus 11788 :

2 alleles

99.2% missing data

1	(0.304) 0.309 0.299

4	(0.696) 0.691 0.701

Locus 11789 :

2 alleles

99.2% missing data

4	(0.384) 0.378 0.391

3	(0.616) 0.622 0.609

Locus 11790 :

2 alleles

96.5% missing data

1	(0.770) 0.762 0.784

4	(0.230) 0.238 0.216

Locus 11791 :

2 alleles

99.2% missing data

4	(0.178) 0.178 0.179

1	(0.822) 0.822 0.821

Locus 11792 :

2 alleles

99.2% missing data

4	(0.585) 0.584 0.583

2	(0.415) 0.416 0.417

Locus 11793 :

2 alleles

98.4% missing data

4	(0.751) 0.749 0.755

1	(0.249) 0.251 0.245

Locus 11794 :

2 alleles

97.3% missing data

3	(0.826) 0.820 0.837

1	(0.174) 0.180 0.163

Locus 11795 :

2 alleles

98.8% missing data

4	(0.724) 0.733 0.716

2	(0.276) 0.267 0.284

Locus 11796 :

2 alleles

99.2% missing data

3	(0.401) 0.411 0.395

1	(0.599) 0.589 0.605

Locus 11797 :

2 alleles

95.7% missing data

3	(0.244) 0.228 0.254

2	(0.756) 0.772 0.746

Locus 11798 :

2 alleles

89.8% missing data

3	(0.915) 0.912 0.928

1	(0.085) 0.088 0.072

Locus 11799 :

2 alleles

99.2% missing data

4	(0.198) 0.192 0.204

3	(0.802) 0.808 0.796

Locus 11800 :

2 alleles

99.2% missing data

1	(0.550) 0.552 0.548

4	(0.450) 0.448 0.452

Locus 11801 :

2 alleles

99.2% missing data

1	(0.682) 0.685 0.683

4	(0.318) 0.315 0.317

Locus 11802 :

2 alleles

99.2% missing data

4	(0.579) 0.575 0.584

2	(0.421) 0.425 0.416

Locus 11803 :

2 alleles

99.2% missing data

4	(0.357) 0.352 0.359

3	(0.643) 0.648 0.641

Locus 11804 :

2 alleles

99.2% missing data

2	(0.504) 0.492 0.507

3	(0.496) 0.508 0.493

Locus 11805 :

2 alleles

93.0% missing data

4	(0.865) 0.864 0.876

1	(0.135) 0.136 0.124

Locus 11806 :

2 alleles

99.2% missing data

3	(0.388) 0.388 0.390

1	(0.612) 0.612 0.610

Locus 11807 :

2 alleles

99.2% missing data

1	(0.629) 0.630 0.630

2	(0.371) 0.370 0.370

Locus 11808 :

2 alleles

99.2% missing data

3	(0.663) 0.665 0.669

2	(0.337) 0.335 0.331

Locus 11809 :

2 alleles

98.8% missing data

1	(0.573) 0.564 0.578

3	(0.427) 0.436 0.422

Locus 11810 :

2 alleles

99.2% missing data

1	(0.659) 0.651 0.661

2	(0.341) 0.349 0.339

Locus 11811 :

2 alleles

99.2% missing data

2	(0.297) 0.293 0.300

1	(0.703) 0.707 0.700

Locus 11812 :

2 alleles

99.2% missing data

2	(0.404) 0.403 0.402

4	(0.596) 0.597 0.598

Locus 11813 :

2 alleles

97.3% missing data

4	(0.868) 0.879 0.864

1	(0.132) 0.121 0.136

Locus 11814 :

2 alleles

99.2% missing data

4	(0.532) 0.526 0.534

3	(0.468) 0.474 0.466

Locus 11815 :

2 alleles

99.2% missing data

4	(0.748) 0.738 0.751

1	(0.252) 0.262 0.249

Locus 11816 :

2 alleles

99.2% missing data

2	(0.282) 0.280 0.288

1	(0.718) 0.720 0.712

Locus 11817 :

2 alleles

98.4% missing data

4	(0.524) 0.521 0.534

1	(0.476) 0.479 0.466

Locus 11818 :

2 alleles

99.2% missing data

2	(0.290) 0.295 0.288

4	(0.710) 0.705 0.712

Locus 11819 :

2 alleles

96.9% missing data

1	(0.795) 0.785 0.811

2	(0.205) 0.215 0.189

Locus 11820 :

2 alleles

99.2% missing data

4	(0.212) 0.209 0.219

3	(0.788) 0.791 0.781

Locus 11821 :

2 alleles

99.2% missing data

2	(0.540) 0.536 0.543

4	(0.460) 0.464 0.457

Locus 11822 :

2 alleles

96.1% missing data

3	(0.176) 0.162 0.177

2	(0.824) 0.838 0.823

Locus 11823 :

2 alleles

99.2% missing data

3	(0.474) 0.480 0.480

1	(0.526) 0.520 0.520

Locus 11824 :

2 alleles

91.0% missing data

3	(0.917) 0.926 0.916

4	(0.083) 0.074 0.084

Locus 11825 :

2 alleles

99.2% missing data

1	(0.737) 0.732 0.744

4	(0.263) 0.268 0.256

Locus 11826 :

2 alleles

99.2% missing data

4	(0.772) 0.772 0.768

3	(0.228) 0.228 0.232

Locus 11827 :

2 alleles

99.2% missing data

4	(0.704) 0.700 0.703

3	(0.296) 0.300 0.297

Locus 11828 :

2 alleles

99.2% missing data

1	(0.451) 0.453 0.449

4	(0.549) 0.547 0.551

Locus 11829 :

2 alleles

99.2% missing data

4	(0.621) 0.625 0.617

1	(0.379) 0.375 0.383

Locus 11830 :

2 alleles

98.8% missing data

4	(0.369) 0.356 0.371

3	(0.631) 0.644 0.629

Locus 11831 :

2 alleles

99.2% missing data

1	(0.710) 0.707 0.713

4	(0.290) 0.293 0.287

Locus 11832 :

2 alleles

99.2% missing data

3	(0.280) 0.278 0.286

1	(0.720) 0.722 0.714

Locus 11833 :

2 alleles

98.8% missing data

1	(0.486) 0.487 0.489

2	(0.514) 0.513 0.511

Locus 11834 :

2 alleles

99.2% missing data

4	(0.822) 0.824 0.815

3	(0.178) 0.176 0.185

Locus 11835 :

2 alleles

0.0% missing data

3	(0.990) 0.989 0.995

2	(0.010) 0.011 0.005

Locus 11836 :

2 alleles

98.8% missing data

4	(0.431) 0.429 0.433

2	(0.569) 0.571 0.567

Locus 11837 :

2 alleles

99.2% missing data

4	(0.674) 0.673 0.673

2	(0.326) 0.327 0.327

Locus 11838 :

2 alleles

98.0% missing data

4	(0.750) 0.742 0.762

1	(0.250) 0.258 0.238

Locus 11839 :

2 alleles

90.6% missing data

2	(0.903) 0.923 0.893

3	(0.097) 0.077 0.107

Locus 11840 :

2 alleles

98.8% missing data

1	(0.357) 0.360 0.354

2	(0.643) 0.640 0.646

Locus 11841 :

2 alleles

93.0% missing data

1	(0.904) 0.903 0.908

4	(0.096) 0.097 0.092

Locus 11842 :

2 alleles

98.0% missing data

4	(0.828) 0.821 0.833

2	(0.172) 0.179 0.167

Locus 11843 :

2 alleles

98.0% missing data

1	(0.853) 0.854 0.853

3	(0.147) 0.146 0.147

Locus 11844 :

2 alleles

99.2% missing data

2	(0.594) 0.591 0.600

4	(0.406) 0.409 0.400

Locus 11845 :

2 alleles

99.2% missing data

2	(0.725) 0.728 0.721

4	(0.275) 0.272 0.279

Locus 11846 :

2 alleles

99.2% missing data

1	(0.483) 0.488 0.472

2	(0.517) 0.512 0.528

Locus 11847 :

2 alleles

99.2% missing data

3	(0.675) 0.671 0.683

2	(0.325) 0.329 0.317

Locus 11848 :

2 alleles

8.2% missing data

2	(0.981) 0.972 0.994

3	(0.019) 0.028 0.006

Locus 11849 :

3 alleles

98.0% missing data

4	(0.605) 0.608 0.604

2	(0.182) 0.180 0.183

3	(0.212) 0.212 0.213

Locus 11850 :

2 alleles

98.0% missing data

2	(0.646) 0.661 0.641

3	(0.354) 0.339 0.359

Locus 11851 :

2 alleles

8.2% missing data

2	(0.980) 0.973 0.993

4	(0.020) 0.027 0.007

Locus 11852 :

2 alleles

99.2% missing data

2	(0.400) 0.402 0.401

1	(0.600) 0.598 0.599

Locus 11853 :

2 alleles

99.2% missing data

4	(0.428) 0.429 0.421

1	(0.572) 0.571 0.579

Locus 11854 :

2 alleles

98.4% missing data

2	(0.473) 0.485 0.465

3	(0.527) 0.515 0.535

Locus 11855 :

2 alleles

99.2% missing data

2	(0.570) 0.563 0.581

4	(0.430) 0.437 0.419

Locus 11856 :

3 alleles

98.4% missing data

2	(0.286) 0.286 0.283

3	(0.312) 0.308 0.321

1	(0.402) 0.407 0.396

Locus 11857 :

2 alleles

99.2% missing data

1	(0.530) 0.525 0.526

2	(0.470) 0.475 0.474

Locus 11858 :

2 alleles

98.8% missing data

3	(0.706) 0.712 0.700

4	(0.294) 0.288 0.300

Locus 11859 :

2 alleles

99.2% missing data

3	(0.620) 0.622 0.627

4	(0.380) 0.378 0.373

Locus 11860 :

2 alleles

99.2% missing data

4	(0.437) 0.438 0.434

3	(0.563) 0.562 0.566

Locus 11861 :

2 alleles

99.2% missing data

1	(0.337) 0.332 0.343

2	(0.663) 0.668 0.657

Locus 11862 :

2 alleles

98.8% missing data

1	(0.690) 0.688 0.695

4	(0.310) 0.312 0.305

Locus 11863 :

2 alleles

91.0% missing data

3	(0.917) 0.932 0.906

2	(0.083) 0.068 0.094

Locus 11864 :

2 alleles

99.2% missing data

4	(0.321) 0.312 0.328

3	(0.679) 0.688 0.672

Locus 11865 :

2 alleles

99.2% missing data

4	(0.419) 0.420 0.420

2	(0.581) 0.580 0.580

Locus 11866 :

2 alleles

99.2% missing data

1	(0.433) 0.431 0.433

2	(0.567) 0.569 0.567

Locus 11867 :

2 alleles

98.4% missing data

4	(0.869) 0.866 0.868

3	(0.131) 0.134 0.132

Locus 11868 :

2 alleles

92.6% missing data

3	(0.881) 0.863 0.898

4	(0.119) 0.137 0.102

Locus 11869 :

3 alleles

98.0% missing data

4	(0.410) 0.421 0.407

2	(0.270) 0.260 0.277

1	(0.321) 0.319 0.316

Locus 11870 :

2 alleles

99.2% missing data

4	(0.369) 0.373 0.359

3	(0.631) 0.627 0.641

Locus 11871 :

2 alleles

99.2% missing data

2	(0.473) 0.477 0.471

3	(0.527) 0.523 0.529

Locus 11872 :

2 alleles

99.2% missing data

3	(0.558) 0.552 0.560

1	(0.442) 0.448 0.440

Locus 11873 :

2 alleles

99.2% missing data

2	(0.692) 0.688 0.693

1	(0.308) 0.312 0.307

Locus 11874 :

2 alleles

98.0% missing data

2	(0.534) 0.539 0.528

4	(0.466) 0.461 0.472

Locus 11875 :

2 alleles

98.8% missing data

4	(0.497) 0.496 0.499

1	(0.503) 0.504 0.501

Locus 11876 :

2 alleles

99.2% missing data

4	(0.497) 0.499 0.498

3	(0.503) 0.501 0.502

Locus 11877 :

2 alleles

97.7% missing data

3	(0.194) 0.198 0.190

4	(0.806) 0.802 0.810

Locus 11878 :

2 alleles

99.2% missing data

4	(0.546) 0.545 0.551

3	(0.454) 0.455 0.449

Locus 11879 :

2 alleles

99.2% missing data

4	(0.509) 0.507 0.518

3	(0.491) 0.493 0.482

Locus 11880 :

2 alleles

95.7% missing data

2	(0.309) 0.319 0.295

3	(0.691) 0.681 0.705

Locus 11881 :

2 alleles

99.2% missing data

1	(0.739) 0.737 0.739

2	(0.261) 0.263 0.261

Locus 11882 :

2 alleles

0.4% missing data

2	(0.969) 0.925 0.990

1	(0.031) 0.075 0.010

Locus 11883 :

2 alleles

99.2% missing data

3	(0.238) 0.234 0.244

2	(0.762) 0.766 0.756

Locus 11884 :

2 alleles

99.2% missing data

2	(0.480) 0.486 0.482

3	(0.520) 0.514 0.518

Locus 11885 :

2 alleles

99.2% missing data

2	(0.540) 0.540 0.535

4	(0.460) 0.460 0.465

Locus 11886 :

2 alleles

99.2% missing data

4	(0.678) 0.681 0.675

2	(0.322) 0.319 0.325

Locus 11887 :

2 alleles

99.2% missing data

2	(0.539) 0.526 0.547

1	(0.461) 0.474 0.453

Locus 11888 :

2 alleles

99.2% missing data

4	(0.450) 0.444 0.449

3	(0.550) 0.556 0.551

Locus 11889 :

2 alleles

99.2% missing data

4	(0.581) 0.582 0.580

3	(0.419) 0.418 0.420

Locus 11890 :

2 alleles

97.3% missing data

4	(0.759) 0.764 0.764

2	(0.241) 0.236 0.236

Locus 11891 :

2 alleles

95.3% missing data

4	(0.780) 0.786 0.781

1	(0.220) 0.214 0.219

Locus 11892 :

2 alleles

99.2% missing data

1	(0.414) 0.410 0.411

3	(0.586) 0.590 0.589

Locus 11893 :

2 alleles

99.2% missing data

4	(0.267) 0.271 0.264

2	(0.733) 0.729 0.736

Locus 11894 :

2 alleles

99.2% missing data

4	(0.167) 0.164 0.167

3	(0.833) 0.836 0.833

Locus 11895 :

2 alleles

99.2% missing data

1	(0.521) 0.518 0.521

3	(0.479) 0.482 0.479

Locus 11896 :

2 alleles

86.7% missing data

4	(0.944) 0.944 0.951

1	(0.056) 0.056 0.049

Locus 11897 :

2 alleles

71.1% missing data

3	(0.971) 0.978 0.974

1	(0.029) 0.022 0.026

Locus 11898 :

2 alleles

99.2% missing data

3	(0.246) 0.246 0.255

4	(0.754) 0.754 0.745

Locus 11899 :

2 alleles

99.2% missing data

1	(0.515) 0.517 0.513

4	(0.485) 0.483 0.487

Locus 11900 :

2 alleles

98.8% missing data

3	(0.473) 0.466 0.477

2	(0.527) 0.534 0.523

Locus 11901 :

2 alleles

99.2% missing data

3	(0.569) 0.569 0.569

2	(0.431) 0.431 0.431

Locus 11902 :

2 alleles

99.2% missing data

3	(0.239) 0.239 0.239

2	(0.761) 0.761 0.761

Locus 11903 :

2 alleles

99.2% missing data

2	(0.550) 0.553 0.555

4	(0.450) 0.447 0.445

Locus 11904 :

2 alleles

99.2% missing data

2	(0.487) 0.475 0.489

3	(0.513) 0.525 0.511

Locus 11905 :

2 alleles

99.2% missing data

1	(0.758) 0.758 0.752

3	(0.242) 0.242 0.248

Locus 11906 :

3 alleles

98.0% missing data

4	(0.271) 0.264 0.276

1	(0.204) 0.202 0.203

3	(0.525) 0.534 0.521

Locus 11907 :

2 alleles

99.2% missing data

2	(0.577) 0.575 0.583

4	(0.423) 0.425 0.417

Locus 11908 :

2 alleles

99.2% missing data

1	(0.411) 0.413 0.409

4	(0.589) 0.587 0.591

Locus 11909 :

2 alleles

99.2% missing data

1	(0.257) 0.264 0.255

2	(0.743) 0.736 0.745

Locus 11910 :

2 alleles

98.8% missing data

3	(0.516) 0.525 0.515

1	(0.484) 0.475 0.485

Locus 11911 :

2 alleles

99.2% missing data

3	(0.333) 0.330 0.335

4	(0.667) 0.670 0.665

Locus 11912 :

2 alleles

98.8% missing data

3	(0.656) 0.653 0.657

2	(0.344) 0.347 0.343

Locus 11913 :

2 alleles

99.2% missing data

3	(0.631) 0.633 0.634

2	(0.369) 0.367 0.366

Locus 11914 :

2 alleles

99.2% missing data

2	(0.357) 0.351 0.360

1	(0.643) 0.649 0.640

Locus 11915 :

2 alleles

90.6% missing data

3	(0.911) 0.927 0.906

2	(0.089) 0.073 0.094

Locus 11916 :

2 alleles

98.0% missing data

3	(0.256) 0.255 0.255

4	(0.744) 0.745 0.745

Locus 11917 :

2 alleles

99.2% missing data

3	(0.492) 0.490 0.494

1	(0.508) 0.510 0.506

Locus 11918 :

3 alleles

92.2% missing data

1	(0.798) 0.799 0.821

4	(0.122) 0.119 0.113

2	(0.080) 0.082 0.066

Locus 11919 :

2 alleles

95.3% missing data

1	(0.883) 0.883 0.886

4	(0.117) 0.117 0.114

Locus 11920 :

2 alleles

96.9% missing data

3	(0.305) 0.310 0.298

2	(0.695) 0.690 0.702

Locus 11921 :

2 alleles

97.3% missing data

1	(0.664) 0.655 0.670

3	(0.336) 0.345 0.330

Locus 11922 :

2 alleles

99.2% missing data

1	(0.254) 0.252 0.253

2	(0.746) 0.748 0.747

Locus 11923 :

2 alleles

99.2% missing data

1	(0.252) 0.255 0.254

3	(0.748) 0.745 0.746

Locus 11924 :

2 alleles

99.2% missing data

4	(0.461) 0.452 0.469

2	(0.539) 0.548 0.531

Locus 11925 :

2 alleles

98.4% missing data

1	(0.537) 0.536 0.545

3	(0.463) 0.464 0.455

Locus 11926 :

2 alleles

98.8% missing data

1	(0.504) 0.506 0.508

3	(0.496) 0.494 0.492

Locus 11927 :

2 alleles

98.4% missing data

3	(0.503) 0.492 0.521

1	(0.497) 0.508 0.479

Locus 11928 :

2 alleles

99.2% missing data

3	(0.240) 0.243 0.242

1	(0.760) 0.757 0.758

Locus 11929 :

2 alleles

97.7% missing data

1	(0.706) 0.707 0.712

2	(0.294) 0.293 0.288

Locus 11930 :

2 alleles

91.8% missing data

3	(0.620) 0.578 0.655

1	(0.380) 0.422 0.345

Locus 11931 :

2 alleles

99.2% missing data

3	(0.461) 0.463 0.462

2	(0.539) 0.537 0.538

Locus 11932 :

2 alleles

97.7% missing data

1	(0.726) 0.734 0.724

3	(0.274) 0.266 0.276

Locus 11933 :

2 alleles

99.2% missing data

1	(0.394) 0.386 0.396

3	(0.606) 0.614 0.604

Locus 11934 :

2 alleles

98.8% missing data

3	(0.501) 0.495 0.503

1	(0.499) 0.505 0.497

Locus 11935 :

2 alleles

95.3% missing data

1	(0.804) 0.804 0.810

3	(0.196) 0.196 0.190

Locus 11936 :

2 alleles

98.8% missing data

2	(0.460) 0.456 0.459

1	(0.540) 0.544 0.541

Locus 11937 :

2 alleles

96.1% missing data

4	(0.890) 0.898 0.886

1	(0.110) 0.102 0.114

Locus 11938 :

2 alleles

99.2% missing data

2	(0.215) 0.211 0.217

3	(0.785) 0.789 0.783

Locus 11939 :

2 alleles

99.2% missing data

1	(0.453) 0.457 0.454

2	(0.547) 0.543 0.546

Locus 11940 :

3 alleles

90.6% missing data

1	(0.838) 0.866 0.820

2	(0.062) 0.050 0.071

4	(0.100) 0.083 0.109

Locus 11941 :

2 alleles

99.2% missing data

2	(0.426) 0.423 0.431

1	(0.574) 0.577 0.569

Locus 11942 :

2 alleles

99.2% missing data

2	(0.261) 0.262 0.266

1	(0.739) 0.738 0.734

Locus 11943 :

2 alleles

99.2% missing data

3	(0.225) 0.234 0.217

1	(0.775) 0.766 0.783

Locus 11944 :

2 alleles

99.2% missing data

1	(0.469) 0.464 0.465

2	(0.531) 0.536 0.535

Locus 11945 :

2 alleles

99.2% missing data

3	(0.329) 0.328 0.328

4	(0.671) 0.672 0.672

Locus 11946 :

2 alleles

91.4% missing data

3	(0.883) 0.904 0.870

1	(0.117) 0.096 0.130

Locus 11947 :

2 alleles

99.2% missing data

2	(0.526) 0.526 0.538

3	(0.474) 0.474 0.462

Locus 11948 :

2 alleles

99.2% missing data

2	(0.381) 0.385 0.380

3	(0.619) 0.615 0.620

Locus 11949 :

2 alleles

94.5% missing data

3	(0.672) 0.695 0.644

1	(0.328) 0.305 0.356

Locus 11950 :

2 alleles

98.8% missing data

2	(0.723) 0.720 0.728

3	(0.277) 0.280 0.272

Locus 11951 :

2 alleles

99.2% missing data

1	(0.796) 0.799 0.795

2	(0.204) 0.201 0.205

Locus 11952 :

3 alleles

88.3% missing data

1	(0.778) 0.820 0.747

3	(0.079) 0.064 0.086

4	(0.143) 0.117 0.167

Locus 11953 :

2 alleles

99.2% missing data

1	(0.197) 0.193 0.200

2	(0.803) 0.807 0.800

Locus 11954 :

2 alleles

99.2% missing data

1	(0.678) 0.685 0.672

3	(0.322) 0.315 0.328

Locus 11955 :

2 alleles

99.2% missing data

1	(0.443) 0.453 0.439

3	(0.557) 0.547 0.561

Locus 11956 :

2 alleles

99.2% missing data

4	(0.240) 0.231 0.245

2	(0.760) 0.769 0.755

Locus 11957 :

2 alleles

98.0% missing data

2	(0.257) 0.255 0.259

4	(0.743) 0.745 0.741

Locus 11958 :

2 alleles

99.2% missing data

3	(0.762) 0.755 0.765

2	(0.238) 0.245 0.235

Locus 11959 :

2 alleles

99.2% missing data

2	(0.372) 0.376 0.369

3	(0.628) 0.624 0.631

Locus 11960 :

2 alleles

95.3% missing data

2	(0.566) 0.580 0.548

1	(0.434) 0.420 0.452

Locus 11961 :

2 alleles

99.2% missing data

1	(0.806) 0.802 0.810

3	(0.194) 0.198 0.190

Locus 11962 :

2 alleles

98.8% missing data

1	(0.618) 0.606 0.627

3	(0.382) 0.394 0.373

Locus 11963 :

2 alleles

98.8% missing data

4	(0.641) 0.640 0.647

1	(0.359) 0.360 0.353

Locus 11964 :

2 alleles

98.4% missing data

3	(0.370) 0.369 0.370

2	(0.630) 0.631 0.630

Locus 11965 :

2 alleles

99.2% missing data

4	(0.262) 0.264 0.265

3	(0.738) 0.736 0.735

Locus 11966 :

2 alleles

99.2% missing data

4	(0.660) 0.654 0.661

1	(0.340) 0.346 0.339

Locus 11967 :

2 alleles

98.0% missing data

1	(0.399) 0.404 0.396

4	(0.601) 0.596 0.604

Locus 11968 :

2 alleles

97.7% missing data

3	(0.834) 0.823 0.840

1	(0.166) 0.177 0.160

Locus 11969 :

2 alleles

99.2% missing data

4	(0.423) 0.416 0.428

1	(0.577) 0.584 0.572

Locus 11970 :

2 alleles

99.2% missing data

1	(0.294) 0.298 0.298

4	(0.706) 0.702 0.702

Locus 11971 :

2 alleles

99.2% missing data

2	(0.654) 0.648 0.659

3	(0.346) 0.352 0.341

Locus 11972 :

2 alleles

99.2% missing data

2	(0.746) 0.743 0.749

1	(0.254) 0.257 0.251

Locus 11973 :

2 alleles

83.6% missing data

3	(0.964) 0.966 0.968

4	(0.036) 0.034 0.032

Locus 11974 :

2 alleles

99.2% missing data

3	(0.648) 0.645 0.653

2	(0.352) 0.355 0.347

Locus 11975 :

2 alleles

99.2% missing data

3	(0.366) 0.362 0.370

1	(0.634) 0.638 0.630

Locus 11976 :

2 alleles

91.0% missing data

2	(0.907) 0.917 0.903

3	(0.093) 0.083 0.097

Locus 11977 :

2 alleles

97.7% missing data

3	(0.236) 0.245 0.228

1	(0.764) 0.755 0.772

Locus 11978 :

2 alleles

99.2% missing data

2	(0.294) 0.292 0.303

1	(0.706) 0.708 0.697

Locus 11979 :

3 alleles

96.9% missing data

2	(0.249) 0.251 0.245

1	(0.215) 0.218 0.211

3	(0.536) 0.531 0.544

Locus 11980 :

2 alleles

98.4% missing data

4	(0.562) 0.577 0.553

2	(0.438) 0.423 0.447

Locus 11981 :

3 alleles

98.8% missing data

1	(0.304) 0.307 0.309

3	(0.230) 0.228 0.234

4	(0.467) 0.466 0.457

Locus 11982 :

2 alleles

99.2% missing data

4	(0.406) 0.404 0.411

3	(0.594) 0.596 0.589

Locus 11983 :

2 alleles

96.1% missing data

4	(0.252) 0.239 0.255

1	(0.748) 0.761 0.745

Locus 11984 :

2 alleles

96.1% missing data

1	(0.194) 0.181 0.203

3	(0.806) 0.819 0.797

Locus 11985 :

2 alleles

99.2% missing data

3	(0.658) 0.664 0.656

2	(0.342) 0.336 0.344

Locus 11986 :

2 alleles

98.8% missing data

2	(0.588) 0.585 0.587

3	(0.412) 0.415 0.413

Locus 11987 :

2 alleles

98.4% missing data

3	(0.564) 0.573 0.561

4	(0.436) 0.427 0.439

Locus 11988 :

2 alleles

93.0% missing data

2	(0.814) 0.813 0.836

1	(0.186) 0.187 0.164

Locus 11989 :

2 alleles

96.5% missing data

1	(0.687) 0.707 0.675

2	(0.313) 0.293 0.325

Locus 11990 :

2 alleles

99.2% missing data

1	(0.758) 0.761 0.753

3	(0.242) 0.239 0.247

Locus 11991 :

3 alleles

95.7% missing data

3	(0.182) 0.166 0.180

1	(0.682) 0.707 0.677

4	(0.136) 0.127 0.143

Locus 11992 :

2 alleles

98.0% missing data

4	(0.256) 0.254 0.254

2	(0.744) 0.746 0.746

Locus 11993 :

2 alleles

99.2% missing data

3	(0.615) 0.610 0.611

1	(0.385) 0.390 0.389

Locus 11994 :

2 alleles

99.2% missing data

3	(0.701) 0.700 0.698

2	(0.299) 0.300 0.302

Locus 11995 :

2 alleles

98.0% missing data

4	(0.806) 0.807 0.804

3	(0.194) 0.193 0.196

Locus 11996 :

2 alleles

99.2% missing data

3	(0.525) 0.523 0.520

2	(0.475) 0.477 0.480

Locus 11997 :

2 alleles

78.1% missing data

2	(0.950) 0.953 0.952

1	(0.050) 0.047 0.048

Locus 11998 :

4 alleles

98.0% missing data

4	(0.244) 0.251 0.244

3	(0.278) 0.274 0.280

1	(0.243) 0.240 0.247

2	(0.235) 0.235 0.230

Locus 11999 :

2 alleles

96.1% missing data

3	(0.824) 0.830 0.826

1	(0.176) 0.170 0.174

Locus 12000 :

2 alleles

99.2% missing data

2	(0.500) 0.499 0.499

4	(0.500) 0.501 0.501

Locus 12001 :

2 alleles

96.5% missing data

3	(0.739) 0.744 0.749

2	(0.261) 0.256 0.251

Locus 12002 :

2 alleles

99.2% missing data

1	(0.524) 0.518 0.525

3	(0.476) 0.482 0.475

Locus 12003 :

2 alleles

99.2% missing data

4	(0.319) 0.316 0.327

1	(0.681) 0.684 0.673

Locus 12004 :

2 alleles

97.7% missing data

2	(0.669) 0.676 0.670

3	(0.331) 0.324 0.330

Locus 12005 :

2 alleles

99.2% missing data

3	(0.592) 0.592 0.593

4	(0.408) 0.408 0.407

Locus 12006 :

2 alleles

99.2% missing data

1	(0.559) 0.556 0.567

4	(0.441) 0.444 0.433

Locus 12007 :

2 alleles

89.1% missing data

3	(0.745) 0.791 0.702

1	(0.255) 0.209 0.298

Locus 12008 :

2 alleles

99.2% missing data

3	(0.662) 0.657 0.662

1	(0.338) 0.343 0.338

Locus 12009 :

2 alleles

96.1% missing data

3	(0.348) 0.352 0.343

2	(0.652) 0.648 0.657

Locus 12010 :

2 alleles

99.2% missing data

1	(0.525) 0.517 0.529

3	(0.475) 0.483 0.471

Locus 12011 :

2 alleles

99.2% missing data

1	(0.367) 0.368 0.372

3	(0.633) 0.632 0.628

Locus 12012 :

2 alleles

99.2% missing data

2	(0.720) 0.721 0.725

3	(0.280) 0.279 0.275

Locus 12013 :

2 alleles

99.2% missing data

4	(0.386) 0.390 0.387

1	(0.614) 0.610 0.613

Locus 12014 :

2 alleles

99.2% missing data

1	(0.237) 0.236 0.244

2	(0.763) 0.764 0.756

Locus 12015 :

2 alleles

99.2% missing data

1	(0.356) 0.362 0.347

3	(0.644) 0.638 0.653

Locus 12016 :

2 alleles

99.2% missing data

2	(0.625) 0.627 0.627

1	(0.375) 0.373 0.373

Locus 12017 :

2 alleles

99.2% missing data

4	(0.401) 0.401 0.398

2	(0.599) 0.599 0.602

Locus 12018 :

2 alleles

99.2% missing data

4	(0.536) 0.540 0.535

1	(0.464) 0.460 0.465

Locus 12019 :

3 alleles

98.0% missing data

1	(0.424) 0.428 0.427

3	(0.140) 0.139 0.142

2	(0.436) 0.434 0.430

Locus 12020 :

2 alleles

99.2% missing data

1	(0.416) 0.420 0.412

2	(0.584) 0.580 0.588

Locus 12021 :

2 alleles

99.2% missing data

4	(0.579) 0.572 0.583

2	(0.421) 0.428 0.417

Locus 12022 :

2 alleles

96.9% missing data

4	(0.260) 0.267 0.244

2	(0.740) 0.733 0.756

Locus 12023 :

2 alleles

98.4% missing data

1	(0.896) 0.897 0.895

3	(0.104) 0.103 0.105

Locus 12024 :

2 alleles

94.5% missing data

4	(0.609) 0.638 0.581

3	(0.391) 0.362 0.419

Locus 12025 :

2 alleles

98.8% missing data

1	(0.275) 0.269 0.279

4	(0.725) 0.731 0.721

Locus 12026 :

2 alleles

98.4% missing data

2	(0.545) 0.546 0.542

4	(0.455) 0.454 0.458

Locus 12027 :

2 alleles

97.3% missing data

4	(0.648) 0.652 0.639

1	(0.352) 0.348 0.361

Locus 12028 :

2 alleles

99.2% missing data

4	(0.534) 0.537 0.535

2	(0.466) 0.463 0.465

Locus 12029 :

2 alleles

99.2% missing data

2	(0.477) 0.474 0.481

3	(0.523) 0.526 0.519

Locus 12030 :

2 alleles

99.2% missing data

1	(0.388) 0.384 0.392

2	(0.612) 0.616 0.608

Locus 12031 :

2 alleles

97.7% missing data

4	(0.249) 0.251 0.241

2	(0.751) 0.749 0.759

Locus 12032 :

2 alleles

97.7% missing data

3	(0.257) 0.245 0.259

1	(0.743) 0.755 0.741

Locus 12033 :

2 alleles

99.2% missing data

3	(0.421) 0.420 0.418

4	(0.579) 0.580 0.582

Locus 12034 :

2 alleles

98.0% missing data

3	(0.493) 0.494 0.499

1	(0.507) 0.506 0.501

Locus 12035 :

2 alleles

97.3% missing data

4	(0.367) 0.350 0.379

2	(0.633) 0.650 0.621

Locus 12036 :

2 alleles

99.2% missing data

3	(0.244) 0.239 0.250

1	(0.756) 0.761 0.750

Locus 12037 :

2 alleles

98.0% missing data

4	(0.378) 0.382 0.361

1	(0.622) 0.618 0.639

Locus 12038 :

3 alleles

87.5% missing data

4	(0.580) 0.609 0.561

1	(0.362) 0.339 0.380

2	(0.058) 0.051 0.060

Locus 12039 :

2 alleles

96.9% missing data

2	(0.526) 0.510 0.529

1	(0.474) 0.490 0.471

Locus 12040 :

2 alleles

97.3% missing data

4	(0.654) 0.670 0.637

2	(0.346) 0.330 0.363

Locus 12041 :

2 alleles

9.4% missing data

3	(0.985) 0.979 0.995

4	(0.015) 0.021 0.005

Locus 12042 :

2 alleles

98.4% missing data

4	(0.804) 0.813 0.792

2	(0.196) 0.187 0.208

Locus 12043 :

2 alleles

99.2% missing data

3	(0.783) 0.780 0.785

1	(0.217) 0.220 0.215

Locus 12044 :

2 alleles

99.2% missing data

1	(0.390) 0.383 0.393

3	(0.610) 0.617 0.607

Locus 12045 :

2 alleles

96.1% missing data

2	(0.176) 0.169 0.180

1	(0.824) 0.831 0.820

Locus 12046 :

2 alleles

99.2% missing data

1	(0.456) 0.447 0.462

4	(0.544) 0.553 0.538

Locus 12047 :

2 alleles

99.2% missing data

4	(0.716) 0.707 0.715

2	(0.284) 0.293 0.285

Locus 12048 :

2 alleles

99.2% missing data

3	(0.708) 0.705 0.705

1	(0.292) 0.295 0.295

Locus 12049 :

2 alleles

99.2% missing data

4	(0.684) 0.684 0.678

3	(0.316) 0.316 0.322

Locus 12050 :

2 alleles

99.2% missing data

3	(0.598) 0.596 0.603

1	(0.402) 0.404 0.397

Locus 12051 :

2 alleles

99.2% missing data

2	(0.367) 0.356 0.373

3	(0.633) 0.644 0.627

Locus 12052 :

2 alleles

99.2% missing data

2	(0.522) 0.517 0.520

3	(0.478) 0.483 0.480

Locus 12053 :

2 alleles

99.2% missing data

2	(0.297) 0.291 0.298

3	(0.703) 0.709 0.702

Locus 12054 :

3 alleles

97.3% missing data

4	(0.200) 0.196 0.197

2	(0.550) 0.546 0.570

1	(0.250) 0.257 0.233

Locus 12055 :

2 alleles

99.2% missing data

1	(0.713) 0.712 0.711

4	(0.287) 0.288 0.289

Locus 12056 :

2 alleles

91.0% missing data

1	(0.886) 0.908 0.878

3	(0.114) 0.092 0.122

Locus 12057 :

3 alleles

98.4% missing data

1	(0.327) 0.328 0.328

2	(0.529) 0.533 0.523

3	(0.144) 0.138 0.149

Locus 12058 :

2 alleles

99.2% missing data

3	(0.286) 0.282 0.293

4	(0.714) 0.718 0.707

Locus 12059 :

2 alleles

99.2% missing data

2	(0.602) 0.602 0.598

3	(0.398) 0.398 0.402

Locus 12060 :

2 alleles

98.8% missing data

4	(0.624) 0.623 0.629

2	(0.376) 0.377 0.371

Locus 12061 :

2 alleles

99.2% missing data

3	(0.151) 0.155 0.150

4	(0.849) 0.845 0.850

Locus 12062 :

2 alleles

97.7% missing data

4	(0.402) 0.394 0.408

3	(0.598) 0.606 0.592

Locus 12063 :

2 alleles

99.2% missing data

4	(0.627) 0.627 0.634

1	(0.373) 0.373 0.366

Locus 12064 :

2 alleles

99.2% missing data

4	(0.683) 0.672 0.681

1	(0.317) 0.328 0.319

Locus 12065 :

2 alleles

99.2% missing data

3	(0.773) 0.766 0.777

4	(0.227) 0.234 0.223

Locus 12066 :

2 alleles

96.1% missing data

1	(0.853) 0.866 0.850

4	(0.147) 0.134 0.150

Locus 12067 :

2 alleles

14.5% missing data

1	(0.985) 0.988 0.992

3	(0.015) 0.012 0.008

Locus 12068 :

2 alleles

99.2% missing data

2	(0.430) 0.431 0.431

4	(0.570) 0.569 0.569

Locus 12069 :

2 alleles

99.2% missing data

3	(0.405) 0.405 0.402

1	(0.595) 0.595 0.598

Locus 12070 :

2 alleles

99.2% missing data

4	(0.304) 0.295 0.310

2	(0.696) 0.705 0.690

Locus 12071 :

2 alleles

99.2% missing data

3	(0.592) 0.595 0.587

1	(0.408) 0.405 0.413

Locus 12072 :

2 alleles

99.2% missing data

4	(0.364) 0.372 0.355

3	(0.636) 0.628 0.645

Locus 12073 :

2 alleles

95.3% missing data

3	(0.262) 0.269 0.243

2	(0.738) 0.731 0.757

Locus 12074 :

2 alleles

97.3% missing data

2	(0.340) 0.345 0.340

4	(0.660) 0.655 0.660

Locus 12075 :

2 alleles

99.2% missing data

2	(0.546) 0.543 0.548

3	(0.454) 0.457 0.452

Locus 12076 :

2 alleles

99.2% missing data

2	(0.673) 0.670 0.672

1	(0.327) 0.330 0.328

Locus 12077 :

2 alleles

96.1% missing data

1	(0.847) 0.860 0.838

2	(0.153) 0.140 0.162

Locus 12078 :

2 alleles

97.7% missing data

4	(0.238) 0.233 0.238

1	(0.762) 0.767 0.762

Locus 12079 :

2 alleles

0.4% missing data

3	(0.987) 0.991 0.993

4	(0.013) 0.009 0.007

Locus 12080 :

2 alleles

99.2% missing data

1	(0.861) 0.865 0.857

3	(0.139) 0.135 0.143

Locus 12081 :

2 alleles

99.2% missing data

2	(0.717) 0.715 0.716

4	(0.283) 0.285 0.284

Locus 12082 :

2 alleles

14.8% missing data

1	(0.980) 0.984 0.989

4	(0.020) 0.016 0.011

Locus 12083 :

2 alleles

8.2% missing data

4	(0.986) 0.976 0.996

1	(0.014) 0.024 0.004

Locus 12084 :

2 alleles

99.2% missing data

4	(0.290) 0.289 0.296

2	(0.710) 0.711 0.704

Locus 12085 :

2 alleles

99.2% missing data

4	(0.673) 0.672 0.672

3	(0.327) 0.328 0.328

Locus 12086 :

2 alleles

99.2% missing data

3	(0.453) 0.448 0.458

1	(0.547) 0.552 0.542

Locus 12087 :

2 alleles

95.7% missing data

1	(0.757) 0.774 0.749

3	(0.243) 0.226 0.251

Locus 12088 :

2 alleles

99.2% missing data

4	(0.289) 0.287 0.285

3	(0.711) 0.713 0.715

Locus 12089 :

2 alleles

99.2% missing data

4	(0.412) 0.403 0.416

3	(0.588) 0.597 0.584

Locus 12090 :

2 alleles

98.8% missing data

2	(0.539) 0.532 0.551

4	(0.461) 0.468 0.449

Locus 12091 :

2 alleles

99.2% missing data

2	(0.540) 0.550 0.540

1	(0.460) 0.450 0.460

Locus 12092 :

2 alleles

99.2% missing data

2	(0.367) 0.363 0.371

3	(0.633) 0.637 0.629

Locus 12093 :

2 alleles

98.8% missing data

2	(0.406) 0.404 0.407

3	(0.594) 0.596 0.593

Locus 12094 :

2 alleles

99.2% missing data

1	(0.551) 0.555 0.556

2	(0.449) 0.445 0.444

Locus 12095 :

2 alleles

99.2% missing data

1	(0.516) 0.523 0.516

3	(0.484) 0.477 0.484

Locus 12096 :

2 alleles

99.2% missing data

1	(0.525) 0.521 0.529

4	(0.475) 0.479 0.471

Locus 12097 :

2 alleles

98.0% missing data

3	(0.282) 0.286 0.273

2	(0.718) 0.714 0.727

Locus 12098 :

2 alleles

0.0% missing data

2	(0.952) 0.963 0.951

3	(0.048) 0.037 0.049

Locus 12099 :

2 alleles

99.2% missing data

2	(0.416) 0.411 0.424

1	(0.584) 0.589 0.576

Locus 12100 :

3 alleles

98.8% missing data

4	(0.453) 0.452 0.449

1	(0.160) 0.159 0.165

3	(0.387) 0.390 0.386

Locus 12101 :

2 alleles

99.2% missing data

2	(0.406) 0.401 0.415

3	(0.594) 0.599 0.585

Locus 12102 :

2 alleles

96.1% missing data

2	(0.739) 0.759 0.738

3	(0.261) 0.241 0.262

Locus 12103 :

2 alleles

97.7% missing data

2	(0.226) 0.212 0.232

1	(0.774) 0.788 0.768

Locus 12104 :

2 alleles

96.5% missing data

1	(0.799) 0.816 0.798

3	(0.201) 0.184 0.202

Locus 12105 :

2 alleles

98.8% missing data

4	(0.443) 0.437 0.446

1	(0.557) 0.563 0.554

Locus 12106 :

2 alleles

98.8% missing data

1	(0.724) 0.723 0.721

3	(0.276) 0.277 0.279

Locus 12107 :

2 alleles

99.2% missing data

1	(0.392) 0.392 0.391

4	(0.608) 0.608 0.609

Locus 12108 :

2 alleles

99.2% missing data

2	(0.490) 0.489 0.490

3	(0.510) 0.511 0.510

Locus 12109 :

2 alleles

97.7% missing data

3	(0.327) 0.317 0.330

2	(0.673) 0.683 0.670

Locus 12110 :

2 alleles

99.2% missing data

1	(0.579) 0.573 0.577

3	(0.421) 0.427 0.423

Locus 12111 :

3 alleles

97.7% missing data

1	(0.183) 0.191 0.178

4	(0.178) 0.174 0.175

2	(0.639) 0.635 0.647

Locus 12112 :

2 alleles

99.2% missing data

1	(0.754) 0.758 0.751

2	(0.246) 0.242 0.249

Locus 12113 :

2 alleles

96.1% missing data

3	(0.831) 0.837 0.834

1	(0.169) 0.163 0.166

Locus 12114 :

2 alleles

96.9% missing data

1	(0.828) 0.829 0.829

3	(0.172) 0.171 0.171

Locus 12115 :

2 alleles

99.2% missing data

1	(0.489) 0.497 0.485

3	(0.511) 0.503 0.515

Locus 12116 :

2 alleles

98.4% missing data

2	(0.735) 0.736 0.738

3	(0.265) 0.264 0.262

Locus 12117 :

2 alleles

8.6% missing data

3	(0.984) 0.987 0.992

2	(0.016) 0.013 0.008

Locus 12118 :

2 alleles

97.3% missing data

1	(0.380) 0.363 0.392

2	(0.620) 0.637 0.608

Locus 12119 :

2 alleles

98.4% missing data

3	(0.470) 0.462 0.479

1	(0.530) 0.538 0.521

Locus 12120 :

2 alleles

93.0% missing data

1	(0.886) 0.883 0.900

4	(0.114) 0.117 0.100

Locus 12121 :

2 alleles

99.2% missing data

4	(0.400) 0.390 0.407

2	(0.600) 0.610 0.593

Locus 12122 :

2 alleles

99.2% missing data

2	(0.680) 0.680 0.675

3	(0.320) 0.320 0.325

Locus 12123 :

2 alleles

99.2% missing data

1	(0.618) 0.611 0.620

3	(0.382) 0.389 0.380

Locus 12124 :

2 alleles

93.4% missing data

3	(0.921) 0.914 0.931

4	(0.079) 0.086 0.069

Locus 12125 :

2 alleles

99.2% missing data

3	(0.687) 0.687 0.686

1	(0.313) 0.313 0.314

Locus 12126 :

2 alleles

99.2% missing data

2	(0.574) 0.571 0.568

3	(0.426) 0.429 0.432

Locus 12127 :

2 alleles

99.2% missing data

4	(0.748) 0.746 0.748

1	(0.252) 0.254 0.252

Locus 12128 :

2 alleles

96.1% missing data

2	(0.163) 0.151 0.174

4	(0.837) 0.849 0.826

Locus 12129 :

2 alleles

8.2% missing data

3	(0.984) 0.976 0.995

2	(0.016) 0.024 0.005

Locus 12130 :

2 alleles

99.2% missing data

2	(0.444) 0.438 0.453

1	(0.556) 0.562 0.547

Locus 12131 :

2 alleles

98.8% missing data

3	(0.285) 0.277 0.284

2	(0.715) 0.723 0.716

Locus 12132 :

2 alleles

99.2% missing data

2	(0.519) 0.516 0.529

1	(0.481) 0.484 0.471

Locus 12133 :

2 alleles

99.2% missing data

2	(0.645) 0.648 0.649

4	(0.355) 0.352 0.351

Locus 12134 :

2 alleles

0.4% missing data

4	(0.978) 0.984 0.980

3	(0.022) 0.016 0.020

Locus 12135 :

2 alleles

99.2% missing data

3	(0.305) 0.300 0.310

2	(0.695) 0.700 0.690

Locus 12136 :

2 alleles

98.0% missing data

1	(0.864) 0.857 0.866

3	(0.136) 0.143 0.134

Locus 12137 :

2 alleles

99.2% missing data

2	(0.537) 0.532 0.540

1	(0.463) 0.468 0.460

Locus 12138 :

2 alleles

99.2% missing data

1	(0.532) 0.526 0.537

2	(0.468) 0.474 0.463

Locus 12139 :

2 alleles

0.0% missing data

2	(0.985) 0.988 0.992

1	(0.015) 0.012 0.008

Locus 12140 :

2 alleles

99.2% missing data

2	(0.614) 0.608 0.612

4	(0.386) 0.392 0.388

Locus 12141 :

2 alleles

99.2% missing data

3	(0.635) 0.629 0.635

1	(0.365) 0.371 0.365

Locus 12142 :

2 alleles

92.6% missing data

3	(0.863) 0.848 0.884

1	(0.137) 0.152 0.116

Locus 12143 :

2 alleles

99.2% missing data

3	(0.461) 0.460 0.464

2	(0.539) 0.540 0.536

Locus 12144 :

2 alleles

99.2% missing data

4	(0.614) 0.607 0.616

2	(0.386) 0.393 0.384

Locus 12145 :

2 alleles

99.2% missing data

3	(0.496) 0.503 0.493

2	(0.504) 0.497 0.507

Locus 12146 :

2 alleles

96.5% missing data

3	(0.765) 0.761 0.773

1	(0.235) 0.239 0.227

Locus 12147 :

2 alleles

97.7% missing data

1	(0.787) 0.791 0.789

4	(0.213) 0.209 0.211

Locus 12148 :

2 alleles

96.1% missing data

4	(0.756) 0.773 0.752

2	(0.244) 0.227 0.248

Locus 12149 :

2 alleles

99.2% missing data

1	(0.669) 0.667 0.674

3	(0.331) 0.333 0.326

Locus 12150 :

2 alleles

99.2% missing data

1	(0.374) 0.375 0.383

2	(0.626) 0.625 0.617

Locus 12151 :

2 alleles

92.6% missing data

1	(0.910) 0.913 0.916

2	(0.090) 0.087 0.084

Locus 12152 :

2 alleles

99.2% missing data

1	(0.313) 0.308 0.313

4	(0.687) 0.692 0.687

Locus 12153 :

2 alleles

95.7% missing data

1	(0.684) 0.691 0.681

3	(0.316) 0.309 0.319

Locus 12154 :

2 alleles

99.2% missing data

3	(0.562) 0.561 0.562

2	(0.438) 0.439 0.438

Locus 12155 :

2 alleles

99.2% missing data

4	(0.518) 0.510 0.525

2	(0.482) 0.490 0.475

Locus 12156 :

2 alleles

99.2% missing data

2	(0.477) 0.477 0.476

1	(0.523) 0.523 0.524

Locus 12157 :

2 alleles

99.2% missing data

1	(0.642) 0.633 0.645

3	(0.358) 0.367 0.355

Locus 12158 :

3 alleles

98.8% missing data

3	(0.602) 0.601 0.591

1	(0.213) 0.212 0.218

4	(0.185) 0.186 0.191

Locus 12159 :

2 alleles

84.4% missing data

1	(0.589) 0.664 0.503

3	(0.411) 0.336 0.497

Locus 12160 :

2 alleles

96.9% missing data

2	(0.233) 0.224 0.233

1	(0.767) 0.776 0.767

Locus 12161 :

2 alleles

99.2% missing data

1	(0.724) 0.720 0.725

4	(0.276) 0.280 0.275

Locus 12162 :

2 alleles

98.0% missing data

1	(0.735) 0.740 0.734

4	(0.265) 0.260 0.266

Locus 12163 :

2 alleles

99.2% missing data

4	(0.227) 0.224 0.226

3	(0.773) 0.776 0.774

Locus 12164 :

2 alleles

98.4% missing data

2	(0.819) 0.819 0.822

3	(0.181) 0.181 0.178

Locus 12165 :

2 alleles

99.2% missing data

1	(0.321) 0.315 0.322

4	(0.679) 0.685 0.678

Locus 12166 :

2 alleles

99.2% missing data

1	(0.231) 0.238 0.223

4	(0.769) 0.762 0.777

Locus 12167 :

2 alleles

96.1% missing data

2	(0.844) 0.849 0.847

4	(0.156) 0.151 0.153

Locus 12168 :

2 alleles

98.0% missing data

3	(0.473) 0.458 0.480

2	(0.527) 0.542 0.520

Locus 12169 :

2 alleles

98.8% missing data

1	(0.592) 0.585 0.599

4	(0.408) 0.415 0.401

Locus 12170 :

2 alleles

99.2% missing data

1	(0.328) 0.329 0.338

3	(0.672) 0.671 0.662

Locus 12171 :

2 alleles

99.2% missing data

1	(0.588) 0.590 0.597

3	(0.412) 0.410 0.403

Locus 12172 :

2 alleles

99.2% missing data

3	(0.589) 0.582 0.598

1	(0.411) 0.418 0.402

Locus 12173 :

2 alleles

99.2% missing data

2	(0.413) 0.408 0.414

4	(0.587) 0.592 0.586

Locus 12174 :

2 alleles

98.8% missing data

3	(0.377) 0.381 0.381

2	(0.623) 0.619 0.619

Locus 12175 :

2 alleles

88.3% missing data

1	(0.799) 0.801 0.789

3	(0.201) 0.199 0.211

Locus 12176 :

2 alleles

99.2% missing data

3	(0.488) 0.488 0.484

1	(0.512) 0.512 0.516

Locus 12177 :

2 alleles

98.0% missing data

1	(0.606) 0.612 0.610

3	(0.394) 0.388 0.390

Locus 12178 :

2 alleles

99.2% missing data

2	(0.399) 0.393 0.406

3	(0.601) 0.607 0.594

Locus 12179 :

2 alleles

92.2% missing data

3	(0.665) 0.696 0.634

1	(0.335) 0.304 0.366

Locus 12180 :

2 alleles

99.2% missing data

1	(0.260) 0.256 0.266

3	(0.740) 0.744 0.734

Locus 12181 :

2 alleles

97.3% missing data

3	(0.719) 0.709 0.738

1	(0.281) 0.291 0.262

Locus 12182 :

2 alleles

98.4% missing data

2	(0.576) 0.581 0.574

3	(0.424) 0.419 0.426

Locus 12183 :

2 alleles

98.0% missing data

3	(0.299) 0.294 0.296

4	(0.701) 0.706 0.704

Locus 12184 :

2 alleles

99.2% missing data

4	(0.896) 0.898 0.889

1	(0.104) 0.102 0.111

Locus 12185 :

2 alleles

97.7% missing data

2	(0.625) 0.609 0.640

1	(0.375) 0.391 0.360

Locus 12186 :

2 alleles

99.2% missing data

3	(0.624) 0.619 0.627

1	(0.376) 0.381 0.373

Locus 12187 :

2 alleles

98.4% missing data

1	(0.340) 0.332 0.348

3	(0.660) 0.668 0.652

Locus 12188 :

2 alleles

99.2% missing data

3	(0.706) 0.708 0.701

1	(0.294) 0.292 0.299

Locus 12189 :

2 alleles

98.0% missing data

3	(0.333) 0.328 0.329

2	(0.667) 0.672 0.671

Locus 12190 :

2 alleles

99.2% missing data

1	(0.158) 0.164 0.162

3	(0.842) 0.836 0.838

Locus 12191 :

2 alleles

99.2% missing data

2	(0.426) 0.425 0.425

3	(0.574) 0.575 0.575

Locus 12192 :

2 alleles

99.2% missing data

2	(0.602) 0.605 0.602

4	(0.398) 0.395 0.398

Locus 12193 :

2 alleles

98.8% missing data

3	(0.406) 0.405 0.413

4	(0.594) 0.595 0.587

Locus 12194 :

2 alleles

99.2% missing data

1	(0.345) 0.348 0.335

2	(0.655) 0.652 0.665

Locus 12195 :

2 alleles

99.2% missing data

4	(0.453) 0.453 0.454

3	(0.547) 0.547 0.546

Locus 12196 :

2 alleles

95.3% missing data

4	(0.250) 0.254 0.231

2	(0.750) 0.746 0.769

Locus 12197 :

2 alleles

98.8% missing data

4	(0.348) 0.344 0.350

1	(0.652) 0.656 0.650

Locus 12198 :

2 alleles

99.2% missing data

1	(0.339) 0.334 0.340

3	(0.661) 0.666 0.660

Locus 12199 :

2 alleles

98.8% missing data

3	(0.581) 0.576 0.584

2	(0.419) 0.424 0.416

Locus 12200 :

2 alleles

99.2% missing data

3	(0.577) 0.566 0.583

1	(0.423) 0.434 0.417

Locus 12201 :

2 alleles

99.2% missing data

4	(0.541) 0.535 0.545

1	(0.459) 0.465 0.455

Locus 12202 :

2 alleles

99.2% missing data

4	(0.567) 0.561 0.571

3	(0.433) 0.439 0.429

Locus 12203 :

2 alleles

96.1% missing data

1	(0.181) 0.172 0.185

2	(0.819) 0.828 0.815

Locus 12204 :

2 alleles

99.2% missing data

4	(0.628) 0.630 0.627

2	(0.372) 0.370 0.373

Locus 12205 :

2 alleles

96.5% missing data

3	(0.222) 0.213 0.229

4	(0.778) 0.787 0.771

Locus 12206 :

2 alleles

91.0% missing data

1	(0.918) 0.925 0.921

3	(0.082) 0.075 0.079

Locus 12207 :

2 alleles

97.7% missing data

3	(0.363) 0.360 0.358

1	(0.637) 0.640 0.642

Locus 12208 :

2 alleles

97.7% missing data

4	(0.654) 0.660 0.650

3	(0.346) 0.340 0.350

Locus 12209 :

2 alleles

98.8% missing data

2	(0.505) 0.502 0.506

3	(0.495) 0.498 0.494

Locus 12210 :

2 alleles

0.0% missing data

2	(0.987) 0.984 0.996

3	(0.013) 0.016 0.004

Locus 12211 :

2 alleles

98.0% missing data

3	(0.642) 0.642 0.651

2	(0.358) 0.358 0.349

Locus 12212 :

2 alleles

98.0% missing data

2	(0.552) 0.553 0.563

4	(0.448) 0.447 0.437

Locus 12213 :

2 alleles

99.2% missing data

2	(0.572) 0.565 0.576

3	(0.428) 0.435 0.424

Locus 12214 :

2 alleles

99.2% missing data

3	(0.505) 0.510 0.501

2	(0.495) 0.490 0.499

Locus 12215 :

2 alleles

99.2% missing data

4	(0.728) 0.721 0.729

1	(0.272) 0.279 0.271

Locus 12216 :

2 alleles

94.1% missing data

3	(0.870) 0.863 0.884

2	(0.130) 0.137 0.116

Locus 12217 :

2 alleles

99.2% missing data

3	(0.404) 0.404 0.405

2	(0.596) 0.596 0.595

Locus 12218 :

2 alleles

99.2% missing data

2	(0.252) 0.250 0.253

4	(0.748) 0.750 0.747

Locus 12219 :

2 alleles

99.2% missing data

3	(0.313) 0.310 0.320

1	(0.687) 0.690 0.680

Locus 12220 :

2 alleles

99.2% missing data

1	(0.389) 0.392 0.381

4	(0.611) 0.608 0.619

Locus 12221 :

2 alleles

99.2% missing data

1	(0.351) 0.350 0.351

3	(0.649) 0.650 0.649

Locus 12222 :

2 alleles

97.3% missing data

3	(0.203) 0.193 0.207

2	(0.797) 0.807 0.793

Locus 12223 :

2 alleles

98.8% missing data

3	(0.494) 0.490 0.507

1	(0.506) 0.510 0.493

Locus 12224 :

2 alleles

99.2% missing data

4	(0.815) 0.818 0.810

3	(0.185) 0.182 0.190

Locus 12225 :

2 alleles

99.2% missing data

1	(0.345) 0.337 0.350

3	(0.655) 0.663 0.650

Locus 12226 :

2 alleles

8.6% missing data

4	(0.985) 0.980 0.995

1	(0.015) 0.020 0.005

Locus 12227 :

2 alleles

99.2% missing data

1	(0.508) 0.512 0.502

3	(0.492) 0.488 0.498

Locus 12228 :

2 alleles

99.2% missing data

2	(0.269) 0.279 0.264

3	(0.731) 0.721 0.736

Locus 12229 :

2 alleles

99.2% missing data

4	(0.297) 0.299 0.301

3	(0.703) 0.701 0.699

Locus 12230 :

3 alleles

98.8% missing data

3	(0.281) 0.282 0.283

1	(0.456) 0.449 0.454

2	(0.263) 0.269 0.263

Locus 12231 :

2 alleles

99.2% missing data

1	(0.564) 0.561 0.566

3	(0.436) 0.439 0.434

Locus 12232 :

2 alleles

99.2% missing data

3	(0.342) 0.340 0.350

1	(0.658) 0.660 0.650

Locus 12233 :

2 alleles

91.0% missing data

3	(0.925) 0.933 0.925

1	(0.075) 0.067 0.075

Locus 12234 :

2 alleles

98.4% missing data

1	(0.587) 0.578 0.588

2	(0.413) 0.422 0.412

Locus 12235 :

2 alleles

99.2% missing data

4	(0.623) 0.620 0.629

3	(0.377) 0.380 0.371

Locus 12236 :

2 alleles

99.2% missing data

1	(0.174) 0.173 0.178

3	(0.826) 0.827 0.822

Locus 12237 :

2 alleles

99.2% missing data

1	(0.403) 0.398 0.406

4	(0.597) 0.602 0.594

Locus 12238 :

2 alleles

99.2% missing data

2	(0.706) 0.702 0.706

1	(0.294) 0.298 0.294

Locus 12239 :

3 alleles

89.1% missing data

2	(0.830) 0.803 0.865

1	(0.050) 0.053 0.040

4	(0.121) 0.144 0.096

Locus 12240 :

2 alleles

98.8% missing data

2	(0.650) 0.648 0.660

4	(0.350) 0.352 0.340

Locus 12241 :

2 alleles

0.0% missing data

3	(0.983) 0.981 0.995

4	(0.017) 0.019 0.005

Locus 12242 :

2 alleles

98.4% missing data

2	(0.500) 0.519 0.488

1	(0.500) 0.481 0.512

Locus 12243 :

2 alleles

99.2% missing data

1	(0.498) 0.499 0.494

3	(0.502) 0.501 0.506

Locus 12244 :

2 alleles

98.8% missing data

1	(0.615) 0.614 0.618

2	(0.385) 0.386 0.382

Locus 12245 :

2 alleles

99.2% missing data

1	(0.483) 0.470 0.493

2	(0.517) 0.530 0.507

Locus 12246 :

2 alleles

99.2% missing data

1	(0.324) 0.318 0.332

4	(0.676) 0.682 0.668

Locus 12247 :

2 alleles

99.2% missing data

2	(0.545) 0.551 0.541

1	(0.455) 0.449 0.459

Locus 12248 :

2 alleles

99.2% missing data

3	(0.508) 0.510 0.508

1	(0.492) 0.490 0.492

Locus 12249 :

2 alleles

99.2% missing data

1	(0.370) 0.369 0.375

2	(0.630) 0.631 0.625

Locus 12250 :

2 alleles

75.8% missing data

4	(0.935) 0.925 0.951

1	(0.065) 0.075 0.049

Locus 12251 :

2 alleles

99.2% missing data

1	(0.432) 0.430 0.429

3	(0.568) 0.570 0.571

Locus 12252 :

2 alleles

99.2% missing data

1	(0.816) 0.814 0.819

4	(0.184) 0.186 0.181

Locus 12253 :

2 alleles

99.2% missing data

1	(0.583) 0.586 0.584

3	(0.417) 0.414 0.416

Locus 12254 :

2 alleles

99.2% missing data

4	(0.248) 0.244 0.256

2	(0.752) 0.756 0.744

Locus 12255 :

2 alleles

98.8% missing data

1	(0.560) 0.559 0.560

2	(0.440) 0.441 0.440

Locus 12256 :

2 alleles

99.2% missing data

1	(0.365) 0.360 0.365

3	(0.635) 0.640 0.635

Locus 12257 :

2 alleles

99.2% missing data

2	(0.232) 0.232 0.241

1	(0.768) 0.768 0.759

Locus 12258 :

2 alleles

99.2% missing data

2	(0.248) 0.256 0.242

3	(0.752) 0.744 0.758

Locus 12259 :

2 alleles

98.8% missing data

3	(0.211) 0.207 0.210

1	(0.789) 0.793 0.790

Locus 12260 :

2 alleles

99.2% missing data

2	(0.474) 0.476 0.470

3	(0.526) 0.524 0.530

Locus 12261 :

2 alleles

96.1% missing data

3	(0.872) 0.876 0.872

1	(0.128) 0.124 0.128

Locus 12262 :

2 alleles

99.2% missing data

1	(0.583) 0.575 0.586

2	(0.417) 0.425 0.414

Locus 12263 :

2 alleles

98.4% missing data

3	(0.633) 0.630 0.636

2	(0.367) 0.370 0.364

Locus 12264 :

2 alleles

99.2% missing data

2	(0.658) 0.658 0.667

1	(0.342) 0.342 0.333

Locus 12265 :

2 alleles

99.2% missing data

4	(0.292) 0.286 0.296

3	(0.708) 0.714 0.704

Locus 12266 :

2 alleles

98.0% missing data

2	(0.789) 0.796 0.783

4	(0.211) 0.204 0.217

Locus 12267 :

3 alleles

98.8% missing data

4	(0.392) 0.392 0.396

3	(0.258) 0.255 0.257

2	(0.350) 0.353 0.347

Locus 12268 :

2 alleles

99.2% missing data

3	(0.329) 0.328 0.341

4	(0.671) 0.672 0.659

Locus 12269 :

2 alleles

99.2% missing data

1	(0.483) 0.483 0.486

4	(0.517) 0.517 0.514

Locus 12270 :

2 alleles

99.2% missing data

3	(0.479) 0.486 0.474

2	(0.521) 0.514 0.526

Locus 12271 :

2 alleles

99.2% missing data

1	(0.477) 0.470 0.481

3	(0.523) 0.530 0.519

Locus 12272 :

2 alleles

99.2% missing data

1	(0.406) 0.412 0.405

3	(0.594) 0.588 0.595

Locus 12273 :

2 alleles

99.2% missing data

3	(0.388) 0.385 0.391

1	(0.612) 0.615 0.609

Locus 12274 :

2 alleles

97.3% missing data

3	(0.745) 0.753 0.744

1	(0.255) 0.247 0.256

Locus 12275 :

2 alleles

99.2% missing data

4	(0.399) 0.402 0.403

3	(0.601) 0.598 0.597

Locus 12276 :

2 alleles

99.2% missing data

2	(0.426) 0.425 0.433

3	(0.574) 0.575 0.567

Locus 12277 :

2 alleles

94.5% missing data

4	(0.477) 0.508 0.451

3	(0.523) 0.492 0.549

Locus 12278 :

3 alleles

98.4% missing data

3	(0.200) 0.194 0.205

4	(0.617) 0.617 0.620

2	(0.183) 0.188 0.175

Locus 12279 :

2 alleles

99.2% missing data

1	(0.606) 0.607 0.614

3	(0.394) 0.393 0.386

Locus 12280 :

2 alleles

99.2% missing data

3	(0.239) 0.239 0.243

2	(0.761) 0.761 0.757

Locus 12281 :

2 alleles

99.2% missing data

2	(0.635) 0.640 0.625

1	(0.365) 0.360 0.375

Locus 12282 :

2 alleles

99.2% missing data

1	(0.317) 0.313 0.324

4	(0.683) 0.687 0.676

Locus 12283 :

2 alleles

94.1% missing data

2	(0.837) 0.840 0.850

1	(0.163) 0.160 0.150

Locus 12284 :

2 alleles

99.2% missing data

2	(0.509) 0.502 0.514

1	(0.491) 0.498 0.486

Locus 12285 :

2 alleles

95.3% missing data

1	(0.143) 0.144 0.138

3	(0.857) 0.856 0.862

Locus 12286 :

2 alleles

91.0% missing data

1	(0.916) 0.925 0.916

2	(0.084) 0.075 0.084

Locus 12287 :

2 alleles

99.2% missing data

2	(0.317) 0.313 0.322

3	(0.683) 0.687 0.678

Locus 12288 :

2 alleles

96.1% missing data

1	(0.148) 0.134 0.155

3	(0.852) 0.866 0.845

Locus 12289 :

2 alleles

99.2% missing data

3	(0.252) 0.247 0.262

1	(0.748) 0.753 0.738

Locus 12290 :

3 alleles

95.7% missing data

2	(0.766) 0.773 0.761

1	(0.129) 0.122 0.136

3	(0.104) 0.105 0.103

Locus 12291 :

2 alleles

94.1% missing data

1	(0.289) 0.281 0.287

2	(0.711) 0.719 0.713

Locus 12292 :

2 alleles

98.8% missing data

4	(0.750) 0.752 0.754

1	(0.250) 0.248 0.246

Locus 12293 :

2 alleles

99.2% missing data

4	(0.357) 0.359 0.352

1	(0.643) 0.641 0.648

Locus 12294 :

2 alleles

99.2% missing data

4	(0.240) 0.236 0.242

1	(0.760) 0.764 0.758

Locus 12295 :

2 alleles

98.8% missing data

2	(0.532) 0.530 0.529

4	(0.468) 0.470 0.471

Locus 12296 :

2 alleles

99.2% missing data

1	(0.357) 0.354 0.357

2	(0.643) 0.646 0.643

Locus 12297 :

2 alleles

98.8% missing data

1	(0.571) 0.569 0.572

2	(0.429) 0.431 0.428

Locus 12298 :

2 alleles

96.9% missing data

2	(0.742) 0.742 0.754

4	(0.258) 0.258 0.246

Locus 12299 :

2 alleles

98.0% missing data

2	(0.816) 0.816 0.815

1	(0.184) 0.184 0.185

Locus 12300 :

2 alleles

0.4% missing data

2	(0.985) 0.989 0.991

3	(0.015) 0.011 0.009

Locus 12301 :

2 alleles

99.2% missing data

1	(0.497) 0.488 0.497

2	(0.503) 0.512 0.503

Locus 12302 :

2 alleles

99.2% missing data

2	(0.382) 0.380 0.384

1	(0.618) 0.620 0.616

Locus 12303 :

2 alleles

99.2% missing data

2	(0.564) 0.557 0.569

1	(0.436) 0.443 0.431

Locus 12304 :

2 alleles

96.5% missing data

4	(0.137) 0.135 0.133

2	(0.863) 0.865 0.867

Locus 12305 :

2 alleles

81.2% missing data

2	(0.415) 0.437 0.385

1	(0.585) 0.563 0.615

Locus 12306 :

2 alleles

93.8% missing data

3	(0.659) 0.624 0.695

1	(0.341) 0.376 0.305

Locus 12307 :

2 alleles

99.2% missing data

4	(0.651) 0.647 0.652

2	(0.349) 0.353 0.348

Locus 12308 :

2 alleles

99.2% missing data

4	(0.649) 0.644 0.656

3	(0.351) 0.356 0.344

Locus 12309 :

2 alleles

99.2% missing data

2	(0.395) 0.393 0.401

1	(0.605) 0.607 0.599

Locus 12310 :

2 alleles

99.2% missing data

4	(0.361) 0.356 0.361

1	(0.639) 0.644 0.639

Locus 12311 :

2 alleles

99.2% missing data

4	(0.429) 0.434 0.427

3	(0.571) 0.566 0.573

Locus 12312 :

2 alleles

99.2% missing data

1	(0.446) 0.447 0.445

2	(0.554) 0.553 0.555

Locus 12313 :

2 alleles

98.8% missing data

4	(0.644) 0.649 0.650

3	(0.356) 0.351 0.350

Locus 12314 :

2 alleles

90.6% missing data

2	(0.887) 0.905 0.880

4	(0.113) 0.095 0.120

Locus 12315 :

2 alleles

99.2% missing data

3	(0.488) 0.490 0.481

2	(0.512) 0.510 0.519

Locus 12316 :

2 alleles

98.0% missing data

4	(0.381) 0.390 0.364

1	(0.619) 0.610 0.636

Locus 12317 :

2 alleles

99.2% missing data

3	(0.194) 0.190 0.195

4	(0.806) 0.810 0.805

Locus 12318 :

2 alleles

99.2% missing data

4	(0.405) 0.411 0.407

1	(0.595) 0.589 0.593

Locus 12319 :

2 alleles

99.2% missing data

3	(0.657) 0.658 0.654

4	(0.343) 0.342 0.346

Locus 12320 :

2 alleles

98.8% missing data

2	(0.674) 0.674 0.668

3	(0.326) 0.326 0.332

Locus 12321 :

2 alleles

99.2% missing data

4	(0.279) 0.280 0.282

2	(0.721) 0.720 0.718

Locus 12322 :

2 alleles

98.8% missing data

1	(0.246) 0.250 0.256

4	(0.754) 0.750 0.744

Locus 12323 :

2 alleles

99.2% missing data

3	(0.621) 0.626 0.612

4	(0.379) 0.374 0.388

Locus 12324 :

2 alleles

99.2% missing data

1	(0.426) 0.433 0.426

2	(0.574) 0.567 0.574

Locus 12325 :

4 alleles

96.5% missing data

4	(0.136) 0.137 0.132

2	(0.560) 0.553 0.584

1	(0.082) 0.084 0.083

3	(0.222) 0.226 0.201

Locus 12326 :

2 alleles

99.2% missing data

4	(0.451) 0.456 0.446

3	(0.549) 0.544 0.554

Locus 12327 :

2 alleles

99.2% missing data

3	(0.516) 0.520 0.508

1	(0.484) 0.480 0.492

Locus 12328 :

2 alleles

99.2% missing data

3	(0.575) 0.578 0.576

4	(0.425) 0.422 0.424

Locus 12329 :

2 alleles

99.2% missing data

3	(0.524) 0.528 0.522

1	(0.476) 0.472 0.478

Locus 12330 :

2 alleles

99.2% missing data

1	(0.414) 0.409 0.412

2	(0.586) 0.591 0.588

Locus 12331 :

3 alleles

96.5% missing data

2	(0.356) 0.341 0.374

3	(0.147) 0.139 0.147

1	(0.497) 0.520 0.479

Locus 12332 :

2 alleles

99.2% missing data

4	(0.205) 0.201 0.215

1	(0.795) 0.799 0.785

Locus 12333 :

2 alleles

7.8% missing data

1	(0.982) 0.977 0.994

4	(0.018) 0.023 0.006

Locus 12334 :

2 alleles

91.0% missing data

4	(0.903) 0.919 0.892

2	(0.097) 0.081 0.108

Locus 12335 :

2 alleles

99.2% missing data

2	(0.693) 0.697 0.692

1	(0.307) 0.303 0.308

Locus 12336 :

2 alleles

98.8% missing data

3	(0.533) 0.530 0.541

4	(0.467) 0.470 0.459

Locus 12337 :

2 alleles

99.2% missing data

3	(0.577) 0.569 0.588

1	(0.423) 0.431 0.412

Locus 12338 :

2 alleles

96.1% missing data

4	(0.211) 0.202 0.222

2	(0.789) 0.798 0.778

Locus 12339 :

2 alleles

99.2% missing data

2	(0.502) 0.499 0.500

4	(0.498) 0.501 0.500

Locus 12340 :

2 alleles

99.2% missing data

2	(0.265) 0.271 0.263

4	(0.735) 0.729 0.737

Locus 12341 :

2 alleles

96.1% missing data

2	(0.247) 0.230 0.251

1	(0.753) 0.770 0.749

Locus 12342 :

2 alleles

99.2% missing data

4	(0.502) 0.494 0.505

1	(0.498) 0.506 0.495

Locus 12343 :

2 alleles

99.2% missing data

4	(0.459) 0.453 0.461

3	(0.541) 0.547 0.539

Locus 12344 :

2 alleles

96.9% missing data

3	(0.698) 0.688 0.718

1	(0.302) 0.312 0.282

Locus 12345 :

2 alleles

99.2% missing data

1	(0.497) 0.495 0.500

4	(0.503) 0.505 0.500

Locus 12346 :

2 alleles

97.7% missing data

3	(0.390) 0.391 0.383

2	(0.610) 0.609 0.617

Locus 12347 :

2 alleles

99.2% missing data

3	(0.772) 0.772 0.768

4	(0.228) 0.228 0.232

Locus 12348 :

3 alleles

98.4% missing data

2	(0.335) 0.334 0.331

4	(0.332) 0.332 0.336

3	(0.333) 0.335 0.333

Locus 12349 :

2 alleles

96.1% missing data

1	(0.848) 0.859 0.841

3	(0.152) 0.141 0.159

Locus 12350 :

2 alleles

99.2% missing data

1	(0.425) 0.432 0.422

4	(0.575) 0.568 0.578

Locus 12351 :

2 alleles

99.2% missing data

1	(0.414) 0.417 0.415

4	(0.586) 0.583 0.585

Locus 12352 :

2 alleles

99.2% missing data

3	(0.387) 0.382 0.390

2	(0.613) 0.618 0.610

Locus 12353 :

2 alleles

98.0% missing data

3	(0.689) 0.690 0.695

2	(0.311) 0.310 0.305

Locus 12354 :

2 alleles

99.2% missing data

2	(0.315) 0.312 0.327

3	(0.685) 0.688 0.673

Locus 12355 :

2 alleles

99.2% missing data

3	(0.265) 0.265 0.267

2	(0.735) 0.735 0.733

Locus 12356 :

2 alleles

98.8% missing data

3	(0.638) 0.638 0.641

1	(0.362) 0.362 0.359

Locus 12357 :

2 alleles

99.2% missing data

1	(0.657) 0.658 0.652

4	(0.343) 0.342 0.348

Locus 12358 :

2 alleles

99.2% missing data

4	(0.441) 0.432 0.450

3	(0.559) 0.568 0.550

Locus 12359 :

2 alleles

98.8% missing data

1	(0.337) 0.333 0.349

4	(0.663) 0.667 0.651

Locus 12360 :

2 alleles

98.4% missing data

3	(0.783) 0.785 0.782

1	(0.217) 0.215 0.218

Locus 12361 :

2 alleles

99.2% missing data

1	(0.323) 0.321 0.330

3	(0.677) 0.679 0.670

Locus 12362 :

2 alleles

98.8% missing data

1	(0.713) 0.713 0.713

3	(0.287) 0.287 0.287

Locus 12363 :

2 alleles

99.2% missing data

3	(0.354) 0.348 0.358

2	(0.646) 0.652 0.642

Locus 12364 :

2 alleles

98.8% missing data

4	(0.605) 0.604 0.596

3	(0.395) 0.396 0.404

Locus 12365 :

2 alleles

99.2% missing data

3	(0.171) 0.172 0.174

2	(0.829) 0.828 0.826

Locus 12366 :

2 alleles

93.0% missing data

4	(0.890) 0.885 0.902

1	(0.110) 0.115 0.098

Locus 12367 :

3 alleles

98.4% missing data

1	(0.374) 0.378 0.379

4	(0.168) 0.163 0.174

3	(0.458) 0.459 0.447

Locus 12368 :

2 alleles

89.1% missing data

1	(0.921) 0.918 0.934

3	(0.079) 0.082 0.066

Locus 12369 :

2 alleles

98.0% missing data

1	(0.688) 0.686 0.689

3	(0.312) 0.314 0.311

Locus 12370 :

2 alleles

99.2% missing data

1	(0.310) 0.319 0.308

4	(0.690) 0.681 0.692

Locus 12371 :

2 alleles

99.2% missing data

3	(0.784) 0.785 0.784

4	(0.216) 0.215 0.216

Locus 12372 :

2 alleles

99.2% missing data

2	(0.841) 0.836 0.842

1	(0.159) 0.164 0.158

Locus 12373 :

2 alleles

99.2% missing data

1	(0.478) 0.478 0.475

4	(0.522) 0.522 0.525

Locus 12374 :

2 alleles

99.2% missing data

1	(0.695) 0.700 0.692

3	(0.305) 0.300 0.308

Locus 12375 :

2 alleles

99.2% missing data

4	(0.508) 0.501 0.515

3	(0.492) 0.499 0.485

Locus 12376 :

2 alleles

99.2% missing data

2	(0.358) 0.366 0.353

1	(0.642) 0.634 0.647

Locus 12377 :

2 alleles

98.8% missing data

4	(0.476) 0.479 0.471

1	(0.524) 0.521 0.529

Locus 12378 :

2 alleles

99.2% missing data

3	(0.266) 0.261 0.274

4	(0.734) 0.739 0.726

Locus 12379 :

2 alleles

99.2% missing data

1	(0.690) 0.696 0.684

3	(0.310) 0.304 0.316

Locus 12380 :

2 alleles

95.3% missing data

1	(0.903) 0.896 0.910

4	(0.097) 0.104 0.090

Locus 12381 :

2 alleles

91.0% missing data

3	(0.888) 0.907 0.878

4	(0.112) 0.093 0.122

Locus 12382 :

2 alleles

99.2% missing data

1	(0.352) 0.348 0.355

3	(0.648) 0.652 0.645

Locus 12383 :

2 alleles

98.8% missing data

3	(0.358) 0.346 0.362

2	(0.642) 0.654 0.638

Locus 12384 :

2 alleles

97.3% missing data

2	(0.247) 0.251 0.238

1	(0.753) 0.749 0.762

Locus 12385 :

2 alleles

99.2% missing data

3	(0.276) 0.278 0.286

1	(0.724) 0.722 0.714

Locus 12386 :

2 alleles

99.2% missing data

4	(0.704) 0.703 0.701

2	(0.296) 0.297 0.299

Locus 12387 :

2 alleles

99.2% missing data

4	(0.647) 0.645 0.645

2	(0.353) 0.355 0.355

Locus 12388 :

2 alleles

99.2% missing data

2	(0.551) 0.550 0.548

4	(0.449) 0.450 0.452

Locus 12389 :

3 alleles

98.0% missing data

2	(0.146) 0.139 0.157

4	(0.271) 0.268 0.271

3	(0.583) 0.592 0.572

Locus 12390 :

2 alleles

99.2% missing data

4	(0.598) 0.598 0.598

3	(0.402) 0.402 0.402

Locus 12391 :

2 alleles

99.2% missing data

3	(0.655) 0.646 0.657

1	(0.345) 0.354 0.343

Locus 12392 :

2 alleles

99.2% missing data

4	(0.459) 0.458 0.466

3	(0.541) 0.542 0.534

Locus 12393 :

2 alleles

99.2% missing data

3	(0.628) 0.627 0.624

2	(0.372) 0.373 0.376

Locus 12394 :

2 alleles

99.2% missing data

3	(0.463) 0.458 0.470

4	(0.537) 0.542 0.530

Locus 12395 :

2 alleles

99.2% missing data

3	(0.245) 0.251 0.237

1	(0.755) 0.749 0.763

Locus 12396 :

2 alleles

98.4% missing data

2	(0.426) 0.425 0.426

1	(0.574) 0.575 0.574

Locus 12397 :

2 alleles

99.2% missing data

4	(0.419) 0.418 0.417

2	(0.581) 0.582 0.583

Locus 12398 :

2 alleles

99.2% missing data

2	(0.721) 0.720 0.724

1	(0.279) 0.280 0.276

Locus 12399 :

2 alleles

99.2% missing data

2	(0.771) 0.770 0.771

4	(0.229) 0.230 0.229

Locus 12400 :

2 alleles

99.2% missing data

1	(0.350) 0.343 0.356

4	(0.650) 0.657 0.644

Locus 12401 :

2 alleles

98.4% missing data

3	(0.224) 0.227 0.219

1	(0.776) 0.773 0.781

Locus 12402 :

2 alleles

96.5% missing data

2	(0.701) 0.712 0.703

1	(0.299) 0.288 0.297

Locus 12403 :

2 alleles

99.2% missing data

2	(0.726) 0.728 0.721

3	(0.274) 0.272 0.279

Locus 12404 :

2 alleles

99.2% missing data

2	(0.499) 0.493 0.508

4	(0.501) 0.507 0.492

Locus 12405 :

2 alleles

98.8% missing data

3	(0.578) 0.574 0.574

1	(0.422) 0.426 0.426

Locus 12406 :

2 alleles

98.8% missing data

3	(0.239) 0.237 0.243

4	(0.761) 0.763 0.757

Locus 12407 :

2 alleles

99.2% missing data

2	(0.445) 0.448 0.440

1	(0.555) 0.552 0.560

Locus 12408 :

2 alleles

99.2% missing data

4	(0.534) 0.531 0.530

3	(0.466) 0.469 0.470

Locus 12409 :

2 alleles

99.2% missing data

2	(0.437) 0.441 0.434

1	(0.563) 0.559 0.566

Locus 12410 :

2 alleles

99.2% missing data

1	(0.430) 0.423 0.431

3	(0.570) 0.577 0.569

Locus 12411 :

2 alleles

99.2% missing data

4	(0.260) 0.259 0.263

1	(0.740) 0.741 0.737

Locus 12412 :

2 alleles

91.0% missing data

2	(0.881) 0.900 0.869

1	(0.119) 0.100 0.131

Locus 12413 :

2 alleles

99.2% missing data

3	(0.719) 0.713 0.720

1	(0.281) 0.287 0.280

Locus 12414 :

2 alleles

99.2% missing data

2	(0.837) 0.839 0.830

1	(0.163) 0.161 0.170

Locus 12415 :

2 alleles

99.2% missing data

2	(0.208) 0.205 0.213

1	(0.792) 0.795 0.787

Locus 12416 :

2 alleles

99.2% missing data

2	(0.558) 0.557 0.562

4	(0.442) 0.443 0.438

Locus 12417 :

2 alleles

99.2% missing data

3	(0.239) 0.239 0.245

2	(0.761) 0.761 0.755

Locus 12418 :

2 alleles

97.3% missing data

2	(0.430) 0.443 0.415

4	(0.570) 0.557 0.585

Locus 12419 :

2 alleles

99.2% missing data

1	(0.466) 0.476 0.456

2	(0.534) 0.524 0.544

Locus 12420 :

2 alleles

99.2% missing data

1	(0.638) 0.632 0.637

4	(0.362) 0.368 0.363

Locus 12421 :

3 alleles

98.8% missing data

1	(0.485) 0.483 0.486

4	(0.129) 0.125 0.141

3	(0.385) 0.392 0.373

Locus 12422 :

2 alleles

99.2% missing data

3	(0.597) 0.590 0.599

4	(0.403) 0.410 0.401

Locus 12423 :

2 alleles

99.2% missing data

4	(0.341) 0.348 0.338

3	(0.659) 0.652 0.662

Locus 12424 :

2 alleles

98.8% missing data

1	(0.575) 0.564 0.579

2	(0.425) 0.436 0.421

Locus 12425 :

2 alleles

99.2% missing data

1	(0.457) 0.457 0.462

4	(0.543) 0.543 0.538

Locus 12426 :

2 alleles

99.2% missing data

4	(0.722) 0.725 0.719

1	(0.278) 0.275 0.281

Locus 12427 :

3 alleles

98.8% missing data

3	(0.411) 0.406 0.418

1	(0.370) 0.372 0.364

4	(0.219) 0.222 0.219

Locus 12428 :

2 alleles

99.2% missing data

1	(0.387) 0.390 0.389

3	(0.613) 0.610 0.611

Locus 12429 :

2 alleles

92.6% missing data

2	(0.137) 0.136 0.123

3	(0.863) 0.864 0.877

Locus 12430 :

2 alleles

99.2% missing data

4	(0.503) 0.492 0.501

2	(0.497) 0.508 0.499

Locus 12431 :

2 alleles

99.2% missing data

2	(0.534) 0.531 0.536

1	(0.466) 0.469 0.464

Locus 12432 :

2 alleles

99.2% missing data

1	(0.461) 0.462 0.460

4	(0.539) 0.538 0.540

Locus 12433 :

2 alleles

98.8% missing data

1	(0.507) 0.508 0.505

3	(0.493) 0.492 0.495

Locus 12434 :

2 alleles

98.4% missing data

3	(0.791) 0.791 0.796

1	(0.209) 0.209 0.204

Locus 12435 :

2 alleles

98.8% missing data

2	(0.500) 0.504 0.504

4	(0.500) 0.496 0.496

Locus 12436 :

2 alleles

99.2% missing data

2	(0.670) 0.670 0.672

4	(0.330) 0.330 0.328

Locus 12437 :

2 alleles

99.2% missing data

2	(0.583) 0.587 0.576

1	(0.417) 0.413 0.424

Locus 12438 :

2 alleles

99.2% missing data

3	(0.569) 0.572 0.575

1	(0.431) 0.428 0.425

Locus 12439 :

2 alleles

99.2% missing data

2	(0.438) 0.433 0.438

4	(0.562) 0.567 0.562

Locus 12440 :

2 alleles

99.2% missing data

1	(0.702) 0.707 0.692

2	(0.298) 0.293 0.308

Locus 12441 :

2 alleles

98.4% missing data

2	(0.250) 0.240 0.262

1	(0.750) 0.760 0.738

Locus 12442 :

2 alleles

99.2% missing data

2	(0.493) 0.494 0.489

3	(0.507) 0.506 0.511

Locus 12443 :

2 alleles

99.2% missing data

4	(0.290) 0.289 0.288

2	(0.710) 0.711 0.712

Locus 12444 :

2 alleles

98.8% missing data

1	(0.390) 0.380 0.394

3	(0.610) 0.620 0.606

Locus 12445 :

2 alleles

2.7% missing data

4	(0.988) 0.992 0.993

3	(0.012) 0.008 0.007

Locus 12446 :

2 alleles

99.2% missing data

3	(0.460) 0.461 0.451

1	(0.540) 0.539 0.549

Locus 12447 :

2 alleles

98.0% missing data

3	(0.393) 0.396 0.392

1	(0.607) 0.604 0.608

Locus 12448 :

2 alleles

99.2% missing data

3	(0.608) 0.611 0.601

2	(0.392) 0.389 0.399

Locus 12449 :

2 alleles

99.2% missing data

2	(0.514) 0.512 0.518

3	(0.486) 0.488 0.482

Locus 12450 :

2 alleles

93.0% missing data

2	(0.905) 0.899 0.917

4	(0.095) 0.101 0.083

Locus 12451 :

2 alleles

99.2% missing data

1	(0.324) 0.321 0.327

2	(0.676) 0.679 0.673

Locus 12452 :

2 alleles

99.2% missing data

1	(0.248) 0.246 0.251

3	(0.752) 0.754 0.749

Locus 12453 :

2 alleles

99.2% missing data

4	(0.205) 0.206 0.199

1	(0.795) 0.794 0.801

Locus 12454 :

2 alleles

98.8% missing data

3	(0.570) 0.564 0.579

4	(0.430) 0.436 0.421

Locus 12455 :

2 alleles

99.2% missing data

2	(0.547) 0.539 0.552

1	(0.453) 0.461 0.448

Locus 12456 :

2 alleles

99.2% missing data

4	(0.484) 0.484 0.476

2	(0.516) 0.516 0.524

Locus 12457 :

2 alleles

99.2% missing data

4	(0.439) 0.433 0.443

3	(0.561) 0.567 0.557

Locus 12458 :

2 alleles

93.4% missing data

3	(0.926) 0.926 0.930

2	(0.074) 0.074 0.070

Locus 12459 :

2 alleles

99.2% missing data

3	(0.201) 0.198 0.198

1	(0.799) 0.802 0.802

Locus 12460 :

2 alleles

0.0% missing data

3	(0.959) 0.969 0.958

2	(0.041) 0.031 0.042

Locus 12461 :

2 alleles

99.2% missing data

1	(0.244) 0.248 0.251

3	(0.756) 0.752 0.749

Locus 12462 :

2 alleles

99.2% missing data

3	(0.535) 0.538 0.528

4	(0.465) 0.462 0.472

Locus 12463 :

2 alleles

99.2% missing data

3	(0.390) 0.393 0.388

1	(0.610) 0.607 0.612

Locus 12464 :

2 alleles

99.2% missing data

3	(0.646) 0.651 0.641

2	(0.354) 0.349 0.359

Locus 12465 :

2 alleles

98.8% missing data

2	(0.327) 0.321 0.337

1	(0.673) 0.679 0.663

Locus 12466 :

2 alleles

99.2% missing data

2	(0.715) 0.714 0.721

4	(0.285) 0.286 0.279

Locus 12467 :

2 alleles

99.2% missing data

2	(0.679) 0.685 0.675

4	(0.321) 0.315 0.325

Locus 12468 :

2 alleles

99.2% missing data

2	(0.836) 0.825 0.840

4	(0.164) 0.175 0.160

Locus 12469 :

2 alleles

99.2% missing data

4	(0.650) 0.641 0.653

1	(0.350) 0.359 0.347

Locus 12470 :

2 alleles

99.2% missing data

1	(0.632) 0.635 0.634

3	(0.368) 0.365 0.366

Locus 12471 :

2 alleles

99.2% missing data

2	(0.344) 0.338 0.350

4	(0.656) 0.662 0.650

Locus 12472 :

2 alleles

99.2% missing data

3	(0.595) 0.593 0.598

4	(0.405) 0.407 0.402

Locus 12473 :

2 alleles

99.2% missing data

3	(0.512) 0.508 0.522

1	(0.488) 0.492 0.478

Locus 12474 :

2 alleles

99.2% missing data

4	(0.291) 0.297 0.285

3	(0.709) 0.703 0.715

Locus 12475 :

2 alleles

99.2% missing data

3	(0.418) 0.410 0.426

4	(0.582) 0.590 0.574

Locus 12476 :

2 alleles

94.9% missing data

4	(0.843) 0.839 0.857

3	(0.157) 0.161 0.143

Locus 12477 :

2 alleles

99.2% missing data

1	(0.656) 0.657 0.655

4	(0.344) 0.343 0.345

Locus 12478 :

2 alleles

99.2% missing data

4	(0.436) 0.431 0.444

3	(0.564) 0.569 0.556

Locus 12479 :

2 alleles

99.2% missing data

4	(0.682) 0.676 0.682

1	(0.318) 0.324 0.318

Locus 12480 :

2 alleles

98.8% missing data

2	(0.704) 0.703 0.702

1	(0.296) 0.297 0.298

Locus 12481 :

2 alleles

99.2% missing data

4	(0.675) 0.672 0.677

3	(0.325) 0.328 0.323

Locus 12482 :

2 alleles

99.2% missing data

3	(0.593) 0.599 0.592

4	(0.407) 0.401 0.408

Locus 12483 :

2 alleles

98.4% missing data

2	(0.832) 0.836 0.830

1	(0.168) 0.164 0.170

Locus 12484 :

2 alleles

99.2% missing data

1	(0.407) 0.403 0.414

3	(0.593) 0.597 0.586

Locus 12485 :

2 alleles

91.4% missing data

3	(0.775) 0.747 0.805

1	(0.225) 0.253 0.195

Locus 12486 :

2 alleles

91.0% missing data

4	(0.878) 0.902 0.869

3	(0.122) 0.098 0.131

Locus 12487 :

2 alleles

98.8% missing data

3	(0.221) 0.214 0.226

2	(0.779) 0.786 0.774

Locus 12488 :

2 alleles

8.6% missing data

2	(0.965) 0.930 0.989

3	(0.035) 0.070 0.011

Locus 12489 :

2 alleles

99.2% missing data

4	(0.580) 0.578 0.591

3	(0.420) 0.422 0.409

Locus 12490 :

2 alleles

99.2% missing data

1	(0.750) 0.748 0.753

4	(0.250) 0.252 0.247

Locus 12491 :

2 alleles

99.2% missing data

4	(0.630) 0.623 0.640

3	(0.370) 0.377 0.360

Locus 12492 :

2 alleles

99.2% missing data

1	(0.193) 0.189 0.191

3	(0.807) 0.811 0.809

Locus 12493 :

2 alleles

99.2% missing data

2	(0.767) 0.770 0.759

1	(0.233) 0.230 0.241

Locus 12494 :

2 alleles

99.2% missing data

1	(0.521) 0.523 0.521

3	(0.479) 0.477 0.479

Locus 12495 :

2 alleles

97.7% missing data

1	(0.610) 0.631 0.603

3	(0.390) 0.369 0.397

Locus 12496 :

2 alleles

99.2% missing data

1	(0.652) 0.643 0.658

2	(0.348) 0.357 0.342

Locus 12497 :

2 alleles

97.3% missing data

1	(0.521) 0.522 0.542

2	(0.479) 0.478 0.458

Locus 12498 :

2 alleles

99.2% missing data

1	(0.517) 0.517 0.513

3	(0.483) 0.483 0.487

Locus 12499 :

2 alleles

99.2% missing data

3	(0.757) 0.755 0.761

2	(0.243) 0.245 0.239

Locus 12500 :

2 alleles

99.2% missing data

4	(0.550) 0.544 0.556

3	(0.450) 0.456 0.444

Locus 12501 :

2 alleles

99.2% missing data

2	(0.838) 0.836 0.838

4	(0.162) 0.164 0.162

Locus 12502 :

2 alleles

99.2% missing data

1	(0.511) 0.514 0.505

2	(0.489) 0.486 0.495

Locus 12503 :

2 alleles

99.2% missing data

4	(0.291) 0.298 0.292

2	(0.709) 0.702 0.708

Locus 12504 :

2 alleles

99.2% missing data

3	(0.368) 0.367 0.368

2	(0.632) 0.633 0.632

Locus 12505 :

2 alleles

98.8% missing data

1	(0.388) 0.386 0.397

3	(0.612) 0.614 0.603

Locus 12506 :

3 alleles

97.7% missing data

1	(0.570) 0.573 0.572

2	(0.225) 0.220 0.228

4	(0.205) 0.206 0.200

Locus 12507 :

2 alleles

99.2% missing data

4	(0.618) 0.614 0.620

1	(0.382) 0.386 0.380

Locus 12508 :

2 alleles

97.7% missing data

4	(0.535) 0.526 0.533

1	(0.465) 0.474 0.467

Locus 12509 :

2 alleles

96.1% missing data

4	(0.204) 0.193 0.207

3	(0.796) 0.807 0.793

Locus 12510 :

3 alleles

97.3% missing data

4	(0.338) 0.345 0.344

1	(0.356) 0.351 0.357

2	(0.306) 0.304 0.299

Locus 12511 :

2 alleles

98.8% missing data

3	(0.392) 0.391 0.390

1	(0.608) 0.609 0.610

Locus 12512 :

3 alleles

97.7% missing data

1	(0.573) 0.584 0.572

2	(0.177) 0.169 0.179

4	(0.251) 0.247 0.248

Locus 12513 :

2 alleles

99.2% missing data

3	(0.716) 0.713 0.721

4	(0.284) 0.287 0.279

Locus 12514 :

2 alleles

99.2% missing data

1	(0.697) 0.696 0.694

4	(0.303) 0.304 0.306

Locus 12515 :

3 alleles

88.3% missing data

1	(0.796) 0.804 0.804

2	(0.140) 0.133 0.139

4	(0.064) 0.063 0.057

Locus 12516 :

2 alleles

99.2% missing data

4	(0.489) 0.493 0.487

1	(0.511) 0.507 0.513

Locus 12517 :

2 alleles

92.6% missing data

4	(0.881) 0.874 0.899

2	(0.119) 0.126 0.101

Locus 12518 :

3 alleles

96.5% missing data

1	(0.654) 0.667 0.650

4	(0.169) 0.154 0.179

2	(0.178) 0.179 0.171

Locus 12519 :

2 alleles

98.4% missing data

3	(0.498) 0.501 0.496

1	(0.502) 0.499 0.504

Locus 12520 :

2 alleles

99.2% missing data

2	(0.577) 0.571 0.582

1	(0.423) 0.429 0.418

Locus 12521 :

2 alleles

98.4% missing data

1	(0.650) 0.650 0.652

4	(0.350) 0.350 0.348

Locus 12522 :

2 alleles

99.2% missing data

2	(0.415) 0.408 0.422

4	(0.585) 0.592 0.578

Locus 12523 :

2 alleles

98.8% missing data

4	(0.415) 0.417 0.419

1	(0.585) 0.583 0.581

Locus 12524 :

2 alleles

99.2% missing data

4	(0.657) 0.665 0.648

2	(0.343) 0.335 0.352

Locus 12525 :

2 alleles

93.0% missing data

1	(0.908) 0.899 0.924

4	(0.092) 0.101 0.076

Locus 12526 :

2 alleles

98.4% missing data

3	(0.505) 0.510 0.502

1	(0.495) 0.490 0.498

Locus 12527 :

3 alleles

92.2% missing data

4	(0.816) 0.817 0.820

1	(0.053) 0.052 0.051

2	(0.132) 0.132 0.129

Locus 12528 :

2 alleles

98.8% missing data

3	(0.524) 0.518 0.533

4	(0.476) 0.482 0.467

Locus 12529 :

2 alleles

99.2% missing data

2	(0.414) 0.409 0.421

1	(0.586) 0.591 0.579

Locus 12530 :

2 alleles

99.2% missing data

1	(0.459) 0.458 0.464

2	(0.541) 0.542 0.536

Locus 12531 :

2 alleles

99.2% missing data

1	(0.438) 0.436 0.444

4	(0.562) 0.564 0.556

Locus 12532 :

2 alleles

14.5% missing data

1	(0.989) 0.981 0.997

2	(0.011) 0.019 0.003

Locus 12533 :

2 alleles

98.0% missing data

2	(0.797) 0.794 0.799

1	(0.203) 0.206 0.201

Locus 12534 :

2 alleles

97.7% missing data

1	(0.154) 0.162 0.147

2	(0.846) 0.838 0.853

Locus 12535 :

2 alleles

98.8% missing data

1	(0.823) 0.818 0.827

2	(0.177) 0.182 0.173

Locus 12536 :

3 alleles

98.8% missing data

2	(0.407) 0.407 0.408

1	(0.271) 0.278 0.268

4	(0.322) 0.315 0.324

Locus 12537 :

2 alleles

99.2% missing data

2	(0.490) 0.489 0.490

1	(0.510) 0.511 0.510

Locus 12538 :

3 alleles

98.8% missing data

4	(0.584) 0.591 0.578

2	(0.130) 0.126 0.137

1	(0.287) 0.283 0.286

Locus 12539 :

2 alleles

99.2% missing data

1	(0.510) 0.512 0.510

2	(0.490) 0.488 0.490

Locus 12540 :

2 alleles

98.8% missing data

1	(0.534) 0.531 0.542

4	(0.466) 0.469 0.458

Locus 12541 :

2 alleles

99.2% missing data

4	(0.377) 0.371 0.377

1	(0.623) 0.629 0.623

Locus 12542 :

2 alleles

99.2% missing data

2	(0.566) 0.561 0.571

1	(0.434) 0.439 0.429

Locus 12543 :

2 alleles

96.5% missing data

3	(0.875) 0.883 0.872

4	(0.125) 0.117 0.128

Locus 12544 :

2 alleles

96.1% missing data

1	(0.844) 0.836 0.856

4	(0.156) 0.164 0.144

Locus 12545 :

2 alleles

98.8% missing data

1	(0.387) 0.383 0.400

2	(0.613) 0.617 0.600

Locus 12546 :

2 alleles

8.2% missing data

3	(0.985) 0.978 0.995

1	(0.015) 0.022 0.005

Locus 12547 :

3 alleles

96.1% missing data

1	(0.151) 0.138 0.158

4	(0.655) 0.677 0.650

2	(0.194) 0.185 0.192

Locus 12548 :

2 alleles

98.8% missing data

3	(0.393) 0.387 0.394

1	(0.607) 0.613 0.606

Locus 12549 :

2 alleles

99.2% missing data

2	(0.628) 0.626 0.626

1	(0.372) 0.374 0.374

Locus 12550 :

2 alleles

99.2% missing data

3	(0.421) 0.420 0.427

1	(0.579) 0.580 0.573

Locus 12551 :

2 alleles

99.2% missing data

1	(0.368) 0.363 0.377

2	(0.632) 0.637 0.623

Locus 12552 :

2 alleles

98.0% missing data

2	(0.591) 0.592 0.596

4	(0.409) 0.408 0.404

Locus 12553 :

3 alleles

95.3% missing data

1	(0.275) 0.273 0.282

3	(0.522) 0.536 0.511

4	(0.204) 0.191 0.207

Locus 12554 :

4 alleles

93.8% missing data

3	(0.174) 0.177 0.168

1	(0.629) 0.622 0.654

2	(0.104) 0.104 0.096

4	(0.093) 0.097 0.081

Locus 12555 :

2 alleles

99.2% missing data

3	(0.428) 0.431 0.428

1	(0.572) 0.569 0.572

Locus 12556 :

2 alleles

99.2% missing data

2	(0.764) 0.763 0.760

3	(0.236) 0.237 0.240

Locus 12557 :

3 alleles

98.8% missing data

3	(0.254) 0.252 0.258

1	(0.287) 0.285 0.291

4	(0.459) 0.463 0.451

Locus 12558 :

3 alleles

97.3% missing data

3	(0.269) 0.264 0.269

1	(0.569) 0.581 0.564

2	(0.163) 0.156 0.167

Locus 12559 :

2 alleles

99.2% missing data

1	(0.512) 0.517 0.517

4	(0.488) 0.483 0.483

Locus 12560 :

2 alleles

98.8% missing data

1	(0.515) 0.518 0.520

2	(0.485) 0.482 0.480

Locus 12561 :

2 alleles

99.2% missing data

3	(0.379) 0.377 0.383

1	(0.621) 0.623 0.617

Locus 12562 :

2 alleles

99.2% missing data

2	(0.271) 0.271 0.269

1	(0.729) 0.729 0.731

Locus 12563 :

3 alleles

98.8% missing data

1	(0.301) 0.302 0.298

2	(0.429) 0.431 0.427

3	(0.270) 0.267 0.275

Locus 12564 :

2 alleles

99.2% missing data

2	(0.616) 0.612 0.617

3	(0.384) 0.388 0.383

Locus 12565 :

2 alleles

98.0% missing data

3	(0.367) 0.360 0.368

4	(0.633) 0.640 0.632

Locus 12566 :

2 alleles

99.2% missing data

4	(0.435) 0.431 0.435

3	(0.565) 0.569 0.565

Locus 12567 :

2 alleles

99.2% missing data

3	(0.318) 0.312 0.322

1	(0.682) 0.688 0.678

Locus 12568 :

2 alleles

98.4% missing data

3	(0.806) 0.799 0.811

1	(0.194) 0.201 0.189

Locus 12569 :

2 alleles

99.2% missing data

2	(0.334) 0.332 0.341

4	(0.666) 0.668 0.659

Locus 12570 :

2 alleles

0.0% missing data

1	(0.990) 0.985 0.997

4	(0.010) 0.015 0.003

Locus 12571 :

2 alleles

91.0% missing data

3	(0.918) 0.929 0.909

2	(0.082) 0.071 0.091

Locus 12572 :

2 alleles

93.0% missing data

1	(0.890) 0.882 0.906

4	(0.110) 0.118 0.094

Locus 12573 :

2 alleles

99.2% missing data

1	(0.405) 0.406 0.409

4	(0.595) 0.594 0.591

Locus 12574 :

2 alleles

98.4% missing data

4	(0.155) 0.157 0.152

3	(0.845) 0.843 0.848

Locus 12575 :

2 alleles

99.2% missing data

3	(0.511) 0.516 0.513

2	(0.489) 0.484 0.487

Locus 12576 :

2 alleles

98.8% missing data

3	(0.248) 0.261 0.247

1	(0.752) 0.739 0.753

Locus 12577 :

2 alleles

98.8% missing data

3	(0.612) 0.609 0.616

2	(0.388) 0.391 0.384

Locus 12578 :

3 alleles

95.3% missing data

1	(0.625) 0.646 0.617

2	(0.224) 0.209 0.240

3	(0.150) 0.145 0.143

Locus 12579 :

2 alleles

99.2% missing data

3	(0.468) 0.471 0.463

4	(0.532) 0.529 0.537

Locus 12580 :

2 alleles

99.2% missing data

1	(0.352) 0.351 0.352

4	(0.648) 0.649 0.648

Locus 12581 :

2 alleles

0.0% missing data

4	(0.983) 0.987 0.989

3	(0.017) 0.013 0.011

Locus 12582 :

2 alleles

98.8% missing data

4	(0.525) 0.529 0.523

3	(0.475) 0.471 0.477

Locus 12583 :

3 alleles

90.2% missing data

1	(0.100) 0.081 0.117

3	(0.857) 0.887 0.832

2	(0.043) 0.032 0.051

Locus 12584 :

3 alleles

98.8% missing data

4	(0.272) 0.269 0.277

1	(0.417) 0.415 0.414

2	(0.311) 0.316 0.309

Locus 12585 :

2 alleles

99.2% missing data

3	(0.601) 0.592 0.602

2	(0.399) 0.408 0.398

Locus 12586 :

2 alleles

99.2% missing data

1	(0.784) 0.785 0.784

4	(0.216) 0.215 0.216

Locus 12587 :

2 alleles

99.2% missing data

1	(0.527) 0.529 0.526

2	(0.473) 0.471 0.474

Locus 12588 :

4 alleles

98.4% missing data

1	(0.302) 0.305 0.303

3	(0.217) 0.214 0.221

4	(0.315) 0.311 0.321

2	(0.165) 0.170 0.155

Locus 12589 :

2 alleles

99.2% missing data

2	(0.469) 0.469 0.468

3	(0.531) 0.531 0.532

Locus 12590 :

3 alleles

97.7% missing data

2	(0.182) 0.179 0.182

3	(0.714) 0.723 0.708

1	(0.103) 0.098 0.110

Locus 12591 :

2 alleles

99.2% missing data

2	(0.235) 0.243 0.234

3	(0.765) 0.757 0.766

Locus 12592 :

2 alleles

94.9% missing data

1	(0.910) 0.901 0.925

2	(0.090) 0.099 0.075

Locus 12593 :

3 alleles

96.1% missing data

1	(0.384) 0.389 0.379

4	(0.469) 0.465 0.479

3	(0.147) 0.146 0.143

Locus 12594 :

2 alleles

99.2% missing data

3	(0.428) 0.420 0.433

1	(0.572) 0.580 0.567

Locus 12595 :

2 alleles

98.8% missing data

3	(0.610) 0.603 0.611

2	(0.390) 0.397 0.389

Locus 12596 :

2 alleles

99.2% missing data

1	(0.561) 0.564 0.557

3	(0.439) 0.436 0.443

Locus 12597 :

2 alleles

98.8% missing data

1	(0.279) 0.272 0.280

4	(0.721) 0.728 0.720

Locus 12598 :

2 alleles

98.4% missing data

2	(0.715) 0.702 0.722

1	(0.285) 0.298 0.278

Locus 12599 :

2 alleles

93.0% missing data

3	(0.885) 0.886 0.894

2	(0.115) 0.114 0.106

Locus 12600 :

2 alleles

98.8% missing data

3	(0.187) 0.185 0.189

1	(0.813) 0.815 0.811

Locus 12601 :

2 alleles

91.0% missing data

1	(0.912) 0.925 0.904

3	(0.088) 0.075 0.096

Locus 12602 :

2 alleles

99.2% missing data

2	(0.487) 0.482 0.489

1	(0.513) 0.518 0.511

Locus 12603 :

2 alleles

99.2% missing data

4	(0.505) 0.506 0.500

2	(0.495) 0.494 0.500

Locus 12604 :

3 alleles

98.8% missing data

1	(0.314) 0.307 0.321

2	(0.168) 0.170 0.167

3	(0.518) 0.523 0.512

Locus 12605 :

2 alleles

99.2% missing data

4	(0.758) 0.753 0.756

1	(0.242) 0.247 0.244

Locus 12606 :

2 alleles

99.2% missing data

2	(0.526) 0.522 0.533

1	(0.474) 0.478 0.467

Locus 12607 :

2 alleles

99.2% missing data

4	(0.714) 0.712 0.717

1	(0.286) 0.288 0.283

Locus 12608 :

2 alleles

98.8% missing data

4	(0.630) 0.634 0.620

1	(0.370) 0.366 0.380

Locus 12609 :

2 alleles

99.2% missing data

3	(0.449) 0.449 0.458

2	(0.551) 0.551 0.542

Locus 12610 :

2 alleles

99.2% missing data

1	(0.557) 0.550 0.559

2	(0.443) 0.450 0.441

Locus 12611 :

2 alleles

8.2% missing data

3	(0.987) 0.979 0.996

1	(0.013) 0.021 0.004

Locus 12612 :

2 alleles

98.8% missing data

2	(0.697) 0.703 0.692

4	(0.303) 0.297 0.308

Locus 12613 :

2 alleles

98.8% missing data

2	(0.463) 0.463 0.465

1	(0.537) 0.537 0.535

Locus 12614 :

3 alleles

98.8% missing data

3	(0.192) 0.197 0.191

1	(0.243) 0.239 0.254

2	(0.565) 0.564 0.554

Locus 12615 :

2 alleles

99.2% missing data

1	(0.370) 0.369 0.369

3	(0.630) 0.631 0.631

Locus 12616 :

2 alleles

93.8% missing data

2	(0.822) 0.811 0.841

3	(0.178) 0.189 0.159

Locus 12617 :

2 alleles

99.2% missing data

2	(0.670) 0.669 0.666

3	(0.330) 0.331 0.334

Locus 12618 :

2 alleles

98.8% missing data

1	(0.590) 0.591 0.595

3	(0.410) 0.409 0.405

Locus 12619 :

2 alleles

98.8% missing data

3	(0.271) 0.263 0.279

1	(0.729) 0.737 0.721

Locus 12620 :

2 alleles

98.8% missing data

1	(0.410) 0.408 0.413

3	(0.590) 0.592 0.587

Locus 12621 :

2 alleles

98.8% missing data

1	(0.542) 0.540 0.546

2	(0.458) 0.460 0.454

Locus 12622 :

2 alleles

98.8% missing data

1	(0.485) 0.491 0.486

2	(0.515) 0.509 0.514

Locus 12623 :

2 alleles

99.2% missing data

3	(0.442) 0.436 0.448

4	(0.558) 0.564 0.552

Locus 12624 :

2 alleles

99.2% missing data

3	(0.531) 0.531 0.532

4	(0.469) 0.469 0.468

Locus 12625 :

2 alleles

99.2% missing data

3	(0.653) 0.647 0.656

4	(0.347) 0.353 0.344

Locus 12626 :

2 alleles

98.8% missing data

3	(0.572) 0.559 0.579

4	(0.428) 0.441 0.421

Locus 12627 :

2 alleles

99.2% missing data

3	(0.481) 0.487 0.474

1	(0.519) 0.513 0.526

Locus 12628 :

2 alleles

99.2% missing data

1	(0.320) 0.321 0.324

2	(0.680) 0.679 0.676

Locus 12629 :

2 alleles

99.2% missing data

4	(0.670) 0.664 0.677

1	(0.330) 0.336 0.323

Locus 12630 :

2 alleles

98.8% missing data

3	(0.245) 0.244 0.250

1	(0.755) 0.756 0.750

Locus 12631 :

3 alleles

98.4% missing data

2	(0.415) 0.417 0.410

3	(0.348) 0.347 0.344

1	(0.237) 0.236 0.246

Locus 12632 :

2 alleles

99.2% missing data

3	(0.556) 0.558 0.564

1	(0.444) 0.442 0.436

Locus 12633 :

2 alleles

98.4% missing data

2	(0.714) 0.717 0.712

3	(0.286) 0.283 0.288

Locus 12634 :

2 alleles

99.2% missing data

3	(0.359) 0.353 0.364

4	(0.641) 0.647 0.636

Locus 12635 :

2 alleles

99.2% missing data

1	(0.865) 0.856 0.870

3	(0.135) 0.144 0.130

Locus 12636 :

2 alleles

99.2% missing data

1	(0.712) 0.704 0.719

3	(0.288) 0.296 0.281

Locus 12637 :

2 alleles

98.8% missing data

3	(0.451) 0.449 0.455

1	(0.549) 0.551 0.545

Locus 12638 :

2 alleles

99.2% missing data

3	(0.518) 0.512 0.520

1	(0.482) 0.488 0.480

Locus 12639 :

2 alleles

99.2% missing data

2	(0.816) 0.817 0.815

4	(0.184) 0.183 0.185

Locus 12640 :

2 alleles

99.2% missing data

2	(0.364) 0.362 0.370

1	(0.636) 0.638 0.630

Locus 12641 :

2 alleles

98.0% missing data

1	(0.729) 0.723 0.735

2	(0.271) 0.277 0.265

Locus 12642 :

3 alleles

98.0% missing data

4	(0.337) 0.343 0.326

2	(0.277) 0.277 0.272

1	(0.386) 0.380 0.402

Locus 12643 :

2 alleles

99.2% missing data

2	(0.366) 0.362 0.376

1	(0.634) 0.638 0.624

Locus 12644 :

2 alleles

99.2% missing data

1	(0.477) 0.478 0.476

4	(0.523) 0.522 0.524

Locus 12645 :

2 alleles

99.2% missing data

2	(0.354) 0.351 0.359

1	(0.646) 0.649 0.641

Locus 12646 :

2 alleles

99.2% missing data

4	(0.612) 0.615 0.609

2	(0.388) 0.385 0.391

Locus 12647 :

2 alleles

0.0% missing data

4	(0.148) 0.129 0.163

3	(0.852) 0.871 0.837

Locus 12648 :

2 alleles

96.5% missing data

1	(0.292) 0.275 0.293

4	(0.708) 0.725 0.707

Locus 12649 :

2 alleles

99.2% missing data

1	(0.493) 0.483 0.495

4	(0.507) 0.517 0.505

Locus 12650 :

2 alleles

99.2% missing data

1	(0.560) 0.557 0.561

4	(0.440) 0.443 0.439

Locus 12651 :

2 alleles

7.0% missing data

4	(0.988) 0.983 0.996

3	(0.012) 0.017 0.004

Locus 12652 :

2 alleles

98.8% missing data

1	(0.679) 0.677 0.673

2	(0.321) 0.323 0.327

Locus 12653 :

2 alleles

98.4% missing data

4	(0.648) 0.652 0.650

1	(0.352) 0.348 0.350

Locus 12654 :

2 alleles

98.8% missing data

1	(0.780) 0.787 0.780

4	(0.220) 0.213 0.220

Locus 12655 :

2 alleles

99.2% missing data

1	(0.846) 0.850 0.841

3	(0.154) 0.150 0.159

Locus 12656 :

2 alleles

98.8% missing data

2	(0.697) 0.694 0.693

1	(0.303) 0.306 0.307

Locus 12657 :

2 alleles

93.0% missing data

3	(0.896) 0.892 0.906

4	(0.104) 0.108 0.094

Locus 12658 :

2 alleles

90.2% missing data

4	(0.845) 0.876 0.815

3	(0.155) 0.124 0.185

Locus 12659 :

2 alleles

99.2% missing data

3	(0.194) 0.202 0.188

1	(0.806) 0.798 0.812

Locus 12660 :

2 alleles

99.2% missing data

2	(0.227) 0.230 0.226

3	(0.773) 0.770 0.774

Locus 12661 :

2 alleles

99.2% missing data

3	(0.614) 0.622 0.610

2	(0.386) 0.378 0.390

Locus 12662 :

3 alleles

98.8% missing data

2	(0.466) 0.467 0.458

4	(0.251) 0.245 0.255

1	(0.283) 0.289 0.287

Locus 12663 :

2 alleles

92.2% missing data

1	(0.834) 0.811 0.860

4	(0.166) 0.189 0.140

Locus 12664 :

2 alleles

8.2% missing data

1	(0.986) 0.978 0.996

2	(0.014) 0.022 0.004

Locus 12665 :

2 alleles

8.6% missing data

2	(0.982) 0.975 0.995

3	(0.018) 0.025 0.005

Locus 12666 :

2 alleles

99.2% missing data

3	(0.361) 0.363 0.363

1	(0.639) 0.637 0.637

Locus 12667 :

2 alleles

98.8% missing data

1	(0.772) 0.765 0.781

2	(0.228) 0.235 0.219

Locus 12668 :

2 alleles

98.8% missing data

2	(0.440) 0.435 0.441

1	(0.560) 0.565 0.559

Locus 12669 :

2 alleles

99.2% missing data

3	(0.532) 0.534 0.529

1	(0.468) 0.466 0.471

Locus 12670 :

2 alleles

98.8% missing data

1	(0.664) 0.663 0.666

3	(0.336) 0.337 0.334

Locus 12671 :

2 alleles

96.1% missing data

4	(0.778) 0.788 0.777

1	(0.222) 0.212 0.223

Locus 12672 :

2 alleles

99.2% missing data

1	(0.277) 0.274 0.279

2	(0.723) 0.726 0.721

Locus 12673 :

3 alleles

98.8% missing data

1	(0.274) 0.276 0.278

3	(0.360) 0.364 0.358

2	(0.366) 0.361 0.364

Locus 12674 :

2 alleles

91.0% missing data

4	(0.935) 0.947 0.929

2	(0.065) 0.053 0.071

Locus 12675 :

2 alleles

99.2% missing data

4	(0.627) 0.627 0.625

1	(0.373) 0.373 0.375

Locus 12676 :

2 alleles

99.2% missing data

3	(0.356) 0.356 0.355

4	(0.644) 0.644 0.645

Locus 12677 :

2 alleles

99.2% missing data

1	(0.510) 0.500 0.514

4	(0.490) 0.500 0.486

Locus 12678 :

2 alleles

99.2% missing data

3	(0.364) 0.359 0.366

1	(0.636) 0.641 0.634

Locus 12679 :

2 alleles

98.8% missing data

4	(0.710) 0.710 0.709

1	(0.290) 0.290 0.291

Locus 12680 :

2 alleles

99.2% missing data

1	(0.594) 0.589 0.597

2	(0.406) 0.411 0.403

Locus 12681 :

3 alleles

98.8% missing data

2	(0.425) 0.421 0.421

1	(0.283) 0.284 0.292

3	(0.292) 0.296 0.287

Locus 12682 :

2 alleles

99.2% missing data

2	(0.714) 0.711 0.710

3	(0.286) 0.289 0.290

Locus 12683 :

2 alleles

99.2% missing data

1	(0.349) 0.359 0.343

3	(0.651) 0.641 0.657

Locus 12684 :

2 alleles

96.5% missing data

3	(0.687) 0.673 0.702

1	(0.313) 0.327 0.298

Locus 12685 :

2 alleles

99.2% missing data

1	(0.285) 0.281 0.289

3	(0.715) 0.719 0.711

Locus 12686 :

2 alleles

96.5% missing data

3	(0.139) 0.132 0.143

1	(0.861) 0.868 0.857

Locus 12687 :

2 alleles

99.2% missing data

3	(0.254) 0.256 0.246

1	(0.746) 0.744 0.754

Locus 12688 :

2 alleles

98.0% missing data

3	(0.660) 0.660 0.660

1	(0.340) 0.340 0.340

Locus 12689 :

3 alleles

92.6% missing data

2	(0.795) 0.788 0.806

1	(0.109) 0.109 0.108

3	(0.096) 0.103 0.086

Locus 12690 :

2 alleles

98.8% missing data

4	(0.609) 0.605 0.610

1	(0.391) 0.395 0.390

Locus 12691 :

2 alleles

98.8% missing data

1	(0.572) 0.577 0.570

3	(0.428) 0.423 0.430

Locus 12692 :

2 alleles

90.6% missing data

1	(0.130) 0.106 0.153

2	(0.870) 0.894 0.847

Locus 12693 :

2 alleles

99.2% missing data

2	(0.362) 0.361 0.371

4	(0.638) 0.639 0.629

Locus 12694 :

2 alleles

98.0% missing data

1	(0.674) 0.665 0.685

3	(0.326) 0.335 0.315

Locus 12695 :

2 alleles

99.2% missing data

1	(0.426) 0.420 0.427

3	(0.574) 0.580 0.573

Locus 12696 :

2 alleles

99.2% missing data

1	(0.367) 0.368 0.369

2	(0.633) 0.632 0.631

Locus 12697 :

2 alleles

99.2% missing data

4	(0.417) 0.415 0.423

2	(0.583) 0.585 0.577

Locus 12698 :

2 alleles

99.2% missing data

1	(0.353) 0.353 0.354

2	(0.647) 0.647 0.646

Locus 12699 :

2 alleles

96.5% missing data

1	(0.207) 0.205 0.206

4	(0.793) 0.795 0.794

Locus 12700 :

2 alleles

99.2% missing data

1	(0.558) 0.558 0.560

4	(0.442) 0.442 0.440

Locus 12701 :

2 alleles

99.2% missing data

1	(0.544) 0.545 0.549

4	(0.456) 0.455 0.451

Locus 12702 :

2 alleles

99.2% missing data

2	(0.400) 0.395 0.402

4	(0.600) 0.605 0.598

Locus 12703 :

2 alleles

96.5% missing data

3	(0.880) 0.877 0.886

2	(0.120) 0.123 0.114

Locus 12704 :

2 alleles

99.2% missing data

4	(0.342) 0.336 0.347

2	(0.658) 0.664 0.653

Locus 12705 :

2 alleles

99.2% missing data

4	(0.409) 0.411 0.410

1	(0.591) 0.589 0.590

Locus 12706 :

2 alleles

99.2% missing data

1	(0.847) 0.840 0.847

2	(0.153) 0.160 0.153

Locus 12707 :

3 alleles

98.8% missing data

4	(0.289) 0.284 0.298

1	(0.251) 0.252 0.248

2	(0.460) 0.463 0.454

Locus 12708 :

2 alleles

11.7% missing data

4	(0.983) 0.975 0.995

2	(0.017) 0.025 0.005

Locus 12709 :

2 alleles

98.4% missing data

3	(0.858) 0.853 0.861

4	(0.142) 0.147 0.139

Locus 12710 :

2 alleles

99.2% missing data

3	(0.316) 0.313 0.322

4	(0.684) 0.687 0.678

Locus 12711 :

2 alleles

99.2% missing data

2	(0.444) 0.441 0.444

4	(0.556) 0.559 0.556

Locus 12712 :

2 alleles

99.2% missing data

2	(0.610) 0.614 0.601

4	(0.390) 0.386 0.399

Locus 12713 :

2 alleles

98.8% missing data

1	(0.427) 0.418 0.432

4	(0.573) 0.582 0.568

Locus 12714 :

2 alleles

0.0% missing data

1	(0.988) 0.983 0.996

3	(0.012) 0.017 0.004

Locus 12715 :

2 alleles

99.2% missing data

4	(0.264) 0.263 0.264

1	(0.736) 0.737 0.736

Locus 12716 :

2 alleles

99.2% missing data

4	(0.366) 0.370 0.369

2	(0.634) 0.630 0.631

Locus 12717 :

2 alleles

97.7% missing data

2	(0.451) 0.440 0.464

1	(0.549) 0.560 0.536

Locus 12718 :

2 alleles

97.3% missing data

4	(0.286) 0.277 0.289

3	(0.714) 0.723 0.711

Locus 12719 :

2 alleles

91.0% missing data

4	(0.927) 0.942 0.919

3	(0.073) 0.058 0.081

Locus 12720 :

2 alleles

99.2% missing data

2	(0.466) 0.467 0.458

1	(0.534) 0.533 0.542

Locus 12721 :

2 alleles

96.1% missing data

3	(0.882) 0.890 0.874

4	(0.118) 0.110 0.126

Locus 12722 :

2 alleles

99.2% missing data

1	(0.445) 0.442 0.455

2	(0.555) 0.558 0.545

Locus 12723 :

2 alleles

98.4% missing data

4	(0.260) 0.253 0.265

2	(0.740) 0.747 0.735

Locus 12724 :

2 alleles

99.2% missing data

1	(0.616) 0.616 0.617

2	(0.384) 0.384 0.383

Locus 12725 :

2 alleles

99.2% missing data

4	(0.604) 0.603 0.597

3	(0.396) 0.397 0.403

Locus 12726 :

2 alleles

99.2% missing data

3	(0.310) 0.310 0.316

1	(0.690) 0.690 0.684

Locus 12727 :

2 alleles

99.2% missing data

4	(0.448) 0.454 0.450

3	(0.552) 0.546 0.550

Locus 12728 :

2 alleles

98.0% missing data

1	(0.567) 0.571 0.558

4	(0.433) 0.429 0.442

Locus 12729 :

2 alleles

98.4% missing data

4	(0.288) 0.283 0.290

2	(0.712) 0.717 0.710

Locus 12730 :

2 alleles

98.8% missing data

2	(0.405) 0.414 0.410

1	(0.595) 0.586 0.590

Locus 12731 :

2 alleles

98.8% missing data

1	(0.551) 0.542 0.555

3	(0.449) 0.458 0.445

Locus 12732 :

2 alleles

98.8% missing data

3	(0.328) 0.321 0.329

4	(0.672) 0.679 0.671

Locus 12733 :

2 alleles

98.0% missing data

4	(0.574) 0.565 0.583

3	(0.426) 0.435 0.417

Locus 12734 :

2 alleles

98.8% missing data

3	(0.622) 0.633 0.619

2	(0.378) 0.367 0.381

Locus 12735 :

2 alleles

99.2% missing data

3	(0.348) 0.355 0.351

2	(0.652) 0.645 0.649

Locus 12736 :

2 alleles

99.2% missing data

2	(0.401) 0.401 0.412

1	(0.599) 0.599 0.588

Locus 12737 :

2 alleles

98.8% missing data

2	(0.415) 0.409 0.420

3	(0.585) 0.591 0.580

Locus 12738 :

2 alleles

98.4% missing data

2	(0.697) 0.705 0.694

3	(0.303) 0.295 0.306

Locus 12739 :

2 alleles

99.2% missing data

4	(0.208) 0.204 0.217

3	(0.792) 0.796 0.783

Locus 12740 :

2 alleles

99.2% missing data

2	(0.606) 0.608 0.605

4	(0.394) 0.392 0.395

Locus 12741 :

2 alleles

4.3% missing data

1	(0.979) 0.966 0.994

4	(0.021) 0.034 0.006

Locus 12742 :

2 alleles

98.8% missing data

2	(0.450) 0.448 0.450

3	(0.550) 0.552 0.550

Locus 12743 :

2 alleles

99.2% missing data

1	(0.745) 0.739 0.751

2	(0.255) 0.261 0.249

Locus 12744 :

2 alleles

98.4% missing data

1	(0.689) 0.699 0.677

3	(0.311) 0.301 0.323

Locus 12745 :

2 alleles

95.7% missing data

4	(0.905) 0.903 0.907

1	(0.095) 0.097 0.093

Locus 12746 :

2 alleles

99.2% missing data

3	(0.649) 0.652 0.647

1	(0.351) 0.348 0.353

Locus 12747 :

2 alleles

99.2% missing data

3	(0.361) 0.361 0.361

2	(0.639) 0.639 0.639

Locus 12748 :

2 alleles

99.2% missing data

2	(0.408) 0.408 0.413

1	(0.592) 0.592 0.587

Locus 12749 :

2 alleles

99.2% missing data

4	(0.243) 0.247 0.239

2	(0.757) 0.753 0.761

Locus 12750 :

2 alleles

98.0% missing data

1	(0.747) 0.748 0.753

3	(0.253) 0.252 0.247

Locus 12751 :

2 alleles

96.5% missing data

1	(0.281) 0.283 0.282

4	(0.719) 0.717 0.718

Locus 12752 :

2 alleles

98.8% missing data

1	(0.396) 0.398 0.398

3	(0.604) 0.602 0.602

Locus 12753 :

2 alleles

97.3% missing data

1	(0.139) 0.135 0.139

3	(0.861) 0.865 0.861

Locus 12754 :

2 alleles

98.8% missing data

3	(0.605) 0.607 0.610

2	(0.395) 0.393 0.390

Locus 12755 :

2 alleles

99.2% missing data

1	(0.665) 0.664 0.664

4	(0.335) 0.336 0.336

Locus 12756 :

2 alleles

99.2% missing data

2	(0.801) 0.798 0.801

1	(0.199) 0.202 0.199

Locus 12757 :

2 alleles

99.2% missing data

4	(0.396) 0.398 0.391

1	(0.604) 0.602 0.609

Locus 12758 :

2 alleles

99.2% missing data

1	(0.275) 0.275 0.277

2	(0.725) 0.725 0.723

Locus 12759 :

2 alleles

99.2% missing data

4	(0.449) 0.453 0.455

1	(0.551) 0.547 0.545

Locus 12760 :

2 alleles

99.2% missing data

1	(0.290) 0.290 0.295

3	(0.710) 0.710 0.705

Locus 12761 :

2 alleles

99.2% missing data

2	(0.490) 0.494 0.492

4	(0.510) 0.506 0.508

Locus 12762 :

2 alleles

99.2% missing data

2	(0.452) 0.446 0.462

4	(0.548) 0.554 0.538

Locus 12763 :

2 alleles

98.4% missing data

3	(0.156) 0.151 0.161

4	(0.844) 0.849 0.839

Locus 12764 :

2 alleles

99.2% missing data

2	(0.508) 0.510 0.515

1	(0.492) 0.490 0.485

Locus 12765 :

2 alleles

9.0% missing data

4	(0.984) 0.977 0.995

1	(0.016) 0.023 0.005

Locus 12766 :

2 alleles

98.8% missing data

3	(0.681) 0.684 0.683

2	(0.319) 0.316 0.317

Locus 12767 :

2 alleles

7.8% missing data

2	(0.971) 0.948 0.991

1	(0.029) 0.052 0.009

Locus 12768 :

2 alleles

99.2% missing data

4	(0.469) 0.467 0.468

2	(0.531) 0.533 0.532

Locus 12769 :

2 alleles

99.2% missing data

3	(0.557) 0.551 0.557

1	(0.443) 0.449 0.443

Locus 12770 :

3 alleles

98.8% missing data

4	(0.273) 0.270 0.275

1	(0.276) 0.276 0.274

3	(0.451) 0.454 0.450

Locus 12771 :

2 alleles

8.2% missing data

1	(0.985) 0.978 0.995

4	(0.015) 0.022 0.005

Locus 12772 :

2 alleles

97.7% missing data

4	(0.442) 0.437 0.444

2	(0.558) 0.563 0.556

Locus 12773 :

3 alleles

98.4% missing data

2	(0.258) 0.253 0.263

3	(0.373) 0.377 0.371

1	(0.369) 0.370 0.366

Locus 12774 :

2 alleles

99.2% missing data

2	(0.534) 0.526 0.537

4	(0.466) 0.474 0.463

Locus 12775 :

2 alleles

98.8% missing data

2	(0.648) 0.643 0.652

3	(0.352) 0.357 0.348

Locus 12776 :

2 alleles

90.6% missing data

4	(0.125) 0.106 0.140

2	(0.875) 0.894 0.860

Locus 12777 :

2 alleles

99.2% missing data

4	(0.318) 0.313 0.323

2	(0.682) 0.687 0.677

Locus 12778 :

2 alleles

99.2% missing data

3	(0.336) 0.331 0.341

1	(0.664) 0.669 0.659

Locus 12779 :

2 alleles

99.2% missing data

4	(0.458) 0.453 0.463

3	(0.542) 0.547 0.537

Locus 12780 :

2 alleles

96.9% missing data

4	(0.867) 0.860 0.875

1	(0.133) 0.140 0.125

Locus 12781 :

2 alleles

99.2% missing data

4	(0.528) 0.527 0.532

3	(0.472) 0.473 0.468

Locus 12782 :

3 alleles

98.4% missing data

3	(0.319) 0.322 0.327

4	(0.309) 0.311 0.306

1	(0.372) 0.368 0.367

Locus 12783 :

2 alleles

97.7% missing data

2	(0.667) 0.661 0.683

3	(0.333) 0.339 0.317

Locus 12784 :

2 alleles

96.9% missing data

1	(0.793) 0.786 0.802

3	(0.207) 0.214 0.198

Locus 12785 :

2 alleles

99.2% missing data

1	(0.530) 0.539 0.525

4	(0.470) 0.461 0.475

Locus 12786 :

2 alleles

99.2% missing data

1	(0.562) 0.562 0.562

4	(0.438) 0.438 0.438

Locus 12787 :

2 alleles

99.2% missing data

4	(0.479) 0.478 0.477

1	(0.521) 0.522 0.523

Locus 12788 :

2 alleles

97.7% missing data

2	(0.790) 0.782 0.804

4	(0.210) 0.218 0.196

Locus 12789 :

2 alleles

99.2% missing data

2	(0.420) 0.420 0.426

3	(0.580) 0.580 0.574

Locus 12790 :

2 alleles

99.2% missing data

3	(0.564) 0.565 0.561

2	(0.436) 0.435 0.439

Locus 12791 :

2 alleles

98.8% missing data

1	(0.695) 0.698 0.694

4	(0.305) 0.302 0.306

Locus 12792 :

2 alleles

99.2% missing data

4	(0.510) 0.502 0.518

2	(0.490) 0.498 0.482

Locus 12793 :

3 alleles

98.8% missing data

4	(0.347) 0.344 0.357

3	(0.385) 0.391 0.375

1	(0.268) 0.265 0.268

Locus 12794 :

2 alleles

99.2% missing data

4	(0.543) 0.541 0.550

1	(0.457) 0.459 0.450

Locus 12795 :

2 alleles

99.2% missing data

1	(0.423) 0.414 0.431

2	(0.577) 0.586 0.569

Locus 12796 :

2 alleles

99.2% missing data

1	(0.385) 0.384 0.385

4	(0.615) 0.616 0.615

Locus 12797 :

2 alleles

98.8% missing data

4	(0.420) 0.419 0.419

2	(0.580) 0.581 0.581

Locus 12798 :

2 alleles

96.5% missing data

1	(0.871) 0.883 0.862

2	(0.129) 0.117 0.138

Locus 12799 :

2 alleles

99.2% missing data

3	(0.352) 0.352 0.359

2	(0.648) 0.648 0.641

Locus 12800 :

2 alleles

99.2% missing data

2	(0.571) 0.562 0.585

1	(0.429) 0.438 0.415

Locus 12801 :

2 alleles

99.2% missing data

1	(0.341) 0.339 0.342

2	(0.659) 0.661 0.658

Locus 12802 :

2 alleles

98.0% missing data

3	(0.640) 0.642 0.642

2	(0.360) 0.358 0.358

Locus 12803 :

2 alleles

96.5% missing data

2	(0.688) 0.696 0.687

3	(0.312) 0.304 0.313

Locus 12804 :

2 alleles

99.2% missing data

3	(0.626) 0.630 0.623

2	(0.374) 0.370 0.377

Locus 12805 :

2 alleles

99.2% missing data

1	(0.336) 0.340 0.343

4	(0.664) 0.660 0.657

Locus 12806 :

2 alleles

99.2% missing data

3	(0.703) 0.701 0.711

2	(0.297) 0.299 0.289

Locus 12807 :

3 alleles

98.8% missing data

3	(0.478) 0.479 0.479

1	(0.311) 0.305 0.312

4	(0.211) 0.216 0.209

Locus 12808 :

2 alleles

98.8% missing data

4	(0.441) 0.436 0.442

2	(0.559) 0.564 0.558

Locus 12809 :

2 alleles

99.2% missing data

2	(0.782) 0.780 0.780

3	(0.218) 0.220 0.220

Locus 12810 :

2 alleles

99.2% missing data

2	(0.489) 0.479 0.496

1	(0.511) 0.521 0.504

Locus 12811 :

2 alleles

99.2% missing data

2	(0.792) 0.788 0.795

1	(0.208) 0.212 0.205

Locus 12812 :

2 alleles

98.8% missing data

1	(0.482) 0.474 0.490

4	(0.518) 0.526 0.510

Locus 12813 :

2 alleles

99.2% missing data

4	(0.408) 0.410 0.410

1	(0.592) 0.590 0.590

Locus 12814 :

2 alleles

98.8% missing data

1	(0.655) 0.654 0.662

4	(0.345) 0.346 0.338

Locus 12815 :

2 alleles

99.2% missing data

1	(0.333) 0.325 0.343

2	(0.667) 0.675 0.657

Locus 12816 :

2 alleles

99.2% missing data

3	(0.493) 0.493 0.491

4	(0.507) 0.507 0.509

Locus 12817 :

2 alleles

99.2% missing data

2	(0.653) 0.646 0.660

3	(0.347) 0.354 0.340

Locus 12818 :

2 alleles

98.8% missing data

4	(0.696) 0.703 0.691

2	(0.304) 0.297 0.309

Locus 12819 :

2 alleles

8.2% missing data

2	(0.986) 0.978 0.996

1	(0.014) 0.022 0.004

Locus 12820 :

2 alleles

99.2% missing data

1	(0.558) 0.563 0.547

2	(0.442) 0.437 0.453

Locus 12821 :

2 alleles

98.8% missing data

3	(0.586) 0.578 0.592

4	(0.414) 0.422 0.408

Locus 12822 :

2 alleles

94.9% missing data

1	(0.756) 0.753 0.773

3	(0.244) 0.247 0.227

Locus 12823 :

3 alleles

97.7% missing data

4	(0.421) 0.421 0.427

1	(0.319) 0.314 0.324

3	(0.260) 0.264 0.249

Locus 12824 :

2 alleles

98.8% missing data

1	(0.437) 0.440 0.436

3	(0.563) 0.560 0.564

Locus 12825 :

2 alleles

99.2% missing data

2	(0.394) 0.395 0.394

4	(0.606) 0.605 0.606

Locus 12826 :

2 alleles

8.2% missing data

4	(0.984) 0.977 0.994

3	(0.016) 0.023 0.006

Locus 12827 :

2 alleles

99.2% missing data

2	(0.487) 0.484 0.489

3	(0.513) 0.516 0.511

Locus 12828 :

2 alleles

99.2% missing data

2	(0.417) 0.412 0.420

1	(0.583) 0.588 0.580

Locus 12829 :

2 alleles

98.8% missing data

2	(0.489) 0.495 0.485

1	(0.511) 0.505 0.515

Locus 12830 :

2 alleles

98.8% missing data

4	(0.365) 0.354 0.372

2	(0.635) 0.646 0.628

Locus 12831 :

2 alleles

99.2% missing data

1	(0.524) 0.521 0.528

2	(0.476) 0.479 0.472

Locus 12832 :

2 alleles

98.8% missing data

2	(0.623) 0.628 0.623

4	(0.377) 0.372 0.377

Locus 12833 :

2 alleles

95.7% missing data

3	(0.466) 0.449 0.467

2	(0.534) 0.551 0.533

Locus 12834 :

2 alleles

99.2% missing data

2	(0.282) 0.280 0.286

4	(0.718) 0.720 0.714

Locus 12835 :

2 alleles

99.2% missing data

2	(0.440) 0.434 0.443

4	(0.560) 0.566 0.557

Locus 12836 :

2 alleles

99.2% missing data

4	(0.399) 0.403 0.396

2	(0.601) 0.597 0.604

Locus 12837 :

2 alleles

99.2% missing data

2	(0.532) 0.529 0.530

3	(0.468) 0.471 0.470

Locus 12838 :

2 alleles

94.1% missing data

1	(0.612) 0.633 0.589

4	(0.388) 0.367 0.411

Locus 12839 :

2 alleles

0.0% missing data

2	(0.977) 0.982 0.980

1	(0.023) 0.018 0.020

Locus 12840 :

2 alleles

99.2% missing data

4	(0.406) 0.403 0.413

2	(0.594) 0.597 0.587

Locus 12841 :

3 alleles

98.4% missing data

1	(0.371) 0.383 0.365

2	(0.154) 0.150 0.166

4	(0.475) 0.467 0.469

Locus 12842 :

2 alleles

99.2% missing data

2	(0.371) 0.367 0.374

1	(0.629) 0.633 0.626

Locus 12843 :

2 alleles

98.8% missing data

2	(0.656) 0.662 0.654

1	(0.344) 0.338 0.346

Locus 12844 :

2 alleles

98.8% missing data

2	(0.493) 0.485 0.500

4	(0.507) 0.515 0.500

Locus 12845 :

2 alleles

99.2% missing data

4	(0.773) 0.778 0.771

3	(0.227) 0.222 0.229

Locus 12846 :

2 alleles

99.2% missing data

4	(0.434) 0.434 0.438

2	(0.566) 0.566 0.562

Locus 12847 :

2 alleles

97.7% missing data

2	(0.740) 0.738 0.740

3	(0.260) 0.262 0.260

Locus 12848 :

2 alleles

99.2% missing data

2	(0.425) 0.426 0.428

1	(0.575) 0.574 0.572

Locus 12849 :

2 alleles

95.7% missing data

4	(0.852) 0.862 0.852

1	(0.148) 0.138 0.148

Locus 12850 :

2 alleles

98.4% missing data

4	(0.635) 0.646 0.631

3	(0.365) 0.354 0.369

Locus 12851 :

2 alleles

93.0% missing data

2	(0.904) 0.905 0.910

3	(0.096) 0.095 0.090

Locus 12852 :

2 alleles

99.2% missing data

3	(0.668) 0.673 0.661

4	(0.332) 0.327 0.339

Locus 12853 :

2 alleles

96.5% missing data

3	(0.717) 0.732 0.715

2	(0.283) 0.268 0.285

Locus 12854 :

2 alleles

99.2% missing data

3	(0.591) 0.583 0.593

2	(0.409) 0.417 0.407

Locus 12855 :

2 alleles

99.2% missing data

2	(0.515) 0.512 0.517

1	(0.485) 0.488 0.483

Locus 12856 :

2 alleles

98.8% missing data

2	(0.490) 0.477 0.493

1	(0.510) 0.523 0.507

Locus 12857 :

2 alleles

99.2% missing data

4	(0.464) 0.460 0.464

1	(0.536) 0.540 0.536

Locus 12858 :

2 alleles

99.2% missing data

1	(0.177) 0.175 0.179

3	(0.823) 0.825 0.821

Locus 12859 :

2 alleles

98.8% missing data

3	(0.786) 0.789 0.785

2	(0.214) 0.211 0.215

Locus 12860 :

2 alleles

99.2% missing data

2	(0.274) 0.270 0.277

4	(0.726) 0.730 0.723

Locus 12861 :

2 alleles

8.2% missing data

2	(0.983) 0.975 0.995

4	(0.017) 0.025 0.005

Locus 12862 :

2 alleles

99.2% missing data

1	(0.287) 0.292 0.282

2	(0.713) 0.708 0.718

Locus 12863 :

2 alleles

99.2% missing data

4	(0.568) 0.560 0.571

3	(0.432) 0.440 0.429

Locus 12864 :

2 alleles

97.7% missing data

2	(0.183) 0.183 0.181

3	(0.817) 0.817 0.819

Locus 12865 :

2 alleles

96.5% missing data

3	(0.848) 0.862 0.843

2	(0.152) 0.138 0.157

Locus 12866 :

2 alleles

99.2% missing data

1	(0.520) 0.518 0.527

4	(0.480) 0.482 0.473

Locus 12867 :

2 alleles

99.2% missing data

1	(0.597) 0.595 0.597

4	(0.403) 0.405 0.403

Locus 12868 :

2 alleles

0.0% missing data

3	(0.990) 0.984 0.997

2	(0.010) 0.016 0.003

Locus 12869 :

2 alleles

99.2% missing data

3	(0.286) 0.291 0.286

1	(0.714) 0.709 0.714

Locus 12870 :

2 alleles

99.2% missing data

2	(0.292) 0.287 0.304

1	(0.708) 0.713 0.696

Locus 12871 :

2 alleles

99.2% missing data

3	(0.490) 0.489 0.491

1	(0.510) 0.511 0.509

Locus 12872 :

2 alleles

99.2% missing data

2	(0.341) 0.341 0.344

1	(0.659) 0.659 0.656

Locus 12873 :

2 alleles

99.2% missing data

4	(0.373) 0.370 0.378

1	(0.627) 0.630 0.622

Locus 12874 :

2 alleles

99.2% missing data

1	(0.655) 0.649 0.663

4	(0.345) 0.351 0.337

Locus 12875 :

2 alleles

99.2% missing data

1	(0.422) 0.425 0.425

2	(0.578) 0.575 0.575

Locus 12876 :

2 alleles

99.2% missing data

2	(0.314) 0.319 0.309

3	(0.686) 0.681 0.691

Locus 12877 :

2 alleles

0.0% missing data

3	(0.987) 0.983 0.997

2	(0.013) 0.017 0.003

Locus 12878 :

2 alleles

99.2% missing data

4	(0.487) 0.477 0.492

3	(0.513) 0.523 0.508

Locus 12879 :

2 alleles

0.8% missing data

1	(0.958) 0.904 0.987

2	(0.042) 0.096 0.013

Locus 12880 :

2 alleles

97.3% missing data

2	(0.747) 0.762 0.746

4	(0.253) 0.238 0.254

Locus 12881 :

2 alleles

99.2% missing data

2	(0.678) 0.680 0.670

3	(0.322) 0.320 0.330

Locus 12882 :

2 alleles

99.2% missing data

3	(0.309) 0.314 0.313

1	(0.691) 0.686 0.687

Locus 12883 :

2 alleles

94.9% missing data

4	(0.378) 0.366 0.390

2	(0.622) 0.634 0.610

Locus 12884 :

2 alleles

98.8% missing data

4	(0.687) 0.692 0.691

2	(0.313) 0.308 0.309

Locus 12885 :

2 alleles

99.2% missing data

3	(0.531) 0.523 0.535

1	(0.469) 0.477 0.465

Locus 12886 :

2 alleles

98.8% missing data

3	(0.231) 0.232 0.236

4	(0.769) 0.768 0.764

Locus 12887 :

2 alleles

99.2% missing data

2	(0.457) 0.455 0.462

3	(0.543) 0.545 0.538

Locus 12888 :

3 alleles

94.1% missing data

4	(0.491) 0.461 0.520

2	(0.387) 0.420 0.361

3	(0.122) 0.119 0.119

Locus 12889 :

2 alleles

99.2% missing data

2	(0.375) 0.373 0.379

3	(0.625) 0.627 0.621

Locus 12890 :

2 alleles

99.2% missing data

4	(0.362) 0.365 0.359

2	(0.638) 0.635 0.641

Locus 12891 :

2 alleles

99.2% missing data

1	(0.453) 0.453 0.448

3	(0.547) 0.547 0.552

Locus 12892 :

2 alleles

96.5% missing data

1	(0.914) 0.919 0.908

3	(0.086) 0.081 0.092

Locus 12893 :

2 alleles

99.2% missing data

4	(0.280) 0.278 0.283

1	(0.720) 0.722 0.717

Locus 12894 :

2 alleles

99.2% missing data

1	(0.378) 0.382 0.381

2	(0.622) 0.618 0.619

Locus 12895 :

2 alleles

0.0% missing data

2	(0.979) 0.984 0.985

3	(0.021) 0.016 0.015

Locus 12896 :

3 alleles

97.7% missing data

4	(0.643) 0.652 0.640

2	(0.153) 0.147 0.156

3	(0.204) 0.201 0.204

Locus 12897 :

2 alleles

99.2% missing data

2	(0.511) 0.505 0.517

3	(0.489) 0.495 0.483

Locus 12898 :

3 alleles

98.4% missing data

3	(0.412) 0.409 0.416

1	(0.341) 0.346 0.332

4	(0.247) 0.245 0.253

Locus 12899 :

2 alleles

99.2% missing data

3	(0.505) 0.510 0.502

1	(0.495) 0.490 0.498

Locus 12900 :

3 alleles

90.6% missing data

1	(0.849) 0.871 0.844

3	(0.081) 0.064 0.088

4	(0.070) 0.064 0.069

Locus 12901 :

2 alleles

99.2% missing data

4	(0.643) 0.644 0.639

2	(0.357) 0.356 0.361

Locus 12902 :

2 alleles

99.2% missing data

1	(0.673) 0.667 0.678

4	(0.327) 0.333 0.322

Locus 12903 :

2 alleles

98.0% missing data

3	(0.780) 0.777 0.784

4	(0.220) 0.223 0.216

Locus 12904 :

2 alleles

98.8% missing data

3	(0.405) 0.400 0.412

4	(0.595) 0.600 0.588

Locus 12905 :

3 alleles

98.4% missing data

4	(0.277) 0.281 0.281

3	(0.541) 0.535 0.545

1	(0.181) 0.184 0.174

Locus 12906 :

2 alleles

99.2% missing data

1	(0.601) 0.598 0.604

3	(0.399) 0.402 0.396

Locus 12907 :

2 alleles

91.4% missing data

3	(0.869) 0.868 0.886

1	(0.131) 0.132 0.114

Locus 12908 :

2 alleles

99.2% missing data

3	(0.763) 0.761 0.762

2	(0.237) 0.239 0.238

Locus 12909 :

2 alleles

98.0% missing data

2	(0.709) 0.705 0.723

4	(0.291) 0.295 0.277

Locus 12910 :

2 alleles

99.2% missing data

4	(0.298) 0.292 0.300

1	(0.702) 0.708 0.700

Locus 12911 :

2 alleles

99.2% missing data

3	(0.204) 0.209 0.202

2	(0.796) 0.791 0.798

Locus 12912 :

2 alleles

96.1% missing data

1	(0.816) 0.820 0.825

3	(0.184) 0.180 0.175

Locus 12913 :

2 alleles

99.2% missing data

3	(0.338) 0.332 0.342

4	(0.662) 0.668 0.658

Locus 12914 :

3 alleles

98.8% missing data

4	(0.332) 0.327 0.335

3	(0.306) 0.304 0.308

1	(0.363) 0.369 0.357

Locus 12915 :

2 alleles

95.7% missing data

4	(0.745) 0.748 0.746

3	(0.255) 0.252 0.254

Locus 12916 :

2 alleles

99.2% missing data

1	(0.733) 0.729 0.738

2	(0.267) 0.271 0.262

Locus 12917 :

2 alleles

99.2% missing data

1	(0.592) 0.587 0.595

2	(0.408) 0.413 0.405

Locus 12918 :

2 alleles

99.2% missing data

4	(0.490) 0.486 0.496

2	(0.510) 0.514 0.504

Locus 12919 :

2 alleles

99.2% missing data

2	(0.587) 0.588 0.582

1	(0.413) 0.412 0.418

Locus 12920 :

2 alleles

99.2% missing data

2	(0.505) 0.516 0.499

4	(0.495) 0.484 0.501

Locus 12921 :

2 alleles

99.2% missing data

1	(0.675) 0.681 0.674

3	(0.325) 0.319 0.326

Locus 12922 :

2 alleles

99.2% missing data

2	(0.802) 0.794 0.808

1	(0.198) 0.206 0.192

Locus 12923 :

2 alleles

98.8% missing data

3	(0.659) 0.656 0.663

4	(0.341) 0.344 0.337

Locus 12924 :

2 alleles

96.9% missing data

3	(0.800) 0.802 0.803

1	(0.200) 0.198 0.197

Locus 12925 :

2 alleles

99.2% missing data

3	(0.668) 0.660 0.668

4	(0.332) 0.340 0.332

Locus 12926 :

2 alleles

97.7% missing data

2	(0.629) 0.643 0.624

3	(0.371) 0.357 0.376

Locus 12927 :

2 alleles

99.2% missing data

1	(0.480) 0.480 0.475

2	(0.520) 0.520 0.525

Locus 12928 :

2 alleles

98.8% missing data

2	(0.631) 0.620 0.643

4	(0.369) 0.380 0.357

Locus 12929 :

2 alleles

99.2% missing data

2	(0.431) 0.430 0.435

4	(0.569) 0.570 0.565

Locus 12930 :

2 alleles

96.1% missing data

2	(0.847) 0.854 0.849

1	(0.153) 0.146 0.151

Locus 12931 :

2 alleles

99.2% missing data

1	(0.554) 0.559 0.553

3	(0.446) 0.441 0.447

Locus 12932 :

2 alleles

94.9% missing data

1	(0.807) 0.809 0.815

4	(0.193) 0.191 0.185

Locus 12933 :

2 alleles

99.2% missing data

4	(0.630) 0.630 0.623

3	(0.370) 0.370 0.377

Locus 12934 :

2 alleles

99.2% missing data

2	(0.465) 0.460 0.470

4	(0.535) 0.540 0.530

Locus 12935 :

2 alleles

99.2% missing data

1	(0.473) 0.474 0.471

4	(0.527) 0.526 0.529

Locus 12936 :

2 alleles

99.2% missing data

3	(0.280) 0.279 0.282

2	(0.720) 0.721 0.718

Locus 12937 :

3 alleles

98.4% missing data

3	(0.288) 0.278 0.297

2	(0.461) 0.468 0.452

4	(0.251) 0.254 0.251

Locus 12938 :

2 alleles

99.2% missing data

4	(0.677) 0.671 0.675

2	(0.323) 0.329 0.325

Locus 12939 :

2 alleles

99.2% missing data

3	(0.577) 0.573 0.581

2	(0.423) 0.427 0.419

Locus 12940 :

2 alleles

99.2% missing data

3	(0.638) 0.643 0.630

1	(0.362) 0.357 0.370

Locus 12941 :

2 alleles

95.3% missing data

2	(0.831) 0.823 0.849

3	(0.169) 0.177 0.151

Locus 12942 :

2 alleles

99.2% missing data

1	(0.508) 0.502 0.511

2	(0.492) 0.498 0.489

Locus 12943 :

2 alleles

8.6% missing data

2	(0.978) 0.974 0.992

1	(0.022) 0.026 0.008

Locus 12944 :

2 alleles

99.2% missing data

2	(0.386) 0.379 0.390

1	(0.614) 0.621 0.610

Locus 12945 :

2 alleles

96.5% missing data

2	(0.828) 0.832 0.834

3	(0.172) 0.168 0.166

Locus 12946 :

2 alleles

99.2% missing data

4	(0.802) 0.807 0.797

1	(0.198) 0.193 0.203

Locus 12947 :

2 alleles

98.8% missing data

3	(0.514) 0.516 0.516

1	(0.486) 0.484 0.484

Locus 12948 :

2 alleles

99.2% missing data

3	(0.275) 0.278 0.277

4	(0.725) 0.722 0.723

Locus 12949 :

2 alleles

99.2% missing data

2	(0.461) 0.452 0.465

1	(0.539) 0.548 0.535

Locus 12950 :

2 alleles

99.2% missing data

2	(0.593) 0.587 0.592

3	(0.407) 0.413 0.408

Locus 12951 :

2 alleles

98.8% missing data

1	(0.427) 0.433 0.428

3	(0.573) 0.567 0.572

Locus 12952 :

2 alleles

97.7% missing data

4	(0.761) 0.761 0.766

2	(0.239) 0.239 0.234

Locus 12953 :

2 alleles

99.2% missing data

3	(0.632) 0.624 0.631

4	(0.368) 0.376 0.369

Locus 12954 :

2 alleles

98.0% missing data

2	(0.171) 0.170 0.177

4	(0.829) 0.830 0.823

Locus 12955 :

2 alleles

99.2% missing data

3	(0.590) 0.589 0.591

2	(0.410) 0.411 0.409

Locus 12956 :

2 alleles

98.8% missing data

3	(0.353) 0.352 0.354

2	(0.647) 0.648 0.646

Locus 12957 :

2 alleles

98.4% missing data

4	(0.164) 0.154 0.166

2	(0.836) 0.846 0.834

Locus 12958 :

2 alleles

98.4% missing data

2	(0.672) 0.663 0.678

4	(0.328) 0.337 0.322

Locus 12959 :

2 alleles

0.4% missing data

2	(0.966) 0.973 0.968

3	(0.034) 0.027 0.032

Locus 12960 :

2 alleles

99.2% missing data

3	(0.469) 0.462 0.477

2	(0.531) 0.538 0.523

Locus 12961 :

2 alleles

96.1% missing data

4	(0.836) 0.850 0.834

1	(0.164) 0.150 0.166

Locus 12962 :

2 alleles

99.2% missing data

1	(0.512) 0.512 0.514

2	(0.488) 0.488 0.486

Locus 12963 :

2 alleles

94.9% missing data

4	(0.224) 0.225 0.217

2	(0.776) 0.775 0.783

Locus 12964 :

2 alleles

99.2% missing data

2	(0.573) 0.574 0.568

4	(0.427) 0.426 0.432

Locus 12965 :

2 alleles

99.2% missing data

4	(0.558) 0.556 0.560

1	(0.442) 0.444 0.440

Locus 12966 :

2 alleles

99.2% missing data

4	(0.458) 0.463 0.459

3	(0.542) 0.537 0.541

Locus 12967 :

2 alleles

98.8% missing data

3	(0.591) 0.579 0.593

2	(0.409) 0.421 0.407

Locus 12968 :

2 alleles

96.5% missing data

2	(0.133) 0.133 0.126

1	(0.867) 0.867 0.874

Locus 12969 :

2 alleles

99.2% missing data

3	(0.277) 0.282 0.273

1	(0.723) 0.718 0.727

Locus 12970 :

2 alleles

99.2% missing data

2	(0.520) 0.522 0.519

4	(0.480) 0.478 0.481

Locus 12971 :

2 alleles

99.2% missing data

1	(0.613) 0.608 0.619

3	(0.387) 0.392 0.381

Locus 12972 :

2 alleles

99.2% missing data

4	(0.483) 0.484 0.484

1	(0.517) 0.516 0.516

Locus 12973 :

2 alleles

96.1% missing data

1	(0.863) 0.868 0.865

4	(0.137) 0.132 0.135

Locus 12974 :

2 alleles

97.3% missing data

1	(0.325) 0.328 0.313

4	(0.675) 0.672 0.687

Locus 12975 :

2 alleles

97.7% missing data

2	(0.141) 0.127 0.149

4	(0.859) 0.873 0.851

Locus 12976 :

2 alleles

99.2% missing data

2	(0.606) 0.599 0.607

4	(0.394) 0.401 0.393

Locus 12977 :

2 alleles

99.2% missing data

1	(0.403) 0.402 0.399

3	(0.597) 0.598 0.601

Locus 12978 :

2 alleles

98.0% missing data

3	(0.644) 0.640 0.656

2	(0.356) 0.360 0.344

Locus 12979 :

2 alleles

98.8% missing data

1	(0.655) 0.654 0.645

4	(0.345) 0.346 0.355

Locus 12980 :

3 alleles

98.8% missing data

4	(0.440) 0.433 0.445

2	(0.377) 0.378 0.379

3	(0.184) 0.189 0.177

Locus 12981 :

2 alleles

96.9% missing data

1	(0.231) 0.229 0.222

4	(0.769) 0.771 0.778

Locus 12982 :

2 alleles

99.2% missing data

3	(0.623) 0.625 0.627

4	(0.377) 0.375 0.373

Locus 12983 :

2 alleles

99.2% missing data

2	(0.473) 0.479 0.469

3	(0.527) 0.521 0.531

Locus 12984 :

2 alleles

96.5% missing data

4	(0.228) 0.211 0.235

2	(0.772) 0.789 0.765

Locus 12985 :

2 alleles

99.2% missing data

2	(0.325) 0.316 0.335

3	(0.675) 0.684 0.665

Locus 12986 :

2 alleles

99.2% missing data

3	(0.344) 0.343 0.351

4	(0.656) 0.657 0.649

Locus 12987 :

2 alleles

96.9% missing data

4	(0.693) 0.705 0.687

3	(0.307) 0.295 0.313

Locus 12988 :

3 alleles

98.8% missing data

4	(0.435) 0.437 0.431

3	(0.301) 0.300 0.305

2	(0.264) 0.263 0.264

Locus 12989 :

2 alleles

96.1% missing data

2	(0.142) 0.128 0.151

4	(0.858) 0.872 0.849

Locus 12990 :

2 alleles

98.8% missing data

4	(0.383) 0.387 0.386

2	(0.617) 0.613 0.614

Locus 12991 :

2 alleles

99.2% missing data

2	(0.312) 0.322 0.310

1	(0.688) 0.678 0.690

Locus 12992 :

2 alleles

99.2% missing data

4	(0.660) 0.664 0.655

3	(0.340) 0.336 0.345

Locus 12993 :

2 alleles

38.7% missing data

3	(0.979) 0.984 0.986

1	(0.021) 0.016 0.014

Locus 12994 :

2 alleles

99.2% missing data

2	(0.522) 0.532 0.516

4	(0.478) 0.468 0.484

Locus 12995 :

2 alleles

99.2% missing data

3	(0.313) 0.304 0.316

2	(0.687) 0.696 0.684

Locus 12996 :

2 alleles

98.8% missing data

4	(0.573) 0.574 0.566

2	(0.427) 0.426 0.434

Locus 12997 :

3 alleles

97.7% missing data

1	(0.157) 0.151 0.157

4	(0.634) 0.646 0.631

3	(0.209) 0.203 0.212

Locus 12998 :

2 alleles

99.2% missing data

3	(0.734) 0.731 0.736

1	(0.266) 0.269 0.264

Locus 12999 :

2 alleles

99.2% missing data

3	(0.555) 0.548 0.564

1	(0.445) 0.452 0.436

Locus 13000 :

2 alleles

99.2% missing data

1	(0.567) 0.561 0.571

3	(0.433) 0.439 0.429

Locus 13001 :

2 alleles

99.2% missing data

1	(0.675) 0.671 0.681

4	(0.325) 0.329 0.319

Locus 13002 :

2 alleles

99.2% missing data

1	(0.755) 0.757 0.758

2	(0.245) 0.243 0.242

Locus 13003 :

2 alleles

0.0% missing data

3	(0.988) 0.983 0.996

1	(0.012) 0.017 0.004

Locus 13004 :

2 alleles

99.2% missing data

2	(0.560) 0.559 0.559

3	(0.440) 0.441 0.441

Locus 13005 :

2 alleles

99.2% missing data

2	(0.569) 0.571 0.563

3	(0.431) 0.429 0.437

Locus 13006 :

2 alleles

99.2% missing data

4	(0.678) 0.672 0.677

3	(0.322) 0.328 0.323

Locus 13007 :

2 alleles

99.2% missing data

2	(0.260) 0.258 0.267

4	(0.740) 0.742 0.733

Locus 13008 :

2 alleles

99.2% missing data

1	(0.384) 0.381 0.385

4	(0.616) 0.619 0.615

Locus 13009 :

2 alleles

96.1% missing data

3	(0.718) 0.732 0.718

4	(0.282) 0.268 0.282

Locus 13010 :

2 alleles

99.2% missing data

3	(0.447) 0.440 0.446

1	(0.553) 0.560 0.554

Locus 13011 :

2 alleles

99.2% missing data

1	(0.174) 0.177 0.175

3	(0.826) 0.823 0.825

Locus 13012 :

2 alleles

99.2% missing data

4	(0.604) 0.604 0.598

2	(0.396) 0.396 0.402

Locus 13013 :

2 alleles

99.2% missing data

3	(0.266) 0.260 0.271

1	(0.734) 0.740 0.729

Locus 13014 :

2 alleles

99.2% missing data

3	(0.777) 0.776 0.779

2	(0.223) 0.224 0.221

Locus 13015 :

2 alleles

94.5% missing data

3	(0.909) 0.901 0.920

2	(0.091) 0.099 0.080

Locus 13016 :

2 alleles

91.0% missing data

1	(0.925) 0.938 0.916

3	(0.075) 0.062 0.084

Locus 13017 :

2 alleles

99.2% missing data

1	(0.385) 0.388 0.380

4	(0.615) 0.612 0.620

Locus 13018 :

2 alleles

98.4% missing data

1	(0.341) 0.342 0.340

2	(0.659) 0.658 0.660

Locus 13019 :

2 alleles

96.1% missing data

3	(0.904) 0.912 0.901

2	(0.096) 0.088 0.099

Locus 13020 :

2 alleles

99.2% missing data

4	(0.594) 0.596 0.595

1	(0.406) 0.404 0.405

Locus 13021 :

2 alleles

99.2% missing data

3	(0.667) 0.673 0.658

1	(0.333) 0.327 0.342

Locus 13022 :

2 alleles

98.8% missing data

1	(0.612) 0.609 0.604

4	(0.388) 0.391 0.396

Locus 13023 :

2 alleles

99.2% missing data

4	(0.705) 0.705 0.709

2	(0.295) 0.295 0.291

Locus 13024 :

2 alleles

99.2% missing data

1	(0.258) 0.256 0.259

3	(0.742) 0.744 0.741

Locus 13025 :

2 alleles

99.2% missing data

3	(0.519) 0.518 0.524

1	(0.481) 0.482 0.476

Locus 13026 :

3 alleles

98.8% missing data

2	(0.494) 0.498 0.485

3	(0.240) 0.238 0.240

1	(0.266) 0.263 0.275

Locus 13027 :

2 alleles

98.8% missing data

3	(0.401) 0.402 0.408

4	(0.599) 0.598 0.592

Locus 13028 :

2 alleles

99.2% missing data

3	(0.603) 0.610 0.599

2	(0.397) 0.390 0.401

Locus 13029 :

2 alleles

98.8% missing data

4	(0.634) 0.631 0.635

3	(0.366) 0.369 0.365

Locus 13030 :

2 alleles

99.2% missing data

4	(0.316) 0.324 0.313

1	(0.684) 0.676 0.687

Locus 13031 :

2 alleles

99.2% missing data

2	(0.485) 0.488 0.480

4	(0.515) 0.512 0.520

Locus 13032 :

2 alleles

99.2% missing data

3	(0.209) 0.209 0.213

2	(0.791) 0.791 0.787

Locus 13033 :

2 alleles

0.0% missing data

4	(0.981) 0.985 0.991

1	(0.019) 0.015 0.009

Locus 13034 :

2 alleles

99.2% missing data

2	(0.773) 0.774 0.772

1	(0.227) 0.226 0.228

Locus 13035 :

2 alleles

44.9% missing data

3	(0.986) 0.989 0.990

4	(0.014) 0.011 0.010

Locus 13036 :

2 alleles

99.2% missing data

3	(0.487) 0.490 0.488

1	(0.513) 0.510 0.512

Locus 13037 :

2 alleles

99.2% missing data

3	(0.448) 0.441 0.455

4	(0.552) 0.559 0.545

Locus 13038 :

3 alleles

98.8% missing data

4	(0.462) 0.455 0.462

2	(0.225) 0.228 0.232

3	(0.313) 0.317 0.306

Locus 13039 :

3 alleles

98.4% missing data

1	(0.212) 0.201 0.222

4	(0.571) 0.578 0.563

2	(0.217) 0.221 0.216

Locus 13040 :

2 alleles

98.0% missing data

3	(0.832) 0.827 0.835

1	(0.168) 0.173 0.165

Locus 13041 :

2 alleles

98.8% missing data

1	(0.720) 0.721 0.718

2	(0.280) 0.279 0.282

Locus 13042 :

3 alleles

98.4% missing data

1	(0.296) 0.298 0.291

4	(0.356) 0.355 0.362

3	(0.348) 0.347 0.347

Locus 13043 :

2 alleles

99.2% missing data

3	(0.498) 0.500 0.501

1	(0.502) 0.500 0.499

Locus 13044 :

2 alleles

98.4% missing data

2	(0.687) 0.687 0.693

4	(0.313) 0.313 0.307

Locus 13045 :

3 alleles

98.8% missing data

3	(0.517) 0.511 0.520

2	(0.159) 0.155 0.165

1	(0.324) 0.334 0.315

Locus 13046 :

3 alleles

98.8% missing data

4	(0.226) 0.225 0.227

2	(0.561) 0.559 0.559

1	(0.213) 0.216 0.214

Locus 13047 :

2 alleles

99.2% missing data

2	(0.605) 0.603 0.608

1	(0.395) 0.397 0.392

Locus 13048 :

2 alleles

98.8% missing data

3	(0.645) 0.647 0.640

4	(0.355) 0.353 0.360

Locus 13049 :

2 alleles

98.0% missing data

4	(0.619) 0.627 0.612

1	(0.381) 0.373 0.388

Locus 13050 :

2 alleles

99.2% missing data

3	(0.650) 0.643 0.657

2	(0.350) 0.357 0.343

Locus 13051 :

2 alleles

98.4% missing data

3	(0.746) 0.754 0.740

2	(0.254) 0.246 0.260

Locus 13052 :

2 alleles

99.2% missing data

1	(0.542) 0.544 0.546

4	(0.458) 0.456 0.454

Locus 13053 :

2 alleles

98.8% missing data

1	(0.685) 0.674 0.697

3	(0.315) 0.326 0.303

Locus 13054 :

3 alleles

97.3% missing data

2	(0.104) 0.098 0.107

1	(0.687) 0.684 0.694

4	(0.208) 0.218 0.199

Locus 13055 :

2 alleles

9.8% missing data

1	(0.987) 0.978 0.996

2	(0.013) 0.022 0.004

Locus 13056 :

2 alleles

99.2% missing data

4	(0.458) 0.461 0.456

1	(0.542) 0.539 0.544

Locus 13057 :

2 alleles

98.8% missing data

4	(0.329) 0.335 0.326

3	(0.671) 0.665 0.674

Locus 13058 :

2 alleles

96.1% missing data

1	(0.834) 0.847 0.827

2	(0.166) 0.153 0.173

Locus 13059 :

2 alleles

97.3% missing data

2	(0.200) 0.200 0.197

1	(0.800) 0.800 0.803

Locus 13060 :

2 alleles

99.2% missing data

4	(0.524) 0.522 0.525

2	(0.476) 0.478 0.475

Locus 13061 :

2 alleles

98.8% missing data

1	(0.618) 0.626 0.613

4	(0.382) 0.374 0.387

Locus 13062 :

2 alleles

99.2% missing data

1	(0.354) 0.352 0.359

3	(0.646) 0.648 0.641

Locus 13063 :

2 alleles

98.0% missing data

1	(0.737) 0.738 0.737

3	(0.263) 0.262 0.263

Locus 13064 :

2 alleles

99.2% missing data

4	(0.711) 0.710 0.714

2	(0.289) 0.290 0.286

Locus 13065 :

2 alleles

99.2% missing data

1	(0.521) 0.519 0.521

4	(0.479) 0.481 0.479

Locus 13066 :

2 alleles

99.2% missing data

1	(0.467) 0.465 0.469

4	(0.533) 0.535 0.531

Locus 13067 :

2 alleles

0.8% missing data

3	(0.990) 0.985 0.997

1	(0.010) 0.015 0.003

Locus 13068 :

2 alleles

96.1% missing data

2	(0.103) 0.094 0.108

4	(0.897) 0.906 0.892

Locus 13069 :

2 alleles

93.0% missing data

1	(0.837) 0.831 0.847

4	(0.163) 0.169 0.153

Locus 13070 :

2 alleles

98.8% missing data

3	(0.611) 0.614 0.608

1	(0.389) 0.386 0.392

Locus 13071 :

2 alleles

93.8% missing data

4	(0.877) 0.875 0.889

3	(0.123) 0.125 0.111

Locus 13072 :

2 alleles

99.2% missing data

1	(0.587) 0.578 0.592

4	(0.413) 0.422 0.408

Locus 13073 :

2 alleles

99.2% missing data

1	(0.454) 0.454 0.462

4	(0.546) 0.546 0.538

Locus 13074 :

2 alleles

99.2% missing data

2	(0.471) 0.463 0.475

4	(0.529) 0.537 0.525

Locus 13075 :

2 alleles

99.2% missing data

4	(0.513) 0.506 0.521

3	(0.487) 0.494 0.479

Locus 13076 :

2 alleles

99.2% missing data

3	(0.510) 0.510 0.514

2	(0.490) 0.490 0.486

Locus 13077 :

2 alleles

99.2% missing data

3	(0.379) 0.379 0.380

1	(0.621) 0.621 0.620

Locus 13078 :

2 alleles

99.2% missing data

2	(0.498) 0.499 0.496

4	(0.502) 0.501 0.504

Locus 13079 :

2 alleles

98.0% missing data

3	(0.653) 0.647 0.661

1	(0.347) 0.353 0.339

Locus 13080 :

2 alleles

99.2% missing data

4	(0.749) 0.750 0.745

1	(0.251) 0.250 0.255

Locus 13081 :

2 alleles

99.2% missing data

4	(0.362) 0.358 0.360

2	(0.638) 0.642 0.640

Locus 13082 :

2 alleles

98.0% missing data

2	(0.476) 0.466 0.474

3	(0.524) 0.534 0.526

Locus 13083 :

2 alleles

0.4% missing data

2	(0.972) 0.977 0.979

3	(0.028) 0.023 0.021

Locus 13084 :

2 alleles

93.4% missing data

1	(0.822) 0.815 0.838

3	(0.178) 0.185 0.162

Locus 13085 :

2 alleles

93.0% missing data

4	(0.899) 0.892 0.912

2	(0.101) 0.108 0.088

Locus 13086 :

2 alleles

99.2% missing data

3	(0.577) 0.576 0.580

1	(0.423) 0.424 0.420

Locus 13087 :

2 alleles

99.2% missing data

1	(0.479) 0.479 0.480

4	(0.521) 0.521 0.520

Locus 13088 :

3 alleles

95.7% missing data

4	(0.077) 0.071 0.083

3	(0.770) 0.786 0.762

1	(0.153) 0.142 0.155

Locus 13089 :

2 alleles

99.2% missing data

3	(0.442) 0.444 0.439

1	(0.558) 0.556 0.561

Locus 13090 :

2 alleles

99.2% missing data

2	(0.650) 0.644 0.656

1	(0.350) 0.356 0.344

Locus 13091 :

2 alleles

99.2% missing data

3	(0.704) 0.704 0.711

4	(0.296) 0.296 0.289

Locus 13092 :

2 alleles

99.2% missing data

1	(0.608) 0.604 0.611

2	(0.392) 0.396 0.389

Locus 13093 :

2 alleles

98.4% missing data

1	(0.502) 0.508 0.502

4	(0.498) 0.492 0.498

Locus 13094 :

2 alleles

99.2% missing data

1	(0.621) 0.619 0.612

4	(0.379) 0.381 0.388

Locus 13095 :

3 alleles

98.8% missing data

1	(0.297) 0.299 0.299

4	(0.380) 0.371 0.381

2	(0.323) 0.329 0.320

Locus 13096 :

2 alleles

99.2% missing data

2	(0.591) 0.586 0.589

3	(0.409) 0.414 0.411

Locus 13097 :

2 alleles

95.7% missing data

2	(0.422) 0.397 0.434

3	(0.578) 0.603 0.566

Locus 13098 :

2 alleles

99.2% missing data

2	(0.283) 0.281 0.283

3	(0.717) 0.719 0.717

Locus 13099 :

2 alleles

99.2% missing data

1	(0.627) 0.627 0.619

3	(0.373) 0.373 0.381

Locus 13100 :

2 alleles

98.8% missing data

1	(0.477) 0.472 0.486

4	(0.523) 0.528 0.514

Locus 13101 :

2 alleles

99.2% missing data

4	(0.726) 0.727 0.726

2	(0.274) 0.273 0.274

Locus 13102 :

2 alleles

99.2% missing data

3	(0.573) 0.575 0.573

4	(0.427) 0.425 0.427

Locus 13103 :

2 alleles

99.2% missing data

3	(0.464) 0.466 0.467

1	(0.536) 0.534 0.533

Locus 13104 :

2 alleles

8.2% missing data

3	(0.980) 0.972 0.994

4	(0.020) 0.028 0.006

Locus 13105 :

2 alleles

98.4% missing data

1	(0.421) 0.408 0.429

4	(0.579) 0.592 0.571

Locus 13106 :

2 alleles

99.2% missing data

3	(0.445) 0.438 0.440

2	(0.555) 0.562 0.560

Locus 13107 :

2 alleles

97.3% missing data

3	(0.146) 0.147 0.143

1	(0.854) 0.853 0.857

Locus 13108 :

2 alleles

99.2% missing data

2	(0.308) 0.305 0.308

3	(0.692) 0.695 0.692

Locus 13109 :

2 alleles

92.2% missing data

1	(0.826) 0.813 0.853

4	(0.174) 0.187 0.147

Locus 13110 :

2 alleles

99.2% missing data

1	(0.505) 0.500 0.507

3	(0.495) 0.500 0.493

Locus 13111 :

2 alleles

99.2% missing data

3	(0.561) 0.560 0.560

2	(0.439) 0.440 0.440

Locus 13112 :

2 alleles

99.2% missing data

3	(0.426) 0.421 0.426

1	(0.574) 0.579 0.574

Locus 13113 :

2 alleles

99.2% missing data

4	(0.549) 0.544 0.551

3	(0.451) 0.456 0.449

Locus 13114 :

3 alleles

98.8% missing data

1	(0.372) 0.366 0.373

2	(0.227) 0.225 0.236

3	(0.401) 0.410 0.391

Locus 13115 :

2 alleles

98.0% missing data

3	(0.825) 0.824 0.832

1	(0.175) 0.176 0.168

Locus 13116 :

2 alleles

99.2% missing data

1	(0.389) 0.383 0.397

3	(0.611) 0.617 0.603

Locus 13117 :

2 alleles

98.0% missing data

1	(0.216) 0.224 0.213

4	(0.784) 0.776 0.787

Locus 13118 :

2 alleles

98.4% missing data

2	(0.595) 0.601 0.591

1	(0.405) 0.399 0.409

Locus 13119 :

2 alleles

98.8% missing data

1	(0.439) 0.437 0.431

3	(0.561) 0.563 0.569

Locus 13120 :

2 alleles

99.2% missing data

2	(0.629) 0.620 0.639

1	(0.371) 0.380 0.361

Locus 13121 :

2 alleles

99.2% missing data

3	(0.268) 0.272 0.270

1	(0.732) 0.728 0.730

Locus 13122 :

2 alleles

99.2% missing data

3	(0.368) 0.368 0.378

4	(0.632) 0.632 0.622

Locus 13123 :

2 alleles

98.8% missing data

3	(0.616) 0.620 0.617

2	(0.384) 0.380 0.383

Locus 13124 :

2 alleles

99.2% missing data

4	(0.434) 0.433 0.435

2	(0.566) 0.567 0.565

Locus 13125 :

2 alleles

98.0% missing data

3	(0.805) 0.807 0.799

2	(0.195) 0.193 0.201

Locus 13126 :

3 alleles

98.4% missing data

1	(0.378) 0.377 0.373

2	(0.369) 0.364 0.370

3	(0.253) 0.259 0.256

Locus 13127 :

2 alleles

99.2% missing data

3	(0.820) 0.809 0.824

2	(0.180) 0.191 0.176

Locus 13128 :

2 alleles

99.2% missing data

3	(0.344) 0.353 0.339

1	(0.656) 0.647 0.661

Locus 13129 :

2 alleles

99.2% missing data

1	(0.536) 0.531 0.536

3	(0.464) 0.469 0.464

Locus 13130 :

2 alleles

99.2% missing data

3	(0.669) 0.677 0.663

2	(0.331) 0.323 0.337

Locus 13131 :

2 alleles

98.8% missing data

2	(0.817) 0.820 0.817

4	(0.183) 0.180 0.183

Locus 13132 :

2 alleles

99.2% missing data

1	(0.237) 0.232 0.246

3	(0.763) 0.768 0.754

Locus 13133 :

2 alleles

99.2% missing data

4	(0.414) 0.412 0.418

3	(0.586) 0.588 0.582

Locus 13134 :

2 alleles

99.2% missing data

1	(0.430) 0.429 0.425

3	(0.570) 0.571 0.575

Locus 13135 :

2 alleles

99.2% missing data

4	(0.207) 0.209 0.208

3	(0.793) 0.791 0.792

Locus 13136 :

2 alleles

99.2% missing data

2	(0.421) 0.426 0.422

3	(0.579) 0.574 0.578

Locus 13137 :

2 alleles

99.2% missing data

3	(0.656) 0.651 0.665

1	(0.344) 0.349 0.335

Locus 13138 :

2 alleles

98.8% missing data

1	(0.713) 0.712 0.710

4	(0.287) 0.288 0.290

Locus 13139 :

2 alleles

99.2% missing data

3	(0.440) 0.442 0.436

2	(0.560) 0.558 0.564

Locus 13140 :

2 alleles

99.2% missing data

1	(0.815) 0.817 0.806

4	(0.185) 0.183 0.194

Locus 13141 :

2 alleles

0.0% missing data

4	(0.987) 0.990 0.993

3	(0.013) 0.010 0.007

Locus 13142 :

2 alleles

99.2% missing data

1	(0.273) 0.271 0.277

4	(0.727) 0.729 0.723

Locus 13143 :

2 alleles

99.2% missing data

4	(0.578) 0.574 0.580

2	(0.422) 0.426 0.420

Locus 13144 :

2 alleles

99.2% missing data

4	(0.766) 0.770 0.762

2	(0.234) 0.230 0.238

Locus 13145 :

2 alleles

0.4% missing data

1	(0.985) 0.981 0.995

4	(0.015) 0.019 0.005

Locus 13146 :

2 alleles

98.4% missing data

1	(0.845) 0.842 0.849

3	(0.155) 0.158 0.151

Locus 13147 :

2 alleles

99.2% missing data

1	(0.297) 0.291 0.298

3	(0.703) 0.709 0.702

Locus 13148 :

2 alleles

99.2% missing data

3	(0.333) 0.335 0.335

1	(0.667) 0.665 0.665

Locus 13149 :

2 alleles

98.8% missing data

1	(0.791) 0.793 0.786

4	(0.209) 0.207 0.214

Locus 13150 :

2 alleles

98.8% missing data

3	(0.605) 0.610 0.603

1	(0.395) 0.390 0.397

Locus 13151 :

3 alleles

98.8% missing data

2	(0.250) 0.247 0.258

1	(0.462) 0.462 0.461

4	(0.288) 0.291 0.281

Locus 13152 :

2 alleles

99.2% missing data

2	(0.558) 0.563 0.553

4	(0.442) 0.437 0.447

Locus 13153 :

2 alleles

99.2% missing data

3	(0.594) 0.589 0.595

1	(0.406) 0.411 0.405

Locus 13154 :

2 alleles

98.8% missing data

3	(0.305) 0.303 0.304

1	(0.695) 0.697 0.696

Locus 13155 :

2 alleles

99.2% missing data

3	(0.694) 0.691 0.695

2	(0.306) 0.309 0.305

Locus 13156 :

2 alleles

99.2% missing data

2	(0.359) 0.358 0.361

1	(0.641) 0.642 0.639

Locus 13157 :

2 alleles

99.2% missing data

3	(0.613) 0.611 0.615

4	(0.387) 0.389 0.385

Locus 13158 :

2 alleles

93.0% missing data

1	(0.902) 0.906 0.909

4	(0.098) 0.094 0.091

Locus 13159 :

2 alleles

8.2% missing data

3	(0.986) 0.989 0.992

1	(0.014) 0.011 0.008

Locus 13160 :

2 alleles

98.8% missing data

4	(0.384) 0.386 0.385

2	(0.616) 0.614 0.615

Locus 13161 :

2 alleles

99.2% missing data

4	(0.570) 0.565 0.573

1	(0.430) 0.435 0.427

Locus 13162 :

2 alleles

99.2% missing data

2	(0.585) 0.585 0.578

4	(0.415) 0.415 0.422

Locus 13163 :

2 alleles

98.8% missing data

1	(0.756) 0.754 0.758

3	(0.244) 0.246 0.242

Locus 13164 :

2 alleles

98.8% missing data

3	(0.464) 0.470 0.460

4	(0.536) 0.530 0.540

Locus 13165 :

2 alleles

99.2% missing data

1	(0.365) 0.360 0.373

4	(0.635) 0.640 0.627

Locus 13166 :

2 alleles

98.8% missing data

1	(0.562) 0.556 0.566

3	(0.438) 0.444 0.434

Locus 13167 :

2 alleles

99.2% missing data

4	(0.739) 0.735 0.743

1	(0.261) 0.265 0.257

Locus 13168 :

2 alleles

99.2% missing data

4	(0.534) 0.530 0.533

1	(0.466) 0.470 0.467

Locus 13169 :

2 alleles

99.2% missing data

3	(0.661) 0.661 0.658

1	(0.339) 0.339 0.342

Locus 13170 :

2 alleles

99.2% missing data

3	(0.841) 0.837 0.846

2	(0.159) 0.163 0.154

Locus 13171 :

2 alleles

99.2% missing data

1	(0.586) 0.573 0.589

2	(0.414) 0.427 0.411

Locus 13172 :

2 alleles

99.2% missing data

1	(0.601) 0.606 0.600

4	(0.399) 0.394 0.400

Locus 13173 :

2 alleles

99.2% missing data

1	(0.445) 0.444 0.450

3	(0.555) 0.556 0.550

Locus 13174 :

3 alleles

98.8% missing data

3	(0.138) 0.136 0.140

1	(0.528) 0.531 0.531

2	(0.334) 0.333 0.329

Locus 13175 :

2 alleles

99.2% missing data

4	(0.692) 0.689 0.691

1	(0.308) 0.311 0.309

Locus 13176 :

2 alleles

99.2% missing data

1	(0.438) 0.435 0.447

2	(0.562) 0.565 0.553

Locus 13177 :

2 alleles

98.4% missing data

1	(0.712) 0.722 0.706

3	(0.288) 0.278 0.294

Locus 13178 :

2 alleles

99.2% missing data

1	(0.210) 0.211 0.209

2	(0.790) 0.789 0.791

Locus 13179 :

2 alleles

99.2% missing data

4	(0.708) 0.703 0.713

2	(0.292) 0.297 0.287

Locus 13180 :

2 alleles

98.0% missing data

2	(0.730) 0.730 0.734

4	(0.270) 0.270 0.266

Locus 13181 :

2 alleles

99.2% missing data

1	(0.371) 0.366 0.374

3	(0.629) 0.634 0.626

Locus 13182 :

2 alleles

94.9% missing data

1	(0.821) 0.831 0.822

3	(0.179) 0.169 0.178

Locus 13183 :

2 alleles

95.3% missing data

2	(0.817) 0.809 0.833

1	(0.183) 0.191 0.167

Locus 13184 :

3 alleles

98.0% missing data

2	(0.431) 0.427 0.430

4	(0.283) 0.283 0.284

1	(0.285) 0.290 0.286

Locus 13185 :

3 alleles

98.8% missing data

2	(0.512) 0.499 0.518

3	(0.181) 0.190 0.174

4	(0.307) 0.311 0.308

Locus 13186 :

2 alleles

98.0% missing data

3	(0.711) 0.722 0.705

1	(0.289) 0.278 0.295

Locus 13187 :

2 alleles

99.2% missing data

3	(0.419) 0.406 0.426

2	(0.581) 0.594 0.574

Locus 13188 :

2 alleles

99.2% missing data

1	(0.444) 0.449 0.446

4	(0.556) 0.551 0.554

Locus 13189 :

2 alleles

99.2% missing data

2	(0.369) 0.369 0.364

1	(0.631) 0.631 0.636

Locus 13190 :

2 alleles

98.8% missing data

1	(0.468) 0.470 0.472

2	(0.532) 0.530 0.528

Locus 13191 :

2 alleles

99.2% missing data

1	(0.764) 0.762 0.771

2	(0.236) 0.238 0.229

Locus 13192 :

2 alleles

99.2% missing data

4	(0.327) 0.328 0.326

3	(0.673) 0.672 0.674

Locus 13193 :

2 alleles

99.2% missing data

3	(0.725) 0.728 0.718

2	(0.275) 0.272 0.282

Locus 13194 :

2 alleles

99.2% missing data

1	(0.326) 0.323 0.332

2	(0.674) 0.677 0.668

Locus 13195 :

2 alleles

99.2% missing data

1	(0.757) 0.761 0.754

3	(0.243) 0.239 0.246

Locus 13196 :

2 alleles

98.8% missing data

1	(0.476) 0.482 0.474

4	(0.524) 0.518 0.526

Locus 13197 :

2 alleles

99.2% missing data

4	(0.253) 0.255 0.252

2	(0.747) 0.745 0.748

Locus 13198 :

2 alleles

98.8% missing data

1	(0.393) 0.385 0.398

2	(0.607) 0.615 0.602

Locus 13199 :

2 alleles

99.2% missing data

4	(0.374) 0.372 0.372

2	(0.626) 0.628 0.628

Locus 13200 :

2 alleles

99.2% missing data

1	(0.624) 0.627 0.625

4	(0.376) 0.373 0.375

Locus 13201 :

2 alleles

93.0% missing data

3	(0.409) 0.457 0.370

2	(0.591) 0.543 0.630

Locus 13202 :

2 alleles

99.2% missing data

4	(0.343) 0.345 0.342

1	(0.657) 0.655 0.658

Locus 13203 :

2 alleles

98.8% missing data

3	(0.363) 0.363 0.362

4	(0.637) 0.637 0.638

Locus 13204 :

2 alleles

99.2% missing data

1	(0.678) 0.677 0.682

2	(0.322) 0.323 0.318

Locus 13205 :

2 alleles

99.2% missing data

1	(0.680) 0.677 0.681

4	(0.320) 0.323 0.319

Locus 13206 :

2 alleles

99.2% missing data

3	(0.167) 0.163 0.171

1	(0.833) 0.837 0.829

Locus 13207 :

2 alleles

99.2% missing data

1	(0.511) 0.505 0.518

2	(0.489) 0.495 0.482

Locus 13208 :

2 alleles

99.2% missing data

1	(0.752) 0.756 0.749

4	(0.248) 0.244 0.251

Locus 13209 :

2 alleles

99.2% missing data

3	(0.322) 0.316 0.326

1	(0.678) 0.684 0.674

Locus 13210 :

2 alleles

99.2% missing data

2	(0.421) 0.420 0.424

3	(0.579) 0.580 0.576

Locus 13211 :

2 alleles

91.4% missing data

1	(0.904) 0.924 0.895

3	(0.096) 0.076 0.105

Locus 13212 :

2 alleles

99.2% missing data

1	(0.649) 0.656 0.640

3	(0.351) 0.344 0.360

Locus 13213 :

2 alleles

98.8% missing data

2	(0.524) 0.516 0.533

4	(0.476) 0.484 0.467

Locus 13214 :

2 alleles

97.7% missing data

2	(0.625) 0.618 0.642

1	(0.375) 0.382 0.358

Locus 13215 :

2 alleles

98.8% missing data

1	(0.492) 0.481 0.496

4	(0.508) 0.519 0.504

Locus 13216 :

2 alleles

99.2% missing data

2	(0.420) 0.421 0.419

4	(0.580) 0.579 0.581

Locus 13217 :

2 alleles

98.8% missing data

4	(0.717) 0.712 0.729

2	(0.283) 0.288 0.271

Locus 13218 :

2 alleles

99.2% missing data

3	(0.759) 0.757 0.751

4	(0.241) 0.243 0.249

Locus 13219 :

2 alleles

99.2% missing data

4	(0.339) 0.335 0.344

2	(0.661) 0.665 0.656

Locus 13220 :

2 alleles

99.2% missing data

2	(0.299) 0.302 0.299

4	(0.701) 0.698 0.701

Locus 13221 :

2 alleles

98.0% missing data

4	(0.576) 0.565 0.585

1	(0.424) 0.435 0.415

Locus 13222 :

2 alleles

8.2% missing data

4	(0.983) 0.973 0.995

3	(0.017) 0.027 0.005

Locus 13223 :

2 alleles

99.2% missing data

4	(0.457) 0.448 0.463

2	(0.543) 0.552 0.537

Locus 13224 :

2 alleles

98.8% missing data

2	(0.557) 0.559 0.555

3	(0.443) 0.441 0.445

Locus 13225 :

3 alleles

98.0% missing data

2	(0.569) 0.577 0.566

4	(0.230) 0.214 0.237

3	(0.201) 0.209 0.197

Locus 13226 :

2 alleles

99.2% missing data

2	(0.502) 0.511 0.496

3	(0.498) 0.489 0.504

Locus 13227 :

2 alleles

99.2% missing data

1	(0.258) 0.256 0.262

2	(0.742) 0.744 0.738

Locus 13228 :

2 alleles

93.0% missing data

1	(0.861) 0.856 0.880

2	(0.139) 0.144 0.120

Locus 13229 :

2 alleles

97.7% missing data

4	(0.372) 0.380 0.361

3	(0.628) 0.620 0.639

Locus 13230 :

2 alleles

99.2% missing data

4	(0.665) 0.669 0.663

2	(0.335) 0.331 0.337

Locus 13231 :

2 alleles

97.3% missing data

2	(0.279) 0.282 0.267

1	(0.721) 0.718 0.733

Locus 13232 :

2 alleles

99.2% missing data

4	(0.655) 0.661 0.650

3	(0.345) 0.339 0.350

Locus 13233 :

2 alleles

99.2% missing data

2	(0.823) 0.825 0.814

4	(0.177) 0.175 0.186

Locus 13234 :

2 alleles

99.2% missing data

4	(0.464) 0.464 0.464

3	(0.536) 0.536 0.536

Locus 13235 :

2 alleles

99.2% missing data

1	(0.168) 0.167 0.176

4	(0.832) 0.833 0.824

Locus 13236 :

2 alleles

96.1% missing data

4	(0.881) 0.882 0.885

3	(0.119) 0.118 0.115

Locus 13237 :

2 alleles

98.4% missing data

2	(0.332) 0.325 0.331

4	(0.668) 0.675 0.669

Locus 13238 :

3 alleles

98.0% missing data

4	(0.434) 0.447 0.436

2	(0.191) 0.184 0.193

1	(0.375) 0.369 0.371

Locus 13239 :

2 alleles

99.2% missing data

2	(0.696) 0.704 0.689

3	(0.304) 0.296 0.311

Locus 13240 :

3 alleles

95.7% missing data

3	(0.678) 0.695 0.670

2	(0.189) 0.178 0.200

1	(0.133) 0.127 0.129

Locus 13241 :

2 alleles

99.2% missing data

4	(0.542) 0.539 0.554

1	(0.458) 0.461 0.446

Locus 13242 :

2 alleles

89.5% missing data

3	(0.937) 0.928 0.950

4	(0.063) 0.072 0.050

Locus 13243 :

2 alleles

98.8% missing data

2	(0.618) 0.609 0.624

1	(0.382) 0.391 0.376

Locus 13244 :

2 alleles

97.7% missing data

3	(0.799) 0.802 0.803

1	(0.201) 0.198 0.197

Locus 13245 :

2 alleles

99.2% missing data

2	(0.321) 0.318 0.324

4	(0.679) 0.682 0.676

Locus 13246 :

2 alleles

99.2% missing data

2	(0.428) 0.427 0.430

4	(0.572) 0.573 0.570

Locus 13247 :

2 alleles

93.0% missing data

3	(0.905) 0.898 0.917

2	(0.095) 0.102 0.083

Locus 13248 :

2 alleles

99.2% missing data

4	(0.326) 0.332 0.319

2	(0.674) 0.668 0.681

Locus 13249 :

2 alleles

98.8% missing data

4	(0.388) 0.381 0.387

3	(0.612) 0.619 0.613

Locus 13250 :

2 alleles

99.2% missing data

3	(0.806) 0.807 0.803

4	(0.194) 0.193 0.197

Locus 13251 :

2 alleles

99.2% missing data

3	(0.572) 0.573 0.576

2	(0.428) 0.427 0.424

Locus 13252 :

2 alleles

97.3% missing data

4	(0.357) 0.351 0.356

2	(0.643) 0.649 0.644

Locus 13253 :

2 alleles

99.2% missing data

2	(0.458) 0.459 0.458

3	(0.542) 0.541 0.542

Locus 13254 :

2 alleles

99.2% missing data

4	(0.520) 0.521 0.521

2	(0.480) 0.479 0.479

Locus 13255 :

2 alleles

98.0% missing data

1	(0.843) 0.843 0.843

3	(0.157) 0.157 0.157

Locus 13256 :

2 alleles

99.2% missing data

2	(0.337) 0.338 0.342

3	(0.663) 0.662 0.658

Locus 13257 :

2 alleles

99.2% missing data

2	(0.322) 0.316 0.321

4	(0.678) 0.684 0.679

Locus 13258 :

2 alleles

99.2% missing data

2	(0.371) 0.375 0.369

4	(0.629) 0.625 0.631

Locus 13259 :

2 alleles

98.8% missing data

4	(0.717) 0.713 0.713

1	(0.283) 0.287 0.287

Locus 13260 :

2 alleles

99.2% missing data

1	(0.310) 0.307 0.319

2	(0.690) 0.693 0.681

Locus 13261 :

3 alleles

98.0% missing data

4	(0.516) 0.526 0.517

2	(0.211) 0.209 0.209

3	(0.273) 0.265 0.274

Locus 13262 :

2 alleles

99.2% missing data

2	(0.267) 0.263 0.272

1	(0.733) 0.737 0.728

Locus 13263 :

3 alleles

98.0% missing data

3	(0.203) 0.208 0.202

2	(0.539) 0.545 0.537

4	(0.258) 0.248 0.261

Locus 13264 :

2 alleles

91.0% missing data

2	(0.745) 0.748 0.741

3	(0.255) 0.252 0.259

Locus 13265 :

3 alleles

98.8% missing data

2	(0.244) 0.246 0.248

4	(0.364) 0.363 0.358

1	(0.391) 0.392 0.394

Locus 13266 :

2 alleles

99.2% missing data

4	(0.800) 0.805 0.792

1	(0.200) 0.195 0.208

Locus 13267 :

2 alleles

99.2% missing data

1	(0.488) 0.486 0.488

3	(0.512) 0.514 0.512

Locus 13268 :

2 alleles

98.4% missing data

2	(0.506) 0.518 0.503

4	(0.494) 0.482 0.497

Locus 13269 :

2 alleles

97.7% missing data

3	(0.219) 0.217 0.214

1	(0.781) 0.783 0.786

Locus 13270 :

2 alleles

96.1% missing data

2	(0.896) 0.902 0.889

1	(0.104) 0.098 0.111

Locus 13271 :

2 alleles

99.2% missing data

2	(0.640) 0.644 0.636

4	(0.360) 0.356 0.364

Locus 13272 :

3 alleles

98.8% missing data

4	(0.418) 0.419 0.422

1	(0.302) 0.295 0.304

2	(0.281) 0.286 0.274

Locus 13273 :

2 alleles

99.2% missing data

1	(0.380) 0.382 0.383

3	(0.620) 0.618 0.617

Locus 13274 :

2 alleles

91.0% missing data

1	(0.924) 0.941 0.916

4	(0.076) 0.059 0.084

Locus 13275 :

2 alleles

90.6% missing data

2	(0.908) 0.921 0.901

1	(0.092) 0.079 0.099

Locus 13276 :

2 alleles

99.2% missing data

1	(0.511) 0.512 0.509

4	(0.489) 0.488 0.491

Locus 13277 :

2 alleles

99.2% missing data

4	(0.292) 0.289 0.292

2	(0.708) 0.711 0.708

Locus 13278 :

2 alleles

99.2% missing data

3	(0.486) 0.488 0.489

2	(0.514) 0.512 0.511

Locus 13279 :

2 alleles

99.2% missing data

4	(0.454) 0.451 0.460

3	(0.546) 0.549 0.540

Locus 13280 :

2 alleles

99.2% missing data

2	(0.633) 0.641 0.628

4	(0.367) 0.359 0.372

Locus 13281 :

2 alleles

99.2% missing data

3	(0.566) 0.557 0.570

4	(0.434) 0.443 0.430

Locus 13282 :

2 alleles

0.0% missing data

4	(0.987) 0.991 0.993

3	(0.013) 0.009 0.007

Locus 13283 :

2 alleles

98.8% missing data

1	(0.695) 0.690 0.699

2	(0.305) 0.310 0.301

Locus 13284 :

2 alleles

99.2% missing data

4	(0.429) 0.420 0.436

1	(0.571) 0.580 0.564

Locus 13285 :

2 alleles

98.4% missing data

1	(0.649) 0.640 0.661

4	(0.351) 0.360 0.339

Locus 13286 :

2 alleles

99.2% missing data

3	(0.425) 0.421 0.439

2	(0.575) 0.579 0.561

Locus 13287 :

2 alleles

99.2% missing data

2	(0.423) 0.419 0.428

4	(0.577) 0.581 0.572

Locus 13288 :

2 alleles

98.0% missing data

3	(0.643) 0.640 0.651

4	(0.357) 0.360 0.349

Locus 13289 :

2 alleles

96.1% missing data

1	(0.827) 0.844 0.822

3	(0.173) 0.156 0.178

Locus 13290 :

2 alleles

99.2% missing data

2	(0.203) 0.207 0.200

4	(0.797) 0.793 0.800

Locus 13291 :

2 alleles

98.8% missing data

1	(0.354) 0.346 0.358

3	(0.646) 0.654 0.642

Locus 13292 :

2 alleles

94.5% missing data

2	(0.838) 0.840 0.852

1	(0.162) 0.160 0.148

Locus 13293 :

2 alleles

99.2% missing data

4	(0.406) 0.403 0.412

1	(0.594) 0.597 0.588

Locus 13294 :

2 alleles

99.2% missing data

3	(0.492) 0.488 0.488

2	(0.508) 0.512 0.512

Locus 13295 :

2 alleles

99.2% missing data

3	(0.353) 0.345 0.357

4	(0.647) 0.655 0.643

Locus 13296 :

2 alleles

99.2% missing data

2	(0.725) 0.722 0.727

4	(0.275) 0.278 0.273

Locus 13297 :

2 alleles

98.4% missing data

3	(0.694) 0.702 0.694

4	(0.306) 0.298 0.306

Locus 13298 :

2 alleles

99.2% missing data

3	(0.478) 0.472 0.483

4	(0.522) 0.528 0.517

Locus 13299 :

2 alleles

99.2% missing data

4	(0.287) 0.286 0.297

1	(0.713) 0.714 0.703

Locus 13300 :

2 alleles

98.8% missing data

4	(0.583) 0.572 0.597

3	(0.417) 0.428 0.403

Locus 13301 :

2 alleles

98.0% missing data

4	(0.413) 0.412 0.415

2	(0.587) 0.588 0.585

Locus 13302 :

2 alleles

99.2% missing data

2	(0.268) 0.273 0.266

4	(0.732) 0.727 0.734

Locus 13303 :

3 alleles

98.8% missing data

2	(0.515) 0.517 0.512

4	(0.292) 0.287 0.296

1	(0.193) 0.196 0.191

Locus 13304 :

2 alleles

99.2% missing data

4	(0.689) 0.692 0.685

3	(0.311) 0.308 0.315

Locus 13305 :

2 alleles

98.8% missing data

4	(0.372) 0.364 0.377

3	(0.628) 0.636 0.623

Locus 13306 :

2 alleles

99.2% missing data

1	(0.779) 0.782 0.777

4	(0.221) 0.218 0.223

Locus 13307 :

2 alleles

98.8% missing data

3	(0.749) 0.740 0.754

4	(0.251) 0.260 0.246

Locus 13308 :

2 alleles

99.2% missing data

3	(0.519) 0.513 0.524

2	(0.481) 0.487 0.476

Locus 13309 :

3 alleles

98.8% missing data

3	(0.666) 0.668 0.664

4	(0.222) 0.217 0.229

2	(0.112) 0.115 0.107

Locus 13310 :

2 alleles

98.8% missing data

4	(0.697) 0.698 0.698

2	(0.303) 0.302 0.302

Locus 13311 :

2 alleles

98.8% missing data

3	(0.389) 0.393 0.389

1	(0.611) 0.607 0.611

Locus 13312 :

3 alleles

98.8% missing data

1	(0.305) 0.303 0.305

2	(0.396) 0.401 0.400

3	(0.299) 0.296 0.296

Locus 13313 :

2 alleles

98.8% missing data

3	(0.296) 0.295 0.306

4	(0.704) 0.705 0.694

Locus 13314 :

2 alleles

99.2% missing data

4	(0.580) 0.574 0.580

3	(0.420) 0.426 0.420

Locus 13315 :

2 alleles

99.2% missing data

2	(0.633) 0.626 0.642

4	(0.367) 0.374 0.358

Locus 13316 :

2 alleles

99.2% missing data

2	(0.524) 0.517 0.525

3	(0.476) 0.483 0.475

Locus 13317 :

2 alleles

98.8% missing data

2	(0.733) 0.737 0.730

4	(0.267) 0.263 0.270

Locus 13318 :

2 alleles

98.8% missing data

3	(0.573) 0.571 0.577

1	(0.427) 0.429 0.423

Locus 13319 :

2 alleles

99.2% missing data

3	(0.605) 0.608 0.603

2	(0.395) 0.392 0.397

Locus 13320 :

2 alleles

99.2% missing data

3	(0.633) 0.637 0.631

2	(0.367) 0.363 0.369

Locus 13321 :

2 alleles

99.2% missing data

3	(0.793) 0.786 0.797

4	(0.207) 0.214 0.203

Locus 13322 :

2 alleles

99.2% missing data

2	(0.565) 0.562 0.566

4	(0.435) 0.438 0.434

Locus 13323 :

2 alleles

99.2% missing data

1	(0.395) 0.391 0.402

3	(0.605) 0.609 0.598

Locus 13324 :

2 alleles

99.2% missing data

4	(0.366) 0.359 0.375

2	(0.634) 0.641 0.625

Locus 13325 :

2 alleles

96.1% missing data

2	(0.172) 0.159 0.178

3	(0.828) 0.841 0.822

Locus 13326 :

2 alleles

99.2% missing data

2	(0.487) 0.486 0.483

3	(0.513) 0.514 0.517

Locus 13327 :

2 alleles

96.9% missing data

3	(0.786) 0.778 0.799

1	(0.214) 0.222 0.201

Locus 13328 :

2 alleles

98.8% missing data

2	(0.564) 0.559 0.567

4	(0.436) 0.441 0.433

Locus 13329 :

2 alleles

98.8% missing data

2	(0.561) 0.561 0.571

1	(0.439) 0.439 0.429

Locus 13330 :

2 alleles

99.2% missing data

1	(0.653) 0.650 0.654

3	(0.347) 0.350 0.346

Locus 13331 :

2 alleles

99.2% missing data

1	(0.691) 0.696 0.690

4	(0.309) 0.304 0.310

Locus 13332 :

2 alleles

4.7% missing data

1	(0.980) 0.968 0.994

4	(0.020) 0.032 0.006

Locus 13333 :

2 alleles

99.2% missing data

3	(0.621) 0.621 0.622

4	(0.379) 0.379 0.378

Locus 13334 :

2 alleles

99.2% missing data

3	(0.572) 0.568 0.577

4	(0.428) 0.432 0.423

Locus 13335 :

2 alleles

99.2% missing data

4	(0.734) 0.732 0.730

1	(0.266) 0.268 0.270

Locus 13336 :

2 alleles

98.0% missing data

1	(0.653) 0.660 0.652

4	(0.347) 0.340 0.348

Locus 13337 :

3 alleles

98.8% missing data

2	(0.180) 0.184 0.182

4	(0.376) 0.375 0.379

1	(0.444) 0.442 0.439

Locus 13338 :

2 alleles

99.2% missing data

2	(0.532) 0.535 0.524

1	(0.468) 0.465 0.476

Locus 13339 :

2 alleles

99.2% missing data

1	(0.519) 0.515 0.526

4	(0.481) 0.485 0.474

Locus 13340 :

2 alleles

99.2% missing data

1	(0.628) 0.626 0.628

4	(0.372) 0.374 0.372

Locus 13341 :

2 alleles

99.2% missing data

4	(0.499) 0.499 0.496

1	(0.501) 0.501 0.504

Locus 13342 :

2 alleles

98.4% missing data

3	(0.723) 0.730 0.721

2	(0.277) 0.270 0.279

Locus 13343 :

2 alleles

99.2% missing data

2	(0.429) 0.430 0.432

1	(0.571) 0.570 0.568

Locus 13344 :

2 alleles

97.3% missing data

1	(0.844) 0.844 0.847

3	(0.156) 0.156 0.153

Locus 13345 :

2 alleles

90.6% missing data

3	(0.911) 0.928 0.905

1	(0.089) 0.072 0.095

Locus 13346 :

2 alleles

99.2% missing data

4	(0.305) 0.302 0.311

3	(0.695) 0.698 0.689

Locus 13347 :

2 alleles

99.2% missing data

2	(0.600) 0.597 0.601

1	(0.400) 0.403 0.399

Locus 13348 :

2 alleles

99.2% missing data

3	(0.582) 0.581 0.582

1	(0.418) 0.419 0.418

Locus 13349 :

2 alleles

98.8% missing data

1	(0.564) 0.561 0.563

2	(0.436) 0.439 0.437

Locus 13350 :

2 alleles

99.2% missing data

2	(0.612) 0.608 0.610

1	(0.388) 0.392 0.390

Locus 13351 :

2 alleles

98.4% missing data

3	(0.643) 0.642 0.646

1	(0.357) 0.358 0.354

Locus 13352 :

3 alleles

96.5% missing data

2	(0.652) 0.673 0.644

4	(0.214) 0.194 0.222

3	(0.135) 0.133 0.134

Locus 13353 :

2 alleles

99.2% missing data

1	(0.697) 0.699 0.693

2	(0.303) 0.301 0.307

Locus 13354 :

2 alleles

99.2% missing data

1	(0.539) 0.530 0.549

4	(0.461) 0.470 0.451

Locus 13355 :

2 alleles

97.3% missing data

1	(0.726) 0.729 0.735

2	(0.274) 0.271 0.265

Locus 13356 :

2 alleles

98.4% missing data

3	(0.259) 0.265 0.253

1	(0.741) 0.735 0.747

Locus 13357 :

2 alleles

99.2% missing data

3	(0.278) 0.281 0.280

2	(0.722) 0.719 0.720

Locus 13358 :

2 alleles

99.2% missing data

1	(0.791) 0.784 0.798

3	(0.209) 0.216 0.202

Locus 13359 :

3 alleles

98.4% missing data

1	(0.333) 0.324 0.338

4	(0.317) 0.326 0.318

3	(0.350) 0.350 0.344

Locus 13360 :

2 alleles

91.8% missing data

2	(0.783) 0.795 0.780

3	(0.217) 0.205 0.220

Locus 13361 :

2 alleles

99.2% missing data

1	(0.407) 0.403 0.413

4	(0.593) 0.597 0.587

Locus 13362 :

2 alleles

99.2% missing data

1	(0.559) 0.552 0.561

2	(0.441) 0.448 0.439

Locus 13363 :

2 alleles

99.2% missing data

1	(0.805) 0.801 0.807

2	(0.195) 0.199 0.193

Locus 13364 :

2 alleles

99.2% missing data

4	(0.307) 0.304 0.307

1	(0.693) 0.696 0.693

Locus 13365 :

2 alleles

99.2% missing data

3	(0.634) 0.633 0.637

2	(0.366) 0.367 0.363

Locus 13366 :

2 alleles

97.7% missing data

1	(0.308) 0.315 0.295

3	(0.692) 0.685 0.705

Locus 13367 :

2 alleles

96.1% missing data

1	(0.152) 0.142 0.153

4	(0.848) 0.858 0.847

Locus 13368 :

2 alleles

99.2% missing data

2	(0.672) 0.666 0.671

3	(0.328) 0.334 0.329

Locus 13369 :

2 alleles

99.2% missing data

3	(0.752) 0.755 0.744

1	(0.248) 0.245 0.256

Locus 13370 :

2 alleles

99.2% missing data

1	(0.538) 0.534 0.543

3	(0.462) 0.466 0.457

Locus 13371 :

2 alleles

99.2% missing data

3	(0.258) 0.260 0.258

2	(0.742) 0.740 0.742

Locus 13372 :

2 alleles

98.8% missing data

1	(0.641) 0.648 0.638

4	(0.359) 0.352 0.362

Locus 13373 :

2 alleles

99.2% missing data

3	(0.490) 0.484 0.494

2	(0.510) 0.516 0.506

Locus 13374 :

2 alleles

99.2% missing data

1	(0.438) 0.442 0.432

4	(0.562) 0.558 0.568

Locus 13375 :

2 alleles

99.2% missing data

1	(0.461) 0.458 0.458

3	(0.539) 0.542 0.542

Locus 13376 :

2 alleles

98.4% missing data

1	(0.719) 0.730 0.712

4	(0.281) 0.270 0.288

Locus 13377 :

2 alleles

99.2% missing data

3	(0.533) 0.532 0.533

2	(0.467) 0.468 0.467

Locus 13378 :

2 alleles

98.8% missing data

4	(0.569) 0.574 0.572

3	(0.431) 0.426 0.428

Locus 13379 :

2 alleles

98.0% missing data

1	(0.580) 0.586 0.581

3	(0.420) 0.414 0.419

Locus 13380 :

2 alleles

96.1% missing data

3	(0.758) 0.767 0.762

2	(0.242) 0.233 0.238

Locus 13381 :

2 alleles

99.2% missing data

2	(0.600) 0.600 0.603

4	(0.400) 0.400 0.397

Locus 13382 :

2 alleles

99.2% missing data

4	(0.734) 0.737 0.726

1	(0.266) 0.263 0.274

Locus 13383 :

4 alleles

98.4% missing data

3	(0.173) 0.174 0.181

4	(0.311) 0.313 0.303

1	(0.233) 0.233 0.236

2	(0.282) 0.280 0.280

Locus 13384 :

2 alleles

99.2% missing data

4	(0.514) 0.514 0.522

3	(0.486) 0.486 0.478

Locus 13385 :

2 alleles

99.2% missing data

3	(0.347) 0.346 0.347

1	(0.653) 0.654 0.653

Locus 13386 :

2 alleles

99.2% missing data

4	(0.816) 0.817 0.812

2	(0.184) 0.183 0.188

Locus 13387 :

2 alleles

99.2% missing data

3	(0.651) 0.645 0.658

4	(0.349) 0.355 0.342

Locus 13388 :

2 alleles

99.2% missing data

4	(0.441) 0.443 0.441

3	(0.559) 0.557 0.559

Locus 13389 :

2 alleles

99.2% missing data

2	(0.477) 0.472 0.481

3	(0.523) 0.528 0.519

Locus 13390 :

2 alleles

99.2% missing data

1	(0.580) 0.573 0.582

4	(0.420) 0.427 0.418

Locus 13391 :

2 alleles

99.2% missing data

1	(0.264) 0.271 0.265

4	(0.736) 0.729 0.735

Locus 13392 :

2 alleles

99.2% missing data

4	(0.571) 0.570 0.573

2	(0.429) 0.430 0.427

Locus 13393 :

2 alleles

99.2% missing data

2	(0.542) 0.537 0.540

1	(0.458) 0.463 0.460

Locus 13394 :

2 alleles

99.2% missing data

3	(0.394) 0.387 0.399

1	(0.606) 0.613 0.601

Locus 13395 :

2 alleles

99.2% missing data

4	(0.388) 0.385 0.392

2	(0.612) 0.615 0.608

Locus 13396 :

2 alleles

96.1% missing data

4	(0.209) 0.199 0.210

1	(0.791) 0.801 0.790

Locus 13397 :

2 alleles

99.2% missing data

3	(0.263) 0.263 0.263

1	(0.737) 0.737 0.737

Locus 13398 :

2 alleles

99.2% missing data

3	(0.750) 0.749 0.744

1	(0.250) 0.251 0.256

Locus 13399 :

2 alleles

99.2% missing data

3	(0.181) 0.178 0.178

2	(0.819) 0.822 0.822

Locus 13400 :

2 alleles

99.2% missing data

2	(0.515) 0.515 0.520

1	(0.485) 0.485 0.480

Locus 13401 :

2 alleles

99.2% missing data

3	(0.582) 0.588 0.573

1	(0.418) 0.412 0.427

Locus 13402 :

2 alleles

99.2% missing data

3	(0.416) 0.420 0.414

1	(0.584) 0.580 0.586

Locus 13403 :

2 alleles

99.2% missing data

2	(0.537) 0.539 0.540

1	(0.463) 0.461 0.460

Locus 13404 :

2 alleles

99.2% missing data

1	(0.232) 0.227 0.237

3	(0.768) 0.773 0.763

Locus 13405 :

2 alleles

99.2% missing data

1	(0.466) 0.466 0.460

3	(0.534) 0.534 0.540

Locus 13406 :

2 alleles

99.2% missing data

1	(0.657) 0.660 0.650

3	(0.343) 0.340 0.350

Locus 13407 :

2 alleles

98.8% missing data

1	(0.551) 0.555 0.549

3	(0.449) 0.445 0.451

Locus 13408 :

3 alleles

98.8% missing data

3	(0.392) 0.392 0.390

2	(0.343) 0.337 0.347

1	(0.265) 0.271 0.263

Locus 13409 :

2 alleles

99.2% missing data

2	(0.437) 0.430 0.439

3	(0.563) 0.570 0.561

Locus 13410 :

2 alleles

99.2% missing data

4	(0.464) 0.467 0.461

3	(0.536) 0.533 0.539

Locus 13411 :

2 alleles

99.2% missing data

2	(0.701) 0.697 0.700

1	(0.299) 0.303 0.300

Locus 13412 :

2 alleles

99.2% missing data

4	(0.312) 0.308 0.317

3	(0.688) 0.692 0.683

Locus 13413 :

2 alleles

98.0% missing data

2	(0.783) 0.777 0.793

1	(0.217) 0.223 0.207

Locus 13414 :

2 alleles

98.4% missing data

4	(0.537) 0.549 0.529

1	(0.463) 0.451 0.471

Locus 13415 :

2 alleles

99.2% missing data

3	(0.853) 0.847 0.860

1	(0.147) 0.153 0.140

Locus 13416 :

3 alleles

98.8% missing data

1	(0.239) 0.236 0.241

3	(0.366) 0.368 0.367

4	(0.395) 0.396 0.392

Locus 13417 :

2 alleles

99.2% missing data

1	(0.773) 0.773 0.773

3	(0.227) 0.227 0.227

Locus 13418 :

2 alleles

99.2% missing data

3	(0.567) 0.567 0.570

2	(0.433) 0.433 0.430

Locus 13419 :

2 alleles

99.2% missing data

1	(0.557) 0.558 0.555

4	(0.443) 0.442 0.445

Locus 13420 :

2 alleles

99.2% missing data

3	(0.371) 0.371 0.370

1	(0.629) 0.629 0.630

Locus 13421 :

2 alleles

90.6% missing data

3	(0.904) 0.913 0.901

1	(0.096) 0.087 0.099

Locus 13422 :

2 alleles

98.8% missing data

4	(0.472) 0.467 0.482

2	(0.528) 0.533 0.518

Locus 13423 :

2 alleles

97.3% missing data

4	(0.723) 0.722 0.731

1	(0.277) 0.278 0.269

Locus 13424 :

2 alleles

99.2% missing data

3	(0.424) 0.419 0.430

2	(0.576) 0.581 0.570

Locus 13425 :

2 alleles

97.7% missing data

2	(0.215) 0.218 0.208

4	(0.785) 0.782 0.792

Locus 13426 :

2 alleles

99.2% missing data

2	(0.626) 0.624 0.629

3	(0.374) 0.376 0.371

Locus 13427 :

2 alleles

99.2% missing data

1	(0.177) 0.179 0.177

2	(0.823) 0.821 0.823

Locus 13428 :

2 alleles

99.2% missing data

1	(0.320) 0.315 0.326

2	(0.680) 0.685 0.674

Locus 13429 :

2 alleles

99.2% missing data

2	(0.743) 0.739 0.745

1	(0.257) 0.261 0.255

Locus 13430 :

3 alleles

91.0% missing data

2	(0.802) 0.835 0.784

3	(0.091) 0.077 0.101

1	(0.107) 0.088 0.116

Locus 13431 :

2 alleles

99.2% missing data

4	(0.199) 0.200 0.204

3	(0.801) 0.800 0.796

Locus 13432 :

2 alleles

98.8% missing data

2	(0.369) 0.384 0.366

3	(0.631) 0.616 0.634

Locus 13433 :

2 alleles

99.2% missing data

4	(0.622) 0.620 0.626

1	(0.378) 0.380 0.374

Locus 13434 :

2 alleles

95.7% missing data

4	(0.834) 0.837 0.838

1	(0.166) 0.163 0.162

Locus 13435 :

2 alleles

99.2% missing data

1	(0.394) 0.389 0.400

2	(0.606) 0.611 0.600

Locus 13436 :

2 alleles

8.2% missing data

3	(0.986) 0.978 0.995

1	(0.014) 0.022 0.005

Locus 13437 :

2 alleles

99.2% missing data

2	(0.678) 0.678 0.683

1	(0.322) 0.322 0.317

Locus 13438 :

2 alleles

99.2% missing data

2	(0.483) 0.479 0.488

4	(0.517) 0.521 0.512

Locus 13439 :

2 alleles

99.2% missing data

1	(0.756) 0.749 0.760

3	(0.244) 0.251 0.240

Locus 13440 :

2 alleles

99.2% missing data

4	(0.443) 0.437 0.442

2	(0.557) 0.563 0.558

Locus 13441 :

2 alleles

99.2% missing data

2	(0.672) 0.668 0.674

3	(0.328) 0.332 0.326

Locus 13442 :

2 alleles

99.2% missing data

1	(0.843) 0.838 0.847

4	(0.157) 0.162 0.153

Locus 13443 :

2 alleles

98.8% missing data

2	(0.531) 0.525 0.537

4	(0.469) 0.475 0.463

Locus 13444 :

2 alleles

99.2% missing data

1	(0.309) 0.303 0.317

4	(0.691) 0.697 0.683

Locus 13445 :

2 alleles

99.2% missing data

3	(0.413) 0.422 0.411

4	(0.587) 0.578 0.589

Locus 13446 :

2 alleles

99.2% missing data

3	(0.565) 0.561 0.570

2	(0.435) 0.439 0.430

Locus 13447 :

2 alleles

99.2% missing data

2	(0.630) 0.622 0.634

3	(0.370) 0.378 0.366

Locus 13448 :

2 alleles

99.2% missing data

2	(0.508) 0.504 0.510

1	(0.492) 0.496 0.490

Locus 13449 :

2 alleles

97.7% missing data

4	(0.757) 0.757 0.759

2	(0.243) 0.243 0.241

Locus 13450 :

2 alleles

99.2% missing data

2	(0.428) 0.425 0.431

4	(0.572) 0.575 0.569

Locus 13451 :

2 alleles

98.8% missing data

1	(0.327) 0.318 0.329

4	(0.673) 0.682 0.671

Locus 13452 :

2 alleles

98.4% missing data

1	(0.633) 0.640 0.621

3	(0.367) 0.360 0.379

Locus 13453 :

2 alleles

98.0% missing data

4	(0.616) 0.615 0.630

3	(0.384) 0.385 0.370

Locus 13454 :

2 alleles

90.6% missing data

1	(0.931) 0.946 0.921

4	(0.069) 0.054 0.079

Locus 13455 :

2 alleles

0.4% missing data

1	(0.975) 0.975 0.982

4	(0.025) 0.025 0.018

Locus 13456 :

2 alleles

0.0% missing data

4	(0.987) 0.986 0.995

1	(0.013) 0.014 0.005

Locus 13457 :

2 alleles

99.2% missing data

4	(0.684) 0.673 0.688

3	(0.316) 0.327 0.312

Locus 13458 :

2 alleles

0.8% missing data

3	(0.981) 0.979 0.991

2	(0.019) 0.021 0.009

Locus 13459 :

2 alleles

99.2% missing data

1	(0.772) 0.773 0.766

3	(0.228) 0.227 0.234

Locus 13460 :

2 alleles

99.2% missing data

4	(0.640) 0.638 0.643

1	(0.360) 0.362 0.357

Locus 13461 :

2 alleles

0.0% missing data

4	(0.977) 0.961 0.993

3	(0.023) 0.039 0.007

Locus 13462 :

3 alleles

98.8% missing data

2	(0.309) 0.307 0.312

4	(0.438) 0.430 0.440

3	(0.254) 0.263 0.248

Locus 13463 :

2 alleles

99.2% missing data

2	(0.697) 0.699 0.690

3	(0.303) 0.301 0.310

Locus 13464 :

2 alleles

1.2% missing data

3	(0.987) 0.990 0.993

2	(0.013) 0.010 0.007

Locus 13465 :

2 alleles

98.8% missing data

2	(0.713) 0.707 0.723

3	(0.287) 0.293 0.277

Locus 13466 :

2 alleles

0.4% missing data

2	(0.988) 0.985 0.996

3	(0.012) 0.015 0.004

Locus 13467 :

2 alleles

99.2% missing data

1	(0.289) 0.290 0.294

3	(0.711) 0.710 0.706

Locus 13468 :

2 alleles

99.2% missing data

2	(0.439) 0.436 0.439

1	(0.561) 0.564 0.561

Locus 13469 :

2 alleles

99.2% missing data

3	(0.521) 0.510 0.528

1	(0.479) 0.490 0.472

Locus 13470 :

2 alleles

99.2% missing data

4	(0.483) 0.477 0.483

2	(0.517) 0.523 0.517

Locus 13471 :

2 alleles

99.2% missing data

2	(0.379) 0.373 0.384

1	(0.621) 0.627 0.616

Locus 13472 :

2 alleles

99.2% missing data

3	(0.379) 0.376 0.386

4	(0.621) 0.624 0.614

Locus 13473 :

2 alleles

99.2% missing data

2	(0.595) 0.597 0.596

4	(0.405) 0.403 0.404

Locus 13474 :

2 alleles

99.2% missing data

4	(0.683) 0.687 0.678

3	(0.317) 0.313 0.322

Locus 13475 :

2 alleles

99.2% missing data

1	(0.577) 0.575 0.583

3	(0.423) 0.425 0.417

Locus 13476 :

2 alleles

99.2% missing data

2	(0.724) 0.724 0.720

4	(0.276) 0.276 0.280

Locus 13477 :

2 alleles

93.8% missing data

2	(0.841) 0.841 0.851

4	(0.159) 0.159 0.149

Locus 13478 :

2 alleles

91.0% missing data

1	(0.902) 0.918 0.889

3	(0.098) 0.082 0.111

Locus 13479 :

2 alleles

99.2% missing data

3	(0.691) 0.698 0.681

2	(0.309) 0.302 0.319

Locus 13480 :

2 alleles

91.0% missing data

1	(0.933) 0.944 0.926

3	(0.067) 0.056 0.074

Locus 13481 :

3 alleles

98.8% missing data

2	(0.526) 0.525 0.525

1	(0.245) 0.245 0.246

3	(0.229) 0.231 0.229

Locus 13482 :

2 alleles

96.9% missing data

1	(0.802) 0.807 0.801

2	(0.198) 0.193 0.199

Locus 13483 :

2 alleles

99.2% missing data

3	(0.281) 0.278 0.288

1	(0.719) 0.722 0.712

Locus 13484 :

2 alleles

99.2% missing data

1	(0.340) 0.341 0.337

3	(0.660) 0.659 0.663

Locus 13485 :

2 alleles

98.8% missing data

2	(0.620) 0.620 0.614

3	(0.380) 0.380 0.386

Locus 13486 :

2 alleles

99.2% missing data

3	(0.412) 0.418 0.408

2	(0.588) 0.582 0.592

Locus 13487 :

2 alleles

98.0% missing data

4	(0.766) 0.776 0.762

3	(0.234) 0.224 0.238

Locus 13488 :

2 alleles

99.2% missing data

4	(0.385) 0.391 0.386

3	(0.615) 0.609 0.614

Locus 13489 :

2 alleles

99.2% missing data

2	(0.191) 0.195 0.191

4	(0.809) 0.805 0.809

Locus 13490 :

2 alleles

98.0% missing data

1	(0.876) 0.880 0.872

2	(0.124) 0.120 0.128

Locus 13491 :

2 alleles

99.2% missing data

3	(0.805) 0.797 0.808

1	(0.195) 0.203 0.192

Locus 13492 :

2 alleles

94.1% missing data

3	(0.348) 0.321 0.376

4	(0.652) 0.679 0.624

Locus 13493 :

2 alleles

99.2% missing data

2	(0.442) 0.433 0.447

3	(0.558) 0.567 0.553

Locus 13494 :

2 alleles

97.7% missing data

3	(0.556) 0.570 0.536

4	(0.444) 0.430 0.464

Locus 13495 :

2 alleles

99.2% missing data

4	(0.155) 0.153 0.159

2	(0.845) 0.847 0.841

Locus 13496 :

2 alleles

98.8% missing data

2	(0.440) 0.434 0.448

1	(0.560) 0.566 0.552

Locus 13497 :

2 alleles

99.2% missing data

1	(0.694) 0.689 0.697

2	(0.306) 0.311 0.303

Locus 13498 :

2 alleles

99.2% missing data

4	(0.256) 0.251 0.262

3	(0.744) 0.749 0.738

Locus 13499 :

2 alleles

94.5% missing data

2	(0.860) 0.853 0.872

3	(0.140) 0.147 0.128

Locus 13500 :

2 alleles

96.9% missing data

2	(0.733) 0.747 0.729

4	(0.267) 0.253 0.271

Locus 13501 :

2 alleles

99.2% missing data

3	(0.523) 0.528 0.525

2	(0.477) 0.472 0.475

Locus 13502 :

2 alleles

97.7% missing data

2	(0.756) 0.757 0.759

3	(0.244) 0.243 0.241

Locus 13503 :

2 alleles

99.2% missing data

1	(0.245) 0.242 0.248

3	(0.755) 0.758 0.752

Locus 13504 :

2 alleles

99.2% missing data

3	(0.433) 0.433 0.424

4	(0.567) 0.567 0.576

Locus 13505 :

2 alleles

99.2% missing data

3	(0.255) 0.251 0.259

1	(0.745) 0.749 0.741

Locus 13506 :

2 alleles

99.2% missing data

2	(0.804) 0.797 0.810

1	(0.196) 0.203 0.190

Locus 13507 :

2 alleles

98.8% missing data

2	(0.514) 0.519 0.508

1	(0.486) 0.481 0.492

Locus 13508 :

2 alleles

99.2% missing data

3	(0.572) 0.575 0.568

1	(0.428) 0.425 0.432

Locus 13509 :

2 alleles

99.2% missing data

3	(0.310) 0.301 0.320

1	(0.690) 0.699 0.680

Locus 13510 :

2 alleles

99.2% missing data

3	(0.435) 0.425 0.439

4	(0.565) 0.575 0.561

Locus 13511 :

2 alleles

99.2% missing data

2	(0.696) 0.692 0.693

4	(0.304) 0.308 0.307

Locus 13512 :

2 alleles

99.2% missing data

1	(0.571) 0.575 0.570

3	(0.429) 0.425 0.430

Locus 13513 :

2 alleles

99.2% missing data

2	(0.426) 0.419 0.436

4	(0.574) 0.581 0.564

Locus 13514 :

2 alleles

97.7% missing data

3	(0.712) 0.720 0.714

4	(0.288) 0.280 0.286

Locus 13515 :

2 alleles

99.2% missing data

1	(0.523) 0.523 0.523

3	(0.477) 0.477 0.477

Locus 13516 :

2 alleles

99.2% missing data

3	(0.444) 0.444 0.448

4	(0.556) 0.556 0.552

Locus 13517 :

2 alleles

99.2% missing data

2	(0.289) 0.290 0.288

1	(0.711) 0.710 0.712

Locus 13518 :

2 alleles

0.4% missing data

2	(0.989) 0.991 0.993

3	(0.011) 0.009 0.007

Locus 13519 :

2 alleles

99.2% missing data

2	(0.429) 0.425 0.435

3	(0.571) 0.575 0.565

Locus 13520 :

2 alleles

7.0% missing data

2	(0.970) 0.938 0.991

3	(0.030) 0.062 0.009

Locus 13521 :

2 alleles

96.1% missing data

2	(0.879) 0.881 0.884

4	(0.121) 0.119 0.116

Locus 13522 :

2 alleles

99.2% missing data

1	(0.624) 0.625 0.625

3	(0.376) 0.375 0.375

Locus 13523 :

2 alleles

95.3% missing data

1	(0.639) 0.639 0.639

3	(0.361) 0.361 0.361

Locus 13524 :

2 alleles

99.2% missing data

4	(0.615) 0.611 0.622

1	(0.385) 0.389 0.378

Locus 13525 :

2 alleles

99.2% missing data

1	(0.602) 0.602 0.599

2	(0.398) 0.398 0.401

Locus 13526 :

2 alleles

3.5% missing data

4	(0.982) 0.986 0.991

3	(0.018) 0.014 0.009

Locus 13527 :

2 alleles

98.8% missing data

1	(0.457) 0.466 0.457

2	(0.543) 0.534 0.543

Locus 13528 :

2 alleles

99.2% missing data

4	(0.554) 0.548 0.557

3	(0.446) 0.452 0.443

Locus 13529 :

2 alleles

99.2% missing data

3	(0.281) 0.276 0.281

2	(0.719) 0.724 0.719

Locus 13530 :

2 alleles

99.2% missing data

4	(0.523) 0.527 0.524

1	(0.477) 0.473 0.476

Locus 13531 :

2 alleles

97.7% missing data

4	(0.345) 0.347 0.332

2	(0.655) 0.653 0.668

Locus 13532 :

2 alleles

99.2% missing data

2	(0.624) 0.631 0.623

3	(0.376) 0.369 0.377

Locus 13533 :

2 alleles

97.7% missing data

2	(0.639) 0.632 0.651

4	(0.361) 0.368 0.349

Locus 13534 :

2 alleles

99.2% missing data

2	(0.368) 0.369 0.370

4	(0.632) 0.631 0.630

Locus 13535 :

2 alleles

99.2% missing data

3	(0.126) 0.122 0.129

2	(0.874) 0.878 0.871

Locus 13536 :

3 alleles

98.0% missing data

1	(0.198) 0.200 0.200

4	(0.467) 0.468 0.469

2	(0.335) 0.332 0.330

Locus 13537 :

2 alleles

99.2% missing data

1	(0.458) 0.463 0.456

2	(0.542) 0.537 0.544

Locus 13538 :

2 alleles

99.2% missing data

3	(0.471) 0.466 0.468

4	(0.529) 0.534 0.532

Locus 13539 :

2 alleles

98.0% missing data

4	(0.779) 0.770 0.785

1	(0.221) 0.230 0.215

Locus 13540 :

2 alleles

99.2% missing data

2	(0.451) 0.448 0.456

1	(0.549) 0.552 0.544

Locus 13541 :

2 alleles

99.2% missing data

2	(0.361) 0.361 0.364

4	(0.639) 0.639 0.636

Locus 13542 :

2 alleles

99.2% missing data

3	(0.750) 0.747 0.756

1	(0.250) 0.253 0.244

Locus 13543 :

2 alleles

99.2% missing data

3	(0.498) 0.495 0.501

4	(0.502) 0.505 0.499

Locus 13544 :

2 alleles

8.6% missing data

4	(0.984) 0.977 0.995

3	(0.016) 0.023 0.005

Locus 13545 :

2 alleles

0.4% missing data

4	(0.984) 0.981 0.995

1	(0.016) 0.019 0.005

Locus 13546 :

2 alleles

99.2% missing data

3	(0.414) 0.413 0.422

2	(0.586) 0.587 0.578

Locus 13547 :

2 alleles

98.8% missing data

3	(0.403) 0.402 0.402

2	(0.597) 0.598 0.598

Locus 13548 :

2 alleles

96.9% missing data

1	(0.108) 0.099 0.115

2	(0.892) 0.901 0.885

Locus 13549 :

2 alleles

99.2% missing data

3	(0.708) 0.700 0.714

2	(0.292) 0.300 0.286

Locus 13550 :

2 alleles

99.2% missing data

3	(0.771) 0.774 0.766

4	(0.229) 0.226 0.234

Locus 13551 :

2 alleles

99.2% missing data

1	(0.270) 0.270 0.273

2	(0.730) 0.730 0.727

Locus 13552 :

2 alleles

99.2% missing data

3	(0.476) 0.475 0.479

1	(0.524) 0.525 0.521

Locus 13553 :

2 alleles

99.2% missing data

1	(0.648) 0.646 0.656

3	(0.352) 0.354 0.344

Locus 13554 :

2 alleles

99.2% missing data

4	(0.640) 0.648 0.642

3	(0.360) 0.352 0.358

Locus 13555 :

2 alleles

99.2% missing data

1	(0.487) 0.491 0.486

3	(0.513) 0.509 0.514

Locus 13556 :

2 alleles

99.2% missing data

1	(0.257) 0.253 0.259

3	(0.743) 0.747 0.741

Locus 13557 :

2 alleles

99.2% missing data

2	(0.522) 0.530 0.519

3	(0.478) 0.470 0.481

Locus 13558 :

2 alleles

99.2% missing data

3	(0.492) 0.494 0.488

4	(0.508) 0.506 0.512

Locus 13559 :

2 alleles

8.2% missing data

2	(0.987) 0.980 0.996

1	(0.013) 0.020 0.004

Locus 13560 :

2 alleles

93.8% missing data

3	(0.896) 0.889 0.908

4	(0.104) 0.111 0.092

Locus 13561 :

2 alleles

99.2% missing data

3	(0.578) 0.578 0.578

4	(0.422) 0.422 0.422

Locus 13562 :

2 alleles

99.2% missing data

4	(0.528) 0.523 0.536

3	(0.472) 0.477 0.464

Locus 13563 :

2 alleles

98.4% missing data

3	(0.584) 0.594 0.578

1	(0.416) 0.406 0.422

Locus 13564 :

2 alleles

92.6% missing data

1	(0.910) 0.914 0.915

3	(0.090) 0.086 0.085

Locus 13565 :

2 alleles

99.2% missing data

1	(0.548) 0.548 0.545

3	(0.452) 0.452 0.455

Locus 13566 :

2 alleles

99.2% missing data

1	(0.641) 0.640 0.636

4	(0.359) 0.360 0.364

Locus 13567 :

2 alleles

99.2% missing data

4	(0.286) 0.286 0.292

1	(0.714) 0.714 0.708

Locus 13568 :

2 alleles

96.5% missing data

3	(0.335) 0.316 0.333

1	(0.665) 0.684 0.667

Locus 13569 :

2 alleles

99.2% missing data

2	(0.328) 0.331 0.327

3	(0.672) 0.669 0.673

Locus 13570 :

2 alleles

98.8% missing data

1	(0.501) 0.496 0.513

3	(0.499) 0.504 0.487

Locus 13571 :

2 alleles

99.2% missing data

3	(0.262) 0.260 0.261

1	(0.738) 0.740 0.739

Locus 13572 :

2 alleles

99.2% missing data

1	(0.791) 0.797 0.790

4	(0.209) 0.203 0.210

Locus 13573 :

2 alleles

99.2% missing data

4	(0.670) 0.671 0.663

2	(0.330) 0.329 0.337

Locus 13574 :

2 alleles

99.2% missing data

3	(0.408) 0.407 0.414

4	(0.592) 0.593 0.586

Locus 13575 :

2 alleles

99.2% missing data

1	(0.651) 0.649 0.654

2	(0.349) 0.351 0.346

Locus 13576 :

2 alleles

99.2% missing data

1	(0.673) 0.664 0.681

4	(0.327) 0.336 0.319

Locus 13577 :

2 alleles

97.7% missing data

3	(0.753) 0.761 0.758

1	(0.247) 0.239 0.242

Locus 13578 :

2 alleles

98.8% missing data

1	(0.380) 0.377 0.384

2	(0.620) 0.623 0.616

Locus 13579 :

2 alleles

99.2% missing data

2	(0.336) 0.338 0.338

4	(0.664) 0.662 0.662

Locus 13580 :

3 alleles

98.8% missing data

1	(0.462) 0.463 0.457

3	(0.301) 0.294 0.313

2	(0.237) 0.243 0.229

Locus 13581 :

2 alleles

99.2% missing data

3	(0.387) 0.382 0.391

1	(0.613) 0.618 0.609

Locus 13582 :

2 alleles

98.4% missing data

4	(0.617) 0.621 0.620

1	(0.383) 0.379 0.380

Locus 13583 :

3 alleles

98.4% missing data

3	(0.316) 0.310 0.318

2	(0.313) 0.309 0.314

1	(0.371) 0.380 0.368

Locus 13584 :

2 alleles

99.2% missing data

1	(0.334) 0.332 0.335

4	(0.666) 0.668 0.665

Locus 13585 :

2 alleles

99.2% missing data

4	(0.556) 0.551 0.560

1	(0.444) 0.449 0.440

Locus 13586 :

2 alleles

99.2% missing data

2	(0.391) 0.385 0.393

4	(0.609) 0.615 0.607

Locus 13587 :

2 alleles

98.8% missing data

3	(0.651) 0.658 0.652

1	(0.349) 0.342 0.348

Locus 13588 :

2 alleles

99.2% missing data

2	(0.548) 0.546 0.553

1	(0.452) 0.454 0.447

Locus 13589 :

2 alleles

98.4% missing data

1	(0.656) 0.661 0.654

4	(0.344) 0.339 0.346

Locus 13590 :

2 alleles

99.2% missing data

3	(0.349) 0.352 0.348

1	(0.651) 0.648 0.652

Locus 13591 :

2 alleles

99.2% missing data

4	(0.382) 0.382 0.380

2	(0.618) 0.618 0.620

Locus 13592 :

2 alleles

99.2% missing data

4	(0.375) 0.376 0.375

2	(0.625) 0.624 0.625

Locus 13593 :

2 alleles

99.2% missing data

4	(0.208) 0.205 0.213

1	(0.792) 0.795 0.787

Locus 13594 :

2 alleles

99.2% missing data

4	(0.420) 0.424 0.419

2	(0.580) 0.576 0.581

Locus 13595 :

2 alleles

99.2% missing data

2	(0.322) 0.321 0.322

1	(0.678) 0.679 0.678

Locus 13596 :

2 alleles

99.2% missing data

4	(0.383) 0.383 0.377

3	(0.617) 0.617 0.623

Locus 13597 :

2 alleles

94.5% missing data

4	(0.833) 0.846 0.832

3	(0.167) 0.154 0.168

Locus 13598 :

2 alleles

99.2% missing data

2	(0.465) 0.467 0.463

1	(0.535) 0.533 0.537

Locus 13599 :

3 alleles

98.8% missing data

3	(0.206) 0.212 0.207

1	(0.231) 0.223 0.241

4	(0.563) 0.566 0.553

Locus 13600 :

2 alleles

94.5% missing data

1	(0.180) 0.180 0.165

3	(0.820) 0.820 0.835

Locus 13601 :

2 alleles

99.2% missing data

2	(0.382) 0.385 0.384

4	(0.618) 0.615 0.616

Locus 13602 :

2 alleles

98.8% missing data

3	(0.466) 0.467 0.467

2	(0.534) 0.533 0.533

Locus 13603 :

2 alleles

99.2% missing data

3	(0.331) 0.330 0.333

4	(0.669) 0.670 0.667

Locus 13604 :

2 alleles

99.2% missing data

4	(0.593) 0.590 0.602

3	(0.407) 0.410 0.398

Locus 13605 :

2 alleles

98.4% missing data

1	(0.539) 0.533 0.547

2	(0.461) 0.467 0.453

Locus 13606 :

2 alleles

99.2% missing data

3	(0.558) 0.557 0.550

4	(0.442) 0.443 0.450

Locus 13607 :

2 alleles

98.8% missing data

3	(0.633) 0.638 0.630

4	(0.367) 0.362 0.370

Locus 13608 :

2 alleles

98.0% missing data

1	(0.600) 0.603 0.598

2	(0.400) 0.397 0.402

Locus 13609 :

2 alleles

99.2% missing data

1	(0.288) 0.285 0.296

3	(0.712) 0.715 0.704

Locus 13610 :

2 alleles

99.2% missing data

3	(0.552) 0.553 0.557

4	(0.448) 0.447 0.443

Locus 13611 :

3 alleles

98.8% missing data

2	(0.509) 0.501 0.518

3	(0.228) 0.234 0.222

4	(0.262) 0.264 0.259

Locus 13612 :

2 alleles

99.2% missing data

3	(0.512) 0.508 0.507

1	(0.488) 0.492 0.493

Locus 13613 :

2 alleles

99.2% missing data

4	(0.602) 0.598 0.601

3	(0.398) 0.402 0.399

Locus 13614 :

2 alleles

93.8% missing data

1	(0.909) 0.906 0.915

3	(0.091) 0.094 0.085

Locus 13615 :

2 alleles

94.9% missing data

3	(0.896) 0.899 0.897

4	(0.104) 0.101 0.103

Locus 13616 :

2 alleles

96.1% missing data

1	(0.819) 0.832 0.812

3	(0.181) 0.168 0.188

Locus 13617 :

2 alleles

93.0% missing data

2	(0.854) 0.850 0.866

4	(0.146) 0.150 0.134

Locus 13618 :

2 alleles

98.8% missing data

4	(0.511) 0.510 0.517

1	(0.489) 0.490 0.483

Locus 13619 :

2 alleles

99.2% missing data

4	(0.383) 0.377 0.381

1	(0.617) 0.623 0.619

Locus 13620 :

2 alleles

96.9% missing data

1	(0.227) 0.228 0.227

4	(0.773) 0.772 0.773

Locus 13621 :

2 alleles

99.2% missing data

1	(0.248) 0.245 0.254

3	(0.752) 0.755 0.746

Locus 13622 :

2 alleles

98.4% missing data

3	(0.654) 0.658 0.656

2	(0.346) 0.342 0.344

Locus 13623 :

2 alleles

99.2% missing data

4	(0.573) 0.576 0.567

3	(0.427) 0.424 0.433

Locus 13624 :

2 alleles

8.2% missing data

3	(0.989) 0.981 0.997

4	(0.011) 0.019 0.003

Locus 13625 :

2 alleles

99.2% missing data

2	(0.856) 0.853 0.865

3	(0.144) 0.147 0.135

Locus 13626 :

2 alleles

99.2% missing data

4	(0.363) 0.358 0.371

1	(0.637) 0.642 0.629

Locus 13627 :

2 alleles

8.2% missing data

2	(0.984) 0.976 0.995

4	(0.016) 0.024 0.005

Locus 13628 :

2 alleles

99.2% missing data

2	(0.705) 0.705 0.705

3	(0.295) 0.295 0.295

Locus 13629 :

2 alleles

99.2% missing data

3	(0.598) 0.590 0.605

1	(0.402) 0.410 0.395

Locus 13630 :

2 alleles

99.2% missing data

1	(0.490) 0.491 0.490

3	(0.510) 0.509 0.510

Locus 13631 :

2 alleles

99.2% missing data

4	(0.319) 0.316 0.324

2	(0.681) 0.684 0.676

Locus 13632 :

2 alleles

99.2% missing data

4	(0.549) 0.548 0.552

1	(0.451) 0.452 0.448

Locus 13633 :

2 alleles

99.2% missing data

1	(0.414) 0.408 0.424

4	(0.586) 0.592 0.576

Locus 13634 :

2 alleles

98.8% missing data

1	(0.494) 0.500 0.490

4	(0.506) 0.500 0.510

Locus 13635 :

2 alleles

99.2% missing data

3	(0.801) 0.804 0.797

1	(0.199) 0.196 0.203

Locus 13636 :

2 alleles

99.2% missing data

1	(0.471) 0.471 0.477

4	(0.529) 0.529 0.523

Locus 13637 :

2 alleles

99.2% missing data

1	(0.323) 0.319 0.331

2	(0.677) 0.681 0.669

Locus 13638 :

2 alleles

99.2% missing data

1	(0.414) 0.415 0.417

2	(0.586) 0.585 0.583

Locus 13639 :

2 alleles

98.8% missing data

4	(0.628) 0.625 0.639

2	(0.372) 0.375 0.361

Locus 13640 :

2 alleles

98.0% missing data

1	(0.849) 0.858 0.846

3	(0.151) 0.142 0.154

Locus 13641 :

2 alleles

99.2% missing data

1	(0.284) 0.289 0.282

3	(0.716) 0.711 0.718

Locus 13642 :

2 alleles

99.2% missing data

2	(0.691) 0.692 0.691

3	(0.309) 0.308 0.309

Locus 13643 :

2 alleles

99.2% missing data

1	(0.280) 0.285 0.276

3	(0.720) 0.715 0.724

Locus 13644 :

2 alleles

99.2% missing data

3	(0.265) 0.264 0.270

1	(0.735) 0.736 0.730

Locus 13645 :

2 alleles

98.4% missing data

1	(0.410) 0.423 0.404

3	(0.590) 0.577 0.596

Locus 13646 :

2 alleles

99.2% missing data

1	(0.323) 0.325 0.328

3	(0.677) 0.675 0.672

Locus 13647 :

2 alleles

99.2% missing data

1	(0.458) 0.464 0.457

3	(0.542) 0.536 0.543

Locus 13648 :

2 alleles

99.2% missing data

4	(0.676) 0.686 0.672

1	(0.324) 0.314 0.328

Locus 13649 :

2 alleles

99.2% missing data

1	(0.563) 0.562 0.569

3	(0.437) 0.438 0.431

Locus 13650 :

2 alleles

99.2% missing data

4	(0.637) 0.639 0.631

1	(0.363) 0.361 0.369

Locus 13651 :

2 alleles

99.2% missing data

3	(0.292) 0.289 0.295

1	(0.708) 0.711 0.705

Locus 13652 :

2 alleles

99.2% missing data

1	(0.438) 0.432 0.444

4	(0.562) 0.568 0.556

Locus 13653 :

2 alleles

8.2% missing data

1	(0.984) 0.977 0.995

2	(0.016) 0.023 0.005

Locus 13654 :

2 alleles

97.7% missing data

1	(0.118) 0.114 0.126

4	(0.882) 0.886 0.874

Locus 13655 :

2 alleles

99.2% missing data

1	(0.577) 0.572 0.579

3	(0.423) 0.428 0.421

Locus 13656 :

2 alleles

98.8% missing data

4	(0.560) 0.563 0.560

2	(0.440) 0.437 0.440

Locus 13657 :

3 alleles

98.4% missing data

1	(0.280) 0.277 0.282

2	(0.451) 0.453 0.448

4	(0.269) 0.270 0.270

Locus 13658 :

2 alleles

99.2% missing data

4	(0.365) 0.358 0.369

3	(0.635) 0.642 0.631

Locus 13659 :

2 alleles

99.2% missing data

1	(0.114) 0.109 0.119

3	(0.886) 0.891 0.881

Locus 13660 :

2 alleles

97.7% missing data

1	(0.865) 0.874 0.858

2	(0.135) 0.126 0.142

Locus 13661 :

2 alleles

99.2% missing data

2	(0.276) 0.272 0.281

3	(0.724) 0.728 0.719

Locus 13662 :

3 alleles

98.8% missing data

3	(0.311) 0.313 0.309

1	(0.351) 0.349 0.351

2	(0.338) 0.338 0.339

Locus 13663 :

2 alleles

99.2% missing data

3	(0.696) 0.701 0.685

1	(0.304) 0.299 0.315

Locus 13664 :

2 alleles

98.8% missing data

2	(0.421) 0.423 0.419

1	(0.579) 0.577 0.581

Locus 13665 :

2 alleles

99.2% missing data

2	(0.227) 0.226 0.230

4	(0.773) 0.774 0.770

Locus 13666 :

3 alleles

97.7% missing data

1	(0.431) 0.450 0.422

4	(0.164) 0.167 0.161

2	(0.405) 0.382 0.417

Locus 13667 :

2 alleles

99.2% missing data

1	(0.597) 0.595 0.598

3	(0.403) 0.405 0.402

Locus 13668 :

2 alleles

98.8% missing data

3	(0.387) 0.390 0.384

1	(0.613) 0.610 0.616

Locus 13669 :

3 alleles

90.2% missing data

1	(0.793) 0.831 0.774

4	(0.100) 0.081 0.110

2	(0.107) 0.088 0.116

Locus 13670 :

2 alleles

99.2% missing data

2	(0.753) 0.746 0.756

1	(0.247) 0.254 0.244

Locus 13671 :

2 alleles

99.2% missing data

4	(0.179) 0.187 0.173

2	(0.821) 0.813 0.827

Locus 13672 :

2 alleles

99.2% missing data

4	(0.763) 0.763 0.759

3	(0.237) 0.237 0.241

Locus 13673 :

2 alleles

98.8% missing data

4	(0.650) 0.656 0.652

1	(0.350) 0.344 0.348

Locus 13674 :

2 alleles

99.2% missing data

4	(0.547) 0.550 0.543

1	(0.453) 0.450 0.457

Locus 13675 :

2 alleles

97.3% missing data

3	(0.833) 0.825 0.845

4	(0.167) 0.175 0.155

Locus 13676 :

2 alleles

99.2% missing data

1	(0.243) 0.251 0.240

2	(0.757) 0.749 0.760

Locus 13677 :

2 alleles

99.2% missing data

1	(0.491) 0.491 0.493

3	(0.509) 0.509 0.507

Locus 13678 :

2 alleles

98.8% missing data

4	(0.472) 0.470 0.473

3	(0.528) 0.530 0.527

Locus 13679 :

2 alleles

96.5% missing data

1	(0.650) 0.673 0.640

4	(0.350) 0.327 0.360

Locus 13680 :

2 alleles

99.2% missing data

3	(0.604) 0.598 0.612

4	(0.396) 0.402 0.388

Locus 13681 :

2 alleles

98.8% missing data

1	(0.504) 0.501 0.503

4	(0.496) 0.499 0.497

Locus 13682 :

2 alleles

98.4% missing data

1	(0.553) 0.548 0.557

3	(0.447) 0.452 0.443

Locus 13683 :

2 alleles

96.1% missing data

1	(0.181) 0.172 0.181

3	(0.819) 0.828 0.819

Locus 13684 :

2 alleles

99.2% missing data

2	(0.327) 0.326 0.325

4	(0.673) 0.674 0.675

Locus 13685 :

2 alleles

93.0% missing data

3	(0.852) 0.839 0.876

4	(0.148) 0.161 0.124

Locus 13686 :

2 alleles

99.2% missing data

1	(0.778) 0.781 0.771

4	(0.222) 0.219 0.229

Locus 13687 :

2 alleles

99.2% missing data

3	(0.491) 0.490 0.496

1	(0.509) 0.510 0.504

Locus 13688 :

2 alleles

0.4% missing data

1	(0.987) 0.984 0.996

4	(0.013) 0.016 0.004

Locus 13689 :

2 alleles

99.2% missing data

4	(0.392) 0.392 0.389

2	(0.608) 0.608 0.611

Locus 13690 :

2 alleles

99.2% missing data

2	(0.205) 0.199 0.211

3	(0.795) 0.801 0.789

Locus 13691 :

2 alleles

96.5% missing data

4	(0.274) 0.259 0.283

3	(0.726) 0.741 0.717

Locus 13692 :

2 alleles

98.8% missing data

1	(0.214) 0.220 0.213

4	(0.786) 0.780 0.787

Locus 13693 :

2 alleles

98.8% missing data

1	(0.305) 0.308 0.302

4	(0.695) 0.692 0.698

Locus 13694 :

2 alleles

98.0% missing data

3	(0.757) 0.756 0.759

1	(0.243) 0.244 0.241

Locus 13695 :

2 alleles

98.4% missing data

1	(0.270) 0.262 0.277

3	(0.730) 0.738 0.723

Locus 13696 :

2 alleles

99.2% missing data

1	(0.304) 0.300 0.312

2	(0.696) 0.700 0.688

Locus 13697 :

2 alleles

99.2% missing data

3	(0.538) 0.533 0.539

1	(0.462) 0.467 0.461

Locus 13698 :

2 alleles

99.2% missing data

4	(0.350) 0.347 0.353

2	(0.650) 0.653 0.647

Locus 13699 :

3 alleles

98.4% missing data

2	(0.303) 0.300 0.307

1	(0.412) 0.411 0.420

3	(0.285) 0.289 0.273

Locus 13700 :

2 alleles

97.3% missing data

3	(0.593) 0.599 0.601

4	(0.407) 0.401 0.399

Locus 13701 :

2 alleles

7.8% missing data

3	(0.978) 0.961 0.993

4	(0.022) 0.039 0.007

Locus 13702 :

2 alleles

97.7% missing data

1	(0.246) 0.244 0.254

3	(0.754) 0.756 0.746

Locus 13703 :

2 alleles

99.2% missing data

4	(0.461) 0.460 0.461

2	(0.539) 0.540 0.539

Locus 13704 :

2 alleles

99.2% missing data

4	(0.715) 0.706 0.715

1	(0.285) 0.294 0.285

Locus 13705 :

2 alleles

96.1% missing data

2	(0.190) 0.176 0.194

3	(0.810) 0.824 0.806

Locus 13706 :

2 alleles

99.2% missing data

2	(0.691) 0.687 0.693

3	(0.309) 0.313 0.307

Locus 13707 :

2 alleles

99.2% missing data

1	(0.517) 0.519 0.512

2	(0.483) 0.481 0.488

Locus 13708 :

2 alleles

99.2% missing data

3	(0.327) 0.325 0.330

2	(0.673) 0.675 0.670

Locus 13709 :

2 alleles

93.0% missing data

3	(0.896) 0.897 0.903

4	(0.104) 0.103 0.097

Locus 13710 :

3 alleles

96.9% missing data

2	(0.254) 0.241 0.265

1	(0.503) 0.525 0.495

4	(0.243) 0.234 0.240

Locus 13711 :

2 alleles

98.8% missing data

2	(0.468) 0.465 0.472

3	(0.532) 0.535 0.528

Locus 13712 :

2 alleles

99.2% missing data

1	(0.507) 0.507 0.503

2	(0.493) 0.493 0.497

Locus 13713 :

2 alleles

99.2% missing data

1	(0.536) 0.538 0.526

4	(0.464) 0.462 0.474

Locus 13714 :

2 alleles

99.2% missing data

2	(0.462) 0.464 0.462

4	(0.538) 0.536 0.538

Locus 13715 :

2 alleles

99.2% missing data

1	(0.475) 0.478 0.471

4	(0.525) 0.522 0.529

Locus 13716 :

2 alleles

95.7% missing data

1	(0.882) 0.879 0.889

2	(0.118) 0.121 0.111

Locus 13717 :

2 alleles

99.2% missing data

4	(0.683) 0.681 0.679

2	(0.317) 0.319 0.321

Locus 13718 :

2 alleles

95.3% missing data

1	(0.742) 0.736 0.765

3	(0.258) 0.264 0.235

Locus 13719 :

2 alleles

99.2% missing data

2	(0.471) 0.477 0.470

1	(0.529) 0.523 0.530

Locus 13720 :

2 alleles

99.2% missing data

2	(0.592) 0.600 0.588

1	(0.408) 0.400 0.412

Locus 13721 :

2 alleles

9.4% missing data

2	(0.984) 0.988 0.992

1	(0.016) 0.012 0.008

Locus 13722 :

2 alleles

99.2% missing data

3	(0.533) 0.527 0.535

2	(0.467) 0.473 0.465

Locus 13723 :

2 alleles

98.8% missing data

3	(0.471) 0.459 0.477

1	(0.529) 0.541 0.523

Locus 13724 :

2 alleles

99.2% missing data

3	(0.327) 0.333 0.332

2	(0.673) 0.667 0.668

Locus 13725 :

2 alleles

98.8% missing data

1	(0.821) 0.815 0.829

2	(0.179) 0.185 0.171

Locus 13726 :

2 alleles

99.2% missing data

3	(0.698) 0.702 0.699

2	(0.302) 0.298 0.301

Locus 13727 :

2 alleles

99.2% missing data

1	(0.472) 0.470 0.470

2	(0.528) 0.530 0.530

Locus 13728 :

2 alleles

99.2% missing data

2	(0.450) 0.447 0.451

3	(0.550) 0.553 0.549

Locus 13729 :

2 alleles

0.4% missing data

1	(0.948) 0.960 0.945

4	(0.052) 0.040 0.055

Locus 13730 :

3 alleles

98.8% missing data

4	(0.237) 0.240 0.240

2	(0.553) 0.553 0.549

1	(0.210) 0.206 0.210

Locus 13731 :

2 alleles

99.2% missing data

1	(0.284) 0.284 0.288

2	(0.716) 0.716 0.712

Locus 13732 :

2 alleles

95.3% missing data

2	(0.198) 0.204 0.180

4	(0.802) 0.796 0.820

Locus 13733 :

2 alleles

99.2% missing data

1	(0.720) 0.716 0.725

3	(0.280) 0.284 0.275

Locus 13734 :

2 alleles

99.2% missing data

1	(0.661) 0.661 0.661

3	(0.339) 0.339 0.339

Locus 13735 :

2 alleles

99.2% missing data

3	(0.446) 0.441 0.446

2	(0.554) 0.559 0.554

Locus 13736 :

2 alleles

99.2% missing data

3	(0.464) 0.463 0.467

1	(0.536) 0.537 0.533

Locus 13737 :

2 alleles

99.2% missing data

4	(0.358) 0.361 0.362

1	(0.642) 0.639 0.638

Locus 13738 :

3 alleles

94.5% missing data

4	(0.797) 0.797 0.802

3	(0.112) 0.111 0.107

1	(0.092) 0.092 0.091

Locus 13739 :

2 alleles

98.8% missing data

4	(0.528) 0.527 0.519

3	(0.472) 0.473 0.481

Locus 13740 :

2 alleles

99.2% missing data

4	(0.364) 0.367 0.363

1	(0.636) 0.633 0.637

Locus 13741 :

2 alleles

99.2% missing data

1	(0.406) 0.398 0.412

3	(0.594) 0.602 0.588

Locus 13742 :

2 alleles

99.2% missing data

4	(0.263) 0.258 0.273

1	(0.737) 0.742 0.727

Locus 13743 :

2 alleles

92.6% missing data

2	(0.919) 0.922 0.923

1	(0.081) 0.078 0.077

Locus 13744 :

4 alleles

98.4% missing data

2	(0.314) 0.309 0.312

3	(0.225) 0.232 0.219

1	(0.156) 0.159 0.156

4	(0.305) 0.300 0.313

Locus 13745 :

2 alleles

96.1% missing data

3	(0.182) 0.167 0.192

4	(0.818) 0.833 0.808

Locus 13746 :

2 alleles

99.2% missing data

4	(0.781) 0.781 0.774

3	(0.219) 0.219 0.226

Locus 13747 :

2 alleles

96.5% missing data

3	(0.159) 0.152 0.159

1	(0.841) 0.848 0.841

Locus 13748 :

2 alleles

99.2% missing data

3	(0.615) 0.606 0.619

2	(0.385) 0.394 0.381

Locus 13749 :

2 alleles

98.8% missing data

1	(0.615) 0.612 0.615

3	(0.385) 0.388 0.385

Locus 13750 :

2 alleles

99.2% missing data

3	(0.471) 0.471 0.465

4	(0.529) 0.529 0.535

Locus 13751 :

2 alleles

96.1% missing data

1	(0.229) 0.214 0.237

3	(0.771) 0.786 0.763

Locus 13752 :

2 alleles

99.2% missing data

2	(0.480) 0.483 0.476

1	(0.520) 0.517 0.524

Locus 13753 :

2 alleles

99.2% missing data

1	(0.482) 0.479 0.488

3	(0.518) 0.521 0.512

Locus 13754 :

2 alleles

92.6% missing data

3	(0.921) 0.914 0.934

4	(0.079) 0.086 0.066

Locus 13755 :

2 alleles

99.2% missing data

1	(0.711) 0.714 0.704

3	(0.289) 0.286 0.296

Locus 13756 :

2 alleles

92.6% missing data

4	(0.795) 0.796 0.796

3	(0.205) 0.204 0.204

Locus 13757 :

2 alleles

98.8% missing data

2	(0.476) 0.465 0.483

3	(0.524) 0.535 0.517

Locus 13758 :

2 alleles

93.0% missing data

1	(0.436) 0.469 0.402

2	(0.564) 0.531 0.598

Locus 13759 :

2 alleles

99.2% missing data

3	(0.456) 0.462 0.455

1	(0.544) 0.538 0.545

Locus 13760 :

2 alleles

99.2% missing data

4	(0.356) 0.355 0.356

3	(0.644) 0.645 0.644

Locus 13761 :

2 alleles

99.2% missing data

4	(0.393) 0.391 0.393

3	(0.607) 0.609 0.607

Locus 13762 :

2 alleles

92.6% missing data

2	(0.844) 0.835 0.868

3	(0.156) 0.165 0.132

Locus 13763 :

2 alleles

99.2% missing data

3	(0.823) 0.826 0.818

1	(0.177) 0.174 0.182

Locus 13764 :

2 alleles

99.2% missing data

1	(0.227) 0.235 0.224

2	(0.773) 0.765 0.776

Locus 13765 :

2 alleles

99.2% missing data

3	(0.232) 0.228 0.242

1	(0.768) 0.772 0.758

Locus 13766 :

2 alleles

91.0% missing data

1	(0.909) 0.927 0.901

3	(0.091) 0.073 0.099

Locus 13767 :

2 alleles

99.2% missing data

3	(0.269) 0.273 0.267

1	(0.731) 0.727 0.733

Locus 13768 :

2 alleles

99.2% missing data

4	(0.573) 0.564 0.572

2	(0.427) 0.436 0.428

Locus 13769 :

2 alleles

98.8% missing data

2	(0.477) 0.485 0.476

1	(0.523) 0.515 0.524

Locus 13770 :

2 alleles

99.2% missing data

4	(0.533) 0.532 0.539

1	(0.467) 0.468 0.461

Locus 13771 :

2 alleles

99.2% missing data

4	(0.517) 0.514 0.511

2	(0.483) 0.486 0.489

Locus 13772 :

2 alleles

99.2% missing data

1	(0.632) 0.623 0.635

4	(0.368) 0.377 0.365

Locus 13773 :

2 alleles

98.4% missing data

1	(0.613) 0.607 0.625

2	(0.387) 0.393 0.375

Locus 13774 :

3 alleles

98.8% missing data

4	(0.403) 0.412 0.406

3	(0.334) 0.332 0.328

2	(0.263) 0.256 0.266

Locus 13775 :

2 alleles

99.2% missing data

4	(0.159) 0.157 0.162

3	(0.841) 0.843 0.838

Locus 13776 :

2 alleles

99.2% missing data

2	(0.397) 0.403 0.391

1	(0.603) 0.597 0.609

Locus 13777 :

2 alleles

98.8% missing data

4	(0.400) 0.392 0.398

2	(0.600) 0.608 0.602

Locus 13778 :

2 alleles

8.2% missing data

4	(0.974) 0.942 0.992

3	(0.026) 0.058 0.008

Locus 13779 :

2 alleles

96.5% missing data

1	(0.689) 0.708 0.684

2	(0.311) 0.292 0.316

Locus 13780 :

2 alleles

99.2% missing data

1	(0.520) 0.527 0.518

4	(0.480) 0.473 0.482

Locus 13781 :

2 alleles

99.2% missing data

2	(0.437) 0.440 0.439

4	(0.563) 0.560 0.561

Locus 13782 :

2 alleles

99.2% missing data

3	(0.563) 0.564 0.556

1	(0.437) 0.436 0.444

Locus 13783 :

2 alleles

0.0% missing data

4	(0.985) 0.981 0.996

1	(0.015) 0.019 0.004

Locus 13784 :

2 alleles

99.2% missing data

3	(0.640) 0.640 0.638

2	(0.360) 0.360 0.362

Locus 13785 :

2 alleles

99.2% missing data

2	(0.504) 0.503 0.507

3	(0.496) 0.497 0.493

Locus 13786 :

2 alleles

99.2% missing data

3	(0.365) 0.366 0.363

2	(0.635) 0.634 0.637

Locus 13787 :

2 alleles

99.2% missing data

3	(0.586) 0.588 0.589

2	(0.414) 0.412 0.411

Locus 13788 :

2 alleles

98.4% missing data

3	(0.183) 0.187 0.180

1	(0.817) 0.813 0.820

Locus 13789 :

2 alleles

99.2% missing data

1	(0.511) 0.503 0.520

3	(0.489) 0.497 0.480

Locus 13790 :

3 alleles

98.4% missing data

2	(0.511) 0.508 0.512

1	(0.278) 0.273 0.280

3	(0.210) 0.219 0.208

Locus 13791 :

2 alleles

98.4% missing data

3	(0.808) 0.812 0.800

1	(0.192) 0.188 0.200

Locus 13792 :

2 alleles

99.2% missing data

2	(0.412) 0.408 0.410

3	(0.588) 0.592 0.590

Locus 13793 :

2 alleles

99.2% missing data

1	(0.751) 0.755 0.743

3	(0.249) 0.245 0.257

Locus 13794 :

2 alleles

99.2% missing data

4	(0.621) 0.623 0.618

1	(0.379) 0.377 0.382

Locus 13795 :

2 alleles

91.0% missing data

3	(0.918) 0.933 0.908

1	(0.082) 0.067 0.092

Locus 13796 :

2 alleles

99.2% missing data

1	(0.616) 0.610 0.624

4	(0.384) 0.390 0.376

Locus 13797 :

2 alleles

99.2% missing data

2	(0.358) 0.369 0.352

3	(0.642) 0.631 0.648

Locus 13798 :

2 alleles

99.2% missing data

3	(0.530) 0.527 0.532

2	(0.470) 0.473 0.468

Locus 13799 :

2 alleles

98.4% missing data

4	(0.318) 0.326 0.313

2	(0.682) 0.674 0.687

Locus 13800 :

2 alleles

99.2% missing data

1	(0.748) 0.745 0.747

4	(0.252) 0.255 0.253

Locus 13801 :

2 alleles

99.2% missing data

4	(0.598) 0.600 0.600

2	(0.402) 0.400 0.400

Locus 13802 :

2 alleles

99.2% missing data

2	(0.221) 0.227 0.220

1	(0.779) 0.773 0.780

Locus 13803 :

2 alleles

99.2% missing data

1	(0.827) 0.834 0.823

3	(0.173) 0.166 0.177

Locus 13804 :

2 alleles

96.9% missing data

3	(0.151) 0.154 0.148

1	(0.849) 0.846 0.852

Locus 13805 :

2 alleles

99.2% missing data

3	(0.418) 0.414 0.425

2	(0.582) 0.586 0.575

Locus 13806 :

2 alleles

97.3% missing data

2	(0.854) 0.862 0.845

4	(0.146) 0.138 0.155

Locus 13807 :

2 alleles

99.2% missing data

3	(0.475) 0.477 0.472

4	(0.525) 0.523 0.528

Locus 13808 :

2 alleles

99.2% missing data

4	(0.732) 0.735 0.729

2	(0.268) 0.265 0.271

Locus 13809 :

2 alleles

96.9% missing data

1	(0.723) 0.718 0.741

3	(0.277) 0.282 0.259

Locus 13810 :

2 alleles

99.2% missing data

4	(0.378) 0.380 0.381

3	(0.622) 0.620 0.619

Locus 13811 :

2 alleles

99.2% missing data

4	(0.700) 0.691 0.707

2	(0.300) 0.309 0.293

Locus 13812 :

2 alleles

99.2% missing data

4	(0.314) 0.314 0.313

3	(0.686) 0.686 0.687

Locus 13813 :

2 alleles

97.3% missing data

4	(0.835) 0.831 0.846

1	(0.165) 0.169 0.154

Locus 13814 :

2 alleles

99.2% missing data

4	(0.515) 0.515 0.517

3	(0.485) 0.485 0.483

Locus 13815 :

2 alleles

99.2% missing data

1	(0.611) 0.606 0.612

3	(0.389) 0.394 0.388

Locus 13816 :

2 alleles

99.2% missing data

3	(0.525) 0.532 0.522

4	(0.475) 0.468 0.478

Locus 13817 :

2 alleles

99.2% missing data

3	(0.457) 0.453 0.457

4	(0.543) 0.547 0.543

Locus 13818 :

2 alleles

99.2% missing data

3	(0.669) 0.669 0.663

2	(0.331) 0.331 0.337

Locus 13819 :

2 alleles

99.2% missing data

4	(0.755) 0.745 0.767

3	(0.245) 0.255 0.233

Locus 13820 :

2 alleles

98.0% missing data

1	(0.748) 0.748 0.754

3	(0.252) 0.252 0.246

Locus 13821 :

2 alleles

99.2% missing data

4	(0.488) 0.491 0.489

1	(0.512) 0.509 0.511

Locus 13822 :

2 alleles

99.2% missing data

2	(0.487) 0.492 0.490

1	(0.513) 0.508 0.510

Locus 13823 :

2 alleles

99.2% missing data

4	(0.540) 0.543 0.547

1	(0.460) 0.457 0.453

Locus 13824 :

2 alleles

93.0% missing data

1	(0.878) 0.869 0.896

4	(0.122) 0.131 0.104

Locus 13825 :

2 alleles

96.9% missing data

3	(0.836) 0.839 0.837

2	(0.164) 0.161 0.163

Locus 13826 :

2 alleles

99.2% missing data

3	(0.485) 0.489 0.486

2	(0.515) 0.511 0.514

Locus 13827 :

2 alleles

97.7% missing data

1	(0.576) 0.590 0.572

2	(0.424) 0.410 0.428

Locus 13828 :

2 alleles

99.2% missing data

2	(0.311) 0.308 0.315

3	(0.689) 0.692 0.685

Locus 13829 :

2 alleles

99.2% missing data

2	(0.593) 0.588 0.599

3	(0.407) 0.412 0.401

Locus 13830 :

2 alleles

87.5% missing data

2	(0.908) 0.900 0.930

1	(0.092) 0.100 0.070

Locus 13831 :

2 alleles

88.7% missing data

2	(0.920) 0.911 0.936

1	(0.080) 0.089 0.064

Locus 13832 :

2 alleles

98.0% missing data

1	(0.764) 0.759 0.771

4	(0.236) 0.241 0.229

Locus 13833 :

3 alleles

98.8% missing data

3	(0.219) 0.217 0.221

1	(0.631) 0.629 0.630

4	(0.151) 0.153 0.149

Locus 13834 :

2 alleles

99.2% missing data

1	(0.680) 0.679 0.683

2	(0.320) 0.321 0.317

Locus 13835 :

2 alleles

99.2% missing data

3	(0.381) 0.386 0.382

2	(0.619) 0.614 0.618

Locus 13836 :

2 alleles

98.8% missing data

3	(0.301) 0.294 0.308

1	(0.699) 0.706 0.692

Locus 13837 :

2 alleles

99.2% missing data

2	(0.345) 0.348 0.349

1	(0.655) 0.652 0.651

Locus 13838 :

2 alleles

94.1% missing data

1	(0.852) 0.865 0.840

4	(0.148) 0.135 0.160

Locus 13839 :

2 alleles

99.2% missing data

1	(0.386) 0.377 0.393

2	(0.614) 0.623 0.607

Locus 13840 :

3 alleles

94.9% missing data

1	(0.518) 0.513 0.519

4	(0.146) 0.144 0.140

2	(0.336) 0.343 0.342

Locus 13841 :

2 alleles

99.2% missing data

1	(0.767) 0.767 0.766

2	(0.233) 0.233 0.234

Locus 13842 :

2 alleles

90.6% missing data

1	(0.913) 0.929 0.899

2	(0.087) 0.071 0.101

Locus 13843 :

2 alleles

94.5% missing data

4	(0.858) 0.863 0.855

2	(0.142) 0.137 0.145

Locus 13844 :

2 alleles

99.2% missing data

2	(0.372) 0.374 0.376

4	(0.628) 0.626 0.624

Locus 13845 :

2 alleles

99.2% missing data

3	(0.550) 0.551 0.555

1	(0.450) 0.449 0.445

Locus 13846 :

2 alleles

98.4% missing data

1	(0.725) 0.732 0.722

4	(0.275) 0.268 0.278

Locus 13847 :

2 alleles

99.2% missing data

1	(0.634) 0.632 0.638

4	(0.366) 0.368 0.362

Locus 13848 :

2 alleles

99.2% missing data

1	(0.477) 0.481 0.478

3	(0.523) 0.519 0.522

Locus 13849 :

2 alleles

96.9% missing data

1	(0.787) 0.789 0.792

4	(0.213) 0.211 0.208

Locus 13850 :

2 alleles

99.2% missing data

4	(0.596) 0.597 0.597

1	(0.404) 0.403 0.403

Locus 13851 :

2 alleles

99.2% missing data

1	(0.274) 0.274 0.275

4	(0.726) 0.726 0.725

Locus 13852 :

3 alleles

98.8% missing data

2	(0.371) 0.376 0.371

4	(0.239) 0.236 0.243

1	(0.390) 0.388 0.386

Locus 13853 :

2 alleles

96.9% missing data

2	(0.265) 0.263 0.252

4	(0.735) 0.737 0.748

Locus 13854 :

2 alleles

98.8% missing data

1	(0.609) 0.615 0.600

2	(0.391) 0.385 0.400

Locus 13855 :

2 alleles

99.2% missing data

3	(0.644) 0.636 0.645

2	(0.356) 0.364 0.355

Locus 13856 :

2 alleles

91.0% missing data

4	(0.923) 0.938 0.913

2	(0.077) 0.062 0.087

Locus 13857 :

2 alleles

99.2% missing data

2	(0.578) 0.578 0.577

1	(0.422) 0.422 0.423

Locus 13858 :

2 alleles

99.2% missing data

3	(0.285) 0.289 0.283

4	(0.715) 0.711 0.717

Locus 13859 :

2 alleles

99.2% missing data

1	(0.784) 0.785 0.783

3	(0.216) 0.215 0.217

Locus 13860 :

2 alleles

99.2% missing data

3	(0.748) 0.754 0.739

4	(0.252) 0.246 0.261

Locus 13861 :

2 alleles

99.2% missing data

4	(0.220) 0.218 0.219

2	(0.780) 0.782 0.781

Locus 13862 :

2 alleles

0.0% missing data

1	(0.987) 0.983 0.996

4	(0.013) 0.017 0.004

Locus 13863 :

2 alleles

99.2% missing data

4	(0.860) 0.854 0.865

2	(0.140) 0.146 0.135

Locus 13864 :

2 alleles

98.8% missing data

4	(0.717) 0.719 0.722

3	(0.283) 0.281 0.278

Locus 13865 :

2 alleles

99.2% missing data

4	(0.739) 0.741 0.732

2	(0.261) 0.259 0.268

Locus 13866 :

2 alleles

98.8% missing data

1	(0.660) 0.666 0.657

2	(0.340) 0.334 0.343

Locus 13867 :

2 alleles

99.2% missing data

4	(0.493) 0.485 0.493

3	(0.507) 0.515 0.507

Locus 13868 :

2 alleles

99.2% missing data

1	(0.677) 0.680 0.671

2	(0.323) 0.320 0.329

Locus 13869 :

2 alleles

98.8% missing data

4	(0.356) 0.362 0.352

1	(0.644) 0.638 0.648

Locus 13870 :

2 alleles

99.2% missing data

3	(0.542) 0.538 0.547

1	(0.458) 0.462 0.453

Locus 13871 :

2 alleles

99.2% missing data

1	(0.613) 0.609 0.612

2	(0.387) 0.391 0.388

Locus 13872 :

2 alleles

98.8% missing data

4	(0.527) 0.537 0.523

2	(0.473) 0.463 0.477

Locus 13873 :

2 alleles

99.2% missing data

3	(0.649) 0.648 0.650

1	(0.351) 0.352 0.350

Locus 13874 :

2 alleles

99.2% missing data

4	(0.268) 0.267 0.269

1	(0.732) 0.733 0.731

Locus 13875 :

2 alleles

99.2% missing data

4	(0.523) 0.517 0.529

3	(0.477) 0.483 0.471

Locus 13876 :

2 alleles

0.4% missing data

2	(0.985) 0.981 0.995

3	(0.015) 0.019 0.005

Locus 13877 :

2 alleles

99.2% missing data

1	(0.417) 0.415 0.419

3	(0.583) 0.585 0.581

Locus 13878 :

2 alleles

96.9% missing data

2	(0.088) 0.086 0.087

4	(0.912) 0.914 0.913

Locus 13879 :

2 alleles

99.2% missing data

2	(0.427) 0.425 0.434

1	(0.573) 0.575 0.566

Locus 13880 :

2 alleles

98.8% missing data

2	(0.704) 0.701 0.703

4	(0.296) 0.299 0.297

Locus 13881 :

2 alleles

99.2% missing data

4	(0.240) 0.233 0.249

2	(0.760) 0.767 0.751

Locus 13882 :

2 alleles

98.8% missing data

3	(0.817) 0.819 0.810

4	(0.183) 0.181 0.190

Locus 13883 :

2 alleles

99.2% missing data

2	(0.232) 0.239 0.228

3	(0.768) 0.761 0.772

Locus 13884 :

2 alleles

99.2% missing data

1	(0.222) 0.223 0.222

2	(0.778) 0.777 0.778

Locus 13885 :

2 alleles

99.2% missing data

1	(0.789) 0.792 0.785

3	(0.211) 0.208 0.215

Locus 13886 :

2 alleles

99.2% missing data

4	(0.743) 0.744 0.739

2	(0.257) 0.256 0.261

Locus 13887 :

2 alleles

99.2% missing data

1	(0.443) 0.445 0.445

3	(0.557) 0.555 0.555

Locus 13888 :

2 alleles

93.0% missing data

1	(0.914) 0.905 0.928

2	(0.086) 0.095 0.072

Locus 13889 :

2 alleles

99.2% missing data

1	(0.583) 0.591 0.574

2	(0.417) 0.409 0.426

Locus 13890 :

2 alleles

99.2% missing data

3	(0.200) 0.203 0.198

2	(0.800) 0.797 0.802

Locus 13891 :

2 alleles

99.2% missing data

1	(0.528) 0.528 0.526

3	(0.472) 0.472 0.474

Locus 13892 :

2 alleles

98.4% missing data

4	(0.607) 0.618 0.596

2	(0.393) 0.382 0.404

Locus 13893 :

2 alleles

97.3% missing data

3	(0.871) 0.872 0.870

2	(0.129) 0.128 0.130

Locus 13894 :

2 alleles

98.8% missing data

4	(0.513) 0.517 0.512

3	(0.487) 0.483 0.488

Locus 13895 :

2 alleles

99.2% missing data

1	(0.814) 0.807 0.815

3	(0.186) 0.193 0.185

Locus 13896 :

2 alleles

98.4% missing data

4	(0.563) 0.560 0.573

1	(0.437) 0.440 0.427

Locus 13897 :

2 alleles

99.2% missing data

4	(0.698) 0.694 0.697

2	(0.302) 0.306 0.303

Locus 13898 :

2 alleles

99.2% missing data

4	(0.775) 0.775 0.778

3	(0.225) 0.225 0.222

Locus 13899 :

2 alleles

99.2% missing data

4	(0.491) 0.498 0.487

2	(0.509) 0.502 0.513

Locus 13900 :

2 alleles

99.2% missing data

1	(0.482) 0.479 0.478

2	(0.518) 0.521 0.522

Locus 13901 :

2 alleles

0.0% missing data

3	(0.989) 0.983 0.997

2	(0.011) 0.017 0.003

Locus 13902 :

2 alleles

99.2% missing data

3	(0.563) 0.554 0.565

2	(0.437) 0.446 0.435

Locus 13903 :

2 alleles

99.2% missing data

4	(0.721) 0.726 0.718

1	(0.279) 0.274 0.282

Locus 13904 :

2 alleles

97.3% missing data

2	(0.157) 0.159 0.150

1	(0.843) 0.841 0.850

Locus 13905 :

2 alleles

92.6% missing data

2	(0.848) 0.839 0.868

4	(0.152) 0.161 0.132

Locus 13906 :

2 alleles

98.4% missing data

1	(0.488) 0.479 0.494

2	(0.512) 0.521 0.506

Locus 13907 :

2 alleles

99.2% missing data

4	(0.778) 0.782 0.774

3	(0.222) 0.218 0.226

Locus 13908 :

2 alleles

99.2% missing data

3	(0.535) 0.534 0.532

1	(0.465) 0.466 0.468

Locus 13909 :

2 alleles

99.2% missing data

1	(0.768) 0.769 0.771

4	(0.232) 0.231 0.229

Locus 13910 :

3 alleles

96.9% missing data

2	(0.146) 0.149 0.142

1	(0.345) 0.342 0.343

4	(0.509) 0.510 0.515

Locus 13911 :

2 alleles

99.2% missing data

3	(0.538) 0.543 0.541

2	(0.462) 0.457 0.459

Locus 13912 :

2 alleles

99.2% missing data

3	(0.332) 0.340 0.327

2	(0.668) 0.660 0.673

Locus 13913 :

3 alleles

89.8% missing data

4	(0.764) 0.806 0.744

2	(0.101) 0.083 0.106

3	(0.135) 0.111 0.150

Locus 13914 :

2 alleles

99.2% missing data

2	(0.650) 0.651 0.645

4	(0.350) 0.349 0.355

Locus 13915 :

2 alleles

98.8% missing data

2	(0.599) 0.587 0.609

1	(0.401) 0.413 0.391

Locus 13916 :

2 alleles

0.0% missing data

4	(0.987) 0.982 0.996

2	(0.013) 0.018 0.004

Locus 13917 :

2 alleles

99.2% missing data

3	(0.576) 0.570 0.575

1	(0.424) 0.430 0.425

Locus 13918 :

2 alleles

99.2% missing data

1	(0.288) 0.287 0.293

2	(0.712) 0.713 0.707

Locus 13919 :

2 alleles

99.2% missing data

2	(0.236) 0.238 0.235

3	(0.764) 0.762 0.765

Locus 13920 :

2 alleles

99.2% missing data

3	(0.851) 0.851 0.848

2	(0.149) 0.149 0.152

Locus 13921 :

2 alleles

98.0% missing data

1	(0.652) 0.657 0.648

3	(0.348) 0.343 0.352

Locus 13922 :

2 alleles

99.2% missing data

4	(0.451) 0.451 0.448

2	(0.549) 0.549 0.552

Locus 13923 :

2 alleles

99.2% missing data

2	(0.288) 0.279 0.298

3	(0.712) 0.721 0.702

Locus 13924 :

2 alleles

92.2% missing data

4	(0.834) 0.817 0.859

3	(0.166) 0.183 0.141

Locus 13925 :

3 alleles

98.8% missing data

4	(0.330) 0.323 0.338

3	(0.498) 0.501 0.496

1	(0.171) 0.176 0.167

Locus 13926 :

3 alleles

95.7% missing data

4	(0.710) 0.703 0.727

3	(0.142) 0.142 0.143

1	(0.148) 0.155 0.130

Locus 13927 :

2 alleles

99.2% missing data

1	(0.661) 0.668 0.651

4	(0.339) 0.332 0.349

Locus 13928 :

2 alleles

96.9% missing data

2	(0.834) 0.837 0.836

3	(0.166) 0.163 0.164

Locus 13929 :

2 alleles

97.3% missing data

1	(0.800) 0.794 0.811

3	(0.200) 0.206 0.189

Locus 13930 :

2 alleles

95.3% missing data

1	(0.794) 0.792 0.807

2	(0.206) 0.208 0.193

Locus 13931 :

2 alleles

99.2% missing data

2	(0.748) 0.751 0.745

3	(0.252) 0.249 0.255

Locus 13932 :

2 alleles

99.2% missing data

1	(0.467) 0.463 0.470

3	(0.533) 0.537 0.530

Locus 13933 :

2 alleles

99.2% missing data

4	(0.552) 0.556 0.551

2	(0.448) 0.444 0.449

Locus 13934 :

2 alleles

98.4% missing data

1	(0.646) 0.650 0.638

3	(0.354) 0.350 0.362

Locus 13935 :

2 alleles

98.8% missing data

2	(0.203) 0.200 0.210

1	(0.797) 0.800 0.790

Locus 13936 :

2 alleles

99.2% missing data

1	(0.533) 0.539 0.533

4	(0.467) 0.461 0.467

Locus 13937 :

2 alleles

99.2% missing data

2	(0.610) 0.602 0.616

4	(0.390) 0.398 0.384

Locus 13938 :

2 alleles

95.3% missing data

4	(0.153) 0.155 0.147

3	(0.847) 0.845 0.853

Locus 13939 :

2 alleles

99.2% missing data

4	(0.320) 0.319 0.328

3	(0.680) 0.681 0.672

Locus 13940 :

2 alleles

98.0% missing data

3	(0.700) 0.702 0.705

4	(0.300) 0.298 0.295

Locus 13941 :

2 alleles

8.6% missing data

1	(0.986) 0.988 0.993

3	(0.014) 0.012 0.007

Locus 13942 :

2 alleles

99.2% missing data

1	(0.769) 0.770 0.766

2	(0.231) 0.230 0.234

Locus 13943 :

2 alleles

99.2% missing data

2	(0.640) 0.643 0.642

3	(0.360) 0.357 0.358

Locus 13944 :

2 alleles

99.2% missing data

4	(0.774) 0.782 0.769

1	(0.226) 0.218 0.231

Locus 13945 :

2 alleles

99.2% missing data

4	(0.225) 0.228 0.225

3	(0.775) 0.772 0.775

Locus 13946 :

2 alleles

98.8% missing data

2	(0.715) 0.717 0.713

4	(0.285) 0.283 0.287

Locus 13947 :

2 alleles

97.7% missing data

3	(0.309) 0.313 0.302

4	(0.691) 0.687 0.698

Locus 13948 :

2 alleles

98.8% missing data

2	(0.791) 0.787 0.793

3	(0.209) 0.213 0.207

Locus 13949 :

2 alleles

99.2% missing data

2	(0.474) 0.472 0.479

1	(0.526) 0.528 0.521

Locus 13950 :

2 alleles

97.7% missing data

1	(0.822) 0.824 0.825

4	(0.178) 0.176 0.175

Locus 13951 :

2 alleles

99.2% missing data

4	(0.472) 0.471 0.475

2	(0.528) 0.529 0.525

Locus 13952 :

2 alleles

96.9% missing data

3	(0.162) 0.159 0.166

1	(0.838) 0.841 0.834

Locus 13953 :

2 alleles

99.2% missing data

3	(0.261) 0.259 0.266

1	(0.739) 0.741 0.734

Locus 13954 :

3 alleles

98.0% missing data

3	(0.427) 0.428 0.426

2	(0.439) 0.439 0.436

1	(0.134) 0.132 0.138

Locus 13955 :

2 alleles

99.2% missing data

1	(0.586) 0.576 0.589

2	(0.414) 0.424 0.411

Locus 13956 :

2 alleles

91.0% missing data

4	(0.914) 0.925 0.911

1	(0.086) 0.075 0.089

Locus 13957 :

2 alleles

99.2% missing data

3	(0.325) 0.320 0.332

2	(0.675) 0.680 0.668

Locus 13958 :

2 alleles

99.2% missing data

2	(0.387) 0.389 0.385

1	(0.613) 0.611 0.615

Locus 13959 :

2 alleles

98.4% missing data

1	(0.808) 0.807 0.812

2	(0.192) 0.193 0.188

Locus 13960 :

2 alleles

99.2% missing data

2	(0.509) 0.514 0.508

4	(0.491) 0.486 0.492

Locus 13961 :

2 alleles

99.2% missing data

3	(0.655) 0.645 0.658

1	(0.345) 0.355 0.342

Locus 13962 :

2 alleles

96.5% missing data

2	(0.756) 0.767 0.756

3	(0.244) 0.233 0.244

Locus 13963 :

2 alleles

99.2% missing data

4	(0.786) 0.782 0.794

3	(0.214) 0.218 0.206

Locus 13964 :

2 alleles

99.2% missing data

3	(0.684) 0.681 0.689

2	(0.316) 0.319 0.311

Locus 13965 :

2 alleles

98.8% missing data

1	(0.572) 0.573 0.564

3	(0.428) 0.427 0.436

Locus 13966 :

2 alleles

98.8% missing data

2	(0.177) 0.180 0.174

1	(0.823) 0.820 0.826

Locus 13967 :

2 alleles

99.2% missing data

1	(0.324) 0.319 0.326

2	(0.676) 0.681 0.674

Locus 13968 :

2 alleles

96.1% missing data

2	(0.861) 0.869 0.857

1	(0.139) 0.131 0.143

Locus 13969 :

2 alleles

99.2% missing data

3	(0.243) 0.244 0.252

4	(0.757) 0.756 0.748

Locus 13970 :

2 alleles

99.2% missing data

1	(0.324) 0.324 0.328

2	(0.676) 0.676 0.672

Locus 13971 :

2 alleles

99.2% missing data

4	(0.227) 0.224 0.231

1	(0.773) 0.776 0.769

Locus 13972 :

2 alleles

97.7% missing data

3	(0.252) 0.252 0.245

4	(0.748) 0.748 0.755

Locus 13973 :

2 alleles

96.1% missing data

4	(0.871) 0.881 0.862

3	(0.129) 0.119 0.138

Locus 13974 :

2 alleles

99.2% missing data

1	(0.779) 0.773 0.783

3	(0.221) 0.227 0.217

Locus 13975 :

2 alleles

99.2% missing data

3	(0.572) 0.579 0.570

1	(0.428) 0.421 0.430

Locus 13976 :

2 alleles

96.5% missing data

3	(0.803) 0.810 0.805

4	(0.197) 0.190 0.195

Locus 13977 :

2 alleles

99.2% missing data

3	(0.536) 0.535 0.537

2	(0.464) 0.465 0.463

Locus 13978 :

2 alleles

99.2% missing data

1	(0.454) 0.456 0.452

4	(0.546) 0.544 0.548

Locus 13979 :

2 alleles

99.2% missing data

2	(0.790) 0.789 0.796

1	(0.210) 0.211 0.204

Locus 13980 :

2 alleles

98.8% missing data

2	(0.707) 0.701 0.714

4	(0.293) 0.299 0.286

Locus 13981 :

2 alleles

84.4% missing data

1	(0.568) 0.653 0.483

3	(0.432) 0.347 0.517

Locus 13982 :

2 alleles

99.2% missing data

2	(0.688) 0.693 0.693

4	(0.312) 0.307 0.307

Locus 13983 :

2 alleles

97.7% missing data

3	(0.706) 0.712 0.704

4	(0.294) 0.288 0.296

Locus 13984 :

2 alleles

99.2% missing data

4	(0.321) 0.321 0.321

2	(0.679) 0.679 0.679

Locus 13985 :

2 alleles

84.8% missing data

4	(0.560) 0.641 0.478

1	(0.440) 0.359 0.522

Locus 13986 :

2 alleles

99.2% missing data

1	(0.512) 0.505 0.511

2	(0.488) 0.495 0.489

Locus 13987 :

2 alleles

99.2% missing data

2	(0.388) 0.390 0.393

3	(0.612) 0.610 0.607

Locus 13988 :

2 alleles

93.0% missing data

3	(0.942) 0.935 0.952

2	(0.058) 0.065 0.048

Locus 13989 :

2 alleles

99.2% missing data

4	(0.551) 0.550 0.547

1	(0.449) 0.450 0.453

Locus 13990 :

2 alleles

98.8% missing data

1	(0.372) 0.371 0.367

4	(0.628) 0.629 0.633

Locus 13991 :

2 alleles

98.8% missing data

1	(0.526) 0.532 0.527

3	(0.474) 0.468 0.473

Locus 13992 :

3 alleles

98.8% missing data

1	(0.202) 0.201 0.205

4	(0.485) 0.476 0.490

3	(0.313) 0.323 0.305

Locus 13993 :

2 alleles

99.2% missing data

3	(0.124) 0.131 0.118

1	(0.876) 0.869 0.882

Locus 13994 :

2 alleles

99.2% missing data

4	(0.459) 0.455 0.462

2	(0.541) 0.545 0.538

Locus 13995 :

2 alleles

99.2% missing data

3	(0.669) 0.669 0.672

1	(0.331) 0.331 0.328

Locus 13996 :

2 alleles

99.2% missing data

2	(0.587) 0.586 0.588

3	(0.413) 0.414 0.412

Locus 13997 :

2 alleles

99.2% missing data

2	(0.604) 0.597 0.611

4	(0.396) 0.403 0.389

Locus 13998 :

2 alleles

99.2% missing data

1	(0.325) 0.322 0.322

3	(0.675) 0.678 0.678

Locus 13999 :

2 alleles

99.2% missing data

1	(0.701) 0.698 0.704

3	(0.299) 0.302 0.296

Locus 14000 :

2 alleles

90.6% missing data

2	(0.893) 0.911 0.877

3	(0.107) 0.089 0.123

Locus 14001 :

2 alleles

99.2% missing data

4	(0.733) 0.723 0.740

3	(0.267) 0.277 0.260

Locus 14002 :

2 alleles

89.1% missing data

3	(0.714) 0.765 0.668

1	(0.286) 0.235 0.332

Locus 14003 :

2 alleles

97.3% missing data

1	(0.112) 0.112 0.108

2	(0.888) 0.888 0.892

Locus 14004 :

2 alleles

99.2% missing data

1	(0.314) 0.310 0.315

2	(0.686) 0.690 0.685

Locus 14005 :

2 alleles

99.2% missing data

3	(0.476) 0.481 0.473

4	(0.524) 0.519 0.527

Locus 14006 :

2 alleles

96.9% missing data

4	(0.848) 0.838 0.857

2	(0.152) 0.162 0.143

Locus 14007 :

3 alleles

98.4% missing data

2	(0.471) 0.472 0.464

3	(0.344) 0.343 0.349

1	(0.185) 0.186 0.187

Locus 14008 :

2 alleles

99.2% missing data

3	(0.121) 0.126 0.119

4	(0.879) 0.874 0.881

Locus 14009 :

2 alleles

96.9% missing data

1	(0.760) 0.760 0.767

2	(0.240) 0.240 0.233

Locus 14010 :

2 alleles

99.2% missing data

3	(0.481) 0.474 0.488

4	(0.519) 0.526 0.512

Locus 14011 :

2 alleles

96.9% missing data

3	(0.387) 0.388 0.379

1	(0.613) 0.612 0.621

Locus 14012 :

2 alleles

89.8% missing data

1	(0.836) 0.870 0.796

3	(0.164) 0.130 0.204

Locus 14013 :

2 alleles

98.8% missing data

3	(0.700) 0.696 0.709

1	(0.300) 0.304 0.291

Locus 14014 :

2 alleles

98.4% missing data

2	(0.729) 0.725 0.737

1	(0.271) 0.275 0.263

Locus 14015 :

2 alleles

99.2% missing data

1	(0.439) 0.436 0.444

4	(0.561) 0.564 0.556

Locus 14016 :

2 alleles

99.2% missing data

2	(0.539) 0.541 0.538

4	(0.461) 0.459 0.462

Locus 14017 :

2 alleles

96.1% missing data

4	(0.810) 0.823 0.803

3	(0.190) 0.177 0.197

Locus 14018 :

2 alleles

99.2% missing data

2	(0.226) 0.218 0.234

1	(0.774) 0.782 0.766

Locus 14019 :

2 alleles

99.2% missing data

1	(0.673) 0.673 0.672

3	(0.327) 0.327 0.328

Locus 14020 :

2 alleles

98.8% missing data

3	(0.412) 0.414 0.419

2	(0.588) 0.586 0.581

Locus 14021 :

3 alleles

97.3% missing data

1	(0.501) 0.512 0.504

3	(0.222) 0.219 0.220

2	(0.278) 0.269 0.276

Locus 14022 :

2 alleles

99.2% missing data

3	(0.556) 0.548 0.564

2	(0.444) 0.452 0.436

Locus 14023 :

2 alleles

99.2% missing data

1	(0.400) 0.408 0.395

3	(0.600) 0.592 0.605

Locus 14024 :

2 alleles

99.2% missing data

1	(0.570) 0.568 0.576

2	(0.430) 0.432 0.424

Locus 14025 :

2 alleles

99.2% missing data

1	(0.485) 0.496 0.475

2	(0.515) 0.504 0.525

Locus 14026 :

2 alleles

98.4% missing data

4	(0.833) 0.836 0.830

1	(0.167) 0.164 0.170

Locus 14027 :

2 alleles

99.2% missing data

1	(0.649) 0.643 0.653

4	(0.351) 0.357 0.347

Locus 14028 :

2 alleles

98.8% missing data

3	(0.308) 0.309 0.305

1	(0.692) 0.691 0.695

Locus 14029 :

2 alleles

99.2% missing data

2	(0.255) 0.258 0.253

1	(0.745) 0.742 0.747

Locus 14030 :

2 alleles

98.4% missing data

1	(0.795) 0.794 0.798

4	(0.205) 0.206 0.202

Locus 14031 :

2 alleles

99.2% missing data

2	(0.667) 0.665 0.671

4	(0.333) 0.335 0.329

Locus 14032 :

2 alleles

93.8% missing data

3	(0.869) 0.858 0.892

1	(0.131) 0.142 0.108

Locus 14033 :

2 alleles

98.8% missing data

4	(0.375) 0.364 0.384

3	(0.625) 0.636 0.616

Locus 14034 :

2 alleles

99.2% missing data

4	(0.509) 0.516 0.513

1	(0.491) 0.484 0.487

Locus 14035 :

2 alleles

99.2% missing data

3	(0.254) 0.256 0.253

1	(0.746) 0.744 0.747

Locus 14036 :

2 alleles

99.2% missing data

1	(0.607) 0.605 0.610

3	(0.393) 0.395 0.390

Locus 14037 :

2 alleles

99.2% missing data

2	(0.232) 0.225 0.239

3	(0.768) 0.775 0.761

Locus 14038 :

2 alleles

89.8% missing data

1	(0.936) 0.948 0.928

3	(0.064) 0.052 0.072

Locus 14039 :

2 alleles

98.8% missing data

3	(0.625) 0.619 0.632

1	(0.375) 0.381 0.368

Locus 14040 :

2 alleles

99.2% missing data

1	(0.236) 0.232 0.238

2	(0.764) 0.768 0.762

Locus 14041 :

2 alleles

99.2% missing data

3	(0.410) 0.413 0.400

2	(0.590) 0.587 0.600

Locus 14042 :

2 alleles

99.2% missing data

2	(0.453) 0.444 0.455

4	(0.547) 0.556 0.545

Locus 14043 :

2 alleles

99.2% missing data

4	(0.552) 0.560 0.551

2	(0.448) 0.440 0.449

Locus 14044 :

2 alleles

93.0% missing data

1	(0.858) 0.851 0.880

3	(0.142) 0.149 0.120

Locus 14045 :

2 alleles

99.2% missing data

2	(0.590) 0.580 0.594

1	(0.410) 0.420 0.406

Locus 14046 :

2 alleles

99.2% missing data

2	(0.835) 0.830 0.838

1	(0.165) 0.170 0.162

Locus 14047 :

2 alleles

92.6% missing data

2	(0.897) 0.888 0.910

1	(0.103) 0.112 0.090

Locus 14048 :

2 alleles

99.2% missing data

4	(0.383) 0.376 0.390

2	(0.617) 0.624 0.610

Locus 14049 :

2 alleles

99.2% missing data

2	(0.436) 0.443 0.436

3	(0.564) 0.557 0.564

Locus 14050 :

2 alleles

96.9% missing data

4	(0.870) 0.869 0.876

3	(0.130) 0.131 0.124

Locus 14051 :

3 alleles

98.8% missing data

1	(0.275) 0.271 0.280

2	(0.393) 0.391 0.391

3	(0.332) 0.337 0.329

Locus 14052 :

2 alleles

99.2% missing data

4	(0.674) 0.672 0.677

1	(0.326) 0.328 0.323

Locus 14053 :

2 alleles

99.2% missing data

2	(0.631) 0.628 0.626

1	(0.369) 0.372 0.374

Locus 14054 :

2 alleles

99.2% missing data

2	(0.402) 0.397 0.410

1	(0.598) 0.603 0.590

Locus 14055 :

2 alleles

97.3% missing data

1	(0.259) 0.269 0.251

3	(0.741) 0.731 0.749

Locus 14056 :

2 alleles

99.2% missing data

3	(0.216) 0.209 0.223

4	(0.784) 0.791 0.777

Locus 14057 :

2 alleles

99.2% missing data

1	(0.464) 0.459 0.466

3	(0.536) 0.541 0.534

Locus 14058 :

2 alleles

97.7% missing data

1	(0.244) 0.236 0.250

4	(0.756) 0.764 0.750

Locus 14059 :

2 alleles

99.2% missing data

2	(0.644) 0.645 0.646

1	(0.356) 0.355 0.354

Locus 14060 :

2 alleles

99.2% missing data

1	(0.583) 0.581 0.582

3	(0.417) 0.419 0.418

Locus 14061 :

2 alleles

99.2% missing data

1	(0.310) 0.306 0.305

3	(0.690) 0.694 0.695

Locus 14062 :

2 alleles

99.2% missing data

3	(0.459) 0.457 0.461

2	(0.541) 0.543 0.539

Locus 14063 :

3 alleles

98.8% missing data

2	(0.246) 0.240 0.247

1	(0.295) 0.300 0.289

3	(0.460) 0.460 0.464

Locus 14064 :

2 alleles

99.2% missing data

3	(0.602) 0.601 0.600

1	(0.398) 0.399 0.400

Locus 14065 :

2 alleles

99.2% missing data

1	(0.566) 0.555 0.568

2	(0.434) 0.445 0.432

Locus 14066 :

2 alleles

98.8% missing data

1	(0.703) 0.698 0.705

4	(0.297) 0.302 0.295

Locus 14067 :

2 alleles

99.2% missing data

4	(0.396) 0.390 0.406

1	(0.604) 0.610 0.594

Locus 14068 :

2 alleles

99.2% missing data

4	(0.515) 0.512 0.518

3	(0.485) 0.488 0.482

Locus 14069 :

2 alleles

98.8% missing data

3	(0.690) 0.694 0.686

1	(0.310) 0.306 0.314

Locus 14070 :

2 alleles

98.8% missing data

1	(0.615) 0.606 0.616

2	(0.385) 0.394 0.384

Locus 14071 :

2 alleles

98.8% missing data

3	(0.724) 0.724 0.724

2	(0.276) 0.276 0.276

Locus 14072 :

2 alleles

99.2% missing data

2	(0.451) 0.442 0.456

4	(0.549) 0.558 0.544

Locus 14073 :

2 alleles

98.8% missing data

1	(0.637) 0.630 0.642

3	(0.363) 0.370 0.358

Locus 14074 :

2 alleles

99.2% missing data

4	(0.638) 0.635 0.638

3	(0.362) 0.365 0.362

Locus 14075 :

2 alleles

99.2% missing data

1	(0.775) 0.773 0.776

2	(0.225) 0.227 0.224

Locus 14076 :

2 alleles

99.2% missing data

1	(0.326) 0.329 0.317

4	(0.674) 0.671 0.683

Locus 14077 :

2 alleles

96.9% missing data

3	(0.838) 0.841 0.841

1	(0.162) 0.159 0.159

Locus 14078 :

2 alleles

98.4% missing data

1	(0.630) 0.631 0.624

2	(0.370) 0.369 0.376

Locus 14079 :

2 alleles

98.8% missing data

2	(0.391) 0.389 0.399

1	(0.609) 0.611 0.601

Locus 14080 :

2 alleles

98.8% missing data

2	(0.713) 0.708 0.714

3	(0.287) 0.292 0.286

Locus 14081 :

2 alleles

99.2% missing data

4	(0.667) 0.668 0.669

2	(0.333) 0.332 0.331

Locus 14082 :

2 alleles

99.2% missing data

2	(0.339) 0.348 0.333

3	(0.661) 0.652 0.667

Locus 14083 :

2 alleles

98.8% missing data

2	(0.592) 0.590 0.601

3	(0.408) 0.410 0.399

Locus 14084 :

2 alleles

99.2% missing data

2	(0.318) 0.305 0.326

3	(0.682) 0.695 0.674

Locus 14085 :

2 alleles

99.2% missing data

2	(0.414) 0.415 0.418

4	(0.586) 0.585 0.582

Locus 14086 :

2 alleles

99.2% missing data

1	(0.656) 0.657 0.654

3	(0.344) 0.343 0.346

Locus 14087 :

2 alleles

99.2% missing data

3	(0.385) 0.382 0.388

2	(0.615) 0.618 0.612

Locus 14088 :

2 alleles

98.8% missing data

2	(0.557) 0.559 0.544

1	(0.443) 0.441 0.456

Locus 14089 :

2 alleles

99.2% missing data

2	(0.677) 0.671 0.678

1	(0.323) 0.329 0.322

Locus 14090 :

2 alleles

99.2% missing data

3	(0.273) 0.270 0.280

1	(0.727) 0.730 0.720

Locus 14091 :

2 alleles

95.3% missing data

2	(0.888) 0.883 0.898

1	(0.112) 0.117 0.102

Locus 14092 :

2 alleles

99.2% missing data

4	(0.471) 0.468 0.476

2	(0.529) 0.532 0.524

Locus 14093 :

2 alleles

99.2% missing data

3	(0.409) 0.407 0.414

2	(0.591) 0.593 0.586

Locus 14094 :

2 alleles

99.2% missing data

3	(0.664) 0.664 0.666

4	(0.336) 0.336 0.334

Locus 14095 :

2 alleles

99.2% missing data

1	(0.793) 0.798 0.788

2	(0.207) 0.202 0.212

Locus 14096 :

2 alleles

99.2% missing data

4	(0.396) 0.407 0.387

3	(0.604) 0.593 0.613

Locus 14097 :

2 alleles

96.1% missing data

2	(0.744) 0.745 0.755

1	(0.256) 0.255 0.245

Locus 14098 :

2 alleles

99.2% missing data

1	(0.617) 0.607 0.621

4	(0.383) 0.393 0.379

Locus 14099 :

2 alleles

99.2% missing data

3	(0.590) 0.585 0.589

2	(0.410) 0.415 0.411

Locus 14100 :

2 alleles

99.2% missing data

1	(0.656) 0.658 0.651

4	(0.344) 0.342 0.349

Locus 14101 :

2 alleles

99.2% missing data

3	(0.297) 0.298 0.297

1	(0.703) 0.702 0.703

Locus 14102 :

2 alleles

99.2% missing data

1	(0.696) 0.692 0.696

4	(0.304) 0.308 0.304

Locus 14103 :

2 alleles

99.2% missing data

3	(0.584) 0.583 0.582

4	(0.416) 0.417 0.418

Locus 14104 :

2 alleles

99.2% missing data

3	(0.668) 0.656 0.675

4	(0.332) 0.344 0.325

Locus 14105 :

2 alleles

99.2% missing data

2	(0.445) 0.449 0.440

3	(0.555) 0.551 0.560

Locus 14106 :

2 alleles

99.2% missing data

4	(0.490) 0.487 0.492

1	(0.510) 0.513 0.508

Locus 14107 :

2 alleles

99.2% missing data

4	(0.781) 0.781 0.776

3	(0.219) 0.219 0.224

Locus 14108 :

2 alleles

99.2% missing data

1	(0.674) 0.672 0.676

2	(0.326) 0.328 0.324

Locus 14109 :

2 alleles

98.8% missing data

2	(0.434) 0.432 0.436

3	(0.566) 0.568 0.564

Locus 14110 :

2 alleles

97.7% missing data

3	(0.259) 0.263 0.250

1	(0.741) 0.737 0.750

Locus 14111 :

2 alleles

99.2% missing data

1	(0.369) 0.365 0.373

4	(0.631) 0.635 0.627

Locus 14112 :

2 alleles

99.2% missing data

2	(0.550) 0.547 0.549

3	(0.450) 0.453 0.451

Locus 14113 :

2 alleles

99.2% missing data

4	(0.568) 0.567 0.572

1	(0.432) 0.433 0.428

Locus 14114 :

2 alleles

99.2% missing data

2	(0.562) 0.551 0.569

1	(0.438) 0.449 0.431

Locus 14115 :

2 alleles

98.8% missing data

2	(0.795) 0.797 0.796

4	(0.205) 0.203 0.204

Locus 14116 :

2 alleles

99.2% missing data

1	(0.591) 0.588 0.600

4	(0.409) 0.412 0.400

Locus 14117 :

2 alleles

98.8% missing data

3	(0.263) 0.263 0.261

4	(0.737) 0.737 0.739

Locus 14118 :

2 alleles

98.8% missing data

1	(0.670) 0.667 0.674

4	(0.330) 0.333 0.326

Locus 14119 :

2 alleles

99.2% missing data

4	(0.632) 0.628 0.632

3	(0.368) 0.372 0.368

Locus 14120 :

2 alleles

99.2% missing data

4	(0.720) 0.722 0.718

2	(0.280) 0.278 0.282

Locus 14121 :

2 alleles

98.8% missing data

3	(0.483) 0.479 0.497

4	(0.517) 0.521 0.503

Locus 14122 :

2 alleles

99.2% missing data

2	(0.280) 0.285 0.277

4	(0.720) 0.715 0.723

Locus 14123 :

2 alleles

99.2% missing data

1	(0.435) 0.431 0.437

4	(0.565) 0.569 0.563

Locus 14124 :

2 alleles

91.0% missing data

2	(0.924) 0.938 0.914

3	(0.076) 0.062 0.086

Locus 14125 :

2 alleles

96.1% missing data

1	(0.779) 0.797 0.774

2	(0.221) 0.203 0.226

Locus 14126 :

2 alleles

99.2% missing data

3	(0.402) 0.406 0.403

1	(0.598) 0.594 0.597

Locus 14127 :

2 alleles

99.2% missing data

3	(0.391) 0.384 0.397

2	(0.609) 0.616 0.603

Locus 14128 :

2 alleles

99.2% missing data

1	(0.631) 0.634 0.627

2	(0.369) 0.366 0.373

Locus 14129 :

2 alleles

98.8% missing data

1	(0.555) 0.541 0.559

2	(0.445) 0.459 0.441

Locus 14130 :

2 alleles

96.9% missing data

2	(0.354) 0.354 0.365

3	(0.646) 0.646 0.635

Locus 14131 :

2 alleles

99.2% missing data

1	(0.558) 0.550 0.563

4	(0.442) 0.450 0.437

Locus 14132 :

2 alleles

99.2% missing data

3	(0.343) 0.342 0.337

1	(0.657) 0.658 0.663

Locus 14133 :

2 alleles

94.5% missing data

1	(0.605) 0.635 0.576

2	(0.395) 0.365 0.424

Locus 14134 :

2 alleles

99.2% missing data

1	(0.469) 0.462 0.475

3	(0.531) 0.538 0.525

Locus 14135 :

2 alleles

99.2% missing data

4	(0.249) 0.242 0.254

2	(0.751) 0.758 0.746

Locus 14136 :

2 alleles

93.0% missing data

2	(0.904) 0.903 0.911

4	(0.096) 0.097 0.089

Locus 14137 :

2 alleles

98.8% missing data

1	(0.520) 0.510 0.523

2	(0.480) 0.490 0.477

Locus 14138 :

2 alleles

97.7% missing data

2	(0.718) 0.728 0.712

3	(0.282) 0.272 0.288

Locus 14139 :

2 alleles

99.2% missing data

2	(0.522) 0.526 0.525

3	(0.478) 0.474 0.475

Locus 14140 :

2 alleles

99.2% missing data

1	(0.396) 0.403 0.394

3	(0.604) 0.597 0.606

Locus 14141 :

2 alleles

98.4% missing data

3	(0.556) 0.559 0.550

1	(0.444) 0.441 0.450

Locus 14142 :

3 alleles

44.9% missing data

3	(0.927) 0.889 0.962

1	(0.054) 0.095 0.025

4	(0.019) 0.016 0.013

Locus 14143 :

2 alleles

99.2% missing data

3	(0.447) 0.441 0.456

2	(0.553) 0.559 0.544

Locus 14144 :

3 alleles

98.4% missing data

3	(0.570) 0.566 0.574

4	(0.222) 0.227 0.214

1	(0.208) 0.207 0.212

Locus 14145 :

2 alleles

99.2% missing data

3	(0.432) 0.431 0.437

1	(0.568) 0.569 0.563

Locus 14146 :

2 alleles

99.2% missing data

1	(0.447) 0.451 0.441

2	(0.553) 0.549 0.559

Locus 14147 :

2 alleles

99.2% missing data

3	(0.279) 0.271 0.279

1	(0.721) 0.729 0.721

Locus 14148 :

2 alleles

99.2% missing data

2	(0.348) 0.343 0.347

1	(0.652) 0.657 0.653

Locus 14149 :

2 alleles

98.4% missing data

4	(0.272) 0.275 0.267

1	(0.728) 0.725 0.733

Locus 14150 :

2 alleles

97.7% missing data

1	(0.604) 0.601 0.609

4	(0.396) 0.399 0.391

Locus 14151 :

2 alleles

98.8% missing data

4	(0.587) 0.590 0.590

3	(0.413) 0.410 0.410

Locus 14152 :

2 alleles

99.2% missing data

3	(0.410) 0.407 0.406

2	(0.590) 0.593 0.594

Locus 14153 :

2 alleles

99.2% missing data

4	(0.641) 0.638 0.645

2	(0.359) 0.362 0.355

Locus 14154 :

2 alleles

55.5% missing data

4	(0.894) 0.898 0.898

1	(0.106) 0.102 0.102

Locus 14155 :

2 alleles

95.7% missing data

4	(0.857) 0.855 0.866

3	(0.143) 0.145 0.134

Locus 14156 :

2 alleles

98.0% missing data

3	(0.507) 0.494 0.524

1	(0.493) 0.506 0.476

Locus 14157 :

2 alleles

98.8% missing data

3	(0.535) 0.541 0.538

1	(0.465) 0.459 0.462

Locus 14158 :

2 alleles

0.0% missing data

4	(0.985) 0.982 0.996

1	(0.015) 0.018 0.004

Locus 14159 :

2 alleles

99.2% missing data

1	(0.472) 0.468 0.481

2	(0.528) 0.532 0.519

Locus 14160 :

2 alleles

96.9% missing data

1	(0.700) 0.699 0.713

2	(0.300) 0.301 0.287

Locus 14161 :

3 alleles

98.8% missing data

4	(0.293) 0.289 0.293

3	(0.270) 0.268 0.274

1	(0.437) 0.443 0.433

Locus 14162 :

2 alleles

98.8% missing data

2	(0.452) 0.445 0.450

1	(0.548) 0.555 0.550

Locus 14163 :

2 alleles

97.7% missing data

2	(0.544) 0.533 0.563

3	(0.456) 0.467 0.437

Locus 14164 :

2 alleles

99.2% missing data

4	(0.471) 0.475 0.467

3	(0.529) 0.525 0.533

Locus 14165 :

3 alleles

98.8% missing data

3	(0.253) 0.252 0.258

4	(0.314) 0.316 0.306

1	(0.433) 0.432 0.436

Locus 14166 :

2 alleles

99.2% missing data

3	(0.690) 0.693 0.682

1	(0.310) 0.307 0.318

Locus 14167 :

2 alleles

98.8% missing data

3	(0.375) 0.367 0.385

2	(0.625) 0.633 0.615

Locus 14168 :

2 alleles

97.7% missing data

1	(0.697) 0.691 0.706

2	(0.303) 0.309 0.294

Locus 14169 :

2 alleles

96.9% missing data

4	(0.748) 0.734 0.763

1	(0.252) 0.266 0.237

Locus 14170 :

2 alleles

99.2% missing data

4	(0.689) 0.697 0.682

3	(0.311) 0.303 0.318

Locus 14171 :

2 alleles

99.2% missing data

1	(0.589) 0.585 0.592

2	(0.411) 0.415 0.408

Locus 14172 :

2 alleles

91.0% missing data

1	(0.922) 0.939 0.912

4	(0.078) 0.061 0.088

Locus 14173 :

2 alleles

99.2% missing data

3	(0.573) 0.567 0.577

2	(0.427) 0.433 0.423

Locus 14174 :

2 alleles

99.2% missing data

4	(0.559) 0.551 0.564

3	(0.441) 0.449 0.436

Locus 14175 :

2 alleles

98.4% missing data

4	(0.806) 0.797 0.812

1	(0.194) 0.203 0.188

Locus 14176 :

2 alleles

99.2% missing data

1	(0.776) 0.775 0.775

3	(0.224) 0.225 0.225

Locus 14177 :

2 alleles

92.6% missing data

2	(0.554) 0.519 0.589

4	(0.446) 0.481 0.411

Locus 14178 :

2 alleles

0.0% missing data

2	(0.969) 0.921 0.991

1	(0.031) 0.079 0.009

Locus 14179 :

2 alleles

99.2% missing data

2	(0.477) 0.467 0.478

4	(0.523) 0.533 0.522

Locus 14180 :

2 alleles

98.8% missing data

4	(0.764) 0.767 0.762

2	(0.236) 0.233 0.238

Locus 14181 :

2 alleles

97.7% missing data

3	(0.742) 0.737 0.755

1	(0.258) 0.263 0.245

Locus 14182 :

2 alleles

99.2% missing data

4	(0.605) 0.606 0.606

2	(0.395) 0.394 0.394

Locus 14183 :

2 alleles

99.2% missing data

1	(0.292) 0.292 0.304

4	(0.708) 0.708 0.696

Locus 14184 :

2 alleles

97.7% missing data

4	(0.434) 0.423 0.434

1	(0.566) 0.577 0.566

Locus 14185 :

2 alleles

96.5% missing data

3	(0.784) 0.795 0.783

4	(0.216) 0.205 0.217

Locus 14186 :

2 alleles

99.2% missing data

1	(0.729) 0.722 0.729

2	(0.271) 0.278 0.271

Locus 14187 :

2 alleles

91.0% missing data

1	(0.919) 0.926 0.920

2	(0.081) 0.074 0.080

Locus 14188 :

2 alleles

98.8% missing data

3	(0.482) 0.482 0.482

1	(0.518) 0.518 0.518

Locus 14189 :

2 alleles

99.2% missing data

3	(0.728) 0.734 0.725

2	(0.272) 0.266 0.275

Locus 14190 :

2 alleles

0.0% missing data

2	(0.985) 0.982 0.996

1	(0.015) 0.018 0.004

Locus 14191 :

2 alleles

99.2% missing data

4	(0.334) 0.337 0.333

1	(0.666) 0.663 0.667

Locus 14192 :

2 alleles

98.8% missing data

3	(0.658) 0.660 0.658

2	(0.342) 0.340 0.342

Locus 14193 :

2 alleles

99.2% missing data

2	(0.456) 0.450 0.461

3	(0.544) 0.550 0.539

Locus 14194 :

2 alleles

99.2% missing data

3	(0.357) 0.357 0.354

1	(0.643) 0.643 0.646

Locus 14195 :

2 alleles

99.2% missing data

1	(0.652) 0.646 0.658

2	(0.348) 0.354 0.342

Locus 14196 :

2 alleles

98.0% missing data

1	(0.337) 0.335 0.334

2	(0.663) 0.665 0.666

Locus 14197 :

2 alleles

98.4% missing data

3	(0.596) 0.596 0.600

4	(0.404) 0.404 0.400

Locus 14198 :

2 alleles

99.2% missing data

3	(0.752) 0.754 0.749

1	(0.248) 0.246 0.251

Locus 14199 :

2 alleles

99.2% missing data

4	(0.539) 0.532 0.539

2	(0.461) 0.468 0.461

Locus 14200 :

2 alleles

92.6% missing data

4	(0.943) 0.951 0.935

2	(0.057) 0.049 0.065

Locus 14201 :

2 alleles

99.2% missing data

4	(0.551) 0.548 0.552

1	(0.449) 0.452 0.448

Locus 14202 :

2 alleles

98.4% missing data

4	(0.351) 0.343 0.369

1	(0.649) 0.657 0.631

Locus 14203 :

2 alleles

96.5% missing data

3	(0.831) 0.833 0.838

1	(0.169) 0.167 0.162

Locus 14204 :

2 alleles

99.2% missing data

4	(0.739) 0.745 0.735

1	(0.261) 0.255 0.265

Locus 14205 :

2 alleles

99.2% missing data

3	(0.654) 0.655 0.647

1	(0.346) 0.345 0.353

Locus 14206 :

2 alleles

99.2% missing data

3	(0.536) 0.537 0.533

4	(0.464) 0.463 0.467

Locus 14207 :

2 alleles

98.8% missing data

4	(0.627) 0.622 0.638

3	(0.373) 0.378 0.362

Locus 14208 :

2 alleles

99.2% missing data

4	(0.491) 0.491 0.494

1	(0.509) 0.509 0.506

Locus 14209 :

2 alleles

98.8% missing data

2	(0.410) 0.410 0.413

1	(0.590) 0.590 0.587

Locus 14210 :

2 alleles

98.0% missing data

4	(0.379) 0.367 0.388

2	(0.621) 0.633 0.612

Locus 14211 :

2 alleles

99.2% missing data

1	(0.524) 0.518 0.527

4	(0.476) 0.482 0.473

Locus 14212 :

2 alleles

94.9% missing data

1	(0.898) 0.906 0.892

2	(0.102) 0.094 0.108

Locus 14213 :

2 alleles

99.2% missing data

2	(0.658) 0.661 0.656

1	(0.342) 0.339 0.344

Locus 14214 :

2 alleles

99.2% missing data

4	(0.477) 0.480 0.479

2	(0.523) 0.520 0.521

Locus 14215 :

2 alleles

98.8% missing data

2	(0.430) 0.427 0.433

1	(0.570) 0.573 0.567

Locus 14216 :

2 alleles

92.2% missing data

1	(0.944) 0.946 0.945

2	(0.056) 0.054 0.055

Locus 14217 :

2 alleles

99.2% missing data

2	(0.741) 0.740 0.736

1	(0.259) 0.260 0.264

Locus 14218 :

2 alleles

99.2% missing data

4	(0.681) 0.681 0.680

3	(0.319) 0.319 0.320

Locus 14219 :

2 alleles

99.2% missing data

1	(0.406) 0.412 0.400

2	(0.594) 0.588 0.600

Locus 14220 :

2 alleles

99.2% missing data

3	(0.475) 0.472 0.482

1	(0.525) 0.528 0.518

Locus 14221 :

2 alleles

99.2% missing data

4	(0.333) 0.333 0.334

2	(0.667) 0.667 0.666

Locus 14222 :

2 alleles

98.0% missing data

2	(0.812) 0.809 0.814

4	(0.188) 0.191 0.186

Locus 14223 :

2 alleles

96.9% missing data

1	(0.819) 0.807 0.835

3	(0.181) 0.193 0.165

Locus 14224 :

2 alleles

98.8% missing data

2	(0.449) 0.444 0.452

4	(0.551) 0.556 0.548

Locus 14225 :

2 alleles

97.7% missing data

2	(0.812) 0.805 0.821

3	(0.188) 0.195 0.179

Locus 14226 :

2 alleles

99.2% missing data

2	(0.495) 0.488 0.507

1	(0.505) 0.512 0.493

Locus 14227 :

2 alleles

99.2% missing data

2	(0.725) 0.725 0.726

1	(0.275) 0.275 0.274

Locus 14228 :

2 alleles

98.8% missing data

3	(0.379) 0.374 0.383

2	(0.621) 0.626 0.617

Locus 14229 :

2 alleles

99.2% missing data

2	(0.381) 0.388 0.375

4	(0.619) 0.612 0.625

Locus 14230 :

2 alleles

99.2% missing data

4	(0.384) 0.380 0.385

3	(0.616) 0.620 0.615

Locus 14231 :

2 alleles

99.2% missing data

4	(0.677) 0.675 0.684

2	(0.323) 0.325 0.316

Locus 14232 :

2 alleles

98.8% missing data

2	(0.542) 0.540 0.558

1	(0.458) 0.460 0.442

Locus 14233 :

3 alleles

98.8% missing data

4	(0.314) 0.311 0.324

1	(0.255) 0.260 0.245

2	(0.431) 0.428 0.431

Locus 14234 :

2 alleles

98.0% missing data

3	(0.749) 0.756 0.744

1	(0.251) 0.244 0.256

Locus 14235 :

2 alleles

99.2% missing data

4	(0.583) 0.575 0.588

2	(0.417) 0.425 0.412

Locus 14236 :

2 alleles

99.2% missing data

1	(0.389) 0.382 0.389

4	(0.611) 0.618 0.611

Locus 14237 :

2 alleles

99.2% missing data

3	(0.574) 0.576 0.571

2	(0.426) 0.424 0.429

Locus 14238 :

2 alleles

99.2% missing data

2	(0.358) 0.357 0.361

4	(0.642) 0.643 0.639

Locus 14239 :

2 alleles

98.0% missing data

1	(0.451) 0.451 0.452

2	(0.549) 0.549 0.548

Locus 14240 :

2 alleles

97.7% missing data

2	(0.809) 0.803 0.819

4	(0.191) 0.197 0.181

Locus 14241 :

2 alleles

98.8% missing data

2	(0.523) 0.515 0.535

1	(0.477) 0.485 0.465

Locus 14242 :

2 alleles

93.0% missing data

3	(0.892) 0.886 0.909

1	(0.108) 0.114 0.091

Locus 14243 :

2 alleles

98.8% missing data

3	(0.589) 0.588 0.595

2	(0.411) 0.412 0.405

Locus 14244 :

3 alleles

90.6% missing data

4	(0.857) 0.871 0.847

2	(0.074) 0.062 0.084

1	(0.069) 0.067 0.069

Locus 14245 :

2 alleles

99.2% missing data

4	(0.442) 0.444 0.444

3	(0.558) 0.556 0.556

Locus 14246 :

2 alleles

89.8% missing data

1	(0.852) 0.878 0.821

4	(0.148) 0.122 0.179

Locus 14247 :

3 alleles

98.4% missing data

2	(0.538) 0.528 0.542

1	(0.255) 0.265 0.247

4	(0.207) 0.208 0.211

Locus 14248 :

2 alleles

99.2% missing data

4	(0.356) 0.357 0.355

3	(0.644) 0.643 0.645

Locus 14249 :

2 alleles

99.2% missing data

1	(0.665) 0.671 0.659

4	(0.335) 0.329 0.341

Locus 14250 :

2 alleles

98.8% missing data

1	(0.751) 0.746 0.753

4	(0.249) 0.254 0.247

Locus 14251 :

2 alleles

99.2% missing data

1	(0.241) 0.242 0.243

2	(0.759) 0.758 0.757

Locus 14252 :

2 alleles

99.2% missing data

3	(0.408) 0.407 0.408

1	(0.592) 0.593 0.592

Locus 14253 :

2 alleles

99.2% missing data

4	(0.465) 0.467 0.467

1	(0.535) 0.533 0.533

Locus 14254 :

2 alleles

99.2% missing data

4	(0.399) 0.390 0.405

3	(0.601) 0.610 0.595

Locus 14255 :

2 alleles

0.0% missing data

4	(0.868) 0.892 0.850

1	(0.132) 0.108 0.150

Locus 14256 :

2 alleles

91.0% missing data

4	(0.884) 0.907 0.878

2	(0.116) 0.093 0.122

Locus 14257 :

2 alleles

0.0% missing data

4	(0.987) 0.989 0.993

3	(0.013) 0.011 0.007

Locus 14258 :

2 alleles

98.8% missing data

1	(0.388) 0.384 0.396

3	(0.612) 0.616 0.604

Locus 14259 :

2 alleles

99.2% missing data

2	(0.272) 0.267 0.273

4	(0.728) 0.733 0.727

Locus 14260 :

2 alleles

99.2% missing data

3	(0.731) 0.727 0.733

2	(0.269) 0.273 0.267

Locus 14261 :

2 alleles

99.2% missing data

3	(0.485) 0.487 0.481

1	(0.515) 0.513 0.519

Locus 14262 :

2 alleles

99.2% missing data

4	(0.355) 0.347 0.358

1	(0.645) 0.653 0.642

Locus 14263 :

2 alleles

98.4% missing data

2	(0.833) 0.833 0.839

3	(0.167) 0.167 0.161

Locus 14264 :

2 alleles

98.0% missing data

1	(0.762) 0.768 0.760

3	(0.238) 0.232 0.240

Locus 14265 :

2 alleles

99.2% missing data

4	(0.221) 0.223 0.220

2	(0.779) 0.777 0.780

Locus 14266 :

2 alleles

95.3% missing data

1	(0.826) 0.828 0.831

3	(0.174) 0.172 0.169

Locus 14267 :

2 alleles

99.2% missing data

4	(0.388) 0.399 0.386

2	(0.612) 0.601 0.614

Locus 14268 :

2 alleles

96.1% missing data

4	(0.851) 0.860 0.847

1	(0.149) 0.140 0.153

Locus 14269 :

2 alleles

98.8% missing data

1	(0.645) 0.647 0.644

4	(0.355) 0.353 0.356

Locus 14270 :

2 alleles

99.2% missing data

2	(0.403) 0.396 0.409

3	(0.597) 0.604 0.591

Locus 14271 :

2 alleles

99.2% missing data

1	(0.261) 0.258 0.267

3	(0.739) 0.742 0.733

Locus 14272 :

2 alleles

98.8% missing data

4	(0.692) 0.684 0.693

3	(0.308) 0.316 0.307

Locus 14273 :

2 alleles

8.2% missing data

4	(0.986) 0.978 0.996

3	(0.014) 0.022 0.004

Locus 14274 :

2 alleles

0.0% missing data

3	(0.986) 0.990 0.993

2	(0.014) 0.010 0.007

Locus 14275 :

2 alleles

99.2% missing data

4	(0.543) 0.537 0.545

2	(0.457) 0.463 0.455

Locus 14276 :

2 alleles

99.2% missing data

3	(0.504) 0.500 0.510

4	(0.496) 0.500 0.490

Locus 14277 :

2 alleles

99.2% missing data

1	(0.520) 0.524 0.515

4	(0.480) 0.476 0.485

Locus 14278 :

2 alleles

99.2% missing data

1	(0.697) 0.696 0.702

2	(0.303) 0.304 0.298

Locus 14279 :

2 alleles

98.8% missing data

1	(0.708) 0.708 0.704

2	(0.292) 0.292 0.296

Locus 14280 :

2 alleles

98.8% missing data

3	(0.535) 0.537 0.526

4	(0.465) 0.463 0.474

Locus 14281 :

2 alleles

0.0% missing data

1	(0.982) 0.963 0.994

3	(0.018) 0.037 0.006

Locus 14282 :

2 alleles

99.2% missing data

1	(0.687) 0.688 0.688

2	(0.313) 0.312 0.312

Locus 14283 :

2 alleles

99.2% missing data

1	(0.841) 0.846 0.837

4	(0.159) 0.154 0.163

Locus 14284 :

2 alleles

99.2% missing data

1	(0.651) 0.652 0.654

2	(0.349) 0.348 0.346

Locus 14285 :

3 alleles

97.3% missing data

4	(0.150) 0.153 0.146

2	(0.231) 0.228 0.230

3	(0.619) 0.619 0.624

Locus 14286 :

2 alleles

99.2% missing data

3	(0.222) 0.223 0.220

1	(0.778) 0.777 0.780

Locus 14287 :

2 alleles

99.2% missing data

1	(0.537) 0.543 0.530

3	(0.463) 0.457 0.470

Locus 14288 :

2 alleles

98.8% missing data

4	(0.763) 0.767 0.762

3	(0.237) 0.233 0.238

Locus 14289 :

2 alleles

97.3% missing data

1	(0.797) 0.796 0.803

2	(0.203) 0.204 0.197

Locus 14290 :

2 alleles

99.2% missing data

1	(0.704) 0.703 0.701

4	(0.296) 0.297 0.299

Locus 14291 :

2 alleles

98.4% missing data

4	(0.522) 0.521 0.520

3	(0.478) 0.479 0.480

Locus 14292 :

2 alleles

99.2% missing data

4	(0.354) 0.353 0.353

1	(0.646) 0.647 0.647

Locus 14293 :

2 alleles

99.2% missing data

3	(0.608) 0.609 0.608

2	(0.392) 0.391 0.392

Locus 14294 :

2 alleles

99.2% missing data

2	(0.331) 0.322 0.341

4	(0.669) 0.678 0.659

Locus 14295 :

2 alleles

99.2% missing data

4	(0.714) 0.718 0.711

1	(0.286) 0.282 0.289

Locus 14296 :

2 alleles

99.2% missing data

2	(0.709) 0.703 0.707

3	(0.291) 0.297 0.293

Locus 14297 :

2 alleles

98.8% missing data

1	(0.693) 0.691 0.690

4	(0.307) 0.309 0.310

Locus 14298 :

2 alleles

99.2% missing data

4	(0.712) 0.712 0.710

3	(0.288) 0.288 0.290

Locus 14299 :

2 alleles

98.4% missing data

4	(0.736) 0.741 0.738

2	(0.264) 0.259 0.262

Locus 14300 :

2 alleles

99.2% missing data

1	(0.464) 0.458 0.468

2	(0.536) 0.542 0.532

Locus 14301 :

2 alleles

98.4% missing data

3	(0.423) 0.428 0.415

1	(0.577) 0.572 0.585

Locus 14302 :

2 alleles

99.2% missing data

1	(0.264) 0.269 0.262

3	(0.736) 0.731 0.738

Locus 14303 :

2 alleles

97.7% missing data

4	(0.788) 0.792 0.786

2	(0.212) 0.208 0.214

Locus 14304 :

2 alleles

97.7% missing data

3	(0.205) 0.200 0.198

4	(0.795) 0.800 0.802

Locus 14305 :

2 alleles

99.2% missing data

1	(0.813) 0.808 0.807

2	(0.187) 0.192 0.193

Locus 14306 :

3 alleles

98.4% missing data

3	(0.485) 0.479 0.491

2	(0.209) 0.217 0.202

4	(0.306) 0.304 0.307

Locus 14307 :

3 alleles

97.7% missing data

2	(0.387) 0.390 0.402

1	(0.294) 0.287 0.290

3	(0.319) 0.323 0.308

Locus 14308 :

2 alleles

99.2% missing data

1	(0.248) 0.248 0.253

2	(0.752) 0.752 0.747

Locus 14309 :

3 alleles

98.4% missing data

4	(0.180) 0.177 0.181

2	(0.417) 0.420 0.417

1	(0.403) 0.403 0.402

Locus 14310 :

2 alleles

96.5% missing data

1	(0.409) 0.407 0.413

4	(0.591) 0.593 0.587

Locus 14311 :

2 alleles

99.2% missing data

1	(0.665) 0.666 0.659

2	(0.335) 0.334 0.341

Locus 14312 :

2 alleles

98.8% missing data

1	(0.406) 0.397 0.410

4	(0.594) 0.603 0.590

Locus 14313 :

2 alleles

99.2% missing data

3	(0.487) 0.484 0.492

4	(0.513) 0.516 0.508

Locus 14314 :

2 alleles

98.0% missing data

1	(0.777) 0.781 0.779

4	(0.223) 0.219 0.221

Locus 14315 :

3 alleles

98.4% missing data

3	(0.478) 0.472 0.486

1	(0.267) 0.267 0.266

2	(0.254) 0.261 0.248

Locus 14316 :

2 alleles

98.4% missing data

4	(0.638) 0.636 0.644

1	(0.362) 0.364 0.356

Locus 14317 :

2 alleles

0.0% missing data

4	(0.988) 0.990 0.993

1	(0.012) 0.010 0.007

Locus 14318 :

2 alleles

99.2% missing data

2	(0.574) 0.577 0.582

4	(0.426) 0.423 0.418

Locus 14319 :

2 alleles

99.2% missing data

2	(0.423) 0.431 0.419

3	(0.577) 0.569 0.581

Locus 14320 :

2 alleles

99.2% missing data

1	(0.395) 0.401 0.386

4	(0.605) 0.599 0.614

Locus 14321 :

2 alleles

99.2% missing data

2	(0.425) 0.419 0.426

1	(0.575) 0.581 0.574

Locus 14322 :

2 alleles

91.0% missing data

2	(0.889) 0.904 0.883

3	(0.111) 0.096 0.117

Locus 14323 :

2 alleles

99.2% missing data

3	(0.585) 0.578 0.588

1	(0.415) 0.422 0.412

Locus 14324 :

2 alleles

99.2% missing data

4	(0.287) 0.289 0.287

2	(0.713) 0.711 0.713

Locus 14325 :

2 alleles

96.5% missing data

2	(0.340) 0.344 0.333

4	(0.660) 0.656 0.667

Locus 14326 :

2 alleles

99.2% missing data

3	(0.444) 0.442 0.446

2	(0.556) 0.558 0.554

Locus 14327 :

2 alleles

99.2% missing data

4	(0.644) 0.643 0.649

3	(0.356) 0.357 0.351

Locus 14328 :

2 alleles

99.2% missing data

4	(0.652) 0.645 0.663

1	(0.348) 0.355 0.337

Locus 14329 :

2 alleles

99.2% missing data

3	(0.481) 0.479 0.483

1	(0.519) 0.521 0.517

Locus 14330 :

2 alleles

98.4% missing data

2	(0.739) 0.735 0.747

1	(0.261) 0.265 0.253

Locus 14331 :

3 alleles

98.4% missing data

2	(0.287) 0.285 0.301

4	(0.364) 0.358 0.366

3	(0.349) 0.357 0.334

Locus 14332 :

2 alleles

99.2% missing data

3	(0.621) 0.606 0.623

1	(0.379) 0.394 0.377

Locus 14333 :

2 alleles

99.2% missing data

2	(0.337) 0.337 0.339

1	(0.663) 0.663 0.661

Locus 14334 :

2 alleles

8.2% missing data

4	(0.985) 0.978 0.995

2	(0.015) 0.022 0.005

Locus 14335 :

2 alleles

97.3% missing data

2	(0.636) 0.628 0.659

3	(0.364) 0.372 0.341

Locus 14336 :

2 alleles

99.2% missing data

1	(0.414) 0.417 0.411

2	(0.586) 0.583 0.589

Locus 14337 :

2 alleles

98.0% missing data

3	(0.843) 0.843 0.843

2	(0.157) 0.157 0.157

Locus 14338 :

2 alleles

99.2% missing data

1	(0.513) 0.515 0.508

2	(0.487) 0.485 0.492

Locus 14339 :

2 alleles

99.2% missing data

2	(0.498) 0.500 0.502

3	(0.502) 0.500 0.498

Locus 14340 :

2 alleles

99.2% missing data

3	(0.519) 0.515 0.530

2	(0.481) 0.485 0.470

Locus 14341 :

2 alleles

94.1% missing data

2	(0.868) 0.870 0.874

4	(0.132) 0.130 0.126

Locus 14342 :

2 alleles

99.2% missing data

3	(0.471) 0.462 0.475

2	(0.529) 0.538 0.525

Locus 14343 :

2 alleles

98.8% missing data

4	(0.339) 0.333 0.331

2	(0.661) 0.667 0.669

Locus 14344 :

2 alleles

99.2% missing data

3	(0.714) 0.712 0.719

4	(0.286) 0.288 0.281

Locus 14345 :

2 alleles

99.2% missing data

3	(0.223) 0.218 0.231

2	(0.777) 0.782 0.769

Locus 14346 :

2 alleles

99.2% missing data

3	(0.656) 0.655 0.655

1	(0.344) 0.345 0.345

Locus 14347 :

2 alleles

99.2% missing data

4	(0.703) 0.699 0.704

1	(0.297) 0.301 0.296

Locus 14348 :

2 alleles

96.1% missing data

2	(0.882) 0.881 0.883

1	(0.118) 0.119 0.117

Locus 14349 :

2 alleles

99.2% missing data

4	(0.728) 0.725 0.722

1	(0.272) 0.275 0.278

Locus 14350 :

2 alleles

99.2% missing data

1	(0.583) 0.581 0.584

3	(0.417) 0.419 0.416

Locus 14351 :

2 alleles

98.8% missing data

1	(0.760) 0.756 0.763

4	(0.240) 0.244 0.237

Locus 14352 :

2 alleles

97.7% missing data

2	(0.842) 0.850 0.832

4	(0.158) 0.150 0.168

Locus 14353 :

2 alleles

99.2% missing data

1	(0.782) 0.780 0.784

4	(0.218) 0.220 0.216

Locus 14354 :

2 alleles

99.2% missing data

2	(0.686) 0.692 0.683

1	(0.314) 0.308 0.317

Locus 14355 :

2 alleles

98.0% missing data

3	(0.556) 0.548 0.572

2	(0.444) 0.452 0.428

Locus 14356 :

2 alleles

99.2% missing data

3	(0.618) 0.611 0.620

1	(0.382) 0.389 0.380

Locus 14357 :

2 alleles

99.2% missing data

4	(0.284) 0.291 0.285

2	(0.716) 0.709 0.715

Locus 14358 :

2 alleles

99.2% missing data

3	(0.504) 0.501 0.503

4	(0.496) 0.499 0.497

Locus 14359 :

2 alleles

99.2% missing data

3	(0.468) 0.468 0.466

2	(0.532) 0.532 0.534

Locus 14360 :

2 alleles

99.2% missing data

1	(0.490) 0.490 0.487

4	(0.510) 0.510 0.513

Locus 14361 :

2 alleles

99.2% missing data

1	(0.459) 0.457 0.463

2	(0.541) 0.543 0.537

Locus 14362 :

2 alleles

99.2% missing data

4	(0.458) 0.452 0.457

3	(0.542) 0.548 0.543

Locus 14363 :

2 alleles

99.2% missing data

2	(0.646) 0.638 0.652

4	(0.354) 0.362 0.348

Locus 14364 :

2 alleles

99.2% missing data

4	(0.450) 0.455 0.450

3	(0.550) 0.545 0.550

Locus 14365 :

2 alleles

99.2% missing data

2	(0.402) 0.398 0.403

3	(0.598) 0.602 0.597

Locus 14366 :

2 alleles

98.8% missing data

3	(0.545) 0.543 0.554

1	(0.455) 0.457 0.446

Locus 14367 :

2 alleles

8.6% missing data

4	(0.983) 0.976 0.995

1	(0.017) 0.024 0.005

Locus 14368 :

2 alleles

91.0% missing data

4	(0.927) 0.940 0.917

2	(0.073) 0.060 0.083

Locus 14369 :

2 alleles

99.2% missing data

1	(0.542) 0.547 0.536

2	(0.458) 0.453 0.464

Locus 14370 :

3 alleles

98.8% missing data

4	(0.275) 0.274 0.279

2	(0.556) 0.560 0.542

1	(0.169) 0.167 0.179

Locus 14371 :

3 alleles

88.7% missing data

3	(0.836) 0.829 0.863

4	(0.065) 0.061 0.060

2	(0.099) 0.110 0.077

Locus 14372 :

2 alleles

97.7% missing data

4	(0.113) 0.106 0.117

1	(0.887) 0.894 0.883

Locus 14373 :

2 alleles

99.2% missing data

2	(0.749) 0.742 0.754

3	(0.251) 0.258 0.246

Locus 14374 :

2 alleles

99.2% missing data

3	(0.456) 0.449 0.461

1	(0.544) 0.551 0.539

Locus 14375 :

2 alleles

99.2% missing data

2	(0.459) 0.451 0.457

1	(0.541) 0.549 0.543

Locus 14376 :

2 alleles

98.8% missing data

1	(0.635) 0.642 0.632

4	(0.365) 0.358 0.368

Locus 14377 :

2 alleles

98.8% missing data

3	(0.455) 0.449 0.463

1	(0.545) 0.551 0.537

Locus 14378 :

2 alleles

99.2% missing data

2	(0.589) 0.589 0.587

4	(0.411) 0.411 0.413

Locus 14379 :

2 alleles

99.2% missing data

3	(0.346) 0.337 0.352

2	(0.654) 0.663 0.648

Locus 14380 :

2 alleles

98.8% missing data

3	(0.433) 0.437 0.438

1	(0.567) 0.563 0.562

Locus 14381 :

2 alleles

99.2% missing data

2	(0.407) 0.413 0.403

1	(0.593) 0.587 0.597

Locus 14382 :

2 alleles

99.2% missing data

1	(0.578) 0.570 0.581

2	(0.422) 0.430 0.419

Locus 14383 :

2 alleles

98.8% missing data

3	(0.421) 0.420 0.426

4	(0.579) 0.580 0.574

Locus 14384 :

2 alleles

99.2% missing data

1	(0.594) 0.596 0.586

4	(0.406) 0.404 0.414

Locus 14385 :

3 alleles

98.8% missing data

2	(0.339) 0.336 0.343

1	(0.307) 0.309 0.307

3	(0.354) 0.355 0.349

Locus 14386 :

2 alleles

99.2% missing data

3	(0.311) 0.307 0.317

4	(0.689) 0.693 0.683

Locus 14387 :

2 alleles

99.2% missing data

4	(0.536) 0.530 0.544

1	(0.464) 0.470 0.456

Locus 14388 :

2 alleles

99.2% missing data

1	(0.767) 0.770 0.765

2	(0.233) 0.230 0.235

Locus 14389 :

2 alleles

91.0% missing data

3	(0.083) 0.068 0.098

1	(0.917) 0.932 0.902

Locus 14390 :

2 alleles

99.2% missing data

1	(0.818) 0.816 0.817

4	(0.182) 0.184 0.183

Locus 14391 :

2 alleles

8.6% missing data

4	(0.983) 0.975 0.995

1	(0.017) 0.025 0.005

Locus 14392 :

2 alleles

99.2% missing data

3	(0.529) 0.531 0.527

4	(0.471) 0.469 0.473

Locus 14393 :

2 alleles

98.8% missing data

2	(0.465) 0.463 0.468

3	(0.535) 0.537 0.532

Locus 14394 :

2 alleles

99.2% missing data

4	(0.462) 0.462 0.463

2	(0.538) 0.538 0.537

Locus 14395 :

2 alleles

99.2% missing data

3	(0.518) 0.510 0.523

1	(0.482) 0.490 0.477

Locus 14396 :

2 alleles

99.2% missing data

3	(0.396) 0.397 0.401

1	(0.604) 0.603 0.599

Locus 14397 :

2 alleles

83.2% missing data

2	(0.931) 0.914 0.949

4	(0.069) 0.086 0.051

Locus 14398 :

3 alleles

98.4% missing data

2	(0.405) 0.402 0.407

4	(0.285) 0.284 0.292

1	(0.309) 0.314 0.301

Locus 14399 :

2 alleles

99.2% missing data

2	(0.504) 0.511 0.494

1	(0.496) 0.489 0.506

Locus 14400 :

2 alleles

99.2% missing data

3	(0.509) 0.503 0.514

2	(0.491) 0.497 0.486

Locus 14401 :

2 alleles

98.4% missing data

4	(0.318) 0.316 0.319

2	(0.682) 0.684 0.681

Locus 14402 :

2 alleles

99.2% missing data

1	(0.408) 0.404 0.413

4	(0.592) 0.596 0.587

Locus 14403 :

2 alleles

95.3% missing data

1	(0.819) 0.821 0.825

3	(0.181) 0.179 0.175

Locus 14404 :

2 alleles

99.2% missing data

3	(0.650) 0.639 0.655

4	(0.350) 0.361 0.345

Locus 14405 :

2 alleles

99.2% missing data

3	(0.355) 0.356 0.357

2	(0.645) 0.644 0.643

Locus 14406 :

2 alleles

97.7% missing data

3	(0.686) 0.690 0.686

4	(0.314) 0.310 0.314

Locus 14407 :

2 alleles

94.9% missing data

3	(0.742) 0.764 0.730

1	(0.258) 0.236 0.270

Locus 14408 :

2 alleles

98.8% missing data

4	(0.471) 0.469 0.472

2	(0.529) 0.531 0.528

Locus 14409 :

2 alleles

99.2% missing data

2	(0.780) 0.783 0.780

1	(0.220) 0.217 0.220

Locus 14410 :

2 alleles

96.9% missing data

4	(0.730) 0.722 0.744

1	(0.270) 0.278 0.256

Locus 14411 :

2 alleles

98.8% missing data

3	(0.328) 0.327 0.338

1	(0.672) 0.673 0.662

Locus 14412 :

2 alleles

91.0% missing data

4	(0.921) 0.935 0.909

2	(0.079) 0.065 0.091

Locus 14413 :

2 alleles

8.2% missing data

4	(0.985) 0.978 0.996

3	(0.015) 0.022 0.004

Locus 14414 :

2 alleles

95.3% missing data

2	(0.788) 0.779 0.801

4	(0.212) 0.221 0.199

Locus 14415 :

2 alleles

98.8% missing data

2	(0.466) 0.463 0.468

1	(0.534) 0.537 0.532

Locus 14416 :

2 alleles

99.2% missing data

3	(0.867) 0.870 0.866

1	(0.133) 0.130 0.134

Locus 14417 :

2 alleles

98.8% missing data

1	(0.488) 0.489 0.496

4	(0.512) 0.511 0.504

Locus 14418 :

3 alleles

98.4% missing data

3	(0.402) 0.392 0.413

1	(0.361) 0.366 0.356

4	(0.238) 0.242 0.231

Locus 14419 :

2 alleles

99.2% missing data

2	(0.812) 0.812 0.814

3	(0.188) 0.188 0.186

Locus 14420 :

2 alleles

93.0% missing data

1	(0.125) 0.125 0.118

4	(0.875) 0.875 0.882

Locus 14421 :

2 alleles

98.8% missing data

1	(0.416) 0.417 0.416

3	(0.584) 0.583 0.584

Locus 14422 :

2 alleles

99.2% missing data

3	(0.711) 0.710 0.707

1	(0.289) 0.290 0.293

Locus 14423 :

2 alleles

99.2% missing data

2	(0.230) 0.227 0.232

4	(0.770) 0.773 0.768

Locus 14424 :

2 alleles

99.2% missing data

3	(0.570) 0.572 0.577

1	(0.430) 0.428 0.423

Locus 14425 :

2 alleles

92.2% missing data

4	(0.578) 0.536 0.619

3	(0.422) 0.464 0.381

Locus 14426 :

3 alleles

98.4% missing data

2	(0.412) 0.412 0.423

1	(0.283) 0.278 0.281

3	(0.305) 0.309 0.296

Locus 14427 :

2 alleles

99.2% missing data

2	(0.359) 0.354 0.360

4	(0.641) 0.646 0.640

Locus 14428 :

2 alleles

98.4% missing data

4	(0.709) 0.717 0.705

3	(0.291) 0.283 0.295

Locus 14429 :

2 alleles

98.4% missing data

2	(0.828) 0.823 0.832

1	(0.172) 0.177 0.168

Locus 14430 :

2 alleles

96.9% missing data

2	(0.751) 0.748 0.766

3	(0.249) 0.252 0.234

Locus 14431 :

2 alleles

99.2% missing data

4	(0.450) 0.449 0.451

3	(0.550) 0.551 0.549

Locus 14432 :

2 alleles

99.2% missing data

3	(0.492) 0.477 0.490

4	(0.508) 0.523 0.510

Locus 14433 :

3 alleles

98.0% missing data

3	(0.132) 0.131 0.136

2	(0.574) 0.573 0.584

1	(0.295) 0.296 0.280

Locus 14434 :

2 alleles

99.2% missing data

1	(0.281) 0.281 0.281

2	(0.719) 0.719 0.719

Locus 14435 :

2 alleles

99.2% missing data

4	(0.635) 0.642 0.635

3	(0.365) 0.358 0.365

Locus 14436 :

2 alleles

99.2% missing data

3	(0.703) 0.711 0.700

1	(0.297) 0.289 0.300

Locus 14437 :

3 alleles

98.8% missing data

2	(0.327) 0.323 0.333

1	(0.316) 0.321 0.309

4	(0.357) 0.356 0.358

Locus 14438 :

2 alleles

99.2% missing data

4	(0.321) 0.319 0.326

2	(0.679) 0.681 0.674

Locus 14439 :

2 alleles

99.2% missing data

4	(0.711) 0.715 0.703

1	(0.289) 0.285 0.297

Locus 14440 :

2 alleles

99.2% missing data

4	(0.400) 0.408 0.401

3	(0.600) 0.592 0.599

Locus 14441 :

3 alleles

98.4% missing data

3	(0.268) 0.266 0.268

2	(0.447) 0.445 0.448

4	(0.285) 0.288 0.285

Locus 14442 :

2 alleles

99.2% missing data

4	(0.618) 0.616 0.622

2	(0.382) 0.384 0.378

Locus 14443 :

2 alleles

99.2% missing data

4	(0.449) 0.452 0.447

2	(0.551) 0.548 0.553

Locus 14444 :

2 alleles

99.2% missing data

2	(0.148) 0.143 0.155

3	(0.852) 0.857 0.845

Locus 14445 :

2 alleles

98.0% missing data

3	(0.615) 0.612 0.607

4	(0.385) 0.388 0.393

Locus 14446 :

2 alleles

98.4% missing data

4	(0.583) 0.577 0.602

1	(0.417) 0.423 0.398

Locus 14447 :

2 alleles

99.2% missing data

3	(0.357) 0.356 0.359

4	(0.643) 0.644 0.641

Locus 14448 :

2 alleles

99.2% missing data

4	(0.653) 0.653 0.651

2	(0.347) 0.347 0.349

Locus 14449 :

2 alleles

93.4% missing data

4	(0.889) 0.882 0.906

2	(0.111) 0.118 0.094

Locus 14450 :

2 alleles

99.2% missing data

2	(0.816) 0.812 0.812

4	(0.184) 0.188 0.188

Locus 14451 :

2 alleles

99.2% missing data

1	(0.660) 0.660 0.660

4	(0.340) 0.340 0.340

Locus 14452 :

2 alleles

99.2% missing data

2	(0.504) 0.499 0.505

4	(0.496) 0.501 0.495

Locus 14453 :

2 alleles

98.4% missing data

4	(0.852) 0.857 0.843

1	(0.148) 0.143 0.157

Locus 14454 :

2 alleles

98.8% missing data

4	(0.672) 0.674 0.672

1	(0.328) 0.326 0.328

Locus 14455 :

2 alleles

99.2% missing data

4	(0.400) 0.398 0.401

2	(0.600) 0.602 0.599

Locus 14456 :

2 alleles

99.2% missing data

4	(0.623) 0.620 0.630

3	(0.377) 0.380 0.370

Locus 14457 :

2 alleles

98.8% missing data

1	(0.298) 0.292 0.305

4	(0.702) 0.708 0.695

Locus 14458 :

2 alleles

99.2% missing data

3	(0.434) 0.436 0.439

2	(0.566) 0.564 0.561

Locus 14459 :

2 alleles

96.9% missing data

3	(0.360) 0.370 0.351

4	(0.640) 0.630 0.649

Locus 14460 :

2 alleles

99.2% missing data

4	(0.512) 0.515 0.512

2	(0.488) 0.485 0.488

Locus 14461 :

2 alleles

99.2% missing data

3	(0.244) 0.246 0.244

1	(0.756) 0.754 0.756

Locus 14462 :

2 alleles

96.1% missing data

2	(0.762) 0.767 0.768

3	(0.238) 0.233 0.232

Locus 14463 :

2 alleles

98.0% missing data

3	(0.438) 0.436 0.454

1	(0.562) 0.564 0.546

Locus 14464 :

2 alleles

99.2% missing data

2	(0.541) 0.539 0.539

3	(0.459) 0.461 0.461

Locus 14465 :

2 alleles

99.2% missing data

4	(0.408) 0.404 0.413

1	(0.592) 0.596 0.587

Locus 14466 :

2 alleles

99.2% missing data

2	(0.285) 0.285 0.288

1	(0.715) 0.715 0.712

Locus 14467 :

2 alleles

99.2% missing data

3	(0.444) 0.446 0.440

4	(0.556) 0.554 0.560

Locus 14468 :

2 alleles

99.2% missing data

1	(0.612) 0.614 0.605

3	(0.388) 0.386 0.395

Locus 14469 :

2 alleles

98.0% missing data

3	(0.748) 0.756 0.748

4	(0.252) 0.244 0.252

Locus 14470 :

2 alleles

8.2% missing data

4	(0.975) 0.948 0.992

3	(0.025) 0.052 0.008

Locus 14471 :

2 alleles

99.2% missing data

2	(0.394) 0.393 0.393

4	(0.606) 0.607 0.607

Locus 14472 :

2 alleles

99.2% missing data

3	(0.388) 0.393 0.381

4	(0.612) 0.607 0.619

Locus 14473 :

2 alleles

99.2% missing data

2	(0.388) 0.388 0.389

4	(0.612) 0.612 0.611

Locus 14474 :

2 alleles

0.0% missing data

3	(0.985) 0.980 0.995

2	(0.015) 0.020 0.005

Locus 14475 :

2 alleles

99.2% missing data

4	(0.552) 0.547 0.562

1	(0.448) 0.453 0.438

Locus 14476 :

2 alleles

99.2% missing data

2	(0.644) 0.644 0.644

3	(0.356) 0.356 0.356

Locus 14477 :

2 alleles

99.2% missing data

3	(0.583) 0.589 0.574

4	(0.417) 0.411 0.426

Locus 14478 :

2 alleles

99.2% missing data

4	(0.513) 0.513 0.508

2	(0.487) 0.487 0.492

Locus 14479 :

2 alleles

97.3% missing data

2	(0.822) 0.818 0.834

3	(0.178) 0.182 0.166

Locus 14480 :

2 alleles

99.2% missing data

4	(0.431) 0.431 0.437

3	(0.569) 0.569 0.563

Locus 14481 :

2 alleles

98.8% missing data

2	(0.483) 0.481 0.486

4	(0.517) 0.519 0.514

Locus 14482 :

2 alleles

99.2% missing data

3	(0.310) 0.310 0.315

2	(0.690) 0.690 0.685

Locus 14483 :

2 alleles

99.2% missing data

4	(0.531) 0.527 0.536

3	(0.469) 0.473 0.464

Locus 14484 :

2 alleles

8.6% missing data

3	(0.982) 0.972 0.995

2	(0.018) 0.028 0.005

Locus 14485 :

2 alleles

90.6% missing data

4	(0.893) 0.907 0.890

1	(0.107) 0.093 0.110

Locus 14486 :

2 alleles

99.2% missing data

1	(0.460) 0.455 0.467

3	(0.540) 0.545 0.533

Locus 14487 :

2 alleles

99.2% missing data

4	(0.299) 0.296 0.306

1	(0.701) 0.704 0.694

Locus 14488 :

2 alleles

10.2% missing data

2	(0.985) 0.977 0.996

1	(0.015) 0.023 0.004

Locus 14489 :

2 alleles

98.4% missing data

4	(0.809) 0.812 0.810

1	(0.191) 0.188 0.190

Locus 14490 :

2 alleles

99.2% missing data

4	(0.746) 0.749 0.739

3	(0.254) 0.251 0.261

Locus 14491 :

3 alleles

98.0% missing data

2	(0.186) 0.189 0.185

1	(0.216) 0.209 0.221

4	(0.598) 0.603 0.593

Locus 14492 :

2 alleles

95.3% missing data

4	(0.754) 0.769 0.755

1	(0.246) 0.231 0.245

Locus 14493 :

2 alleles

99.2% missing data

2	(0.557) 0.559 0.560

4	(0.443) 0.441 0.440

Locus 14494 :

2 alleles

8.2% missing data

4	(0.982) 0.974 0.995

3	(0.018) 0.026 0.005

Locus 14495 :

2 alleles

93.0% missing data

2	(0.855) 0.850 0.871

4	(0.145) 0.150 0.129

Locus 14496 :

2 alleles

98.8% missing data

4	(0.382) 0.386 0.380

3	(0.618) 0.614 0.620

Locus 14497 :

2 alleles

99.2% missing data

3	(0.465) 0.456 0.474

2	(0.535) 0.544 0.526

Locus 14498 :

3 alleles

98.4% missing data

4	(0.257) 0.252 0.261

2	(0.360) 0.363 0.357

3	(0.383) 0.385 0.382

Locus 14499 :

2 alleles

98.8% missing data

3	(0.687) 0.689 0.687

4	(0.313) 0.311 0.313

Locus 14500 :

2 alleles

99.2% missing data

3	(0.253) 0.254 0.256

4	(0.747) 0.746 0.744

Locus 14501 :

2 alleles

99.2% missing data

4	(0.678) 0.682 0.680

2	(0.322) 0.318 0.320

Locus 14502 :

2 alleles

99.2% missing data

1	(0.654) 0.656 0.654

2	(0.346) 0.344 0.346

Locus 14503 :

2 alleles

99.2% missing data

2	(0.535) 0.527 0.537

1	(0.465) 0.473 0.463

Locus 14504 :

2 alleles

99.2% missing data

4	(0.330) 0.329 0.328

2	(0.670) 0.671 0.672

Locus 14505 :

2 alleles

99.2% missing data

1	(0.414) 0.413 0.416

4	(0.586) 0.587 0.584

Locus 14506 :

2 alleles

99.2% missing data

3	(0.696) 0.695 0.691

1	(0.304) 0.305 0.309

Locus 14507 :

2 alleles

96.9% missing data

4	(0.599) 0.598 0.605

1	(0.401) 0.402 0.395

Locus 14508 :

2 alleles

99.2% missing data

3	(0.508) 0.510 0.506

4	(0.492) 0.490 0.494

Locus 14509 :

2 alleles

98.8% missing data

3	(0.650) 0.647 0.655

1	(0.350) 0.353 0.345

Locus 14510 :

2 alleles

97.7% missing data

2	(0.258) 0.255 0.249

4	(0.742) 0.745 0.751

Locus 14511 :

2 alleles

8.2% missing data

4	(0.981) 0.973 0.994

2	(0.019) 0.027 0.006

Locus 14512 :

2 alleles

94.9% missing data

1	(0.825) 0.825 0.834

3	(0.175) 0.175 0.166

Locus 14513 :

2 alleles

99.2% missing data

1	(0.586) 0.587 0.581

2	(0.414) 0.413 0.419

Locus 14514 :

3 alleles

98.8% missing data

3	(0.588) 0.585 0.586

4	(0.270) 0.266 0.276

2	(0.141) 0.149 0.138

Locus 14515 :

2 alleles

91.4% missing data

1	(0.703) 0.703 0.707

2	(0.297) 0.297 0.293

Locus 14516 :

2 alleles

99.2% missing data

1	(0.215) 0.215 0.217

4	(0.785) 0.785 0.783

Locus 14517 :

2 alleles

99.2% missing data

3	(0.471) 0.461 0.477

2	(0.529) 0.539 0.523

Locus 14518 :

2 alleles

99.2% missing data

2	(0.261) 0.256 0.268

3	(0.739) 0.744 0.732

Locus 14519 :

2 alleles

99.2% missing data

4	(0.564) 0.564 0.564

3	(0.436) 0.436 0.436

Locus 14520 :

2 alleles

99.2% missing data

3	(0.418) 0.418 0.428

1	(0.582) 0.582 0.572

Locus 14521 :

2 alleles

99.2% missing data

4	(0.597) 0.597 0.603

2	(0.403) 0.403 0.397

Locus 14522 :

2 alleles

99.2% missing data

4	(0.513) 0.518 0.506

1	(0.487) 0.482 0.494

Locus 14523 :

2 alleles

99.2% missing data

4	(0.545) 0.549 0.546

3	(0.455) 0.451 0.454

Locus 14524 :

2 alleles

0.0% missing data

1	(0.985) 0.980 0.996

4	(0.015) 0.020 0.004

Locus 14525 :

2 alleles

98.0% missing data

4	(0.784) 0.776 0.794

1	(0.216) 0.224 0.206

Locus 14526 :

2 alleles

99.2% missing data

3	(0.618) 0.621 0.615

2	(0.382) 0.379 0.385

Locus 14527 :

2 alleles

99.2% missing data

2	(0.752) 0.755 0.747

1	(0.248) 0.245 0.253

Locus 14528 :

2 alleles

83.6% missing data

3	(0.939) 0.930 0.954

2	(0.061) 0.070 0.046

Locus 14529 :

2 alleles

96.9% missing data

3	(0.754) 0.747 0.773

4	(0.246) 0.253 0.227

Locus 14530 :

2 alleles

98.4% missing data

2	(0.719) 0.723 0.724

4	(0.281) 0.277 0.276

Locus 14531 :

2 alleles

99.2% missing data

1	(0.608) 0.600 0.609

3	(0.392) 0.400 0.391

Locus 14532 :

2 alleles

98.8% missing data

4	(0.378) 0.375 0.389

1	(0.622) 0.625 0.611

Locus 14533 :

2 alleles

97.7% missing data

1	(0.656) 0.658 0.663

3	(0.344) 0.342 0.337

Locus 14534 :

2 alleles

99.2% missing data

3	(0.615) 0.617 0.617

4	(0.385) 0.383 0.383

Locus 14535 :

2 alleles

98.4% missing data

4	(0.640) 0.638 0.641

2	(0.360) 0.362 0.359

Locus 14536 :

2 alleles

99.2% missing data

2	(0.794) 0.790 0.795

1	(0.206) 0.210 0.205

Locus 14537 :

2 alleles

99.2% missing data

4	(0.484) 0.490 0.480

3	(0.516) 0.510 0.520

Locus 14538 :

2 alleles

82.0% missing data

3	(0.468) 0.538 0.401

4	(0.532) 0.462 0.599

Locus 14539 :

2 alleles

96.5% missing data

1	(0.803) 0.814 0.802

4	(0.197) 0.186 0.198

Locus 14540 :

2 alleles

95.3% missing data

4	(0.447) 0.436 0.448

3	(0.553) 0.564 0.552

Locus 14541 :

2 alleles

98.4% missing data

2	(0.779) 0.784 0.774

1	(0.221) 0.216 0.226

Locus 14542 :

2 alleles

99.2% missing data

4	(0.301) 0.304 0.302

1	(0.699) 0.696 0.698

Locus 14543 :

2 alleles

91.0% missing data

4	(0.898) 0.918 0.889

1	(0.102) 0.082 0.111

Locus 14544 :

2 alleles

99.2% missing data

1	(0.533) 0.527 0.543

4	(0.467) 0.473 0.457

Locus 14545 :

3 alleles

98.8% missing data

4	(0.537) 0.526 0.543

3	(0.184) 0.191 0.183

1	(0.279) 0.283 0.274

Locus 14546 :

2 alleles

99.2% missing data

2	(0.700) 0.707 0.695

4	(0.300) 0.293 0.305

Locus 14547 :

2 alleles

99.2% missing data

2	(0.268) 0.266 0.275

4	(0.732) 0.734 0.725

Locus 14548 :

2 alleles

98.8% missing data

2	(0.449) 0.441 0.453

4	(0.551) 0.559 0.547

Locus 14549 :

2 alleles

99.2% missing data

3	(0.654) 0.650 0.660

1	(0.346) 0.350 0.340

Locus 14550 :

2 alleles

99.2% missing data

1	(0.288) 0.296 0.281

2	(0.712) 0.704 0.719

Locus 14551 :

2 alleles

99.2% missing data

3	(0.821) 0.823 0.816

1	(0.179) 0.177 0.184

Locus 14552 :

2 alleles

98.8% missing data

3	(0.531) 0.527 0.542

2	(0.469) 0.473 0.458

Locus 14553 :

2 alleles

99.2% missing data

2	(0.655) 0.652 0.658

3	(0.345) 0.348 0.342

Locus 14554 :

2 alleles

98.8% missing data

3	(0.810) 0.806 0.816

2	(0.190) 0.194 0.184

Locus 14555 :

2 alleles

93.4% missing data

3	(0.880) 0.881 0.883

1	(0.120) 0.119 0.117

Locus 14556 :

2 alleles

95.3% missing data

2	(0.341) 0.333 0.332

3	(0.659) 0.667 0.668

Locus 14557 :

2 alleles

98.0% missing data

1	(0.755) 0.753 0.761

2	(0.245) 0.247 0.239

Locus 14558 :

2 alleles

97.7% missing data

3	(0.452) 0.457 0.436

1	(0.548) 0.543 0.564

Locus 14559 :

2 alleles

99.2% missing data

2	(0.732) 0.728 0.728

4	(0.268) 0.272 0.272

Locus 14560 :

2 alleles

99.2% missing data

3	(0.267) 0.266 0.270

2	(0.733) 0.734 0.730

Locus 14561 :

2 alleles

98.8% missing data

4	(0.634) 0.632 0.636

3	(0.366) 0.368 0.364

Locus 14562 :

2 alleles

99.2% missing data

3	(0.323) 0.321 0.330

4	(0.677) 0.679 0.670

Locus 14563 :

2 alleles

99.2% missing data

3	(0.481) 0.471 0.487

4	(0.519) 0.529 0.513

Locus 14564 :

2 alleles

99.2% missing data

4	(0.505) 0.510 0.506

1	(0.495) 0.490 0.494

Locus 14565 :

3 alleles

96.1% missing data

1	(0.214) 0.210 0.198

3	(0.424) 0.426 0.431

4	(0.362) 0.364 0.371

Locus 14566 :

2 alleles

99.2% missing data

1	(0.570) 0.562 0.574

2	(0.430) 0.438 0.426

Locus 14567 :

2 alleles

99.2% missing data

3	(0.514) 0.513 0.524

2	(0.486) 0.487 0.476

Locus 14568 :

2 alleles

99.2% missing data

1	(0.632) 0.628 0.637

4	(0.368) 0.372 0.363

Locus 14569 :

2 alleles

99.2% missing data

4	(0.519) 0.514 0.522

2	(0.481) 0.486 0.478

Locus 14570 :

2 alleles

96.5% missing data

2	(0.843) 0.850 0.842

4	(0.157) 0.150 0.158

Locus 14571 :

2 alleles

99.2% missing data

2	(0.715) 0.719 0.713

4	(0.285) 0.281 0.287

Locus 14572 :

2 alleles

98.0% missing data

2	(0.754) 0.763 0.753

3	(0.246) 0.237 0.247

Locus 14573 :

2 alleles

99.2% missing data

3	(0.377) 0.372 0.377

4	(0.623) 0.628 0.623

Locus 14574 :

2 alleles

98.8% missing data

1	(0.217) 0.211 0.221

2	(0.783) 0.789 0.779

Locus 14575 :

2 alleles

98.0% missing data

3	(0.828) 0.836 0.821

1	(0.172) 0.164 0.179

Locus 14576 :

2 alleles

99.2% missing data

4	(0.355) 0.351 0.355

2	(0.645) 0.649 0.645

Locus 14577 :

2 alleles

99.2% missing data

3	(0.455) 0.451 0.458

2	(0.545) 0.549 0.542

Locus 14578 :

2 alleles

90.6% missing data

2	(0.885) 0.905 0.873

4	(0.115) 0.095 0.127

Locus 14579 :

2 alleles

96.1% missing data

1	(0.244) 0.234 0.241

2	(0.756) 0.766 0.759

Locus 14580 :

2 alleles

98.4% missing data

2	(0.806) 0.807 0.809

3	(0.194) 0.193 0.191

Locus 14581 :

2 alleles

95.7% missing data

3	(0.773) 0.789 0.770

4	(0.227) 0.211 0.230

Locus 14582 :

3 alleles

98.8% missing data

4	(0.459) 0.465 0.453

1	(0.242) 0.236 0.244

2	(0.300) 0.299 0.302

Locus 14583 :

2 alleles

99.2% missing data

1	(0.119) 0.116 0.124

4	(0.881) 0.884 0.876

Locus 14584 :

2 alleles

99.2% missing data

4	(0.504) 0.503 0.503

1	(0.496) 0.497 0.497

Locus 14585 :

2 alleles

99.2% missing data

2	(0.188) 0.185 0.191

4	(0.812) 0.815 0.809

Locus 14586 :

2 alleles

99.2% missing data

4	(0.562) 0.556 0.574

2	(0.438) 0.444 0.426

Locus 14587 :

2 alleles

93.0% missing data

4	(0.920) 0.916 0.931

2	(0.080) 0.084 0.069

Locus 14588 :

2 alleles

99.2% missing data

1	(0.593) 0.589 0.592

2	(0.407) 0.411 0.408

Locus 14589 :

2 alleles

86.7% missing data

4	(0.651) 0.712 0.584

2	(0.349) 0.288 0.416

Locus 14590 :

2 alleles

99.2% missing data

2	(0.544) 0.548 0.538

3	(0.456) 0.452 0.462

Locus 14591 :

2 alleles

99.2% missing data

4	(0.368) 0.380 0.366

1	(0.632) 0.620 0.634

Locus 14592 :

2 alleles

99.2% missing data

1	(0.567) 0.565 0.569

4	(0.433) 0.435 0.431

Locus 14593 :

2 alleles

99.2% missing data

2	(0.712) 0.703 0.713

4	(0.288) 0.297 0.287

Locus 14594 :

3 alleles

98.4% missing data

1	(0.315) 0.311 0.326

4	(0.387) 0.385 0.386

2	(0.298) 0.305 0.288

Locus 14595 :

2 alleles

99.2% missing data

2	(0.636) 0.638 0.633

1	(0.364) 0.362 0.367

Locus 14596 :

2 alleles

99.2% missing data

3	(0.528) 0.521 0.528

4	(0.472) 0.479 0.472

Locus 14597 :

2 alleles

99.2% missing data

2	(0.295) 0.299 0.290

1	(0.705) 0.701 0.710

Locus 14598 :

2 alleles

99.2% missing data

3	(0.215) 0.216 0.212

1	(0.785) 0.784 0.788

Locus 14599 :

2 alleles

90.6% missing data

2	(0.920) 0.936 0.909

3	(0.080) 0.064 0.091

Locus 14600 :

2 alleles

96.1% missing data

4	(0.910) 0.918 0.901

1	(0.090) 0.082 0.099

Locus 14601 :

2 alleles

97.7% missing data

2	(0.544) 0.557 0.535

4	(0.456) 0.443 0.465

Locus 14602 :

2 alleles

99.2% missing data

1	(0.518) 0.528 0.515

3	(0.482) 0.472 0.485

Locus 14603 :

2 alleles

99.2% missing data

3	(0.627) 0.628 0.625

1	(0.373) 0.372 0.375

Locus 14604 :

2 alleles

99.2% missing data

3	(0.671) 0.668 0.667

2	(0.329) 0.332 0.333

Locus 14605 :

2 alleles

94.5% missing data

3	(0.582) 0.606 0.563

2	(0.418) 0.394 0.437

Locus 14606 :

3 alleles

98.4% missing data

2	(0.366) 0.365 0.378

4	(0.364) 0.368 0.353

3	(0.270) 0.267 0.269

Locus 14607 :

2 alleles

98.8% missing data

2	(0.210) 0.209 0.214

4	(0.790) 0.791 0.786

Locus 14608 :

2 alleles

95.7% missing data

2	(0.854) 0.868 0.844

3	(0.146) 0.132 0.156

Locus 14609 :

2 alleles

8.2% missing data

2	(0.984) 0.989 0.991

3	(0.016) 0.011 0.009

Locus 14610 :

2 alleles

99.2% missing data

4	(0.560) 0.565 0.555

1	(0.440) 0.435 0.445

Locus 14611 :

2 alleles

99.2% missing data

2	(0.333) 0.333 0.332

1	(0.667) 0.667 0.668

Locus 14612 :

2 alleles

98.8% missing data

3	(0.295) 0.298 0.295

2	(0.705) 0.702 0.705

Locus 14613 :

2 alleles

99.2% missing data

3	(0.658) 0.657 0.655

4	(0.342) 0.343 0.345

Locus 14614 :

2 alleles

95.7% missing data

1	(0.255) 0.242 0.267

3	(0.745) 0.758 0.733

Locus 14615 :

2 alleles

98.0% missing data

4	(0.429) 0.440 0.415

2	(0.571) 0.560 0.585

Locus 14616 :

2 alleles

98.0% missing data

3	(0.776) 0.776 0.777

4	(0.224) 0.224 0.223

Locus 14617 :

2 alleles

99.2% missing data

3	(0.445) 0.438 0.451

4	(0.555) 0.562 0.549

Locus 14618 :

2 alleles

99.2% missing data

2	(0.394) 0.393 0.397

1	(0.606) 0.607 0.603

Locus 14619 :

2 alleles

99.2% missing data

1	(0.594) 0.594 0.586

2	(0.406) 0.406 0.414

Locus 14620 :

2 alleles

94.9% missing data

2	(0.884) 0.889 0.880

3	(0.116) 0.111 0.120

Locus 14621 :

2 alleles

99.2% missing data

1	(0.688) 0.683 0.692

2	(0.312) 0.317 0.308

Locus 14622 :

2 alleles

98.8% missing data

1	(0.773) 0.770 0.766

3	(0.227) 0.230 0.234

Locus 14623 :

2 alleles

99.2% missing data

1	(0.327) 0.332 0.325

2	(0.673) 0.668 0.675

Locus 14624 :

2 alleles

99.2% missing data

4	(0.624) 0.616 0.623

2	(0.376) 0.384 0.377

Locus 14625 :

2 alleles

99.2% missing data

2	(0.367) 0.369 0.367

4	(0.633) 0.631 0.633

Locus 14626 :

2 alleles

99.2% missing data

4	(0.404) 0.407 0.400

2	(0.596) 0.593 0.600

Locus 14627 :

2 alleles

99.2% missing data

2	(0.565) 0.562 0.564

4	(0.435) 0.438 0.436

Locus 14628 :

2 alleles

99.2% missing data

3	(0.571) 0.570 0.565

2	(0.429) 0.430 0.435

Locus 14629 :

2 alleles

99.2% missing data

2	(0.516) 0.510 0.524

3	(0.484) 0.490 0.476

Locus 14630 :

2 alleles

99.2% missing data

3	(0.460) 0.457 0.455

2	(0.540) 0.543 0.545

Locus 14631 :

2 alleles

98.8% missing data

4	(0.737) 0.746 0.736

2	(0.263) 0.254 0.264

Locus 14632 :

2 alleles

0.4% missing data

3	(0.986) 0.990 0.993

2	(0.014) 0.010 0.007

Locus 14633 :

2 alleles

99.2% missing data

3	(0.469) 0.475 0.469

4	(0.531) 0.525 0.531

Locus 14634 :

2 alleles

97.7% missing data

3	(0.234) 0.237 0.230

4	(0.766) 0.763 0.770

Locus 14635 :

2 alleles

99.2% missing data

3	(0.491) 0.486 0.490

2	(0.509) 0.514 0.510

Locus 14636 :

2 alleles

99.2% missing data

4	(0.756) 0.749 0.762

1	(0.244) 0.251 0.238

Locus 14637 :

2 alleles

99.2% missing data

1	(0.401) 0.401 0.402

2	(0.599) 0.599 0.598

Locus 14638 :

2 alleles

99.2% missing data

1	(0.508) 0.508 0.511

4	(0.492) 0.492 0.489

Locus 14639 :

2 alleles

99.2% missing data

4	(0.330) 0.329 0.333

3	(0.670) 0.671 0.667

Locus 14640 :

2 alleles

66.4% missing data

1	(0.974) 0.980 0.977

2	(0.026) 0.020 0.023

Locus 14641 :

2 alleles

99.2% missing data

4	(0.623) 0.617 0.629

3	(0.377) 0.383 0.371

Locus 14642 :

2 alleles

99.2% missing data

2	(0.541) 0.541 0.541

3	(0.459) 0.459 0.459

Locus 14643 :

2 alleles

99.2% missing data

1	(0.307) 0.309 0.312

4	(0.693) 0.691 0.688

Locus 14644 :

2 alleles

99.2% missing data

2	(0.475) 0.474 0.482

4	(0.525) 0.526 0.518

Locus 14645 :

2 alleles

99.2% missing data

2	(0.482) 0.475 0.490

3	(0.518) 0.525 0.510

Locus 14646 :

2 alleles

99.2% missing data

1	(0.333) 0.330 0.336

2	(0.667) 0.670 0.664

Locus 14647 :

2 alleles

93.4% missing data

1	(0.902) 0.905 0.908

4	(0.098) 0.095 0.092

Locus 14648 :

2 alleles

99.2% missing data

4	(0.459) 0.464 0.454

3	(0.541) 0.536 0.546

Locus 14649 :

3 alleles

98.8% missing data

2	(0.252) 0.250 0.254

1	(0.301) 0.298 0.303

4	(0.447) 0.451 0.442

Locus 14650 :

2 alleles

99.2% missing data

3	(0.707) 0.707 0.705

2	(0.293) 0.293 0.295

Locus 14651 :

2 alleles

97.7% missing data

4	(0.686) 0.695 0.671

2	(0.314) 0.305 0.329

Locus 14652 :

3 alleles

92.6% missing data

4	(0.756) 0.752 0.779

3	(0.104) 0.104 0.096

1	(0.140) 0.143 0.125

Locus 14653 :

2 alleles

99.2% missing data

1	(0.604) 0.603 0.602

2	(0.396) 0.397 0.398

Locus 14654 :

2 alleles

99.2% missing data

2	(0.429) 0.423 0.433

3	(0.571) 0.577 0.567

Locus 14655 :

2 alleles

99.2% missing data

4	(0.532) 0.533 0.527

2	(0.468) 0.467 0.473

Locus 14656 :

2 alleles

99.2% missing data

2	(0.264) 0.262 0.266

4	(0.736) 0.738 0.734

Locus 14657 :

2 alleles

99.2% missing data

2	(0.257) 0.256 0.265

4	(0.743) 0.744 0.735

Locus 14658 :

2 alleles

98.0% missing data

2	(0.718) 0.722 0.722

3	(0.282) 0.278 0.278

Locus 14659 :

2 alleles

99.2% missing data

4	(0.248) 0.253 0.247

1	(0.752) 0.747 0.753

Locus 14660 :

2 alleles

99.2% missing data

2	(0.469) 0.470 0.471

4	(0.531) 0.530 0.529

Locus 14661 :

2 alleles

99.2% missing data

2	(0.500) 0.496 0.505

3	(0.500) 0.504 0.495

Locus 14662 :

2 alleles

99.2% missing data

4	(0.445) 0.446 0.440

1	(0.555) 0.554 0.560

Locus 14663 :

2 alleles

98.8% missing data

4	(0.712) 0.701 0.716

3	(0.288) 0.299 0.284

Locus 14664 :

2 alleles

99.2% missing data

4	(0.604) 0.604 0.601

1	(0.396) 0.396 0.399

Locus 14665 :

2 alleles

99.2% missing data

4	(0.589) 0.591 0.597

3	(0.411) 0.409 0.403

Locus 14666 :

2 alleles

99.2% missing data

3	(0.579) 0.581 0.578

2	(0.421) 0.419 0.422

Locus 14667 :

2 alleles

96.9% missing data

1	(0.822) 0.803 0.836

3	(0.178) 0.197 0.164

Locus 14668 :

2 alleles

99.2% missing data

3	(0.463) 0.457 0.472

4	(0.537) 0.543 0.528

Locus 14669 :

2 alleles

99.2% missing data

2	(0.722) 0.721 0.725

4	(0.278) 0.279 0.275

Locus 14670 :

2 alleles

98.8% missing data

1	(0.450) 0.443 0.457

3	(0.550) 0.557 0.543

Locus 14671 :

2 alleles

98.8% missing data

2	(0.612) 0.603 0.615

3	(0.388) 0.397 0.385

Locus 14672 :

2 alleles

99.2% missing data

1	(0.426) 0.423 0.424

2	(0.574) 0.577 0.576

Locus 14673 :

2 alleles

98.8% missing data

4	(0.756) 0.760 0.751

3	(0.244) 0.240 0.249

Locus 14674 :

2 alleles

99.2% missing data

3	(0.481) 0.477 0.486

4	(0.519) 0.523 0.514

Locus 14675 :

2 alleles

99.2% missing data

3	(0.486) 0.488 0.481

2	(0.514) 0.512 0.519

Locus 14676 :

2 alleles

99.2% missing data

3	(0.347) 0.341 0.350

4	(0.653) 0.659 0.650

Locus 14677 :

2 alleles

98.8% missing data

1	(0.565) 0.558 0.573

4	(0.435) 0.442 0.427

Locus 14678 :

2 alleles

99.2% missing data

4	(0.195) 0.190 0.195

3	(0.805) 0.810 0.805

Locus 14679 :

2 alleles

99.2% missing data

1	(0.426) 0.416 0.428

3	(0.574) 0.584 0.572

Locus 14680 :

2 alleles

99.2% missing data

1	(0.581) 0.570 0.588

2	(0.419) 0.430 0.412

Locus 14681 :

2 alleles

99.2% missing data

4	(0.300) 0.304 0.299

3	(0.700) 0.696 0.701

Locus 14682 :

2 alleles

96.1% missing data

4	(0.785) 0.804 0.777

2	(0.215) 0.196 0.223

Locus 14683 :

2 alleles

99.2% missing data

3	(0.636) 0.634 0.636

1	(0.364) 0.366 0.364

Locus 14684 :

2 alleles

99.2% missing data

4	(0.725) 0.729 0.720

1	(0.275) 0.271 0.280

Locus 14685 :

2 alleles

99.2% missing data

4	(0.203) 0.197 0.211

1	(0.797) 0.803 0.789

Locus 14686 :

2 alleles

98.8% missing data

4	(0.641) 0.634 0.649

1	(0.359) 0.366 0.351

Locus 14687 :

2 alleles

99.2% missing data

1	(0.481) 0.488 0.475

3	(0.519) 0.512 0.525

Locus 14688 :

2 alleles

99.2% missing data

4	(0.283) 0.286 0.281

1	(0.717) 0.714 0.719

Locus 14689 :

2 alleles

99.2% missing data

4	(0.433) 0.434 0.432

1	(0.567) 0.566 0.568

Locus 14690 :

2 alleles

99.2% missing data

4	(0.573) 0.576 0.573

3	(0.427) 0.424 0.427

Locus 14691 :

2 alleles

99.2% missing data

3	(0.295) 0.293 0.296

4	(0.705) 0.707 0.704

Locus 14692 :

2 alleles

98.4% missing data

4	(0.618) 0.618 0.623

3	(0.382) 0.382 0.377

Locus 14693 :

2 alleles

99.2% missing data

3	(0.511) 0.515 0.507

2	(0.489) 0.485 0.493

Locus 14694 :

2 alleles

0.0% missing data

4	(0.984) 0.981 0.994

1	(0.016) 0.019 0.006

Locus 14695 :

2 alleles

99.2% missing data

3	(0.309) 0.313 0.314

4	(0.691) 0.687 0.686

Locus 14696 :

2 alleles

99.2% missing data

2	(0.169) 0.166 0.176

1	(0.831) 0.834 0.824

Locus 14697 :

2 alleles

99.2% missing data

3	(0.483) 0.478 0.486

2	(0.517) 0.522 0.514

Locus 14698 :

2 alleles

99.2% missing data

4	(0.572) 0.567 0.581

3	(0.428) 0.433 0.419

Locus 14699 :

2 alleles

98.0% missing data

3	(0.748) 0.740 0.758

1	(0.252) 0.260 0.242

Locus 14700 :

2 alleles

99.2% missing data

4	(0.406) 0.400 0.409

2	(0.594) 0.600 0.591

Locus 14701 :

2 alleles

99.2% missing data

2	(0.377) 0.378 0.386

4	(0.623) 0.622 0.614

Locus 14702 :

2 alleles

98.8% missing data

3	(0.645) 0.639 0.650

2	(0.355) 0.361 0.350

Locus 14703 :

2 alleles

99.2% missing data

1	(0.562) 0.559 0.559

4	(0.438) 0.441 0.441

Locus 14704 :

3 alleles

63.3% missing data

4	(0.948) 0.945 0.970

3	(0.028) 0.029 0.016

2	(0.024) 0.026 0.014

Locus 14705 :

2 alleles

0.0% missing data

3	(0.986) 0.982 0.996

2	(0.014) 0.018 0.004

Locus 14706 :

2 alleles

99.2% missing data

2	(0.629) 0.625 0.621

1	(0.371) 0.375 0.379

Locus 14707 :

2 alleles

99.2% missing data

2	(0.279) 0.279 0.283

3	(0.721) 0.721 0.717

Locus 14708 :

2 alleles

99.2% missing data

1	(0.485) 0.482 0.491

2	(0.515) 0.518 0.509

Locus 14709 :

2 alleles

99.2% missing data

2	(0.338) 0.337 0.340

4	(0.662) 0.663 0.660

Locus 14710 :

2 alleles

99.2% missing data

3	(0.672) 0.669 0.680

1	(0.328) 0.331 0.320

Locus 14711 :

2 alleles

97.3% missing data

2	(0.685) 0.682 0.703

4	(0.315) 0.318 0.297

Locus 14712 :

2 alleles

98.8% missing data

4	(0.591) 0.587 0.602

1	(0.409) 0.413 0.398

Locus 14713 :

2 alleles

98.4% missing data

4	(0.463) 0.470 0.463

1	(0.537) 0.530 0.537

Locus 14714 :

2 alleles

99.2% missing data

2	(0.488) 0.488 0.483

3	(0.512) 0.512 0.517

Locus 14715 :

2 alleles

99.2% missing data

4	(0.386) 0.386 0.387

1	(0.614) 0.614 0.613

Locus 14716 :

2 alleles

99.2% missing data

3	(0.693) 0.689 0.693

4	(0.307) 0.311 0.307

Locus 14717 :

2 alleles

99.2% missing data

1	(0.476) 0.483 0.472

4	(0.524) 0.517 0.528

Locus 14718 :

2 alleles

99.2% missing data

2	(0.816) 0.818 0.811

1	(0.184) 0.182 0.189

Locus 14719 :

2 alleles

98.8% missing data

1	(0.598) 0.604 0.594

4	(0.402) 0.396 0.406

Locus 14720 :

2 alleles

99.2% missing data

2	(0.316) 0.314 0.318

3	(0.684) 0.686 0.682

Locus 14721 :

2 alleles

99.2% missing data

3	(0.527) 0.527 0.533

1	(0.473) 0.473 0.467

Locus 14722 :

3 alleles

92.6% missing data

3	(0.802) 0.791 0.819

4	(0.092) 0.091 0.086

2	(0.107) 0.118 0.095

Locus 14723 :

2 alleles

99.2% missing data

3	(0.415) 0.413 0.419

2	(0.585) 0.587 0.581

Locus 14724 :

2 alleles

99.2% missing data

3	(0.487) 0.487 0.481

1	(0.513) 0.513 0.519

Locus 14725 :

2 alleles

98.8% missing data

3	(0.774) 0.776 0.765

1	(0.226) 0.224 0.235

Locus 14726 :

2 alleles

98.8% missing data

2	(0.519) 0.518 0.527

3	(0.481) 0.482 0.473

Locus 14727 :

3 alleles

98.8% missing data

4	(0.387) 0.378 0.396

1	(0.347) 0.350 0.340

2	(0.265) 0.271 0.264

Locus 14728 :

3 alleles

98.4% missing data

1	(0.658) 0.649 0.656

3	(0.119) 0.128 0.114

4	(0.223) 0.222 0.230

Locus 14729 :

2 alleles

98.4% missing data

4	(0.813) 0.818 0.816

3	(0.187) 0.182 0.184

Locus 14730 :

2 alleles

0.8% missing data

4	(0.975) 0.980 0.983

3	(0.025) 0.020 0.017

Locus 14731 :

2 alleles

98.8% missing data

1	(0.427) 0.420 0.437

4	(0.573) 0.580 0.563

Locus 14732 :

2 alleles

96.9% missing data

3	(0.801) 0.814 0.794

4	(0.199) 0.186 0.206

Locus 14733 :

2 alleles

95.3% missing data

4	(0.480) 0.513 0.458

1	(0.520) 0.487 0.542

Locus 14734 :

2 alleles

99.2% missing data

3	(0.772) 0.770 0.773

2	(0.228) 0.230 0.227

Locus 14735 :

2 alleles

97.3% missing data

3	(0.824) 0.825 0.827

1	(0.176) 0.175 0.173

Locus 14736 :

2 alleles

98.8% missing data

3	(0.121) 0.119 0.124

4	(0.879) 0.881 0.876

Locus 14737 :

2 alleles

98.8% missing data

2	(0.647) 0.644 0.653

1	(0.353) 0.356 0.347

Locus 14738 :

4 alleles

98.0% missing data

3	(0.348) 0.346 0.355

2	(0.175) 0.176 0.179

4	(0.309) 0.309 0.299

1	(0.168) 0.169 0.167

Locus 14739 :

2 alleles

99.2% missing data

2	(0.328) 0.323 0.334

3	(0.672) 0.677 0.666

Locus 14740 :

2 alleles

99.2% missing data

4	(0.581) 0.585 0.579

3	(0.419) 0.415 0.421

Locus 14741 :

2 alleles

99.2% missing data

3	(0.534) 0.539 0.529

4	(0.466) 0.461 0.471

Locus 14742 :

2 alleles

99.2% missing data

1	(0.735) 0.732 0.732

3	(0.265) 0.268 0.268

Locus 14743 :

2 alleles

99.2% missing data

3	(0.186) 0.185 0.191

4	(0.814) 0.815 0.809

Locus 14744 :

2 alleles

97.7% missing data

3	(0.813) 0.811 0.819

1	(0.187) 0.189 0.181

Locus 14745 :

2 alleles

99.2% missing data

4	(0.492) 0.489 0.493

2	(0.508) 0.511 0.507

Locus 14746 :

2 alleles

99.2% missing data

3	(0.303) 0.297 0.309

4	(0.697) 0.703 0.691

Locus 14747 :

2 alleles

99.2% missing data

4	(0.734) 0.738 0.730

3	(0.266) 0.262 0.270

Locus 14748 :

2 alleles

99.2% missing data

2	(0.518) 0.520 0.523

3	(0.482) 0.480 0.477

Locus 14749 :

2 alleles

99.2% missing data

4	(0.691) 0.689 0.692

3	(0.309) 0.311 0.308

Locus 14750 :

2 alleles

99.2% missing data

4	(0.471) 0.477 0.468

1	(0.529) 0.523 0.532

Locus 14751 :

2 alleles

98.8% missing data

2	(0.550) 0.550 0.559

4	(0.450) 0.450 0.441

Locus 14752 :

2 alleles

98.8% missing data

2	(0.346) 0.349 0.343

4	(0.654) 0.651 0.657

Locus 14753 :

2 alleles

99.2% missing data

1	(0.626) 0.623 0.628

4	(0.374) 0.377 0.372

Locus 14754 :

2 alleles

98.8% missing data

3	(0.695) 0.699 0.700

4	(0.305) 0.301 0.300

Locus 14755 :

3 alleles

98.8% missing data

3	(0.387) 0.377 0.389

4	(0.272) 0.278 0.266

1	(0.341) 0.345 0.344

Locus 14756 :

2 alleles

99.2% missing data

1	(0.758) 0.762 0.756

2	(0.242) 0.238 0.244

Locus 14757 :

2 alleles

99.2% missing data

1	(0.584) 0.587 0.579

2	(0.416) 0.413 0.421

Locus 14758 :

2 alleles

97.7% missing data

3	(0.346) 0.329 0.351

1	(0.654) 0.671 0.649

Locus 14759 :

2 alleles

99.2% missing data

3	(0.736) 0.738 0.732

2	(0.264) 0.262 0.268

Locus 14760 :

2 alleles

98.8% missing data

3	(0.630) 0.631 0.635

2	(0.370) 0.369 0.365

Locus 14761 :

2 alleles

99.2% missing data

3	(0.209) 0.206 0.217

2	(0.791) 0.794 0.783

Locus 14762 :

2 alleles

97.3% missing data

4	(0.301) 0.311 0.276

3	(0.699) 0.689 0.724

Locus 14763 :

2 alleles

99.2% missing data

1	(0.537) 0.528 0.539

4	(0.463) 0.472 0.461

Locus 14764 :

2 alleles

99.2% missing data

3	(0.342) 0.334 0.342

4	(0.658) 0.666 0.658

Locus 14765 :

2 alleles

98.4% missing data

4	(0.645) 0.641 0.656

1	(0.355) 0.359 0.344

Locus 14766 :

2 alleles

99.2% missing data

2	(0.740) 0.734 0.740

3	(0.260) 0.266 0.260

Locus 14767 :

2 alleles

96.1% missing data

2	(0.175) 0.167 0.180

1	(0.825) 0.833 0.820

Locus 14768 :

2 alleles

99.2% missing data

4	(0.656) 0.658 0.654

3	(0.344) 0.342 0.346

Locus 14769 :

2 alleles

99.2% missing data

3	(0.635) 0.631 0.634

1	(0.365) 0.369 0.366

Locus 14770 :

2 alleles

99.2% missing data

1	(0.709) 0.702 0.710

4	(0.291) 0.298 0.290

Locus 14771 :

2 alleles

99.2% missing data

1	(0.539) 0.543 0.535

3	(0.461) 0.457 0.465

Locus 14772 :

2 alleles

96.1% missing data

2	(0.286) 0.266 0.282

1	(0.714) 0.734 0.718

Locus 14773 :

2 alleles

99.2% missing data

3	(0.513) 0.507 0.519

4	(0.487) 0.493 0.481

Locus 14774 :

2 alleles

99.2% missing data

3	(0.734) 0.731 0.741

4	(0.266) 0.269 0.259

Locus 14775 :

2 alleles

99.2% missing data

1	(0.577) 0.577 0.579

4	(0.423) 0.423 0.421

Locus 14776 :

2 alleles

99.2% missing data

3	(0.274) 0.279 0.271

2	(0.726) 0.721 0.729

Locus 14777 :

2 alleles

98.4% missing data

1	(0.805) 0.802 0.808

2	(0.195) 0.198 0.192

Locus 14778 :

2 alleles

97.7% missing data

3	(0.883) 0.877 0.888

2	(0.117) 0.123 0.112

Locus 14779 :

2 alleles

99.2% missing data

2	(0.628) 0.628 0.624

4	(0.372) 0.372 0.376

Locus 14780 :

2 alleles

99.2% missing data

4	(0.459) 0.461 0.454

3	(0.541) 0.539 0.546

Locus 14781 :

2 alleles

98.8% missing data

2	(0.604) 0.603 0.600

3	(0.396) 0.397 0.400

Locus 14782 :

2 alleles

94.9% missing data

1	(0.085) 0.090 0.081

3	(0.915) 0.910 0.919

Locus 14783 :

2 alleles

99.2% missing data

4	(0.526) 0.514 0.533

1	(0.474) 0.486 0.467

Locus 14784 :

2 alleles

92.6% missing data

3	(0.935) 0.934 0.936

1	(0.065) 0.066 0.064

Locus 14785 :

2 alleles

99.2% missing data

2	(0.587) 0.584 0.579

4	(0.413) 0.416 0.421

Locus 14786 :

2 alleles

99.2% missing data

3	(0.271) 0.262 0.276

1	(0.729) 0.738 0.724

Locus 14787 :

2 alleles

99.2% missing data

3	(0.704) 0.704 0.696

1	(0.296) 0.296 0.304

Locus 14788 :

2 alleles

97.7% missing data

3	(0.816) 0.815 0.823

1	(0.184) 0.185 0.177

Locus 14789 :

2 alleles

99.2% missing data

2	(0.369) 0.368 0.367

3	(0.631) 0.632 0.633

Locus 14790 :

2 alleles

99.2% missing data

3	(0.267) 0.276 0.259

2	(0.733) 0.724 0.741

Locus 14791 :

2 alleles

99.2% missing data

4	(0.463) 0.463 0.469

2	(0.537) 0.537 0.531

Locus 14792 :

2 alleles

96.5% missing data

1	(0.706) 0.713 0.703

2	(0.294) 0.287 0.297

Locus 14793 :

2 alleles

99.2% missing data

2	(0.336) 0.333 0.335

4	(0.664) 0.667 0.665

Locus 14794 :

2 alleles

99.2% missing data

4	(0.504) 0.497 0.506

1	(0.496) 0.503 0.494

Locus 14795 :

2 alleles

98.8% missing data

2	(0.575) 0.576 0.579

1	(0.425) 0.424 0.421

Locus 14796 :

2 alleles

99.2% missing data

1	(0.351) 0.346 0.359

4	(0.649) 0.654 0.641

Locus 14797 :

3 alleles

98.4% missing data

2	(0.305) 0.295 0.312

3	(0.303) 0.302 0.304

4	(0.392) 0.403 0.385

Locus 14798 :

3 alleles

98.8% missing data

3	(0.283) 0.287 0.284

1	(0.122) 0.118 0.130

2	(0.595) 0.595 0.587

Locus 14799 :

2 alleles

99.2% missing data

4	(0.565) 0.561 0.572

3	(0.435) 0.439 0.428

Locus 14800 :

2 alleles

98.8% missing data

1	(0.681) 0.682 0.674

3	(0.319) 0.318 0.326

Locus 14801 :

2 alleles

98.8% missing data

4	(0.706) 0.702 0.713

2	(0.294) 0.298 0.287

Locus 14802 :

2 alleles

99.2% missing data

1	(0.661) 0.664 0.659

2	(0.339) 0.336 0.341

Locus 14803 :

2 alleles

99.2% missing data

2	(0.555) 0.551 0.555

4	(0.445) 0.449 0.445

Locus 14804 :

2 alleles

99.2% missing data

1	(0.559) 0.561 0.555

2	(0.441) 0.439 0.445

Locus 14805 :

2 alleles

99.2% missing data

2	(0.606) 0.610 0.600

3	(0.394) 0.390 0.400

Locus 14806 :

2 alleles

99.2% missing data

2	(0.300) 0.300 0.307

1	(0.700) 0.700 0.693

Locus 14807 :

2 alleles

99.2% missing data

2	(0.556) 0.555 0.554

3	(0.444) 0.445 0.446

Locus 14808 :

2 alleles

99.2% missing data

4	(0.300) 0.294 0.308

3	(0.700) 0.706 0.692

Locus 14809 :

2 alleles

99.2% missing data

2	(0.365) 0.366 0.369

1	(0.635) 0.634 0.631

Locus 14810 :

2 alleles

99.2% missing data

2	(0.484) 0.482 0.483

4	(0.516) 0.518 0.517

Locus 14811 :

2 alleles

99.2% missing data

4	(0.455) 0.449 0.456

3	(0.545) 0.551 0.544

Locus 14812 :

2 alleles

99.2% missing data

2	(0.847) 0.844 0.849

1	(0.153) 0.156 0.151

Locus 14813 :

2 alleles

99.2% missing data

4	(0.459) 0.453 0.464

1	(0.541) 0.547 0.536

Locus 14814 :

2 alleles

99.2% missing data

2	(0.189) 0.189 0.189

4	(0.811) 0.811 0.811

Locus 14815 :

2 alleles

98.8% missing data

3	(0.271) 0.265 0.270

1	(0.729) 0.735 0.730

Locus 14816 :

2 alleles

99.2% missing data

3	(0.453) 0.451 0.451

1	(0.547) 0.549 0.549

Locus 14817 :

2 alleles

99.2% missing data

4	(0.514) 0.514 0.514

1	(0.486) 0.486 0.486

Locus 14818 :

2 alleles

98.8% missing data

3	(0.387) 0.386 0.385

4	(0.613) 0.614 0.615

Locus 14819 :

2 alleles

99.2% missing data

3	(0.273) 0.272 0.279

2	(0.727) 0.728 0.721

Locus 14820 :

2 alleles

98.4% missing data

3	(0.615) 0.610 0.609

2	(0.385) 0.390 0.391

Locus 14821 :

2 alleles

99.2% missing data

3	(0.251) 0.251 0.252

1	(0.749) 0.749 0.748

Locus 14822 :

2 alleles

96.5% missing data

4	(0.147) 0.136 0.154

3	(0.853) 0.864 0.846

Locus 14823 :

2 alleles

99.2% missing data

3	(0.504) 0.509 0.502

4	(0.496) 0.491 0.498

Locus 14824 :

2 alleles

98.4% missing data

3	(0.565) 0.565 0.573

1	(0.435) 0.435 0.427

Locus 14825 :

2 alleles

99.2% missing data

2	(0.496) 0.490 0.502

4	(0.504) 0.510 0.498

Locus 14826 :

2 alleles

99.2% missing data

1	(0.455) 0.447 0.467

2	(0.545) 0.553 0.533

Locus 14827 :

2 alleles

99.2% missing data

3	(0.702) 0.698 0.699

2	(0.298) 0.302 0.301

Locus 14828 :

2 alleles

99.2% missing data

1	(0.460) 0.457 0.467

2	(0.540) 0.543 0.533

Locus 14829 :

2 alleles

98.4% missing data

3	(0.389) 0.397 0.385

2	(0.611) 0.603 0.615

Locus 14830 :

2 alleles

99.2% missing data

4	(0.547) 0.551 0.539

3	(0.453) 0.449 0.461

Locus 14831 :

2 alleles

99.2% missing data

1	(0.564) 0.570 0.564

2	(0.436) 0.430 0.436

Locus 14832 :

2 alleles

99.2% missing data

2	(0.533) 0.530 0.541

3	(0.467) 0.470 0.459

Locus 14833 :

2 alleles

99.2% missing data

4	(0.397) 0.397 0.404

2	(0.603) 0.603 0.596

Locus 14834 :

2 alleles

98.4% missing data

4	(0.556) 0.559 0.560

2	(0.444) 0.441 0.440

Locus 14835 :

2 alleles

99.2% missing data

4	(0.719) 0.717 0.721

1	(0.281) 0.283 0.279

Locus 14836 :

2 alleles

99.2% missing data

3	(0.604) 0.597 0.607

4	(0.396) 0.403 0.393

Locus 14837 :

2 alleles

98.8% missing data

2	(0.345) 0.348 0.339

4	(0.655) 0.652 0.661

Locus 14838 :

2 alleles

98.4% missing data

2	(0.416) 0.403 0.419

1	(0.584) 0.597 0.581

Locus 14839 :

2 alleles

98.0% missing data

4	(0.733) 0.722 0.742

3	(0.267) 0.278 0.258

Locus 14840 :

2 alleles

99.2% missing data

3	(0.207) 0.202 0.214

1	(0.793) 0.798 0.786

Locus 14841 :

2 alleles

99.2% missing data

2	(0.771) 0.773 0.766

1	(0.229) 0.227 0.234

Locus 14842 :

2 alleles

99.2% missing data

1	(0.336) 0.335 0.342

3	(0.664) 0.665 0.658

Locus 14843 :

2 alleles

99.2% missing data

3	(0.667) 0.676 0.657

4	(0.333) 0.324 0.343

Locus 14844 :

2 alleles

99.2% missing data

2	(0.522) 0.521 0.521

3	(0.478) 0.479 0.479

Locus 14845 :

2 alleles

99.2% missing data

2	(0.512) 0.507 0.516

1	(0.488) 0.493 0.484

Locus 14846 :

2 alleles

98.8% missing data

4	(0.549) 0.554 0.548

3	(0.451) 0.446 0.452

Locus 14847 :

2 alleles

99.2% missing data

1	(0.442) 0.431 0.445

4	(0.558) 0.569 0.555

Locus 14848 :

2 alleles

99.2% missing data

3	(0.292) 0.287 0.301

2	(0.708) 0.713 0.699

Locus 14849 :

2 alleles

92.2% missing data

3	(0.104) 0.110 0.090

1	(0.896) 0.890 0.910

Locus 14850 :

2 alleles

99.2% missing data

1	(0.643) 0.646 0.641

4	(0.357) 0.354 0.359

Locus 14851 :

2 alleles

91.0% missing data

2	(0.931) 0.945 0.921

4	(0.069) 0.055 0.079

Locus 14852 :

2 alleles

98.8% missing data

3	(0.615) 0.603 0.622

2	(0.385) 0.397 0.378

Locus 14853 :

2 alleles

90.2% missing data

3	(0.919) 0.922 0.924

1	(0.081) 0.078 0.076

Locus 14854 :

2 alleles

99.2% missing data

3	(0.734) 0.729 0.732

4	(0.266) 0.271 0.268

Locus 14855 :

2 alleles

99.2% missing data

1	(0.595) 0.597 0.586

2	(0.405) 0.403 0.414

Locus 14856 :

2 alleles

98.0% missing data

3	(0.629) 0.622 0.645

2	(0.371) 0.378 0.355

Locus 14857 :

2 alleles

98.8% missing data

2	(0.752) 0.748 0.750

3	(0.248) 0.252 0.250

Locus 14858 :

2 alleles

99.2% missing data

2	(0.483) 0.485 0.484

4	(0.517) 0.515 0.516

Locus 14859 :

2 alleles

99.2% missing data

2	(0.710) 0.712 0.705

3	(0.290) 0.288 0.295

Locus 14860 :

2 alleles

99.2% missing data

1	(0.604) 0.603 0.606

2	(0.396) 0.397 0.394

Locus 14861 :

2 alleles

99.2% missing data

2	(0.247) 0.247 0.247

3	(0.753) 0.753 0.753

Locus 14862 :

2 alleles

99.2% missing data

2	(0.250) 0.250 0.253

4	(0.750) 0.750 0.747

Locus 14863 :

2 alleles

99.2% missing data

3	(0.772) 0.767 0.777

4	(0.228) 0.233 0.223

Locus 14864 :

2 alleles

99.2% missing data

3	(0.695) 0.690 0.695

1	(0.305) 0.310 0.305

Locus 14865 :

2 alleles

99.2% missing data

2	(0.245) 0.245 0.245

3	(0.755) 0.755 0.755

Locus 14866 :

3 alleles

98.4% missing data

2	(0.234) 0.235 0.235

1	(0.442) 0.453 0.433

3	(0.324) 0.312 0.332

Locus 14867 :

2 alleles

97.7% missing data

3	(0.180) 0.169 0.185

1	(0.820) 0.831 0.815

Locus 14868 :

2 alleles

99.2% missing data

4	(0.450) 0.448 0.449

3	(0.550) 0.552 0.551

Locus 14869 :

2 alleles

99.2% missing data

2	(0.384) 0.384 0.376

1	(0.616) 0.616 0.624

Locus 14870 :

2 alleles

99.2% missing data

1	(0.487) 0.484 0.490

2	(0.513) 0.516 0.510

Locus 14871 :

2 alleles

99.2% missing data

3	(0.474) 0.475 0.477

2	(0.526) 0.525 0.523

Locus 14872 :

2 alleles

99.2% missing data

2	(0.599) 0.596 0.595

1	(0.401) 0.404 0.405

Locus 14873 :

2 alleles

99.2% missing data

2	(0.493) 0.486 0.498

1	(0.507) 0.514 0.502

Locus 14874 :

2 alleles

99.2% missing data

4	(0.275) 0.275 0.283

3	(0.725) 0.725 0.717

Locus 14875 :

2 alleles

96.5% missing data

1	(0.183) 0.173 0.186

4	(0.817) 0.827 0.814

Locus 14876 :

2 alleles

99.2% missing data

2	(0.662) 0.659 0.665

1	(0.338) 0.341 0.335

Locus 14877 :

2 alleles

99.2% missing data

4	(0.673) 0.679 0.671

3	(0.327) 0.321 0.329

Locus 14878 :

2 alleles

99.2% missing data

2	(0.174) 0.170 0.181

1	(0.826) 0.830 0.819

Locus 14879 :

2 alleles

91.4% missing data

4	(0.903) 0.909 0.910

3	(0.097) 0.091 0.090

Locus 14880 :

2 alleles

99.2% missing data

3	(0.773) 0.766 0.775

4	(0.227) 0.234 0.225

Locus 14881 :

2 alleles

96.9% missing data

3	(0.814) 0.805 0.819

1	(0.186) 0.195 0.181

Locus 14882 :

2 alleles

98.8% missing data

3	(0.766) 0.768 0.758

2	(0.234) 0.232 0.242

Locus 14883 :

2 alleles

98.4% missing data

3	(0.689) 0.684 0.697

4	(0.311) 0.316 0.303

Locus 14884 :

2 alleles

99.2% missing data

4	(0.552) 0.553 0.556

3	(0.448) 0.447 0.444

Locus 14885 :

2 alleles

99.2% missing data

2	(0.829) 0.825 0.831

4	(0.171) 0.175 0.169

Locus 14886 :

2 alleles

99.2% missing data

3	(0.504) 0.500 0.499

2	(0.496) 0.500 0.501

Locus 14887 :

3 alleles

98.8% missing data

1	(0.292) 0.287 0.293

3	(0.331) 0.344 0.327

2	(0.377) 0.369 0.380

Locus 14888 :

2 alleles

99.2% missing data

1	(0.387) 0.384 0.393

3	(0.613) 0.616 0.607

Locus 14889 :

2 alleles

99.2% missing data

3	(0.297) 0.299 0.293

2	(0.703) 0.701 0.707

Locus 14890 :

2 alleles

99.2% missing data

4	(0.496) 0.504 0.488

3	(0.504) 0.496 0.512

Locus 14891 :

2 alleles

99.2% missing data

2	(0.653) 0.650 0.648

1	(0.347) 0.350 0.352

Locus 14892 :

2 alleles

98.8% missing data

2	(0.217) 0.214 0.223

3	(0.783) 0.786 0.777

Locus 14893 :

2 alleles

99.2% missing data

2	(0.301) 0.296 0.303

3	(0.699) 0.704 0.697

Locus 14894 :

2 alleles

99.2% missing data

2	(0.667) 0.676 0.667

4	(0.333) 0.324 0.333

Locus 14895 :

2 alleles

99.2% missing data

4	(0.277) 0.279 0.280

1	(0.723) 0.721 0.720

Locus 14896 :

2 alleles

99.2% missing data

4	(0.485) 0.478 0.488

1	(0.515) 0.522 0.512

Locus 14897 :

2 alleles

98.8% missing data

4	(0.410) 0.404 0.411

2	(0.590) 0.596 0.589

Locus 14898 :

2 alleles

98.8% missing data

3	(0.443) 0.442 0.445

2	(0.557) 0.558 0.555

Locus 14899 :

2 alleles

98.8% missing data

3	(0.477) 0.483 0.482

4	(0.523) 0.517 0.518

Locus 14900 :

2 alleles

96.1% missing data

4	(0.529) 0.541 0.522

1	(0.471) 0.459 0.478

Locus 14901 :

2 alleles

99.2% missing data

4	(0.425) 0.421 0.424

1	(0.575) 0.579 0.576

Locus 14902 :

2 alleles

99.2% missing data

3	(0.589) 0.596 0.586

2	(0.411) 0.404 0.414

Locus 14903 :

3 alleles

98.8% missing data

4	(0.278) 0.288 0.279

3	(0.324) 0.321 0.323

2	(0.398) 0.391 0.397

Locus 14904 :

2 alleles

98.8% missing data

4	(0.575) 0.571 0.576

2	(0.425) 0.429 0.424

Locus 14905 :

2 alleles

98.8% missing data

3	(0.438) 0.437 0.437

2	(0.562) 0.563 0.563

Locus 14906 :

2 alleles

97.7% missing data

2	(0.417) 0.415 0.406

3	(0.583) 0.585 0.594

Locus 14907 :

2 alleles

98.8% missing data

1	(0.751) 0.752 0.749

4	(0.249) 0.248 0.251

Locus 14908 :

2 alleles

98.8% missing data

4	(0.559) 0.566 0.554

1	(0.441) 0.434 0.446

Locus 14909 :

2 alleles

98.4% missing data

2	(0.603) 0.609 0.598

1	(0.397) 0.391 0.402

Locus 14910 :

2 alleles

99.2% missing data

4	(0.330) 0.324 0.332

1	(0.670) 0.676 0.668

Locus 14911 :

3 alleles

94.5% missing data

4	(0.667) 0.670 0.676

1	(0.135) 0.132 0.129

2	(0.199) 0.198 0.195

Locus 14912 :

2 alleles

99.2% missing data

1	(0.660) 0.658 0.656

2	(0.340) 0.342 0.344

Locus 14913 :

2 alleles

99.2% missing data

1	(0.367) 0.373 0.361

2	(0.633) 0.627 0.639

Locus 14914 :

2 alleles

98.8% missing data

4	(0.456) 0.448 0.459

1	(0.544) 0.552 0.541

Locus 14915 :

2 alleles

98.8% missing data

2	(0.680) 0.679 0.690

4	(0.320) 0.321 0.310

Locus 14916 :

2 alleles

98.8% missing data

4	(0.427) 0.419 0.436

1	(0.573) 0.581 0.564

Locus 14917 :

2 alleles

98.8% missing data

1	(0.651) 0.657 0.648

2	(0.349) 0.343 0.352

Locus 14918 :

2 alleles

99.2% missing data

1	(0.627) 0.629 0.618

4	(0.373) 0.371 0.382

Locus 14919 :

2 alleles

99.2% missing data

1	(0.700) 0.696 0.704

4	(0.300) 0.304 0.296

Locus 14920 :

2 alleles

97.7% missing data

2	(0.618) 0.636 0.611

4	(0.382) 0.364 0.389

Locus 14921 :

3 alleles

98.8% missing data

1	(0.204) 0.209 0.195

3	(0.497) 0.500 0.500

2	(0.299) 0.291 0.305

Locus 14922 :

2 alleles

91.0% missing data

3	(0.910) 0.925 0.903

2	(0.090) 0.075 0.097

Locus 14923 :

2 alleles

99.2% missing data

2	(0.419) 0.419 0.408

4	(0.581) 0.581 0.592

Locus 14924 :

2 alleles

99.2% missing data

4	(0.229) 0.228 0.232

2	(0.771) 0.772 0.768

Locus 14925 :

2 alleles

99.2% missing data

1	(0.431) 0.437 0.428

2	(0.569) 0.563 0.572

Locus 14926 :

2 alleles

97.7% missing data

1	(0.475) 0.484 0.460

2	(0.525) 0.516 0.540

Locus 14927 :

2 alleles

99.2% missing data

2	(0.386) 0.389 0.386

3	(0.614) 0.611 0.614

Locus 14928 :

2 alleles

99.2% missing data

1	(0.567) 0.561 0.570

4	(0.433) 0.439 0.430

Locus 14929 :

2 alleles

99.2% missing data

1	(0.483) 0.481 0.484

2	(0.517) 0.519 0.516

Locus 14930 :

2 alleles

89.8% missing data

3	(0.104) 0.083 0.122

4	(0.896) 0.917 0.878

Locus 14931 :

2 alleles

98.8% missing data

4	(0.737) 0.743 0.740

2	(0.263) 0.257 0.260

Locus 14932 :

2 alleles

98.8% missing data

3	(0.522) 0.517 0.519

2	(0.478) 0.483 0.481

Locus 14933 :

2 alleles

99.2% missing data

4	(0.294) 0.299 0.293

3	(0.706) 0.701 0.707

Locus 14934 :

2 alleles

99.2% missing data

3	(0.534) 0.534 0.531

4	(0.466) 0.466 0.469

Locus 14935 :

2 alleles

99.2% missing data

4	(0.481) 0.476 0.488

1	(0.519) 0.524 0.512

Locus 14936 :

2 alleles

99.2% missing data

1	(0.355) 0.345 0.362

4	(0.645) 0.655 0.638

Locus 14937 :

2 alleles

98.4% missing data

2	(0.669) 0.661 0.675

4	(0.331) 0.339 0.325

Locus 14938 :

2 alleles

99.2% missing data

1	(0.467) 0.466 0.465

4	(0.533) 0.534 0.535

Locus 14939 :

2 alleles

98.4% missing data

3	(0.617) 0.616 0.625

4	(0.383) 0.384 0.375

Locus 14940 :

2 alleles

99.2% missing data

1	(0.534) 0.531 0.537

4	(0.466) 0.469 0.463

Locus 14941 :

2 alleles

99.2% missing data

1	(0.412) 0.405 0.420

3	(0.588) 0.595 0.580

Locus 14942 :

2 alleles

99.2% missing data

3	(0.314) 0.314 0.313

1	(0.686) 0.686 0.687

Locus 14943 :

2 alleles

95.3% missing data

2	(0.866) 0.879 0.860

3	(0.134) 0.121 0.140

Locus 14944 :

2 alleles

99.2% missing data

1	(0.622) 0.616 0.631

2	(0.378) 0.384 0.369

Locus 14945 :

2 alleles

98.8% missing data

4	(0.429) 0.420 0.439

2	(0.571) 0.580 0.561

Locus 14946 :

2 alleles

99.2% missing data

2	(0.428) 0.435 0.423

3	(0.572) 0.565 0.577

Locus 14947 :

2 alleles

98.8% missing data

1	(0.470) 0.476 0.478

2	(0.530) 0.524 0.522

Locus 14948 :

2 alleles

98.8% missing data

3	(0.433) 0.433 0.434

1	(0.567) 0.567 0.566

Locus 14949 :

3 alleles

98.8% missing data

2	(0.506) 0.501 0.512

1	(0.298) 0.310 0.287

4	(0.196) 0.190 0.201

Locus 14950 :

2 alleles

99.2% missing data

3	(0.247) 0.246 0.253

4	(0.753) 0.754 0.747

Locus 14951 :

2 alleles

98.4% missing data

3	(0.576) 0.572 0.586

4	(0.424) 0.428 0.414

Locus 14952 :

2 alleles

97.7% missing data

4	(0.888) 0.890 0.887

2	(0.112) 0.110 0.113

Locus 14953 :

2 alleles

98.8% missing data

3	(0.623) 0.627 0.619

2	(0.377) 0.373 0.381

Locus 14954 :

2 alleles

99.2% missing data

3	(0.573) 0.574 0.569

4	(0.427) 0.426 0.431

Locus 14955 :

2 alleles

99.2% missing data

2	(0.235) 0.240 0.236

4	(0.765) 0.760 0.764

Locus 14956 :

2 alleles

97.3% missing data

3	(0.411) 0.410 0.402

1	(0.589) 0.590 0.598

Locus 14957 :

2 alleles

83.2% missing data

2	(0.493) 0.559 0.432

4	(0.507) 0.441 0.568

Locus 14958 :

2 alleles

99.2% missing data

2	(0.506) 0.502 0.511

4	(0.494) 0.498 0.489

Locus 14959 :

2 alleles

96.9% missing data

3	(0.719) 0.721 0.715

2	(0.281) 0.279 0.285

Locus 14960 :

2 alleles

99.2% missing data

2	(0.287) 0.289 0.279

1	(0.713) 0.711 0.721

Locus 14961 :

2 alleles

99.2% missing data

2	(0.769) 0.774 0.767

4	(0.231) 0.226 0.233

Locus 14962 :

3 alleles

98.8% missing data

1	(0.271) 0.274 0.265

2	(0.413) 0.412 0.407

3	(0.317) 0.314 0.328

Locus 14963 :

3 alleles

98.8% missing data

2	(0.249) 0.250 0.253

1	(0.290) 0.285 0.291

4	(0.461) 0.466 0.456

Locus 14964 :

2 alleles

90.2% missing data

3	(0.925) 0.941 0.916

1	(0.075) 0.059 0.084

Locus 14965 :

2 alleles

99.2% missing data

2	(0.619) 0.617 0.620

3	(0.381) 0.383 0.380

Locus 14966 :

2 alleles

99.2% missing data

4	(0.502) 0.498 0.503

3	(0.498) 0.502 0.497

Locus 14967 :

2 alleles

99.2% missing data

3	(0.545) 0.549 0.542

2	(0.455) 0.451 0.458

Locus 14968 :

2 alleles

97.7% missing data

4	(0.262) 0.268 0.255

3	(0.738) 0.732 0.745

Locus 14969 :

2 alleles

95.7% missing data

4	(0.366) 0.366 0.358

1	(0.634) 0.634 0.642

Locus 14970 :

2 alleles

0.0% missing data

4	(0.986) 0.983 0.996

1	(0.014) 0.017 0.004

Locus 14971 :

2 alleles

98.0% missing data

3	(0.615) 0.608 0.625

1	(0.385) 0.392 0.375

Locus 14972 :

2 alleles

99.2% missing data

3	(0.665) 0.669 0.653

4	(0.335) 0.331 0.347

Locus 14973 :

2 alleles

98.8% missing data

3	(0.446) 0.440 0.460

2	(0.554) 0.560 0.540

Locus 14974 :

2 alleles

26.6% missing data

3	(0.985) 0.987 0.990

1	(0.015) 0.013 0.010

Locus 14975 :

2 alleles

99.2% missing data

1	(0.277) 0.274 0.282

2	(0.723) 0.726 0.718

Locus 14976 :

3 alleles

98.8% missing data

2	(0.379) 0.379 0.379

4	(0.225) 0.230 0.220

3	(0.396) 0.391 0.401

Locus 14977 :

2 alleles

99.2% missing data

1	(0.311) 0.305 0.309

3	(0.689) 0.695 0.691

Locus 14978 :

2 alleles

99.2% missing data

3	(0.326) 0.324 0.330

2	(0.674) 0.676 0.670

Locus 14979 :

2 alleles

97.7% missing data

1	(0.640) 0.660 0.635

2	(0.360) 0.340 0.365

Locus 14980 :

2 alleles

99.2% missing data

2	(0.414) 0.416 0.414

3	(0.586) 0.584 0.586

Locus 14981 :

2 alleles

98.8% missing data

3	(0.607) 0.604 0.613

2	(0.393) 0.396 0.387

Locus 14982 :

2 alleles

98.0% missing data

3	(0.227) 0.217 0.236

2	(0.773) 0.783 0.764

Locus 14983 :

2 alleles

99.2% missing data

4	(0.609) 0.608 0.604

2	(0.391) 0.392 0.396

Locus 14984 :

2 alleles

99.2% missing data

3	(0.704) 0.701 0.698

2	(0.296) 0.299 0.302

Locus 14985 :

2 alleles

97.7% missing data

1	(0.748) 0.755 0.749

3	(0.252) 0.245 0.251

Locus 14986 :

2 alleles

99.2% missing data

3	(0.500) 0.495 0.500

1	(0.500) 0.505 0.500

Locus 14987 :

2 alleles

98.8% missing data

1	(0.574) 0.566 0.581

3	(0.426) 0.434 0.419

Locus 14988 :

2 alleles

98.8% missing data

3	(0.681) 0.686 0.680

4	(0.319) 0.314 0.320

Locus 14989 :

2 alleles

99.2% missing data

3	(0.437) 0.437 0.442

1	(0.563) 0.563 0.558

Locus 14990 :

2 alleles

96.1% missing data

2	(0.120) 0.109 0.124

3	(0.880) 0.891 0.876

Locus 14991 :

2 alleles

99.2% missing data

1	(0.471) 0.462 0.478

2	(0.529) 0.538 0.522

Locus 14992 :

2 alleles

98.4% missing data

1	(0.664) 0.662 0.674

3	(0.336) 0.338 0.326

Locus 14993 :

2 alleles

99.2% missing data

3	(0.520) 0.529 0.515

1	(0.480) 0.471 0.485

Locus 14994 :

2 alleles

99.2% missing data

3	(0.627) 0.628 0.622

1	(0.373) 0.372 0.378

Locus 14995 :

3 alleles

98.8% missing data

4	(0.658) 0.659 0.658

1	(0.148) 0.146 0.150

3	(0.194) 0.195 0.192

Locus 14996 :

2 alleles

99.2% missing data

1	(0.486) 0.490 0.489

3	(0.514) 0.510 0.511

Locus 14997 :

2 alleles

99.2% missing data

4	(0.813) 0.813 0.811

3	(0.187) 0.187 0.189

Locus 14998 :

2 alleles

99.2% missing data

3	(0.299) 0.303 0.296

2	(0.701) 0.697 0.704

Locus 14999 :

2 alleles

96.1% missing data

3	(0.823) 0.824 0.826

2	(0.177) 0.176 0.174

Locus 15000 :

2 alleles

99.2% missing data

1	(0.569) 0.562 0.567

4	(0.431) 0.438 0.433

Locus 15001 :

2 alleles

99.2% missing data

2	(0.686) 0.688 0.683

1	(0.314) 0.312 0.317

Locus 15002 :

2 alleles

99.2% missing data

2	(0.204) 0.201 0.204

1	(0.796) 0.799 0.796

Locus 15003 :

2 alleles

99.2% missing data

1	(0.433) 0.432 0.438

4	(0.567) 0.568 0.562

Locus 15004 :

2 alleles

96.5% missing data

1	(0.874) 0.880 0.871

3	(0.126) 0.120 0.129

Locus 15005 :

4 alleles

98.4% missing data

2	(0.148) 0.145 0.150

1	(0.357) 0.354 0.362

4	(0.390) 0.392 0.383

3	(0.105) 0.110 0.105

Locus 15006 :

2 alleles

99.2% missing data

3	(0.291) 0.297 0.288

4	(0.709) 0.703 0.712

Locus 15007 :

2 alleles

99.2% missing data

2	(0.654) 0.652 0.655

3	(0.346) 0.348 0.345

Locus 15008 :

2 alleles

99.2% missing data

4	(0.570) 0.563 0.581

1	(0.430) 0.437 0.419

Locus 15009 :

2 alleles

99.2% missing data

2	(0.385) 0.387 0.387

1	(0.615) 0.613 0.613

Locus 15010 :

2 alleles

99.2% missing data

4	(0.395) 0.398 0.394

2	(0.605) 0.602 0.606

Locus 15011 :

2 alleles

99.2% missing data

1	(0.393) 0.388 0.399

4	(0.607) 0.612 0.601

Locus 15012 :

2 alleles

98.4% missing data

2	(0.600) 0.603 0.597

3	(0.400) 0.397 0.403

Locus 15013 :

2 alleles

99.2% missing data

1	(0.522) 0.522 0.524

2	(0.478) 0.478 0.476

Locus 15014 :

2 alleles

99.2% missing data

1	(0.592) 0.592 0.591

3	(0.408) 0.408 0.409

Locus 15015 :

2 alleles

99.2% missing data

3	(0.401) 0.398 0.404

1	(0.599) 0.602 0.596

Locus 15016 :

2 alleles

98.8% missing data

2	(0.417) 0.414 0.432

1	(0.583) 0.586 0.568

Locus 15017 :

2 alleles

99.2% missing data

3	(0.541) 0.535 0.546

1	(0.459) 0.465 0.454

Locus 15018 :

2 alleles

99.2% missing data

3	(0.439) 0.442 0.442

1	(0.561) 0.558 0.558

Locus 15019 :

2 alleles

99.2% missing data

1	(0.340) 0.327 0.353

3	(0.660) 0.673 0.647

Locus 15020 :

2 alleles

99.2% missing data

1	(0.609) 0.601 0.620

2	(0.391) 0.399 0.380

Locus 15021 :

2 alleles

98.4% missing data

3	(0.556) 0.554 0.567

4	(0.444) 0.446 0.433

Locus 15022 :

2 alleles

99.2% missing data

3	(0.682) 0.675 0.683

2	(0.318) 0.325 0.317

Locus 15023 :

2 alleles

99.2% missing data

1	(0.546) 0.547 0.542

2	(0.454) 0.453 0.458

Locus 15024 :

2 alleles

99.2% missing data

3	(0.278) 0.273 0.284

1	(0.722) 0.727 0.716

Locus 15025 :

2 alleles

99.2% missing data

1	(0.708) 0.707 0.710

2	(0.292) 0.293 0.290

Locus 15026 :

2 alleles

99.2% missing data

1	(0.540) 0.545 0.542

3	(0.460) 0.455 0.458

Locus 15027 :

2 alleles

99.2% missing data

1	(0.554) 0.548 0.560

3	(0.446) 0.452 0.440

Locus 15028 :

2 alleles

98.8% missing data

1	(0.796) 0.798 0.790

2	(0.204) 0.202 0.210

Locus 15029 :

2 alleles

98.0% missing data

1	(0.738) 0.739 0.736

2	(0.262) 0.261 0.264

Locus 15030 :

2 alleles

99.2% missing data

1	(0.509) 0.503 0.513

3	(0.491) 0.497 0.487

Locus 15031 :

2 alleles

99.2% missing data

1	(0.476) 0.480 0.472

3	(0.524) 0.520 0.528

Locus 15032 :

2 alleles

99.2% missing data

1	(0.625) 0.619 0.627

2	(0.375) 0.381 0.373

Locus 15033 :

2 alleles

98.8% missing data

1	(0.727) 0.722 0.733

3	(0.273) 0.278 0.267

Locus 15034 :

2 alleles

98.8% missing data

3	(0.796) 0.797 0.790

4	(0.204) 0.203 0.210

Locus 15035 :

2 alleles

99.2% missing data

3	(0.597) 0.596 0.605

2	(0.403) 0.404 0.395

Locus 15036 :

2 alleles

99.2% missing data

3	(0.420) 0.415 0.428

2	(0.580) 0.585 0.572

Locus 15037 :

2 alleles

99.2% missing data

3	(0.548) 0.542 0.548

1	(0.452) 0.458 0.452

Locus 15038 :

2 alleles

99.2% missing data

3	(0.363) 0.362 0.369

4	(0.637) 0.638 0.631

Locus 15039 :

2 alleles

98.8% missing data

1	(0.269) 0.264 0.269

2	(0.731) 0.736 0.731

Locus 15040 :

2 alleles

98.4% missing data

3	(0.691) 0.682 0.700

2	(0.309) 0.318 0.300

Locus 15041 :

2 alleles

99.2% missing data

4	(0.396) 0.396 0.397

2	(0.604) 0.604 0.603

Locus 15042 :

2 alleles

99.2% missing data

1	(0.617) 0.617 0.613

4	(0.383) 0.383 0.387

Locus 15043 :

2 alleles

99.2% missing data

4	(0.464) 0.463 0.470

1	(0.536) 0.537 0.530

Locus 15044 :

2 alleles

93.0% missing data

1	(0.882) 0.886 0.891

4	(0.118) 0.114 0.109

Locus 15045 :

2 alleles

96.1% missing data

3	(0.151) 0.141 0.157

1	(0.849) 0.859 0.843

Locus 15046 :

2 alleles

99.2% missing data

1	(0.493) 0.487 0.499

3	(0.507) 0.513 0.501

Locus 15047 :

2 alleles

91.0% missing data

2	(0.926) 0.936 0.920

1	(0.074) 0.064 0.080

Locus 15048 :

2 alleles

99.2% missing data

1	(0.430) 0.432 0.431

2	(0.570) 0.568 0.569

Locus 15049 :

2 alleles

99.2% missing data

1	(0.343) 0.340 0.344

2	(0.657) 0.660 0.656

Locus 15050 :

2 alleles

98.8% missing data

3	(0.524) 0.521 0.522

2	(0.476) 0.479 0.478

Locus 15051 :

2 alleles

99.2% missing data

1	(0.496) 0.502 0.494

4	(0.504) 0.498 0.506

Locus 15052 :

2 alleles

99.2% missing data

4	(0.667) 0.660 0.671

2	(0.333) 0.340 0.329

Locus 15053 :

2 alleles

99.2% missing data

1	(0.693) 0.687 0.688

3	(0.307) 0.313 0.312

Locus 15054 :

2 alleles

98.8% missing data

3	(0.357) 0.359 0.353

4	(0.643) 0.641 0.647

Locus 15055 :

2 alleles

99.2% missing data

1	(0.765) 0.772 0.763

3	(0.235) 0.228 0.237

Locus 15056 :

3 alleles

90.2% missing data

2	(0.775) 0.816 0.764

3	(0.120) 0.101 0.124

1	(0.104) 0.083 0.112

Locus 15057 :

2 alleles

99.2% missing data

1	(0.419) 0.420 0.419

4	(0.581) 0.580 0.581

Locus 15058 :

2 alleles

0.0% missing data

2	(0.987) 0.990 0.993

3	(0.013) 0.010 0.007

Locus 15059 :

2 alleles

99.2% missing data

1	(0.393) 0.389 0.398

2	(0.607) 0.611 0.602

Locus 15060 :

2 alleles

99.2% missing data

2	(0.489) 0.485 0.491

3	(0.511) 0.515 0.509

Locus 15061 :

2 alleles

99.2% missing data

4	(0.547) 0.556 0.540

1	(0.453) 0.444 0.460

Locus 15062 :

2 alleles

99.2% missing data

2	(0.680) 0.673 0.683

4	(0.320) 0.327 0.317

Locus 15063 :

2 alleles

99.2% missing data

1	(0.590) 0.587 0.593

2	(0.410) 0.413 0.407

Locus 15064 :

2 alleles

99.2% missing data

3	(0.321) 0.323 0.317

4	(0.679) 0.677 0.683

Locus 15065 :

2 alleles

97.3% missing data

1	(0.434) 0.433 0.442

2	(0.566) 0.567 0.558

Locus 15066 :

2 alleles

91.0% missing data

1	(0.870) 0.883 0.868

4	(0.130) 0.117 0.132

Locus 15067 :

2 alleles

99.2% missing data

1	(0.297) 0.294 0.301

4	(0.703) 0.706 0.699

Locus 15068 :

2 alleles

99.2% missing data

2	(0.649) 0.656 0.643

4	(0.351) 0.344 0.357

Locus 15069 :

2 alleles

0.0% missing data

3	(0.987) 0.982 0.996

4	(0.013) 0.018 0.004

Locus 15070 :

2 alleles

98.8% missing data

4	(0.532) 0.538 0.529

1	(0.468) 0.462 0.471

Locus 15071 :

2 alleles

97.7% missing data

4	(0.269) 0.271 0.263

1	(0.731) 0.729 0.737

Locus 15072 :

2 alleles

99.2% missing data

4	(0.591) 0.591 0.591

3	(0.409) 0.409 0.409

Locus 15073 :

2 alleles

98.8% missing data

3	(0.450) 0.446 0.464

1	(0.550) 0.554 0.536

Locus 15074 :

2 alleles

99.2% missing data

1	(0.817) 0.823 0.814

2	(0.183) 0.177 0.186

Locus 15075 :

2 alleles

99.2% missing data

3	(0.598) 0.599 0.592

1	(0.402) 0.401 0.408

Locus 15076 :

2 alleles

99.2% missing data

4	(0.637) 0.642 0.635

3	(0.363) 0.358 0.365

Locus 15077 :

2 alleles

99.2% missing data

1	(0.404) 0.405 0.406

3	(0.596) 0.595 0.594

Locus 15078 :

2 alleles

98.8% missing data

4	(0.451) 0.445 0.455

3	(0.549) 0.555 0.545

Locus 15079 :

2 alleles

99.2% missing data

2	(0.553) 0.556 0.547

4	(0.447) 0.444 0.453

Locus 15080 :

2 alleles

98.4% missing data

2	(0.618) 0.626 0.617

1	(0.382) 0.374 0.383

Locus 15081 :

2 alleles

99.2% missing data

3	(0.287) 0.288 0.284

4	(0.713) 0.712 0.716

Locus 15082 :

2 alleles

91.0% missing data

4	(0.888) 0.908 0.880

3	(0.112) 0.092 0.120

Locus 15083 :

2 alleles

98.4% missing data

4	(0.588) 0.602 0.589

1	(0.412) 0.398 0.411

Locus 15084 :

2 alleles

99.2% missing data

3	(0.515) 0.509 0.520

2	(0.485) 0.491 0.480

Locus 15085 :

2 alleles

99.2% missing data

3	(0.489) 0.479 0.492

4	(0.511) 0.521 0.508

Locus 15086 :

2 alleles

99.2% missing data

4	(0.544) 0.550 0.542

1	(0.456) 0.450 0.458

Locus 15087 :

2 alleles

99.2% missing data

2	(0.495) 0.493 0.502

1	(0.505) 0.507 0.498

Locus 15088 :

2 alleles

99.2% missing data

1	(0.429) 0.433 0.430

4	(0.571) 0.567 0.570

Locus 15089 :

2 alleles

99.2% missing data

3	(0.188) 0.187 0.188

2	(0.812) 0.813 0.812

Locus 15090 :

2 alleles

99.2% missing data

2	(0.447) 0.446 0.443

4	(0.553) 0.554 0.557

Locus 15091 :

2 alleles

99.2% missing data

1	(0.752) 0.749 0.753

4	(0.248) 0.251 0.247

Locus 15092 :

2 alleles

99.2% missing data

4	(0.648) 0.651 0.644

3	(0.352) 0.349 0.356

Locus 15093 :

2 alleles

99.2% missing data

4	(0.412) 0.408 0.415

3	(0.588) 0.592 0.585

Locus 15094 :

2 alleles

99.2% missing data

1	(0.662) 0.657 0.666

4	(0.338) 0.343 0.334

Locus 15095 :

2 alleles

99.2% missing data

3	(0.446) 0.448 0.448

4	(0.554) 0.552 0.552

Locus 15096 :

3 alleles

95.7% missing data

3	(0.154) 0.143 0.160

1	(0.160) 0.155 0.157

2	(0.686) 0.703 0.682

Locus 15097 :

2 alleles

99.2% missing data

1	(0.589) 0.590 0.581

4	(0.411) 0.410 0.419

Locus 15098 :

2 alleles

99.2% missing data

1	(0.535) 0.531 0.538

2	(0.465) 0.469 0.462

Locus 15099 :

2 alleles

99.2% missing data

3	(0.255) 0.260 0.252

1	(0.745) 0.740 0.748

Locus 15100 :

2 alleles

99.2% missing data

2	(0.214) 0.216 0.214

3	(0.786) 0.784 0.786

Locus 15101 :

2 alleles

99.2% missing data

2	(0.518) 0.517 0.521

3	(0.482) 0.483 0.479

Locus 15102 :

2 alleles

99.2% missing data

1	(0.354) 0.355 0.354

2	(0.646) 0.645 0.646

Locus 15103 :

2 alleles

99.2% missing data

1	(0.228) 0.227 0.230

2	(0.772) 0.773 0.770

Locus 15104 :

2 alleles

99.2% missing data

2	(0.404) 0.403 0.410

3	(0.596) 0.597 0.590

Locus 15105 :

2 alleles

99.2% missing data

1	(0.281) 0.285 0.280

2	(0.719) 0.715 0.720

Locus 15106 :

2 alleles

99.2% missing data

1	(0.195) 0.191 0.197

2	(0.805) 0.809 0.803

Locus 15107 :

2 alleles

98.8% missing data

3	(0.626) 0.631 0.630

2	(0.374) 0.369 0.370

Locus 15108 :

2 alleles

99.2% missing data

4	(0.476) 0.476 0.479

3	(0.524) 0.524 0.521

Locus 15109 :

2 alleles

99.2% missing data

1	(0.211) 0.217 0.205

3	(0.789) 0.783 0.795

Locus 15110 :

2 alleles

91.4% missing data

1	(0.911) 0.928 0.904

3	(0.089) 0.072 0.096

Locus 15111 :

2 alleles

99.2% missing data

1	(0.717) 0.715 0.721

4	(0.283) 0.285 0.279

Locus 15112 :

2 alleles

99.2% missing data

2	(0.418) 0.410 0.427

3	(0.582) 0.590 0.573

Locus 15113 :

2 alleles

99.2% missing data

4	(0.667) 0.666 0.667

1	(0.333) 0.334 0.333

Locus 15114 :

2 alleles

99.2% missing data

3	(0.462) 0.465 0.457

2	(0.538) 0.535 0.543

Locus 15115 :

2 alleles

96.5% missing data

1	(0.144) 0.140 0.143

2	(0.856) 0.860 0.857

Locus 15116 :

2 alleles

99.2% missing data

2	(0.588) 0.588 0.591

1	(0.412) 0.412 0.409

Locus 15117 :

2 alleles

97.3% missing data

2	(0.787) 0.801 0.779

4	(0.213) 0.199 0.221

Locus 15118 :

2 alleles

99.2% missing data

1	(0.460) 0.454 0.463

2	(0.540) 0.546 0.537

Locus 15119 :

2 alleles

99.2% missing data

2	(0.616) 0.617 0.612

3	(0.384) 0.383 0.388

Locus 15120 :

2 alleles

91.0% missing data

1	(0.924) 0.936 0.918

2	(0.076) 0.064 0.082

Locus 15121 :

2 alleles

99.2% missing data

1	(0.273) 0.278 0.270

4	(0.727) 0.722 0.730

Locus 15122 :

2 alleles

99.2% missing data

3	(0.408) 0.404 0.411

1	(0.592) 0.596 0.589

Locus 15123 :

2 alleles

99.2% missing data

3	(0.343) 0.338 0.351

1	(0.657) 0.662 0.649

Locus 15124 :

2 alleles

91.0% missing data

1	(0.907) 0.927 0.901

2	(0.093) 0.073 0.099

Locus 15125 :

2 alleles

97.3% missing data

2	(0.642) 0.634 0.660

4	(0.358) 0.366 0.340

Locus 15126 :

2 alleles

98.4% missing data

2	(0.706) 0.703 0.707

1	(0.294) 0.297 0.293

Locus 15127 :

2 alleles

99.2% missing data

2	(0.637) 0.640 0.640

4	(0.363) 0.360 0.360

Locus 15128 :

2 alleles

89.8% missing data

2	(0.930) 0.938 0.933

3	(0.070) 0.062 0.067

Locus 15129 :

2 alleles

99.2% missing data

4	(0.400) 0.401 0.396

1	(0.600) 0.599 0.604

Locus 15130 :

2 alleles

99.2% missing data

4	(0.317) 0.314 0.319

2	(0.683) 0.686 0.681

Locus 15131 :

2 alleles

99.2% missing data

2	(0.696) 0.695 0.697

1	(0.304) 0.305 0.303

Locus 15132 :

2 alleles

99.2% missing data

2	(0.283) 0.286 0.281

4	(0.717) 0.714 0.719

Locus 15133 :

2 alleles

8.2% missing data

2	(0.989) 0.983 0.997

4	(0.011) 0.017 0.003

Locus 15134 :

2 alleles

96.1% missing data

1	(0.328) 0.302 0.345

2	(0.672) 0.698 0.655

Locus 15135 :

2 alleles

99.2% missing data

4	(0.685) 0.690 0.686

2	(0.315) 0.310 0.314

Locus 15136 :

2 alleles

98.8% missing data

1	(0.543) 0.541 0.544

2	(0.457) 0.459 0.456

Locus 15137 :

2 alleles

99.2% missing data

1	(0.694) 0.696 0.692

2	(0.306) 0.304 0.308

Locus 15138 :

3 alleles

98.8% missing data

2	(0.330) 0.327 0.331

4	(0.360) 0.364 0.357

3	(0.310) 0.310 0.312

Locus 15139 :

2 alleles

99.2% missing data

4	(0.447) 0.449 0.446

2	(0.553) 0.551 0.554

Locus 15140 :

2 alleles

99.2% missing data

4	(0.140) 0.145 0.135

1	(0.860) 0.855 0.865

Locus 15141 :

2 alleles

99.2% missing data

2	(0.434) 0.433 0.439

4	(0.566) 0.567 0.561

Locus 15142 :

2 alleles

8.6% missing data

4	(0.984) 0.976 0.995

2	(0.016) 0.024 0.005

Locus 15143 :

2 alleles

99.2% missing data

1	(0.611) 0.608 0.619

4	(0.389) 0.392 0.381

Locus 15144 :

2 alleles

98.8% missing data

4	(0.610) 0.607 0.609

1	(0.390) 0.393 0.391

Locus 15145 :

2 alleles

99.2% missing data

1	(0.703) 0.705 0.704

3	(0.297) 0.295 0.296

Locus 15146 :

2 alleles

99.2% missing data

3	(0.544) 0.547 0.543

4	(0.456) 0.453 0.457

Locus 15147 :

2 alleles

99.2% missing data

4	(0.381) 0.380 0.384

1	(0.619) 0.620 0.616

Locus 15148 :

2 alleles

99.2% missing data

1	(0.710) 0.715 0.712

4	(0.290) 0.285 0.288

Locus 15149 :

2 alleles

97.3% missing data

2	(0.783) 0.784 0.790

4	(0.217) 0.216 0.210

Locus 15150 :

2 alleles

8.2% missing data

2	(0.987) 0.979 0.996

4	(0.013) 0.021 0.004

Locus 15151 :

2 alleles

99.2% missing data

4	(0.312) 0.308 0.318

1	(0.688) 0.692 0.682

Locus 15152 :

2 alleles

99.2% missing data

1	(0.677) 0.684 0.674

3	(0.323) 0.316 0.326

Locus 15153 :

2 alleles

98.8% missing data

1	(0.501) 0.506 0.500

3	(0.499) 0.494 0.500

Locus 15154 :

2 alleles

85.9% missing data

3	(0.616) 0.692 0.543

1	(0.384) 0.308 0.457

Locus 15155 :

2 alleles

98.8% missing data

4	(0.689) 0.694 0.683

3	(0.311) 0.306 0.317

Locus 15156 :

2 alleles

99.2% missing data

4	(0.573) 0.564 0.578

1	(0.427) 0.436 0.422

Locus 15157 :

3 alleles

98.4% missing data

4	(0.436) 0.437 0.445

2	(0.217) 0.214 0.218

3	(0.347) 0.350 0.337

Locus 15158 :

2 alleles

99.2% missing data

3	(0.486) 0.480 0.492

1	(0.514) 0.520 0.508

Locus 15159 :

2 alleles

98.0% missing data

4	(0.741) 0.742 0.739

2	(0.259) 0.258 0.261

Locus 15160 :

2 alleles

8.2% missing data

2	(0.982) 0.985 0.991

4	(0.018) 0.015 0.009

Locus 15161 :

2 alleles

96.1% missing data

2	(0.133) 0.122 0.141

3	(0.867) 0.878 0.859

Locus 15162 :

2 alleles

99.2% missing data

2	(0.577) 0.584 0.577

4	(0.423) 0.416 0.423

Locus 15163 :

2 alleles

99.2% missing data

1	(0.632) 0.635 0.632

2	(0.368) 0.365 0.368

Locus 15164 :

2 alleles

99.2% missing data

4	(0.486) 0.492 0.487

1	(0.514) 0.508 0.513

Locus 15165 :

2 alleles

99.2% missing data

3	(0.335) 0.332 0.339

4	(0.665) 0.668 0.661

Locus 15166 :

2 alleles

99.2% missing data

3	(0.483) 0.481 0.485

2	(0.517) 0.519 0.515

Locus 15167 :

2 alleles

98.8% missing data

3	(0.591) 0.590 0.591

2	(0.409) 0.410 0.409

Locus 15168 :

2 alleles

99.2% missing data

1	(0.338) 0.337 0.340

3	(0.662) 0.663 0.660

Locus 15169 :

2 alleles

92.2% missing data

3	(0.928) 0.932 0.930

4	(0.072) 0.068 0.070

Locus 15170 :

2 alleles

99.2% missing data

4	(0.701) 0.702 0.697

2	(0.299) 0.298 0.303

Locus 15171 :

2 alleles

99.2% missing data

2	(0.455) 0.455 0.462

1	(0.545) 0.545 0.538

Locus 15172 :

2 alleles

98.8% missing data

3	(0.578) 0.577 0.581

4	(0.422) 0.423 0.419

Locus 15173 :

2 alleles

99.2% missing data

4	(0.527) 0.518 0.534

1	(0.473) 0.482 0.466

Locus 15174 :

2 alleles

99.2% missing data

1	(0.445) 0.443 0.449

2	(0.555) 0.557 0.551

Locus 15175 :

2 alleles

98.8% missing data

3	(0.641) 0.631 0.648

1	(0.359) 0.369 0.352

Locus 15176 :

2 alleles

99.2% missing data

4	(0.575) 0.577 0.575

3	(0.425) 0.423 0.425

Locus 15177 :

2 alleles

99.2% missing data

3	(0.670) 0.671 0.665

2	(0.330) 0.329 0.335

Locus 15178 :

2 alleles

96.1% missing data

1	(0.123) 0.113 0.128

4	(0.877) 0.887 0.872

Locus 15179 :

2 alleles

92.6% missing data

4	(0.901) 0.900 0.909

1	(0.099) 0.100 0.091

Locus 15180 :

2 alleles

97.7% missing data

2	(0.281) 0.277 0.274

3	(0.719) 0.723 0.726

Locus 15181 :

2 alleles

99.2% missing data

3	(0.251) 0.254 0.245

1	(0.749) 0.746 0.755

Locus 15182 :

2 alleles

99.2% missing data

1	(0.497) 0.493 0.505

4	(0.503) 0.507 0.495

Locus 15183 :

2 alleles

99.2% missing data

1	(0.553) 0.553 0.549

3	(0.447) 0.447 0.451

Locus 15184 :

2 alleles

1.6% missing data

3	(0.987) 0.985 0.995

1	(0.013) 0.015 0.005

Locus 15185 :

2 alleles

99.2% missing data

2	(0.275) 0.272 0.278

3	(0.725) 0.728 0.722

Locus 15186 :

2 alleles

8.2% missing data

3	(0.966) 0.933 0.989

4	(0.034) 0.067 0.011

Locus 15187 :

2 alleles

99.2% missing data

3	(0.374) 0.376 0.376

2	(0.626) 0.624 0.624

Locus 15188 :

3 alleles

98.8% missing data

3	(0.289) 0.288 0.294

4	(0.346) 0.349 0.346

2	(0.365) 0.363 0.360

Locus 15189 :

2 alleles

99.2% missing data

2	(0.417) 0.420 0.417

3	(0.583) 0.580 0.583

Locus 15190 :

2 alleles

96.1% missing data

4	(0.190) 0.175 0.197

1	(0.810) 0.825 0.803

Locus 15191 :

2 alleles

99.2% missing data

4	(0.686) 0.679 0.682

1	(0.314) 0.321 0.318

Locus 15192 :

2 alleles

99.2% missing data

4	(0.479) 0.483 0.474

1	(0.521) 0.517 0.526

Locus 15193 :

2 alleles

99.2% missing data

3	(0.431) 0.432 0.431

4	(0.569) 0.568 0.569

Locus 15194 :

2 alleles

91.0% missing data

3	(0.879) 0.901 0.871

4	(0.121) 0.099 0.129

Locus 15195 :

2 alleles

97.3% missing data

2	(0.740) 0.734 0.752

3	(0.260) 0.266 0.248

Locus 15196 :

2 alleles

8.2% missing data

3	(0.987) 0.980 0.996

1	(0.013) 0.020 0.004

Locus 15197 :

2 alleles

99.2% missing data

3	(0.662) 0.661 0.662

4	(0.338) 0.339 0.338

Locus 15198 :

3 alleles

98.8% missing data

1	(0.262) 0.252 0.264

4	(0.536) 0.547 0.529

2	(0.202) 0.201 0.207

Locus 15199 :

2 alleles

98.0% missing data

3	(0.699) 0.697 0.704

1	(0.301) 0.303 0.296

Locus 15200 :

2 alleles

98.4% missing data

3	(0.618) 0.620 0.611

2	(0.382) 0.380 0.389

Locus 15201 :

2 alleles

99.2% missing data

3	(0.405) 0.399 0.415

4	(0.595) 0.601 0.585

Locus 15202 :

2 alleles

99.2% missing data

2	(0.268) 0.270 0.268

3	(0.732) 0.730 0.732

Locus 15203 :

2 alleles

99.2% missing data

3	(0.630) 0.630 0.624

2	(0.370) 0.370 0.376

Locus 15204 :

2 alleles

99.2% missing data

2	(0.580) 0.580 0.580

1	(0.420) 0.420 0.420

Locus 15205 :

2 alleles

99.2% missing data

3	(0.326) 0.317 0.330

1	(0.674) 0.683 0.670

Locus 15206 :

2 alleles

95.3% missing data

4	(0.558) 0.562 0.559

3	(0.442) 0.438 0.441

Locus 15207 :

2 alleles

0.0% missing data

1	(0.984) 0.980 0.995

2	(0.016) 0.020 0.005

Locus 15208 :

2 alleles

99.2% missing data

3	(0.778) 0.781 0.772

2	(0.222) 0.219 0.228

Locus 15209 :

2 alleles

99.2% missing data

3	(0.647) 0.641 0.657

1	(0.353) 0.359 0.343

Locus 15210 :

2 alleles

90.6% missing data

4	(0.896) 0.916 0.889

3	(0.104) 0.084 0.111

Locus 15211 :

2 alleles

98.8% missing data

3	(0.672) 0.672 0.674

4	(0.328) 0.328 0.326

Locus 15212 :

2 alleles

98.8% missing data

2	(0.504) 0.502 0.508

3	(0.496) 0.498 0.492

Locus 15213 :

2 alleles

99.2% missing data

1	(0.312) 0.317 0.313

2	(0.688) 0.683 0.687

Locus 15214 :

2 alleles

0.4% missing data

3	(0.979) 0.962 0.994

2	(0.021) 0.038 0.006

Locus 15215 :

2 alleles

98.4% missing data

1	(0.590) 0.599 0.586

3	(0.410) 0.401 0.414

Locus 15216 :

2 alleles

0.0% missing data

2	(0.987) 0.990 0.992

1	(0.013) 0.010 0.008

Locus 15217 :

2 alleles

99.2% missing data

2	(0.329) 0.327 0.333

4	(0.671) 0.673 0.667

Locus 15218 :

2 alleles

99.2% missing data

4	(0.689) 0.688 0.687

3	(0.311) 0.312 0.313

Locus 15219 :

2 alleles

99.2% missing data

1	(0.693) 0.688 0.690

3	(0.307) 0.312 0.310

Locus 15220 :

2 alleles

99.2% missing data

4	(0.487) 0.482 0.487

3	(0.513) 0.518 0.513

Locus 15221 :

2 alleles

98.8% missing data

1	(0.721) 0.715 0.728

3	(0.279) 0.285 0.272

Locus 15222 :

2 alleles

99.2% missing data

2	(0.760) 0.754 0.760

4	(0.240) 0.246 0.240

Locus 15223 :

2 alleles

98.8% missing data

3	(0.593) 0.588 0.599

4	(0.407) 0.412 0.401

Locus 15224 :

2 alleles

99.2% missing data

1	(0.318) 0.317 0.318

3	(0.682) 0.683 0.682

Locus 15225 :

2 alleles

99.2% missing data

3	(0.466) 0.461 0.474

2	(0.534) 0.539 0.526

Locus 15226 :

2 alleles

99.2% missing data

4	(0.525) 0.523 0.529

3	(0.475) 0.477 0.471

Locus 15227 :

2 alleles

96.9% missing data

1	(0.860) 0.856 0.868

3	(0.140) 0.144 0.132

Locus 15228 :

2 alleles

99.2% missing data

1	(0.711) 0.717 0.707

2	(0.289) 0.283 0.293

Locus 15229 :

2 alleles

98.8% missing data

1	(0.589) 0.593 0.590

4	(0.411) 0.407 0.410

Locus 15230 :

2 alleles

99.2% missing data

4	(0.586) 0.579 0.589

3	(0.414) 0.421 0.411

Locus 15231 :

2 alleles

99.2% missing data

1	(0.624) 0.635 0.615

3	(0.376) 0.365 0.385

Locus 15232 :

2 alleles

99.2% missing data

1	(0.463) 0.459 0.466

3	(0.537) 0.541 0.534

Locus 15233 :

2 alleles

91.4% missing data

3	(0.893) 0.894 0.905

2	(0.107) 0.106 0.095

Locus 15234 :

2 alleles

99.2% missing data

1	(0.517) 0.509 0.519

4	(0.483) 0.491 0.481

Locus 15235 :

2 alleles

98.8% missing data

1	(0.708) 0.701 0.713

3	(0.292) 0.299 0.287

Locus 15236 :

3 alleles

98.8% missing data

4	(0.434) 0.433 0.433

2	(0.221) 0.217 0.226

1	(0.345) 0.350 0.341

Locus 15237 :

2 alleles

99.2% missing data

3	(0.153) 0.157 0.150

4	(0.847) 0.843 0.850

Locus 15238 :

2 alleles

90.6% missing data

1	(0.908) 0.924 0.902

2	(0.092) 0.076 0.098

Locus 15239 :

2 alleles

99.2% missing data

3	(0.365) 0.367 0.360

2	(0.635) 0.633 0.640

Locus 15240 :

2 alleles

99.2% missing data

3	(0.346) 0.350 0.349

1	(0.654) 0.650 0.651

Locus 15241 :

2 alleles

99.2% missing data

3	(0.646) 0.646 0.649

1	(0.354) 0.354 0.351

Locus 15242 :

2 alleles

95.3% missing data

3	(0.829) 0.835 0.838

2	(0.171) 0.165 0.162

Locus 15243 :

3 alleles

98.8% missing data

3	(0.421) 0.417 0.422

1	(0.288) 0.292 0.295

2	(0.291) 0.291 0.283

Locus 15244 :

2 alleles

99.2% missing data

4	(0.534) 0.539 0.534

3	(0.466) 0.461 0.466

Locus 15245 :

2 alleles

99.2% missing data

4	(0.654) 0.655 0.650

2	(0.346) 0.345 0.350

Locus 15246 :

2 alleles

99.2% missing data

2	(0.815) 0.816 0.813

1	(0.185) 0.184 0.187

Locus 15247 :

2 alleles

99.2% missing data

2	(0.612) 0.611 0.611

1	(0.388) 0.389 0.389

Locus 15248 :

2 alleles

99.2% missing data

3	(0.387) 0.380 0.393

2	(0.613) 0.620 0.607

Locus 15249 :

2 alleles

99.2% missing data

4	(0.201) 0.199 0.212

1	(0.799) 0.801 0.788

Locus 15250 :

2 alleles

91.0% missing data

3	(0.932) 0.938 0.927

4	(0.068) 0.062 0.073

Locus 15251 :

2 alleles

99.2% missing data

3	(0.482) 0.480 0.488

4	(0.518) 0.520 0.512

Locus 15252 :

2 alleles

95.7% missing data

1	(0.854) 0.849 0.862

3	(0.146) 0.151 0.138

Locus 15253 :

2 alleles

99.2% missing data

1	(0.327) 0.324 0.334

2	(0.673) 0.676 0.666

Locus 15254 :

2 alleles

99.2% missing data

4	(0.492) 0.484 0.495

1	(0.508) 0.516 0.505

Locus 15255 :

2 alleles

99.2% missing data

3	(0.567) 0.571 0.570

1	(0.433) 0.429 0.430

Locus 15256 :

2 alleles

99.2% missing data

3	(0.247) 0.244 0.253

1	(0.753) 0.756 0.747

Locus 15257 :

2 alleles

2.3% missing data

3	(0.987) 0.980 0.996

1	(0.013) 0.020 0.004

Locus 15258 :

2 alleles

99.2% missing data

3	(0.739) 0.743 0.735

4	(0.261) 0.257 0.265

Locus 15259 :

2 alleles

99.2% missing data

1	(0.321) 0.316 0.329

2	(0.679) 0.684 0.671

Locus 15260 :

2 alleles

99.2% missing data

3	(0.316) 0.317 0.310

1	(0.684) 0.683 0.690

Locus 15261 :

2 alleles

99.2% missing data

3	(0.154) 0.156 0.160

4	(0.846) 0.844 0.840

Locus 15262 :

2 alleles

99.2% missing data

4	(0.568) 0.571 0.565

1	(0.432) 0.429 0.435

Locus 15263 :

2 alleles

99.2% missing data

4	(0.578) 0.578 0.579

2	(0.422) 0.422 0.421

Locus 15264 :

2 alleles

99.2% missing data

2	(0.445) 0.446 0.447

3	(0.555) 0.554 0.553

Locus 15265 :

2 alleles

99.2% missing data

3	(0.360) 0.366 0.362

2	(0.640) 0.634 0.638

Locus 15266 :

2 alleles

99.2% missing data

4	(0.531) 0.537 0.521

2	(0.469) 0.463 0.479

Locus 15267 :

2 alleles

99.2% missing data

4	(0.452) 0.453 0.457

3	(0.548) 0.547 0.543

Locus 15268 :

2 alleles

98.8% missing data

4	(0.393) 0.384 0.403

2	(0.607) 0.616 0.597

Locus 15269 :

2 alleles

99.2% missing data

1	(0.359) 0.366 0.354

3	(0.641) 0.634 0.646

Locus 15270 :

2 alleles

99.2% missing data

4	(0.413) 0.409 0.411

1	(0.587) 0.591 0.589

Locus 15271 :

2 alleles

99.2% missing data

3	(0.275) 0.273 0.276

4	(0.725) 0.727 0.724

Locus 15272 :

3 alleles

98.8% missing data

3	(0.505) 0.499 0.500

2	(0.197) 0.197 0.202

1	(0.298) 0.304 0.298

Locus 15273 :

2 alleles

99.2% missing data

1	(0.593) 0.593 0.596

3	(0.407) 0.407 0.404

Locus 15274 :

2 alleles

99.2% missing data

3	(0.580) 0.578 0.585

4	(0.420) 0.422 0.415

Locus 15275 :

2 alleles

99.2% missing data

4	(0.363) 0.357 0.374

3	(0.637) 0.643 0.626

Locus 15276 :

2 alleles

99.2% missing data

3	(0.509) 0.502 0.514

2	(0.491) 0.498 0.486

Locus 15277 :

2 alleles

99.2% missing data

1	(0.611) 0.607 0.616

4	(0.389) 0.393 0.384

Locus 15278 :

2 alleles

95.3% missing data

4	(0.903) 0.896 0.913

2	(0.097) 0.104 0.087

Locus 15279 :

2 alleles

99.2% missing data

1	(0.778) 0.772 0.784

2	(0.222) 0.228 0.216

Locus 15280 :

2 alleles

93.0% missing data

3	(0.869) 0.857 0.888

1	(0.131) 0.143 0.112

Locus 15281 :

2 alleles

99.2% missing data

3	(0.555) 0.553 0.550

2	(0.445) 0.447 0.450

Locus 15282 :

2 alleles

96.1% missing data

1	(0.919) 0.927 0.913

3	(0.081) 0.073 0.087

Locus 15283 :

2 alleles

98.0% missing data

3	(0.447) 0.447 0.457

2	(0.553) 0.553 0.543

Locus 15284 :

2 alleles

99.2% missing data

3	(0.439) 0.439 0.442

2	(0.561) 0.561 0.558

Locus 15285 :

2 alleles

99.2% missing data

3	(0.263) 0.255 0.266

2	(0.737) 0.745 0.734

Locus 15286 :

2 alleles

99.2% missing data

3	(0.621) 0.617 0.618

2	(0.379) 0.383 0.382

Locus 15287 :

3 alleles

98.8% missing data

3	(0.487) 0.482 0.486

1	(0.280) 0.290 0.275

2	(0.234) 0.227 0.239

Locus 15288 :

2 alleles

91.4% missing data

2	(0.926) 0.934 0.924

3	(0.074) 0.066 0.076

Locus 15289 :

2 alleles

98.8% missing data

1	(0.376) 0.373 0.377

3	(0.624) 0.627 0.623

Locus 15290 :

2 alleles

99.2% missing data

3	(0.631) 0.628 0.629

2	(0.369) 0.372 0.371

Locus 15291 :

2 alleles

99.2% missing data

2	(0.618) 0.629 0.608

3	(0.382) 0.371 0.392

Locus 15292 :

2 alleles

93.0% missing data

3	(0.854) 0.853 0.864

1	(0.146) 0.147 0.136

Locus 15293 :

2 alleles

0.0% missing data

2	(0.986) 0.982 0.995

3	(0.014) 0.018 0.005

Locus 15294 :

2 alleles

98.8% missing data

4	(0.696) 0.704 0.694

1	(0.304) 0.296 0.306

Locus 15295 :

2 alleles

99.2% missing data

3	(0.166) 0.164 0.171

1	(0.834) 0.836 0.829

Locus 15296 :

2 alleles

98.8% missing data

4	(0.311) 0.304 0.308

1	(0.689) 0.696 0.692

Locus 15297 :

3 alleles

94.5% missing data

1	(0.348) 0.326 0.370

3	(0.544) 0.563 0.528

4	(0.108) 0.111 0.102

Locus 15298 :

2 alleles

97.7% missing data

1	(0.682) 0.684 0.680

2	(0.318) 0.316 0.320

Locus 15299 :

2 alleles

99.2% missing data

1	(0.634) 0.630 0.639

4	(0.366) 0.370 0.361

Locus 15300 :

2 alleles

99.2% missing data

4	(0.417) 0.420 0.416

3	(0.583) 0.580 0.584

Locus 15301 :

2 alleles

98.4% missing data

2	(0.523) 0.525 0.532

1	(0.477) 0.475 0.468

Locus 15302 :

2 alleles

99.2% missing data

2	(0.328) 0.321 0.333

1	(0.672) 0.679 0.667

Locus 15303 :

2 alleles

98.4% missing data

4	(0.894) 0.894 0.895

1	(0.106) 0.106 0.105

Locus 15304 :

2 alleles

99.2% missing data

1	(0.387) 0.389 0.382

4	(0.613) 0.611 0.618

Locus 15305 :

2 alleles

99.2% missing data

1	(0.789) 0.781 0.794

3	(0.211) 0.219 0.206

Locus 15306 :

2 alleles

99.2% missing data

3	(0.714) 0.708 0.716

1	(0.286) 0.292 0.284

Locus 15307 :

2 alleles

8.2% missing data

2	(0.980) 0.971 0.994

4	(0.020) 0.029 0.006

Locus 15308 :

2 alleles

99.2% missing data

2	(0.453) 0.454 0.448

3	(0.547) 0.546 0.552

Locus 15309 :

3 alleles

98.8% missing data

3	(0.521) 0.515 0.523

4	(0.195) 0.199 0.196

1	(0.284) 0.285 0.281

Locus 15310 :

2 alleles

99.2% missing data

4	(0.636) 0.635 0.638

3	(0.364) 0.365 0.362

Locus 15311 :

2 alleles

99.2% missing data

1	(0.465) 0.459 0.469

3	(0.535) 0.541 0.531

Locus 15312 :

2 alleles

99.2% missing data

2	(0.341) 0.341 0.345

1	(0.659) 0.659 0.655

Locus 15313 :

2 alleles

98.8% missing data

1	(0.490) 0.490 0.496

4	(0.510) 0.510 0.504

Locus 15314 :

2 alleles

99.2% missing data

3	(0.449) 0.456 0.446

4	(0.551) 0.544 0.554

Locus 15315 :

3 alleles

98.8% missing data

1	(0.402) 0.399 0.407

4	(0.323) 0.320 0.322

2	(0.275) 0.281 0.272

Locus 15316 :

2 alleles

99.2% missing data

1	(0.448) 0.442 0.449

4	(0.552) 0.558 0.551

Locus 15317 :

2 alleles

99.2% missing data

4	(0.488) 0.480 0.490

3	(0.512) 0.520 0.510

Locus 15318 :

2 alleles

98.8% missing data

4	(0.778) 0.777 0.780

1	(0.222) 0.223 0.220

Locus 15319 :

2 alleles

98.4% missing data

4	(0.173) 0.176 0.179

3	(0.827) 0.824 0.821

Locus 15320 :

2 alleles

96.9% missing data

1	(0.786) 0.777 0.799

4	(0.214) 0.223 0.201

Locus 15321 :

2 alleles

98.0% missing data

2	(0.295) 0.297 0.286

4	(0.705) 0.703 0.714

Locus 15322 :

2 alleles

99.2% missing data

1	(0.570) 0.560 0.571

4	(0.430) 0.440 0.429

Locus 15323 :

2 alleles

99.2% missing data

1	(0.234) 0.231 0.233

2	(0.766) 0.769 0.767

Locus 15324 :

3 alleles

98.8% missing data

4	(0.542) 0.535 0.536

3	(0.237) 0.239 0.244

2	(0.221) 0.226 0.221

Locus 15325 :

2 alleles

99.2% missing data

1	(0.573) 0.576 0.571

4	(0.427) 0.424 0.429

Locus 15326 :

2 alleles

99.2% missing data

1	(0.377) 0.378 0.375

3	(0.623) 0.622 0.625

Locus 15327 :

2 alleles

99.2% missing data

3	(0.513) 0.514 0.511

4	(0.487) 0.486 0.489

Locus 15328 :

2 alleles

99.2% missing data

3	(0.617) 0.610 0.623

4	(0.383) 0.390 0.377

Locus 15329 :

3 alleles

98.4% missing data

3	(0.274) 0.272 0.272

4	(0.420) 0.415 0.429

2	(0.306) 0.313 0.300

Locus 15330 :

2 alleles

99.2% missing data

2	(0.389) 0.383 0.384

4	(0.611) 0.617 0.616

Locus 15331 :

2 alleles

98.4% missing data

3	(0.659) 0.665 0.664

4	(0.341) 0.335 0.336

Locus 15332 :

2 alleles

98.0% missing data

4	(0.461) 0.463 0.450

3	(0.539) 0.537 0.550

Locus 15333 :

2 alleles

84.8% missing data

2	(0.533) 0.616 0.453

1	(0.467) 0.384 0.547

Locus 15334 :

2 alleles

99.2% missing data

4	(0.842) 0.837 0.845

1	(0.158) 0.163 0.155

Locus 15335 :

2 alleles

91.0% missing data

3	(0.918) 0.929 0.908

2	(0.082) 0.071 0.092

Locus 15336 :

2 alleles

73.4% missing data

4	(0.958) 0.957 0.969

3	(0.042) 0.043 0.031

Locus 15337 :

2 alleles

8.2% missing data

1	(0.990) 0.983 0.997

2	(0.010) 0.017 0.003

Locus 15338 :

2 alleles

98.4% missing data

4	(0.414) 0.410 0.409

3	(0.586) 0.590 0.591

Locus 15339 :

2 alleles

99.2% missing data

1	(0.342) 0.339 0.341

3	(0.658) 0.661 0.659

Locus 15340 :

2 alleles

98.0% missing data

1	(0.671) 0.678 0.666

4	(0.329) 0.322 0.334

Locus 15341 :

2 alleles

99.2% missing data

4	(0.485) 0.489 0.484

3	(0.515) 0.511 0.516

Locus 15342 :

2 alleles

91.4% missing data

4	(0.943) 0.945 0.947

1	(0.057) 0.055 0.053

Locus 15343 :

2 alleles

96.1% missing data

4	(0.822) 0.835 0.816

2	(0.178) 0.165 0.184

Locus 15344 :

3 alleles

98.4% missing data

4	(0.479) 0.467 0.492

3	(0.283) 0.295 0.276

1	(0.239) 0.238 0.232

Locus 15345 :

2 alleles

99.2% missing data

4	(0.487) 0.480 0.494

1	(0.513) 0.520 0.506

Locus 15346 :

2 alleles

96.5% missing data

1	(0.869) 0.872 0.872

3	(0.131) 0.128 0.128

Locus 15347 :

2 alleles

99.2% missing data

1	(0.290) 0.289 0.294

4	(0.710) 0.711 0.706

Locus 15348 :

2 alleles

96.9% missing data

4	(0.140) 0.135 0.136

1	(0.860) 0.865 0.864

Locus 15349 :

2 alleles

99.2% missing data

4	(0.525) 0.527 0.522

1	(0.475) 0.473 0.478

Locus 15350 :

2 alleles

99.2% missing data

2	(0.566) 0.563 0.570

1	(0.434) 0.437 0.430

Locus 15351 :

2 alleles

99.2% missing data

1	(0.490) 0.502 0.485

4	(0.510) 0.498 0.515

Locus 15352 :

3 alleles

98.4% missing data

4	(0.267) 0.261 0.285

3	(0.330) 0.337 0.318

1	(0.403) 0.402 0.398

Locus 15353 :

2 alleles

98.8% missing data

1	(0.682) 0.683 0.676

3	(0.318) 0.317 0.324

Locus 15354 :

2 alleles

99.2% missing data

2	(0.716) 0.709 0.714

3	(0.284) 0.291 0.286

Locus 15355 :

2 alleles

99.2% missing data

2	(0.536) 0.530 0.548

4	(0.464) 0.470 0.452

Locus 15356 :

2 alleles

99.2% missing data

1	(0.615) 0.609 0.621

4	(0.385) 0.391 0.379

Locus 15357 :

2 alleles

99.2% missing data

3	(0.534) 0.533 0.538

1	(0.466) 0.467 0.462

Locus 15358 :

2 alleles

99.2% missing data

4	(0.332) 0.326 0.343

1	(0.668) 0.674 0.657

Locus 15359 :

2 alleles

99.2% missing data

1	(0.584) 0.584 0.580

3	(0.416) 0.416 0.420

Locus 15360 :

2 alleles

99.2% missing data

3	(0.385) 0.383 0.379

4	(0.615) 0.617 0.621

Locus 15361 :

2 alleles

91.0% missing data

3	(0.905) 0.923 0.894

4	(0.095) 0.077 0.106

Locus 15362 :

2 alleles

98.8% missing data

2	(0.490) 0.486 0.495

1	(0.510) 0.514 0.505

Locus 15363 :

2 alleles

98.8% missing data

1	(0.525) 0.527 0.521

2	(0.475) 0.473 0.479

Locus 15364 :

2 alleles

99.2% missing data

1	(0.567) 0.574 0.559

2	(0.433) 0.426 0.441

Locus 15365 :

2 alleles

99.2% missing data

3	(0.618) 0.610 0.622

4	(0.382) 0.390 0.378

Locus 15366 :

2 alleles

8.6% missing data

1	(0.985) 0.978 0.995

4	(0.015) 0.022 0.005

Locus 15367 :

3 alleles

98.8% missing data

3	(0.285) 0.277 0.290

1	(0.300) 0.308 0.291

4	(0.415) 0.415 0.420

Locus 15368 :

2 alleles

98.4% missing data

2	(0.604) 0.611 0.602

4	(0.396) 0.389 0.398

Locus 15369 :

2 alleles

99.2% missing data

3	(0.410) 0.407 0.412

1	(0.590) 0.593 0.588

Locus 15370 :

2 alleles

99.2% missing data

3	(0.233) 0.232 0.237

1	(0.767) 0.768 0.763

Locus 15371 :

3 alleles

98.4% missing data

1	(0.255) 0.250 0.265

3	(0.452) 0.450 0.449

4	(0.294) 0.300 0.286

Locus 15372 :

2 alleles

92.6% missing data

3	(0.894) 0.890 0.903

4	(0.106) 0.110 0.097

Locus 15373 :

2 alleles

96.5% missing data

2	(0.794) 0.800 0.792

4	(0.206) 0.200 0.208

Locus 15374 :

2 alleles

98.8% missing data

1	(0.736) 0.731 0.746

4	(0.264) 0.269 0.254

Locus 15375 :

2 alleles

99.2% missing data

1	(0.633) 0.629 0.632

2	(0.367) 0.371 0.368

Locus 15376 :

2 alleles

99.2% missing data

2	(0.459) 0.461 0.464

4	(0.541) 0.539 0.536

Locus 15377 :

2 alleles

99.2% missing data

1	(0.735) 0.728 0.734

4	(0.265) 0.272 0.266

Locus 15378 :

2 alleles

99.2% missing data

1	(0.433) 0.430 0.432

2	(0.567) 0.570 0.568

Locus 15379 :

2 alleles

99.2% missing data

3	(0.291) 0.296 0.293

1	(0.709) 0.704 0.707

Locus 15380 :

2 alleles

99.2% missing data

2	(0.366) 0.365 0.374

4	(0.634) 0.635 0.626

Locus 15381 :

2 alleles

99.2% missing data

4	(0.308) 0.311 0.306

1	(0.692) 0.689 0.694

Locus 15382 :

2 alleles

98.8% missing data

1	(0.601) 0.604 0.598

4	(0.399) 0.396 0.402

Locus 15383 :

2 alleles

97.3% missing data

4	(0.783) 0.776 0.792

1	(0.217) 0.224 0.208

Locus 15384 :

2 alleles

99.2% missing data

2	(0.700) 0.693 0.698

4	(0.300) 0.307 0.302

Locus 15385 :

2 alleles

98.8% missing data

4	(0.368) 0.363 0.373

1	(0.632) 0.637 0.627

Locus 15386 :

2 alleles

97.3% missing data

3	(0.665) 0.652 0.684

4	(0.335) 0.348 0.316

Locus 15387 :

2 alleles

99.2% missing data

4	(0.690) 0.700 0.686

1	(0.310) 0.300 0.314

Locus 15388 :

2 alleles

99.2% missing data

3	(0.323) 0.319 0.326

2	(0.677) 0.681 0.674

Locus 15389 :

2 alleles

99.2% missing data

3	(0.571) 0.564 0.574

1	(0.429) 0.436 0.426

Locus 15390 :

2 alleles

8.6% missing data

4	(0.983) 0.975 0.995

2	(0.017) 0.025 0.005

Locus 15391 :

2 alleles

98.8% missing data

1	(0.354) 0.351 0.352

2	(0.646) 0.649 0.648

Locus 15392 :

2 alleles

93.0% missing data

1	(0.921) 0.914 0.930

3	(0.079) 0.086 0.070

Locus 15393 :

2 alleles

99.2% missing data

3	(0.341) 0.339 0.344

4	(0.659) 0.661 0.656

Locus 15394 :

2 alleles

98.8% missing data

3	(0.671) 0.665 0.679

4	(0.329) 0.335 0.321

Locus 15395 :

2 alleles

97.7% missing data

3	(0.725) 0.721 0.729

1	(0.275) 0.279 0.271

Locus 15396 :

2 alleles

98.4% missing data

3	(0.473) 0.479 0.470

4	(0.527) 0.521 0.530

Locus 15397 :

2 alleles

98.0% missing data

1	(0.355) 0.340 0.366

2	(0.645) 0.660 0.634

Locus 15398 :

2 alleles

99.2% missing data

4	(0.520) 0.525 0.519

3	(0.480) 0.475 0.481

Locus 15399 :

2 alleles

95.7% missing data

4	(0.811) 0.819 0.813

1	(0.189) 0.181 0.187

Locus 15400 :

3 alleles

98.8% missing data

1	(0.385) 0.384 0.392

3	(0.309) 0.307 0.303

2	(0.306) 0.310 0.305

Locus 15401 :

2 alleles

99.2% missing data

3	(0.500) 0.503 0.500

4	(0.500) 0.497 0.500

Locus 15402 :

2 alleles

98.8% missing data

1	(0.546) 0.556 0.544

3	(0.454) 0.444 0.456

Locus 15403 :

2 alleles

99.2% missing data

2	(0.761) 0.763 0.762

4	(0.239) 0.237 0.238

Locus 15404 :

2 alleles

99.2% missing data

3	(0.526) 0.525 0.521

1	(0.474) 0.475 0.479

Locus 15405 :

2 alleles

97.3% missing data

4	(0.864) 0.863 0.865

3	(0.136) 0.137 0.135

Locus 15406 :

3 alleles

73.0% missing data

1	(0.928) 0.921 0.948

2	(0.033) 0.032 0.026

3	(0.039) 0.047 0.026

Locus 15407 :

2 alleles

96.1% missing data

2	(0.838) 0.839 0.845

1	(0.162) 0.161 0.155

Locus 15408 :

2 alleles

98.8% missing data

2	(0.710) 0.703 0.714

1	(0.290) 0.297 0.286

Locus 15409 :

2 alleles

99.2% missing data

1	(0.606) 0.605 0.608

4	(0.394) 0.395 0.392

Locus 15410 :

2 alleles

96.5% missing data

1	(0.312) 0.295 0.313

3	(0.688) 0.705 0.687

Locus 15411 :

3 alleles

98.8% missing data

2	(0.305) 0.304 0.312

1	(0.175) 0.173 0.178

4	(0.520) 0.523 0.511

Locus 15412 :

2 alleles

99.2% missing data

4	(0.674) 0.671 0.677

3	(0.326) 0.329 0.323

Locus 15413 :

3 alleles

98.8% missing data

3	(0.318) 0.318 0.318

2	(0.378) 0.373 0.385

4	(0.304) 0.309 0.297

Locus 15414 :

2 alleles

99.2% missing data

4	(0.587) 0.582 0.595

1	(0.413) 0.418 0.405

Locus 15415 :

2 alleles

99.2% missing data

4	(0.299) 0.299 0.303

1	(0.701) 0.701 0.697

Locus 15416 :

2 alleles

98.4% missing data

4	(0.691) 0.684 0.695

3	(0.309) 0.316 0.305

Locus 15417 :

2 alleles

99.2% missing data

1	(0.849) 0.850 0.840

4	(0.151) 0.150 0.160

Locus 15418 :

2 alleles

99.2% missing data

1	(0.661) 0.660 0.663

3	(0.339) 0.340 0.337

Locus 15419 :

2 alleles

99.2% missing data

3	(0.361) 0.353 0.371

4	(0.639) 0.647 0.629

Locus 15420 :

2 alleles

99.2% missing data

4	(0.425) 0.423 0.426

3	(0.575) 0.577 0.574

Locus 15421 :

2 alleles

99.2% missing data

1	(0.349) 0.353 0.348

3	(0.651) 0.647 0.652

Locus 15422 :

2 alleles

8.2% missing data

3	(0.973) 0.947 0.991

1	(0.027) 0.053 0.009

Locus 15423 :

2 alleles

98.0% missing data

2	(0.720) 0.725 0.723

1	(0.280) 0.275 0.277

Locus 15424 :

2 alleles

99.2% missing data

3	(0.456) 0.451 0.457

1	(0.544) 0.549 0.543

Locus 15425 :

2 alleles

99.2% missing data

3	(0.350) 0.345 0.357

1	(0.650) 0.655 0.643

Locus 15426 :

2 alleles

99.2% missing data

1	(0.314) 0.321 0.309

4	(0.686) 0.679 0.691

Locus 15427 :

2 alleles

98.4% missing data

4	(0.232) 0.228 0.233

1	(0.768) 0.772 0.767

Locus 15428 :

2 alleles

99.2% missing data

2	(0.841) 0.846 0.837

1	(0.159) 0.154 0.163

Locus 15429 :

2 alleles

98.0% missing data

1	(0.827) 0.829 0.828

3	(0.173) 0.171 0.172

Locus 15430 :

2 alleles

99.2% missing data

3	(0.396) 0.392 0.398

2	(0.604) 0.608 0.602

Locus 15431 :

2 alleles

98.4% missing data

4	(0.577) 0.582 0.570

3	(0.423) 0.418 0.430

Locus 15432 :

2 alleles

98.8% missing data

1	(0.818) 0.816 0.819

2	(0.182) 0.184 0.181

Locus 15433 :

2 alleles

99.2% missing data

4	(0.276) 0.273 0.276

1	(0.724) 0.727 0.724

Locus 15434 :

2 alleles

99.2% missing data

2	(0.605) 0.608 0.601

4	(0.395) 0.392 0.399

Locus 15435 :

2 alleles

99.2% missing data

4	(0.397) 0.393 0.402

3	(0.603) 0.607 0.598

Locus 15436 :

2 alleles

96.1% missing data

1	(0.778) 0.790 0.770

2	(0.222) 0.210 0.230

Locus 15437 :

2 alleles

99.2% missing data

1	(0.492) 0.491 0.496

4	(0.508) 0.509 0.504

Locus 15438 :

2 alleles

98.4% missing data

4	(0.845) 0.840 0.848

2	(0.155) 0.160 0.152

Locus 15439 :

2 alleles

99.2% missing data

2	(0.717) 0.724 0.714

1	(0.283) 0.276 0.286

Locus 15440 :

2 alleles

96.1% missing data

2	(0.782) 0.795 0.774

4	(0.218) 0.205 0.226

Locus 15441 :

2 alleles

92.6% missing data

1	(0.886) 0.884 0.898

3	(0.114) 0.116 0.102

Locus 15442 :

2 alleles

99.2% missing data

4	(0.368) 0.366 0.363

1	(0.632) 0.634 0.637

Locus 15443 :

3 alleles

96.1% missing data

4	(0.681) 0.698 0.674

1	(0.144) 0.140 0.146

2	(0.175) 0.162 0.181

Locus 15444 :

2 alleles

99.2% missing data

3	(0.224) 0.222 0.229

1	(0.776) 0.778 0.771

Locus 15445 :

2 alleles

99.2% missing data

3	(0.599) 0.606 0.591

4	(0.401) 0.394 0.409

Locus 15446 :

2 alleles

91.8% missing data

3	(0.909) 0.923 0.899

1	(0.091) 0.077 0.101

Locus 15447 :

2 alleles

97.7% missing data

4	(0.721) 0.721 0.730

2	(0.279) 0.279 0.270

Locus 15448 :

2 alleles

97.7% missing data

1	(0.834) 0.827 0.844

3	(0.166) 0.173 0.156

Locus 15449 :

2 alleles

99.2% missing data

1	(0.392) 0.389 0.397

3	(0.608) 0.611 0.603

Locus 15450 :

2 alleles

99.2% missing data

4	(0.156) 0.153 0.163

1	(0.844) 0.847 0.837

Locus 15451 :

2 alleles

99.2% missing data

4	(0.590) 0.591 0.592

1	(0.410) 0.409 0.408

Locus 15452 :

2 alleles

99.2% missing data

3	(0.530) 0.522 0.534

1	(0.470) 0.478 0.466

Locus 15453 :

2 alleles

97.7% missing data

3	(0.456) 0.457 0.444

1	(0.544) 0.543 0.556

Locus 15454 :

2 alleles

98.8% missing data

4	(0.606) 0.609 0.605

3	(0.394) 0.391 0.395

Locus 15455 :

2 alleles

99.2% missing data

2	(0.480) 0.478 0.486

3	(0.520) 0.522 0.514

Locus 15456 :

3 alleles

98.8% missing data

4	(0.395) 0.389 0.394

1	(0.448) 0.457 0.444

3	(0.157) 0.154 0.162

Locus 15457 :

2 alleles

99.2% missing data

2	(0.233) 0.237 0.230

1	(0.767) 0.763 0.770

Locus 15458 :

2 alleles

99.2% missing data

2	(0.554) 0.557 0.563

3	(0.446) 0.443 0.437

Locus 15459 :

2 alleles

99.2% missing data

1	(0.336) 0.328 0.347

4	(0.664) 0.672 0.653

Locus 15460 :

2 alleles

98.0% missing data

1	(0.793) 0.793 0.795

4	(0.207) 0.207 0.205

Locus 15461 :

2 alleles

99.2% missing data

4	(0.681) 0.683 0.674

2	(0.319) 0.317 0.326

Locus 15462 :

2 alleles

99.2% missing data

3	(0.659) 0.666 0.652

1	(0.341) 0.334 0.348

Locus 15463 :

2 alleles

99.2% missing data

4	(0.652) 0.651 0.646

1	(0.348) 0.349 0.354

Locus 15464 :

2 alleles

99.2% missing data

2	(0.686) 0.695 0.683

3	(0.314) 0.305 0.317

Locus 15465 :

2 alleles

9.4% missing data

3	(0.989) 0.983 0.997

1	(0.011) 0.017 0.003

Locus 15466 :

2 alleles

99.2% missing data

3	(0.877) 0.875 0.882

2	(0.123) 0.125 0.118

Locus 15467 :

3 alleles

98.4% missing data

1	(0.288) 0.287 0.287

4	(0.395) 0.400 0.389

3	(0.318) 0.314 0.324

Locus 15468 :

2 alleles

99.2% missing data

3	(0.546) 0.542 0.551

1	(0.454) 0.458 0.449

Locus 15469 :

2 alleles

99.2% missing data

3	(0.462) 0.466 0.455

2	(0.538) 0.534 0.545

Locus 15470 :

2 alleles

99.2% missing data

2	(0.509) 0.507 0.516

3	(0.491) 0.493 0.484

Locus 15471 :

2 alleles

99.2% missing data

3	(0.611) 0.604 0.615

4	(0.389) 0.396 0.385

Locus 15472 :

2 alleles

99.2% missing data

1	(0.414) 0.409 0.421

2	(0.586) 0.591 0.579

Locus 15473 :

2 alleles

98.8% missing data

3	(0.507) 0.503 0.510

2	(0.493) 0.497 0.490

Locus 15474 :

2 alleles

99.2% missing data

4	(0.347) 0.349 0.353

2	(0.653) 0.651 0.647

Locus 15475 :

2 alleles

99.2% missing data

4	(0.581) 0.571 0.587

1	(0.419) 0.429 0.413

Locus 15476 :

3 alleles

98.4% missing data

2	(0.376) 0.370 0.383

4	(0.350) 0.349 0.345

1	(0.274) 0.280 0.272

Locus 15477 :

2 alleles

99.2% missing data

4	(0.550) 0.541 0.554

1	(0.450) 0.459 0.446

Locus 15478 :

2 alleles

96.1% missing data

2	(0.755) 0.766 0.760

4	(0.245) 0.234 0.240

Locus 15479 :

2 alleles

99.2% missing data

3	(0.301) 0.297 0.312

4	(0.699) 0.703 0.688

Locus 15480 :

2 alleles

98.4% missing data

4	(0.651) 0.655 0.646

1	(0.349) 0.345 0.354

Locus 15481 :

2 alleles

99.2% missing data

2	(0.391) 0.398 0.398

4	(0.609) 0.602 0.602

Locus 15482 :

2 alleles

99.2% missing data

1	(0.405) 0.401 0.409

4	(0.595) 0.599 0.591

Locus 15483 :

2 alleles

99.2% missing data

1	(0.717) 0.718 0.712

4	(0.283) 0.282 0.288

Locus 15484 :

2 alleles

99.2% missing data

1	(0.322) 0.331 0.318

2	(0.678) 0.669 0.682

Locus 15485 :

2 alleles

99.2% missing data

2	(0.539) 0.534 0.546

4	(0.461) 0.466 0.454

Locus 15486 :

3 alleles

98.8% missing data

4	(0.309) 0.307 0.307

3	(0.464) 0.469 0.454

1	(0.227) 0.224 0.239

Locus 15487 :

2 alleles

96.1% missing data

4	(0.797) 0.801 0.801

2	(0.203) 0.199 0.199

Locus 15488 :

2 alleles

97.7% missing data

1	(0.771) 0.771 0.772

3	(0.229) 0.229 0.228

Locus 15489 :

2 alleles

99.2% missing data

4	(0.756) 0.752 0.760

3	(0.244) 0.248 0.240

Locus 15490 :

2 alleles

98.8% missing data

3	(0.389) 0.382 0.393

2	(0.611) 0.618 0.607

Locus 15491 :

2 alleles

99.2% missing data

2	(0.480) 0.477 0.480

1	(0.520) 0.523 0.520

Locus 15492 :

2 alleles

99.2% missing data

4	(0.366) 0.375 0.362

1	(0.634) 0.625 0.638

Locus 15493 :

2 alleles

96.1% missing data

3	(0.814) 0.818 0.820

4	(0.186) 0.182 0.180

Locus 15494 :

2 alleles

99.2% missing data

4	(0.408) 0.406 0.406

3	(0.592) 0.594 0.594

Locus 15495 :

2 alleles

99.2% missing data

4	(0.313) 0.303 0.316

1	(0.687) 0.697 0.684

Locus 15496 :

2 alleles

99.2% missing data

1	(0.614) 0.608 0.615

3	(0.386) 0.392 0.385

Locus 15497 :

2 alleles

99.2% missing data

1	(0.590) 0.592 0.586

4	(0.410) 0.408 0.414

Locus 15498 :

2 alleles

99.2% missing data

4	(0.556) 0.551 0.563

1	(0.444) 0.449 0.437

Locus 15499 :

2 alleles

99.2% missing data

3	(0.604) 0.604 0.608

4	(0.396) 0.396 0.392

Locus 15500 :

3 alleles

98.8% missing data

4	(0.241) 0.241 0.244

2	(0.182) 0.175 0.185

1	(0.578) 0.584 0.571

Locus 15501 :

2 alleles

99.2% missing data

4	(0.380) 0.376 0.385

2	(0.620) 0.624 0.615

Locus 15502 :

2 alleles

0.0% missing data

3	(0.985) 0.988 0.993

2	(0.015) 0.012 0.007

Locus 15503 :

2 alleles

96.5% missing data

1	(0.826) 0.815 0.843

4	(0.174) 0.185 0.157

Locus 15504 :

2 alleles

96.1% missing data

3	(0.137) 0.124 0.140

4	(0.863) 0.876 0.860

Locus 15505 :

2 alleles

93.0% missing data

4	(0.849) 0.844 0.862

3	(0.151) 0.156 0.138

Locus 15506 :

2 alleles

78.1% missing data

2	(0.952) 0.942 0.969

4	(0.048) 0.058 0.031

Locus 15507 :

2 alleles

98.0% missing data

3	(0.645) 0.639 0.657

2	(0.355) 0.361 0.343

Locus 15508 :

2 alleles

96.5% missing data

1	(0.757) 0.770 0.755

3	(0.243) 0.230 0.245

Locus 15509 :

2 alleles

98.8% missing data

3	(0.237) 0.228 0.243

2	(0.763) 0.772 0.757

Locus 15510 :

2 alleles

99.2% missing data

4	(0.380) 0.374 0.385

3	(0.620) 0.626 0.615

Locus 15511 :

2 alleles

0.4% missing data

1	(0.992) 0.994 0.994

4	(0.008) 0.006 0.006

Locus 15512 :

2 alleles

97.7% missing data

1	(0.224) 0.226 0.217

3	(0.776) 0.774 0.783

Locus 15513 :

2 alleles

99.2% missing data

3	(0.423) 0.423 0.421

1	(0.577) 0.577 0.579

Locus 15514 :

2 alleles

98.8% missing data

1	(0.692) 0.685 0.700

2	(0.308) 0.315 0.300

Locus 15515 :

2 alleles

99.2% missing data

4	(0.766) 0.769 0.758

2	(0.234) 0.231 0.242

Locus 15516 :

2 alleles

99.2% missing data

3	(0.593) 0.597 0.586

1	(0.407) 0.403 0.414

Locus 15517 :

2 alleles

96.9% missing data

2	(0.865) 0.880 0.858

1	(0.135) 0.120 0.142

Locus 15518 :

2 alleles

99.2% missing data

3	(0.371) 0.369 0.371

4	(0.629) 0.631 0.629

Locus 15519 :

2 alleles

99.2% missing data

4	(0.499) 0.494 0.509

1	(0.501) 0.506 0.491

Locus 15520 :

2 alleles

99.2% missing data

3	(0.604) 0.606 0.600

1	(0.396) 0.394 0.400

Locus 15521 :

2 alleles

99.2% missing data

1	(0.416) 0.421 0.411

3	(0.584) 0.579 0.589

Locus 15522 :

2 alleles

99.2% missing data

3	(0.432) 0.435 0.434

4	(0.568) 0.565 0.566

Locus 15523 :

2 alleles

99.2% missing data

3	(0.342) 0.344 0.338

4	(0.658) 0.656 0.662

Locus 15524 :

2 alleles

98.8% missing data

2	(0.405) 0.404 0.411

1	(0.595) 0.596 0.589

Locus 15525 :

2 alleles

99.2% missing data

4	(0.608) 0.602 0.612

3	(0.392) 0.398 0.388

Locus 15526 :

3 alleles

96.9% missing data

3	(0.475) 0.478 0.461

4	(0.391) 0.387 0.401

1	(0.134) 0.135 0.138

Locus 15527 :

2 alleles

99.2% missing data

4	(0.572) 0.573 0.575

1	(0.428) 0.427 0.425

Locus 15528 :

2 alleles

99.2% missing data

1	(0.755) 0.756 0.751

3	(0.245) 0.244 0.249

Locus 15529 :

2 alleles

99.2% missing data

4	(0.585) 0.582 0.588

1	(0.415) 0.418 0.412

Locus 15530 :

2 alleles

90.6% missing data

2	(0.873) 0.886 0.867

3	(0.127) 0.114 0.133

Locus 15531 :

2 alleles

99.2% missing data

1	(0.515) 0.514 0.517

4	(0.485) 0.486 0.483

Locus 15532 :

2 alleles

99.2% missing data

3	(0.270) 0.267 0.266

1	(0.730) 0.733 0.734

Locus 15533 :

2 alleles

99.2% missing data

1	(0.284) 0.280 0.289

3	(0.716) 0.720 0.711

Locus 15534 :

2 alleles

93.8% missing data

3	(0.885) 0.882 0.892

2	(0.115) 0.118 0.108

Locus 15535 :

2 alleles

99.2% missing data

4	(0.545) 0.549 0.547

3	(0.455) 0.451 0.453

Locus 15536 :

2 alleles

99.2% missing data

1	(0.462) 0.460 0.468

2	(0.538) 0.540 0.532

Locus 15537 :

2 alleles

99.2% missing data

3	(0.760) 0.762 0.752

2	(0.240) 0.238 0.248

Locus 15538 :

2 alleles

99.2% missing data

1	(0.460) 0.456 0.461

3	(0.540) 0.544 0.539

Locus 15539 :

2 alleles

94.5% missing data

4	(0.833) 0.849 0.824

3	(0.167) 0.151 0.176

Locus 15540 :

2 alleles

99.2% missing data

1	(0.515) 0.508 0.520

2	(0.485) 0.492 0.480

Locus 15541 :

2 alleles

99.2% missing data

1	(0.253) 0.262 0.250

4	(0.747) 0.738 0.750

Locus 15542 :

2 alleles

98.8% missing data

3	(0.573) 0.578 0.566

2	(0.427) 0.422 0.434

Locus 15543 :

2 alleles

99.2% missing data

2	(0.389) 0.393 0.392

3	(0.611) 0.607 0.608

Locus 15544 :

2 alleles

91.0% missing data

2	(0.877) 0.896 0.869

4	(0.123) 0.104 0.131

Locus 15545 :

2 alleles

99.2% missing data

4	(0.320) 0.318 0.313

3	(0.680) 0.682 0.687

Locus 15546 :

2 alleles

99.2% missing data

3	(0.373) 0.376 0.370

4	(0.627) 0.624 0.630

Locus 15547 :

2 alleles

99.2% missing data

2	(0.532) 0.530 0.530

3	(0.468) 0.470 0.470

Locus 15548 :

3 alleles

98.4% missing data

1	(0.494) 0.482 0.498

2	(0.247) 0.256 0.245

4	(0.259) 0.262 0.257

Locus 15549 :

2 alleles

99.2% missing data

3	(0.353) 0.342 0.357

1	(0.647) 0.658 0.643

Locus 15550 :

2 alleles

99.2% missing data

3	(0.444) 0.448 0.441

1	(0.556) 0.552 0.559

Locus 15551 :

2 alleles

99.2% missing data

3	(0.487) 0.486 0.486

2	(0.513) 0.514 0.514

Locus 15552 :

2 alleles

96.5% missing data

2	(0.743) 0.753 0.743

3	(0.257) 0.247 0.257

Locus 15553 :

3 alleles

98.4% missing data

4	(0.340) 0.347 0.337

3	(0.268) 0.259 0.270

1	(0.392) 0.393 0.393

Locus 15554 :

2 alleles

99.2% missing data

4	(0.543) 0.545 0.547

2	(0.457) 0.455 0.453

Locus 15555 :

2 alleles

99.2% missing data

2	(0.525) 0.532 0.518

3	(0.475) 0.468 0.482

Locus 15556 :

2 alleles

99.2% missing data

3	(0.389) 0.384 0.386

1	(0.611) 0.616 0.614

Locus 15557 :

2 alleles

98.4% missing data

1	(0.739) 0.747 0.734

3	(0.261) 0.253 0.266

Locus 15558 :

2 alleles

98.8% missing data

4	(0.363) 0.356 0.368

2	(0.637) 0.644 0.632

Locus 15559 :

2 alleles

99.2% missing data

2	(0.423) 0.418 0.424

4	(0.577) 0.582 0.576

Locus 15560 :

2 alleles

99.2% missing data

1	(0.336) 0.334 0.342

4	(0.664) 0.666 0.658

Locus 15561 :

2 alleles

99.2% missing data

1	(0.436) 0.426 0.440

2	(0.564) 0.574 0.560

Locus 15562 :

2 alleles

99.2% missing data

2	(0.418) 0.424 0.416

4	(0.582) 0.576 0.584

Locus 15563 :

2 alleles

99.2% missing data

3	(0.525) 0.524 0.527

1	(0.475) 0.476 0.473

Locus 15564 :

2 alleles

98.0% missing data

3	(0.280) 0.278 0.284

1	(0.720) 0.722 0.716

Locus 15565 :

2 alleles

96.1% missing data

4	(0.843) 0.853 0.840

1	(0.157) 0.147 0.160

Locus 15566 :

2 alleles

99.2% missing data

4	(0.642) 0.646 0.645

1	(0.358) 0.354 0.355

Locus 15567 :

2 alleles

99.2% missing data

3	(0.321) 0.318 0.325

1	(0.679) 0.682 0.675

Locus 15568 :

2 alleles

99.2% missing data

3	(0.566) 0.560 0.573

2	(0.434) 0.440 0.427

Locus 15569 :

2 alleles

99.2% missing data

1	(0.324) 0.322 0.326

4	(0.676) 0.678 0.674

Locus 15570 :

2 alleles

99.2% missing data

2	(0.546) 0.550 0.541

4	(0.454) 0.450 0.459

Locus 15571 :

2 alleles

96.1% missing data

3	(0.865) 0.870 0.869

2	(0.135) 0.130 0.131

Locus 15572 :

2 alleles

98.8% missing data

4	(0.467) 0.467 0.470

2	(0.533) 0.533 0.530

Locus 15573 :

2 alleles

98.0% missing data

1	(0.740) 0.734 0.746

2	(0.260) 0.266 0.254

Locus 15574 :

2 alleles

98.4% missing data

1	(0.468) 0.459 0.466

4	(0.532) 0.541 0.534

Locus 15575 :

2 alleles

99.2% missing data

1	(0.500) 0.501 0.506

2	(0.500) 0.499 0.494

Locus 15576 :

2 alleles

98.4% missing data

2	(0.259) 0.260 0.257

4	(0.741) 0.740 0.743

Locus 15577 :

2 alleles

99.2% missing data

3	(0.222) 0.223 0.227

2	(0.778) 0.777 0.773

Locus 15578 :

2 alleles

98.4% missing data

1	(0.752) 0.751 0.749

4	(0.248) 0.249 0.251

Locus 15579 :

2 alleles

98.0% missing data

4	(0.623) 0.624 0.624

3	(0.377) 0.376 0.376

Locus 15580 :

2 alleles

99.2% missing data

3	(0.279) 0.279 0.287

1	(0.721) 0.721 0.713

Locus 15581 :

2 alleles

99.2% missing data

3	(0.692) 0.701 0.690

4	(0.308) 0.299 0.310

Locus 15582 :

2 alleles

99.2% missing data

1	(0.703) 0.699 0.708

2	(0.297) 0.301 0.292

Locus 15583 :

2 alleles

99.2% missing data

4	(0.548) 0.549 0.547

3	(0.452) 0.451 0.453

Locus 15584 :

2 alleles

99.2% missing data

3	(0.667) 0.669 0.662

1	(0.333) 0.331 0.338

Locus 15585 :

2 alleles

99.2% missing data

3	(0.699) 0.702 0.693

4	(0.301) 0.298 0.307

Locus 15586 :

2 alleles

96.5% missing data

4	(0.664) 0.653 0.684

1	(0.336) 0.347 0.316

Locus 15587 :

2 alleles

99.2% missing data

3	(0.330) 0.331 0.332

4	(0.670) 0.669 0.668

Locus 15588 :

2 alleles

99.2% missing data

1	(0.564) 0.565 0.571

3	(0.436) 0.435 0.429

Locus 15589 :

2 alleles

96.5% missing data

4	(0.778) 0.780 0.778

3	(0.222) 0.220 0.222

Locus 15590 :

2 alleles

99.2% missing data

4	(0.257) 0.265 0.257

2	(0.743) 0.735 0.743

Locus 15591 :

2 alleles

90.6% missing data

3	(0.910) 0.925 0.902

1	(0.090) 0.075 0.098

Locus 15592 :

2 alleles

98.4% missing data

3	(0.822) 0.819 0.829

2	(0.178) 0.181 0.171

Locus 15593 :

2 alleles

0.0% missing data

4	(0.948) 0.959 0.945

1	(0.052) 0.041 0.055

Locus 15594 :

2 alleles

96.9% missing data

4	(0.763) 0.757 0.779

3	(0.237) 0.243 0.221

Locus 15595 :

2 alleles

98.0% missing data

3	(0.638) 0.634 0.648

1	(0.362) 0.366 0.352

Locus 15596 :

2 alleles

99.2% missing data

1	(0.322) 0.318 0.326

4	(0.678) 0.682 0.674

Locus 15597 :

2 alleles

99.2% missing data

1	(0.676) 0.674 0.680

2	(0.324) 0.326 0.320

Locus 15598 :

2 alleles

96.1% missing data

1	(0.827) 0.840 0.820

4	(0.173) 0.160 0.180

Locus 15599 :

2 alleles

99.2% missing data

3	(0.488) 0.479 0.496

4	(0.512) 0.521 0.504

Locus 15600 :

2 alleles

96.5% missing data

4	(0.842) 0.844 0.843

2	(0.158) 0.156 0.157

Locus 15601 :

2 alleles

96.1% missing data

1	(0.188) 0.175 0.196

4	(0.812) 0.825 0.804

Locus 15602 :

2 alleles

98.0% missing data

4	(0.553) 0.562 0.553

3	(0.447) 0.438 0.447

Locus 15603 :

2 alleles

99.2% missing data

3	(0.591) 0.582 0.596

1	(0.409) 0.418 0.404

Locus 15604 :

2 alleles

96.5% missing data

3	(0.782) 0.794 0.775

4	(0.218) 0.206 0.225

Locus 15605 :

2 alleles

99.2% missing data

1	(0.330) 0.329 0.335

4	(0.670) 0.671 0.665

Locus 15606 :

2 alleles

99.2% missing data

4	(0.482) 0.487 0.472

1	(0.518) 0.513 0.528

Locus 15607 :

2 alleles

99.2% missing data

1	(0.582) 0.582 0.582

3	(0.418) 0.418 0.418

Locus 15608 :

2 alleles

99.2% missing data

4	(0.771) 0.766 0.767

2	(0.229) 0.234 0.233

Locus 15609 :

2 alleles

99.2% missing data

1	(0.408) 0.410 0.403

2	(0.592) 0.590 0.597

Locus 15610 :

2 alleles

99.2% missing data

2	(0.680) 0.679 0.678

3	(0.320) 0.321 0.322

Locus 15611 :

2 alleles

99.2% missing data

2	(0.466) 0.459 0.472

4	(0.534) 0.541 0.528

Locus 15612 :

2 alleles

99.2% missing data

2	(0.556) 0.548 0.562

3	(0.444) 0.452 0.438

Locus 15613 :

2 alleles

96.9% missing data

3	(0.227) 0.213 0.237

4	(0.773) 0.787 0.763

Locus 15614 :

2 alleles

99.2% missing data

2	(0.592) 0.594 0.590

1	(0.408) 0.406 0.410

Locus 15615 :

2 alleles

92.6% missing data

4	(0.888) 0.883 0.900

2	(0.112) 0.117 0.100

Locus 15616 :

2 alleles

99.2% missing data

4	(0.440) 0.447 0.437

2	(0.560) 0.553 0.563

Locus 15617 :

2 alleles

99.2% missing data

4	(0.473) 0.475 0.468

2	(0.527) 0.525 0.532

Locus 15618 :

2 alleles

99.2% missing data

2	(0.845) 0.843 0.848

4	(0.155) 0.157 0.152

Locus 15619 :

2 alleles

99.2% missing data

1	(0.684) 0.685 0.689

3	(0.316) 0.315 0.311

Locus 15620 :

2 alleles

99.2% missing data

2	(0.417) 0.416 0.416

1	(0.583) 0.584 0.584

Locus 15621 :

2 alleles

98.8% missing data

4	(0.512) 0.508 0.522

3	(0.488) 0.492 0.478

Locus 15622 :

2 alleles

99.2% missing data

4	(0.500) 0.496 0.499

2	(0.500) 0.504 0.501

Locus 15623 :

2 alleles

96.9% missing data

3	(0.791) 0.783 0.806

4	(0.209) 0.217 0.194

Locus 15624 :

2 alleles

99.2% missing data

3	(0.401) 0.405 0.394

4	(0.599) 0.595 0.606

Locus 15625 :

3 alleles

98.8% missing data

4	(0.280) 0.286 0.276

1	(0.464) 0.455 0.468

2	(0.256) 0.259 0.255

Locus 15626 :

2 alleles

99.2% missing data

4	(0.253) 0.250 0.258

1	(0.747) 0.750 0.742

Locus 15627 :

2 alleles

97.7% missing data

4	(0.637) 0.641 0.636

1	(0.363) 0.359 0.364

Locus 15628 :

2 alleles

99.2% missing data

2	(0.397) 0.397 0.392

3	(0.603) 0.603 0.608

Locus 15629 :

2 alleles

99.2% missing data

3	(0.275) 0.277 0.276

4	(0.725) 0.723 0.724

Locus 15630 :

2 alleles

95.3% missing data

1	(0.332) 0.326 0.343

3	(0.668) 0.674 0.657

Locus 15631 :

2 alleles

99.2% missing data

4	(0.604) 0.601 0.604

1	(0.396) 0.399 0.396

Locus 15632 :

2 alleles

96.9% missing data

1	(0.082) 0.084 0.076

4	(0.918) 0.916 0.924

Locus 15633 :

2 alleles

98.8% missing data

4	(0.339) 0.347 0.337

1	(0.661) 0.653 0.663

Locus 15634 :

2 alleles

99.2% missing data

2	(0.235) 0.236 0.236

1	(0.765) 0.764 0.764

Locus 15635 :

2 alleles

98.8% missing data

3	(0.814) 0.815 0.812

4	(0.186) 0.185 0.188

Locus 15636 :

2 alleles

99.2% missing data

2	(0.326) 0.330 0.326

1	(0.674) 0.670 0.674

Locus 15637 :

2 alleles

8.6% missing data

2	(0.975) 0.961 0.992

1	(0.025) 0.039 0.008

Locus 15638 :

2 alleles

96.9% missing data

4	(0.345) 0.346 0.339

3	(0.655) 0.654 0.661

Locus 15639 :

2 alleles

84.0% missing data

2	(0.963) 0.965 0.965

1	(0.037) 0.035 0.035

Locus 15640 :

2 alleles

98.8% missing data

2	(0.880) 0.882 0.874

4	(0.120) 0.118 0.126

Locus 15641 :

2 alleles

99.2% missing data

4	(0.411) 0.413 0.415

2	(0.589) 0.587 0.585

Locus 15642 :

2 alleles

99.2% missing data

4	(0.470) 0.474 0.472

1	(0.530) 0.526 0.528

Locus 15643 :

2 alleles

99.2% missing data

1	(0.556) 0.553 0.558

4	(0.444) 0.447 0.442

Locus 15644 :

2 alleles

98.0% missing data

3	(0.221) 0.220 0.218

4	(0.779) 0.780 0.782

Locus 15645 :

2 alleles

99.2% missing data

4	(0.353) 0.362 0.345

1	(0.647) 0.638 0.655

Locus 15646 :

2 alleles

99.2% missing data

3	(0.348) 0.348 0.352

4	(0.652) 0.652 0.648

Locus 15647 :

2 alleles

99.2% missing data

4	(0.514) 0.509 0.519

3	(0.486) 0.491 0.481

Locus 15648 :

2 alleles

99.2% missing data

2	(0.702) 0.700 0.702

4	(0.298) 0.300 0.298

Locus 15649 :

2 alleles

99.2% missing data

3	(0.525) 0.526 0.524

1	(0.475) 0.474 0.476

Locus 15650 :

2 alleles

98.0% missing data

1	(0.592) 0.598 0.592

2	(0.408) 0.402 0.408

Locus 15651 :

2 alleles

99.2% missing data

3	(0.597) 0.591 0.601

2	(0.403) 0.409 0.399

Locus 15652 :

2 alleles

97.3% missing data

2	(0.827) 0.829 0.827

1	(0.173) 0.171 0.173

Locus 15653 :

2 alleles

99.2% missing data

1	(0.388) 0.386 0.396

4	(0.612) 0.614 0.604

Locus 15654 :

2 alleles

99.2% missing data

1	(0.504) 0.499 0.503

3	(0.496) 0.501 0.497

Locus 15655 :

2 alleles

97.3% missing data

4	(0.274) 0.262 0.274

1	(0.726) 0.738 0.726

Locus 15656 :

2 alleles

99.2% missing data

3	(0.557) 0.558 0.556

1	(0.443) 0.442 0.444

Locus 15657 :

2 alleles

90.6% missing data

1	(0.898) 0.915 0.887

3	(0.102) 0.085 0.113

Locus 15658 :

2 alleles

98.0% missing data

2	(0.592) 0.591 0.595

4	(0.408) 0.409 0.405

Locus 15659 :

2 alleles

99.2% missing data

2	(0.369) 0.358 0.371

4	(0.631) 0.642 0.629

Locus 15660 :

2 alleles

96.5% missing data

4	(0.732) 0.738 0.732

1	(0.268) 0.262 0.268

Locus 15661 :

2 alleles

99.2% missing data

4	(0.896) 0.896 0.891

1	(0.104) 0.104 0.109

Locus 15662 :

2 alleles

90.2% missing data

4	(0.930) 0.944 0.922

2	(0.070) 0.056 0.078

Locus 15663 :

2 alleles

99.2% missing data

1	(0.229) 0.231 0.228

3	(0.771) 0.769 0.772

Locus 15664 :

2 alleles

98.8% missing data

3	(0.667) 0.661 0.677

4	(0.333) 0.339 0.323

Locus 15665 :

2 alleles

99.2% missing data

1	(0.457) 0.461 0.454

4	(0.543) 0.539 0.546

Locus 15666 :

2 alleles

91.0% missing data

3	(0.947) 0.958 0.940

1	(0.053) 0.042 0.060

Locus 15667 :

2 alleles

98.8% missing data

4	(0.538) 0.543 0.538

2	(0.462) 0.457 0.462

Locus 15668 :

2 alleles

99.2% missing data

4	(0.418) 0.414 0.424

1	(0.582) 0.586 0.576

Locus 15669 :

2 alleles

99.2% missing data

2	(0.446) 0.444 0.442

4	(0.554) 0.556 0.558

Locus 15670 :

2 alleles

99.2% missing data

4	(0.354) 0.362 0.351

1	(0.646) 0.638 0.649

Locus 15671 :

2 alleles

90.6% missing data

4	(0.912) 0.919 0.912

3	(0.088) 0.081 0.088

Locus 15672 :

3 alleles

98.8% missing data

4	(0.680) 0.671 0.684

3	(0.173) 0.170 0.178

2	(0.146) 0.159 0.139

Locus 15673 :

2 alleles

99.2% missing data

3	(0.326) 0.322 0.326

2	(0.674) 0.678 0.674

Locus 15674 :

2 alleles

98.0% missing data

4	(0.644) 0.637 0.655

2	(0.356) 0.363 0.345

Locus 15675 :

2 alleles

96.9% missing data

1	(0.867) 0.861 0.879

3	(0.133) 0.139 0.121

Locus 15676 :

2 alleles

99.2% missing data

1	(0.695) 0.690 0.700

3	(0.305) 0.310 0.300

Locus 15677 :

3 alleles

97.3% missing data

2	(0.502) 0.501 0.504

3	(0.252) 0.245 0.262

1	(0.246) 0.254 0.234

Locus 15678 :

2 alleles

99.2% missing data

3	(0.308) 0.314 0.302

1	(0.692) 0.686 0.698

Locus 15679 :

2 alleles

99.2% missing data

4	(0.219) 0.221 0.216

1	(0.781) 0.779 0.784

Locus 15680 :

2 alleles

99.2% missing data

2	(0.730) 0.732 0.725

4	(0.270) 0.268 0.275

Locus 15681 :

2 alleles

8.2% missing data

4	(0.983) 0.974 0.995

1	(0.017) 0.026 0.005

Locus 15682 :

2 alleles

98.0% missing data

3	(0.463) 0.461 0.469

4	(0.537) 0.539 0.531

Locus 15683 :

2 alleles

99.2% missing data

4	(0.430) 0.430 0.437

1	(0.570) 0.570 0.563

Locus 15684 :

2 alleles

98.8% missing data

4	(0.376) 0.373 0.373

1	(0.624) 0.627 0.627

Locus 15685 :

3 alleles

89.5% missing data

3	(0.827) 0.858 0.811

2	(0.098) 0.082 0.103

4	(0.074) 0.060 0.086

Locus 15686 :

2 alleles

99.2% missing data

3	(0.516) 0.517 0.523

1	(0.484) 0.483 0.477

Locus 15687 :

2 alleles

99.2% missing data

4	(0.466) 0.461 0.475

1	(0.534) 0.539 0.525

Locus 15688 :

2 alleles

99.2% missing data

4	(0.734) 0.731 0.737

1	(0.266) 0.269 0.263

Locus 15689 :

2 alleles

99.2% missing data

4	(0.424) 0.427 0.421

1	(0.576) 0.573 0.579

Locus 15690 :

3 alleles

98.4% missing data

4	(0.582) 0.573 0.589

2	(0.202) 0.204 0.202

3	(0.217) 0.223 0.209

Locus 15691 :

2 alleles

99.2% missing data

3	(0.505) 0.515 0.496

1	(0.495) 0.485 0.504

Locus 15692 :

2 alleles

98.8% missing data

4	(0.122) 0.115 0.127

2	(0.878) 0.885 0.873

Locus 15693 :

2 alleles

99.2% missing data

3	(0.455) 0.447 0.455

4	(0.545) 0.553 0.545

Locus 15694 :

2 alleles

99.2% missing data

4	(0.653) 0.646 0.657

1	(0.347) 0.354 0.343

Locus 15695 :

2 alleles

98.8% missing data

1	(0.269) 0.263 0.276

4	(0.731) 0.737 0.724

Locus 15696 :

2 alleles

99.2% missing data

1	(0.150) 0.146 0.154

4	(0.850) 0.854 0.846

Locus 15697 :

2 alleles

96.1% missing data

3	(0.893) 0.901 0.882

1	(0.107) 0.099 0.118

Locus 15698 :

2 alleles

99.2% missing data

3	(0.481) 0.483 0.483

2	(0.519) 0.517 0.517

Locus 15699 :

3 alleles

98.0% missing data

1	(0.502) 0.514 0.501

3	(0.281) 0.271 0.286

2	(0.217) 0.215 0.213

Locus 15700 :

2 alleles

8.2% missing data

2	(0.984) 0.977 0.995

1	(0.016) 0.023 0.005

Locus 15701 :

2 alleles

99.2% missing data

2	(0.656) 0.654 0.657

1	(0.344) 0.346 0.343

Locus 15702 :

2 alleles

99.2% missing data

4	(0.338) 0.334 0.339

3	(0.662) 0.666 0.661

Locus 15703 :

2 alleles

91.0% missing data

4	(0.925) 0.940 0.916

2	(0.075) 0.060 0.084

Locus 15704 :

2 alleles

99.2% missing data

1	(0.393) 0.391 0.393

4	(0.607) 0.609 0.607

Locus 15705 :

3 alleles

98.8% missing data

2	(0.548) 0.537 0.552

3	(0.198) 0.197 0.201

4	(0.254) 0.265 0.247

Locus 15706 :

2 alleles

98.4% missing data

2	(0.397) 0.402 0.385

1	(0.603) 0.598 0.615

Locus 15707 :

2 alleles

91.0% missing data

2	(0.892) 0.915 0.882

3	(0.108) 0.085 0.118

Locus 15708 :

2 alleles

91.0% missing data

4	(0.860) 0.884 0.853

1	(0.140) 0.116 0.147

Locus 15709 :

2 alleles

98.8% missing data

4	(0.724) 0.728 0.720

2	(0.276) 0.272 0.280

Locus 15710 :

3 alleles

98.4% missing data

3	(0.322) 0.326 0.323

4	(0.222) 0.226 0.214

1	(0.455) 0.448 0.464

Locus 15711 :

2 alleles

98.8% missing data

3	(0.801) 0.795 0.804

2	(0.199) 0.205 0.196

Locus 15712 :

2 alleles

99.2% missing data

3	(0.717) 0.713 0.725

4	(0.283) 0.287 0.275

Locus 15713 :

2 alleles

99.2% missing data

2	(0.275) 0.275 0.283

3	(0.725) 0.725 0.717

Locus 15714 :

2 alleles

96.1% missing data

2	(0.761) 0.780 0.757

4	(0.239) 0.220 0.243

Locus 15715 :

2 alleles

99.2% missing data

1	(0.681) 0.675 0.680

3	(0.319) 0.325 0.320

Locus 15716 :

2 alleles

99.2% missing data

2	(0.433) 0.428 0.440

3	(0.567) 0.572 0.560

Locus 15717 :

2 alleles

99.2% missing data

2	(0.681) 0.683 0.682

3	(0.319) 0.317 0.318

Locus 15718 :

3 alleles

98.8% missing data

3	(0.193) 0.194 0.202

2	(0.175) 0.181 0.170

4	(0.631) 0.625 0.628

Locus 15719 :

2 alleles

96.5% missing data

1	(0.880) 0.887 0.873

2	(0.120) 0.113 0.127

Locus 15720 :

2 alleles

99.2% missing data

4	(0.563) 0.558 0.567

2	(0.437) 0.442 0.433

Locus 15721 :

2 alleles

99.2% missing data

2	(0.393) 0.395 0.404

4	(0.607) 0.605 0.596

Locus 15722 :

2 alleles

98.4% missing data

4	(0.660) 0.660 0.656

1	(0.340) 0.340 0.344

Locus 15723 :

2 alleles

99.2% missing data

4	(0.233) 0.231 0.236

3	(0.767) 0.769 0.764

Locus 15724 :

2 alleles

96.9% missing data

1	(0.685) 0.676 0.704

4	(0.315) 0.324 0.296

Locus 15725 :

2 alleles

98.8% missing data

2	(0.188) 0.181 0.196

3	(0.812) 0.819 0.804

Locus 15726 :

2 alleles

98.8% missing data

1	(0.277) 0.273 0.280

2	(0.723) 0.727 0.720

Locus 15727 :

2 alleles

98.8% missing data

4	(0.559) 0.562 0.560

2	(0.441) 0.438 0.440

Locus 15728 :

2 alleles

99.2% missing data

4	(0.523) 0.523 0.527

1	(0.477) 0.477 0.473

Locus 15729 :

2 alleles

98.8% missing data

1	(0.242) 0.252 0.235

2	(0.758) 0.748 0.765

Locus 15730 :

2 alleles

98.8% missing data

1	(0.754) 0.760 0.747

4	(0.246) 0.240 0.253

Locus 15731 :

2 alleles

98.8% missing data

4	(0.645) 0.639 0.647

3	(0.355) 0.361 0.353

Locus 15732 :

2 alleles

99.2% missing data

2	(0.774) 0.771 0.773

3	(0.226) 0.229 0.227

Locus 15733 :

2 alleles

99.2% missing data

4	(0.493) 0.493 0.493

3	(0.507) 0.507 0.507

Locus 15734 :

2 alleles

99.2% missing data

4	(0.700) 0.691 0.707

1	(0.300) 0.309 0.293

Locus 15735 :

2 alleles

99.2% missing data

4	(0.743) 0.742 0.740

3	(0.257) 0.258 0.260

Locus 15736 :

2 alleles

96.5% missing data

1	(0.862) 0.868 0.857

2	(0.138) 0.132 0.143

Locus 15737 :

2 alleles

99.2% missing data

4	(0.610) 0.616 0.605

3	(0.390) 0.384 0.395

Locus 15738 :

2 alleles

98.0% missing data

4	(0.696) 0.697 0.702

3	(0.304) 0.303 0.298

Locus 15739 :

2 alleles

98.0% missing data

4	(0.226) 0.228 0.234

3	(0.774) 0.772 0.766

Locus 15740 :

2 alleles

99.2% missing data

4	(0.537) 0.539 0.540

2	(0.463) 0.461 0.460

Locus 15741 :

2 alleles

96.9% missing data

4	(0.812) 0.814 0.810

3	(0.188) 0.186 0.190

Locus 15742 :

2 alleles

99.2% missing data

3	(0.386) 0.395 0.387

1	(0.614) 0.605 0.613

Locus 15743 :

2 alleles

99.2% missing data

4	(0.591) 0.584 0.592

2	(0.409) 0.416 0.408

Locus 15744 :

2 alleles

99.2% missing data

4	(0.511) 0.506 0.517

3	(0.489) 0.494 0.483

Locus 15745 :

2 alleles

98.8% missing data

4	(0.664) 0.659 0.674

1	(0.336) 0.341 0.326

Locus 15746 :

2 alleles

99.2% missing data

4	(0.607) 0.602 0.611

1	(0.393) 0.398 0.389

Locus 15747 :

2 alleles

98.8% missing data

1	(0.679) 0.683 0.674

3	(0.321) 0.317 0.326

Locus 15748 :

2 alleles

99.2% missing data

3	(0.437) 0.436 0.439

1	(0.563) 0.564 0.561

Locus 15749 :

2 alleles

99.2% missing data

4	(0.486) 0.488 0.484

1	(0.514) 0.512 0.516

Locus 15750 :

2 alleles

96.1% missing data

3	(0.930) 0.926 0.933

4	(0.070) 0.074 0.067

Locus 15751 :

2 alleles

98.8% missing data

1	(0.742) 0.747 0.738

4	(0.258) 0.253 0.262

Locus 15752 :

2 alleles

99.2% missing data

4	(0.336) 0.325 0.339

1	(0.664) 0.675 0.661

Locus 15753 :

2 alleles

97.3% missing data

1	(0.416) 0.420 0.404

3	(0.584) 0.580 0.596

Locus 15754 :

2 alleles

99.2% missing data

1	(0.287) 0.285 0.287

4	(0.713) 0.715 0.713

Locus 15755 :

2 alleles

98.0% missing data

1	(0.501) 0.504 0.511

3	(0.499) 0.496 0.489

Locus 15756 :

2 alleles

99.2% missing data

2	(0.205) 0.202 0.205

3	(0.795) 0.798 0.795

Locus 15757 :

2 alleles

99.2% missing data

3	(0.268) 0.275 0.265

4	(0.732) 0.725 0.735

Locus 15758 :

2 alleles

99.2% missing data

3	(0.611) 0.613 0.609

2	(0.389) 0.387 0.391

Locus 15759 :

2 alleles

98.8% missing data

2	(0.378) 0.382 0.373

4	(0.622) 0.618 0.627

Locus 15760 :

2 alleles

99.2% missing data

2	(0.209) 0.211 0.212

4	(0.791) 0.789 0.788

Locus 15761 :

3 alleles

98.8% missing data

2	(0.629) 0.636 0.618

4	(0.195) 0.190 0.202

3	(0.177) 0.173 0.180

Locus 15762 :

2 alleles

99.2% missing data

1	(0.460) 0.459 0.456

2	(0.540) 0.541 0.544

Locus 15763 :

2 alleles

98.4% missing data

4	(0.634) 0.635 0.632

2	(0.366) 0.365 0.368

Locus 15764 :

2 alleles

99.2% missing data

1	(0.231) 0.222 0.235

2	(0.769) 0.778 0.765

Locus 15765 :

3 alleles

96.1% missing data

1	(0.147) 0.143 0.148

4	(0.147) 0.147 0.145

2	(0.705) 0.710 0.706

Locus 15766 :

2 alleles

97.7% missing data

4	(0.310) 0.310 0.301

3	(0.690) 0.690 0.699

Locus 15767 :

2 alleles

99.2% missing data

1	(0.219) 0.214 0.223

3	(0.781) 0.786 0.777

Locus 15768 :

2 alleles

99.2% missing data

4	(0.676) 0.673 0.680

1	(0.324) 0.327 0.320

Locus 15769 :

2 alleles

99.2% missing data

3	(0.560) 0.552 0.564

4	(0.440) 0.448 0.436

Locus 15770 :

2 alleles

99.2% missing data

1	(0.452) 0.449 0.459

3	(0.548) 0.551 0.541

Locus 15771 :

2 alleles

99.2% missing data

3	(0.427) 0.430 0.424

4	(0.573) 0.570 0.576

Locus 15772 :

2 alleles

97.7% missing data

1	(0.206) 0.197 0.219

4	(0.794) 0.803 0.781

Locus 15773 :

2 alleles

99.2% missing data

4	(0.383) 0.375 0.392

1	(0.617) 0.625 0.608

Locus 15774 :

2 alleles

98.4% missing data

1	(0.560) 0.562 0.560

2	(0.440) 0.438 0.440

Locus 15775 :

2 alleles

98.8% missing data

4	(0.665) 0.668 0.664

1	(0.335) 0.332 0.336

Locus 15776 :

2 alleles

98.4% missing data

4	(0.530) 0.517 0.531

1	(0.470) 0.483 0.469

Locus 15777 :

2 alleles

99.2% missing data

4	(0.662) 0.661 0.670

2	(0.338) 0.339 0.330

Locus 15778 :

2 alleles

99.2% missing data

4	(0.448) 0.458 0.437

3	(0.552) 0.542 0.563

Locus 15779 :

2 alleles

99.2% missing data

4	(0.527) 0.536 0.526

2	(0.473) 0.464 0.474

Locus 15780 :

2 alleles

97.7% missing data

4	(0.322) 0.307 0.327

2	(0.678) 0.693 0.673

Locus 15781 :

2 alleles

96.1% missing data

4	(0.758) 0.762 0.762

1	(0.242) 0.238 0.238

Locus 15782 :

2 alleles

99.2% missing data

3	(0.408) 0.419 0.406

1	(0.592) 0.581 0.594

Locus 15783 :

2 alleles

99.2% missing data

2	(0.702) 0.705 0.694

1	(0.298) 0.295 0.306

Locus 15784 :

2 alleles

99.2% missing data

4	(0.394) 0.392 0.394

2	(0.606) 0.608 0.606

Locus 15785 :

2 alleles

93.4% missing data

1	(0.894) 0.886 0.904

2	(0.106) 0.114 0.096

Locus 15786 :

2 alleles

99.2% missing data

2	(0.477) 0.472 0.486

1	(0.523) 0.528 0.514

Locus 15787 :

2 alleles

99.2% missing data

2	(0.409) 0.410 0.409

3	(0.591) 0.590 0.591

Locus 15788 :

2 alleles

98.4% missing data

2	(0.647) 0.636 0.657

1	(0.353) 0.364 0.343

Locus 15789 :

2 alleles

99.2% missing data

2	(0.539) 0.534 0.541

1	(0.461) 0.466 0.459

Locus 15790 :

2 alleles

99.2% missing data

3	(0.555) 0.554 0.550

1	(0.445) 0.446 0.450

Locus 15791 :

2 alleles

99.2% missing data

1	(0.338) 0.337 0.336

3	(0.662) 0.663 0.664

Locus 15792 :

2 alleles

99.2% missing data

1	(0.597) 0.594 0.591

3	(0.403) 0.406 0.409

Locus 15793 :

2 alleles

98.8% missing data

4	(0.553) 0.549 0.563

3	(0.447) 0.451 0.437

Locus 15794 :

2 alleles

99.2% missing data

2	(0.519) 0.517 0.520

1	(0.481) 0.483 0.480

Locus 15795 :

2 alleles

99.2% missing data

1	(0.559) 0.555 0.565

4	(0.441) 0.445 0.435

Locus 15796 :

2 alleles

98.0% missing data

1	(0.218) 0.215 0.223

4	(0.782) 0.785 0.777

Locus 15797 :

2 alleles

99.2% missing data

3	(0.760) 0.763 0.757

1	(0.240) 0.237 0.243

Locus 15798 :

2 alleles

99.2% missing data

1	(0.376) 0.376 0.387

3	(0.624) 0.624 0.613

Locus 15799 :

2 alleles

98.8% missing data

4	(0.674) 0.682 0.670

3	(0.326) 0.318 0.330

Locus 15800 :

2 alleles

98.0% missing data

1	(0.525) 0.521 0.525

3	(0.475) 0.479 0.475

Locus 15801 :

2 alleles

99.2% missing data

4	(0.769) 0.765 0.768

3	(0.231) 0.235 0.232

Locus 15802 :

2 alleles

99.2% missing data

1	(0.570) 0.575 0.565

3	(0.430) 0.425 0.435

Locus 15803 :

2 alleles

98.8% missing data

4	(0.757) 0.758 0.758

2	(0.243) 0.242 0.242

Locus 15804 :

2 alleles

99.2% missing data

1	(0.519) 0.515 0.521

3	(0.481) 0.485 0.479

Locus 15805 :

2 alleles

99.2% missing data

4	(0.616) 0.620 0.613

2	(0.384) 0.380 0.387

Locus 15806 :

2 alleles

99.2% missing data

2	(0.476) 0.477 0.479

3	(0.524) 0.523 0.521

Locus 15807 :

2 alleles

98.0% missing data

4	(0.654) 0.662 0.648

2	(0.346) 0.338 0.352

Locus 15808 :

2 alleles

99.2% missing data

4	(0.344) 0.339 0.346

1	(0.656) 0.661 0.654

Locus 15809 :

3 alleles

98.0% missing data

4	(0.206) 0.214 0.196

2	(0.390) 0.378 0.400

3	(0.405) 0.408 0.404

Locus 15810 :

2 alleles

99.2% missing data

2	(0.343) 0.347 0.339

4	(0.657) 0.653 0.661

Locus 15811 :

2 alleles

99.2% missing data

2	(0.603) 0.597 0.609

1	(0.397) 0.403 0.391

Locus 15812 :

2 alleles

99.2% missing data

3	(0.312) 0.309 0.316

2	(0.688) 0.691 0.684

Locus 15813 :

2 alleles

99.2% missing data

4	(0.727) 0.720 0.731

3	(0.273) 0.280 0.269

Locus 15814 :

2 alleles

98.4% missing data

4	(0.419) 0.416 0.418

3	(0.581) 0.584 0.582

Locus 15815 :

2 alleles

98.8% missing data

2	(0.820) 0.813 0.825

4	(0.180) 0.187 0.175

Locus 15816 :

2 alleles

99.2% missing data

2	(0.711) 0.705 0.714

4	(0.289) 0.295 0.286

Locus 15817 :

2 alleles

99.2% missing data

2	(0.436) 0.431 0.438

3	(0.564) 0.569 0.562

Locus 15818 :

2 alleles

99.2% missing data

4	(0.718) 0.711 0.720

1	(0.282) 0.289 0.280

Locus 15819 :

2 alleles

99.2% missing data

2	(0.425) 0.426 0.425

4	(0.575) 0.574 0.575

Locus 15820 :

2 alleles

99.2% missing data

4	(0.522) 0.521 0.519

1	(0.478) 0.479 0.481

Locus 15821 :

2 alleles

99.2% missing data

3	(0.391) 0.393 0.386

2	(0.609) 0.607 0.614

Locus 15822 :

2 alleles

90.6% missing data

4	(0.897) 0.916 0.889

1	(0.103) 0.084 0.111

Locus 15823 :

2 alleles

99.2% missing data

3	(0.570) 0.566 0.572

2	(0.430) 0.434 0.428

Locus 15824 :

2 alleles

91.0% missing data

1	(0.874) 0.893 0.869

2	(0.126) 0.107 0.131

Locus 15825 :

2 alleles

89.5% missing data

2	(0.867) 0.888 0.853

1	(0.133) 0.112 0.147

Locus 15826 :

2 alleles

98.8% missing data

2	(0.593) 0.596 0.598

4	(0.407) 0.404 0.402

Locus 15827 :

2 alleles

99.2% missing data

1	(0.558) 0.553 0.559

2	(0.442) 0.447 0.441

Locus 15828 :

2 alleles

91.4% missing data

1	(0.938) 0.944 0.940

2	(0.062) 0.056 0.060

Locus 15829 :

2 alleles

98.8% missing data

4	(0.341) 0.346 0.337

2	(0.659) 0.654 0.663

Locus 15830 :

2 alleles

99.2% missing data

2	(0.352) 0.348 0.359

1	(0.648) 0.652 0.641

Locus 15831 :

2 alleles

98.0% missing data

1	(0.330) 0.326 0.333

2	(0.670) 0.674 0.667

Locus 15832 :

2 alleles

91.0% missing data

2	(0.897) 0.915 0.887

1	(0.103) 0.085 0.113

Locus 15833 :

2 alleles

99.2% missing data

4	(0.585) 0.588 0.584

3	(0.415) 0.412 0.416

Locus 15834 :

2 alleles

99.2% missing data

3	(0.515) 0.509 0.511

2	(0.485) 0.491 0.489

Locus 15835 :

2 alleles

99.2% missing data

2	(0.405) 0.410 0.408

3	(0.595) 0.590 0.592

Locus 15836 :

2 alleles

99.2% missing data

1	(0.339) 0.333 0.343

3	(0.661) 0.667 0.657

Locus 15837 :

2 alleles

98.8% missing data

3	(0.538) 0.532 0.544

4	(0.462) 0.468 0.456

Locus 15838 :

2 alleles

99.2% missing data

2	(0.704) 0.700 0.703

1	(0.296) 0.300 0.297

Locus 15839 :

2 alleles

98.8% missing data

4	(0.459) 0.469 0.454

2	(0.541) 0.531 0.546

Locus 15840 :

2 alleles

99.2% missing data

4	(0.518) 0.514 0.522

2	(0.482) 0.486 0.478

Locus 15841 :

2 alleles

99.2% missing data

4	(0.595) 0.586 0.597

3	(0.405) 0.414 0.403

Locus 15842 :

2 alleles

99.2% missing data

1	(0.092) 0.088 0.098

4	(0.908) 0.912 0.902

Locus 15843 :

2 alleles

99.2% missing data

3	(0.162) 0.167 0.161

4	(0.838) 0.833 0.839

Locus 15844 :

2 alleles

99.2% missing data

3	(0.453) 0.449 0.452

2	(0.547) 0.551 0.548

Locus 15845 :

2 alleles

99.2% missing data

1	(0.345) 0.347 0.349

4	(0.655) 0.653 0.651

Locus 15846 :

2 alleles

99.2% missing data

1	(0.753) 0.750 0.759

2	(0.247) 0.250 0.241

Locus 15847 :

2 alleles

98.8% missing data

2	(0.687) 0.684 0.696

1	(0.313) 0.316 0.304

Locus 15848 :

2 alleles

99.2% missing data

3	(0.411) 0.407 0.410

1	(0.589) 0.593 0.590

Locus 15849 :

2 alleles

99.2% missing data

3	(0.678) 0.676 0.680

4	(0.322) 0.324 0.320

Locus 15850 :

2 alleles

99.2% missing data

4	(0.475) 0.467 0.481

1	(0.525) 0.533 0.519

Locus 15851 :

2 alleles

99.2% missing data

4	(0.392) 0.396 0.390

3	(0.608) 0.604 0.610

Locus 15852 :

2 alleles

99.2% missing data

4	(0.710) 0.699 0.709

2	(0.290) 0.301 0.291

Locus 15853 :

2 alleles

99.2% missing data

3	(0.562) 0.560 0.562

2	(0.438) 0.440 0.438

Locus 15854 :

2 alleles

99.2% missing data

4	(0.496) 0.491 0.500

3	(0.504) 0.509 0.500

Locus 15855 :

3 alleles

97.3% missing data

4	(0.681) 0.679 0.678

2	(0.120) 0.120 0.125

1	(0.199) 0.201 0.197

Locus 15856 :

2 alleles

95.3% missing data

4	(0.853) 0.860 0.852

3	(0.147) 0.140 0.148

Locus 15857 :

2 alleles

99.2% missing data

3	(0.411) 0.412 0.405

4	(0.589) 0.588 0.595

Locus 15858 :

2 alleles

99.2% missing data

1	(0.261) 0.260 0.258

2	(0.739) 0.740 0.742

Locus 15859 :

2 alleles

99.2% missing data

4	(0.596) 0.596 0.595

3	(0.404) 0.404 0.405

Locus 15860 :

3 alleles

98.4% missing data

1	(0.303) 0.308 0.306

3	(0.216) 0.215 0.224

2	(0.480) 0.476 0.470

Locus 15861 :

2 alleles

99.2% missing data

4	(0.393) 0.389 0.399

1	(0.607) 0.611 0.601

Locus 15862 :

3 alleles

96.1% missing data

1	(0.768) 0.762 0.777

2	(0.085) 0.096 0.080

3	(0.147) 0.142 0.144

Locus 15863 :

2 alleles

98.8% missing data

3	(0.495) 0.501 0.492

4	(0.505) 0.499 0.508

Locus 15864 :

2 alleles

98.4% missing data

2	(0.577) 0.588 0.571

1	(0.423) 0.412 0.429

Locus 15865 :

2 alleles

99.2% missing data

4	(0.532) 0.534 0.531

3	(0.468) 0.466 0.469

Locus 15866 :

2 alleles

99.2% missing data

2	(0.395) 0.391 0.402

1	(0.605) 0.609 0.598

Locus 15867 :

2 alleles

99.2% missing data

4	(0.336) 0.336 0.338

1	(0.664) 0.664 0.662

Locus 15868 :

2 alleles

99.2% missing data

3	(0.344) 0.335 0.350

1	(0.656) 0.665 0.650

Locus 15869 :

2 alleles

99.2% missing data

3	(0.615) 0.618 0.611

1	(0.385) 0.382 0.389

Locus 15870 :

2 alleles

99.2% missing data

4	(0.347) 0.347 0.345

3	(0.653) 0.653 0.655

Locus 15871 :

2 alleles

98.8% missing data

2	(0.660) 0.658 0.661

1	(0.340) 0.342 0.339

Locus 15872 :

2 alleles

99.2% missing data

3	(0.386) 0.391 0.388

4	(0.614) 0.609 0.612

Locus 15873 :

2 alleles

99.2% missing data

4	(0.258) 0.259 0.259

2	(0.742) 0.741 0.741

Locus 15874 :

2 alleles

99.2% missing data

4	(0.692) 0.702 0.684

3	(0.308) 0.298 0.316

Locus 15875 :

2 alleles

99.2% missing data

2	(0.596) 0.588 0.593

1	(0.404) 0.412 0.407

Locus 15876 :

2 alleles

99.2% missing data

3	(0.334) 0.336 0.338

1	(0.666) 0.664 0.662

Locus 15877 :

2 alleles

99.2% missing data

3	(0.523) 0.518 0.520

4	(0.477) 0.482 0.480

Locus 15878 :

2 alleles

99.2% missing data

3	(0.388) 0.386 0.396

4	(0.612) 0.614 0.604

Locus 15879 :

2 alleles

99.2% missing data

4	(0.646) 0.645 0.644

3	(0.354) 0.355 0.356

Locus 15880 :

2 alleles

99.2% missing data

4	(0.441) 0.444 0.443

1	(0.559) 0.556 0.557

Locus 15881 :

2 alleles

99.2% missing data

4	(0.607) 0.611 0.600

1	(0.393) 0.389 0.400

Locus 15882 :

2 alleles

99.2% missing data

3	(0.517) 0.510 0.519

2	(0.483) 0.490 0.481

Locus 15883 :

2 alleles

96.1% missing data

4	(0.835) 0.844 0.833

1	(0.165) 0.156 0.167

Locus 15884 :

2 alleles

98.8% missing data

3	(0.441) 0.439 0.448

1	(0.559) 0.561 0.552

Locus 15885 :

2 alleles

99.2% missing data

2	(0.248) 0.243 0.253

1	(0.752) 0.757 0.747

Locus 15886 :

2 alleles

99.2% missing data

3	(0.656) 0.651 0.653

1	(0.344) 0.349 0.347

Locus 15887 :

2 alleles

99.2% missing data

3	(0.262) 0.260 0.262

4	(0.738) 0.740 0.738

Locus 15888 :

2 alleles

91.4% missing data

3	(0.950) 0.955 0.949

2	(0.050) 0.045 0.051

Locus 15889 :

2 alleles

99.2% missing data

3	(0.446) 0.450 0.440

2	(0.554) 0.550 0.560

Locus 15890 :

2 alleles

99.2% missing data

4	(0.648) 0.649 0.641

1	(0.352) 0.351 0.359

Locus 15891 :

3 alleles

96.5% missing data

1	(0.543) 0.541 0.553

3	(0.342) 0.346 0.330

4	(0.115) 0.113 0.117

Locus 15892 :

2 alleles

98.0% missing data

1	(0.894) 0.894 0.892

3	(0.106) 0.106 0.108

Locus 15893 :

2 alleles

97.7% missing data

1	(0.291) 0.289 0.289

3	(0.709) 0.711 0.711

Locus 15894 :

3 alleles

96.5% missing data

3	(0.684) 0.682 0.698

1	(0.153) 0.150 0.148

4	(0.163) 0.168 0.154

Locus 15895 :

2 alleles

99.2% missing data

1	(0.502) 0.505 0.496

3	(0.498) 0.495 0.504

Locus 15896 :

2 alleles

98.0% missing data

1	(0.749) 0.753 0.748

4	(0.251) 0.247 0.252

Locus 15897 :

2 alleles

98.8% missing data

3	(0.430) 0.417 0.432

1	(0.570) 0.583 0.568

Locus 15898 :

2 alleles

95.7% missing data

4	(0.143) 0.129 0.161

1	(0.857) 0.871 0.839

Locus 15899 :

2 alleles

99.2% missing data

3	(0.711) 0.705 0.718

4	(0.289) 0.295 0.282

Locus 15900 :

2 alleles

99.2% missing data

1	(0.558) 0.557 0.564

4	(0.442) 0.443 0.436

Locus 15901 :

2 alleles

99.2% missing data

1	(0.693) 0.688 0.693

4	(0.307) 0.312 0.307

Locus 15902 :

3 alleles

90.2% missing data

3	(0.813) 0.845 0.796

2	(0.071) 0.058 0.079

1	(0.116) 0.098 0.124

Locus 15903 :

2 alleles

99.2% missing data

1	(0.204) 0.196 0.215

4	(0.796) 0.804 0.785

Locus 15904 :

2 alleles

99.2% missing data

2	(0.336) 0.334 0.341

1	(0.664) 0.666 0.659

Locus 15905 :

2 alleles

99.2% missing data

2	(0.706) 0.700 0.706

3	(0.294) 0.300 0.294

Locus 15906 :

2 alleles

12.9% missing data

3	(0.988) 0.985 0.995

2	(0.012) 0.015 0.005

Locus 15907 :

2 alleles

8.6% missing data

2	(0.985) 0.978 0.996

4	(0.015) 0.022 0.004

Locus 15908 :

2 alleles

96.5% missing data

1	(0.230) 0.212 0.235

3	(0.770) 0.788 0.765

Locus 15909 :

2 alleles

96.5% missing data

4	(0.659) 0.670 0.652

1	(0.341) 0.330 0.348

Locus 15910 :

2 alleles

99.2% missing data

2	(0.531) 0.531 0.534

4	(0.469) 0.469 0.466

Locus 15911 :

2 alleles

96.1% missing data

1	(0.756) 0.771 0.751

4	(0.244) 0.229 0.249

Locus 15912 :

2 alleles

99.2% missing data

3	(0.539) 0.535 0.547

2	(0.461) 0.465 0.453

Locus 15913 :

2 alleles

94.5% missing data

1	(0.868) 0.881 0.861

4	(0.132) 0.119 0.139

Locus 15914 :

2 alleles

98.4% missing data

4	(0.620) 0.631 0.611

2	(0.380) 0.369 0.389

Locus 15915 :

2 alleles

95.7% missing data

4	(0.153) 0.141 0.161

2	(0.847) 0.859 0.839

Locus 15916 :

2 alleles

99.2% missing data

3	(0.539) 0.535 0.544

4	(0.461) 0.465 0.456

Locus 15917 :

2 alleles

98.8% missing data

2	(0.444) 0.450 0.446

1	(0.556) 0.550 0.554

Locus 15918 :

2 alleles

99.2% missing data

1	(0.680) 0.679 0.676

4	(0.320) 0.321 0.324

Locus 15919 :

2 alleles

97.7% missing data

4	(0.566) 0.577 0.565

1	(0.434) 0.423 0.435

Locus 15920 :

2 alleles

99.2% missing data

1	(0.488) 0.482 0.498

3	(0.512) 0.518 0.502

Locus 15921 :

2 alleles

99.2% missing data

2	(0.628) 0.626 0.622

3	(0.372) 0.374 0.378

Locus 15922 :

2 alleles

98.8% missing data

2	(0.608) 0.597 0.618

1	(0.392) 0.403 0.382

Locus 15923 :

2 alleles

99.2% missing data

1	(0.670) 0.668 0.670

3	(0.330) 0.332 0.330

Locus 15924 :

2 alleles

99.2% missing data

4	(0.676) 0.671 0.677

1	(0.324) 0.329 0.323

Locus 15925 :

3 alleles

98.0% missing data

1	(0.639) 0.642 0.637

3	(0.149) 0.144 0.155

2	(0.212) 0.214 0.208

Locus 15926 :

2 alleles

99.2% missing data

3	(0.499) 0.499 0.494

1	(0.501) 0.501 0.506

Locus 15927 :

2 alleles

99.2% missing data

3	(0.293) 0.288 0.297

1	(0.707) 0.712 0.703

Locus 15928 :

2 alleles

93.0% missing data

2	(0.901) 0.905 0.905

1	(0.099) 0.095 0.095

Locus 15929 :

2 alleles

99.2% missing data

3	(0.471) 0.472 0.468

2	(0.529) 0.528 0.532

Locus 15930 :

2 alleles

96.9% missing data

1	(0.796) 0.801 0.798

4	(0.204) 0.199 0.202

Locus 15931 :

2 alleles

98.8% missing data

3	(0.686) 0.676 0.698

1	(0.314) 0.324 0.302

Locus 15932 :

3 alleles

97.3% missing data

3	(0.634) 0.644 0.632

1	(0.236) 0.227 0.240

4	(0.130) 0.129 0.128

Locus 15933 :

2 alleles

99.2% missing data

1	(0.668) 0.666 0.666

4	(0.332) 0.334 0.334

Locus 15934 :

2 alleles

99.2% missing data

4	(0.548) 0.545 0.555

3	(0.452) 0.455 0.445

Locus 15935 :

2 alleles

98.8% missing data

2	(0.579) 0.585 0.576

3	(0.421) 0.415 0.424

Locus 15936 :

2 alleles

98.4% missing data

3	(0.527) 0.540 0.520

4	(0.473) 0.460 0.480

Locus 15937 :

2 alleles

98.8% missing data

4	(0.598) 0.604 0.598

3	(0.402) 0.396 0.402

Locus 15938 :

2 alleles

98.8% missing data

2	(0.644) 0.630 0.650

3	(0.356) 0.370 0.350

Locus 15939 :

2 alleles

99.2% missing data

2	(0.472) 0.479 0.469

1	(0.528) 0.521 0.531

Locus 15940 :

2 alleles

99.2% missing data

2	(0.617) 0.607 0.623

4	(0.383) 0.393 0.377

Locus 15941 :

2 alleles

96.9% missing data

1	(0.851) 0.863 0.845

3	(0.149) 0.137 0.155

Locus 15942 :

2 alleles

99.2% missing data

3	(0.292) 0.293 0.295

2	(0.708) 0.707 0.705

Locus 15943 :

2 alleles

99.2% missing data

2	(0.457) 0.453 0.458

3	(0.543) 0.547 0.542

Locus 15944 :

2 alleles

41.4% missing data

4	(0.979) 0.976 0.990

2	(0.021) 0.024 0.010

Locus 15945 :

2 alleles

99.2% missing data

2	(0.535) 0.542 0.535

4	(0.465) 0.458 0.465

Locus 15946 :

2 alleles

99.2% missing data

3	(0.619) 0.619 0.618

4	(0.381) 0.381 0.382

Locus 15947 :

2 alleles

99.2% missing data

3	(0.639) 0.648 0.632

2	(0.361) 0.352 0.368

Locus 15948 :

2 alleles

5.1% missing data

3	(0.981) 0.967 0.994

1	(0.019) 0.033 0.006

Locus 15949 :

2 alleles

99.2% missing data

1	(0.590) 0.586 0.593

3	(0.410) 0.414 0.407

Locus 15950 :

2 alleles

98.0% missing data

4	(0.843) 0.845 0.846

3	(0.157) 0.155 0.154

Locus 15951 :

2 alleles

98.8% missing data

1	(0.484) 0.479 0.486

3	(0.516) 0.521 0.514

Locus 15952 :

2 alleles

98.4% missing data

2	(0.518) 0.518 0.530

3	(0.482) 0.482 0.470

Locus 15953 :

2 alleles

99.2% missing data

1	(0.421) 0.425 0.420

4	(0.579) 0.575 0.580

Locus 15954 :

2 alleles

98.0% missing data

3	(0.903) 0.906 0.898

1	(0.097) 0.094 0.102

Locus 15955 :

2 alleles

0.0% missing data

1	(0.988) 0.990 0.994

4	(0.012) 0.010 0.006

Locus 15956 :

2 alleles

99.2% missing data

3	(0.463) 0.461 0.468

2	(0.537) 0.539 0.532

Locus 15957 :

2 alleles

99.2% missing data

2	(0.619) 0.617 0.620

1	(0.381) 0.383 0.380

Locus 15958 :

2 alleles

99.2% missing data

3	(0.363) 0.359 0.367

4	(0.637) 0.641 0.633

Locus 15959 :

2 alleles

99.2% missing data

1	(0.579) 0.575 0.578

2	(0.421) 0.425 0.422

Locus 15960 :

2 alleles

99.2% missing data

1	(0.166) 0.165 0.172

4	(0.834) 0.835 0.828

Locus 15961 :

2 alleles

99.2% missing data

4	(0.219) 0.225 0.220

1	(0.781) 0.775 0.780

Locus 15962 :

2 alleles

98.4% missing data

1	(0.299) 0.288 0.307

2	(0.701) 0.712 0.693

Locus 15963 :

2 alleles

99.2% missing data

3	(0.359) 0.359 0.363

4	(0.641) 0.641 0.637

Locus 15964 :

2 alleles

98.8% missing data

3	(0.639) 0.639 0.639

1	(0.361) 0.361 0.361

Locus 15965 :

3 alleles

98.4% missing data

4	(0.436) 0.436 0.444

2	(0.188) 0.185 0.189

3	(0.375) 0.378 0.367

Locus 15966 :

2 alleles

99.2% missing data

1	(0.579) 0.589 0.577

3	(0.421) 0.411 0.423

Locus 15967 :

3 alleles

98.8% missing data

3	(0.138) 0.136 0.140

1	(0.321) 0.324 0.316

2	(0.541) 0.540 0.544

Locus 15968 :

2 alleles

99.2% missing data

3	(0.346) 0.347 0.348

4	(0.654) 0.653 0.652

Locus 15969 :

2 alleles

96.1% missing data

2	(0.148) 0.141 0.155

1	(0.852) 0.859 0.845

Locus 15970 :

3 alleles

95.7% missing data

1	(0.129) 0.117 0.135

4	(0.610) 0.620 0.607

2	(0.261) 0.263 0.258

Locus 15971 :

2 alleles

98.0% missing data

1	(0.817) 0.817 0.824

3	(0.183) 0.183 0.176

Locus 15972 :

2 alleles

98.4% missing data

3	(0.595) 0.600 0.597

4	(0.405) 0.400 0.403

Locus 15973 :

2 alleles

99.2% missing data

4	(0.611) 0.609 0.614

3	(0.389) 0.391 0.386

Locus 15974 :

2 alleles

99.2% missing data

4	(0.395) 0.397 0.399

3	(0.605) 0.603 0.601

Locus 15975 :

2 alleles

91.0% missing data

4	(0.921) 0.935 0.913

3	(0.079) 0.065 0.087

Locus 15976 :

2 alleles

97.7% missing data

4	(0.698) 0.699 0.691

1	(0.302) 0.301 0.309

Locus 15977 :

2 alleles

99.2% missing data

3	(0.490) 0.489 0.488

4	(0.510) 0.511 0.512

Locus 15978 :

2 alleles

96.1% missing data

4	(0.171) 0.159 0.178

1	(0.829) 0.841 0.822

Locus 15979 :

2 alleles

99.2% missing data

1	(0.347) 0.343 0.346

3	(0.653) 0.657 0.654

Locus 15980 :

2 alleles

99.2% missing data

4	(0.587) 0.587 0.593

1	(0.413) 0.413 0.407

Locus 15981 :

2 alleles

96.1% missing data

1	(0.791) 0.796 0.785

4	(0.209) 0.204 0.215

Locus 15982 :

2 alleles

93.0% missing data

3	(0.886) 0.886 0.893

1	(0.114) 0.114 0.107

Locus 15983 :

2 alleles

99.2% missing data

1	(0.422) 0.416 0.427

4	(0.578) 0.584 0.573

Locus 15984 :

2 alleles

99.2% missing data

1	(0.419) 0.421 0.421

3	(0.581) 0.579 0.579

Locus 15985 :

3 alleles

98.4% missing data

1	(0.418) 0.408 0.421

3	(0.322) 0.324 0.326

4	(0.260) 0.268 0.252

Locus 15986 :

2 alleles

91.0% missing data

4	(0.873) 0.898 0.865

1	(0.127) 0.102 0.135

Locus 15987 :

2 alleles

98.8% missing data

3	(0.547) 0.548 0.541

1	(0.453) 0.452 0.459

Locus 15988 :

2 alleles

98.0% missing data

4	(0.465) 0.459 0.461

1	(0.535) 0.541 0.539

Locus 15989 :

2 alleles

99.2% missing data

1	(0.391) 0.394 0.390

4	(0.609) 0.606 0.610

Locus 15990 :

2 alleles

99.2% missing data

4	(0.550) 0.543 0.556

2	(0.450) 0.457 0.444

Locus 15991 :

2 alleles

97.7% missing data

3	(0.798) 0.802 0.800

4	(0.202) 0.198 0.200

Locus 15992 :

2 alleles

99.2% missing data

3	(0.781) 0.776 0.785

2	(0.219) 0.224 0.215

Locus 15993 :

2 alleles

97.7% missing data

1	(0.653) 0.653 0.654

4	(0.347) 0.347 0.346

Locus 15994 :

2 alleles

99.2% missing data

1	(0.435) 0.432 0.440

3	(0.565) 0.568 0.560

Locus 15995 :

2 alleles

99.2% missing data

4	(0.373) 0.371 0.373

1	(0.627) 0.629 0.627

Locus 15996 :

2 alleles

99.2% missing data

4	(0.606) 0.606 0.598

3	(0.394) 0.394 0.402

Locus 15997 :

3 alleles

92.6% missing data

1	(0.260) 0.257 0.261

4	(0.650) 0.647 0.661

2	(0.090) 0.096 0.079

Locus 15998 :

2 alleles

8.2% missing data

4	(0.980) 0.973 0.994

2	(0.020) 0.027 0.006

Locus 15999 :

2 alleles

98.8% missing data

2	(0.649) 0.643 0.650

1	(0.351) 0.357 0.350

Locus 16000 :

2 alleles

99.2% missing data

4	(0.398) 0.395 0.399

1	(0.602) 0.605 0.601

Locus 16001 :

2 alleles

99.2% missing data

4	(0.511) 0.505 0.520

1	(0.489) 0.495 0.480

Locus 16002 :

2 alleles

97.7% missing data

1	(0.664) 0.671 0.660

2	(0.336) 0.329 0.340

Locus 16003 :

2 alleles

99.2% missing data

1	(0.823) 0.827 0.817

4	(0.177) 0.173 0.183

Locus 16004 :

2 alleles

99.2% missing data

4	(0.479) 0.481 0.483

3	(0.521) 0.519 0.517

Locus 16005 :

2 alleles

99.2% missing data

4	(0.555) 0.554 0.558

1	(0.445) 0.446 0.442

Locus 16006 :

2 alleles

98.8% missing data

3	(0.504) 0.505 0.507

1	(0.496) 0.495 0.493

Locus 16007 :

2 alleles

97.7% missing data

4	(0.636) 0.631 0.654

1	(0.364) 0.369 0.346

Locus 16008 :

2 alleles

98.8% missing data

1	(0.165) 0.160 0.171

4	(0.835) 0.840 0.829

Locus 16009 :

2 alleles

95.3% missing data

1	(0.844) 0.852 0.839

4	(0.156) 0.148 0.161

Locus 16010 :

2 alleles

92.2% missing data

2	(0.833) 0.812 0.857

1	(0.167) 0.188 0.143

Locus 16011 :

2 alleles

98.8% missing data

4	(0.804) 0.797 0.807

3	(0.196) 0.203 0.193

Locus 16012 :

2 alleles

91.0% missing data

1	(0.905) 0.922 0.896

4	(0.095) 0.078 0.104

Locus 16013 :

2 alleles

99.2% missing data

4	(0.613) 0.605 0.615

3	(0.387) 0.395 0.385

Locus 16014 :

2 alleles

97.7% missing data

2	(0.298) 0.304 0.289

3	(0.702) 0.696 0.711

Locus 16015 :

2 alleles

93.8% missing data

3	(0.853) 0.848 0.866

2	(0.147) 0.152 0.134

Locus 16016 :

2 alleles

0.0% missing data

3	(0.984) 0.988 0.992

4	(0.016) 0.012 0.008

Locus 16017 :

2 alleles

99.2% missing data

2	(0.366) 0.368 0.364

4	(0.634) 0.632 0.636

Locus 16018 :

2 alleles

99.2% missing data

4	(0.482) 0.477 0.486

2	(0.518) 0.523 0.514

Locus 16019 :

2 alleles

99.2% missing data

4	(0.622) 0.623 0.626

1	(0.378) 0.377 0.374

Locus 16020 :

2 alleles

98.8% missing data

3	(0.534) 0.527 0.542

1	(0.466) 0.473 0.458

Locus 16021 :

2 alleles

99.2% missing data

4	(0.663) 0.666 0.661

3	(0.337) 0.334 0.339

Locus 16022 :

3 alleles

98.8% missing data

1	(0.298) 0.288 0.299

4	(0.446) 0.452 0.433

2	(0.256) 0.260 0.268

Locus 16023 :

2 alleles

99.2% missing data

4	(0.697) 0.690 0.699

2	(0.303) 0.310 0.301

Locus 16024 :

2 alleles

98.8% missing data

3	(0.235) 0.229 0.239

4	(0.765) 0.771 0.761

Locus 16025 :

2 alleles

99.2% missing data

2	(0.660) 0.665 0.658

4	(0.340) 0.335 0.342

Locus 16026 :

3 alleles

96.9% missing data

3	(0.155) 0.159 0.141

4	(0.182) 0.197 0.175

1	(0.663) 0.644 0.684

Locus 16027 :

2 alleles

99.2% missing data

4	(0.423) 0.422 0.427

2	(0.577) 0.578 0.573

Locus 16028 :

2 alleles

99.2% missing data

1	(0.580) 0.576 0.582

2	(0.420) 0.424 0.418

Locus 16029 :

2 alleles

0.0% missing data

3	(0.980) 0.960 0.993

4	(0.020) 0.040 0.007

Locus 16030 :

2 alleles

99.2% missing data

4	(0.341) 0.338 0.341

2	(0.659) 0.662 0.659

Locus 16031 :

2 alleles

98.8% missing data

2	(0.498) 0.499 0.496

1	(0.502) 0.501 0.504

Locus 16032 :

2 alleles

99.2% missing data

3	(0.273) 0.274 0.280

4	(0.727) 0.726 0.720

Locus 16033 :

2 alleles

99.2% missing data

3	(0.599) 0.593 0.605

1	(0.401) 0.407 0.395

Locus 16034 :

2 alleles

98.8% missing data

2	(0.758) 0.759 0.758

4	(0.242) 0.241 0.242

Locus 16035 :

2 alleles

99.2% missing data

4	(0.576) 0.579 0.571

2	(0.424) 0.421 0.429

Locus 16036 :

2 alleles

99.2% missing data

2	(0.279) 0.273 0.281

1	(0.721) 0.727 0.719

Locus 16037 :

2 alleles

97.7% missing data

2	(0.479) 0.479 0.477

4	(0.521) 0.521 0.523

Locus 16038 :

2 alleles

98.8% missing data

4	(0.832) 0.838 0.827

2	(0.168) 0.162 0.173

Locus 16039 :

3 alleles

78.1% missing data

4	(0.906) 0.917 0.910

2	(0.041) 0.037 0.044

1	(0.052) 0.046 0.047

Locus 16040 :

2 alleles

98.4% missing data

2	(0.567) 0.564 0.571

4	(0.433) 0.436 0.429

Locus 16041 :

2 alleles

99.2% missing data

2	(0.600) 0.605 0.596

3	(0.400) 0.395 0.404

Locus 16042 :

2 alleles

0.0% missing data

1	(0.987) 0.983 0.997

3	(0.013) 0.017 0.003

Locus 16043 :

2 alleles

99.2% missing data

3	(0.542) 0.546 0.538

2	(0.458) 0.454 0.462

Locus 16044 :

2 alleles

99.2% missing data

4	(0.527) 0.529 0.532

1	(0.473) 0.471 0.468

Locus 16045 :

2 alleles

99.2% missing data

3	(0.797) 0.792 0.804

2	(0.203) 0.208 0.196

Locus 16046 :

2 alleles

99.2% missing data

1	(0.372) 0.376 0.373

2	(0.628) 0.624 0.627

Locus 16047 :

2 alleles

99.2% missing data

4	(0.408) 0.405 0.412

2	(0.592) 0.595 0.588

Locus 16048 :

2 alleles

87.1% missing data

4	(0.934) 0.935 0.943

2	(0.066) 0.065 0.057

Locus 16049 :

2 alleles

99.2% missing data

2	(0.548) 0.544 0.546

3	(0.452) 0.456 0.454

Locus 16050 :

2 alleles

99.2% missing data

2	(0.563) 0.559 0.564

3	(0.437) 0.441 0.436

Locus 16051 :

2 alleles

99.2% missing data

4	(0.512) 0.510 0.511

1	(0.488) 0.490 0.489

Locus 16052 :

2 alleles

8.6% missing data

3	(0.984) 0.975 0.995

2	(0.016) 0.025 0.005

Locus 16053 :

2 alleles

99.2% missing data

3	(0.351) 0.343 0.355

2	(0.649) 0.657 0.645

Locus 16054 :

2 alleles

98.8% missing data

4	(0.420) 0.410 0.431

1	(0.580) 0.590 0.569

Locus 16055 :

3 alleles

98.8% missing data

1	(0.282) 0.289 0.274

2	(0.356) 0.345 0.367

4	(0.362) 0.366 0.359

Locus 16056 :

3 alleles

94.9% missing data

1	(0.688) 0.697 0.684

4	(0.173) 0.171 0.170

2	(0.139) 0.132 0.146

Locus 16057 :

2 alleles

98.0% missing data

2	(0.655) 0.664 0.650

3	(0.345) 0.336 0.350

Locus 16058 :

2 alleles

99.2% missing data

2	(0.330) 0.328 0.338

1	(0.670) 0.672 0.662

Locus 16059 :

2 alleles

98.4% missing data

4	(0.695) 0.696 0.695

2	(0.305) 0.304 0.305

Locus 16060 :

2 alleles

98.4% missing data

2	(0.654) 0.661 0.654

3	(0.346) 0.339 0.346

Locus 16061 :

2 alleles

93.4% missing data

4	(0.694) 0.698 0.689

1	(0.306) 0.302 0.311

Locus 16062 :

2 alleles

98.8% missing data

4	(0.748) 0.743 0.744

1	(0.252) 0.257 0.256

Locus 16063 :

2 alleles

99.2% missing data

3	(0.629) 0.617 0.636

2	(0.371) 0.383 0.364

Locus 16064 :

2 alleles

99.2% missing data

4	(0.191) 0.189 0.197

2	(0.809) 0.811 0.803

Locus 16065 :

2 alleles

8.2% missing data

3	(0.985) 0.977 0.996

1	(0.015) 0.023 0.004

Locus 16066 :

2 alleles

96.9% missing data

1	(0.784) 0.782 0.789

4	(0.216) 0.218 0.211

Locus 16067 :

2 alleles

99.2% missing data

2	(0.683) 0.687 0.679

4	(0.317) 0.313 0.321

Locus 16068 :

2 alleles

99.2% missing data

3	(0.441) 0.441 0.441

2	(0.559) 0.559 0.559

Locus 16069 :

2 alleles

98.8% missing data

4	(0.674) 0.679 0.670

1	(0.326) 0.321 0.330

Locus 16070 :

2 alleles

99.2% missing data

3	(0.280) 0.276 0.282

2	(0.720) 0.724 0.718

Locus 16071 :

2 alleles

99.2% missing data

1	(0.478) 0.473 0.488

3	(0.522) 0.527 0.512

Locus 16072 :

2 alleles

96.1% missing data

3	(0.900) 0.909 0.894

1	(0.100) 0.091 0.106

Locus 16073 :

2 alleles

99.2% missing data

1	(0.407) 0.411 0.410

3	(0.593) 0.589 0.590

Locus 16074 :

2 alleles

99.2% missing data

3	(0.476) 0.482 0.476

2	(0.524) 0.518 0.524

Locus 16075 :

2 alleles

99.2% missing data

3	(0.572) 0.572 0.573

2	(0.428) 0.428 0.427

Locus 16076 :

2 alleles

99.2% missing data

2	(0.437) 0.428 0.441

3	(0.563) 0.572 0.559

Locus 16077 :

2 alleles

93.0% missing data

2	(0.856) 0.856 0.871

1	(0.144) 0.144 0.129

Locus 16078 :

2 alleles

98.8% missing data

2	(0.800) 0.801 0.796

4	(0.200) 0.199 0.204

Locus 16079 :

2 alleles

99.2% missing data

3	(0.637) 0.640 0.640

1	(0.363) 0.360 0.360

Locus 16080 :

2 alleles

18.0% missing data

1	(0.985) 0.979 0.994

2	(0.015) 0.021 0.006

Locus 16081 :

2 alleles

99.2% missing data

2	(0.704) 0.706 0.705

3	(0.296) 0.294 0.295

Locus 16082 :

2 alleles

99.2% missing data

3	(0.513) 0.513 0.512

4	(0.487) 0.487 0.488

Locus 16083 :

2 alleles

99.2% missing data

1	(0.639) 0.640 0.633

2	(0.361) 0.360 0.367

Locus 16084 :

2 alleles

99.2% missing data

2	(0.576) 0.573 0.574

4	(0.424) 0.427 0.426

Locus 16085 :

2 alleles

99.2% missing data

2	(0.418) 0.415 0.421

3	(0.582) 0.585 0.579

Locus 16086 :

2 alleles

99.2% missing data

4	(0.586) 0.585 0.587

1	(0.414) 0.415 0.413

Locus 16087 :

2 alleles

99.2% missing data

2	(0.282) 0.275 0.285

3	(0.718) 0.725 0.715

Locus 16088 :

2 alleles

99.2% missing data

2	(0.607) 0.603 0.612

1	(0.393) 0.397 0.388

Locus 16089 :

2 alleles

98.0% missing data

1	(0.620) 0.625 0.627

4	(0.380) 0.375 0.373

Locus 16090 :

2 alleles

98.8% missing data

1	(0.602) 0.602 0.604

3	(0.398) 0.398 0.396

Locus 16091 :

2 alleles

98.8% missing data

1	(0.448) 0.455 0.452

4	(0.552) 0.545 0.548

Locus 16092 :

2 alleles

99.2% missing data

1	(0.652) 0.657 0.661

3	(0.348) 0.343 0.339

Locus 16093 :

2 alleles

99.2% missing data

1	(0.433) 0.434 0.429

4	(0.567) 0.566 0.571

Locus 16094 :

2 alleles

99.2% missing data

3	(0.506) 0.503 0.511

4	(0.494) 0.497 0.489

Locus 16095 :

2 alleles

99.2% missing data

1	(0.628) 0.628 0.622

3	(0.372) 0.372 0.378

Locus 16096 :

2 alleles

99.2% missing data

2	(0.191) 0.187 0.194

4	(0.809) 0.813 0.806

Locus 16097 :

2 alleles

99.2% missing data

3	(0.731) 0.723 0.739

4	(0.269) 0.277 0.261

Locus 16098 :

2 alleles

99.2% missing data

1	(0.576) 0.577 0.571

3	(0.424) 0.423 0.429

Locus 16099 :

2 alleles

99.2% missing data

2	(0.361) 0.350 0.366

3	(0.639) 0.650 0.634

Locus 16100 :

2 alleles

96.5% missing data

4	(0.805) 0.810 0.805

1	(0.195) 0.190 0.195

Locus 16101 :

2 alleles

99.2% missing data

2	(0.435) 0.439 0.426

1	(0.565) 0.561 0.574

Locus 16102 :

2 alleles

99.2% missing data

4	(0.545) 0.547 0.536

1	(0.455) 0.453 0.464

Locus 16103 :

2 alleles

99.2% missing data

4	(0.633) 0.631 0.639

1	(0.367) 0.369 0.361

Locus 16104 :

2 alleles

8.2% missing data

2	(0.985) 0.977 0.995

3	(0.015) 0.023 0.005

Locus 16105 :

2 alleles

98.8% missing data

4	(0.440) 0.439 0.434

3	(0.560) 0.561 0.566

Locus 16106 :

2 alleles

8.2% missing data

1	(0.982) 0.986 0.988

3	(0.018) 0.014 0.012

Locus 16107 :

2 alleles

0.0% missing data

2	(0.989) 0.983 0.997

4	(0.011) 0.017 0.003

Locus 16108 :

2 alleles

99.2% missing data

1	(0.790) 0.787 0.799

2	(0.210) 0.213 0.201

Locus 16109 :

2 alleles

99.2% missing data

2	(0.673) 0.676 0.676

4	(0.327) 0.324 0.324

Locus 16110 :

2 alleles

99.2% missing data

3	(0.623) 0.626 0.620

1	(0.377) 0.374 0.380

Locus 16111 :

2 alleles

99.2% missing data

3	(0.549) 0.550 0.549

4	(0.451) 0.450 0.451

Locus 16112 :

2 alleles

99.2% missing data

3	(0.305) 0.306 0.303

1	(0.695) 0.694 0.697

Locus 16113 :

2 alleles

99.2% missing data

2	(0.390) 0.385 0.392

3	(0.610) 0.615 0.608

Locus 16114 :

2 alleles

99.2% missing data

1	(0.359) 0.360 0.365

3	(0.641) 0.640 0.635

Locus 16115 :

2 alleles

99.2% missing data

1	(0.550) 0.543 0.552

2	(0.450) 0.457 0.448

Locus 16116 :

2 alleles

97.7% missing data

3	(0.264) 0.251 0.273

4	(0.736) 0.749 0.727

Locus 16117 :

2 alleles

99.2% missing data

3	(0.646) 0.643 0.651

4	(0.354) 0.357 0.349

Locus 16118 :

2 alleles

99.2% missing data

3	(0.510) 0.506 0.517

1	(0.490) 0.494 0.483

Locus 16119 :

2 alleles

98.8% missing data

4	(0.569) 0.578 0.572

1	(0.431) 0.422 0.428

Locus 16120 :

2 alleles

96.9% missing data

2	(0.291) 0.293 0.277

1	(0.709) 0.707 0.723

Locus 16121 :

2 alleles

97.7% missing data

3	(0.626) 0.632 0.617

2	(0.374) 0.368 0.383

Locus 16122 :

2 alleles

97.7% missing data

1	(0.201) 0.208 0.197

3	(0.799) 0.792 0.803

Locus 16123 :

2 alleles

98.4% missing data

1	(0.721) 0.729 0.717

3	(0.279) 0.271 0.283

Locus 16124 :

2 alleles

98.0% missing data

3	(0.779) 0.777 0.783

1	(0.221) 0.223 0.217

Locus 16125 :

2 alleles

98.8% missing data

2	(0.377) 0.373 0.378

3	(0.623) 0.627 0.622

Locus 16126 :

2 alleles

99.2% missing data

2	(0.382) 0.384 0.380

1	(0.618) 0.616 0.620

Locus 16127 :

2 alleles

98.4% missing data

3	(0.845) 0.852 0.840

1	(0.155) 0.148 0.160

Locus 16128 :

2 alleles

90.2% missing data

4	(0.827) 0.839 0.831

3	(0.173) 0.161 0.169

Locus 16129 :

2 alleles

99.2% missing data

3	(0.363) 0.366 0.358

1	(0.637) 0.634 0.642

Locus 16130 :

2 alleles

99.2% missing data

3	(0.471) 0.476 0.466

1	(0.529) 0.524 0.534

Locus 16131 :

2 alleles

99.2% missing data

1	(0.608) 0.607 0.608

3	(0.392) 0.393 0.392

Locus 16132 :

2 alleles

99.2% missing data

2	(0.355) 0.354 0.358

4	(0.645) 0.646 0.642

Locus 16133 :

2 alleles

99.2% missing data

1	(0.518) 0.516 0.519

4	(0.482) 0.484 0.481

Locus 16134 :

2 alleles

98.8% missing data

3	(0.726) 0.718 0.732

1	(0.274) 0.282 0.268

Locus 16135 :

2 alleles

99.2% missing data

1	(0.335) 0.330 0.344

4	(0.665) 0.670 0.656

Locus 16136 :

2 alleles

99.2% missing data

4	(0.413) 0.418 0.411

1	(0.587) 0.582 0.589

Locus 16137 :

2 alleles

96.9% missing data

3	(0.841) 0.829 0.851

4	(0.159) 0.171 0.149

Locus 16138 :

2 alleles

97.7% missing data

4	(0.253) 0.262 0.243

3	(0.747) 0.738 0.757

Locus 16139 :

2 alleles

99.2% missing data

4	(0.514) 0.514 0.517

1	(0.486) 0.486 0.483

Locus 16140 :

2 alleles

99.2% missing data

3	(0.391) 0.394 0.385

4	(0.609) 0.606 0.615

Locus 16141 :

2 alleles

99.2% missing data

2	(0.758) 0.756 0.764

4	(0.242) 0.244 0.236

Locus 16142 :

2 alleles

99.2% missing data

4	(0.358) 0.362 0.356

1	(0.642) 0.638 0.644

Locus 16143 :

2 alleles

99.2% missing data

4	(0.599) 0.595 0.600

1	(0.401) 0.405 0.400

Locus 16144 :

2 alleles

98.4% missing data

1	(0.755) 0.766 0.748

2	(0.245) 0.234 0.252

Locus 16145 :

2 alleles

95.7% missing data

4	(0.778) 0.774 0.789

1	(0.222) 0.226 0.211

Locus 16146 :

2 alleles

99.2% missing data

2	(0.752) 0.755 0.744

1	(0.248) 0.245 0.256

Locus 16147 :

2 alleles

90.6% missing data

2	(0.881) 0.903 0.876

1	(0.119) 0.097 0.124

Locus 16148 :

2 alleles

99.2% missing data

2	(0.552) 0.553 0.553

1	(0.448) 0.447 0.447

Locus 16149 :

2 alleles

99.2% missing data

4	(0.279) 0.280 0.288

3	(0.721) 0.720 0.712

Locus 16150 :

2 alleles

98.8% missing data

3	(0.658) 0.657 0.656

1	(0.342) 0.343 0.344

Locus 16151 :

2 alleles

99.2% missing data

2	(0.420) 0.417 0.422

1	(0.580) 0.583 0.578

Locus 16152 :

2 alleles

99.2% missing data

2	(0.478) 0.475 0.477

1	(0.522) 0.525 0.523

Locus 16153 :

3 alleles

98.8% missing data

2	(0.332) 0.329 0.330

4	(0.373) 0.371 0.381

1	(0.294) 0.300 0.289

Locus 16154 :

2 alleles

99.2% missing data

3	(0.561) 0.560 0.557

1	(0.439) 0.440 0.443

Locus 16155 :

2 alleles

91.0% missing data

2	(0.879) 0.902 0.867

1	(0.121) 0.098 0.133

Locus 16156 :

2 alleles

98.4% missing data

2	(0.715) 0.724 0.710

3	(0.285) 0.276 0.290

Locus 16157 :

2 alleles

99.2% missing data

4	(0.357) 0.365 0.351

1	(0.643) 0.635 0.649

Locus 16158 :

2 alleles

98.8% missing data

3	(0.612) 0.617 0.603

2	(0.388) 0.383 0.397

Locus 16159 :

2 alleles

99.2% missing data

1	(0.187) 0.192 0.180

4	(0.813) 0.808 0.820

Locus 16160 :

2 alleles

98.8% missing data

3	(0.629) 0.626 0.625

4	(0.371) 0.374 0.375

Locus 16161 :

2 alleles

8.2% missing data

1	(0.987) 0.979 0.997

3	(0.013) 0.021 0.003

Locus 16162 :

2 alleles

97.7% missing data

1	(0.202) 0.200 0.199

3	(0.798) 0.800 0.801

Locus 16163 :

2 alleles

99.2% missing data

1	(0.601) 0.599 0.602

3	(0.399) 0.401 0.398

Locus 16164 :

2 alleles

98.8% missing data

3	(0.720) 0.719 0.723

1	(0.280) 0.281 0.277

Locus 16165 :

2 alleles

99.2% missing data

1	(0.550) 0.550 0.554

4	(0.450) 0.450 0.446

Locus 16166 :

2 alleles

99.2% missing data

2	(0.584) 0.589 0.587

3	(0.416) 0.411 0.413

Locus 16167 :

2 alleles

96.5% missing data

1	(0.813) 0.831 0.806

3	(0.187) 0.169 0.194

Locus 16168 :

2 alleles

99.2% missing data

3	(0.260) 0.263 0.262

1	(0.740) 0.737 0.738

Locus 16169 :

2 alleles

98.8% missing data

1	(0.526) 0.524 0.522

3	(0.474) 0.476 0.478

Locus 16170 :

2 alleles

99.2% missing data

1	(0.588) 0.584 0.589

3	(0.412) 0.416 0.411

Locus 16171 :

2 alleles

99.2% missing data

2	(0.422) 0.415 0.424

4	(0.578) 0.585 0.576

Locus 16172 :

2 alleles

98.8% missing data

4	(0.570) 0.570 0.567

1	(0.430) 0.430 0.433

Locus 16173 :

2 alleles

99.2% missing data

1	(0.645) 0.640 0.653

2	(0.355) 0.360 0.347

Locus 16174 :

2 alleles

99.2% missing data

3	(0.537) 0.529 0.545

2	(0.463) 0.471 0.455

Locus 16175 :

2 alleles

96.5% missing data

2	(0.195) 0.188 0.199

4	(0.805) 0.812 0.801

Locus 16176 :

2 alleles

99.2% missing data

1	(0.490) 0.489 0.489

3	(0.510) 0.511 0.511

Locus 16177 :

2 alleles

99.2% missing data

3	(0.527) 0.519 0.531

4	(0.473) 0.481 0.469

Locus 16178 :

2 alleles

96.9% missing data

4	(0.898) 0.892 0.907

3	(0.102) 0.108 0.093

Locus 16179 :

2 alleles

99.2% missing data

1	(0.584) 0.588 0.580

3	(0.416) 0.412 0.420

Locus 16180 :

2 alleles

98.8% missing data

4	(0.618) 0.615 0.618

2	(0.382) 0.385 0.382

Locus 16181 :

2 alleles

98.8% missing data

3	(0.672) 0.673 0.675

1	(0.328) 0.327 0.325

Locus 16182 :

2 alleles

99.2% missing data

1	(0.656) 0.654 0.658

2	(0.344) 0.346 0.342

Locus 16183 :

2 alleles

96.9% missing data

3	(0.652) 0.640 0.672

4	(0.348) 0.360 0.328

Locus 16184 :

2 alleles

99.2% missing data

1	(0.513) 0.512 0.520

2	(0.487) 0.488 0.480

Locus 16185 :

2 alleles

99.2% missing data

2	(0.531) 0.537 0.521

1	(0.469) 0.463 0.479

Locus 16186 :

2 alleles

96.1% missing data

1	(0.182) 0.171 0.185

2	(0.818) 0.829 0.815

Locus 16187 :

2 alleles

99.2% missing data

1	(0.284) 0.284 0.290

2	(0.716) 0.716 0.710

Locus 16188 :

2 alleles

99.2% missing data

2	(0.441) 0.445 0.434

4	(0.559) 0.555 0.566

Locus 16189 :

2 alleles

99.2% missing data

4	(0.407) 0.407 0.409

1	(0.593) 0.593 0.591

Locus 16190 :

2 alleles

99.2% missing data

4	(0.277) 0.271 0.278

1	(0.723) 0.729 0.722

Locus 16191 :

2 alleles

99.2% missing data

4	(0.514) 0.507 0.520

3	(0.486) 0.493 0.480

Locus 16192 :

2 alleles

97.7% missing data

1	(0.191) 0.184 0.193

4	(0.809) 0.816 0.807

Locus 16193 :

2 alleles

99.2% missing data

1	(0.469) 0.465 0.474

2	(0.531) 0.535 0.526

Locus 16194 :

2 alleles

97.3% missing data

1	(0.296) 0.295 0.297

2	(0.704) 0.705 0.703

Locus 16195 :

2 alleles

99.2% missing data

2	(0.694) 0.692 0.691

3	(0.306) 0.308 0.309

Locus 16196 :

2 alleles

0.0% missing data

3	(0.986) 0.983 0.996

2	(0.014) 0.017 0.004

Locus 16197 :

2 alleles

99.2% missing data

4	(0.402) 0.401 0.409

3	(0.598) 0.599 0.591

Locus 16198 :

3 alleles

98.4% missing data

4	(0.190) 0.189 0.192

1	(0.311) 0.315 0.309

3	(0.499) 0.496 0.499

Locus 16199 :

2 alleles

99.2% missing data

1	(0.138) 0.146 0.138

2	(0.862) 0.854 0.862

Locus 16200 :

2 alleles

98.8% missing data

1	(0.452) 0.452 0.453

3	(0.548) 0.548 0.547

Locus 16201 :

2 alleles

99.2% missing data

1	(0.238) 0.238 0.244

2	(0.762) 0.762 0.756

Locus 16202 :

2 alleles

96.5% missing data

3	(0.832) 0.834 0.833

4	(0.168) 0.166 0.167

Locus 16203 :

2 alleles

99.2% missing data

3	(0.453) 0.448 0.462

1	(0.547) 0.552 0.538

Locus 16204 :

2 alleles

98.8% missing data

4	(0.263) 0.257 0.273

1	(0.737) 0.743 0.727

Locus 16205 :

2 alleles

99.2% missing data

4	(0.692) 0.686 0.693

3	(0.308) 0.314 0.307

Locus 16206 :

2 alleles

99.2% missing data

4	(0.573) 0.576 0.574

3	(0.427) 0.424 0.426

Locus 16207 :

2 alleles

99.2% missing data

3	(0.517) 0.513 0.522

4	(0.483) 0.487 0.478

Locus 16208 :

2 alleles

99.2% missing data

3	(0.331) 0.324 0.336

4	(0.669) 0.676 0.664

Locus 16209 :

2 alleles

98.0% missing data

1	(0.255) 0.251 0.249

4	(0.745) 0.749 0.751

Locus 16210 :

2 alleles

98.4% missing data

1	(0.691) 0.696 0.691

4	(0.309) 0.304 0.309

Locus 16211 :

2 alleles

99.2% missing data

3	(0.167) 0.170 0.166

1	(0.833) 0.830 0.834

Locus 16212 :

2 alleles

99.2% missing data

2	(0.561) 0.557 0.561

4	(0.439) 0.443 0.439

Locus 16213 :

2 alleles

99.2% missing data

3	(0.449) 0.447 0.446

4	(0.551) 0.553 0.554

Locus 16214 :

2 alleles

98.8% missing data

2	(0.239) 0.242 0.234

1	(0.761) 0.758 0.766

Locus 16215 :

2 alleles

99.2% missing data

3	(0.230) 0.226 0.236

1	(0.770) 0.774 0.764

Locus 16216 :

2 alleles

96.9% missing data

1	(0.187) 0.182 0.184

3	(0.813) 0.818 0.816

Locus 16217 :

2 alleles

99.2% missing data

3	(0.366) 0.374 0.362

1	(0.634) 0.626 0.638

Locus 16218 :

3 alleles

98.4% missing data

2	(0.240) 0.239 0.245

4	(0.428) 0.428 0.429

1	(0.332) 0.334 0.325

Locus 16219 :

2 alleles

99.2% missing data

4	(0.252) 0.252 0.257

3	(0.748) 0.748 0.743

Locus 16220 :

2 alleles

99.2% missing data

4	(0.574) 0.571 0.573

1	(0.426) 0.429 0.427

Locus 16221 :

2 alleles

97.7% missing data

1	(0.668) 0.659 0.688

4	(0.332) 0.341 0.312

Locus 16222 :

2 alleles

99.2% missing data

4	(0.599) 0.591 0.606

1	(0.401) 0.409 0.394

Locus 16223 :

2 alleles

99.2% missing data

3	(0.530) 0.520 0.536

1	(0.470) 0.480 0.464

Locus 16224 :

2 alleles

99.2% missing data

1	(0.343) 0.338 0.341

3	(0.657) 0.662 0.659

Locus 16225 :

2 alleles

99.2% missing data

1	(0.701) 0.693 0.705

2	(0.299) 0.307 0.295

Locus 16226 :

2 alleles

99.2% missing data

4	(0.198) 0.209 0.193

3	(0.802) 0.791 0.807

Locus 16227 :

2 alleles

99.2% missing data

3	(0.258) 0.264 0.253

1	(0.742) 0.736 0.747

Locus 16228 :

2 alleles

95.7% missing data

3	(0.736) 0.748 0.727

1	(0.264) 0.252 0.273

Locus 16229 :

2 alleles

99.2% missing data

3	(0.733) 0.731 0.738

4	(0.267) 0.269 0.262

Locus 16230 :

2 alleles

96.9% missing data

2	(0.889) 0.879 0.897

1	(0.111) 0.121 0.103

Locus 16231 :

2 alleles

99.2% missing data

1	(0.445) 0.442 0.453

3	(0.555) 0.558 0.547

Locus 16232 :

2 alleles

97.3% missing data

4	(0.240) 0.242 0.240

1	(0.760) 0.758 0.760

Locus 16233 :

2 alleles

98.8% missing data

2	(0.513) 0.503 0.522

3	(0.487) 0.497 0.478

Locus 16234 :

2 alleles

99.2% missing data

2	(0.360) 0.362 0.368

1	(0.640) 0.638 0.632

Locus 16235 :

2 alleles

97.7% missing data

3	(0.254) 0.254 0.252

1	(0.746) 0.746 0.748

Locus 16236 :

2 alleles

99.2% missing data

2	(0.374) 0.376 0.379

3	(0.626) 0.624 0.621

Locus 16237 :

2 alleles

99.2% missing data

4	(0.552) 0.561 0.547

3	(0.448) 0.439 0.453

Locus 16238 :

2 alleles

97.7% missing data

4	(0.736) 0.731 0.747

3	(0.264) 0.269 0.253

Locus 16239 :

2 alleles

98.8% missing data

3	(0.367) 0.363 0.372

4	(0.633) 0.637 0.628

Locus 16240 :

2 alleles

97.3% missing data

1	(0.283) 0.270 0.283

3	(0.717) 0.730 0.717

Locus 16241 :

2 alleles

98.8% missing data

1	(0.752) 0.751 0.753

3	(0.248) 0.249 0.247

Locus 16242 :

2 alleles

99.2% missing data

4	(0.555) 0.558 0.554

2	(0.445) 0.442 0.446

Locus 16243 :

2 alleles

97.7% missing data

3	(0.823) 0.826 0.819

1	(0.177) 0.174 0.181

Locus 16244 :

2 alleles

99.2% missing data

1	(0.624) 0.624 0.631

3	(0.376) 0.376 0.369

Locus 16245 :

3 alleles

97.3% missing data

2	(0.723) 0.738 0.713

3	(0.157) 0.148 0.162

4	(0.120) 0.114 0.125

Locus 16246 :

2 alleles

95.3% missing data

4	(0.895) 0.907 0.887

3	(0.105) 0.093 0.113

Locus 16247 :

2 alleles

99.2% missing data

2	(0.458) 0.461 0.450

3	(0.542) 0.539 0.550

Locus 16248 :

2 alleles

99.2% missing data

3	(0.553) 0.555 0.555

2	(0.447) 0.445 0.445

Locus 16249 :

3 alleles

98.8% missing data

3	(0.175) 0.177 0.177

2	(0.416) 0.419 0.417

4	(0.409) 0.405 0.406

Locus 16250 :

2 alleles

99.2% missing data

1	(0.375) 0.372 0.381

2	(0.625) 0.628 0.619

Locus 16251 :

2 alleles

99.2% missing data

1	(0.600) 0.609 0.590

2	(0.400) 0.391 0.410

Locus 16252 :

2 alleles

97.7% missing data

1	(0.882) 0.875 0.892

4	(0.118) 0.125 0.108

Locus 16253 :

2 alleles

99.2% missing data

2	(0.432) 0.432 0.440

3	(0.568) 0.568 0.560

Locus 16254 :

2 alleles

99.2% missing data

4	(0.344) 0.348 0.343

1	(0.656) 0.652 0.657

Locus 16255 :

2 alleles

99.2% missing data

2	(0.255) 0.261 0.252

4	(0.745) 0.739 0.748

Locus 16256 :

2 alleles

97.7% missing data

4	(0.204) 0.210 0.193

1	(0.796) 0.790 0.807

Locus 16257 :

2 alleles

98.8% missing data

2	(0.609) 0.609 0.610

4	(0.391) 0.391 0.390

Locus 16258 :

2 alleles

0.0% missing data

2	(0.984) 0.987 0.992

3	(0.016) 0.013 0.008

Locus 16259 :

2 alleles

99.2% missing data

4	(0.505) 0.505 0.501

1	(0.495) 0.495 0.499

Locus 16260 :

2 alleles

99.2% missing data

1	(0.543) 0.542 0.543

2	(0.457) 0.458 0.457

Locus 16261 :

2 alleles

98.8% missing data

1	(0.384) 0.382 0.384

4	(0.616) 0.618 0.616

Locus 16262 :

2 alleles

97.3% missing data

1	(0.328) 0.325 0.318

2	(0.672) 0.675 0.682

Locus 16263 :

2 alleles

99.2% missing data

1	(0.282) 0.281 0.285

2	(0.718) 0.719 0.715

Locus 16264 :

2 alleles

88.7% missing data

3	(0.789) 0.831 0.739

4	(0.211) 0.169 0.261

Locus 16265 :

2 alleles

97.7% missing data

4	(0.209) 0.205 0.210

3	(0.791) 0.795 0.790

Locus 16266 :

2 alleles

99.2% missing data

2	(0.386) 0.375 0.393

1	(0.614) 0.625 0.607

Locus 16267 :

4 alleles

96.9% missing data

4	(0.474) 0.473 0.481

2	(0.181) 0.175 0.183

1	(0.228) 0.241 0.214

3	(0.116) 0.111 0.121

Locus 16268 :

2 alleles

99.2% missing data

3	(0.585) 0.591 0.583

1	(0.415) 0.409 0.417

Locus 16269 :

2 alleles

97.3% missing data

4	(0.811) 0.805 0.824

1	(0.189) 0.195 0.176

Locus 16270 :

2 alleles

99.2% missing data

1	(0.551) 0.548 0.549

4	(0.449) 0.452 0.451

Locus 16271 :

2 alleles

99.2% missing data

4	(0.537) 0.541 0.533

1	(0.463) 0.459 0.467

Locus 16272 :

2 alleles

99.2% missing data

3	(0.593) 0.586 0.600

4	(0.407) 0.414 0.400

Locus 16273 :

2 alleles

97.7% missing data

3	(0.312) 0.318 0.299

4	(0.688) 0.682 0.701

Locus 16274 :

2 alleles

99.2% missing data

4	(0.622) 0.629 0.614

1	(0.378) 0.371 0.386

Locus 16275 :

2 alleles

98.4% missing data

1	(0.364) 0.357 0.376

4	(0.636) 0.643 0.624

Locus 16276 :

2 alleles

91.0% missing data

1	(0.928) 0.941 0.918

3	(0.072) 0.059 0.082

Locus 16277 :

2 alleles

99.2% missing data

3	(0.340) 0.345 0.332

1	(0.660) 0.655 0.668

Locus 16278 :

2 alleles

98.4% missing data

3	(0.680) 0.684 0.681

1	(0.320) 0.316 0.319

Locus 16279 :

2 alleles

99.2% missing data

2	(0.467) 0.459 0.473

1	(0.533) 0.541 0.527

Locus 16280 :

2 alleles

99.2% missing data

3	(0.370) 0.376 0.369

4	(0.630) 0.624 0.631

Locus 16281 :

2 alleles

99.2% missing data

1	(0.264) 0.258 0.260

3	(0.736) 0.742 0.740

Locus 16282 :

2 alleles

94.5% missing data

1	(0.324) 0.351 0.294

4	(0.676) 0.649 0.706

Locus 16283 :

2 alleles

99.2% missing data

2	(0.627) 0.623 0.627

3	(0.373) 0.377 0.373

Locus 16284 :

3 alleles

98.4% missing data

2	(0.300) 0.291 0.309

1	(0.189) 0.186 0.191

4	(0.512) 0.523 0.500

Locus 16285 :

3 alleles

98.8% missing data

4	(0.463) 0.458 0.463

3	(0.244) 0.242 0.241

1	(0.293) 0.300 0.296

Locus 16286 :

2 alleles

99.2% missing data

2	(0.529) 0.519 0.535

4	(0.471) 0.481 0.465

Locus 16287 :

2 alleles

99.2% missing data

4	(0.444) 0.442 0.452

2	(0.556) 0.558 0.548

Locus 16288 :

2 alleles

96.5% missing data

4	(0.876) 0.888 0.868

1	(0.124) 0.112 0.132

Locus 16289 :

2 alleles

99.2% missing data

4	(0.408) 0.409 0.405

1	(0.592) 0.591 0.595

Locus 16290 :

3 alleles

98.8% missing data

4	(0.547) 0.543 0.551

3	(0.171) 0.176 0.168

2	(0.282) 0.282 0.280

Locus 16291 :

2 alleles

99.2% missing data

1	(0.648) 0.645 0.654

4	(0.352) 0.355 0.346

Locus 16292 :

2 alleles

89.1% missing data

1	(0.945) 0.951 0.944

4	(0.055) 0.049 0.056

Locus 16293 :

2 alleles

99.2% missing data

3	(0.580) 0.583 0.579

4	(0.420) 0.417 0.421

Locus 16294 :

2 alleles

99.2% missing data

1	(0.399) 0.398 0.402

2	(0.601) 0.602 0.598

Locus 16295 :

2 alleles

99.2% missing data

4	(0.525) 0.520 0.531

2	(0.475) 0.480 0.469

Locus 16296 :

2 alleles

99.2% missing data

3	(0.536) 0.536 0.535

2	(0.464) 0.464 0.465

Locus 16297 :

2 alleles

98.4% missing data

3	(0.602) 0.597 0.610

4	(0.398) 0.403 0.390

Locus 16298 :

2 alleles

99.2% missing data

1	(0.392) 0.386 0.404

2	(0.608) 0.614 0.596

Locus 16299 :

3 alleles

98.4% missing data

3	(0.278) 0.268 0.288

1	(0.338) 0.331 0.338

2	(0.383) 0.401 0.374

Locus 16300 :

2 alleles

99.2% missing data

1	(0.240) 0.238 0.246

4	(0.760) 0.762 0.754

Locus 16301 :

3 alleles

98.8% missing data

1	(0.338) 0.341 0.335

3	(0.510) 0.514 0.509

2	(0.153) 0.145 0.156

Locus 16302 :

3 alleles

98.8% missing data

2	(0.259) 0.258 0.265

1	(0.216) 0.209 0.220

4	(0.524) 0.534 0.515

Locus 16303 :

2 alleles

99.2% missing data

4	(0.647) 0.655 0.641

1	(0.353) 0.345 0.359

Locus 16304 :

2 alleles

91.0% missing data

1	(0.919) 0.936 0.910

2	(0.081) 0.064 0.090

Locus 16305 :

2 alleles

0.0% missing data

1	(0.901) 0.743 0.970

2	(0.099) 0.257 0.030

Locus 16306 :

2 alleles

99.2% missing data

1	(0.602) 0.603 0.598

3	(0.398) 0.397 0.402

Locus 16307 :

3 alleles

97.7% missing data

1	(0.610) 0.620 0.611

3	(0.192) 0.180 0.194

4	(0.198) 0.200 0.196

Locus 16308 :

2 alleles

92.6% missing data

1	(0.906) 0.904 0.911

4	(0.094) 0.096 0.089

Locus 16309 :

2 alleles

99.2% missing data

3	(0.422) 0.408 0.433

4	(0.578) 0.592 0.567

Locus 16310 :

2 alleles

8.2% missing data

1	(0.988) 0.981 0.997

3	(0.012) 0.019 0.003

Locus 16311 :

2 alleles

99.2% missing data

4	(0.557) 0.556 0.560

2	(0.443) 0.444 0.440

Locus 16312 :

2 alleles

99.2% missing data

2	(0.342) 0.344 0.345

1	(0.658) 0.656 0.655

Locus 16313 :

3 alleles

98.8% missing data

3	(0.414) 0.422 0.409

2	(0.327) 0.323 0.326

1	(0.259) 0.255 0.264

Locus 16314 :

2 alleles

98.8% missing data

4	(0.647) 0.651 0.651

3	(0.353) 0.349 0.349

Locus 16315 :

2 alleles

99.2% missing data

1	(0.528) 0.526 0.524

3	(0.472) 0.474 0.476

Locus 16316 :

2 alleles

99.2% missing data

4	(0.228) 0.230 0.229

3	(0.772) 0.770 0.771

Locus 16317 :

2 alleles

99.2% missing data

1	(0.305) 0.308 0.297

4	(0.695) 0.692 0.703

Locus 16318 :

2 alleles

97.3% missing data

1	(0.347) 0.353 0.343

2	(0.653) 0.647 0.657

Locus 16319 :

2 alleles

96.9% missing data

4	(0.851) 0.853 0.852

1	(0.149) 0.147 0.148

Locus 16320 :

2 alleles

99.2% missing data

1	(0.446) 0.451 0.442

3	(0.554) 0.549 0.558

Locus 16321 :

2 alleles

99.2% missing data

2	(0.308) 0.313 0.300

3	(0.692) 0.687 0.700

Locus 16322 :

2 alleles

98.8% missing data

1	(0.442) 0.438 0.454

3	(0.558) 0.562 0.546

Locus 16323 :

2 alleles

99.2% missing data

3	(0.492) 0.488 0.497

4	(0.508) 0.512 0.503

Locus 16324 :

2 alleles

97.3% missing data

3	(0.697) 0.698 0.713

1	(0.303) 0.302 0.287

Locus 16325 :

2 alleles

98.8% missing data

4	(0.722) 0.725 0.715

2	(0.278) 0.275 0.285

Locus 16326 :

2 alleles

99.2% missing data

3	(0.301) 0.295 0.310

1	(0.699) 0.705 0.690

Locus 16327 :

2 alleles

99.2% missing data

4	(0.252) 0.254 0.251

3	(0.748) 0.746 0.749

Locus 16328 :

2 alleles

99.2% missing data

1	(0.531) 0.527 0.540

2	(0.469) 0.473 0.460

Locus 16329 :

2 alleles

99.2% missing data

3	(0.538) 0.531 0.540

1	(0.462) 0.469 0.460

Locus 16330 :

2 alleles

96.1% missing data

4	(0.131) 0.127 0.132

3	(0.869) 0.873 0.868

Locus 16331 :

2 alleles

99.2% missing data

3	(0.450) 0.456 0.447

2	(0.550) 0.544 0.553

Locus 16332 :

2 alleles

98.4% missing data

3	(0.646) 0.650 0.640

1	(0.354) 0.350 0.360

Locus 16333 :

2 alleles

99.2% missing data

4	(0.479) 0.489 0.470

3	(0.521) 0.511 0.530

Locus 16334 :

2 alleles

99.2% missing data

2	(0.440) 0.438 0.438

4	(0.560) 0.562 0.562

Locus 16335 :

2 alleles

99.2% missing data

3	(0.560) 0.553 0.564

2	(0.440) 0.447 0.436

Locus 16336 :

2 alleles

99.2% missing data

3	(0.692) 0.697 0.687

1	(0.308) 0.303 0.313

Locus 16337 :

3 alleles

98.8% missing data

4	(0.215) 0.220 0.212

3	(0.483) 0.483 0.480

2	(0.303) 0.296 0.308

Locus 16338 :

2 alleles

99.2% missing data

4	(0.495) 0.496 0.493

3	(0.505) 0.504 0.507

Locus 16339 :

2 alleles

99.2% missing data

3	(0.703) 0.701 0.703

1	(0.297) 0.299 0.297

Locus 16340 :

2 alleles

99.2% missing data

1	(0.335) 0.333 0.338

3	(0.665) 0.667 0.662

Locus 16341 :

2 alleles

99.2% missing data

2	(0.804) 0.808 0.800

4	(0.196) 0.192 0.200

Locus 16342 :

2 alleles

0.0% missing data

2	(0.980) 0.985 0.984

3	(0.020) 0.015 0.016

Locus 16343 :

2 alleles

98.8% missing data

1	(0.406) 0.405 0.410

3	(0.594) 0.595 0.590

Locus 16344 :

2 alleles

91.0% missing data

3	(0.914) 0.928 0.907

1	(0.086) 0.072 0.093

Locus 16345 :

2 alleles

99.2% missing data

2	(0.509) 0.512 0.511

3	(0.491) 0.488 0.489

Locus 16346 :

2 alleles

98.8% missing data

1	(0.779) 0.773 0.778

3	(0.221) 0.227 0.222

Locus 16347 :

2 alleles

98.0% missing data

2	(0.518) 0.526 0.517

4	(0.482) 0.474 0.483

Locus 16348 :

2 alleles

93.8% missing data

1	(0.824) 0.819 0.845

3	(0.176) 0.181 0.155

Locus 16349 :

2 alleles

99.2% missing data

1	(0.355) 0.356 0.357

3	(0.645) 0.644 0.643

Locus 16350 :

2 alleles

98.0% missing data

1	(0.471) 0.467 0.482

3	(0.529) 0.533 0.518

Locus 16351 :

3 alleles

95.3% missing data

2	(0.307) 0.298 0.297

1	(0.517) 0.539 0.521

4	(0.177) 0.164 0.182

Locus 16352 :

2 alleles

99.2% missing data

2	(0.841) 0.832 0.843

3	(0.159) 0.168 0.157

Locus 16353 :

2 alleles

99.2% missing data

4	(0.258) 0.264 0.258

3	(0.742) 0.736 0.742

Locus 16354 :

2 alleles

99.2% missing data

4	(0.467) 0.460 0.471

3	(0.533) 0.540 0.529

Locus 16355 :

2 alleles

99.2% missing data

1	(0.585) 0.581 0.587

2	(0.415) 0.419 0.413

Locus 16356 :

2 alleles

99.2% missing data

3	(0.368) 0.367 0.378

1	(0.632) 0.633 0.622

Locus 16357 :

2 alleles

99.2% missing data

1	(0.722) 0.723 0.721

3	(0.278) 0.277 0.279

Locus 16358 :

2 alleles

93.0% missing data

1	(0.901) 0.895 0.915

4	(0.099) 0.105 0.085

Locus 16359 :

2 alleles

98.8% missing data

1	(0.411) 0.403 0.425

3	(0.589) 0.597 0.575

Locus 16360 :

2 alleles

98.8% missing data

2	(0.393) 0.390 0.393

4	(0.607) 0.610 0.607

Locus 16361 :

2 alleles

99.2% missing data

1	(0.483) 0.479 0.481

2	(0.517) 0.521 0.519

Locus 16362 :

2 alleles

94.5% missing data

1	(0.891) 0.887 0.903

4	(0.109) 0.113 0.097

Locus 16363 :

2 alleles

99.2% missing data

3	(0.372) 0.370 0.370

1	(0.628) 0.630 0.630

Locus 16364 :

2 alleles

98.8% missing data

2	(0.350) 0.352 0.354

1	(0.650) 0.648 0.646

Locus 16365 :

2 alleles

99.2% missing data

4	(0.695) 0.698 0.686

1	(0.305) 0.302 0.314

Locus 16366 :

2 alleles

99.2% missing data

4	(0.762) 0.757 0.762

2	(0.238) 0.243 0.238

Locus 16367 :

2 alleles

99.2% missing data

3	(0.478) 0.485 0.469

4	(0.522) 0.515 0.531

Locus 16368 :

2 alleles

96.5% missing data

1	(0.672) 0.685 0.668

3	(0.328) 0.315 0.332

Locus 16369 :

2 alleles

99.2% missing data

2	(0.378) 0.385 0.371

3	(0.622) 0.615 0.629

Locus 16370 :

2 alleles

99.2% missing data

2	(0.674) 0.673 0.673

3	(0.326) 0.327 0.327

Locus 16371 :

2 alleles

98.8% missing data

1	(0.789) 0.791 0.789

3	(0.211) 0.209 0.211

Locus 16372 :

2 alleles

99.2% missing data

4	(0.251) 0.254 0.248

2	(0.749) 0.746 0.752

Locus 16373 :

2 alleles

99.2% missing data

4	(0.550) 0.544 0.548

1	(0.450) 0.456 0.452

Locus 16374 :

2 alleles

99.2% missing data

2	(0.436) 0.427 0.438

3	(0.564) 0.573 0.562

Locus 16375 :

2 alleles

98.0% missing data

4	(0.455) 0.443 0.460

1	(0.545) 0.557 0.540

Locus 16376 :

2 alleles

98.8% missing data

4	(0.797) 0.797 0.803

3	(0.203) 0.203 0.197

Locus 16377 :

2 alleles

98.4% missing data

3	(0.584) 0.590 0.587

4	(0.416) 0.410 0.413

Locus 16378 :

2 alleles

99.2% missing data

1	(0.402) 0.398 0.402

3	(0.598) 0.602 0.598

Locus 16379 :

2 alleles

99.2% missing data

1	(0.511) 0.521 0.505

3	(0.489) 0.479 0.495

Locus 16380 :

3 alleles

94.9% missing data

2	(0.136) 0.132 0.132

3	(0.730) 0.727 0.749

4	(0.134) 0.140 0.119

Locus 16381 :

2 alleles

99.2% missing data

2	(0.355) 0.348 0.365

4	(0.645) 0.652 0.635

Locus 16382 :

2 alleles

99.2% missing data

2	(0.387) 0.380 0.387

3	(0.613) 0.620 0.613

Locus 16383 :

2 alleles

97.7% missing data

3	(0.748) 0.749 0.751

4	(0.252) 0.251 0.249

Locus 16384 :

2 alleles

99.2% missing data

1	(0.454) 0.447 0.464

4	(0.546) 0.553 0.536

Locus 16385 :

2 alleles

98.4% missing data

1	(0.615) 0.622 0.617

2	(0.385) 0.378 0.383

Locus 16386 :

2 alleles

98.8% missing data

1	(0.703) 0.696 0.712

4	(0.297) 0.304 0.288

Locus 16387 :

2 alleles

98.4% missing data

1	(0.739) 0.743 0.732

2	(0.261) 0.257 0.268

Locus 16388 :

2 alleles

99.2% missing data

1	(0.564) 0.559 0.569

2	(0.436) 0.441 0.431

Locus 16389 :

2 alleles

99.2% missing data

1	(0.372) 0.376 0.376

4	(0.628) 0.624 0.624

Locus 16390 :

2 alleles

99.2% missing data

1	(0.189) 0.188 0.191

3	(0.811) 0.812 0.809

Locus 16391 :

2 alleles

99.2% missing data

1	(0.641) 0.636 0.644

3	(0.359) 0.364 0.356

Locus 16392 :

2 alleles

99.2% missing data

1	(0.568) 0.564 0.570

2	(0.432) 0.436 0.430

Locus 16393 :

2 alleles

98.4% missing data

1	(0.551) 0.558 0.544

4	(0.449) 0.442 0.456

Locus 16394 :

2 alleles

95.7% missing data

3	(0.904) 0.912 0.902

4	(0.096) 0.088 0.098

Locus 16395 :

2 alleles

0.4% missing data

2	(0.984) 0.988 0.992

3	(0.016) 0.012 0.008

Locus 16396 :

2 alleles

99.2% missing data

1	(0.724) 0.716 0.725

4	(0.276) 0.284 0.275

Locus 16397 :

2 alleles

96.9% missing data

1	(0.789) 0.784 0.797

4	(0.211) 0.216 0.203

Locus 16398 :

2 alleles

0.0% missing data

4	(0.984) 0.980 0.995

1	(0.016) 0.020 0.005

Locus 16399 :

2 alleles

96.1% missing data

1	(0.853) 0.858 0.857

2	(0.147) 0.142 0.143

Locus 16400 :

2 alleles

98.0% missing data

1	(0.687) 0.688 0.688

2	(0.313) 0.312 0.312

Locus 16401 :

3 alleles

98.8% missing data

3	(0.431) 0.431 0.428

2	(0.221) 0.211 0.228

1	(0.348) 0.357 0.344

Locus 16402 :

2 alleles

99.2% missing data

2	(0.610) 0.605 0.609

4	(0.390) 0.395 0.391

Locus 16403 :

2 alleles

99.2% missing data

3	(0.678) 0.674 0.678

2	(0.322) 0.326 0.322

Locus 16404 :

2 alleles

99.2% missing data

2	(0.473) 0.479 0.478

1	(0.527) 0.521 0.522

Locus 16405 :

2 alleles

98.8% missing data

1	(0.475) 0.472 0.480

4	(0.525) 0.528 0.520

Locus 16406 :

2 alleles

99.2% missing data

1	(0.293) 0.291 0.296

4	(0.707) 0.709 0.704

Locus 16407 :

3 alleles

98.8% missing data

4	(0.253) 0.255 0.256

1	(0.625) 0.617 0.624

2	(0.122) 0.128 0.120

Locus 16408 :

2 alleles

99.2% missing data

4	(0.534) 0.523 0.540

3	(0.466) 0.477 0.460

Locus 16409 :

2 alleles

91.0% missing data

3	(0.918) 0.933 0.910

4	(0.082) 0.067 0.090

Locus 16410 :

3 alleles

98.8% missing data

4	(0.461) 0.468 0.449

1	(0.160) 0.157 0.167

2	(0.379) 0.374 0.384

Locus 16411 :

2 alleles

99.2% missing data

1	(0.396) 0.394 0.404

3	(0.604) 0.606 0.596

Locus 16412 :

2 alleles

99.2% missing data

3	(0.505) 0.503 0.504

1	(0.495) 0.497 0.496

Locus 16413 :

2 alleles

99.2% missing data

4	(0.455) 0.450 0.457

1	(0.545) 0.550 0.543

Locus 16414 :

2 alleles

99.2% missing data

4	(0.603) 0.595 0.610

3	(0.397) 0.405 0.390

Locus 16415 :

2 alleles

99.2% missing data

3	(0.356) 0.361 0.365

2	(0.644) 0.639 0.635

Locus 16416 :

2 alleles

99.2% missing data

1	(0.672) 0.680 0.670

2	(0.328) 0.320 0.330

Locus 16417 :

2 alleles

99.2% missing data

3	(0.470) 0.462 0.476

4	(0.530) 0.538 0.524

Locus 16418 :

2 alleles

99.2% missing data

2	(0.505) 0.505 0.503

3	(0.495) 0.495 0.497

Locus 16419 :

2 alleles

98.8% missing data

1	(0.558) 0.563 0.561

3	(0.442) 0.437 0.439

Locus 16420 :

2 alleles

99.2% missing data

2	(0.370) 0.373 0.372

3	(0.630) 0.627 0.628

Locus 16421 :

2 alleles

95.7% missing data

2	(0.077) 0.072 0.077

1	(0.923) 0.928 0.923

Locus 16422 :

2 alleles

99.2% missing data

1	(0.553) 0.558 0.554

3	(0.447) 0.442 0.446

Locus 16423 :

2 alleles

99.2% missing data

3	(0.313) 0.311 0.320

2	(0.687) 0.689 0.680

Locus 16424 :

2 alleles

98.8% missing data

3	(0.477) 0.473 0.473

1	(0.523) 0.527 0.527

Locus 16425 :

2 alleles

99.2% missing data

3	(0.555) 0.552 0.561

1	(0.445) 0.448 0.439

Locus 16426 :

2 alleles

98.0% missing data

3	(0.817) 0.816 0.812

1	(0.183) 0.184 0.188

Locus 16427 :

2 alleles

99.2% missing data

2	(0.317) 0.311 0.319

3	(0.683) 0.689 0.681

Locus 16428 :

2 alleles

99.2% missing data

4	(0.633) 0.639 0.631

1	(0.367) 0.361 0.369

Locus 16429 :

2 alleles

99.2% missing data

1	(0.413) 0.409 0.413

2	(0.587) 0.591 0.587

Locus 16430 :

2 alleles

99.2% missing data

1	(0.189) 0.188 0.190

3	(0.811) 0.812 0.810

Locus 16431 :

2 alleles

98.8% missing data

3	(0.596) 0.601 0.592

1	(0.404) 0.399 0.408

Locus 16432 :

2 alleles

99.2% missing data

3	(0.378) 0.372 0.385

4	(0.622) 0.628 0.615

Locus 16433 :

2 alleles

99.2% missing data

4	(0.367) 0.364 0.373

2	(0.633) 0.636 0.627

Locus 16434 :

2 alleles

99.2% missing data

3	(0.267) 0.270 0.263

2	(0.733) 0.730 0.737

Locus 16435 :

2 alleles

99.2% missing data

1	(0.503) 0.500 0.506

4	(0.497) 0.500 0.494

Locus 16436 :

2 alleles

99.2% missing data

3	(0.519) 0.513 0.524

1	(0.481) 0.487 0.476

Locus 16437 :

2 alleles

98.4% missing data

2	(0.589) 0.592 0.589

4	(0.411) 0.408 0.411

Locus 16438 :

2 alleles

99.2% missing data

4	(0.339) 0.339 0.348

3	(0.661) 0.661 0.652

Locus 16439 :

2 alleles

99.2% missing data

3	(0.657) 0.656 0.663

4	(0.343) 0.344 0.337

Locus 16440 :

2 alleles

99.2% missing data

2	(0.564) 0.567 0.557

4	(0.436) 0.433 0.443

Locus 16441 :

2 alleles

99.2% missing data

4	(0.418) 0.415 0.424

3	(0.582) 0.585 0.576

Locus 16442 :

2 alleles

99.2% missing data

3	(0.382) 0.381 0.385

4	(0.618) 0.619 0.615

Locus 16443 :

2 alleles

91.0% missing data

1	(0.915) 0.930 0.907

2	(0.085) 0.070 0.093

Locus 16444 :

2 alleles

99.2% missing data

1	(0.770) 0.766 0.772

3	(0.230) 0.234 0.228

Locus 16445 :

2 alleles

99.2% missing data

2	(0.345) 0.341 0.347

3	(0.655) 0.659 0.653

Locus 16446 :

2 alleles

99.2% missing data

1	(0.595) 0.587 0.600

4	(0.405) 0.413 0.400

Locus 16447 :

3 alleles

98.4% missing data

1	(0.314) 0.312 0.320

4	(0.520) 0.517 0.520

2	(0.166) 0.171 0.160

Locus 16448 :

2 alleles

98.8% missing data

3	(0.751) 0.744 0.758

2	(0.249) 0.256 0.242

Locus 16449 :

2 alleles

90.6% missing data

2	(0.829) 0.857 0.827

3	(0.171) 0.143 0.173

Locus 16450 :

2 alleles

99.2% missing data

1	(0.437) 0.429 0.442

2	(0.563) 0.571 0.558

Locus 16451 :

2 alleles

99.2% missing data

3	(0.323) 0.314 0.336

2	(0.677) 0.686 0.664

Locus 16452 :

2 alleles

96.9% missing data

3	(0.723) 0.714 0.742

4	(0.277) 0.286 0.258

Locus 16453 :

2 alleles

98.8% missing data

3	(0.796) 0.804 0.791

1	(0.204) 0.196 0.209

Locus 16454 :

2 alleles

99.2% missing data

2	(0.806) 0.807 0.802

3	(0.194) 0.193 0.198

Locus 16455 :

2 alleles

99.2% missing data

2	(0.689) 0.684 0.692

3	(0.311) 0.316 0.308

Locus 16456 :

2 alleles

99.2% missing data

2	(0.690) 0.683 0.696

4	(0.310) 0.317 0.304

Locus 16457 :

2 alleles

98.8% missing data

3	(0.823) 0.814 0.827

4	(0.177) 0.186 0.173

Locus 16458 :

2 alleles

97.3% missing data

2	(0.734) 0.732 0.744

4	(0.266) 0.268 0.256

Locus 16459 :

2 alleles

98.4% missing data

2	(0.767) 0.764 0.762

4	(0.233) 0.236 0.238

Locus 16460 :

2 alleles

98.8% missing data

1	(0.196) 0.190 0.200

4	(0.804) 0.810 0.800

Locus 16461 :

2 alleles

99.2% missing data

3	(0.482) 0.473 0.483

2	(0.518) 0.527 0.517

Locus 16462 :

2 alleles

99.2% missing data

3	(0.432) 0.439 0.423

2	(0.568) 0.561 0.577

Locus 16463 :

2 alleles

91.0% missing data

4	(0.939) 0.953 0.933

3	(0.061) 0.047 0.067

Locus 16464 :

2 alleles

98.0% missing data

4	(0.860) 0.856 0.866

2	(0.140) 0.144 0.134

Locus 16465 :

2 alleles

99.2% missing data

2	(0.416) 0.420 0.415

3	(0.584) 0.580 0.585

Locus 16466 :

2 alleles

98.0% missing data

3	(0.762) 0.763 0.769

2	(0.238) 0.237 0.231

Locus 16467 :

2 alleles

99.2% missing data

2	(0.544) 0.541 0.549

4	(0.456) 0.459 0.451

Locus 16468 :

2 alleles

99.2% missing data

1	(0.451) 0.451 0.451

2	(0.549) 0.549 0.549

Locus 16469 :

2 alleles

99.2% missing data

2	(0.451) 0.445 0.461

3	(0.549) 0.555 0.539

Locus 16470 :

2 alleles

91.0% missing data

1	(0.941) 0.948 0.937

2	(0.059) 0.052 0.063

Locus 16471 :

2 alleles

99.2% missing data

4	(0.520) 0.526 0.517

1	(0.480) 0.474 0.483

Locus 16472 :

2 alleles

99.2% missing data

3	(0.467) 0.462 0.466

1	(0.533) 0.538 0.534

Locus 16473 :

2 alleles

99.2% missing data

3	(0.482) 0.482 0.486

4	(0.518) 0.518 0.514

Locus 16474 :

2 alleles

99.2% missing data

2	(0.634) 0.639 0.629

1	(0.366) 0.361 0.371

Locus 16475 :

2 alleles

99.2% missing data

2	(0.679) 0.681 0.678

4	(0.321) 0.319 0.322

Locus 16476 :

2 alleles

99.2% missing data

4	(0.504) 0.501 0.503

1	(0.496) 0.499 0.497

Locus 16477 :

2 alleles

98.4% missing data

2	(0.706) 0.706 0.705

1	(0.294) 0.294 0.295

Locus 16478 :

2 alleles

98.4% missing data

2	(0.807) 0.803 0.809

4	(0.193) 0.197 0.191

Locus 16479 :

2 alleles

97.7% missing data

3	(0.788) 0.791 0.789

2	(0.212) 0.209 0.211

Locus 16480 :

2 alleles

99.2% missing data

2	(0.644) 0.642 0.643

1	(0.356) 0.358 0.357

Locus 16481 :

2 alleles

98.8% missing data

3	(0.407) 0.407 0.412

1	(0.593) 0.593 0.588

Locus 16482 :

2 alleles

99.2% missing data

1	(0.207) 0.206 0.208

4	(0.793) 0.794 0.792

Locus 16483 :

2 alleles

99.2% missing data

1	(0.418) 0.421 0.420

4	(0.582) 0.579 0.580

Locus 16484 :

2 alleles

0.0% missing data

4	(0.986) 0.982 0.996

1	(0.014) 0.018 0.004

Locus 16485 :

2 alleles

98.4% missing data

3	(0.559) 0.551 0.564

4	(0.441) 0.449 0.436

Locus 16486 :

2 alleles

99.2% missing data

1	(0.238) 0.229 0.240

2	(0.762) 0.771 0.760

Locus 16487 :

2 alleles

99.2% missing data

4	(0.595) 0.590 0.598

1	(0.405) 0.410 0.402

Locus 16488 :

2 alleles

96.1% missing data

1	(0.807) 0.799 0.827

3	(0.193) 0.201 0.173

Locus 16489 :

2 alleles

99.2% missing data

1	(0.537) 0.535 0.537

2	(0.463) 0.465 0.463

Locus 16490 :

2 alleles

99.2% missing data

3	(0.490) 0.493 0.489

4	(0.510) 0.507 0.511

Locus 16491 :

2 alleles

99.2% missing data

2	(0.735) 0.729 0.733

3	(0.265) 0.271 0.267

Locus 16492 :

2 alleles

99.2% missing data

3	(0.361) 0.365 0.356

2	(0.639) 0.635 0.644

Locus 16493 :

2 alleles

99.2% missing data

2	(0.257) 0.254 0.263

1	(0.743) 0.746 0.737

Locus 16494 :

2 alleles

98.8% missing data

1	(0.447) 0.443 0.457

2	(0.553) 0.557 0.543

Locus 16495 :

2 alleles

99.2% missing data

3	(0.431) 0.424 0.434

4	(0.569) 0.576 0.566

Locus 16496 :

2 alleles

98.0% missing data

1	(0.518) 0.533 0.518

2	(0.482) 0.467 0.482

Locus 16497 :

2 alleles

98.4% missing data

3	(0.830) 0.835 0.821

1	(0.170) 0.165 0.179

Locus 16498 :

2 alleles

99.2% missing data

2	(0.465) 0.467 0.468

4	(0.535) 0.533 0.532

Locus 16499 :

2 alleles

98.8% missing data

1	(0.655) 0.648 0.660

4	(0.345) 0.352 0.340

Locus 16500 :

2 alleles

97.7% missing data

3	(0.261) 0.260 0.257

4	(0.739) 0.740 0.743

Locus 16501 :

2 alleles

99.2% missing data

3	(0.624) 0.626 0.620

1	(0.376) 0.374 0.380

Locus 16502 :

2 alleles

99.2% missing data

4	(0.456) 0.459 0.459

2	(0.544) 0.541 0.541

Locus 16503 :

2 alleles

99.2% missing data

1	(0.646) 0.640 0.653

4	(0.354) 0.360 0.347

Locus 16504 :

2 alleles

99.2% missing data

3	(0.398) 0.401 0.395

2	(0.602) 0.599 0.605

Locus 16505 :

2 alleles

98.4% missing data

2	(0.204) 0.197 0.207

4	(0.796) 0.803 0.793

Locus 16506 :

3 alleles

98.8% missing data

4	(0.243) 0.241 0.239

3	(0.446) 0.440 0.453

2	(0.311) 0.319 0.308

Locus 16507 :

2 alleles

99.2% missing data

2	(0.325) 0.330 0.325

4	(0.675) 0.670 0.675

Locus 16508 :

2 alleles

99.2% missing data

1	(0.124) 0.117 0.131

3	(0.876) 0.883 0.869

Locus 16509 :

2 alleles

99.2% missing data

3	(0.430) 0.432 0.428

2	(0.570) 0.568 0.572

Locus 16510 :

2 alleles

99.2% missing data

4	(0.471) 0.465 0.473

3	(0.529) 0.535 0.527

Locus 16511 :

2 alleles

96.9% missing data

1	(0.832) 0.828 0.841

4	(0.168) 0.172 0.159

Locus 16512 :

2 alleles

99.2% missing data

2	(0.474) 0.468 0.480

1	(0.526) 0.532 0.520

Locus 16513 :

2 alleles

99.2% missing data

1	(0.398) 0.392 0.404

3	(0.602) 0.608 0.596

Locus 16514 :

2 alleles

8.2% missing data

2	(0.984) 0.977 0.995

4	(0.016) 0.023 0.005

Locus 16515 :

2 alleles

98.0% missing data

4	(0.298) 0.287 0.312

1	(0.702) 0.713 0.688

Locus 16516 :

2 alleles

99.2% missing data

2	(0.554) 0.544 0.559

3	(0.446) 0.456 0.441

Locus 16517 :

2 alleles

98.8% missing data

4	(0.848) 0.843 0.851

1	(0.152) 0.157 0.149

Locus 16518 :

2 alleles

99.2% missing data

2	(0.472) 0.470 0.476

4	(0.528) 0.530 0.524

Locus 16519 :

2 alleles

98.8% missing data

4	(0.508) 0.503 0.506

2	(0.492) 0.497 0.494

Locus 16520 :

2 alleles

93.0% missing data

3	(0.123) 0.128 0.107

4	(0.877) 0.872 0.893

Locus 16521 :

2 alleles

98.8% missing data

3	(0.624) 0.619 0.630

1	(0.376) 0.381 0.370

Locus 16522 :

2 alleles

99.2% missing data

2	(0.589) 0.596 0.581

3	(0.411) 0.404 0.419

Locus 16523 :

2 alleles

99.2% missing data

1	(0.286) 0.285 0.290

3	(0.714) 0.715 0.710

Locus 16524 :

2 alleles

99.2% missing data

1	(0.711) 0.713 0.710

3	(0.289) 0.287 0.290

Locus 16525 :

2 alleles

98.8% missing data

1	(0.743) 0.747 0.742

3	(0.257) 0.253 0.258

Locus 16526 :

2 alleles

98.8% missing data

1	(0.766) 0.765 0.763

3	(0.234) 0.235 0.237

Locus 16527 :

2 alleles

99.2% missing data

4	(0.662) 0.658 0.662

3	(0.338) 0.342 0.338

Locus 16528 :

3 alleles

98.8% missing data

4	(0.327) 0.328 0.327

3	(0.491) 0.484 0.493

2	(0.182) 0.188 0.180

Locus 16529 :

2 alleles

99.2% missing data

4	(0.577) 0.584 0.570

2	(0.423) 0.416 0.430

Locus 16530 :

2 alleles

99.2% missing data

3	(0.382) 0.379 0.390

4	(0.618) 0.621 0.610

Locus 16531 :

2 alleles

99.2% missing data

4	(0.782) 0.787 0.779

1	(0.218) 0.213 0.221

Locus 16532 :

2 alleles

98.4% missing data

2	(0.621) 0.613 0.626

4	(0.379) 0.387 0.374

Locus 16533 :

2 alleles

99.2% missing data

2	(0.319) 0.324 0.321

1	(0.681) 0.676 0.679

Locus 16534 :

2 alleles

99.2% missing data

2	(0.110) 0.113 0.114

3	(0.890) 0.887 0.886

Locus 16535 :

2 alleles

99.2% missing data

1	(0.490) 0.484 0.493

4	(0.510) 0.516 0.507

Locus 16536 :

2 alleles

99.2% missing data

2	(0.310) 0.306 0.320

4	(0.690) 0.694 0.680

Locus 16537 :

2 alleles

99.2% missing data

2	(0.282) 0.275 0.283

4	(0.718) 0.725 0.717

Locus 16538 :

2 alleles

99.2% missing data

1	(0.729) 0.733 0.728

3	(0.271) 0.267 0.272

Locus 16539 :

2 alleles

99.2% missing data

1	(0.700) 0.705 0.699

2	(0.300) 0.295 0.301

Locus 16540 :

2 alleles

99.2% missing data

1	(0.408) 0.409 0.411

2	(0.592) 0.591 0.589

Locus 16541 :

2 alleles

99.2% missing data

4	(0.446) 0.455 0.437

1	(0.554) 0.545 0.563

Locus 16542 :

2 alleles

99.2% missing data

1	(0.469) 0.461 0.476

3	(0.531) 0.539 0.524

Locus 16543 :

2 alleles

99.2% missing data

2	(0.532) 0.531 0.538

1	(0.468) 0.469 0.462

Locus 16544 :

2 alleles

99.2% missing data

2	(0.672) 0.670 0.670

4	(0.328) 0.330 0.330

Locus 16545 :

2 alleles

99.2% missing data

3	(0.520) 0.524 0.513

2	(0.480) 0.476 0.487

Locus 16546 :

2 alleles

99.2% missing data

3	(0.464) 0.463 0.463

4	(0.536) 0.537 0.537

Locus 16547 :

2 alleles

99.2% missing data

1	(0.324) 0.321 0.331

3	(0.676) 0.679 0.669

Locus 16548 :

2 alleles

98.8% missing data

1	(0.644) 0.651 0.648

4	(0.356) 0.349 0.352

Locus 16549 :

2 alleles

99.2% missing data

1	(0.256) 0.263 0.252

3	(0.744) 0.737 0.748

Locus 16550 :

2 alleles

99.2% missing data

1	(0.206) 0.215 0.206

4	(0.794) 0.785 0.794

Locus 16551 :

2 alleles

99.2% missing data

3	(0.528) 0.533 0.524

4	(0.472) 0.467 0.476

Locus 16552 :

2 alleles

99.2% missing data

2	(0.391) 0.388 0.390

3	(0.609) 0.612 0.610

Locus 16553 :

2 alleles

1.6% missing data

1	(0.987) 0.990 0.993

4	(0.013) 0.010 0.007

Locus 16554 :

4 alleles

98.4% missing data

4	(0.286) 0.286 0.290

1	(0.360) 0.355 0.356

3	(0.093) 0.090 0.099

2	(0.261) 0.269 0.255

Locus 16555 :

3 alleles

98.8% missing data

3	(0.413) 0.408 0.419

2	(0.356) 0.363 0.342

1	(0.231) 0.229 0.239

Locus 16556 :

2 alleles

99.2% missing data

3	(0.609) 0.608 0.604

1	(0.391) 0.392 0.396

Locus 16557 :

2 alleles

98.4% missing data

2	(0.764) 0.758 0.771

3	(0.236) 0.242 0.229

Locus 16558 :

2 alleles

99.2% missing data

3	(0.605) 0.602 0.609

1	(0.395) 0.398 0.391

Locus 16559 :

2 alleles

98.8% missing data

3	(0.730) 0.731 0.726

1	(0.270) 0.269 0.274

Locus 16560 :

2 alleles

99.2% missing data

3	(0.517) 0.513 0.517

1	(0.483) 0.487 0.483

Locus 16561 :

2 alleles

99.2% missing data

4	(0.470) 0.465 0.472

1	(0.530) 0.535 0.528

Locus 16562 :

2 alleles

99.2% missing data

3	(0.683) 0.675 0.687

2	(0.317) 0.325 0.313

Locus 16563 :

3 alleles

98.8% missing data

2	(0.482) 0.485 0.483

1	(0.218) 0.214 0.222

3	(0.300) 0.301 0.294

Locus 16564 :

2 alleles

97.7% missing data

1	(0.807) 0.812 0.804

2	(0.193) 0.188 0.196

Locus 16565 :

2 alleles

99.2% missing data

1	(0.401) 0.395 0.409

3	(0.599) 0.605 0.591

Locus 16566 :

2 alleles

99.2% missing data

2	(0.421) 0.418 0.423

4	(0.579) 0.582 0.577

Locus 16567 :

2 alleles

99.2% missing data

4	(0.712) 0.717 0.712

1	(0.288) 0.283 0.288

Locus 16568 :

2 alleles

99.2% missing data

3	(0.628) 0.627 0.626

2	(0.372) 0.373 0.374

Locus 16569 :

2 alleles

98.4% missing data

3	(0.856) 0.855 0.858

1	(0.144) 0.145 0.142

Locus 16570 :

2 alleles

99.2% missing data

1	(0.208) 0.219 0.201

3	(0.792) 0.781 0.799

Locus 16571 :

2 alleles

99.2% missing data

3	(0.738) 0.744 0.734

2	(0.262) 0.256 0.266

Locus 16572 :

2 alleles

98.0% missing data

2	(0.867) 0.875 0.861

4	(0.133) 0.125 0.139

Locus 16573 :

2 alleles

0.0% missing data

2	(0.987) 0.991 0.993

3	(0.013) 0.009 0.007

Locus 16574 :

2 alleles

96.9% missing data

1	(0.826) 0.816 0.840

4	(0.174) 0.184 0.160

Locus 16575 :

2 alleles

99.2% missing data

4	(0.289) 0.283 0.292

1	(0.711) 0.717 0.708

Locus 16576 :

3 alleles

98.4% missing data

3	(0.372) 0.368 0.377

4	(0.390) 0.384 0.391

1	(0.239) 0.248 0.231

Locus 16577 :

2 alleles

99.2% missing data

2	(0.351) 0.353 0.350

4	(0.649) 0.647 0.650

Locus 16578 :

2 alleles

95.7% missing data

4	(0.278) 0.262 0.287

1	(0.722) 0.738 0.713

Locus 16579 :

2 alleles

98.0% missing data

1	(0.695) 0.698 0.693

3	(0.305) 0.302 0.307

Locus 16580 :

2 alleles

99.2% missing data

3	(0.271) 0.272 0.270

1	(0.729) 0.728 0.730

Locus 16581 :

2 alleles

99.2% missing data

3	(0.288) 0.283 0.296

1	(0.712) 0.717 0.704

Locus 16582 :

2 alleles

98.0% missing data

3	(0.513) 0.511 0.521

2	(0.487) 0.489 0.479

Locus 16583 :

2 alleles

99.2% missing data

2	(0.573) 0.569 0.582

1	(0.427) 0.431 0.418

Locus 16584 :

2 alleles

93.4% missing data

3	(0.796) 0.799 0.812

2	(0.204) 0.201 0.188

Locus 16585 :

2 alleles

99.2% missing data

4	(0.491) 0.497 0.485

3	(0.509) 0.503 0.515

Locus 16586 :

2 alleles

98.8% missing data

4	(0.790) 0.786 0.799

2	(0.210) 0.214 0.201

Locus 16587 :

2 alleles

98.0% missing data

1	(0.287) 0.280 0.295

3	(0.713) 0.720 0.705

Locus 16588 :

2 alleles

99.2% missing data

1	(0.485) 0.483 0.491

3	(0.515) 0.517 0.509

Locus 16589 :

3 alleles

98.8% missing data

4	(0.208) 0.216 0.207

3	(0.473) 0.467 0.477

1	(0.319) 0.316 0.316

Locus 16590 :

2 alleles

98.8% missing data

2	(0.335) 0.335 0.330

1	(0.665) 0.665 0.670

Locus 16591 :

2 alleles

99.2% missing data

3	(0.528) 0.527 0.530

2	(0.472) 0.473 0.470

Locus 16592 :

2 alleles

98.0% missing data

1	(0.731) 0.727 0.740

3	(0.269) 0.273 0.260

Locus 16593 :

2 alleles

99.2% missing data

3	(0.686) 0.682 0.691

4	(0.314) 0.318 0.309

Locus 16594 :

2 alleles

99.2% missing data

3	(0.384) 0.387 0.379

1	(0.616) 0.613 0.621

Locus 16595 :

2 alleles

98.8% missing data

1	(0.424) 0.419 0.429

4	(0.576) 0.581 0.571

Locus 16596 :

2 alleles

99.2% missing data

3	(0.368) 0.366 0.373

2	(0.632) 0.634 0.627

Locus 16597 :

2 alleles

99.2% missing data

2	(0.306) 0.299 0.308

3	(0.694) 0.701 0.692

Locus 16598 :

2 alleles

98.4% missing data

4	(0.295) 0.286 0.294

3	(0.705) 0.714 0.706

Locus 16599 :

2 alleles

99.2% missing data

4	(0.312) 0.309 0.315

3	(0.688) 0.691 0.685

Locus 16600 :

2 alleles

0.0% missing data

2	(0.988) 0.983 0.996

1	(0.012) 0.017 0.004

Locus 16601 :

2 alleles

99.2% missing data

2	(0.499) 0.498 0.501

3	(0.501) 0.502 0.499

Locus 16602 :

2 alleles

99.2% missing data

3	(0.631) 0.631 0.633

1	(0.369) 0.369 0.367

Locus 16603 :

2 alleles

99.2% missing data

3	(0.498) 0.499 0.501

4	(0.502) 0.501 0.499

Locus 16604 :

2 alleles

85.2% missing data

2	(0.927) 0.923 0.943

3	(0.073) 0.077 0.057

Locus 16605 :

2 alleles

98.8% missing data

2	(0.533) 0.528 0.545

3	(0.467) 0.472 0.455

Locus 16606 :

2 alleles

98.8% missing data

4	(0.684) 0.680 0.690

3	(0.316) 0.320 0.310

Locus 16607 :

2 alleles

98.8% missing data

2	(0.511) 0.511 0.512

3	(0.489) 0.489 0.488

Locus 16608 :

2 alleles

99.2% missing data

2	(0.248) 0.246 0.252

1	(0.752) 0.754 0.748

Locus 16609 :

2 alleles

99.2% missing data

2	(0.589) 0.592 0.586

3	(0.411) 0.408 0.414

Locus 16610 :

2 alleles

99.2% missing data

2	(0.487) 0.493 0.488

3	(0.513) 0.507 0.512

Locus 16611 :

2 alleles

99.2% missing data

3	(0.339) 0.338 0.341

1	(0.661) 0.662 0.659

Locus 16612 :

2 alleles

99.2% missing data

3	(0.589) 0.586 0.586

2	(0.411) 0.414 0.414

Locus 16613 :

2 alleles

99.2% missing data

1	(0.360) 0.364 0.356

3	(0.640) 0.636 0.644

Locus 16614 :

2 alleles

89.8% missing data

1	(0.950) 0.953 0.952

3	(0.050) 0.047 0.048

Locus 16615 :

2 alleles

98.8% missing data

2	(0.641) 0.634 0.648

4	(0.359) 0.366 0.352

Locus 16616 :

2 alleles

99.2% missing data

3	(0.671) 0.659 0.676

2	(0.329) 0.341 0.324

Locus 16617 :

2 alleles

99.2% missing data

3	(0.515) 0.513 0.523

4	(0.485) 0.487 0.477

Locus 16618 :

2 alleles

99.2% missing data

4	(0.719) 0.715 0.724

1	(0.281) 0.285 0.276

Locus 16619 :

2 alleles

99.2% missing data

1	(0.682) 0.677 0.690

2	(0.318) 0.323 0.310

Locus 16620 :

2 alleles

99.2% missing data

3	(0.536) 0.526 0.536

2	(0.464) 0.474 0.464

Locus 16621 :

2 alleles

99.2% missing data

3	(0.709) 0.700 0.713

1	(0.291) 0.300 0.287

Locus 16622 :

2 alleles

99.2% missing data

2	(0.395) 0.394 0.391

3	(0.605) 0.606 0.609

Locus 16623 :

2 alleles

99.2% missing data

3	(0.682) 0.680 0.683

4	(0.318) 0.320 0.317

Locus 16624 :

2 alleles

98.0% missing data

3	(0.468) 0.472 0.472

1	(0.532) 0.528 0.528

Locus 16625 :

2 alleles

99.2% missing data

4	(0.469) 0.469 0.469

1	(0.531) 0.531 0.531

Locus 16626 :

2 alleles

98.8% missing data

1	(0.666) 0.660 0.670

2	(0.334) 0.340 0.330

Locus 16627 :

2 alleles

93.0% missing data

3	(0.861) 0.861 0.871

1	(0.139) 0.139 0.129

Locus 16628 :

2 alleles

8.6% missing data

3	(0.982) 0.975 0.994

4	(0.018) 0.025 0.006

Locus 16629 :

2 alleles

99.2% missing data

3	(0.711) 0.713 0.706

4	(0.289) 0.287 0.294

Locus 16630 :

2 alleles

98.8% missing data

2	(0.468) 0.472 0.475

4	(0.532) 0.528 0.525

Locus 16631 :

3 alleles

96.9% missing data

1	(0.319) 0.328 0.315

2	(0.173) 0.164 0.177

4	(0.508) 0.508 0.508

Locus 16632 :

2 alleles

99.2% missing data

1	(0.733) 0.724 0.740

2	(0.267) 0.276 0.260

Locus 16633 :

2 alleles

98.8% missing data

3	(0.183) 0.177 0.189

2	(0.817) 0.823 0.811

Locus 16634 :

2 alleles

99.2% missing data

1	(0.359) 0.362 0.350

3	(0.641) 0.638 0.650

Locus 16635 :

2 alleles

99.2% missing data

2	(0.330) 0.328 0.331

1	(0.670) 0.672 0.669

Locus 16636 :

2 alleles

99.2% missing data

4	(0.458) 0.454 0.464

2	(0.542) 0.546 0.536

Locus 16637 :

2 alleles

97.3% missing data

3	(0.198) 0.197 0.200

1	(0.802) 0.803 0.800

Locus 16638 :

2 alleles

99.2% missing data

3	(0.479) 0.481 0.482

2	(0.521) 0.519 0.518

Locus 16639 :

2 alleles

85.2% missing data

4	(0.873) 0.860 0.895

2	(0.127) 0.140 0.105

Locus 16640 :

2 alleles

99.2% missing data

2	(0.318) 0.309 0.329

3	(0.682) 0.691 0.671

Locus 16641 :

2 alleles

98.8% missing data

2	(0.601) 0.598 0.606

1	(0.399) 0.402 0.394

Locus 16642 :

2 alleles

99.2% missing data

3	(0.334) 0.332 0.340

2	(0.666) 0.668 0.660

Locus 16643 :

2 alleles

98.4% missing data

4	(0.691) 0.694 0.690

3	(0.309) 0.306 0.310

Locus 16644 :

2 alleles

98.8% missing data

4	(0.408) 0.406 0.408

3	(0.592) 0.594 0.592

Locus 16645 :

2 alleles

0.0% missing data

4	(0.984) 0.980 0.995

1	(0.016) 0.020 0.005

Locus 16646 :

2 alleles

98.8% missing data

4	(0.695) 0.690 0.700

3	(0.305) 0.310 0.300

Locus 16647 :

2 alleles

99.2% missing data

4	(0.305) 0.304 0.308

3	(0.695) 0.696 0.692

Locus 16648 :

2 alleles

96.5% missing data

2	(0.847) 0.838 0.860

1	(0.153) 0.162 0.140

Locus 16649 :

2 alleles

99.2% missing data

4	(0.410) 0.416 0.413

3	(0.590) 0.584 0.587

Locus 16650 :

3 alleles

98.8% missing data

4	(0.326) 0.325 0.332

1	(0.438) 0.438 0.435

3	(0.236) 0.237 0.233

Locus 16651 :

2 alleles

99.2% missing data

4	(0.732) 0.731 0.733

1	(0.268) 0.269 0.267

Locus 16652 :

2 alleles

99.2% missing data

4	(0.247) 0.253 0.244

3	(0.753) 0.747 0.756

Locus 16653 :

2 alleles

99.2% missing data

3	(0.519) 0.514 0.520

2	(0.481) 0.486 0.480

Locus 16654 :

2 alleles

98.8% missing data

4	(0.401) 0.398 0.404

1	(0.599) 0.602 0.596

Locus 16655 :

2 alleles

99.2% missing data

1	(0.513) 0.514 0.514

3	(0.487) 0.486 0.486

Locus 16656 :

2 alleles

99.2% missing data

4	(0.319) 0.318 0.322

1	(0.681) 0.682 0.678

Locus 16657 :

2 alleles

99.2% missing data

3	(0.671) 0.674 0.666

2	(0.329) 0.326 0.334

Locus 16658 :

2 alleles

99.2% missing data

3	(0.544) 0.545 0.541

1	(0.456) 0.455 0.459

Locus 16659 :

2 alleles

99.2% missing data

1	(0.652) 0.649 0.655

3	(0.348) 0.351 0.345

Locus 16660 :

2 alleles

99.2% missing data

1	(0.407) 0.401 0.416

4	(0.593) 0.599 0.584

Locus 16661 :

2 alleles

98.8% missing data

4	(0.521) 0.523 0.521

1	(0.479) 0.477 0.479

Locus 16662 :

2 alleles

99.2% missing data

3	(0.612) 0.608 0.616

1	(0.388) 0.392 0.384

Locus 16663 :

2 alleles

99.2% missing data

2	(0.754) 0.754 0.758

1	(0.246) 0.246 0.242

Locus 16664 :

2 alleles

98.8% missing data

4	(0.526) 0.519 0.538

2	(0.474) 0.481 0.462

Locus 16665 :

2 alleles

99.2% missing data

4	(0.696) 0.694 0.693

2	(0.304) 0.306 0.307

Locus 16666 :

2 alleles

99.2% missing data

3	(0.753) 0.753 0.750

2	(0.247) 0.247 0.250

Locus 16667 :

2 alleles

99.2% missing data

3	(0.422) 0.423 0.425

2	(0.578) 0.577 0.575

Locus 16668 :

2 alleles

99.2% missing data

3	(0.505) 0.496 0.512

1	(0.495) 0.504 0.488

Locus 16669 :

2 alleles

99.2% missing data

3	(0.417) 0.414 0.417

4	(0.583) 0.586 0.583

Locus 16670 :

2 alleles

93.8% missing data

4	(0.884) 0.887 0.890

3	(0.116) 0.113 0.110

Locus 16671 :

2 alleles

99.2% missing data

2	(0.238) 0.237 0.243

3	(0.762) 0.763 0.757

Locus 16672 :

2 alleles

99.2% missing data

3	(0.648) 0.650 0.648

4	(0.352) 0.350 0.352

Locus 16673 :

2 alleles

98.0% missing data

4	(0.706) 0.707 0.712

1	(0.294) 0.293 0.288

Locus 16674 :

2 alleles

97.7% missing data

3	(0.133) 0.139 0.129

1	(0.867) 0.861 0.871

Locus 16675 :

2 alleles

99.2% missing data

1	(0.648) 0.642 0.652

4	(0.352) 0.358 0.348

Locus 16676 :

2 alleles

99.2% missing data

4	(0.341) 0.344 0.337

1	(0.659) 0.656 0.663

Locus 16677 :

2 alleles

99.2% missing data

2	(0.372) 0.372 0.383

4	(0.628) 0.628 0.617

Locus 16678 :

3 alleles

93.0% missing data

3	(0.813) 0.805 0.833

1	(0.120) 0.122 0.111

2	(0.067) 0.073 0.056

Locus 16679 :

2 alleles

99.2% missing data

3	(0.346) 0.344 0.351

1	(0.654) 0.656 0.649

Locus 16680 :

2 alleles

99.2% missing data

1	(0.523) 0.524 0.523

4	(0.477) 0.476 0.477

Locus 16681 :

2 alleles

99.2% missing data

1	(0.630) 0.637 0.627

3	(0.370) 0.363 0.373

Locus 16682 :

2 alleles

99.2% missing data

1	(0.323) 0.321 0.326

4	(0.677) 0.679 0.674

Locus 16683 :

2 alleles

99.2% missing data

3	(0.542) 0.535 0.543

2	(0.458) 0.465 0.457

Locus 16684 :

2 alleles

99.2% missing data

4	(0.686) 0.685 0.686

3	(0.314) 0.315 0.314

Locus 16685 :

2 alleles

99.2% missing data

1	(0.437) 0.433 0.444

4	(0.563) 0.567 0.556

Locus 16686 :

2 alleles

99.2% missing data

2	(0.477) 0.474 0.476

4	(0.523) 0.526 0.524

Locus 16687 :

2 alleles

99.2% missing data

4	(0.531) 0.537 0.528

1	(0.469) 0.463 0.472

Locus 16688 :

2 alleles

99.2% missing data

3	(0.349) 0.355 0.351

4	(0.651) 0.645 0.649

Locus 16689 :

2 alleles

99.2% missing data

3	(0.402) 0.397 0.411

4	(0.598) 0.603 0.589

Locus 16690 :

2 alleles

96.1% missing data

1	(0.848) 0.856 0.851

4	(0.152) 0.144 0.149

Locus 16691 :

2 alleles

91.0% missing data

4	(0.935) 0.947 0.928

2	(0.065) 0.053 0.072

Locus 16692 :

2 alleles

99.2% missing data

4	(0.450) 0.446 0.458

2	(0.550) 0.554 0.542

Locus 16693 :

2 alleles

97.7% missing data

1	(0.218) 0.216 0.212

2	(0.782) 0.784 0.788

Locus 16694 :

2 alleles

99.2% missing data

3	(0.376) 0.370 0.381

4	(0.624) 0.630 0.619

Locus 16695 :

2 alleles

99.2% missing data

2	(0.502) 0.490 0.510

4	(0.498) 0.510 0.490

Locus 16696 :

2 alleles

6.2% missing data

1	(0.984) 0.978 0.995

2	(0.016) 0.022 0.005

Locus 16697 :

2 alleles

8.2% missing data

2	(0.982) 0.975 0.995

3	(0.018) 0.025 0.005

Locus 16698 :

2 alleles

99.2% missing data

4	(0.578) 0.574 0.583

3	(0.422) 0.426 0.417

Locus 16699 :

2 alleles

99.2% missing data

4	(0.620) 0.621 0.618

3	(0.380) 0.379 0.382

Locus 16700 :

2 alleles

99.2% missing data

2	(0.389) 0.389 0.394

3	(0.611) 0.611 0.606

Locus 16701 :

2 alleles

99.2% missing data

2	(0.344) 0.342 0.352

1	(0.656) 0.658 0.648

Locus 16702 :

2 alleles

99.2% missing data

4	(0.687) 0.689 0.684

3	(0.313) 0.311 0.316

Locus 16703 :

2 alleles

99.2% missing data

3	(0.423) 0.420 0.425

2	(0.577) 0.580 0.575

Locus 16704 :

2 alleles

99.2% missing data

4	(0.297) 0.292 0.303

2	(0.703) 0.708 0.697

Locus 16705 :

2 alleles

97.3% missing data

2	(0.336) 0.335 0.330

3	(0.664) 0.665 0.670

Locus 16706 :

2 alleles

99.2% missing data

4	(0.272) 0.275 0.269

3	(0.728) 0.725 0.731

Locus 16707 :

2 alleles

99.2% missing data

4	(0.704) 0.706 0.705

3	(0.296) 0.294 0.295

Locus 16708 :

2 alleles

99.2% missing data

1	(0.658) 0.654 0.671

4	(0.342) 0.346 0.329

Locus 16709 :

2 alleles

99.2% missing data

3	(0.361) 0.362 0.365

1	(0.639) 0.638 0.635

Locus 16710 :

2 alleles

96.1% missing data

1	(0.825) 0.836 0.819

4	(0.175) 0.164 0.181

Locus 16711 :

2 alleles

99.2% missing data

3	(0.513) 0.509 0.517

1	(0.487) 0.491 0.483

Locus 16712 :

2 alleles

96.9% missing data

2	(0.091) 0.088 0.092

1	(0.909) 0.912 0.908

Locus 16713 :

2 alleles

99.2% missing data

2	(0.463) 0.461 0.463

3	(0.537) 0.539 0.537

Locus 16714 :

2 alleles

99.2% missing data

2	(0.620) 0.619 0.611

3	(0.380) 0.381 0.389

Locus 16715 :

2 alleles

99.2% missing data

4	(0.653) 0.655 0.652

3	(0.347) 0.345 0.348

Locus 16716 :

2 alleles

98.4% missing data

1	(0.242) 0.238 0.250

4	(0.758) 0.762 0.750

Locus 16717 :

2 alleles

98.4% missing data

2	(0.683) 0.692 0.682

1	(0.317) 0.308 0.318

Locus 16718 :

2 alleles

95.3% missing data

3	(0.131) 0.135 0.122

4	(0.869) 0.865 0.878

Locus 16719 :

2 alleles

98.8% missing data

3	(0.690) 0.691 0.695

2	(0.310) 0.309 0.305

Locus 16720 :

2 alleles

99.2% missing data

2	(0.366) 0.357 0.377

4	(0.634) 0.643 0.623

Locus 16721 :

2 alleles

99.2% missing data

3	(0.736) 0.738 0.735

1	(0.264) 0.262 0.265

Locus 16722 :

2 alleles

99.2% missing data

4	(0.667) 0.659 0.668

2	(0.333) 0.341 0.332

Locus 16723 :

3 alleles

98.8% missing data

4	(0.585) 0.580 0.588

1	(0.073) 0.080 0.070

3	(0.342) 0.340 0.342

Locus 16724 :

2 alleles

99.2% missing data

4	(0.245) 0.250 0.240

1	(0.755) 0.750 0.760

Locus 16725 :

2 alleles

96.1% missing data

2	(0.907) 0.918 0.903

1	(0.093) 0.082 0.097

Locus 16726 :

3 alleles

92.6% missing data

4	(0.821) 0.824 0.834

1	(0.075) 0.070 0.071

3	(0.104) 0.105 0.094

Locus 16727 :

2 alleles

98.8% missing data

2	(0.784) 0.782 0.775

3	(0.216) 0.218 0.225

Locus 16728 :

2 alleles

99.2% missing data

1	(0.619) 0.620 0.619

2	(0.381) 0.380 0.381

Locus 16729 :

2 alleles

99.2% missing data

3	(0.438) 0.436 0.436

4	(0.562) 0.564 0.564

Locus 16730 :

2 alleles

98.8% missing data

2	(0.593) 0.594 0.596

3	(0.407) 0.406 0.404

Locus 16731 :

2 alleles

90.6% missing data

2	(0.621) 0.577 0.668

1	(0.379) 0.423 0.332

Locus 16732 :

2 alleles

99.2% missing data

1	(0.465) 0.458 0.475

4	(0.535) 0.542 0.525

Locus 16733 :

2 alleles

99.2% missing data

3	(0.324) 0.325 0.328

4	(0.676) 0.675 0.672

Locus 16734 :

2 alleles

99.2% missing data

3	(0.509) 0.506 0.510

4	(0.491) 0.494 0.490

Locus 16735 :

2 alleles

99.2% missing data

4	(0.387) 0.378 0.392

1	(0.613) 0.622 0.608

Locus 16736 :

2 alleles

99.2% missing data

3	(0.392) 0.401 0.388

1	(0.608) 0.599 0.612

Locus 16737 :

2 alleles

99.2% missing data

1	(0.391) 0.390 0.387

2	(0.609) 0.610 0.613

Locus 16738 :

2 alleles

99.2% missing data

4	(0.391) 0.391 0.393

3	(0.609) 0.609 0.607

Locus 16739 :

2 alleles

98.0% missing data

2	(0.424) 0.427 0.433

3	(0.576) 0.573 0.567

Locus 16740 :

2 alleles

99.2% missing data

4	(0.144) 0.147 0.146

3	(0.856) 0.853 0.854

Locus 16741 :

3 alleles

96.5% missing data

1	(0.202) 0.193 0.207

4	(0.611) 0.631 0.604

2	(0.187) 0.176 0.189

Locus 16742 :

2 alleles

99.2% missing data

2	(0.290) 0.288 0.286

3	(0.710) 0.712 0.714

Locus 16743 :

2 alleles

96.5% missing data

2	(0.801) 0.808 0.803

3	(0.199) 0.192 0.197

Locus 16744 :

2 alleles

97.3% missing data

1	(0.903) 0.905 0.906

3	(0.097) 0.095 0.094

Locus 16745 :

2 alleles

99.2% missing data

2	(0.556) 0.563 0.544

4	(0.444) 0.437 0.456

Locus 16746 :

2 alleles

98.8% missing data

2	(0.728) 0.720 0.730

1	(0.272) 0.280 0.270

Locus 16747 :

2 alleles

86.3% missing data

3	(0.949) 0.939 0.964

4	(0.051) 0.061 0.036

Locus 16748 :

2 alleles

99.2% missing data

3	(0.367) 0.372 0.369

4	(0.633) 0.628 0.631

Locus 16749 :

2 alleles

99.2% missing data

4	(0.762) 0.766 0.755

3	(0.238) 0.234 0.245

Locus 16750 :

2 alleles

99.2% missing data

4	(0.468) 0.462 0.471

3	(0.532) 0.538 0.529

Locus 16751 :

2 alleles

99.2% missing data

2	(0.264) 0.272 0.259

4	(0.736) 0.728 0.741

Locus 16752 :

2 alleles

99.2% missing data

2	(0.737) 0.731 0.740

4	(0.263) 0.269 0.260

Locus 16753 :

2 alleles

89.8% missing data

1	(0.925) 0.941 0.915

3	(0.075) 0.059 0.085

Locus 16754 :

2 alleles

99.2% missing data

2	(0.532) 0.536 0.534

3	(0.468) 0.464 0.466

Locus 16755 :

2 alleles

96.5% missing data

4	(0.872) 0.871 0.872

2	(0.128) 0.129 0.128

Locus 16756 :

2 alleles

99.2% missing data

4	(0.671) 0.674 0.669

2	(0.329) 0.326 0.331

Locus 16757 :

2 alleles

99.2% missing data

4	(0.485) 0.486 0.484

2	(0.515) 0.514 0.516

Locus 16758 :

2 alleles

98.4% missing data

2	(0.666) 0.656 0.675

3	(0.334) 0.344 0.325

Locus 16759 :

2 alleles

99.2% missing data

1	(0.473) 0.477 0.474

3	(0.527) 0.523 0.526

Locus 16760 :

2 alleles

98.8% missing data

1	(0.315) 0.319 0.317

2	(0.685) 0.681 0.683

Locus 16761 :

2 alleles

98.0% missing data

3	(0.623) 0.635 0.621

1	(0.377) 0.365 0.379

Locus 16762 :

2 alleles

99.2% missing data

3	(0.593) 0.590 0.594

1	(0.407) 0.410 0.406

Locus 16763 :

3 alleles

98.8% missing data

1	(0.263) 0.264 0.271

3	(0.222) 0.219 0.226

4	(0.514) 0.516 0.503

Locus 16764 :

2 alleles

99.2% missing data

2	(0.520) 0.528 0.516

4	(0.480) 0.472 0.484

Locus 16765 :

2 alleles

99.2% missing data

2	(0.262) 0.261 0.261

1	(0.738) 0.739 0.739

Locus 16766 :

2 alleles

99.2% missing data

2	(0.502) 0.499 0.504

4	(0.498) 0.501 0.496

Locus 16767 :

2 alleles

99.2% missing data

3	(0.367) 0.372 0.360

2	(0.633) 0.628 0.640

Locus 16768 :

2 alleles

99.2% missing data

3	(0.519) 0.524 0.519

2	(0.481) 0.476 0.481

Locus 16769 :

2 alleles

99.2% missing data

4	(0.432) 0.432 0.429

2	(0.568) 0.568 0.571

Locus 16770 :

2 alleles

98.8% missing data

3	(0.638) 0.639 0.642

2	(0.362) 0.361 0.358

Locus 16771 :

2 alleles

99.2% missing data

4	(0.543) 0.546 0.547

2	(0.457) 0.454 0.453

Locus 16772 :

2 alleles

97.3% missing data

4	(0.814) 0.804 0.826

1	(0.186) 0.196 0.174

Locus 16773 :

2 alleles

13.3% missing data

2	(0.987) 0.989 0.992

4	(0.013) 0.011 0.008

Locus 16774 :

2 alleles

99.2% missing data

3	(0.637) 0.643 0.635

4	(0.363) 0.357 0.365

Locus 16775 :

2 alleles

99.2% missing data

3	(0.479) 0.478 0.476

2	(0.521) 0.522 0.524

Locus 16776 :

2 alleles

99.2% missing data

2	(0.314) 0.321 0.319

3	(0.686) 0.679 0.681

Locus 16777 :

2 alleles

99.2% missing data

1	(0.538) 0.529 0.542

4	(0.462) 0.471 0.458

Locus 16778 :

2 alleles

81.2% missing data

2	(0.971) 0.969 0.980

3	(0.029) 0.031 0.020

Locus 16779 :

2 alleles

99.2% missing data

4	(0.789) 0.790 0.783

1	(0.211) 0.210 0.217

Locus 16780 :

2 alleles

99.2% missing data

1	(0.534) 0.532 0.531

4	(0.466) 0.468 0.469

Locus 16781 :

2 alleles

97.7% missing data

2	(0.298) 0.294 0.289

4	(0.702) 0.706 0.711

Locus 16782 :

2 alleles

99.2% missing data

2	(0.497) 0.498 0.493

4	(0.503) 0.502 0.507

Locus 16783 :

2 alleles

93.8% missing data

4	(0.935) 0.937 0.938

3	(0.065) 0.063 0.062

Locus 16784 :

2 alleles

99.2% missing data

3	(0.484) 0.485 0.478

4	(0.516) 0.515 0.522

Locus 16785 :

2 alleles

96.5% missing data

3	(0.157) 0.149 0.162

4	(0.843) 0.851 0.838

Locus 16786 :

2 alleles

99.2% missing data

3	(0.285) 0.288 0.277

1	(0.715) 0.712 0.723

Locus 16787 :

2 alleles

99.2% missing data

4	(0.322) 0.320 0.327

1	(0.678) 0.680 0.673

Locus 16788 :

2 alleles

96.1% missing data

1	(0.880) 0.885 0.880

2	(0.120) 0.115 0.120

Locus 16789 :

2 alleles

97.7% missing data

3	(0.686) 0.679 0.707

4	(0.314) 0.321 0.293

Locus 16790 :

2 alleles

99.2% missing data

2	(0.448) 0.444 0.458

4	(0.552) 0.556 0.542

Locus 16791 :

2 alleles

96.1% missing data

4	(0.756) 0.763 0.758

3	(0.244) 0.237 0.242

Locus 16792 :

2 alleles

99.2% missing data

3	(0.267) 0.265 0.268

4	(0.733) 0.735 0.732

Locus 16793 :

2 alleles

98.8% missing data

4	(0.242) 0.238 0.248

3	(0.758) 0.762 0.752

Locus 16794 :

2 alleles

99.2% missing data

1	(0.402) 0.406 0.399

3	(0.598) 0.594 0.601

Locus 16795 :

2 alleles

99.2% missing data

4	(0.585) 0.577 0.589

1	(0.415) 0.423 0.411

Locus 16796 :

2 alleles

99.2% missing data

1	(0.723) 0.724 0.728

3	(0.277) 0.276 0.272

Locus 16797 :

3 alleles

98.4% missing data

3	(0.501) 0.498 0.508

2	(0.367) 0.363 0.365

1	(0.132) 0.139 0.127

Locus 16798 :

2 alleles

98.8% missing data

3	(0.759) 0.759 0.762

1	(0.241) 0.241 0.238

Locus 16799 :

2 alleles

95.3% missing data

4	(0.079) 0.078 0.081

1	(0.921) 0.922 0.919

Locus 16800 :

2 alleles

99.2% missing data

1	(0.585) 0.579 0.580

3	(0.415) 0.421 0.420

Locus 16801 :

2 alleles

98.4% missing data

1	(0.817) 0.812 0.817

4	(0.183) 0.188 0.183

Locus 16802 :

2 alleles

99.2% missing data

2	(0.528) 0.531 0.526

4	(0.472) 0.469 0.474

Locus 16803 :

2 alleles

99.2% missing data

2	(0.586) 0.584 0.583

3	(0.414) 0.416 0.417

Locus 16804 :

2 alleles

99.2% missing data

1	(0.445) 0.440 0.446

3	(0.555) 0.560 0.554

Locus 16805 :

2 alleles

99.2% missing data

1	(0.305) 0.304 0.306

3	(0.695) 0.696 0.694

Locus 16806 :

2 alleles

98.8% missing data

3	(0.283) 0.281 0.281

2	(0.717) 0.719 0.719

Locus 16807 :

2 alleles

99.2% missing data

3	(0.610) 0.610 0.616

2	(0.390) 0.390 0.384

Locus 16808 :

2 alleles

90.2% missing data

1	(0.819) 0.800 0.846

2	(0.181) 0.200 0.154

Locus 16809 :

2 alleles

99.2% missing data

1	(0.488) 0.491 0.485

2	(0.512) 0.509 0.515

Locus 16810 :

2 alleles

99.2% missing data

2	(0.399) 0.400 0.403

1	(0.601) 0.600 0.597

Locus 16811 :

2 alleles

99.2% missing data

2	(0.673) 0.668 0.672

1	(0.327) 0.332 0.328

Locus 16812 :

2 alleles

99.2% missing data

1	(0.705) 0.696 0.713

4	(0.295) 0.304 0.287

Locus 16813 :

2 alleles

98.8% missing data

1	(0.258) 0.251 0.271

4	(0.742) 0.749 0.729

Locus 16814 :

2 alleles

99.2% missing data

3	(0.163) 0.157 0.169

4	(0.837) 0.843 0.831

Locus 16815 :

2 alleles

99.2% missing data

3	(0.536) 0.535 0.537

4	(0.464) 0.465 0.463

Locus 16816 :

2 alleles

99.2% missing data

4	(0.487) 0.485 0.487

1	(0.513) 0.515 0.513

Locus 16817 :

2 alleles

99.2% missing data

1	(0.724) 0.720 0.730

4	(0.276) 0.280 0.270

Locus 16818 :

2 alleles

99.2% missing data

4	(0.362) 0.362 0.364

3	(0.638) 0.638 0.636

Locus 16819 :

2 alleles

99.2% missing data

2	(0.290) 0.285 0.291

3	(0.710) 0.715 0.709

Locus 16820 :

2 alleles

98.8% missing data

1	(0.304) 0.302 0.304

4	(0.696) 0.698 0.696

Locus 16821 :

2 alleles

98.8% missing data

3	(0.548) 0.544 0.553

1	(0.452) 0.456 0.447

Locus 16822 :

2 alleles

91.8% missing data

2	(0.202) 0.223 0.182

1	(0.798) 0.777 0.818

Locus 16823 :

2 alleles

99.2% missing data

2	(0.227) 0.230 0.224

3	(0.773) 0.770 0.776

Locus 16824 :

2 alleles

99.2% missing data

2	(0.676) 0.675 0.678

1	(0.324) 0.325 0.322

Locus 16825 :

2 alleles

96.1% missing data

1	(0.305) 0.311 0.297

3	(0.695) 0.689 0.703

Locus 16826 :

2 alleles

99.2% missing data

2	(0.393) 0.396 0.391

4	(0.607) 0.604 0.609

Locus 16827 :

2 alleles

99.2% missing data

3	(0.617) 0.625 0.615

2	(0.383) 0.375 0.385

Locus 16828 :

2 alleles

99.2% missing data

1	(0.436) 0.437 0.438

4	(0.564) 0.563 0.562

Locus 16829 :

2 alleles

99.2% missing data

4	(0.748) 0.745 0.743

2	(0.252) 0.255 0.257

Locus 16830 :

2 alleles

99.2% missing data

3	(0.362) 0.367 0.356

2	(0.638) 0.633 0.644

Locus 16831 :

2 alleles

99.2% missing data

2	(0.464) 0.453 0.465

3	(0.536) 0.547 0.535

Locus 16832 :

2 alleles

99.2% missing data

4	(0.633) 0.633 0.634

1	(0.367) 0.367 0.366

Locus 16833 :

2 alleles

98.8% missing data

1	(0.318) 0.308 0.329

4	(0.682) 0.692 0.671

Locus 16834 :

2 alleles

99.2% missing data

3	(0.507) 0.500 0.512

1	(0.493) 0.500 0.488

Locus 16835 :

2 alleles

96.1% missing data

4	(0.811) 0.824 0.808

1	(0.189) 0.176 0.192

Locus 16836 :

2 alleles

99.2% missing data

1	(0.534) 0.532 0.532

3	(0.466) 0.468 0.468

Locus 16837 :

2 alleles

93.4% missing data

1	(0.903) 0.893 0.916

3	(0.097) 0.107 0.084

Locus 16838 :

2 alleles

99.2% missing data

3	(0.495) 0.488 0.502

1	(0.505) 0.512 0.498

Locus 16839 :

2 alleles

99.2% missing data

1	(0.442) 0.446 0.435

4	(0.558) 0.554 0.565

Locus 16840 :

3 alleles

97.7% missing data

3	(0.667) 0.660 0.675

2	(0.149) 0.151 0.144

1	(0.185) 0.189 0.180

Locus 16841 :

2 alleles

97.7% missing data

4	(0.142) 0.140 0.146

2	(0.858) 0.860 0.854

Locus 16842 :

2 alleles

99.2% missing data

3	(0.272) 0.276 0.271

1	(0.728) 0.724 0.729

Locus 16843 :

2 alleles

99.2% missing data

3	(0.673) 0.676 0.671

2	(0.327) 0.324 0.329

Locus 16844 :

2 alleles

98.8% missing data

3	(0.552) 0.543 0.559

4	(0.448) 0.457 0.441

Locus 16845 :

2 alleles

99.2% missing data

2	(0.680) 0.672 0.690

4	(0.320) 0.328 0.310

Locus 16846 :

2 alleles

99.2% missing data

3	(0.543) 0.540 0.544

1	(0.457) 0.460 0.456

Locus 16847 :

2 alleles

99.2% missing data

4	(0.300) 0.299 0.312

3	(0.700) 0.701 0.688

Locus 16848 :

2 alleles

99.2% missing data

3	(0.326) 0.328 0.329

4	(0.674) 0.672 0.671

Locus 16849 :

2 alleles

99.2% missing data

4	(0.433) 0.436 0.426

1	(0.567) 0.564 0.574

Locus 16850 :

2 alleles

99.2% missing data

3	(0.712) 0.715 0.713

1	(0.288) 0.285 0.287

Locus 16851 :

2 alleles

99.2% missing data

3	(0.649) 0.651 0.642

1	(0.351) 0.349 0.358

Locus 16852 :

2 alleles

99.2% missing data

1	(0.291) 0.289 0.293

3	(0.709) 0.711 0.707

Locus 16853 :

2 alleles

99.2% missing data

1	(0.413) 0.416 0.412

2	(0.587) 0.584 0.588

Locus 16854 :

2 alleles

96.1% missing data

1	(0.734) 0.749 0.736

3	(0.266) 0.251 0.264

Locus 16855 :

2 alleles

99.2% missing data

2	(0.396) 0.400 0.393

3	(0.604) 0.600 0.607

Locus 16856 :

2 alleles

99.2% missing data

1	(0.314) 0.316 0.308

3	(0.686) 0.684 0.692

Locus 16857 :

2 alleles

97.7% missing data

2	(0.328) 0.329 0.317

4	(0.672) 0.671 0.683

Locus 16858 :

2 alleles

99.2% missing data

2	(0.403) 0.400 0.404

1	(0.597) 0.600 0.596

Locus 16859 :

2 alleles

0.0% missing data

2	(0.915) 0.765 0.974

3	(0.085) 0.235 0.026

Locus 16860 :

2 alleles

99.2% missing data

2	(0.383) 0.379 0.381

4	(0.617) 0.621 0.619

Locus 16861 :

2 alleles

97.7% missing data

1	(0.728) 0.720 0.743

4	(0.272) 0.280 0.257

Locus 16862 :

2 alleles

99.2% missing data

2	(0.502) 0.503 0.504

3	(0.498) 0.497 0.496

Locus 16863 :

2 alleles

99.2% missing data

2	(0.728) 0.734 0.727

3	(0.272) 0.266 0.273

Locus 16864 :

2 alleles

99.2% missing data

2	(0.488) 0.493 0.485

3	(0.512) 0.507 0.515

Locus 16865 :

2 alleles

99.2% missing data

2	(0.347) 0.350 0.344

4	(0.653) 0.650 0.656

Locus 16866 :

2 alleles

99.2% missing data

2	(0.552) 0.552 0.554

1	(0.448) 0.448 0.446

Locus 16867 :

2 alleles

99.2% missing data

1	(0.584) 0.582 0.580

4	(0.416) 0.418 0.420

Locus 16868 :

2 alleles

99.2% missing data

3	(0.438) 0.439 0.438

4	(0.562) 0.561 0.562

Locus 16869 :

3 alleles

98.8% missing data

3	(0.232) 0.227 0.235

2	(0.266) 0.270 0.266

4	(0.502) 0.502 0.499

Locus 16870 :

2 alleles

98.8% missing data

1	(0.630) 0.625 0.636

2	(0.370) 0.375 0.364

Locus 16871 :

2 alleles

98.8% missing data

2	(0.527) 0.532 0.524

3	(0.473) 0.468 0.476

Locus 16872 :

2 alleles

98.4% missing data

2	(0.557) 0.562 0.558

3	(0.443) 0.438 0.442

Locus 16873 :

2 alleles

99.2% missing data

3	(0.435) 0.432 0.436

4	(0.565) 0.568 0.564

Locus 16874 :

2 alleles

98.4% missing data

1	(0.720) 0.727 0.710

2	(0.280) 0.273 0.290

Locus 16875 :

2 alleles

99.2% missing data

2	(0.324) 0.322 0.327

3	(0.676) 0.678 0.673

Locus 16876 :

2 alleles

97.7% missing data

4	(0.234) 0.234 0.230

1	(0.766) 0.766 0.770

Locus 16877 :

2 alleles

98.4% missing data

1	(0.602) 0.591 0.606

4	(0.398) 0.409 0.394

Locus 16878 :

2 alleles

99.2% missing data

1	(0.393) 0.387 0.397

4	(0.607) 0.613 0.603

Locus 16879 :

2 alleles

99.2% missing data

1	(0.439) 0.433 0.444

4	(0.561) 0.567 0.556

Locus 16880 :

2 alleles

99.2% missing data

4	(0.244) 0.244 0.248

2	(0.756) 0.756 0.752

Locus 16881 :

2 alleles

77.0% missing data

4	(0.959) 0.960 0.970

1	(0.041) 0.040 0.030

Locus 16882 :

2 alleles

94.1% missing data

2	(0.856) 0.845 0.875

1	(0.144) 0.155 0.125

Locus 16883 :

2 alleles

99.2% missing data

1	(0.787) 0.783 0.792

2	(0.213) 0.217 0.208

Locus 16884 :

2 alleles

96.9% missing data

3	(0.163) 0.158 0.165

1	(0.837) 0.842 0.835

Locus 16885 :

2 alleles

99.2% missing data

1	(0.682) 0.681 0.677

4	(0.318) 0.319 0.323

Locus 16886 :

2 alleles

99.2% missing data

3	(0.287) 0.279 0.289

1	(0.713) 0.721 0.711

Locus 16887 :

2 alleles

99.2% missing data

4	(0.721) 0.721 0.718

1	(0.279) 0.279 0.282

Locus 16888 :

2 alleles

99.2% missing data

2	(0.605) 0.606 0.597

3	(0.395) 0.394 0.403

Locus 16889 :

2 alleles

99.2% missing data

4	(0.327) 0.327 0.331

1	(0.673) 0.673 0.669

Locus 16890 :

2 alleles

98.0% missing data

2	(0.797) 0.801 0.788

3	(0.203) 0.199 0.212

Locus 16891 :

2 alleles

99.2% missing data

3	(0.383) 0.391 0.377

2	(0.617) 0.609 0.623

Locus 16892 :

2 alleles

98.8% missing data

4	(0.745) 0.752 0.738

3	(0.255) 0.248 0.262

Locus 16893 :

2 alleles

99.2% missing data

1	(0.598) 0.593 0.602

3	(0.402) 0.407 0.398

Locus 16894 :

2 alleles

99.2% missing data

4	(0.311) 0.308 0.310

1	(0.689) 0.692 0.690

Locus 16895 :

2 alleles

1.2% missing data

2	(0.987) 0.983 0.996

1	(0.013) 0.017 0.004

Locus 16896 :

2 alleles

99.2% missing data

1	(0.382) 0.382 0.385

2	(0.618) 0.618 0.615

Locus 16897 :

2 alleles

99.2% missing data

4	(0.618) 0.611 0.617

1	(0.382) 0.389 0.383

Locus 16898 :

2 alleles

99.2% missing data

3	(0.560) 0.563 0.558

2	(0.440) 0.437 0.442

Locus 16899 :

2 alleles

98.8% missing data

3	(0.373) 0.374 0.371

1	(0.627) 0.626 0.629

Locus 16900 :

2 alleles

96.9% missing data

3	(0.809) 0.814 0.810

2	(0.191) 0.186 0.190

Locus 16901 :

2 alleles

99.2% missing data

3	(0.260) 0.263 0.261

2	(0.740) 0.737 0.739

Locus 16902 :

2 alleles

99.2% missing data

2	(0.485) 0.479 0.487

4	(0.515) 0.521 0.513

Locus 16903 :

2 alleles

96.5% missing data

1	(0.758) 0.770 0.749

3	(0.242) 0.230 0.251

Locus 16904 :

2 alleles

92.6% missing data

4	(0.939) 0.939 0.940

1	(0.061) 0.061 0.060

Locus 16905 :

2 alleles

9.0% missing data

1	(0.986) 0.979 0.996

4	(0.014) 0.021 0.004

Locus 16906 :

2 alleles

99.2% missing data

1	(0.227) 0.230 0.222

4	(0.773) 0.770 0.778

Locus 16907 :

2 alleles

99.2% missing data

1	(0.367) 0.369 0.371

2	(0.633) 0.631 0.629

Locus 16908 :

2 alleles

99.2% missing data

3	(0.380) 0.385 0.380

4	(0.620) 0.615 0.620

Locus 16909 :

2 alleles

99.2% missing data

4	(0.522) 0.520 0.525

3	(0.478) 0.480 0.475

Locus 16910 :

2 alleles

99.2% missing data

1	(0.681) 0.671 0.686

3	(0.319) 0.329 0.314

Locus 16911 :

2 alleles

99.2% missing data

1	(0.625) 0.627 0.631

4	(0.375) 0.373 0.369

Locus 16912 :

2 alleles

99.2% missing data

4	(0.431) 0.421 0.435

3	(0.569) 0.579 0.565

Locus 16913 :

2 alleles

98.4% missing data

2	(0.626) 0.627 0.635

3	(0.374) 0.373 0.365

Locus 16914 :

2 alleles

99.2% missing data

3	(0.668) 0.674 0.664

2	(0.332) 0.326 0.336

Locus 16915 :

2 alleles

0.4% missing data

2	(0.984) 0.980 0.995

3	(0.016) 0.020 0.005

Locus 16916 :

3 alleles

97.3% missing data

2	(0.165) 0.166 0.163

4	(0.600) 0.607 0.605

1	(0.235) 0.228 0.233

Locus 16917 :

2 alleles

99.2% missing data

4	(0.688) 0.681 0.691

2	(0.312) 0.319 0.309

Locus 16918 :

2 alleles

99.2% missing data

2	(0.752) 0.757 0.754

3	(0.248) 0.243 0.246

Locus 16919 :

2 alleles

99.2% missing data

4	(0.347) 0.340 0.353

1	(0.653) 0.660 0.647

Locus 16920 :

2 alleles

99.2% missing data

3	(0.348) 0.342 0.355

2	(0.652) 0.658 0.645

Locus 16921 :

3 alleles

95.7% missing data

2	(0.652) 0.674 0.640

3	(0.173) 0.162 0.177

1	(0.174) 0.164 0.183

Locus 16922 :

2 alleles

99.2% missing data

3	(0.736) 0.730 0.733

4	(0.264) 0.270 0.267

Locus 16923 :

2 alleles

99.2% missing data

4	(0.365) 0.368 0.367

3	(0.635) 0.632 0.633

Locus 16924 :

2 alleles

7.8% missing data

3	(0.978) 0.958 0.993

2	(0.022) 0.042 0.007

Locus 16925 :

3 alleles

98.8% missing data

1	(0.578) 0.573 0.582

3	(0.155) 0.155 0.157

2	(0.268) 0.272 0.261

Locus 16926 :

2 alleles

99.2% missing data

4	(0.293) 0.298 0.292

3	(0.707) 0.702 0.708

Locus 16927 :

2 alleles

98.0% missing data

3	(0.741) 0.742 0.741

2	(0.259) 0.258 0.259

Locus 16928 :

2 alleles

99.2% missing data

3	(0.414) 0.413 0.420

1	(0.586) 0.587 0.580

Locus 16929 :

2 alleles

99.2% missing data

2	(0.532) 0.534 0.531

1	(0.468) 0.466 0.469

Locus 16930 :

2 alleles

99.2% missing data

2	(0.453) 0.454 0.455

3	(0.547) 0.546 0.545

Locus 16931 :

2 alleles

99.2% missing data

1	(0.373) 0.379 0.367

2	(0.627) 0.621 0.633

Locus 16932 :

2 alleles

99.2% missing data

3	(0.299) 0.307 0.297

2	(0.701) 0.693 0.703

Locus 16933 :

2 alleles

99.2% missing data

4	(0.595) 0.591 0.602

3	(0.405) 0.409 0.398

Locus 16934 :

2 alleles

95.3% missing data

3	(0.853) 0.842 0.872

4	(0.147) 0.158 0.128

Locus 16935 :

3 alleles

98.4% missing data

1	(0.366) 0.355 0.375

3	(0.229) 0.228 0.226

2	(0.405) 0.417 0.399

Locus 16936 :

2 alleles

99.2% missing data

2	(0.701) 0.693 0.703

3	(0.299) 0.307 0.297

Locus 16937 :

2 alleles

98.4% missing data

1	(0.669) 0.671 0.670

3	(0.331) 0.329 0.330

Locus 16938 :

2 alleles

97.7% missing data

1	(0.662) 0.676 0.656

4	(0.338) 0.324 0.344

Locus 16939 :

2 alleles

99.2% missing data

2	(0.234) 0.241 0.236

4	(0.766) 0.759 0.764

Locus 16940 :

2 alleles

99.2% missing data

3	(0.543) 0.543 0.546

4	(0.457) 0.457 0.454

Locus 16941 :

2 alleles

99.2% missing data

3	(0.720) 0.713 0.730

2	(0.280) 0.287 0.270

Locus 16942 :

2 alleles

0.0% missing data

1	(0.990) 0.985 0.997

4	(0.010) 0.015 0.003

Locus 16943 :

2 alleles

99.2% missing data

3	(0.482) 0.479 0.496

1	(0.518) 0.521 0.504

Locus 16944 :

2 alleles

98.0% missing data

3	(0.799) 0.796 0.803

4	(0.201) 0.204 0.197

Locus 16945 :

2 alleles

99.2% missing data

3	(0.586) 0.586 0.588

2	(0.414) 0.414 0.412

Locus 16946 :

2 alleles

99.2% missing data

4	(0.493) 0.495 0.495

2	(0.507) 0.505 0.505

Locus 16947 :

2 alleles

99.2% missing data

2	(0.678) 0.675 0.683

1	(0.322) 0.325 0.317

Locus 16948 :

2 alleles

97.7% missing data

2	(0.847) 0.841 0.854

1	(0.153) 0.159 0.146

Locus 16949 :

2 alleles

0.0% missing data

4	(0.982) 0.987 0.989

1	(0.018) 0.013 0.011

Locus 16950 :

2 alleles

99.2% missing data

3	(0.267) 0.269 0.274

2	(0.733) 0.731 0.726

Locus 16951 :

2 alleles

99.2% missing data

4	(0.397) 0.391 0.402

3	(0.603) 0.609 0.598

Locus 16952 :

2 alleles

99.2% missing data

4	(0.679) 0.675 0.683

1	(0.321) 0.325 0.317

Locus 16953 :

2 alleles

98.8% missing data

3	(0.449) 0.451 0.442

1	(0.551) 0.549 0.558

Locus 16954 :

2 alleles

8.2% missing data

3	(0.984) 0.975 0.996

4	(0.016) 0.025 0.004

Locus 16955 :

2 alleles

99.2% missing data

2	(0.652) 0.655 0.649

4	(0.348) 0.345 0.351

Locus 16956 :

2 alleles

98.8% missing data

4	(0.738) 0.743 0.735

3	(0.262) 0.257 0.265

Locus 16957 :

2 alleles

99.2% missing data

2	(0.568) 0.567 0.569

4	(0.432) 0.433 0.431

Locus 16958 :

2 alleles

99.2% missing data

4	(0.540) 0.540 0.541

2	(0.460) 0.460 0.459

Locus 16959 :

2 alleles

95.7% missing data

3	(0.781) 0.798 0.764

4	(0.219) 0.202 0.236

Locus 16960 :

2 alleles

96.1% missing data

3	(0.710) 0.725 0.709

4	(0.290) 0.275 0.291

Locus 16961 :

2 alleles

99.2% missing data

2	(0.540) 0.535 0.543

1	(0.460) 0.465 0.457

Locus 16962 :

2 alleles

97.7% missing data

4	(0.522) 0.516 0.539

3	(0.478) 0.484 0.461

Locus 16963 :

2 alleles

99.2% missing data

1	(0.644) 0.638 0.650

2	(0.356) 0.362 0.350

Locus 16964 :

2 alleles

99.2% missing data

3	(0.417) 0.421 0.412

4	(0.583) 0.579 0.588

Locus 16965 :

2 alleles

99.2% missing data

4	(0.724) 0.729 0.718

2	(0.276) 0.271 0.282

Locus 16966 :

2 alleles

99.2% missing data

3	(0.596) 0.597 0.590

4	(0.404) 0.403 0.410

Locus 16967 :

2 alleles

99.2% missing data

2	(0.628) 0.621 0.636

3	(0.372) 0.379 0.364

Locus 16968 :

2 alleles

99.2% missing data

3	(0.368) 0.363 0.373

2	(0.632) 0.637 0.627

Locus 16969 :

2 alleles

95.3% missing data

1	(0.895) 0.890 0.907

2	(0.105) 0.110 0.093

Locus 16970 :

2 alleles

99.2% missing data

1	(0.554) 0.549 0.553

2	(0.446) 0.451 0.447

Locus 16971 :

2 alleles

98.8% missing data

3	(0.357) 0.358 0.355

2	(0.643) 0.642 0.645

Locus 16972 :

2 alleles

99.2% missing data

3	(0.475) 0.472 0.481

4	(0.525) 0.528 0.519

Locus 16973 :

2 alleles

99.2% missing data

1	(0.467) 0.466 0.466

4	(0.533) 0.534 0.534

Locus 16974 :

2 alleles

99.2% missing data

4	(0.285) 0.288 0.285

1	(0.715) 0.712 0.715

Locus 16975 :

3 alleles

98.8% missing data

3	(0.319) 0.318 0.327

1	(0.443) 0.445 0.437

4	(0.238) 0.238 0.236

Locus 16976 :

2 alleles

99.2% missing data

1	(0.471) 0.463 0.479

4	(0.529) 0.537 0.521

Locus 16977 :

2 alleles

98.4% missing data

3	(0.529) 0.537 0.520

1	(0.471) 0.463 0.480

Locus 16978 :

2 alleles

99.2% missing data

3	(0.506) 0.507 0.505

2	(0.494) 0.493 0.495

Locus 16979 :

2 alleles

99.2% missing data

2	(0.375) 0.375 0.377

4	(0.625) 0.625 0.623

Locus 16980 :

2 alleles

0.4% missing data

4	(0.987) 0.981 0.996

1	(0.013) 0.019 0.004

Locus 16981 :

2 alleles

99.2% missing data

3	(0.366) 0.359 0.368

2	(0.634) 0.641 0.632

Locus 16982 :

2 alleles

99.2% missing data

3	(0.636) 0.629 0.638

1	(0.364) 0.371 0.362

Locus 16983 :

2 alleles

99.2% missing data

2	(0.450) 0.460 0.452

1	(0.550) 0.540 0.548

Locus 16984 :

2 alleles

97.3% missing data

2	(0.778) 0.762 0.792

3	(0.222) 0.238 0.208

Locus 16985 :

2 alleles

99.2% missing data

4	(0.314) 0.314 0.325

1	(0.686) 0.686 0.675

Locus 16986 :

2 alleles

99.2% missing data

3	(0.604) 0.606 0.600

4	(0.396) 0.394 0.400

Locus 16987 :

2 alleles

97.7% missing data

3	(0.538) 0.527 0.542

1	(0.462) 0.473 0.458

Locus 16988 :

2 alleles

99.2% missing data

3	(0.321) 0.322 0.324

1	(0.679) 0.678 0.676

Locus 16989 :

3 alleles

98.8% missing data

3	(0.386) 0.377 0.393

4	(0.307) 0.313 0.303

2	(0.307) 0.310 0.304

Locus 16990 :

2 alleles

99.2% missing data

3	(0.629) 0.627 0.628

2	(0.371) 0.373 0.372

Locus 16991 :

2 alleles

99.2% missing data

4	(0.417) 0.417 0.422

1	(0.583) 0.583 0.578

Locus 16992 :

2 alleles

99.2% missing data

4	(0.459) 0.449 0.464

3	(0.541) 0.551 0.536

Locus 16993 :

2 alleles

98.4% missing data

1	(0.712) 0.709 0.717

2	(0.288) 0.291 0.283

Locus 16994 :

2 alleles

99.2% missing data

1	(0.531) 0.527 0.530

3	(0.469) 0.473 0.470

Locus 16995 :

2 alleles

98.0% missing data

2	(0.170) 0.169 0.169

3	(0.830) 0.831 0.831

Locus 16996 :

2 alleles

99.2% missing data

3	(0.458) 0.454 0.467

2	(0.542) 0.546 0.533

Locus 16997 :

2 alleles

99.2% missing data

1	(0.634) 0.645 0.632

2	(0.366) 0.355 0.368

Locus 16998 :

2 alleles

99.2% missing data

3	(0.580) 0.578 0.582

2	(0.420) 0.422 0.418

Locus 16999 :

2 alleles

99.2% missing data

2	(0.473) 0.476 0.466

3	(0.527) 0.524 0.534

Locus 17000 :

2 alleles

99.2% missing data

2	(0.543) 0.538 0.542

4	(0.457) 0.462 0.458

Locus 17001 :

2 alleles

99.2% missing data

4	(0.398) 0.401 0.399

3	(0.602) 0.599 0.601

Locus 17002 :

2 alleles

99.2% missing data

1	(0.490) 0.492 0.494

3	(0.510) 0.508 0.506

Locus 17003 :

2 alleles

99.2% missing data

4	(0.457) 0.463 0.451

3	(0.543) 0.537 0.549

Locus 17004 :

2 alleles

98.8% missing data

1	(0.500) 0.499 0.493

4	(0.500) 0.501 0.507

Locus 17005 :

2 alleles

99.2% missing data

3	(0.365) 0.374 0.359

1	(0.635) 0.626 0.641

Locus 17006 :

2 alleles

99.2% missing data

2	(0.666) 0.666 0.667

3	(0.334) 0.334 0.333

Locus 17007 :

2 alleles

99.2% missing data

1	(0.419) 0.416 0.420

3	(0.581) 0.584 0.580

Locus 17008 :

2 alleles

99.2% missing data

2	(0.337) 0.331 0.345

1	(0.663) 0.669 0.655

Locus 17009 :

2 alleles

99.2% missing data

3	(0.817) 0.810 0.826

4	(0.183) 0.190 0.174

Locus 17010 :

2 alleles

99.2% missing data

3	(0.730) 0.728 0.728

4	(0.270) 0.272 0.272

Locus 17011 :

2 alleles

99.2% missing data

1	(0.586) 0.583 0.590

3	(0.414) 0.417 0.410

Locus 17012 :

2 alleles

96.5% missing data

1	(0.859) 0.863 0.860

2	(0.141) 0.137 0.140

Locus 17013 :

2 alleles

99.2% missing data

2	(0.544) 0.546 0.538

1	(0.456) 0.454 0.462

Locus 17014 :

2 alleles

96.5% missing data

3	(0.758) 0.754 0.772

1	(0.242) 0.246 0.228

Locus 17015 :

2 alleles

99.2% missing data

1	(0.717) 0.722 0.709

4	(0.283) 0.278 0.291

Locus 17016 :

2 alleles

96.5% missing data

1	(0.792) 0.802 0.790

3	(0.208) 0.198 0.210

Locus 17017 :

2 alleles

92.6% missing data

3	(0.898) 0.897 0.907

4	(0.102) 0.103 0.093

Locus 17018 :

2 alleles

99.2% missing data

4	(0.589) 0.582 0.591

2	(0.411) 0.418 0.409

Locus 17019 :

2 alleles

91.0% missing data

4	(0.949) 0.957 0.946

1	(0.051) 0.043 0.054

Locus 17020 :

2 alleles

99.2% missing data

2	(0.385) 0.390 0.380

1	(0.615) 0.610 0.620

Locus 17021 :

2 alleles

99.2% missing data

4	(0.716) 0.719 0.705

2	(0.284) 0.281 0.295

Locus 17022 :

2 alleles

91.0% missing data

4	(0.911) 0.919 0.911

1	(0.089) 0.081 0.089

Locus 17023 :

2 alleles

99.2% missing data

4	(0.142) 0.139 0.149

1	(0.858) 0.861 0.851

Locus 17024 :

2 alleles

88.7% missing data

1	(0.884) 0.908 0.862

3	(0.116) 0.092 0.138

Locus 17025 :

2 alleles

99.2% missing data

2	(0.262) 0.263 0.263

3	(0.738) 0.737 0.737

Locus 17026 :

3 alleles

98.8% missing data

4	(0.302) 0.301 0.310

1	(0.137) 0.137 0.145

2	(0.561) 0.562 0.545

Locus 17027 :

2 alleles

98.4% missing data

1	(0.562) 0.560 0.571

2	(0.438) 0.440 0.429

Locus 17028 :

2 alleles

99.2% missing data

3	(0.549) 0.547 0.545

2	(0.451) 0.453 0.455

Locus 17029 :

2 alleles

99.2% missing data

1	(0.419) 0.413 0.430

2	(0.581) 0.587 0.570

Locus 17030 :

2 alleles

93.0% missing data

4	(0.916) 0.917 0.921

2	(0.084) 0.083 0.079

Locus 17031 :

2 alleles

97.7% missing data

1	(0.634) 0.631 0.645

2	(0.366) 0.369 0.355

Locus 17032 :

2 alleles

99.2% missing data

2	(0.363) 0.364 0.356

4	(0.637) 0.636 0.644

Locus 17033 :

2 alleles

99.2% missing data

1	(0.415) 0.412 0.414

4	(0.585) 0.588 0.586

Locus 17034 :

2 alleles

96.1% missing data

1	(0.810) 0.822 0.808

4	(0.190) 0.178 0.192

Locus 17035 :

2 alleles

99.2% missing data

3	(0.412) 0.413 0.417

4	(0.588) 0.587 0.583

Locus 17036 :

2 alleles

91.0% missing data

1	(0.895) 0.907 0.894

4	(0.105) 0.093 0.106

Locus 17037 :

3 alleles

98.4% missing data

1	(0.396) 0.392 0.402

3	(0.325) 0.323 0.328

4	(0.279) 0.285 0.270

Locus 17038 :

2 alleles

99.2% missing data

1	(0.354) 0.350 0.354

2	(0.646) 0.650 0.646

Locus 17039 :

2 alleles

99.2% missing data

2	(0.225) 0.225 0.225

1	(0.775) 0.775 0.775

Locus 17040 :

2 alleles

99.2% missing data

3	(0.630) 0.629 0.626

1	(0.370) 0.371 0.374

Locus 17041 :

2 alleles

97.3% missing data

2	(0.781) 0.782 0.784

4	(0.219) 0.218 0.216

Locus 17042 :

3 alleles

98.8% missing data

3	(0.312) 0.317 0.310

4	(0.270) 0.268 0.271

1	(0.418) 0.415 0.419

Locus 17043 :

2 alleles

99.2% missing data

2	(0.713) 0.709 0.712

4	(0.287) 0.291 0.288

Locus 17044 :

2 alleles

74.6% missing data

1	(0.602) 0.616 0.587

4	(0.398) 0.384 0.413

Locus 17045 :

2 alleles

98.8% missing data

1	(0.503) 0.507 0.501

4	(0.497) 0.493 0.499

Locus 17046 :

2 alleles

99.2% missing data

3	(0.226) 0.217 0.228

4	(0.774) 0.783 0.772

Locus 17047 :

2 alleles

94.5% missing data

3	(0.858) 0.858 0.870

1	(0.142) 0.142 0.130

Locus 17048 :

2 alleles

99.2% missing data

2	(0.645) 0.641 0.650

3	(0.355) 0.359 0.350

Locus 17049 :

2 alleles

98.8% missing data

4	(0.394) 0.393 0.389

2	(0.606) 0.607 0.611

Locus 17050 :

2 alleles

98.8% missing data

1	(0.571) 0.578 0.564

4	(0.429) 0.422 0.436

Locus 17051 :

2 alleles

98.4% missing data

1	(0.574) 0.566 0.575

3	(0.426) 0.434 0.425

Locus 17052 :

2 alleles

99.2% missing data

1	(0.507) 0.507 0.507

2	(0.493) 0.493 0.493

Locus 17053 :

2 alleles

95.7% missing data

2	(0.517) 0.512 0.526

1	(0.483) 0.488 0.474

Locus 17054 :

2 alleles

98.8% missing data

4	(0.715) 0.712 0.723

1	(0.285) 0.288 0.277

Locus 17055 :

2 alleles

99.2% missing data

4	(0.558) 0.557 0.559

1	(0.442) 0.443 0.441

Locus 17056 :

2 alleles

98.4% missing data

2	(0.698) 0.701 0.699

3	(0.302) 0.299 0.301

Locus 17057 :

2 alleles

82.0% missing data

1	(0.408) 0.483 0.337

3	(0.592) 0.517 0.663

Locus 17058 :

2 alleles

99.2% missing data

3	(0.340) 0.341 0.337

1	(0.660) 0.659 0.663

Locus 17059 :

2 alleles

99.2% missing data

3	(0.554) 0.554 0.553

2	(0.446) 0.446 0.447

Locus 17060 :

2 alleles

99.2% missing data

1	(0.469) 0.468 0.469

2	(0.531) 0.532 0.531

Locus 17061 :

2 alleles

99.2% missing data

3	(0.730) 0.731 0.729

2	(0.270) 0.269 0.271

Locus 17062 :

2 alleles

98.8% missing data

3	(0.517) 0.516 0.524

4	(0.483) 0.484 0.476

Locus 17063 :

2 alleles

98.4% missing data

1	(0.761) 0.760 0.758

3	(0.239) 0.240 0.242

Locus 17064 :

2 alleles

99.2% missing data

2	(0.623) 0.629 0.616

4	(0.377) 0.371 0.384

Locus 17065 :

2 alleles

99.2% missing data

2	(0.426) 0.431 0.428

3	(0.574) 0.569 0.572

Locus 17066 :

2 alleles

99.2% missing data

2	(0.631) 0.626 0.629

3	(0.369) 0.374 0.371

Locus 17067 :

2 alleles

90.6% missing data

4	(0.925) 0.939 0.913

3	(0.075) 0.061 0.087

Locus 17068 :

2 alleles

99.2% missing data

3	(0.392) 0.390 0.397

2	(0.608) 0.610 0.603

Locus 17069 :

2 alleles

99.2% missing data

3	(0.762) 0.754 0.763

1	(0.238) 0.246 0.237

Locus 17070 :

2 alleles

96.1% missing data

4	(0.832) 0.844 0.829

3	(0.168) 0.156 0.171

Locus 17071 :

2 alleles

99.2% missing data

1	(0.436) 0.426 0.446

2	(0.564) 0.574 0.554

Locus 17072 :

3 alleles

98.8% missing data

1	(0.414) 0.404 0.413

4	(0.246) 0.244 0.254

3	(0.340) 0.352 0.333

Locus 17073 :

3 alleles

98.8% missing data

2	(0.350) 0.348 0.358

1	(0.328) 0.326 0.327

3	(0.322) 0.326 0.315

Locus 17074 :

2 alleles

99.2% missing data

1	(0.708) 0.703 0.716

4	(0.292) 0.297 0.284

Locus 17075 :

2 alleles

99.2% missing data

4	(0.445) 0.444 0.455

3	(0.555) 0.556 0.545

Locus 17076 :

2 alleles

99.2% missing data

2	(0.705) 0.703 0.707

3	(0.295) 0.297 0.293

Locus 17077 :

2 alleles

99.2% missing data

2	(0.373) 0.366 0.380

3	(0.627) 0.634 0.620

Locus 17078 :

2 alleles

98.8% missing data

3	(0.592) 0.586 0.606

2	(0.408) 0.414 0.394

Locus 17079 :

2 alleles

98.8% missing data

3	(0.576) 0.574 0.585

1	(0.424) 0.426 0.415

Locus 17080 :

2 alleles

97.7% missing data

3	(0.250) 0.237 0.255

1	(0.750) 0.763 0.745

Locus 17081 :

2 alleles

96.9% missing data

1	(0.782) 0.782 0.786

3	(0.218) 0.218 0.214

Locus 17082 :

2 alleles

98.8% missing data

3	(0.731) 0.729 0.735

2	(0.269) 0.271 0.265

Locus 17083 :

2 alleles

99.2% missing data

1	(0.442) 0.443 0.446

2	(0.558) 0.557 0.554

Locus 17084 :

2 alleles

90.6% missing data

2	(0.858) 0.874 0.848

4	(0.142) 0.126 0.152

Locus 17085 :

2 alleles

99.2% missing data

4	(0.689) 0.683 0.693

2	(0.311) 0.317 0.307

Locus 17086 :

2 alleles

99.2% missing data

1	(0.187) 0.191 0.186

2	(0.813) 0.809 0.814

Locus 17087 :

2 alleles

99.2% missing data

3	(0.277) 0.270 0.282

4	(0.723) 0.730 0.718

Locus 17088 :

2 alleles

93.0% missing data

4	(0.881) 0.873 0.895

1	(0.119) 0.127 0.105

Locus 17089 :

2 alleles

99.2% missing data

3	(0.351) 0.347 0.355

1	(0.649) 0.653 0.645

Locus 17090 :

2 alleles

99.2% missing data

3	(0.283) 0.280 0.284

1	(0.717) 0.720 0.716

Locus 17091 :

2 alleles

98.8% missing data

4	(0.546) 0.546 0.546

3	(0.454) 0.454 0.454

Locus 17092 :

2 alleles

93.0% missing data

1	(0.877) 0.875 0.886

4	(0.123) 0.125 0.114

Locus 17093 :

2 alleles

99.2% missing data

4	(0.460) 0.456 0.463

3	(0.540) 0.544 0.537

Locus 17094 :

2 alleles

99.2% missing data

2	(0.247) 0.247 0.243

1	(0.753) 0.753 0.757

Locus 17095 :

3 alleles

98.0% missing data

4	(0.246) 0.244 0.250

3	(0.300) 0.291 0.309

1	(0.454) 0.465 0.441

Locus 17096 :

2 alleles

99.2% missing data

2	(0.451) 0.452 0.449

1	(0.549) 0.548 0.551

Locus 17097 :

2 alleles

90.6% missing data

4	(0.924) 0.937 0.917

1	(0.076) 0.063 0.083

Locus 17098 :

2 alleles

99.2% missing data

1	(0.482) 0.474 0.485

2	(0.518) 0.526 0.515

Locus 17099 :

2 alleles

99.2% missing data

2	(0.583) 0.588 0.579

1	(0.417) 0.412 0.421

Locus 17100 :

2 alleles

99.2% missing data

4	(0.447) 0.445 0.445

3	(0.553) 0.555 0.555

Locus 17101 :

2 alleles

99.2% missing data

4	(0.602) 0.594 0.604

1	(0.398) 0.406 0.396

Locus 17102 :

2 alleles

99.2% missing data

4	(0.727) 0.730 0.724

2	(0.273) 0.270 0.276

Locus 17103 :

2 alleles

99.2% missing data

2	(0.744) 0.735 0.746

1	(0.256) 0.265 0.254

Locus 17104 :

2 alleles

98.4% missing data

4	(0.698) 0.700 0.693

1	(0.302) 0.300 0.307

Locus 17105 :

2 alleles

99.2% missing data

4	(0.519) 0.518 0.522

3	(0.481) 0.482 0.478

Locus 17106 :

2 alleles

98.8% missing data

3	(0.582) 0.575 0.589

2	(0.418) 0.425 0.411

Locus 17107 :

2 alleles

98.8% missing data

4	(0.549) 0.555 0.544

1	(0.451) 0.445 0.456

Locus 17108 :

2 alleles

99.2% missing data

2	(0.548) 0.554 0.549

3	(0.452) 0.446 0.451

Locus 17109 :

2 alleles

98.8% missing data

4	(0.432) 0.426 0.437

3	(0.568) 0.574 0.563

Locus 17110 :

2 alleles

99.2% missing data

2	(0.760) 0.758 0.755

1	(0.240) 0.242 0.245

Locus 17111 :

2 alleles

99.2% missing data

1	(0.444) 0.441 0.446

3	(0.556) 0.559 0.554

Locus 17112 :

3 alleles

96.1% missing data

4	(0.719) 0.734 0.716

3	(0.159) 0.148 0.165

1	(0.121) 0.118 0.119

Locus 17113 :

2 alleles

98.8% missing data

2	(0.456) 0.453 0.460

3	(0.544) 0.547 0.540

Locus 17114 :

2 alleles

99.2% missing data

3	(0.733) 0.732 0.731

1	(0.267) 0.268 0.269

Locus 17115 :

2 alleles

99.2% missing data

3	(0.484) 0.482 0.488

4	(0.516) 0.518 0.512

Locus 17116 :

2 alleles

96.9% missing data

3	(0.692) 0.701 0.690

1	(0.308) 0.299 0.310

Locus 17117 :

2 alleles

98.8% missing data

2	(0.592) 0.589 0.600

4	(0.408) 0.411 0.400

Locus 17118 :

2 alleles

95.3% missing data

3	(0.901) 0.902 0.905

4	(0.099) 0.098 0.095

Locus 17119 :

2 alleles

99.2% missing data

3	(0.767) 0.762 0.775

2	(0.233) 0.238 0.225

Locus 17120 :

3 alleles

98.8% missing data

2	(0.326) 0.330 0.329

1	(0.295) 0.291 0.299

4	(0.379) 0.378 0.371

Locus 17121 :

2 alleles

99.2% missing data

1	(0.605) 0.605 0.598

2	(0.395) 0.395 0.402

Locus 17122 :

2 alleles

99.2% missing data

4	(0.511) 0.511 0.509

2	(0.489) 0.489 0.491

Locus 17123 :

2 alleles

99.2% missing data

2	(0.229) 0.229 0.234

4	(0.771) 0.771 0.766

Locus 17124 :

2 alleles

97.7% missing data

2	(0.245) 0.247 0.238

4	(0.755) 0.753 0.762

Locus 17125 :

2 alleles

99.2% missing data

2	(0.344) 0.343 0.356

1	(0.656) 0.657 0.644

Locus 17126 :

2 alleles

99.2% missing data

4	(0.474) 0.474 0.468

1	(0.526) 0.526 0.532

Locus 17127 :

2 alleles

98.8% missing data

1	(0.337) 0.331 0.353

3	(0.663) 0.669 0.647

Locus 17128 :

2 alleles

99.2% missing data

1	(0.482) 0.485 0.480

2	(0.518) 0.515 0.520

Locus 17129 :

2 alleles

99.2% missing data

4	(0.463) 0.462 0.466

3	(0.537) 0.538 0.534

Locus 17130 :

2 alleles

99.2% missing data

4	(0.661) 0.655 0.668

1	(0.339) 0.345 0.332

Locus 17131 :

2 alleles

99.2% missing data

2	(0.260) 0.251 0.267

4	(0.740) 0.749 0.733

Locus 17132 :

2 alleles

99.2% missing data

1	(0.242) 0.246 0.240

4	(0.758) 0.754 0.760

Locus 17133 :

2 alleles

99.2% missing data

1	(0.644) 0.648 0.644

3	(0.356) 0.352 0.356

Locus 17134 :

2 alleles

99.2% missing data

2	(0.184) 0.186 0.187

1	(0.816) 0.814 0.813

Locus 17135 :

2 alleles

99.2% missing data

3	(0.492) 0.484 0.496

1	(0.508) 0.516 0.504

Locus 17136 :

2 alleles

96.9% missing data

2	(0.212) 0.217 0.202

3	(0.788) 0.783 0.798

Locus 17137 :

2 alleles

99.2% missing data

2	(0.643) 0.643 0.638

1	(0.357) 0.357 0.362

Locus 17138 :

2 alleles

99.2% missing data

3	(0.536) 0.544 0.534

2	(0.464) 0.456 0.466

Locus 17139 :

2 alleles

99.2% missing data

1	(0.222) 0.229 0.221

2	(0.778) 0.771 0.779

Locus 17140 :

2 alleles

99.2% missing data

3	(0.332) 0.334 0.329

1	(0.668) 0.666 0.671

Locus 17141 :

2 alleles

95.7% missing data

3	(0.308) 0.288 0.318

2	(0.692) 0.712 0.682

Locus 17142 :

2 alleles

99.2% missing data

1	(0.396) 0.394 0.400

3	(0.604) 0.606 0.600

Locus 17143 :

2 alleles

97.3% missing data

2	(0.658) 0.661 0.669

1	(0.342) 0.339 0.331

Locus 17144 :

2 alleles

99.2% missing data

2	(0.438) 0.440 0.434

3	(0.562) 0.560 0.566

Locus 17145 :

2 alleles

99.2% missing data

3	(0.712) 0.714 0.704

1	(0.288) 0.286 0.296

Locus 17146 :

2 alleles

99.2% missing data

3	(0.505) 0.500 0.506

1	(0.495) 0.500 0.494

Locus 17147 :

2 alleles

99.2% missing data

1	(0.337) 0.333 0.341

2	(0.663) 0.667 0.659

Locus 17148 :

2 alleles

97.7% missing data

4	(0.151) 0.153 0.149

1	(0.849) 0.847 0.851

Locus 17149 :

2 alleles

99.2% missing data

4	(0.676) 0.674 0.673

1	(0.324) 0.326 0.327

Locus 17150 :

2 alleles

96.9% missing data

3	(0.781) 0.785 0.785

2	(0.219) 0.215 0.215

Locus 17151 :

2 alleles

99.2% missing data

2	(0.820) 0.823 0.815

4	(0.180) 0.177 0.185

Locus 17152 :

2 alleles

99.2% missing data

1	(0.515) 0.511 0.514

3	(0.485) 0.489 0.486

Locus 17153 :

2 alleles

95.7% missing data

2	(0.819) 0.828 0.815

1	(0.181) 0.172 0.185

Locus 17154 :

2 alleles

99.2% missing data

3	(0.555) 0.554 0.552

4	(0.445) 0.446 0.448

Locus 17155 :

2 alleles

98.8% missing data

1	(0.787) 0.781 0.789

4	(0.213) 0.219 0.211

Locus 17156 :

2 alleles

98.8% missing data

4	(0.545) 0.537 0.544

1	(0.455) 0.463 0.456

Locus 17157 :

2 alleles

98.0% missing data

3	(0.722) 0.722 0.725

2	(0.278) 0.278 0.275

Locus 17158 :

2 alleles

99.2% missing data

2	(0.447) 0.456 0.438

1	(0.553) 0.544 0.562

Locus 17159 :

2 alleles

96.5% missing data

3	(0.737) 0.752 0.734

2	(0.263) 0.248 0.266

Locus 17160 :

2 alleles

99.2% missing data

1	(0.249) 0.255 0.244

2	(0.751) 0.745 0.756

Locus 17161 :

2 alleles

99.2% missing data

3	(0.525) 0.527 0.528

1	(0.475) 0.473 0.472

Locus 17162 :

3 alleles

98.8% missing data

4	(0.349) 0.342 0.355

3	(0.314) 0.312 0.318

1	(0.337) 0.346 0.327

Locus 17163 :

2 alleles

98.8% missing data

4	(0.510) 0.514 0.515

3	(0.490) 0.486 0.485

Locus 17164 :

2 alleles

99.2% missing data

4	(0.372) 0.379 0.372

2	(0.628) 0.621 0.628

Locus 17165 :

2 alleles

99.2% missing data

3	(0.545) 0.539 0.542

1	(0.455) 0.461 0.458

Locus 17166 :

2 alleles

0.0% missing data

4	(0.987) 0.991 0.993

1	(0.013) 0.009 0.007

Locus 17167 :

2 alleles

99.2% missing data

1	(0.293) 0.290 0.293

2	(0.707) 0.710 0.707

Locus 17168 :

2 alleles

99.2% missing data

4	(0.604) 0.612 0.600

1	(0.396) 0.388 0.400

Locus 17169 :

2 alleles

99.2% missing data

1	(0.722) 0.723 0.716

2	(0.278) 0.277 0.284

Locus 17170 :

2 alleles

96.1% missing data

4	(0.874) 0.875 0.879

3	(0.126) 0.125 0.121

Locus 17171 :

2 alleles

91.0% missing data

2	(0.912) 0.921 0.911

3	(0.088) 0.079 0.089

Locus 17172 :

2 alleles

99.2% missing data

4	(0.530) 0.534 0.529

1	(0.470) 0.466 0.471

Locus 17173 :

2 alleles

98.8% missing data

4	(0.490) 0.491 0.477

3	(0.510) 0.509 0.523

Locus 17174 :

2 alleles

99.2% missing data

3	(0.335) 0.333 0.339

2	(0.665) 0.667 0.661

Locus 17175 :

2 alleles

99.2% missing data

4	(0.422) 0.426 0.413

2	(0.578) 0.574 0.587

Locus 17176 :

2 alleles

99.2% missing data

4	(0.561) 0.564 0.558

2	(0.439) 0.436 0.442

Locus 17177 :

2 alleles

98.4% missing data

3	(0.545) 0.543 0.536

4	(0.455) 0.457 0.464

Locus 17178 :

2 alleles

99.2% missing data

3	(0.545) 0.544 0.550

2	(0.455) 0.456 0.450

Locus 17179 :

2 alleles

96.1% missing data

2	(0.842) 0.842 0.849

3	(0.158) 0.158 0.151

Locus 17180 :

3 alleles

98.4% missing data

3	(0.602) 0.596 0.600

4	(0.280) 0.280 0.286

2	(0.118) 0.124 0.114

Locus 17181 :

2 alleles

93.0% missing data

4	(0.888) 0.884 0.900

3	(0.112) 0.116 0.100

Locus 17182 :

2 alleles

99.2% missing data

1	(0.524) 0.524 0.526

2	(0.476) 0.476 0.474

Locus 17183 :

2 alleles

99.2% missing data

4	(0.588) 0.581 0.593

1	(0.412) 0.419 0.407

Locus 17184 :

2 alleles

96.1% missing data

3	(0.803) 0.815 0.802

1	(0.197) 0.185 0.198

Locus 17185 :

2 alleles

98.8% missing data

3	(0.578) 0.579 0.585

4	(0.422) 0.421 0.415

Locus 17186 :

2 alleles

99.2% missing data

3	(0.495) 0.492 0.495

2	(0.505) 0.508 0.505

Locus 17187 :

2 alleles

99.2% missing data

3	(0.315) 0.315 0.316

4	(0.685) 0.685 0.684

Locus 17188 :

2 alleles

99.2% missing data

3	(0.331) 0.332 0.329

2	(0.669) 0.668 0.671

Locus 17189 :

2 alleles

99.2% missing data

4	(0.404) 0.401 0.411

3	(0.596) 0.599 0.589

Locus 17190 :

2 alleles

96.5% missing data

3	(0.837) 0.850 0.833

4	(0.163) 0.150 0.167

Locus 17191 :

2 alleles

95.3% missing data

4	(0.113) 0.111 0.105

3	(0.887) 0.889 0.895

Locus 17192 :

2 alleles

99.2% missing data

1	(0.468) 0.469 0.468

3	(0.532) 0.531 0.532

Locus 17193 :

2 alleles

99.2% missing data

3	(0.160) 0.167 0.162

1	(0.840) 0.833 0.838

Locus 17194 :

2 alleles

91.0% missing data

2	(0.924) 0.937 0.914

4	(0.076) 0.063 0.086

Locus 17195 :

2 alleles

98.8% missing data

3	(0.437) 0.432 0.440

2	(0.563) 0.568 0.560

Locus 17196 :

2 alleles

98.0% missing data

3	(0.820) 0.822 0.818

4	(0.180) 0.178 0.182

Locus 17197 :

2 alleles

99.2% missing data

3	(0.720) 0.712 0.725

4	(0.280) 0.288 0.275

Locus 17198 :

2 alleles

98.8% missing data

1	(0.567) 0.571 0.565

4	(0.433) 0.429 0.435

Locus 17199 :

2 alleles

97.7% missing data

1	(0.279) 0.278 0.276

3	(0.721) 0.722 0.724

Locus 17200 :

2 alleles

99.2% missing data

4	(0.392) 0.389 0.395

2	(0.608) 0.611 0.605

Locus 17201 :

2 alleles

99.2% missing data

2	(0.482) 0.480 0.476

3	(0.518) 0.520 0.524

Locus 17202 :

2 alleles

99.2% missing data

4	(0.627) 0.627 0.626

2	(0.373) 0.373 0.374

Locus 17203 :

4 alleles

92.2% missing data

2	(0.738) 0.730 0.767

4	(0.086) 0.084 0.079

3	(0.095) 0.094 0.087

1	(0.082) 0.092 0.067

Locus 17204 :

2 alleles

99.2% missing data

4	(0.599) 0.594 0.601

2	(0.401) 0.406 0.399

Locus 17205 :

2 alleles

99.2% missing data

3	(0.640) 0.639 0.644

1	(0.360) 0.361 0.356

Locus 17206 :

2 alleles

99.2% missing data

3	(0.353) 0.344 0.354

4	(0.647) 0.656 0.646

Locus 17207 :

2 alleles

99.2% missing data

1	(0.505) 0.514 0.502

3	(0.495) 0.486 0.498

Locus 17208 :

2 alleles

98.4% missing data

1	(0.703) 0.708 0.706

3	(0.297) 0.292 0.294

Locus 17209 :

2 alleles

93.4% missing data

1	(0.863) 0.850 0.882

3	(0.137) 0.150 0.118

Locus 17210 :

2 alleles

99.2% missing data

3	(0.553) 0.556 0.552

2	(0.447) 0.444 0.448

Locus 17211 :

2 alleles

99.2% missing data

3	(0.768) 0.760 0.777

4	(0.232) 0.240 0.223

Locus 17212 :

2 alleles

99.2% missing data

1	(0.587) 0.586 0.595

3	(0.413) 0.414 0.405

Locus 17213 :

2 alleles

99.2% missing data

1	(0.296) 0.293 0.303

3	(0.704) 0.707 0.697

Locus 17214 :

2 alleles

99.2% missing data

1	(0.651) 0.652 0.650

3	(0.349) 0.348 0.350

Locus 17215 :

2 alleles

99.2% missing data

1	(0.550) 0.553 0.554

3	(0.450) 0.447 0.446

Locus 17216 :

2 alleles

99.2% missing data

4	(0.488) 0.493 0.486

3	(0.512) 0.507 0.514

Locus 17217 :

2 alleles

99.2% missing data

1	(0.151) 0.149 0.162

2	(0.849) 0.851 0.838

Locus 17218 :

2 alleles

98.8% missing data

2	(0.427) 0.420 0.433

3	(0.573) 0.580 0.567

Locus 17219 :

2 alleles

93.8% missing data

3	(0.869) 0.862 0.883

1	(0.131) 0.138 0.117

Locus 17220 :

2 alleles

98.8% missing data

2	(0.454) 0.455 0.454

3	(0.546) 0.545 0.546

Locus 17221 :

2 alleles

91.4% missing data

3	(0.905) 0.902 0.920

1	(0.095) 0.098 0.080

Locus 17222 :

3 alleles

98.8% missing data

2	(0.333) 0.325 0.344

4	(0.284) 0.286 0.278

3	(0.383) 0.389 0.378

Locus 17223 :

2 alleles

99.2% missing data

3	(0.424) 0.423 0.426

2	(0.576) 0.577 0.574

Locus 17224 :

2 alleles

97.3% missing data

1	(0.150) 0.142 0.156

2	(0.850) 0.858 0.844

Locus 17225 :

2 alleles

99.2% missing data

1	(0.342) 0.343 0.349

2	(0.658) 0.657 0.651

Locus 17226 :

2 alleles

98.0% missing data

1	(0.794) 0.797 0.795

3	(0.206) 0.203 0.205

Locus 17227 :

2 alleles

95.7% missing data

3	(0.788) 0.795 0.795

1	(0.212) 0.205 0.205

Locus 17228 :

2 alleles

94.5% missing data

4	(0.803) 0.789 0.823

1	(0.197) 0.211 0.177

Locus 17229 :

2 alleles

99.2% missing data

4	(0.424) 0.423 0.424

2	(0.576) 0.577 0.576

Locus 17230 :

2 alleles

99.2% missing data

1	(0.518) 0.506 0.530

2	(0.482) 0.494 0.470

Locus 17231 :

2 alleles

98.8% missing data

3	(0.511) 0.517 0.510

2	(0.489) 0.483 0.490

Locus 17232 :

2 alleles

94.5% missing data

4	(0.771) 0.767 0.782

1	(0.229) 0.233 0.218

Locus 17233 :

3 alleles

92.2% missing data

4	(0.789) 0.783 0.817

3	(0.122) 0.120 0.110

1	(0.089) 0.097 0.073

Locus 17234 :

2 alleles

96.5% missing data

1	(0.808) 0.819 0.806

4	(0.192) 0.181 0.194

Locus 17235 :

2 alleles

98.8% missing data

1	(0.633) 0.636 0.634

4	(0.367) 0.364 0.366

Locus 17236 :

2 alleles

98.4% missing data

1	(0.716) 0.719 0.719

2	(0.284) 0.281 0.281

Locus 17237 :

3 alleles

96.1% missing data

1	(0.221) 0.220 0.215

2	(0.583) 0.578 0.596

4	(0.196) 0.202 0.190

Locus 17238 :

2 alleles

99.2% missing data

3	(0.627) 0.625 0.628

1	(0.373) 0.375 0.372

Locus 17239 :

2 alleles

99.2% missing data

4	(0.587) 0.586 0.590

1	(0.413) 0.414 0.410

Locus 17240 :

2 alleles

99.2% missing data

1	(0.658) 0.662 0.650

2	(0.342) 0.338 0.350

Locus 17241 :

2 alleles

99.2% missing data

1	(0.596) 0.592 0.599

2	(0.404) 0.408 0.401

Locus 17242 :

2 alleles

99.2% missing data

1	(0.121) 0.117 0.123

4	(0.879) 0.883 0.877

Locus 17243 :

2 alleles

98.4% missing data

4	(0.598) 0.598 0.606

3	(0.402) 0.402 0.394

Locus 17244 :

2 alleles

98.4% missing data

1	(0.655) 0.652 0.661

4	(0.345) 0.348 0.339

Locus 17245 :

2 alleles

99.2% missing data

1	(0.446) 0.446 0.449

2	(0.554) 0.554 0.551

Locus 17246 :

3 alleles

98.8% missing data

2	(0.322) 0.315 0.325

3	(0.190) 0.196 0.186

4	(0.488) 0.490 0.488

Locus 17247 :

2 alleles

96.5% missing data

2	(0.853) 0.853 0.858

1	(0.147) 0.147 0.142

Locus 17248 :

2 alleles

75.8% missing data

2	(0.951) 0.952 0.963

1	(0.049) 0.048 0.037

Locus 17249 :

2 alleles

98.4% missing data

2	(0.583) 0.596 0.580

4	(0.417) 0.404 0.420

Locus 17250 :

2 alleles

97.7% missing data

2	(0.523) 0.534 0.517

1	(0.477) 0.466 0.483

Locus 17251 :

2 alleles

98.8% missing data

1	(0.672) 0.671 0.673

2	(0.328) 0.329 0.327

Locus 17252 :

2 alleles

90.6% missing data

4	(0.923) 0.931 0.926

1	(0.077) 0.069 0.074

Locus 17253 :

2 alleles

98.4% missing data

1	(0.579) 0.574 0.579

3	(0.421) 0.426 0.421

Locus 17254 :

2 alleles

8.6% missing data

1	(0.984) 0.976 0.995

2	(0.016) 0.024 0.005

Locus 17255 :

2 alleles

91.0% missing data

1	(0.912) 0.917 0.916

3	(0.088) 0.083 0.084

Locus 17256 :

4 alleles

97.7% missing data

1	(0.372) 0.385 0.371

4	(0.375) 0.366 0.370

2	(0.141) 0.137 0.142

3	(0.112) 0.112 0.117

Locus 17257 :

2 alleles

99.2% missing data

1	(0.391) 0.396 0.390

2	(0.609) 0.604 0.610

Locus 17258 :

2 alleles

99.2% missing data

2	(0.567) 0.563 0.563

1	(0.433) 0.437 0.437

Locus 17259 :

2 alleles

99.2% missing data

2	(0.547) 0.546 0.546

3	(0.453) 0.454 0.454

Locus 17260 :

2 alleles

98.8% missing data

1	(0.349) 0.350 0.355

4	(0.651) 0.650 0.645

Locus 17261 :

2 alleles

99.2% missing data

3	(0.842) 0.844 0.836

2	(0.158) 0.156 0.164

Locus 17262 :

2 alleles

93.8% missing data

3	(0.920) 0.910 0.931

2	(0.080) 0.090 0.069

Locus 17263 :

2 alleles

98.8% missing data

3	(0.541) 0.545 0.539

4	(0.459) 0.455 0.461

Locus 17264 :

3 alleles

98.8% missing data

3	(0.226) 0.226 0.229

2	(0.234) 0.231 0.236

4	(0.539) 0.543 0.535

Locus 17265 :

2 alleles

99.2% missing data

1	(0.780) 0.774 0.784

4	(0.220) 0.226 0.216

Locus 17266 :

2 alleles

99.2% missing data

1	(0.641) 0.640 0.638

3	(0.359) 0.360 0.362

Locus 17267 :

2 alleles

99.2% missing data

4	(0.387) 0.385 0.386

1	(0.613) 0.615 0.614

Locus 17268 :

3 alleles

98.8% missing data

2	(0.299) 0.299 0.298

1	(0.381) 0.380 0.381

4	(0.320) 0.321 0.321

Locus 17269 :

2 alleles

98.8% missing data

1	(0.711) 0.711 0.710

4	(0.289) 0.289 0.290

Locus 17270 :

2 alleles

99.2% missing data

3	(0.446) 0.446 0.438

1	(0.554) 0.554 0.562

Locus 17271 :

2 alleles

98.8% missing data

4	(0.780) 0.775 0.784

1	(0.220) 0.225 0.216

Locus 17272 :

2 alleles

99.2% missing data

1	(0.674) 0.677 0.669

4	(0.326) 0.323 0.331

Locus 17273 :

2 alleles

96.1% missing data

4	(0.781) 0.791 0.774

1	(0.219) 0.209 0.226

Locus 17274 :

2 alleles

98.8% missing data

4	(0.845) 0.852 0.843

1	(0.155) 0.148 0.157

Locus 17275 :

3 alleles

98.4% missing data

4	(0.295) 0.290 0.304

1	(0.306) 0.304 0.310

3	(0.398) 0.407 0.387

Locus 17276 :

3 alleles

98.0% missing data

2	(0.391) 0.383 0.406

1	(0.292) 0.296 0.286

3	(0.316) 0.321 0.307

Locus 17277 :

2 alleles

0.4% missing data

4	(0.987) 0.991 0.993

1	(0.013) 0.009 0.007

Locus 17278 :

2 alleles

99.2% missing data

2	(0.657) 0.653 0.653

3	(0.343) 0.347 0.347

Locus 17279 :

2 alleles

99.2% missing data

3	(0.779) 0.775 0.785

4	(0.221) 0.225 0.215

Locus 17280 :

2 alleles

98.8% missing data

3	(0.240) 0.240 0.237

1	(0.760) 0.760 0.763

Locus 17281 :

3 alleles

94.1% missing data

1	(0.699) 0.682 0.725

2	(0.162) 0.162 0.153

4	(0.139) 0.156 0.122

Locus 17282 :

3 alleles

98.8% missing data

4	(0.411) 0.416 0.411

2	(0.178) 0.173 0.179

1	(0.411) 0.411 0.410

Locus 17283 :

2 alleles

99.2% missing data

3	(0.429) 0.424 0.436

2	(0.571) 0.576 0.564

Locus 17284 :

2 alleles

99.2% missing data

2	(0.576) 0.578 0.573

1	(0.424) 0.422 0.427

Locus 17285 :

2 alleles

99.2% missing data

1	(0.530) 0.529 0.525

4	(0.470) 0.471 0.475

Locus 17286 :

2 alleles

99.2% missing data

4	(0.432) 0.435 0.432

3	(0.568) 0.565 0.568

Locus 17287 :

2 alleles

99.2% missing data

4	(0.553) 0.552 0.552

1	(0.447) 0.448 0.448

Locus 17288 :

2 alleles

99.2% missing data

1	(0.218) 0.215 0.226

2	(0.782) 0.785 0.774

Locus 17289 :

2 alleles

99.2% missing data

4	(0.429) 0.426 0.433

2	(0.571) 0.574 0.567

Locus 17290 :

2 alleles

99.2% missing data

2	(0.651) 0.644 0.659

1	(0.349) 0.356 0.341

Locus 17291 :

2 alleles

99.2% missing data

3	(0.624) 0.633 0.621

1	(0.376) 0.367 0.379

Locus 17292 :

2 alleles

98.4% missing data

4	(0.573) 0.577 0.573

1	(0.427) 0.423 0.427

Locus 17293 :

2 alleles

96.1% missing data

4	(0.391) 0.394 0.375

1	(0.609) 0.606 0.625

Locus 17294 :

2 alleles

99.2% missing data

4	(0.729) 0.732 0.726

3	(0.271) 0.268 0.274

Locus 17295 :

2 alleles

93.8% missing data

1	(0.863) 0.864 0.873

4	(0.137) 0.136 0.127

Locus 17296 :

3 alleles

98.8% missing data

4	(0.495) 0.494 0.493

3	(0.200) 0.198 0.203

1	(0.306) 0.308 0.304

Locus 17297 :

2 alleles

99.2% missing data

3	(0.607) 0.615 0.600

4	(0.393) 0.385 0.400

Locus 17298 :

2 alleles

85.9% missing data

4	(0.758) 0.756 0.768

3	(0.242) 0.244 0.232

Locus 17299 :

2 alleles

99.2% missing data

4	(0.634) 0.634 0.637

3	(0.366) 0.366 0.363

Locus 17300 :

2 alleles

99.2% missing data

3	(0.263) 0.256 0.267

1	(0.737) 0.744 0.733

Locus 17301 :

2 alleles

99.2% missing data

4	(0.319) 0.317 0.323

2	(0.681) 0.683 0.677

Locus 17302 :

2 alleles

98.4% missing data

1	(0.709) 0.705 0.715

3	(0.291) 0.295 0.285

Locus 17303 :

2 alleles

76.2% missing data

4	(0.961) 0.964 0.968

3	(0.039) 0.036 0.032

Locus 17304 :

2 alleles

97.7% missing data

3	(0.624) 0.615 0.636

1	(0.376) 0.385 0.364

Locus 17305 :

2 alleles

0.0% missing data

1	(0.989) 0.984 0.997

4	(0.011) 0.016 0.003

Locus 17306 :

3 alleles

98.4% missing data

4	(0.297) 0.301 0.296

3	(0.392) 0.393 0.380

1	(0.311) 0.306 0.324

Locus 17307 :

2 alleles

93.8% missing data

1	(0.931) 0.932 0.935

2	(0.069) 0.068 0.065

Locus 17308 :

2 alleles

99.2% missing data

1	(0.592) 0.590 0.595

2	(0.408) 0.410 0.405

Locus 17309 :

3 alleles

98.8% missing data

1	(0.246) 0.249 0.248

3	(0.425) 0.415 0.420

4	(0.329) 0.336 0.331

Locus 17310 :

2 alleles

99.2% missing data

4	(0.445) 0.446 0.442

2	(0.555) 0.554 0.558

Locus 17311 :

2 alleles

99.2% missing data

2	(0.288) 0.288 0.288

3	(0.712) 0.712 0.712

Locus 17312 :

2 alleles

99.2% missing data

1	(0.583) 0.583 0.578

2	(0.417) 0.417 0.422

Locus 17313 :

2 alleles

99.2% missing data

1	(0.379) 0.378 0.384

3	(0.621) 0.622 0.616

Locus 17314 :

2 alleles

98.8% missing data

3	(0.467) 0.462 0.466

4	(0.533) 0.538 0.534

Locus 17315 :

2 alleles

96.1% missing data

1	(0.741) 0.746 0.740

3	(0.259) 0.254 0.260

Locus 17316 :

2 alleles

99.2% missing data

3	(0.357) 0.361 0.348

1	(0.643) 0.639 0.652

Locus 17317 :

2 alleles

99.2% missing data

1	(0.365) 0.360 0.367

4	(0.635) 0.640 0.633

Locus 17318 :

2 alleles

98.8% missing data

1	(0.597) 0.587 0.602

3	(0.403) 0.413 0.398

Locus 17319 :

2 alleles

91.8% missing data

2	(0.936) 0.933 0.946

1	(0.064) 0.067 0.054

Locus 17320 :

2 alleles

93.8% missing data

1	(0.873) 0.874 0.880

2	(0.127) 0.126 0.120

Locus 17321 :

2 alleles

99.2% missing data

4	(0.606) 0.611 0.611

3	(0.394) 0.389 0.389

Locus 17322 :

2 alleles

99.2% missing data

3	(0.670) 0.669 0.669

4	(0.330) 0.331 0.331

Locus 17323 :

2 alleles

99.2% missing data

2	(0.213) 0.212 0.216

1	(0.787) 0.788 0.784

Locus 17324 :

2 alleles

99.2% missing data

4	(0.359) 0.354 0.358

3	(0.641) 0.646 0.642

Locus 17325 :

2 alleles

99.2% missing data

3	(0.311) 0.314 0.311

4	(0.689) 0.686 0.689

Locus 17326 :

2 alleles

99.2% missing data

4	(0.460) 0.462 0.460

3	(0.540) 0.538 0.540

Locus 17327 :

2 alleles

98.8% missing data

4	(0.555) 0.552 0.546

2	(0.445) 0.448 0.454

Locus 17328 :

2 alleles

99.2% missing data

1	(0.287) 0.294 0.280

2	(0.713) 0.706 0.720

Locus 17329 :

2 alleles

98.8% missing data

2	(0.508) 0.522 0.501

3	(0.492) 0.478 0.499

Locus 17330 :

2 alleles

98.4% missing data

1	(0.700) 0.697 0.699

3	(0.300) 0.303 0.301

Locus 17331 :

2 alleles

99.2% missing data

3	(0.468) 0.465 0.472

1	(0.532) 0.535 0.528

Locus 17332 :

4 alleles

97.7% missing data

2	(0.251) 0.243 0.265

3	(0.195) 0.198 0.192

4	(0.275) 0.270 0.272

1	(0.279) 0.289 0.270

Locus 17333 :

2 alleles

84.8% missing data

1	(0.626) 0.695 0.542

3	(0.374) 0.305 0.458

Locus 17334 :

2 alleles

99.2% missing data

3	(0.413) 0.423 0.407

2	(0.587) 0.577 0.593

Locus 17335 :

2 alleles

99.2% missing data

3	(0.530) 0.522 0.536

4	(0.470) 0.478 0.464

Locus 17336 :

2 alleles

91.0% missing data

3	(0.917) 0.929 0.913

4	(0.083) 0.071 0.087

Locus 17337 :

2 alleles

90.6% missing data

4	(0.912) 0.930 0.900

3	(0.088) 0.070 0.100

Locus 17338 :

2 alleles

99.2% missing data

2	(0.357) 0.357 0.356

4	(0.643) 0.643 0.644

Locus 17339 :

2 alleles

98.8% missing data

2	(0.469) 0.458 0.481

1	(0.531) 0.542 0.519

Locus 17340 :

2 alleles

97.3% missing data

1	(0.335) 0.338 0.332

2	(0.665) 0.662 0.668

Locus 17341 :

2 alleles

99.2% missing data

1	(0.340) 0.336 0.341

3	(0.660) 0.664 0.659

Locus 17342 :

2 alleles

99.2% missing data

4	(0.468) 0.463 0.478

2	(0.532) 0.537 0.522

Locus 17343 :

2 alleles

99.2% missing data

1	(0.308) 0.312 0.306

4	(0.692) 0.688 0.694

Locus 17344 :

2 alleles

98.8% missing data

1	(0.577) 0.568 0.589

4	(0.423) 0.432 0.411

Locus 17345 :

2 alleles

91.0% missing data

3	(0.906) 0.902 0.918

1	(0.094) 0.098 0.082

Locus 17346 :

3 alleles

98.8% missing data

3	(0.188) 0.192 0.190

2	(0.405) 0.398 0.409

1	(0.407) 0.410 0.401

Locus 17347 :

2 alleles

96.5% missing data

1	(0.925) 0.919 0.929

2	(0.075) 0.081 0.071

Locus 17348 :

2 alleles

99.2% missing data

1	(0.560) 0.562 0.553

4	(0.440) 0.438 0.447

Locus 17349 :

2 alleles

99.2% missing data

1	(0.493) 0.486 0.499

4	(0.507) 0.514 0.501

Locus 17350 :

2 alleles

98.8% missing data

3	(0.643) 0.643 0.649

1	(0.357) 0.357 0.351

Locus 17351 :

2 alleles

99.2% missing data

1	(0.335) 0.339 0.331

2	(0.665) 0.661 0.669

Locus 17352 :

2 alleles

99.2% missing data

1	(0.453) 0.447 0.453

2	(0.547) 0.553 0.547

Locus 17353 :

2 alleles

99.2% missing data

1	(0.630) 0.627 0.634

3	(0.370) 0.373 0.366

Locus 17354 :

2 alleles

95.3% missing data

1	(0.827) 0.836 0.818

3	(0.173) 0.164 0.182

Locus 17355 :

2 alleles

99.2% missing data

2	(0.307) 0.306 0.313

1	(0.693) 0.694 0.687

Locus 17356 :

3 alleles

98.8% missing data

1	(0.303) 0.302 0.306

4	(0.468) 0.463 0.474

2	(0.229) 0.235 0.220

Locus 17357 :

2 alleles

98.8% missing data

3	(0.608) 0.615 0.609

1	(0.392) 0.385 0.391

Locus 17358 :

2 alleles

98.8% missing data

4	(0.600) 0.601 0.601

1	(0.400) 0.399 0.399

Locus 17359 :

2 alleles

99.2% missing data

2	(0.597) 0.599 0.596

4	(0.403) 0.401 0.404

Locus 17360 :

2 alleles

99.2% missing data

1	(0.647) 0.645 0.644

4	(0.353) 0.355 0.356

Locus 17361 :

2 alleles

97.3% missing data

4	(0.213) 0.212 0.205

1	(0.787) 0.788 0.795

Locus 17362 :

2 alleles

93.4% missing data

2	(0.929) 0.928 0.937

1	(0.071) 0.072 0.063

Locus 17363 :

2 alleles

99.2% missing data

1	(0.465) 0.460 0.465

3	(0.535) 0.540 0.535

Locus 17364 :

2 alleles

98.8% missing data

2	(0.850) 0.845 0.853

1	(0.150) 0.155 0.147

Locus 17365 :

2 alleles

99.2% missing data

3	(0.635) 0.625 0.639

1	(0.365) 0.375 0.361

Locus 17366 :

2 alleles

99.2% missing data

4	(0.519) 0.526 0.515

1	(0.481) 0.474 0.485

Locus 17367 :

2 alleles

99.2% missing data

1	(0.547) 0.547 0.547

4	(0.453) 0.453 0.453

Locus 17368 :

2 alleles

99.2% missing data

3	(0.594) 0.594 0.593

1	(0.406) 0.406 0.407

Locus 17369 :

2 alleles

99.2% missing data

1	(0.496) 0.488 0.505

3	(0.504) 0.512 0.495

Locus 17370 :

2 alleles

68.4% missing data

2	(0.817) 0.835 0.806

1	(0.183) 0.165 0.194

Locus 17371 :

2 alleles

97.3% missing data

2	(0.771) 0.768 0.762

3	(0.229) 0.232 0.238

Locus 17372 :

2 alleles

98.8% missing data

3	(0.683) 0.683 0.676

1	(0.317) 0.317 0.324

Locus 17373 :

3 alleles

98.8% missing data

2	(0.327) 0.320 0.325

4	(0.379) 0.376 0.391

1	(0.294) 0.304 0.284

Locus 17374 :

2 alleles

99.2% missing data

4	(0.390) 0.393 0.389

3	(0.610) 0.607 0.611

Locus 17375 :

3 alleles

98.8% missing data

2	(0.342) 0.343 0.347

1	(0.505) 0.494 0.506

3	(0.153) 0.163 0.147

Locus 17376 :

2 alleles

99.2% missing data

4	(0.445) 0.436 0.447

1	(0.555) 0.564 0.553

Locus 17377 :

2 alleles

99.2% missing data

1	(0.524) 0.527 0.528

2	(0.476) 0.473 0.472

Locus 17378 :

2 alleles

98.8% missing data

3	(0.445) 0.450 0.442

2	(0.555) 0.550 0.558

Locus 17379 :

2 alleles

98.4% missing data

1	(0.765) 0.770 0.760

2	(0.235) 0.230 0.240

Locus 17380 :

2 alleles

99.2% missing data

2	(0.669) 0.670 0.678

1	(0.331) 0.330 0.322

Locus 17381 :

2 alleles

98.4% missing data

3	(0.667) 0.671 0.670

4	(0.333) 0.329 0.330

Locus 17382 :

2 alleles

99.2% missing data

1	(0.356) 0.347 0.356

3	(0.644) 0.653 0.644

Locus 17383 :

2 alleles

99.2% missing data

1	(0.460) 0.458 0.464

3	(0.540) 0.542 0.536

Locus 17384 :

2 alleles

98.8% missing data

1	(0.694) 0.699 0.690

2	(0.306) 0.301 0.310

Locus 17385 :

2 alleles

99.2% missing data

1	(0.403) 0.401 0.402

4	(0.597) 0.599 0.598

Locus 17386 :

2 alleles

99.2% missing data

1	(0.648) 0.650 0.641

4	(0.352) 0.350 0.359

Locus 17387 :

2 alleles

99.2% missing data

2	(0.169) 0.172 0.173

1	(0.831) 0.828 0.827

Locus 17388 :

2 alleles

99.2% missing data

1	(0.372) 0.370 0.374

2	(0.628) 0.630 0.626

Locus 17389 :

2 alleles

98.4% missing data

1	(0.484) 0.482 0.493

3	(0.516) 0.518 0.507

Locus 17390 :

2 alleles

96.9% missing data

2	(0.332) 0.333 0.326

4	(0.668) 0.667 0.674

Locus 17391 :

2 alleles

95.7% missing data

2	(0.803) 0.816 0.800

4	(0.197) 0.184 0.200

Locus 17392 :

2 alleles

99.2% missing data

1	(0.669) 0.665 0.668

3	(0.331) 0.335 0.332

Locus 17393 :

2 alleles

0.0% missing data

3	(0.989) 0.984 0.997

2	(0.011) 0.016 0.003

Locus 17394 :

2 alleles

97.3% missing data

2	(0.279) 0.287 0.265

4	(0.721) 0.713 0.735

Locus 17395 :

2 alleles

99.2% missing data

4	(0.498) 0.496 0.499

3	(0.502) 0.504 0.501

Locus 17396 :

2 alleles

99.2% missing data

1	(0.547) 0.551 0.543

4	(0.453) 0.449 0.457

Locus 17397 :

2 alleles

99.2% missing data

2	(0.578) 0.578 0.575

4	(0.422) 0.422 0.425

Locus 17398 :

2 alleles

97.7% missing data

3	(0.791) 0.799 0.791

1	(0.209) 0.201 0.209

Locus 17399 :

2 alleles

96.5% missing data

1	(0.853) 0.855 0.855

3	(0.147) 0.145 0.145

Locus 17400 :

2 alleles

97.3% missing data

4	(0.696) 0.702 0.700

2	(0.304) 0.298 0.300

Locus 17401 :

2 alleles

98.8% missing data

2	(0.774) 0.768 0.780

1	(0.226) 0.232 0.220

Locus 17402 :

2 alleles

99.2% missing data

1	(0.672) 0.671 0.675

3	(0.328) 0.329 0.325

Locus 17403 :

2 alleles

99.2% missing data

1	(0.476) 0.475 0.476

2	(0.524) 0.525 0.524

Locus 17404 :

2 alleles

99.2% missing data

1	(0.309) 0.312 0.310

3	(0.691) 0.688 0.690

Locus 17405 :

2 alleles

35.5% missing data

2	(0.985) 0.980 0.994

3	(0.015) 0.020 0.006

Locus 17406 :

2 alleles

98.0% missing data

1	(0.406) 0.394 0.414

2	(0.594) 0.606 0.586

Locus 17407 :

2 alleles

99.2% missing data

2	(0.741) 0.738 0.742

1	(0.259) 0.262 0.258

Locus 17408 :

2 alleles

99.2% missing data

2	(0.193) 0.194 0.190

3	(0.807) 0.806 0.810

Locus 17409 :

3 alleles

98.8% missing data

4	(0.243) 0.237 0.250

1	(0.159) 0.163 0.161

2	(0.599) 0.600 0.589

Locus 17410 :

2 alleles

99.2% missing data

1	(0.561) 0.560 0.557

3	(0.439) 0.440 0.443

Locus 17411 :

2 alleles

99.2% missing data

2	(0.605) 0.604 0.609

1	(0.395) 0.396 0.391

Locus 17412 :

2 alleles

99.2% missing data

4	(0.462) 0.459 0.456

1	(0.538) 0.541 0.544

Locus 17413 :

2 alleles

99.2% missing data

2	(0.420) 0.415 0.419

4	(0.580) 0.585 0.581

Locus 17414 :

2 alleles

99.2% missing data

1	(0.535) 0.523 0.547

3	(0.465) 0.477 0.453

Locus 17415 :

2 alleles

91.4% missing data

3	(0.910) 0.915 0.912

1	(0.090) 0.085 0.088

Locus 17416 :

2 alleles

93.0% missing data

4	(0.955) 0.959 0.955

3	(0.045) 0.041 0.045

Locus 17417 :

2 alleles

99.2% missing data

1	(0.540) 0.533 0.542

3	(0.460) 0.467 0.458

Locus 17418 :

2 alleles

99.2% missing data

1	(0.461) 0.456 0.457

4	(0.539) 0.544 0.543

Locus 17419 :

2 alleles

99.2% missing data

1	(0.598) 0.596 0.599

4	(0.402) 0.404 0.401

Locus 17420 :

2 alleles

98.8% missing data

2	(0.736) 0.729 0.742

1	(0.264) 0.271 0.258

Locus 17421 :

2 alleles

97.7% missing data

2	(0.578) 0.582 0.576

4	(0.422) 0.418 0.424

Locus 17422 :

2 alleles

99.2% missing data

2	(0.688) 0.692 0.679

1	(0.312) 0.308 0.321

Locus 17423 :

3 alleles

97.3% missing data

4	(0.163) 0.161 0.165

1	(0.547) 0.548 0.562

2	(0.290) 0.291 0.273

Locus 17424 :

2 alleles

99.2% missing data

3	(0.335) 0.331 0.343

2	(0.665) 0.669 0.657

Locus 17425 :

2 alleles

96.1% missing data

3	(0.847) 0.858 0.840

2	(0.153) 0.142 0.160

Locus 17426 :

2 alleles

99.2% missing data

4	(0.635) 0.628 0.640

2	(0.365) 0.372 0.360

Locus 17427 :

2 alleles

99.2% missing data

4	(0.412) 0.411 0.417

1	(0.588) 0.589 0.583

Locus 17428 :

2 alleles

97.7% missing data

1	(0.204) 0.195 0.213

2	(0.796) 0.805 0.787

Locus 17429 :

4 alleles

98.0% missing data

4	(0.236) 0.230 0.241

3	(0.202) 0.196 0.205

2	(0.254) 0.250 0.255

1	(0.308) 0.324 0.298

Locus 17430 :

2 alleles

99.2% missing data

3	(0.425) 0.430 0.423

2	(0.575) 0.570 0.577

Locus 17431 :

2 alleles

99.2% missing data

1	(0.589) 0.588 0.588

4	(0.411) 0.412 0.412

Locus 17432 :

2 alleles

8.2% missing data

4	(0.983) 0.977 0.994

2	(0.017) 0.023 0.006

Locus 17433 :

2 alleles

99.2% missing data

4	(0.310) 0.304 0.318

3	(0.690) 0.696 0.682

Locus 17434 :

2 alleles

98.8% missing data

2	(0.730) 0.726 0.723

3	(0.270) 0.274 0.277

Locus 17435 :

2 alleles

0.4% missing data

4	(0.987) 0.985 0.995

2	(0.013) 0.015 0.005

Locus 17436 :

2 alleles

99.2% missing data

3	(0.292) 0.292 0.291

4	(0.708) 0.708 0.709

Locus 17437 :

2 alleles

99.2% missing data

1	(0.607) 0.611 0.605

3	(0.393) 0.389 0.395

Locus 17438 :

2 alleles

95.3% missing data

1	(0.847) 0.846 0.856

2	(0.153) 0.154 0.144

Locus 17439 :

2 alleles

98.0% missing data

2	(0.323) 0.322 0.318

4	(0.677) 0.678 0.682

Locus 17440 :

2 alleles

99.2% missing data

1	(0.687) 0.680 0.691

4	(0.313) 0.320 0.309

Locus 17441 :

2 alleles

99.2% missing data

4	(0.586) 0.580 0.592

2	(0.414) 0.420 0.408

Locus 17442 :

2 alleles

99.2% missing data

1	(0.729) 0.729 0.730

3	(0.271) 0.271 0.270

Locus 17443 :

2 alleles

99.2% missing data

4	(0.275) 0.279 0.279

1	(0.725) 0.721 0.721

Locus 17444 :

3 alleles

90.6% missing data

4	(0.863) 0.880 0.857

3	(0.074) 0.060 0.084

2	(0.063) 0.060 0.059

Locus 17445 :

2 alleles

99.2% missing data

4	(0.422) 0.426 0.423

2	(0.578) 0.574 0.577

Locus 17446 :

2 alleles

99.2% missing data

3	(0.599) 0.591 0.602

2	(0.401) 0.409 0.398

Locus 17447 :

2 alleles

99.2% missing data

2	(0.790) 0.781 0.795

1	(0.210) 0.219 0.205

Locus 17448 :

2 alleles

99.2% missing data

3	(0.641) 0.640 0.642

2	(0.359) 0.360 0.358

Locus 17449 :

2 alleles

98.8% missing data

4	(0.587) 0.581 0.595

3	(0.413) 0.419 0.405

Locus 17450 :

2 alleles

93.4% missing data

1	(0.618) 0.580 0.652

3	(0.382) 0.420 0.348

Locus 17451 :

3 alleles

98.8% missing data

1	(0.268) 0.260 0.276

4	(0.273) 0.271 0.269

2	(0.459) 0.468 0.456

Locus 17452 :

2 alleles

99.2% missing data

3	(0.517) 0.525 0.517

4	(0.483) 0.475 0.483

Locus 17453 :

2 alleles

99.2% missing data

2	(0.464) 0.464 0.465

3	(0.536) 0.536 0.535

Locus 17454 :

2 alleles

99.2% missing data

1	(0.261) 0.256 0.266

4	(0.739) 0.744 0.734

Locus 17455 :

2 alleles

96.1% missing data

4	(0.826) 0.830 0.831

2	(0.174) 0.170 0.169

Locus 17456 :

2 alleles

99.2% missing data

3	(0.616) 0.612 0.618

4	(0.384) 0.388 0.382

Locus 17457 :

2 alleles

93.0% missing data

3	(0.896) 0.897 0.900

1	(0.104) 0.103 0.100

Locus 17458 :

2 alleles

98.8% missing data

4	(0.754) 0.767 0.753

2	(0.246) 0.233 0.247

Locus 17459 :

2 alleles

99.2% missing data

3	(0.333) 0.336 0.337

1	(0.667) 0.664 0.663

Locus 17460 :

2 alleles

99.2% missing data

1	(0.363) 0.364 0.369

2	(0.637) 0.636 0.631

Locus 17461 :

2 alleles

97.3% missing data

4	(0.144) 0.141 0.142

1	(0.856) 0.859 0.858

Locus 17462 :

2 alleles

98.8% missing data

2	(0.479) 0.486 0.475

1	(0.521) 0.514 0.525

Locus 17463 :

3 alleles

98.8% missing data

1	(0.266) 0.266 0.268

3	(0.485) 0.484 0.482

2	(0.249) 0.250 0.250

Locus 17464 :

2 alleles

99.2% missing data

4	(0.356) 0.351 0.356

1	(0.644) 0.649 0.644

Locus 17465 :

2 alleles

96.1% missing data

4	(0.158) 0.147 0.169

2	(0.842) 0.853 0.831

Locus 17466 :

2 alleles

99.2% missing data

3	(0.527) 0.525 0.530

1	(0.473) 0.475 0.470

Locus 17467 :

3 alleles

98.8% missing data

1	(0.184) 0.182 0.186

2	(0.277) 0.274 0.286

4	(0.539) 0.544 0.528

Locus 17468 :

2 alleles

99.2% missing data

3	(0.487) 0.481 0.489

1	(0.513) 0.519 0.511

Locus 17469 :

2 alleles

99.2% missing data

3	(0.378) 0.374 0.382

4	(0.622) 0.626 0.618

Locus 17470 :

2 alleles

94.5% missing data

4	(0.853) 0.852 0.863

1	(0.147) 0.148 0.137

Locus 17471 :

2 alleles

99.2% missing data

1	(0.265) 0.270 0.264

4	(0.735) 0.730 0.736

Locus 17472 :

2 alleles

99.2% missing data

4	(0.508) 0.501 0.511

2	(0.492) 0.499 0.489

Locus 17473 :

2 alleles

99.2% missing data

2	(0.673) 0.672 0.677

4	(0.327) 0.328 0.323

Locus 17474 :

2 alleles

9.0% missing data

4	(0.986) 0.981 0.996

2	(0.014) 0.019 0.004

Locus 17475 :

2 alleles

98.8% missing data

2	(0.589) 0.596 0.580

1	(0.411) 0.404 0.420

Locus 17476 :

2 alleles

96.9% missing data

3	(0.168) 0.159 0.179

1	(0.832) 0.841 0.821

Locus 17477 :

2 alleles

96.1% missing data

3	(0.091) 0.085 0.097

4	(0.909) 0.915 0.903

Locus 17478 :

2 alleles

99.2% missing data

1	(0.425) 0.417 0.434

2	(0.575) 0.583 0.566

Locus 17479 :

2 alleles

99.2% missing data

3	(0.232) 0.227 0.236

1	(0.768) 0.773 0.764

Locus 17480 :

2 alleles

95.3% missing data

4	(0.878) 0.869 0.892

1	(0.122) 0.131 0.108

Locus 17481 :

2 alleles

96.5% missing data

4	(0.745) 0.758 0.740

1	(0.255) 0.242 0.260

Locus 17482 :

2 alleles

99.2% missing data

4	(0.248) 0.241 0.259

3	(0.752) 0.759 0.741

Locus 17483 :

2 alleles

99.2% missing data

4	(0.499) 0.499 0.499

1	(0.501) 0.501 0.501

Locus 17484 :

2 alleles

98.8% missing data

1	(0.386) 0.381 0.395

4	(0.614) 0.619 0.605

Locus 17485 :

2 alleles

98.0% missing data

1	(0.682) 0.677 0.691

2	(0.318) 0.323 0.309

Locus 17486 :

2 alleles

99.2% missing data

2	(0.521) 0.519 0.522

1	(0.479) 0.481 0.478

Locus 17487 :

2 alleles

97.3% missing data

1	(0.641) 0.630 0.660

2	(0.359) 0.370 0.340

Locus 17488 :

2 alleles

99.2% missing data

1	(0.624) 0.617 0.631

4	(0.376) 0.383 0.369

Locus 17489 :

2 alleles

99.2% missing data

3	(0.620) 0.620 0.612

1	(0.380) 0.380 0.388

Locus 17490 :

2 alleles

99.2% missing data

3	(0.580) 0.575 0.590

1	(0.420) 0.425 0.410

Locus 17491 :

2 alleles

0.0% missing data

4	(0.985) 0.990 0.993

1	(0.015) 0.010 0.007

Locus 17492 :

2 alleles

99.2% missing data

3	(0.576) 0.580 0.581

1	(0.424) 0.420 0.419

Locus 17493 :

2 alleles

99.2% missing data

3	(0.336) 0.342 0.334

1	(0.664) 0.658 0.666

Locus 17494 :

2 alleles

99.2% missing data

2	(0.408) 0.405 0.409

4	(0.592) 0.595 0.591

Locus 17495 :

2 alleles

98.4% missing data

1	(0.793) 0.793 0.792

3	(0.207) 0.207 0.208

Locus 17496 :

2 alleles

96.1% missing data

2	(0.847) 0.847 0.856

4	(0.153) 0.153 0.144

Locus 17497 :

2 alleles

99.2% missing data

1	(0.429) 0.430 0.426

3	(0.571) 0.570 0.574

Locus 17498 :

2 alleles

99.2% missing data

2	(0.402) 0.406 0.405

4	(0.598) 0.594 0.595

Locus 17499 :

2 alleles

97.7% missing data

4	(0.738) 0.733 0.747

2	(0.262) 0.267 0.253

Locus 17500 :

2 alleles

99.2% missing data

2	(0.355) 0.357 0.353

1	(0.645) 0.643 0.647

Locus 17501 :

2 alleles

98.8% missing data

4	(0.661) 0.656 0.663

2	(0.339) 0.344 0.337

Locus 17502 :

2 alleles

90.6% missing data

1	(0.879) 0.885 0.880

4	(0.121) 0.115 0.120

Locus 17503 :

2 alleles

99.2% missing data

4	(0.206) 0.218 0.196

2	(0.794) 0.782 0.804

Locus 17504 :

2 alleles

99.2% missing data

1	(0.485) 0.482 0.492

2	(0.515) 0.518 0.508

Locus 17505 :

2 alleles

99.2% missing data

4	(0.203) 0.200 0.207

2	(0.797) 0.800 0.793

Locus 17506 :

2 alleles

99.2% missing data

4	(0.639) 0.643 0.637

1	(0.361) 0.357 0.363

Locus 17507 :

2 alleles

99.2% missing data

2	(0.563) 0.557 0.559

4	(0.437) 0.443 0.441

Locus 17508 :

3 alleles

98.4% missing data

3	(0.470) 0.467 0.475

1	(0.158) 0.165 0.151

2	(0.372) 0.367 0.374

Locus 17509 :

2 alleles

99.2% missing data

4	(0.465) 0.463 0.465

1	(0.535) 0.537 0.535

Locus 17510 :

2 alleles

99.2% missing data

1	(0.449) 0.447 0.447

3	(0.551) 0.553 0.553

Locus 17511 :

2 alleles

99.2% missing data

4	(0.705) 0.703 0.713

2	(0.295) 0.297 0.287

Locus 17512 :

2 alleles

99.2% missing data

4	(0.436) 0.438 0.433

1	(0.564) 0.562 0.567

Locus 17513 :

2 alleles

99.2% missing data

3	(0.599) 0.596 0.602

2	(0.401) 0.404 0.398

Locus 17514 :

2 alleles

99.2% missing data

1	(0.466) 0.462 0.472

4	(0.534) 0.538 0.528

Locus 17515 :

2 alleles

99.2% missing data

4	(0.467) 0.465 0.466

1	(0.533) 0.535 0.534

Locus 17516 :

2 alleles

99.2% missing data

1	(0.590) 0.585 0.595

2	(0.410) 0.415 0.405

Locus 17517 :

2 alleles

99.2% missing data

4	(0.446) 0.446 0.440

1	(0.554) 0.554 0.560

Locus 17518 :

2 alleles

96.1% missing data

1	(0.792) 0.796 0.795

3	(0.208) 0.204 0.205

Locus 17519 :

2 alleles

96.1% missing data

2	(0.890) 0.889 0.894

1	(0.110) 0.111 0.106

Locus 17520 :

2 alleles

99.2% missing data

2	(0.592) 0.591 0.592

3	(0.408) 0.409 0.408

Locus 17521 :

2 alleles

99.2% missing data

1	(0.627) 0.621 0.631

4	(0.373) 0.379 0.369

Locus 17522 :

2 alleles

99.2% missing data

3	(0.728) 0.727 0.727

2	(0.272) 0.273 0.273

Locus 17523 :

2 alleles

97.7% missing data

1	(0.698) 0.689 0.711

4	(0.302) 0.311 0.289

Locus 17524 :

2 alleles

99.2% missing data

2	(0.250) 0.249 0.253

1	(0.750) 0.751 0.747

Locus 17525 :

2 alleles

99.2% missing data

3	(0.416) 0.410 0.419

2	(0.584) 0.590 0.581

Locus 17526 :

2 alleles

99.2% missing data

2	(0.333) 0.331 0.338

4	(0.667) 0.669 0.662

Locus 17527 :

2 alleles

96.5% missing data

4	(0.865) 0.876 0.858

1	(0.135) 0.124 0.142

Locus 17528 :

2 alleles

99.2% missing data

2	(0.727) 0.727 0.730

1	(0.273) 0.273 0.270

Locus 17529 :

2 alleles

8.2% missing data

4	(0.983) 0.978 0.995

2	(0.017) 0.022 0.005

Locus 17530 :

2 alleles

99.2% missing data

1	(0.431) 0.427 0.428

4	(0.569) 0.573 0.572

Locus 17531 :

2 alleles

99.2% missing data

4	(0.681) 0.681 0.675

1	(0.319) 0.319 0.325

Locus 17532 :

2 alleles

99.2% missing data

3	(0.550) 0.541 0.556

2	(0.450) 0.459 0.444

Locus 17533 :

2 alleles

94.5% missing data

1	(0.919) 0.910 0.929

2	(0.081) 0.090 0.071

Locus 17534 :

3 alleles

98.4% missing data

3	(0.525) 0.524 0.525

1	(0.303) 0.304 0.303

4	(0.172) 0.172 0.172

Locus 17535 :

2 alleles

98.8% missing data

3	(0.485) 0.486 0.489

4	(0.515) 0.514 0.511

Locus 17536 :

2 alleles

97.7% missing data

1	(0.800) 0.800 0.795

2	(0.200) 0.200 0.205

Locus 17537 :

2 alleles

98.0% missing data

2	(0.820) 0.812 0.824

4	(0.180) 0.188 0.176

Locus 17538 :

2 alleles

99.2% missing data

4	(0.682) 0.688 0.678

3	(0.318) 0.312 0.322

Locus 17539 :

2 alleles

96.1% missing data

2	(0.178) 0.173 0.180

3	(0.822) 0.827 0.820

Locus 17540 :

2 alleles

98.4% missing data

4	(0.634) 0.637 0.631

1	(0.366) 0.363 0.369

Locus 17541 :

2 alleles

99.2% missing data

4	(0.572) 0.573 0.570

2	(0.428) 0.427 0.430

Locus 17542 :

2 alleles

99.2% missing data

2	(0.519) 0.511 0.524

4	(0.481) 0.489 0.476

Locus 17543 :

2 alleles

99.2% missing data

4	(0.566) 0.557 0.572

1	(0.434) 0.443 0.428

Locus 17544 :

2 alleles

98.0% missing data

2	(0.733) 0.726 0.748

1	(0.267) 0.274 0.252

Locus 17545 :

2 alleles

98.4% missing data

2	(0.181) 0.171 0.188

1	(0.819) 0.829 0.812

Locus 17546 :

2 alleles

98.8% missing data

1	(0.274) 0.271 0.275

4	(0.726) 0.729 0.725

Locus 17547 :

2 alleles

99.2% missing data

1	(0.566) 0.569 0.568

4	(0.434) 0.431 0.432

Locus 17548 :

3 alleles

96.5% missing data

4	(0.614) 0.619 0.627

2	(0.107) 0.103 0.105

1	(0.279) 0.278 0.268

Locus 17549 :

2 alleles

99.2% missing data

2	(0.436) 0.433 0.440

1	(0.564) 0.567 0.560

Locus 17550 :

2 alleles

99.2% missing data

1	(0.614) 0.616 0.612

4	(0.386) 0.384 0.388

Locus 17551 :

2 alleles

98.8% missing data

3	(0.355) 0.360 0.353

2	(0.645) 0.640 0.647

Locus 17552 :

2 alleles

99.2% missing data

3	(0.286) 0.280 0.289

4	(0.714) 0.720 0.711

Locus 17553 :

2 alleles

99.2% missing data

1	(0.470) 0.468 0.474

2	(0.530) 0.532 0.526

Locus 17554 :

2 alleles

99.2% missing data

3	(0.667) 0.672 0.666

2	(0.333) 0.328 0.334

Locus 17555 :

2 alleles

99.2% missing data

2	(0.756) 0.760 0.750

1	(0.244) 0.240 0.250

Locus 17556 :

2 alleles

99.2% missing data

4	(0.173) 0.169 0.175

2	(0.827) 0.831 0.825

Locus 17557 :

2 alleles

99.2% missing data

4	(0.784) 0.784 0.779

2	(0.216) 0.216 0.221

Locus 17558 :

2 alleles

99.2% missing data

2	(0.586) 0.584 0.581

1	(0.414) 0.416 0.419

Locus 17559 :

2 alleles

98.0% missing data

4	(0.714) 0.708 0.722

3	(0.286) 0.292 0.278

Locus 17560 :

2 alleles

99.2% missing data

1	(0.412) 0.407 0.420

2	(0.588) 0.593 0.580

Locus 17561 :

3 alleles

96.9% missing data

3	(0.254) 0.254 0.249

2	(0.521) 0.515 0.532

4	(0.225) 0.231 0.218

Locus 17562 :

2 alleles

99.2% missing data

4	(0.501) 0.489 0.503

2	(0.499) 0.511 0.497

Locus 17563 :

2 alleles

99.2% missing data

1	(0.421) 0.412 0.427

3	(0.579) 0.588 0.573

Locus 17564 :

2 alleles

89.1% missing data

1	(0.249) 0.251 0.237

3	(0.751) 0.749 0.763

Locus 17565 :

2 alleles

99.2% missing data

1	(0.480) 0.481 0.479

3	(0.520) 0.519 0.521

Locus 17566 :

2 alleles

99.2% missing data

2	(0.509) 0.508 0.512

3	(0.491) 0.492 0.488

Locus 17567 :

2 alleles

98.8% missing data

2	(0.551) 0.549 0.547

1	(0.449) 0.451 0.453

Locus 17568 :

2 alleles

99.2% missing data

4	(0.541) 0.545 0.539

2	(0.459) 0.455 0.461

Locus 17569 :

2 alleles

92.6% missing data

1	(0.886) 0.880 0.906

4	(0.114) 0.120 0.094

Locus 17570 :

2 alleles

95.3% missing data

1	(0.174) 0.177 0.161

3	(0.826) 0.823 0.839

Locus 17571 :

2 alleles

99.2% missing data

3	(0.547) 0.550 0.549

4	(0.453) 0.450 0.451

Locus 17572 :

2 alleles

96.1% missing data

3	(0.864) 0.876 0.858

1	(0.136) 0.124 0.142

Locus 17573 :

3 alleles

98.8% missing data

4	(0.335) 0.336 0.334

2	(0.196) 0.192 0.201

1	(0.469) 0.471 0.465

Locus 17574 :

2 alleles

99.2% missing data

4	(0.649) 0.650 0.647

2	(0.351) 0.350 0.353

Locus 17575 :

2 alleles

94.9% missing data

1	(0.848) 0.841 0.862

4	(0.152) 0.159 0.138

Locus 17576 :

2 alleles

99.2% missing data

1	(0.567) 0.566 0.568

4	(0.433) 0.434 0.432

Locus 17577 :

2 alleles

98.8% missing data

2	(0.520) 0.506 0.520

4	(0.480) 0.494 0.480

Locus 17578 :

2 alleles

98.8% missing data

1	(0.625) 0.623 0.632

2	(0.375) 0.377 0.368

Locus 17579 :

2 alleles

99.2% missing data

2	(0.264) 0.260 0.273

1	(0.736) 0.740 0.727

Locus 17580 :

2 alleles

99.2% missing data

4	(0.663) 0.652 0.665

2	(0.337) 0.348 0.335

Locus 17581 :

2 alleles

98.4% missing data

4	(0.705) 0.705 0.710

1	(0.295) 0.295 0.290

Locus 17582 :

3 alleles

91.0% missing data

1	(0.670) 0.631 0.711

2	(0.239) 0.276 0.207

3	(0.091) 0.093 0.082

Locus 17583 :

2 alleles

99.2% missing data

1	(0.379) 0.381 0.380

3	(0.621) 0.619 0.620

Locus 17584 :

2 alleles

99.2% missing data

3	(0.500) 0.494 0.505

1	(0.500) 0.506 0.495

Locus 17585 :

2 alleles

99.2% missing data

2	(0.652) 0.661 0.647

3	(0.348) 0.339 0.353

Locus 17586 :

2 alleles

91.4% missing data

2	(0.929) 0.921 0.940

1	(0.071) 0.079 0.060

Locus 17587 :

2 alleles

98.8% missing data

1	(0.351) 0.343 0.357

2	(0.649) 0.657 0.643

Locus 17588 :

2 alleles

99.2% missing data

2	(0.343) 0.342 0.345

4	(0.657) 0.658 0.655

Locus 17589 :

2 alleles

99.2% missing data

4	(0.549) 0.556 0.540

2	(0.451) 0.444 0.460

Locus 17590 :

2 alleles

96.9% missing data

4	(0.892) 0.891 0.896

1	(0.108) 0.109 0.104

Locus 17591 :

2 alleles

97.3% missing data

3	(0.713) 0.729 0.704

4	(0.287) 0.271 0.296

Locus 17592 :

2 alleles

98.4% missing data

4	(0.656) 0.652 0.663

2	(0.344) 0.348 0.337

Locus 17593 :

2 alleles

91.0% missing data

2	(0.930) 0.936 0.930

3	(0.070) 0.064 0.070

Locus 17594 :

3 alleles

98.8% missing data

1	(0.321) 0.317 0.325

4	(0.227) 0.232 0.226

2	(0.452) 0.451 0.450

Locus 17595 :

2 alleles

98.4% missing data

1	(0.736) 0.735 0.739

3	(0.264) 0.265 0.261

Locus 17596 :

3 alleles

98.8% missing data

1	(0.525) 0.521 0.524

2	(0.234) 0.231 0.238

4	(0.240) 0.248 0.238

Locus 17597 :

3 alleles

98.0% missing data

1	(0.307) 0.320 0.298

4	(0.248) 0.239 0.263

3	(0.445) 0.441 0.438

Locus 17598 :

2 alleles

95.3% missing data

4	(0.138) 0.127 0.140

2	(0.862) 0.873 0.860

Locus 17599 :

2 alleles

93.4% missing data

4	(0.700) 0.692 0.727

3	(0.300) 0.308 0.273

Locus 17600 :

2 alleles

99.2% missing data

1	(0.644) 0.638 0.645

2	(0.356) 0.362 0.355

Locus 17601 :

2 alleles

99.2% missing data

1	(0.543) 0.541 0.533

2	(0.457) 0.459 0.467

Locus 17602 :

2 alleles

99.2% missing data

4	(0.202) 0.205 0.206

2	(0.798) 0.795 0.794

Locus 17603 :

2 alleles

98.0% missing data

1	(0.767) 0.775 0.757

3	(0.233) 0.225 0.243

Locus 17604 :

3 alleles

98.0% missing data

2	(0.168) 0.164 0.173

3	(0.322) 0.333 0.322

1	(0.509) 0.503 0.505

Locus 17605 :

2 alleles

98.8% missing data

3	(0.293) 0.292 0.293

4	(0.707) 0.708 0.707

Locus 17606 :

2 alleles

99.2% missing data

3	(0.823) 0.816 0.826

4	(0.177) 0.184 0.174

Locus 17607 :

2 alleles

96.1% missing data

2	(0.198) 0.189 0.196

3	(0.802) 0.811 0.804

Locus 17608 :

2 alleles

99.2% missing data

1	(0.530) 0.538 0.526

4	(0.470) 0.462 0.474

Locus 17609 :

2 alleles

99.2% missing data

2	(0.629) 0.619 0.635

3	(0.371) 0.381 0.365

Locus 17610 :

2 alleles

99.2% missing data

2	(0.356) 0.356 0.360

1	(0.644) 0.644 0.640

Locus 17611 :

3 alleles

98.4% missing data

1	(0.610) 0.612 0.603

3	(0.196) 0.192 0.199

2	(0.194) 0.196 0.198

Locus 17612 :

2 alleles

99.2% missing data

3	(0.562) 0.554 0.568

4	(0.438) 0.446 0.432

Locus 17613 :

2 alleles

98.8% missing data

4	(0.649) 0.645 0.658

2	(0.351) 0.355 0.342

Locus 17614 :

2 alleles

98.8% missing data

4	(0.641) 0.650 0.636

1	(0.359) 0.350 0.364

Locus 17615 :

2 alleles

98.8% missing data

1	(0.458) 0.455 0.465

4	(0.542) 0.545 0.535

Locus 17616 :

2 alleles

96.1% missing data

4	(0.859) 0.871 0.850

2	(0.141) 0.129 0.150

Locus 17617 :

2 alleles

99.2% missing data

4	(0.458) 0.456 0.455

1	(0.542) 0.544 0.545

Locus 17618 :

2 alleles

99.2% missing data

3	(0.627) 0.623 0.633

2	(0.373) 0.377 0.367

Locus 17619 :

2 alleles

8.2% missing data

2	(0.983) 0.973 0.995

3	(0.017) 0.027 0.005

Locus 17620 :

2 alleles

99.2% missing data

4	(0.565) 0.560 0.563

1	(0.435) 0.440 0.437

Locus 17621 :

2 alleles

99.2% missing data

3	(0.602) 0.601 0.606

1	(0.398) 0.399 0.394

Locus 17622 :

2 alleles

99.2% missing data

3	(0.438) 0.433 0.450

1	(0.562) 0.567 0.550

Locus 17623 :

2 alleles

95.7% missing data

2	(0.136) 0.130 0.141

1	(0.864) 0.870 0.859

Locus 17624 :

2 alleles

99.2% missing data

1	(0.477) 0.471 0.478

2	(0.523) 0.529 0.522

Locus 17625 :

3 alleles

98.8% missing data

2	(0.312) 0.309 0.316

4	(0.382) 0.379 0.383

3	(0.306) 0.313 0.302

Locus 17626 :

2 alleles

99.2% missing data

2	(0.655) 0.658 0.656

1	(0.345) 0.342 0.344

Locus 17627 :

2 alleles

94.9% missing data

3	(0.898) 0.899 0.905

2	(0.102) 0.101 0.095

Locus 17628 :

2 alleles

99.2% missing data

4	(0.626) 0.626 0.626

1	(0.374) 0.374 0.374

Locus 17629 :

2 alleles

99.2% missing data

2	(0.416) 0.419 0.423

1	(0.584) 0.581 0.577

Locus 17630 :

2 alleles

99.2% missing data

2	(0.276) 0.274 0.276

1	(0.724) 0.726 0.724

Locus 17631 :

2 alleles

99.2% missing data

3	(0.286) 0.286 0.293

1	(0.714) 0.714 0.707

Locus 17632 :

2 alleles

98.8% missing data

2	(0.815) 0.818 0.819

4	(0.185) 0.182 0.181

Locus 17633 :

3 alleles

98.8% missing data

3	(0.221) 0.229 0.215

2	(0.621) 0.610 0.631

1	(0.158) 0.161 0.155

Locus 17634 :

2 alleles

98.0% missing data

1	(0.514) 0.531 0.505

3	(0.486) 0.469 0.495

Locus 17635 :

2 alleles

98.8% missing data

2	(0.796) 0.798 0.793

1	(0.204) 0.202 0.207

Locus 17636 :

2 alleles

8.2% missing data

3	(0.979) 0.974 0.994

2	(0.021) 0.026 0.006

Locus 17637 :

3 alleles

90.6% missing data

1	(0.845) 0.873 0.829

2	(0.108) 0.091 0.117

3	(0.047) 0.037 0.055

Locus 17638 :

2 alleles

99.2% missing data

4	(0.264) 0.263 0.264

2	(0.736) 0.737 0.736

Locus 17639 :

2 alleles

90.2% missing data

3	(0.931) 0.927 0.941

4	(0.069) 0.073 0.059

Locus 17640 :

3 alleles

93.0% missing data

2	(0.842) 0.822 0.865

1	(0.090) 0.102 0.076

4	(0.069) 0.076 0.059

Locus 17641 :

2 alleles

98.4% missing data

1	(0.612) 0.606 0.621

4	(0.388) 0.394 0.379

Locus 17642 :

2 alleles

99.2% missing data

1	(0.403) 0.399 0.405

2	(0.597) 0.601 0.595

Locus 17643 :

3 alleles

97.7% missing data

3	(0.509) 0.522 0.511

1	(0.369) 0.355 0.370

2	(0.123) 0.123 0.119

Locus 17644 :

2 alleles

99.2% missing data

1	(0.664) 0.664 0.660

3	(0.336) 0.336 0.340

Locus 17645 :

2 alleles

99.2% missing data

1	(0.766) 0.770 0.763

2	(0.234) 0.230 0.237

Locus 17646 :

2 alleles

98.8% missing data

2	(0.735) 0.741 0.736

1	(0.265) 0.259 0.264

Locus 17647 :

2 alleles

99.2% missing data

1	(0.364) 0.368 0.361

4	(0.636) 0.632 0.639

Locus 17648 :

2 alleles

99.2% missing data

3	(0.312) 0.310 0.311

1	(0.688) 0.690 0.689

Locus 17649 :

2 alleles

97.3% missing data

2	(0.870) 0.872 0.869

3	(0.130) 0.128 0.131

Locus 17650 :

2 alleles

99.2% missing data

1	(0.667) 0.665 0.671

3	(0.333) 0.335 0.329

Locus 17651 :

2 alleles

99.2% missing data

3	(0.346) 0.343 0.347

1	(0.654) 0.657 0.653

Locus 17652 :

2 alleles

99.2% missing data

2	(0.473) 0.473 0.479

1	(0.527) 0.527 0.521

Locus 17653 :

2 alleles

98.8% missing data

4	(0.603) 0.590 0.606

3	(0.397) 0.410 0.394

Locus 17654 :

2 alleles

98.0% missing data

2	(0.718) 0.719 0.723

3	(0.282) 0.281 0.277

Locus 17655 :

2 alleles

99.2% missing data

1	(0.774) 0.772 0.770

3	(0.226) 0.228 0.230

Locus 17656 :

2 alleles

98.8% missing data

1	(0.478) 0.481 0.483

4	(0.522) 0.519 0.517

Locus 17657 :

2 alleles

99.2% missing data

4	(0.296) 0.295 0.296

3	(0.704) 0.705 0.704

Locus 17658 :

2 alleles

99.2% missing data

1	(0.468) 0.465 0.469

2	(0.532) 0.535 0.531

Locus 17659 :

2 alleles

98.0% missing data

1	(0.710) 0.721 0.702

2	(0.290) 0.279 0.298

Locus 17660 :

2 alleles

0.0% missing data

4	(0.980) 0.985 0.988

1	(0.020) 0.015 0.012

Locus 17661 :

2 alleles

99.2% missing data

2	(0.448) 0.450 0.450

1	(0.552) 0.550 0.550

Locus 17662 :

2 alleles

99.2% missing data

4	(0.479) 0.485 0.476

3	(0.521) 0.515 0.524

Locus 17663 :

2 alleles

98.8% missing data

4	(0.251) 0.247 0.254

2	(0.749) 0.753 0.746

Locus 17664 :

2 alleles

99.2% missing data

4	(0.331) 0.336 0.330

1	(0.669) 0.664 0.670

Locus 17665 :

2 alleles

98.8% missing data

2	(0.419) 0.422 0.422

1	(0.581) 0.578 0.578

Locus 17666 :

2 alleles

98.4% missing data

3	(0.583) 0.590 0.578

4	(0.417) 0.410 0.422

Locus 17667 :

2 alleles

98.8% missing data

3	(0.639) 0.630 0.640

2	(0.361) 0.370 0.360

Locus 17668 :

2 alleles

96.1% missing data

1	(0.701) 0.722 0.694

4	(0.299) 0.278 0.306

Locus 17669 :

2 alleles

99.2% missing data

4	(0.700) 0.704 0.698

2	(0.300) 0.296 0.302

Locus 17670 :

2 alleles

99.2% missing data

4	(0.697) 0.688 0.698

3	(0.303) 0.312 0.302

Locus 17671 :

2 alleles

99.2% missing data

2	(0.678) 0.685 0.668

4	(0.322) 0.315 0.332

Locus 17672 :

3 alleles

8.6% missing data

4	(0.970) 0.960 0.991

1	(0.016) 0.022 0.005

2	(0.014) 0.018 0.004

Locus 17673 :

2 alleles

99.2% missing data

2	(0.288) 0.284 0.292

3	(0.712) 0.716 0.708

Locus 17674 :

2 alleles

99.2% missing data

4	(0.335) 0.335 0.340

3	(0.665) 0.665 0.660

Locus 17675 :

2 alleles

99.2% missing data

1	(0.586) 0.585 0.580

4	(0.414) 0.415 0.420

Locus 17676 :

2 alleles

99.2% missing data

1	(0.680) 0.681 0.682

3	(0.320) 0.319 0.318

Locus 17677 :

2 alleles

99.2% missing data

1	(0.624) 0.612 0.626

3	(0.376) 0.388 0.374

Locus 17678 :

2 alleles

0.0% missing data

1	(0.986) 0.989 0.992

2	(0.014) 0.011 0.008

Locus 17679 :

2 alleles

99.2% missing data

3	(0.472) 0.466 0.474

4	(0.528) 0.534 0.526

Locus 17680 :

2 alleles

99.2% missing data

2	(0.447) 0.439 0.451

4	(0.553) 0.561 0.549

Locus 17681 :

2 alleles

99.2% missing data

1	(0.400) 0.402 0.404

4	(0.600) 0.598 0.596

Locus 17682 :

2 alleles

99.2% missing data

2	(0.548) 0.547 0.548

4	(0.452) 0.453 0.452

Locus 17683 :

2 alleles

99.2% missing data

1	(0.497) 0.498 0.497

3	(0.503) 0.502 0.503

Locus 17684 :

2 alleles

99.2% missing data

2	(0.474) 0.473 0.472

3	(0.526) 0.527 0.528

Locus 17685 :

2 alleles

99.2% missing data

4	(0.507) 0.503 0.512

1	(0.493) 0.497 0.488

Locus 17686 :

2 alleles

99.2% missing data

4	(0.630) 0.630 0.633

3	(0.370) 0.370 0.367

Locus 17687 :

2 alleles

91.4% missing data

4	(0.860) 0.883 0.849

1	(0.140) 0.117 0.151

Locus 17688 :

2 alleles

99.2% missing data

4	(0.358) 0.359 0.361

1	(0.642) 0.641 0.639

Locus 17689 :

2 alleles

95.7% missing data

2	(0.273) 0.263 0.275

4	(0.727) 0.737 0.725

Locus 17690 :

2 alleles

74.6% missing data

3	(0.969) 0.962 0.981

1	(0.031) 0.038 0.019

Locus 17691 :

2 alleles

99.2% missing data

3	(0.455) 0.461 0.452

4	(0.545) 0.539 0.548

Locus 17692 :

2 alleles

99.2% missing data

3	(0.562) 0.569 0.567

4	(0.438) 0.431 0.433

Locus 17693 :

2 alleles

99.2% missing data

1	(0.607) 0.614 0.600

4	(0.393) 0.386 0.400

Locus 17694 :

2 alleles

99.2% missing data

2	(0.183) 0.189 0.180

4	(0.817) 0.811 0.820

Locus 17695 :

2 alleles

98.8% missing data

4	(0.452) 0.450 0.449

3	(0.548) 0.550 0.551

Locus 17696 :

2 alleles

99.2% missing data

3	(0.424) 0.420 0.429

2	(0.576) 0.580 0.571

Locus 17697 :

3 alleles

98.8% missing data

4	(0.238) 0.238 0.238

3	(0.360) 0.358 0.363

2	(0.402) 0.404 0.399

Locus 17698 :

2 alleles

99.2% missing data

4	(0.576) 0.577 0.573

3	(0.424) 0.423 0.427

Locus 17699 :

3 alleles

90.6% missing data

4	(0.852) 0.872 0.842

2	(0.079) 0.063 0.091

1	(0.069) 0.065 0.068

Locus 17700 :

2 alleles

90.2% missing data

4	(0.927) 0.928 0.933

3	(0.073) 0.072 0.067

Locus 17701 :

2 alleles

98.0% missing data

1	(0.794) 0.795 0.798

2	(0.206) 0.205 0.202

Locus 17702 :

2 alleles

97.7% missing data

2	(0.705) 0.704 0.710

1	(0.295) 0.296 0.290

Locus 17703 :

2 alleles

98.0% missing data

3	(0.411) 0.412 0.422

1	(0.589) 0.588 0.578

Locus 17704 :

2 alleles

99.2% missing data

1	(0.430) 0.427 0.435

4	(0.570) 0.573 0.565

Locus 17705 :

2 alleles

99.2% missing data

3	(0.441) 0.439 0.447

4	(0.559) 0.561 0.553

Locus 17706 :

2 alleles

97.3% missing data

2	(0.754) 0.742 0.768

1	(0.246) 0.258 0.232

Locus 17707 :

2 alleles

99.2% missing data

4	(0.622) 0.617 0.629

3	(0.378) 0.383 0.371

Locus 17708 :

2 alleles

97.7% missing data

3	(0.355) 0.354 0.346

1	(0.645) 0.646 0.654

Locus 17709 :

2 alleles

98.0% missing data

2	(0.296) 0.289 0.298

1	(0.704) 0.711 0.702

Locus 17710 :

2 alleles

99.2% missing data

3	(0.606) 0.592 0.619

2	(0.394) 0.408 0.381

Locus 17711 :

2 alleles

98.8% missing data

3	(0.262) 0.265 0.262

4	(0.738) 0.735 0.738

Locus 17712 :

3 alleles

95.7% missing data

3	(0.280) 0.267 0.291

4	(0.590) 0.611 0.572

2	(0.130) 0.123 0.137

Locus 17713 :

2 alleles

99.2% missing data

2	(0.369) 0.363 0.373

1	(0.631) 0.637 0.627

Locus 17714 :

2 alleles

99.2% missing data

1	(0.145) 0.153 0.139

3	(0.855) 0.847 0.861

Locus 17715 :

2 alleles

99.2% missing data

4	(0.443) 0.443 0.444

1	(0.557) 0.557 0.556

Locus 17716 :

2 alleles

99.2% missing data

2	(0.761) 0.758 0.761

4	(0.239) 0.242 0.239

Locus 17717 :

2 alleles

96.5% missing data

1	(0.628) 0.611 0.647

2	(0.372) 0.389 0.353

Locus 17718 :

2 alleles

0.4% missing data

1	(0.988) 0.985 0.996

4	(0.012) 0.015 0.004

Locus 17719 :

2 alleles

99.2% missing data

4	(0.216) 0.215 0.216

2	(0.784) 0.785 0.784

Locus 17720 :

2 alleles

98.8% missing data

1	(0.412) 0.415 0.408

2	(0.588) 0.585 0.592

Locus 17721 :

2 alleles

67.6% missing data

3	(0.964) 0.967 0.969

2	(0.036) 0.033 0.031

Locus 17722 :

2 alleles

99.2% missing data

3	(0.549) 0.547 0.552

2	(0.451) 0.453 0.448

Locus 17723 :

2 alleles

99.2% missing data

4	(0.145) 0.152 0.143

3	(0.855) 0.848 0.857

Locus 17724 :

2 alleles

99.2% missing data

3	(0.468) 0.468 0.464

4	(0.532) 0.532 0.536

Locus 17725 :

2 alleles

99.2% missing data

2	(0.599) 0.596 0.605

1	(0.401) 0.404 0.395

Locus 17726 :

2 alleles

99.2% missing data

2	(0.714) 0.718 0.708

3	(0.286) 0.282 0.292

Locus 17727 :

2 alleles

99.2% missing data

4	(0.397) 0.395 0.396

3	(0.603) 0.605 0.604

Locus 17728 :

2 alleles

99.2% missing data

3	(0.491) 0.494 0.491

2	(0.509) 0.506 0.509

Locus 17729 :

3 alleles

97.3% missing data

2	(0.617) 0.632 0.611

4	(0.223) 0.210 0.232

3	(0.160) 0.159 0.157

Locus 17730 :

2 alleles

99.2% missing data

4	(0.687) 0.687 0.688

2	(0.313) 0.313 0.312

Locus 17731 :

2 alleles

98.8% missing data

3	(0.551) 0.556 0.555

4	(0.449) 0.444 0.445

Locus 17732 :

2 alleles

99.2% missing data

1	(0.513) 0.519 0.508

3	(0.487) 0.481 0.492

Locus 17733 :

2 alleles

99.2% missing data

3	(0.456) 0.449 0.460

4	(0.544) 0.551 0.540

Locus 17734 :

2 alleles

99.2% missing data

4	(0.645) 0.640 0.647

1	(0.355) 0.360 0.353

Locus 17735 :

3 alleles

98.4% missing data

3	(0.264) 0.259 0.273

2	(0.299) 0.302 0.292

4	(0.437) 0.439 0.435

Locus 17736 :

2 alleles

99.2% missing data

1	(0.627) 0.619 0.630

4	(0.373) 0.381 0.370

Locus 17737 :

2 alleles

98.8% missing data

4	(0.465) 0.465 0.465

1	(0.535) 0.535 0.535

Locus 17738 :

2 alleles

99.2% missing data

4	(0.493) 0.489 0.493

1	(0.507) 0.511 0.507

Locus 17739 :

2 alleles

99.2% missing data

1	(0.730) 0.734 0.720

4	(0.270) 0.266 0.280

Locus 17740 :

2 alleles

97.7% missing data

4	(0.259) 0.261 0.261

3	(0.741) 0.739 0.739

Locus 17741 :

2 alleles

98.4% missing data

4	(0.438) 0.450 0.438

1	(0.562) 0.550 0.562

Locus 17742 :

2 alleles

97.3% missing data

1	(0.220) 0.212 0.225

3	(0.780) 0.788 0.775

Locus 17743 :

2 alleles

99.2% missing data

2	(0.496) 0.494 0.495

3	(0.504) 0.506 0.505

Locus 17744 :

2 alleles

98.4% missing data

3	(0.747) 0.746 0.745

1	(0.253) 0.254 0.255

Locus 17745 :

2 alleles

99.2% missing data

2	(0.559) 0.557 0.561

1	(0.441) 0.443 0.439

Locus 17746 :

2 alleles

98.8% missing data

1	(0.672) 0.669 0.677

4	(0.328) 0.331 0.323

Locus 17747 :

2 alleles

99.2% missing data

1	(0.293) 0.295 0.288

2	(0.707) 0.705 0.712

Locus 17748 :

2 alleles

99.2% missing data

2	(0.513) 0.506 0.519

1	(0.487) 0.494 0.481

Locus 17749 :

2 alleles

99.2% missing data

1	(0.690) 0.691 0.690

4	(0.310) 0.309 0.310

Locus 17750 :

2 alleles

99.2% missing data

1	(0.411) 0.408 0.416

2	(0.589) 0.592 0.584

Locus 17751 :

4 alleles

98.0% missing data

4	(0.197) 0.197 0.197

3	(0.192) 0.194 0.189

1	(0.187) 0.176 0.191

2	(0.424) 0.433 0.422

Locus 17752 :

2 alleles

99.2% missing data

1	(0.218) 0.213 0.226

4	(0.782) 0.787 0.774

Locus 17753 :

2 alleles

89.5% missing data

1	(0.716) 0.762 0.677

4	(0.284) 0.238 0.323

Locus 17754 :

2 alleles

99.2% missing data

2	(0.612) 0.609 0.606

1	(0.388) 0.391 0.394

Locus 17755 :

2 alleles

99.2% missing data

4	(0.329) 0.330 0.328

1	(0.671) 0.670 0.672

Locus 17756 :

2 alleles

98.8% missing data

2	(0.675) 0.671 0.674

1	(0.325) 0.329 0.326

Locus 17757 :

2 alleles

99.2% missing data

4	(0.376) 0.370 0.382

3	(0.624) 0.630 0.618

Locus 17758 :

2 alleles

0.0% missing data

1	(0.989) 0.992 0.994

4	(0.011) 0.008 0.006

Locus 17759 :

2 alleles

92.6% missing data

1	(0.912) 0.915 0.917

2	(0.088) 0.085 0.083

Locus 17760 :

2 alleles

98.8% missing data

2	(0.494) 0.490 0.504

1	(0.506) 0.510 0.496

Locus 17761 :

2 alleles

99.2% missing data

4	(0.622) 0.625 0.619

1	(0.378) 0.375 0.381

Locus 17762 :

2 alleles

99.2% missing data

3	(0.686) 0.677 0.693

1	(0.314) 0.323 0.307

Locus 17763 :

2 alleles

99.2% missing data

1	(0.416) 0.411 0.419

3	(0.584) 0.589 0.581

Locus 17764 :

2 alleles

97.7% missing data

4	(0.882) 0.880 0.883

1	(0.118) 0.120 0.117

Locus 17765 :

3 alleles

98.4% missing data

3	(0.331) 0.339 0.327

1	(0.423) 0.421 0.417

4	(0.246) 0.240 0.255

Locus 17766 :

2 alleles

99.2% missing data

4	(0.599) 0.602 0.600

1	(0.401) 0.398 0.400

Locus 17767 :

2 alleles

97.7% missing data

3	(0.716) 0.723 0.717

2	(0.284) 0.277 0.283

Locus 17768 :

2 alleles

98.8% missing data

3	(0.516) 0.502 0.521

4	(0.484) 0.498 0.479

Locus 17769 :

2 alleles

98.8% missing data

1	(0.286) 0.281 0.291

3	(0.714) 0.719 0.709

Locus 17770 :

2 alleles

96.5% missing data

1	(0.780) 0.794 0.772

4	(0.220) 0.206 0.228

Locus 17771 :

2 alleles

99.2% missing data

3	(0.532) 0.530 0.536

1	(0.468) 0.470 0.464

Locus 17772 :

2 alleles

92.6% missing data

4	(0.881) 0.871 0.898

1	(0.119) 0.129 0.102

Locus 17773 :

2 alleles

98.8% missing data

2	(0.790) 0.794 0.787

1	(0.210) 0.206 0.213

Locus 17774 :

2 alleles

99.2% missing data

4	(0.771) 0.766 0.773

2	(0.229) 0.234 0.227

Locus 17775 :

2 alleles

98.8% missing data

3	(0.717) 0.721 0.714

1	(0.283) 0.279 0.286

Locus 17776 :

2 alleles

8.2% missing data

3	(0.984) 0.978 0.995

4	(0.016) 0.022 0.005

Locus 17777 :

2 alleles

0.8% missing data

3	(0.922) 0.770 0.976

1	(0.078) 0.230 0.024

Locus 17778 :

2 alleles

99.2% missing data

4	(0.466) 0.462 0.467

1	(0.534) 0.538 0.533

Locus 17779 :

2 alleles

99.2% missing data

1	(0.585) 0.584 0.591

3	(0.415) 0.416 0.409

Locus 17780 :

2 alleles

98.8% missing data

4	(0.432) 0.425 0.434

1	(0.568) 0.575 0.566

Locus 17781 :

2 alleles

99.2% missing data

3	(0.502) 0.500 0.501

1	(0.498) 0.500 0.499

Locus 17782 :

2 alleles

99.2% missing data

1	(0.325) 0.326 0.325

2	(0.675) 0.674 0.675

Locus 17783 :

2 alleles

99.2% missing data

4	(0.356) 0.357 0.359

2	(0.644) 0.643 0.641

Locus 17784 :

2 alleles

97.7% missing data

2	(0.385) 0.385 0.374

4	(0.615) 0.615 0.626

Locus 17785 :

2 alleles

97.3% missing data

4	(0.792) 0.806 0.789

1	(0.208) 0.194 0.211

Locus 17786 :

2 alleles

99.2% missing data

4	(0.700) 0.699 0.692

3	(0.300) 0.301 0.308

Locus 17787 :

2 alleles

8.2% missing data

2	(0.987) 0.978 0.996

1	(0.013) 0.022 0.004

Locus 17788 :

2 alleles

98.0% missing data

1	(0.726) 0.720 0.738

3	(0.274) 0.280 0.262

Locus 17789 :

2 alleles

99.2% missing data

3	(0.701) 0.697 0.703

1	(0.299) 0.303 0.297

Locus 17790 :

2 alleles

99.2% missing data

4	(0.472) 0.477 0.472

3	(0.528) 0.523 0.528

Locus 17791 :

2 alleles

99.2% missing data

3	(0.574) 0.578 0.573

2	(0.426) 0.422 0.427

Locus 17792 :

2 alleles

99.2% missing data

4	(0.708) 0.706 0.699

2	(0.292) 0.294 0.301

Locus 17793 :

2 alleles

97.7% missing data

2	(0.692) 0.681 0.708

3	(0.308) 0.319 0.292

Locus 17794 :

2 alleles

99.2% missing data

3	(0.420) 0.425 0.415

4	(0.580) 0.575 0.585

Locus 17795 :

2 alleles

99.2% missing data

1	(0.294) 0.296 0.288

2	(0.706) 0.704 0.712

Locus 17796 :

2 alleles

98.8% missing data

4	(0.429) 0.421 0.436

2	(0.571) 0.579 0.564

Locus 17797 :

2 alleles

99.2% missing data

1	(0.491) 0.497 0.491

2	(0.509) 0.503 0.509

Locus 17798 :

2 alleles

99.2% missing data

3	(0.663) 0.667 0.657

1	(0.337) 0.333 0.343

Locus 17799 :

2 alleles

99.2% missing data

3	(0.509) 0.507 0.511

2	(0.491) 0.493 0.489

Locus 17800 :

2 alleles

96.1% missing data

1	(0.846) 0.846 0.851

3	(0.154) 0.154 0.149

Locus 17801 :

2 alleles

99.2% missing data

4	(0.588) 0.597 0.588

1	(0.412) 0.403 0.412

Locus 17802 :

2 alleles

99.2% missing data

1	(0.648) 0.654 0.648

3	(0.352) 0.346 0.352

Locus 17803 :

3 alleles

98.8% missing data

1	(0.408) 0.407 0.401

4	(0.329) 0.335 0.329

3	(0.263) 0.257 0.269

Locus 17804 :

2 alleles

99.2% missing data

3	(0.363) 0.362 0.363

4	(0.637) 0.638 0.637

Locus 17805 :

2 alleles

99.2% missing data

1	(0.623) 0.619 0.625

2	(0.377) 0.381 0.375

Locus 17806 :

2 alleles

99.2% missing data

1	(0.258) 0.257 0.259

3	(0.742) 0.743 0.741

Locus 17807 :

2 alleles

8.2% missing data

3	(0.987) 0.978 0.996

4	(0.013) 0.022 0.004

Locus 17808 :

2 alleles

99.2% missing data

1	(0.384) 0.384 0.391

4	(0.616) 0.616 0.609

Locus 17809 :

3 alleles

98.8% missing data

4	(0.473) 0.466 0.478

2	(0.283) 0.282 0.285

3	(0.244) 0.251 0.237

Locus 17810 :

2 alleles

98.0% missing data

2	(0.796) 0.790 0.803

1	(0.204) 0.210 0.197

Locus 17811 :

2 alleles

91.4% missing data

4	(0.947) 0.943 0.956

1	(0.053) 0.057 0.044

Locus 17812 :

2 alleles

99.2% missing data

3	(0.559) 0.563 0.555

1	(0.441) 0.437 0.445

Locus 17813 :

2 alleles

96.9% missing data

3	(0.855) 0.849 0.865

1	(0.145) 0.151 0.135

Locus 17814 :

2 alleles

93.8% missing data

4	(0.832) 0.833 0.843

3	(0.168) 0.167 0.157

Locus 17815 :

2 alleles

98.8% missing data

1	(0.596) 0.599 0.600

3	(0.404) 0.401 0.400

Locus 17816 :

2 alleles

99.2% missing data

2	(0.614) 0.615 0.621

3	(0.386) 0.385 0.379

Locus 17817 :

2 alleles

99.2% missing data

1	(0.215) 0.214 0.223

4	(0.785) 0.786 0.777

Locus 17818 :

2 alleles

99.2% missing data

3	(0.274) 0.274 0.279

1	(0.726) 0.726 0.721

Locus 17819 :

2 alleles

99.2% missing data

4	(0.279) 0.277 0.290

3	(0.721) 0.723 0.710

Locus 17820 :

2 alleles

99.2% missing data

1	(0.269) 0.269 0.270

3	(0.731) 0.731 0.730

Locus 17821 :

2 alleles

98.4% missing data

1	(0.634) 0.638 0.632

2	(0.366) 0.362 0.368

Locus 17822 :

2 alleles

99.2% missing data

4	(0.502) 0.503 0.509

1	(0.498) 0.497 0.491

Locus 17823 :

2 alleles

99.2% missing data

1	(0.574) 0.575 0.583

3	(0.426) 0.425 0.417

Locus 17824 :

2 alleles

99.2% missing data

1	(0.377) 0.375 0.378

3	(0.623) 0.625 0.622

Locus 17825 :

2 alleles

98.8% missing data

2	(0.718) 0.715 0.723

4	(0.282) 0.285 0.277

Locus 17826 :

2 alleles

99.2% missing data

4	(0.703) 0.698 0.705

2	(0.297) 0.302 0.295

Locus 17827 :

2 alleles

85.9% missing data

3	(0.936) 0.939 0.945

1	(0.064) 0.061 0.055

Locus 17828 :

2 alleles

95.3% missing data

3	(0.868) 0.865 0.874

2	(0.132) 0.135 0.126

Locus 17829 :

2 alleles

97.7% missing data

4	(0.787) 0.781 0.798

3	(0.213) 0.219 0.202

Locus 17830 :

3 alleles

98.8% missing data

4	(0.409) 0.406 0.414

1	(0.317) 0.317 0.311

3	(0.274) 0.277 0.275

Locus 17831 :

2 alleles

99.2% missing data

3	(0.707) 0.700 0.708

1	(0.293) 0.300 0.292

Locus 17832 :

2 alleles

99.2% missing data

4	(0.299) 0.300 0.296

1	(0.701) 0.700 0.704

Locus 17833 :

2 alleles

96.5% missing data

4	(0.849) 0.861 0.846

2	(0.151) 0.139 0.154

Locus 17834 :

2 alleles

99.2% missing data

3	(0.498) 0.490 0.506

1	(0.502) 0.510 0.494

Locus 17835 :

2 alleles

99.2% missing data

3	(0.615) 0.603 0.618

1	(0.385) 0.397 0.382

Locus 17836 :

2 alleles

97.3% missing data

2	(0.740) 0.748 0.743

4	(0.260) 0.252 0.257

Locus 17837 :

2 alleles

98.8% missing data

3	(0.532) 0.528 0.534

1	(0.468) 0.472 0.466

Locus 17838 :

2 alleles

99.2% missing data

3	(0.260) 0.258 0.265

1	(0.740) 0.742 0.735

Locus 17839 :

2 alleles

99.2% missing data

1	(0.485) 0.487 0.491

4	(0.515) 0.513 0.509

Locus 17840 :

2 alleles

99.2% missing data

3	(0.520) 0.514 0.525

2	(0.480) 0.486 0.475

Locus 17841 :

2 alleles

99.2% missing data

3	(0.545) 0.552 0.539

2	(0.455) 0.448 0.461

Locus 17842 :

2 alleles

98.8% missing data

1	(0.587) 0.575 0.593

3	(0.413) 0.425 0.407

Locus 17843 :

2 alleles

99.2% missing data

4	(0.652) 0.648 0.654

1	(0.348) 0.352 0.346

Locus 17844 :

2 alleles

98.8% missing data

3	(0.647) 0.648 0.645

1	(0.353) 0.352 0.355

Locus 17845 :

2 alleles

99.2% missing data

3	(0.356) 0.362 0.359

2	(0.644) 0.638 0.641

Locus 17846 :

2 alleles

98.0% missing data

3	(0.670) 0.682 0.673

4	(0.330) 0.318 0.327

Locus 17847 :

3 alleles

98.8% missing data

4	(0.323) 0.315 0.324

3	(0.460) 0.460 0.465

2	(0.216) 0.225 0.211

Locus 17848 :

2 alleles

99.2% missing data

3	(0.862) 0.857 0.861

4	(0.138) 0.143 0.139

Locus 17849 :

2 alleles

99.2% missing data

1	(0.616) 0.614 0.617

4	(0.384) 0.386 0.383

Locus 17850 :

2 alleles

98.4% missing data

1	(0.517) 0.514 0.518

4	(0.483) 0.486 0.482

Locus 17851 :

2 alleles

99.2% missing data

2	(0.568) 0.565 0.567

1	(0.432) 0.435 0.433

Locus 17852 :

2 alleles

99.2% missing data

4	(0.761) 0.754 0.763

1	(0.239) 0.246 0.237

Locus 17853 :

2 alleles

99.2% missing data

3	(0.354) 0.362 0.350

1	(0.646) 0.638 0.650

Locus 17854 :

2 alleles

99.2% missing data

2	(0.572) 0.566 0.578

3	(0.428) 0.434 0.422

Locus 17855 :

2 alleles

98.8% missing data

1	(0.375) 0.381 0.374

4	(0.625) 0.619 0.626

Locus 17856 :

2 alleles

99.2% missing data

1	(0.568) 0.565 0.570

2	(0.432) 0.435 0.430

Locus 17857 :

2 alleles

99.2% missing data

1	(0.829) 0.832 0.829

2	(0.171) 0.168 0.171

Locus 17858 :

2 alleles

0.0% missing data

2	(0.987) 0.989 0.993

3	(0.013) 0.011 0.007

Locus 17859 :

2 alleles

99.2% missing data

4	(0.489) 0.488 0.492

2	(0.511) 0.512 0.508

Locus 17860 :

2 alleles

97.7% missing data

4	(0.737) 0.746 0.735

1	(0.263) 0.254 0.265

Locus 17861 :

2 alleles

98.8% missing data

3	(0.652) 0.649 0.659

1	(0.348) 0.351 0.341

Locus 17862 :

2 alleles

99.2% missing data

2	(0.497) 0.502 0.486

3	(0.503) 0.498 0.514

Locus 17863 :

2 alleles

99.2% missing data

3	(0.407) 0.409 0.402

1	(0.593) 0.591 0.598

Locus 17864 :

2 alleles

98.4% missing data

1	(0.755) 0.763 0.747

4	(0.245) 0.237 0.253

Locus 17865 :

2 alleles

99.2% missing data

4	(0.272) 0.268 0.279

1	(0.728) 0.732 0.721

Locus 17866 :

2 alleles

98.8% missing data

2	(0.755) 0.758 0.747

3	(0.245) 0.242 0.253

Locus 17867 :

2 alleles

98.8% missing data

1	(0.746) 0.751 0.741

4	(0.254) 0.249 0.259

Locus 17868 :

3 alleles

98.8% missing data

4	(0.195) 0.190 0.200

3	(0.398) 0.398 0.396

2	(0.407) 0.412 0.404

Locus 17869 :

2 alleles

98.8% missing data

4	(0.605) 0.599 0.606

1	(0.395) 0.401 0.394

Locus 17870 :

2 alleles

93.4% missing data

1	(0.865) 0.868 0.876

3	(0.135) 0.132 0.124

Locus 17871 :

2 alleles

99.2% missing data

2	(0.742) 0.740 0.744

4	(0.258) 0.260 0.256

Locus 17872 :

2 alleles

98.8% missing data

4	(0.759) 0.752 0.762

2	(0.241) 0.248 0.238

Locus 17873 :

2 alleles

99.2% missing data

1	(0.418) 0.421 0.425

4	(0.582) 0.579 0.575

Locus 17874 :

3 alleles

98.8% missing data

4	(0.181) 0.180 0.185

2	(0.350) 0.346 0.353

1	(0.469) 0.474 0.461

Locus 17875 :

2 alleles

96.1% missing data

4	(0.134) 0.125 0.136

2	(0.866) 0.875 0.864

Locus 17876 :

2 alleles

99.2% missing data

4	(0.355) 0.356 0.356

2	(0.645) 0.644 0.644

Locus 17877 :

2 alleles

99.2% missing data

4	(0.506) 0.504 0.508

3	(0.494) 0.496 0.492

Locus 17878 :

2 alleles

91.0% missing data

4	(0.907) 0.918 0.910

1	(0.093) 0.082 0.090

Locus 17879 :

2 alleles

99.2% missing data

1	(0.639) 0.629 0.642

4	(0.361) 0.371 0.358

Locus 17880 :

2 alleles

84.4% missing data

4	(0.960) 0.956 0.969

3	(0.040) 0.044 0.031

Locus 17881 :

2 alleles

0.4% missing data

1	(0.987) 0.991 0.993

2	(0.013) 0.009 0.007

Locus 17882 :

3 alleles

98.8% missing data

1	(0.189) 0.186 0.197

4	(0.416) 0.417 0.417

3	(0.394) 0.397 0.386

Locus 17883 :

2 alleles

99.2% missing data

3	(0.509) 0.521 0.508

1	(0.491) 0.479 0.492

Locus 17884 :

2 alleles

97.7% missing data

4	(0.806) 0.802 0.816

3	(0.194) 0.198 0.184

Locus 17885 :

2 alleles

99.2% missing data

3	(0.185) 0.186 0.188

4	(0.815) 0.814 0.812

Locus 17886 :

2 alleles

91.0% missing data

1	(0.898) 0.916 0.891

3	(0.102) 0.084 0.109

Locus 17887 :

2 alleles

76.6% missing data

4	(0.964) 0.964 0.972

3	(0.036) 0.036 0.028

Locus 17888 :

2 alleles

98.8% missing data

2	(0.169) 0.163 0.176

1	(0.831) 0.837 0.824

Locus 17889 :

2 alleles

93.0% missing data

3	(0.936) 0.939 0.937

4	(0.064) 0.061 0.063

Locus 17890 :

2 alleles

97.7% missing data

3	(0.845) 0.851 0.845

1	(0.155) 0.149 0.155

Locus 17891 :

2 alleles

7.4% missing data

2	(0.978) 0.953 0.993

1	(0.022) 0.047 0.007

Locus 17892 :

2 alleles

99.2% missing data

1	(0.648) 0.648 0.650

4	(0.352) 0.352 0.350

Locus 17893 :

2 alleles

99.2% missing data

3	(0.655) 0.655 0.660

4	(0.345) 0.345 0.340

Locus 17894 :

2 alleles

99.2% missing data

3	(0.212) 0.211 0.221

1	(0.788) 0.789 0.779

Locus 17895 :

2 alleles

99.2% missing data

2	(0.599) 0.598 0.600

4	(0.401) 0.402 0.400

Locus 17896 :

2 alleles

99.2% missing data

3	(0.638) 0.627 0.645

2	(0.362) 0.373 0.355

Locus 17897 :

2 alleles

99.2% missing data

1	(0.623) 0.617 0.623

4	(0.377) 0.383 0.377

Locus 17898 :

2 alleles

98.8% missing data

3	(0.480) 0.472 0.486

4	(0.520) 0.528 0.514

Locus 17899 :

3 alleles

98.0% missing data

4	(0.454) 0.457 0.464

3	(0.288) 0.285 0.291

1	(0.257) 0.258 0.245

Locus 17900 :

2 alleles

96.1% missing data

1	(0.275) 0.263 0.271

3	(0.725) 0.737 0.729

Locus 17901 :

2 alleles

99.2% missing data

2	(0.423) 0.427 0.418

1	(0.577) 0.573 0.582

Locus 17902 :

2 alleles

99.2% missing data

4	(0.561) 0.555 0.567

3	(0.439) 0.445 0.433

Locus 17903 :

2 alleles

99.2% missing data

4	(0.655) 0.653 0.655

1	(0.345) 0.347 0.345

Locus 17904 :

2 alleles

99.2% missing data

2	(0.367) 0.365 0.365

3	(0.633) 0.635 0.635

Locus 17905 :

2 alleles

99.2% missing data

4	(0.425) 0.425 0.431

2	(0.575) 0.575 0.569

Locus 17906 :

2 alleles

99.2% missing data

2	(0.661) 0.655 0.671

3	(0.339) 0.345 0.329

Locus 17907 :

2 alleles

99.2% missing data

3	(0.717) 0.709 0.722

4	(0.283) 0.291 0.278

Locus 17908 :

2 alleles

93.0% missing data

4	(0.085) 0.088 0.079

3	(0.915) 0.912 0.921

Locus 17909 :

2 alleles

99.2% missing data

4	(0.524) 0.526 0.529

3	(0.476) 0.474 0.471

Locus 17910 :

2 alleles

97.7% missing data

3	(0.558) 0.545 0.569

1	(0.442) 0.455 0.431

Locus 17911 :

2 alleles

99.2% missing data

2	(0.291) 0.289 0.290

3	(0.709) 0.711 0.710

Locus 17912 :

2 alleles

99.2% missing data

3	(0.457) 0.455 0.463

2	(0.543) 0.545 0.537

Locus 17913 :

2 alleles

99.2% missing data

1	(0.716) 0.711 0.718

2	(0.284) 0.289 0.282

Locus 17914 :

3 alleles

98.8% missing data

3	(0.265) 0.260 0.266

1	(0.493) 0.501 0.487

4	(0.242) 0.239 0.248

Locus 17915 :

2 alleles

96.1% missing data

4	(0.819) 0.835 0.809

2	(0.181) 0.165 0.191

Locus 17916 :

2 alleles

97.7% missing data

2	(0.762) 0.763 0.771

1	(0.238) 0.237 0.229

Locus 17917 :

2 alleles

96.1% missing data

4	(0.139) 0.129 0.148

1	(0.861) 0.871 0.852

Locus 17918 :

2 alleles

96.5% missing data

1	(0.822) 0.826 0.824

4	(0.178) 0.174 0.176

Locus 17919 :

2 alleles

0.4% missing data

2	(0.987) 0.990 0.994

3	(0.013) 0.010 0.006

Locus 17920 :

2 alleles

99.2% missing data

4	(0.485) 0.478 0.488

2	(0.515) 0.522 0.512

Locus 17921 :

2 alleles

96.9% missing data

4	(0.819) 0.824 0.823

2	(0.181) 0.176 0.177

Locus 17922 :

2 alleles

99.2% missing data

3	(0.449) 0.452 0.448

2	(0.551) 0.548 0.552

Locus 17923 :

2 alleles

98.8% missing data

3	(0.620) 0.622 0.624

2	(0.380) 0.378 0.376

Locus 17924 :

2 alleles

99.2% missing data

4	(0.600) 0.596 0.606

3	(0.400) 0.404 0.394

Locus 17925 :

2 alleles

99.2% missing data

1	(0.324) 0.322 0.333

4	(0.676) 0.678 0.667

Locus 17926 :

2 alleles

96.5% missing data

3	(0.222) 0.232 0.210

1	(0.778) 0.768 0.790

Locus 17927 :

2 alleles

91.0% missing data

2	(0.923) 0.939 0.912

3	(0.077) 0.061 0.088

Locus 17928 :

2 alleles

98.8% missing data

2	(0.672) 0.674 0.673

3	(0.328) 0.326 0.327

Locus 17929 :

2 alleles

99.2% missing data

4	(0.743) 0.742 0.738

1	(0.257) 0.258 0.262

Locus 17930 :

2 alleles

99.2% missing data

1	(0.391) 0.385 0.396

4	(0.609) 0.615 0.604

Locus 17931 :

2 alleles

93.8% missing data

1	(0.853) 0.852 0.865

2	(0.147) 0.148 0.135

Locus 17932 :

2 alleles

99.2% missing data

4	(0.628) 0.632 0.627

1	(0.372) 0.368 0.373

Locus 17933 :

2 alleles

99.2% missing data

3	(0.525) 0.519 0.537

1	(0.475) 0.481 0.463

Locus 17934 :

2 alleles

98.0% missing data

1	(0.640) 0.640 0.642

3	(0.360) 0.360 0.358

Locus 17935 :

2 alleles

99.2% missing data

4	(0.328) 0.321 0.331

1	(0.672) 0.679 0.669

Locus 17936 :

2 alleles

93.0% missing data

3	(0.878) 0.877 0.896

2	(0.122) 0.123 0.104

Locus 17937 :

2 alleles

0.4% missing data

2	(0.989) 0.984 0.997

3	(0.011) 0.016 0.003

Locus 17938 :

2 alleles

98.0% missing data

4	(0.776) 0.784 0.772

1	(0.224) 0.216 0.228

Locus 17939 :

2 alleles

99.2% missing data

2	(0.628) 0.623 0.632

3	(0.372) 0.377 0.368

Locus 17940 :

2 alleles

99.2% missing data

3	(0.177) 0.175 0.182

4	(0.823) 0.825 0.818

Locus 17941 :

2 alleles

96.1% missing data

4	(0.170) 0.163 0.172

1	(0.830) 0.837 0.828

Locus 17942 :

2 alleles

98.8% missing data

3	(0.625) 0.620 0.633

1	(0.375) 0.380 0.367

Locus 17943 :

2 alleles

99.2% missing data

3	(0.652) 0.652 0.653

1	(0.348) 0.348 0.347

Locus 17944 :

2 alleles

99.2% missing data

2	(0.448) 0.442 0.446

1	(0.552) 0.558 0.554

Locus 17945 :

2 alleles

99.2% missing data

3	(0.439) 0.439 0.441

1	(0.561) 0.561 0.559

Locus 17946 :

2 alleles

98.4% missing data

1	(0.698) 0.690 0.708

4	(0.302) 0.310 0.292

Locus 17947 :

2 alleles

99.2% missing data

4	(0.145) 0.145 0.147

2	(0.855) 0.855 0.853

Locus 17948 :

2 alleles

99.2% missing data

4	(0.655) 0.654 0.654

2	(0.345) 0.346 0.346

Locus 17949 :

2 alleles

90.6% missing data

4	(0.939) 0.944 0.938

1	(0.061) 0.056 0.062

Locus 17950 :

2 alleles

98.4% missing data

4	(0.648) 0.641 0.643

3	(0.352) 0.359 0.357

Locus 17951 :

2 alleles

99.2% missing data

2	(0.689) 0.683 0.689

1	(0.311) 0.317 0.311

Locus 17952 :

2 alleles

99.2% missing data

1	(0.570) 0.570 0.566

2	(0.430) 0.430 0.434

Locus 17953 :

2 alleles

96.1% missing data

2	(0.130) 0.120 0.132

1	(0.870) 0.880 0.868

Locus 17954 :

2 alleles

99.2% missing data

3	(0.311) 0.305 0.318

2	(0.689) 0.695 0.682

Locus 17955 :

2 alleles

99.2% missing data

2	(0.646) 0.645 0.640

3	(0.354) 0.355 0.360

Locus 17956 :

2 alleles

99.2% missing data

1	(0.537) 0.546 0.536

2	(0.463) 0.454 0.464

Locus 17957 :

2 alleles

98.8% missing data

1	(0.677) 0.677 0.675

2	(0.323) 0.323 0.325

Locus 17958 :

3 alleles

98.4% missing data

3	(0.214) 0.209 0.221

1	(0.634) 0.634 0.628

2	(0.152) 0.157 0.151

Locus 17959 :

2 alleles

98.4% missing data

4	(0.346) 0.337 0.346

2	(0.654) 0.663 0.654

Locus 17960 :

2 alleles

99.2% missing data

4	(0.358) 0.357 0.361

3	(0.642) 0.643 0.639

Locus 17961 :

2 alleles

99.2% missing data

3	(0.496) 0.491 0.500

2	(0.504) 0.509 0.500

Locus 17962 :

2 alleles

99.2% missing data

4	(0.500) 0.491 0.502

2	(0.500) 0.509 0.498

Locus 17963 :

2 alleles

99.2% missing data

1	(0.311) 0.306 0.319

4	(0.689) 0.694 0.681

Locus 17964 :

2 alleles

99.2% missing data

1	(0.655) 0.650 0.657

2	(0.345) 0.350 0.343

Locus 17965 :

2 alleles

98.8% missing data

2	(0.747) 0.742 0.749

4	(0.253) 0.258 0.251

Locus 17966 :

2 alleles

93.0% missing data

4	(0.916) 0.919 0.915

3	(0.084) 0.081 0.085

Locus 17967 :

2 alleles

99.2% missing data

2	(0.443) 0.440 0.453

4	(0.557) 0.560 0.547

Locus 17968 :

2 alleles

99.2% missing data

3	(0.218) 0.224 0.215

1	(0.782) 0.776 0.785

Locus 17969 :

2 alleles

98.0% missing data

2	(0.251) 0.236 0.261

3	(0.749) 0.764 0.739

Locus 17970 :

2 alleles

99.2% missing data

4	(0.357) 0.359 0.354

2	(0.643) 0.641 0.646

Locus 17971 :

2 alleles

98.8% missing data

1	(0.763) 0.766 0.763

4	(0.237) 0.234 0.237

Locus 17972 :

2 alleles

99.2% missing data

2	(0.416) 0.417 0.417

4	(0.584) 0.583 0.583

Locus 17973 :

3 alleles

98.4% missing data

4	(0.265) 0.262 0.275

3	(0.190) 0.188 0.192

2	(0.545) 0.550 0.532

Locus 17974 :

3 alleles

98.4% missing data

1	(0.370) 0.369 0.375

3	(0.248) 0.253 0.238

4	(0.382) 0.378 0.387

Locus 17975 :

2 alleles

99.2% missing data

1	(0.583) 0.574 0.595

2	(0.417) 0.426 0.405

Locus 17976 :

2 alleles

98.8% missing data

3	(0.755) 0.750 0.757

4	(0.245) 0.250 0.243

Locus 17977 :

2 alleles

91.4% missing data

2	(0.921) 0.919 0.929

4	(0.079) 0.081 0.071

Locus 17978 :

2 alleles

99.2% missing data

2	(0.620) 0.619 0.615

4	(0.380) 0.381 0.385

Locus 17979 :

2 alleles

99.2% missing data

2	(0.673) 0.675 0.664

1	(0.327) 0.325 0.336

Locus 17980 :

2 alleles

99.2% missing data

4	(0.316) 0.318 0.318

3	(0.684) 0.682 0.682

Locus 17981 :

2 alleles

0.0% missing data

2	(0.986) 0.990 0.993

1	(0.014) 0.010 0.007

Locus 17982 :

2 alleles

99.2% missing data

3	(0.385) 0.385 0.394

2	(0.615) 0.615 0.606

Locus 17983 :

2 alleles

99.2% missing data

3	(0.703) 0.698 0.711

2	(0.297) 0.302 0.289

Locus 17984 :

2 alleles

99.2% missing data

2	(0.543) 0.548 0.545

4	(0.457) 0.452 0.455

Locus 17985 :

2 alleles

99.2% missing data

2	(0.320) 0.314 0.326

3	(0.680) 0.686 0.674

Locus 17986 :

2 alleles

98.8% missing data

4	(0.680) 0.672 0.682

1	(0.320) 0.328 0.318

Locus 17987 :

2 alleles

99.2% missing data

4	(0.556) 0.553 0.559

2	(0.444) 0.447 0.441

Locus 17988 :

2 alleles

99.2% missing data

3	(0.741) 0.748 0.740

2	(0.259) 0.252 0.260

Locus 17989 :

2 alleles

99.2% missing data

2	(0.421) 0.423 0.416

3	(0.579) 0.577 0.584

Locus 17990 :

2 alleles

94.9% missing data

3	(0.853) 0.856 0.858

1	(0.147) 0.144 0.142

Locus 17991 :

2 alleles

99.2% missing data

2	(0.256) 0.254 0.264

4	(0.744) 0.746 0.736

Locus 17992 :

2 alleles

92.2% missing data

4	(0.855) 0.878 0.847

2	(0.145) 0.122 0.153

Locus 17993 :

2 alleles

99.2% missing data

2	(0.634) 0.627 0.639

4	(0.366) 0.373 0.361

Locus 17994 :

2 alleles

99.2% missing data

2	(0.455) 0.450 0.463

1	(0.545) 0.550 0.537

Locus 17995 :

2 alleles

97.7% missing data

4	(0.694) 0.695 0.697

1	(0.306) 0.305 0.303

Locus 17996 :

2 alleles

98.8% missing data

3	(0.172) 0.174 0.171

4	(0.828) 0.826 0.829

Locus 17997 :

2 alleles

96.5% missing data

4	(0.867) 0.878 0.862

2	(0.133) 0.122 0.138

Locus 17998 :

2 alleles

98.8% missing data

4	(0.762) 0.766 0.761

1	(0.238) 0.234 0.239

Locus 17999 :

2 alleles

99.2% missing data

1	(0.573) 0.564 0.579

2	(0.427) 0.436 0.421

Locus 18000 :

3 alleles

98.8% missing data

4	(0.326) 0.324 0.330

3	(0.313) 0.315 0.311

2	(0.362) 0.361 0.359

Locus 18001 :

2 alleles

95.7% missing data

3	(0.764) 0.766 0.765

4	(0.236) 0.234 0.235

Locus 18002 :

2 alleles

98.8% missing data

2	(0.328) 0.328 0.327

3	(0.672) 0.672 0.673

Locus 18003 :

2 alleles

98.8% missing data

3	(0.340) 0.333 0.343

4	(0.660) 0.667 0.657

Locus 18004 :

2 alleles

99.2% missing data

3	(0.324) 0.330 0.320

1	(0.676) 0.670 0.680

Locus 18005 :

2 alleles

98.8% missing data

2	(0.527) 0.527 0.532

1	(0.473) 0.473 0.468

Locus 18006 :

2 alleles

84.4% missing data

2	(0.930) 0.930 0.938

3	(0.070) 0.070 0.062

Locus 18007 :

2 alleles

99.2% missing data

4	(0.648) 0.650 0.646

3	(0.352) 0.350 0.354

Locus 18008 :

2 alleles

95.3% missing data

3	(0.835) 0.829 0.850

2	(0.165) 0.171 0.150

Locus 18009 :

2 alleles

99.2% missing data

2	(0.446) 0.443 0.450

1	(0.554) 0.557 0.550

Locus 18010 :

2 alleles

99.2% missing data

2	(0.404) 0.399 0.403

4	(0.596) 0.601 0.597

Locus 18011 :

2 alleles

97.7% missing data

1	(0.201) 0.201 0.204

4	(0.799) 0.799 0.796

Locus 18012 :

2 alleles

99.2% missing data

2	(0.697) 0.690 0.702

4	(0.303) 0.310 0.298

Locus 18013 :

2 alleles

99.2% missing data

4	(0.547) 0.553 0.540

2	(0.453) 0.447 0.460

Locus 18014 :

2 alleles

99.2% missing data

3	(0.721) 0.712 0.725

2	(0.279) 0.288 0.275

Locus 18015 :

2 alleles

98.4% missing data

4	(0.831) 0.833 0.825

2	(0.169) 0.167 0.175

Locus 18016 :

2 alleles

99.2% missing data

3	(0.304) 0.302 0.308

1	(0.696) 0.698 0.692

Locus 18017 :

2 alleles

96.5% missing data

3	(0.761) 0.778 0.760

2	(0.239) 0.222 0.240

Locus 18018 :

2 alleles

99.2% missing data

4	(0.599) 0.596 0.600

2	(0.401) 0.404 0.400

Locus 18019 :

2 alleles

99.2% missing data

1	(0.639) 0.645 0.631

3	(0.361) 0.355 0.369

Locus 18020 :

2 alleles

99.2% missing data

4	(0.391) 0.391 0.398

1	(0.609) 0.609 0.602

Locus 18021 :

2 alleles

99.2% missing data

1	(0.339) 0.334 0.342

3	(0.661) 0.666 0.658

Locus 18022 :

2 alleles

99.2% missing data

4	(0.303) 0.299 0.303

3	(0.697) 0.701 0.697

Locus 18023 :

2 alleles

99.2% missing data

4	(0.330) 0.327 0.335

1	(0.670) 0.673 0.665

Locus 18024 :

2 alleles

99.2% missing data

2	(0.834) 0.826 0.836

1	(0.166) 0.174 0.164

Locus 18025 :

2 alleles

99.2% missing data

4	(0.360) 0.362 0.361

3	(0.640) 0.638 0.639

Locus 18026 :

2 alleles

96.1% missing data

1	(0.226) 0.207 0.246

3	(0.774) 0.793 0.754

Locus 18027 :

2 alleles

99.2% missing data

3	(0.442) 0.439 0.445

2	(0.558) 0.561 0.555

Locus 18028 :

2 alleles

0.4% missing data

4	(0.988) 0.991 0.993

1	(0.012) 0.009 0.007

Locus 18029 :

2 alleles

99.2% missing data

4	(0.331) 0.329 0.333

1	(0.669) 0.671 0.667

Locus 18030 :

2 alleles

99.2% missing data

1	(0.313) 0.308 0.319

2	(0.687) 0.692 0.681

Locus 18031 :

2 alleles

99.2% missing data

4	(0.650) 0.651 0.656

2	(0.350) 0.349 0.344

Locus 18032 :

2 alleles

8.6% missing data

4	(0.984) 0.976 0.995

3	(0.016) 0.024 0.005

Locus 18033 :

2 alleles

99.2% missing data

3	(0.593) 0.596 0.591

2	(0.407) 0.404 0.409

Locus 18034 :

2 alleles

99.2% missing data

3	(0.386) 0.378 0.396

1	(0.614) 0.622 0.604

Locus 18035 :

2 alleles

96.9% missing data

4	(0.825) 0.826 0.833

3	(0.175) 0.174 0.167

Locus 18036 :

2 alleles

99.2% missing data

3	(0.477) 0.480 0.473

4	(0.523) 0.520 0.527

Locus 18037 :

2 alleles

99.2% missing data

3	(0.430) 0.433 0.428

4	(0.570) 0.567 0.572

Locus 18038 :

2 alleles

98.4% missing data

4	(0.799) 0.805 0.795

1	(0.201) 0.195 0.205

Locus 18039 :

3 alleles

92.6% missing data

4	(0.795) 0.786 0.826

1	(0.121) 0.123 0.105

2	(0.083) 0.091 0.069

Locus 18040 :

2 alleles

99.2% missing data

1	(0.543) 0.539 0.547

4	(0.457) 0.461 0.453

Locus 18041 :

3 alleles

98.8% missing data

3	(0.355) 0.358 0.357

4	(0.371) 0.366 0.369

2	(0.274) 0.276 0.273

Locus 18042 :

2 alleles

99.2% missing data

4	(0.793) 0.794 0.788

2	(0.207) 0.206 0.212

Locus 18043 :

2 alleles

98.4% missing data

3	(0.264) 0.263 0.268

4	(0.736) 0.737 0.732

Locus 18044 :

2 alleles

99.2% missing data

3	(0.655) 0.658 0.646

4	(0.345) 0.342 0.354

Locus 18045 :

2 alleles

99.2% missing data

2	(0.557) 0.562 0.552

4	(0.443) 0.438 0.448

Locus 18046 :

2 alleles

98.8% missing data

2	(0.449) 0.453 0.446

4	(0.551) 0.547 0.554

Locus 18047 :

2 alleles

98.8% missing data

4	(0.340) 0.337 0.334

3	(0.660) 0.663 0.666

Locus 18048 :

2 alleles

99.2% missing data

3	(0.576) 0.571 0.583

1	(0.424) 0.429 0.417

Locus 18049 :

2 alleles

99.2% missing data

4	(0.384) 0.389 0.377

2	(0.616) 0.611 0.623

Locus 18050 :

2 alleles

99.2% missing data

3	(0.340) 0.339 0.345

1	(0.660) 0.661 0.655

Locus 18051 :

2 alleles

99.2% missing data

4	(0.472) 0.475 0.471

3	(0.528) 0.525 0.529

Locus 18052 :

2 alleles

99.2% missing data

4	(0.606) 0.603 0.605

1	(0.394) 0.397 0.395

Locus 18053 :

2 alleles

99.2% missing data

2	(0.470) 0.472 0.466

3	(0.530) 0.528 0.534

Locus 18054 :

2 alleles

99.2% missing data

2	(0.738) 0.732 0.737

1	(0.262) 0.268 0.263

Locus 18055 :

2 alleles

99.2% missing data

3	(0.397) 0.406 0.395

1	(0.603) 0.594 0.605

Locus 18056 :

2 alleles

99.2% missing data

1	(0.308) 0.308 0.308

2	(0.692) 0.692 0.692

Locus 18057 :

2 alleles

99.2% missing data

3	(0.869) 0.869 0.867

1	(0.131) 0.131 0.133

Locus 18058 :

2 alleles

99.2% missing data

2	(0.606) 0.601 0.607

3	(0.394) 0.399 0.393

Locus 18059 :

2 alleles

98.8% missing data

3	(0.415) 0.416 0.414

4	(0.585) 0.584 0.586

Locus 18060 :

2 alleles

99.2% missing data

1	(0.587) 0.587 0.584

2	(0.413) 0.413 0.416

Locus 18061 :

2 alleles

99.2% missing data

4	(0.612) 0.614 0.608

2	(0.388) 0.386 0.392

Locus 18062 :

2 alleles

98.0% missing data

1	(0.688) 0.684 0.702

3	(0.312) 0.316 0.298

Locus 18063 :

2 alleles

98.8% missing data

1	(0.482) 0.471 0.484

4	(0.518) 0.529 0.516

Locus 18064 :

2 alleles

99.2% missing data

4	(0.735) 0.733 0.736

3	(0.265) 0.267 0.264

Locus 18065 :

2 alleles

99.2% missing data

4	(0.140) 0.142 0.137

3	(0.860) 0.858 0.863

Locus 18066 :

2 alleles

99.2% missing data

1	(0.338) 0.342 0.339

3	(0.662) 0.658 0.661

Locus 18067 :

2 alleles

99.2% missing data

3	(0.662) 0.661 0.660

4	(0.338) 0.339 0.340

Locus 18068 :

2 alleles

98.8% missing data

4	(0.563) 0.565 0.567

2	(0.437) 0.435 0.433

Locus 18069 :

2 alleles

99.2% missing data

1	(0.639) 0.636 0.641

4	(0.361) 0.364 0.359

Locus 18070 :

2 alleles

98.8% missing data

3	(0.444) 0.455 0.442

4	(0.556) 0.545 0.558

Locus 18071 :

2 alleles

97.7% missing data

1	(0.539) 0.529 0.549

4	(0.461) 0.471 0.451

Locus 18072 :

2 alleles

99.2% missing data

1	(0.793) 0.789 0.796

3	(0.207) 0.211 0.204

Locus 18073 :

2 alleles

99.2% missing data

2	(0.445) 0.445 0.445

3	(0.555) 0.555 0.555

Locus 18074 :

2 alleles

96.9% missing data

4	(0.211) 0.216 0.202

2	(0.789) 0.784 0.798

Locus 18075 :

2 alleles

98.0% missing data

4	(0.138) 0.132 0.141

3	(0.862) 0.868 0.859

Locus 18076 :

2 alleles

99.2% missing data

4	(0.821) 0.816 0.826

3	(0.179) 0.184 0.174

Locus 18077 :

2 alleles

99.2% missing data

2	(0.386) 0.387 0.385

4	(0.614) 0.613 0.615

Locus 18078 :

2 alleles

99.2% missing data

3	(0.278) 0.278 0.274

1	(0.722) 0.722 0.726

Locus 18079 :

2 alleles

91.0% missing data

1	(0.958) 0.962 0.955

4	(0.042) 0.038 0.045

Locus 18080 :

2 alleles

99.2% missing data

3	(0.628) 0.620 0.636

2	(0.372) 0.380 0.364

Locus 18081 :

2 alleles

99.2% missing data

1	(0.311) 0.312 0.307

4	(0.689) 0.688 0.693

Locus 18082 :

2 alleles

99.2% missing data

3	(0.430) 0.429 0.433

4	(0.570) 0.571 0.567

Locus 18083 :

2 alleles

95.3% missing data

3	(0.866) 0.861 0.882

1	(0.134) 0.139 0.118

Locus 18084 :

2 alleles

96.9% missing data

2	(0.300) 0.312 0.284

1	(0.700) 0.688 0.716

Locus 18085 :

2 alleles

99.2% missing data

2	(0.322) 0.320 0.326

1	(0.678) 0.680 0.674

Locus 18086 :

2 alleles

98.8% missing data

3	(0.628) 0.628 0.630

4	(0.372) 0.372 0.370

Locus 18087 :

2 alleles

99.2% missing data

2	(0.627) 0.624 0.626

3	(0.373) 0.376 0.374

Locus 18088 :

2 alleles

98.8% missing data

1	(0.478) 0.476 0.478

4	(0.522) 0.524 0.522

Locus 18089 :

3 alleles

98.4% missing data

2	(0.363) 0.357 0.360

1	(0.430) 0.431 0.432

3	(0.208) 0.211 0.207

Locus 18090 :

2 alleles

99.2% missing data

3	(0.418) 0.413 0.420

4	(0.582) 0.587 0.580

Locus 18091 :

2 alleles

99.2% missing data

2	(0.696) 0.692 0.704

4	(0.304) 0.308 0.296

Locus 18092 :

2 alleles

99.2% missing data

2	(0.178) 0.179 0.187

4	(0.822) 0.821 0.813

Locus 18093 :

2 alleles

99.2% missing data

1	(0.572) 0.571 0.574

2	(0.428) 0.429 0.426

Locus 18094 :

2 alleles

99.2% missing data

4	(0.828) 0.822 0.829

3	(0.172) 0.178 0.171

Locus 18095 :

2 alleles

98.8% missing data

2	(0.334) 0.333 0.340

4	(0.666) 0.667 0.660

Locus 18096 :

2 alleles

99.2% missing data

1	(0.465) 0.472 0.469

2	(0.535) 0.528 0.531

Locus 18097 :

2 alleles

99.2% missing data

4	(0.440) 0.438 0.442

1	(0.560) 0.562 0.558

Locus 18098 :

2 alleles

99.2% missing data

3	(0.576) 0.576 0.581

2	(0.424) 0.424 0.419

Locus 18099 :

2 alleles

94.9% missing data

3	(0.852) 0.852 0.861

1	(0.148) 0.148 0.139

Locus 18100 :

2 alleles

98.8% missing data

4	(0.622) 0.610 0.623

1	(0.378) 0.390 0.377

Locus 18101 :

2 alleles

99.2% missing data

4	(0.485) 0.478 0.490

1	(0.515) 0.522 0.510

Locus 18102 :

2 alleles

99.2% missing data

3	(0.646) 0.651 0.639

1	(0.354) 0.349 0.361

Locus 18103 :

2 alleles

99.2% missing data

4	(0.445) 0.440 0.450

3	(0.555) 0.560 0.550

Locus 18104 :

2 alleles

98.4% missing data

1	(0.542) 0.541 0.543

3	(0.458) 0.459 0.457

Locus 18105 :

2 alleles

99.2% missing data

1	(0.546) 0.548 0.550

3	(0.454) 0.452 0.450

Locus 18106 :

3 alleles

98.8% missing data

4	(0.434) 0.432 0.434

3	(0.301) 0.307 0.295

2	(0.265) 0.261 0.271

Locus 18107 :

2 alleles

99.2% missing data

2	(0.620) 0.610 0.626

3	(0.380) 0.390 0.374

Locus 18108 :

2 alleles

96.9% missing data

3	(0.652) 0.658 0.652

2	(0.348) 0.342 0.348

Locus 18109 :

2 alleles

98.0% missing data

1	(0.728) 0.728 0.735

4	(0.272) 0.272 0.265

Locus 18110 :

2 alleles

97.3% missing data

3	(0.691) 0.682 0.708

1	(0.309) 0.318 0.292

Locus 18111 :

2 alleles

99.2% missing data

1	(0.613) 0.608 0.620

2	(0.387) 0.392 0.380

Locus 18112 :

2 alleles

99.2% missing data

3	(0.586) 0.586 0.585

1	(0.414) 0.414 0.415

Locus 18113 :

2 alleles

99.2% missing data

4	(0.411) 0.415 0.414

2	(0.589) 0.585 0.586

Locus 18114 :

2 alleles

98.4% missing data

4	(0.563) 0.572 0.558

1	(0.437) 0.428 0.442

Locus 18115 :

2 alleles

99.2% missing data

2	(0.662) 0.662 0.666

4	(0.338) 0.338 0.334

Locus 18116 :

2 alleles

99.2% missing data

3	(0.233) 0.231 0.235

2	(0.767) 0.769 0.765

Locus 18117 :

2 alleles

99.2% missing data

4	(0.326) 0.323 0.333

1	(0.674) 0.677 0.667

Locus 18118 :

2 alleles

99.2% missing data

2	(0.528) 0.526 0.525

1	(0.472) 0.474 0.475

Locus 18119 :

2 alleles

96.9% missing data

3	(0.256) 0.258 0.253

1	(0.744) 0.742 0.747

Locus 18120 :

2 alleles

0.0% missing data

2	(0.983) 0.979 0.995

1	(0.017) 0.021 0.005

Locus 18121 :

2 alleles

99.2% missing data

2	(0.332) 0.326 0.329

3	(0.668) 0.674 0.671

Locus 18122 :

2 alleles

99.2% missing data

4	(0.662) 0.650 0.661

3	(0.338) 0.350 0.339

Locus 18123 :

2 alleles

96.5% missing data

2	(0.842) 0.838 0.855

3	(0.158) 0.162 0.145

Locus 18124 :

2 alleles

98.8% missing data

4	(0.301) 0.313 0.294

2	(0.699) 0.687 0.706

Locus 18125 :

2 alleles

99.2% missing data

1	(0.325) 0.317 0.335

4	(0.675) 0.683 0.665

Locus 18126 :

2 alleles

97.7% missing data

1	(0.601) 0.603 0.602

2	(0.399) 0.397 0.398

Locus 18127 :

2 alleles

99.2% missing data

3	(0.248) 0.248 0.255

1	(0.752) 0.752 0.745

Locus 18128 :

2 alleles

99.2% missing data

4	(0.469) 0.466 0.462

1	(0.531) 0.534 0.538

Locus 18129 :

2 alleles

99.2% missing data

2	(0.636) 0.631 0.638

1	(0.364) 0.369 0.362

Locus 18130 :

2 alleles

99.2% missing data

4	(0.625) 0.626 0.622

2	(0.375) 0.374 0.378

Locus 18131 :

2 alleles

98.8% missing data

1	(0.373) 0.382 0.375

2	(0.627) 0.618 0.625

Locus 18132 :

2 alleles

96.5% missing data

3	(0.247) 0.233 0.256

2	(0.753) 0.767 0.744

Locus 18133 :

2 alleles

98.8% missing data

4	(0.791) 0.786 0.797

2	(0.209) 0.214 0.203

Locus 18134 :

2 alleles

99.2% missing data

3	(0.504) 0.508 0.505

2	(0.496) 0.492 0.495

Locus 18135 :

2 alleles

99.2% missing data

2	(0.277) 0.282 0.276

4	(0.723) 0.718 0.724

Locus 18136 :

2 alleles

99.2% missing data

2	(0.419) 0.418 0.420

1	(0.581) 0.582 0.580

Locus 18137 :

2 alleles

98.8% missing data

4	(0.784) 0.778 0.790

2	(0.216) 0.222 0.210

Locus 18138 :

2 alleles

99.2% missing data

3	(0.282) 0.280 0.290

2	(0.718) 0.720 0.710

Locus 18139 :

2 alleles

96.9% missing data

4	(0.822) 0.827 0.825

1	(0.178) 0.173 0.175

Locus 18140 :

2 alleles

99.2% missing data

1	(0.403) 0.403 0.401

2	(0.597) 0.597 0.599

Locus 18141 :

2 alleles

99.2% missing data

1	(0.568) 0.570 0.569

2	(0.432) 0.430 0.431

Locus 18142 :

2 alleles

98.8% missing data

4	(0.371) 0.368 0.370

3	(0.629) 0.632 0.630

Locus 18143 :

2 alleles

99.2% missing data

3	(0.416) 0.416 0.417

2	(0.584) 0.584 0.583

Locus 18144 :

2 alleles

99.2% missing data

4	(0.473) 0.476 0.473

3	(0.527) 0.524 0.527

Locus 18145 :

2 alleles

99.2% missing data

3	(0.593) 0.593 0.600

1	(0.407) 0.407 0.400

Locus 18146 :

2 alleles

99.2% missing data

4	(0.687) 0.679 0.692

3	(0.313) 0.321 0.308

Locus 18147 :

2 alleles

99.2% missing data

4	(0.318) 0.314 0.322

1	(0.682) 0.686 0.678

Locus 18148 :

2 alleles

97.3% missing data

2	(0.773) 0.761 0.776

1	(0.227) 0.239 0.224

Locus 18149 :

2 alleles

98.8% missing data

2	(0.524) 0.513 0.531

4	(0.476) 0.487 0.469

Locus 18150 :

2 alleles

98.4% missing data

1	(0.608) 0.616 0.606

2	(0.392) 0.384 0.394

Locus 18151 :

2 alleles

99.2% missing data

1	(0.802) 0.805 0.802

2	(0.198) 0.195 0.198

Locus 18152 :

2 alleles

99.2% missing data

2	(0.638) 0.629 0.645

4	(0.362) 0.371 0.355

Locus 18153 :

2 alleles

91.4% missing data

2	(0.917) 0.933 0.906

4	(0.083) 0.067 0.094

Locus 18154 :

2 alleles

99.2% missing data

4	(0.222) 0.217 0.228

2	(0.778) 0.783 0.772

Locus 18155 :

2 alleles

99.2% missing data

2	(0.573) 0.566 0.574

3	(0.427) 0.434 0.426

Locus 18156 :

2 alleles

98.8% missing data

4	(0.815) 0.813 0.813

3	(0.185) 0.187 0.187

Locus 18157 :

2 alleles

99.2% missing data

4	(0.653) 0.647 0.651

2	(0.347) 0.353 0.349

Locus 18158 :

2 alleles

99.2% missing data

2	(0.524) 0.519 0.535

3	(0.476) 0.481 0.465

Locus 18159 :

2 alleles

99.2% missing data

1	(0.464) 0.464 0.459

2	(0.536) 0.536 0.541

Locus 18160 :

2 alleles

99.2% missing data

4	(0.528) 0.516 0.537

1	(0.472) 0.484 0.463

Locus 18161 :

2 alleles

98.4% missing data

4	(0.676) 0.671 0.683

2	(0.324) 0.329 0.317

Locus 18162 :

2 alleles

99.2% missing data

2	(0.571) 0.570 0.568

3	(0.429) 0.430 0.432

Locus 18163 :

2 alleles

99.2% missing data

4	(0.375) 0.373 0.379

2	(0.625) 0.627 0.621

Locus 18164 :

2 alleles

99.2% missing data

1	(0.249) 0.255 0.246

2	(0.751) 0.745 0.754

Locus 18165 :

2 alleles

99.2% missing data

1	(0.612) 0.614 0.606

4	(0.388) 0.386 0.394

Locus 18166 :

2 alleles

0.0% missing data

4	(0.988) 0.982 0.997

1	(0.012) 0.018 0.003

Locus 18167 :

2 alleles

91.4% missing data

4	(0.902) 0.910 0.905

1	(0.098) 0.090 0.095

Locus 18168 :

2 alleles

94.9% missing data

2	(0.918) 0.926 0.912

1	(0.082) 0.074 0.088

Locus 18169 :

2 alleles

99.2% missing data

1	(0.691) 0.695 0.688

2	(0.309) 0.305 0.312

Locus 18170 :

2 alleles

99.2% missing data

1	(0.575) 0.580 0.570

2	(0.425) 0.420 0.430

Locus 18171 :

2 alleles

99.2% missing data

2	(0.556) 0.553 0.553

4	(0.444) 0.447 0.447

Locus 18172 :

2 alleles

91.0% missing data

3	(0.937) 0.946 0.929

1	(0.063) 0.054 0.071

Locus 18173 :

2 alleles

99.2% missing data

3	(0.773) 0.780 0.766

4	(0.227) 0.220 0.234

Locus 18174 :

2 alleles

98.8% missing data

3	(0.806) 0.806 0.802

4	(0.194) 0.194 0.198

Locus 18175 :

2 alleles

99.2% missing data

4	(0.560) 0.566 0.555

1	(0.440) 0.434 0.445

Locus 18176 :

2 alleles

99.2% missing data

3	(0.351) 0.353 0.357

1	(0.649) 0.647 0.643

Locus 18177 :

4 alleles

98.4% missing data

4	(0.184) 0.185 0.185

3	(0.189) 0.193 0.185

1	(0.108) 0.103 0.114

2	(0.519) 0.518 0.515

Locus 18178 :

2 alleles

99.2% missing data

2	(0.259) 0.259 0.260

4	(0.741) 0.741 0.740

Locus 18179 :

2 alleles

99.2% missing data

2	(0.606) 0.602 0.610

3	(0.394) 0.398 0.390

Locus 18180 :

2 alleles

97.7% missing data

2	(0.741) 0.742 0.748

4	(0.259) 0.258 0.252

Locus 18181 :

2 alleles

96.5% missing data

2	(0.853) 0.857 0.855

1	(0.147) 0.143 0.145

Locus 18182 :

2 alleles

99.2% missing data

4	(0.485) 0.485 0.482

2	(0.515) 0.515 0.518

Locus 18183 :

2 alleles

99.2% missing data

3	(0.794) 0.801 0.786

2	(0.206) 0.199 0.214

Locus 18184 :

2 alleles

99.2% missing data

1	(0.485) 0.482 0.485

4	(0.515) 0.518 0.515

Locus 18185 :

2 alleles

99.2% missing data

4	(0.578) 0.574 0.579

2	(0.422) 0.426 0.421

Locus 18186 :

2 alleles

95.3% missing data

4	(0.816) 0.816 0.825

3	(0.184) 0.184 0.175

Locus 18187 :

2 alleles

98.8% missing data

1	(0.579) 0.577 0.587

4	(0.421) 0.423 0.413

Locus 18188 :

2 alleles

96.9% missing data

4	(0.196) 0.196 0.188

3	(0.804) 0.804 0.812

Locus 18189 :

2 alleles

98.0% missing data

3	(0.783) 0.783 0.787

1	(0.217) 0.217 0.213

Locus 18190 :

2 alleles

99.2% missing data

1	(0.651) 0.655 0.645

3	(0.349) 0.345 0.355

Locus 18191 :

2 alleles

95.3% missing data

4	(0.846) 0.835 0.863

1	(0.154) 0.165 0.137

Locus 18192 :

2 alleles

0.4% missing data

3	(0.986) 0.983 0.996

2	(0.014) 0.017 0.004

Locus 18193 :

2 alleles

99.2% missing data

4	(0.482) 0.483 0.480

2	(0.518) 0.517 0.520

Locus 18194 :

2 alleles

99.2% missing data

3	(0.658) 0.658 0.652

1	(0.342) 0.342 0.348

Locus 18195 :

2 alleles

99.2% missing data

4	(0.696) 0.689 0.696

2	(0.304) 0.311 0.304

Locus 18196 :

3 alleles

98.4% missing data

1	(0.270) 0.272 0.274

2	(0.295) 0.290 0.297

4	(0.435) 0.439 0.429

Locus 18197 :

2 alleles

99.2% missing data

3	(0.368) 0.361 0.378

1	(0.632) 0.639 0.622

Locus 18198 :

2 alleles

99.2% missing data

3	(0.476) 0.479 0.481

2	(0.524) 0.521 0.519

Locus 18199 :

2 alleles

99.2% missing data

2	(0.478) 0.478 0.478

4	(0.522) 0.522 0.522

Locus 18200 :

2 alleles

99.2% missing data

4	(0.294) 0.295 0.295

2	(0.706) 0.705 0.705

Locus 18201 :

2 alleles

99.2% missing data

2	(0.558) 0.565 0.545

4	(0.442) 0.435 0.455

Locus 18202 :

2 alleles

99.2% missing data

2	(0.256) 0.253 0.257

1	(0.744) 0.747 0.743

Locus 18203 :

3 alleles

98.8% missing data

3	(0.493) 0.493 0.495

4	(0.396) 0.388 0.398

1	(0.111) 0.118 0.106

Locus 18204 :

2 alleles

98.4% missing data

4	(0.794) 0.791 0.792

3	(0.206) 0.209 0.208

Locus 18205 :

2 alleles

99.2% missing data

2	(0.713) 0.708 0.718

3	(0.287) 0.292 0.282

Locus 18206 :

2 alleles

94.9% missing data

4	(0.499) 0.535 0.474

3	(0.501) 0.465 0.526

Locus 18207 :

2 alleles

97.7% missing data

3	(0.750) 0.755 0.749

1	(0.250) 0.245 0.251

Locus 18208 :

2 alleles

90.6% missing data

3	(0.921) 0.930 0.916

1	(0.079) 0.070 0.084

Locus 18209 :

2 alleles

91.0% missing data

1	(0.916) 0.912 0.929

3	(0.084) 0.088 0.071

Locus 18210 :

2 alleles

96.5% missing data

1	(0.858) 0.870 0.850

3	(0.142) 0.130 0.150

Locus 18211 :

2 alleles

98.8% missing data

1	(0.364) 0.361 0.368

4	(0.636) 0.639 0.632

Locus 18212 :

2 alleles

99.2% missing data

2	(0.293) 0.292 0.300

1	(0.707) 0.708 0.700

Locus 18213 :

2 alleles

98.8% missing data

3	(0.407) 0.396 0.418

2	(0.593) 0.604 0.582

Locus 18214 :

2 alleles

93.8% missing data

3	(0.854) 0.851 0.867

1	(0.146) 0.149 0.133

Locus 18215 :

2 alleles

99.2% missing data

4	(0.562) 0.565 0.564

2	(0.438) 0.435 0.436

Locus 18216 :

2 alleles

99.2% missing data

2	(0.707) 0.714 0.708

3	(0.293) 0.286 0.292

Locus 18217 :

2 alleles

98.8% missing data

4	(0.496) 0.494 0.505

3	(0.504) 0.506 0.495

Locus 18218 :

2 alleles

98.8% missing data

3	(0.538) 0.555 0.534

1	(0.462) 0.445 0.466

Locus 18219 :

3 alleles

98.8% missing data

3	(0.428) 0.422 0.428

2	(0.253) 0.255 0.256

1	(0.320) 0.323 0.316

Locus 18220 :

2 alleles

99.2% missing data

2	(0.238) 0.242 0.233

4	(0.762) 0.758 0.767

Locus 18221 :

2 alleles

99.2% missing data

3	(0.437) 0.435 0.441

2	(0.563) 0.565 0.559

Locus 18222 :

2 alleles

98.8% missing data

4	(0.306) 0.312 0.309

3	(0.694) 0.688 0.691

Locus 18223 :

2 alleles

99.2% missing data

1	(0.456) 0.453 0.460

3	(0.544) 0.547 0.540

Locus 18224 :

2 alleles

99.2% missing data

3	(0.504) 0.506 0.506

2	(0.496) 0.494 0.494

Locus 18225 :

2 alleles

98.4% missing data

1	(0.759) 0.758 0.763

3	(0.241) 0.242 0.237

Locus 18226 :

2 alleles

96.9% missing data

2	(0.782) 0.776 0.793

4	(0.218) 0.224 0.207

Locus 18227 :

2 alleles

99.2% missing data

2	(0.439) 0.439 0.432

4	(0.561) 0.561 0.568

Locus 18228 :

2 alleles

99.2% missing data

2	(0.290) 0.285 0.299

4	(0.710) 0.715 0.701

Locus 18229 :

2 alleles

99.2% missing data

1	(0.527) 0.525 0.526

2	(0.473) 0.475 0.474

Locus 18230 :

2 alleles

67.6% missing data

1	(0.748) 0.617 0.844

3	(0.252) 0.383 0.156

Locus 18231 :

2 alleles

99.2% missing data

1	(0.314) 0.318 0.314

3	(0.686) 0.682 0.686

Locus 18232 :

2 alleles

99.2% missing data

4	(0.530) 0.528 0.531

1	(0.470) 0.472 0.469

Locus 18233 :

2 alleles

96.1% missing data

4	(0.891) 0.890 0.894

1	(0.109) 0.110 0.106

Locus 18234 :

2 alleles

99.2% missing data

1	(0.472) 0.477 0.473

3	(0.528) 0.523 0.527

Locus 18235 :

2 alleles

98.8% missing data

1	(0.570) 0.571 0.573

3	(0.430) 0.429 0.427

Locus 18236 :

2 alleles

99.2% missing data

3	(0.397) 0.391 0.399

1	(0.603) 0.609 0.601

Locus 18237 :

2 alleles

99.2% missing data

4	(0.499) 0.499 0.499

2	(0.501) 0.501 0.501

Locus 18238 :

2 alleles

99.2% missing data

2	(0.498) 0.498 0.496

4	(0.502) 0.502 0.504

Locus 18239 :

2 alleles

99.2% missing data

4	(0.581) 0.575 0.586

3	(0.419) 0.425 0.414

Locus 18240 :

2 alleles

0.4% missing data

4	(0.983) 0.979 0.995

1	(0.017) 0.021 0.005

Locus 18241 :

2 alleles

99.2% missing data

4	(0.828) 0.835 0.820

3	(0.172) 0.165 0.180

Locus 18242 :

2 alleles

99.2% missing data

2	(0.581) 0.575 0.593

1	(0.419) 0.425 0.407

Locus 18243 :

2 alleles

99.2% missing data

3	(0.499) 0.508 0.497

1	(0.501) 0.492 0.503

Locus 18244 :

2 alleles

99.2% missing data

3	(0.426) 0.427 0.429

4	(0.574) 0.573 0.571

Locus 18245 :

2 alleles

99.2% missing data

2	(0.222) 0.226 0.225

1	(0.778) 0.774 0.775

Locus 18246 :

2 alleles

99.2% missing data

2	(0.699) 0.692 0.704

3	(0.301) 0.308 0.296

Locus 18247 :

2 alleles

99.2% missing data

3	(0.310) 0.303 0.316

1	(0.690) 0.697 0.684

Locus 18248 :

2 alleles

97.7% missing data

2	(0.497) 0.491 0.495

4	(0.503) 0.509 0.505

Locus 18249 :

2 alleles

99.2% missing data

4	(0.615) 0.611 0.618

2	(0.385) 0.389 0.382

Locus 18250 :

2 alleles

99.2% missing data

3	(0.671) 0.671 0.666

2	(0.329) 0.329 0.334

Locus 18251 :

2 alleles

89.8% missing data

2	(0.911) 0.904 0.928

4	(0.089) 0.096 0.072

Locus 18252 :

2 alleles

99.2% missing data

4	(0.689) 0.684 0.693

1	(0.311) 0.316 0.307

Locus 18253 :

2 alleles

99.2% missing data

4	(0.753) 0.752 0.755

3	(0.247) 0.248 0.245

Locus 18254 :

2 alleles

99.2% missing data

2	(0.463) 0.465 0.466

1	(0.537) 0.535 0.534

Locus 18255 :

2 alleles

97.7% missing data

1	(0.647) 0.646 0.652

4	(0.353) 0.354 0.348

Locus 18256 :

2 alleles

99.2% missing data

1	(0.360) 0.362 0.359

2	(0.640) 0.638 0.641

Locus 18257 :

2 alleles

99.2% missing data

3	(0.786) 0.788 0.778

2	(0.214) 0.212 0.222

Locus 18258 :

2 alleles

99.2% missing data

4	(0.601) 0.597 0.602

3	(0.399) 0.403 0.398

Locus 18259 :

2 alleles

98.8% missing data

4	(0.640) 0.644 0.642

1	(0.360) 0.356 0.358

Locus 18260 :

2 alleles

99.2% missing data

4	(0.400) 0.399 0.400

3	(0.600) 0.601 0.600

Locus 18261 :

2 alleles

99.2% missing data

3	(0.362) 0.360 0.363

1	(0.638) 0.640 0.637

Locus 18262 :

2 alleles

93.4% missing data

3	(0.826) 0.838 0.827

1	(0.174) 0.162 0.173

Locus 18263 :

2 alleles

98.8% missing data

1	(0.753) 0.759 0.747

2	(0.247) 0.241 0.253

Locus 18264 :

2 alleles

99.2% missing data

2	(0.600) 0.593 0.601

1	(0.400) 0.407 0.399

Locus 18265 :

2 alleles

99.2% missing data

1	(0.500) 0.501 0.496

2	(0.500) 0.499 0.504

Locus 18266 :

2 alleles

99.2% missing data

2	(0.560) 0.552 0.563

3	(0.440) 0.448 0.437

Locus 18267 :

2 alleles

97.3% missing data

1	(0.677) 0.675 0.692

3	(0.323) 0.325 0.308

Locus 18268 :

2 alleles

99.2% missing data

3	(0.487) 0.491 0.486

2	(0.513) 0.509 0.514

Locus 18269 :

2 alleles

99.2% missing data

4	(0.682) 0.690 0.673

2	(0.318) 0.310 0.327

Locus 18270 :

3 alleles

98.8% missing data

2	(0.595) 0.587 0.603

4	(0.269) 0.271 0.263

1	(0.136) 0.142 0.134

Locus 18271 :

2 alleles

99.2% missing data

1	(0.295) 0.296 0.295

3	(0.705) 0.704 0.705

Locus 18272 :

2 alleles

99.2% missing data

3	(0.534) 0.532 0.532

1	(0.466) 0.468 0.468

Locus 18273 :

2 alleles

99.2% missing data

2	(0.603) 0.612 0.603

1	(0.397) 0.388 0.397

Locus 18274 :

2 alleles

99.2% missing data

1	(0.386) 0.383 0.391

4	(0.614) 0.617 0.609

Locus 18275 :

2 alleles

99.2% missing data

4	(0.501) 0.491 0.502

2	(0.499) 0.509 0.498

Locus 18276 :

2 alleles

99.2% missing data

2	(0.424) 0.425 0.425

4	(0.576) 0.575 0.575

Locus 18277 :

2 alleles

91.0% missing data

4	(0.858) 0.886 0.850

1	(0.142) 0.114 0.150

Locus 18278 :

2 alleles

99.2% missing data

1	(0.729) 0.732 0.725

4	(0.271) 0.268 0.275

Locus 18279 :

2 alleles

99.2% missing data

1	(0.571) 0.577 0.569

4	(0.429) 0.423 0.431

Locus 18280 :

2 alleles

98.8% missing data

2	(0.634) 0.635 0.624

3	(0.366) 0.365 0.376

Locus 18281 :

2 alleles

99.2% missing data

4	(0.704) 0.699 0.711

3	(0.296) 0.301 0.289

Locus 18282 :

2 alleles

99.2% missing data

4	(0.552) 0.542 0.558

1	(0.448) 0.458 0.442

Locus 18283 :

2 alleles

98.8% missing data

2	(0.711) 0.705 0.722

3	(0.289) 0.295 0.278

Locus 18284 :

2 alleles

99.2% missing data

3	(0.454) 0.448 0.457

1	(0.546) 0.552 0.543

Locus 18285 :

2 alleles

99.2% missing data

3	(0.565) 0.565 0.559

4	(0.435) 0.435 0.441

Locus 18286 :

2 alleles

98.0% missing data

4	(0.325) 0.328 0.320

2	(0.675) 0.672 0.680

Locus 18287 :

2 alleles

99.2% missing data

2	(0.702) 0.703 0.698

1	(0.298) 0.297 0.302

Locus 18288 :

2 alleles

0.0% missing data

4	(0.989) 0.991 0.994

1	(0.011) 0.009 0.006

Locus 18289 :

2 alleles

98.4% missing data

2	(0.608) 0.602 0.619

4	(0.392) 0.398 0.381

Locus 18290 :

2 alleles

98.8% missing data

3	(0.647) 0.657 0.639

4	(0.353) 0.343 0.361

Locus 18291 :

2 alleles

99.2% missing data

4	(0.632) 0.635 0.631

3	(0.368) 0.365 0.369

Locus 18292 :

2 alleles

99.2% missing data

2	(0.586) 0.587 0.584

4	(0.414) 0.413 0.416

Locus 18293 :

2 alleles

93.0% missing data

3	(0.884) 0.874 0.902

2	(0.116) 0.126 0.098

Locus 18294 :

2 alleles

99.2% missing data

4	(0.656) 0.653 0.660

3	(0.344) 0.347 0.340

Locus 18295 :

2 alleles

98.8% missing data

2	(0.590) 0.587 0.594

3	(0.410) 0.413 0.406

Locus 18296 :

2 alleles

99.2% missing data

4	(0.300) 0.303 0.304

2	(0.700) 0.697 0.696

Locus 18297 :

2 alleles

99.2% missing data

2	(0.497) 0.497 0.505

4	(0.503) 0.503 0.495

Locus 18298 :

2 alleles

99.2% missing data

3	(0.366) 0.366 0.364

1	(0.634) 0.634 0.636

Locus 18299 :

2 alleles

99.2% missing data

1	(0.285) 0.282 0.289

4	(0.715) 0.718 0.711

Locus 18300 :

2 alleles

99.2% missing data

1	(0.299) 0.297 0.305

2	(0.701) 0.703 0.695

Locus 18301 :

2 alleles

99.2% missing data

4	(0.429) 0.428 0.424

3	(0.571) 0.572 0.576

Locus 18302 :

2 alleles

99.2% missing data

1	(0.371) 0.375 0.371

3	(0.629) 0.625 0.629

Locus 18303 :

2 alleles

98.8% missing data

3	(0.571) 0.567 0.574

1	(0.429) 0.433 0.426

Locus 18304 :

2 alleles

99.2% missing data

4	(0.408) 0.409 0.416

3	(0.592) 0.591 0.584

Locus 18305 :

2 alleles

99.2% missing data

3	(0.334) 0.327 0.342

4	(0.666) 0.673 0.658

Locus 18306 :

2 alleles

99.2% missing data

1	(0.314) 0.311 0.320

4	(0.686) 0.689 0.680

Locus 18307 :

2 alleles

99.2% missing data

2	(0.650) 0.650 0.653

1	(0.350) 0.350 0.347

Locus 18308 :

2 alleles

98.4% missing data

3	(0.762) 0.763 0.757

4	(0.238) 0.237 0.243

Locus 18309 :

2 alleles

99.2% missing data

3	(0.596) 0.593 0.599

1	(0.404) 0.407 0.401

Locus 18310 :

2 alleles

99.2% missing data

3	(0.113) 0.108 0.120

1	(0.887) 0.892 0.880

Locus 18311 :

2 alleles

99.2% missing data

2	(0.260) 0.263 0.258

3	(0.740) 0.737 0.742

Locus 18312 :

2 alleles

97.3% missing data

4	(0.777) 0.775 0.786

1	(0.223) 0.225 0.214

Locus 18313 :

3 alleles

98.8% missing data

3	(0.222) 0.219 0.225

4	(0.176) 0.177 0.176

2	(0.603) 0.604 0.598

Locus 18314 :

2 alleles

99.2% missing data

4	(0.607) 0.602 0.612

2	(0.393) 0.398 0.388

Locus 18315 :

2 alleles

99.2% missing data

2	(0.408) 0.410 0.409

3	(0.592) 0.590 0.591

Locus 18316 :

2 alleles

99.2% missing data

2	(0.220) 0.218 0.225

1	(0.780) 0.782 0.775

Locus 18317 :

2 alleles

99.2% missing data

3	(0.703) 0.704 0.698

2	(0.297) 0.296 0.302

Locus 18318 :

2 alleles

98.8% missing data

1	(0.702) 0.705 0.698

3	(0.298) 0.295 0.302

Locus 18319 :

2 alleles

91.0% missing data

3	(0.892) 0.901 0.890

1	(0.108) 0.099 0.110

Locus 18320 :

2 alleles

99.2% missing data

3	(0.440) 0.445 0.431

2	(0.560) 0.555 0.569

Locus 18321 :

2 alleles

96.1% missing data

4	(0.823) 0.827 0.821

2	(0.177) 0.173 0.179

Locus 18322 :

2 alleles

99.2% missing data

3	(0.656) 0.654 0.657

2	(0.344) 0.346 0.343

Locus 18323 :

2 alleles

99.2% missing data

2	(0.656) 0.653 0.655

4	(0.344) 0.347 0.345

Locus 18324 :

2 alleles

99.2% missing data

1	(0.570) 0.567 0.578

3	(0.430) 0.433 0.422

Locus 18325 :

2 alleles

99.2% missing data

1	(0.499) 0.500 0.501

3	(0.501) 0.500 0.499

Locus 18326 :

2 alleles

99.2% missing data

2	(0.602) 0.604 0.604

4	(0.398) 0.396 0.396

Locus 18327 :

2 alleles

99.2% missing data

1	(0.758) 0.757 0.761

3	(0.242) 0.243 0.239

Locus 18328 :

2 alleles

99.2% missing data

1	(0.703) 0.697 0.702

3	(0.297) 0.303 0.298

Locus 18329 :

2 alleles

99.2% missing data

4	(0.410) 0.410 0.407

3	(0.590) 0.590 0.593

Locus 18330 :

2 alleles

99.2% missing data

2	(0.429) 0.421 0.430

1	(0.571) 0.579 0.570

Locus 18331 :

2 alleles

96.5% missing data

4	(0.766) 0.768 0.774

3	(0.234) 0.232 0.226

Locus 18332 :

2 alleles

98.4% missing data

2	(0.522) 0.522 0.523

1	(0.478) 0.478 0.477

Locus 18333 :

2 alleles

99.2% missing data

2	(0.431) 0.425 0.433

3	(0.569) 0.575 0.567

Locus 18334 :

3 alleles

97.3% missing data

2	(0.482) 0.494 0.461

1	(0.304) 0.293 0.333

3	(0.214) 0.213 0.205

Locus 18335 :

2 alleles

99.2% missing data

2	(0.519) 0.516 0.516

3	(0.481) 0.484 0.484

Locus 18336 :

2 alleles

99.2% missing data

1	(0.585) 0.585 0.583

3	(0.415) 0.415 0.417

Locus 18337 :

2 alleles

99.2% missing data

1	(0.534) 0.535 0.534

3	(0.466) 0.465 0.466

Locus 18338 :

2 alleles

97.7% missing data

1	(0.872) 0.866 0.878

3	(0.128) 0.134 0.122

Locus 18339 :

2 alleles

99.2% missing data

4	(0.449) 0.445 0.451

3	(0.551) 0.555 0.549

Locus 18340 :

2 alleles

99.2% missing data

1	(0.520) 0.522 0.516

2	(0.480) 0.478 0.484

Locus 18341 :

2 alleles

98.4% missing data

3	(0.693) 0.689 0.702

4	(0.307) 0.311 0.298

Locus 18342 :

2 alleles

93.0% missing data

1	(0.883) 0.870 0.901

2	(0.117) 0.130 0.099

Locus 18343 :

2 alleles

95.3% missing data

2	(0.870) 0.869 0.878

3	(0.130) 0.131 0.122

Locus 18344 :

2 alleles

99.2% missing data

4	(0.608) 0.604 0.605

1	(0.392) 0.396 0.395

Locus 18345 :

2 alleles

99.2% missing data

4	(0.541) 0.543 0.537

1	(0.459) 0.457 0.463

Locus 18346 :

3 alleles

98.0% missing data

2	(0.408) 0.414 0.400

4	(0.417) 0.410 0.423

3	(0.175) 0.176 0.177

Locus 18347 :

2 alleles

99.2% missing data

2	(0.562) 0.558 0.571

3	(0.438) 0.442 0.429

Locus 18348 :

2 alleles

6.6% missing data

1	(0.983) 0.978 0.995

4	(0.017) 0.022 0.005

Locus 18349 :

2 alleles

99.2% missing data

1	(0.452) 0.446 0.465

3	(0.548) 0.554 0.535

Locus 18350 :

2 alleles

99.2% missing data

2	(0.476) 0.480 0.477

1	(0.524) 0.520 0.523

Locus 18351 :

3 alleles

98.8% missing data

2	(0.193) 0.189 0.198

4	(0.583) 0.587 0.574

1	(0.224) 0.224 0.227

Locus 18352 :

2 alleles

99.2% missing data

3	(0.672) 0.677 0.669

1	(0.328) 0.323 0.331

Locus 18353 :

3 alleles

98.8% missing data

2	(0.244) 0.241 0.245

3	(0.313) 0.309 0.319

1	(0.443) 0.450 0.436

Locus 18354 :

2 alleles

99.2% missing data

1	(0.302) 0.300 0.307

3	(0.698) 0.700 0.693

Locus 18355 :

2 alleles

98.8% missing data

3	(0.399) 0.392 0.404

1	(0.601) 0.608 0.596

Locus 18356 :

2 alleles

98.0% missing data

4	(0.231) 0.226 0.226

3	(0.769) 0.774 0.774

Locus 18357 :

2 alleles

98.8% missing data

1	(0.567) 0.569 0.568

2	(0.433) 0.431 0.432

Locus 18358 :

2 alleles

99.2% missing data

2	(0.255) 0.255 0.260

1	(0.745) 0.745 0.740

Locus 18359 :

2 alleles

99.2% missing data

3	(0.541) 0.534 0.547

2	(0.459) 0.466 0.453

Locus 18360 :

2 alleles

99.2% missing data

3	(0.417) 0.423 0.418

1	(0.583) 0.577 0.582

Locus 18361 :

2 alleles

95.3% missing data

1	(0.801) 0.799 0.814

2	(0.199) 0.201 0.186

Locus 18362 :

2 alleles

99.2% missing data

3	(0.640) 0.635 0.642

4	(0.360) 0.365 0.358

Locus 18363 :

2 alleles

99.2% missing data

4	(0.354) 0.349 0.357

1	(0.646) 0.651 0.643

Locus 18364 :

2 alleles

98.8% missing data

4	(0.544) 0.543 0.540

1	(0.456) 0.457 0.460

Locus 18365 :

2 alleles

99.2% missing data

1	(0.411) 0.415 0.414

3	(0.589) 0.585 0.586

Locus 18366 :

2 alleles

99.2% missing data

2	(0.652) 0.648 0.656

3	(0.348) 0.352 0.344

Locus 18367 :

2 alleles

99.2% missing data

1	(0.312) 0.310 0.310

4	(0.688) 0.690 0.690

Locus 18368 :

2 alleles

98.8% missing data

1	(0.576) 0.579 0.574

4	(0.424) 0.421 0.426

Locus 18369 :

3 alleles

98.0% missing data

1	(0.178) 0.170 0.188

2	(0.271) 0.272 0.273

4	(0.551) 0.558 0.539

Locus 18370 :

2 alleles

99.2% missing data

1	(0.646) 0.647 0.649

2	(0.354) 0.353 0.351

Locus 18371 :

2 alleles

99.2% missing data

3	(0.415) 0.419 0.415

1	(0.585) 0.581 0.585

Locus 18372 :

3 alleles

93.0% missing data

2	(0.707) 0.707 0.727

3	(0.199) 0.198 0.182

1	(0.094) 0.095 0.091

Locus 18373 :

2 alleles

12.5% missing data

4	(0.990) 0.992 0.994

3	(0.010) 0.008 0.006

Locus 18374 :

2 alleles

96.1% missing data

3	(0.827) 0.839 0.820

4	(0.173) 0.161 0.180

Locus 18375 :

2 alleles

99.2% missing data

2	(0.643) 0.645 0.645

4	(0.357) 0.355 0.355

Locus 18376 :

2 alleles

9.0% missing data

3	(0.969) 0.935 0.990

1	(0.031) 0.065 0.010

Locus 18377 :

2 alleles

99.2% missing data

4	(0.219) 0.213 0.230

1	(0.781) 0.787 0.770

Locus 18378 :

2 alleles

99.2% missing data

3	(0.514) 0.515 0.511

4	(0.486) 0.485 0.489

Locus 18379 :

2 alleles

99.2% missing data

2	(0.326) 0.322 0.330

1	(0.674) 0.678 0.670

Locus 18380 :

2 alleles

99.2% missing data

4	(0.584) 0.583 0.587

1	(0.416) 0.417 0.413

Locus 18381 :

2 alleles

98.4% missing data

1	(0.716) 0.720 0.715

3	(0.284) 0.280 0.285

Locus 18382 :

2 alleles

99.2% missing data

3	(0.637) 0.638 0.640

4	(0.363) 0.362 0.360

Locus 18383 :

2 alleles

99.2% missing data

2	(0.596) 0.589 0.600

3	(0.404) 0.411 0.400

Locus 18384 :

2 alleles

99.2% missing data

4	(0.351) 0.356 0.350

1	(0.649) 0.644 0.650

Locus 18385 :

2 alleles

99.2% missing data

2	(0.175) 0.169 0.179

1	(0.825) 0.831 0.821

Locus 18386 :

2 alleles

99.2% missing data

2	(0.676) 0.683 0.671

1	(0.324) 0.317 0.329

Locus 18387 :

2 alleles

99.2% missing data

1	(0.588) 0.594 0.575

3	(0.412) 0.406 0.425

Locus 18388 :

2 alleles

96.1% missing data

3	(0.833) 0.839 0.838

4	(0.167) 0.161 0.162

Locus 18389 :

2 alleles

99.2% missing data

1	(0.288) 0.281 0.292

3	(0.712) 0.719 0.708

Locus 18390 :

2 alleles

99.2% missing data

2	(0.554) 0.551 0.556

4	(0.446) 0.449 0.444

Locus 18391 :

2 alleles

99.2% missing data

1	(0.316) 0.317 0.311

3	(0.684) 0.683 0.689

Locus 18392 :

3 alleles

98.8% missing data

3	(0.244) 0.248 0.239

1	(0.513) 0.508 0.512

2	(0.244) 0.243 0.249

Locus 18393 :

2 alleles

99.2% missing data

4	(0.589) 0.592 0.592

1	(0.411) 0.408 0.408

Locus 18394 :

2 alleles

99.2% missing data

4	(0.464) 0.462 0.466

2	(0.536) 0.538 0.534

Locus 18395 :

2 alleles

98.0% missing data

2	(0.835) 0.839 0.833

3	(0.165) 0.161 0.167

Locus 18396 :

2 alleles

99.2% missing data

1	(0.371) 0.368 0.374

3	(0.629) 0.632 0.626

Locus 18397 :

2 alleles

99.2% missing data

2	(0.556) 0.556 0.554

1	(0.444) 0.444 0.446

Locus 18398 :

3 alleles

98.4% missing data

3	(0.374) 0.382 0.373

1	(0.275) 0.273 0.271

2	(0.351) 0.345 0.356

Locus 18399 :

2 alleles

99.2% missing data

4	(0.688) 0.688 0.687

1	(0.312) 0.312 0.313

Locus 18400 :

3 alleles

98.8% missing data

2	(0.139) 0.136 0.146

3	(0.176) 0.177 0.171

1	(0.685) 0.688 0.683

Locus 18401 :

2 alleles

98.8% missing data

3	(0.562) 0.556 0.567

4	(0.438) 0.444 0.433

Locus 18402 :

2 alleles

98.4% missing data

3	(0.674) 0.677 0.675

2	(0.326) 0.323 0.325

Locus 18403 :

2 alleles

99.2% missing data

4	(0.478) 0.464 0.482

1	(0.522) 0.536 0.518

Locus 18404 :

2 alleles

99.2% missing data

3	(0.571) 0.563 0.576

1	(0.429) 0.437 0.424

Locus 18405 :

2 alleles

89.1% missing data

2	(0.845) 0.876 0.809

3	(0.155) 0.124 0.191

Locus 18406 :

2 alleles

99.2% missing data

1	(0.549) 0.546 0.557

3	(0.451) 0.454 0.443

Locus 18407 :

2 alleles

99.2% missing data

1	(0.378) 0.370 0.383

3	(0.622) 0.630 0.617

Locus 18408 :

2 alleles

17.2% missing data

1	(0.983) 0.975 0.995

3	(0.017) 0.025 0.005

Locus 18409 :

2 alleles

0.4% missing data

4	(0.985) 0.989 0.992

1	(0.015) 0.011 0.008

Locus 18410 :

2 alleles

0.0% missing data

3	(0.894) 0.740 0.969

2	(0.106) 0.260 0.031

Locus 18411 :

2 alleles

99.2% missing data

1	(0.307) 0.309 0.307

2	(0.693) 0.691 0.693

Locus 18412 :

2 alleles

99.2% missing data

3	(0.659) 0.658 0.659

2	(0.341) 0.342 0.341

Locus 18413 :

2 alleles

99.2% missing data

4	(0.742) 0.739 0.744

1	(0.258) 0.261 0.256

Locus 18414 :

2 alleles

99.2% missing data

4	(0.498) 0.500 0.494

3	(0.502) 0.500 0.506

Locus 18415 :

2 alleles

99.2% missing data

2	(0.582) 0.578 0.582

1	(0.418) 0.422 0.418

Locus 18416 :

2 alleles

99.2% missing data

2	(0.352) 0.354 0.358

4	(0.648) 0.646 0.642

Locus 18417 :

2 alleles

99.2% missing data

2	(0.367) 0.364 0.366

4	(0.633) 0.636 0.634

Locus 18418 :

2 alleles

91.0% missing data

3	(0.886) 0.899 0.886

2	(0.114) 0.101 0.114

Locus 18419 :

2 alleles

93.4% missing data

3	(0.884) 0.877 0.895

2	(0.116) 0.123 0.105

Locus 18420 :

2 alleles

98.8% missing data

3	(0.636) 0.642 0.638

1	(0.364) 0.358 0.362

Locus 18421 :

2 alleles

8.2% missing data

2	(0.983) 0.975 0.995

4	(0.017) 0.025 0.005

Locus 18422 :

2 alleles

99.2% missing data

1	(0.565) 0.568 0.556

4	(0.435) 0.432 0.444

Locus 18423 :

2 alleles

99.2% missing data

1	(0.226) 0.225 0.232

4	(0.774) 0.775 0.768

Locus 18424 :

2 alleles

99.2% missing data

1	(0.418) 0.416 0.424

3	(0.582) 0.584 0.576

Locus 18425 :

2 alleles

99.2% missing data

1	(0.599) 0.602 0.595

4	(0.401) 0.398 0.405

Locus 18426 :

2 alleles

99.2% missing data

1	(0.413) 0.406 0.418

3	(0.587) 0.594 0.582

Locus 18427 :

2 alleles

96.1% missing data

3	(0.185) 0.168 0.189

1	(0.815) 0.832 0.811

Locus 18428 :

2 alleles

98.8% missing data

2	(0.590) 0.592 0.589

3	(0.410) 0.408 0.411

Locus 18429 :

2 alleles

99.2% missing data

3	(0.577) 0.574 0.580

4	(0.423) 0.426 0.420

Locus 18430 :

2 alleles

96.5% missing data

4	(0.632) 0.627 0.650

1	(0.368) 0.373 0.350

Locus 18431 :

2 alleles

99.2% missing data

2	(0.357) 0.359 0.357

1	(0.643) 0.641 0.643

Locus 18432 :

2 alleles

99.2% missing data

3	(0.271) 0.277 0.270

4	(0.729) 0.723 0.730

Locus 18433 :

2 alleles

99.2% missing data

2	(0.627) 0.621 0.628

3	(0.373) 0.379 0.372

Locus 18434 :

2 alleles

98.4% missing data

3	(0.535) 0.539 0.532

1	(0.465) 0.461 0.468

Locus 18435 :

2 alleles

0.8% missing data

4	(0.985) 0.989 0.992

3	(0.015) 0.011 0.008

Locus 18436 :

2 alleles

99.2% missing data

2	(0.408) 0.415 0.401

3	(0.592) 0.585 0.599

Locus 18437 :

2 alleles

99.2% missing data

1	(0.518) 0.524 0.519

2	(0.482) 0.476 0.481

Locus 18438 :

2 alleles

99.2% missing data

2	(0.637) 0.639 0.632

3	(0.363) 0.361 0.368

Locus 18439 :

2 alleles

99.2% missing data

1	(0.432) 0.436 0.427

2	(0.568) 0.564 0.573

Locus 18440 :

2 alleles

99.2% missing data

3	(0.170) 0.173 0.168

4	(0.830) 0.827 0.832

Locus 18441 :

3 alleles

98.8% missing data

2	(0.394) 0.393 0.397

1	(0.267) 0.268 0.263

3	(0.339) 0.338 0.340

Locus 18442 :

2 alleles

99.2% missing data

3	(0.815) 0.808 0.820

1	(0.185) 0.192 0.180

Locus 18443 :

2 alleles

99.2% missing data

1	(0.460) 0.456 0.460

3	(0.540) 0.544 0.540

Locus 18444 :

2 alleles

99.2% missing data

4	(0.525) 0.521 0.526

1	(0.475) 0.479 0.474

Locus 18445 :

2 alleles

99.2% missing data

3	(0.282) 0.282 0.280

2	(0.718) 0.718 0.720

Locus 18446 :

2 alleles

99.2% missing data

1	(0.778) 0.782 0.777

4	(0.222) 0.218 0.223

Locus 18447 :

2 alleles

99.2% missing data

2	(0.530) 0.537 0.521

3	(0.470) 0.463 0.479

Locus 18448 :

2 alleles

99.2% missing data

2	(0.279) 0.278 0.283

3	(0.721) 0.722 0.717

Locus 18449 :

2 alleles

99.2% missing data

4	(0.345) 0.349 0.346

3	(0.655) 0.651 0.654

Locus 18450 :

2 alleles

94.1% missing data

2	(0.556) 0.558 0.554

3	(0.444) 0.442 0.446

Locus 18451 :

2 alleles

98.8% missing data

1	(0.368) 0.376 0.369

3	(0.632) 0.624 0.631

Locus 18452 :

2 alleles

99.2% missing data

2	(0.571) 0.575 0.576

3	(0.429) 0.425 0.424

Locus 18453 :

2 alleles

99.2% missing data

4	(0.487) 0.491 0.478

3	(0.513) 0.509 0.522

Locus 18454 :

2 alleles

99.2% missing data

3	(0.425) 0.422 0.431

2	(0.575) 0.578 0.569

Locus 18455 :

2 alleles

99.2% missing data

1	(0.475) 0.474 0.478

4	(0.525) 0.526 0.522

Locus 18456 :

2 alleles

99.2% missing data

3	(0.391) 0.392 0.404

2	(0.609) 0.608 0.596

Locus 18457 :

3 alleles

98.8% missing data

4	(0.251) 0.248 0.257

3	(0.379) 0.376 0.378

1	(0.370) 0.376 0.365

Locus 18458 :

3 alleles

96.1% missing data

4	(0.137) 0.140 0.130

3	(0.556) 0.547 0.575

2	(0.306) 0.312 0.296

Locus 18459 :

2 alleles

99.2% missing data

2	(0.613) 0.609 0.616

3	(0.387) 0.391 0.384

Locus 18460 :

2 alleles

98.4% missing data

4	(0.679) 0.685 0.668

1	(0.321) 0.315 0.332

Locus 18461 :

2 alleles

99.2% missing data

4	(0.423) 0.422 0.420

2	(0.577) 0.578 0.580

Locus 18462 :

2 alleles

99.2% missing data

4	(0.447) 0.448 0.440

1	(0.553) 0.552 0.560

Locus 18463 :

2 alleles

98.8% missing data

2	(0.407) 0.410 0.402

3	(0.593) 0.590 0.598

Locus 18464 :

2 alleles

8.2% missing data

1	(0.986) 0.979 0.996

2	(0.014) 0.021 0.004

Locus 18465 :

2 alleles

99.2% missing data

1	(0.511) 0.518 0.507

2	(0.489) 0.482 0.493

Locus 18466 :

2 alleles

98.0% missing data

1	(0.362) 0.371 0.354

4	(0.638) 0.629 0.646

Locus 18467 :

2 alleles

99.2% missing data

3	(0.712) 0.706 0.721

4	(0.288) 0.294 0.279

Locus 18468 :

2 alleles

99.2% missing data

1	(0.717) 0.724 0.712

3	(0.283) 0.276 0.288

Locus 18469 :

2 alleles

99.2% missing data

4	(0.497) 0.500 0.502

1	(0.503) 0.500 0.498

Locus 18470 :

2 alleles

98.4% missing data

1	(0.728) 0.720 0.738

3	(0.272) 0.280 0.262

Locus 18471 :

3 alleles

96.1% missing data

1	(0.724) 0.730 0.734

4	(0.076) 0.080 0.074

2	(0.199) 0.191 0.192

Locus 18472 :

2 alleles

99.2% missing data

1	(0.368) 0.369 0.365

2	(0.632) 0.631 0.635

Locus 18473 :

2 alleles

99.2% missing data

4	(0.175) 0.176 0.176

2	(0.825) 0.824 0.824

Locus 18474 :

3 alleles

98.8% missing data

3	(0.207) 0.213 0.206

2	(0.573) 0.572 0.567

1	(0.220) 0.216 0.227

Locus 18475 :

2 alleles

8.2% missing data

4	(0.986) 0.978 0.996

3	(0.014) 0.022 0.004

Locus 18476 :

2 alleles

99.2% missing data

3	(0.565) 0.562 0.568

1	(0.435) 0.438 0.432

Locus 18477 :

2 alleles

99.2% missing data

3	(0.278) 0.273 0.283

1	(0.722) 0.727 0.717

Locus 18478 :

2 alleles

98.4% missing data

3	(0.743) 0.750 0.745

1	(0.257) 0.250 0.255

Locus 18479 :

2 alleles

99.2% missing data

2	(0.525) 0.520 0.529

1	(0.475) 0.480 0.471

Locus 18480 :

2 alleles

96.1% missing data

3	(0.761) 0.775 0.752

1	(0.239) 0.225 0.248

Locus 18481 :

2 alleles

99.2% missing data

4	(0.560) 0.564 0.561

3	(0.440) 0.436 0.439

Locus 18482 :

2 alleles

99.2% missing data

1	(0.658) 0.652 0.656

2	(0.342) 0.348 0.344

Locus 18483 :

2 alleles

99.2% missing data

1	(0.297) 0.295 0.300

2	(0.703) 0.705 0.700

Locus 18484 :

2 alleles

99.2% missing data

3	(0.420) 0.423 0.425

4	(0.580) 0.577 0.575

Locus 18485 :

2 alleles

99.2% missing data

1	(0.547) 0.544 0.551

2	(0.453) 0.456 0.449

Locus 18486 :

2 alleles

99.2% missing data

4	(0.493) 0.485 0.498

1	(0.507) 0.515 0.502

Locus 18487 :

2 alleles

99.2% missing data

3	(0.746) 0.748 0.744

1	(0.254) 0.252 0.256

Locus 18488 :

3 alleles

98.4% missing data

4	(0.297) 0.297 0.296

1	(0.265) 0.258 0.270

3	(0.438) 0.445 0.435

Locus 18489 :

2 alleles

98.4% missing data

1	(0.698) 0.714 0.688

4	(0.302) 0.286 0.312

Locus 18490 :

2 alleles

99.2% missing data

4	(0.220) 0.215 0.226

3	(0.780) 0.785 0.774

Locus 18491 :

2 alleles

73.8% missing data

1	(0.958) 0.947 0.975

3	(0.042) 0.053 0.025

Locus 18492 :

2 alleles

99.2% missing data

3	(0.314) 0.323 0.310

1	(0.686) 0.677 0.690

Locus 18493 :

2 alleles

98.8% missing data

2	(0.347) 0.342 0.342

1	(0.653) 0.658 0.658

Locus 18494 :

2 alleles

98.4% missing data

2	(0.788) 0.791 0.788

1	(0.212) 0.209 0.212

Locus 18495 :

2 alleles

99.2% missing data

2	(0.625) 0.631 0.621

4	(0.375) 0.369 0.379

Locus 18496 :

2 alleles

99.2% missing data

4	(0.266) 0.262 0.269

3	(0.734) 0.738 0.731

Locus 18497 :

2 alleles

99.2% missing data

2	(0.468) 0.471 0.467

1	(0.532) 0.529 0.533

Locus 18498 :

2 alleles

99.2% missing data

3	(0.740) 0.738 0.742

4	(0.260) 0.262 0.258

Locus 18499 :

3 alleles

98.0% missing data

1	(0.234) 0.227 0.236

4	(0.342) 0.329 0.348

3	(0.424) 0.444 0.416

Locus 18500 :

2 alleles

99.2% missing data

4	(0.682) 0.673 0.687

2	(0.318) 0.327 0.313

Locus 18501 :

2 alleles

97.7% missing data

4	(0.160) 0.163 0.156

3	(0.840) 0.837 0.844

Locus 18502 :

2 alleles

97.7% missing data

1	(0.135) 0.134 0.135

4	(0.865) 0.866 0.865

Locus 18503 :

2 alleles

98.4% missing data

2	(0.316) 0.317 0.314

3	(0.684) 0.683 0.686

Locus 18504 :

2 alleles

99.2% missing data

4	(0.255) 0.249 0.261

2	(0.745) 0.751 0.739

Locus 18505 :

2 alleles

99.2% missing data

4	(0.707) 0.709 0.700

3	(0.293) 0.291 0.300

Locus 18506 :

3 alleles

98.8% missing data

3	(0.296) 0.289 0.302

4	(0.454) 0.457 0.451

1	(0.250) 0.253 0.247

Locus 18507 :

2 alleles

97.7% missing data

4	(0.726) 0.719 0.737

3	(0.274) 0.281 0.263

Locus 18508 :

2 alleles

98.8% missing data

4	(0.763) 0.768 0.751

2	(0.237) 0.232 0.249

Locus 18509 :

2 alleles

99.2% missing data

4	(0.601) 0.603 0.598

2	(0.399) 0.397 0.402

Locus 18510 :

2 alleles

98.0% missing data

2	(0.512) 0.510 0.507

1	(0.488) 0.490 0.493

Locus 18511 :

2 alleles

88.7% missing data

1	(0.737) 0.747 0.731

3	(0.263) 0.253 0.269

Locus 18512 :

2 alleles

99.2% missing data

2	(0.396) 0.393 0.398

1	(0.604) 0.607 0.602

Locus 18513 :

2 alleles

99.2% missing data

3	(0.641) 0.640 0.644

1	(0.359) 0.360 0.356

Locus 18514 :

2 alleles

99.2% missing data

4	(0.334) 0.339 0.325

3	(0.666) 0.661 0.675

Locus 18515 :

2 alleles

99.2% missing data

4	(0.771) 0.766 0.777

2	(0.229) 0.234 0.223

Locus 18516 :

2 alleles

99.2% missing data

1	(0.564) 0.565 0.561

4	(0.436) 0.435 0.439

Locus 18517 :

2 alleles

99.2% missing data

1	(0.424) 0.429 0.423

3	(0.576) 0.571 0.577

Locus 18518 :

2 alleles

97.3% missing data

4	(0.712) 0.722 0.710

2	(0.288) 0.278 0.290

Locus 18519 :

2 alleles

99.2% missing data

4	(0.408) 0.408 0.407

2	(0.592) 0.592 0.593

Locus 18520 :

2 alleles

98.8% missing data

4	(0.741) 0.736 0.742

3	(0.259) 0.264 0.258

Locus 18521 :

2 alleles

2.3% missing data

1	(0.987) 0.982 0.996

2	(0.013) 0.018 0.004

Locus 18522 :

2 alleles

91.0% missing data

1	(0.888) 0.908 0.879

3	(0.112) 0.092 0.121

Locus 18523 :

2 alleles

91.0% missing data

3	(0.946) 0.959 0.940

1	(0.054) 0.041 0.060

Locus 18524 :

2 alleles

99.2% missing data

2	(0.501) 0.504 0.500

1	(0.499) 0.496 0.500

Locus 18525 :

2 alleles

99.2% missing data

1	(0.543) 0.542 0.545

3	(0.457) 0.458 0.455

Locus 18526 :

2 alleles

96.1% missing data

2	(0.175) 0.170 0.173

3	(0.825) 0.830 0.827

Locus 18527 :

2 alleles

99.2% missing data

3	(0.477) 0.479 0.483

4	(0.523) 0.521 0.517

Locus 18528 :

2 alleles

99.2% missing data

3	(0.470) 0.469 0.470

2	(0.530) 0.531 0.530

Locus 18529 :

2 alleles

0.4% missing data

2	(0.964) 0.973 0.967

3	(0.036) 0.027 0.033

Locus 18530 :

2 alleles

93.0% missing data

1	(0.912) 0.916 0.915

4	(0.088) 0.084 0.085

Locus 18531 :

2 alleles

99.2% missing data

2	(0.485) 0.480 0.491

4	(0.515) 0.520 0.509

Locus 18532 :

3 alleles

97.3% missing data

2	(0.549) 0.562 0.539

3	(0.271) 0.265 0.276

4	(0.180) 0.173 0.185

Locus 18533 :

2 alleles

99.2% missing data

1	(0.468) 0.463 0.475

3	(0.532) 0.537 0.525

Locus 18534 :

3 alleles

98.8% missing data

1	(0.155) 0.152 0.155

3	(0.554) 0.548 0.560

2	(0.291) 0.300 0.286

Locus 18535 :

2 alleles

97.7% missing data

3	(0.169) 0.164 0.177

1	(0.831) 0.836 0.823

Locus 18536 :

2 alleles

99.2% missing data

2	(0.350) 0.352 0.350

3	(0.650) 0.648 0.650

Locus 18537 :

2 alleles

0.0% missing data

1	(0.987) 0.982 0.996

3	(0.013) 0.018 0.004

Locus 18538 :

2 alleles

97.3% missing data

2	(0.749) 0.755 0.745

4	(0.251) 0.245 0.255

Locus 18539 :

2 alleles

99.2% missing data

1	(0.688) 0.694 0.684

2	(0.312) 0.306 0.316

Locus 18540 :

2 alleles

98.4% missing data

2	(0.588) 0.591 0.581

3	(0.412) 0.409 0.419

Locus 18541 :

3 alleles

95.3% missing data

2	(0.297) 0.291 0.294

1	(0.130) 0.123 0.132

3	(0.573) 0.586 0.573

Locus 18542 :

2 alleles

98.8% missing data

4	(0.476) 0.476 0.479

2	(0.524) 0.524 0.521

Locus 18543 :

2 alleles

91.0% missing data

2	(0.912) 0.923 0.908

4	(0.088) 0.077 0.092

Locus 18544 :

2 alleles

98.8% missing data

1	(0.510) 0.509 0.514

2	(0.490) 0.491 0.486

Locus 18545 :

2 alleles

98.4% missing data

2	(0.388) 0.397 0.390

4	(0.612) 0.603 0.610

Locus 18546 :

2 alleles

99.2% missing data

2	(0.397) 0.394 0.395

4	(0.603) 0.606 0.605

Locus 18547 :

2 alleles

98.8% missing data

4	(0.415) 0.406 0.421

1	(0.585) 0.594 0.579

Locus 18548 :

2 alleles

99.2% missing data

3	(0.765) 0.761 0.763

1	(0.235) 0.239 0.237

Locus 18549 :

2 alleles

99.2% missing data

4	(0.401) 0.397 0.411

2	(0.599) 0.603 0.589

Locus 18550 :

3 alleles

98.8% missing data

4	(0.240) 0.240 0.244

3	(0.470) 0.478 0.465

2	(0.290) 0.281 0.291

Locus 18551 :

2 alleles

99.2% missing data

1	(0.347) 0.350 0.346

2	(0.653) 0.650 0.654

Locus 18552 :

2 alleles

99.2% missing data

1	(0.644) 0.638 0.640

4	(0.356) 0.362 0.360

Locus 18553 :

2 alleles

99.2% missing data

1	(0.523) 0.520 0.520

4	(0.477) 0.480 0.480

Locus 18554 :

2 alleles

99.2% missing data

3	(0.370) 0.371 0.361

1	(0.630) 0.629 0.639

Locus 18555 :

2 alleles

99.2% missing data

1	(0.363) 0.364 0.360

4	(0.637) 0.636 0.640

Locus 18556 :

2 alleles

99.2% missing data

2	(0.450) 0.451 0.451

4	(0.550) 0.549 0.549

Locus 18557 :

2 alleles

99.2% missing data

1	(0.538) 0.538 0.538

4	(0.462) 0.462 0.462

Locus 18558 :

2 alleles

99.2% missing data

1	(0.498) 0.500 0.491

4	(0.502) 0.500 0.509

Locus 18559 :

2 alleles

98.8% missing data

3	(0.804) 0.803 0.800

1	(0.196) 0.197 0.200

Locus 18560 :

2 alleles

97.3% missing data

3	(0.312) 0.302 0.316

1	(0.688) 0.698 0.684

Locus 18561 :

2 alleles

99.2% missing data

2	(0.450) 0.442 0.457

4	(0.550) 0.558 0.543

Locus 18562 :

2 alleles

8.6% missing data

2	(0.977) 0.979 0.987

3	(0.023) 0.021 0.013

Locus 18563 :

2 alleles

99.2% missing data

4	(0.387) 0.384 0.390

3	(0.613) 0.616 0.610

Locus 18564 :

2 alleles

99.2% missing data

4	(0.663) 0.660 0.666

2	(0.337) 0.340 0.334

Locus 18565 :

2 alleles

98.8% missing data

2	(0.610) 0.607 0.617

3	(0.390) 0.393 0.383

Locus 18566 :

2 alleles

99.2% missing data

3	(0.306) 0.302 0.311

2	(0.694) 0.698 0.689

Locus 18567 :

2 alleles

97.7% missing data

4	(0.776) 0.776 0.781

1	(0.224) 0.224 0.219

Locus 18568 :

2 alleles

98.8% missing data

1	(0.356) 0.346 0.361

4	(0.644) 0.654 0.639

Locus 18569 :

2 alleles

99.2% missing data

4	(0.280) 0.269 0.285

2	(0.720) 0.731 0.715

Locus 18570 :

2 alleles

99.2% missing data

3	(0.584) 0.575 0.584

1	(0.416) 0.425 0.416

Locus 18571 :

2 alleles

99.2% missing data

1	(0.532) 0.539 0.528

2	(0.468) 0.461 0.472

Locus 18572 :

2 alleles

98.4% missing data

2	(0.286) 0.279 0.294

1	(0.714) 0.721 0.706

Locus 18573 :

2 alleles

98.4% missing data

1	(0.790) 0.789 0.785

4	(0.210) 0.211 0.215

Locus 18574 :

2 alleles

99.2% missing data

4	(0.586) 0.589 0.586

3	(0.414) 0.411 0.414

Locus 18575 :

2 alleles

99.2% missing data

3	(0.336) 0.329 0.346

2	(0.664) 0.671 0.654

Locus 18576 :

2 alleles

99.2% missing data

4	(0.783) 0.782 0.780

1	(0.217) 0.218 0.220

Locus 18577 :

2 alleles

99.2% missing data

4	(0.479) 0.477 0.477

2	(0.521) 0.523 0.523

Locus 18578 :

2 alleles

94.9% missing data

3	(0.820) 0.818 0.832

4	(0.180) 0.182 0.168

Locus 18579 :

3 alleles

98.4% missing data

3	(0.444) 0.439 0.452

4	(0.277) 0.279 0.279

1	(0.279) 0.282 0.269

Locus 18580 :

2 alleles

99.2% missing data

4	(0.697) 0.690 0.701

1	(0.303) 0.310 0.299

Locus 18581 :

2 alleles

99.2% missing data

2	(0.300) 0.293 0.310

4	(0.700) 0.707 0.690

Locus 18582 :

2 alleles

97.7% missing data

1	(0.537) 0.542 0.529

4	(0.463) 0.458 0.471

Locus 18583 :

2 alleles

8.2% missing data

2	(0.986) 0.980 0.996

1	(0.014) 0.020 0.004

Locus 18584 :

2 alleles

99.2% missing data

1	(0.696) 0.690 0.694

4	(0.304) 0.310 0.306

Locus 18585 :

2 alleles

99.2% missing data

3	(0.525) 0.518 0.531

4	(0.475) 0.482 0.469

Locus 18586 :

2 alleles

98.8% missing data

2	(0.426) 0.424 0.429

1	(0.574) 0.576 0.571

Locus 18587 :

2 alleles

99.2% missing data

1	(0.535) 0.530 0.533

3	(0.465) 0.470 0.467

Locus 18588 :

2 alleles

97.7% missing data

2	(0.743) 0.749 0.747

1	(0.257) 0.251 0.253

Locus 18589 :

2 alleles

98.4% missing data

1	(0.201) 0.200 0.203

2	(0.799) 0.800 0.797

Locus 18590 :

2 alleles

93.0% missing data

1	(0.915) 0.914 0.921

3	(0.085) 0.086 0.079

Locus 18591 :

2 alleles

97.3% missing data

2	(0.270) 0.274 0.262

3	(0.730) 0.726 0.738

Locus 18592 :

2 alleles

98.8% missing data

3	(0.635) 0.640 0.638

1	(0.365) 0.360 0.362

Locus 18593 :

2 alleles

97.7% missing data

2	(0.740) 0.736 0.751

1	(0.260) 0.264 0.249

Locus 18594 :

3 alleles

98.8% missing data

1	(0.523) 0.527 0.510

3	(0.321) 0.319 0.331

4	(0.155) 0.154 0.158

Locus 18595 :

2 alleles

99.2% missing data

3	(0.662) 0.664 0.657

1	(0.338) 0.336 0.343

Locus 18596 :

2 alleles

99.2% missing data

1	(0.779) 0.782 0.775

2	(0.221) 0.218 0.225

Locus 18597 :

2 alleles

99.2% missing data

2	(0.497) 0.504 0.494

4	(0.503) 0.496 0.506

Locus 18598 :

2 alleles

99.2% missing data

3	(0.627) 0.626 0.636

1	(0.373) 0.374 0.364

Locus 18599 :

2 alleles

99.2% missing data

3	(0.646) 0.641 0.653

2	(0.354) 0.359 0.347

Locus 18600 :

2 alleles

98.8% missing data

1	(0.444) 0.443 0.436

2	(0.556) 0.557 0.564

Locus 18601 :

2 alleles

99.2% missing data

1	(0.660) 0.654 0.667

3	(0.340) 0.346 0.333

Locus 18602 :

2 alleles

93.8% missing data

4	(0.878) 0.878 0.886

1	(0.122) 0.122 0.114

Locus 18603 :

2 alleles

98.8% missing data

2	(0.450) 0.453 0.458

3	(0.550) 0.547 0.542

Locus 18604 :

3 alleles

98.8% missing data

3	(0.169) 0.166 0.173

1	(0.346) 0.345 0.347

2	(0.486) 0.490 0.481

Locus 18605 :

2 alleles

99.2% missing data

3	(0.737) 0.731 0.740

1	(0.263) 0.269 0.260

Locus 18606 :

2 alleles

99.2% missing data

2	(0.253) 0.253 0.261

3	(0.747) 0.747 0.739

Locus 18607 :

2 alleles

99.2% missing data

1	(0.434) 0.434 0.437

3	(0.566) 0.566 0.563

Locus 18608 :

2 alleles

99.2% missing data

2	(0.713) 0.713 0.713

1	(0.287) 0.287 0.287

Locus 18609 :

2 alleles

99.2% missing data

3	(0.578) 0.570 0.583

4	(0.422) 0.430 0.417

Locus 18610 :

2 alleles

91.0% missing data

1	(0.900) 0.912 0.894

4	(0.100) 0.088 0.106

Locus 18611 :

2 alleles

99.2% missing data

4	(0.752) 0.754 0.746

1	(0.248) 0.246 0.254

Locus 18612 :

2 alleles

0.0% missing data

4	(0.981) 0.985 0.988

1	(0.019) 0.015 0.012

Locus 18613 :

2 alleles

96.1% missing data

3	(0.499) 0.493 0.500

4	(0.501) 0.507 0.500

Locus 18614 :

2 alleles

99.2% missing data

2	(0.615) 0.618 0.618

3	(0.385) 0.382 0.382

Locus 18615 :

2 alleles

98.0% missing data

3	(0.709) 0.717 0.703

1	(0.291) 0.283 0.297

Locus 18616 :

2 alleles

99.2% missing data

1	(0.461) 0.462 0.464

2	(0.539) 0.538 0.536

Locus 18617 :

2 alleles

0.8% missing data

4	(0.985) 0.982 0.995

2	(0.015) 0.018 0.005

Locus 18618 :

2 alleles

99.2% missing data

2	(0.644) 0.640 0.642

3	(0.356) 0.360 0.358

Locus 18619 :

2 alleles

98.8% missing data

2	(0.477) 0.473 0.480

4	(0.523) 0.527 0.520

Locus 18620 :

2 alleles

0.0% missing data

3	(0.985) 0.989 0.992

2	(0.015) 0.011 0.008

Locus 18621 :

2 alleles

99.2% missing data

1	(0.564) 0.563 0.563

3	(0.436) 0.437 0.437

Locus 18622 :

2 alleles

99.2% missing data

4	(0.190) 0.185 0.197

1	(0.810) 0.815 0.803

Locus 18623 :

2 alleles

8.2% missing data

3	(0.968) 0.935 0.990

2	(0.032) 0.065 0.010

Locus 18624 :

2 alleles

99.2% missing data

2	(0.326) 0.325 0.326

4	(0.674) 0.675 0.674

Locus 18625 :

2 alleles

96.5% missing data

4	(0.482) 0.469 0.490

2	(0.518) 0.531 0.510

Locus 18626 :

2 alleles

98.8% missing data

3	(0.168) 0.165 0.167

1	(0.832) 0.835 0.833

Locus 18627 :

2 alleles

91.8% missing data

2	(0.871) 0.877 0.880

1	(0.129) 0.123 0.120

Locus 18628 :

2 alleles

99.2% missing data

4	(0.508) 0.507 0.507

3	(0.492) 0.493 0.493

Locus 18629 :

3 alleles

98.8% missing data

4	(0.556) 0.541 0.554

2	(0.158) 0.166 0.162

3	(0.286) 0.293 0.284

Locus 18630 :

2 alleles

99.2% missing data

4	(0.698) 0.698 0.687

1	(0.302) 0.302 0.313

Locus 18631 :

2 alleles

99.2% missing data

4	(0.525) 0.527 0.521

1	(0.475) 0.473 0.479

Locus 18632 :

2 alleles

98.8% missing data

4	(0.811) 0.806 0.813

2	(0.189) 0.194 0.187

Locus 18633 :

2 alleles

99.2% missing data

4	(0.728) 0.729 0.719

3	(0.272) 0.271 0.281

Locus 18634 :

2 alleles

98.8% missing data

4	(0.467) 0.461 0.467

2	(0.533) 0.539 0.533

Locus 18635 :

2 alleles

98.8% missing data

2	(0.312) 0.305 0.317

1	(0.688) 0.695 0.683

Locus 18636 :

2 alleles

99.2% missing data

4	(0.733) 0.734 0.731

3	(0.267) 0.266 0.269

Locus 18637 :

2 alleles

98.4% missing data

2	(0.392) 0.400 0.393

1	(0.608) 0.600 0.607

Locus 18638 :

2 alleles

99.2% missing data

3	(0.455) 0.447 0.465

1	(0.545) 0.553 0.535

Locus 18639 :

2 alleles

99.2% missing data

1	(0.254) 0.254 0.257

4	(0.746) 0.746 0.743

Locus 18640 :

2 alleles

99.2% missing data

2	(0.449) 0.447 0.445

3	(0.551) 0.553 0.555

Locus 18641 :

2 alleles

99.2% missing data

2	(0.569) 0.565 0.581

3	(0.431) 0.435 0.419

Locus 18642 :

2 alleles

99.2% missing data

2	(0.504) 0.498 0.512

1	(0.496) 0.502 0.488

Locus 18643 :

2 alleles

99.2% missing data

1	(0.388) 0.391 0.386

2	(0.612) 0.609 0.614

Locus 18644 :

2 alleles

99.2% missing data

4	(0.668) 0.668 0.669

1	(0.332) 0.332 0.331

Locus 18645 :

2 alleles

99.2% missing data

3	(0.550) 0.549 0.543

4	(0.450) 0.451 0.457

Locus 18646 :

2 alleles

97.7% missing data

3	(0.328) 0.330 0.319

1	(0.672) 0.670 0.681

Locus 18647 :

2 alleles

99.2% missing data

3	(0.430) 0.424 0.436

1	(0.570) 0.576 0.564

Locus 18648 :

2 alleles

99.2% missing data

4	(0.493) 0.501 0.486

1	(0.507) 0.499 0.514

Locus 18649 :

3 alleles

98.0% missing data

4	(0.242) 0.227 0.245

1	(0.577) 0.587 0.574

2	(0.181) 0.186 0.181

Locus 18650 :

2 alleles

99.2% missing data

4	(0.595) 0.590 0.599

3	(0.405) 0.410 0.401

Locus 18651 :

2 alleles

99.2% missing data

1	(0.761) 0.763 0.758

4	(0.239) 0.237 0.242

Locus 18652 :

2 alleles

99.2% missing data

2	(0.350) 0.348 0.356

1	(0.650) 0.652 0.644

Locus 18653 :

2 alleles

99.2% missing data

3	(0.577) 0.581 0.572

2	(0.423) 0.419 0.428

Locus 18654 :

2 alleles

99.2% missing data

1	(0.663) 0.665 0.665

4	(0.337) 0.335 0.335

Locus 18655 :

3 alleles

98.8% missing data

3	(0.175) 0.175 0.174

4	(0.383) 0.381 0.385

2	(0.442) 0.445 0.442

Locus 18656 :

3 alleles

98.4% missing data

4	(0.460) 0.453 0.474

3	(0.217) 0.218 0.211

1	(0.323) 0.330 0.315

Locus 18657 :

2 alleles

99.2% missing data

3	(0.688) 0.688 0.685

1	(0.312) 0.312 0.315

Locus 18658 :

2 alleles

99.2% missing data

2	(0.253) 0.249 0.258

4	(0.747) 0.751 0.742

Locus 18659 :

2 alleles

99.2% missing data

3	(0.618) 0.613 0.616

4	(0.382) 0.387 0.384

Locus 18660 :

2 alleles

9.0% missing data

4	(0.974) 0.951 0.992

2	(0.026) 0.049 0.008

Locus 18661 :

2 alleles

99.2% missing data

2	(0.726) 0.723 0.728

1	(0.274) 0.277 0.272

Locus 18662 :

2 alleles

0.4% missing data

1	(0.989) 0.984 0.996

2	(0.011) 0.016 0.004

Locus 18663 :

2 alleles

99.2% missing data

2	(0.638) 0.633 0.644

1	(0.362) 0.367 0.356

Locus 18664 :

2 alleles

99.2% missing data

2	(0.491) 0.489 0.494

4	(0.509) 0.511 0.506

Locus 18665 :

2 alleles

98.4% missing data

1	(0.263) 0.263 0.279

2	(0.737) 0.737 0.721

Locus 18666 :

2 alleles

98.4% missing data

2	(0.197) 0.203 0.195

1	(0.803) 0.797 0.805

Locus 18667 :

3 alleles

98.4% missing data

2	(0.581) 0.578 0.576

4	(0.144) 0.135 0.154

3	(0.275) 0.288 0.269

Locus 18668 :

2 alleles

97.7% missing data

1	(0.718) 0.718 0.720

3	(0.282) 0.282 0.280

Locus 18669 :

2 alleles

90.2% missing data

2	(0.886) 0.907 0.865

1	(0.114) 0.093 0.135

Locus 18670 :

2 alleles

0.0% missing data

2	(0.987) 0.989 0.992

3	(0.013) 0.011 0.008

Locus 18671 :

3 alleles

98.8% missing data

2	(0.473) 0.473 0.470

3	(0.194) 0.186 0.203

4	(0.333) 0.341 0.327

Locus 18672 :

3 alleles

98.8% missing data

2	(0.233) 0.224 0.236

3	(0.266) 0.265 0.264

4	(0.501) 0.511 0.500

Locus 18673 :

2 alleles

99.2% missing data

3	(0.384) 0.386 0.383

2	(0.616) 0.614 0.617

Locus 18674 :

3 alleles

98.8% missing data

3	(0.640) 0.641 0.626

1	(0.194) 0.193 0.199

4	(0.167) 0.166 0.174

Locus 18675 :

3 alleles

98.8% missing data

3	(0.286) 0.288 0.289

1	(0.261) 0.257 0.262

2	(0.453) 0.454 0.449

Locus 18676 :

2 alleles

99.2% missing data

3	(0.707) 0.701 0.713

1	(0.293) 0.299 0.287

Locus 18677 :

2 alleles

99.2% missing data

3	(0.612) 0.619 0.607

2	(0.388) 0.381 0.393

Locus 18678 :

2 alleles

99.2% missing data

2	(0.436) 0.436 0.432

1	(0.564) 0.564 0.568

Locus 18679 :

2 alleles

98.8% missing data

2	(0.231) 0.232 0.225

3	(0.769) 0.768 0.775

Locus 18680 :

2 alleles

99.2% missing data

1	(0.810) 0.813 0.804

3	(0.190) 0.187 0.196

Locus 18681 :

2 alleles

96.1% missing data

4	(0.199) 0.186 0.196

1	(0.801) 0.814 0.804

Locus 18682 :

2 alleles

99.2% missing data

1	(0.873) 0.878 0.866

3	(0.127) 0.122 0.134

Locus 18683 :

2 alleles

99.2% missing data

1	(0.397) 0.408 0.395

2	(0.603) 0.592 0.605

Locus 18684 :

2 alleles

8.6% missing data

2	(0.985) 0.970 0.995

1	(0.015) 0.030 0.005

Locus 18685 :

2 alleles

98.4% missing data

3	(0.373) 0.363 0.385

2	(0.627) 0.637 0.615

Locus 18686 :

2 alleles

89.1% missing data

4	(0.851) 0.881 0.817

3	(0.149) 0.119 0.183

Locus 18687 :

2 alleles

99.2% missing data

1	(0.744) 0.742 0.742

4	(0.256) 0.258 0.258

Locus 18688 :

2 alleles

96.9% missing data

4	(0.659) 0.649 0.682

1	(0.341) 0.351 0.318

Locus 18689 :

2 alleles

99.2% missing data

1	(0.459) 0.451 0.467

3	(0.541) 0.549 0.533

Locus 18690 :

2 alleles

99.2% missing data

3	(0.330) 0.332 0.329

2	(0.670) 0.668 0.671

Locus 18691 :

3 alleles

98.8% missing data

1	(0.370) 0.378 0.361

2	(0.372) 0.367 0.375

3	(0.258) 0.255 0.264

Locus 18692 :

2 alleles

97.3% missing data

2	(0.805) 0.799 0.820

4	(0.195) 0.201 0.180

Locus 18693 :

2 alleles

99.2% missing data

3	(0.498) 0.503 0.491

1	(0.502) 0.497 0.509

Locus 18694 :

2 alleles

8.6% missing data

1	(0.986) 0.980 0.995

4	(0.014) 0.020 0.005

Locus 18695 :

2 alleles

98.8% missing data

1	(0.622) 0.626 0.624

3	(0.378) 0.374 0.376

Locus 18696 :

3 alleles

98.8% missing data

1	(0.457) 0.450 0.461

3	(0.235) 0.240 0.230

2	(0.308) 0.309 0.309

Locus 18697 :

2 alleles

90.6% missing data

2	(0.890) 0.907 0.883

3	(0.110) 0.093 0.117

Locus 18698 :

2 alleles

98.8% missing data

1	(0.500) 0.502 0.507

2	(0.500) 0.498 0.493

Locus 18699 :

2 alleles

95.7% missing data

4	(0.237) 0.226 0.246

1	(0.763) 0.774 0.754

Locus 18700 :

2 alleles

97.7% missing data

3	(0.843) 0.835 0.851

1	(0.157) 0.165 0.149

Locus 18701 :

2 alleles

99.2% missing data

2	(0.236) 0.233 0.243

1	(0.764) 0.767 0.757

Locus 18702 :

2 alleles

97.3% missing data

3	(0.624) 0.632 0.628

4	(0.376) 0.368 0.372

Locus 18703 :

3 alleles

98.8% missing data

3	(0.148) 0.146 0.152

4	(0.552) 0.553 0.545

2	(0.300) 0.301 0.303

Locus 18704 :

2 alleles

99.2% missing data

3	(0.437) 0.426 0.444

1	(0.563) 0.574 0.556

Locus 18705 :

2 alleles

99.2% missing data

1	(0.625) 0.625 0.629

4	(0.375) 0.375 0.371

Locus 18706 :

2 alleles

99.2% missing data

2	(0.594) 0.599 0.592

3	(0.406) 0.401 0.408

Locus 18707 :

2 alleles

99.2% missing data

1	(0.236) 0.232 0.238

2	(0.764) 0.768 0.762

Locus 18708 :

2 alleles

99.2% missing data

3	(0.765) 0.758 0.768

1	(0.235) 0.242 0.232

Locus 18709 :

2 alleles

97.7% missing data

1	(0.283) 0.284 0.282

4	(0.717) 0.716 0.718

Locus 18710 :

2 alleles

97.3% missing data

1	(0.222) 0.232 0.214

2	(0.778) 0.768 0.786

Locus 18711 :

2 alleles

98.0% missing data

4	(0.619) 0.631 0.614

2	(0.381) 0.369 0.386

Locus 18712 :

2 alleles

91.0% missing data

4	(0.900) 0.917 0.892

3	(0.100) 0.083 0.108

Locus 18713 :

2 alleles

99.2% missing data

2	(0.714) 0.708 0.712

4	(0.286) 0.292 0.288

Locus 18714 :

2 alleles

93.0% missing data

3	(0.916) 0.916 0.921

2	(0.084) 0.084 0.079

Locus 18715 :

3 alleles

98.4% missing data

1	(0.605) 0.607 0.608

4	(0.154) 0.162 0.149

3	(0.241) 0.230 0.243

Locus 18716 :

2 alleles

97.3% missing data

3	(0.704) 0.700 0.717

4	(0.296) 0.300 0.283

Locus 18717 :

2 alleles

94.9% missing data

1	(0.632) 0.622 0.654

3	(0.368) 0.378 0.346

Locus 18718 :

2 alleles

98.8% missing data

3	(0.564) 0.570 0.563

4	(0.436) 0.430 0.437

Locus 18719 :

2 alleles

99.2% missing data

3	(0.465) 0.461 0.463

1	(0.535) 0.539 0.537

Locus 18720 :

2 alleles

99.2% missing data

1	(0.381) 0.376 0.381

4	(0.619) 0.624 0.619

Locus 18721 :

3 alleles

96.5% missing data

4	(0.377) 0.372 0.384

1	(0.163) 0.157 0.162

3	(0.460) 0.472 0.454

Locus 18722 :

2 alleles

96.9% missing data

4	(0.895) 0.901 0.895

3	(0.105) 0.099 0.105

Locus 18723 :

2 alleles

96.9% missing data

2	(0.273) 0.261 0.278

4	(0.727) 0.739 0.722

Locus 18724 :

2 alleles

97.3% missing data

3	(0.759) 0.757 0.771

4	(0.241) 0.243 0.229

Locus 18725 :

3 alleles

98.8% missing data

3	(0.421) 0.421 0.430

1	(0.336) 0.335 0.333

4	(0.243) 0.244 0.237

Locus 18726 :

2 alleles

99.2% missing data

2	(0.678) 0.674 0.681

4	(0.322) 0.326 0.319

Locus 18727 :

2 alleles

97.7% missing data

2	(0.811) 0.815 0.809

3	(0.189) 0.185 0.191

Locus 18728 :

2 alleles

98.4% missing data

4	(0.528) 0.530 0.521

3	(0.472) 0.470 0.479

Locus 18729 :

2 alleles

99.2% missing data

3	(0.283) 0.286 0.283

4	(0.717) 0.714 0.717

Locus 18730 :

2 alleles

99.2% missing data

2	(0.603) 0.602 0.607

3	(0.397) 0.398 0.393

Locus 18731 :

2 alleles

82.4% missing data

3	(0.425) 0.499 0.360

1	(0.575) 0.501 0.640

Locus 18732 :

2 alleles

98.0% missing data

4	(0.717) 0.713 0.729

1	(0.283) 0.287 0.271

Locus 18733 :

2 alleles

99.2% missing data

4	(0.530) 0.521 0.537

1	(0.470) 0.479 0.463

Locus 18734 :

2 alleles

99.2% missing data

1	(0.529) 0.521 0.533

2	(0.471) 0.479 0.467

Locus 18735 :

2 alleles

99.2% missing data

2	(0.585) 0.582 0.582

3	(0.415) 0.418 0.418

Locus 18736 :

2 alleles

98.8% missing data

1	(0.359) 0.369 0.352

3	(0.641) 0.631 0.648

Locus 18737 :

2 alleles

99.2% missing data

2	(0.324) 0.327 0.317

3	(0.676) 0.673 0.683

Locus 18738 :

2 alleles

99.2% missing data

1	(0.517) 0.510 0.521

4	(0.483) 0.490 0.479

Locus 18739 :

2 alleles

99.2% missing data

3	(0.730) 0.722 0.735

4	(0.270) 0.278 0.265

Locus 18740 :

2 alleles

99.2% missing data

1	(0.485) 0.491 0.481

3	(0.515) 0.509 0.519

Locus 18741 :

2 alleles

99.2% missing data

2	(0.432) 0.435 0.430

4	(0.568) 0.565 0.570

Locus 18742 :

2 alleles

90.6% missing data

1	(0.932) 0.938 0.933

3	(0.068) 0.062 0.067

Locus 18743 :

2 alleles

99.2% missing data

4	(0.557) 0.551 0.565

3	(0.443) 0.449 0.435

Locus 18744 :

2 alleles

99.2% missing data

1	(0.574) 0.581 0.566

4	(0.426) 0.419 0.434

Locus 18745 :

2 alleles

98.0% missing data

3	(0.766) 0.771 0.769

2	(0.234) 0.229 0.231

Locus 18746 :

2 alleles

96.5% missing data

4	(0.720) 0.740 0.712

1	(0.280) 0.260 0.288

Locus 18747 :

2 alleles

96.9% missing data

4	(0.593) 0.598 0.587

3	(0.407) 0.402 0.413

Locus 18748 :

2 alleles

98.8% missing data

3	(0.505) 0.510 0.504

4	(0.495) 0.490 0.496

Locus 18749 :

2 alleles

0.0% missing data

4	(0.988) 0.991 0.994

1	(0.012) 0.009 0.006

Locus 18750 :

2 alleles

99.2% missing data

1	(0.306) 0.302 0.310

3	(0.694) 0.698 0.690

Locus 18751 :

2 alleles

99.2% missing data

4	(0.696) 0.702 0.689

1	(0.304) 0.298 0.311

Locus 18752 :

2 alleles

98.8% missing data

4	(0.672) 0.668 0.678

2	(0.328) 0.332 0.322

Locus 18753 :

2 alleles

99.2% missing data

3	(0.693) 0.696 0.690

4	(0.307) 0.304 0.310

Locus 18754 :

2 alleles

97.7% missing data

1	(0.735) 0.732 0.745

4	(0.265) 0.268 0.255

Locus 18755 :

2 alleles

99.2% missing data

1	(0.704) 0.705 0.708

2	(0.296) 0.295 0.292

Locus 18756 :

2 alleles

98.8% missing data

1	(0.623) 0.614 0.624

4	(0.377) 0.386 0.376

Locus 18757 :

2 alleles

93.0% missing data

4	(0.824) 0.816 0.849

2	(0.176) 0.184 0.151

Locus 18758 :

2 alleles

99.2% missing data

3	(0.459) 0.459 0.455

1	(0.541) 0.541 0.545

Locus 18759 :

2 alleles

99.2% missing data

1	(0.202) 0.197 0.209

3	(0.798) 0.803 0.791

Locus 18760 :

2 alleles

98.8% missing data

4	(0.136) 0.132 0.148

3	(0.864) 0.868 0.852

Locus 18761 :

2 alleles

98.8% missing data

2	(0.551) 0.551 0.564

3	(0.449) 0.449 0.436

Locus 18762 :

2 alleles

99.2% missing data

4	(0.519) 0.520 0.521

3	(0.481) 0.480 0.479

Locus 18763 :

2 alleles

99.2% missing data

1	(0.269) 0.274 0.270

4	(0.731) 0.726 0.730

Locus 18764 :

2 alleles

99.2% missing data

3	(0.561) 0.549 0.568

1	(0.439) 0.451 0.432

Locus 18765 :

3 alleles

98.8% missing data

2	(0.255) 0.258 0.259

3	(0.442) 0.440 0.440

1	(0.303) 0.302 0.301

Locus 18766 :

2 alleles

99.2% missing data

4	(0.328) 0.328 0.330

3	(0.672) 0.672 0.670

Locus 18767 :

2 alleles

96.1% missing data

2	(0.821) 0.832 0.816

4	(0.179) 0.168 0.184

Locus 18768 :

2 alleles

99.2% missing data

1	(0.456) 0.450 0.455

3	(0.544) 0.550 0.545

Locus 18769 :

2 alleles

98.0% missing data

4	(0.804) 0.797 0.817

1	(0.196) 0.203 0.183

Locus 18770 :

2 alleles

96.9% missing data

3	(0.796) 0.789 0.809

2	(0.204) 0.211 0.191

Locus 18771 :

2 alleles

99.2% missing data

3	(0.655) 0.646 0.657

2	(0.345) 0.354 0.343

Locus 18772 :

2 alleles

99.2% missing data

3	(0.411) 0.407 0.415

2	(0.589) 0.593 0.585

Locus 18773 :

2 alleles

99.2% missing data

1	(0.707) 0.712 0.704

4	(0.293) 0.288 0.296

Locus 18774 :

2 alleles

97.7% missing data

2	(0.483) 0.483 0.467

3	(0.517) 0.517 0.533

Locus 18775 :

2 alleles

91.0% missing data

3	(0.900) 0.919 0.892

4	(0.100) 0.081 0.108

Locus 18776 :

2 alleles

96.9% missing data

1	(0.797) 0.798 0.805

4	(0.203) 0.202 0.195

Locus 18777 :

2 alleles

94.5% missing data

3	(0.868) 0.868 0.872

2	(0.132) 0.132 0.128

Locus 18778 :

2 alleles

99.2% missing data

3	(0.401) 0.409 0.389

1	(0.599) 0.591 0.611

Locus 18779 :

2 alleles

98.4% missing data

4	(0.461) 0.456 0.469

3	(0.539) 0.544 0.531

Locus 18780 :

3 alleles

90.6% missing data

3	(0.845) 0.873 0.833

1	(0.077) 0.062 0.083

2	(0.078) 0.064 0.084

Locus 18781 :

2 alleles

99.2% missing data

4	(0.369) 0.370 0.368

1	(0.631) 0.630 0.632

Locus 18782 :

3 alleles

98.0% missing data

2	(0.140) 0.140 0.142

4	(0.395) 0.394 0.402

1	(0.464) 0.466 0.456

Locus 18783 :

2 alleles

99.2% missing data

1	(0.362) 0.368 0.355

4	(0.638) 0.632 0.645

Locus 18784 :

2 alleles

90.6% missing data

3	(0.887) 0.903 0.884

4	(0.113) 0.097 0.116

Locus 18785 :

2 alleles

98.8% missing data

4	(0.342) 0.336 0.339

2	(0.658) 0.664 0.661

Locus 18786 :

2 alleles

99.2% missing data

4	(0.427) 0.424 0.429

1	(0.573) 0.576 0.571

Locus 18787 :

2 alleles

99.2% missing data

1	(0.376) 0.375 0.380

3	(0.624) 0.625 0.620

Locus 18788 :

2 alleles

99.2% missing data

3	(0.198) 0.200 0.201

2	(0.802) 0.800 0.799

Locus 18789 :

2 alleles

98.0% missing data

4	(0.273) 0.263 0.279

2	(0.727) 0.737 0.721

Locus 18790 :

2 alleles

99.2% missing data

3	(0.434) 0.432 0.438

1	(0.566) 0.568 0.562

Locus 18791 :

2 alleles

98.8% missing data

1	(0.319) 0.313 0.325

3	(0.681) 0.687 0.675

Locus 18792 :

2 alleles

98.0% missing data

3	(0.433) 0.428 0.432

2	(0.567) 0.572 0.568

Locus 18793 :

2 alleles

98.8% missing data

2	(0.420) 0.417 0.418

3	(0.580) 0.583 0.582

Locus 18794 :

2 alleles

98.8% missing data

2	(0.564) 0.565 0.553

4	(0.436) 0.435 0.447

Locus 18795 :

2 alleles

99.2% missing data

3	(0.493) 0.498 0.495

2	(0.507) 0.502 0.505

Locus 18796 :

2 alleles

98.8% missing data

2	(0.734) 0.736 0.734

4	(0.266) 0.264 0.266

Locus 18797 :

2 alleles

99.2% missing data

3	(0.270) 0.278 0.273

1	(0.730) 0.722 0.727

Locus 18798 :

4 alleles

98.4% missing data

1	(0.253) 0.251 0.257

2	(0.375) 0.376 0.366

4	(0.235) 0.232 0.242

3	(0.137) 0.142 0.135

Locus 18799 :

2 alleles

98.8% missing data

3	(0.201) 0.197 0.204

1	(0.799) 0.803 0.796

Locus 18800 :

2 alleles

98.4% missing data

3	(0.807) 0.808 0.801

4	(0.193) 0.192 0.199

Locus 18801 :

3 alleles

95.3% missing data

1	(0.114) 0.118 0.106

3	(0.513) 0.512 0.516

2	(0.373) 0.371 0.379

Locus 18802 :

2 alleles

99.2% missing data

2	(0.532) 0.526 0.539

1	(0.468) 0.474 0.461

Locus 18803 :

2 alleles

99.2% missing data

3	(0.662) 0.666 0.658

4	(0.338) 0.334 0.342

Locus 18804 :

2 alleles

98.4% missing data

4	(0.581) 0.591 0.578

2	(0.419) 0.409 0.422

Locus 18805 :

3 alleles

98.8% missing data

2	(0.204) 0.196 0.208

3	(0.379) 0.386 0.371

4	(0.417) 0.418 0.420

Locus 18806 :

2 alleles

97.7% missing data

1	(0.860) 0.853 0.868

3	(0.140) 0.147 0.132

Locus 18807 :

2 alleles

99.2% missing data

2	(0.387) 0.385 0.399

4	(0.613) 0.615 0.601

Locus 18808 :

3 alleles

98.4% missing data

4	(0.246) 0.240 0.243

2	(0.372) 0.377 0.375

1	(0.383) 0.383 0.383

Locus 18809 :

2 alleles

98.8% missing data

3	(0.486) 0.484 0.486

2	(0.514) 0.516 0.514

Locus 18810 :

2 alleles

99.2% missing data

4	(0.723) 0.724 0.722

3	(0.277) 0.276 0.278

Locus 18811 :

2 alleles

99.2% missing data

3	(0.430) 0.427 0.432

4	(0.570) 0.573 0.568

Locus 18812 :

2 alleles

99.2% missing data

3	(0.280) 0.286 0.280

2	(0.720) 0.714 0.720

Locus 18813 :

2 alleles

98.0% missing data

4	(0.753) 0.756 0.752

1	(0.247) 0.244 0.248

Locus 18814 :

2 alleles

99.2% missing data

3	(0.632) 0.628 0.633

1	(0.368) 0.372 0.367

Locus 18815 :

3 alleles

98.4% missing data

3	(0.353) 0.350 0.350

4	(0.265) 0.267 0.269

1	(0.383) 0.383 0.381

Locus 18816 :

2 alleles

99.2% missing data

4	(0.326) 0.326 0.330

3	(0.674) 0.674 0.670

Locus 18817 :

2 alleles

99.2% missing data

3	(0.342) 0.335 0.347

4	(0.658) 0.665 0.653

Locus 18818 :

2 alleles

96.9% missing data

1	(0.800) 0.815 0.794

4	(0.200) 0.185 0.206

Locus 18819 :

2 alleles

99.2% missing data

3	(0.380) 0.370 0.381

1	(0.620) 0.630 0.619

Locus 18820 :

2 alleles

91.0% missing data

2	(0.896) 0.910 0.889

3	(0.104) 0.090 0.111

Locus 18821 :

2 alleles

99.2% missing data

1	(0.320) 0.323 0.315

3	(0.680) 0.677 0.685

Locus 18822 :

2 alleles

96.9% missing data

2	(0.840) 0.835 0.852

3	(0.160) 0.165 0.148

Locus 18823 :

3 alleles

98.4% missing data

1	(0.487) 0.489 0.488

4	(0.165) 0.164 0.172

2	(0.348) 0.347 0.340

Locus 18824 :

2 alleles

99.2% missing data

1	(0.474) 0.471 0.480

4	(0.526) 0.529 0.520

Locus 18825 :

2 alleles

99.2% missing data

3	(0.609) 0.604 0.610

1	(0.391) 0.396 0.390

Locus 18826 :

3 alleles

98.8% missing data

1	(0.417) 0.413 0.424

3	(0.257) 0.253 0.254

2	(0.326) 0.334 0.322

Locus 18827 :

2 alleles

99.2% missing data

2	(0.604) 0.605 0.602

1	(0.396) 0.395 0.398

Locus 18828 :

2 alleles

99.2% missing data

1	(0.682) 0.680 0.675

4	(0.318) 0.320 0.325

Locus 18829 :

2 alleles

99.2% missing data

3	(0.218) 0.211 0.221

4	(0.782) 0.789 0.779

Locus 18830 :

2 alleles

99.2% missing data

1	(0.503) 0.503 0.503

2	(0.497) 0.497 0.497

Locus 18831 :

3 alleles

98.8% missing data

1	(0.232) 0.230 0.241

4	(0.349) 0.349 0.348

2	(0.419) 0.421 0.411

Locus 18832 :

2 alleles

98.8% missing data

3	(0.577) 0.582 0.581

1	(0.423) 0.418 0.419

Locus 18833 :

2 alleles

99.2% missing data

2	(0.370) 0.368 0.368

1	(0.630) 0.632 0.632

Locus 18834 :

2 alleles

96.1% missing data

3	(0.705) 0.699 0.720

1	(0.295) 0.301 0.280

Locus 18835 :

2 alleles

99.2% missing data

4	(0.461) 0.469 0.459

3	(0.539) 0.531 0.541

Locus 18836 :

2 alleles

99.2% missing data

4	(0.800) 0.801 0.800

1	(0.200) 0.199 0.200

Locus 18837 :

2 alleles

99.2% missing data

2	(0.573) 0.578 0.569

3	(0.427) 0.422 0.431

Locus 18838 :

2 alleles

99.2% missing data

1	(0.350) 0.348 0.350

3	(0.650) 0.652 0.650

Locus 18839 :

2 alleles

99.2% missing data

1	(0.461) 0.453 0.465

2	(0.539) 0.547 0.535

Locus 18840 :

2 alleles

98.4% missing data

2	(0.380) 0.385 0.374

1	(0.620) 0.615 0.626

Locus 18841 :

2 alleles

99.2% missing data

3	(0.651) 0.659 0.648

1	(0.349) 0.341 0.352

Locus 18842 :

2 alleles

99.2% missing data

1	(0.424) 0.423 0.425

3	(0.576) 0.577 0.575

Locus 18843 :

2 alleles

98.4% missing data

3	(0.778) 0.778 0.778

1	(0.222) 0.222 0.222

Locus 18844 :

2 alleles

98.8% missing data

3	(0.540) 0.542 0.529

2	(0.460) 0.458 0.471

Locus 18845 :

2 alleles

98.8% missing data

2	(0.635) 0.628 0.642

3	(0.365) 0.372 0.358

Locus 18846 :

2 alleles

98.4% missing data

2	(0.456) 0.458 0.463

1	(0.544) 0.542 0.537

Locus 18847 :

2 alleles

99.2% missing data

1	(0.455) 0.454 0.451

2	(0.545) 0.546 0.549

Locus 18848 :

2 alleles

90.2% missing data

1	(0.815) 0.827 0.815

2	(0.185) 0.173 0.185

Locus 18849 :

2 alleles

99.2% missing data

2	(0.435) 0.433 0.434

3	(0.565) 0.567 0.566

Locus 18850 :

2 alleles

99.2% missing data

2	(0.567) 0.560 0.574

1	(0.433) 0.440 0.426

Locus 18851 :

2 alleles

99.2% missing data

3	(0.472) 0.466 0.479

1	(0.528) 0.534 0.521

Locus 18852 :

2 alleles

99.2% missing data

4	(0.460) 0.462 0.465

3	(0.540) 0.538 0.535

Locus 18853 :

2 alleles

99.2% missing data

4	(0.508) 0.509 0.509

1	(0.492) 0.491 0.491

Locus 18854 :

2 alleles

98.8% missing data

2	(0.275) 0.289 0.275

1	(0.725) 0.711 0.725

Locus 18855 :

2 alleles

99.2% missing data

3	(0.831) 0.825 0.834

1	(0.169) 0.175 0.166

Locus 18856 :

3 alleles

97.3% missing data

4	(0.155) 0.155 0.150

3	(0.509) 0.501 0.531

2	(0.336) 0.344 0.319

Locus 18857 :

2 alleles

99.2% missing data

4	(0.650) 0.639 0.658

3	(0.350) 0.361 0.342

Locus 18858 :

2 alleles

98.8% missing data

3	(0.307) 0.299 0.317

4	(0.693) 0.701 0.683

Locus 18859 :

2 alleles

96.9% missing data

3	(0.790) 0.791 0.800

1	(0.210) 0.209 0.200

Locus 18860 :

2 alleles

99.2% missing data

3	(0.747) 0.746 0.748

1	(0.253) 0.254 0.252

Locus 18861 :

2 alleles

99.2% missing data

2	(0.549) 0.550 0.550

1	(0.451) 0.450 0.450

Locus 18862 :

2 alleles

99.2% missing data

2	(0.736) 0.732 0.736

1	(0.264) 0.268 0.264

Locus 18863 :

2 alleles

91.0% missing data

4	(0.922) 0.929 0.922

2	(0.078) 0.071 0.078

Locus 18864 :

3 alleles

98.8% missing data

2	(0.200) 0.201 0.203

4	(0.469) 0.468 0.471

1	(0.332) 0.331 0.326

Locus 18865 :

2 alleles

99.2% missing data

4	(0.751) 0.750 0.758

3	(0.249) 0.250 0.242

Locus 18866 :

2 alleles

99.2% missing data

2	(0.372) 0.378 0.369

3	(0.628) 0.622 0.631

Locus 18867 :

2 alleles

99.2% missing data

1	(0.722) 0.725 0.719

3	(0.278) 0.275 0.281

Locus 18868 :

2 alleles

98.8% missing data

4	(0.393) 0.391 0.401

1	(0.607) 0.609 0.599

Locus 18869 :

3 alleles

98.8% missing data

4	(0.581) 0.580 0.576

1	(0.121) 0.120 0.125

3	(0.298) 0.301 0.299

Locus 18870 :

2 alleles

94.9% missing data

3	(0.706) 0.724 0.679

2	(0.294) 0.276 0.321

Locus 18871 :

2 alleles

99.2% missing data

3	(0.739) 0.736 0.746

1	(0.261) 0.264 0.254

Locus 18872 :

2 alleles

99.2% missing data

3	(0.665) 0.667 0.664

2	(0.335) 0.333 0.336

Locus 18873 :

2 alleles

99.2% missing data

1	(0.254) 0.252 0.260

3	(0.746) 0.748 0.740

Locus 18874 :

2 alleles

99.2% missing data

2	(0.212) 0.208 0.213

3	(0.788) 0.792 0.787

Locus 18875 :

2 alleles

96.1% missing data

2	(0.185) 0.183 0.188

4	(0.815) 0.817 0.812

Locus 18876 :

2 alleles

99.2% missing data

1	(0.681) 0.680 0.684

3	(0.319) 0.320 0.316

Locus 18877 :

2 alleles

99.2% missing data

2	(0.622) 0.616 0.625

4	(0.378) 0.384 0.375

Locus 18878 :

2 alleles

99.2% missing data

1	(0.265) 0.259 0.271

3	(0.735) 0.741 0.729

Locus 18879 :

2 alleles

99.2% missing data

1	(0.417) 0.408 0.429

4	(0.583) 0.592 0.571

Locus 18880 :

2 alleles

99.2% missing data

2	(0.407) 0.410 0.412

3	(0.593) 0.590 0.588

Locus 18881 :

2 alleles

99.2% missing data

3	(0.436) 0.447 0.427

1	(0.564) 0.553 0.573

Locus 18882 :

2 alleles

99.2% missing data

3	(0.434) 0.431 0.432

4	(0.566) 0.569 0.568

Locus 18883 :

2 alleles

99.2% missing data

1	(0.495) 0.493 0.497

3	(0.505) 0.507 0.503

Locus 18884 :

2 alleles

99.2% missing data

1	(0.544) 0.547 0.543

3	(0.456) 0.453 0.457

Locus 18885 :

2 alleles

91.0% missing data

4	(0.918) 0.933 0.909

1	(0.082) 0.067 0.091

Locus 18886 :

2 alleles

99.2% missing data

4	(0.756) 0.750 0.756

3	(0.244) 0.250 0.244

Locus 18887 :

2 alleles

99.2% missing data

1	(0.404) 0.404 0.401

3	(0.596) 0.596 0.599

Locus 18888 :

2 alleles

99.2% missing data

4	(0.445) 0.446 0.440

2	(0.555) 0.554 0.560

Locus 18889 :

2 alleles

99.2% missing data

1	(0.574) 0.577 0.568

2	(0.426) 0.423 0.432

Locus 18890 :

2 alleles

99.2% missing data

1	(0.504) 0.504 0.507

3	(0.496) 0.496 0.493

Locus 18891 :

2 alleles

91.4% missing data

4	(0.939) 0.951 0.929

2	(0.061) 0.049 0.071

Locus 18892 :

2 alleles

98.8% missing data

4	(0.555) 0.549 0.555

2	(0.445) 0.451 0.445

Locus 18893 :

3 alleles

98.4% missing data

4	(0.195) 0.196 0.194

2	(0.439) 0.436 0.443

3	(0.366) 0.368 0.363

Locus 18894 :

2 alleles

98.8% missing data

1	(0.531) 0.527 0.539

4	(0.469) 0.473 0.461

Locus 18895 :

2 alleles

99.2% missing data

1	(0.395) 0.393 0.394

3	(0.605) 0.607 0.606

Locus 18896 :

2 alleles

99.2% missing data

1	(0.476) 0.479 0.475

3	(0.524) 0.521 0.525

Locus 18897 :

2 alleles

98.4% missing data

1	(0.593) 0.600 0.586

3	(0.407) 0.400 0.414

Locus 18898 :

2 alleles

98.8% missing data

4	(0.581) 0.583 0.584

1	(0.419) 0.417 0.416

Locus 18899 :

2 alleles

98.4% missing data

1	(0.596) 0.599 0.595

2	(0.404) 0.401 0.405

Locus 18900 :

2 alleles

99.2% missing data

3	(0.625) 0.626 0.614

1	(0.375) 0.374 0.386

Locus 18901 :

2 alleles

8.6% missing data

1	(0.988) 0.984 0.996

4	(0.012) 0.016 0.004

Locus 18902 :

3 alleles

98.8% missing data

1	(0.373) 0.379 0.368

2	(0.315) 0.308 0.325

3	(0.312) 0.313 0.307

Locus 18903 :

2 alleles

99.2% missing data

3	(0.691) 0.680 0.694

2	(0.309) 0.320 0.306

Locus 18904 :

2 alleles

99.2% missing data

1	(0.594) 0.603 0.591

4	(0.406) 0.397 0.409

Locus 18905 :

2 alleles

98.8% missing data

4	(0.515) 0.516 0.522

3	(0.485) 0.484 0.478

Locus 18906 :

2 alleles

99.2% missing data

3	(0.418) 0.421 0.411

2	(0.582) 0.579 0.589

Locus 18907 :

2 alleles

99.2% missing data

4	(0.166) 0.163 0.166

1	(0.834) 0.837 0.834

Locus 18908 :

2 alleles

99.2% missing data

3	(0.597) 0.597 0.595

4	(0.403) 0.403 0.405

Locus 18909 :

2 alleles

99.2% missing data

2	(0.419) 0.433 0.417

4	(0.581) 0.567 0.583

Locus 18910 :

2 alleles

98.4% missing data

4	(0.711) 0.702 0.713

1	(0.289) 0.298 0.287

Locus 18911 :

2 alleles

99.2% missing data

4	(0.556) 0.549 0.559

1	(0.444) 0.451 0.441

Locus 18912 :

2 alleles

99.2% missing data

3	(0.553) 0.549 0.554

1	(0.447) 0.451 0.446

Locus 18913 :

2 alleles

99.2% missing data

1	(0.272) 0.273 0.281

3	(0.728) 0.727 0.719

Locus 18914 :

2 alleles

99.2% missing data

1	(0.472) 0.469 0.476

3	(0.528) 0.531 0.524

Locus 18915 :

2 alleles

97.7% missing data

1	(0.654) 0.654 0.665

3	(0.346) 0.346 0.335

Locus 18916 :

2 alleles

98.8% missing data

2	(0.457) 0.461 0.458

1	(0.543) 0.539 0.542

Locus 18917 :

2 alleles

99.2% missing data

2	(0.396) 0.389 0.403

3	(0.604) 0.611 0.597

Locus 18918 :

2 alleles

99.2% missing data

4	(0.703) 0.703 0.703

3	(0.297) 0.297 0.297

Locus 18919 :

3 alleles

92.2% missing data

1	(0.755) 0.742 0.780

3	(0.137) 0.142 0.129

2	(0.108) 0.117 0.091

Locus 18920 :

2 alleles

97.3% missing data

2	(0.319) 0.328 0.317

4	(0.681) 0.672 0.683

Locus 18921 :

2 alleles

99.2% missing data

4	(0.344) 0.344 0.346

3	(0.656) 0.656 0.654

Locus 18922 :

2 alleles

99.2% missing data

1	(0.472) 0.469 0.482

4	(0.528) 0.531 0.518

Locus 18923 :

2 alleles

99.2% missing data

1	(0.300) 0.304 0.304

3	(0.700) 0.696 0.696

Locus 18924 :

2 alleles

99.2% missing data

3	(0.313) 0.320 0.307

4	(0.687) 0.680 0.693

Locus 18925 :

2 alleles

91.0% missing data

2	(0.082) 0.069 0.089

3	(0.918) 0.931 0.911

Locus 18926 :

2 alleles

99.2% missing data

2	(0.420) 0.417 0.423

3	(0.580) 0.583 0.577

Locus 18927 :

2 alleles

98.4% missing data

2	(0.655) 0.658 0.659

4	(0.345) 0.342 0.341

Locus 18928 :

2 alleles

99.2% missing data

2	(0.674) 0.673 0.674

1	(0.326) 0.327 0.326

Locus 18929 :

2 alleles

7.8% missing data

3	(0.976) 0.952 0.992

1	(0.024) 0.048 0.008

Locus 18930 :

2 alleles

99.2% missing data

1	(0.314) 0.322 0.314

3	(0.686) 0.678 0.686

Locus 18931 :

2 alleles

98.8% missing data

1	(0.586) 0.586 0.592

4	(0.414) 0.414 0.408

Locus 18932 :

3 alleles

98.4% missing data

1	(0.599) 0.593 0.600

2	(0.150) 0.155 0.149

3	(0.251) 0.251 0.251

Locus 18933 :

2 alleles

99.2% missing data

1	(0.482) 0.475 0.483

3	(0.518) 0.525 0.517

Locus 18934 :

2 alleles

98.4% missing data

1	(0.660) 0.662 0.666

3	(0.340) 0.338 0.334

Locus 18935 :

2 alleles

99.2% missing data

3	(0.831) 0.825 0.837

1	(0.169) 0.175 0.163

Locus 18936 :

2 alleles

99.2% missing data

2	(0.481) 0.472 0.486

3	(0.519) 0.528 0.514

Locus 18937 :

2 alleles

99.2% missing data

2	(0.565) 0.562 0.574

1	(0.435) 0.438 0.426

Locus 18938 :

2 alleles

99.2% missing data

1	(0.572) 0.571 0.577

3	(0.428) 0.429 0.423

Locus 18939 :

2 alleles

99.2% missing data

3	(0.556) 0.558 0.562

1	(0.444) 0.442 0.438

Locus 18940 :

2 alleles

99.2% missing data

3	(0.433) 0.429 0.433

1	(0.567) 0.571 0.567

Locus 18941 :

2 alleles

99.2% missing data

4	(0.451) 0.452 0.451

3	(0.549) 0.548 0.549

Locus 18942 :

2 alleles

98.8% missing data

3	(0.637) 0.633 0.642

2	(0.363) 0.367 0.358

Locus 18943 :

2 alleles

1.2% missing data

4	(0.987) 0.990 0.993

1	(0.013) 0.010 0.007

Locus 18944 :

2 alleles

99.2% missing data

4	(0.489) 0.485 0.494

1	(0.511) 0.515 0.506

Locus 18945 :

2 alleles

99.2% missing data

3	(0.560) 0.554 0.560

2	(0.440) 0.446 0.440

Locus 18946 :

2 alleles

95.3% missing data

3	(0.134) 0.140 0.125

1	(0.866) 0.860 0.875

Locus 18947 :

2 alleles

99.2% missing data

1	(0.299) 0.302 0.301

2	(0.701) 0.698 0.699

Locus 18948 :

2 alleles

98.4% missing data

1	(0.394) 0.403 0.391

3	(0.606) 0.597 0.609

Locus 18949 :

2 alleles

99.2% missing data

4	(0.446) 0.447 0.450

1	(0.554) 0.553 0.550

Locus 18950 :

2 alleles

96.1% missing data

2	(0.801) 0.805 0.806

3	(0.199) 0.195 0.194

Locus 18951 :

3 alleles

90.2% missing data

3	(0.797) 0.834 0.783

1	(0.101) 0.083 0.108

2	(0.102) 0.084 0.109

Locus 18952 :

2 alleles

99.2% missing data

3	(0.343) 0.351 0.342

1	(0.657) 0.649 0.658

Locus 18953 :

2 alleles

99.2% missing data

2	(0.687) 0.682 0.690

1	(0.313) 0.318 0.310

Locus 18954 :

2 alleles

99.2% missing data

3	(0.647) 0.643 0.652

4	(0.353) 0.357 0.348

Locus 18955 :

2 alleles

99.2% missing data

3	(0.534) 0.528 0.538

1	(0.466) 0.472 0.462

Locus 18956 :

2 alleles

99.2% missing data

2	(0.528) 0.530 0.526

1	(0.472) 0.470 0.474

Locus 18957 :

2 alleles

99.2% missing data

3	(0.580) 0.583 0.579

4	(0.420) 0.417 0.421

Locus 18958 :

2 alleles

99.2% missing data

1	(0.416) 0.414 0.420

3	(0.584) 0.586 0.580

Locus 18959 :

2 alleles

7.0% missing data

3	(0.975) 0.951 0.992

4	(0.025) 0.049 0.008

Locus 18960 :

2 alleles

98.0% missing data

1	(0.660) 0.645 0.670

3	(0.340) 0.355 0.330

Locus 18961 :

2 alleles

99.2% missing data

3	(0.521) 0.518 0.525

2	(0.479) 0.482 0.475

Locus 18962 :

2 alleles

99.2% missing data

3	(0.843) 0.844 0.837

1	(0.157) 0.156 0.163

Locus 18963 :

2 alleles

98.8% missing data

3	(0.310) 0.312 0.309

1	(0.690) 0.688 0.691

Locus 18964 :

2 alleles

97.3% missing data

2	(0.855) 0.848 0.865

1	(0.145) 0.152 0.135

Locus 18965 :

2 alleles

98.8% missing data

2	(0.514) 0.502 0.521

1	(0.486) 0.498 0.479

Locus 18966 :

2 alleles

99.2% missing data

3	(0.639) 0.641 0.636

1	(0.361) 0.359 0.364

Locus 18967 :

2 alleles

98.8% missing data

2	(0.623) 0.616 0.625

4	(0.377) 0.384 0.375

Locus 18968 :

2 alleles

98.8% missing data

2	(0.816) 0.821 0.809

3	(0.184) 0.179 0.191

Locus 18969 :

2 alleles

64.8% missing data

3	(0.968) 0.959 0.983

1	(0.032) 0.041 0.017

Locus 18970 :

2 alleles

98.8% missing data

4	(0.337) 0.335 0.334

3	(0.663) 0.665 0.666

Locus 18971 :

2 alleles

98.8% missing data

4	(0.641) 0.636 0.648

3	(0.359) 0.364 0.352

Locus 18972 :

3 alleles

98.8% missing data

1	(0.372) 0.375 0.361

2	(0.219) 0.218 0.227

3	(0.409) 0.406 0.413

Locus 18973 :

2 alleles

99.2% missing data

1	(0.451) 0.447 0.462

2	(0.549) 0.553 0.538

Locus 18974 :

2 alleles

96.9% missing data

1	(0.585) 0.576 0.600

2	(0.415) 0.424 0.400

Locus 18975 :

3 alleles

98.8% missing data

1	(0.466) 0.458 0.464

2	(0.363) 0.357 0.369

3	(0.171) 0.185 0.167

Locus 18976 :

2 alleles

97.3% missing data

1	(0.164) 0.161 0.165

4	(0.836) 0.839 0.835

Locus 18977 :

3 alleles

97.7% missing data

4	(0.571) 0.582 0.572

2	(0.125) 0.119 0.132

3	(0.304) 0.299 0.296

Locus 18978 :

2 alleles

95.7% missing data

4	(0.835) 0.847 0.823

2	(0.165) 0.153 0.177

Locus 18979 :

2 alleles

99.2% missing data

3	(0.606) 0.610 0.605

2	(0.394) 0.390 0.395

Locus 18980 :

2 alleles

98.4% missing data

2	(0.683) 0.690 0.672

4	(0.317) 0.310 0.328

Locus 18981 :

2 alleles

99.2% missing data

1	(0.811) 0.801 0.811

3	(0.189) 0.199 0.189

Locus 18982 :

2 alleles

98.4% missing data

4	(0.672) 0.675 0.669

2	(0.328) 0.325 0.331

Locus 18983 :

2 alleles

99.2% missing data

2	(0.505) 0.501 0.509

4	(0.495) 0.499 0.491

Locus 18984 :

2 alleles

99.2% missing data

2	(0.367) 0.367 0.369

1	(0.633) 0.633 0.631

Locus 18985 :

2 alleles

99.2% missing data

2	(0.246) 0.253 0.245

1	(0.754) 0.747 0.755

Locus 18986 :

2 alleles

96.5% missing data

4	(0.742) 0.753 0.742

1	(0.258) 0.247 0.258

Locus 18987 :

2 alleles

96.5% missing data

4	(0.310) 0.321 0.303

2	(0.690) 0.679 0.697

Locus 18988 :

2 alleles

97.7% missing data

2	(0.787) 0.778 0.796

1	(0.213) 0.222 0.204

Locus 18989 :

2 alleles

99.2% missing data

2	(0.421) 0.423 0.419

3	(0.579) 0.577 0.581

Locus 18990 :

2 alleles

99.2% missing data

2	(0.774) 0.772 0.773

3	(0.226) 0.228 0.227

Locus 18991 :

2 alleles

97.7% missing data

3	(0.200) 0.192 0.210

2	(0.800) 0.808 0.790

Locus 18992 :

2 alleles

96.5% missing data

1	(0.872) 0.873 0.873

2	(0.128) 0.127 0.127

Locus 18993 :

2 alleles

92.6% missing data

2	(0.845) 0.847 0.857

3	(0.155) 0.153 0.143

Locus 18994 :

2 alleles

98.8% missing data

2	(0.685) 0.683 0.683

1	(0.315) 0.317 0.317

Locus 18995 :

2 alleles

98.4% missing data

3	(0.601) 0.606 0.593

4	(0.399) 0.394 0.407

Locus 18996 :

2 alleles

98.8% missing data

4	(0.597) 0.604 0.596

3	(0.403) 0.396 0.404

Locus 18997 :

2 alleles

99.2% missing data

3	(0.396) 0.406 0.393

2	(0.604) 0.594 0.607

Locus 18998 :

2 alleles

99.2% missing data

4	(0.506) 0.505 0.499

3	(0.494) 0.495 0.501

Locus 18999 :

2 alleles

99.2% missing data

2	(0.395) 0.392 0.395

1	(0.605) 0.608 0.605

Locus 19000 :

2 alleles

99.2% missing data

2	(0.246) 0.242 0.249

3	(0.754) 0.758 0.751

Locus 19001 :

2 alleles

99.2% missing data

1	(0.481) 0.487 0.469

2	(0.519) 0.513 0.531

Locus 19002 :

2 alleles

98.8% missing data

2	(0.752) 0.747 0.757

1	(0.248) 0.253 0.243

Locus 19003 :

3 alleles

94.5% missing data

1	(0.705) 0.693 0.730

2	(0.153) 0.149 0.143

4	(0.143) 0.158 0.127

Locus 19004 :

2 alleles

97.3% missing data

2	(0.178) 0.175 0.179

1	(0.822) 0.825 0.821

Locus 19005 :

2 alleles

97.7% missing data

2	(0.826) 0.817 0.830

1	(0.174) 0.183 0.170

Locus 19006 :

2 alleles

99.2% missing data

4	(0.750) 0.744 0.755

2	(0.250) 0.256 0.245

Locus 19007 :

2 alleles

98.8% missing data

4	(0.390) 0.395 0.386

2	(0.610) 0.605 0.614

Locus 19008 :

2 alleles

96.9% missing data

2	(0.272) 0.260 0.288

3	(0.728) 0.740 0.712

Locus 19009 :

2 alleles

99.2% missing data

4	(0.586) 0.584 0.586

3	(0.414) 0.416 0.414

Locus 19010 :

2 alleles

99.2% missing data

1	(0.556) 0.559 0.555

3	(0.444) 0.441 0.445

Locus 19011 :

2 alleles

98.8% missing data

4	(0.301) 0.296 0.306

2	(0.699) 0.704 0.694

Locus 19012 :

2 alleles

97.7% missing data

2	(0.384) 0.370 0.387

1	(0.616) 0.630 0.613

Locus 19013 :

2 alleles

98.8% missing data

4	(0.121) 0.119 0.129

2	(0.879) 0.881 0.871

Locus 19014 :

2 alleles

99.2% missing data

1	(0.385) 0.387 0.389

2	(0.615) 0.613 0.611

Locus 19015 :

2 alleles

99.2% missing data

3	(0.744) 0.743 0.742

4	(0.256) 0.257 0.258

Locus 19016 :

2 alleles

99.2% missing data

3	(0.627) 0.617 0.629

2	(0.373) 0.383 0.371

Locus 19017 :

2 alleles

96.9% missing data

2	(0.346) 0.351 0.349

3	(0.654) 0.649 0.651

Locus 19018 :

2 alleles

99.2% missing data

1	(0.531) 0.531 0.528

4	(0.469) 0.469 0.472

Locus 19019 :

2 alleles

99.2% missing data

4	(0.555) 0.558 0.552

1	(0.445) 0.442 0.448

Locus 19020 :

2 alleles

0.0% missing data

1	(0.985) 0.982 0.996

4	(0.015) 0.018 0.004

Locus 19021 :

2 alleles

97.7% missing data

1	(0.837) 0.838 0.835

3	(0.163) 0.162 0.165

Locus 19022 :

2 alleles

8.2% missing data

4	(0.985) 0.980 0.995

3	(0.015) 0.020 0.005

Locus 19023 :

2 alleles

96.5% missing data

4	(0.817) 0.824 0.819

1	(0.183) 0.176 0.181

Locus 19024 :

3 alleles

98.8% missing data

2	(0.334) 0.335 0.330

4	(0.283) 0.282 0.279

3	(0.382) 0.383 0.391

Locus 19025 :

2 alleles

99.2% missing data

4	(0.574) 0.576 0.577

1	(0.426) 0.424 0.423

Locus 19026 :

2 alleles

97.7% missing data

4	(0.160) 0.154 0.163

3	(0.840) 0.846 0.837

Locus 19027 :

2 alleles

98.4% missing data

1	(0.655) 0.657 0.658

4	(0.345) 0.343 0.342

Locus 19028 :

2 alleles

97.7% missing data

2	(0.628) 0.633 0.634

3	(0.372) 0.367 0.366

Locus 19029 :

2 alleles

99.2% missing data

3	(0.346) 0.346 0.346

2	(0.654) 0.654 0.654

Locus 19030 :

2 alleles

99.2% missing data

3	(0.458) 0.449 0.459

1	(0.542) 0.551 0.541

Locus 19031 :

2 alleles

98.8% missing data

2	(0.336) 0.327 0.349

1	(0.664) 0.673 0.651

Locus 19032 :

2 alleles

98.8% missing data

2	(0.811) 0.817 0.807

3	(0.189) 0.183 0.193

Locus 19033 :

2 alleles

99.2% missing data

1	(0.470) 0.470 0.466

3	(0.530) 0.530 0.534

Locus 19034 :

2 alleles

96.1% missing data

3	(0.855) 0.859 0.857

1	(0.145) 0.141 0.143

Locus 19035 :

2 alleles

98.8% missing data

2	(0.464) 0.459 0.466

3	(0.536) 0.541 0.534

Locus 19036 :

3 alleles

98.8% missing data

3	(0.165) 0.159 0.169

4	(0.259) 0.259 0.262

2	(0.576) 0.582 0.569

Locus 19037 :

2 alleles

98.8% missing data

2	(0.496) 0.492 0.501

4	(0.504) 0.508 0.499

Locus 19038 :

3 alleles

96.9% missing data

3	(0.226) 0.230 0.221

2	(0.107) 0.108 0.106

4	(0.666) 0.662 0.672

Locus 19039 :

2 alleles

98.0% missing data

1	(0.652) 0.658 0.653

3	(0.348) 0.342 0.347

Locus 19040 :

2 alleles

99.2% missing data

2	(0.471) 0.468 0.472

3	(0.529) 0.532 0.528

Locus 19041 :

2 alleles

99.2% missing data

2	(0.674) 0.668 0.676

4	(0.326) 0.332 0.324

Locus 19042 :

2 alleles

99.2% missing data

1	(0.692) 0.693 0.696

3	(0.308) 0.307 0.304

Locus 19043 :

2 alleles

99.2% missing data

3	(0.387) 0.388 0.394

2	(0.613) 0.612 0.606

Locus 19044 :

2 alleles

97.3% missing data

4	(0.212) 0.201 0.213

2	(0.788) 0.799 0.787

Locus 19045 :

2 alleles

99.2% missing data

2	(0.729) 0.736 0.724

4	(0.271) 0.264 0.276

Locus 19046 :

2 alleles

98.0% missing data

2	(0.770) 0.773 0.773

1	(0.230) 0.227 0.227

Locus 19047 :

2 alleles

98.8% missing data

3	(0.621) 0.615 0.626

2	(0.379) 0.385 0.374

Locus 19048 :

2 alleles

93.0% missing data

3	(0.874) 0.871 0.886

4	(0.126) 0.129 0.114

Locus 19049 :

2 alleles

99.2% missing data

1	(0.490) 0.488 0.495

3	(0.510) 0.512 0.505

Locus 19050 :

2 alleles

98.8% missing data

4	(0.527) 0.531 0.533

2	(0.473) 0.469 0.467

Locus 19051 :

2 alleles

98.8% missing data

1	(0.607) 0.602 0.612

2	(0.393) 0.398 0.388

Locus 19052 :

2 alleles

99.2% missing data

4	(0.523) 0.519 0.526

2	(0.477) 0.481 0.474

Locus 19053 :

2 alleles

94.1% missing data

3	(0.258) 0.258 0.255

2	(0.742) 0.742 0.745

Locus 19054 :

2 alleles

98.8% missing data

3	(0.665) 0.667 0.672

1	(0.335) 0.333 0.328

Locus 19055 :

2 alleles

92.6% missing data

4	(0.898) 0.901 0.906

1	(0.102) 0.099 0.094

Locus 19056 :

2 alleles

97.3% missing data

4	(0.876) 0.871 0.881

3	(0.124) 0.129 0.119

Locus 19057 :

3 alleles

98.8% missing data

3	(0.528) 0.524 0.528

1	(0.202) 0.212 0.192

4	(0.270) 0.264 0.280

Locus 19058 :

2 alleles

99.2% missing data

2	(0.481) 0.487 0.477

4	(0.519) 0.513 0.523

Locus 19059 :

2 alleles

98.4% missing data

4	(0.597) 0.594 0.604

1	(0.403) 0.406 0.396

Locus 19060 :

2 alleles

99.2% missing data

2	(0.617) 0.615 0.622

1	(0.383) 0.385 0.378

Locus 19061 :

2 alleles

99.2% missing data

1	(0.443) 0.444 0.443

3	(0.557) 0.556 0.557

Locus 19062 :

2 alleles

99.2% missing data

4	(0.358) 0.364 0.360

3	(0.642) 0.636 0.640

Locus 19063 :

2 alleles

99.2% missing data

1	(0.623) 0.615 0.626

2	(0.377) 0.385 0.374

Locus 19064 :

2 alleles

99.2% missing data

2	(0.395) 0.390 0.401

3	(0.605) 0.610 0.599

Locus 19065 :

2 alleles

99.2% missing data

3	(0.537) 0.538 0.536

1	(0.463) 0.462 0.464

Locus 19066 :

2 alleles

99.2% missing data

3	(0.568) 0.569 0.572

2	(0.432) 0.431 0.428

Locus 19067 :

2 alleles

0.4% missing data

4	(0.986) 0.990 0.993

1	(0.014) 0.010 0.007

Locus 19068 :

2 alleles

99.2% missing data

3	(0.678) 0.678 0.674

4	(0.322) 0.322 0.326

Locus 19069 :

2 alleles

99.2% missing data

2	(0.395) 0.392 0.400

4	(0.605) 0.608 0.600

Locus 19070 :

2 alleles

98.0% missing data

2	(0.722) 0.728 0.724

4	(0.278) 0.272 0.276

Locus 19071 :

2 alleles

98.8% missing data

4	(0.303) 0.303 0.301

2	(0.697) 0.697 0.699

Locus 19072 :

3 alleles

90.2% missing data

2	(0.854) 0.880 0.830

1	(0.065) 0.052 0.075

3	(0.081) 0.068 0.094

Locus 19073 :

2 alleles

96.1% missing data

4	(0.180) 0.169 0.182

3	(0.820) 0.831 0.818

Locus 19074 :

2 alleles

99.2% missing data

2	(0.189) 0.188 0.188

4	(0.811) 0.812 0.812

Locus 19075 :

2 alleles

99.2% missing data

4	(0.458) 0.454 0.461

2	(0.542) 0.546 0.539

Locus 19076 :

2 alleles

98.4% missing data

3	(0.584) 0.586 0.581

1	(0.416) 0.414 0.419

Locus 19077 :

2 alleles

99.2% missing data

1	(0.727) 0.724 0.734

3	(0.273) 0.276 0.266

Locus 19078 :

2 alleles

98.8% missing data

3	(0.772) 0.773 0.767

4	(0.228) 0.227 0.233

Locus 19079 :

2 alleles

92.6% missing data

4	(0.830) 0.836 0.842

3	(0.170) 0.164 0.158

Locus 19080 :

2 alleles

99.2% missing data

3	(0.213) 0.223 0.212

1	(0.787) 0.777 0.788

Locus 19081 :

2 alleles

96.1% missing data

3	(0.325) 0.331 0.319

2	(0.675) 0.669 0.681

Locus 19082 :

2 alleles

96.1% missing data

2	(0.182) 0.171 0.185

4	(0.818) 0.829 0.815

Locus 19083 :

3 alleles

98.4% missing data

3	(0.585) 0.584 0.583

2	(0.195) 0.194 0.200

4	(0.220) 0.222 0.217

Locus 19084 :

2 alleles

98.4% missing data

4	(0.745) 0.752 0.739

1	(0.255) 0.248 0.261

Locus 19085 :

2 alleles

6.6% missing data

2	(0.968) 0.934 0.990

4	(0.032) 0.066 0.010

Locus 19086 :

2 alleles

99.2% missing data

3	(0.439) 0.439 0.438

1	(0.561) 0.561 0.562

Locus 19087 :

2 alleles

99.2% missing data

1	(0.687) 0.684 0.684

4	(0.313) 0.316 0.316

Locus 19088 :

2 alleles

99.2% missing data

2	(0.531) 0.539 0.529

3	(0.469) 0.461 0.471

Locus 19089 :

2 alleles

99.2% missing data

3	(0.742) 0.738 0.747

4	(0.258) 0.262 0.253

Locus 19090 :

2 alleles

99.2% missing data

3	(0.448) 0.445 0.449

1	(0.552) 0.555 0.551

Locus 19091 :

2 alleles

99.2% missing data

4	(0.385) 0.382 0.380

1	(0.615) 0.618 0.620

Locus 19092 :

2 alleles

98.8% missing data

2	(0.712) 0.711 0.711

1	(0.288) 0.289 0.289

Locus 19093 :

2 alleles

99.2% missing data

1	(0.442) 0.443 0.443

3	(0.558) 0.557 0.557

Locus 19094 :

2 alleles

98.4% missing data

4	(0.404) 0.412 0.397

1	(0.596) 0.588 0.603

Locus 19095 :

2 alleles

95.7% missing data

4	(0.671) 0.662 0.682

1	(0.329) 0.338 0.318

Locus 19096 :

2 alleles

91.0% missing data

1	(0.738) 0.740 0.747

2	(0.262) 0.260 0.253

Locus 19097 :

2 alleles

99.2% missing data

1	(0.657) 0.655 0.659

2	(0.343) 0.345 0.341

Locus 19098 :

2 alleles

99.2% missing data

4	(0.766) 0.758 0.772

2	(0.234) 0.242 0.228

Locus 19099 :

3 alleles

95.7% missing data

4	(0.201) 0.194 0.202

1	(0.661) 0.678 0.655

3	(0.138) 0.128 0.143

Locus 19100 :

3 alleles

97.3% missing data

3	(0.319) 0.323 0.308

4	(0.560) 0.558 0.571

2	(0.121) 0.120 0.121

Locus 19101 :

2 alleles

98.4% missing data

1	(0.555) 0.551 0.563

2	(0.445) 0.449 0.437

Locus 19102 :

2 alleles

96.5% missing data

1	(0.846) 0.851 0.851

2	(0.154) 0.149 0.149

Locus 19103 :

2 alleles

97.7% missing data

2	(0.458) 0.466 0.440

4	(0.542) 0.534 0.560

Locus 19104 :

2 alleles

99.2% missing data

2	(0.573) 0.565 0.580

3	(0.427) 0.435 0.420

Locus 19105 :

2 alleles

98.0% missing data

2	(0.827) 0.823 0.824

1	(0.173) 0.177 0.176

Locus 19106 :

2 alleles

99.2% missing data

3	(0.491) 0.490 0.494

2	(0.509) 0.510 0.506

Locus 19107 :

2 alleles

91.0% missing data

3	(0.862) 0.890 0.849

1	(0.138) 0.110 0.151

Locus 19108 :

2 alleles

99.2% missing data

1	(0.341) 0.334 0.346

2	(0.659) 0.666 0.654

Locus 19109 :

2 alleles

99.2% missing data

4	(0.591) 0.587 0.598

2	(0.409) 0.413 0.402

Locus 19110 :

2 alleles

99.2% missing data

2	(0.709) 0.704 0.713

1	(0.291) 0.296 0.287

Locus 19111 :

2 alleles

99.2% missing data

3	(0.420) 0.423 0.420

4	(0.580) 0.577 0.580

Locus 19112 :

2 alleles

99.2% missing data

3	(0.451) 0.452 0.447

4	(0.549) 0.548 0.553

Locus 19113 :

2 alleles

96.5% missing data

2	(0.154) 0.141 0.160

4	(0.846) 0.859 0.840

Locus 19114 :

3 alleles

98.8% missing data

4	(0.241) 0.238 0.244

2	(0.486) 0.483 0.488

1	(0.273) 0.279 0.267

Locus 19115 :

2 alleles

99.2% missing data

3	(0.753) 0.754 0.741

4	(0.247) 0.246 0.259

Locus 19116 :

2 alleles

97.3% missing data

3	(0.775) 0.774 0.783

4	(0.225) 0.226 0.217

Locus 19117 :

2 alleles

99.2% missing data

1	(0.738) 0.737 0.742

2	(0.262) 0.263 0.258

Locus 19118 :

2 alleles

96.1% missing data

1	(0.849) 0.861 0.844

4	(0.151) 0.139 0.156

Locus 19119 :

2 alleles

99.2% missing data

4	(0.353) 0.348 0.353

1	(0.647) 0.652 0.647

Locus 19120 :

2 alleles

99.2% missing data

4	(0.421) 0.413 0.425

1	(0.579) 0.587 0.575

Locus 19121 :

2 alleles

97.7% missing data

1	(0.802) 0.798 0.811

4	(0.198) 0.202 0.189

Locus 19122 :

2 alleles

99.2% missing data

1	(0.654) 0.661 0.646

4	(0.346) 0.339 0.354

Locus 19123 :

2 alleles

99.2% missing data

4	(0.766) 0.769 0.764

2	(0.234) 0.231 0.236

Locus 19124 :

2 alleles

98.4% missing data

4	(0.482) 0.476 0.494

2	(0.518) 0.524 0.506

Locus 19125 :

2 alleles

99.2% missing data

3	(0.558) 0.558 0.561

1	(0.442) 0.442 0.439

Locus 19126 :

2 alleles

99.2% missing data

4	(0.751) 0.747 0.758

2	(0.249) 0.253 0.242

Locus 19127 :

2 alleles

96.1% missing data

2	(0.102) 0.104 0.103

3	(0.898) 0.896 0.897

Locus 19128 :

2 alleles

99.2% missing data

4	(0.637) 0.634 0.642

2	(0.363) 0.366 0.358

Locus 19129 :

2 alleles

97.7% missing data

4	(0.302) 0.309 0.293

2	(0.698) 0.691 0.707

Locus 19130 :

2 alleles

96.9% missing data

4	(0.573) 0.553 0.584

2	(0.427) 0.447 0.416

Locus 19131 :

2 alleles

91.4% missing data

2	(0.911) 0.909 0.920

1	(0.089) 0.091 0.080

Locus 19132 :

2 alleles

97.3% missing data

1	(0.832) 0.837 0.831

4	(0.168) 0.163 0.169

Locus 19133 :

2 alleles

98.4% missing data

2	(0.759) 0.758 0.762

3	(0.241) 0.242 0.238

Locus 19134 :

2 alleles

96.1% missing data

2	(0.306) 0.295 0.311

3	(0.694) 0.705 0.689

Locus 19135 :

2 alleles

98.8% missing data

1	(0.638) 0.643 0.635

2	(0.362) 0.357 0.365

Locus 19136 :

2 alleles

99.2% missing data

4	(0.305) 0.297 0.312

3	(0.695) 0.703 0.688

Locus 19137 :

2 alleles

98.8% missing data

1	(0.341) 0.348 0.342

3	(0.659) 0.652 0.658

Locus 19138 :

2 alleles

99.2% missing data

2	(0.304) 0.307 0.311

3	(0.696) 0.693 0.689

Locus 19139 :

2 alleles

96.9% missing data

4	(0.775) 0.783 0.768

1	(0.225) 0.217 0.232

Locus 19140 :

2 alleles

98.8% missing data

1	(0.422) 0.425 0.420

3	(0.578) 0.575 0.580

Locus 19141 :

2 alleles

99.2% missing data

2	(0.551) 0.554 0.543

1	(0.449) 0.446 0.457

Locus 19142 :

2 alleles

8.2% missing data

1	(0.981) 0.973 0.994

3	(0.019) 0.027 0.006

Locus 19143 :

2 alleles

98.8% missing data

1	(0.416) 0.416 0.409

2	(0.584) 0.584 0.591

Locus 19144 :

2 alleles

99.2% missing data

1	(0.356) 0.355 0.349

4	(0.644) 0.645 0.651

Locus 19145 :

2 alleles

99.2% missing data

2	(0.699) 0.689 0.702

4	(0.301) 0.311 0.298

Locus 19146 :

2 alleles

99.2% missing data

4	(0.399) 0.399 0.400

2	(0.601) 0.601 0.600

Locus 19147 :

2 alleles

97.3% missing data

2	(0.342) 0.342 0.328

4	(0.658) 0.658 0.672

Locus 19148 :

2 alleles

99.2% missing data

2	(0.728) 0.725 0.731

3	(0.272) 0.275 0.269

Locus 19149 :

3 alleles

97.7% missing data

2	(0.228) 0.220 0.238

3	(0.310) 0.315 0.313

4	(0.462) 0.465 0.449

Locus 19150 :

2 alleles

98.8% missing data

1	(0.405) 0.401 0.409

2	(0.595) 0.599 0.591

Locus 19151 :

3 alleles

94.5% missing data

2	(0.657) 0.673 0.655

3	(0.140) 0.131 0.147

4	(0.203) 0.196 0.197

Locus 19152 :

2 alleles

99.2% missing data

4	(0.683) 0.684 0.687

2	(0.317) 0.316 0.313

Locus 19153 :

2 alleles

99.2% missing data

4	(0.857) 0.860 0.851

2	(0.143) 0.140 0.149

Locus 19154 :

2 alleles

98.8% missing data

3	(0.585) 0.576 0.597

2	(0.415) 0.424 0.403

Locus 19155 :

2 alleles

99.2% missing data

3	(0.763) 0.766 0.761

2	(0.237) 0.234 0.239

Locus 19156 :

2 alleles

99.2% missing data

4	(0.571) 0.575 0.569

2	(0.429) 0.425 0.431

Locus 19157 :

2 alleles

96.1% missing data

3	(0.909) 0.920 0.901

2	(0.091) 0.080 0.099

Locus 19158 :

2 alleles

95.7% missing data

4	(0.784) 0.786 0.779

1	(0.216) 0.214 0.221

Locus 19159 :

2 alleles

95.7% missing data

2	(0.751) 0.760 0.741

3	(0.249) 0.240 0.259

Locus 19160 :

2 alleles

90.2% missing data

3	(0.839) 0.869 0.815

2	(0.161) 0.131 0.185

Locus 19161 :

2 alleles

98.8% missing data

3	(0.836) 0.837 0.832

4	(0.164) 0.163 0.168

Locus 19162 :

2 alleles

99.2% missing data

4	(0.477) 0.477 0.473

2	(0.523) 0.523 0.527

Locus 19163 :

2 alleles

99.2% missing data

1	(0.749) 0.742 0.755

3	(0.251) 0.258 0.245

Locus 19164 :

2 alleles

98.8% missing data

3	(0.517) 0.509 0.520

2	(0.483) 0.491 0.480

Locus 19165 :

2 alleles

99.2% missing data

4	(0.322) 0.332 0.319

2	(0.678) 0.668 0.681

Locus 19166 :

2 alleles

99.2% missing data

2	(0.592) 0.591 0.590

4	(0.408) 0.409 0.410

Locus 19167 :

2 alleles

99.2% missing data

1	(0.437) 0.441 0.435

4	(0.563) 0.559 0.565

Locus 19168 :

2 alleles

99.2% missing data

3	(0.424) 0.432 0.421

1	(0.576) 0.568 0.579

Locus 19169 :

2 alleles

98.4% missing data

4	(0.821) 0.821 0.821

2	(0.179) 0.179 0.179

Locus 19170 :

2 alleles

98.8% missing data

4	(0.558) 0.562 0.558

1	(0.442) 0.438 0.442

Locus 19171 :

2 alleles

99.2% missing data

3	(0.515) 0.517 0.514

4	(0.485) 0.483 0.486

Locus 19172 :

2 alleles

99.2% missing data

2	(0.170) 0.165 0.174

1	(0.830) 0.835 0.826

Locus 19173 :

2 alleles

99.2% missing data

4	(0.434) 0.433 0.445

1	(0.566) 0.567 0.555

Locus 19174 :

2 alleles

1.2% missing data

1	(0.912) 0.749 0.973

2	(0.088) 0.251 0.027

Locus 19175 :

2 alleles

9.8% missing data

4	(0.964) 0.934 0.987

2	(0.036) 0.066 0.013

Locus 19176 :

2 alleles

98.4% missing data

1	(0.573) 0.588 0.563

3	(0.427) 0.412 0.437

Locus 19177 :

2 alleles

99.2% missing data

2	(0.372) 0.372 0.374

3	(0.628) 0.628 0.626

Locus 19178 :

2 alleles

99.2% missing data

2	(0.511) 0.514 0.513

1	(0.489) 0.486 0.487

Locus 19179 :

2 alleles

99.2% missing data

1	(0.729) 0.730 0.726

2	(0.271) 0.270 0.274

Locus 19180 :

2 alleles

98.0% missing data

2	(0.575) 0.577 0.585

4	(0.425) 0.423 0.415

Locus 19181 :

2 alleles

99.2% missing data

2	(0.696) 0.702 0.695

4	(0.304) 0.298 0.305

Locus 19182 :

2 alleles

98.8% missing data

2	(0.714) 0.710 0.713

4	(0.286) 0.290 0.287

Locus 19183 :

2 alleles

98.8% missing data

3	(0.231) 0.223 0.240

4	(0.769) 0.777 0.760

Locus 19184 :

2 alleles

98.8% missing data

2	(0.706) 0.710 0.708

1	(0.294) 0.290 0.292

Locus 19185 :

2 alleles

99.2% missing data

1	(0.570) 0.571 0.570

4	(0.430) 0.429 0.430

Locus 19186 :

2 alleles

99.2% missing data

1	(0.374) 0.371 0.377

4	(0.626) 0.629 0.623

Locus 19187 :

2 alleles

98.8% missing data

2	(0.595) 0.594 0.600

1	(0.405) 0.406 0.400

Locus 19188 :

2 alleles

99.2% missing data

3	(0.589) 0.589 0.590

1	(0.411) 0.411 0.410

Locus 19189 :

3 alleles

98.0% missing data

4	(0.357) 0.343 0.375

1	(0.405) 0.417 0.395

2	(0.238) 0.240 0.230

Locus 19190 :

2 alleles

98.0% missing data

3	(0.859) 0.857 0.858

4	(0.141) 0.143 0.142

Locus 19191 :

2 alleles

99.2% missing data

2	(0.497) 0.494 0.507

1	(0.503) 0.506 0.493

Locus 19192 :

2 alleles

98.0% missing data

4	(0.760) 0.766 0.752

2	(0.240) 0.234 0.248

Locus 19193 :

2 alleles

99.2% missing data

4	(0.540) 0.533 0.535

2	(0.460) 0.467 0.465

Locus 19194 :

2 alleles

97.7% missing data

1	(0.682) 0.682 0.688

2	(0.318) 0.318 0.312

Locus 19195 :

2 alleles

99.2% missing data

1	(0.670) 0.668 0.678

2	(0.330) 0.332 0.322

Locus 19196 :

2 alleles

0.0% missing data

4	(0.924) 0.800 0.977

2	(0.076) 0.200 0.023

Locus 19197 :

2 alleles

97.3% missing data

2	(0.744) 0.747 0.746

1	(0.256) 0.253 0.254

Locus 19198 :

2 alleles

98.4% missing data

1	(0.224) 0.222 0.227

2	(0.776) 0.778 0.773

Locus 19199 :

2 alleles

99.2% missing data

2	(0.384) 0.379 0.385

4	(0.616) 0.621 0.615

Locus 19200 :

2 alleles

99.2% missing data

3	(0.383) 0.379 0.380

1	(0.617) 0.621 0.620

Locus 19201 :

3 alleles

98.8% missing data

1	(0.208) 0.205 0.209

2	(0.606) 0.614 0.599

4	(0.186) 0.181 0.193

Locus 19202 :

2 alleles

99.2% missing data

3	(0.502) 0.490 0.506

1	(0.498) 0.510 0.494

Locus 19203 :

2 alleles

99.2% missing data

1	(0.479) 0.478 0.482

3	(0.521) 0.522 0.518

Locus 19204 :

2 alleles

98.8% missing data

2	(0.555) 0.553 0.564

3	(0.445) 0.447 0.436

Locus 19205 :

2 alleles

99.2% missing data

3	(0.324) 0.325 0.323

2	(0.676) 0.675 0.677

Locus 19206 :

2 alleles

99.2% missing data

4	(0.428) 0.424 0.438

1	(0.572) 0.576 0.562

Locus 19207 :

2 alleles

93.0% missing data

3	(0.860) 0.859 0.872

4	(0.140) 0.141 0.128

Locus 19208 :

2 alleles

96.1% missing data

4	(0.263) 0.246 0.275

3	(0.737) 0.754 0.725

Locus 19209 :

2 alleles

98.0% missing data

3	(0.607) 0.605 0.608

2	(0.393) 0.395 0.392

Locus 19210 :

2 alleles

99.2% missing data

2	(0.475) 0.480 0.473

4	(0.525) 0.520 0.527

Locus 19211 :

2 alleles

98.8% missing data

1	(0.569) 0.576 0.569

3	(0.431) 0.424 0.431

Locus 19212 :

2 alleles

99.2% missing data

3	(0.245) 0.254 0.244

2	(0.755) 0.746 0.756

Locus 19213 :

2 alleles

99.2% missing data

2	(0.405) 0.399 0.408

1	(0.595) 0.601 0.592

Locus 19214 :

2 alleles

98.4% missing data

3	(0.607) 0.612 0.612

4	(0.393) 0.388 0.388

Locus 19215 :

2 alleles

99.2% missing data

3	(0.715) 0.720 0.714

2	(0.285) 0.280 0.286

Locus 19216 :

2 alleles

99.2% missing data

3	(0.441) 0.446 0.438

2	(0.559) 0.554 0.562

Locus 19217 :

2 alleles

96.1% missing data

3	(0.626) 0.621 0.653

2	(0.374) 0.379 0.347

Locus 19218 :

2 alleles

97.3% missing data

3	(0.277) 0.263 0.287

1	(0.723) 0.737 0.713

Locus 19219 :

2 alleles

98.8% missing data

1	(0.625) 0.624 0.629

4	(0.375) 0.376 0.371

Locus 19220 :

2 alleles

99.2% missing data

4	(0.159) 0.158 0.161

1	(0.841) 0.842 0.839

Locus 19221 :

3 alleles

98.8% missing data

2	(0.438) 0.436 0.441

1	(0.331) 0.326 0.334

3	(0.231) 0.238 0.226

Locus 19222 :

2 alleles

99.2% missing data

2	(0.771) 0.766 0.776

3	(0.229) 0.234 0.224

Locus 19223 :

2 alleles

98.8% missing data

1	(0.617) 0.618 0.620

2	(0.383) 0.382 0.380

Locus 19224 :

2 alleles

99.2% missing data

1	(0.325) 0.314 0.329

4	(0.675) 0.686 0.671

Locus 19225 :

2 alleles

99.2% missing data

1	(0.383) 0.376 0.387

4	(0.617) 0.624 0.613

Locus 19226 :

2 alleles

98.8% missing data

3	(0.758) 0.755 0.765

4	(0.242) 0.245 0.235

Locus 19227 :

2 alleles

99.2% missing data

3	(0.494) 0.491 0.501

2	(0.506) 0.509 0.499

Locus 19228 :

2 alleles

98.8% missing data

4	(0.553) 0.555 0.547

2	(0.447) 0.445 0.453

Locus 19229 :

2 alleles

99.2% missing data

2	(0.758) 0.752 0.761

4	(0.242) 0.248 0.239

Locus 19230 :

2 alleles

99.2% missing data

2	(0.355) 0.357 0.350

3	(0.645) 0.643 0.650

Locus 19231 :

3 alleles

98.4% missing data

4	(0.513) 0.504 0.523

3	(0.322) 0.325 0.318

1	(0.166) 0.171 0.159

Locus 19232 :

2 alleles

99.2% missing data

2	(0.291) 0.289 0.296

3	(0.709) 0.711 0.704

Locus 19233 :

3 alleles

98.8% missing data

3	(0.525) 0.527 0.523

4	(0.263) 0.264 0.263

1	(0.212) 0.209 0.214

Locus 19234 :

2 alleles

94.5% missing data

4	(0.858) 0.851 0.876

3	(0.142) 0.149 0.124

Locus 19235 :

2 alleles

99.2% missing data

3	(0.701) 0.703 0.696

4	(0.299) 0.297 0.304

Locus 19236 :

2 alleles

96.5% missing data

2	(0.800) 0.803 0.799

3	(0.200) 0.197 0.201

Locus 19237 :

2 alleles

98.4% missing data

2	(0.667) 0.661 0.679

4	(0.333) 0.339 0.321

Locus 19238 :

2 alleles

99.2% missing data

4	(0.505) 0.500 0.507

3	(0.495) 0.500 0.493

Locus 19239 :

2 alleles

99.2% missing data

1	(0.156) 0.159 0.157

4	(0.844) 0.841 0.843

Locus 19240 :

2 alleles

99.2% missing data

1	(0.464) 0.468 0.458

2	(0.536) 0.532 0.542

Locus 19241 :

2 alleles

98.4% missing data

2	(0.418) 0.418 0.431

3	(0.582) 0.582 0.569

Locus 19242 :

2 alleles

99.2% missing data

1	(0.608) 0.611 0.602

3	(0.392) 0.389 0.398

Locus 19243 :

3 alleles

98.8% missing data

4	(0.404) 0.402 0.408

3	(0.269) 0.267 0.271

2	(0.328) 0.331 0.321

Locus 19244 :

2 alleles

99.2% missing data

3	(0.254) 0.259 0.252

4	(0.746) 0.741 0.748

Locus 19245 :

3 alleles

98.0% missing data

3	(0.179) 0.181 0.174

4	(0.603) 0.612 0.602

1	(0.218) 0.207 0.224

Locus 19246 :

2 alleles

99.2% missing data

4	(0.642) 0.642 0.651

2	(0.358) 0.358 0.349

Locus 19247 :

2 alleles

99.2% missing data

3	(0.492) 0.492 0.490

2	(0.508) 0.508 0.510

Locus 19248 :

2 alleles

98.8% missing data

3	(0.513) 0.512 0.512

2	(0.487) 0.488 0.488

Locus 19249 :

2 alleles

99.2% missing data

2	(0.538) 0.530 0.540

1	(0.462) 0.470 0.460

Locus 19250 :

2 alleles

99.2% missing data

2	(0.748) 0.737 0.750

1	(0.252) 0.263 0.250

Locus 19251 :

2 alleles

90.6% missing data

2	(0.945) 0.949 0.946

4	(0.055) 0.051 0.054

Locus 19252 :

2 alleles

98.0% missing data

2	(0.714) 0.702 0.728

4	(0.286) 0.298 0.272

Locus 19253 :

2 alleles

90.6% missing data

3	(0.904) 0.921 0.886

1	(0.096) 0.079 0.114

Locus 19254 :

2 alleles

99.2% missing data

1	(0.312) 0.310 0.320

2	(0.688) 0.690 0.680

Locus 19255 :

2 alleles

96.1% missing data

2	(0.812) 0.816 0.816

3	(0.188) 0.184 0.184

Locus 19256 :

2 alleles

99.2% missing data

2	(0.442) 0.442 0.441

3	(0.558) 0.558 0.559

Locus 19257 :

2 alleles

99.2% missing data

2	(0.392) 0.396 0.390

3	(0.608) 0.604 0.610

Locus 19258 :

2 alleles

98.8% missing data

1	(0.590) 0.590 0.587

2	(0.410) 0.410 0.413

Locus 19259 :

4 alleles

94.5% missing data

4	(0.189) 0.193 0.193

2	(0.185) 0.178 0.185

3	(0.526) 0.540 0.516

1	(0.100) 0.089 0.105

Locus 19260 :

2 alleles

98.8% missing data

2	(0.618) 0.611 0.620

4	(0.382) 0.389 0.380

Locus 19261 :

2 alleles

99.2% missing data

4	(0.758) 0.754 0.757

3	(0.242) 0.246 0.243

Locus 19262 :

2 alleles

99.2% missing data

2	(0.698) 0.698 0.699

1	(0.302) 0.302 0.301

Locus 19263 :

2 alleles

99.2% missing data

2	(0.350) 0.347 0.352

4	(0.650) 0.653 0.648

Locus 19264 :

2 alleles

99.2% missing data

2	(0.510) 0.510 0.511

4	(0.490) 0.490 0.489

Locus 19265 :

2 alleles

99.2% missing data

2	(0.508) 0.515 0.498

3	(0.492) 0.485 0.502

Locus 19266 :

2 alleles

99.2% missing data

4	(0.545) 0.537 0.549

3	(0.455) 0.463 0.451

Locus 19267 :

2 alleles

99.2% missing data

4	(0.197) 0.201 0.201

3	(0.803) 0.799 0.799

Locus 19268 :

2 alleles

99.2% missing data

4	(0.383) 0.384 0.381

1	(0.617) 0.616 0.619

Locus 19269 :

2 alleles

98.4% missing data

2	(0.466) 0.463 0.462

3	(0.534) 0.537 0.538

Locus 19270 :

2 alleles

99.2% missing data

2	(0.694) 0.691 0.695

3	(0.306) 0.309 0.305

Locus 19271 :

2 alleles

99.2% missing data

4	(0.425) 0.424 0.425

2	(0.575) 0.576 0.575

Locus 19272 :

2 alleles

99.2% missing data

3	(0.484) 0.486 0.483

2	(0.516) 0.514 0.517

Locus 19273 :

2 alleles

96.1% missing data

1	(0.163) 0.157 0.160

3	(0.837) 0.843 0.840

Locus 19274 :

2 alleles

98.8% missing data

2	(0.481) 0.482 0.483

3	(0.519) 0.518 0.517

Locus 19275 :

2 alleles

99.2% missing data

2	(0.767) 0.758 0.773

4	(0.233) 0.242 0.227

Locus 19276 :

2 alleles

98.4% missing data

3	(0.432) 0.445 0.430

1	(0.568) 0.555 0.570

Locus 19277 :

2 alleles

99.2% missing data

2	(0.724) 0.723 0.723

3	(0.276) 0.277 0.277

Locus 19278 :

2 alleles

99.2% missing data

1	(0.460) 0.465 0.451

4	(0.540) 0.535 0.549

Locus 19279 :

2 alleles

98.4% missing data

2	(0.628) 0.633 0.629

3	(0.372) 0.367 0.371

Locus 19280 :

2 alleles

98.8% missing data

3	(0.674) 0.674 0.677

1	(0.326) 0.326 0.323

Locus 19281 :

2 alleles

98.8% missing data

3	(0.838) 0.842 0.837

4	(0.162) 0.158 0.163

Locus 19282 :

2 alleles

99.2% missing data

4	(0.418) 0.420 0.415

1	(0.582) 0.580 0.585

Locus 19283 :

2 alleles

96.1% missing data

3	(0.167) 0.158 0.173

4	(0.833) 0.842 0.827

Locus 19284 :

2 alleles

99.2% missing data

3	(0.337) 0.342 0.336

2	(0.663) 0.658 0.664

Locus 19285 :

2 alleles

99.2% missing data

4	(0.666) 0.666 0.666

1	(0.334) 0.334 0.334

Locus 19286 :

2 alleles

99.2% missing data

2	(0.643) 0.635 0.644

1	(0.357) 0.365 0.356

Locus 19287 :

3 alleles

98.8% missing data

2	(0.200) 0.196 0.202

3	(0.293) 0.294 0.291

4	(0.506) 0.509 0.506

Locus 19288 :

2 alleles

99.2% missing data

3	(0.571) 0.563 0.573

4	(0.429) 0.437 0.427

Locus 19289 :

2 alleles

99.2% missing data

2	(0.721) 0.708 0.725

4	(0.279) 0.292 0.275

Locus 19290 :

2 alleles

0.4% missing data

2	(0.987) 0.982 0.996

3	(0.013) 0.018 0.004

Locus 19291 :

2 alleles

90.2% missing data

4	(0.911) 0.929 0.901

1	(0.089) 0.071 0.099

Locus 19292 :

2 alleles

99.2% missing data

2	(0.724) 0.719 0.724

3	(0.276) 0.281 0.276

Locus 19293 :

2 alleles

96.5% missing data

2	(0.208) 0.206 0.200

3	(0.792) 0.794 0.800

Locus 19294 :

2 alleles

97.7% missing data

2	(0.525) 0.523 0.530

4	(0.475) 0.477 0.470

Locus 19295 :

2 alleles

0.0% missing data

4	(0.987) 0.982 0.996

3	(0.013) 0.018 0.004

Locus 19296 :

2 alleles

98.8% missing data

2	(0.647) 0.646 0.657

3	(0.353) 0.354 0.343

Locus 19297 :

2 alleles

99.2% missing data

1	(0.476) 0.474 0.476

4	(0.524) 0.526 0.524

Locus 19298 :

2 alleles

99.2% missing data

2	(0.344) 0.349 0.345

1	(0.656) 0.651 0.655

Locus 19299 :

2 alleles

99.2% missing data

3	(0.340) 0.340 0.343

2	(0.660) 0.660 0.657

Locus 19300 :

2 alleles

99.2% missing data

3	(0.648) 0.641 0.650

1	(0.352) 0.359 0.350

Locus 19301 :

2 alleles

99.2% missing data

1	(0.380) 0.380 0.376

3	(0.620) 0.620 0.624

Locus 19302 :

3 alleles

98.8% missing data

3	(0.342) 0.343 0.339

1	(0.276) 0.274 0.283

2	(0.382) 0.383 0.378

Locus 19303 :

2 alleles

99.2% missing data

1	(0.339) 0.337 0.340

2	(0.661) 0.663 0.660

Locus 19304 :

2 alleles

99.2% missing data

3	(0.523) 0.525 0.523

2	(0.477) 0.475 0.477

Locus 19305 :

2 alleles

98.0% missing data

4	(0.909) 0.905 0.913

3	(0.091) 0.095 0.087

Locus 19306 :

3 alleles

98.8% missing data

4	(0.641) 0.631 0.641

3	(0.182) 0.191 0.178

2	(0.177) 0.178 0.180

Locus 19307 :

2 alleles

99.2% missing data

2	(0.571) 0.569 0.572

1	(0.429) 0.431 0.428

Locus 19308 :

2 alleles

98.0% missing data

1	(0.570) 0.565 0.581

2	(0.430) 0.435 0.419

Locus 19309 :

2 alleles

99.2% missing data

1	(0.610) 0.614 0.616

3	(0.390) 0.386 0.384

Locus 19310 :

2 alleles

98.8% missing data

1	(0.681) 0.673 0.690

3	(0.319) 0.327 0.310

Locus 19311 :

2 alleles

98.4% missing data

3	(0.701) 0.703 0.700

2	(0.299) 0.297 0.300

Locus 19312 :

2 alleles

98.0% missing data

4	(0.675) 0.673 0.676

1	(0.325) 0.327 0.324

Locus 19313 :

2 alleles

99.2% missing data

4	(0.590) 0.590 0.596

2	(0.410) 0.410 0.404

Locus 19314 :

2 alleles

99.2% missing data

3	(0.407) 0.416 0.396

4	(0.593) 0.584 0.604

Locus 19315 :

2 alleles

99.2% missing data

1	(0.581) 0.583 0.580

2	(0.419) 0.417 0.420

Locus 19316 :

2 alleles

99.2% missing data

3	(0.398) 0.393 0.403

2	(0.602) 0.607 0.597

Locus 19317 :

2 alleles

99.2% missing data

1	(0.474) 0.478 0.469

4	(0.526) 0.522 0.531

Locus 19318 :

2 alleles

99.2% missing data

2	(0.638) 0.635 0.635

1	(0.362) 0.365 0.365

Locus 19319 :

2 alleles

98.0% missing data

2	(0.610) 0.616 0.614

1	(0.390) 0.384 0.386

Locus 19320 :

2 alleles

96.5% missing data

1	(0.848) 0.862 0.839

3	(0.152) 0.138 0.161

Locus 19321 :

2 alleles

99.2% missing data

2	(0.385) 0.379 0.395

1	(0.615) 0.621 0.605

Locus 19322 :

3 alleles

98.8% missing data

2	(0.341) 0.344 0.340

3	(0.400) 0.395 0.400

1	(0.259) 0.261 0.260

Locus 19323 :

2 alleles

98.8% missing data

3	(0.275) 0.275 0.271

2	(0.725) 0.725 0.729

Locus 19324 :

2 alleles

99.2% missing data

2	(0.574) 0.575 0.573

3	(0.426) 0.425 0.427

Locus 19325 :

2 alleles

99.2% missing data

2	(0.367) 0.363 0.372

1	(0.633) 0.637 0.628

Locus 19326 :

2 alleles

99.2% missing data

4	(0.269) 0.266 0.276

1	(0.731) 0.734 0.724

Locus 19327 :

2 alleles

98.8% missing data

1	(0.519) 0.517 0.526

2	(0.481) 0.483 0.474

Locus 19328 :

2 alleles

98.8% missing data

3	(0.624) 0.630 0.623

2	(0.376) 0.370 0.377

Locus 19329 :

2 alleles

98.4% missing data

2	(0.267) 0.278 0.258

4	(0.733) 0.722 0.742

Locus 19330 :

2 alleles

99.2% missing data

1	(0.389) 0.380 0.401

2	(0.611) 0.620 0.599

Locus 19331 :

2 alleles

98.8% missing data

2	(0.667) 0.664 0.671

3	(0.333) 0.336 0.329

Locus 19332 :

2 alleles

98.8% missing data

2	(0.563) 0.566 0.563

3	(0.437) 0.434 0.437

Locus 19333 :

2 alleles

98.4% missing data

2	(0.624) 0.631 0.623

3	(0.376) 0.369 0.377

Locus 19334 :

2 alleles

98.4% missing data

1	(0.514) 0.525 0.507

3	(0.486) 0.475 0.493

Locus 19335 :

2 alleles

70.7% missing data

2	(0.983) 0.984 0.984

1	(0.017) 0.016 0.016

Locus 19336 :

2 alleles

97.3% missing data

3	(0.195) 0.192 0.197

4	(0.805) 0.808 0.803

Locus 19337 :

2 alleles

99.2% missing data

1	(0.487) 0.492 0.489

3	(0.513) 0.508 0.511

Locus 19338 :

2 alleles

99.2% missing data

2	(0.490) 0.495 0.486

1	(0.510) 0.505 0.514

Locus 19339 :

2 alleles

99.2% missing data

4	(0.516) 0.511 0.513

2	(0.484) 0.489 0.487

Locus 19340 :

2 alleles

99.2% missing data

1	(0.548) 0.549 0.549

4	(0.452) 0.451 0.451

Locus 19341 :

3 alleles

96.5% missing data

2	(0.668) 0.666 0.688

3	(0.169) 0.165 0.162

1	(0.163) 0.169 0.150

Locus 19342 :

2 alleles

99.2% missing data

4	(0.682) 0.685 0.682

1	(0.318) 0.315 0.318

Locus 19343 :

2 alleles

99.2% missing data

3	(0.750) 0.753 0.744

2	(0.250) 0.247 0.256

Locus 19344 :

2 alleles

99.2% missing data

1	(0.668) 0.676 0.662

4	(0.332) 0.324 0.338

Locus 19345 :

2 alleles

99.2% missing data

1	(0.789) 0.790 0.784

3	(0.211) 0.210 0.216

Locus 19346 :

2 alleles

99.2% missing data

4	(0.615) 0.608 0.617

3	(0.385) 0.392 0.383

Locus 19347 :

2 alleles

98.8% missing data

3	(0.414) 0.413 0.410

4	(0.586) 0.587 0.590

Locus 19348 :

2 alleles

99.2% missing data

1	(0.521) 0.516 0.528

4	(0.479) 0.484 0.472

Locus 19349 :

2 alleles

99.2% missing data

2	(0.402) 0.405 0.397

4	(0.598) 0.595 0.603

Locus 19350 :

2 alleles

99.2% missing data

1	(0.607) 0.608 0.600

2	(0.393) 0.392 0.400

Locus 19351 :

2 alleles

99.2% missing data

1	(0.647) 0.653 0.644

2	(0.353) 0.347 0.356

Locus 19352 :

2 alleles

99.2% missing data

2	(0.419) 0.412 0.423

3	(0.581) 0.588 0.577

Locus 19353 :

2 alleles

98.4% missing data

3	(0.580) 0.581 0.586

1	(0.420) 0.419 0.414

Locus 19354 :

2 alleles

99.2% missing data

3	(0.663) 0.658 0.672

4	(0.337) 0.342 0.328

Locus 19355 :

2 alleles

99.2% missing data

4	(0.331) 0.334 0.329

2	(0.669) 0.666 0.671

Locus 19356 :

2 alleles

0.8% missing data

2	(0.987) 0.991 0.993

4	(0.013) 0.009 0.007

Locus 19357 :

2 alleles

98.8% missing data

4	(0.688) 0.700 0.688

2	(0.312) 0.300 0.312

Locus 19358 :

2 alleles

99.2% missing data

1	(0.625) 0.619 0.632

2	(0.375) 0.381 0.368

Locus 19359 :

2 alleles

99.2% missing data

4	(0.580) 0.585 0.576

1	(0.420) 0.415 0.424

Locus 19360 :

2 alleles

99.2% missing data

2	(0.637) 0.640 0.636

1	(0.363) 0.360 0.364

Locus 19361 :

2 alleles

98.8% missing data

3	(0.409) 0.409 0.408

4	(0.591) 0.591 0.592

Locus 19362 :

2 alleles

99.2% missing data

3	(0.636) 0.633 0.639

1	(0.364) 0.367 0.361

Locus 19363 :

2 alleles

99.2% missing data

4	(0.675) 0.673 0.668

2	(0.325) 0.327 0.332

Locus 19364 :

2 alleles

99.2% missing data

4	(0.229) 0.224 0.236

1	(0.771) 0.776 0.764

Locus 19365 :

2 alleles

99.2% missing data

2	(0.651) 0.647 0.650

1	(0.349) 0.353 0.350

Locus 19366 :

2 alleles

99.2% missing data

1	(0.155) 0.152 0.158

3	(0.845) 0.848 0.842

Locus 19367 :

2 alleles

99.2% missing data

1	(0.548) 0.540 0.556

2	(0.452) 0.460 0.444

Locus 19368 :

3 alleles

98.8% missing data

4	(0.234) 0.230 0.241

3	(0.553) 0.563 0.544

2	(0.213) 0.207 0.215

Locus 19369 :

2 alleles

98.0% missing data

3	(0.544) 0.538 0.556

4	(0.456) 0.462 0.444

Locus 19370 :

2 alleles

99.2% missing data

4	(0.266) 0.259 0.272

3	(0.734) 0.741 0.728

Locus 19371 :

2 alleles

97.7% missing data

2	(0.722) 0.724 0.725

1	(0.278) 0.276 0.275

Locus 19372 :

2 alleles

96.1% missing data

4	(0.224) 0.208 0.231

1	(0.776) 0.792 0.769

Locus 19373 :

2 alleles

91.0% missing data

1	(0.889) 0.907 0.883

4	(0.111) 0.093 0.117

Locus 19374 :

2 alleles

99.2% missing data

4	(0.270) 0.266 0.271

3	(0.730) 0.734 0.729

Locus 19375 :

2 alleles

99.2% missing data

3	(0.652) 0.653 0.652

4	(0.348) 0.347 0.348

Locus 19376 :

3 alleles

90.2% missing data

2	(0.888) 0.910 0.873

1	(0.057) 0.044 0.066

4	(0.055) 0.046 0.061

Locus 19377 :

3 alleles

98.8% missing data

3	(0.341) 0.336 0.338

1	(0.302) 0.300 0.305

2	(0.357) 0.363 0.356

Locus 19378 :

2 alleles

93.4% missing data

4	(0.630) 0.634 0.633

1	(0.370) 0.366 0.367

Locus 19379 :

2 alleles

98.8% missing data

2	(0.528) 0.521 0.537

3	(0.472) 0.479 0.463

Locus 19380 :

3 alleles

96.1% missing data

2	(0.144) 0.148 0.137

4	(0.714) 0.701 0.733

3	(0.142) 0.151 0.131

Locus 19381 :

2 alleles

98.4% missing data

3	(0.625) 0.625 0.633

4	(0.375) 0.375 0.367

Locus 19382 :

2 alleles

99.2% missing data

4	(0.661) 0.659 0.667

3	(0.339) 0.341 0.333

Locus 19383 :

2 alleles

99.2% missing data

4	(0.343) 0.333 0.350

3	(0.657) 0.667 0.650

Locus 19384 :

2 alleles

98.4% missing data

3	(0.477) 0.485 0.476

1	(0.523) 0.515 0.524

Locus 19385 :

2 alleles

99.2% missing data

4	(0.418) 0.416 0.421

1	(0.582) 0.584 0.579

Locus 19386 :

2 alleles

99.2% missing data

1	(0.276) 0.281 0.280

4	(0.724) 0.719 0.720

Locus 19387 :

2 alleles

99.2% missing data

4	(0.393) 0.391 0.393

2	(0.607) 0.609 0.607

Locus 19388 :

2 alleles

97.3% missing data

2	(0.739) 0.748 0.743

4	(0.261) 0.252 0.257

Locus 19389 :

3 alleles

98.4% missing data

2	(0.388) 0.380 0.402

3	(0.213) 0.220 0.204

4	(0.400) 0.400 0.394

Locus 19390 :

2 alleles

96.1% missing data

2	(0.856) 0.859 0.862

3	(0.144) 0.141 0.138

Locus 19391 :

3 alleles

98.8% missing data

4	(0.165) 0.166 0.164

2	(0.396) 0.388 0.403

1	(0.439) 0.446 0.433

Locus 19392 :

2 alleles

99.2% missing data

3	(0.394) 0.381 0.397

1	(0.606) 0.619 0.603

Locus 19393 :

2 alleles

99.2% missing data

4	(0.576) 0.580 0.565

1	(0.424) 0.420 0.435

Locus 19394 :

2 alleles

99.2% missing data

3	(0.540) 0.531 0.545

4	(0.460) 0.469 0.455

Locus 19395 :

2 alleles

99.2% missing data

3	(0.519) 0.513 0.518

2	(0.481) 0.487 0.482

Locus 19396 :

2 alleles

5.9% missing data

3	(0.988) 0.990 0.993

2	(0.012) 0.010 0.007

Locus 19397 :

2 alleles

90.2% missing data

3	(0.894) 0.913 0.887

2	(0.106) 0.087 0.113

Locus 19398 :

2 alleles

99.2% missing data

4	(0.293) 0.290 0.301

3	(0.707) 0.710 0.699

Locus 19399 :

2 alleles

99.2% missing data

4	(0.171) 0.169 0.178

1	(0.829) 0.831 0.822

Locus 19400 :

2 alleles

99.2% missing data

3	(0.590) 0.589 0.589

1	(0.410) 0.411 0.411

Locus 19401 :

2 alleles

99.2% missing data

1	(0.467) 0.466 0.464

3	(0.533) 0.534 0.536

Locus 19402 :

2 alleles

98.8% missing data

2	(0.453) 0.453 0.451

3	(0.547) 0.547 0.549

Locus 19403 :

2 alleles

99.2% missing data

2	(0.459) 0.458 0.457

4	(0.541) 0.542 0.543

Locus 19404 :

2 alleles

99.2% missing data

1	(0.533) 0.528 0.533

3	(0.467) 0.472 0.467

Locus 19405 :

2 alleles

93.8% missing data

3	(0.784) 0.778 0.805

4	(0.216) 0.222 0.195

Locus 19406 :

2 alleles

90.6% missing data

2	(0.881) 0.887 0.882

1	(0.119) 0.113 0.118

Locus 19407 :

2 alleles

96.9% missing data

4	(0.853) 0.855 0.854

3	(0.147) 0.145 0.146

Locus 19408 :

2 alleles

99.2% missing data

2	(0.588) 0.582 0.592

3	(0.412) 0.418 0.408

Locus 19409 :

2 alleles

53.1% missing data

4	(0.976) 0.980 0.983

2	(0.024) 0.020 0.017

Locus 19410 :

2 alleles

99.2% missing data

2	(0.662) 0.665 0.658

4	(0.338) 0.335 0.342

Locus 19411 :

2 alleles

96.1% missing data

4	(0.818) 0.831 0.814

3	(0.182) 0.169 0.186

Locus 19412 :

2 alleles

99.2% missing data

3	(0.331) 0.327 0.334

1	(0.669) 0.673 0.666

Locus 19413 :

2 alleles

91.0% missing data

3	(0.933) 0.944 0.925

4	(0.067) 0.056 0.075

Locus 19414 :

2 alleles

96.1% missing data

4	(0.836) 0.838 0.840

3	(0.164) 0.162 0.160

Locus 19415 :

2 alleles

98.4% missing data

3	(0.627) 0.633 0.624

4	(0.373) 0.367 0.376

Locus 19416 :

2 alleles

99.2% missing data

1	(0.294) 0.291 0.292

4	(0.706) 0.709 0.708

Locus 19417 :

2 alleles

99.2% missing data

3	(0.753) 0.746 0.759

4	(0.247) 0.254 0.241

Locus 19418 :

2 alleles

99.2% missing data

3	(0.549) 0.543 0.558

4	(0.451) 0.457 0.442

Locus 19419 :

2 alleles

99.2% missing data

4	(0.494) 0.496 0.499

3	(0.506) 0.504 0.501

Locus 19420 :

2 alleles

97.3% missing data

3	(0.285) 0.294 0.270

4	(0.715) 0.706 0.730

Locus 19421 :

2 alleles

99.2% missing data

3	(0.465) 0.463 0.464

1	(0.535) 0.537 0.536

Locus 19422 :

2 alleles

98.4% missing data

1	(0.503) 0.492 0.515

4	(0.497) 0.508 0.485

Locus 19423 :

2 alleles

91.4% missing data

2	(0.909) 0.926 0.902

3	(0.091) 0.074 0.098

Locus 19424 :

2 alleles

99.2% missing data

3	(0.704) 0.700 0.709

1	(0.296) 0.300 0.291

Locus 19425 :

2 alleles

99.2% missing data

1	(0.413) 0.413 0.416

2	(0.587) 0.587 0.584

Locus 19426 :

2 alleles

99.2% missing data

3	(0.441) 0.438 0.437

2	(0.559) 0.562 0.563

Locus 19427 :

2 alleles

99.2% missing data

4	(0.512) 0.518 0.501

3	(0.488) 0.482 0.499

Locus 19428 :

2 alleles

99.2% missing data

1	(0.470) 0.461 0.481

3	(0.530) 0.539 0.519

Locus 19429 :

2 alleles

99.2% missing data

3	(0.576) 0.577 0.583

4	(0.424) 0.423 0.417

Locus 19430 :

2 alleles

99.2% missing data

3	(0.382) 0.374 0.383

1	(0.618) 0.626 0.617

Locus 19431 :

2 alleles

99.2% missing data

4	(0.555) 0.553 0.550

3	(0.445) 0.447 0.450

Locus 19432 :

2 alleles

99.2% missing data

4	(0.666) 0.672 0.660

2	(0.334) 0.328 0.340

Locus 19433 :

2 alleles

99.2% missing data

1	(0.700) 0.698 0.696

2	(0.300) 0.302 0.304

Locus 19434 :

2 alleles

99.2% missing data

1	(0.679) 0.675 0.680

2	(0.321) 0.325 0.320

Locus 19435 :

2 alleles

99.2% missing data

4	(0.275) 0.271 0.283

1	(0.725) 0.729 0.717

Locus 19436 :

2 alleles

91.4% missing data

4	(0.775) 0.747 0.807

3	(0.225) 0.253 0.193

Locus 19437 :

2 alleles

99.2% missing data

4	(0.565) 0.563 0.567

3	(0.435) 0.437 0.433

Locus 19438 :

2 alleles

99.2% missing data

1	(0.460) 0.464 0.458

3	(0.540) 0.536 0.542

Locus 19439 :

2 alleles

92.6% missing data

4	(0.921) 0.913 0.931

1	(0.079) 0.087 0.069

Locus 19440 :

2 alleles

99.2% missing data

3	(0.426) 0.421 0.434

4	(0.574) 0.579 0.566

Locus 19441 :

2 alleles

99.2% missing data

2	(0.520) 0.522 0.516

4	(0.480) 0.478 0.484

Locus 19442 :

2 alleles

99.2% missing data

1	(0.566) 0.562 0.569

2	(0.434) 0.438 0.431

Locus 19443 :

2 alleles

98.0% missing data

4	(0.800) 0.807 0.795

1	(0.200) 0.193 0.205

Locus 19444 :

2 alleles

94.5% missing data

4	(0.912) 0.910 0.918

1	(0.088) 0.090 0.082

Locus 19445 :

2 alleles

99.2% missing data

3	(0.568) 0.559 0.571

4	(0.432) 0.441 0.429

Locus 19446 :

2 alleles

99.2% missing data

2	(0.492) 0.495 0.487

1	(0.508) 0.505 0.513

Locus 19447 :

2 alleles

99.2% missing data

1	(0.292) 0.292 0.291

4	(0.708) 0.708 0.709

Locus 19448 :

2 alleles

99.2% missing data

2	(0.486) 0.485 0.480

3	(0.514) 0.515 0.520

Locus 19449 :

2 alleles

99.2% missing data

3	(0.673) 0.676 0.668

2	(0.327) 0.324 0.332

Locus 19450 :

2 alleles

99.2% missing data

3	(0.442) 0.438 0.444

4	(0.558) 0.562 0.556

Locus 19451 :

2 alleles

99.2% missing data

2	(0.667) 0.658 0.671

1	(0.333) 0.342 0.329

Locus 19452 :

2 alleles

91.0% missing data

2	(0.889) 0.910 0.878

4	(0.111) 0.090 0.122

Locus 19453 :

3 alleles

98.8% missing data

4	(0.422) 0.415 0.431

1	(0.178) 0.183 0.171

3	(0.400) 0.402 0.398

Locus 19454 :

2 alleles

99.2% missing data

4	(0.426) 0.422 0.431

1	(0.574) 0.578 0.569

Locus 19455 :

2 alleles

91.0% missing data

1	(0.942) 0.954 0.933

2	(0.058) 0.046 0.067

Locus 19456 :

2 alleles

99.2% missing data

4	(0.730) 0.723 0.730

2	(0.270) 0.277 0.270

Locus 19457 :

2 alleles

94.5% missing data

2	(0.333) 0.328 0.335

3	(0.667) 0.672 0.665

Locus 19458 :

2 alleles

99.2% missing data

2	(0.394) 0.386 0.399

4	(0.606) 0.614 0.601

Locus 19459 :

2 alleles

99.2% missing data

4	(0.370) 0.365 0.374

1	(0.630) 0.635 0.626

Locus 19460 :

3 alleles

98.8% missing data

1	(0.417) 0.423 0.414

3	(0.453) 0.442 0.459

4	(0.130) 0.135 0.127

Locus 19461 :

2 alleles

98.8% missing data

1	(0.628) 0.632 0.617

4	(0.372) 0.368 0.383

Locus 19462 :

2 alleles

98.8% missing data

4	(0.362) 0.360 0.372

3	(0.638) 0.640 0.628

Locus 19463 :

2 alleles

98.8% missing data

4	(0.588) 0.580 0.589

1	(0.412) 0.420 0.411

Locus 19464 :

2 alleles

99.2% missing data

2	(0.215) 0.219 0.215

4	(0.785) 0.781 0.785

Locus 19465 :

2 alleles

98.4% missing data

1	(0.396) 0.403 0.390

4	(0.604) 0.597 0.610

Locus 19466 :

2 alleles

99.2% missing data

4	(0.611) 0.614 0.605

2	(0.389) 0.386 0.395

Locus 19467 :

2 alleles

99.2% missing data

4	(0.582) 0.581 0.578

3	(0.418) 0.419 0.422

Locus 19468 :

2 alleles

99.2% missing data

1	(0.512) 0.513 0.507

2	(0.488) 0.487 0.493

Locus 19469 :

2 alleles

99.2% missing data

4	(0.307) 0.307 0.315

1	(0.693) 0.693 0.685

Locus 19470 :

2 alleles

99.2% missing data

3	(0.317) 0.309 0.319

4	(0.683) 0.691 0.681

Locus 19471 :

2 alleles

99.2% missing data

2	(0.686) 0.682 0.691

1	(0.314) 0.318 0.309

Locus 19472 :

2 alleles

99.2% missing data

4	(0.253) 0.252 0.260

3	(0.747) 0.748 0.740

Locus 19473 :

2 alleles

0.0% missing data

1	(0.983) 0.987 0.989

3	(0.017) 0.013 0.011

Locus 19474 :

2 alleles

99.2% missing data

3	(0.786) 0.790 0.785

4	(0.214) 0.210 0.215

Locus 19475 :

2 alleles

98.8% missing data

3	(0.187) 0.183 0.193

2	(0.813) 0.817 0.807

Locus 19476 :

2 alleles

99.2% missing data

4	(0.519) 0.528 0.517

2	(0.481) 0.472 0.483

Locus 19477 :

2 alleles

98.8% missing data

2	(0.803) 0.801 0.807

4	(0.197) 0.199 0.193

Locus 19478 :

3 alleles

98.8% missing data

3	(0.089) 0.092 0.088

4	(0.191) 0.185 0.203

2	(0.720) 0.723 0.709

Locus 19479 :

2 alleles

0.0% missing data

4	(0.976) 0.983 0.980

1	(0.024) 0.017 0.020

Locus 19480 :

2 alleles

99.2% missing data

4	(0.222) 0.221 0.233

2	(0.778) 0.779 0.767

Locus 19481 :

2 alleles

99.2% missing data

1	(0.197) 0.192 0.201

4	(0.803) 0.808 0.799

Locus 19482 :

2 alleles

99.2% missing data

4	(0.629) 0.628 0.632

3	(0.371) 0.372 0.368

Locus 19483 :

2 alleles

99.2% missing data

3	(0.699) 0.692 0.702

2	(0.301) 0.308 0.298

Locus 19484 :

2 alleles

99.2% missing data

4	(0.555) 0.553 0.553

2	(0.445) 0.447 0.447

Locus 19485 :

2 alleles

97.7% missing data

3	(0.639) 0.644 0.640

2	(0.361) 0.356 0.360

Locus 19486 :

2 alleles

98.8% missing data

3	(0.590) 0.588 0.597

1	(0.410) 0.412 0.403

Locus 19487 :

2 alleles

98.0% missing data

3	(0.644) 0.662 0.645

1	(0.356) 0.338 0.355

Locus 19488 :

2 alleles

98.4% missing data

4	(0.562) 0.562 0.565

3	(0.438) 0.438 0.435

Locus 19489 :

2 alleles

92.6% missing data

2	(0.937) 0.941 0.939

4	(0.063) 0.059 0.061

Locus 19490 :

2 alleles

99.2% missing data

4	(0.338) 0.337 0.335

1	(0.662) 0.663 0.665

Locus 19491 :

2 alleles

96.1% missing data

4	(0.137) 0.128 0.141

3	(0.863) 0.872 0.859

Locus 19492 :

2 alleles

96.5% missing data

3	(0.827) 0.835 0.825

4	(0.173) 0.165 0.175

Locus 19493 :

4 alleles

91.4% missing data

3	(0.070) 0.069 0.064

1	(0.237) 0.223 0.261

4	(0.461) 0.489 0.431

2	(0.232) 0.219 0.244

Locus 19494 :

2 alleles

98.8% missing data

1	(0.522) 0.515 0.530

3	(0.478) 0.485 0.470

Locus 19495 :

2 alleles

94.1% missing data

2	(0.873) 0.877 0.879

4	(0.127) 0.123 0.121

Locus 19496 :

2 alleles

99.2% missing data

4	(0.271) 0.271 0.275

2	(0.729) 0.729 0.725

Locus 19497 :

2 alleles

99.2% missing data

2	(0.215) 0.214 0.213

4	(0.785) 0.786 0.787

Locus 19498 :

2 alleles

99.2% missing data

4	(0.347) 0.347 0.347

1	(0.653) 0.653 0.653

Locus 19499 :

2 alleles

98.4% missing data

3	(0.408) 0.403 0.413

1	(0.592) 0.597 0.587

Locus 19500 :

2 alleles

0.0% missing data

3	(0.988) 0.985 0.996

1	(0.012) 0.015 0.004

Locus 19501 :

2 alleles

99.2% missing data

3	(0.563) 0.562 0.563

2	(0.437) 0.438 0.437

Locus 19502 :

2 alleles

99.2% missing data

2	(0.550) 0.544 0.551

4	(0.450) 0.456 0.449

Locus 19503 :

3 alleles

98.0% missing data

4	(0.245) 0.241 0.258

3	(0.356) 0.347 0.353

1	(0.399) 0.412 0.389

Locus 19504 :

3 alleles

98.0% missing data

2	(0.235) 0.234 0.236

4	(0.448) 0.458 0.446

3	(0.317) 0.308 0.318

Locus 19505 :

2 alleles

99.2% missing data

2	(0.428) 0.426 0.426

4	(0.572) 0.574 0.574

Locus 19506 :

2 alleles

99.2% missing data

3	(0.460) 0.463 0.451

4	(0.540) 0.537 0.549

Locus 19507 :

2 alleles

99.2% missing data

4	(0.440) 0.434 0.446

3	(0.560) 0.566 0.554

Locus 19508 :

2 alleles

99.2% missing data

3	(0.255) 0.255 0.253

1	(0.745) 0.745 0.747

Locus 19509 :

2 alleles

99.2% missing data

2	(0.382) 0.376 0.383

4	(0.618) 0.624 0.617

Locus 19510 :

2 alleles

99.2% missing data

1	(0.420) 0.414 0.422

3	(0.580) 0.586 0.578

Locus 19511 :

2 alleles

99.2% missing data

2	(0.398) 0.407 0.391

1	(0.602) 0.593 0.609

Locus 19512 :

2 alleles

79.3% missing data

1	(0.538) 0.538 0.537

2	(0.462) 0.462 0.463

Locus 19513 :

2 alleles

96.9% missing data

4	(0.481) 0.494 0.465

2	(0.519) 0.506 0.535

Locus 19514 :

2 alleles

8.6% missing data

4	(0.985) 0.977 0.995

3	(0.015) 0.023 0.005

Locus 19515 :

2 alleles

99.2% missing data

4	(0.563) 0.568 0.565

2	(0.437) 0.432 0.435

Locus 19516 :

2 alleles

99.2% missing data

2	(0.246) 0.244 0.245

3	(0.754) 0.756 0.755

Locus 19517 :

2 alleles

98.0% missing data

1	(0.830) 0.822 0.838

4	(0.170) 0.178 0.162

Locus 19518 :

2 alleles

99.2% missing data

2	(0.738) 0.738 0.741

3	(0.262) 0.262 0.259

Locus 19519 :

2 alleles

99.2% missing data

2	(0.341) 0.346 0.341

4	(0.659) 0.654 0.659

Locus 19520 :

2 alleles

99.2% missing data

1	(0.505) 0.507 0.505

2	(0.495) 0.493 0.495

Locus 19521 :

3 alleles

96.5% missing data

3	(0.171) 0.165 0.174

1	(0.665) 0.682 0.657

4	(0.164) 0.153 0.169

Locus 19522 :

2 alleles

97.7% missing data

1	(0.589) 0.583 0.603

4	(0.411) 0.417 0.397

Locus 19523 :

2 alleles

98.8% missing data

2	(0.756) 0.753 0.761

1	(0.244) 0.247 0.239

Locus 19524 :

2 alleles

97.3% missing data

1	(0.285) 0.283 0.276

2	(0.715) 0.717 0.724

Locus 19525 :

3 alleles

92.2% missing data

2	(0.812) 0.790 0.832

4	(0.058) 0.057 0.058

3	(0.129) 0.153 0.110

Locus 19526 :

2 alleles

99.2% missing data

3	(0.364) 0.373 0.357

1	(0.636) 0.627 0.643

Locus 19527 :

2 alleles

99.2% missing data

2	(0.360) 0.359 0.361

4	(0.640) 0.641 0.639

Locus 19528 :

2 alleles

99.2% missing data

3	(0.411) 0.416 0.412

2	(0.589) 0.584 0.588

Locus 19529 :

2 alleles

99.2% missing data

4	(0.290) 0.287 0.298

3	(0.710) 0.713 0.702

Locus 19530 :

2 alleles

97.7% missing data

1	(0.736) 0.731 0.744

3	(0.264) 0.269 0.256

Locus 19531 :

2 alleles

99.2% missing data

3	(0.702) 0.702 0.710

2	(0.298) 0.298 0.290

Locus 19532 :

2 alleles

96.1% missing data

1	(0.183) 0.171 0.189

4	(0.817) 0.829 0.811

Locus 19533 :

2 alleles

90.6% missing data

4	(0.901) 0.919 0.897

3	(0.099) 0.081 0.103

Locus 19534 :

2 alleles

99.2% missing data

4	(0.489) 0.479 0.503

1	(0.511) 0.521 0.497

Locus 19535 :

2 alleles

99.2% missing data

1	(0.800) 0.800 0.793

4	(0.200) 0.200 0.207

Locus 19536 :

2 alleles

99.2% missing data

1	(0.342) 0.339 0.344

2	(0.658) 0.661 0.656

Locus 19537 :

2 alleles

99.2% missing data

1	(0.570) 0.563 0.579

2	(0.430) 0.437 0.421

Locus 19538 :

2 alleles

99.2% missing data

4	(0.567) 0.563 0.571

1	(0.433) 0.437 0.429

Locus 19539 :

2 alleles

96.1% missing data

1	(0.782) 0.799 0.775

3	(0.218) 0.201 0.225

Locus 19540 :

2 alleles

96.1% missing data

3	(0.205) 0.192 0.212

2	(0.795) 0.808 0.788

Locus 19541 :

2 alleles

95.7% missing data

4	(0.895) 0.892 0.900

3	(0.105) 0.108 0.100

Locus 19542 :

2 alleles

99.2% missing data

3	(0.399) 0.399 0.401

4	(0.601) 0.601 0.599

Locus 19543 :

2 alleles

99.2% missing data

2	(0.402) 0.401 0.404

3	(0.598) 0.599 0.596

Locus 19544 :

2 alleles

98.8% missing data

2	(0.683) 0.676 0.692

4	(0.317) 0.324 0.308

Locus 19545 :

2 alleles

98.8% missing data

3	(0.325) 0.319 0.327

1	(0.675) 0.681 0.673

Locus 19546 :

3 alleles

98.8% missing data

4	(0.300) 0.302 0.296

2	(0.329) 0.326 0.329

1	(0.371) 0.372 0.375

Locus 19547 :

2 alleles

99.2% missing data

4	(0.751) 0.755 0.749

3	(0.249) 0.245 0.251

Locus 19548 :

2 alleles

98.8% missing data

2	(0.703) 0.702 0.701

4	(0.297) 0.298 0.299

Locus 19549 :

2 alleles

99.2% missing data

2	(0.742) 0.742 0.734

4	(0.258) 0.258 0.266

Locus 19550 :

2 alleles

96.5% missing data

2	(0.833) 0.844 0.829

3	(0.167) 0.156 0.171

Locus 19551 :

2 alleles

96.1% missing data

4	(0.793) 0.806 0.785

2	(0.207) 0.194 0.215

Locus 19552 :

3 alleles

98.8% missing data

4	(0.366) 0.368 0.364

1	(0.344) 0.339 0.342

3	(0.290) 0.293 0.294

Locus 19553 :

2 alleles

99.2% missing data

1	(0.355) 0.361 0.356

3	(0.645) 0.639 0.644

Locus 19554 :

2 alleles

99.2% missing data

2	(0.475) 0.471 0.488

4	(0.525) 0.529 0.512

Locus 19555 :

2 alleles

99.2% missing data

4	(0.299) 0.297 0.299

1	(0.701) 0.703 0.701

Locus 19556 :

2 alleles

99.2% missing data

1	(0.403) 0.393 0.405

4	(0.597) 0.607 0.595

Locus 19557 :

2 alleles

99.2% missing data

4	(0.663) 0.666 0.662

2	(0.337) 0.334 0.338

Locus 19558 :

2 alleles

91.0% missing data

3	(0.877) 0.894 0.869

4	(0.123) 0.106 0.131

Locus 19559 :

2 alleles

99.2% missing data

1	(0.534) 0.538 0.533

2	(0.466) 0.462 0.467

Locus 19560 :

2 alleles

98.8% missing data

2	(0.731) 0.730 0.727

3	(0.269) 0.270 0.273

Locus 19561 :

2 alleles

99.2% missing data

1	(0.504) 0.504 0.499

4	(0.496) 0.496 0.501

Locus 19562 :

3 alleles

98.8% missing data

4	(0.341) 0.344 0.337

3	(0.440) 0.439 0.440

1	(0.219) 0.217 0.223

Locus 19563 :

2 alleles

18.0% missing data

1	(0.985) 0.972 0.995

4	(0.015) 0.028 0.005

Locus 19564 :

3 alleles

92.6% missing data

3	(0.853) 0.854 0.861

2	(0.081) 0.081 0.076

4	(0.065) 0.065 0.063

Locus 19565 :

2 alleles

98.4% missing data

4	(0.405) 0.403 0.402

2	(0.595) 0.597 0.598

Locus 19566 :

2 alleles

94.9% missing data

4	(0.829) 0.840 0.825

2	(0.171) 0.160 0.175

Locus 19567 :

2 alleles

98.0% missing data

4	(0.143) 0.143 0.146

3	(0.857) 0.857 0.854

Locus 19568 :

2 alleles

99.2% missing data

3	(0.510) 0.504 0.512

4	(0.490) 0.496 0.488

Locus 19569 :

2 alleles

98.0% missing data

4	(0.389) 0.386 0.386

1	(0.611) 0.614 0.614

Locus 19570 :

2 alleles

99.2% missing data

3	(0.550) 0.547 0.551

4	(0.450) 0.453 0.449

Locus 19571 :

2 alleles

99.2% missing data

3	(0.491) 0.485 0.495

4	(0.509) 0.515 0.505

Locus 19572 :

2 alleles

99.2% missing data

4	(0.427) 0.431 0.426

2	(0.573) 0.569 0.574

Locus 19573 :

2 alleles

97.7% missing data

4	(0.491) 0.489 0.490

2	(0.509) 0.511 0.510

Locus 19574 :

2 alleles

99.2% missing data

1	(0.617) 0.607 0.622

4	(0.383) 0.393 0.378

Locus 19575 :

2 alleles

97.3% missing data

1	(0.378) 0.379 0.375

2	(0.622) 0.621 0.625

Locus 19576 :

2 alleles

99.2% missing data

3	(0.666) 0.668 0.663

1	(0.334) 0.332 0.337

Locus 19577 :

2 alleles

99.2% missing data

3	(0.131) 0.128 0.139

1	(0.869) 0.872 0.861

Locus 19578 :

2 alleles

96.1% missing data

3	(0.809) 0.825 0.801

2	(0.191) 0.175 0.199

Locus 19579 :

2 alleles

99.2% missing data

2	(0.774) 0.767 0.777

4	(0.226) 0.233 0.223

Locus 19580 :

2 alleles

98.4% missing data

4	(0.618) 0.626 0.618

3	(0.382) 0.374 0.382

Locus 19581 :

3 alleles

89.5% missing data

2	(0.833) 0.869 0.810

4	(0.087) 0.069 0.101

1	(0.080) 0.063 0.088

Locus 19582 :

2 alleles

97.3% missing data

3	(0.783) 0.782 0.788

2	(0.217) 0.218 0.212

Locus 19583 :

2 alleles

99.2% missing data

2	(0.332) 0.327 0.341

4	(0.668) 0.673 0.659

Locus 19584 :

2 alleles

99.2% missing data

4	(0.706) 0.698 0.709

1	(0.294) 0.302 0.291

Locus 19585 :

2 alleles

97.3% missing data

4	(0.483) 0.472 0.484

2	(0.517) 0.528 0.516

Locus 19586 :

2 alleles

99.2% missing data

3	(0.500) 0.497 0.508

2	(0.500) 0.503 0.492

Locus 19587 :

2 alleles

97.7% missing data

3	(0.188) 0.186 0.187

4	(0.812) 0.814 0.813

Locus 19588 :

2 alleles

99.2% missing data

3	(0.432) 0.438 0.430

4	(0.568) 0.562 0.570

Locus 19589 :

2 alleles

90.6% missing data

3	(0.917) 0.935 0.909

4	(0.083) 0.065 0.091

Locus 19590 :

2 alleles

98.8% missing data

2	(0.786) 0.789 0.783

3	(0.214) 0.211 0.217

Locus 19591 :

2 alleles

0.0% missing data

2	(0.919) 0.770 0.976

3	(0.081) 0.230 0.024

Locus 19592 :

2 alleles

0.0% missing data

2	(0.990) 0.993 0.994

1	(0.010) 0.007 0.006

Locus 19593 :

2 alleles

99.2% missing data

2	(0.518) 0.511 0.526

4	(0.482) 0.489 0.474

Locus 19594 :

2 alleles

99.2% missing data

3	(0.675) 0.667 0.681

4	(0.325) 0.333 0.319

Locus 19595 :

2 alleles

99.2% missing data

4	(0.724) 0.724 0.723

2	(0.276) 0.276 0.277

Locus 19596 :

2 alleles

99.2% missing data

3	(0.599) 0.594 0.595

1	(0.401) 0.406 0.405

Locus 19597 :

2 alleles

99.2% missing data

3	(0.296) 0.297 0.294

2	(0.704) 0.703 0.706

Locus 19598 :

2 alleles

99.2% missing data

4	(0.470) 0.468 0.469

3	(0.530) 0.532 0.531

Locus 19599 :

2 alleles

93.8% missing data

4	(0.872) 0.870 0.885

2	(0.128) 0.130 0.115

Locus 19600 :

2 alleles

98.4% missing data

1	(0.669) 0.665 0.676

3	(0.331) 0.335 0.324

Locus 19601 :

2 alleles

99.2% missing data

3	(0.608) 0.601 0.611

4	(0.392) 0.399 0.389

Locus 19602 :

2 alleles

99.2% missing data

3	(0.473) 0.476 0.465

1	(0.527) 0.524 0.535

Locus 19603 :

2 alleles

99.2% missing data

3	(0.671) 0.671 0.666

4	(0.329) 0.329 0.334

Locus 19604 :

2 alleles

99.2% missing data

4	(0.570) 0.572 0.571

2	(0.430) 0.428 0.429

Locus 19605 :

2 alleles

96.9% missing data

3	(0.196) 0.192 0.195

2	(0.804) 0.808 0.805

Locus 19606 :

2 alleles

98.4% missing data

3	(0.628) 0.636 0.627

2	(0.372) 0.364 0.373

Locus 19607 :

2 alleles

98.8% missing data

2	(0.556) 0.559 0.548

1	(0.444) 0.441 0.452

Locus 19608 :

2 alleles

99.2% missing data

3	(0.401) 0.403 0.398

1	(0.599) 0.597 0.602

Locus 19609 :

2 alleles

99.2% missing data

2	(0.430) 0.428 0.425

4	(0.570) 0.572 0.575

Locus 19610 :

2 alleles

99.2% missing data

2	(0.416) 0.414 0.422

4	(0.584) 0.586 0.578

Locus 19611 :

2 alleles

91.0% missing data

2	(0.676) 0.634 0.719

1	(0.324) 0.366 0.281

Locus 19612 :

2 alleles

99.2% missing data

3	(0.661) 0.659 0.656

1	(0.339) 0.341 0.344

Locus 19613 :

2 alleles

90.2% missing data

4	(0.951) 0.962 0.944

3	(0.049) 0.038 0.056

Locus 19614 :

2 alleles

96.5% missing data

2	(0.106) 0.114 0.097

3	(0.894) 0.886 0.903

Locus 19615 :

2 alleles

99.2% missing data

3	(0.446) 0.450 0.445

4	(0.554) 0.550 0.555

Locus 19616 :

2 alleles

96.1% missing data

4	(0.858) 0.868 0.852

2	(0.142) 0.132 0.148

Locus 19617 :

2 alleles

99.2% missing data

1	(0.384) 0.379 0.391

2	(0.616) 0.621 0.609

Locus 19618 :

2 alleles

8.2% missing data

3	(0.985) 0.978 0.996

4	(0.015) 0.022 0.004

Locus 19619 :

2 alleles

99.2% missing data

3	(0.520) 0.522 0.520

1	(0.480) 0.478 0.480

Locus 19620 :

3 alleles

98.8% missing data

4	(0.352) 0.351 0.349

1	(0.344) 0.342 0.351

2	(0.305) 0.307 0.300

Locus 19621 :

2 alleles

99.2% missing data

3	(0.302) 0.302 0.304

2	(0.698) 0.698 0.696

Locus 19622 :

2 alleles

98.0% missing data

3	(0.898) 0.900 0.893

4	(0.102) 0.100 0.107

Locus 19623 :

2 alleles

99.2% missing data

1	(0.328) 0.334 0.329

4	(0.672) 0.666 0.671

Locus 19624 :

2 alleles

99.2% missing data

3	(0.611) 0.610 0.613

1	(0.389) 0.390 0.387

Locus 19625 :

2 alleles

99.2% missing data

2	(0.639) 0.636 0.645

3	(0.361) 0.364 0.355

Locus 19626 :

2 alleles

98.8% missing data

3	(0.534) 0.539 0.541

4	(0.466) 0.461 0.459

Locus 19627 :

2 alleles

98.4% missing data

4	(0.362) 0.371 0.365

1	(0.638) 0.629 0.635

Locus 19628 :

2 alleles

91.0% missing data

4	(0.889) 0.885 0.906

2	(0.111) 0.115 0.094

Locus 19629 :

2 alleles

97.7% missing data

4	(0.733) 0.731 0.735

2	(0.267) 0.269 0.265

Locus 19630 :

2 alleles

98.8% missing data

1	(0.531) 0.535 0.530

4	(0.469) 0.465 0.470

Locus 19631 :

2 alleles

99.2% missing data

3	(0.599) 0.595 0.607

1	(0.401) 0.405 0.393

Locus 19632 :

2 alleles

99.2% missing data

1	(0.601) 0.597 0.604

3	(0.399) 0.403 0.396

Locus 19633 :

2 alleles

99.2% missing data

2	(0.353) 0.349 0.359

1	(0.647) 0.651 0.641

Locus 19634 :

2 alleles

99.2% missing data

2	(0.448) 0.441 0.453

4	(0.552) 0.559 0.547

Locus 19635 :

2 alleles

98.8% missing data

1	(0.341) 0.336 0.340

4	(0.659) 0.664 0.660

Locus 19636 :

2 alleles

97.3% missing data

4	(0.771) 0.774 0.770

2	(0.229) 0.226 0.230

Locus 19637 :

2 alleles

99.2% missing data

4	(0.656) 0.648 0.661

1	(0.344) 0.352 0.339

Locus 19638 :

2 alleles

99.2% missing data

3	(0.778) 0.778 0.780

1	(0.222) 0.222 0.220

Locus 19639 :

2 alleles

91.0% missing data

1	(0.906) 0.923 0.896

4	(0.094) 0.077 0.104

Locus 19640 :

2 alleles

99.2% missing data

3	(0.677) 0.680 0.674

2	(0.323) 0.320 0.326

Locus 19641 :

2 alleles

99.2% missing data

2	(0.394) 0.393 0.391

3	(0.606) 0.607 0.609

Locus 19642 :

2 alleles

99.2% missing data

4	(0.132) 0.133 0.137

3	(0.868) 0.867 0.863

Locus 19643 :

2 alleles

96.5% missing data

2	(0.831) 0.834 0.835

4	(0.169) 0.166 0.165

Locus 19644 :

2 alleles

99.2% missing data

3	(0.646) 0.647 0.641

2	(0.354) 0.353 0.359

Locus 19645 :

2 alleles

99.2% missing data

4	(0.655) 0.657 0.651

2	(0.345) 0.343 0.349

Locus 19646 :

2 alleles

99.2% missing data

4	(0.772) 0.762 0.778

2	(0.228) 0.238 0.222

Locus 19647 :

2 alleles

99.2% missing data

3	(0.495) 0.496 0.496

2	(0.505) 0.504 0.504

Locus 19648 :

2 alleles

99.2% missing data

2	(0.385) 0.384 0.386

1	(0.615) 0.616 0.614

Locus 19649 :

2 alleles

96.5% missing data

3	(0.834) 0.833 0.843

2	(0.166) 0.167 0.157

Locus 19650 :

2 alleles

99.2% missing data

1	(0.585) 0.588 0.579

4	(0.415) 0.412 0.421

Locus 19651 :

2 alleles

99.2% missing data

2	(0.483) 0.489 0.483

4	(0.517) 0.511 0.517

Locus 19652 :

2 alleles

99.2% missing data

4	(0.455) 0.454 0.450

3	(0.545) 0.546 0.550

Locus 19653 :

2 alleles

99.2% missing data

4	(0.621) 0.621 0.613

2	(0.379) 0.379 0.387

Locus 19654 :

2 alleles

99.2% missing data

4	(0.496) 0.485 0.499

3	(0.504) 0.515 0.501

Locus 19655 :

2 alleles

95.7% missing data

1	(0.231) 0.217 0.234

3	(0.769) 0.783 0.766

Locus 19656 :

2 alleles

99.2% missing data

2	(0.301) 0.303 0.300

4	(0.699) 0.697 0.700

Locus 19657 :

2 alleles

99.2% missing data

1	(0.446) 0.447 0.445

2	(0.554) 0.553 0.555

Locus 19658 :

2 alleles

99.2% missing data

1	(0.316) 0.309 0.322

3	(0.684) 0.691 0.678

Locus 19659 :

2 alleles

99.2% missing data

4	(0.621) 0.615 0.627

2	(0.379) 0.385 0.373

Locus 19660 :

3 alleles

98.4% missing data

3	(0.246) 0.242 0.254

4	(0.340) 0.334 0.340

1	(0.413) 0.423 0.406

Locus 19661 :

2 alleles

99.2% missing data

4	(0.422) 0.419 0.428

3	(0.578) 0.581 0.572

Locus 19662 :

2 alleles

99.2% missing data

3	(0.574) 0.570 0.577

4	(0.426) 0.430 0.423

Locus 19663 :

2 alleles

99.2% missing data

4	(0.494) 0.492 0.488

2	(0.506) 0.508 0.512

Locus 19664 :

3 alleles

98.8% missing data

2	(0.360) 0.355 0.365

1	(0.444) 0.453 0.434

4	(0.196) 0.192 0.201

Locus 19665 :

2 alleles

98.0% missing data

3	(0.858) 0.853 0.866

1	(0.142) 0.147 0.134

Locus 19666 :

2 alleles

99.2% missing data

4	(0.568) 0.563 0.571

3	(0.432) 0.437 0.429

Locus 19667 :

2 alleles

99.2% missing data

4	(0.271) 0.278 0.266

2	(0.729) 0.722 0.734

Locus 19668 :

2 alleles

98.8% missing data

3	(0.392) 0.398 0.382

2	(0.608) 0.602 0.618

Locus 19669 :

2 alleles

99.2% missing data

2	(0.445) 0.443 0.451

1	(0.555) 0.557 0.549

Locus 19670 :

2 alleles

99.2% missing data

3	(0.331) 0.333 0.325

2	(0.669) 0.667 0.675

Locus 19671 :

2 alleles

99.2% missing data

3	(0.241) 0.241 0.244

4	(0.759) 0.759 0.756

Locus 19672 :

2 alleles

99.2% missing data

2	(0.547) 0.552 0.549

3	(0.453) 0.448 0.451

Locus 19673 :

2 alleles

98.8% missing data

4	(0.747) 0.745 0.751

3	(0.253) 0.255 0.249

Locus 19674 :

2 alleles

96.9% missing data

1	(0.757) 0.759 0.768

2	(0.243) 0.241 0.232

Locus 19675 :

2 alleles

98.8% missing data

3	(0.627) 0.626 0.635

1	(0.373) 0.374 0.365

Locus 19676 :

2 alleles

10.2% missing data

3	(0.983) 0.986 0.992

2	(0.017) 0.014 0.008

Locus 19677 :

2 alleles

96.9% missing data

2	(0.685) 0.678 0.696

3	(0.315) 0.322 0.304

Locus 19678 :

2 alleles

92.6% missing data

4	(0.861) 0.854 0.874

3	(0.139) 0.146 0.126

Locus 19679 :

2 alleles

98.4% missing data

4	(0.657) 0.654 0.658

3	(0.343) 0.346 0.342

Locus 19680 :

2 alleles

99.2% missing data

2	(0.800) 0.801 0.794

1	(0.200) 0.199 0.206

Locus 19681 :

2 alleles

98.8% missing data

4	(0.281) 0.278 0.281

1	(0.719) 0.722 0.719

Locus 19682 :

2 alleles

96.5% missing data

3	(0.804) 0.790 0.820

4	(0.196) 0.210 0.180

Locus 19683 :

3 alleles

98.8% missing data

4	(0.264) 0.262 0.266

3	(0.367) 0.364 0.375

2	(0.369) 0.374 0.359

Locus 19684 :

2 alleles

98.0% missing data

1	(0.746) 0.747 0.749

2	(0.254) 0.253 0.251

Locus 19685 :

2 alleles

98.0% missing data

4	(0.723) 0.721 0.723

2	(0.277) 0.279 0.277

Locus 19686 :

2 alleles

99.2% missing data

4	(0.281) 0.276 0.286

1	(0.719) 0.724 0.714

Locus 19687 :

2 alleles

98.0% missing data

3	(0.173) 0.176 0.172

2	(0.827) 0.824 0.828

Locus 19688 :

2 alleles

99.2% missing data

4	(0.682) 0.677 0.681

1	(0.318) 0.323 0.319

Locus 19689 :

2 alleles

99.2% missing data

2	(0.486) 0.480 0.489

1	(0.514) 0.520 0.511

Locus 19690 :

2 alleles

99.2% missing data

4	(0.511) 0.506 0.517

3	(0.489) 0.494 0.483

Locus 19691 :

2 alleles

99.2% missing data

3	(0.230) 0.227 0.232

2	(0.770) 0.773 0.768

Locus 19692 :

2 alleles

99.2% missing data

1	(0.316) 0.314 0.321

2	(0.684) 0.686 0.679

Locus 19693 :

2 alleles

99.2% missing data

1	(0.447) 0.444 0.458

2	(0.553) 0.556 0.542

Locus 19694 :

2 alleles

99.2% missing data

1	(0.618) 0.615 0.627

3	(0.382) 0.385 0.373

Locus 19695 :

2 alleles

99.2% missing data

1	(0.644) 0.638 0.647

2	(0.356) 0.362 0.353

Locus 19696 :

2 alleles

99.2% missing data

2	(0.351) 0.350 0.356

3	(0.649) 0.650 0.644

Locus 19697 :

2 alleles

98.0% missing data

1	(0.418) 0.420 0.411

2	(0.582) 0.580 0.589

Locus 19698 :

2 alleles

99.2% missing data

2	(0.374) 0.369 0.376

1	(0.626) 0.631 0.624

Locus 19699 :

2 alleles

99.2% missing data

1	(0.529) 0.528 0.526

3	(0.471) 0.472 0.474

Locus 19700 :

2 alleles

99.2% missing data

3	(0.696) 0.691 0.694

4	(0.304) 0.309 0.306

Locus 19701 :

2 alleles

99.2% missing data

1	(0.691) 0.686 0.696

2	(0.309) 0.314 0.304

Locus 19702 :

2 alleles

98.8% missing data

1	(0.372) 0.370 0.375

4	(0.628) 0.630 0.625

Locus 19703 :

2 alleles

99.2% missing data

4	(0.650) 0.649 0.648

2	(0.350) 0.351 0.352

Locus 19704 :

2 alleles

98.0% missing data

2	(0.643) 0.636 0.664

4	(0.357) 0.364 0.336

Locus 19705 :

2 alleles

83.6% missing data

4	(0.518) 0.574 0.456

3	(0.482) 0.426 0.544

Locus 19706 :

2 alleles

99.2% missing data

1	(0.492) 0.484 0.499

2	(0.508) 0.516 0.501

Locus 19707 :

2 alleles

99.2% missing data

3	(0.836) 0.832 0.841

1	(0.164) 0.168 0.159

Locus 19708 :

2 alleles

99.2% missing data

1	(0.269) 0.267 0.274

3	(0.731) 0.733 0.726

Locus 19709 :

2 alleles

96.1% missing data

1	(0.884) 0.890 0.878

2	(0.116) 0.110 0.122

Locus 19710 :

2 alleles

98.8% missing data

3	(0.626) 0.620 0.634

1	(0.374) 0.380 0.366

Locus 19711 :

2 alleles

98.8% missing data

1	(0.530) 0.528 0.532

3	(0.470) 0.472 0.468

Locus 19712 :

2 alleles

74.2% missing data

2	(0.041) 0.046 0.026

1	(0.959) 0.954 0.974

Locus 19713 :

2 alleles

98.8% missing data

4	(0.758) 0.756 0.757

1	(0.242) 0.244 0.243

Locus 19714 :

2 alleles

99.2% missing data

3	(0.574) 0.577 0.568

4	(0.426) 0.423 0.432

Locus 19715 :

2 alleles

99.2% missing data

4	(0.680) 0.677 0.685

1	(0.320) 0.323 0.315

Locus 19716 :

2 alleles

99.2% missing data

2	(0.299) 0.301 0.298

3	(0.701) 0.699 0.702

Locus 19717 :

2 alleles

99.2% missing data

2	(0.607) 0.604 0.607

1	(0.393) 0.396 0.393

Locus 19718 :

2 alleles

99.2% missing data

4	(0.335) 0.335 0.337

3	(0.665) 0.665 0.663

Locus 19719 :

2 alleles

99.2% missing data

1	(0.476) 0.478 0.478

2	(0.524) 0.522 0.522

Locus 19720 :

2 alleles

99.2% missing data

2	(0.279) 0.279 0.282

3	(0.721) 0.721 0.718

Locus 19721 :

2 alleles

99.2% missing data

4	(0.683) 0.678 0.690

1	(0.317) 0.322 0.310

Locus 19722 :

2 alleles

98.8% missing data

1	(0.665) 0.671 0.663

2	(0.335) 0.329 0.337

Locus 19723 :

2 alleles

99.2% missing data

2	(0.378) 0.375 0.386

4	(0.622) 0.625 0.614

Locus 19724 :

2 alleles

85.2% missing data

2	(0.959) 0.958 0.967

4	(0.041) 0.042 0.033

Locus 19725 :

2 alleles

99.2% missing data

1	(0.663) 0.661 0.661

3	(0.337) 0.339 0.339

Locus 19726 :

2 alleles

99.2% missing data

2	(0.680) 0.671 0.687

1	(0.320) 0.329 0.313

Locus 19727 :

2 alleles

0.0% missing data

2	(0.981) 0.964 0.995

3	(0.019) 0.036 0.005

Locus 19728 :

2 alleles

99.2% missing data

4	(0.360) 0.354 0.362

1	(0.640) 0.646 0.638

Locus 19729 :

2 alleles

93.4% missing data

3	(0.126) 0.127 0.116

1	(0.874) 0.873 0.884

Locus 19730 :

2 alleles

91.4% missing data

3	(0.923) 0.922 0.936

2	(0.077) 0.078 0.064

Locus 19731 :

2 alleles

99.2% missing data

3	(0.535) 0.540 0.538

2	(0.465) 0.460 0.462

Locus 19732 :

2 alleles

98.8% missing data

2	(0.645) 0.634 0.653

3	(0.355) 0.366 0.347

Locus 19733 :

2 alleles

99.2% missing data

3	(0.445) 0.442 0.452

2	(0.555) 0.558 0.548

Locus 19734 :

2 alleles

99.2% missing data

3	(0.751) 0.742 0.754

2	(0.249) 0.258 0.246

Locus 19735 :

2 alleles

99.2% missing data

1	(0.635) 0.636 0.631

2	(0.365) 0.364 0.369

Locus 19736 :

2 alleles

99.2% missing data

1	(0.490) 0.488 0.495

2	(0.510) 0.512 0.505

Locus 19737 :

2 alleles

99.2% missing data

1	(0.697) 0.693 0.695

3	(0.303) 0.307 0.305

Locus 19738 :

2 alleles

99.2% missing data

1	(0.257) 0.261 0.256

3	(0.743) 0.739 0.744

Locus 19739 :

2 alleles

99.2% missing data

2	(0.384) 0.378 0.387

3	(0.616) 0.622 0.613

Locus 19740 :

2 alleles

99.2% missing data

4	(0.634) 0.636 0.634

1	(0.366) 0.364 0.366

Locus 19741 :

2 alleles

99.2% missing data

3	(0.738) 0.741 0.732

2	(0.262) 0.259 0.268

Locus 19742 :

2 alleles

98.8% missing data

1	(0.438) 0.435 0.444

2	(0.562) 0.565 0.556

Locus 19743 :

2 alleles

99.2% missing data

2	(0.686) 0.688 0.683

1	(0.314) 0.312 0.317

Locus 19744 :

2 alleles

97.3% missing data

1	(0.336) 0.336 0.327

4	(0.664) 0.664 0.673

Locus 19745 :

2 alleles

99.2% missing data

2	(0.548) 0.541 0.556

3	(0.452) 0.459 0.444

Locus 19746 :

2 alleles

99.2% missing data

4	(0.353) 0.350 0.357

3	(0.647) 0.650 0.643

Locus 19747 :

2 alleles

99.2% missing data

1	(0.527) 0.518 0.530

2	(0.473) 0.482 0.470

Locus 19748 :

2 alleles

99.2% missing data

3	(0.412) 0.409 0.418

2	(0.588) 0.591 0.582

Locus 19749 :

2 alleles

99.2% missing data

4	(0.767) 0.770 0.758

2	(0.233) 0.230 0.242

Locus 19750 :

2 alleles

99.2% missing data

4	(0.501) 0.503 0.497

3	(0.499) 0.497 0.503

Locus 19751 :

2 alleles

8.6% missing data

4	(0.987) 0.981 0.996

2	(0.013) 0.019 0.004

Locus 19752 :

2 alleles

98.0% missing data

2	(0.583) 0.577 0.596

4	(0.417) 0.423 0.404

Locus 19753 :

2 alleles

99.2% missing data

3	(0.208) 0.202 0.216

4	(0.792) 0.798 0.784

Locus 19754 :

2 alleles

98.8% missing data

3	(0.733) 0.740 0.734

4	(0.267) 0.260 0.266

Locus 19755 :

3 alleles

88.3% missing data

1	(0.715) 0.737 0.697

3	(0.221) 0.212 0.231

4	(0.064) 0.051 0.072

Locus 19756 :

3 alleles

98.8% missing data

1	(0.486) 0.485 0.488

2	(0.229) 0.224 0.234

4	(0.285) 0.290 0.278

Locus 19757 :

2 alleles

98.8% missing data

3	(0.561) 0.561 0.558

2	(0.439) 0.439 0.442

Locus 19758 :

2 alleles

2.3% missing data

2	(0.925) 0.807 0.977

4	(0.075) 0.193 0.023

Locus 19759 :

2 alleles

98.8% missing data

2	(0.782) 0.791 0.775

4	(0.218) 0.209 0.225

Locus 19760 :

2 alleles

99.2% missing data

1	(0.291) 0.283 0.297

2	(0.709) 0.717 0.703

Locus 19761 :

2 alleles

99.2% missing data

4	(0.349) 0.347 0.355

3	(0.651) 0.653 0.645

Locus 19762 :

2 alleles

99.2% missing data

2	(0.568) 0.563 0.574

3	(0.432) 0.437 0.426

Locus 19763 :

2 alleles

99.2% missing data

1	(0.596) 0.599 0.591

4	(0.404) 0.401 0.409

Locus 19764 :

2 alleles

93.0% missing data

2	(0.931) 0.933 0.933

4	(0.069) 0.067 0.067

Locus 19765 :

2 alleles

99.2% missing data

4	(0.524) 0.524 0.519

3	(0.476) 0.476 0.481

Locus 19766 :

2 alleles

99.2% missing data

4	(0.494) 0.500 0.489

2	(0.506) 0.500 0.511

Locus 19767 :

2 alleles

99.2% missing data

4	(0.766) 0.766 0.767

2	(0.234) 0.234 0.233

Locus 19768 :

2 alleles

99.2% missing data

1	(0.259) 0.254 0.264

2	(0.741) 0.746 0.736

Locus 19769 :

2 alleles

98.4% missing data

4	(0.307) 0.304 0.302

2	(0.693) 0.696 0.698

Locus 19770 :

2 alleles

98.4% missing data

2	(0.830) 0.833 0.825

4	(0.170) 0.167 0.175

Locus 19771 :

2 alleles

98.8% missing data

4	(0.543) 0.534 0.547

2	(0.457) 0.466 0.453

Locus 19772 :

2 alleles

99.2% missing data

2	(0.370) 0.371 0.368

4	(0.630) 0.629 0.632

Locus 19773 :

2 alleles

99.2% missing data

4	(0.538) 0.537 0.543

2	(0.462) 0.463 0.457

Locus 19774 :

2 alleles

98.4% missing data

4	(0.778) 0.788 0.769

1	(0.222) 0.212 0.231

Locus 19775 :

2 alleles

99.2% missing data

4	(0.652) 0.645 0.657

3	(0.348) 0.355 0.343

Locus 19776 :

2 alleles

98.4% missing data

2	(0.757) 0.757 0.758

4	(0.243) 0.243 0.242

Locus 19777 :

2 alleles

99.2% missing data

2	(0.809) 0.812 0.805

4	(0.191) 0.188 0.195

Locus 19778 :

3 alleles

98.0% missing data

2	(0.536) 0.538 0.539

3	(0.267) 0.267 0.262

4	(0.196) 0.194 0.199

Locus 19779 :

2 alleles

99.2% missing data

3	(0.467) 0.464 0.478

2	(0.533) 0.536 0.522

Locus 19780 :

2 alleles

97.7% missing data

1	(0.787) 0.794 0.787

3	(0.213) 0.206 0.213

Locus 19781 :

2 alleles

99.2% missing data

3	(0.425) 0.426 0.430

1	(0.575) 0.574 0.570

Locus 19782 :

2 alleles

11.7% missing data

2	(0.975) 0.948 0.992

1	(0.025) 0.052 0.008

Locus 19783 :

2 alleles

98.0% missing data

2	(0.197) 0.209 0.187

1	(0.803) 0.791 0.813

Locus 19784 :

2 alleles

2.3% missing data

2	(0.987) 0.991 0.992

3	(0.013) 0.009 0.008

Locus 19785 :

2 alleles

99.2% missing data

2	(0.712) 0.705 0.715

1	(0.288) 0.295 0.285

Locus 19786 :

2 alleles

98.8% missing data

2	(0.486) 0.478 0.495

4	(0.514) 0.522 0.505

Locus 19787 :

2 alleles

99.2% missing data

3	(0.519) 0.520 0.516

1	(0.481) 0.480 0.484

Locus 19788 :

3 alleles

98.8% missing data

4	(0.243) 0.244 0.244

2	(0.251) 0.250 0.256

3	(0.505) 0.506 0.499

Locus 19789 :

2 alleles

99.2% missing data

1	(0.764) 0.762 0.773

4	(0.236) 0.238 0.227

Locus 19790 :

2 alleles

99.2% missing data

2	(0.755) 0.757 0.752

4	(0.245) 0.243 0.248

Locus 19791 :

2 alleles

98.8% missing data

4	(0.539) 0.535 0.547

3	(0.461) 0.465 0.453

Locus 19792 :

2 alleles

99.2% missing data

4	(0.241) 0.238 0.241

1	(0.759) 0.762 0.759

Locus 19793 :

2 alleles

97.3% missing data

3	(0.707) 0.709 0.719

2	(0.293) 0.291 0.281

Locus 19794 :

2 alleles

99.2% missing data

4	(0.638) 0.639 0.633

2	(0.362) 0.361 0.367

Locus 19795 :

2 alleles

99.2% missing data

2	(0.743) 0.735 0.744

1	(0.257) 0.265 0.256

Locus 19796 :

2 alleles

99.2% missing data

3	(0.724) 0.716 0.728

4	(0.276) 0.284 0.272

Locus 19797 :

2 alleles

99.2% missing data

4	(0.646) 0.650 0.643

3	(0.354) 0.350 0.357

Locus 19798 :

2 alleles

99.2% missing data

3	(0.507) 0.505 0.507

2	(0.493) 0.495 0.493

Locus 19799 :

2 alleles

99.2% missing data

2	(0.443) 0.440 0.445

4	(0.557) 0.560 0.555

Locus 19800 :

2 alleles

99.2% missing data

2	(0.626) 0.627 0.627

1	(0.374) 0.373 0.373

Locus 19801 :

2 alleles

99.2% missing data

1	(0.646) 0.640 0.653

3	(0.354) 0.360 0.347

Locus 19802 :

2 alleles

99.2% missing data

4	(0.383) 0.383 0.388

2	(0.617) 0.617 0.612

Locus 19803 :

2 alleles

94.9% missing data

2	(0.888) 0.881 0.897

3	(0.112) 0.119 0.103

Locus 19804 :

2 alleles

91.4% missing data

3	(0.930) 0.927 0.941

2	(0.070) 0.073 0.059

Locus 19805 :

2 alleles

98.8% missing data

1	(0.499) 0.502 0.501

3	(0.501) 0.498 0.499

Locus 19806 :

2 alleles

99.2% missing data

2	(0.533) 0.535 0.530

3	(0.467) 0.465 0.470

Locus 19807 :

2 alleles

99.2% missing data

4	(0.426) 0.425 0.421

1	(0.574) 0.575 0.579

Locus 19808 :

2 alleles

99.2% missing data

4	(0.549) 0.549 0.545

2	(0.451) 0.451 0.455

Locus 19809 :

2 alleles

98.8% missing data

2	(0.754) 0.747 0.765

4	(0.246) 0.253 0.235

Locus 19810 :

2 alleles

99.2% missing data

3	(0.418) 0.414 0.418

1	(0.582) 0.586 0.582

Locus 19811 :

2 alleles

91.0% missing data

3	(0.914) 0.929 0.907

4	(0.086) 0.071 0.093

Locus 19812 :

2 alleles

98.8% missing data

4	(0.239) 0.236 0.238

2	(0.761) 0.764 0.762

Locus 19813 :

2 alleles

99.2% missing data

1	(0.233) 0.239 0.225

4	(0.767) 0.761 0.775

Locus 19814 :

2 alleles

99.2% missing data

4	(0.402) 0.395 0.407

3	(0.598) 0.605 0.593

Locus 19815 :

2 alleles

99.2% missing data

1	(0.530) 0.536 0.524

3	(0.470) 0.464 0.476

Locus 19816 :

2 alleles

99.2% missing data

1	(0.238) 0.234 0.248

3	(0.762) 0.766 0.752

Locus 19817 :

2 alleles

99.2% missing data

3	(0.519) 0.514 0.523

2	(0.481) 0.486 0.477

Locus 19818 :

2 alleles

99.2% missing data

3	(0.728) 0.719 0.731

1	(0.272) 0.281 0.269

Locus 19819 :

2 alleles

99.2% missing data

2	(0.740) 0.732 0.743

4	(0.260) 0.268 0.257

Locus 19820 :

2 alleles

90.6% missing data

2	(0.937) 0.950 0.927

1	(0.063) 0.050 0.073

Locus 19821 :

2 alleles

99.2% missing data

2	(0.576) 0.571 0.571

3	(0.424) 0.429 0.429

Locus 19822 :

2 alleles

98.8% missing data

2	(0.393) 0.388 0.405

4	(0.607) 0.612 0.595

Locus 19823 :

2 alleles

99.2% missing data

1	(0.481) 0.481 0.479

4	(0.519) 0.519 0.521

Locus 19824 :

2 alleles

99.2% missing data

1	(0.152) 0.161 0.144

4	(0.848) 0.839 0.856

Locus 19825 :

2 alleles

98.0% missing data

2	(0.791) 0.793 0.794

1	(0.209) 0.207 0.206

Locus 19826 :

2 alleles

99.2% missing data

2	(0.272) 0.274 0.276

4	(0.728) 0.726 0.724

Locus 19827 :

2 alleles

90.6% missing data

3	(0.917) 0.926 0.919

1	(0.083) 0.074 0.081

Locus 19828 :

2 alleles

0.0% missing data

4	(0.987) 0.990 0.993

3	(0.013) 0.010 0.007

Locus 19829 :

2 alleles

99.2% missing data

2	(0.392) 0.399 0.392

4	(0.608) 0.601 0.608

Locus 19830 :

2 alleles

98.8% missing data

2	(0.334) 0.341 0.328

4	(0.666) 0.659 0.672

Locus 19831 :

2 alleles

99.2% missing data

2	(0.442) 0.438 0.442

4	(0.558) 0.562 0.558

Locus 19832 :

2 alleles

99.2% missing data

2	(0.592) 0.599 0.593

4	(0.408) 0.401 0.407

Locus 19833 :

2 alleles

0.0% missing data

2	(0.905) 0.749 0.972

3	(0.095) 0.251 0.028

Locus 19834 :

2 alleles

99.2% missing data

2	(0.543) 0.539 0.544

3	(0.457) 0.461 0.456

Locus 19835 :

2 alleles

99.2% missing data

4	(0.589) 0.586 0.595

2	(0.411) 0.414 0.405

Locus 19836 :

2 alleles

98.0% missing data

4	(0.729) 0.727 0.735

1	(0.271) 0.273 0.265

Locus 19837 :

2 alleles

99.2% missing data

1	(0.566) 0.562 0.569

3	(0.434) 0.438 0.431

Locus 19838 :

2 alleles

97.7% missing data

3	(0.358) 0.359 0.341

4	(0.642) 0.641 0.659

Locus 19839 :

2 alleles

99.2% missing data

4	(0.721) 0.719 0.720

2	(0.279) 0.281 0.280

Locus 19840 :

2 alleles

99.2% missing data

2	(0.575) 0.573 0.574

4	(0.425) 0.427 0.426

Locus 19841 :

2 alleles

99.2% missing data

4	(0.597) 0.596 0.595

1	(0.403) 0.404 0.405

Locus 19842 :

2 alleles

99.2% missing data

2	(0.716) 0.716 0.712

3	(0.284) 0.284 0.288

Locus 19843 :

2 alleles

97.7% missing data

1	(0.749) 0.744 0.760

2	(0.251) 0.256 0.240

Locus 19844 :

2 alleles

99.2% missing data

1	(0.602) 0.600 0.605

2	(0.398) 0.400 0.395

Locus 19845 :

2 alleles

98.8% missing data

4	(0.264) 0.262 0.266

1	(0.736) 0.738 0.734

Locus 19846 :

2 alleles

98.8% missing data

2	(0.413) 0.409 0.424

1	(0.587) 0.591 0.576

Locus 19847 :

2 alleles

98.8% missing data

1	(0.441) 0.441 0.449

2	(0.559) 0.559 0.551

Locus 19848 :

2 alleles

99.2% missing data

2	(0.417) 0.419 0.421

1	(0.583) 0.581 0.579

Locus 19849 :

2 alleles

99.2% missing data

3	(0.520) 0.518 0.521

2	(0.480) 0.482 0.479

Locus 19850 :

2 alleles

96.1% missing data

3	(0.564) 0.580 0.541

4	(0.436) 0.420 0.459

Locus 19851 :

2 alleles

99.2% missing data

4	(0.767) 0.761 0.771

1	(0.233) 0.239 0.229

Locus 19852 :

2 alleles

98.8% missing data

3	(0.475) 0.478 0.470

1	(0.525) 0.522 0.530

Locus 19853 :

2 alleles

99.2% missing data

2	(0.115) 0.111 0.119

1	(0.885) 0.889 0.881

Locus 19854 :

2 alleles

99.2% missing data

4	(0.635) 0.638 0.632

2	(0.365) 0.362 0.368

Locus 19855 :

3 alleles

98.8% missing data

4	(0.334) 0.337 0.334

2	(0.376) 0.376 0.375

1	(0.289) 0.287 0.290

Locus 19856 :

2 alleles

99.2% missing data

1	(0.186) 0.190 0.184

4	(0.814) 0.810 0.816

Locus 19857 :

2 alleles

91.0% missing data

1	(0.920) 0.933 0.911

3	(0.080) 0.067 0.089

Locus 19858 :

2 alleles

99.2% missing data

3	(0.739) 0.730 0.744

1	(0.261) 0.270 0.256

Locus 19859 :

2 alleles

99.2% missing data

1	(0.653) 0.655 0.651

4	(0.347) 0.345 0.349

Locus 19860 :

2 alleles

98.0% missing data

2	(0.638) 0.649 0.634

1	(0.362) 0.351 0.366

Locus 19861 :

2 alleles

99.2% missing data

2	(0.502) 0.505 0.503

4	(0.498) 0.495 0.497

Locus 19862 :

2 alleles

99.2% missing data

4	(0.576) 0.571 0.580

2	(0.424) 0.429 0.420

Locus 19863 :

2 alleles

99.2% missing data

1	(0.636) 0.632 0.642

3	(0.364) 0.368 0.358

Locus 19864 :

2 alleles

99.2% missing data

3	(0.362) 0.361 0.366

4	(0.638) 0.639 0.634

Locus 19865 :

2 alleles

99.2% missing data

3	(0.490) 0.489 0.489

2	(0.510) 0.511 0.511

Locus 19866 :

2 alleles

99.2% missing data

3	(0.465) 0.467 0.464

2	(0.535) 0.533 0.536

Locus 19867 :

2 alleles

99.2% missing data

1	(0.472) 0.467 0.476

3	(0.528) 0.533 0.524

Locus 19868 :

2 alleles

99.2% missing data

2	(0.506) 0.500 0.513

3	(0.494) 0.500 0.487

Locus 19869 :

2 alleles

99.2% missing data

1	(0.412) 0.403 0.415

3	(0.588) 0.597 0.585

Locus 19870 :

2 alleles

99.2% missing data

1	(0.535) 0.537 0.527

2	(0.465) 0.463 0.473

Locus 19871 :

2 alleles

98.4% missing data

1	(0.787) 0.794 0.775

3	(0.213) 0.206 0.225

Locus 19872 :

2 alleles

99.2% missing data

4	(0.433) 0.430 0.433

2	(0.567) 0.570 0.567

Locus 19873 :

2 alleles

99.2% missing data

2	(0.664) 0.657 0.664

3	(0.336) 0.343 0.336

Locus 19874 :

3 alleles

98.4% missing data

2	(0.278) 0.273 0.283

3	(0.185) 0.185 0.193

1	(0.536) 0.542 0.524

Locus 19875 :

2 alleles

1.6% missing data

4	(0.987) 0.990 0.993

3	(0.013) 0.010 0.007

Locus 19876 :

2 alleles

95.3% missing data

1	(0.100) 0.104 0.089

3	(0.900) 0.896 0.911

Locus 19877 :

2 alleles

99.2% missing data

2	(0.498) 0.497 0.501

4	(0.502) 0.503 0.499

Locus 19878 :

2 alleles

99.2% missing data

3	(0.538) 0.546 0.537

4	(0.462) 0.454 0.463

Locus 19879 :

2 alleles

99.2% missing data

1	(0.666) 0.662 0.669

4	(0.334) 0.338 0.331

Locus 19880 :

2 alleles

99.2% missing data

3	(0.264) 0.259 0.269

2	(0.736) 0.741 0.731

Locus 19881 :

2 alleles

90.2% missing data

3	(0.942) 0.951 0.934

1	(0.058) 0.049 0.066

Locus 19882 :

2 alleles

94.5% missing data

2	(0.890) 0.888 0.895

1	(0.110) 0.112 0.105

Locus 19883 :

2 alleles

99.2% missing data

1	(0.310) 0.312 0.313

3	(0.690) 0.688 0.687

Locus 19884 :

3 alleles

89.1% missing data

1	(0.227) 0.197 0.252

4	(0.671) 0.721 0.632

3	(0.102) 0.082 0.116

Locus 19885 :

2 alleles

99.2% missing data

1	(0.772) 0.774 0.771

2	(0.228) 0.226 0.229

Locus 19886 :

2 alleles

99.2% missing data

2	(0.249) 0.256 0.245

4	(0.751) 0.744 0.755

Locus 19887 :

2 alleles

91.0% missing data

2	(0.854) 0.877 0.844

3	(0.146) 0.123 0.156

Locus 19888 :

2 alleles

99.2% missing data

3	(0.412) 0.405 0.416

1	(0.588) 0.595 0.584

Locus 19889 :

2 alleles

99.2% missing data

3	(0.212) 0.221 0.207

1	(0.788) 0.779 0.793

Locus 19890 :

2 alleles

95.7% missing data

1	(0.840) 0.835 0.847

3	(0.160) 0.165 0.153

Locus 19891 :

2 alleles

98.8% missing data

3	(0.360) 0.358 0.363

1	(0.640) 0.642 0.637

Locus 19892 :

2 alleles

99.2% missing data

4	(0.512) 0.514 0.513

3	(0.488) 0.486 0.487

Locus 19893 :

2 alleles

99.2% missing data

2	(0.812) 0.817 0.804

1	(0.188) 0.183 0.196

Locus 19894 :

2 alleles

99.2% missing data

1	(0.593) 0.598 0.593

3	(0.407) 0.402 0.407

Locus 19895 :

2 alleles

93.4% missing data

2	(0.842) 0.843 0.857

4	(0.158) 0.157 0.143

Locus 19896 :

2 alleles

99.2% missing data

2	(0.443) 0.440 0.453

4	(0.557) 0.560 0.547

Locus 19897 :

3 alleles

98.8% missing data

3	(0.331) 0.329 0.334

2	(0.452) 0.447 0.458

1	(0.218) 0.224 0.208

Locus 19898 :

2 alleles

99.2% missing data

3	(0.316) 0.317 0.322

1	(0.684) 0.683 0.678

Locus 19899 :

2 alleles

96.9% missing data

3	(0.431) 0.408 0.446

1	(0.569) 0.592 0.554

Locus 19900 :

2 alleles

99.2% missing data

4	(0.583) 0.576 0.594

1	(0.417) 0.424 0.406

Locus 19901 :

2 alleles

99.2% missing data

4	(0.701) 0.706 0.697

1	(0.299) 0.294 0.303

Locus 19902 :

2 alleles

99.2% missing data

2	(0.597) 0.596 0.597

1	(0.403) 0.404 0.403

Locus 19903 :

2 alleles

99.2% missing data

4	(0.488) 0.491 0.485

1	(0.512) 0.509 0.515

Locus 19904 :

2 alleles

99.2% missing data

2	(0.496) 0.493 0.500

4	(0.504) 0.507 0.500

Locus 19905 :

2 alleles

99.2% missing data

3	(0.341) 0.338 0.345

4	(0.659) 0.662 0.655

Locus 19906 :

2 alleles

99.2% missing data

3	(0.437) 0.439 0.437

2	(0.563) 0.561 0.563

Locus 19907 :

2 alleles

98.0% missing data

3	(0.871) 0.881 0.865

2	(0.129) 0.119 0.135

Locus 19908 :

2 alleles

99.2% missing data

1	(0.356) 0.351 0.363

2	(0.644) 0.649 0.637

Locus 19909 :

2 alleles

99.2% missing data

3	(0.412) 0.409 0.411

4	(0.588) 0.591 0.589

Locus 19910 :

2 alleles

99.2% missing data

4	(0.586) 0.582 0.598

3	(0.414) 0.418 0.402

Locus 19911 :

2 alleles

99.2% missing data

2	(0.312) 0.306 0.314

1	(0.688) 0.694 0.686

Locus 19912 :

2 alleles

99.2% missing data

1	(0.720) 0.717 0.724

3	(0.280) 0.283 0.276

Locus 19913 :

2 alleles

98.0% missing data

2	(0.743) 0.746 0.747

3	(0.257) 0.254 0.253

Locus 19914 :

2 alleles

0.0% missing data

2	(0.985) 0.989 0.992

3	(0.015) 0.011 0.008

Locus 19915 :

2 alleles

99.2% missing data

3	(0.407) 0.405 0.410

1	(0.593) 0.595 0.590

Locus 19916 :

2 alleles

99.2% missing data

4	(0.690) 0.684 0.695

3	(0.310) 0.316 0.305

Locus 19917 :

2 alleles

99.2% missing data

4	(0.276) 0.274 0.282

1	(0.724) 0.726 0.718

Locus 19918 :

2 alleles

99.2% missing data

1	(0.449) 0.445 0.451

4	(0.551) 0.555 0.549

Locus 19919 :

3 alleles

96.5% missing data

3	(0.649) 0.668 0.642

1	(0.256) 0.242 0.258

4	(0.095) 0.089 0.100

Locus 19920 :

2 alleles

99.2% missing data

3	(0.658) 0.656 0.662

1	(0.342) 0.344 0.338

Locus 19921 :

2 alleles

96.5% missing data

3	(0.316) 0.326 0.297

1	(0.684) 0.674 0.703

Locus 19922 :

2 alleles

99.2% missing data

4	(0.404) 0.397 0.411

3	(0.596) 0.603 0.589

Locus 19923 :

2 alleles

99.2% missing data

1	(0.321) 0.321 0.324

2	(0.679) 0.679 0.676

Locus 19924 :

2 alleles

99.2% missing data

4	(0.501) 0.500 0.500

1	(0.499) 0.500 0.500

Locus 19925 :

2 alleles

99.2% missing data

4	(0.585) 0.580 0.590

3	(0.415) 0.420 0.410

Locus 19926 :

2 alleles

99.2% missing data

2	(0.594) 0.585 0.598

3	(0.406) 0.415 0.402

Locus 19927 :

2 alleles

99.2% missing data

3	(0.150) 0.146 0.153

2	(0.850) 0.854 0.847

Locus 19928 :

2 alleles

98.0% missing data

3	(0.254) 0.243 0.253

1	(0.746) 0.757 0.747

Locus 19929 :

2 alleles

99.2% missing data

4	(0.224) 0.233 0.221

1	(0.776) 0.767 0.779

Locus 19930 :

2 alleles

99.2% missing data

2	(0.453) 0.449 0.462

3	(0.547) 0.551 0.538

Locus 19931 :

2 alleles

99.2% missing data

3	(0.624) 0.620 0.633

1	(0.376) 0.380 0.367

Locus 19932 :

2 alleles

99.2% missing data

1	(0.325) 0.326 0.327

2	(0.675) 0.674 0.673

Locus 19933 :

2 alleles

99.2% missing data

1	(0.637) 0.639 0.628

4	(0.363) 0.361 0.372

Locus 19934 :

2 alleles

99.2% missing data

2	(0.338) 0.346 0.339

4	(0.662) 0.654 0.661

Locus 19935 :

2 alleles

90.6% missing data

1	(0.887) 0.911 0.869

4	(0.113) 0.089 0.131

Locus 19936 :

2 alleles

99.2% missing data

1	(0.538) 0.543 0.534

3	(0.462) 0.457 0.466

Locus 19937 :

2 alleles

99.2% missing data

4	(0.308) 0.309 0.310

1	(0.692) 0.691 0.690

Locus 19938 :

2 alleles

99.2% missing data

1	(0.385) 0.390 0.378

2	(0.615) 0.610 0.622

Locus 19939 :

2 alleles

99.2% missing data

1	(0.726) 0.724 0.720

2	(0.274) 0.276 0.280

Locus 19940 :

2 alleles

99.2% missing data

3	(0.639) 0.638 0.639

2	(0.361) 0.362 0.361

Locus 19941 :

2 alleles

99.2% missing data

1	(0.573) 0.568 0.570

4	(0.427) 0.432 0.430

Locus 19942 :

2 alleles

99.2% missing data

2	(0.418) 0.423 0.415

3	(0.582) 0.577 0.585

Locus 19943 :

2 alleles

99.2% missing data

1	(0.241) 0.248 0.238

3	(0.759) 0.752 0.762

Locus 19944 :

2 alleles

0.0% missing data

2	(0.984) 0.979 0.995

3	(0.016) 0.021 0.005

Locus 19945 :

2 alleles

99.2% missing data

1	(0.238) 0.237 0.245

3	(0.762) 0.763 0.755

Locus 19946 :

2 alleles

99.2% missing data

4	(0.864) 0.855 0.867

2	(0.136) 0.145 0.133

Locus 19947 :

2 alleles

98.4% missing data

2	(0.820) 0.825 0.817

1	(0.180) 0.175 0.183

Locus 19948 :

2 alleles

99.2% missing data

4	(0.443) 0.437 0.440

1	(0.557) 0.563 0.560

Locus 19949 :

2 alleles

92.6% missing data

2	(0.138) 0.145 0.126

1	(0.862) 0.855 0.874

Locus 19950 :

2 alleles

99.2% missing data

3	(0.507) 0.507 0.498

2	(0.493) 0.493 0.502

Locus 19951 :

2 alleles

98.8% missing data

2	(0.503) 0.497 0.511

3	(0.497) 0.503 0.489

Locus 19952 :

2 alleles

91.8% missing data

3	(0.742) 0.710 0.781

1	(0.258) 0.290 0.219

Locus 19953 :

2 alleles

98.8% missing data

3	(0.243) 0.248 0.246

4	(0.757) 0.752 0.754

Locus 19954 :

2 alleles

96.5% missing data

2	(0.799) 0.794 0.813

4	(0.201) 0.206 0.187

Locus 19955 :

2 alleles

99.2% missing data

1	(0.432) 0.426 0.436

3	(0.568) 0.574 0.564

Locus 19956 :

2 alleles

93.0% missing data

3	(0.929) 0.927 0.933

4	(0.071) 0.073 0.067

Locus 19957 :

2 alleles

99.2% missing data

1	(0.465) 0.466 0.463

3	(0.535) 0.534 0.537

Locus 19958 :

2 alleles

98.8% missing data

4	(0.794) 0.789 0.798

3	(0.206) 0.211 0.202

Locus 19959 :

2 alleles

99.2% missing data

3	(0.499) 0.493 0.509

2	(0.501) 0.507 0.491

Locus 19960 :

2 alleles

99.2% missing data

1	(0.663) 0.659 0.664

3	(0.337) 0.341 0.336

Locus 19961 :

2 alleles

75.4% missing data

1	(0.961) 0.953 0.976

4	(0.039) 0.047 0.024

Locus 19962 :

2 alleles

98.8% missing data

1	(0.346) 0.339 0.352

3	(0.654) 0.661 0.648

Locus 19963 :

2 alleles

99.2% missing data

1	(0.599) 0.597 0.593

2	(0.401) 0.403 0.407

Locus 19964 :

2 alleles

98.8% missing data

1	(0.628) 0.638 0.625

4	(0.372) 0.362 0.375

Locus 19965 :

2 alleles

99.2% missing data

1	(0.312) 0.316 0.308

2	(0.688) 0.684 0.692

Locus 19966 :

2 alleles

99.2% missing data

2	(0.712) 0.715 0.706

4	(0.288) 0.285 0.294

Locus 19967 :

2 alleles

99.2% missing data

3	(0.444) 0.449 0.438

1	(0.556) 0.551 0.562

Locus 19968 :

2 alleles

99.2% missing data

4	(0.584) 0.587 0.581

1	(0.416) 0.413 0.419

Locus 19969 :

2 alleles

99.2% missing data

3	(0.362) 0.366 0.360

2	(0.638) 0.634 0.640

Locus 19970 :

2 alleles

98.8% missing data

1	(0.232) 0.234 0.232

4	(0.768) 0.766 0.768

Locus 19971 :

2 alleles

99.2% missing data

4	(0.534) 0.536 0.533

2	(0.466) 0.464 0.467

Locus 19972 :

2 alleles

99.2% missing data

4	(0.633) 0.633 0.642

1	(0.367) 0.367 0.358

Locus 19973 :

2 alleles

0.4% missing data

2	(0.983) 0.979 0.995

4	(0.017) 0.021 0.005

Locus 19974 :

2 alleles

98.8% missing data

3	(0.477) 0.490 0.478

4	(0.523) 0.510 0.522

Locus 19975 :

2 alleles

99.2% missing data

3	(0.500) 0.500 0.494

1	(0.500) 0.500 0.506

Locus 19976 :

2 alleles

96.1% missing data

1	(0.888) 0.891 0.891

3	(0.112) 0.109 0.109

Locus 19977 :

2 alleles

98.8% missing data

3	(0.479) 0.478 0.485

2	(0.521) 0.522 0.515

Locus 19978 :

2 alleles

98.8% missing data

3	(0.303) 0.306 0.302

2	(0.697) 0.694 0.698

Locus 19979 :

2 alleles

99.2% missing data

4	(0.469) 0.471 0.468

3	(0.531) 0.529 0.532

Locus 19980 :

2 alleles

99.2% missing data

1	(0.155) 0.153 0.161

2	(0.845) 0.847 0.839

Locus 19981 :

2 alleles

99.2% missing data

4	(0.653) 0.655 0.642

3	(0.347) 0.345 0.358

Locus 19982 :

2 alleles

99.2% missing data

2	(0.591) 0.586 0.592

4	(0.409) 0.414 0.408

Locus 19983 :

2 alleles

99.2% missing data

1	(0.285) 0.280 0.284

2	(0.715) 0.720 0.716

Locus 19984 :

2 alleles

96.9% missing data

4	(0.262) 0.265 0.252

3	(0.738) 0.735 0.748

Locus 19985 :

2 alleles

99.2% missing data

1	(0.652) 0.658 0.641

2	(0.348) 0.342 0.359

Locus 19986 :

2 alleles

97.3% missing data

1	(0.866) 0.872 0.861

2	(0.134) 0.128 0.139

Locus 19987 :

3 alleles

98.8% missing data

3	(0.259) 0.259 0.262

2	(0.255) 0.255 0.257

4	(0.486) 0.487 0.481

Locus 19988 :

2 alleles

99.2% missing data

3	(0.755) 0.751 0.757

1	(0.245) 0.249 0.243

Locus 19989 :

2 alleles

99.2% missing data

3	(0.499) 0.506 0.492

1	(0.501) 0.494 0.508

Locus 19990 :

2 alleles

99.2% missing data

1	(0.510) 0.510 0.509

4	(0.490) 0.490 0.491

Locus 19991 :

2 alleles

99.2% missing data

2	(0.368) 0.363 0.374

3	(0.632) 0.637 0.626

Locus 19992 :

2 alleles

99.2% missing data

4	(0.649) 0.648 0.645

3	(0.351) 0.352 0.355

Locus 19993 :

2 alleles

99.2% missing data

2	(0.453) 0.451 0.457

1	(0.547) 0.549 0.543

Locus 19994 :

2 alleles

99.2% missing data

1	(0.540) 0.545 0.537

3	(0.460) 0.455 0.463

Locus 19995 :

2 alleles

99.2% missing data

2	(0.700) 0.696 0.700

1	(0.300) 0.304 0.300

Locus 19996 :

2 alleles

99.2% missing data

4	(0.384) 0.383 0.389

1	(0.616) 0.617 0.611

Locus 19997 :

2 alleles

99.2% missing data

1	(0.610) 0.607 0.612

4	(0.390) 0.393 0.388

Locus 19998 :

2 alleles

99.2% missing data

3	(0.526) 0.521 0.523

1	(0.474) 0.479 0.477

Locus 19999 :

2 alleles

99.2% missing data

1	(0.475) 0.475 0.473

2	(0.525) 0.525 0.527

Locus 20000 :

2 alleles

98.8% missing data

1	(0.796) 0.786 0.804

3	(0.204) 0.214 0.196

Locus 20001 :

2 alleles

99.2% missing data

2	(0.603) 0.592 0.610

1	(0.397) 0.408 0.390

Locus 20002 :

2 alleles

98.8% missing data

1	(0.530) 0.535 0.529

3	(0.470) 0.465 0.471

Locus 20003 :

2 alleles

98.8% missing data

2	(0.521) 0.517 0.528

3	(0.479) 0.483 0.472

Locus 20004 :

2 alleles

97.7% missing data

3	(0.376) 0.378 0.365

2	(0.624) 0.622 0.635

Locus 20005 :

2 alleles

98.8% missing data

2	(0.656) 0.646 0.657

1	(0.344) 0.354 0.343

Locus 20006 :

2 alleles

98.8% missing data

3	(0.494) 0.493 0.498

2	(0.506) 0.507 0.502

Locus 20007 :

2 alleles

99.2% missing data

2	(0.194) 0.190 0.203

3	(0.806) 0.810 0.797

Locus 20008 :

2 alleles

99.2% missing data

2	(0.397) 0.396 0.402

1	(0.603) 0.604 0.598

Locus 20009 :

2 alleles

99.2% missing data

2	(0.223) 0.219 0.227

4	(0.777) 0.781 0.773

Locus 20010 :

2 alleles

99.2% missing data

3	(0.208) 0.207 0.207

1	(0.792) 0.793 0.793

Locus 20011 :

2 alleles

99.2% missing data

2	(0.667) 0.672 0.664

4	(0.333) 0.328 0.336

Locus 20012 :

2 alleles

99.2% missing data

1	(0.538) 0.542 0.535

4	(0.462) 0.458 0.465

Locus 20013 :

2 alleles

98.8% missing data

2	(0.371) 0.368 0.369

3	(0.629) 0.632 0.631

Locus 20014 :

2 alleles

98.8% missing data

1	(0.643) 0.641 0.639

3	(0.357) 0.359 0.361

Locus 20015 :

2 alleles

99.2% missing data

3	(0.493) 0.493 0.496

1	(0.507) 0.507 0.504

Locus 20016 :

3 alleles

98.4% missing data

2	(0.312) 0.310 0.316

1	(0.199) 0.199 0.196

4	(0.489) 0.491 0.488

Locus 20017 :

2 alleles

99.2% missing data

4	(0.551) 0.553 0.546

2	(0.449) 0.447 0.454

Locus 20018 :

2 alleles

91.0% missing data

2	(0.935) 0.947 0.926

4	(0.065) 0.053 0.074

Locus 20019 :

2 alleles

99.2% missing data

2	(0.367) 0.368 0.371

1	(0.633) 0.632 0.629

Locus 20020 :

2 alleles

99.2% missing data

1	(0.437) 0.436 0.436

3	(0.563) 0.564 0.564

Locus 20021 :

2 alleles

99.2% missing data

2	(0.437) 0.439 0.435

3	(0.563) 0.561 0.565

Locus 20022 :

2 alleles

96.1% missing data

1	(0.762) 0.776 0.755

2	(0.238) 0.224 0.245

Locus 20023 :

2 alleles

99.2% missing data

1	(0.436) 0.439 0.430

2	(0.564) 0.561 0.570

Locus 20024 :

2 alleles

7.0% missing data

1	(0.967) 0.933 0.989

4	(0.033) 0.067 0.011

Locus 20025 :

2 alleles

96.5% missing data

2	(0.336) 0.341 0.326

3	(0.664) 0.659 0.674

Locus 20026 :

2 alleles

99.2% missing data

4	(0.532) 0.535 0.527

1	(0.468) 0.465 0.473

Locus 20027 :

2 alleles

99.2% missing data

3	(0.486) 0.489 0.487

2	(0.514) 0.511 0.513

Locus 20028 :

2 alleles

97.7% missing data

3	(0.600) 0.604 0.605

2	(0.400) 0.396 0.395

Locus 20029 :

2 alleles

99.2% missing data

1	(0.390) 0.386 0.399

3	(0.610) 0.614 0.601

Locus 20030 :

2 alleles

99.2% missing data

1	(0.477) 0.475 0.476

3	(0.523) 0.525 0.524

Locus 20031 :

2 alleles

99.2% missing data

3	(0.281) 0.283 0.286

1	(0.719) 0.717 0.714

Locus 20032 :

2 alleles

99.2% missing data

1	(0.674) 0.686 0.670

4	(0.326) 0.314 0.330

Locus 20033 :

2 alleles

99.2% missing data

4	(0.717) 0.705 0.724

1	(0.283) 0.295 0.276

Locus 20034 :

2 alleles

99.2% missing data

3	(0.337) 0.334 0.341

2	(0.663) 0.666 0.659

Locus 20035 :

2 alleles

4.7% missing data

4	(0.984) 0.981 0.995

2	(0.016) 0.019 0.005

Locus 20036 :

2 alleles

93.8% missing data

4	(0.860) 0.855 0.875

2	(0.140) 0.145 0.125

Locus 20037 :

2 alleles

99.2% missing data

1	(0.529) 0.534 0.526

4	(0.471) 0.466 0.474

Locus 20038 :

2 alleles

14.1% missing data

3	(0.983) 0.986 0.992

4	(0.017) 0.014 0.008

Locus 20039 :

2 alleles

99.2% missing data

1	(0.233) 0.236 0.231

2	(0.767) 0.764 0.769

Locus 20040 :

2 alleles

99.2% missing data

4	(0.463) 0.464 0.465

2	(0.537) 0.536 0.535

Locus 20041 :

2 alleles

96.1% missing data

4	(0.845) 0.848 0.849

1	(0.155) 0.152 0.151

Locus 20042 :

2 alleles

99.2% missing data

2	(0.555) 0.551 0.564

3	(0.445) 0.449 0.436

Locus 20043 :

2 alleles

89.8% missing data

1	(0.816) 0.848 0.784

3	(0.184) 0.152 0.216

Locus 20044 :

2 alleles

48.4% missing data

1	(0.950) 0.956 0.959

2	(0.050) 0.044 0.041

Locus 20045 :

2 alleles

99.2% missing data

3	(0.849) 0.850 0.841

1	(0.151) 0.150 0.159

Locus 20046 :

2 alleles

99.2% missing data

3	(0.555) 0.553 0.550

2	(0.445) 0.447 0.450

Locus 20047 :

2 alleles

99.2% missing data

4	(0.682) 0.685 0.679

1	(0.318) 0.315 0.321

Locus 20048 :

2 alleles

98.8% missing data

4	(0.707) 0.706 0.703

3	(0.293) 0.294 0.297

Locus 20049 :

2 alleles

98.8% missing data

1	(0.635) 0.641 0.636

3	(0.365) 0.359 0.364

Locus 20050 :

2 alleles

99.2% missing data

2	(0.752) 0.746 0.749

4	(0.248) 0.254 0.251

Locus 20051 :

2 alleles

99.2% missing data

2	(0.664) 0.669 0.657

3	(0.336) 0.331 0.343

Locus 20052 :

2 alleles

98.0% missing data

1	(0.787) 0.792 0.784

3	(0.213) 0.208 0.216

Locus 20053 :

2 alleles

99.2% missing data

1	(0.480) 0.468 0.484

2	(0.520) 0.532 0.516

Locus 20054 :

2 alleles

99.2% missing data

3	(0.348) 0.344 0.348

4	(0.652) 0.656 0.652

Locus 20055 :

2 alleles

98.8% missing data

2	(0.396) 0.399 0.397

4	(0.604) 0.601 0.603

Locus 20056 :

2 alleles

91.8% missing data

3	(0.920) 0.927 0.921

4	(0.080) 0.073 0.079

Locus 20057 :

2 alleles

96.9% missing data

1	(0.741) 0.737 0.753

3	(0.259) 0.263 0.247

Locus 20058 :

2 alleles

2.3% missing data

3	(0.987) 0.990 0.993

2	(0.013) 0.010 0.007

Locus 20059 :

2 alleles

99.2% missing data

3	(0.635) 0.630 0.636

4	(0.365) 0.370 0.364

Locus 20060 :

2 alleles

99.2% missing data

3	(0.424) 0.418 0.428

2	(0.576) 0.582 0.572

Locus 20061 :

2 alleles

99.2% missing data

4	(0.217) 0.214 0.220

1	(0.783) 0.786 0.780

Locus 20062 :

2 alleles

99.2% missing data

2	(0.605) 0.602 0.611

3	(0.395) 0.398 0.389

Locus 20063 :

2 alleles

0.8% missing data

4	(0.989) 0.984 0.996

1	(0.011) 0.016 0.004

Locus 20064 :

2 alleles

99.2% missing data

4	(0.497) 0.495 0.500

3	(0.503) 0.505 0.500

Locus 20065 :

2 alleles

99.2% missing data

2	(0.495) 0.498 0.495

1	(0.505) 0.502 0.505

Locus 20066 :

2 alleles

99.2% missing data

4	(0.293) 0.288 0.294

2	(0.707) 0.712 0.706

Locus 20067 :

2 alleles

99.2% missing data

2	(0.439) 0.444 0.448

3	(0.561) 0.556 0.552

Locus 20068 :

2 alleles

97.3% missing data

3	(0.756) 0.758 0.766

1	(0.244) 0.242 0.234

Locus 20069 :

2 alleles

99.2% missing data

1	(0.268) 0.265 0.269

4	(0.732) 0.735 0.731

Locus 20070 :

2 alleles

99.2% missing data

1	(0.413) 0.406 0.414

2	(0.587) 0.594 0.586

Locus 20071 :

2 alleles

96.5% missing data

4	(0.257) 0.248 0.259

3	(0.743) 0.752 0.741

Locus 20072 :

2 alleles

99.2% missing data

3	(0.324) 0.323 0.332

4	(0.676) 0.677 0.668

Locus 20073 :

2 alleles

98.8% missing data

3	(0.733) 0.725 0.736

1	(0.267) 0.275 0.264

Locus 20074 :

2 alleles

99.2% missing data

1	(0.672) 0.674 0.672

2	(0.328) 0.326 0.328

Locus 20075 :

2 alleles

98.8% missing data

2	(0.652) 0.646 0.654

4	(0.348) 0.354 0.346

Locus 20076 :

2 alleles

87.1% missing data

4	(0.725) 0.778 0.657

2	(0.275) 0.222 0.343

Locus 20077 :

2 alleles

99.2% missing data

4	(0.242) 0.239 0.245

2	(0.758) 0.761 0.755

Locus 20078 :

2 alleles

99.2% missing data

1	(0.723) 0.719 0.726

4	(0.277) 0.281 0.274

Locus 20079 :

2 alleles

98.0% missing data

2	(0.715) 0.717 0.717

1	(0.285) 0.283 0.283

Locus 20080 :

2 alleles

99.2% missing data

1	(0.619) 0.622 0.611

3	(0.381) 0.378 0.389

Locus 20081 :

2 alleles

99.2% missing data

1	(0.476) 0.474 0.476

4	(0.524) 0.526 0.524

Locus 20082 :

2 alleles

99.2% missing data

4	(0.681) 0.676 0.680

3	(0.319) 0.324 0.320

Locus 20083 :

2 alleles

99.2% missing data

2	(0.196) 0.199 0.199

3	(0.804) 0.801 0.801

Locus 20084 :

2 alleles

98.8% missing data

4	(0.536) 0.544 0.540

2	(0.464) 0.456 0.460

Locus 20085 :

2 alleles

98.8% missing data

3	(0.626) 0.630 0.612

4	(0.374) 0.370 0.388

Locus 20086 :

2 alleles

99.2% missing data

1	(0.680) 0.684 0.679

3	(0.320) 0.316 0.321

Locus 20087 :

2 alleles

93.0% missing data

3	(0.890) 0.891 0.898

2	(0.110) 0.109 0.102

Locus 20088 :

2 alleles

99.2% missing data

4	(0.595) 0.594 0.591

2	(0.405) 0.406 0.409

Locus 20089 :

2 alleles

98.8% missing data

1	(0.512) 0.501 0.521

3	(0.488) 0.499 0.479

Locus 20090 :

2 alleles

99.2% missing data

4	(0.493) 0.494 0.491

3	(0.507) 0.506 0.509

Locus 20091 :

2 alleles

99.2% missing data

4	(0.127) 0.132 0.124

2	(0.873) 0.868 0.876

Locus 20092 :

2 alleles

99.2% missing data

1	(0.583) 0.588 0.573

2	(0.417) 0.412 0.427

Locus 20093 :

2 alleles

99.2% missing data

4	(0.217) 0.220 0.214

3	(0.783) 0.780 0.786

Locus 20094 :

2 alleles

99.2% missing data

2	(0.516) 0.524 0.512

4	(0.484) 0.476 0.488

Locus 20095 :

2 alleles

99.2% missing data

4	(0.392) 0.384 0.389

3	(0.608) 0.616 0.611

Locus 20096 :

2 alleles

98.4% missing data

2	(0.373) 0.374 0.371

3	(0.627) 0.626 0.629

Locus 20097 :

2 alleles

98.8% missing data

1	(0.433) 0.432 0.433

2	(0.567) 0.568 0.567

Locus 20098 :

3 alleles

98.8% missing data

3	(0.373) 0.373 0.371

1	(0.425) 0.429 0.424

2	(0.202) 0.198 0.205

Locus 20099 :

2 alleles

99.2% missing data

2	(0.577) 0.581 0.575

4	(0.423) 0.419 0.425

Locus 20100 :

2 alleles

99.2% missing data

1	(0.315) 0.311 0.324

4	(0.685) 0.689 0.676

Locus 20101 :

2 alleles

99.2% missing data

3	(0.721) 0.718 0.727

4	(0.279) 0.282 0.273

Locus 20102 :

2 alleles

99.2% missing data

3	(0.679) 0.680 0.676

4	(0.321) 0.320 0.324

Locus 20103 :

2 alleles

99.2% missing data

1	(0.468) 0.468 0.473

3	(0.532) 0.532 0.527

Locus 20104 :

2 alleles

99.2% missing data

2	(0.319) 0.315 0.326

3	(0.681) 0.685 0.674

Locus 20105 :

2 alleles

99.2% missing data

1	(0.475) 0.468 0.480

2	(0.525) 0.532 0.520

Locus 20106 :

2 alleles

99.2% missing data

3	(0.477) 0.473 0.486

4	(0.523) 0.527 0.514

Locus 20107 :

2 alleles

97.7% missing data

3	(0.098) 0.093 0.104

4	(0.902) 0.907 0.896

Locus 20108 :

2 alleles

99.2% missing data

2	(0.560) 0.558 0.559

1	(0.440) 0.442 0.441

Locus 20109 :

2 alleles

99.2% missing data

3	(0.252) 0.257 0.252

4	(0.748) 0.743 0.748

Locus 20110 :

3 alleles

98.8% missing data

3	(0.206) 0.207 0.200

4	(0.157) 0.159 0.157

1	(0.637) 0.634 0.643

Locus 20111 :

2 alleles

99.2% missing data

4	(0.795) 0.797 0.793

2	(0.205) 0.203 0.207

Locus 20112 :

2 alleles

97.7% missing data

1	(0.288) 0.296 0.278

4	(0.712) 0.704 0.722

Locus 20113 :

2 alleles

98.0% missing data

4	(0.332) 0.332 0.328

3	(0.668) 0.668 0.672

Locus 20114 :

2 alleles

98.8% missing data

3	(0.652) 0.651 0.651

2	(0.348) 0.349 0.349

Locus 20115 :

2 alleles

93.4% missing data

2	(0.899) 0.898 0.910

3	(0.101) 0.102 0.090

Locus 20116 :

3 alleles

97.3% missing data

2	(0.445) 0.461 0.444

1	(0.277) 0.271 0.272

3	(0.278) 0.267 0.284

Locus 20117 :

2 alleles

99.2% missing data

4	(0.701) 0.691 0.706

2	(0.299) 0.309 0.294

Locus 20118 :

2 alleles

99.2% missing data

3	(0.229) 0.228 0.232

4	(0.771) 0.772 0.768

Locus 20119 :

2 alleles

22.7% missing data

1	(0.957) 0.968 0.958

2	(0.043) 0.032 0.042

Locus 20120 :

2 alleles

97.3% missing data

2	(0.318) 0.320 0.303

1	(0.682) 0.680 0.697

Locus 20121 :

2 alleles

8.6% missing data

4	(0.971) 0.935 0.990

1	(0.029) 0.065 0.010

Locus 20122 :

2 alleles

98.8% missing data

3	(0.309) 0.303 0.310

4	(0.691) 0.697 0.690

Locus 20123 :

2 alleles

99.2% missing data

2	(0.450) 0.449 0.456

1	(0.550) 0.551 0.544

Locus 20124 :

2 alleles

88.3% missing data

4	(0.745) 0.799 0.698

1	(0.255) 0.201 0.302

Locus 20125 :

2 alleles

99.2% missing data

1	(0.660) 0.662 0.655

4	(0.340) 0.338 0.345

Locus 20126 :

2 alleles

96.9% missing data

4	(0.902) 0.903 0.903

1	(0.098) 0.097 0.097

Locus 20127 :

2 alleles

83.6% missing data

2	(0.472) 0.537 0.409

4	(0.528) 0.463 0.591

Locus 20128 :

2 alleles

97.7% missing data

4	(0.150) 0.141 0.156

3	(0.850) 0.859 0.844

Locus 20129 :

3 alleles

98.0% missing data

3	(0.491) 0.496 0.486

4	(0.309) 0.314 0.311

1	(0.200) 0.190 0.204

Locus 20130 :

2 alleles

99.2% missing data

2	(0.446) 0.449 0.444

3	(0.554) 0.551 0.556

Locus 20131 :

2 alleles

95.7% missing data

2	(0.227) 0.220 0.230

1	(0.773) 0.780 0.770

Locus 20132 :

2 alleles

99.2% missing data

3	(0.419) 0.418 0.416

2	(0.581) 0.582 0.584

Locus 20133 :

2 alleles

96.9% missing data

2	(0.126) 0.126 0.124

3	(0.874) 0.874 0.876

Locus 20134 :

2 alleles

99.2% missing data

2	(0.222) 0.224 0.226

3	(0.778) 0.776 0.774

Locus 20135 :

2 alleles

98.8% missing data

2	(0.618) 0.617 0.621

1	(0.382) 0.383 0.379

Locus 20136 :

2 alleles

97.7% missing data

4	(0.261) 0.247 0.271

2	(0.739) 0.753 0.729

Locus 20137 :

2 alleles

99.2% missing data

3	(0.333) 0.333 0.334

4	(0.667) 0.667 0.666

Locus 20138 :

3 alleles

98.0% missing data

1	(0.458) 0.453 0.471

4	(0.226) 0.228 0.220

2	(0.316) 0.320 0.309

Locus 20139 :

2 alleles

99.2% missing data

3	(0.552) 0.548 0.557

2	(0.448) 0.452 0.443

Locus 20140 :

2 alleles

98.8% missing data

4	(0.476) 0.474 0.481

2	(0.524) 0.526 0.519

Locus 20141 :

2 alleles

99.2% missing data

4	(0.352) 0.348 0.360

2	(0.648) 0.652 0.640

Locus 20142 :

2 alleles

99.2% missing data

4	(0.406) 0.408 0.409

1	(0.594) 0.592 0.591

Locus 20143 :

2 alleles

99.2% missing data

2	(0.428) 0.428 0.431

4	(0.572) 0.572 0.569

Locus 20144 :

3 alleles

98.8% missing data

3	(0.364) 0.360 0.367

1	(0.423) 0.425 0.417

2	(0.213) 0.215 0.216

Locus 20145 :

2 alleles

99.2% missing data

3	(0.527) 0.532 0.524

2	(0.473) 0.468 0.476

Locus 20146 :

2 alleles

91.4% missing data

2	(0.917) 0.922 0.922

3	(0.083) 0.078 0.078

Locus 20147 :

2 alleles

99.2% missing data

1	(0.652) 0.648 0.653

3	(0.348) 0.352 0.347

Locus 20148 :

2 alleles

99.2% missing data

3	(0.573) 0.578 0.574

2	(0.427) 0.422 0.426

Locus 20149 :

3 alleles

96.5% missing data

3	(0.671) 0.688 0.668

1	(0.157) 0.143 0.165

2	(0.172) 0.169 0.168

Locus 20150 :

2 alleles

99.2% missing data

3	(0.650) 0.644 0.648

4	(0.350) 0.356 0.352

Locus 20151 :

2 alleles

99.2% missing data

4	(0.453) 0.448 0.457

3	(0.547) 0.552 0.543

Locus 20152 :

2 alleles

98.8% missing data

1	(0.695) 0.699 0.694

3	(0.305) 0.301 0.306

Locus 20153 :

2 alleles

99.2% missing data

1	(0.461) 0.467 0.454

3	(0.539) 0.533 0.546

Locus 20154 :

2 alleles

99.2% missing data

1	(0.459) 0.458 0.456

3	(0.541) 0.542 0.544

Locus 20155 :

2 alleles

99.2% missing data

2	(0.387) 0.389 0.383

4	(0.613) 0.611 0.617

Locus 20156 :

2 alleles

99.2% missing data

3	(0.607) 0.604 0.612

2	(0.393) 0.396 0.388

Locus 20157 :

2 alleles

99.2% missing data

2	(0.694) 0.695 0.694

1	(0.306) 0.305 0.306

Locus 20158 :

2 alleles

99.2% missing data

3	(0.449) 0.457 0.448

2	(0.551) 0.543 0.552

Locus 20159 :

2 alleles

98.8% missing data

2	(0.514) 0.515 0.514

4	(0.486) 0.485 0.486

Locus 20160 :

2 alleles

99.2% missing data

1	(0.457) 0.448 0.458

3	(0.543) 0.552 0.542

Locus 20161 :

2 alleles

98.8% missing data

3	(0.472) 0.481 0.467

2	(0.528) 0.519 0.533

Locus 20162 :

2 alleles

99.2% missing data

1	(0.662) 0.654 0.662

3	(0.338) 0.346 0.338

Locus 20163 :

2 alleles

99.2% missing data

1	(0.530) 0.525 0.533

3	(0.470) 0.475 0.467

Locus 20164 :

2 alleles

99.2% missing data

1	(0.785) 0.787 0.779

4	(0.215) 0.213 0.221

Locus 20165 :

2 alleles

99.2% missing data

2	(0.492) 0.492 0.498

1	(0.508) 0.508 0.502

Locus 20166 :

2 alleles

97.7% missing data

2	(0.279) 0.267 0.285

4	(0.721) 0.733 0.715

Locus 20167 :

2 alleles

99.2% missing data

2	(0.595) 0.595 0.592

4	(0.405) 0.405 0.408

Locus 20168 :

2 alleles

99.2% missing data

1	(0.335) 0.326 0.346

2	(0.665) 0.674 0.654

Locus 20169 :

2 alleles

99.2% missing data

3	(0.803) 0.799 0.802

4	(0.197) 0.201 0.198

Locus 20170 :

2 alleles

99.2% missing data

4	(0.503) 0.493 0.508

3	(0.497) 0.507 0.492

Locus 20171 :

2 alleles

99.2% missing data

4	(0.725) 0.724 0.726

1	(0.275) 0.276 0.274

Locus 20172 :

2 alleles

97.7% missing data

1	(0.703) 0.702 0.710

3	(0.297) 0.298 0.290

Locus 20173 :

2 alleles

99.2% missing data

4	(0.554) 0.554 0.555

2	(0.446) 0.446 0.445

Locus 20174 :

2 alleles

99.2% missing data

3	(0.566) 0.571 0.558

1	(0.434) 0.429 0.442

Locus 20175 :

2 alleles

99.2% missing data

4	(0.182) 0.175 0.190

3	(0.818) 0.825 0.810

Locus 20176 :

2 alleles

97.7% missing data

4	(0.326) 0.320 0.316

3	(0.674) 0.680 0.684

Locus 20177 :

2 alleles

99.2% missing data

2	(0.156) 0.164 0.156

3	(0.844) 0.836 0.844

Locus 20178 :

2 alleles

98.8% missing data

1	(0.551) 0.549 0.555

2	(0.449) 0.451 0.445

Locus 20179 :

2 alleles

99.2% missing data

4	(0.501) 0.499 0.499

3	(0.499) 0.501 0.501

Locus 20180 :

2 alleles

98.8% missing data

3	(0.692) 0.693 0.681

2	(0.308) 0.307 0.319

Locus 20181 :

2 alleles

8.2% missing data

3	(0.982) 0.975 0.995

4	(0.018) 0.025 0.005

Locus 20182 :

2 alleles

99.2% missing data

3	(0.398) 0.402 0.391

1	(0.602) 0.598 0.609

Locus 20183 :

2 alleles

99.2% missing data

1	(0.621) 0.624 0.615

2	(0.379) 0.376 0.385

Locus 20184 :

2 alleles

98.8% missing data

2	(0.490) 0.490 0.499

3	(0.510) 0.510 0.501

Locus 20185 :

2 alleles

99.2% missing data

4	(0.416) 0.409 0.421

2	(0.584) 0.591 0.579

Locus 20186 :

2 alleles

99.2% missing data

2	(0.484) 0.484 0.483

1	(0.516) 0.516 0.517

Locus 20187 :

2 alleles

99.2% missing data

1	(0.441) 0.444 0.440

3	(0.559) 0.556 0.560

Locus 20188 :

2 alleles

98.8% missing data

3	(0.851) 0.855 0.843

1	(0.149) 0.145 0.157

Locus 20189 :

2 alleles

98.8% missing data

1	(0.570) 0.565 0.577

4	(0.430) 0.435 0.423

Locus 20190 :

2 alleles

99.2% missing data

2	(0.451) 0.446 0.454

3	(0.549) 0.554 0.546

Locus 20191 :

2 alleles

91.0% missing data

4	(0.911) 0.919 0.912

1	(0.089) 0.081 0.088

Locus 20192 :

2 alleles

98.8% missing data

4	(0.384) 0.388 0.376

2	(0.616) 0.612 0.624

Locus 20193 :

2 alleles

99.2% missing data

3	(0.505) 0.507 0.505

1	(0.495) 0.493 0.495

Locus 20194 :

2 alleles

99.2% missing data

2	(0.722) 0.724 0.716

4	(0.278) 0.276 0.284

Locus 20195 :

2 alleles

99.2% missing data

1	(0.449) 0.451 0.448

3	(0.551) 0.549 0.552

Locus 20196 :

2 alleles

97.7% missing data

1	(0.181) 0.177 0.179

2	(0.819) 0.823 0.821

Locus 20197 :

2 alleles

99.2% missing data

4	(0.505) 0.496 0.513

2	(0.495) 0.504 0.487

Locus 20198 :

3 alleles

98.8% missing data

4	(0.245) 0.237 0.249

1	(0.316) 0.314 0.318

2	(0.439) 0.449 0.432

Locus 20199 :

2 alleles

99.2% missing data

3	(0.354) 0.351 0.360

4	(0.646) 0.649 0.640

Locus 20200 :

2 alleles

99.2% missing data

2	(0.545) 0.552 0.547

1	(0.455) 0.448 0.453

Locus 20201 :

2 alleles

98.8% missing data

2	(0.350) 0.343 0.355

3	(0.650) 0.657 0.645

Locus 20202 :

2 alleles

99.2% missing data

2	(0.518) 0.521 0.508

3	(0.482) 0.479 0.492

Locus 20203 :

2 alleles

99.2% missing data

1	(0.397) 0.400 0.396

2	(0.603) 0.600 0.604

Locus 20204 :

2 alleles

98.8% missing data

4	(0.378) 0.377 0.371

2	(0.622) 0.623 0.629

Locus 20205 :

2 alleles

99.2% missing data

4	(0.406) 0.405 0.413

1	(0.594) 0.595 0.587

Locus 20206 :

2 alleles

98.8% missing data

3	(0.834) 0.840 0.830

2	(0.166) 0.160 0.170

Locus 20207 :

2 alleles

96.9% missing data

1	(0.206) 0.207 0.200

3	(0.794) 0.793 0.800

Locus 20208 :

2 alleles

99.2% missing data

4	(0.348) 0.348 0.348

1	(0.652) 0.652 0.652

Locus 20209 :

2 alleles

99.2% missing data

4	(0.581) 0.580 0.581

3	(0.419) 0.420 0.419

Locus 20210 :

2 alleles

99.2% missing data

2	(0.706) 0.700 0.713

1	(0.294) 0.300 0.287

Locus 20211 :

2 alleles

96.9% missing data

1	(0.797) 0.797 0.801

3	(0.203) 0.203 0.199

Locus 20212 :

2 alleles

98.8% missing data

2	(0.730) 0.721 0.735

1	(0.270) 0.279 0.265

Locus 20213 :

2 alleles

92.6% missing data

3	(0.910) 0.910 0.913

1	(0.090) 0.090 0.087

Locus 20214 :

2 alleles

99.2% missing data

3	(0.536) 0.526 0.539

1	(0.464) 0.474 0.461

Locus 20215 :

2 alleles

99.2% missing data

2	(0.701) 0.704 0.702

3	(0.299) 0.296 0.298

Locus 20216 :

3 alleles

98.4% missing data

4	(0.245) 0.247 0.246

1	(0.435) 0.440 0.427

3	(0.320) 0.313 0.327

Locus 20217 :

2 alleles

99.2% missing data

3	(0.355) 0.359 0.352

1	(0.645) 0.641 0.648

Locus 20218 :

3 alleles

98.8% missing data

4	(0.225) 0.229 0.224

3	(0.277) 0.279 0.279

1	(0.498) 0.492 0.498

Locus 20219 :

2 alleles

99.2% missing data

1	(0.551) 0.544 0.555

4	(0.449) 0.456 0.445

Locus 20220 :

3 alleles

98.8% missing data

3	(0.314) 0.308 0.323

1	(0.476) 0.479 0.472

4	(0.210) 0.213 0.205

Locus 20221 :

2 alleles

99.2% missing data

1	(0.763) 0.766 0.757

4	(0.237) 0.234 0.243

Locus 20222 :

2 alleles

99.2% missing data

1	(0.441) 0.442 0.442

3	(0.559) 0.558 0.558

Locus 20223 :

2 alleles

99.2% missing data

1	(0.475) 0.479 0.475

3	(0.525) 0.521 0.525

Locus 20224 :

2 alleles

99.2% missing data

3	(0.589) 0.586 0.597

2	(0.411) 0.414 0.403

Locus 20225 :

2 alleles

99.2% missing data

3	(0.566) 0.562 0.568

1	(0.434) 0.438 0.432

Locus 20226 :

2 alleles

99.2% missing data

3	(0.462) 0.461 0.467

4	(0.538) 0.539 0.533

Locus 20227 :

2 alleles

99.2% missing data

4	(0.428) 0.428 0.429

3	(0.572) 0.572 0.571

Locus 20228 :

2 alleles

99.2% missing data

3	(0.299) 0.296 0.305

2	(0.701) 0.704 0.695

Locus 20229 :

2 alleles

98.8% missing data

3	(0.607) 0.600 0.616

2	(0.393) 0.400 0.384

Locus 20230 :

2 alleles

98.8% missing data

2	(0.609) 0.612 0.608

4	(0.391) 0.388 0.392

Locus 20231 :

2 alleles

98.0% missing data

2	(0.534) 0.545 0.534

1	(0.466) 0.455 0.466

Locus 20232 :

2 alleles

99.2% missing data

1	(0.687) 0.674 0.687

3	(0.313) 0.326 0.313

Locus 20233 :

3 alleles

96.9% missing data

4	(0.290) 0.289 0.291

1	(0.495) 0.488 0.508

2	(0.215) 0.222 0.201

Locus 20234 :

2 alleles

99.2% missing data

2	(0.269) 0.268 0.267

1	(0.731) 0.732 0.733

Locus 20235 :

2 alleles

93.0% missing data

1	(0.878) 0.879 0.888

2	(0.122) 0.121 0.112

Locus 20236 :

2 alleles

98.8% missing data

3	(0.522) 0.519 0.530

1	(0.478) 0.481 0.470

Locus 20237 :

2 alleles

9.0% missing data

2	(0.986) 0.982 0.995

3	(0.014) 0.018 0.005

Locus 20238 :

2 alleles

99.2% missing data

3	(0.253) 0.247 0.265

1	(0.747) 0.753 0.735

Locus 20239 :

2 alleles

99.2% missing data

2	(0.424) 0.426 0.423

1	(0.576) 0.574 0.577

Locus 20240 :

3 alleles

98.8% missing data

2	(0.195) 0.190 0.197

3	(0.572) 0.572 0.575

1	(0.233) 0.239 0.228

Locus 20241 :

2 alleles

96.1% missing data

2	(0.224) 0.210 0.228

1	(0.776) 0.790 0.772

Locus 20242 :

2 alleles

99.2% missing data

1	(0.703) 0.700 0.704

3	(0.297) 0.300 0.296

Locus 20243 :

2 alleles

97.3% missing data

2	(0.325) 0.329 0.315

3	(0.675) 0.671 0.685

Locus 20244 :

2 alleles

99.2% missing data

1	(0.408) 0.404 0.412

3	(0.592) 0.596 0.588

Locus 20245 :

2 alleles

99.2% missing data

3	(0.280) 0.282 0.277

1	(0.720) 0.718 0.723

Locus 20246 :

2 alleles

99.2% missing data

2	(0.498) 0.489 0.503

3	(0.502) 0.511 0.497

Locus 20247 :

2 alleles

96.9% missing data

3	(0.841) 0.846 0.844

1	(0.159) 0.154 0.156

Locus 20248 :

2 alleles

95.3% missing data

1	(0.906) 0.908 0.907

4	(0.094) 0.092 0.093

Locus 20249 :

3 alleles

90.2% missing data

1	(0.765) 0.809 0.737

2	(0.139) 0.113 0.161

4	(0.095) 0.078 0.101

Locus 20250 :

2 alleles

82.8% missing data

1	(0.441) 0.516 0.380

3	(0.559) 0.484 0.620

Locus 20251 :

2 alleles

96.1% missing data

2	(0.144) 0.147 0.138

3	(0.856) 0.853 0.862

Locus 20252 :

3 alleles

98.8% missing data

3	(0.113) 0.110 0.117

1	(0.345) 0.342 0.344

2	(0.542) 0.548 0.539

Locus 20253 :

2 alleles

99.2% missing data

4	(0.573) 0.567 0.578

3	(0.427) 0.433 0.422

Locus 20254 :

3 alleles

98.4% missing data

4	(0.360) 0.354 0.376

3	(0.206) 0.212 0.202

2	(0.434) 0.434 0.423

Locus 20255 :

2 alleles

99.2% missing data

3	(0.500) 0.499 0.506

2	(0.500) 0.501 0.494

Locus 20256 :

2 alleles

99.2% missing data

4	(0.278) 0.275 0.283

1	(0.722) 0.725 0.717

Locus 20257 :

3 alleles

98.8% missing data

1	(0.360) 0.360 0.363

4	(0.214) 0.211 0.218

3	(0.425) 0.429 0.419

Locus 20258 :

2 alleles

91.8% missing data

4	(0.836) 0.821 0.853

1	(0.164) 0.179 0.147

Locus 20259 :

2 alleles

95.3% missing data

4	(0.141) 0.153 0.126

3	(0.859) 0.847 0.874

Locus 20260 :

2 alleles

99.2% missing data

3	(0.672) 0.669 0.679

1	(0.328) 0.331 0.321

Locus 20261 :

2 alleles

99.2% missing data

1	(0.477) 0.471 0.479

3	(0.523) 0.529 0.521

Locus 20262 :

2 alleles

97.7% missing data

3	(0.112) 0.112 0.113

2	(0.888) 0.888 0.887

Locus 20263 :

3 alleles

94.5% missing data

2	(0.206) 0.201 0.197

1	(0.709) 0.708 0.726

4	(0.085) 0.091 0.077

Locus 20264 :

2 alleles

99.2% missing data

2	(0.655) 0.654 0.654

4	(0.345) 0.346 0.346

Locus 20265 :

2 alleles

98.8% missing data

2	(0.645) 0.650 0.646

1	(0.355) 0.350 0.354

Locus 20266 :

2 alleles

99.2% missing data

4	(0.585) 0.587 0.586

3	(0.415) 0.413 0.414

Locus 20267 :

2 alleles

99.2% missing data

2	(0.340) 0.333 0.346

3	(0.660) 0.667 0.654

Locus 20268 :

2 alleles

99.2% missing data

3	(0.412) 0.416 0.409

1	(0.588) 0.584 0.591

Locus 20269 :

2 alleles

98.4% missing data

2	(0.624) 0.624 0.627

3	(0.376) 0.376 0.373

Locus 20270 :

2 alleles

99.2% missing data

2	(0.667) 0.658 0.666

1	(0.333) 0.342 0.334

Locus 20271 :

2 alleles

96.1% missing data

3	(0.817) 0.822 0.820

2	(0.183) 0.178 0.180

Locus 20272 :

2 alleles

99.2% missing data

1	(0.740) 0.739 0.738

3	(0.260) 0.261 0.262

Locus 20273 :

2 alleles

99.2% missing data

1	(0.549) 0.544 0.543

3	(0.451) 0.456 0.457

Locus 20274 :

2 alleles

99.2% missing data

1	(0.412) 0.412 0.417

3	(0.588) 0.588 0.583

Locus 20275 :

2 alleles

98.8% missing data

3	(0.696) 0.692 0.694

4	(0.304) 0.308 0.306

Locus 20276 :

2 alleles

99.2% missing data

2	(0.224) 0.227 0.229

1	(0.776) 0.773 0.771

Locus 20277 :

2 alleles

99.2% missing data

2	(0.604) 0.600 0.605

3	(0.396) 0.400 0.395

Locus 20278 :

2 alleles

99.2% missing data

1	(0.447) 0.445 0.452

3	(0.553) 0.555 0.548

Locus 20279 :

2 alleles

99.2% missing data

3	(0.595) 0.597 0.591

4	(0.405) 0.403 0.409

Locus 20280 :

2 alleles

99.2% missing data

3	(0.439) 0.441 0.448

1	(0.561) 0.559 0.552

Locus 20281 :

2 alleles

99.2% missing data

3	(0.685) 0.680 0.689

1	(0.315) 0.320 0.311

Locus 20282 :

2 alleles

91.0% missing data

4	(0.894) 0.912 0.882

2	(0.106) 0.088 0.118

Locus 20283 :

2 alleles

99.2% missing data

3	(0.310) 0.310 0.309

4	(0.690) 0.690 0.691

Locus 20284 :

3 alleles

98.8% missing data

1	(0.133) 0.133 0.135

3	(0.473) 0.472 0.472

4	(0.395) 0.395 0.393

Locus 20285 :

2 alleles

99.2% missing data

4	(0.386) 0.383 0.384

3	(0.614) 0.617 0.616

Locus 20286 :

2 alleles

90.6% missing data

2	(0.880) 0.890 0.874

3	(0.120) 0.110 0.126

Locus 20287 :

2 alleles

99.2% missing data

3	(0.334) 0.333 0.336

1	(0.666) 0.667 0.664

Locus 20288 :

2 alleles

99.2% missing data

2	(0.388) 0.386 0.390

1	(0.612) 0.614 0.610

Locus 20289 :

3 alleles

98.8% missing data

2	(0.191) 0.191 0.197

3	(0.343) 0.339 0.339

4	(0.466) 0.469 0.464

Locus 20290 :

2 alleles

98.0% missing data

3	(0.730) 0.729 0.726

1	(0.270) 0.271 0.274

Locus 20291 :

2 alleles

96.5% missing data

3	(0.145) 0.143 0.141

1	(0.855) 0.857 0.859

Locus 20292 :

2 alleles

99.2% missing data

1	(0.390) 0.387 0.389

3	(0.610) 0.613 0.611

Locus 20293 :

3 alleles

98.4% missing data

2	(0.330) 0.311 0.343

1	(0.460) 0.477 0.450

3	(0.210) 0.212 0.207

Locus 20294 :

2 alleles

98.4% missing data

1	(0.354) 0.347 0.362

3	(0.646) 0.653 0.638

Locus 20295 :

2 alleles

99.2% missing data

1	(0.637) 0.637 0.635

2	(0.363) 0.363 0.365

Locus 20296 :

2 alleles

99.2% missing data

1	(0.708) 0.698 0.708

3	(0.292) 0.302 0.292

Locus 20297 :

2 alleles

99.2% missing data

3	(0.414) 0.412 0.418

4	(0.586) 0.588 0.582

Locus 20298 :

2 alleles

98.0% missing data

2	(0.763) 0.762 0.766

3	(0.237) 0.238 0.234

Locus 20299 :

2 alleles

98.4% missing data

4	(0.497) 0.513 0.489

3	(0.503) 0.487 0.511

Locus 20300 :

2 alleles

98.4% missing data

3	(0.477) 0.478 0.485

2	(0.523) 0.522 0.515

Locus 20301 :

2 alleles

96.5% missing data

3	(0.167) 0.157 0.172

4	(0.833) 0.843 0.828

Locus 20302 :

3 alleles

98.8% missing data

3	(0.377) 0.377 0.372

4	(0.375) 0.366 0.384

2	(0.248) 0.257 0.244

Locus 20303 :

2 alleles

96.1% missing data

4	(0.869) 0.871 0.873

1	(0.131) 0.129 0.127

Locus 20304 :

2 alleles

99.2% missing data

4	(0.666) 0.659 0.669

1	(0.334) 0.341 0.331

Locus 20305 :

2 alleles

94.1% missing data

4	(0.898) 0.893 0.911

1	(0.102) 0.107 0.089

Locus 20306 :

2 alleles

99.2% missing data

1	(0.224) 0.225 0.224

4	(0.776) 0.775 0.776

Locus 20307 :

2 alleles

98.4% missing data

4	(0.261) 0.253 0.263

1	(0.739) 0.747 0.737

Locus 20308 :

2 alleles

98.0% missing data

3	(0.489) 0.501 0.488

1	(0.511) 0.499 0.512

Locus 20309 :

2 alleles

99.2% missing data

4	(0.309) 0.308 0.315

1	(0.691) 0.692 0.685

Locus 20310 :

2 alleles

89.5% missing data

3	(0.948) 0.945 0.958

1	(0.052) 0.055 0.042

Locus 20311 :

3 alleles

98.8% missing data

4	(0.337) 0.334 0.339

2	(0.377) 0.371 0.381

3	(0.287) 0.295 0.280

Locus 20312 :

2 alleles

99.2% missing data

2	(0.498) 0.497 0.500

4	(0.502) 0.503 0.500

Locus 20313 :

2 alleles

98.4% missing data

4	(0.554) 0.554 0.547

2	(0.446) 0.446 0.453

Locus 20314 :

2 alleles

99.2% missing data

1	(0.448) 0.444 0.454

4	(0.552) 0.556 0.546

Locus 20315 :

2 alleles

99.2% missing data

3	(0.697) 0.702 0.697

1	(0.303) 0.298 0.303

Locus 20316 :

3 alleles

98.8% missing data

4	(0.368) 0.367 0.369

1	(0.137) 0.135 0.144

3	(0.495) 0.497 0.486

Locus 20317 :

2 alleles

99.2% missing data

1	(0.454) 0.454 0.452

4	(0.546) 0.546 0.548

Locus 20318 :

2 alleles

98.0% missing data

3	(0.845) 0.843 0.849

1	(0.155) 0.157 0.151

Locus 20319 :

2 alleles

99.2% missing data

1	(0.702) 0.703 0.706

3	(0.298) 0.297 0.294

Locus 20320 :

3 alleles

98.4% missing data

3	(0.537) 0.541 0.539

1	(0.324) 0.317 0.324

4	(0.139) 0.142 0.136

Locus 20321 :

2 alleles

99.2% missing data

1	(0.295) 0.294 0.295

4	(0.705) 0.706 0.705

Locus 20322 :

2 alleles

99.2% missing data

1	(0.589) 0.595 0.586

3	(0.411) 0.405 0.414

Locus 20323 :

2 alleles

8.2% missing data

4	(0.987) 0.981 0.996

1	(0.013) 0.019 0.004

Locus 20324 :

2 alleles

99.2% missing data

1	(0.377) 0.375 0.382

2	(0.623) 0.625 0.618

Locus 20325 :

2 alleles

99.2% missing data

4	(0.472) 0.463 0.467

1	(0.528) 0.537 0.533

Locus 20326 :

3 alleles

98.8% missing data

1	(0.259) 0.261 0.255

2	(0.221) 0.211 0.232

3	(0.520) 0.529 0.513

Locus 20327 :

2 alleles

99.2% missing data

1	(0.563) 0.565 0.562

3	(0.437) 0.435 0.438

Locus 20328 :

2 alleles

98.8% missing data

4	(0.428) 0.425 0.439

3	(0.572) 0.575 0.561

Locus 20329 :

2 alleles

9.0% missing data

4	(0.985) 0.986 0.992

3	(0.015) 0.014 0.008

Locus 20330 :

2 alleles

96.5% missing data

3	(0.595) 0.585 0.612

4	(0.405) 0.415 0.388

Locus 20331 :

2 alleles

99.2% missing data

3	(0.622) 0.633 0.611

4	(0.378) 0.367 0.389

Locus 20332 :

2 alleles

99.2% missing data

3	(0.569) 0.572 0.569

1	(0.431) 0.428 0.431

Locus 20333 :

2 alleles

99.2% missing data

3	(0.273) 0.274 0.273

4	(0.727) 0.726 0.727

Locus 20334 :

3 alleles

98.4% missing data

3	(0.214) 0.213 0.223

2	(0.223) 0.225 0.217

1	(0.562) 0.562 0.560

Locus 20335 :

2 alleles

99.2% missing data

1	(0.684) 0.680 0.693

3	(0.316) 0.320 0.307

Locus 20336 :

2 alleles

98.4% missing data

1	(0.459) 0.458 0.460

3	(0.541) 0.542 0.540

Locus 20337 :

2 alleles

97.7% missing data

1	(0.500) 0.484 0.510

2	(0.500) 0.516 0.490

Locus 20338 :

2 alleles

99.2% missing data

4	(0.655) 0.660 0.653

2	(0.345) 0.340 0.347

Locus 20339 :

2 alleles

98.4% missing data

4	(0.341) 0.332 0.346

2	(0.659) 0.668 0.654

Locus 20340 :

2 alleles

94.9% missing data

3	(0.248) 0.251 0.236

4	(0.752) 0.749 0.764

Locus 20341 :

2 alleles

99.2% missing data

4	(0.204) 0.204 0.206

3	(0.796) 0.796 0.794

Locus 20342 :

2 alleles

99.2% missing data

1	(0.417) 0.423 0.414

3	(0.583) 0.577 0.586

Locus 20343 :

2 alleles

99.2% missing data

4	(0.490) 0.486 0.488

1	(0.510) 0.514 0.512

Locus 20344 :

2 alleles

99.2% missing data

3	(0.551) 0.546 0.554

1	(0.449) 0.454 0.446

Locus 20345 :

2 alleles

99.2% missing data

2	(0.588) 0.590 0.583

1	(0.412) 0.410 0.417

Locus 20346 :

3 alleles

98.8% missing data

4	(0.381) 0.385 0.385

3	(0.486) 0.483 0.479

1	(0.133) 0.131 0.137

Locus 20347 :

2 alleles

99.2% missing data

1	(0.586) 0.579 0.585

2	(0.414) 0.421 0.415

Locus 20348 :

2 alleles

99.2% missing data

3	(0.602) 0.596 0.603

2	(0.398) 0.404 0.397

Locus 20349 :

2 alleles

99.2% missing data

3	(0.613) 0.611 0.614

4	(0.387) 0.389 0.386

Locus 20350 :

2 alleles

99.2% missing data

4	(0.699) 0.702 0.690

2	(0.301) 0.298 0.310

Locus 20351 :

2 alleles

96.1% missing data

3	(0.225) 0.210 0.230

1	(0.775) 0.790 0.770

Locus 20352 :

2 alleles

99.2% missing data

4	(0.449) 0.452 0.452

2	(0.551) 0.548 0.548

Locus 20353 :

2 alleles

99.2% missing data

3	(0.577) 0.578 0.582

1	(0.423) 0.422 0.418

Locus 20354 :

2 alleles

99.2% missing data

1	(0.338) 0.338 0.342

4	(0.662) 0.662 0.658

Locus 20355 :

2 alleles

96.1% missing data

3	(0.765) 0.774 0.768

2	(0.235) 0.226 0.232

Locus 20356 :

2 alleles

99.2% missing data

3	(0.565) 0.565 0.562

4	(0.435) 0.435 0.438

Locus 20357 :

2 alleles

96.5% missing data

4	(0.881) 0.886 0.880

1	(0.119) 0.114 0.120

Locus 20358 :

2 alleles

99.2% missing data

4	(0.612) 0.605 0.615

3	(0.388) 0.395 0.385

Locus 20359 :

2 alleles

99.2% missing data

2	(0.234) 0.230 0.239

4	(0.766) 0.770 0.761

Locus 20360 :

2 alleles

99.2% missing data

2	(0.637) 0.633 0.639

3	(0.363) 0.367 0.361

Locus 20361 :

2 alleles

91.0% missing data

2	(0.927) 0.942 0.918

1	(0.073) 0.058 0.082

Locus 20362 :

2 alleles

8.2% missing data

1	(0.982) 0.976 0.995

4	(0.018) 0.024 0.005

Locus 20363 :

2 alleles

96.1% missing data

4	(0.856) 0.855 0.862

3	(0.144) 0.145 0.138

Locus 20364 :

2 alleles

98.8% missing data

2	(0.587) 0.591 0.585

3	(0.413) 0.409 0.415

Locus 20365 :

2 alleles

98.8% missing data

2	(0.523) 0.521 0.517

1	(0.477) 0.479 0.483

Locus 20366 :

2 alleles

98.4% missing data

1	(0.562) 0.565 0.559

3	(0.438) 0.435 0.441

Locus 20367 :

2 alleles

99.2% missing data

2	(0.451) 0.449 0.458

1	(0.549) 0.551 0.542

Locus 20368 :

2 alleles

98.0% missing data

2	(0.745) 0.743 0.749

4	(0.255) 0.257 0.251

Locus 20369 :

2 alleles

99.2% missing data

1	(0.380) 0.376 0.383

2	(0.620) 0.624 0.617

Locus 20370 :

2 alleles

99.2% missing data

1	(0.411) 0.409 0.418

4	(0.589) 0.591 0.582

Locus 20371 :

2 alleles

0.4% missing data

4	(0.984) 0.987 0.992

1	(0.016) 0.013 0.008

Locus 20372 :

2 alleles

99.2% missing data

4	(0.572) 0.568 0.576

3	(0.428) 0.432 0.424

Locus 20373 :

2 alleles

99.2% missing data

3	(0.392) 0.390 0.398

4	(0.608) 0.610 0.602

Locus 20374 :

2 alleles

96.5% missing data

2	(0.482) 0.485 0.472

3	(0.518) 0.515 0.528

Locus 20375 :

2 alleles

99.2% missing data

4	(0.729) 0.722 0.732

3	(0.271) 0.278 0.268

Locus 20376 :

2 alleles

98.4% missing data

3	(0.465) 0.465 0.474

4	(0.535) 0.535 0.526

Locus 20377 :

2 alleles

97.3% missing data

1	(0.298) 0.284 0.308

4	(0.702) 0.716 0.692

Locus 20378 :

3 alleles

98.0% missing data

1	(0.437) 0.441 0.432

3	(0.211) 0.208 0.217

4	(0.352) 0.351 0.350

Locus 20379 :

2 alleles

99.2% missing data

3	(0.719) 0.721 0.718

2	(0.281) 0.279 0.282

Locus 20380 :

2 alleles

98.8% missing data

3	(0.388) 0.389 0.392

1	(0.612) 0.611 0.608

Locus 20381 :

2 alleles

99.2% missing data

2	(0.270) 0.278 0.261

4	(0.730) 0.722 0.739

Locus 20382 :

2 alleles

99.2% missing data

2	(0.646) 0.644 0.648

4	(0.354) 0.356 0.352

Locus 20383 :

2 alleles

97.7% missing data

1	(0.107) 0.107 0.109

2	(0.893) 0.893 0.891

Locus 20384 :

2 alleles

99.2% missing data

2	(0.434) 0.439 0.428

4	(0.566) 0.561 0.572

Locus 20385 :

2 alleles

99.2% missing data

2	(0.506) 0.504 0.504

4	(0.494) 0.496 0.496

Locus 20386 :

2 alleles

99.2% missing data

2	(0.542) 0.543 0.540

1	(0.458) 0.457 0.460

Locus 20387 :

2 alleles

99.2% missing data

4	(0.715) 0.713 0.719

1	(0.285) 0.287 0.281

Locus 20388 :

2 alleles

99.2% missing data

3	(0.395) 0.385 0.395

4	(0.605) 0.615 0.605

Locus 20389 :

2 alleles

99.2% missing data

4	(0.416) 0.414 0.419

2	(0.584) 0.586 0.581

Locus 20390 :

2 alleles

99.2% missing data

2	(0.442) 0.444 0.440

4	(0.558) 0.556 0.560

Locus 20391 :

2 alleles

99.2% missing data

1	(0.330) 0.326 0.332

2	(0.670) 0.674 0.668

Locus 20392 :

2 alleles

99.2% missing data

2	(0.567) 0.557 0.569

3	(0.433) 0.443 0.431

Locus 20393 :

2 alleles

99.2% missing data

3	(0.453) 0.452 0.453

2	(0.547) 0.548 0.547

Locus 20394 :

2 alleles

99.2% missing data

4	(0.401) 0.397 0.404

1	(0.599) 0.603 0.596

Locus 20395 :

2 alleles

99.2% missing data

1	(0.254) 0.248 0.260

2	(0.746) 0.752 0.740

Locus 20396 :

2 alleles

97.7% missing data

1	(0.254) 0.258 0.240

4	(0.746) 0.742 0.760

Locus 20397 :

2 alleles

93.0% missing data

4	(0.269) 0.265 0.269

1	(0.731) 0.735 0.731

Locus 20398 :

2 alleles

98.8% missing data

2	(0.610) 0.601 0.622

1	(0.390) 0.399 0.378

Locus 20399 :

2 alleles

0.4% missing data

2	(0.989) 0.985 0.997

3	(0.011) 0.015 0.003

Locus 20400 :

2 alleles

99.2% missing data

2	(0.211) 0.210 0.219

3	(0.789) 0.790 0.781

Locus 20401 :

2 alleles

99.2% missing data

4	(0.540) 0.545 0.540

1	(0.460) 0.455 0.460

Locus 20402 :

2 alleles

98.4% missing data

1	(0.829) 0.827 0.830

3	(0.171) 0.173 0.170

Locus 20403 :

2 alleles

99.2% missing data

2	(0.677) 0.672 0.676

1	(0.323) 0.328 0.324

Locus 20404 :

2 alleles

92.6% missing data

1	(0.898) 0.892 0.912

2	(0.102) 0.108 0.088

Locus 20405 :

2 alleles

97.7% missing data

2	(0.229) 0.229 0.221

1	(0.771) 0.771 0.779

Locus 20406 :

2 alleles

98.8% missing data

2	(0.870) 0.875 0.869

3	(0.130) 0.125 0.131

Locus 20407 :

2 alleles

0.4% missing data

2	(0.980) 0.985 0.988

3	(0.020) 0.015 0.012

Locus 20408 :

2 alleles

99.2% missing data

3	(0.318) 0.314 0.319

2	(0.682) 0.686 0.681

Locus 20409 :

2 alleles

96.1% missing data

4	(0.794) 0.799 0.796

2	(0.206) 0.201 0.204

Locus 20410 :

2 alleles

98.4% missing data

2	(0.831) 0.828 0.833

1	(0.169) 0.172 0.167

Locus 20411 :

2 alleles

99.2% missing data

4	(0.313) 0.313 0.316

2	(0.687) 0.687 0.684

Locus 20412 :

2 alleles

99.2% missing data

2	(0.782) 0.781 0.772

1	(0.218) 0.219 0.228

Locus 20413 :

2 alleles

99.2% missing data

2	(0.499) 0.502 0.497

3	(0.501) 0.498 0.503

Locus 20414 :

2 alleles

0.0% missing data

3	(0.985) 0.989 0.993

2	(0.015) 0.011 0.007

Locus 20415 :

2 alleles

99.2% missing data

2	(0.758) 0.763 0.758

3	(0.242) 0.237 0.242

Locus 20416 :

2 alleles

96.1% missing data

1	(0.119) 0.108 0.121

2	(0.881) 0.892 0.879

Locus 20417 :

2 alleles

99.2% missing data

2	(0.486) 0.485 0.479

3	(0.514) 0.515 0.521

Locus 20418 :

2 alleles

99.2% missing data

4	(0.550) 0.550 0.550

2	(0.450) 0.450 0.450

Locus 20419 :

2 alleles

91.4% missing data

3	(0.913) 0.917 0.916

1	(0.087) 0.083 0.084

Locus 20420 :

3 alleles

98.0% missing data

4	(0.557) 0.563 0.556

3	(0.323) 0.324 0.315

1	(0.120) 0.113 0.129

Locus 20421 :

2 alleles

99.2% missing data

3	(0.556) 0.557 0.558

2	(0.444) 0.443 0.442

Locus 20422 :

3 alleles

92.2% missing data

4	(0.685) 0.689 0.692

2	(0.078) 0.076 0.073

1	(0.237) 0.236 0.235

Locus 20423 :

2 alleles

93.0% missing data

1	(0.913) 0.913 0.918

4	(0.087) 0.087 0.082

Locus 20424 :

2 alleles

99.2% missing data

1	(0.453) 0.451 0.452

2	(0.547) 0.549 0.548

Locus 20425 :

2 alleles

99.2% missing data

3	(0.255) 0.257 0.259

2	(0.745) 0.743 0.741

Locus 20426 :

2 alleles

99.2% missing data

4	(0.438) 0.436 0.437

1	(0.562) 0.564 0.563

Locus 20427 :

2 alleles

91.0% missing data

4	(0.906) 0.918 0.900

2	(0.094) 0.082 0.100

Locus 20428 :

2 alleles

99.2% missing data

2	(0.265) 0.256 0.273

1	(0.735) 0.744 0.727

Locus 20429 :

2 alleles

99.2% missing data

1	(0.544) 0.547 0.531

4	(0.456) 0.453 0.469

Locus 20430 :

2 alleles

99.2% missing data

3	(0.380) 0.380 0.386

1	(0.620) 0.620 0.614

Locus 20431 :

2 alleles

99.2% missing data

4	(0.761) 0.767 0.759

3	(0.239) 0.233 0.241

Locus 20432 :

2 alleles

98.8% missing data

3	(0.466) 0.468 0.466

4	(0.534) 0.532 0.534

Locus 20433 :

2 alleles

97.7% missing data

2	(0.597) 0.590 0.610

1	(0.403) 0.410 0.390

Locus 20434 :

2 alleles

99.2% missing data

1	(0.711) 0.704 0.712

3	(0.289) 0.296 0.288

Locus 20435 :

2 alleles

98.8% missing data

4	(0.724) 0.719 0.728

1	(0.276) 0.281 0.272

Locus 20436 :

2 alleles

99.2% missing data

4	(0.627) 0.630 0.617

2	(0.373) 0.370 0.383

Locus 20437 :

2 alleles

91.0% missing data

3	(0.923) 0.937 0.914

2	(0.077) 0.063 0.086

Locus 20438 :

2 alleles

98.4% missing data

2	(0.718) 0.726 0.713

1	(0.282) 0.274 0.287

Locus 20439 :

2 alleles

99.2% missing data

1	(0.467) 0.470 0.457

3	(0.533) 0.530 0.543

Locus 20440 :

2 alleles

98.8% missing data

2	(0.729) 0.732 0.729

1	(0.271) 0.268 0.271

Locus 20441 :

2 alleles

99.2% missing data

3	(0.717) 0.715 0.726

4	(0.283) 0.285 0.274

Locus 20442 :

2 alleles

99.2% missing data

2	(0.503) 0.501 0.503

4	(0.497) 0.499 0.497

Locus 20443 :

2 alleles

99.2% missing data

3	(0.400) 0.398 0.411

2	(0.600) 0.602 0.589

Locus 20444 :

2 alleles

98.0% missing data

4	(0.665) 0.669 0.663

3	(0.335) 0.331 0.337

Locus 20445 :

2 alleles

93.4% missing data

2	(0.923) 0.913 0.933

1	(0.077) 0.087 0.067

Locus 20446 :

2 alleles

99.2% missing data

2	(0.445) 0.437 0.450

3	(0.555) 0.563 0.550

Locus 20447 :

2 alleles

99.2% missing data

3	(0.374) 0.371 0.375

2	(0.626) 0.629 0.625

Locus 20448 :

2 alleles

99.2% missing data

3	(0.685) 0.690 0.675

4	(0.315) 0.310 0.325

Locus 20449 :

2 alleles

99.2% missing data

4	(0.323) 0.321 0.328

3	(0.677) 0.679 0.672

Locus 20450 :

2 alleles

99.2% missing data

2	(0.672) 0.673 0.679

4	(0.328) 0.327 0.321

Locus 20451 :

2 alleles

99.2% missing data

2	(0.615) 0.618 0.613

3	(0.385) 0.382 0.387

Locus 20452 :

2 alleles

97.7% missing data

2	(0.643) 0.657 0.642

3	(0.357) 0.343 0.358

Locus 20453 :

2 alleles

99.2% missing data

3	(0.538) 0.536 0.539

4	(0.462) 0.464 0.461

Locus 20454 :

2 alleles

99.2% missing data

2	(0.305) 0.299 0.308

4	(0.695) 0.701 0.692

Locus 20455 :

2 alleles

90.6% missing data

3	(0.913) 0.929 0.906

4	(0.087) 0.071 0.094

Locus 20456 :

2 alleles

99.2% missing data

4	(0.361) 0.359 0.359

1	(0.639) 0.641 0.641

Locus 20457 :

2 alleles

98.8% missing data

4	(0.730) 0.723 0.743

3	(0.270) 0.277 0.257

Locus 20458 :

2 alleles

99.2% missing data

4	(0.581) 0.579 0.584

1	(0.419) 0.421 0.416

Locus 20459 :

2 alleles

99.2% missing data

1	(0.533) 0.537 0.531

4	(0.467) 0.463 0.469

Locus 20460 :

2 alleles

99.2% missing data

1	(0.423) 0.423 0.428

2	(0.577) 0.577 0.572

Locus 20461 :

2 alleles

99.2% missing data

2	(0.451) 0.456 0.450

1	(0.549) 0.544 0.550

Locus 20462 :

2 alleles

99.2% missing data

4	(0.542) 0.534 0.547

1	(0.458) 0.466 0.453

Locus 20463 :

2 alleles

97.7% missing data

4	(0.263) 0.269 0.254

3	(0.737) 0.731 0.746

Locus 20464 :

2 alleles

99.2% missing data

2	(0.702) 0.698 0.706

4	(0.298) 0.302 0.294

Locus 20465 :

2 alleles

99.2% missing data

4	(0.391) 0.389 0.390

1	(0.609) 0.611 0.610

Locus 20466 :

2 alleles

99.2% missing data

2	(0.438) 0.437 0.445

1	(0.562) 0.563 0.555

Locus 20467 :

2 alleles

99.2% missing data

4	(0.749) 0.750 0.746

1	(0.251) 0.250 0.254

Locus 20468 :

2 alleles

99.2% missing data

1	(0.362) 0.357 0.369

2	(0.638) 0.643 0.631

Locus 20469 :

2 alleles

99.2% missing data

3	(0.712) 0.714 0.710

1	(0.288) 0.286 0.290

Locus 20470 :

2 alleles

92.2% missing data

2	(0.937) 0.940 0.937

1	(0.063) 0.060 0.063

Locus 20471 :

2 alleles

99.2% missing data

2	(0.548) 0.552 0.546

1	(0.452) 0.448 0.454

Locus 20472 :

2 alleles

97.7% missing data

2	(0.135) 0.129 0.138

3	(0.865) 0.871 0.862

Locus 20473 :

2 alleles

99.2% missing data

2	(0.353) 0.347 0.357

3	(0.647) 0.653 0.643

Locus 20474 :

2 alleles

99.2% missing data

2	(0.300) 0.296 0.296

1	(0.700) 0.704 0.704

Locus 20475 :

2 alleles

98.4% missing data

2	(0.819) 0.826 0.815

3	(0.181) 0.174 0.185

Locus 20476 :

2 alleles

98.8% missing data

4	(0.622) 0.619 0.619

2	(0.378) 0.381 0.381

Locus 20477 :

2 alleles

99.2% missing data

1	(0.515) 0.508 0.518

4	(0.485) 0.492 0.482

Locus 20478 :

2 alleles

99.2% missing data

4	(0.285) 0.279 0.294

1	(0.715) 0.721 0.706

Locus 20479 :

2 alleles

99.2% missing data

3	(0.264) 0.262 0.266

4	(0.736) 0.738 0.734

Locus 20480 :

2 alleles

99.2% missing data

3	(0.433) 0.436 0.433

1	(0.567) 0.564 0.567

Locus 20481 :

2 alleles

99.2% missing data

3	(0.301) 0.312 0.300

4	(0.699) 0.688 0.700

Locus 20482 :

2 alleles

98.8% missing data

1	(0.677) 0.672 0.685

4	(0.323) 0.328 0.315

Locus 20483 :

2 alleles

99.2% missing data

2	(0.278) 0.285 0.277

1	(0.722) 0.715 0.723

Locus 20484 :

2 alleles

99.2% missing data

1	(0.503) 0.504 0.503

2	(0.497) 0.496 0.497

Locus 20485 :

2 alleles

98.8% missing data

3	(0.476) 0.471 0.476

1	(0.524) 0.529 0.524

Locus 20486 :

2 alleles

99.2% missing data

4	(0.247) 0.248 0.251

3	(0.753) 0.752 0.749

Locus 20487 :

3 alleles

98.8% missing data

3	(0.270) 0.273 0.267

1	(0.361) 0.362 0.362

4	(0.369) 0.365 0.371

Locus 20488 :

2 alleles

99.2% missing data

1	(0.479) 0.478 0.477

3	(0.521) 0.522 0.523

Locus 20489 :

2 alleles

99.2% missing data

3	(0.256) 0.255 0.262

2	(0.744) 0.745 0.738

Locus 20490 :

2 alleles

91.0% missing data

3	(0.911) 0.926 0.900

1	(0.089) 0.074 0.100

Locus 20491 :

2 alleles

99.2% missing data

1	(0.115) 0.117 0.119

3	(0.885) 0.883 0.881

Locus 20492 :

2 alleles

96.1% missing data

2	(0.877) 0.883 0.872

4	(0.123) 0.117 0.128

Locus 20493 :

2 alleles

91.0% missing data

4	(0.925) 0.941 0.918

2	(0.075) 0.059 0.082

Locus 20494 :

2 alleles

97.7% missing data

1	(0.299) 0.298 0.291

4	(0.701) 0.702 0.709

Locus 20495 :

2 alleles

98.4% missing data

1	(0.698) 0.696 0.706

2	(0.302) 0.304 0.294

Locus 20496 :

2 alleles

99.2% missing data

1	(0.537) 0.537 0.529

4	(0.463) 0.463 0.471

Locus 20497 :

2 alleles

99.2% missing data

2	(0.351) 0.349 0.354

1	(0.649) 0.651 0.646

Locus 20498 :

2 alleles

99.2% missing data

4	(0.629) 0.629 0.633

3	(0.371) 0.371 0.367

Locus 20499 :

2 alleles

97.7% missing data

3	(0.727) 0.735 0.734

1	(0.273) 0.265 0.266

Locus 20500 :

2 alleles

8.2% missing data

1	(0.985) 0.978 0.995

2	(0.015) 0.022 0.005

Locus 20501 :

2 alleles

91.0% missing data

2	(0.956) 0.968 0.946

4	(0.044) 0.032 0.054

Locus 20502 :

2 alleles

99.2% missing data

2	(0.407) 0.404 0.417

1	(0.593) 0.596 0.583

Locus 20503 :

2 alleles

99.2% missing data

3	(0.497) 0.498 0.495

2	(0.503) 0.502 0.505

Locus 20504 :

2 alleles

99.2% missing data

4	(0.550) 0.551 0.546

3	(0.450) 0.449 0.454

Locus 20505 :

2 alleles

98.0% missing data

1	(0.604) 0.594 0.609

4	(0.396) 0.406 0.391

Locus 20506 :

2 alleles

98.8% missing data

4	(0.493) 0.488 0.496

2	(0.507) 0.512 0.504

Locus 20507 :

2 alleles

91.4% missing data

4	(0.902) 0.897 0.916

1	(0.098) 0.103 0.084

Locus 20508 :

2 alleles

98.8% missing data

3	(0.235) 0.228 0.241

4	(0.765) 0.772 0.759

Locus 20509 :

2 alleles

99.2% missing data

1	(0.433) 0.440 0.429

2	(0.567) 0.560 0.571

Locus 20510 :

2 alleles

98.8% missing data

3	(0.583) 0.582 0.587

4	(0.417) 0.418 0.413

Locus 20511 :

2 alleles

96.5% missing data

3	(0.221) 0.221 0.213

1	(0.779) 0.779 0.787

Locus 20512 :

2 alleles

89.8% missing data

4	(0.920) 0.938 0.903

3	(0.080) 0.062 0.097

Locus 20513 :

2 alleles

98.8% missing data

1	(0.415) 0.414 0.416

2	(0.585) 0.586 0.584

Locus 20514 :

2 alleles

98.8% missing data

1	(0.643) 0.646 0.636

2	(0.357) 0.354 0.364

Locus 20515 :

2 alleles

97.7% missing data

1	(0.732) 0.732 0.741

4	(0.268) 0.268 0.259

Locus 20516 :

2 alleles

98.8% missing data

4	(0.392) 0.384 0.398

2	(0.608) 0.616 0.602

Locus 20517 :

2 alleles

99.2% missing data

1	(0.689) 0.684 0.689

4	(0.311) 0.316 0.311

Locus 20518 :

2 alleles

99.2% missing data

2	(0.376) 0.375 0.386

4	(0.624) 0.625 0.614

Locus 20519 :

2 alleles

96.1% missing data

3	(0.190) 0.184 0.187

4	(0.810) 0.816 0.813

Locus 20520 :

2 alleles

98.8% missing data

4	(0.572) 0.582 0.570

1	(0.428) 0.418 0.430

Locus 20521 :

2 alleles

99.2% missing data

4	(0.601) 0.604 0.600

1	(0.399) 0.396 0.400

Locus 20522 :

2 alleles

98.8% missing data

4	(0.667) 0.654 0.665

1	(0.333) 0.346 0.335

Locus 20523 :

2 alleles

99.2% missing data

4	(0.323) 0.313 0.326

1	(0.677) 0.687 0.674

Locus 20524 :

2 alleles

98.4% missing data

3	(0.184) 0.180 0.193

4	(0.816) 0.820 0.807

Locus 20525 :

2 alleles

99.2% missing data

3	(0.665) 0.670 0.657

4	(0.335) 0.330 0.343

Locus 20526 :

2 alleles

98.0% missing data

4	(0.300) 0.297 0.297

2	(0.700) 0.703 0.703

Locus 20527 :

2 alleles

99.2% missing data

4	(0.692) 0.694 0.693

2	(0.308) 0.306 0.307

Locus 20528 :

2 alleles

99.2% missing data

2	(0.841) 0.841 0.835

1	(0.159) 0.159 0.165

Locus 20529 :

2 alleles

97.3% missing data

4	(0.764) 0.771 0.767

2	(0.236) 0.229 0.233

Locus 20530 :

3 alleles

98.8% missing data

1	(0.306) 0.300 0.308

3	(0.533) 0.538 0.525

2	(0.162) 0.162 0.166

Locus 20531 :

2 alleles

99.2% missing data

1	(0.431) 0.429 0.438

3	(0.569) 0.571 0.562

Locus 20532 :

2 alleles

96.9% missing data

2	(0.781) 0.776 0.793

1	(0.219) 0.224 0.207

Locus 20533 :

2 alleles

91.8% missing data

4	(0.559) 0.558 0.568

1	(0.441) 0.442 0.432

Locus 20534 :

2 alleles

99.2% missing data

4	(0.380) 0.378 0.384

3	(0.620) 0.622 0.616

Locus 20535 :

2 alleles

99.2% missing data

3	(0.384) 0.388 0.381

4	(0.616) 0.612 0.619

Locus 20536 :

2 alleles

99.2% missing data

4	(0.292) 0.291 0.295

3	(0.708) 0.709 0.705

Locus 20537 :

2 alleles

99.2% missing data

4	(0.333) 0.328 0.336

1	(0.667) 0.672 0.664

Locus 20538 :

2 alleles

98.4% missing data

3	(0.779) 0.782 0.775

4	(0.221) 0.218 0.225

Locus 20539 :

2 alleles

99.2% missing data

2	(0.430) 0.432 0.427

4	(0.570) 0.568 0.573

Locus 20540 :

2 alleles

99.2% missing data

4	(0.788) 0.779 0.789

3	(0.212) 0.221 0.211

Locus 20541 :

2 alleles

99.2% missing data

4	(0.690) 0.685 0.688

2	(0.310) 0.315 0.312

Locus 20542 :

2 alleles

97.3% missing data

1	(0.777) 0.779 0.778

2	(0.223) 0.221 0.222

Locus 20543 :

2 alleles

97.7% missing data

3	(0.758) 0.763 0.758

1	(0.242) 0.237 0.242

Locus 20544 :

2 alleles

99.2% missing data

2	(0.339) 0.335 0.342

3	(0.661) 0.665 0.658

Locus 20545 :

3 alleles

98.4% missing data

3	(0.426) 0.424 0.426

1	(0.239) 0.242 0.239

4	(0.335) 0.334 0.335

Locus 20546 :

2 alleles

99.2% missing data

1	(0.499) 0.498 0.495

4	(0.501) 0.502 0.505

Locus 20547 :

2 alleles

99.2% missing data

1	(0.279) 0.277 0.287

3	(0.721) 0.723 0.713

Locus 20548 :

2 alleles

99.2% missing data

4	(0.507) 0.504 0.515

1	(0.493) 0.496 0.485

Locus 20549 :

2 alleles

99.2% missing data

3	(0.544) 0.541 0.547

1	(0.456) 0.459 0.453

Locus 20550 :

2 alleles

96.5% missing data

1	(0.806) 0.810 0.806

4	(0.194) 0.190 0.194

Locus 20551 :

2 alleles

97.7% missing data

4	(0.439) 0.431 0.449

3	(0.561) 0.569 0.551

Locus 20552 :

2 alleles

98.4% missing data

4	(0.471) 0.464 0.476

2	(0.529) 0.536 0.524

Locus 20553 :

2 alleles

97.7% missing data

4	(0.824) 0.818 0.835

1	(0.176) 0.182 0.165

Locus 20554 :

2 alleles

99.2% missing data

2	(0.372) 0.363 0.376

3	(0.628) 0.637 0.624

Locus 20555 :

2 alleles

98.0% missing data

1	(0.854) 0.849 0.858

2	(0.146) 0.151 0.142

Locus 20556 :

2 alleles

99.2% missing data

3	(0.307) 0.296 0.310

1	(0.693) 0.704 0.690

Locus 20557 :

2 alleles

99.2% missing data

3	(0.290) 0.288 0.292

4	(0.710) 0.712 0.708

Locus 20558 :

2 alleles

99.2% missing data

4	(0.284) 0.278 0.289

3	(0.716) 0.722 0.711

Locus 20559 :

2 alleles

98.8% missing data

3	(0.608) 0.614 0.604

2	(0.392) 0.386 0.396

Locus 20560 :

2 alleles

93.8% missing data

4	(0.866) 0.869 0.880

1	(0.134) 0.131 0.120

Locus 20561 :

2 alleles

99.2% missing data

2	(0.619) 0.618 0.613

1	(0.381) 0.382 0.387

Locus 20562 :

2 alleles

99.2% missing data

2	(0.663) 0.664 0.665

4	(0.337) 0.336 0.335

Locus 20563 :

2 alleles

98.0% missing data

4	(0.802) 0.797 0.815

1	(0.198) 0.203 0.185

Locus 20564 :

2 alleles

84.4% missing data

1	(0.484) 0.547 0.426

4	(0.516) 0.453 0.574

Locus 20565 :

3 alleles

96.9% missing data

1	(0.656) 0.655 0.668

2	(0.213) 0.216 0.200

3	(0.131) 0.129 0.132

Locus 20566 :

2 alleles

96.9% missing data

4	(0.805) 0.808 0.810

1	(0.195) 0.192 0.190

Locus 20567 :

2 alleles

99.2% missing data

3	(0.572) 0.572 0.564

2	(0.428) 0.428 0.436

Locus 20568 :

2 alleles

99.2% missing data

2	(0.393) 0.388 0.402

3	(0.607) 0.612 0.598

Locus 20569 :

2 alleles

99.2% missing data

2	(0.480) 0.486 0.476

4	(0.520) 0.514 0.524

Locus 20570 :

2 alleles

99.2% missing data

4	(0.443) 0.448 0.450

3	(0.557) 0.552 0.550

Locus 20571 :

2 alleles

99.2% missing data

4	(0.645) 0.644 0.646

2	(0.355) 0.356 0.354

Locus 20572 :

2 alleles

99.2% missing data

2	(0.291) 0.292 0.291

4	(0.709) 0.708 0.709

Locus 20573 :

2 alleles

8.2% missing data

4	(0.983) 0.975 0.995

3	(0.017) 0.025 0.005

Locus 20574 :

2 alleles

92.2% missing data

1	(0.877) 0.879 0.881

3	(0.123) 0.121 0.119

Locus 20575 :

2 alleles

99.2% missing data

3	(0.610) 0.605 0.610

2	(0.390) 0.395 0.390

Locus 20576 :

2 alleles

98.8% missing data

3	(0.451) 0.449 0.448

4	(0.549) 0.551 0.552

Locus 20577 :

2 alleles

98.8% missing data

2	(0.387) 0.386 0.392

1	(0.613) 0.614 0.608

Locus 20578 :

2 alleles

98.8% missing data

4	(0.736) 0.731 0.737

3	(0.264) 0.269 0.263

Locus 20579 :

2 alleles

99.2% missing data

4	(0.323) 0.320 0.326

3	(0.677) 0.680 0.674

Locus 20580 :

3 alleles

98.8% missing data

4	(0.258) 0.262 0.255

1	(0.339) 0.346 0.335

3	(0.404) 0.392 0.410

Locus 20581 :

2 alleles

97.7% missing data

1	(0.164) 0.163 0.160

4	(0.836) 0.837 0.840

Locus 20582 :

2 alleles

99.2% missing data

4	(0.593) 0.593 0.596

1	(0.407) 0.407 0.404

Locus 20583 :

2 alleles

99.2% missing data

1	(0.324) 0.326 0.321

4	(0.676) 0.674 0.679

Locus 20584 :

2 alleles

99.2% missing data

3	(0.473) 0.473 0.473

1	(0.527) 0.527 0.527

Locus 20585 :

2 alleles

97.3% missing data

3	(0.844) 0.836 0.848

2	(0.156) 0.164 0.152

Locus 20586 :

2 alleles

97.7% missing data

4	(0.189) 0.181 0.195

3	(0.811) 0.819 0.805

Locus 20587 :

3 alleles

98.8% missing data

2	(0.182) 0.175 0.181

4	(0.584) 0.584 0.587

3	(0.234) 0.241 0.232

Locus 20588 :

2 alleles

97.7% missing data

2	(0.245) 0.245 0.243

4	(0.755) 0.755 0.757

Locus 20589 :

2 alleles

99.2% missing data

2	(0.314) 0.317 0.323

3	(0.686) 0.683 0.677

Locus 20590 :

2 alleles

99.2% missing data

4	(0.502) 0.502 0.500

3	(0.498) 0.498 0.500

Locus 20591 :

3 alleles

98.8% missing data

3	(0.434) 0.435 0.429

2	(0.190) 0.189 0.192

4	(0.377) 0.376 0.379

Locus 20592 :

2 alleles

99.2% missing data

2	(0.365) 0.362 0.367

3	(0.635) 0.638 0.633

Locus 20593 :

2 alleles

99.2% missing data

1	(0.654) 0.654 0.648

3	(0.346) 0.346 0.352

Locus 20594 :

2 alleles

98.4% missing data

1	(0.548) 0.552 0.556

2	(0.452) 0.448 0.444

Locus 20595 :

2 alleles

99.2% missing data

4	(0.490) 0.493 0.494

2	(0.510) 0.507 0.506

Locus 20596 :

2 alleles

99.2% missing data

1	(0.321) 0.322 0.325

4	(0.679) 0.678 0.675

Locus 20597 :

2 alleles

99.2% missing data

3	(0.227) 0.227 0.233

4	(0.773) 0.773 0.767

Locus 20598 :

2 alleles

99.2% missing data

2	(0.236) 0.233 0.244

4	(0.764) 0.767 0.756

Locus 20599 :

2 alleles

98.8% missing data

4	(0.469) 0.467 0.462

1	(0.531) 0.533 0.538

Locus 20600 :

2 alleles

96.1% missing data

4	(0.839) 0.849 0.834

3	(0.161) 0.151 0.166

Locus 20601 :

2 alleles

48.8% missing data

4	(0.978) 0.982 0.985

1	(0.022) 0.018 0.015

Locus 20602 :

2 alleles

99.2% missing data

3	(0.435) 0.436 0.433

1	(0.565) 0.564 0.567

Locus 20603 :

2 alleles

99.2% missing data

3	(0.809) 0.804 0.816

1	(0.191) 0.196 0.184

Locus 20604 :

2 alleles

99.2% missing data

4	(0.539) 0.545 0.535

3	(0.461) 0.455 0.465

Locus 20605 :

2 alleles

99.2% missing data

3	(0.258) 0.259 0.264

2	(0.742) 0.741 0.736

Locus 20606 :

2 alleles

97.7% missing data

1	(0.733) 0.735 0.734

4	(0.267) 0.265 0.266

Locus 20607 :

2 alleles

99.2% missing data

3	(0.381) 0.377 0.384

4	(0.619) 0.623 0.616

Locus 20608 :

2 alleles

95.7% missing data

3	(0.441) 0.435 0.449

1	(0.559) 0.565 0.551

Locus 20609 :

2 alleles

98.8% missing data

2	(0.594) 0.590 0.599

3	(0.406) 0.410 0.401

Locus 20610 :

2 alleles

98.8% missing data

4	(0.364) 0.368 0.358

1	(0.636) 0.632 0.642

Locus 20611 :

2 alleles

98.4% missing data

4	(0.682) 0.691 0.675

1	(0.318) 0.309 0.325

Locus 20612 :

2 alleles

99.2% missing data

1	(0.180) 0.179 0.185

4	(0.820) 0.821 0.815

Locus 20613 :

2 alleles

98.0% missing data

2	(0.764) 0.758 0.772

3	(0.236) 0.242 0.228

Locus 20614 :

2 alleles

99.2% missing data

1	(0.512) 0.507 0.514

2	(0.488) 0.493 0.486

Locus 20615 :

2 alleles

99.2% missing data

2	(0.384) 0.381 0.385

4	(0.616) 0.619 0.615

Locus 20616 :

2 alleles

99.2% missing data

4	(0.634) 0.637 0.632

1	(0.366) 0.363 0.368

Locus 20617 :

2 alleles

99.2% missing data

3	(0.711) 0.706 0.714

1	(0.289) 0.294 0.286

Locus 20618 :

2 alleles

98.4% missing data

2	(0.308) 0.309 0.310

3	(0.692) 0.691 0.690

Locus 20619 :

2 alleles

99.2% missing data

1	(0.671) 0.671 0.673

3	(0.329) 0.329 0.327

Locus 20620 :

2 alleles

99.2% missing data

1	(0.669) 0.671 0.673

4	(0.331) 0.329 0.327

Locus 20621 :

2 alleles

95.7% missing data

2	(0.239) 0.242 0.226

3	(0.761) 0.758 0.774

Locus 20622 :

2 alleles

99.2% missing data

3	(0.475) 0.474 0.479

4	(0.525) 0.526 0.521

Locus 20623 :

2 alleles

98.8% missing data

1	(0.341) 0.339 0.340

3	(0.659) 0.661 0.660

Locus 20624 :

2 alleles

96.5% missing data

4	(0.577) 0.568 0.593

3	(0.423) 0.432 0.407

Locus 20625 :

2 alleles

96.9% missing data

1	(0.160) 0.156 0.165

3	(0.840) 0.844 0.835

Locus 20626 :

2 alleles

90.6% missing data

2	(0.934) 0.946 0.923

1	(0.066) 0.054 0.077

Locus 20627 :

2 alleles

98.0% missing data

1	(0.651) 0.641 0.661

4	(0.349) 0.359 0.339

Locus 20628 :

2 alleles

99.2% missing data

1	(0.386) 0.389 0.384

3	(0.614) 0.611 0.616

Locus 20629 :

3 alleles

97.3% missing data

4	(0.205) 0.204 0.202

1	(0.612) 0.599 0.625

2	(0.183) 0.197 0.173

Locus 20630 :

2 alleles

98.8% missing data

1	(0.684) 0.683 0.688

4	(0.316) 0.317 0.312

Locus 20631 :

3 alleles

95.3% missing data

1	(0.189) 0.175 0.195

3	(0.678) 0.698 0.671

4	(0.133) 0.126 0.134

Locus 20632 :

2 alleles

98.8% missing data

4	(0.418) 0.410 0.418

3	(0.582) 0.590 0.582

Locus 20633 :

2 alleles

99.2% missing data

1	(0.465) 0.462 0.466

4	(0.535) 0.538 0.534

Locus 20634 :

2 alleles

96.1% missing data

3	(0.750) 0.765 0.743

4	(0.250) 0.235 0.257

Locus 20635 :

2 alleles

96.1% missing data

4	(0.885) 0.894 0.877

3	(0.115) 0.106 0.123

Locus 20636 :

2 alleles

99.2% missing data

4	(0.420) 0.415 0.423

2	(0.580) 0.585 0.577

Locus 20637 :

2 alleles

99.2% missing data

1	(0.543) 0.548 0.539

4	(0.457) 0.452 0.461

Locus 20638 :

2 alleles

99.2% missing data

3	(0.722) 0.718 0.737

2	(0.278) 0.282 0.263

Locus 20639 :

2 alleles

99.2% missing data

1	(0.524) 0.516 0.529

2	(0.476) 0.484 0.471

Locus 20640 :

2 alleles

99.2% missing data

2	(0.710) 0.710 0.706

1	(0.290) 0.290 0.294

Locus 20641 :

2 alleles

99.2% missing data

4	(0.563) 0.556 0.559

1	(0.437) 0.444 0.441

Locus 20642 :

2 alleles

99.2% missing data

1	(0.619) 0.612 0.626

3	(0.381) 0.388 0.374

Locus 20643 :

4 alleles

98.4% missing data

3	(0.196) 0.200 0.196

2	(0.095) 0.093 0.099

1	(0.443) 0.437 0.440

4	(0.266) 0.270 0.266

Locus 20644 :

2 alleles

99.2% missing data

3	(0.375) 0.370 0.383

4	(0.625) 0.630 0.617

Locus 20645 :

2 alleles

99.2% missing data

1	(0.600) 0.599 0.601

3	(0.400) 0.401 0.399

Locus 20646 :

2 alleles

99.2% missing data

4	(0.672) 0.672 0.665

1	(0.328) 0.328 0.335

Locus 20647 :

2 alleles

99.2% missing data

2	(0.328) 0.327 0.327

1	(0.672) 0.673 0.673

Locus 20648 :

2 alleles

98.8% missing data

1	(0.226) 0.222 0.227

4	(0.774) 0.778 0.773

Locus 20649 :

2 alleles

99.2% missing data

4	(0.369) 0.361 0.377

1	(0.631) 0.639 0.623

Locus 20650 :

2 alleles

98.0% missing data

4	(0.586) 0.593 0.590

1	(0.414) 0.407 0.410

Locus 20651 :

2 alleles

99.2% missing data

1	(0.361) 0.358 0.367

3	(0.639) 0.642 0.633

Locus 20652 :

2 alleles

99.2% missing data

4	(0.742) 0.737 0.741

2	(0.258) 0.263 0.259

Locus 20653 :

2 alleles

99.2% missing data

3	(0.235) 0.236 0.237

2	(0.765) 0.764 0.763

Locus 20654 :

2 alleles

99.2% missing data

1	(0.333) 0.338 0.330

3	(0.667) 0.662 0.670

Locus 20655 :

2 alleles

98.8% missing data

4	(0.374) 0.374 0.372

3	(0.626) 0.626 0.628

Locus 20656 :

2 alleles

99.2% missing data

1	(0.371) 0.367 0.378

3	(0.629) 0.633 0.622

Locus 20657 :

2 alleles

99.2% missing data

1	(0.642) 0.631 0.644

3	(0.358) 0.369 0.356

Locus 20658 :

2 alleles

99.2% missing data

4	(0.686) 0.692 0.682

1	(0.314) 0.308 0.318

Locus 20659 :

2 alleles

99.2% missing data

4	(0.475) 0.471 0.477

1	(0.525) 0.529 0.523

Locus 20660 :

2 alleles

99.2% missing data

4	(0.666) 0.667 0.665

1	(0.334) 0.333 0.335

Locus 20661 :

2 alleles

93.8% missing data

3	(0.807) 0.798 0.835

1	(0.193) 0.202 0.165

Locus 20662 :

2 alleles

99.2% missing data

3	(0.590) 0.587 0.593

1	(0.410) 0.413 0.407

Locus 20663 :

2 alleles

99.2% missing data

4	(0.493) 0.484 0.497

2	(0.507) 0.516 0.503

Locus 20664 :

2 alleles

99.2% missing data

3	(0.561) 0.564 0.563

2	(0.439) 0.436 0.437

Locus 20665 :

2 alleles

97.7% missing data

2	(0.742) 0.756 0.734

4	(0.258) 0.244 0.266

Locus 20666 :

2 alleles

99.2% missing data

2	(0.474) 0.472 0.473

3	(0.526) 0.528 0.527

Locus 20667 :

2 alleles

98.8% missing data

1	(0.481) 0.481 0.479

3	(0.519) 0.519 0.521

Locus 20668 :

2 alleles

97.7% missing data

3	(0.766) 0.763 0.770

4	(0.234) 0.237 0.230

Locus 20669 :

2 alleles

99.2% missing data

1	(0.477) 0.473 0.479

4	(0.523) 0.527 0.521

Locus 20670 :

2 alleles

98.0% missing data

4	(0.314) 0.296 0.319

3	(0.686) 0.704 0.681

Locus 20671 :

2 alleles

99.2% missing data

1	(0.745) 0.747 0.737

4	(0.255) 0.253 0.263

Locus 20672 :

2 alleles

98.8% missing data

3	(0.396) 0.391 0.395

4	(0.604) 0.609 0.605

Locus 20673 :

2 alleles

99.2% missing data

4	(0.746) 0.744 0.749

3	(0.254) 0.256 0.251

Locus 20674 :

2 alleles

98.8% missing data

4	(0.599) 0.599 0.606

1	(0.401) 0.401 0.394

Locus 20675 :

2 alleles

99.2% missing data

3	(0.756) 0.750 0.760

2	(0.244) 0.250 0.240

Locus 20676 :

2 alleles

99.2% missing data

4	(0.379) 0.379 0.379

1	(0.621) 0.621 0.621

Locus 20677 :

2 alleles

0.0% missing data

4	(0.984) 0.979 0.996

1	(0.016) 0.021 0.004

Locus 20678 :

2 alleles

98.8% missing data

1	(0.623) 0.627 0.624

4	(0.377) 0.373 0.376

Locus 20679 :

2 alleles

98.0% missing data

4	(0.329) 0.324 0.325

1	(0.671) 0.676 0.675

Locus 20680 :

2 alleles

99.2% missing data

3	(0.378) 0.376 0.383

1	(0.622) 0.624 0.617

Locus 20681 :

3 alleles

98.8% missing data

4	(0.480) 0.480 0.477

2	(0.240) 0.240 0.242

3	(0.280) 0.280 0.281

Locus 20682 :

2 alleles

97.7% missing data

4	(0.170) 0.170 0.169

1	(0.830) 0.830 0.831

Locus 20683 :

2 alleles

99.2% missing data

4	(0.530) 0.532 0.533

1	(0.470) 0.468 0.467

Locus 20684 :

2 alleles

99.2% missing data

3	(0.567) 0.574 0.560

4	(0.433) 0.426 0.440

Locus 20685 :

2 alleles

98.0% missing data

1	(0.709) 0.712 0.717

3	(0.291) 0.288 0.283

Locus 20686 :

2 alleles

99.2% missing data

1	(0.320) 0.321 0.323

4	(0.680) 0.679 0.677

Locus 20687 :

2 alleles

99.2% missing data

4	(0.275) 0.273 0.273

3	(0.725) 0.727 0.727

Locus 20688 :

2 alleles

99.2% missing data

1	(0.460) 0.462 0.451

2	(0.540) 0.538 0.549

Locus 20689 :

2 alleles

99.2% missing data

2	(0.748) 0.743 0.747

3	(0.252) 0.257 0.253

Locus 20690 :

2 alleles

14.1% missing data

4	(0.984) 0.984 0.993

1	(0.016) 0.016 0.007

Locus 20691 :

2 alleles

98.8% missing data

4	(0.544) 0.543 0.546

2	(0.456) 0.457 0.454

Locus 20692 :

2 alleles

99.2% missing data

3	(0.312) 0.304 0.323

4	(0.688) 0.696 0.677

Locus 20693 :

2 alleles

99.2% missing data

1	(0.465) 0.465 0.462

4	(0.535) 0.535 0.538

Locus 20694 :

2 alleles

99.2% missing data

3	(0.453) 0.449 0.450

2	(0.547) 0.551 0.550

Locus 20695 :

2 alleles

99.2% missing data

1	(0.628) 0.634 0.622

4	(0.372) 0.366 0.378

Locus 20696 :

2 alleles

96.1% missing data

4	(0.830) 0.842 0.827

1	(0.170) 0.158 0.173

Locus 20697 :

2 alleles

99.2% missing data

3	(0.212) 0.207 0.217

1	(0.788) 0.793 0.783

Locus 20698 :

2 alleles

99.2% missing data

4	(0.354) 0.351 0.358

2	(0.646) 0.649 0.642

Locus 20699 :

2 alleles

99.2% missing data

1	(0.513) 0.508 0.519

4	(0.487) 0.492 0.481

Locus 20700 :

2 alleles

99.2% missing data

3	(0.415) 0.417 0.411

4	(0.585) 0.583 0.589

Locus 20701 :

2 alleles

99.2% missing data

3	(0.523) 0.525 0.523

1	(0.477) 0.475 0.477

Locus 20702 :

3 alleles

90.6% missing data

4	(0.873) 0.891 0.867

2	(0.073) 0.066 0.073

3	(0.053) 0.043 0.060

Locus 20703 :

2 alleles

99.2% missing data

4	(0.573) 0.577 0.565

3	(0.427) 0.423 0.435

Locus 20704 :

2 alleles

91.0% missing data

2	(0.909) 0.926 0.901

3	(0.091) 0.074 0.099

Locus 20705 :

2 alleles

96.9% missing data

4	(0.208) 0.195 0.212

3	(0.792) 0.805 0.788

Locus 20706 :

3 alleles

98.8% missing data

4	(0.107) 0.107 0.106

2	(0.618) 0.622 0.611

1	(0.275) 0.271 0.283

Locus 20707 :

2 alleles

99.2% missing data

2	(0.214) 0.213 0.223

3	(0.786) 0.787 0.777

Locus 20708 :

2 alleles

0.0% missing data

1	(0.986) 0.983 0.996

4	(0.014) 0.017 0.004

Locus 20709 :

2 alleles

99.2% missing data

1	(0.516) 0.521 0.512

2	(0.484) 0.479 0.488

Locus 20710 :

2 alleles

99.2% missing data

4	(0.651) 0.648 0.657

2	(0.349) 0.352 0.343

Locus 20711 :

2 alleles

99.2% missing data

3	(0.554) 0.556 0.554

1	(0.446) 0.444 0.446

Locus 20712 :

2 alleles

95.3% missing data

1	(0.901) 0.902 0.903

4	(0.099) 0.098 0.097

Locus 20713 :

2 alleles

99.2% missing data

4	(0.499) 0.504 0.491

1	(0.501) 0.496 0.509

Locus 20714 :

3 alleles

96.1% missing data

1	(0.167) 0.162 0.168

3	(0.329) 0.349 0.317

2	(0.504) 0.489 0.515

Locus 20715 :

2 alleles

91.0% missing data

2	(0.918) 0.934 0.908

4	(0.082) 0.066 0.092

Locus 20716 :

3 alleles

98.8% missing data

2	(0.102) 0.101 0.108

1	(0.356) 0.366 0.353

3	(0.542) 0.534 0.539

Locus 20717 :

2 alleles

99.2% missing data

4	(0.470) 0.467 0.472

1	(0.530) 0.533 0.528

Locus 20718 :

2 alleles

99.2% missing data

4	(0.773) 0.765 0.777

3	(0.227) 0.235 0.223

Locus 20719 :

2 alleles

99.2% missing data

2	(0.530) 0.531 0.531

1	(0.470) 0.469 0.469

Locus 20720 :

2 alleles

99.2% missing data

2	(0.645) 0.640 0.652

1	(0.355) 0.360 0.348

Locus 20721 :

2 alleles

99.2% missing data

1	(0.266) 0.272 0.267

3	(0.734) 0.728 0.733

Locus 20722 :

2 alleles

99.2% missing data

4	(0.535) 0.535 0.531

2	(0.465) 0.465 0.469

Locus 20723 :

2 alleles

99.2% missing data

1	(0.655) 0.659 0.647

3	(0.345) 0.341 0.353

Locus 20724 :

2 alleles

99.2% missing data

4	(0.512) 0.515 0.512

3	(0.488) 0.485 0.488

Locus 20725 :

2 alleles

95.3% missing data

1	(0.346) 0.342 0.354

2	(0.654) 0.658 0.646

Locus 20726 :

2 alleles

99.2% missing data

1	(0.465) 0.466 0.463

4	(0.535) 0.534 0.537

Locus 20727 :

2 alleles

99.2% missing data

3	(0.380) 0.376 0.382

2	(0.620) 0.624 0.618

Locus 20728 :

2 alleles

99.2% missing data

1	(0.603) 0.600 0.608

4	(0.397) 0.400 0.392

Locus 20729 :

2 alleles

99.2% missing data

1	(0.582) 0.584 0.584

2	(0.418) 0.416 0.416

Locus 20730 :

2 alleles

8.2% missing data

4	(0.986) 0.980 0.996

1	(0.014) 0.020 0.004

Locus 20731 :

2 alleles

98.4% missing data

4	(0.778) 0.772 0.784

3	(0.222) 0.228 0.216

Locus 20732 :

2 alleles

0.4% missing data

3	(0.987) 0.991 0.993

2	(0.013) 0.009 0.007

Locus 20733 :

2 alleles

99.2% missing data

2	(0.344) 0.338 0.349

4	(0.656) 0.662 0.651

Locus 20734 :

3 alleles

98.8% missing data

1	(0.169) 0.175 0.166

4	(0.295) 0.301 0.293

3	(0.536) 0.524 0.541

Locus 20735 :

2 alleles

99.2% missing data

4	(0.685) 0.686 0.684

3	(0.315) 0.314 0.316

Locus 20736 :

2 alleles

98.8% missing data

1	(0.608) 0.608 0.609

4	(0.392) 0.392 0.391

Locus 20737 :

2 alleles

96.9% missing data

1	(0.816) 0.811 0.826

4	(0.184) 0.189 0.174

Locus 20738 :

2 alleles

99.2% missing data

4	(0.299) 0.293 0.304

1	(0.701) 0.707 0.696

Locus 20739 :

3 alleles

98.8% missing data

2	(0.252) 0.248 0.252

4	(0.155) 0.152 0.163

3	(0.593) 0.600 0.585

Locus 20740 :

2 alleles

98.8% missing data

2	(0.628) 0.631 0.629

4	(0.372) 0.369 0.371

Locus 20741 :

2 alleles

98.8% missing data

2	(0.483) 0.483 0.486

4	(0.517) 0.517 0.514

Locus 20742 :

2 alleles

99.2% missing data

4	(0.497) 0.500 0.495

2	(0.503) 0.500 0.505

Locus 20743 :

2 alleles

97.7% missing data

4	(0.172) 0.167 0.170

1	(0.828) 0.833 0.830

Locus 20744 :

3 alleles

98.8% missing data

1	(0.362) 0.358 0.370

2	(0.248) 0.241 0.249

4	(0.390) 0.400 0.381

Locus 20745 :

2 alleles

99.2% missing data

3	(0.823) 0.812 0.818

1	(0.177) 0.188 0.182

Locus 20746 :

2 alleles

99.2% missing data

1	(0.433) 0.437 0.428

2	(0.567) 0.563 0.572

Locus 20747 :

2 alleles

99.2% missing data

2	(0.551) 0.553 0.549

4	(0.449) 0.447 0.451

Locus 20748 :

2 alleles

99.2% missing data

2	(0.366) 0.365 0.375

4	(0.634) 0.635 0.625

Locus 20749 :

2 alleles

99.2% missing data

2	(0.488) 0.490 0.491

4	(0.512) 0.510 0.509

Locus 20750 :

2 alleles

98.8% missing data

4	(0.511) 0.503 0.522

2	(0.489) 0.497 0.478

Locus 20751 :

2 alleles

91.0% missing data

2	(0.890) 0.909 0.881

1	(0.110) 0.091 0.119

Locus 20752 :

2 alleles

99.2% missing data

3	(0.600) 0.598 0.605

2	(0.400) 0.402 0.395

Locus 20753 :

2 alleles

99.2% missing data

4	(0.372) 0.368 0.382

3	(0.628) 0.632 0.618

Locus 20754 :

2 alleles

96.9% missing data

4	(0.251) 0.255 0.241

3	(0.749) 0.745 0.759

Locus 20755 :

2 alleles

98.8% missing data

2	(0.188) 0.186 0.194

3	(0.812) 0.814 0.806

Locus 20756 :

2 alleles

99.2% missing data

3	(0.407) 0.401 0.410

4	(0.593) 0.599 0.590

Locus 20757 :

2 alleles

97.3% missing data

1	(0.400) 0.382 0.422

2	(0.600) 0.618 0.578

Locus 20758 :

2 alleles

99.2% missing data

2	(0.410) 0.411 0.409

4	(0.590) 0.589 0.591

Locus 20759 :

2 alleles

99.2% missing data

3	(0.427) 0.417 0.430

1	(0.573) 0.583 0.570

Locus 20760 :

2 alleles

99.2% missing data

4	(0.427) 0.433 0.423

3	(0.573) 0.567 0.577

Locus 20761 :

3 alleles

98.4% missing data

3	(0.613) 0.619 0.604

2	(0.211) 0.205 0.216

4	(0.175) 0.175 0.180

Locus 20762 :

2 alleles

99.2% missing data

4	(0.583) 0.586 0.579

1	(0.417) 0.414 0.421

Locus 20763 :

2 alleles

99.2% missing data

4	(0.657) 0.653 0.662

2	(0.343) 0.347 0.338

Locus 20764 :

2 alleles

99.2% missing data

4	(0.303) 0.304 0.304

2	(0.697) 0.696 0.696

Locus 20765 :

2 alleles

99.2% missing data

1	(0.390) 0.384 0.397

2	(0.610) 0.616 0.603

Locus 20766 :

2 alleles

99.2% missing data

4	(0.345) 0.350 0.352

3	(0.655) 0.650 0.648

Locus 20767 :

2 alleles

99.2% missing data

2	(0.473) 0.470 0.473

4	(0.527) 0.530 0.527

Locus 20768 :

2 alleles

99.2% missing data

1	(0.502) 0.504 0.496

4	(0.498) 0.496 0.504

Locus 20769 :

2 alleles

97.3% missing data

4	(0.777) 0.778 0.777

2	(0.223) 0.222 0.223

Locus 20770 :

2 alleles

99.2% missing data

3	(0.520) 0.522 0.517

4	(0.480) 0.478 0.483

Locus 20771 :

2 alleles

94.1% missing data

3	(0.870) 0.868 0.878

4	(0.130) 0.132 0.122

Locus 20772 :

3 alleles

96.5% missing data

1	(0.242) 0.242 0.245

3	(0.655) 0.666 0.645

2	(0.103) 0.092 0.110

Locus 20773 :

2 alleles

98.8% missing data

3	(0.670) 0.666 0.669

1	(0.330) 0.334 0.331

Locus 20774 :

2 alleles

99.2% missing data

1	(0.490) 0.488 0.497

2	(0.510) 0.512 0.503

Locus 20775 :

2 alleles

99.2% missing data

2	(0.491) 0.497 0.491

1	(0.509) 0.503 0.509

Locus 20776 :

2 alleles

98.4% missing data

3	(0.713) 0.724 0.709

1	(0.287) 0.276 0.291

Locus 20777 :

2 alleles

99.2% missing data

1	(0.195) 0.187 0.202

3	(0.805) 0.813 0.798

Locus 20778 :

2 alleles

97.3% missing data

1	(0.287) 0.290 0.281

2	(0.713) 0.710 0.719

Locus 20779 :

3 alleles

98.8% missing data

1	(0.346) 0.345 0.345

3	(0.430) 0.428 0.437

4	(0.224) 0.227 0.218

Locus 20780 :

2 alleles

99.2% missing data

4	(0.244) 0.241 0.250

1	(0.756) 0.759 0.750

Locus 20781 :

2 alleles

17.6% missing data

2	(0.982) 0.972 0.994

4	(0.018) 0.028 0.006

Locus 20782 :

3 alleles

98.8% missing data

2	(0.266) 0.264 0.268

3	(0.517) 0.523 0.512

4	(0.217) 0.213 0.220

Locus 20783 :

2 alleles

99.2% missing data

2	(0.328) 0.330 0.332

1	(0.672) 0.670 0.668

Locus 20784 :

2 alleles

99.2% missing data

2	(0.705) 0.700 0.700

4	(0.295) 0.300 0.300

Locus 20785 :

2 alleles

99.2% missing data

1	(0.228) 0.228 0.234

2	(0.772) 0.772 0.766

Locus 20786 :

2 alleles

99.2% missing data

4	(0.661) 0.661 0.660

2	(0.339) 0.339 0.340

Locus 20787 :

2 alleles

99.2% missing data

3	(0.251) 0.253 0.248

2	(0.749) 0.747 0.752

Locus 20788 :

3 alleles

98.4% missing data

3	(0.593) 0.588 0.596

1	(0.226) 0.225 0.221

4	(0.181) 0.187 0.182

Locus 20789 :

2 alleles

92.2% missing data

4	(0.896) 0.893 0.904

1	(0.104) 0.107 0.096

Locus 20790 :

2 alleles

97.3% missing data

3	(0.211) 0.211 0.206

4	(0.789) 0.789 0.794

Locus 20791 :

2 alleles

98.8% missing data

2	(0.526) 0.522 0.532

1	(0.474) 0.478 0.468

Locus 20792 :

3 alleles

98.8% missing data

2	(0.389) 0.402 0.381

1	(0.340) 0.336 0.346

3	(0.271) 0.261 0.274

Locus 20793 :

2 alleles

99.2% missing data

4	(0.489) 0.492 0.485

1	(0.511) 0.508 0.515

Locus 20794 :

2 alleles

98.8% missing data

1	(0.341) 0.347 0.340

4	(0.659) 0.653 0.660

Locus 20795 :

2 alleles

98.8% missing data

3	(0.342) 0.338 0.355

1	(0.658) 0.662 0.645

Locus 20796 :

2 alleles

99.2% missing data

2	(0.524) 0.525 0.526

1	(0.476) 0.475 0.474

Locus 20797 :

2 alleles

99.2% missing data

2	(0.550) 0.546 0.554

1	(0.450) 0.454 0.446

Locus 20798 :

2 alleles

99.2% missing data

3	(0.347) 0.344 0.356

4	(0.653) 0.656 0.644

Locus 20799 :

2 alleles

99.2% missing data

3	(0.689) 0.686 0.690

1	(0.311) 0.314 0.310

Locus 20800 :

2 alleles

99.2% missing data

2	(0.522) 0.522 0.520

4	(0.478) 0.478 0.480

Locus 20801 :

2 alleles

99.2% missing data

1	(0.718) 0.719 0.716

2	(0.282) 0.281 0.284

Locus 20802 :

2 alleles

2.3% missing data

4	(0.981) 0.968 0.994

2	(0.019) 0.032 0.006

Locus 20803 :

2 alleles

96.9% missing data

1	(0.206) 0.204 0.201

4	(0.794) 0.796 0.799

Locus 20804 :

2 alleles

99.2% missing data

2	(0.494) 0.493 0.497

1	(0.506) 0.507 0.503

Locus 20805 :

2 alleles

4.7% missing data

1	(0.959) 0.956 0.970

4	(0.041) 0.044 0.030

Locus 20806 :

2 alleles

99.2% missing data

4	(0.719) 0.712 0.726

2	(0.281) 0.288 0.274

Locus 20807 :

2 alleles

98.8% missing data

3	(0.622) 0.623 0.624

2	(0.378) 0.377 0.376

Locus 20808 :

2 alleles

99.2% missing data

3	(0.252) 0.251 0.255

1	(0.748) 0.749 0.745

Locus 20809 :

2 alleles

99.2% missing data

3	(0.551) 0.552 0.556

1	(0.449) 0.448 0.444

Locus 20810 :

2 alleles

96.9% missing data

3	(0.263) 0.260 0.258

1	(0.737) 0.740 0.742

Locus 20811 :

3 alleles

92.6% missing data

1	(0.602) 0.592 0.616

3	(0.131) 0.133 0.117

2	(0.266) 0.275 0.268

Locus 20812 :

2 alleles

8.2% missing data

4	(0.988) 0.981 0.997

2	(0.012) 0.019 0.003

Locus 20813 :

2 alleles

99.2% missing data

3	(0.403) 0.402 0.401

2	(0.597) 0.598 0.599

Locus 20814 :

2 alleles

0.0% missing data

2	(0.983) 0.987 0.992

3	(0.017) 0.013 0.008

Locus 20815 :

2 alleles

99.2% missing data

2	(0.679) 0.684 0.673

3	(0.321) 0.316 0.327

Locus 20816 :

2 alleles

99.2% missing data

1	(0.391) 0.387 0.398

4	(0.609) 0.613 0.602

Locus 20817 :

3 alleles

96.1% missing data

1	(0.219) 0.217 0.218

4	(0.664) 0.665 0.668

2	(0.117) 0.118 0.115

Locus 20818 :

2 alleles

99.2% missing data

1	(0.373) 0.369 0.372

4	(0.627) 0.631 0.628

Locus 20819 :

2 alleles

99.2% missing data

1	(0.508) 0.504 0.508

4	(0.492) 0.496 0.492

Locus 20820 :

2 alleles

99.2% missing data

4	(0.529) 0.534 0.520

1	(0.471) 0.466 0.480

Locus 20821 :

2 alleles

0.0% missing data

4	(0.908) 0.753 0.972

2	(0.092) 0.247 0.028

Locus 20822 :

2 alleles

91.0% missing data

3	(0.931) 0.944 0.924

2	(0.069) 0.056 0.076

Locus 20823 :

2 alleles

99.2% missing data

2	(0.181) 0.175 0.185

4	(0.819) 0.825 0.815

Locus 20824 :

2 alleles

8.2% missing data

4	(0.979) 0.971 0.993

3	(0.021) 0.029 0.007

Locus 20825 :

2 alleles

99.2% missing data

4	(0.792) 0.788 0.792

1	(0.208) 0.212 0.208

Locus 20826 :

2 alleles

99.2% missing data

2	(0.455) 0.457 0.455

4	(0.545) 0.543 0.545

Locus 20827 :

2 alleles

96.1% missing data

1	(0.858) 0.866 0.853

3	(0.142) 0.134 0.147

Locus 20828 :

2 alleles

8.2% missing data

3	(0.987) 0.983 0.996

4	(0.013) 0.017 0.004

Locus 20829 :

2 alleles

99.2% missing data

2	(0.268) 0.269 0.272

1	(0.732) 0.731 0.728

Locus 20830 :

2 alleles

0.0% missing data

4	(0.950) 0.961 0.948

1	(0.050) 0.039 0.052

Locus 20831 :

2 alleles

98.0% missing data

3	(0.706) 0.708 0.710

2	(0.294) 0.292 0.290

Locus 20832 :

2 alleles

99.2% missing data

1	(0.321) 0.313 0.325

4	(0.679) 0.687 0.675

Locus 20833 :

2 alleles

98.8% missing data

3	(0.492) 0.498 0.494

2	(0.508) 0.502 0.506

Locus 20834 :

2 alleles

99.2% missing data

2	(0.309) 0.316 0.311

4	(0.691) 0.684 0.689

Locus 20835 :

3 alleles

98.8% missing data

1	(0.427) 0.419 0.428

2	(0.414) 0.415 0.418

3	(0.159) 0.166 0.154

Locus 20836 :

2 alleles

99.2% missing data

4	(0.235) 0.237 0.230

1	(0.765) 0.763 0.770

Locus 20837 :

2 alleles

0.0% missing data

3	(0.987) 0.990 0.993

2	(0.013) 0.010 0.007

Locus 20838 :

2 alleles

99.2% missing data

2	(0.636) 0.627 0.647

4	(0.364) 0.373 0.353

Locus 20839 :

2 alleles

99.2% missing data

2	(0.798) 0.800 0.795

4	(0.202) 0.200 0.205

Locus 20840 :

3 alleles

9.0% missing data

2	(0.958) 0.928 0.987

1	(0.016) 0.021 0.005

3	(0.026) 0.052 0.008

Locus 20841 :

2 alleles

99.2% missing data

2	(0.548) 0.536 0.548

4	(0.452) 0.464 0.452

Locus 20842 :

2 alleles

98.8% missing data

2	(0.539) 0.542 0.535

3	(0.461) 0.458 0.465

Locus 20843 :

2 alleles

99.2% missing data

1	(0.470) 0.465 0.481

4	(0.530) 0.535 0.519

Locus 20844 :

2 alleles

98.4% missing data

4	(0.544) 0.545 0.545

1	(0.456) 0.455 0.455

Locus 20845 :

2 alleles

96.5% missing data

3	(0.163) 0.154 0.165

4	(0.837) 0.846 0.835

Locus 20846 :

2 alleles

92.2% missing data

4	(0.897) 0.902 0.904

3	(0.103) 0.098 0.096

Locus 20847 :

2 alleles

99.2% missing data

1	(0.368) 0.366 0.364

3	(0.632) 0.634 0.636

Locus 20848 :

2 alleles

99.2% missing data

3	(0.132) 0.128 0.133

4	(0.868) 0.872 0.867

Locus 20849 :

2 alleles

99.2% missing data

3	(0.499) 0.501 0.498

2	(0.501) 0.499 0.502

Locus 20850 :

2 alleles

99.2% missing data

2	(0.565) 0.561 0.565

3	(0.435) 0.439 0.435

Locus 20851 :

2 alleles

99.2% missing data

1	(0.346) 0.342 0.347

2	(0.654) 0.658 0.653

Locus 20852 :

2 alleles

98.8% missing data

3	(0.478) 0.478 0.484

4	(0.522) 0.522 0.516

Locus 20853 :

2 alleles

98.4% missing data

1	(0.794) 0.800 0.791

4	(0.206) 0.200 0.209

Locus 20854 :

2 alleles

97.7% missing data

3	(0.227) 0.225 0.229

4	(0.773) 0.775 0.771

Locus 20855 :

2 alleles

99.2% missing data

1	(0.280) 0.282 0.283

2	(0.720) 0.718 0.717

Locus 20856 :

2 alleles

99.2% missing data

4	(0.784) 0.779 0.784

3	(0.216) 0.221 0.216

Locus 20857 :

2 alleles

99.2% missing data

4	(0.498) 0.503 0.498

2	(0.502) 0.497 0.502

Locus 20858 :

3 alleles

98.0% missing data

4	(0.417) 0.416 0.425

1	(0.213) 0.214 0.213

3	(0.370) 0.369 0.362

Locus 20859 :

2 alleles

96.1% missing data

2	(0.117) 0.105 0.121

4	(0.883) 0.895 0.879

Locus 20860 :

2 alleles

99.2% missing data

3	(0.441) 0.441 0.440

2	(0.559) 0.559 0.560

Locus 20861 :

2 alleles

99.2% missing data

4	(0.445) 0.445 0.446

3	(0.555) 0.555 0.554

Locus 20862 :

2 alleles

91.0% missing data

2	(0.829) 0.850 0.826

1	(0.171) 0.150 0.174

Locus 20863 :

2 alleles

99.2% missing data

2	(0.695) 0.686 0.693

1	(0.305) 0.314 0.307

Locus 20864 :

2 alleles

99.2% missing data

4	(0.576) 0.572 0.582

3	(0.424) 0.428 0.418

Locus 20865 :

2 alleles

99.2% missing data

4	(0.356) 0.351 0.368

2	(0.644) 0.649 0.632

Locus 20866 :

2 alleles

98.8% missing data

4	(0.684) 0.690 0.691

3	(0.316) 0.310 0.309

Locus 20867 :

2 alleles

98.8% missing data

4	(0.389) 0.393 0.388

1	(0.611) 0.607 0.612

Locus 20868 :

3 alleles

97.7% missing data

3	(0.388) 0.391 0.386

4	(0.460) 0.460 0.460

2	(0.152) 0.148 0.154

Locus 20869 :

2 alleles

98.8% missing data

2	(0.647) 0.643 0.648

4	(0.353) 0.357 0.352

Locus 20870 :

3 alleles

98.8% missing data

2	(0.386) 0.379 0.386

4	(0.283) 0.280 0.280

3	(0.331) 0.341 0.334

Locus 20871 :

2 alleles

98.8% missing data

2	(0.353) 0.348 0.360

3	(0.647) 0.652 0.640

Locus 20872 :

3 alleles

98.8% missing data

1	(0.258) 0.259 0.263

2	(0.259) 0.263 0.255

3	(0.483) 0.478 0.482

Locus 20873 :

2 alleles

99.2% missing data

1	(0.637) 0.635 0.642

4	(0.363) 0.365 0.358

Locus 20874 :

2 alleles

98.4% missing data

4	(0.596) 0.594 0.605

2	(0.404) 0.406 0.395

Locus 20875 :

2 alleles

91.0% missing data

2	(0.906) 0.924 0.896

3	(0.094) 0.076 0.104

Locus 20876 :

2 alleles

98.8% missing data

3	(0.682) 0.677 0.691

1	(0.318) 0.323 0.309

Locus 20877 :

2 alleles

98.8% missing data

4	(0.655) 0.646 0.657

3	(0.345) 0.354 0.343

Locus 20878 :

2 alleles

95.7% missing data

4	(0.750) 0.753 0.760

2	(0.250) 0.247 0.240

Locus 20879 :

2 alleles

97.7% missing data

3	(0.518) 0.507 0.528

2	(0.482) 0.493 0.472

Locus 20880 :

2 alleles

99.2% missing data

1	(0.530) 0.527 0.533

2	(0.470) 0.473 0.467

Locus 20881 :

2 alleles

99.2% missing data

4	(0.500) 0.495 0.505

1	(0.500) 0.505 0.495

Locus 20882 :

2 alleles

99.2% missing data

3	(0.463) 0.461 0.470

1	(0.537) 0.539 0.530

Locus 20883 :

2 alleles

99.2% missing data

4	(0.369) 0.363 0.373

2	(0.631) 0.637 0.627

Locus 20884 :

2 alleles

98.8% missing data

4	(0.465) 0.468 0.467

3	(0.535) 0.532 0.533

Locus 20885 :

2 alleles

99.2% missing data

3	(0.447) 0.447 0.448

4	(0.553) 0.553 0.552

Locus 20886 :

2 alleles

99.2% missing data

1	(0.527) 0.532 0.524

4	(0.473) 0.468 0.476

Locus 20887 :

2 alleles

99.2% missing data

3	(0.738) 0.728 0.743

2	(0.262) 0.272 0.257

Locus 20888 :

2 alleles

96.9% missing data

2	(0.836) 0.840 0.838

4	(0.164) 0.160 0.162

Locus 20889 :

2 alleles

99.2% missing data

2	(0.550) 0.549 0.551

4	(0.450) 0.451 0.449

Locus 20890 :

2 alleles

99.2% missing data

2	(0.547) 0.552 0.550

3	(0.453) 0.448 0.450

Locus 20891 :

2 alleles

99.2% missing data

2	(0.575) 0.570 0.574

4	(0.425) 0.430 0.426

Locus 20892 :

2 alleles

99.2% missing data

4	(0.524) 0.523 0.526

3	(0.476) 0.477 0.474

Locus 20893 :

2 alleles

98.8% missing data

1	(0.148) 0.145 0.150

3	(0.852) 0.855 0.850

Locus 20894 :

2 alleles

96.9% missing data

4	(0.822) 0.830 0.816

1	(0.178) 0.170 0.184

Locus 20895 :

2 alleles

96.5% missing data

4	(0.803) 0.817 0.798

1	(0.197) 0.183 0.202

Locus 20896 :

2 alleles

99.2% missing data

4	(0.572) 0.568 0.571

1	(0.428) 0.432 0.429

Locus 20897 :

2 alleles

99.2% missing data

1	(0.563) 0.560 0.566

4	(0.437) 0.440 0.434

Locus 20898 :

2 alleles

99.2% missing data

2	(0.646) 0.648 0.647

3	(0.354) 0.352 0.353

Locus 20899 :

2 alleles

99.2% missing data

2	(0.803) 0.809 0.795

4	(0.197) 0.191 0.205

Locus 20900 :

2 alleles

99.2% missing data

3	(0.297) 0.294 0.297

4	(0.703) 0.706 0.703

Locus 20901 :

2 alleles

99.2% missing data

4	(0.114) 0.108 0.119

3	(0.886) 0.892 0.881

Locus 20902 :

2 alleles

99.2% missing data

3	(0.270) 0.271 0.276

2	(0.730) 0.729 0.724

Locus 20903 :

2 alleles

99.2% missing data

4	(0.534) 0.527 0.538

1	(0.466) 0.473 0.462

Locus 20904 :

2 alleles

98.8% missing data

4	(0.423) 0.422 0.431

2	(0.577) 0.578 0.569

Locus 20905 :

2 alleles

99.2% missing data

3	(0.762) 0.758 0.762

4	(0.238) 0.242 0.238

Locus 20906 :

2 alleles

99.2% missing data

3	(0.592) 0.596 0.591

4	(0.408) 0.404 0.409

Locus 20907 :

2 alleles

99.2% missing data

3	(0.572) 0.568 0.573

4	(0.428) 0.432 0.427

Locus 20908 :

2 alleles

99.2% missing data

3	(0.748) 0.756 0.737

4	(0.252) 0.244 0.263

Locus 20909 :

2 alleles

8.6% missing data

2	(0.985) 0.977 0.996

4	(0.015) 0.023 0.004

Locus 20910 :

2 alleles

99.2% missing data

3	(0.678) 0.680 0.673

4	(0.322) 0.320 0.327

Locus 20911 :

2 alleles

99.2% missing data

2	(0.633) 0.624 0.639

1	(0.367) 0.376 0.361

Locus 20912 :

2 alleles

98.8% missing data

4	(0.640) 0.638 0.642

1	(0.360) 0.362 0.358

Locus 20913 :

2 alleles

99.2% missing data

1	(0.251) 0.245 0.257

3	(0.749) 0.755 0.743

Locus 20914 :

2 alleles

99.2% missing data

1	(0.690) 0.689 0.687

4	(0.310) 0.311 0.313

Locus 20915 :

2 alleles

96.9% missing data

2	(0.819) 0.822 0.821

4	(0.181) 0.178 0.179

Locus 20916 :

2 alleles

99.2% missing data

2	(0.454) 0.449 0.450

1	(0.546) 0.551 0.550

Locus 20917 :

2 alleles

98.8% missing data

3	(0.430) 0.431 0.436

4	(0.570) 0.569 0.564

Locus 20918 :

2 alleles

99.2% missing data

4	(0.510) 0.514 0.504

2	(0.490) 0.486 0.496

Locus 20919 :

2 alleles

0.0% missing data

1	(0.986) 0.980 0.996

3	(0.014) 0.020 0.004

Locus 20920 :

2 alleles

98.4% missing data

4	(0.281) 0.278 0.276

3	(0.719) 0.722 0.724

Locus 20921 :

2 alleles

99.2% missing data

1	(0.469) 0.468 0.473

4	(0.531) 0.532 0.527

Locus 20922 :

2 alleles

99.2% missing data

1	(0.654) 0.653 0.654

4	(0.346) 0.347 0.346

Locus 20923 :

2 alleles

99.2% missing data

3	(0.544) 0.538 0.549

4	(0.456) 0.462 0.451

Locus 20924 :

2 alleles

99.2% missing data

1	(0.567) 0.562 0.576

2	(0.433) 0.438 0.424

Locus 20925 :

2 alleles

98.0% missing data

3	(0.654) 0.664 0.649

4	(0.346) 0.336 0.351

Locus 20926 :

2 alleles

98.8% missing data

3	(0.367) 0.369 0.365

1	(0.633) 0.631 0.635

Locus 20927 :

2 alleles

99.2% missing data

1	(0.271) 0.273 0.270

4	(0.729) 0.727 0.730

Locus 20928 :

2 alleles

96.1% missing data

2	(0.179) 0.163 0.190

4	(0.821) 0.837 0.810

Locus 20929 :

2 alleles

98.8% missing data

4	(0.364) 0.362 0.370

3	(0.636) 0.638 0.630

Locus 20930 :

2 alleles

98.0% missing data

1	(0.735) 0.738 0.738

4	(0.265) 0.262 0.262

Locus 20931 :

2 alleles

99.2% missing data

4	(0.324) 0.317 0.332

1	(0.676) 0.683 0.668

Locus 20932 :

2 alleles

98.8% missing data

3	(0.647) 0.639 0.650

4	(0.353) 0.361 0.350

Locus 20933 :

2 alleles

96.1% missing data

4	(0.885) 0.886 0.884

1	(0.115) 0.114 0.116

Locus 20934 :

2 alleles

96.1% missing data

4	(0.749) 0.758 0.755

3	(0.251) 0.242 0.245

Locus 20935 :

3 alleles

98.8% missing data

4	(0.425) 0.425 0.422

2	(0.322) 0.324 0.320

3	(0.253) 0.250 0.258

Locus 20936 :

2 alleles

99.2% missing data

4	(0.472) 0.481 0.467

2	(0.528) 0.519 0.533

Locus 20937 :

2 alleles

99.2% missing data

4	(0.416) 0.410 0.419

2	(0.584) 0.590 0.581

Locus 20938 :

2 alleles

99.2% missing data

3	(0.552) 0.553 0.553

2	(0.448) 0.447 0.447

Locus 20939 :

2 alleles

99.2% missing data

3	(0.735) 0.731 0.742

4	(0.265) 0.269 0.258

Locus 20940 :

2 alleles

99.2% missing data

2	(0.661) 0.654 0.668

3	(0.339) 0.346 0.332

Locus 20941 :

2 alleles

99.2% missing data

1	(0.559) 0.564 0.554

2	(0.441) 0.436 0.446

Locus 20942 :

2 alleles

99.2% missing data

1	(0.536) 0.536 0.535

2	(0.464) 0.464 0.465

Locus 20943 :

2 alleles

99.2% missing data

3	(0.425) 0.424 0.427

1	(0.575) 0.576 0.573

Locus 20944 :

2 alleles

99.2% missing data

3	(0.661) 0.662 0.663

2	(0.339) 0.338 0.337

Locus 20945 :

2 alleles

99.2% missing data

4	(0.627) 0.628 0.632

1	(0.373) 0.372 0.368

Locus 20946 :

2 alleles

91.0% missing data

2	(0.911) 0.918 0.914

3	(0.089) 0.082 0.086

Locus 20947 :

3 alleles

97.3% missing data

1	(0.246) 0.250 0.235

4	(0.122) 0.117 0.125

3	(0.632) 0.633 0.640

Locus 20948 :

2 alleles

91.0% missing data

3	(0.939) 0.944 0.937

1	(0.061) 0.056 0.063

Locus 20949 :

2 alleles

99.2% missing data

4	(0.524) 0.528 0.518

3	(0.476) 0.472 0.482

Locus 20950 :

2 alleles

99.2% missing data

1	(0.464) 0.454 0.466

4	(0.536) 0.546 0.534

Locus 20951 :

2 alleles

99.2% missing data

2	(0.535) 0.530 0.538

3	(0.465) 0.470 0.462

Locus 20952 :

2 alleles

99.2% missing data

3	(0.156) 0.150 0.162

4	(0.844) 0.850 0.838

Locus 20953 :

2 alleles

99.2% missing data

3	(0.636) 0.635 0.635

2	(0.364) 0.365 0.365

Locus 20954 :

2 alleles

96.5% missing data

3	(0.743) 0.741 0.755

4	(0.257) 0.259 0.245

Locus 20955 :

2 alleles

98.8% missing data

3	(0.640) 0.635 0.641

4	(0.360) 0.365 0.359

Locus 20956 :

2 alleles

99.2% missing data

3	(0.558) 0.564 0.555

2	(0.442) 0.436 0.445

Locus 20957 :

3 alleles

96.9% missing data

3	(0.675) 0.666 0.692

1	(0.175) 0.177 0.165

4	(0.150) 0.157 0.143

Locus 20958 :

2 alleles

98.8% missing data

1	(0.781) 0.778 0.783

4	(0.219) 0.222 0.217

Locus 20959 :

2 alleles

99.2% missing data

2	(0.326) 0.327 0.322

4	(0.674) 0.673 0.678

Locus 20960 :

2 alleles

91.0% missing data

2	(0.932) 0.945 0.924

3	(0.068) 0.055 0.076

Locus 20961 :

2 alleles

98.4% missing data

3	(0.367) 0.360 0.371

4	(0.633) 0.640 0.629

Locus 20962 :

2 alleles

96.1% missing data

4	(0.849) 0.850 0.853

2	(0.151) 0.150 0.147

Locus 20963 :

2 alleles

98.0% missing data

1	(0.704) 0.708 0.703

2	(0.296) 0.292 0.297

Locus 20964 :

2 alleles

99.2% missing data

1	(0.518) 0.517 0.518

4	(0.482) 0.483 0.482

Locus 20965 :

2 alleles

99.2% missing data

3	(0.276) 0.284 0.270

4	(0.724) 0.716 0.730

Locus 20966 :

2 alleles

99.2% missing data

3	(0.653) 0.652 0.654

1	(0.347) 0.348 0.346

Locus 20967 :

2 alleles

99.2% missing data

4	(0.609) 0.607 0.604

2	(0.391) 0.393 0.396

Locus 20968 :

2 alleles

90.6% missing data

2	(0.942) 0.954 0.936

4	(0.058) 0.046 0.064

Locus 20969 :

2 alleles

99.2% missing data

4	(0.587) 0.587 0.587

3	(0.413) 0.413 0.413

Locus 20970 :

2 alleles

99.2% missing data

3	(0.553) 0.559 0.544

1	(0.447) 0.441 0.456

Locus 20971 :

2 alleles

98.4% missing data

2	(0.774) 0.779 0.775

1	(0.226) 0.221 0.225

Locus 20972 :

2 alleles

99.2% missing data

3	(0.655) 0.658 0.651

4	(0.345) 0.342 0.349

Locus 20973 :

2 alleles

98.4% missing data

4	(0.171) 0.174 0.168

1	(0.829) 0.826 0.832

Locus 20974 :

2 alleles

99.2% missing data

3	(0.386) 0.389 0.387

4	(0.614) 0.611 0.613

Locus 20975 :

2 alleles

99.2% missing data

3	(0.599) 0.593 0.598

2	(0.401) 0.407 0.402

Locus 20976 :

2 alleles

98.8% missing data

3	(0.669) 0.664 0.679

4	(0.331) 0.336 0.321

Locus 20977 :

2 alleles

99.2% missing data

1	(0.456) 0.451 0.459

2	(0.544) 0.549 0.541

Locus 20978 :

2 alleles

99.2% missing data

1	(0.643) 0.639 0.648

2	(0.357) 0.361 0.352

Locus 20979 :

2 alleles

99.2% missing data

4	(0.477) 0.471 0.480

1	(0.523) 0.529 0.520

Locus 20980 :

2 alleles

98.0% missing data

3	(0.826) 0.827 0.825

1	(0.174) 0.173 0.175

Locus 20981 :

2 alleles

96.9% missing data

1	(0.922) 0.928 0.920

3	(0.078) 0.072 0.080

Locus 20982 :

2 alleles

98.8% missing data

1	(0.670) 0.674 0.670

4	(0.330) 0.326 0.330

Locus 20983 :

2 alleles

99.2% missing data

1	(0.435) 0.433 0.437

4	(0.565) 0.567 0.563

Locus 20984 :

2 alleles

99.2% missing data

4	(0.496) 0.494 0.495

1	(0.504) 0.506 0.505

Locus 20985 :

2 alleles

99.2% missing data

2	(0.636) 0.634 0.645

3	(0.364) 0.366 0.355

Locus 20986 :

2 alleles

9.8% missing data

1	(0.985) 0.977 0.995

2	(0.015) 0.023 0.005

Locus 20987 :

2 alleles

99.2% missing data

2	(0.280) 0.283 0.277

4	(0.720) 0.717 0.723

Locus 20988 :

2 alleles

91.0% missing data

1	(0.932) 0.936 0.933

4	(0.068) 0.064 0.067

Locus 20989 :

2 alleles

99.2% missing data

4	(0.161) 0.160 0.167

1	(0.839) 0.840 0.833

Locus 20990 :

2 alleles

99.2% missing data

4	(0.821) 0.825 0.812

1	(0.179) 0.175 0.188

Locus 20991 :

2 alleles

99.2% missing data

1	(0.455) 0.452 0.458

3	(0.545) 0.548 0.542

Locus 20992 :

2 alleles

97.7% missing data

1	(0.242) 0.245 0.235

4	(0.758) 0.755 0.765

Locus 20993 :

2 alleles

90.6% missing data

3	(0.851) 0.866 0.845

2	(0.149) 0.134 0.155

Locus 20994 :

2 alleles

96.1% missing data

3	(0.832) 0.834 0.841

1	(0.168) 0.166 0.159

Locus 20995 :

3 alleles

98.8% missing data

3	(0.360) 0.358 0.357

2	(0.287) 0.291 0.287

4	(0.353) 0.351 0.356

Locus 20996 :

2 alleles

98.8% missing data

3	(0.444) 0.438 0.457

1	(0.556) 0.562 0.543

Locus 20997 :

2 alleles

99.2% missing data

3	(0.683) 0.673 0.685

1	(0.317) 0.327 0.315

Locus 20998 :

2 alleles

98.8% missing data

2	(0.698) 0.694 0.697

1	(0.302) 0.306 0.303

Locus 20999 :

3 alleles

98.8% missing data

1	(0.338) 0.334 0.342

2	(0.386) 0.393 0.374

3	(0.276) 0.273 0.284

Locus 21000 :

3 alleles

90.2% missing data

1	(0.830) 0.856 0.828

3	(0.056) 0.045 0.063

4	(0.114) 0.099 0.109

Locus 21001 :

2 alleles

96.9% missing data

4	(0.836) 0.842 0.837

3	(0.164) 0.158 0.163

Locus 21002 :

2 alleles

98.8% missing data

1	(0.802) 0.803 0.802

4	(0.198) 0.197 0.198

Locus 21003 :

3 alleles

98.4% missing data

1	(0.509) 0.509 0.515

2	(0.280) 0.278 0.281

4	(0.211) 0.213 0.204

Locus 21004 :

3 alleles

96.1% missing data

4	(0.610) 0.624 0.613

1	(0.215) 0.206 0.218

3	(0.174) 0.170 0.169

Locus 21005 :

2 alleles

98.8% missing data

2	(0.559) 0.548 0.566

3	(0.441) 0.452 0.434

Locus 21006 :

2 alleles

99.2% missing data

3	(0.633) 0.631 0.633

4	(0.367) 0.369 0.367

Locus 21007 :

2 alleles

99.2% missing data

4	(0.560) 0.556 0.560

1	(0.440) 0.444 0.440

Locus 21008 :

2 alleles

98.4% missing data

2	(0.502) 0.505 0.510

1	(0.498) 0.495 0.490

Locus 21009 :

2 alleles

99.2% missing data

1	(0.533) 0.535 0.537

3	(0.467) 0.465 0.463

Locus 21010 :

2 alleles

99.2% missing data

3	(0.545) 0.551 0.543

1	(0.455) 0.449 0.457

Locus 21011 :

2 alleles

99.2% missing data

2	(0.457) 0.455 0.462

1	(0.543) 0.545 0.538

Locus 21012 :

2 alleles

99.2% missing data

1	(0.561) 0.562 0.562

3	(0.439) 0.438 0.438

Locus 21013 :

2 alleles

99.2% missing data

1	(0.582) 0.576 0.589

3	(0.418) 0.424 0.411

Locus 21014 :

2 alleles

99.2% missing data

2	(0.359) 0.365 0.352

4	(0.641) 0.635 0.648

Locus 21015 :

2 alleles

99.2% missing data

4	(0.602) 0.595 0.606

1	(0.398) 0.405 0.394

Locus 21016 :

2 alleles

99.2% missing data

3	(0.686) 0.687 0.689

1	(0.314) 0.313 0.311

Locus 21017 :

2 alleles

99.2% missing data

3	(0.476) 0.470 0.480

4	(0.524) 0.530 0.520

Locus 21018 :

2 alleles

99.2% missing data

3	(0.540) 0.547 0.532

4	(0.460) 0.453 0.468

Locus 21019 :

2 alleles

99.2% missing data

1	(0.764) 0.764 0.755

3	(0.236) 0.236 0.245

Locus 21020 :

2 alleles

98.8% missing data

4	(0.654) 0.650 0.656

3	(0.346) 0.350 0.344

Locus 21021 :

2 alleles

99.2% missing data

4	(0.599) 0.597 0.599

3	(0.401) 0.403 0.401

Locus 21022 :

2 alleles

95.7% missing data

2	(0.744) 0.758 0.746

3	(0.256) 0.242 0.254

Locus 21023 :

2 alleles

97.3% missing data

3	(0.603) 0.616 0.595

4	(0.397) 0.384 0.405

Locus 21024 :

2 alleles

99.2% missing data

3	(0.615) 0.613 0.614

1	(0.385) 0.387 0.386

Locus 21025 :

2 alleles

98.4% missing data

3	(0.674) 0.675 0.676

4	(0.326) 0.325 0.324

Locus 21026 :

2 alleles

99.2% missing data

2	(0.425) 0.422 0.427

3	(0.575) 0.578 0.573

Locus 21027 :

2 alleles

99.2% missing data

2	(0.535) 0.533 0.536

1	(0.465) 0.467 0.464

Locus 21028 :

2 alleles

99.2% missing data

3	(0.280) 0.284 0.280

2	(0.720) 0.716 0.720

Locus 21029 :

2 alleles

97.3% missing data

1	(0.668) 0.665 0.686

3	(0.332) 0.335 0.314

Locus 21030 :

2 alleles

99.2% missing data

2	(0.407) 0.411 0.408

3	(0.593) 0.589 0.592

Locus 21031 :

2 alleles

98.4% missing data

1	(0.377) 0.374 0.385

2	(0.623) 0.626 0.615

Locus 21032 :

2 alleles

99.2% missing data

1	(0.344) 0.350 0.354

4	(0.656) 0.650 0.646

Locus 21033 :

2 alleles

99.2% missing data

2	(0.510) 0.519 0.501

3	(0.490) 0.481 0.499

Locus 21034 :

2 alleles

96.5% missing data

4	(0.839) 0.839 0.841

2	(0.161) 0.161 0.159

Locus 21035 :

2 alleles

91.0% missing data

1	(0.941) 0.946 0.938

3	(0.059) 0.054 0.062

Locus 21036 :

2 alleles

99.2% missing data

3	(0.634) 0.630 0.630

1	(0.366) 0.370 0.370

Locus 21037 :

2 alleles

99.2% missing data

3	(0.674) 0.670 0.677

1	(0.326) 0.330 0.323

Locus 21038 :

2 alleles

98.8% missing data

1	(0.651) 0.646 0.656

4	(0.349) 0.354 0.344

Locus 21039 :

2 alleles

99.2% missing data

2	(0.514) 0.516 0.512

1	(0.486) 0.484 0.488

Locus 21040 :

2 alleles

99.2% missing data

3	(0.209) 0.207 0.217

2	(0.791) 0.793 0.783

Locus 21041 :

2 alleles

99.2% missing data

1	(0.515) 0.516 0.516

4	(0.485) 0.484 0.484

Locus 21042 :

2 alleles

99.2% missing data

2	(0.271) 0.268 0.282

3	(0.729) 0.732 0.718

Locus 21043 :

2 alleles

99.2% missing data

1	(0.282) 0.282 0.286

4	(0.718) 0.718 0.714

Locus 21044 :

2 alleles

99.2% missing data

3	(0.437) 0.439 0.444

4	(0.563) 0.561 0.556

Locus 21045 :

2 alleles

99.2% missing data

3	(0.763) 0.772 0.760

1	(0.237) 0.228 0.240

Locus 21046 :

2 alleles

99.2% missing data

1	(0.734) 0.729 0.738

4	(0.266) 0.271 0.262

Locus 21047 :

2 alleles

99.2% missing data

3	(0.761) 0.770 0.755

4	(0.239) 0.230 0.245

Locus 21048 :

2 alleles

99.2% missing data

3	(0.588) 0.590 0.583

4	(0.412) 0.410 0.417

Locus 21049 :

2 alleles

99.2% missing data

4	(0.693) 0.694 0.691

1	(0.307) 0.306 0.309

Locus 21050 :

2 alleles

99.2% missing data

3	(0.443) 0.439 0.449

4	(0.557) 0.561 0.551

Locus 21051 :

2 alleles

99.2% missing data

3	(0.274) 0.265 0.280

1	(0.726) 0.735 0.720

Locus 21052 :

2 alleles

92.2% missing data

3	(0.913) 0.912 0.919

1	(0.087) 0.088 0.081

Locus 21053 :

2 alleles

99.2% missing data

3	(0.533) 0.539 0.534

2	(0.467) 0.461 0.466

Locus 21054 :

2 alleles

98.8% missing data

1	(0.567) 0.572 0.567

3	(0.433) 0.428 0.433

Locus 21055 :

3 alleles

97.7% missing data

3	(0.211) 0.203 0.212

1	(0.659) 0.661 0.662

4	(0.131) 0.136 0.126

Locus 21056 :

2 alleles

99.2% missing data

1	(0.631) 0.628 0.633

3	(0.369) 0.372 0.367

Locus 21057 :

2 alleles

98.0% missing data

2	(0.541) 0.542 0.549

4	(0.459) 0.458 0.451

Locus 21058 :

2 alleles

98.8% missing data

3	(0.179) 0.171 0.187

1	(0.821) 0.829 0.813

Locus 21059 :

2 alleles

99.2% missing data

3	(0.413) 0.415 0.403

4	(0.587) 0.585 0.597

Locus 21060 :

2 alleles

98.8% missing data

4	(0.465) 0.464 0.457

1	(0.535) 0.536 0.543

Locus 21061 :

2 alleles

99.2% missing data

3	(0.532) 0.538 0.528

2	(0.468) 0.462 0.472

Locus 21062 :

2 alleles

99.2% missing data

4	(0.410) 0.413 0.413

1	(0.590) 0.587 0.587

Locus 21063 :

2 alleles

0.0% missing data

2	(0.986) 0.981 0.996

3	(0.014) 0.019 0.004

Locus 21064 :

2 alleles

97.7% missing data

4	(0.345) 0.350 0.331

2	(0.655) 0.650 0.669

Locus 21065 :

2 alleles

99.2% missing data

2	(0.356) 0.353 0.362

3	(0.644) 0.647 0.638

Locus 21066 :

2 alleles

98.8% missing data

3	(0.576) 0.565 0.579

4	(0.424) 0.435 0.421

Locus 21067 :

2 alleles

99.2% missing data

1	(0.500) 0.496 0.499

3	(0.500) 0.504 0.501

Locus 21068 :

2 alleles

90.6% missing data

2	(0.925) 0.937 0.917

1	(0.075) 0.063 0.083

Locus 21069 :

2 alleles

99.2% missing data

1	(0.301) 0.302 0.310

4	(0.699) 0.698 0.690

Locus 21070 :

2 alleles

99.2% missing data

4	(0.494) 0.497 0.497

1	(0.506) 0.503 0.503

Locus 21071 :

2 alleles

90.2% missing data

2	(0.815) 0.849 0.779

4	(0.185) 0.151 0.221

Locus 21072 :

2 alleles

99.2% missing data

3	(0.627) 0.633 0.625

1	(0.373) 0.367 0.375

Locus 21073 :

3 alleles

98.8% missing data

1	(0.253) 0.262 0.247

3	(0.363) 0.356 0.366

2	(0.384) 0.382 0.387

Locus 21074 :

2 alleles

98.4% missing data

2	(0.379) 0.379 0.377

3	(0.621) 0.621 0.623

Locus 21075 :

2 alleles

98.0% missing data

4	(0.676) 0.680 0.680

1	(0.324) 0.320 0.320

Locus 21076 :

2 alleles

91.4% missing data

4	(0.941) 0.938 0.951

3	(0.059) 0.062 0.049

Locus 21077 :

2 alleles

99.2% missing data

4	(0.567) 0.569 0.559

1	(0.433) 0.431 0.441

Locus 21078 :

2 alleles

98.4% missing data

1	(0.169) 0.166 0.171

3	(0.831) 0.834 0.829

Locus 21079 :

2 alleles

99.2% missing data

2	(0.633) 0.640 0.629

1	(0.367) 0.360 0.371

Locus 21080 :

2 alleles

0.0% missing data

3	(0.988) 0.984 0.996

2	(0.012) 0.016 0.004

Locus 21081 :

2 alleles

99.2% missing data

3	(0.346) 0.347 0.345

4	(0.654) 0.653 0.655

Locus 21082 :

3 alleles

94.5% missing data

2	(0.294) 0.280 0.295

3	(0.211) 0.210 0.214

1	(0.495) 0.510 0.491

Locus 21083 :

2 alleles

99.2% missing data

4	(0.616) 0.611 0.625

3	(0.384) 0.389 0.375

Locus 21084 :

2 alleles

98.8% missing data

4	(0.822) 0.813 0.828

3	(0.178) 0.187 0.172

Locus 21085 :

2 alleles

99.2% missing data

4	(0.260) 0.261 0.266

1	(0.740) 0.739 0.734

Locus 21086 :

2 alleles

99.2% missing data

1	(0.630) 0.622 0.635

2	(0.370) 0.378 0.365

Locus 21087 :

2 alleles

99.2% missing data

1	(0.280) 0.276 0.283

4	(0.720) 0.724 0.717

Locus 21088 :

2 alleles

99.2% missing data

3	(0.643) 0.644 0.644

1	(0.357) 0.356 0.356

Locus 21089 :

2 alleles

96.1% missing data

2	(0.232) 0.219 0.238

1	(0.768) 0.781 0.762

Locus 21090 :

2 alleles

98.8% missing data

1	(0.676) 0.677 0.676

2	(0.324) 0.323 0.324

Locus 21091 :

2 alleles

98.0% missing data

4	(0.820) 0.816 0.823

1	(0.180) 0.184 0.177

Locus 21092 :

2 alleles

1.2% missing data

4	(0.984) 0.988 0.991

1	(0.016) 0.012 0.009

Locus 21093 :

2 alleles

99.2% missing data

1	(0.204) 0.197 0.206

3	(0.796) 0.803 0.794

Locus 21094 :

2 alleles

90.6% missing data

1	(0.942) 0.947 0.943

4	(0.058) 0.053 0.057

Locus 21095 :

2 alleles

97.7% missing data

3	(0.398) 0.397 0.420

1	(0.602) 0.603 0.580

Locus 21096 :

2 alleles

96.9% missing data

1	(0.801) 0.794 0.814

4	(0.199) 0.206 0.186

Locus 21097 :

2 alleles

98.8% missing data

4	(0.565) 0.567 0.559

2	(0.435) 0.433 0.441

Locus 21098 :

2 alleles

96.9% missing data

2	(0.784) 0.794 0.778

1	(0.216) 0.206 0.222

Locus 21099 :

2 alleles

94.5% missing data

4	(0.798) 0.816 0.790

3	(0.202) 0.184 0.210

Locus 21100 :

2 alleles

99.2% missing data

3	(0.649) 0.648 0.651

2	(0.351) 0.352 0.349

Locus 21101 :

2 alleles

91.0% missing data

3	(0.924) 0.932 0.929

1	(0.076) 0.068 0.071

Locus 21102 :

2 alleles

99.2% missing data

3	(0.725) 0.726 0.717

2	(0.275) 0.274 0.283

Locus 21103 :

2 alleles

96.5% missing data

1	(0.798) 0.809 0.794

2	(0.202) 0.191 0.206

Locus 21104 :

2 alleles

99.2% missing data

3	(0.528) 0.531 0.524

2	(0.472) 0.469 0.476

Locus 21105 :

2 alleles

99.2% missing data

4	(0.646) 0.640 0.648

3	(0.354) 0.360 0.352

Locus 21106 :

2 alleles

99.2% missing data

2	(0.466) 0.450 0.471

3	(0.534) 0.550 0.529

Locus 21107 :

2 alleles

98.8% missing data

4	(0.463) 0.459 0.464

1	(0.537) 0.541 0.536

Locus 21108 :

2 alleles

97.7% missing data

1	(0.766) 0.763 0.772

3	(0.234) 0.237 0.228

Locus 21109 :

2 alleles

99.2% missing data

4	(0.751) 0.754 0.744

2	(0.249) 0.246 0.256

Locus 21110 :

2 alleles

97.7% missing data

2	(0.326) 0.306 0.336

3	(0.674) 0.694 0.664

Locus 21111 :

2 alleles

99.2% missing data

2	(0.520) 0.528 0.516

4	(0.480) 0.472 0.484

Locus 21112 :

2 alleles

99.2% missing data

1	(0.707) 0.700 0.712

3	(0.293) 0.300 0.288

Locus 21113 :

2 alleles

99.2% missing data

1	(0.374) 0.373 0.372

3	(0.626) 0.627 0.628

Locus 21114 :

2 alleles

98.8% missing data

3	(0.859) 0.858 0.860

1	(0.141) 0.142 0.140

Locus 21115 :

3 alleles

98.0% missing data

3	(0.529) 0.533 0.533

2	(0.151) 0.152 0.151

1	(0.320) 0.316 0.316

Locus 21116 :

2 alleles

98.8% missing data

4	(0.336) 0.341 0.330

2	(0.664) 0.659 0.670

Locus 21117 :

2 alleles

98.8% missing data

3	(0.826) 0.831 0.823

2	(0.174) 0.169 0.177

Locus 21118 :

2 alleles

98.0% missing data

1	(0.645) 0.642 0.644

3	(0.355) 0.358 0.356

Locus 21119 :

2 alleles

87.1% missing data

4	(0.943) 0.957 0.935

3	(0.057) 0.043 0.065

Locus 21120 :

2 alleles

99.2% missing data

2	(0.787) 0.791 0.784

4	(0.213) 0.209 0.216

Locus 21121 :

2 alleles

99.2% missing data

1	(0.470) 0.468 0.464

4	(0.530) 0.532 0.536

Locus 21122 :

2 alleles

98.8% missing data

4	(0.417) 0.412 0.422

1	(0.583) 0.588 0.578

Locus 21123 :

2 alleles

99.2% missing data

3	(0.601) 0.595 0.608

2	(0.399) 0.405 0.392

Locus 21124 :

2 alleles

99.2% missing data

2	(0.433) 0.427 0.438

1	(0.567) 0.573 0.562

Locus 21125 :

2 alleles

99.2% missing data

1	(0.500) 0.492 0.502

2	(0.500) 0.508 0.498

Locus 21126 :

2 alleles

98.8% missing data

1	(0.423) 0.415 0.429

2	(0.577) 0.585 0.571

Locus 21127 :

2 alleles

97.7% missing data

2	(0.288) 0.287 0.283

3	(0.712) 0.713 0.717

Locus 21128 :

2 alleles

0.0% missing data

3	(0.988) 0.983 0.997

2	(0.012) 0.017 0.003

Locus 21129 :

2 alleles

99.2% missing data

2	(0.491) 0.487 0.484

4	(0.509) 0.513 0.516

Locus 21130 :

2 alleles

97.7% missing data

3	(0.833) 0.832 0.841

2	(0.167) 0.168 0.159

Locus 21131 :

2 alleles

99.2% missing data

2	(0.429) 0.428 0.432

1	(0.571) 0.572 0.568

Locus 21132 :

2 alleles

98.8% missing data

2	(0.758) 0.759 0.752

4	(0.242) 0.241 0.248

Locus 21133 :

2 alleles

99.2% missing data

3	(0.659) 0.665 0.654

2	(0.341) 0.335 0.346

Locus 21134 :

2 alleles

98.8% missing data

4	(0.842) 0.838 0.842

1	(0.158) 0.162 0.158

Locus 21135 :

2 alleles

99.2% missing data

4	(0.536) 0.537 0.536

1	(0.464) 0.463 0.464

Locus 21136 :

2 alleles

98.8% missing data

3	(0.447) 0.454 0.444

1	(0.553) 0.546 0.556

Locus 21137 :

2 alleles

99.2% missing data

2	(0.624) 0.625 0.623

3	(0.376) 0.375 0.377

Locus 21138 :

2 alleles

99.2% missing data

2	(0.437) 0.437 0.427

1	(0.563) 0.563 0.573

Locus 21139 :

2 alleles

99.2% missing data

2	(0.388) 0.385 0.393

4	(0.612) 0.615 0.607

Locus 21140 :

3 alleles

98.0% missing data

3	(0.466) 0.455 0.470

4	(0.187) 0.185 0.193

1	(0.347) 0.360 0.337

Locus 21141 :

2 alleles

99.2% missing data

1	(0.582) 0.581 0.587

2	(0.418) 0.419 0.413

Locus 21142 :

2 alleles

0.0% missing data

2	(0.987) 0.983 0.996

3	(0.013) 0.017 0.004

Locus 21143 :

2 alleles

99.2% missing data

1	(0.364) 0.369 0.364

4	(0.636) 0.631 0.636

Locus 21144 :

2 alleles

98.8% missing data

1	(0.361) 0.360 0.369

3	(0.639) 0.640 0.631

Locus 21145 :

2 alleles

99.2% missing data

1	(0.353) 0.349 0.354

4	(0.647) 0.651 0.646

Locus 21146 :

2 alleles

99.2% missing data

4	(0.729) 0.730 0.727

1	(0.271) 0.270 0.273

Locus 21147 :

2 alleles

0.0% missing data

4	(0.990) 0.985 0.997

3	(0.010) 0.015 0.003

Locus 21148 :

2 alleles

99.2% missing data

3	(0.724) 0.725 0.723

1	(0.276) 0.275 0.277

Locus 21149 :

2 alleles

99.2% missing data

1	(0.546) 0.544 0.544

2	(0.454) 0.456 0.456

Locus 21150 :

2 alleles

96.5% missing data

4	(0.170) 0.158 0.175

2	(0.830) 0.842 0.825

Locus 21151 :

2 alleles

98.8% missing data

1	(0.339) 0.352 0.335

4	(0.661) 0.648 0.665

Locus 21152 :

2 alleles

98.0% missing data

2	(0.717) 0.711 0.727

4	(0.283) 0.289 0.273

Locus 21153 :

2 alleles

99.2% missing data

4	(0.549) 0.555 0.550

3	(0.451) 0.445 0.450

Locus 21154 :

2 alleles

99.2% missing data

3	(0.523) 0.523 0.518

4	(0.477) 0.477 0.482

Locus 21155 :

2 alleles

99.2% missing data

3	(0.460) 0.452 0.466

4	(0.540) 0.548 0.534

Locus 21156 :

2 alleles

0.0% missing data

1	(0.991) 0.985 0.997

4	(0.009) 0.015 0.003

Locus 21157 :

2 alleles

99.2% missing data

4	(0.396) 0.391 0.396

1	(0.604) 0.609 0.604

Locus 21158 :

2 alleles

98.8% missing data

2	(0.443) 0.442 0.452

1	(0.557) 0.558 0.548

Locus 21159 :

2 alleles

99.2% missing data

4	(0.510) 0.511 0.514

3	(0.490) 0.489 0.486

Locus 21160 :

2 alleles

99.2% missing data

1	(0.463) 0.464 0.462

2	(0.537) 0.536 0.538

Locus 21161 :

2 alleles

99.2% missing data

3	(0.487) 0.485 0.485

1	(0.513) 0.515 0.515

Locus 21162 :

2 alleles

99.2% missing data

2	(0.411) 0.409 0.415

4	(0.589) 0.591 0.585

Locus 21163 :

2 alleles

99.2% missing data

1	(0.662) 0.661 0.671

2	(0.338) 0.339 0.329

Locus 21164 :

3 alleles

96.9% missing data

2	(0.208) 0.204 0.197

4	(0.575) 0.572 0.600

3	(0.217) 0.224 0.203

Locus 21165 :

2 alleles

98.8% missing data

2	(0.594) 0.584 0.606

1	(0.406) 0.416 0.394

Locus 21166 :

2 alleles

98.8% missing data

4	(0.644) 0.644 0.645

2	(0.356) 0.356 0.355

Locus 21167 :

2 alleles

93.0% missing data

3	(0.151) 0.152 0.138

2	(0.849) 0.848 0.862

Locus 21168 :

2 alleles

99.2% missing data

4	(0.471) 0.474 0.473

2	(0.529) 0.526 0.527

Locus 21169 :

2 alleles

99.2% missing data

4	(0.173) 0.175 0.170

2	(0.827) 0.825 0.830

Locus 21170 :

2 alleles

99.2% missing data

4	(0.476) 0.474 0.469

3	(0.524) 0.526 0.531

Locus 21171 :

2 alleles

0.4% missing data

3	(0.988) 0.984 0.997

4	(0.012) 0.016 0.003

Locus 21172 :

2 alleles

99.2% missing data

2	(0.666) 0.667 0.663

4	(0.334) 0.333 0.337

Locus 21173 :

3 alleles

98.4% missing data

2	(0.525) 0.520 0.527

1	(0.210) 0.213 0.213

4	(0.265) 0.266 0.260

Locus 21174 :

2 alleles

99.2% missing data

2	(0.330) 0.329 0.336

3	(0.670) 0.671 0.664

Locus 21175 :

2 alleles

99.2% missing data

1	(0.441) 0.445 0.439

3	(0.559) 0.555 0.561

Locus 21176 :

2 alleles

99.2% missing data

2	(0.486) 0.490 0.479

1	(0.514) 0.510 0.521

Locus 21177 :

2 alleles

98.8% missing data

3	(0.485) 0.490 0.478

4	(0.515) 0.510 0.522

Locus 21178 :

2 alleles

99.2% missing data

1	(0.366) 0.368 0.366

4	(0.634) 0.632 0.634

Locus 21179 :

2 alleles

95.7% missing data

4	(0.810) 0.813 0.816

3	(0.190) 0.187 0.184

Locus 21180 :

2 alleles

99.2% missing data

4	(0.642) 0.639 0.644

1	(0.358) 0.361 0.356

Locus 21181 :

2 alleles

99.2% missing data

2	(0.450) 0.446 0.458

4	(0.550) 0.554 0.542

Locus 21182 :

3 alleles

98.8% missing data

4	(0.274) 0.273 0.277

3	(0.247) 0.252 0.246

2	(0.478) 0.475 0.477

Locus 21183 :

2 alleles

99.2% missing data

3	(0.508) 0.509 0.512

1	(0.492) 0.491 0.488

Locus 21184 :

2 alleles

73.4% missing data

1	(0.954) 0.957 0.965

4	(0.046) 0.043 0.035

Locus 21185 :

2 alleles

0.0% missing data

3	(0.989) 0.985 0.997

4	(0.011) 0.015 0.003

Locus 21186 :

2 alleles

99.2% missing data

2	(0.573) 0.568 0.582

4	(0.427) 0.432 0.418

Locus 21187 :

2 alleles

99.2% missing data

1	(0.647) 0.642 0.651

3	(0.353) 0.358 0.349

Locus 21188 :

3 alleles

97.3% missing data

4	(0.198) 0.194 0.193

3	(0.292) 0.292 0.284

1	(0.510) 0.514 0.523

Locus 21189 :

2 alleles

99.2% missing data

1	(0.610) 0.605 0.612

2	(0.390) 0.395 0.388

Locus 21190 :

2 alleles

99.2% missing data

1	(0.345) 0.342 0.347

4	(0.655) 0.658 0.653

Locus 21191 :

2 alleles

99.2% missing data

3	(0.618) 0.619 0.613

1	(0.382) 0.381 0.387

Locus 21192 :

2 alleles

99.2% missing data

2	(0.402) 0.399 0.401

3	(0.598) 0.601 0.599

Locus 21193 :

2 alleles

99.2% missing data

1	(0.330) 0.339 0.328

3	(0.670) 0.661 0.672

Locus 21194 :

2 alleles

99.2% missing data

1	(0.476) 0.479 0.474

3	(0.524) 0.521 0.526

Locus 21195 :

2 alleles

91.0% missing data

2	(0.929) 0.938 0.922

1	(0.071) 0.062 0.078

Locus 21196 :

2 alleles

99.2% missing data

1	(0.575) 0.579 0.573

2	(0.425) 0.421 0.427

Locus 21197 :

2 alleles

99.2% missing data

4	(0.257) 0.251 0.258

3	(0.743) 0.749 0.742

Locus 21198 :

3 alleles

97.3% missing data

2	(0.438) 0.433 0.455

1	(0.399) 0.395 0.393

4	(0.163) 0.171 0.151

Locus 21199 :

2 alleles

98.4% missing data

1	(0.207) 0.205 0.207

3	(0.793) 0.795 0.793

Locus 21200 :

2 alleles

99.2% missing data

3	(0.632) 0.625 0.635

1	(0.368) 0.375 0.365

Locus 21201 :

2 alleles

99.2% missing data

4	(0.770) 0.762 0.771

3	(0.230) 0.238 0.229

Locus 21202 :

3 alleles

96.5% missing data

4	(0.312) 0.313 0.314

2	(0.532) 0.523 0.540

1	(0.156) 0.164 0.147

Locus 21203 :

2 alleles

99.2% missing data

3	(0.655) 0.648 0.657

1	(0.345) 0.352 0.343

Locus 21204 :

2 alleles

99.2% missing data

3	(0.445) 0.439 0.447

1	(0.555) 0.561 0.553

Locus 21205 :

2 alleles

93.0% missing data

4	(0.601) 0.601 0.607

1	(0.399) 0.399 0.393

Locus 21206 :

3 alleles

98.0% missing data

4	(0.386) 0.403 0.376

2	(0.225) 0.219 0.233

1	(0.389) 0.377 0.390

Locus 21207 :

2 alleles

99.2% missing data

3	(0.421) 0.430 0.416

1	(0.579) 0.570 0.584

Locus 21208 :

2 alleles

98.8% missing data

1	(0.880) 0.884 0.871

4	(0.120) 0.116 0.129

Locus 21209 :

2 alleles

94.9% missing data

1	(0.879) 0.879 0.881

4	(0.121) 0.121 0.119

Locus 21210 :

3 alleles

98.8% missing data

3	(0.286) 0.290 0.284

2	(0.396) 0.391 0.402

4	(0.318) 0.319 0.314

Locus 21211 :

2 alleles

99.2% missing data

4	(0.424) 0.423 0.429

1	(0.576) 0.577 0.571

Locus 21212 :

2 alleles

99.2% missing data

3	(0.338) 0.343 0.333

1	(0.662) 0.657 0.667

Locus 21213 :

2 alleles

98.4% missing data

4	(0.732) 0.739 0.724

1	(0.268) 0.261 0.276

Locus 21214 :

2 alleles

99.2% missing data

3	(0.845) 0.847 0.842

4	(0.155) 0.153 0.158

Locus 21215 :

2 alleles

99.2% missing data

4	(0.733) 0.736 0.731

3	(0.267) 0.264 0.269

Locus 21216 :

2 alleles

99.2% missing data

2	(0.644) 0.645 0.646

1	(0.356) 0.355 0.354

Locus 21217 :

2 alleles

98.8% missing data

2	(0.172) 0.170 0.177

1	(0.828) 0.830 0.823

Locus 21218 :

3 alleles

98.4% missing data

3	(0.342) 0.339 0.350

2	(0.320) 0.324 0.311

4	(0.338) 0.337 0.338

Locus 21219 :

2 alleles

99.2% missing data

3	(0.774) 0.774 0.770

1	(0.226) 0.226 0.230

Locus 21220 :

2 alleles

98.4% missing data

2	(0.470) 0.453 0.477

4	(0.530) 0.547 0.523

Locus 21221 :

2 alleles

99.2% missing data

4	(0.599) 0.594 0.601

2	(0.401) 0.406 0.399

Locus 21222 :

2 alleles

99.2% missing data

3	(0.319) 0.314 0.322

4	(0.681) 0.686 0.678

Locus 21223 :

2 alleles

99.2% missing data

4	(0.335) 0.336 0.336

1	(0.665) 0.664 0.664

Locus 21224 :

2 alleles

98.8% missing data

4	(0.634) 0.626 0.631

2	(0.366) 0.374 0.369

Locus 21225 :

3 alleles

98.8% missing data

2	(0.308) 0.311 0.296

4	(0.337) 0.337 0.346

3	(0.355) 0.352 0.358

Locus 21226 :

2 alleles

98.4% missing data

1	(0.761) 0.754 0.768

4	(0.239) 0.246 0.232

Locus 21227 :

2 alleles

99.2% missing data

3	(0.453) 0.453 0.450

4	(0.547) 0.547 0.550

Locus 21228 :

2 alleles

97.7% missing data

1	(0.326) 0.325 0.315

4	(0.674) 0.675 0.685

Locus 21229 :

2 alleles

99.2% missing data

4	(0.399) 0.398 0.403

3	(0.601) 0.602 0.597

Locus 21230 :

2 alleles

98.0% missing data

3	(0.836) 0.841 0.835

4	(0.164) 0.159 0.165

Locus 21231 :

2 alleles

99.2% missing data

2	(0.549) 0.547 0.554

3	(0.451) 0.453 0.446

Locus 21232 :

2 alleles

0.4% missing data

1	(0.986) 0.981 0.996

3	(0.014) 0.019 0.004

Locus 21233 :

2 alleles

99.2% missing data

4	(0.748) 0.746 0.744

3	(0.252) 0.254 0.256

Locus 21234 :

2 alleles

99.2% missing data

4	(0.685) 0.680 0.687

3	(0.315) 0.320 0.313

Locus 21235 :

2 alleles

99.2% missing data

4	(0.609) 0.606 0.614

1	(0.391) 0.394 0.386

Locus 21236 :

2 alleles

99.2% missing data

3	(0.637) 0.636 0.635

4	(0.363) 0.364 0.365

Locus 21237 :

2 alleles

98.8% missing data

1	(0.414) 0.424 0.406

4	(0.586) 0.576 0.594

Locus 21238 :

2 alleles

99.2% missing data

3	(0.425) 0.427 0.426

1	(0.575) 0.573 0.574

Locus 21239 :

2 alleles

98.8% missing data

2	(0.620) 0.631 0.616

3	(0.380) 0.369 0.384

Locus 21240 :

2 alleles

99.2% missing data

3	(0.485) 0.487 0.492

1	(0.515) 0.513 0.508

Locus 21241 :

2 alleles

96.9% missing data

3	(0.234) 0.228 0.242

2	(0.766) 0.772 0.758

Locus 21242 :

2 alleles

99.2% missing data

4	(0.381) 0.382 0.385

3	(0.619) 0.618 0.615

Locus 21243 :

2 alleles

97.7% missing data

2	(0.274) 0.276 0.272

4	(0.726) 0.724 0.728

Locus 21244 :

2 alleles

0.0% missing data

1	(0.914) 0.749 0.974

4	(0.086) 0.251 0.026

Locus 21245 :

2 alleles

98.0% missing data

1	(0.613) 0.621 0.613

3	(0.387) 0.379 0.387

Locus 21246 :

2 alleles

99.2% missing data

1	(0.472) 0.472 0.472

4	(0.528) 0.528 0.528

Locus 21247 :

2 alleles

99.2% missing data

2	(0.427) 0.425 0.421

4	(0.573) 0.575 0.579

Locus 21248 :

2 alleles

99.2% missing data

4	(0.524) 0.527 0.524

2	(0.476) 0.473 0.476

Locus 21249 :

2 alleles

99.2% missing data

1	(0.700) 0.704 0.700

4	(0.300) 0.296 0.300

Locus 21250 :

2 alleles

96.1% missing data

3	(0.199) 0.193 0.203

1	(0.801) 0.807 0.797

Locus 21251 :

2 alleles

99.2% missing data

3	(0.469) 0.460 0.474

1	(0.531) 0.540 0.526

Locus 21252 :

3 alleles

90.6% missing data

1	(0.840) 0.861 0.834

2	(0.061) 0.050 0.068

3	(0.100) 0.089 0.098

Locus 21253 :

3 alleles

98.0% missing data

2	(0.314) 0.321 0.314

1	(0.254) 0.251 0.259

3	(0.432) 0.428 0.428

Locus 21254 :

2 alleles

99.2% missing data

2	(0.194) 0.187 0.197

3	(0.806) 0.813 0.803

Locus 21255 :

2 alleles

96.1% missing data

3	(0.765) 0.761 0.773

1	(0.235) 0.239 0.227

Locus 21256 :

3 alleles

98.8% missing data

4	(0.498) 0.499 0.498

1	(0.284) 0.286 0.283

3	(0.218) 0.214 0.218

Locus 21257 :

2 alleles

99.2% missing data

1	(0.450) 0.447 0.453

3	(0.550) 0.553 0.547

Locus 21258 :

2 alleles

99.2% missing data

3	(0.625) 0.621 0.624

2	(0.375) 0.379 0.376

Locus 21259 :

2 alleles

99.2% missing data

4	(0.225) 0.231 0.222

1	(0.775) 0.769 0.778

Locus 21260 :

2 alleles

99.2% missing data

4	(0.211) 0.210 0.211

3	(0.789) 0.790 0.789

Locus 21261 :

2 alleles

99.2% missing data

4	(0.501) 0.500 0.508

3	(0.499) 0.500 0.492

Locus 21262 :

2 alleles

99.2% missing data

4	(0.492) 0.495 0.481

1	(0.508) 0.505 0.519

Locus 21263 :

2 alleles

98.8% missing data

4	(0.709) 0.710 0.712

3	(0.291) 0.290 0.288

Locus 21264 :

2 alleles

98.8% missing data

2	(0.827) 0.818 0.827

1	(0.173) 0.182 0.173

Locus 21265 :

2 alleles

99.2% missing data

2	(0.367) 0.355 0.374

4	(0.633) 0.645 0.626

Locus 21266 :

2 alleles

98.8% missing data

3	(0.717) 0.713 0.714

4	(0.283) 0.287 0.286

Locus 21267 :

2 alleles

99.2% missing data

2	(0.459) 0.457 0.464

4	(0.541) 0.543 0.536

Locus 21268 :

2 alleles

99.2% missing data

4	(0.313) 0.318 0.316

3	(0.687) 0.682 0.684

Locus 21269 :

2 alleles

99.2% missing data

4	(0.475) 0.476 0.475

1	(0.525) 0.524 0.525

Locus 21270 :

2 alleles

99.2% missing data

2	(0.458) 0.452 0.465

3	(0.542) 0.548 0.535

Locus 21271 :

2 alleles

99.2% missing data

2	(0.164) 0.161 0.169

3	(0.836) 0.839 0.831

Locus 21272 :

2 alleles

97.7% missing data

4	(0.745) 0.744 0.755

1	(0.255) 0.256 0.245

Locus 21273 :

2 alleles

99.2% missing data

3	(0.324) 0.324 0.321

1	(0.676) 0.676 0.679

Locus 21274 :

2 alleles

99.2% missing data

4	(0.549) 0.552 0.544

1	(0.451) 0.448 0.456

Locus 21275 :

2 alleles

98.8% missing data

4	(0.684) 0.672 0.685

3	(0.316) 0.328 0.315

Locus 21276 :

2 alleles

98.8% missing data

4	(0.488) 0.497 0.490

1	(0.512) 0.503 0.510

Locus 21277 :

2 alleles

96.9% missing data

3	(0.842) 0.839 0.847

4	(0.158) 0.161 0.153

Locus 21278 :

3 alleles

98.4% missing data

4	(0.465) 0.463 0.462

2	(0.280) 0.285 0.277

1	(0.255) 0.252 0.261

Locus 21279 :

2 alleles

99.2% missing data

3	(0.289) 0.280 0.300

1	(0.711) 0.720 0.700

Locus 21280 :

2 alleles

99.2% missing data

3	(0.648) 0.641 0.650

1	(0.352) 0.359 0.350

Locus 21281 :

3 alleles

98.8% missing data

2	(0.299) 0.295 0.304

3	(0.505) 0.500 0.501

1	(0.196) 0.205 0.195

Locus 21282 :

2 alleles

98.8% missing data

4	(0.394) 0.385 0.399

1	(0.606) 0.615 0.601

Locus 21283 :

2 alleles

99.2% missing data

4	(0.384) 0.387 0.384

3	(0.616) 0.613 0.616

Locus 21284 :

2 alleles

99.2% missing data

1	(0.610) 0.611 0.614

3	(0.390) 0.389 0.386

Locus 21285 :

2 alleles

96.9% missing data

2	(0.205) 0.209 0.193

4	(0.795) 0.791 0.807

Locus 21286 :

2 alleles

97.7% missing data

2	(0.756) 0.752 0.763

4	(0.244) 0.248 0.237

Locus 21287 :

2 alleles

0.0% missing data

4	(0.989) 0.985 0.996

1	(0.011) 0.015 0.004

Locus 21288 :

2 alleles

93.0% missing data

1	(0.868) 0.861 0.885

4	(0.132) 0.139 0.115

Locus 21289 :

2 alleles

99.2% missing data

4	(0.738) 0.736 0.748

1	(0.262) 0.264 0.252

Locus 21290 :

2 alleles

99.2% missing data

3	(0.351) 0.342 0.353

2	(0.649) 0.658 0.647

Locus 21291 :

2 alleles

99.2% missing data

4	(0.365) 0.359 0.372

1	(0.635) 0.641 0.628

Locus 21292 :

2 alleles

90.6% missing data

1	(0.900) 0.918 0.891

2	(0.100) 0.082 0.109

Locus 21293 :

2 alleles

99.2% missing data

1	(0.496) 0.499 0.500

3	(0.504) 0.501 0.500

Locus 21294 :

2 alleles

98.0% missing data

1	(0.624) 0.618 0.644

2	(0.376) 0.382 0.356

Locus 21295 :

2 alleles

99.2% missing data

4	(0.470) 0.467 0.476

2	(0.530) 0.533 0.524

Locus 21296 :

2 alleles

99.2% missing data

2	(0.321) 0.330 0.318

3	(0.679) 0.670 0.682

Locus 21297 :

2 alleles

98.8% missing data

1	(0.610) 0.615 0.605

2	(0.390) 0.385 0.395

Locus 21298 :

2 alleles

9.0% missing data

2	(0.984) 0.980 0.995

4	(0.016) 0.020 0.005

Locus 21299 :

2 alleles

96.5% missing data

4	(0.825) 0.827 0.829

3	(0.175) 0.173 0.171

Locus 21300 :

2 alleles

99.2% missing data

1	(0.508) 0.500 0.516

3	(0.492) 0.500 0.484

Locus 21301 :

2 alleles

99.2% missing data

1	(0.605) 0.599 0.606

3	(0.395) 0.401 0.394

Locus 21302 :

2 alleles

99.2% missing data

1	(0.331) 0.328 0.331

3	(0.669) 0.672 0.669

Locus 21303 :

3 alleles

97.3% missing data

1	(0.388) 0.403 0.385

4	(0.309) 0.301 0.315

3	(0.303) 0.296 0.299

Locus 21304 :

2 alleles

99.2% missing data

2	(0.583) 0.581 0.584

3	(0.417) 0.419 0.416

Locus 21305 :

2 alleles

95.3% missing data

1	(0.769) 0.785 0.764

4	(0.231) 0.215 0.236

Locus 21306 :

2 alleles

99.2% missing data

2	(0.155) 0.158 0.157

1	(0.845) 0.842 0.843

Locus 21307 :

2 alleles

99.2% missing data

2	(0.662) 0.665 0.655

3	(0.338) 0.335 0.345

Locus 21308 :

2 alleles

99.2% missing data

3	(0.406) 0.399 0.412

1	(0.594) 0.601 0.588

Locus 21309 :

2 alleles

99.2% missing data

1	(0.287) 0.293 0.284

2	(0.713) 0.707 0.716

Locus 21310 :

2 alleles

99.2% missing data

3	(0.691) 0.691 0.682

2	(0.309) 0.309 0.318

Locus 21311 :

2 alleles

99.2% missing data

1	(0.407) 0.401 0.410

2	(0.593) 0.599 0.590

Locus 21312 :

2 alleles

98.8% missing data

3	(0.492) 0.494 0.490

2	(0.508) 0.506 0.510

Locus 21313 :

2 alleles

98.4% missing data

4	(0.816) 0.823 0.813

1	(0.184) 0.177 0.187

Locus 21314 :

2 alleles

99.2% missing data

3	(0.552) 0.550 0.546

1	(0.448) 0.450 0.454

Locus 21315 :

2 alleles

98.8% missing data

4	(0.407) 0.400 0.406

1	(0.593) 0.600 0.594

Locus 21316 :

2 alleles

99.2% missing data

4	(0.634) 0.638 0.632

1	(0.366) 0.362 0.368

Locus 21317 :

3 alleles

92.6% missing data

3	(0.769) 0.768 0.790

1	(0.107) 0.105 0.101

2	(0.124) 0.126 0.109

Locus 21318 :

2 alleles

97.7% missing data

2	(0.811) 0.819 0.808

3	(0.189) 0.181 0.192

Locus 21319 :

2 alleles

99.2% missing data

1	(0.605) 0.599 0.615

2	(0.395) 0.401 0.385

Locus 21320 :

2 alleles

90.6% missing data

3	(0.905) 0.923 0.887

1	(0.095) 0.077 0.113

Locus 21321 :

2 alleles

91.0% missing data

3	(0.931) 0.943 0.921

2	(0.069) 0.057 0.079

Locus 21322 :

2 alleles

99.2% missing data

4	(0.677) 0.678 0.672

1	(0.323) 0.322 0.328

Locus 21323 :

2 alleles

99.2% missing data

4	(0.251) 0.260 0.245

3	(0.749) 0.740 0.755

Locus 21324 :

2 alleles

99.2% missing data

1	(0.581) 0.572 0.586

4	(0.419) 0.428 0.414

Locus 21325 :

2 alleles

7.4% missing data

2	(0.972) 0.955 0.991

3	(0.028) 0.045 0.009

Locus 21326 :

2 alleles

99.2% missing data

2	(0.303) 0.300 0.312

1	(0.697) 0.700 0.688

Locus 21327 :

2 alleles

99.2% missing data

1	(0.634) 0.629 0.633

3	(0.366) 0.371 0.367

Locus 21328 :

2 alleles

98.4% missing data

2	(0.644) 0.651 0.641

1	(0.356) 0.349 0.359

Locus 21329 :

2 alleles

97.7% missing data

4	(0.811) 0.804 0.818

1	(0.189) 0.196 0.182

Locus 21330 :

3 alleles

98.8% missing data

1	(0.253) 0.246 0.263

3	(0.507) 0.507 0.499

2	(0.240) 0.247 0.238

Locus 21331 :

2 alleles

99.2% missing data

2	(0.378) 0.375 0.388

3	(0.622) 0.625 0.612

Locus 21332 :

2 alleles

99.2% missing data

1	(0.538) 0.543 0.527
[truncated: 12,118,921 more chars]
